## [Peer Review File · Nature Communications]

Reviewers' comments:

Reviewer #1 (Remarks to the Author):

In the manuscript entitled "Comparative oncogenomics and iterative mouse modeling identifies combinations of driver genes and drug targets in BRCA1-mutated breast cancer", Annunziato, de Rooter, Henneman, Brambillasca, et al describe the characterization of copy number alterations (CNAs) in BRCA-1 mutated TNBC. Using this dataset as a guiding principle, they develop rapid and flexible mouse models of breast cancer to introduce putative drivers in the identified CNAs and analyze their contribution to tumor initiation. The authors observe the strong effect of Myc overexpression, as well as the contribution of Rb and Pten loss and the overexpression of Met in the number and latency of breast cancers. By performing CNA analysis in WB1P and WB1P-Myc, the authors identify a recurrent amplification of mouse chromosome 3, which is conserved in the syntenic 1q region of human TNBC. By performing a focused screening in organoids, the authors identify Mcl1 as a key survival factor embedded in this region that is important for the survival of Myc overexpressing tumors. Indeed, the authors observe that Myc overexpression leads to Mcl1 amplification and that this can be exploited as a synthetic lethality by using Mcl1 inhibitors in both mouse models of breast cancer and patient-derived xenografts.

Overall, the manuscript is very well written, the experiments are well designed and controlled, and the models developed in the paper will undoubtedly contribute to studying novel drivers in models of breast cancer. Although several of the observations in the manuscript do not come as a major surprise (e.g. Myc overexpression leads to more aggressive tumors), this is the first formal proof for several of such predictions in immunocompetent models of TNBC and as such is a valuable study. In this regard, and considering the vast amount of work already done, this manuscript would only need minor changes to be ready for publication.

Below is a list of the minor concerns:

-The authors use oncogene overexpression to test their contribution to cancer initiation and progression. Although I'm aware this is currently the most reasonable surrogate for such experiments, the authors should assess the levels of Myc or Met achieved by CNA (e.g. in the subset of WB1P tumors that gain chromosome 15) compared to the overexpression systems and comment about this potential drawback in the discussion.

-In line with the previous point, the authors should comment on the possibility that the reduced CNA number in WB1P-Myc (Figure 3C) tumors could also be explained by negative selection of cells with both high levels of Myc and CNAs. Although not strictly necessary, if possible the authors could analyze the levels of CNA burden and Myc protein change over time in the WB1P-Myc model.

-To test the requirement of Mcl1 amplification as a permissive factor for Myc amplification, authors could suppress Mcl1 function (e.g. CRISPR of Mcl1, or treatment with S63845) and test whether this intervention precludes the amplification of Myc.

-Chromosome 1p amplifications in humans tend to be quite broad and it seems unlikely that it is the only relevant driver. While the authors do a thorough job in implicating Mcl-1 as a contributor, they may wish to mention that there may be others to avoid giving the impression that 1p (mouse 3) gain = Mcl1 amplification.

-The authors should discuss the specificity (and potential off target effects) of the Mcl-1 antagonist they use in their study (S63845).

-The authors should consider citing a recent paper from Cambell/Blyth (Cell Death Disease 2018), which seems to support the role of Mcl1 as a driver and target in human breast cancer.

Reviewer #2 (Remarks to the Author):

BRCA1 mutation is one the major genetic pre-dispositions for breast cancer development. BRCA1 mutation breast cancer usually belongs to triple-negative breast cancer subtype, which has no effective treatment at the moment. It is thus of great public interest to further analyze the cooperative genetic events leading to this type of cancer. The authors tried to identify the cancer driver genes collaboratively working with BRCA1 mutant. Targeting the proteins coded by these genes simultaneously would in theory generating better outcomes than individual agent targeting only one specific protein or pathway. The authors eventually discovered that in BRCA1 mutated tumors, Myc amplification or over-expression leads to less DNA copy number alterations but is strongly associated with MCL1 amplification. Thus, PARP1 inhibitor (approved for BRCA1 mutant breast cancer patients) and MCL1 inhibitor synergistically delayed tumor growth in mouse models. This is an overall interesting and strong mouse modeling study, with utilization of quite a few in vivo genetically engineered mouse models and novel combination of GEMM with lentiviral infection method (for non-germline gene expression manipulation). The discovered co-amplification of MCL1 and MYC is also very interesting. I have a few minor questions that I would like the authors to address:

1. Since the major finding is that MYC and MCL1 are co-driver genes for BRCA1 mutation containing breast cancer, can the authors analyze the human breast cancer patient samples, and present the amplification map for these three genes similarly as in Fig. 1A. This will significantly enhance the cancer relevance of the discovery.

2. In Figure 2C, I am surprised that both B1P (Lenti-MycP2Acre) and WB1P (Lenti-Myc) mice develop tumors faster than B1P-Myc mice. Can the authors exam the Myc expression by

immunoblotting at early time points when no tumors developed in these mice yet or at late stage when mice already had tumors?

3. In Figure 4A, the shRNA dropout assay was used to determine which gene(s) was the most important one for tumorigenesis in an organoid assay. However, MCL1 might just be a critical oncogene that is needed for tumor growth but not specifically related to Myc-driven tumor. A control dropout assay by using WB1P tumor cells will be needed to make sure MCL1 is specifically needed for WB1P-Myc but not WB1P tumor.

Reviewer #3 (Remarks to the Author):

Jonkers and colleagues describe new in vivo systems to determine driver events in BRCA1 mutant mammary gland cancers. This entails development of methods for Cas9 mediated loss of function or c-myc overexpression events in combination with BRCA1 and p53 mutation. The work provides proof of concept that candidate driver genes can be more rapidly assessed than by conventional breeding schemes. This in itself represents a significant advance for the field. The results of the study are also notable for strong cooperation between c-Myc or Met oncogene overexpression dramatically reducing tumor latency in the context of BRCA1 mutation. PTEN or RB loss also produced more rapid tumor development. While these results are not entirely surprising, they provide important validation and also result in the unanticipated finding that c-Myc overexpression simplifies the CNA changes observed in B1P tumors. This facilitated identification of MCL1 as a driver change in B1P Myc tumors and that MCL1 inhibition + PARPi significantly delayed or c-myc overexpression tumor formation.

This is an important study and will be of broad interest considering its methodologic advances and the medical implication to BRCA1 mutated cancers. It does suffer somewhat from being diffuse and not going more in depth into the results of its many findings. Additional controls are missing to determine if MCL1 cooperation is mainly through Myc or has any specific relationship to BRCA1. This reduces the quality of the study. Nonetheless, the work is valuable and should be published upon some additional changes to the text.

Specific comments

1. Fig 2A-C. Myc overexpression dramatically reduces tumor latency in the B1P background. However, we don't know if it actually cooperates with BRCA1 mutation. What would the kinetics of tumor development be in Myc, p53 mice? Is this data purely driven by Myc and BRCA1 mutation merely a passenger that is affecting CNA.
2. Again, MCL1 inhibition may be primarily working against Myc rather than BRCA1. There is indeed substantial literature to show cooperation between MCL1 and c-Myc. Are the authors really providing new information with their data?

3. Fig 4G. Can the authors explain why the combination of PARPi and MCL1i is not curative?
4. Fig S8 MCL inhibitor S63845 has minimal additive efficacy over olaparib alone in WB1P-Myc tumors. Significance is not indicated

Reviewer #1, expert in breast cancer mouse models-

In the manuscript entitled “Comparative oncogenomics and iterative mouse modeling identifies combinations of driver genes and drug targets in BRCA1-mutated breast cancer”, Annunziato, de Ruyter, Henneman, Brambillasca, et al describe the characterization of copy number alterations (CNAs) in BRCA-1 mutated TNBC. Using this dataset as a guiding principle, they develop rapid and flexible mouse models of breast cancer to introduce putative drivers in the identified CNAs and analyze their contribution to tumor initiation. The authors observe the strong effect of Myc overexpression, as well as the contribution of Rb and Pten loss and the overexpression of Met in the number and latency of breast cancers. By performing CNA analysis in WB1P and WB1P-Myc, the authors identify a recurrent amplification of mouse chromosome 3, which is conserved in the syntenic 1q region of human TNBC. By performing a focused screening in organoids, the authors identify Mcl1 as a key survival factor embedded in this region that is important for the survival of Myc overexpressing tumors. Indeed, the authors observe that Myc overexpression leads to Mcl1 amplification and that this can be exploited as a synthetic lethality by using Mcl1 inhibitors in both mouse models of breast cancer and patient-derived xenografts.

Overall, the manuscript is very well written, the experiments are well designed and controlled, and the models developed in the paper will undoubtedly contribute to studying novel drivers in models of breast cancer. Although several of the observations in the manuscript do not come as a major surprise (e.g. Myc overexpression leads to more aggressive tumors), this is the first formal proof for several of such predictions in immunocompetent models of TNBC and as such is a valuable study. In this regard, and considering the vast amount of work already done, this manuscript would only need minor changes to be ready for publication.

We would like to thank Reviewer 1 for the appreciation of the solidity our work and its relevance.

Below is a list of the minor concerns:

-The authors use oncogene overexpression to test their contribution to cancer initiation and progression. Although I’m aware this is currently the most reasonable surrogate for such experiments, the authors should assess the levels of Myc or Met achieved by CNA (e.g. in the subset of WB1P tumors that gain chromosome 15) compared to the overexpression systems and comment about this potential drawback in the discussion.

- We agree with the Reviewer that it would be important to compare the expression levels achieved in tumors that spontaneously develop amplification of an endogenous gene with those achieved with our cDNA overexpression systems. To this end, we performed immunoblot analysis of MYC expression in spontaneous WB1P tumors with or without amplification of chromosome 15, tumors from B1P-Myc mice injected with Lenti-Cre, and

tumors from B1P mice injected with Lenti-MycP2ACre. Although the spontaneous amplification of chromosome 15 in WB1P tumors results in an increase of endogenous *Myc* expression, both our overexpression systems result in higher levels of MYC. We have included these results in a new figure panel (Supplementary Figure 3B of the revised manuscript). The *invCAG-Myc-IRES-Luc* allele, which was knocked-in in the *Col1a1* locus, drives MYC expression from a very active CAG promoter upon Cre-mediated recombination, while in the Lenti-MycP2ACre vector the *Myc* cDNA is controlled by an even stronger spleen focus forming virus (SFFV) promoter. The expression levels of MYC provided by both these promoters seem to exceed the MYC levels resulting from amplification of the endogenous *Myc* locus in WB1P tumors. We have commented about this potential drawback in the discussion. In future studies, we plan to test additional promoters in our lentiviral constructs, in order to fine-tune the expression levels of the cDNAs that we want to somatically express in the mouse mammary gland. Moreover, we plan to explore the possibility to alter the activity of the endogenous *Myc* promoter by somatic CRISPRa approaches.

-In line with the previous point, the authors should comment on the possibility that the reduced CNA number in WB1P-Myc (Figure 3C) tumors could also be explained by negative selection of cells with both high levels of *Myc* and CNAs. Although not strictly necessary, if possible the authors could analyze the levels of CNA burden and *Myc* protein change over time in the WB1P-Myc model.

- In the discussion of the revised manuscript we mention the possibility that the simplification of the copy-number profile of WB1P-Myc tumors (compared to WB1P tumors) could also be due to negative selection of *Myc*-overexpressing cells with high levels of CNAs.

-To test the requirement of Mcl1 amplification as a permissive factor for *Myc* amplification, authors could suppress Mcl1 function (e.g. CRISPR of Mcl1, or treatment with S63845) and test whether this intervention precludes the amplification of *Myc*.

- The experiment proposed by Reviewer 1 is elegant and informative but beyond the scope of the present study, as it would require a long time to age WB1P mice in which MCL1 function is suppressed, in order to observe whether *Myc* amplification in the resulting tumors is precluded. Moreover, chronic suppression of MCL1 function might be toxic to mice. Alternatively, MCL1 suppression might protect mice from tumorigenesis. In support of the

latter, it was previously shown by the group of Andreas Strasser (Xiang *et al.*, 2010) that development of MYC-driven acute myeloid leukemia (AML) in mice is blocked by *Mcl1*-haploinsufficiency and can be rescued by expression of *Bcl2*. Notably, *Mcl1*-heterozygous bone marrow cells fail to maintain high *Myc* expression levels, suggesting that these cells depend on MCL1 for protection from MYC-induced apoptosis. Similarly, inducible Cre-mediated deletion of even a single *Mcl1* allele substantially impairs the growth of MYC-driven mouse lymphomas (Kelly *et al.*, 2014).

- To test whether MCL1 is required for sustained MYC expression in our model, we derived organoids from a WB1P-Myc mammary tumor and performed shRNA-mediated knockdown of endogenous *Mcl1*, followed by immunoblot analysis of MYC and MCL1 expression. Similar to Xiang *et al.*, *Mcl1*-depleted WB1P-Myc organoids failed to maintain high *Myc* expression levels. We have included these results in a new figure panel (Supplementary Figure 7E of the revised manuscript). Notably, reduced cell growth was observed during this experiment upon shRNA-mediated *Mcl1* knockdown in WB1P-Myc organoids, in line with the results of our shRNA dropout assay (Figure 4A).

-Chromosome 1p amplifications in humans tend to be quite broad and it seems unlikely that it is the only relevant driver. While the authors do a thorough job in implicating Mcl-1 as a contributor, they may wish to mention that there may be others to avoid giving the impression that 1p (mouse 3) gain = Mcl1 amplification.

- We agree with the Reviewer that other relevant driver genes may reside on human chromosome 1q (mouse chromosome 3), a broad region which has been indeed shown to encompass multiple oncogenes (Orsetti *et al.*, 2006). We mentioned this in the revised discussion and we adjusted the text to avoid readers to conclude that *Mcl1* is the only driver in this region.

-The authors should discuss the specificity (and potential off target effects) of the Mcl-1 antagonist they use in their study (S63845).

- We agree with the Reviewer that it is important to discuss the specificity of S63845. The BH3-mimetic MCL1 antagonist S63845 was identified by an NMR-based fragment screen followed by structure-guided drug discovery (Kotschy *et al.*, 2016). S63845 binds to human MCL1 with a K_d of 0.19 nM, and to mouse MCL1 with a 6-fold lower affinity. However, in contrast to other BH3-mimetics, it does not display detectable binding to other anti-apoptotic family members

like BCL2 or BCL-XL. S63845 disrupts binding of BAK and BAX to MCL1, but not to BCL2 and BCL-XL. Moreover, S63845 was shown to be ineffective in BAX and BAK-deficient cancer cells, demonstrating that its killing effect occurs via its on-target activity. While it cannot be excluded that S63845 might have unknown off-target effects, it seems likely that its pro-apoptotic effects are mediated by MCL1-targeting. To highlight the specificity of S63845, we added one sentence in the Results section of the revised manuscript.

-The authors should consider citing a recent paper from Cambell/Blyth (Cell Death Disease 2018), which seems to support the role of Mcl1 as a driver and target in human breast cancer.

- We would like to thank Reviewer 1 for pointing out this recent paper, which shows strong requirement for MCL1 in breast tumorigenesis. We included a citation to this publication in the Discussion section of the revised manuscript.

Reviewer #2, expert in breast cancer mouse models and bioinformatics-

BRCA1 mutation is one the major genetic pre-dispositions for breast cancer development. BRCA1 mutation breast cancer usually belongs to triple-negative breast cancer subtype, which has no effective treatment at the moment. It is thus of great public interest to further analyze the cooperative genetic events leading to this type of cancer. The authors tried to identify the cancer driver genes collaboratively working with BRCA1 mutant. Targeting the proteins coded by these genes simultaneously would in theory generating better outcomes than individual agent targeting only one specific protein or pathway. The authors eventually discovered that in BRCA1 mutated tumors, Myc amplification or over-expression leads to less DNA copy number alterations but is strongly associated with MCL1 amplification. Thus, PARP1 inhibitor (approved for BRCA1 mutant breast cancer patients) and MCL1 inhibitor synergistically delayed tumor growth in mouse models. This is an overall interesting and strong mouse modeling study, with utilization of quite a few in vivo genetically engineered mouse models and novel combination of GEMM with lentiviral infection method (for non-germline gene expression manipulation). The discovered co-amplification of MCL1 and MYC is also very interesting.

We would like to thank Reviewer 2 for the appreciation of our work and for pointing out the important advance of our work for the readership of *Nature Communications*.

I have a few minor questions that I would like the authors to address:

1. Since the major finding is that MYC and MCL1 are co-driver genes for BRCA1 mutation containing breast cancer, can the authors analyze the human breast cancer patient samples, and present the amplification map for these three genes similarly as in Fig. 1A. This will significantly enhance the cancer relevance of the discovery.

- We agree with the Reviewer that adding this information would enhance the relevance of our findings. We therefore generated a new oncoplot that summarizes the deleterious mutations and copy-number events of *TP53*, *MYC* and *MCL1* in 80 *BRCA1*-mutated human breast tumor samples from four large-scale tumor-sequencing studies. This plot has been included in Supplementary Figure S8C of the revised manuscript.

2. In Figure 2C, I am surprised that both B1P (Lenti-MycP2Acre) and WB1P (Lenti-Myc) mice develop tumors faster than B1P-Myc mice. Can the authors exam the Myc expression by immunoblotting at early time points when no tumors developed in these mice yet or at late stage when mice already had tumors?

- We thank the Reviewer for pointing this out. As already mentioned in our reply to point 1 of Reviewer 1, we addressed this issue by performing immunoblot analysis of MYC expression in tumors from B1P-Myc mice injected with Lenti-Cre and tumors from B1P mice injected with Lenti-MycP2Acre. We have included these data in Supplementary Figure 3B of the revised manuscript. We found higher MYC expression from the viral construct than from the knock-in allele. As discussed above, the SFFV viral promoter of Lenti-MycP2Acre more potently drives MYC expression than the CAG promoter of the *invCAG-Myc-IRES-Luc* allele. Moreover, it cannot be excluded that multiple copies of Lenti-MycP2Acre could integrate in the genome of the B1P cells that originate the tumors, while only a single copy of the *invCAG-Myc-IRES-Luc* knock-in allele is present in the *Col1a1* locus of B1P-Myc mice. These differences likely account for the accelerated tumor latency of B1P mice injected with Lenti-MycP2Acre. We mentioned this in the Results section.

3. In Figure 4A, the shRNA dropout assay was used to determine which gene(s) was the most important one for tumorigenesis in an organoid assay. However, MCL1 might just be a critical oncogene

that is needed for tumor growth but not specifically related to Myc-driven tumor. A control dropout assay by using WB1P tumor cells will be needed to make sure MCL1 is specifically needed for WB1P-Myc but not WB1P tumor.

- The Reviewer is correct in stating that the oncogenic role of MCL1 is probably not restricted to MYC-driven tumors. Indeed, in our manuscript we show that somatic overexpression of MCL1 (via intraductal injection with Lenti-Mcl1P2ACre) accelerates tumorigenesis not only in B1P-Myc mice but also in B1P mice (Figure 4D). Immunoblot analysis of MYC expression in tumors from B1P mice injected with Lenti-Mcl1P2ACre did not detect any increased MYC levels compared to tumors from B1P mice injected with Lenti-Cre, suggesting that the somatic overexpression of MCL1 does not necessarily select for amplification of *Myc* (see Additional File for the Reviewers). Another example of the oncogenic role of MCL1 in a tumor that is not driven by MYC comes from the recent paper from Campbell *et al.*, 2018, where MCL1 seems to be required for mammary tumor development in the MMTV-PyMT mouse model.

Reviewer #3 (Remarks to the Author):

Jonkers and colleagues describe new in vivo systems to determine driver events in BRCA1 mutant mammary gland cancers. This entails development of methods for Cas9 mediated loss of function or c-myc overexpression events in combination with BRCA1 and p53 mutation. The work provides proof of concept that candidate driver genes can be more rapidly assessed than by conventional breeding schemes. This in itself represents a significant advance for the field. The results of the study are also notable for strong cooperation between c-Myc or Met oncogene overexpression dramatically reducing tumor latency in the context of BRCA1 mutation. PTEN or RB loss also produced more rapid tumor development. While these results are not entirely surprising, they provide important validation and also result in the unanticipated finding that c-Myc overexpression simplifies the CNA changes observed in B1P tumors. This facilitated identification of MCL1 as a driver change in B1P Myc tumors and that MCL1 inhibition + PARPi significantly delayed or c-myc overexpression tumor formation.

This is an important study and will be of broad interest considering its methodologic advances and the medical implication to BRCA1 mutated cancers. It does suffer somewhat from being diffuse and not going more in depth into the results of its many findings. Additional controls are missing to determine if MCL1 cooperation is mainly through Myc or has any specific relationship to BRCA1. This reduces the quality of the study. Nonetheless, the work is valuable and should be published upon some additional changes to the text.

We would like to thank Reviewer 3 for appreciating our work and for pointing out the general relevance of the methodological advances we describe.

Specific comments

1. Fig 2A-C. Myc overexpression dramatically reduces tumor latency in the B1P background. However, we don't know if it actually cooperates with BRCA1 mutation. What would the kinetics of tumor development be in Myc, p53 mice? Is this data purely driven by Myc and BRCA1 mutation merely a passenger that is affecting CNA.

- We agree with Reviewer 3 that it would be interesting to look at the tumor latency of mice that overexpress MYC and lack p53 but retain *Brca1* expression. For this reason, we included the Kaplan-Meier curve of the mammary tumor free survival of 20 *WapCre;Brca1^{F/+};Trp53^{F/F};Col1a1^{invCAG-Myc-IRES-Luc/+}* female mice with heterozygous *Brca1^F* alleles in Supplementary Figure 2C of the revised manuscript. These animals developed mammary tumors slightly but significantly slower ($p < 0.001$) than *WapCre;Brca1^{F/F};Trp53^{F/F};Col1a1^{invCAG-Myc-IRES-Luc/+}* mice with homozygous *Brca1^F* alleles, supporting a cooperative role of BRCA1 loss in WB1P-Myc tumors.

2. Again, McCl1 inhibition may be primarily working against Myc rather than BRCA1. There is indeed substantial literature to show cooperation between MCL1 And c-Myc. Are the authors really providing new information with their data?

- We agree with the Reviewer that the cooperation between MCL1 and MYC in tumorigenesis has been reported before (Xiang *et al.*, 2010; Allen *et al.*, 2011). However, we report for the first time on the relevance of this interaction in the context of BRCA1-associated mammary tumorigenesis. Moreover, we demonstrate strong synergy between a PARP inhibitor and an MCL1 antagonist in a patient-derived xenograft (PDX) model of *BRCA1*-mutated breast cancer, a novel finding that warrants future investigation and that might benefit a fraction of *BRCA1*-mutated breast cancer patients with poor response to PARP inhibition.

3. Fig 4G. Can the authors explain why the combination of PARPi and MCL1i is not curative?

- The fact that the combination of olaparib and S63845 is not curative in our PDX setting might be due to several reasons. We don't know if complete inhibition of MCL1 was achieved with the intermittent dosing schedule we employed, and this will warrant future investigation and optimization. Moreover, there might be heterogeneity within the PDX tumor in the response to both drugs. Finally, even with an optimal inhibitor and dosing schedule, treatment could select for tumor cells with acquired resistance to the combination treatment, i.e. for cells that upregulate other anti-apoptotic effectors than MCL1.

4. Fig S8 MCL inhibitor S63845 has minimal additive efficacy over olaparib alone in WB1P-Myc tumors. Significance is not indicated

- We thank the Reviewer for noticing that this information was missing. In the revised version of Supplementary Figure S8 we indicated significance in the Figure legend.

REVIEWERS' COMMENTS:

Reviewer #1 (Remarks to the Author):

The authors have addressed all concerns and the current form of this manuscript is suitable for publication

Reviewer #2 (Remarks to the Author):

The authors have now addressed all my three questions.

1) In BRCA1 mutant patients, the majority of Myc amplification patients do have Mcl1 amplification.

2) Myc expression level is provided in these mouse models and explained that the reduced tumor latency in B1P and WB1P is actually caused by higher expression level of Myc in these experimental models.

3) Mcl1 could potentially works with other genetic events rather than Myc amplification.

REVIEWERS' COMMENTS

Reviewer #1 (Remarks to the Author):

The authors have addressed all concerns and the current form of this manuscript is suitable for publication

Reviewer #2 (Remarks to the Author):

The authors have now addressed all my three questions.

- 1) In BRCA1 mutant patients, the majority of Myc amplification patients do have Mcl1 amplification.
- 2) Myc expression level is provided in these mouse models and explained that the reduced tumor latency in B1P and WB1P is actually caused by higher expression level of Myc in these experimental models.
- 3) Mcl1 could potentially works with other genetic events rather than Myc amplification.

Reviewer #3 (Remarks to the Author):

The authors have satisfactorily addressed the majority of my concerns. I am in favor of publication in Nature Comm

We would like to thank the three reviewers for taking the time to read our manuscript again. We are happy that we could address all concerns raised and we believe that the revised manuscript is significantly improved thanks to their constructive remarks.