# Supplementary material for: Comparative oncogenomics identifies combinations of driver genes and drug targets in BRCA1-mutated breast cancer
Source: Nat Commun. 2019 Jan 23;10:397. doi: 10.1038/s41467-019-08301-2 (PMC6344487; doi:10.1038/s41467-019-08301-2)
Supplement: Supplementary file 1 — Supplementary Information [file 41467_2019_8301_MOESM1_ESM.pdf]

Comparative oncogenomics identifies combinations of driver genes and drug targets in *BRCA1*-mutated breast cancer

Stefano Annunziato, Julian R de Ruiter, Linda Henneman, Chiara S Brambillasca *et al.*

Supplementary Information

Supplementary Figure 1

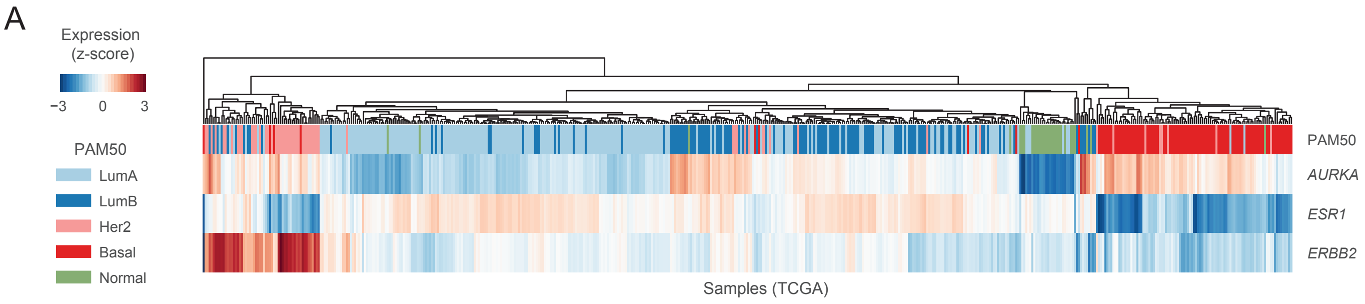

Supplementary Figure 1. Gene expression analysis of human breast cancer samples. (A) Unsupervised clustering (Euclidean distance, average linkage) of the human breast cancer samples from TCGA, using a three-genes signature that distinguishes the PAM50 subtypes (26). LumA, luminal A; LumB, luminal B.

Supplementary Figure 2

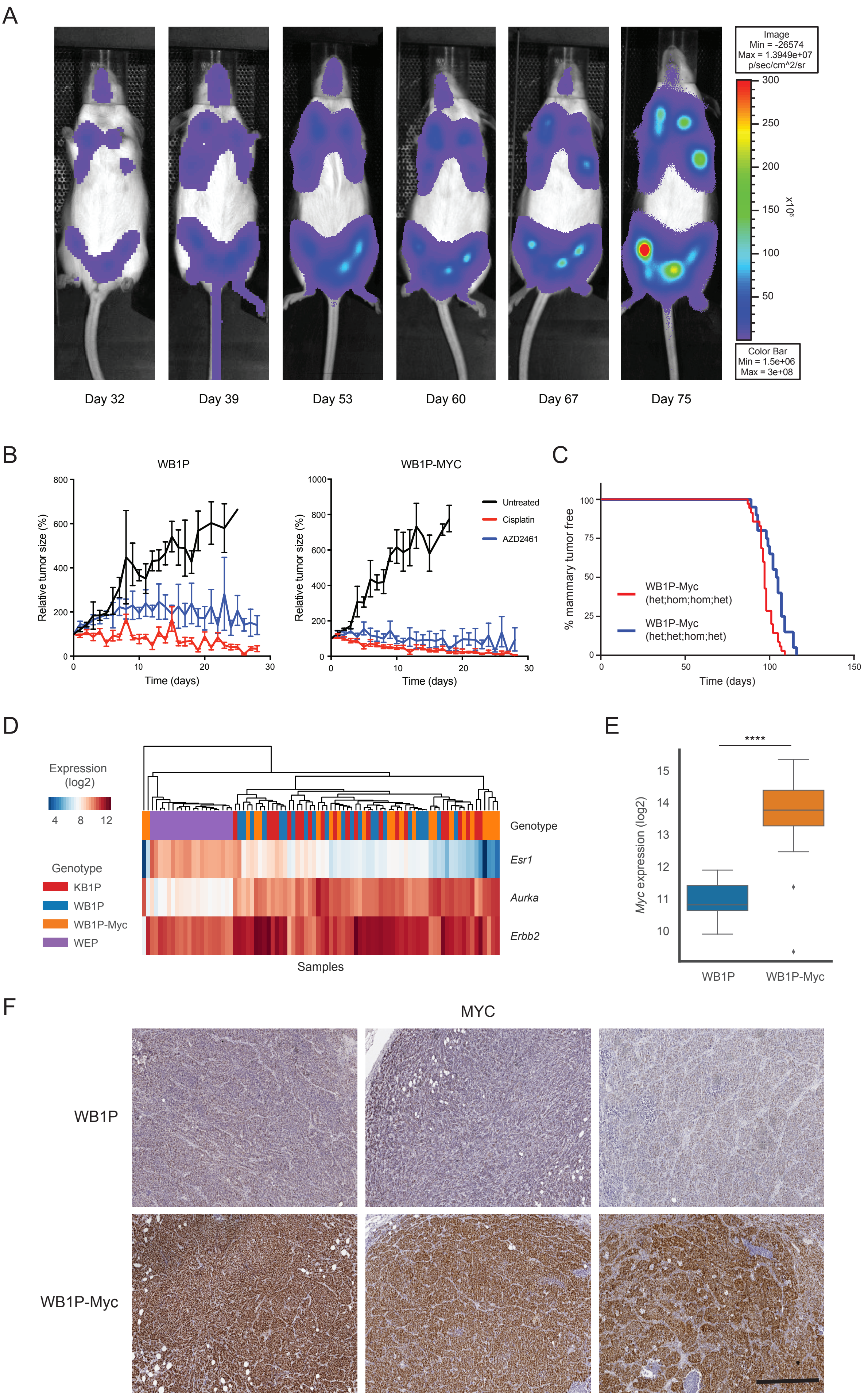

Supplementary Figure 2. Characterization of the WB1P-Myc mouse model. (A) Longitudinal *in vivo* bioluminescence imaging of luciferase expression in a WB1P-Myc female, showing signal build-up over time. **(B)** Response of WB1P and WB1P-Myc tumors to cisplatin and PARP inhibitors, as visualized by tumor volume curves. Small fragments of WB1P and WB1P-Myc tumors were transplanted in the fourth mammary fat pad of nude mice (*n*=36 per donor). When tumors had reached a size of 100 mm^3, mice were treated with 6 mg kg^{-1} cisplatin (administered i.v. on day 0 and day 14), 100 mg kg^{-1} AZD2461 (administered daily by per os, for 28 consecutive days) or vehicle. Error bars represent standard error of the mean. **(C)** Kaplan-Meier curves showing mammary tumor-specific survival for the different genotypes. *WapCre;Brca1$^{F/F}$;Trp53$^{F/F}$;Col1a1$^{invCAG\text{-}Myc\text{-}IRES\text{-}Luc/+}$* (WB1P-Myc*)* females (*n=35*) showed a reduced mammary tumor-specific survival compared to *WapCre;Brca1$^{F/+}$;Trp53$^{F/F}$;Col1a1$^{invCAG\text{-}Myc\text{-}IRES\text{-}Luc/+}$* (*n*=20) littermates (97 days vs 105 days; ****P* < 0.001 by Mantel-Cox test). **(D)** Unsupervised clustering (Euclidean distance, average linkage) of WB1P and WB1P-Myc tumors with tumors derived from published mouse models of luminal (*WapCre;Cdh1$^{F/F}$;Pten$^{F/F}$*, WEP) and basal-like (*K14Cre;Brca1$^{F/F}$;Trp53$^{F/F}$,* KB1P) breast cancer, using a three-genes signature that distinguishes the PAM50 subtypes (26). **(E)** *Myc* expression levels in WB1P and WB1P-Myc tumors; *****P* < 0.0001 (two-sided Mann-Whitney U test). Boxes extend from the third (Q3) to the first (Q1) quartile (interquartile range, IQR), with the line at the median; whiskers extend to Q3 + 1.5 × IQR and to Q1 − 1.5 × IQR. Points beyond the ends of the whiskers are outliers. **(F)** Immunohistochemical detection of MYC in multiple independent WB1P and WB1P-Myc tumors. Bar, 400 µM.

Supplementary Figure 3

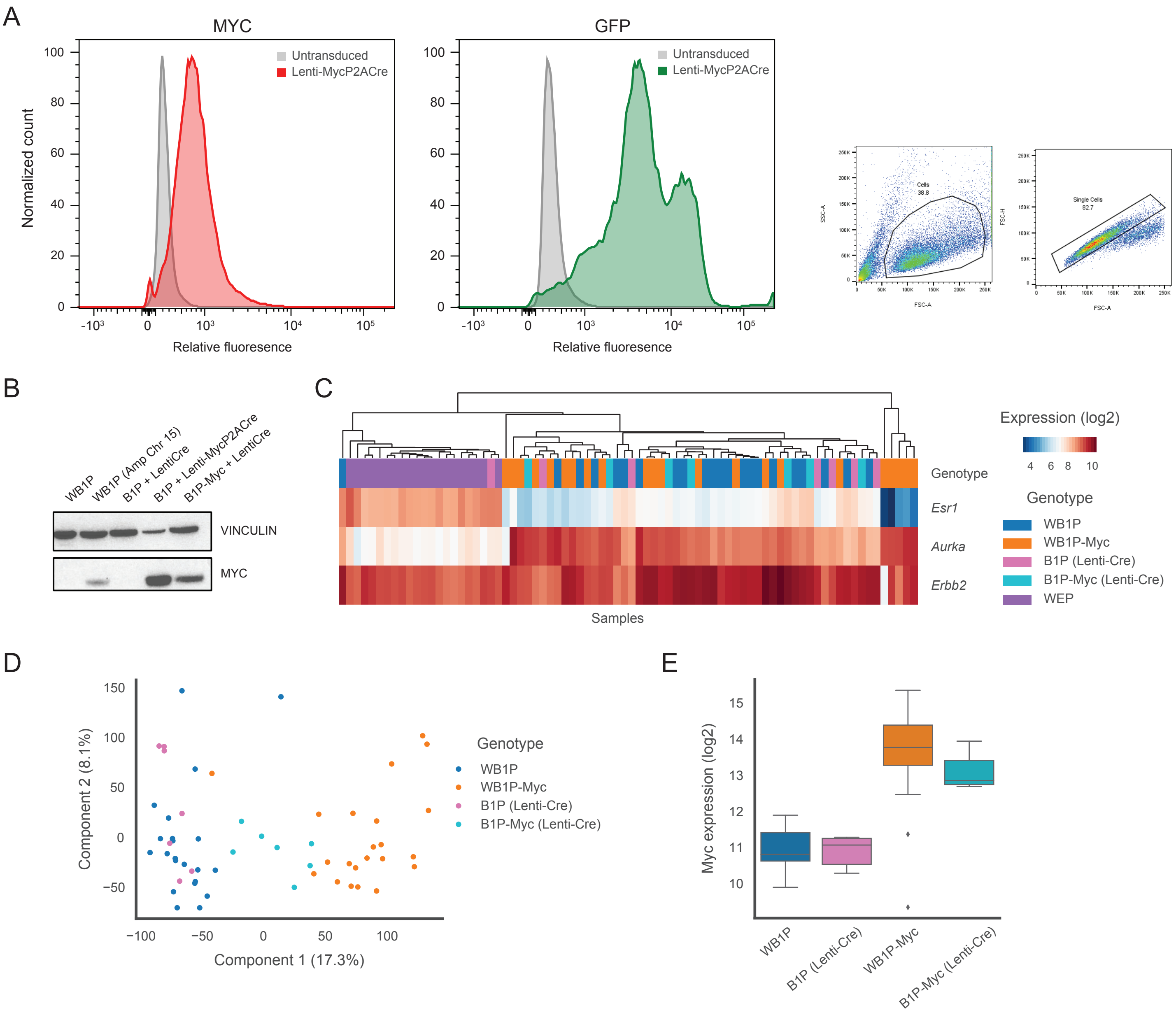

Supplementary Figure 3. Non-germline models with *Myc* overexpression. (A) *In vitro* validation of Lenti-MycP2ACre in Cre-reporter cells (containing a lox-STOP-lox-GFP cassette) 5 days after transduction. Expression of MYC as visualized by FACS using an anti-MYC antibody and FACS analysis of Cre-recombined GFP-positive cells are shown. A figure exemplifying the gating strategy is provided on the right. **(B)** Expression of MYC in independent tumors as visualized by immunoblotting using anti-MYC antibody. Sample order**:** WB1P tumor without chromosome 15 amplification; WB1P tumor with chromosome 15 amplification; tumor from B1P mouse injected with Lenti-Cre; tumor from B1P mouse injected with Lenti-MycP2ACre; tumor from B1P-Myc mouse injected with Lenti-Cre. **(C)** Unsupervised clustering (Euclidean distance, average linkage) of the tumors from germline and somatic models using the three-genes PAM50 signature (26), showing that tumors from the somatic models retain the basal expression profiles observed in tumors from the germline models. **(D)** PCA plot comparing WB1P and WB1P-Myc tumors to tumors from the non-germline models (Lenti-Cre injected B1P and B1P-Myc mice) using global gene expression profiles. **(E)** *Myc* expression levels in tumors from germline and non-germline models. Boxes extend from the third (Q3) to the first (Q1) quartile (interquartile range, IQR), with the line at the median; whiskers extend to Q3 + 1.5 × IQR and to Q1 − 1.5 × IQR. Points beyond the ends of the whiskers are outliers.

Supplementary Figure 4

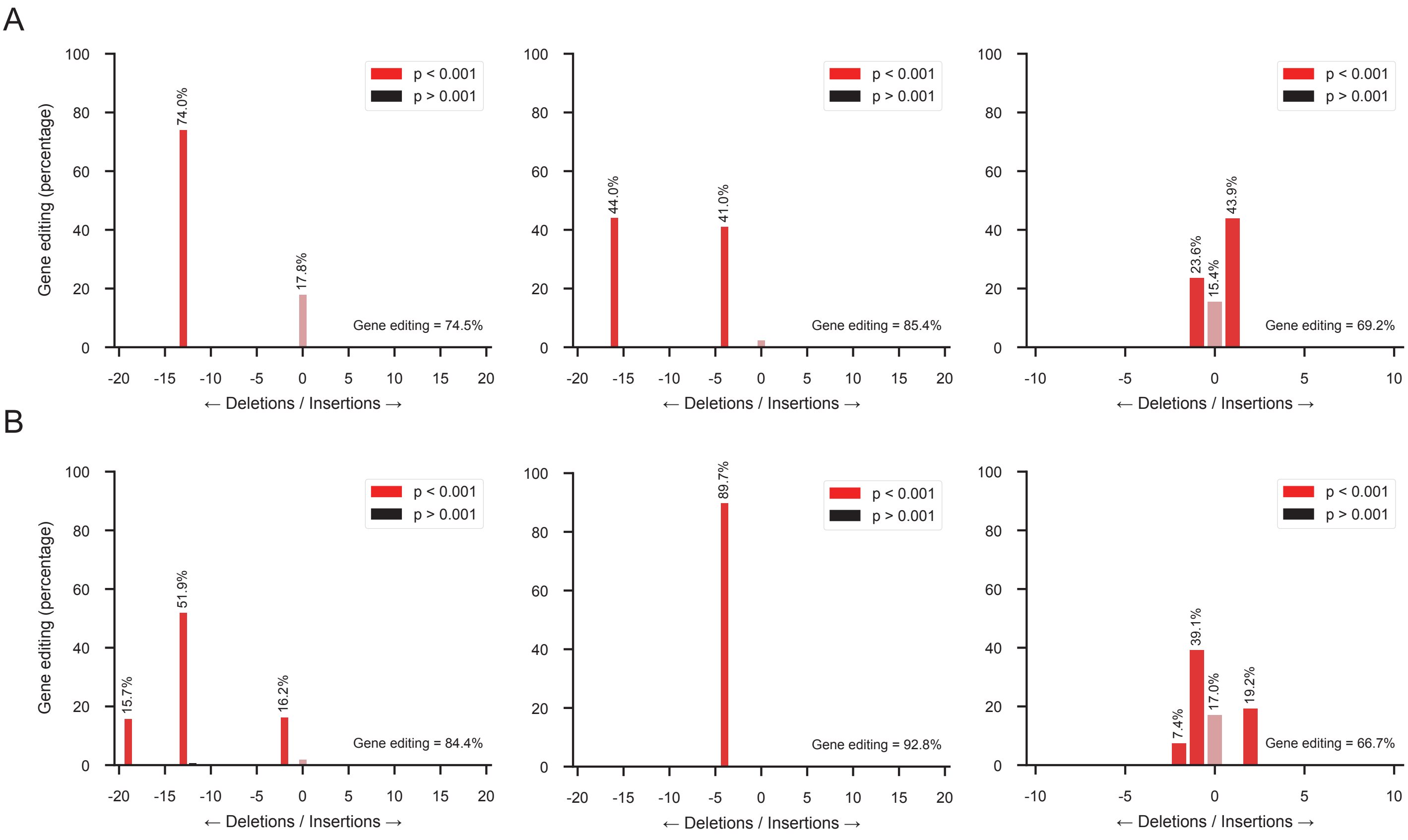

Supplementary Figure 4. CRISPR-mediated somatic gene disruption of *Rb1* and *Pten*. (A-B) TIDE analysis showing the spectrum of insertions/deletions (indels) of the targeted *Rb1* **(A)** and *Pten* **(B)** alleles in multiple independent tumors from WB1P-Cas9 mice injected with Lenti-sg*Rb1*-Myc and Lenti-sg*Pten*-Myc, respectively.

Supplementary Figure 5

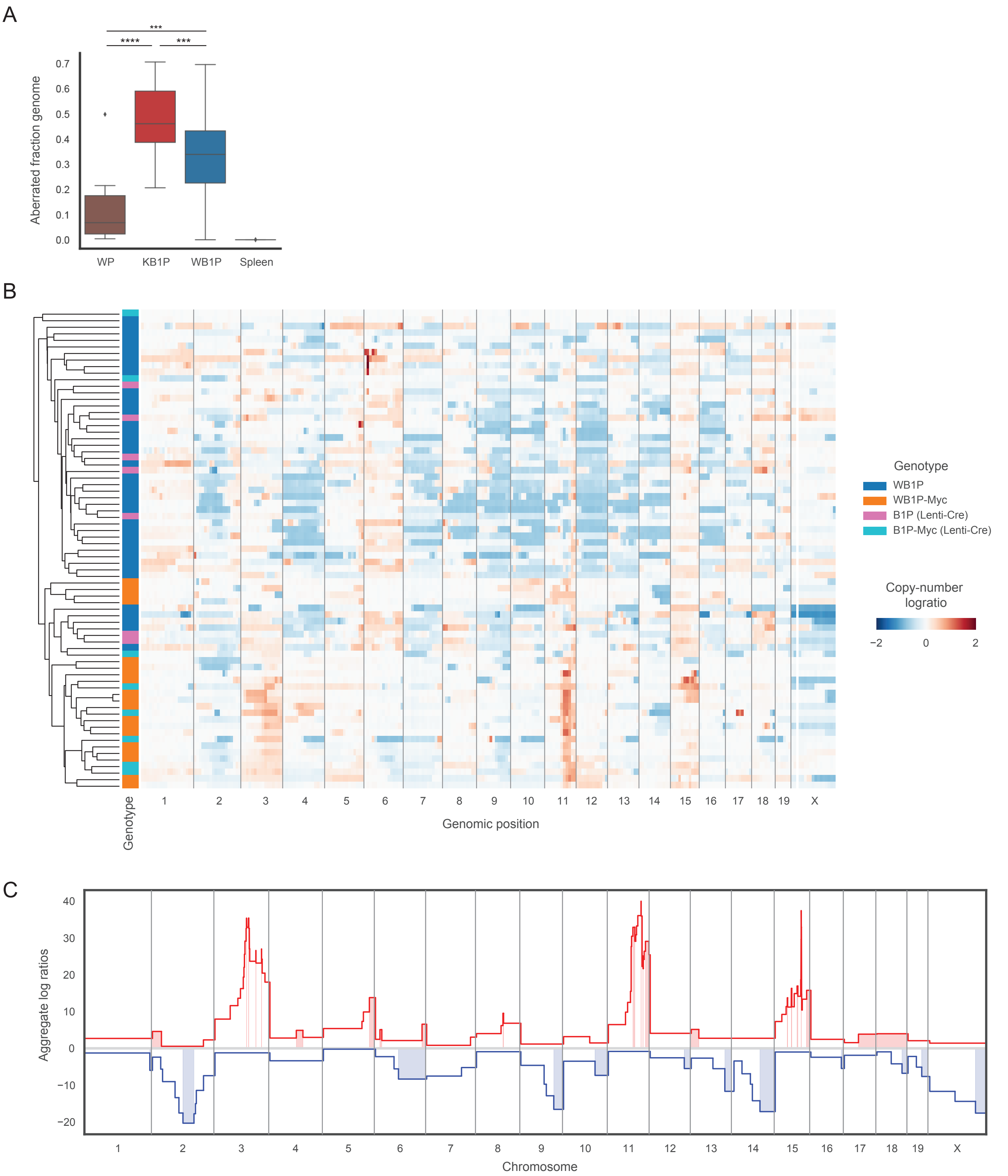

Supplementary Figure 5. Genomic instability of WB1P and WB1P-Myc tumors. (A) Genomic instability scores of WP, WB1P and KB1P tumors; ***P < 0.001 and ****P < 0.0001 (two-sided Mann-Whitney U test). Scores for spleen samples from WB1P mice are shown as reference. Boxes extend from the third (Q3) to the first (Q1) quartile (interquartile range, IQR), with the line at the median; whiskers extend to Q3 + 1.5 × IQR and to Q1 − 1.5 × IQR. Points beyond the ends of the whiskers are outliers. **(B)** Unsupervised clustering (correlation distance, average linkage) of the tumors from somatic and germline models based on their copy-number profiles. Tumors from the somatic models mainly cluster together with their germline counterparts, demonstrating that these tumors have similar patterns of copy-number aberrations. **(C)** Overview of the recurrently aberrated regions identified by RUBIC in the combined set of MYC-driven tumors from the germline and somatic models.

Supplementary Figure 6

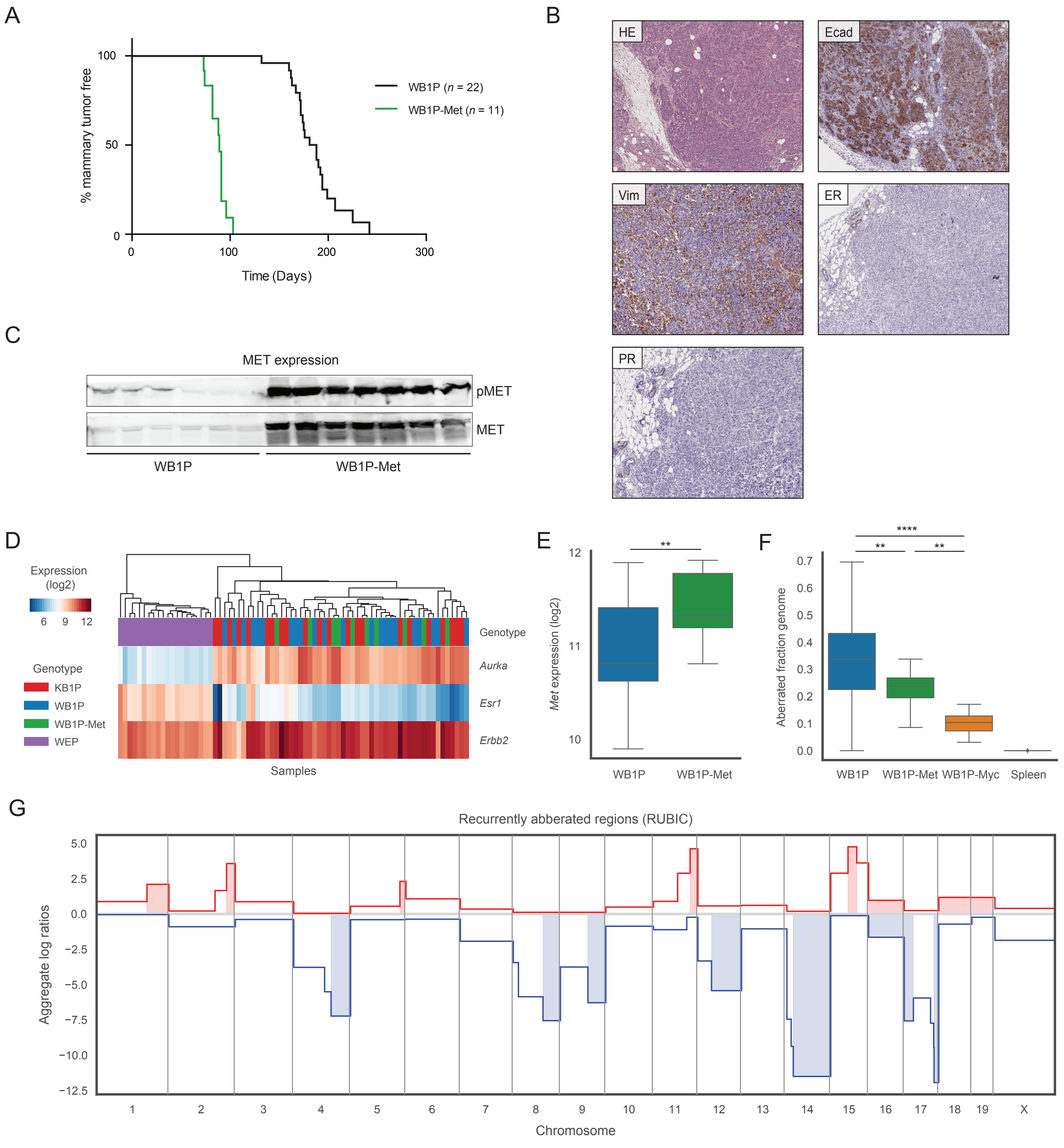

Supplementary Figure 6. Characterization of the WB1P-Met mouse model. (A) Kaplan-Meier curves showing mammary tumor-specific survival for the different genotypes. *WapCre;Brca1*$^{F/F}$*;Trp53*$^{F/F}$*;Col1a1*$^{invCAG\text{-}Met\text{-}IRES\text{-}Luc/+}$ (WB1P-Met) females showed a reduced mammary tumor-specific survival compared to WB1P littermates (89 days vs 188 days; ****P < 0.0001 by Mantel-Cox test). **(B)** Representative hematoxylin and eosin (HE) staining and immunohistochemical detection of E-cadherin, vimentin, ER and PR in WB1P-Met tumors. **(C)** Expression and activity of MET in independent tumors from WB1P and WB1P-Met mice, as visualized by immunoblotting using anti-MET and anti-phosphoMET antibodies. **(D)** Unsupervised clustering (Euclidean distance, average linkage) of WB1P and WB1P-Met tumors with tumors derived from published mouse models of luminal (*WapCre;Cdh1*$^{F/F}$*;Pten*$^{F/F}$, WEP) and basal-like (*K14Cre;Brca1*$^{F/F}$*;Trp53*$^{F/F}$, KB1P) breast cancer, using a three-genes signature that distinguishes the PAM50 subtypes (26). **(E)** *Met* expression levels in WB1P and WB1P-Met tumors; **P < 0.01 (two-sided Mann-Whitney U test). Boxes extend from the third (Q3) to the first (Q1) quartile (interquartile range, IQR), with the line at the median; whiskers extend to Q3 + 1.5 × IQR and to Q1 − 1.5 × IQR. **(F)** Genomic instability of WB1P, WB1P-Met and WB1P-Myc tumors; **P < 0.01 and ****P < 0.0001 (two-sided Mann-Whitney U test). Scores for spleen samples from WB1P mice are shown as reference. **(G)** Genome-wide RUBIC analysis of the CNV profiles of WB1P-Met tumors. Significant amplifications and deletions are marked by light red and blue columns, respectively.

Supplementary Figure 7

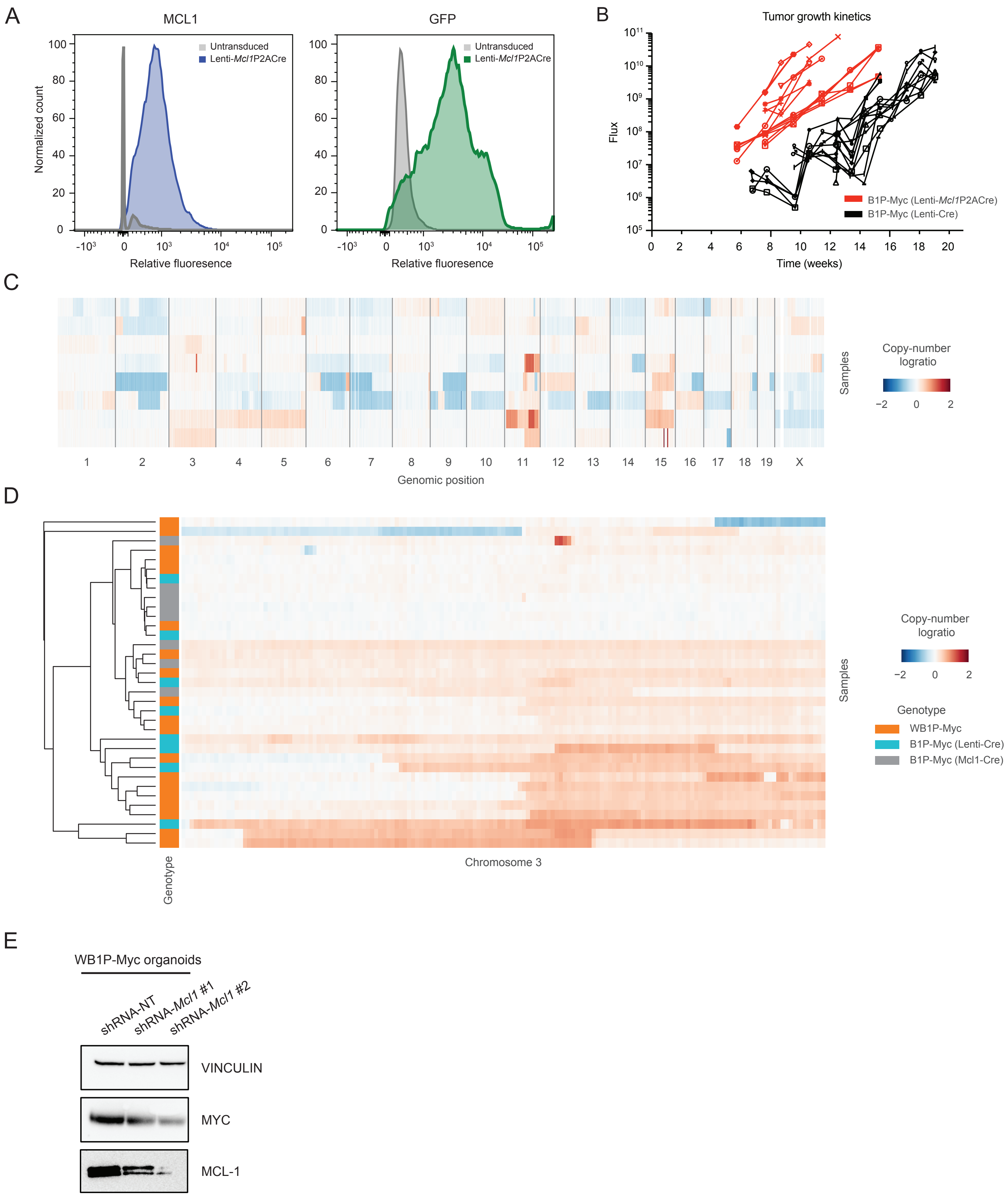

Supplementary Figure 7. Validation of MCL1 as a driver in BRCA1-associated TNBC. (A) *In vitro* validation of Lenti-Mcl1P2ACre in Cre-reporter cells 5 days after transduction. Expression of MCL1 as visualized by FACS using an anti-MCL1 antibody and FACS analysis of Cre-recombined GFP-positive cells are shown. **(B)** Longitudinal *in vivo* bioluminescence imaging of luciferase expression in B1P-Myc animals injected with Lenti-Cre (black lines) or Lenti-Mcl1P2ACre (red lines), showing signal build-up over time. **(C)** Heatmap showing the copy-number logratios for tumors from B1P-Myc females injected with Lenti-Mcl1P2ACre, showing that recurrent gains on chromosomes 11 and 15 are retained, whilst the gain on chromosome 3 is less pronounced. **(D)** Unsupervised clustering (Euclidean distance, average linkage) of MYC-driven tumors from germline and somatic models (WB1P-Myc tumors and tumors from Lenti-Cre injected B1P-Myc mice) together with tumors from B1P-Myc females injected with Lenti-Mcl1P2ACre. The clustering shows that the focal gain surrounding *Mcl1* is lost in the majority of tumors from Lenti-Mcl1P2ACre injected B1P-Myc females. **(E)** Expression of MYC and MCL1 in WB1P-Myc organoids transduced with a non-targeting shRNA or with two independent sh*Mcl1* vectors, as visualized by immunoblotting using anti-MYC antibody 5 days after transduction.

Supplementary Figure 8

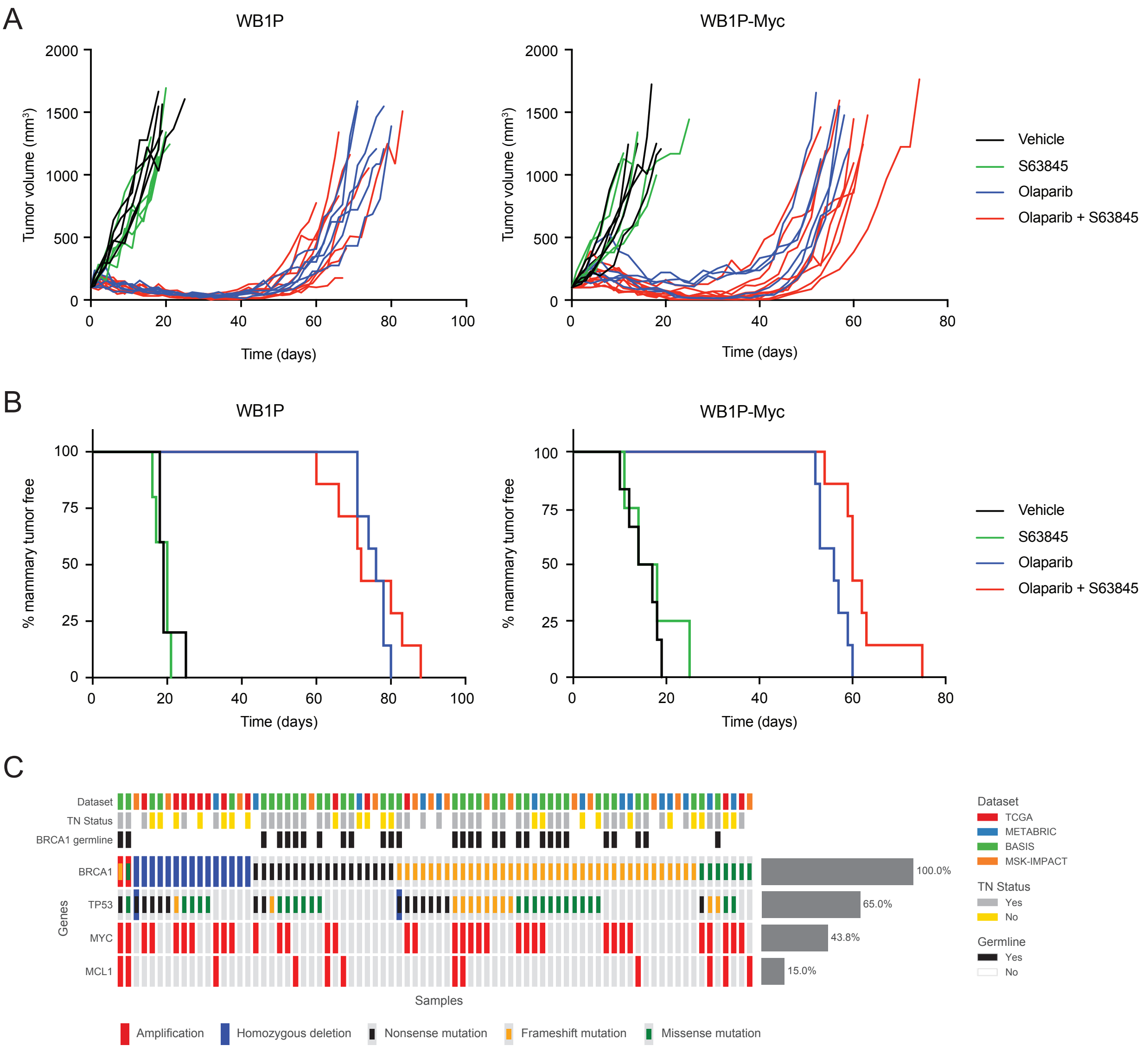

Supplementary Figure 8. Treatment of WB1P and WB1P-Myc tumors with MCL1 and PARP inhibitors. (A-B) Response of organoid-derived WB1P and WB1P-Myc tumors to MCL1 and PARP inhibitors, as visualized by tumor volume curves (**A**) and Kaplan Meier curves (**B**). WB1P and WB1P-Myc organoid lines were transplanted in the fourth mammary fat pad of nude mice (*n*=24 per donor). When tumors had reached a size of 100 mm^3, mice were treated with 25 mg kg^{-1} S63845 (administered i.v. once weekly for 5 weeks), 100 mg kg^{-1} olaparib (administered i.p. daily for 28 consecutive days), both drugs or vehicle. S63845 shows minimal additive efficacy over olaparib alone in WB1P-Myc tumors (*$P < 0.05$ by Mantel-Cox test). **(C)** Overview of the mutations and copy-number events in our panel of 80 *BRCA1*-mutated human breast tumor samples for *TP53*, *MYC* and *MCL1*.

Supplementary Table 1. Mutational landscape of human *BRCA1*-mutated TNBC. Deleterious mutations and copy-number events in 80 *BRCA1*-mutated human breast tumor samples from four large-scale tumor-sequencing studies.

sample	gene	type	value	dataset	mutation
TCGA-E9-A1NC	BRCA1	mutation	nonsense	TCGA	p.Q139*
TCGA-LL-A5YP	BRCA1	mutation	missense	TCGA	p.D96H
TCGA-AN-A0XU	BRCA1	mutation	missense	TCGA	p.G1788V
TCGA-A2-A25B	BRCA1	mutation	nonsense	TCGA	p.E720*
TCGA-D8-A27M	BRCA1	mutation	frameshift	TCGA	p.P1614Qfs*19
MB-0346	BRCA1	mutation	missense	METABRIC	p.R1737T
MB-0420	BRCA1	mutation	frameshift	METABRIC	p.V1654Cfs*4
MB-5070	BRCA1	mutation	frameshift	METABRIC	p.T150Pfs*13
MB-5107	BRCA1	mutation	frameshift	METABRIC	p.E1494Kfs*11
MB-5323	BRCA1	mutation	frameshift	METABRIC	p.E880Rfs*13
MB-5465	BRCA1	mutation	frameshift	METABRIC	p.D560Efs*6
MB-6060	BRCA1	mutation	nonsense	METABRIC	p.S282*
MB-6098	BRCA1	mutation	missense	METABRIC	p.D1692N
MB-6271	BRCA1	mutation	frameshift	METABRIC	p.L502Sfs*2
MB-7032	BRCA1	mutation	frameshift	METABRIC	p.K1667Qfs*11
MB-7038	BRCA1	mutation	frameshift	METABRIC	p.Q202Kfs*32
MB-7048	BRCA1	mutation	frameshift	METABRIC	p.F709Sfs*29
MTS-T0064	BRCA1	mutation	nonsense	METABRIC	p.Y1463*
P-0010035-T01-IM5	BRCA1	mutation	frameshift	MSK-IMPACT	p.G911Efs*89
P-0002915-T01-IM3	BRCA1	mutation	frameshift	MSK-IMPACT	p.E729Rfs*10
P-0005423-T01-IM5	BRCA1	mutation	frameshift	MSK-IMPACT	p.E23Afs*18
P-0011369-T01-IM5	BRCA1	mutation	frameshift	MSK-IMPACT	p.E686*
P-0007037-T01-IM5	BRCA1	mutation	frameshift	MSK-IMPACT	p.G1371Vfs*4
P-0008994-T01-IM5	BRCA1	mutation	frameshift	MSK-IMPACT	p.R71Kfs*10
P-0006504-T02-IM5	BRCA1	mutation	frameshift	MSK-IMPACT	p.L358Kfs*13
P-0010457-T01-IM5	BRCA1	mutation	nonsense	MSK-IMPACT	p.K1724*
P-0003885-T01-IM5	BRCA1	mutation	nonsense	MSK-IMPACT	p.E648*
P-0002237-T01-IM3	BRCA1	mutation	frameshift	MSK-IMPACT	p.E1219Ffs*12
P-0002858-T01-IM3	BRCA1	mutation	missense	MSK-IMPACT	p.Q1395E
P-0010758-T01-IM5	BRCA1	mutation	frameshift	MSK-IMPACT	p.V1654Cfs*4
PD11742	BRCA1	mutation	frameshift	BASIS	p.F1772fs*29
PD7215	BRCA1	mutation	missense	BASIS	p.D1739V
PD9585	BRCA1	mutation	nonsense	BASIS	p.R1443*
PD7067	BRCA1	mutation	nonsense	BASIS	p.E515*
PD23561	BRCA1	mutation	frameshift	BASIS	p.A224fs*4
PD24202	BRCA1	mutation	frameshift	BASIS	p.V1833fs*7
PD8621	BRCA1	mutation	nonsense	BASIS	p.R1835*
PD13296	BRCA1	mutation	frameshift	BASIS	p.?
PD24337	BRCA1	mutation	nonsense	BASIS	p.E1754*
PD9702	BRCA1	mutation	missense	BASIS	p.R1699W
PD6406	BRCA1	mutation	missense	BASIS	p.D1692N
PD9004	BRCA1	mutation	frameshift	BASIS	p.P1464fs*2
PD3905	BRCA1	mutation	frameshift	BASIS	p.?
PD24186	BRCA1	mutation	frameshift	BASIS	p.Q1447fs*16
PD4005	BRCA1	mutation	frameshift	BASIS	p.I1237fs*27
PD13771	BRCA1	mutation	nonsense	BASIS	p.K1183fs*26

PD13297	BRCA1	mutation	nonsense	BASIS	p.E1060*
PD5935	BRCA1	mutation	nonsense	BASIS	p.E1060*
PD11327	BRCA1	mutation	frameshift	BASIS	p.N1029fs*5
PD10014	BRCA1	mutation	frameshift	BASIS	p.K894fs*8
PD3890	BRCA1	mutation	frameshift	BASIS	p.G834fs*12
PD5930	BRCA1	mutation	frameshift	BASIS	p.D825fs*21
PD8980	BRCA1	mutation	frameshift	BASIS	p.D825fs*21
PD14442	BRCA1	mutation	nonsense	BASIS	p.Q563*
PD4006	BRCA1	mutation	nonsense	BASIS	p.Q563*
PD13299	BRCA1	mutation	frameshift	BASIS	p.K519fs*13
PD4107	BRCA1	mutation	frameshift	BASIS	p.V340fs*6
PD23562	BRCA1	mutation	frameshift	BASIS	p.S308fs*1
PD5945	BRCA1	mutation	frameshift	BASIS	p.V233fs*4
PD22355	BRCA1	mutation	nonsense	BASIS	p.Y130*
PD23574	BRCA1	mutation	nonsense	BASIS	p.Y130*
PD23578	BRCA1	mutation	nonsense	BASIS	p.Y130*
PD6413	BRCA1	mutation	frameshift	BASIS	p.G129fs*34
PD6731	BRCA1	mutation	frameshift	BASIS	p.E23fs*17
PD5948	BRCA1	mutation	frameshift	BASIS	p.?
TCGA-BH-A0AW	TP53	mutation	missense	TCGA	p.F270S
TCGA-AO-A0JL	TP53	mutation	nonsense	TCGA	p.R306*
TCGA-BH-A0C0	TP53	mutation	frameshift	TCGA	p.R110Vfs*13
TCGA-BH-A0C0	TP53	mutation	frameshift	TCGA	p.R110Vfs*13
TCGA-LL-A5YP	TP53	mutation	missense	TCGA	p.R280K
TCGA-C8-A12L	TP53	mutation	missense	TCGA	p.C238F
TCGA-BH-A1FU	TP53	mutation	missense	TCGA	p.R175H
TCGA-D8-A27M	TP53	mutation	nonsense	TCGA	p.R196*
TCGA-E2-A1L7	TP53	mutation	missense	TCGA	p.I255F
MB-0346	TP53	mutation	missense	METABRIC	p.R273C
MB-0420	TP53	mutation	nonsense	METABRIC	p.R306*
MB-5465	TP53	mutation	nonsense	METABRIC	p.R213*
MB-6098	TP53	mutation	frameshift	METABRIC	p.S215Cfs*6
MB-6271	TP53	mutation	missense	METABRIC	p.C135F
MB-7048	TP53	mutation	missense	METABRIC	p.R110L
MTS-T0064	TP53	mutation	missense	METABRIC	p.E285D
MTS-T0064	TP53	mutation	nonsense	METABRIC	p.E286*
P-0010035-T01-IM5	TP53	mutation	frameshift	MSK-IMPACT	p.R110Pfs*14
P-0011369-T01-IM5	TP53	mutation	nonsense	MSK-IMPACT	p.R196*
P-0007037-T01-IM5	TP53	mutation	missense	MSK-IMPACT	p.E224D
P-0008994-T01-IM5	TP53	mutation	missense	MSK-IMPACT	p.Y107D
P-0006504-T02-IM5	TP53	mutation	nonsense	MSK-IMPACT	p.R213*
P-0009557-T01-IM5	TP53	mutation	nonsense	MSK-IMPACT	p.S183*
P-0010457-T01-IM5	TP53	mutation	missense	MSK-IMPACT	p.T125R
P-0002237-T01-IM3	TP53	mutation	nonsense	MSK-IMPACT	p.Q100*
P-0002591-T01-IM3	TP53	mutation	nonsense	MSK-IMPACT	p.Q375*
P-0010758-T01-IM5	TP53	mutation	frameshift	MSK-IMPACT	p.P85Vfs*35
PD3905	TP53	mutation	frameshift	BASIS	p.E343fs*3

PD5948	TP53	mutation	frameshift	BASIS	p.G334fs*12
PD4967	TP53	mutation	nonsense	BASIS	p.Q331*
PD24337	TP53	mutation	missense	BASIS	p.R280K
PD8621	TP53	mutation	missense	BASIS	p.R273G
PD22355	TP53	mutation	missense	BASIS	p.V272M
PD10014	TP53	mutation	missense	BASIS	p.I255T
PD6413	TP53	mutation	missense	BASIS	p.R248Q
PD3890	TP53	mutation	missense	BASIS	p.R248W
PD11742	TP53	mutation	missense	BASIS	p.G245S
PD8980	TP53	mutation	missense	BASIS	p.Y220C
PD4005	TP53	mutation	nonsense	BASIS	p.R213*
PD9585	TP53	mutation	frameshift	BASIS	p.N200fs*9
PD4107	TP53	mutation	frameshift	BASIS	p.R196fs*51
PD13297	TP53	mutation	nonsense	BASIS	p.R196*
PD11327	TP53	mutation	missense	BASIS	p.L194R
PD4826	TP53	mutation	nonsense	BASIS	p.Q192*
PD24202	TP53	mutation	frameshift	BASIS	p.D184fs*62
PD6731	TP53	mutation	missense	BASIS	p.R175H
PD23574	TP53	mutation	missense	BASIS	p.R175H
PD9702	TP53	mutation	missense	BASIS	p.R175G
PD6406	TP53	mutation	frameshift	BASIS	p.A159fs*8
PD5945	TP53	mutation	nonsense	BASIS	p.W146*
PD5935	TP53	mutation	missense	BASIS	p.T125R
PD9004	TP53	mutation	frameshift	BASIS	p.V122fs*26
PD23562	TP53	mutation	frameshift	BASIS	p.E68fs*81
PD7215	TP53	mutation	nonsense	BASIS	p.W53*
TCGA-C8-A12L	PIK3CA	mutation	missense	TCGA	p.H1047R
MB-5107	PIK3CA	mutation	missense	METABRIC	p.K111E
MB-5107	PIK3CA	mutation	missense	METABRIC	p.G320A
MB-6060	PIK3CA	mutation	missense	METABRIC	p.H1047R
MB-7032	PIK3CA	mutation	missense	METABRIC	p.H1047R
P-0002915-T01-IM3	PIK3CA	mutation	missense	MSK-IMPACT	p.E970K
P-0005423-T01-IM5	PIK3CA	mutation	missense	MSK-IMPACT	p.E542K
P-0008994-T01-IM5	PIK3CA	mutation	missense	MSK-IMPACT	p.H1047R
P-0003885-T01-IM5	PIK3CA	mutation	missense	MSK-IMPACT	p.E545K
P-0002591-T01-IM3	PIK3CA	mutation	missense	MSK-IMPACT	p.H1047R
P-0002858-T01-IM3	PIK3CA	mutation	missense	MSK-IMPACT	p.H1047R
P-0010758-T01-IM5	PIK3CA	mutation	missense	MSK-IMPACT	p.G122D
PD4967	PIK3CA	mutation	missense	BASIS	p.P104L
PD13299	PIK3CA	mutation	missense	BASIS	p.E542K
PD23561	PIK3CA	mutation	missense	BASIS	p.E542K
PD3905	PIK3CA	mutation	missense	BASIS	p.E542K
PD11327	PIK3CA	mutation	missense	BASIS	p.E545K
PD4967	PIK3CA	mutation	missense	BASIS	p.H1047R
TCGA-EW-A1OX	GATA3	mutation	frameshift	TCGA	p.*444Yfs*63
TCGA-A2-A25B	GATA3	mutation	frameshift	TCGA	p.M400Vfs*106
P-0002915-T01-IM3	GATA3	mutation	frameshift	MSK-IMPACT	p.N333Kfs*19

P-0005423-T01-IM5	GATA3	mutation	frameshift	MSK-IMPACT	p.P408Qfs*97
P-0002023-T01-IM3	GATA3	mutation	frameshift	MSK-IMPACT	p.S401Vfs*106
MB-0346	KMT2D	mutation	missense	METABRIC	p.G4427A
MB-5070	KMT2D	mutation	missense	METABRIC	p.P2108L
MB-6098	KMT2D	mutation	missense	METABRIC	p.M1166V
MB-6271	KMT2D	mutation	missense	METABRIC	p.P710R
MTS-T0064	KMT2D	mutation	missense	METABRIC	p.R466C
MB-6271	NOTCH1	mutation	frameshift	METABRIC	p.H2428Qfs*77
MB-6271	NOTCH1	mutation	frameshift	METABRIC	p.H2428Pfs*79
P-0008994-T01-IM5	NOTCH1	mutation	frameshift	MSK-IMPACT	p.S2486Rfs*103
P-0008994-T01-IM5	NOTCH1	mutation	missense	MSK-IMPACT	p.V2385M
P-0010457-T01-IM5	NOTCH1	mutation	missense	MSK-IMPACT	p.T123M
TCGA-EW-A1OX	ARID1A	mutation	frameshift	TCGA	p.E2250Rfs*28
MB-2827	ARID1A	mutation	missense	METABRIC	p.R1879Q
MB-7038	ARID1A	mutation	missense	METABRIC	p.A1927P
P-0002237-T01-IM3	ARID1A	mutation	missense	MSK-IMPACT	p.D1193A
MB-6060	ATR	mutation	missense	METABRIC	p.I219V
MB-7038	ATR	mutation	missense	METABRIC	p.H1672R
P-0005423-T01-IM5	ATR	mutation	missense	MSK-IMPACT	p.L2599I
P-0002858-T01-IM3	ATR	mutation	missense	MSK-IMPACT	p.E1888K
P-0008994-T01-IM5	CDH1	mutation	frameshift	MSK-IMPACT	p.P825Rfs*21
P-0003885-T01-IM5	CDH1	mutation	missense	MSK-IMPACT	p.E273K
P-0002591-T01-IM3	CDH1	mutation	frameshift	MSK-IMPACT	p.R74Dfs*9
PD14442	CDH1	mutation	nonsense	BASIS	p.Q699*
P-0002915-T01-IM3	CREBBP	mutation	missense	MSK-IMPACT	p.S893W
P-0008994-T01-IM5	CREBBP	mutation	missense	MSK-IMPACT	p.R1960Q
P-0003885-T01-IM5	CREBBP	mutation	missense	MSK-IMPACT	p.E1501K
P-0003885-T01-IM5	CREBBP	mutation	missense	MSK-IMPACT	p.H1351D
P-0007037-T01-IM5	GATA2	mutation	frameshift	MSK-IMPACT	p.D367Efs*17
P-0006504-T02-IM5	GATA2	mutation	missense	MSK-IMPACT	p.S122T
P-0003885-T01-IM5	GATA2	mutation	nonsense	MSK-IMPACT	p.Q239*
P-0003885-T01-IM5	GATA2	mutation	missense	MSK-IMPACT	p.S192F
TCGA-BH-A0AW	SYNE1	mutation	missense	TCGA	p.N4215Y
MB-5465	SYNE1	mutation	missense	METABRIC	p.L125F
MB-6060	SYNE1	mutation	missense	METABRIC	p.R1091W
MB-6098	SYNE1	mutation	nonsense	METABRIC	p.W1139*
TCGA-BH-A0C0	ABCA9	mutation	missense	TCGA	p.N1371S
TCGA-BH-A0C0	ABCA9	mutation	missense	TCGA	p.N1371S
TCGA-A2-A25B	ABCA9	mutation	missense	TCGA	p.K1521E
MB-0346	AKAP9	mutation	missense	METABRIC	p.E1342Q
MB-7038	AKAP9	mutation	missense	METABRIC	p.E2403G
MB-7048	AKAP9	mutation	missense	METABRIC	p.R3233H
MB-2827	FLT3	mutation	missense	METABRIC	p.S976R
P-0002858-T01-IM3	FLT3	mutation	missense	MSK-IMPACT	p.E672K
P-0002858-T01-IM3	FLT3	mutation	missense	MSK-IMPACT	p.S638L
PD4967	MLLT4	mutation	frameshift	BASIS	p.F275fs*7
PD4005	MLLT4	mutation	nonsense	BASIS	p.Q421*

PD13771	MLLT4	mutation	nonsense	BASIS	p.R776*
P-0002858-T01-IM3	NOTCH2	mutation	missense	MSK-IMPACT	p.D2438N
P-0002858-T01-IM3	NOTCH2	mutation	missense	MSK-IMPACT	p.E2344Q
P-0002858-T01-IM3	NOTCH2	mutation	missense	MSK-IMPACT	p.E2276K
TCGA-LL-A5YP	PTEN	mutation	missense	TCGA	p.A126G
P-0009557-T01-IM5	PTEN	mutation	frameshift	MSK-IMPACT	p.T319*
PD10014	PTEN	mutation	missense	BASIS	p.R130Q
TCGA-BH-A0C0	TAF1L	mutation	missense	TCGA	p.L782Q
TCGA-BH-A0C0	TAF1L	mutation	missense	TCGA	p.L782Q
TCGA-C8-A12L	TAF1L	mutation	frameshift	TCGA	p.P255Tfs*33
MB-5465	AHNAK2	mutation	missense	METABRIC	p.A1672V
MB-7048	AHNAK2	mutation	missense	METABRIC	p.S923C
TCGA-AO-A0JL	ARID1B	mutation	missense	TCGA	p.V2083L
MB-7038	ARID1B	mutation	missense	METABRIC	p.G1882E
MB-6060	BRCA2	mutation	missense	METABRIC	p.A3205V
MB-6060	BRCA2	mutation	missense	METABRIC	p.A3205P
TCGA-BH-A0C0	CREB3L2	mutation	missense	TCGA	p.I125M
TCGA-BH-A0C0	CREB3L2	mutation	missense	TCGA	p.I125M
P-0007037-T01-IM5	DOT1L	mutation	missense	MSK-IMPACT	p.M206I
P-0007037-T01-IM5	DOT1L	mutation	missense	MSK-IMPACT	p.Q530E
MB-7038	ERBB3	mutation	missense	METABRIC	p.G113R
MB-7038	ERBB3	mutation	missense	METABRIC	p.A1131T
P-0003885-T01-IM5	ERCC4	mutation	missense	MSK-IMPACT	p.D650H
P-0003885-T01-IM5	ERCC4	mutation	frameshift	MSK-IMPACT	p.E631Kfs*4
P-0002915-T01-IM3	GNAS	mutation	nonsense	MSK-IMPACT	p.R601*
P-0002915-T01-IM3	GNAS	mutation	missense	MSK-IMPACT	p.R601L
TCGA-LL-A5YP	GRIN2A	mutation	missense	TCGA	p.I775T
P-0002858-T01-IM3	GRIN2A	mutation	missense	MSK-IMPACT	p.E691D
TCGA-A2-A25B	HIST1H2BJ	mutation	missense	TCGA	p.M60L
P-0007037-T01-IM5	HIST1H2BJ	mutation	frameshift	MSK-IMPACT	p.D26Efs*16
P-0009557-T01-IM5	IKBKE	mutation	missense	MSK-IMPACT	p.E177Q
P-0003885-T01-IM5	IKBKE	mutation	missense	MSK-IMPACT	p.E168D
P-0002858-T01-IM3	IRS2	mutation	missense	MSK-IMPACT	p.R83Q
P-0010758-T01-IM5	IRS2	mutation	missense	MSK-IMPACT	p.P9R
TCGA-BH-A0AW	JAK2	mutation	missense	TCGA	p.R115I
TCGA-LL-A5YP	JAK2	mutation	missense	TCGA	p.V615L
P-0007037-T01-IM5	KDM5A	mutation	missense	MSK-IMPACT	p.S686F
P-0002858-T01-IM3	KDM5A	mutation	missense	MSK-IMPACT	p.S1066F
MB-5323	MAP3K1	mutation	missense	METABRIC	p.A1194V
P-0002915-T01-IM3	MAP3K1	mutation	frameshift	MSK-IMPACT	p.C571Mfs*4
P-0002915-T01-IM3	MDC1	mutation	missense	MSK-IMPACT	p.S1318A
P-0008994-T01-IM5	MDC1	mutation	frameshift	MSK-IMPACT	p.K183Nfs*9
MB-7038	NCOR1	mutation	missense	METABRIC	p.N877S
P-0003885-T01-IM5	NCOR1	mutation	nonsense	MSK-IMPACT	p.R1275*
P-0002023-T01-IM3	NSD1	mutation	missense	MSK-IMPACT	p.Q2669R
P-0002858-T01-IM3	NSD1	mutation	frameshift	MSK-IMPACT	p.E249Kfs*12
MB-6060	PDE4DIP	mutation	missense	METABRIC	p.R2117Q

MB-7038	PDE4DIP	mutation	missense	METABRIC	p.R419L
P-0002915-T01-IM3	PIK3C2G	mutation	missense	MSK-IMPACT	p.M1350I
P-0003885-T01-IM5	PIK3C2G	mutation	nonsense	MSK-IMPACT	p.S297*
TCGA-E9-A1NC	PIK3CB	mutation	frameshift	TCGA	p.R604Pfs*29
P-0002858-T01-IM3	PIK3CB	mutation	missense	MSK-IMPACT	p.R321Q
P-0011369-T01-IM5	PRDM1	mutation	missense	MSK-IMPACT	p.W199C
PD13296	PRDM1	mutation	nonsense	BASIS	p.C789*
MB-5323	PTPRD	mutation	missense	METABRIC	p.Y751F
MB-6271	PTPRD	mutation	missense	METABRIC	p.N837I
TCGA-BH-A0C0	RANBP17	mutation	missense	TCGA	p.D516N
TCGA-BH-A0C0	RANBP17	mutation	missense	TCGA	p.D516N
TCGA-BH-A0C0	RET	mutation	missense	TCGA	p.P117T
TCGA-BH-A0C0	RET	mutation	missense	TCGA	p.P117T
MB-5070	SETD2	mutation	missense	METABRIC	p.D995E
P-0008994-T01-IM5	SETD2	mutation	missense	MSK-IMPACT	p.R1543W
TCGA-AN-A0XU	SPEN	mutation	frameshift	TCGA	p.D73Pfs*6
PD9004	SPEN	mutation	frameshift	BASIS	p.L2402fs*13
P-0006504-T02-IM5	TET2	mutation	nonsense	MSK-IMPACT	p.R1452*
P-0002858-T01-IM3	TET2	mutation	missense	MSK-IMPACT	p.Q1445K
P-0011369-T01-IM5	ABL1	mutation	missense	MSK-IMPACT	p.E675A
MB-6271	AFF2	mutation	missense	METABRIC	p.L1034I
P-0002858-T01-IM3	AKT1	mutation	missense	MSK-IMPACT	p.S381L
TCGA-E9-A1NC	ALDH2	mutation	frameshift	TCGA	p.K289Efs*45
P-0002237-T01-IM3	ALK	mutation	missense	MSK-IMPACT	p.N1497K
TCGA-BH-A0C0	ARHGAP35	mutation	missense	TCGA	p.P1402T
P-0008994-T01-IM5	ATM	mutation	nonsense	MSK-IMPACT	p.R2598*
P-0002858-T01-IM3	AXIN1	mutation	missense	MSK-IMPACT	p.P61A
MB-0346	BAP1	mutation	frameshift	METABRIC	p.S623Rfs*14
TCGA-E9-A1NC	BCL3	mutation	frameshift	TCGA	p.S448Lfs*19
P-0002858-T01-IM3	BCOR	mutation	missense	MSK-IMPACT	p.R1398T
TCGA-LL-A5YP	BCORL1	mutation	missense	TCGA	p.A1661S
MB-0346	CASP8	mutation	missense	METABRIC	p.I144M
TCGA-LL-A5YP	CD36	mutation	missense	TCGA	p.S324T
TCGA-AO-A0JL	CDH11	mutation	missense	TCGA	p.L569F
P-0002858-T01-IM3	CDK12	mutation	nonsense	MSK-IMPACT	p.S24*
TCGA-EW-A1OX	CHEK1	mutation	frameshift	TCGA	p.W231*
P-0008994-T01-IM5	CHEK2	mutation	missense	MSK-IMPACT	p.R3Q
TCGA-AO-A0JL	CIITA	mutation	missense	TCGA	p.D154H
P-0010758-T01-IM5	CTCF	mutation	missense	MSK-IMPACT	p.E584Q
TCGA-BH-A0AW	CTLA4	mutation	missense	TCGA	p.L196V
TCGA-BH-A0AW	DDR2	mutation	missense	TCGA	p.S123I
P-0007037-T01-IM5	DNMT3A	mutation	missense	MSK-IMPACT	p.L639V
P-0007037-T01-IM5	EGFR	mutation	missense	MSK-IMPACT	p.N413K
TCGA-A2-A25B	FAT3	mutation	frameshift	TCGA	p.L4090Sfs*25
P-0002858-T01-IM3	FGF19	mutation	missense	MSK-IMPACT	p.D198H
P-0002591-T01-IM3	FGFR1	mutation	missense	MSK-IMPACT	p.G48D
P-0003885-T01-IM5	FGFR2	mutation	missense	MSK-IMPACT	p.P253R

P-0003885-T01-IM5	FLCN	mutation	missense	MSK-IMPACT	p.Q31E
P-0010457-T01-IM5	FLT1	mutation	missense	MSK-IMPACT	p.R280L
P-0003885-T01-IM5	FOXP1	mutation	nonsense	MSK-IMPACT	p.E294*
P-0006504-T02-IM5	FYN	mutation	missense	MSK-IMPACT	p.R481Q
TCGA-E9-A1NC	GATA1	mutation	missense	TCGA	p.R307H
TCGA-AO-A0JL	HGF	mutation	missense	TCGA	p.R468C
TCGA-AO-A0JL	ICOSLG	mutation	frameshift	TCGA	p.H147Pfs*7
P-0008994-T01-IM5	IDH1	mutation	missense	MSK-IMPACT	p.T327A
TCGA-C8-A12L	IL7R	mutation	missense	TCGA	p.M164V
P-0010758-T01-IM5	INHBA	mutation	missense	MSK-IMPACT	p.W218C
TCGA-AN-A0XU	ITGAL	mutation	missense	TCGA	p.L969M
P-0008994-T01-IM5	JAK1	mutation	frameshift	MSK-IMPACT	p.P430Rfs*2
P-0008994-T01-IM5	JUN	mutation	missense	MSK-IMPACT	p.P207Q
P-0007037-T01-IM5	KDM5C	mutation	missense	MSK-IMPACT	p.L747P
P-0002858-T01-IM3	KIT	mutation	missense	MSK-IMPACT	p.E88K
P-0010758-T01-IM5	LATS1	mutation	missense	MSK-IMPACT	p.T367S
P-0010457-T01-IM5	LATS2	mutation	missense	MSK-IMPACT	p.K855R
MB-7038	LIFR	mutation	missense	METABRIC	p.H105R
TCGA-AN-A0XU	MAML2	mutation	missense	TCGA	p.P572L
P-0002858-T01-IM3	MDM2	mutation	missense	MSK-IMPACT	p.Q141E
TCGA-C8-A12L	MYH9	mutation	missense	TCGA	p.E912D
TCGA-AO-A0JL	NAV3	mutation	missense	TCGA	p.E1734K
MB-6271	NCOA3	mutation	missense	METABRIC	p.R487H
MB-5070	NCOR2	mutation	missense	METABRIC	p.E871K
PD24202	NF1	mutation	nonsense	BASIS	p.Q589*
P-0011369-T01-IM5	NKX2-1	mutation	missense	MSK-IMPACT	p.A124G
P-0002858-T01-IM3	NTRK1	mutation	missense	MSK-IMPACT	p.F395L
TCGA-E9-A1NC	NUP93	mutation	missense	TCGA	p.E14K
TCGA-C8-A12L	OBSCN	mutation	missense	TCGA	p.T544S
TCGA-BH-A0AW	PAG1	mutation	missense	TCGA	p.V242A
P-0003885-T01-IM5	PAK7	mutation	missense	MSK-IMPACT	p.S114N
P-0007037-T01-IM5	PALB2	mutation	missense	MSK-IMPACT	p.R942T
P-0002858-T01-IM3	PARP1	mutation	missense	MSK-IMPACT	p.D957N
PD8980	PBRM1	mutation	frameshift	BASIS	p.P227fs*2
P-0003885-T01-IM5	PDCD1	mutation	missense	MSK-IMPACT	p.Q245H
TCGA-C8-A12L	PDCD11	mutation	missense	TCGA	p.H417Q
P-0007037-T01-IM5	PDGFRA	mutation	missense	MSK-IMPACT	p.D366H
PD5930	PHF6	mutation	nonsense	BASIS	p.G348*
TCGA-AN-A0XU	PIK3C3	mutation	missense	TCGA	p.P98S
P-0005423-T01-IM5	PMS1	mutation	missense	MSK-IMPACT	p.H81Y
P-0002858-T01-IM3	POLE	mutation	missense	MSK-IMPACT	p.E1299Q
PD6406	PREX2	mutation	missense	BASIS	p.R155Q
P-0007037-T01-IM5	PTCH1	mutation	missense	MSK-IMPACT	p.L209R
TCGA-C8-A12L	PTPN11	mutation	missense	TCGA	p.G13D
TCGA-C8-A12L	PTPN2	mutation	missense	TCGA	p.I164S
P-0003885-T01-IM5	PTPRT	mutation	missense	MSK-IMPACT	p.R450Q
P-0003885-T01-IM5	RAD51	mutation	missense	MSK-IMPACT	p.E50Q

P-0008994-T01-IM5	RAD52	mutation	missense	MSK-IMPACT	p.N99D
P-0002858-T01-IM3	RAD54L	mutation	missense	MSK-IMPACT	p.Q689E
P-0002237-T01-IM3	RBM10	mutation	missense	MSK-IMPACT	p.D20N
TCGA-AN-A0XU	RBMX	mutation	frameshift	TCGA	p.P106Ffs*32
P-0005423-T01-IM5	RICTOR	mutation	missense	MSK-IMPACT	p.I228F
P-0002858-T01-IM3	RIT1	mutation	missense	MSK-IMPACT	p.M90I
TCGA-E2-A1L7	SGK1	mutation	missense	TCGA	p.K137N
P-0003885-T01-IM5	SMAD2	mutation	nonsense	MSK-IMPACT	p.Q168*
TCGA-A2-A25B	SMAD4	mutation	missense	TCGA	p.L109R
TCGA-A2-A25B	SMARCA1	mutation	missense	TCGA	p.S321Y
P-0002858-T01-IM3	SMARCA4	mutation	missense	MSK-IMPACT	p.E566Q
TCGA-AN-A0XU	STAG1	mutation	missense	TCGA	p.N941I
P-0009557-T01-IM5	STAG2	mutation	missense	MSK-IMPACT	p.I900M
TCGA-E9-A1NC	STAT6	mutation	frameshift	TCGA	p.Q281Pfs*92
TCGA-BH-A18R	TBL1XR1	mutation	frameshift	TCGA	p.A142*
P-0002858-T01-IM3	TET1	mutation	missense	MSK-IMPACT	p.D658H
P-0002858-T01-IM3	TGFBR2	mutation	missense	MSK-IMPACT	p.F442Y
TCGA-BH-A1FU	THRAP3	mutation	missense	TCGA	p.R48S
P-0010457-T01-IM5	TOP1	mutation	missense	MSK-IMPACT	p.E213G
TCGA-AO-A0JL	TRIP11	mutation	missense	TCGA	p.M716T
TCGA-LL-A5YP	TRRAP	mutation	missense	TCGA	p.F154S
P-0002915-T01-IM3	TSC2	mutation	missense	MSK-IMPACT	p.K1180E
TCGA-E9-A1NC	TYK2	mutation	frameshift	TCGA	p.T1106Hfs*5
TCGA-BH-A0AW	UHRF1BP1L	mutation	missense	TCGA	p.S752L
MB-6060	USP9X	mutation	nonsense	METABRIC	p.L1321*
P-0002237-T01-IM3	WT1	mutation	missense	MSK-IMPACT	p.P135A
TCGA-C8-A12L	XPO1	mutation	missense	TCGA	p.F444L
P-0002858-T01-IM3	YES1	mutation	missense	MSK-IMPACT	p.E349K
P-0006504-T02-IM5	ZFHX3	mutation	missense	MSK-IMPACT	p.F204C
TCGA-A2-A25B	SDHD	cna	hetloss	TCGA	
TCGA-AN-A0XU	SDHD	cna	gain	TCGA	
TCGA-AO-A0JL	SDHD	cna	hetloss	TCGA	
TCGA-BH-A0C0	SDHD	cna	gain	TCGA	
TCGA-BH-A18R	SDHD	cna	homdel	TCGA	
TCGA-D8-A27M	SDHD	cna	hetloss	TCGA	
TCGA-E2-A1L7	SDHD	cna	hetloss	TCGA	
TCGA-E9-A1NC	SDHD	cna	hetloss	TCGA	
TCGA-EW-A1OX	SDHD	cna	homdel	TCGA	
TCGA-LL-A5YP	SDHD	cna	hetloss	TCGA	
MB-0346	SDHD	cna	hetloss	METABRIC	
MB-5070	SDHD	cna	hetloss	METABRIC	
MB-5107	SDHD	cna	hetloss	METABRIC	
MB-6271	SDHD	cna	hetloss	METABRIC	
MB-7032	SDHD	cna	hetloss	METABRIC	
MB-7048	SDHD	cna	hetloss	METABRIC	
PD11327	SDHD	cna	gain	BASIS	
PD11742	SDHD	cna	hetloss	BASIS	

PD13296	SDHD	cna	hetloss	BASIS
PD13299	SDHD	cna	gain	BASIS
PD13771	SDHD	cna	hetloss	BASIS
PD22355	SDHD	cna	hetloss	BASIS
PD23574	SDHD	cna	gain	BASIS
PD23578	SDHD	cna	hetloss	BASIS
PD24186	SDHD	cna	gain	BASIS
PD24206	SDHD	cna	gain	BASIS
PD24337	SDHD	cna	hetloss	BASIS
PD3905	SDHD	cna	gain	BASIS
PD4005	SDHD	cna	hetloss	BASIS
PD4826	SDHD	cna	hetloss	BASIS
PD5935	SDHD	cna	gain	BASIS
PD5945	SDHD	cna	gain	BASIS
PD5948	SDHD	cna	gain	BASIS
PD6413	SDHD	cna	hetloss	BASIS
PD6731	SDHD	cna	hetloss	BASIS
PD7067	SDHD	cna	gain	BASIS
PD7215	SDHD	cna	gain	BASIS
PD9004	SDHD	cna	hetloss	BASIS
PD9585	SDHD	cna	gain	BASIS
PD9702	SDHD	cna	gain	BASIS
PD11327	SDHD	cna	gain	BASIS
PD11742	SDHD	cna	hetloss	BASIS
PD13296	SDHD	cna	hetloss	BASIS
PD13299	SDHD	cna	gain	BASIS
PD13771	SDHD	cna	hetloss	BASIS
PD22355	SDHD	cna	hetloss	BASIS
PD23574	SDHD	cna	gain	BASIS
PD23578	SDHD	cna	hetloss	BASIS
PD24186	SDHD	cna	gain	BASIS
PD24206	SDHD	cna	gain	BASIS
PD24337	SDHD	cna	hetloss	BASIS
PD3905	SDHD	cna	gain	BASIS
PD4005	SDHD	cna	hetloss	BASIS
PD4826	SDHD	cna	hetloss	BASIS
PD5935	SDHD	cna	gain	BASIS
PD5945	SDHD	cna	gain	BASIS
PD5948	SDHD	cna	gain	BASIS
PD6413	SDHD	cna	hetloss	BASIS
PD6731	SDHD	cna	hetloss	BASIS
PD7067	SDHD	cna	gain	BASIS
PD7215	SDHD	cna	gain	BASIS
PD9004	SDHD	cna	hetloss	BASIS
PD9585	SDHD	cna	gain	BASIS
PD9702	SDHD	cna	gain	BASIS
MB-2827	CKS1B	cna	amp	METABRIC

MB-5070	CKS1B	cna	gain	METABRIC
MB-6060	CKS1B	cna	gain	METABRIC
MB-6098	CKS1B	cna	amp	METABRIC
MB-7032	CKS1B	cna	gain	METABRIC
PD10014	CKS1B	cna	gain	BASIS
PD11327	CKS1B	cna	gain	BASIS
PD13297	CKS1B	cna	gain	BASIS
PD13299	CKS1B	cna	gain	BASIS
PD13771	CKS1B	cna	gain	BASIS
PD14442	CKS1B	cna	gain	BASIS
PD22355	CKS1B	cna	gain	BASIS
PD23562	CKS1B	cna	amp	BASIS
PD23574	CKS1B	cna	amp	BASIS
PD24186	CKS1B	cna	gain	BASIS
PD24206	CKS1B	cna	gain	BASIS
PD3890	CKS1B	cna	gain	BASIS
PD3905	CKS1B	cna	gain	BASIS
PD4005	CKS1B	cna	gain	BASIS
PD4006	CKS1B	cna	amp	BASIS
PD4107	CKS1B	cna	gain	BASIS
PD4826	CKS1B	cna	gain	BASIS
PD5930	CKS1B	cna	amp	BASIS
PD5935	CKS1B	cna	gain	BASIS
PD5945	CKS1B	cna	amp	BASIS
PD5948	CKS1B	cna	gain	BASIS
PD6406	CKS1B	cna	gain	BASIS
PD6413	CKS1B	cna	gain	BASIS
PD7067	CKS1B	cna	amp	BASIS
PD7215	CKS1B	cna	gain	BASIS
PD8621	CKS1B	cna	amp	BASIS
PD8980	CKS1B	cna	gain	BASIS
PD9004	CKS1B	cna	amp	BASIS
PD9702	CKS1B	cna	amp	BASIS
PD10014	CKS1B	cna	hetloss	BASIS
PD11742	CKS1B	cna	hetloss	BASIS
PD13296	CKS1B	cna	hetloss	BASIS
PD13297	CKS1B	cna	hetloss	BASIS
PD22355	CKS1B	cna	hetloss	BASIS
PD23562	CKS1B	cna	hetloss	BASIS
PD23578	CKS1B	cna	hetloss	BASIS
PD24186	CKS1B	cna	gain	BASIS
PD24202	CKS1B	cna	hetloss	BASIS
PD24206	CKS1B	cna	gain	BASIS
PD24337	CKS1B	cna	hetloss	BASIS
PD3890	CKS1B	cna	hetloss	BASIS
PD3905	CKS1B	cna	gain	BASIS
PD4005	CKS1B	cna	hetloss	BASIS

PD4006	CKS1B	cna	homdel	BASIS
PD4107	CKS1B	cna	hetloss	BASIS
PD5930	CKS1B	cna	gain	BASIS
PD5935	CKS1B	cna	hetloss	BASIS
PD5948	CKS1B	cna	hetloss	BASIS
PD6413	CKS1B	cna	hetloss	BASIS
PD6731	CKS1B	cna	hetloss	BASIS
PD7215	CKS1B	cna	hetloss	BASIS
PD8621	CKS1B	cna	hetloss	BASIS
PD9585	CKS1B	cna	hetloss	BASIS
TCGA-A2-A25B	MYC	cna	gain	TCGA
TCGA-AN-A0XU	MYC	cna	amp	TCGA
TCGA-AO-A0JL	MYC	cna	amp	TCGA
TCGA-BH-A0AW	MYC	cna	gain	TCGA
TCGA-BH-A0C0	MYC	cna	amp	TCGA
TCGA-BH-A18R	MYC	cna	amp	TCGA
TCGA-BH-A1FU	MYC	cna	amp	TCGA
TCGA-C8-A12L	MYC	cna	gain	TCGA
TCGA-D8-A27M	MYC	cna	amp	TCGA
TCGA-E2-A1L7	MYC	cna	amp	TCGA
TCGA-E9-A1NC	MYC	cna	amp	TCGA
TCGA-LL-A5YP	MYC	cna	amp	TCGA
MB-0346	MYC	cna	amp	METABRIC
MB-2827	MYC	cna	amp	METABRIC
MB-5107	MYC	cna	amp	METABRIC
MB-5465	MYC	cna	gain	METABRIC
MB-6060	MYC	cna	gain	METABRIC
MB-6098	MYC	cna	amp	METABRIC
MB-7038	MYC	cna	amp	METABRIC
MB-7048	MYC	cna	amp	METABRIC
MB-0420	MYC	cna	gain	METABRIC
MTS-T0064	MYC	cna	amp	METABRIC
P-0002237-T01-IM3	MYC	cna	amp	MSK-IMPACT
P-0010758-T01-IM5	MYC	cna	amp	MSK-IMPACT
PD10014	MYC	cna	gain	BASIS
PD11327	MYC	cna	amp	BASIS
PD11742	MYC	cna	gain	BASIS
PD13296	MYC	cna	hetloss	BASIS
PD13297	MYC	cna	gain	BASIS
PD13299	MYC	cna	amp	BASIS
PD13771	MYC	cna	gain	BASIS
PD23562	MYC	cna	amp	BASIS
PD23574	MYC	cna	amp	BASIS
PD23578	MYC	cna	gain	BASIS
PD24186	MYC	cna	amp	BASIS
PD24202	MYC	cna	gain	BASIS
PD24206	MYC	cna	amp	BASIS

PD24337	MYC	cna	gain	BASIS
PD3890	MYC	cna	amp	BASIS
PD3905	MYC	cna	amp	BASIS
PD4005	MYC	cna	gain	BASIS
PD4006	MYC	cna	gain	BASIS
PD4107	MYC	cna	gain	BASIS
PD4826	MYC	cna	amp	BASIS
PD5930	MYC	cna	gain	BASIS
PD5935	MYC	cna	amp	BASIS
PD5945	MYC	cna	amp	BASIS
PD5948	MYC	cna	amp	BASIS
PD6406	MYC	cna	gain	BASIS
PD6413	MYC	cna	gain	BASIS
PD6731	MYC	cna	gain	BASIS
PD7067	MYC	cna	amp	BASIS
PD7215	MYC	cna	amp	BASIS
PD8621	MYC	cna	gain	BASIS
PD8980	MYC	cna	amp	BASIS
PD9004	MYC	cna	amp	BASIS
PD9585	MYC	cna	gain	BASIS
PD9702	MYC	cna	amp	BASIS
TCGA-A2-A25B	RAD21	cna	gain	TCGA
TCGA-AN-A0XU	RAD21	cna	gain	TCGA
TCGA-AO-A0JL	RAD21	cna	amp	TCGA
TCGA-BH-A0AW	RAD21	cna	gain	TCGA
TCGA-BH-A0C0	RAD21	cna	amp	TCGA
TCGA-BH-A18R	RAD21	cna	amp	TCGA
TCGA-BH-A1FU	RAD21	cna	amp	TCGA
TCGA-C8-A12L	RAD21	cna	gain	TCGA
TCGA-D8-A27M	RAD21	cna	amp	TCGA
TCGA-E2-A1L7	RAD21	cna	gain	TCGA
TCGA-E9-A1NC	RAD21	cna	amp	TCGA
TCGA-LL-A5YP	RAD21	cna	amp	TCGA
MB-0346	RAD21	cna	amp	METABRIC
MB-2827	RAD21	cna	amp	METABRIC
MB-5070	RAD21	cna	amp	METABRIC
MB-5107	RAD21	cna	amp	METABRIC
MB-5465	RAD21	cna	gain	METABRIC
MB-6060	RAD21	cna	gain	METABRIC
MB-6098	RAD21	cna	amp	METABRIC
MB-7038	RAD21	cna	amp	METABRIC
MB-7048	RAD21	cna	gain	METABRIC
MB-0420	RAD21	cna	gain	METABRIC
P-0010758-T01-IM5	RAD21	cna	amp	MSK-IMPACT
PD10014	RAD21	cna	gain	BASIS
PD11327	RAD21	cna	amp	BASIS
PD11742	RAD21	cna	gain	BASIS

PD13296	RAD21	cna	hetloss	BASIS
PD13299	RAD21	cna	amp	BASIS
PD13771	RAD21	cna	gain	BASIS
PD23562	RAD21	cna	amp	BASIS
PD23574	RAD21	cna	amp	BASIS
PD23578	RAD21	cna	gain	BASIS
PD24186	RAD21	cna	amp	BASIS
PD24202	RAD21	cna	gain	BASIS
PD24206	RAD21	cna	amp	BASIS
PD24337	RAD21	cna	gain	BASIS
PD3890	RAD21	cna	gain	BASIS
PD3905	RAD21	cna	gain	BASIS
PD4005	RAD21	cna	gain	BASIS
PD4006	RAD21	cna	gain	BASIS
PD4107	RAD21	cna	gain	BASIS
PD4826	RAD21	cna	amp	BASIS
PD4967	RAD21	cna	gain	BASIS
PD5930	RAD21	cna	gain	BASIS
PD5935	RAD21	cna	gain	BASIS
PD5945	RAD21	cna	amp	BASIS
PD5948	RAD21	cna	amp	BASIS
PD6406	RAD21	cna	gain	BASIS
PD6413	RAD21	cna	gain	BASIS
PD6731	RAD21	cna	gain	BASIS
PD7067	RAD21	cna	amp	BASIS
PD7215	RAD21	cna	amp	BASIS
PD8621	RAD21	cna	gain	BASIS
PD8980	RAD21	cna	gain	BASIS
PD9004	RAD21	cna	amp	BASIS
PD9585	RAD21	cna	gain	BASIS
PD9702	RAD21	cna	amp	BASIS
TCGA-A2-A25B	NDRG1	cna	gain	TCGA
TCGA-AN-A0XU	NDRG1	cna	hetloss	TCGA
TCGA-AO-A0JL	NDRG1	cna	amp	TCGA
TCGA-BH-A0AW	NDRG1	cna	gain	TCGA
TCGA-BH-A0C0	NDRG1	cna	amp	TCGA
TCGA-BH-A18R	NDRG1	cna	amp	TCGA
TCGA-BH-A1FU	NDRG1	cna	gain	TCGA
TCGA-C8-A12L	NDRG1	cna	gain	TCGA
TCGA-D8-A27M	NDRG1	cna	amp	TCGA
TCGA-E2-A1L7	NDRG1	cna	amp	TCGA
TCGA-E9-A1NC	NDRG1	cna	amp	TCGA
TCGA-LL-A5YP	NDRG1	cna	gain	TCGA
MB-0346	NDRG1	cna	amp	METABRIC
MB-2827	NDRG1	cna	amp	METABRIC
MB-5107	NDRG1	cna	amp	METABRIC
MB-5465	NDRG1	cna	gain	METABRIC

MB-6060	NDRG1	cna	gain	METABRIC
MB-6098	NDRG1	cna	amp	METABRIC
MB-7038	NDRG1	cna	amp	METABRIC
MB-7048	NDRG1	cna	amp	METABRIC
MB-0420	NDRG1	cna	gain	METABRIC
MTS-T0064	NDRG1	cna	gain	METABRIC
PD10014	NDRG1	cna	gain	BASIS
PD11327	NDRG1	cna	gain	BASIS
PD11742	NDRG1	cna	gain	BASIS
PD13296	NDRG1	cna	gain	BASIS
PD13297	NDRG1	cna	gain	BASIS
PD13299	NDRG1	cna	gain	BASIS
PD13771	NDRG1	cna	hetloss	BASIS
PD23562	NDRG1	cna	amp	BASIS
PD23574	NDRG1	cna	gain	BASIS
PD23578	NDRG1	cna	gain	BASIS
PD24186	NDRG1	cna	amp	BASIS
PD24202	NDRG1	cna	gain	BASIS
PD24206	NDRG1	cna	amp	BASIS
PD24337	NDRG1	cna	gain	BASIS
PD3890	NDRG1	cna	gain	BASIS
PD3905	NDRG1	cna	amp	BASIS
PD4005	NDRG1	cna	gain	BASIS
PD4006	NDRG1	cna	gain	BASIS
PD4107	NDRG1	cna	gain	BASIS
PD4826	NDRG1	cna	amp	BASIS
PD5930	NDRG1	cna	gain	BASIS
PD5935	NDRG1	cna	amp	BASIS
PD5945	NDRG1	cna	amp	BASIS
PD5948	NDRG1	cna	amp	BASIS
PD6406	NDRG1	cna	gain	BASIS
PD6413	NDRG1	cna	gain	BASIS
PD6731	NDRG1	cna	gain	BASIS
PD7067	NDRG1	cna	gain	BASIS
PD7215	NDRG1	cna	amp	BASIS
PD8621	NDRG1	cna	gain	BASIS
PD8980	NDRG1	cna	amp	BASIS
PD9004	NDRG1	cna	amp	BASIS
PD9585	NDRG1	cna	gain	BASIS
PD9702	NDRG1	cna	gain	BASIS
TCGA-A2-A25B	PTK2	cna	gain	TCGA
TCGA-AN-A0XU	PTK2	cna	hetloss	TCGA
TCGA-AO-A0JL	PTK2	cna	amp	TCGA
TCGA-BH-A0AW	PTK2	cna	gain	TCGA
TCGA-BH-A0C0	PTK2	cna	amp	TCGA
TCGA-BH-A18R	PTK2	cna	amp	TCGA
TCGA-BH-A1FU	PTK2	cna	gain	TCGA

TCGA-C8-A12L	PTK2	cna	gain	TCGA
TCGA-D8-A27M	PTK2	cna	amp	TCGA
TCGA-E2-A1L7	PTK2	cna	amp	TCGA
TCGA-E9-A1NC	PTK2	cna	amp	TCGA
TCGA-LL-A5YP	PTK2	cna	gain	TCGA
MB-0346	PTK2	cna	amp	METABRIC
MB-5107	PTK2	cna	amp	METABRIC
MB-5465	PTK2	cna	gain	METABRIC
MB-6060	PTK2	cna	gain	METABRIC
MB-6098	PTK2	cna	amp	METABRIC
MB-7038	PTK2	cna	amp	METABRIC
MB-7048	PTK2	cna	amp	METABRIC
MB-0420	PTK2	cna	gain	METABRIC
MTS-T0064	PTK2	cna	gain	METABRIC
PD10014	PTK2	cna	gain	BASIS
PD11327	PTK2	cna	gain	BASIS
PD11742	PTK2	cna	gain	BASIS
PD13296	PTK2	cna	gain	BASIS
PD13297	PTK2	cna	gain	BASIS
PD13299	PTK2	cna	gain	BASIS
PD13771	PTK2	cna	hetloss	BASIS
PD23562	PTK2	cna	amp	BASIS
PD23574	PTK2	cna	gain	BASIS
PD23578	PTK2	cna	gain	BASIS
PD24186	PTK2	cna	amp	BASIS
PD24202	PTK2	cna	hetloss	BASIS
PD24206	PTK2	cna	amp	BASIS
PD24337	PTK2	cna	gain	BASIS
PD3890	PTK2	cna	gain	BASIS
PD3905	PTK2	cna	gain	BASIS
PD4005	PTK2	cna	gain	BASIS
PD4006	PTK2	cna	amp	BASIS
PD4107	PTK2	cna	gain	BASIS
PD4826	PTK2	cna	amp	BASIS
PD5930	PTK2	cna	gain	BASIS
PD5935	PTK2	cna	amp	BASIS
PD5945	PTK2	cna	amp	BASIS
PD5948	PTK2	cna	gain	BASIS
PD6406	PTK2	cna	amp	BASIS
PD6413	PTK2	cna	gain	BASIS
PD6731	PTK2	cna	gain	BASIS
PD7067	PTK2	cna	amp	BASIS
PD7215	PTK2	cna	amp	BASIS
PD8621	PTK2	cna	gain	BASIS
PD8980	PTK2	cna	amp	BASIS
PD9004	PTK2	cna	amp	BASIS
PD9585	PTK2	cna	gain	BASIS

PD9702	PTK2	cna	gain	BASIS
TCGA-A2-A25B	RECQL4	cna	gain	TCGA
TCGA-AN-A0XU	RECQL4	cna	gain	TCGA
TCGA-AO-A0JL	RECQL4	cna	amp	TCGA
TCGA-BH-A0AW	RECQL4	cna	gain	TCGA
TCGA-BH-A0C0	RECQL4	cna	amp	TCGA
TCGA-BH-A18R	RECQL4	cna	amp	TCGA
TCGA-BH-A1FU	RECQL4	cna	hetloss	TCGA
TCGA-C8-A12L	RECQL4	cna	gain	TCGA
TCGA-D8-A27M	RECQL4	cna	amp	TCGA
TCGA-E2-A1L7	RECQL4	cna	amp	TCGA
TCGA-E9-A1NC	RECQL4	cna	amp	TCGA
TCGA-LL-A5YP	RECQL4	cna	gain	TCGA
MB-0346	RECQL4	cna	amp	METABRIC
MB-5107	RECQL4	cna	amp	METABRIC
MB-5465	RECQL4	cna	gain	METABRIC
MB-6060	RECQL4	cna	gain	METABRIC
MB-6098	RECQL4	cna	amp	METABRIC
MB-7038	RECQL4	cna	amp	METABRIC
MB-7048	RECQL4	cna	amp	METABRIC
P-0002237-T01-IM3	RECQL4	cna	amp	MSK-IMPACT
P-0010758-T01-IM5	RECQL4	cna	amp	MSK-IMPACT
PD10014	RECQL4	cna	gain	BASIS
PD11327	RECQL4	cna	gain	BASIS
PD11742	RECQL4	cna	gain	BASIS
PD13297	RECQL4	cna	gain	BASIS
PD13299	RECQL4	cna	gain	BASIS
PD13771	RECQL4	cna	hetloss	BASIS
PD22355	RECQL4	cna	gain	BASIS
PD23562	RECQL4	cna	amp	BASIS
PD23574	RECQL4	cna	gain	BASIS
PD23578	RECQL4	cna	hetloss	BASIS
PD24186	RECQL4	cna	amp	BASIS
PD24202	RECQL4	cna	hetloss	BASIS
PD24206	RECQL4	cna	amp	BASIS
PD24337	RECQL4	cna	gain	BASIS
PD3890	RECQL4	cna	gain	BASIS
PD3905	RECQL4	cna	gain	BASIS
PD4005	RECQL4	cna	gain	BASIS
PD4006	RECQL4	cna	amp	BASIS
PD4107	RECQL4	cna	gain	BASIS
PD4826	RECQL4	cna	amp	BASIS
PD5930	RECQL4	cna	gain	BASIS
PD5935	RECQL4	cna	amp	BASIS
PD5945	RECQL4	cna	amp	BASIS
PD5948	RECQL4	cna	gain	BASIS
PD6406	RECQL4	cna	amp	BASIS

PD6413	RECQL4	cna	gain	BASIS
PD6731	RECQL4	cna	gain	BASIS
PD7067	RECQL4	cna	amp	BASIS
PD7215	RECQL4	cna	amp	BASIS
PD8621	RECQL4	cna	gain	BASIS
PD8980	RECQL4	cna	amp	BASIS
PD9004	RECQL4	cna	amp	BASIS
PD9585	RECQL4	cna	gain	BASIS
PD9702	RECQL4	cna	amp	BASIS
TCGA-A2-A25B	RSPO2	cna	gain	TCGA
TCGA-AN-A0XU	RSPO2	cna	gain	TCGA
TCGA-AO-A0JL	RSPO2	cna	amp	TCGA
TCGA-BH-A0AW	RSPO2	cna	gain	TCGA
TCGA-BH-A0C0	RSPO2	cna	amp	TCGA
TCGA-BH-A18R	RSPO2	cna	amp	TCGA
TCGA-BH-A1FU	RSPO2	cna	amp	TCGA
TCGA-C8-A12L	RSPO2	cna	gain	TCGA
TCGA-D8-A27M	RSPO2	cna	amp	TCGA
TCGA-E2-A1L7	RSPO2	cna	gain	TCGA
TCGA-E9-A1NC	RSPO2	cna	amp	TCGA
TCGA-LL-A5YP	RSPO2	cna	amp	TCGA
MB-0346	RSPO2	cna	amp	METABRIC
MB-2827	RSPO2	cna	amp	METABRIC
MB-5070	RSPO2	cna	gain	METABRIC
MB-5107	RSPO2	cna	amp	METABRIC
MB-5465	RSPO2	cna	gain	METABRIC
MB-6060	RSPO2	cna	amp	METABRIC
MB-6098	RSPO2	cna	amp	METABRIC
MB-7038	RSPO2	cna	amp	METABRIC
MB-7048	RSPO2	cna	gain	METABRIC
MB-0420	RSPO2	cna	gain	METABRIC
PD10014	RSPO2	cna	gain	BASIS
PD11327	RSPO2	cna	amp	BASIS
PD11742	RSPO2	cna	gain	BASIS
PD13296	RSPO2	cna	hetloss	BASIS
PD13299	RSPO2	cna	amp	BASIS
PD13771	RSPO2	cna	gain	BASIS
PD23562	RSPO2	cna	amp	BASIS
PD23574	RSPO2	cna	gain	BASIS
PD23578	RSPO2	cna	gain	BASIS
PD24186	RSPO2	cna	amp	BASIS
PD24202	RSPO2	cna	gain	BASIS
PD24206	RSPO2	cna	amp	BASIS
PD24337	RSPO2	cna	gain	BASIS
PD3890	RSPO2	cna	gain	BASIS
PD3905	RSPO2	cna	gain	BASIS
PD4005	RSPO2	cna	gain	BASIS

PD4006	RSPO2	cna	gain	BASIS
PD4107	RSPO2	cna	gain	BASIS
PD4826	RSPO2	cna	amp	BASIS
PD4967	RSPO2	cna	gain	BASIS
PD5930	RSPO2	cna	gain	BASIS
PD5935	RSPO2	cna	gain	BASIS
PD5945	RSPO2	cna	amp	BASIS
PD5948	RSPO2	cna	amp	BASIS
PD6406	RSPO2	cna	gain	BASIS
PD6413	RSPO2	cna	gain	BASIS
PD7067	RSPO2	cna	amp	BASIS
PD7215	RSPO2	cna	amp	BASIS
PD8621	RSPO2	cna	gain	BASIS
PD8980	RSPO2	cna	gain	BASIS
PD9004	RSPO2	cna	amp	BASIS
PD9585	RSPO2	cna	gain	BASIS
PD9702	RSPO2	cna	amp	BASIS
TCGA-A2-A25B	EXT1	cna	gain	TCGA
TCGA-AN-A0XU	EXT1	cna	gain	TCGA
TCGA-AO-A0JL	EXT1	cna	amp	TCGA
TCGA-BH-A0AW	EXT1	cna	gain	TCGA
TCGA-BH-A0C0	EXT1	cna	amp	TCGA
TCGA-BH-A18R	EXT1	cna	amp	TCGA
TCGA-BH-A1FU	EXT1	cna	amp	TCGA
TCGA-C8-A12L	EXT1	cna	gain	TCGA
TCGA-D8-A27M	EXT1	cna	amp	TCGA
TCGA-E2-A1L7	EXT1	cna	gain	TCGA
TCGA-E9-A1NC	EXT1	cna	amp	TCGA
TCGA-LL-A5YP	EXT1	cna	amp	TCGA
MB-0346	EXT1	cna	amp	METABRIC
MB-2827	EXT1	cna	amp	METABRIC
MB-5070	EXT1	cna	amp	METABRIC
MB-5107	EXT1	cna	amp	METABRIC
MB-5465	EXT1	cna	amp	METABRIC
MB-6060	EXT1	cna	gain	METABRIC
MB-6098	EXT1	cna	amp	METABRIC
MB-7038	EXT1	cna	amp	METABRIC
MB-7048	EXT1	cna	gain	METABRIC
MB-0420	EXT1	cna	gain	METABRIC
PD10014	EXT1	cna	gain	BASIS
PD11327	EXT1	cna	amp	BASIS
PD11742	EXT1	cna	gain	BASIS
PD13296	EXT1	cna	hetloss	BASIS
PD13299	EXT1	cna	amp	BASIS
PD13771	EXT1	cna	gain	BASIS
PD23562	EXT1	cna	amp	BASIS
PD23574	EXT1	cna	amp	BASIS

PD23578	EXT1	cna	gain	BASIS
PD24202	EXT1	cna	gain	BASIS
PD24206	EXT1	cna	amp	BASIS
PD24337	EXT1	cna	gain	BASIS
PD3890	EXT1	cna	gain	BASIS
PD3905	EXT1	cna	gain	BASIS
PD4005	EXT1	cna	gain	BASIS
PD4006	EXT1	cna	gain	BASIS
PD4107	EXT1	cna	gain	BASIS
PD4826	EXT1	cna	amp	BASIS
PD5930	EXT1	cna	gain	BASIS
PD5935	EXT1	cna	gain	BASIS
PD5945	EXT1	cna	amp	BASIS
PD5948	EXT1	cna	amp	BASIS
PD6406	EXT1	cna	gain	BASIS
PD6413	EXT1	cna	gain	BASIS
PD6731	EXT1	cna	gain	BASIS
PD7067	EXT1	cna	amp	BASIS
PD7215	EXT1	cna	amp	BASIS
PD8621	EXT1	cna	gain	BASIS
PD8980	EXT1	cna	gain	BASIS
PD9004	EXT1	cna	amp	BASIS
PD9585	EXT1	cna	gain	BASIS
PD9702	EXT1	cna	amp	BASIS
TCGA-AO-A0JL	PIK3R2	cna	gain	TCGA
TCGA-BH-A0AW	PIK3R2	cna	gain	TCGA
TCGA-BH-A0C0	PIK3R2	cna	gain	TCGA
TCGA-BH-A1FU	PIK3R2	cna	hetloss	TCGA
TCGA-C8-A12L	PIK3R2	cna	gain	TCGA
TCGA-D8-A27M	PIK3R2	cna	hetloss	TCGA
TCGA-EW-A1OX	PIK3R2	cna	hetloss	TCGA
TCGA-LL-A5YP	PIK3R2	cna	hetloss	TCGA
MB-0346	PIK3R2	cna	hetloss	METABRIC
MB-2827	PIK3R2	cna	hetloss	METABRIC
MB-6060	PIK3R2	cna	gain	METABRIC
MB-6098	PIK3R2	cna	hetloss	METABRIC
MB-0420	PIK3R2	cna	hetloss	METABRIC
MTS-T0064	PIK3R2	cna	amp	METABRIC
PD13299	PIK3R2	cna	gain	BASIS
PD14442	PIK3R2	cna	hetloss	BASIS
PD22355	PIK3R2	cna	gain	BASIS
PD23562	PIK3R2	cna	gain	BASIS
PD23574	PIK3R2	cna	gain	BASIS
PD24186	PIK3R2	cna	gain	BASIS
PD24206	PIK3R2	cna	hetloss	BASIS
PD3890	PIK3R2	cna	hetloss	BASIS
PD3905	PIK3R2	cna	gain	BASIS

PD4006	PIK3R2	cna	amp	BASIS
PD4107	PIK3R2	cna	gain	BASIS
PD4826	PIK3R2	cna	gain	BASIS
PD4967	PIK3R2	cna	hetloss	BASIS
PD5930	PIK3R2	cna	gain	BASIS
PD5935	PIK3R2	cna	gain	BASIS
PD5945	PIK3R2	cna	amp	BASIS
PD5948	PIK3R2	cna	gain	BASIS
PD7067	PIK3R2	cna	gain	BASIS
PD7215	PIK3R2	cna	amp	BASIS
PD9702	PIK3R2	cna	gain	BASIS
PD13299	PIK3R2	cna	gain	BASIS
PD14442	PIK3R2	cna	hetloss	BASIS
PD22355	PIK3R2	cna	gain	BASIS
PD23562	PIK3R2	cna	gain	BASIS
PD23574	PIK3R2	cna	gain	BASIS
PD24186	PIK3R2	cna	gain	BASIS
PD24206	PIK3R2	cna	hetloss	BASIS
PD3890	PIK3R2	cna	hetloss	BASIS
PD3905	PIK3R2	cna	gain	BASIS
PD4006	PIK3R2	cna	amp	BASIS
PD4107	PIK3R2	cna	gain	BASIS
PD4826	PIK3R2	cna	gain	BASIS
PD4967	PIK3R2	cna	hetloss	BASIS
PD5930	PIK3R2	cna	gain	BASIS
PD5935	PIK3R2	cna	gain	BASIS
PD5945	PIK3R2	cna	amp	BASIS
PD5948	PIK3R2	cna	gain	BASIS
PD7067	PIK3R2	cna	gain	BASIS
PD7215	PIK3R2	cna	amp	BASIS
PD9702	PIK3R2	cna	gain	BASIS
TCGA-A2-A25B	EPPK1	cna	gain	TCGA
TCGA-AN-A0XU	EPPK1	cna	hetloss	TCGA
TCGA-AO-A0JL	EPPK1	cna	amp	TCGA
TCGA-BH-A0AW	EPPK1	cna	gain	TCGA
TCGA-BH-A0C0	EPPK1	cna	amp	TCGA
TCGA-BH-A18R	EPPK1	cna	amp	TCGA
TCGA-BH-A1FU	EPPK1	cna	gain	TCGA
TCGA-C8-A12L	EPPK1	cna	gain	TCGA
TCGA-D8-A27M	EPPK1	cna	amp	TCGA
TCGA-E2-A1L7	EPPK1	cna	amp	TCGA
TCGA-E9-A1NC	EPPK1	cna	amp	TCGA
TCGA-LL-A5YP	EPPK1	cna	gain	TCGA
MB-0346	EPPK1	cna	amp	METABRIC
MB-5107	EPPK1	cna	amp	METABRIC
MB-5465	EPPK1	cna	gain	METABRIC
MB-6060	EPPK1	cna	gain	METABRIC

MB-6098	EPPK1	cna	amp	METABRIC
MB-7038	EPPK1	cna	amp	METABRIC
MB-7048	EPPK1	cna	amp	METABRIC
PD10014	EPPK1	cna	gain	BASIS
PD11327	EPPK1	cna	gain	BASIS
PD11742	EPPK1	cna	gain	BASIS
PD13297	EPPK1	cna	gain	BASIS
PD13299	EPPK1	cna	gain	BASIS
PD13771	EPPK1	cna	hetloss	BASIS
PD22355	EPPK1	cna	gain	BASIS
PD23562	EPPK1	cna	amp	BASIS
PD23574	EPPK1	cna	gain	BASIS
PD23578	EPPK1	cna	hetloss	BASIS
PD24186	EPPK1	cna	amp	BASIS
PD24202	EPPK1	cna	hetloss	BASIS
PD24206	EPPK1	cna	amp	BASIS
PD24337	EPPK1	cna	gain	BASIS
PD3890	EPPK1	cna	gain	BASIS
PD3905	EPPK1	cna	gain	BASIS
PD4005	EPPK1	cna	gain	BASIS
PD4006	EPPK1	cna	amp	BASIS
PD4107	EPPK1	cna	gain	BASIS
PD4826	EPPK1	cna	amp	BASIS
PD5930	EPPK1	cna	gain	BASIS
PD5935	EPPK1	cna	amp	BASIS
PD5945	EPPK1	cna	amp	BASIS
PD5948	EPPK1	cna	gain	BASIS
PD6406	EPPK1	cna	amp	BASIS
PD6413	EPPK1	cna	gain	BASIS
PD6731	EPPK1	cna	gain	BASIS
PD7067	EPPK1	cna	amp	BASIS
PD7215	EPPK1	cna	amp	BASIS
PD8621	EPPK1	cna	gain	BASIS
PD8980	EPPK1	cna	amp	BASIS
PD9004	EPPK1	cna	amp	BASIS
PD9585	EPPK1	cna	gain	BASIS
PD9702	EPPK1	cna	amp	BASIS
TCGA-A2-A25B	PLEC	cna	gain	TCGA
TCGA-AN-A0XU	PLEC	cna	hetloss	TCGA
TCGA-AO-A0JL	PLEC	cna	amp	TCGA
TCGA-BH-A0AW	PLEC	cna	gain	TCGA
TCGA-BH-A0C0	PLEC	cna	amp	TCGA
TCGA-BH-A18R	PLEC	cna	amp	TCGA
TCGA-BH-A1FU	PLEC	cna	gain	TCGA
TCGA-C8-A12L	PLEC	cna	gain	TCGA
TCGA-D8-A27M	PLEC	cna	amp	TCGA
TCGA-E2-A1L7	PLEC	cna	amp	TCGA

TCGA-E9-A1NC	PLEC	cna	amp	TCGA
TCGA-LL-A5YP	PLEC	cna	gain	TCGA
MB-0346	PLEC	cna	amp	METABRIC
MB-5107	PLEC	cna	amp	METABRIC
MB-5465	PLEC	cna	gain	METABRIC
MB-6060	PLEC	cna	gain	METABRIC
MB-6098	PLEC	cna	amp	METABRIC
MB-7038	PLEC	cna	amp	METABRIC
MB-7048	PLEC	cna	amp	METABRIC
PD10014	PLEC	cna	gain	BASIS
PD11327	PLEC	cna	gain	BASIS
PD11742	PLEC	cna	gain	BASIS
PD13297	PLEC	cna	gain	BASIS
PD13299	PLEC	cna	gain	BASIS
PD13771	PLEC	cna	hetloss	BASIS
PD22355	PLEC	cna	gain	BASIS
PD23562	PLEC	cna	amp	BASIS
PD23574	PLEC	cna	gain	BASIS
PD23578	PLEC	cna	hetloss	BASIS
PD24186	PLEC	cna	amp	BASIS
PD24202	PLEC	cna	hetloss	BASIS
PD24206	PLEC	cna	amp	BASIS
PD24337	PLEC	cna	gain	BASIS
PD3890	PLEC	cna	gain	BASIS
PD3905	PLEC	cna	gain	BASIS
PD4005	PLEC	cna	gain	BASIS
PD4006	PLEC	cna	amp	BASIS
PD4107	PLEC	cna	gain	BASIS
PD4826	PLEC	cna	amp	BASIS
PD5930	PLEC	cna	gain	BASIS
PD5935	PLEC	cna	amp	BASIS
PD5945	PLEC	cna	amp	BASIS
PD5948	PLEC	cna	gain	BASIS
PD6406	PLEC	cna	amp	BASIS
PD6413	PLEC	cna	gain	BASIS
PD6731	PLEC	cna	gain	BASIS
PD7067	PLEC	cna	amp	BASIS
PD7215	PLEC	cna	amp	BASIS
PD8621	PLEC	cna	gain	BASIS
PD8980	PLEC	cna	amp	BASIS
PD9004	PLEC	cna	amp	BASIS
PD9585	PLEC	cna	gain	BASIS
PD9702	PLEC	cna	amp	BASIS
TCGA-A2-A25B	H3F3A	cna	amp	TCGA
TCGA-AN-A0XU	H3F3A	cna	gain	TCGA
TCGA-AO-A0JL	H3F3A	cna	gain	TCGA
TCGA-BH-A0AW	H3F3A	cna	gain	TCGA

TCGA-BH-A0C0	H3F3A	cna	gain	TCGA
TCGA-BH-A18R	H3F3A	cna	amp	TCGA
TCGA-BH-A1FU	H3F3A	cna	gain	TCGA
TCGA-C8-A12L	H3F3A	cna	amp	TCGA
TCGA-E2-A1L7	H3F3A	cna	gain	TCGA
TCGA-E9-A1NC	H3F3A	cna	gain	TCGA
TCGA-EW-A1OX	H3F3A	cna	gain	TCGA
TCGA-LL-A5YP	H3F3A	cna	gain	TCGA
MB-0346	H3F3A	cna	gain	METABRIC
MB-2827	H3F3A	cna	amp	METABRIC
MB-5070	H3F3A	cna	gain	METABRIC
MB-5107	H3F3A	cna	gain	METABRIC
MB-5465	H3F3A	cna	gain	METABRIC
MB-6060	H3F3A	cna	gain	METABRIC
MB-6098	H3F3A	cna	gain	METABRIC
MB-7032	H3F3A	cna	gain	METABRIC
MB-7038	H3F3A	cna	amp	METABRIC
MB-7048	H3F3A	cna	gain	METABRIC
MB-0420	H3F3A	cna	gain	METABRIC
PD10014	H3F3A	cna	gain	BASIS
PD11742	H3F3A	cna	gain	BASIS
PD13296	H3F3A	cna	hetloss	BASIS
PD13299	H3F3A	cna	gain	BASIS
PD13771	H3F3A	cna	hetloss	BASIS
PD14442	H3F3A	cna	gain	BASIS
PD22355	H3F3A	cna	gain	BASIS
PD23562	H3F3A	cna	gain	BASIS
PD23574	H3F3A	cna	gain	BASIS
PD24186	H3F3A	cna	amp	BASIS
PD24202	H3F3A	cna	gain	BASIS
PD24206	H3F3A	cna	gain	BASIS
PD3890	H3F3A	cna	gain	BASIS
PD3905	H3F3A	cna	gain	BASIS
PD4005	H3F3A	cna	gain	BASIS
PD4006	H3F3A	cna	amp	BASIS
PD4107	H3F3A	cna	gain	BASIS
PD4826	H3F3A	cna	gain	BASIS
PD5935	H3F3A	cna	gain	BASIS
PD5945	H3F3A	cna	amp	BASIS
PD5948	H3F3A	cna	gain	BASIS
PD6413	H3F3A	cna	gain	BASIS
PD7067	H3F3A	cna	gain	BASIS
PD7215	H3F3A	cna	amp	BASIS
PD8621	H3F3A	cna	gain	BASIS
PD8980	H3F3A	cna	amp	BASIS
PD9004	H3F3A	cna	gain	BASIS
PD9585	H3F3A	cna	hetloss	BASIS

PD9702	H3F3A	cna	amp	BASIS
TCGA-A2-A25B	NBN	cna	gain	TCGA
TCGA-AO-A0JL	NBN	cna	amp	TCGA
TCGA-BH-A0AW	NBN	cna	gain	TCGA
TCGA-BH-A0C0	NBN	cna	amp	TCGA
TCGA-BH-A18R	NBN	cna	amp	TCGA
TCGA-BH-A1FU	NBN	cna	gain	TCGA
TCGA-C8-A12L	NBN	cna	gain	TCGA
TCGA-D8-A27M	NBN	cna	amp	TCGA
TCGA-E2-A1L7	NBN	cna	gain	TCGA
TCGA-E9-A1NC	NBN	cna	amp	TCGA
TCGA-LL-A5YP	NBN	cna	amp	TCGA
MB-0346	NBN	cna	amp	METABRIC
MB-2827	NBN	cna	amp	METABRIC
MB-5107	NBN	cna	gain	METABRIC
MB-5465	NBN	cna	hetloss	METABRIC
MB-6060	NBN	cna	gain	METABRIC
MB-6098	NBN	cna	gain	METABRIC
MB-7038	NBN	cna	gain	METABRIC
MB-7048	NBN	cna	gain	METABRIC
MB-0420	NBN	cna	gain	METABRIC
P-0002237-T01-IM3	NBN	cna	amp	MSK-IMPACT
P-0010758-T01-IM5	NBN	cna	amp	MSK-IMPACT
PD10014	NBN	cna	amp	BASIS
PD11327	NBN	cna	amp	BASIS
PD11742	NBN	cna	gain	BASIS
PD13296	NBN	cna	hetloss	BASIS
PD13299	NBN	cna	amp	BASIS
PD13771	NBN	cna	gain	BASIS
PD23562	NBN	cna	amp	BASIS
PD23574	NBN	cna	gain	BASIS
PD23578	NBN	cna	gain	BASIS
PD24186	NBN	cna	gain	BASIS
PD24202	NBN	cna	gain	BASIS
PD24206	NBN	cna	amp	BASIS
PD3890	NBN	cna	gain	BASIS
PD3905	NBN	cna	gain	BASIS
PD4005	NBN	cna	hetloss	BASIS
PD4006	NBN	cna	gain	BASIS
PD4826	NBN	cna	amp	BASIS
PD5930	NBN	cna	gain	BASIS
PD5935	NBN	cna	gain	BASIS
PD5945	NBN	cna	gain	BASIS
PD5948	NBN	cna	gain	BASIS
PD6406	NBN	cna	gain	BASIS
PD6413	NBN	cna	gain	BASIS
PD7067	NBN	cna	amp	BASIS

PD7215	NBN	cna	amp	BASIS
PD8621	NBN	cna	gain	BASIS
PD8980	NBN	cna	gain	BASIS
PD9004	NBN	cna	gain	BASIS
PD9585	NBN	cna	gain	BASIS
PD9702	NBN	cna	amp	BASIS
TCGA-A2-A25B	NLRP3	cna	amp	TCGA
TCGA-AO-A0JL	NLRP3	cna	amp	TCGA
TCGA-BH-A0AW	NLRP3	cna	gain	TCGA
TCGA-BH-A0C0	NLRP3	cna	hetloss	TCGA
TCGA-BH-A18R	NLRP3	cna	amp	TCGA
TCGA-BH-A1FU	NLRP3	cna	gain	TCGA
TCGA-C8-A12L	NLRP3	cna	amp	TCGA
TCGA-D8-A27M	NLRP3	cna	hetloss	TCGA
TCGA-E2-A1L7	NLRP3	cna	hetloss	TCGA
TCGA-E9-A1NC	NLRP3	cna	gain	TCGA
TCGA-EW-A1OX	NLRP3	cna	gain	TCGA
TCGA-LL-A5YP	NLRP3	cna	gain	TCGA
MB-0346	NLRP3	cna	gain	METABRIC
MB-5465	NLRP3	cna	gain	METABRIC
MB-6060	NLRP3	cna	gain	METABRIC
MB-6098	NLRP3	cna	amp	METABRIC
MB-7032	NLRP3	cna	gain	METABRIC
MB-7038	NLRP3	cna	amp	METABRIC
MB-7048	NLRP3	cna	gain	METABRIC
MB-0420	NLRP3	cna	gain	METABRIC
MTS-T0064	NLRP3	cna	hetloss	METABRIC
PD10014	NLRP3	cna	gain	BASIS
PD11742	NLRP3	cna	gain	BASIS
PD13296	NLRP3	cna	gain	BASIS
PD13299	NLRP3	cna	gain	BASIS
PD13771	NLRP3	cna	hetloss	BASIS
PD14442	NLRP3	cna	gain	BASIS
PD22355	NLRP3	cna	gain	BASIS
PD23562	NLRP3	cna	gain	BASIS
PD23574	NLRP3	cna	gain	BASIS
PD24186	NLRP3	cna	amp	BASIS
PD24202	NLRP3	cna	gain	BASIS
PD24206	NLRP3	cna	gain	BASIS
PD24337	NLRP3	cna	hetloss	BASIS
PD3890	NLRP3	cna	gain	BASIS
PD3905	NLRP3	cna	gain	BASIS
PD4005	NLRP3	cna	gain	BASIS
PD4006	NLRP3	cna	amp	BASIS
PD4107	NLRP3	cna	gain	BASIS
PD4826	NLRP3	cna	gain	BASIS
PD5930	NLRP3	cna	gain	BASIS

PD5935	NLRP3	cna	gain	BASIS
PD5945	NLRP3	cna	amp	BASIS
PD5948	NLRP3	cna	amp	BASIS
PD6406	NLRP3	cna	gain	BASIS
PD7067	NLRP3	cna	gain	BASIS
PD7215	NLRP3	cna	amp	BASIS
PD8621	NLRP3	cna	gain	BASIS
PD8980	NLRP3	cna	amp	BASIS
PD9004	NLRP3	cna	gain	BASIS
PD9585	NLRP3	cna	gain	BASIS
PD9702	NLRP3	cna	amp	BASIS
TCGA-A2-A25B	OBSCN	cna	amp	TCGA
TCGA-AN-A0XU	OBSCN	cna	gain	TCGA
TCGA-AO-A0JL	OBSCN	cna	gain	TCGA
TCGA-BH-A0AW	OBSCN	cna	gain	TCGA
TCGA-BH-A0C0	OBSCN	cna	gain	TCGA
TCGA-BH-A18R	OBSCN	cna	amp	TCGA
TCGA-BH-A1FU	OBSCN	cna	gain	TCGA
TCGA-C8-A12L	OBSCN	cna	amp	TCGA
TCGA-E2-A1L7	OBSCN	cna	gain	TCGA
TCGA-E9-A1NC	OBSCN	cna	gain	TCGA
TCGA-EW-A1OX	OBSCN	cna	gain	TCGA
TCGA-LL-A5YP	OBSCN	cna	gain	TCGA
MB-0346	OBSCN	cna	gain	METABRIC
MB-2827	OBSCN	cna	amp	METABRIC
MB-5070	OBSCN	cna	gain	METABRIC
MB-5107	OBSCN	cna	gain	METABRIC
MB-5465	OBSCN	cna	gain	METABRIC
MB-6060	OBSCN	cna	gain	METABRIC
MB-6098	OBSCN	cna	gain	METABRIC
MB-7032	OBSCN	cna	gain	METABRIC
MB-7038	OBSCN	cna	amp	METABRIC
MB-7048	OBSCN	cna	gain	METABRIC
MB-0420	OBSCN	cna	gain	METABRIC
PD10014	OBSCN	cna	gain	BASIS
PD11742	OBSCN	cna	gain	BASIS
PD13296	OBSCN	cna	hetloss	BASIS
PD13299	OBSCN	cna	gain	BASIS
PD13771	OBSCN	cna	hetloss	BASIS
PD14442	OBSCN	cna	gain	BASIS
PD22355	OBSCN	cna	gain	BASIS
PD23562	OBSCN	cna	gain	BASIS
PD23574	OBSCN	cna	gain	BASIS
PD24186	OBSCN	cna	amp	BASIS
PD24202	OBSCN	cna	gain	BASIS
PD24206	OBSCN	cna	gain	BASIS
PD3890	OBSCN	cna	gain	BASIS

PD3905	OBSCN	cna	gain	BASIS
PD4005	OBSCN	cna	gain	BASIS
PD4006	OBSCN	cna	amp	BASIS
PD4107	OBSCN	cna	gain	BASIS
PD4826	OBSCN	cna	gain	BASIS
PD5935	OBSCN	cna	gain	BASIS
PD5945	OBSCN	cna	amp	BASIS
PD5948	OBSCN	cna	gain	BASIS
PD6413	OBSCN	cna	gain	BASIS
PD7067	OBSCN	cna	gain	BASIS
PD7215	OBSCN	cna	amp	BASIS
PD8621	OBSCN	cna	gain	BASIS
PD8980	OBSCN	cna	amp	BASIS
PD9004	OBSCN	cna	gain	BASIS
PD9585	OBSCN	cna	hetloss	BASIS
PD9702	OBSCN	cna	amp	BASIS
TCGA-A2-A25B	PARP1	cna	amp	TCGA
TCGA-AN-A0XU	PARP1	cna	gain	TCGA
TCGA-AO-A0JL	PARP1	cna	gain	TCGA
TCGA-BH-A0AW	PARP1	cna	gain	TCGA
TCGA-BH-A0C0	PARP1	cna	gain	TCGA
TCGA-BH-A18R	PARP1	cna	amp	TCGA
TCGA-BH-A1FU	PARP1	cna	gain	TCGA
TCGA-C8-A12L	PARP1	cna	amp	TCGA
TCGA-E2-A1L7	PARP1	cna	gain	TCGA
TCGA-E9-A1NC	PARP1	cna	gain	TCGA
TCGA-EW-A1OX	PARP1	cna	gain	TCGA
TCGA-LL-A5YP	PARP1	cna	gain	TCGA
MB-0346	PARP1	cna	gain	METABRIC
MB-2827	PARP1	cna	amp	METABRIC
MB-5070	PARP1	cna	gain	METABRIC
MB-5107	PARP1	cna	gain	METABRIC
MB-5465	PARP1	cna	gain	METABRIC
MB-6060	PARP1	cna	gain	METABRIC
MB-6098	PARP1	cna	gain	METABRIC
MB-7032	PARP1	cna	gain	METABRIC
MB-7038	PARP1	cna	amp	METABRIC
MB-7048	PARP1	cna	gain	METABRIC
MB-0420	PARP1	cna	gain	METABRIC
PD10014	PARP1	cna	gain	BASIS
PD11742	PARP1	cna	gain	BASIS
PD13296	PARP1	cna	hetloss	BASIS
PD13299	PARP1	cna	gain	BASIS
PD13771	PARP1	cna	hetloss	BASIS
PD14442	PARP1	cna	gain	BASIS
PD22355	PARP1	cna	gain	BASIS
PD23562	PARP1	cna	gain	BASIS

PD23574	PARP1	cna	gain	BASIS
PD24186	PARP1	cna	amp	BASIS
PD24202	PARP1	cna	gain	BASIS
PD24206	PARP1	cna	gain	BASIS
PD3890	PARP1	cna	gain	BASIS
PD3905	PARP1	cna	gain	BASIS
PD4005	PARP1	cna	gain	BASIS
PD4006	PARP1	cna	amp	BASIS
PD4107	PARP1	cna	gain	BASIS
PD4826	PARP1	cna	gain	BASIS
PD5935	PARP1	cna	gain	BASIS
PD5945	PARP1	cna	amp	BASIS
PD5948	PARP1	cna	gain	BASIS
PD6413	PARP1	cna	gain	BASIS
PD7067	PARP1	cna	gain	BASIS
PD7215	PARP1	cna	amp	BASIS
PD8621	PARP1	cna	gain	BASIS
PD8980	PARP1	cna	amp	BASIS
PD9004	PARP1	cna	gain	BASIS
PD9585	PARP1	cna	hetloss	BASIS
PD9702	PARP1	cna	amp	BASIS
TCGA-A2-A25B	B4GALT3	cna	gain	TCGA
TCGA-AN-A0XU	B4GALT3	cna	gain	TCGA
TCGA-AO-A0JL	B4GALT3	cna	gain	TCGA
TCGA-BH-A0AW	B4GALT3	cna	gain	TCGA
TCGA-BH-A0C0	B4GALT3	cna	gain	TCGA
TCGA-BH-A18R	B4GALT3	cna	gain	TCGA
TCGA-BH-A1FU	B4GALT3	cna	gain	TCGA
TCGA-C8-A12L	B4GALT3	cna	gain	TCGA
TCGA-D8-A27M	B4GALT3	cna	amp	TCGA
TCGA-E2-A1L7	B4GALT3	cna	gain	TCGA
TCGA-EW-A1OX	B4GALT3	cna	gain	TCGA
TCGA-LL-A5YP	B4GALT3	cna	amp	TCGA
MB-0346	B4GALT3	cna	gain	METABRIC
MB-2827	B4GALT3	cna	amp	METABRIC
MB-5070	B4GALT3	cna	gain	METABRIC
MB-5465	B4GALT3	cna	amp	METABRIC
MB-6060	B4GALT3	cna	gain	METABRIC
MB-6098	B4GALT3	cna	amp	METABRIC
MB-7032	B4GALT3	cna	gain	METABRIC
MB-7038	B4GALT3	cna	gain	METABRIC
MB-0420	B4GALT3	cna	gain	METABRIC
MTS-T0064	B4GALT3	cna	amp	METABRIC
PD10014	B4GALT3	cna	gain	BASIS
PD11327	B4GALT3	cna	gain	BASIS
PD13297	B4GALT3	cna	gain	BASIS
PD13299	B4GALT3	cna	gain	BASIS

PD14442	B4GALT3	cna	gain	BASIS
PD22355	B4GALT3	cna	gain	BASIS
PD23562	B4GALT3	cna	amp	BASIS
PD23574	B4GALT3	cna	amp	BASIS
PD24186	B4GALT3	cna	gain	BASIS
PD24206	B4GALT3	cna	gain	BASIS
PD24337	B4GALT3	cna	gain	BASIS
PD3890	B4GALT3	cna	gain	BASIS
PD3905	B4GALT3	cna	gain	BASIS
PD4005	B4GALT3	cna	gain	BASIS
PD4006	B4GALT3	cna	gain	BASIS
PD4107	B4GALT3	cna	gain	BASIS
PD4826	B4GALT3	cna	gain	BASIS
PD5930	B4GALT3	cna	gain	BASIS
PD5935	B4GALT3	cna	gain	BASIS
PD5945	B4GALT3	cna	amp	BASIS
PD5948	B4GALT3	cna	gain	BASIS
PD6406	B4GALT3	cna	gain	BASIS
PD6413	B4GALT3	cna	gain	BASIS
PD7067	B4GALT3	cna	amp	BASIS
PD7215	B4GALT3	cna	gain	BASIS
PD8621	B4GALT3	cna	amp	BASIS
PD8980	B4GALT3	cna	gain	BASIS
PD9004	B4GALT3	cna	gain	BASIS
PD9702	B4GALT3	cna	gain	BASIS
TCGA-AN-A0XU	KMT2B	cna	gain	TCGA
TCGA-AO-A0JL	KMT2B	cna	hetloss	TCGA
TCGA-BH-A0AW	KMT2B	cna	gain	TCGA
TCGA-BH-A0C0	KMT2B	cna	hetloss	TCGA
TCGA-BH-A1FU	KMT2B	cna	hetloss	TCGA
TCGA-C8-A12L	KMT2B	cna	gain	TCGA
TCGA-D8-A27M	KMT2B	cna	gain	TCGA
TCGA-EW-A1OX	KMT2B	cna	hetloss	TCGA
TCGA-LL-A5YP	KMT2B	cna	gain	TCGA
MB-0346	KMT2B	cna	hetloss	METABRIC
MB-6098	KMT2B	cna	gain	METABRIC
MB-7038	KMT2B	cna	gain	METABRIC
MB-7048	KMT2B	cna	hetloss	METABRIC
PD13296	KMT2B	cna	gain	BASIS
PD13299	KMT2B	cna	gain	BASIS
PD23562	KMT2B	cna	gain	BASIS
PD23574	KMT2B	cna	gain	BASIS
PD24186	KMT2B	cna	gain	BASIS
PD3905	KMT2B	cna	gain	BASIS
PD4006	KMT2B	cna	gain	BASIS
PD4826	KMT2B	cna	gain	BASIS
PD5935	KMT2B	cna	gain	BASIS

PD5945	KMT2B	cna	gain	BASIS
PD5948	KMT2B	cna	gain	BASIS
PD6406	KMT2B	cna	gain	BASIS
PD7067	KMT2B	cna	gain	BASIS
PD7215	KMT2B	cna	gain	BASIS
PD8621	KMT2B	cna	gain	BASIS
PD8980	KMT2B	cna	hetloss	BASIS
PD9004	KMT2B	cna	hetloss	BASIS
PD9585	KMT2B	cna	gain	BASIS
PD9702	KMT2B	cna	gain	BASIS
PD13296	KMT2B	cna	gain	BASIS
PD13299	KMT2B	cna	gain	BASIS
PD23562	KMT2B	cna	gain	BASIS
PD23574	KMT2B	cna	gain	BASIS
PD24186	KMT2B	cna	gain	BASIS
PD3905	KMT2B	cna	gain	BASIS
PD4006	KMT2B	cna	gain	BASIS
PD4826	KMT2B	cna	gain	BASIS
PD5935	KMT2B	cna	gain	BASIS
PD5945	KMT2B	cna	gain	BASIS
PD5948	KMT2B	cna	gain	BASIS
PD6406	KMT2B	cna	gain	BASIS
PD7067	KMT2B	cna	gain	BASIS
PD7215	KMT2B	cna	gain	BASIS
PD8621	KMT2B	cna	gain	BASIS
PD8980	KMT2B	cna	hetloss	BASIS
PD9004	KMT2B	cna	hetloss	BASIS
PD9585	KMT2B	cna	gain	BASIS
PD9702	KMT2B	cna	gain	BASIS
TCGA-A2-A25B	SDHC	cna	gain	TCGA
TCGA-AN-A0XU	SDHC	cna	gain	TCGA
TCGA-AO-A0JL	SDHC	cna	gain	TCGA
TCGA-BH-A0AW	SDHC	cna	gain	TCGA
TCGA-BH-A0C0	SDHC	cna	gain	TCGA
TCGA-BH-A18R	SDHC	cna	gain	TCGA
TCGA-BH-A1FU	SDHC	cna	gain	TCGA
TCGA-C8-A12L	SDHC	cna	gain	TCGA
TCGA-D8-A27M	SDHC	cna	amp	TCGA
TCGA-E2-A1L7	SDHC	cna	gain	TCGA
TCGA-EW-A1OX	SDHC	cna	gain	TCGA
TCGA-LL-A5YP	SDHC	cna	amp	TCGA
MB-0346	SDHC	cna	gain	METABRIC
MB-2827	SDHC	cna	amp	METABRIC
MB-5070	SDHC	cna	gain	METABRIC
MB-5465	SDHC	cna	amp	METABRIC
MB-6060	SDHC	cna	gain	METABRIC
MB-6098	SDHC	cna	amp	METABRIC

MB-7032	SDHC	cna	gain	METABRIC
MB-7038	SDHC	cna	gain	METABRIC
MB-0420	SDHC	cna	gain	METABRIC
MTS-T0064	SDHC	cna	amp	METABRIC
PD10014	SDHC	cna	gain	BASIS
PD11327	SDHC	cna	gain	BASIS
PD13297	SDHC	cna	gain	BASIS
PD13299	SDHC	cna	gain	BASIS
PD14442	SDHC	cna	gain	BASIS
PD22355	SDHC	cna	gain	BASIS
PD23562	SDHC	cna	amp	BASIS
PD23574	SDHC	cna	amp	BASIS
PD24186	SDHC	cna	gain	BASIS
PD24206	SDHC	cna	gain	BASIS
PD24337	SDHC	cna	gain	BASIS
PD3890	SDHC	cna	gain	BASIS
PD3905	SDHC	cna	gain	BASIS
PD4005	SDHC	cna	gain	BASIS
PD4006	SDHC	cna	gain	BASIS
PD4107	SDHC	cna	gain	BASIS
PD4826	SDHC	cna	gain	BASIS
PD5930	SDHC	cna	gain	BASIS
PD5935	SDHC	cna	gain	BASIS
PD5945	SDHC	cna	amp	BASIS
PD5948	SDHC	cna	gain	BASIS
PD6406	SDHC	cna	gain	BASIS
PD6413	SDHC	cna	gain	BASIS
PD7067	SDHC	cna	amp	BASIS
PD7215	SDHC	cna	gain	BASIS
PD8621	SDHC	cna	amp	BASIS
PD8980	SDHC	cna	gain	BASIS
PD9004	SDHC	cna	gain	BASIS
PD9702	SDHC	cna	gain	BASIS
TCGA-A2-A25B	COX6C	cna	gain	TCGA
TCGA-AN-A0XU	COX6C	cna	gain	TCGA
TCGA-AO-A0JL	COX6C	cna	amp	TCGA
TCGA-BH-A0AW	COX6C	cna	gain	TCGA
TCGA-BH-A0C0	COX6C	cna	amp	TCGA
TCGA-BH-A18R	COX6C	cna	amp	TCGA
TCGA-BH-A1FU	COX6C	cna	gain	TCGA
TCGA-C8-A12L	COX6C	cna	gain	TCGA
TCGA-D8-A27M	COX6C	cna	amp	TCGA
TCGA-E2-A1L7	COX6C	cna	gain	TCGA
TCGA-E9-A1NC	COX6C	cna	amp	TCGA
TCGA-LL-A5YP	COX6C	cna	amp	TCGA
MB-0346	COX6C	cna	amp	METABRIC
MB-2827	COX6C	cna	amp	METABRIC

MB-5107	COX6C	cna	gain	METABRIC
MB-6060	COX6C	cna	amp	METABRIC
MB-6098	COX6C	cna	amp	METABRIC
MB-7038	COX6C	cna	gain	METABRIC
MB-7048	COX6C	cna	gain	METABRIC
MB-0420	COX6C	cna	gain	METABRIC
PD10014	COX6C	cna	amp	BASIS
PD11327	COX6C	cna	amp	BASIS
PD11742	COX6C	cna	gain	BASIS
PD13296	COX6C	cna	hetloss	BASIS
PD13299	COX6C	cna	amp	BASIS
PD13771	COX6C	cna	gain	BASIS
PD23562	COX6C	cna	amp	BASIS
PD23574	COX6C	cna	amp	BASIS
PD23578	COX6C	cna	gain	BASIS
PD24186	COX6C	cna	amp	BASIS
PD24206	COX6C	cna	amp	BASIS
PD24337	COX6C	cna	gain	BASIS
PD3890	COX6C	cna	gain	BASIS
PD3905	COX6C	cna	gain	BASIS
PD4006	COX6C	cna	gain	BASIS
PD4107	COX6C	cna	gain	BASIS
PD4826	COX6C	cna	amp	BASIS
PD5930	COX6C	cna	gain	BASIS
PD5935	COX6C	cna	gain	BASIS
PD5945	COX6C	cna	gain	BASIS
PD5948	COX6C	cna	amp	BASIS
PD6406	COX6C	cna	gain	BASIS
PD6413	COX6C	cna	gain	BASIS
PD7067	COX6C	cna	amp	BASIS
PD7215	COX6C	cna	amp	BASIS
PD8621	COX6C	cna	gain	BASIS
PD8980	COX6C	cna	gain	BASIS
PD9004	COX6C	cna	amp	BASIS
PD9585	COX6C	cna	gain	BASIS
PD9702	COX6C	cna	amp	BASIS
TCGA-A2-A25B	AKT3	cna	amp	TCGA
TCGA-AO-A0JL	AKT3	cna	amp	TCGA
TCGA-BH-A0AW	AKT3	cna	gain	TCGA
TCGA-BH-A0C0	AKT3	cna	gain	TCGA
TCGA-BH-A18R	AKT3	cna	amp	TCGA
TCGA-BH-A1FU	AKT3	cna	gain	TCGA
TCGA-C8-A12L	AKT3	cna	amp	TCGA
TCGA-E9-A1NC	AKT3	cna	gain	TCGA
TCGA-EW-A1OX	AKT3	cna	gain	TCGA
TCGA-LL-A5YP	AKT3	cna	gain	TCGA
MB-0346	AKT3	cna	gain	METABRIC

MB-5070	AKT3	cna	gain	METABRIC
MB-5465	AKT3	cna	gain	METABRIC
MB-6060	AKT3	cna	gain	METABRIC
MB-6098	AKT3	cna	amp	METABRIC
MB-7032	AKT3	cna	gain	METABRIC
MB-7038	AKT3	cna	amp	METABRIC
MB-7048	AKT3	cna	gain	METABRIC
MB-0420	AKT3	cna	gain	METABRIC
PD10014	AKT3	cna	gain	BASIS
PD11742	AKT3	cna	gain	BASIS
PD13299	AKT3	cna	gain	BASIS
PD13771	AKT3	cna	hetloss	BASIS
PD14442	AKT3	cna	gain	BASIS
PD22355	AKT3	cna	gain	BASIS
PD23562	AKT3	cna	gain	BASIS
PD23574	AKT3	cna	gain	BASIS
PD24186	AKT3	cna	amp	BASIS
PD24202	AKT3	cna	gain	BASIS
PD24337	AKT3	cna	hetloss	BASIS
PD3890	AKT3	cna	gain	BASIS
PD3905	AKT3	cna	gain	BASIS
PD4005	AKT3	cna	gain	BASIS
PD4006	AKT3	cna	amp	BASIS
PD4107	AKT3	cna	gain	BASIS
PD4826	AKT3	cna	gain	BASIS
PD5930	AKT3	cna	gain	BASIS
PD5935	AKT3	cna	gain	BASIS
PD5945	AKT3	cna	amp	BASIS
PD5948	AKT3	cna	amp	BASIS
PD6406	AKT3	cna	gain	BASIS
PD6413	AKT3	cna	hetloss	BASIS
PD7067	AKT3	cna	gain	BASIS
PD7215	AKT3	cna	amp	BASIS
PD8621	AKT3	cna	gain	BASIS
PD8980	AKT3	cna	amp	BASIS
PD9004	AKT3	cna	gain	BASIS
PD9585	AKT3	cna	gain	BASIS
PD9702	AKT3	cna	amp	BASIS
TCGA-A2-A25B	BRCA1	cna	hetloss	TCGA
TCGA-AN-A0XU	BRCA1	cna	hetloss	TCGA
TCGA-AO-A0JL	BRCA1	cna	homdel	TCGA
TCGA-BH-A0AW	BRCA1	cna	homdel	TCGA
TCGA-BH-A0C0	BRCA1	cna	homdel	TCGA
TCGA-BH-A18R	BRCA1	cna	homdel	TCGA
TCGA-BH-A1FU	BRCA1	cna	homdel	TCGA
TCGA-C8-A12L	BRCA1	cna	homdel	TCGA
TCGA-D8-A27M	BRCA1	cna	hetloss	TCGA

TCGA-E2-A1L7	BRCA1	cna	homdel	TCGA
TCGA-E9-A1NC	BRCA1	cna	hetloss	TCGA
TCGA-EW-A1OX	BRCA1	cna	homdel	TCGA
MB-0346	BRCA1	cna	hetloss	METABRIC
MB-2827	BRCA1	cna	homdel	METABRIC
MB-5070	BRCA1	cna	hetloss	METABRIC
MB-5465	BRCA1	cna	hetloss	METABRIC
MB-6060	BRCA1	cna	hetloss	METABRIC
MB-6098	BRCA1	cna	hetloss	METABRIC
MB-6271	BRCA1	cna	hetloss	METABRIC
MB-7038	BRCA1	cna	hetloss	METABRIC
MB-7048	BRCA1	cna	hetloss	METABRIC
MB-0420	BRCA1	cna	hetloss	METABRIC
P-0002023-T01-IM3	BRCA1	cna	homdel	MSK-IMPACT
P-0002591-T01-IM3	BRCA1	cna	homdel	MSK-IMPACT
P-0009557-T01-IM5	BRCA1	cna	homdel	MSK-IMPACT
PD13296	BRCA1	cna	gain	BASIS
PD13297	BRCA1	cna	hetloss	BASIS
PD13299	BRCA1	cna	gain	BASIS
PD13771	BRCA1	cna	hetloss	BASIS
PD23561	BRCA1	cna	hetloss	BASIS
PD23562	BRCA1	cna	gain	BASIS
PD23574	BRCA1	cna	gain	BASIS
PD24186	BRCA1	cna	gain	BASIS
PD24206	BRCA1	cna	homdel	BASIS
PD24337	BRCA1	cna	hetloss	BASIS
PD3890	BRCA1	cna	hetloss	BASIS
PD3905	BRCA1	cna	gain	BASIS
PD4006	BRCA1	cna	gain	BASIS
PD4826	BRCA1	cna	homdel	BASIS
PD4967	BRCA1	cna	homdel	BASIS
PD5945	BRCA1	cna	amp	BASIS
PD5948	BRCA1	cna	gain	BASIS
PD6406	BRCA1	cna	hetloss	BASIS
PD6731	BRCA1	cna	hetloss	BASIS
PD7067	BRCA1	cna	gain	BASIS
PD7215	BRCA1	cna	gain	BASIS
PD9004	BRCA1	cna	gain	BASIS
PD9585	BRCA1	cna	hetloss	BASIS
PD9702	BRCA1	cna	amp	BASIS
TCGA-A2-A25B	FH	cna	amp	TCGA
TCGA-AO-A0JL	FH	cna	gain	TCGA
TCGA-BH-A0AW	FH	cna	gain	TCGA
TCGA-BH-A0C0	FH	cna	gain	TCGA
TCGA-BH-A18R	FH	cna	amp	TCGA
TCGA-BH-A1FU	FH	cna	gain	TCGA
TCGA-C8-A12L	FH	cna	amp	TCGA

TCGA-E2-A1L7	FH	cna	hetloss	TCGA
TCGA-E9-A1NC	FH	cna	gain	TCGA
TCGA-EW-A1OX	FH	cna	gain	TCGA
TCGA-LL-A5YP	FH	cna	gain	TCGA
MB-0346	FH	cna	gain	METABRIC
MB-5070	FH	cna	gain	METABRIC
MB-5465	FH	cna	gain	METABRIC
MB-6060	FH	cna	gain	METABRIC
MB-6098	FH	cna	amp	METABRIC
MB-7032	FH	cna	gain	METABRIC
MB-7038	FH	cna	amp	METABRIC
MB-7048	FH	cna	gain	METABRIC
MB-0420	FH	cna	gain	METABRIC
PD10014	FH	cna	gain	BASIS
PD11742	FH	cna	gain	BASIS
PD13299	FH	cna	gain	BASIS
PD13771	FH	cna	hetloss	BASIS
PD14442	FH	cna	gain	BASIS
PD22355	FH	cna	gain	BASIS
PD23562	FH	cna	gain	BASIS
PD23574	FH	cna	gain	BASIS
PD24186	FH	cna	amp	BASIS
PD24202	FH	cna	gain	BASIS
PD24337	FH	cna	hetloss	BASIS
PD3890	FH	cna	gain	BASIS
PD3905	FH	cna	gain	BASIS
PD4005	FH	cna	gain	BASIS
PD4006	FH	cna	amp	BASIS
PD4107	FH	cna	gain	BASIS
PD4826	FH	cna	gain	BASIS
PD5935	FH	cna	gain	BASIS
PD5945	FH	cna	amp	BASIS
PD5948	FH	cna	amp	BASIS
PD6406	FH	cna	gain	BASIS
PD6413	FH	cna	gain	BASIS
PD7067	FH	cna	gain	BASIS
PD7215	FH	cna	amp	BASIS
PD8621	FH	cna	gain	BASIS
PD8980	FH	cna	amp	BASIS
PD9004	FH	cna	gain	BASIS
PD9585	FH	cna	gain	BASIS
PD9702	FH	cna	amp	BASIS
TCGA-A2-A25B	RUNX1T1	cna	gain	TCGA
TCGA-AO-A0JL	RUNX1T1	cna	amp	TCGA
TCGA-BH-A0AW	RUNX1T1	cna	gain	TCGA
TCGA-BH-A0C0	RUNX1T1	cna	amp	TCGA
TCGA-BH-A18R	RUNX1T1	cna	amp	TCGA

TCGA-BH-A1FU	RUNX1T1	cna	gain	TCGA
TCGA-C8-A12L	RUNX1T1	cna	gain	TCGA
TCGA-D8-A27M	RUNX1T1	cna	amp	TCGA
TCGA-E2-A1L7	RUNX1T1	cna	gain	TCGA
TCGA-E9-A1NC	RUNX1T1	cna	amp	TCGA
TCGA-LL-A5YP	RUNX1T1	cna	amp	TCGA
MB-0346	RUNX1T1	cna	amp	METABRIC
MB-2827	RUNX1T1	cna	amp	METABRIC
MB-5107	RUNX1T1	cna	gain	METABRIC
MB-5465	RUNX1T1	cna	hetloss	METABRIC
MB-6060	RUNX1T1	cna	amp	METABRIC
MB-6098	RUNX1T1	cna	gain	METABRIC
MB-7038	RUNX1T1	cna	gain	METABRIC
MB-7048	RUNX1T1	cna	gain	METABRIC
MB-0420	RUNX1T1	cna	gain	METABRIC
PD10014	RUNX1T1	cna	amp	BASIS
PD11327	RUNX1T1	cna	amp	BASIS
PD11742	RUNX1T1	cna	gain	BASIS
PD13296	RUNX1T1	cna	hetloss	BASIS
PD13299	RUNX1T1	cna	amp	BASIS
PD13771	RUNX1T1	cna	gain	BASIS
PD23562	RUNX1T1	cna	amp	BASIS
PD23574	RUNX1T1	cna	amp	BASIS
PD23578	RUNX1T1	cna	gain	BASIS
PD24186	RUNX1T1	cna	gain	BASIS
PD24206	RUNX1T1	cna	amp	BASIS
PD3890	RUNX1T1	cna	gain	BASIS
PD3905	RUNX1T1	cna	gain	BASIS
PD4005	RUNX1T1	cna	hetloss	BASIS
PD4006	RUNX1T1	cna	gain	BASIS
PD4826	RUNX1T1	cna	amp	BASIS
PD5930	RUNX1T1	cna	gain	BASIS
PD5935	RUNX1T1	cna	gain	BASIS
PD5945	RUNX1T1	cna	gain	BASIS
PD5948	RUNX1T1	cna	gain	BASIS
PD6406	RUNX1T1	cna	gain	BASIS
PD6413	RUNX1T1	cna	gain	BASIS
PD7067	RUNX1T1	cna	amp	BASIS
PD7215	RUNX1T1	cna	amp	BASIS
PD8621	RUNX1T1	cna	gain	BASIS
PD8980	RUNX1T1	cna	gain	BASIS
PD9004	RUNX1T1	cna	gain	BASIS
PD9585	RUNX1T1	cna	gain	BASIS
PD9702	RUNX1T1	cna	amp	BASIS
TCGA-A2-A25B	DDR2	cna	gain	TCGA
TCGA-AN-A0XU	DDR2	cna	gain	TCGA
TCGA-AO-A0JL	DDR2	cna	gain	TCGA

TCGA-BH-A0AW	DDR2	cna	gain	TCGA
TCGA-BH-A0C0	DDR2	cna	gain	TCGA
TCGA-BH-A18R	DDR2	cna	gain	TCGA
TCGA-BH-A1FU	DDR2	cna	gain	TCGA
TCGA-C8-A12L	DDR2	cna	gain	TCGA
TCGA-D8-A27M	DDR2	cna	amp	TCGA
TCGA-E2-A1L7	DDR2	cna	gain	TCGA
TCGA-EW-A1OX	DDR2	cna	gain	TCGA
TCGA-LL-A5YP	DDR2	cna	amp	TCGA
MB-0346	DDR2	cna	gain	METABRIC
MB-5070	DDR2	cna	gain	METABRIC
MB-5465	DDR2	cna	amp	METABRIC
MB-6060	DDR2	cna	gain	METABRIC
MB-6098	DDR2	cna	amp	METABRIC
MB-7032	DDR2	cna	gain	METABRIC
MB-7038	DDR2	cna	gain	METABRIC
MB-0420	DDR2	cna	gain	METABRIC
MTS-T0064	DDR2	cna	gain	METABRIC
PD10014	DDR2	cna	gain	BASIS
PD11327	DDR2	cna	amp	BASIS
PD13297	DDR2	cna	gain	BASIS
PD13299	DDR2	cna	gain	BASIS
PD14442	DDR2	cna	gain	BASIS
PD22355	DDR2	cna	gain	BASIS
PD23562	DDR2	cna	amp	BASIS
PD23574	DDR2	cna	gain	BASIS
PD23578	DDR2	cna	gain	BASIS
PD24186	DDR2	cna	gain	BASIS
PD24206	DDR2	cna	gain	BASIS
PD3905	DDR2	cna	gain	BASIS
PD4005	DDR2	cna	gain	BASIS
PD4006	DDR2	cna	gain	BASIS
PD4107	DDR2	cna	gain	BASIS
PD4826	DDR2	cna	gain	BASIS
PD5935	DDR2	cna	gain	BASIS
PD5945	DDR2	cna	amp	BASIS
PD5948	DDR2	cna	gain	BASIS
PD6406	DDR2	cna	gain	BASIS
PD6413	DDR2	cna	gain	BASIS
PD7067	DDR2	cna	amp	BASIS
PD7215	DDR2	cna	gain	BASIS
PD8621	DDR2	cna	amp	BASIS
PD8980	DDR2	cna	gain	BASIS
PD9004	DDR2	cna	gain	BASIS
PD9702	DDR2	cna	gain	BASIS
TCGA-A2-A25B	ELF3	cna	amp	TCGA
TCGA-AN-A0XU	ELF3	cna	hetloss	TCGA

TCGA-AO-A0JL	ELF3	cna	gain	TCGA
TCGA-BH-A0AW	ELF3	cna	gain	TCGA
TCGA-BH-A0C0	ELF3	cna	gain	TCGA
TCGA-BH-A18R	ELF3	cna	gain	TCGA
TCGA-BH-A1FU	ELF3	cna	gain	TCGA
TCGA-C8-A12L	ELF3	cna	gain	TCGA
TCGA-E2-A1L7	ELF3	cna	gain	TCGA
TCGA-E9-A1NC	ELF3	cna	gain	TCGA
TCGA-EW-A1OX	ELF3	cna	gain	TCGA
TCGA-LL-A5YP	ELF3	cna	gain	TCGA
MB-0346	ELF3	cna	gain	METABRIC
MB-5107	ELF3	cna	gain	METABRIC
MB-5465	ELF3	cna	gain	METABRIC
MB-6060	ELF3	cna	gain	METABRIC
MB-6098	ELF3	cna	gain	METABRIC
MB-7032	ELF3	cna	gain	METABRIC
MB-7048	ELF3	cna	gain	METABRIC
PD10014	ELF3	cna	gain	BASIS
PD13296	ELF3	cna	hetloss	BASIS
PD13297	ELF3	cna	gain	BASIS
PD13299	ELF3	cna	gain	BASIS
PD14442	ELF3	cna	gain	BASIS
PD23562	ELF3	cna	gain	BASIS
PD23574	ELF3	cna	gain	BASIS
PD23578	ELF3	cna	hetloss	BASIS
PD24186	ELF3	cna	amp	BASIS
PD24206	ELF3	cna	gain	BASIS
PD24337	ELF3	cna	gain	BASIS
PD3890	ELF3	cna	gain	BASIS
PD3905	ELF3	cna	gain	BASIS
PD4005	ELF3	cna	gain	BASIS
PD4006	ELF3	cna	amp	BASIS
PD4107	ELF3	cna	gain	BASIS
PD4826	ELF3	cna	gain	BASIS
PD5935	ELF3	cna	gain	BASIS
PD5945	ELF3	cna	amp	BASIS
PD5948	ELF3	cna	gain	BASIS
PD6406	ELF3	cna	gain	BASIS
PD6413	ELF3	cna	gain	BASIS
PD6731	ELF3	cna	hetloss	BASIS
PD7067	ELF3	cna	gain	BASIS
PD7215	ELF3	cna	amp	BASIS
PD8621	ELF3	cna	gain	BASIS
PD8980	ELF3	cna	amp	BASIS
PD9004	ELF3	cna	gain	BASIS
PD9702	ELF3	cna	amp	BASIS
TCGA-A2-A25B	ERC1	cna	hetloss	TCGA

TCGA-AN-A0XU	ERC1	cna	gain	TCGA
TCGA-AO-A0JL	ERC1	cna	gain	TCGA
TCGA-BH-A0C0	ERC1	cna	hetloss	TCGA
TCGA-BH-A18R	ERC1	cna	gain	TCGA
TCGA-BH-A1FU	ERC1	cna	hetloss	TCGA
TCGA-C8-A12L	ERC1	cna	gain	TCGA
TCGA-D8-A27M	ERC1	cna	amp	TCGA
TCGA-E9-A1NC	ERC1	cna	gain	TCGA
TCGA-LL-A5YP	ERC1	cna	gain	TCGA
MB-0346	ERC1	cna	hetloss	METABRIC
MB-2827	ERC1	cna	amp	METABRIC
MB-5070	ERC1	cna	amp	METABRIC
MB-6060	ERC1	cna	gain	METABRIC
MB-6098	ERC1	cna	gain	METABRIC
MB-7048	ERC1	cna	gain	METABRIC
MB-0420	ERC1	cna	gain	METABRIC
MTS-T0064	ERC1	cna	amp	METABRIC
PD10014	ERC1	cna	hetloss	BASIS
PD11327	ERC1	cna	gain	BASIS
PD11742	ERC1	cna	gain	BASIS
PD13299	ERC1	cna	gain	BASIS
PD13771	ERC1	cna	hetloss	BASIS
PD23562	ERC1	cna	amp	BASIS
PD23574	ERC1	cna	gain	BASIS
PD23578	ERC1	cna	gain	BASIS
PD24186	ERC1	cna	amp	BASIS
PD24202	ERC1	cna	gain	BASIS
PD24206	ERC1	cna	homdel	BASIS
PD24337	ERC1	cna	gain	BASIS
PD3890	ERC1	cna	gain	BASIS
PD3905	ERC1	cna	gain	BASIS
PD4005	ERC1	cna	gain	BASIS
PD4006	ERC1	cna	amp	BASIS
PD4107	ERC1	cna	gain	BASIS
PD4826	ERC1	cna	hetloss	BASIS
PD4967	ERC1	cna	hetloss	BASIS
PD5930	ERC1	cna	gain	BASIS
PD5935	ERC1	cna	gain	BASIS
PD5945	ERC1	cna	amp	BASIS
PD5948	ERC1	cna	gain	BASIS
PD6406	ERC1	cna	gain	BASIS
PD6413	ERC1	cna	gain	BASIS
PD6731	ERC1	cna	hetloss	BASIS
PD7215	ERC1	cna	gain	BASIS
PD8621	ERC1	cna	gain	BASIS
PD9004	ERC1	cna	gain	BASIS
PD9702	ERC1	cna	gain	BASIS

TCGA-A2-A25B	KDM5A	cna	hetloss	TCGA
TCGA-AN-A0XU	KDM5A	cna	gain	TCGA
TCGA-AO-A0JL	KDM5A	cna	gain	TCGA
TCGA-BH-A0C0	KDM5A	cna	hetloss	TCGA
TCGA-BH-A18R	KDM5A	cna	gain	TCGA
TCGA-BH-A1FU	KDM5A	cna	hetloss	TCGA
TCGA-C8-A12L	KDM5A	cna	gain	TCGA
TCGA-D8-A27M	KDM5A	cna	amp	TCGA
TCGA-E9-A1NC	KDM5A	cna	gain	TCGA
TCGA-LL-A5YP	KDM5A	cna	gain	TCGA
MB-0346	KDM5A	cna	hetloss	METABRIC
MB-2827	KDM5A	cna	amp	METABRIC
MB-5070	KDM5A	cna	amp	METABRIC
MB-6060	KDM5A	cna	gain	METABRIC
MB-6098	KDM5A	cna	gain	METABRIC
MB-7048	KDM5A	cna	gain	METABRIC
MB-0420	KDM5A	cna	gain	METABRIC
MTS-T0064	KDM5A	cna	amp	METABRIC
PD10014	KDM5A	cna	hetloss	BASIS
PD11327	KDM5A	cna	gain	BASIS
PD11742	KDM5A	cna	gain	BASIS
PD13299	KDM5A	cna	gain	BASIS
PD13771	KDM5A	cna	hetloss	BASIS
PD22355	KDM5A	cna	hetloss	BASIS
PD23562	KDM5A	cna	amp	BASIS
PD23574	KDM5A	cna	gain	BASIS
PD23578	KDM5A	cna	gain	BASIS
PD24186	KDM5A	cna	amp	BASIS
PD24206	KDM5A	cna	hetloss	BASIS
PD24337	KDM5A	cna	gain	BASIS
PD3890	KDM5A	cna	gain	BASIS
PD3905	KDM5A	cna	gain	BASIS
PD4005	KDM5A	cna	gain	BASIS
PD4006	KDM5A	cna	amp	BASIS
PD4107	KDM5A	cna	amp	BASIS
PD4826	KDM5A	cna	hetloss	BASIS
PD4967	KDM5A	cna	hetloss	BASIS
PD5930	KDM5A	cna	gain	BASIS
PD5935	KDM5A	cna	gain	BASIS
PD5945	KDM5A	cna	amp	BASIS
PD5948	KDM5A	cna	gain	BASIS
PD6406	KDM5A	cna	gain	BASIS
PD6413	KDM5A	cna	gain	BASIS
PD6731	KDM5A	cna	hetloss	BASIS
PD7215	KDM5A	cna	gain	BASIS
PD8621	KDM5A	cna	gain	BASIS
PD9004	KDM5A	cna	gain	BASIS

PD9702	KDM5A	cna	gain	BASIS
TCGA-A2-A25B	NCSTN	cna	gain	TCGA
TCGA-AN-A0XU	NCSTN	cna	gain	TCGA
TCGA-AO-A0JL	NCSTN	cna	gain	TCGA
TCGA-BH-A0AW	NCSTN	cna	gain	TCGA
TCGA-BH-A0C0	NCSTN	cna	gain	TCGA
TCGA-BH-A18R	NCSTN	cna	gain	TCGA
TCGA-BH-A1FU	NCSTN	cna	gain	TCGA
TCGA-C8-A12L	NCSTN	cna	gain	TCGA
TCGA-E2-A1L7	NCSTN	cna	gain	TCGA
TCGA-EW-A1OX	NCSTN	cna	gain	TCGA
TCGA-LL-A5YP	NCSTN	cna	amp	TCGA
MB-0346	NCSTN	cna	gain	METABRIC
MB-2827	NCSTN	cna	amp	METABRIC
MB-5070	NCSTN	cna	gain	METABRIC
MB-5465	NCSTN	cna	amp	METABRIC
MB-6060	NCSTN	cna	gain	METABRIC
MB-6098	NCSTN	cna	amp	METABRIC
MB-7032	NCSTN	cna	gain	METABRIC
MB-7038	NCSTN	cna	gain	METABRIC
MB-0420	NCSTN	cna	amp	METABRIC
PD10014	NCSTN	cna	gain	BASIS
PD11327	NCSTN	cna	amp	BASIS
PD13297	NCSTN	cna	gain	BASIS
PD13299	NCSTN	cna	gain	BASIS
PD14442	NCSTN	cna	gain	BASIS
PD22355	NCSTN	cna	gain	BASIS
PD23562	NCSTN	cna	amp	BASIS
PD23574	NCSTN	cna	amp	BASIS
PD24186	NCSTN	cna	gain	BASIS
PD24206	NCSTN	cna	gain	BASIS
PD3890	NCSTN	cna	gain	BASIS
PD3905	NCSTN	cna	gain	BASIS
PD4005	NCSTN	cna	gain	BASIS
PD4006	NCSTN	cna	gain	BASIS
PD4107	NCSTN	cna	gain	BASIS
PD4826	NCSTN	cna	gain	BASIS
PD5930	NCSTN	cna	gain	BASIS
PD5935	NCSTN	cna	gain	BASIS
PD5945	NCSTN	cna	amp	BASIS
PD5948	NCSTN	cna	gain	BASIS
PD6406	NCSTN	cna	gain	BASIS
PD6413	NCSTN	cna	gain	BASIS
PD7067	NCSTN	cna	amp	BASIS
PD7215	NCSTN	cna	gain	BASIS
PD8621	NCSTN	cna	amp	BASIS
PD8980	NCSTN	cna	gain	BASIS

PD9004	NCSTN	cna	gain	BASIS
PD9702	NCSTN	cna	gain	BASIS
TCGA-A2-A25B	RAD52	cna	hetloss	TCGA
TCGA-AN-A0XU	RAD52	cna	gain	TCGA
TCGA-AO-A0JL	RAD52	cna	gain	TCGA
TCGA-BH-A0C0	RAD52	cna	hetloss	TCGA
TCGA-BH-A18R	RAD52	cna	gain	TCGA
TCGA-BH-A1FU	RAD52	cna	hetloss	TCGA
TCGA-C8-A12L	RAD52	cna	gain	TCGA
TCGA-D8-A27M	RAD52	cna	amp	TCGA
TCGA-E9-A1NC	RAD52	cna	gain	TCGA
TCGA-LL-A5YP	RAD52	cna	gain	TCGA
MB-0346	RAD52	cna	hetloss	METABRIC
MB-2827	RAD52	cna	amp	METABRIC
MB-5070	RAD52	cna	amp	METABRIC
MB-6060	RAD52	cna	gain	METABRIC
MB-6098	RAD52	cna	gain	METABRIC
MB-7048	RAD52	cna	gain	METABRIC
MB-0420	RAD52	cna	gain	METABRIC
MTS-T0064	RAD52	cna	amp	METABRIC
PD10014	RAD52	cna	hetloss	BASIS
PD11327	RAD52	cna	gain	BASIS
PD11742	RAD52	cna	gain	BASIS
PD13299	RAD52	cna	gain	BASIS
PD13771	RAD52	cna	hetloss	BASIS
PD23562	RAD52	cna	amp	BASIS
PD23574	RAD52	cna	gain	BASIS
PD23578	RAD52	cna	gain	BASIS
PD24186	RAD52	cna	amp	BASIS
PD24202	RAD52	cna	gain	BASIS
PD24206	RAD52	cna	hetloss	BASIS
PD24337	RAD52	cna	gain	BASIS
PD3890	RAD52	cna	gain	BASIS
PD3905	RAD52	cna	gain	BASIS
PD4005	RAD52	cna	gain	BASIS
PD4006	RAD52	cna	amp	BASIS
PD4107	RAD52	cna	gain	BASIS
PD4826	RAD52	cna	hetloss	BASIS
PD4967	RAD52	cna	hetloss	BASIS
PD5930	RAD52	cna	gain	BASIS
PD5935	RAD52	cna	gain	BASIS
PD5945	RAD52	cna	amp	BASIS
PD5948	RAD52	cna	gain	BASIS
PD6406	RAD52	cna	gain	BASIS
PD6413	RAD52	cna	gain	BASIS
PD6731	RAD52	cna	hetloss	BASIS
PD7215	RAD52	cna	gain	BASIS

PD8621	RAD52	cna	gain	BASIS
PD9004	RAD52	cna	gain	BASIS
PD9702	RAD52	cna	gain	BASIS
TCGA-A2-A25B	RARA	cna	hetloss	TCGA
TCGA-AN-A0XU	RARA	cna	hetloss	TCGA
TCGA-AO-A0JL	RARA	cna	amp	TCGA
TCGA-BH-A0AW	RARA	cna	homdel	TCGA
TCGA-BH-A0C0	RARA	cna	gain	TCGA
TCGA-BH-A18R	RARA	cna	amp	TCGA
TCGA-BH-A1FU	RARA	cna	homdel	TCGA
TCGA-C8-A12L	RARA	cna	hetloss	TCGA
TCGA-D8-A27M	RARA	cna	hetloss	TCGA
TCGA-E2-A1L7	RARA	cna	hetloss	TCGA
TCGA-E9-A1NC	RARA	cna	hetloss	TCGA
MB-0346	RARA	cna	hetloss	METABRIC
MB-2827	RARA	cna	hetloss	METABRIC
MB-5070	RARA	cna	hetloss	METABRIC
MB-5107	RARA	cna	gain	METABRIC
MB-5465	RARA	cna	hetloss	METABRIC
MB-6060	RARA	cna	amp	METABRIC
MB-6098	RARA	cna	hetloss	METABRIC
MB-6271	RARA	cna	hetloss	METABRIC
MB-7038	RARA	cna	hetloss	METABRIC
MB-7048	RARA	cna	gain	METABRIC
MB-0420	RARA	cna	hetloss	METABRIC
P-0002023-T01-IM3	RARA	cna	amp	MSK-IMPACT
P-0002858-T01-IM3	RARA	cna	amp	MSK-IMPACT
P-0009557-T01-IM5	RARA	cna	homdel	MSK-IMPACT
PD13297	RARA	cna	hetloss	BASIS
PD13299	RARA	cna	gain	BASIS
PD13771	RARA	cna	hetloss	BASIS
PD23561	RARA	cna	hetloss	BASIS
PD23562	RARA	cna	gain	BASIS
PD23574	RARA	cna	gain	BASIS
PD24186	RARA	cna	gain	BASIS
PD24206	RARA	cna	hetloss	BASIS
PD24337	RARA	cna	hetloss	BASIS
PD3890	RARA	cna	hetloss	BASIS
PD3905	RARA	cna	gain	BASIS
PD4005	RARA	cna	hetloss	BASIS
PD4826	RARA	cna	hetloss	BASIS
PD4967	RARA	cna	hetloss	BASIS
PD5945	RARA	cna	amp	BASIS
PD5948	RARA	cna	gain	BASIS
PD6406	RARA	cna	hetloss	BASIS
PD6731	RARA	cna	hetloss	BASIS
PD7067	RARA	cna	gain	BASIS

PD7215	RARA	cna	gain	BASIS
PD9004	RARA	cna	gain	BASIS
PD9585	RARA	cna	hetloss	BASIS
PD9702	RARA	cna	amp	BASIS
TCGA-A2-A25B	STAT3	cna	hetloss	TCGA
TCGA-AN-A0XU	STAT3	cna	hetloss	TCGA
TCGA-AO-A0JL	STAT3	cna	homdel	TCGA
TCGA-BH-A0AW	STAT3	cna	homdel	TCGA
TCGA-BH-A0C0	STAT3	cna	gain	TCGA
TCGA-BH-A18R	STAT3	cna	homdel	TCGA
TCGA-BH-A1FU	STAT3	cna	homdel	TCGA
TCGA-C8-A12L	STAT3	cna	hetloss	TCGA
TCGA-D8-A27M	STAT3	cna	hetloss	TCGA
TCGA-E2-A1L7	STAT3	cna	hetloss	TCGA
TCGA-E9-A1NC	STAT3	cna	hetloss	TCGA
MB-0346	STAT3	cna	hetloss	METABRIC
MB-2827	STAT3	cna	hetloss	METABRIC
MB-5070	STAT3	cna	hetloss	METABRIC
MB-5107	STAT3	cna	gain	METABRIC
MB-5465	STAT3	cna	hetloss	METABRIC
MB-6060	STAT3	cna	hetloss	METABRIC
MB-6098	STAT3	cna	hetloss	METABRIC
MB-6271	STAT3	cna	hetloss	METABRIC
MB-7038	STAT3	cna	hetloss	METABRIC
MB-7048	STAT3	cna	hetloss	METABRIC
MB-0420	STAT3	cna	hetloss	METABRIC
P-0009557-T01-IM5	STAT3	cna	homdel	MSK-IMPACT
PD13296	STAT3	cna	gain	BASIS
PD13297	STAT3	cna	hetloss	BASIS
PD13299	STAT3	cna	gain	BASIS
PD13771	STAT3	cna	hetloss	BASIS
PD22355	STAT3	cna	hetloss	BASIS
PD23561	STAT3	cna	hetloss	BASIS
PD23562	STAT3	cna	gain	BASIS
PD23574	STAT3	cna	gain	BASIS
PD24186	STAT3	cna	gain	BASIS
PD24206	STAT3	cna	hetloss	BASIS
PD24337	STAT3	cna	hetloss	BASIS
PD3890	STAT3	cna	hetloss	BASIS
PD3905	STAT3	cna	gain	BASIS
PD4006	STAT3	cna	gain	BASIS
PD4826	STAT3	cna	hetloss	BASIS
PD4967	STAT3	cna	homdel	BASIS
PD5945	STAT3	cna	amp	BASIS
PD5948	STAT3	cna	gain	BASIS
PD6406	STAT3	cna	hetloss	BASIS
PD6731	STAT3	cna	hetloss	BASIS

PD7067	STAT3	cna	gain	BASIS
PD7215	STAT3	cna	gain	BASIS
PD9004	STAT3	cna	gain	BASIS
PD9585	STAT3	cna	hetloss	BASIS
PD9702	STAT3	cna	amp	BASIS
TCGA-A2-A25B	STAT5A	cna	hetloss	TCGA
TCGA-AN-A0XU	STAT5A	cna	hetloss	TCGA
TCGA-AO-A0JL	STAT5A	cna	homdel	TCGA
TCGA-BH-A0AW	STAT5A	cna	homdel	TCGA
TCGA-BH-A0C0	STAT5A	cna	gain	TCGA
TCGA-BH-A18R	STAT5A	cna	homdel	TCGA
TCGA-BH-A1FU	STAT5A	cna	homdel	TCGA
TCGA-C8-A12L	STAT5A	cna	hetloss	TCGA
TCGA-D8-A27M	STAT5A	cna	hetloss	TCGA
TCGA-E2-A1L7	STAT5A	cna	hetloss	TCGA
TCGA-E9-A1NC	STAT5A	cna	hetloss	TCGA
MB-0346	STAT5A	cna	hetloss	METABRIC
MB-2827	STAT5A	cna	hetloss	METABRIC
MB-5070	STAT5A	cna	hetloss	METABRIC
MB-5107	STAT5A	cna	gain	METABRIC
MB-5465	STAT5A	cna	hetloss	METABRIC
MB-6060	STAT5A	cna	hetloss	METABRIC
MB-6098	STAT5A	cna	hetloss	METABRIC
MB-6271	STAT5A	cna	hetloss	METABRIC
MB-7038	STAT5A	cna	hetloss	METABRIC
MB-7048	STAT5A	cna	hetloss	METABRIC
MB-0420	STAT5A	cna	hetloss	METABRIC
P-0009557-T01-IM5	STAT5A	cna	homdel	MSK-IMPACT
PD13296	STAT5A	cna	gain	BASIS
PD13297	STAT5A	cna	hetloss	BASIS
PD13299	STAT5A	cna	gain	BASIS
PD13771	STAT5A	cna	hetloss	BASIS
PD22355	STAT5A	cna	hetloss	BASIS
PD23561	STAT5A	cna	hetloss	BASIS
PD23562	STAT5A	cna	gain	BASIS
PD23574	STAT5A	cna	gain	BASIS
PD24186	STAT5A	cna	gain	BASIS
PD24206	STAT5A	cna	hetloss	BASIS
PD24337	STAT5A	cna	hetloss	BASIS
PD3890	STAT5A	cna	hetloss	BASIS
PD3905	STAT5A	cna	gain	BASIS
PD4006	STAT5A	cna	gain	BASIS
PD4826	STAT5A	cna	hetloss	BASIS
PD4967	STAT5A	cna	hetloss	BASIS
PD5945	STAT5A	cna	amp	BASIS
PD5948	STAT5A	cna	gain	BASIS
PD6406	STAT5A	cna	hetloss	BASIS

PD6731	STAT5A	cna	hetloss	BASIS
PD7067	STAT5A	cna	gain	BASIS
PD7215	STAT5A	cna	gain	BASIS
PD9004	STAT5A	cna	gain	BASIS
PD9585	STAT5A	cna	hetloss	BASIS
PD9702	STAT5A	cna	amp	BASIS
TCGA-A2-A25B	STAT5B	cna	hetloss	TCGA
TCGA-AN-A0XU	STAT5B	cna	hetloss	TCGA
TCGA-AO-A0JL	STAT5B	cna	homdel	TCGA
TCGA-BH-A0AW	STAT5B	cna	homdel	TCGA
TCGA-BH-A0C0	STAT5B	cna	gain	TCGA
TCGA-BH-A18R	STAT5B	cna	homdel	TCGA
TCGA-BH-A1FU	STAT5B	cna	homdel	TCGA
TCGA-C8-A12L	STAT5B	cna	hetloss	TCGA
TCGA-D8-A27M	STAT5B	cna	hetloss	TCGA
TCGA-E2-A1L7	STAT5B	cna	hetloss	TCGA
TCGA-E9-A1NC	STAT5B	cna	hetloss	TCGA
MB-0346	STAT5B	cna	hetloss	METABRIC
MB-2827	STAT5B	cna	hetloss	METABRIC
MB-5070	STAT5B	cna	hetloss	METABRIC
MB-5107	STAT5B	cna	gain	METABRIC
MB-5465	STAT5B	cna	hetloss	METABRIC
MB-6060	STAT5B	cna	hetloss	METABRIC
MB-6098	STAT5B	cna	hetloss	METABRIC
MB-6271	STAT5B	cna	hetloss	METABRIC
MB-7038	STAT5B	cna	hetloss	METABRIC
MB-7048	STAT5B	cna	hetloss	METABRIC
MB-0420	STAT5B	cna	hetloss	METABRIC
P-0009557-T01-IM5	STAT5B	cna	homdel	MSK-IMPACT
PD13296	STAT5B	cna	gain	BASIS
PD13297	STAT5B	cna	hetloss	BASIS
PD13299	STAT5B	cna	gain	BASIS
PD13771	STAT5B	cna	hetloss	BASIS
PD22355	STAT5B	cna	hetloss	BASIS
PD23561	STAT5B	cna	hetloss	BASIS
PD23562	STAT5B	cna	gain	BASIS
PD23574	STAT5B	cna	gain	BASIS
PD24186	STAT5B	cna	gain	BASIS
PD24206	STAT5B	cna	hetloss	BASIS
PD24337	STAT5B	cna	hetloss	BASIS
PD3890	STAT5B	cna	hetloss	BASIS
PD3905	STAT5B	cna	gain	BASIS
PD4006	STAT5B	cna	gain	BASIS
PD4826	STAT5B	cna	hetloss	BASIS
PD4967	STAT5B	cna	hetloss	BASIS
PD5945	STAT5B	cna	amp	BASIS
PD5948	STAT5B	cna	gain	BASIS

PD6406	STAT5B	cna	hetloss	BASIS
PD6731	STAT5B	cna	hetloss	BASIS
PD7067	STAT5B	cna	gain	BASIS
PD7215	STAT5B	cna	gain	BASIS
PD9004	STAT5B	cna	gain	BASIS
PD9585	STAT5B	cna	hetloss	BASIS
PD9702	STAT5B	cna	amp	BASIS
TCGA-A2-A25B	WNK1	cna	hetloss	TCGA
TCGA-AN-A0XU	WNK1	cna	gain	TCGA
TCGA-AO-A0JL	WNK1	cna	gain	TCGA
TCGA-BH-A0C0	WNK1	cna	hetloss	TCGA
TCGA-BH-A18R	WNK1	cna	gain	TCGA
TCGA-BH-A1FU	WNK1	cna	hetloss	TCGA
TCGA-C8-A12L	WNK1	cna	gain	TCGA
TCGA-D8-A27M	WNK1	cna	amp	TCGA
TCGA-E9-A1NC	WNK1	cna	gain	TCGA
TCGA-LL-A5YP	WNK1	cna	gain	TCGA
MB-0346	WNK1	cna	hetloss	METABRIC
MB-2827	WNK1	cna	amp	METABRIC
MB-5070	WNK1	cna	amp	METABRIC
MB-6060	WNK1	cna	gain	METABRIC
MB-6098	WNK1	cna	gain	METABRIC
MB-7048	WNK1	cna	gain	METABRIC
MB-0420	WNK1	cna	gain	METABRIC
MTS-T0064	WNK1	cna	amp	METABRIC
PD10014	WNK1	cna	hetloss	BASIS
PD11327	WNK1	cna	gain	BASIS
PD11742	WNK1	cna	gain	BASIS
PD13299	WNK1	cna	gain	BASIS
PD13771	WNK1	cna	hetloss	BASIS
PD23562	WNK1	cna	amp	BASIS
PD23574	WNK1	cna	gain	BASIS
PD23578	WNK1	cna	gain	BASIS
PD24186	WNK1	cna	amp	BASIS
PD24202	WNK1	cna	gain	BASIS
PD24206	WNK1	cna	hetloss	BASIS
PD24337	WNK1	cna	gain	BASIS
PD3890	WNK1	cna	gain	BASIS
PD3905	WNK1	cna	gain	BASIS
PD4005	WNK1	cna	gain	BASIS
PD4006	WNK1	cna	amp	BASIS
PD4107	WNK1	cna	amp	BASIS
PD4826	WNK1	cna	hetloss	BASIS
PD4967	WNK1	cna	hetloss	BASIS
PD5930	WNK1	cna	gain	BASIS
PD5935	WNK1	cna	gain	BASIS
PD5945	WNK1	cna	amp	BASIS

PD5948	WNK1	cna	gain	BASIS
PD6406	WNK1	cna	gain	BASIS
PD6413	WNK1	cna	gain	BASIS
PD6731	WNK1	cna	hetloss	BASIS
PD7215	WNK1	cna	gain	BASIS
PD8621	WNK1	cna	gain	BASIS
PD9004	WNK1	cna	gain	BASIS
PD9702	WNK1	cna	gain	BASIS
TCGA-A2-A25B	BRIP1	cna	amp	TCGA
TCGA-AN-A0XU	BRIP1	cna	gain	TCGA
TCGA-AO-A0JL	BRIP1	cna	hetloss	TCGA
TCGA-BH-A0AW	BRIP1	cna	amp	TCGA
TCGA-BH-A0C0	BRIP1	cna	gain	TCGA
TCGA-BH-A18R	BRIP1	cna	amp	TCGA
TCGA-C8-A12L	BRIP1	cna	gain	TCGA
TCGA-D8-A27M	BRIP1	cna	hetloss	TCGA
TCGA-EW-A1OX	BRIP1	cna	amp	TCGA
TCGA-LL-A5YP	BRIP1	cna	amp	TCGA
MB-0346	BRIP1	cna	gain	METABRIC
MB-2827	BRIP1	cna	hetloss	METABRIC
MB-5070	BRIP1	cna	hetloss	METABRIC
MB-5107	BRIP1	cna	amp	METABRIC
MB-5323	BRIP1	cna	gain	METABRIC
MB-5465	BRIP1	cna	amp	METABRIC
MB-6060	BRIP1	cna	amp	METABRIC
MB-6098	BRIP1	cna	hetloss	METABRIC
MB-6271	BRIP1	cna	hetloss	METABRIC
MB-7048	BRIP1	cna	gain	METABRIC
MTS-T0064	BRIP1	cna	gain	METABRIC
P-0002023-T01-IM3	BRIP1	cna	amp	MSK-IMPACT
P-0002858-T01-IM3	BRIP1	cna	amp	MSK-IMPACT
PD11327	BRIP1	cna	gain	BASIS
PD11742	BRIP1	cna	gain	BASIS
PD13297	BRIP1	cna	hetloss	BASIS
PD22355	BRIP1	cna	gain	BASIS
PD23561	BRIP1	cna	gain	BASIS
PD23562	BRIP1	cna	gain	BASIS
PD23574	BRIP1	cna	gain	BASIS
PD24186	BRIP1	cna	gain	BASIS
PD24206	BRIP1	cna	hetloss	BASIS
PD24337	BRIP1	cna	hetloss	BASIS
PD3905	BRIP1	cna	gain	BASIS
PD4006	BRIP1	cna	amp	BASIS
PD4826	BRIP1	cna	amp	BASIS
PD4967	BRIP1	cna	homdel	BASIS
PD5930	BRIP1	cna	gain	BASIS
PD5935	BRIP1	cna	amp	BASIS

PD5945	BRIP1	cna	amp	BASIS
PD5948	BRIP1	cna	gain	BASIS
PD7067	BRIP1	cna	gain	BASIS
PD7215	BRIP1	cna	gain	BASIS
PD8621	BRIP1	cna	gain	BASIS
PD8980	BRIP1	cna	gain	BASIS
PD9004	BRIP1	cna	gain	BASIS
PD9702	BRIP1	cna	gain	BASIS
TCGA-A2-A25B	BTG2	cna	amp	TCGA
TCGA-AN-A0XU	BTG2	cna	hetloss	TCGA
TCGA-AO-A0JL	BTG2	cna	gain	TCGA
TCGA-BH-A0AW	BTG2	cna	gain	TCGA
TCGA-BH-A0C0	BTG2	cna	gain	TCGA
TCGA-BH-A18R	BTG2	cna	gain	TCGA
TCGA-BH-A1FU	BTG2	cna	gain	TCGA
TCGA-C8-A12L	BTG2	cna	gain	TCGA
TCGA-E2-A1L7	BTG2	cna	gain	TCGA
TCGA-E9-A1NC	BTG2	cna	gain	TCGA
TCGA-EW-A1OX	BTG2	cna	gain	TCGA
TCGA-LL-A5YP	BTG2	cna	hetloss	TCGA
MB-0346	BTG2	cna	gain	METABRIC
MB-5107	BTG2	cna	gain	METABRIC
MB-5465	BTG2	cna	gain	METABRIC
MB-6060	BTG2	cna	gain	METABRIC
MB-6098	BTG2	cna	gain	METABRIC
MB-7032	BTG2	cna	gain	METABRIC
MB-7038	BTG2	cna	gain	METABRIC
MB-7048	BTG2	cna	gain	METABRIC
PD10014	BTG2	cna	gain	BASIS
PD13296	BTG2	cna	hetloss	BASIS
PD13299	BTG2	cna	gain	BASIS
PD14442	BTG2	cna	gain	BASIS
PD23562	BTG2	cna	gain	BASIS
PD23574	BTG2	cna	gain	BASIS
PD24186	BTG2	cna	amp	BASIS
PD24206	BTG2	cna	gain	BASIS
PD24337	BTG2	cna	gain	BASIS
PD3890	BTG2	cna	gain	BASIS
PD3905	BTG2	cna	gain	BASIS
PD4005	BTG2	cna	gain	BASIS
PD4006	BTG2	cna	amp	BASIS
PD4107	BTG2	cna	gain	BASIS
PD4826	BTG2	cna	gain	BASIS
PD5935	BTG2	cna	gain	BASIS
PD5945	BTG2	cna	amp	BASIS
PD5948	BTG2	cna	gain	BASIS
PD6406	BTG2	cna	gain	BASIS

PD6413	BTG2	cna	gain	BASIS
PD6731	BTG2	cna	hetloss	BASIS
PD7067	BTG2	cna	gain	BASIS
PD7215	BTG2	cna	amp	BASIS
PD8621	BTG2	cna	gain	BASIS
PD8980	BTG2	cna	amp	BASIS
PD9004	BTG2	cna	gain	BASIS
PD9702	BTG2	cna	amp	BASIS
TCGA-A2-A25B	CDC73	cna	amp	TCGA
TCGA-AN-A0XU	CDC73	cna	hetloss	TCGA
TCGA-AO-A0JL	CDC73	cna	gain	TCGA
TCGA-BH-A0AW	CDC73	cna	gain	TCGA
TCGA-BH-A0C0	CDC73	cna	gain	TCGA
TCGA-BH-A18R	CDC73	cna	gain	TCGA
TCGA-BH-A1FU	CDC73	cna	gain	TCGA
TCGA-C8-A12L	CDC73	cna	amp	TCGA
TCGA-E2-A1L7	CDC73	cna	gain	TCGA
TCGA-E9-A1NC	CDC73	cna	hetloss	TCGA
TCGA-EW-A1OX	CDC73	cna	gain	TCGA
TCGA-LL-A5YP	CDC73	cna	gain	TCGA
MB-0346	CDC73	cna	gain	METABRIC
MB-5107	CDC73	cna	gain	METABRIC
MB-5465	CDC73	cna	amp	METABRIC
MB-6060	CDC73	cna	gain	METABRIC
MB-6098	CDC73	cna	gain	METABRIC
MB-7032	CDC73	cna	gain	METABRIC
MB-7038	CDC73	cna	gain	METABRIC
MB-7048	CDC73	cna	gain	METABRIC
PD10014	CDC73	cna	gain	BASIS
PD11327	CDC73	cna	gain	BASIS
PD13297	CDC73	cna	gain	BASIS
PD13299	CDC73	cna	gain	BASIS
PD14442	CDC73	cna	gain	BASIS
PD22355	CDC73	cna	gain	BASIS
PD23562	CDC73	cna	gain	BASIS
PD24186	CDC73	cna	amp	BASIS
PD24206	CDC73	cna	gain	BASIS
PD24337	CDC73	cna	gain	BASIS
PD3890	CDC73	cna	gain	BASIS
PD3905	CDC73	cna	amp	BASIS
PD4005	CDC73	cna	gain	BASIS
PD4006	CDC73	cna	gain	BASIS
PD4107	CDC73	cna	gain	BASIS
PD4826	CDC73	cna	gain	BASIS
PD5935	CDC73	cna	gain	BASIS
PD5945	CDC73	cna	amp	BASIS
PD5948	CDC73	cna	gain	BASIS

PD6406	CDC73	cna	gain	BASIS
PD6413	CDC73	cna	gain	BASIS
PD7067	CDC73	cna	amp	BASIS
PD7215	CDC73	cna	amp	BASIS
PD8621	CDC73	cna	gain	BASIS
PD8980	CDC73	cna	gain	BASIS
PD9004	CDC73	cna	gain	BASIS
PD9702	CDC73	cna	gain	BASIS
TCGA-A2-A25B	FCGR2B	cna	gain	TCGA
TCGA-AN-A0XU	FCGR2B	cna	gain	TCGA
TCGA-AO-A0JL	FCGR2B	cna	gain	TCGA
TCGA-BH-A0AW	FCGR2B	cna	gain	TCGA
TCGA-BH-A0C0	FCGR2B	cna	gain	TCGA
TCGA-BH-A18R	FCGR2B	cna	gain	TCGA
TCGA-BH-A1FU	FCGR2B	cna	gain	TCGA
TCGA-C8-A12L	FCGR2B	cna	gain	TCGA
TCGA-D8-A27M	FCGR2B	cna	amp	TCGA
TCGA-E2-A1L7	FCGR2B	cna	gain	TCGA
TCGA-EW-A1OX	FCGR2B	cna	gain	TCGA
TCGA-LL-A5YP	FCGR2B	cna	amp	TCGA
MB-0346	FCGR2B	cna	gain	METABRIC
MB-2827	FCGR2B	cna	amp	METABRIC
MB-5070	FCGR2B	cna	gain	METABRIC
MB-5465	FCGR2B	cna	amp	METABRIC
MB-6060	FCGR2B	cna	gain	METABRIC
MB-6098	FCGR2B	cna	amp	METABRIC
MB-7032	FCGR2B	cna	gain	METABRIC
MB-7038	FCGR2B	cna	gain	METABRIC
MB-0420	FCGR2B	cna	gain	METABRIC
PD10014	FCGR2B	cna	gain	BASIS
PD11327	FCGR2B	cna	gain	BASIS
PD13297	FCGR2B	cna	gain	BASIS
PD13299	FCGR2B	cna	gain	BASIS
PD14442	FCGR2B	cna	gain	BASIS
PD22355	FCGR2B	cna	gain	BASIS
PD23562	FCGR2B	cna	amp	BASIS
PD23574	FCGR2B	cna	amp	BASIS
PD24186	FCGR2B	cna	gain	BASIS
PD24206	FCGR2B	cna	gain	BASIS
PD3905	FCGR2B	cna	gain	BASIS
PD4005	FCGR2B	cna	gain	BASIS
PD4006	FCGR2B	cna	gain	BASIS
PD4107	FCGR2B	cna	gain	BASIS
PD4826	FCGR2B	cna	gain	BASIS
PD5935	FCGR2B	cna	gain	BASIS
PD5945	FCGR2B	cna	amp	BASIS
PD5948	FCGR2B	cna	gain	BASIS

PD6406	FCGR2B	cna	gain	BASIS
PD6413	FCGR2B	cna	gain	BASIS
PD7067	FCGR2B	cna	amp	BASIS
PD7215	FCGR2B	cna	gain	BASIS
PD8621	FCGR2B	cna	amp	BASIS
PD8980	FCGR2B	cna	gain	BASIS
PD9004	FCGR2B	cna	gain	BASIS
PD9702	FCGR2B	cna	gain	BASIS
TCGA-A2-A25B	INSRR	cna	gain	TCGA
TCGA-AN-A0XU	INSRR	cna	gain	TCGA
TCGA-AO-A0JL	INSRR	cna	gain	TCGA
TCGA-BH-A0AW	INSRR	cna	gain	TCGA
TCGA-BH-A0C0	INSRR	cna	hetloss	TCGA
TCGA-BH-A18R	INSRR	cna	gain	TCGA
TCGA-BH-A1FU	INSRR	cna	gain	TCGA
TCGA-C8-A12L	INSRR	cna	gain	TCGA
TCGA-E2-A1L7	INSRR	cna	gain	TCGA
TCGA-EW-A1OX	INSRR	cna	gain	TCGA
TCGA-LL-A5YP	INSRR	cna	amp	TCGA
MB-2827	INSRR	cna	amp	METABRIC
MB-5070	INSRR	cna	gain	METABRIC
MB-5465	INSRR	cna	gain	METABRIC
MB-6060	INSRR	cna	gain	METABRIC
MB-6098	INSRR	cna	amp	METABRIC
MB-7032	INSRR	cna	gain	METABRIC
MB-7038	INSRR	cna	gain	METABRIC
PD10014	INSRR	cna	gain	BASIS
PD11327	INSRR	cna	gain	BASIS
PD13297	INSRR	cna	gain	BASIS
PD13299	INSRR	cna	gain	BASIS
PD14442	INSRR	cna	gain	BASIS
PD22355	INSRR	cna	gain	BASIS
PD23562	INSRR	cna	amp	BASIS
PD23574	INSRR	cna	amp	BASIS
PD24186	INSRR	cna	gain	BASIS
PD24206	INSRR	cna	gain	BASIS
PD24337	INSRR	cna	gain	BASIS
PD3890	INSRR	cna	gain	BASIS
PD3905	INSRR	cna	gain	BASIS
PD4005	INSRR	cna	gain	BASIS
PD4006	INSRR	cna	amp	BASIS
PD4107	INSRR	cna	gain	BASIS
PD4826	INSRR	cna	gain	BASIS
PD5930	INSRR	cna	gain	BASIS
PD5935	INSRR	cna	gain	BASIS
PD5945	INSRR	cna	amp	BASIS
PD5948	INSRR	cna	gain	BASIS

PD6406	INSRR	cna	gain	BASIS
PD6413	INSRR	cna	gain	BASIS
PD7067	INSRR	cna	gain	BASIS
PD7215	INSRR	cna	gain	BASIS
PD8621	INSRR	cna	amp	BASIS
PD8980	INSRR	cna	gain	BASIS
PD9004	INSRR	cna	amp	BASIS
PD9702	INSRR	cna	gain	BASIS
TCGA-A2-A25B	LGR6	cna	amp	TCGA
TCGA-AN-A0XU	LGR6	cna	hetloss	TCGA
TCGA-AO-A0JL	LGR6	cna	gain	TCGA
TCGA-BH-A0AW	LGR6	cna	gain	TCGA
TCGA-BH-A0C0	LGR6	cna	gain	TCGA
TCGA-BH-A18R	LGR6	cna	gain	TCGA
TCGA-BH-A1FU	LGR6	cna	gain	TCGA
TCGA-C8-A12L	LGR6	cna	gain	TCGA
TCGA-E2-A1L7	LGR6	cna	gain	TCGA
TCGA-E9-A1NC	LGR6	cna	gain	TCGA
TCGA-EW-A1OX	LGR6	cna	gain	TCGA
TCGA-LL-A5YP	LGR6	cna	gain	TCGA
MB-0346	LGR6	cna	gain	METABRIC
MB-5107	LGR6	cna	gain	METABRIC
MB-5465	LGR6	cna	gain	METABRIC
MB-6060	LGR6	cna	gain	METABRIC
MB-6098	LGR6	cna	gain	METABRIC
MB-7032	LGR6	cna	gain	METABRIC
MB-7048	LGR6	cna	gain	METABRIC
PD10014	LGR6	cna	gain	BASIS
PD13296	LGR6	cna	hetloss	BASIS
PD13297	LGR6	cna	gain	BASIS
PD13299	LGR6	cna	gain	BASIS
PD14442	LGR6	cna	gain	BASIS
PD23562	LGR6	cna	gain	BASIS
PD23574	LGR6	cna	gain	BASIS
PD24186	LGR6	cna	amp	BASIS
PD24206	LGR6	cna	gain	BASIS
PD24337	LGR6	cna	gain	BASIS
PD3890	LGR6	cna	gain	BASIS
PD3905	LGR6	cna	gain	BASIS
PD4005	LGR6	cna	gain	BASIS
PD4006	LGR6	cna	amp	BASIS
PD4107	LGR6	cna	gain	BASIS
PD4826	LGR6	cna	gain	BASIS
PD5935	LGR6	cna	gain	BASIS
PD5945	LGR6	cna	amp	BASIS
PD5948	LGR6	cna	gain	BASIS
PD6406	LGR6	cna	gain	BASIS

PD6413	LGR6	cna	gain	BASIS
PD6731	LGR6	cna	hetloss	BASIS
PD7067	LGR6	cna	gain	BASIS
PD7215	LGR6	cna	amp	BASIS
PD8621	LGR6	cna	gain	BASIS
PD8980	LGR6	cna	amp	BASIS
PD9004	LGR6	cna	gain	BASIS
PD9702	LGR6	cna	amp	BASIS
TCGA-A2-A25B	NTRK1	cna	gain	TCGA
TCGA-AN-A0XU	NTRK1	cna	gain	TCGA
TCGA-AO-A0JL	NTRK1	cna	gain	TCGA
TCGA-BH-A0AW	NTRK1	cna	gain	TCGA
TCGA-BH-A0C0	NTRK1	cna	hetloss	TCGA
TCGA-BH-A18R	NTRK1	cna	gain	TCGA
TCGA-BH-A1FU	NTRK1	cna	gain	TCGA
TCGA-C8-A12L	NTRK1	cna	gain	TCGA
TCGA-E2-A1L7	NTRK1	cna	gain	TCGA
TCGA-EW-A1OX	NTRK1	cna	gain	TCGA
TCGA-LL-A5YP	NTRK1	cna	amp	TCGA
MB-2827	NTRK1	cna	amp	METABRIC
MB-5070	NTRK1	cna	gain	METABRIC
MB-5465	NTRK1	cna	gain	METABRIC
MB-6060	NTRK1	cna	gain	METABRIC
MB-6098	NTRK1	cna	amp	METABRIC
MB-7032	NTRK1	cna	gain	METABRIC
MB-7038	NTRK1	cna	gain	METABRIC
PD10014	NTRK1	cna	gain	BASIS
PD11327	NTRK1	cna	gain	BASIS
PD13297	NTRK1	cna	gain	BASIS
PD13299	NTRK1	cna	gain	BASIS
PD14442	NTRK1	cna	gain	BASIS
PD22355	NTRK1	cna	gain	BASIS
PD23562	NTRK1	cna	amp	BASIS
PD23574	NTRK1	cna	amp	BASIS
PD24186	NTRK1	cna	gain	BASIS
PD24206	NTRK1	cna	gain	BASIS
PD24337	NTRK1	cna	gain	BASIS
PD3890	NTRK1	cna	gain	BASIS
PD3905	NTRK1	cna	gain	BASIS
PD4005	NTRK1	cna	gain	BASIS
PD4006	NTRK1	cna	amp	BASIS
PD4107	NTRK1	cna	gain	BASIS
PD4826	NTRK1	cna	gain	BASIS
PD5930	NTRK1	cna	gain	BASIS
PD5935	NTRK1	cna	gain	BASIS
PD5945	NTRK1	cna	amp	BASIS
PD5948	NTRK1	cna	gain	BASIS

PD6406	NTRK1	cna	gain	BASIS
PD6413	NTRK1	cna	gain	BASIS
PD7067	NTRK1	cna	gain	BASIS
PD7215	NTRK1	cna	gain	BASIS
PD8621	NTRK1	cna	amp	BASIS
PD8980	NTRK1	cna	gain	BASIS
PD9004	NTRK1	cna	amp	BASIS
PD9702	NTRK1	cna	gain	BASIS
TCGA-A2-A25B	PBX1	cna	gain	TCGA
TCGA-AN-A0XU	PBX1	cna	gain	TCGA
TCGA-AO-A0JL	PBX1	cna	gain	TCGA
TCGA-BH-A0AW	PBX1	cna	gain	TCGA
TCGA-BH-A0C0	PBX1	cna	gain	TCGA
TCGA-BH-A18R	PBX1	cna	gain	TCGA
TCGA-BH-A1FU	PBX1	cna	gain	TCGA
TCGA-C8-A12L	PBX1	cna	gain	TCGA
TCGA-E2-A1L7	PBX1	cna	gain	TCGA
TCGA-EW-A1OX	PBX1	cna	gain	TCGA
TCGA-LL-A5YP	PBX1	cna	amp	TCGA
MB-0346	PBX1	cna	gain	METABRIC
MB-5070	PBX1	cna	gain	METABRIC
MB-5465	PBX1	cna	amp	METABRIC
MB-6060	PBX1	cna	gain	METABRIC
MB-6098	PBX1	cna	amp	METABRIC
MB-7032	PBX1	cna	gain	METABRIC
MB-7038	PBX1	cna	gain	METABRIC
MB-0420	PBX1	cna	gain	METABRIC
MTS-T0064	PBX1	cna	gain	METABRIC
PD10014	PBX1	cna	gain	BASIS
PD11327	PBX1	cna	amp	BASIS
PD13297	PBX1	cna	gain	BASIS
PD13299	PBX1	cna	gain	BASIS
PD14442	PBX1	cna	gain	BASIS
PD22355	PBX1	cna	gain	BASIS
PD23562	PBX1	cna	amp	BASIS
PD23574	PBX1	cna	gain	BASIS
PD24186	PBX1	cna	gain	BASIS
PD24206	PBX1	cna	gain	BASIS
PD24337	PBX1	cna	hetloss	BASIS
PD3905	PBX1	cna	gain	BASIS
PD4005	PBX1	cna	gain	BASIS
PD4006	PBX1	cna	gain	BASIS
PD4107	PBX1	cna	gain	BASIS
PD4826	PBX1	cna	gain	BASIS
PD5935	PBX1	cna	gain	BASIS
PD5945	PBX1	cna	amp	BASIS
PD5948	PBX1	cna	gain	BASIS

PD6406	PBX1	cna	gain	BASIS
PD6413	PBX1	cna	gain	BASIS
PD7067	PBX1	cna	amp	BASIS
PD7215	PBX1	cna	gain	BASIS
PD8621	PBX1	cna	gain	BASIS
PD8980	PBX1	cna	gain	BASIS
PD9004	PBX1	cna	gain	BASIS
PD9702	PBX1	cna	gain	BASIS
TCGA-A2-A25B	PRKAR1A	cna	gain	TCGA
TCGA-AN-A0XU	PRKAR1A	cna	gain	TCGA
TCGA-AO-A0JL	PRKAR1A	cna	hetloss	TCGA
TCGA-BH-A0AW	PRKAR1A	cna	amp	TCGA
TCGA-BH-A0C0	PRKAR1A	cna	gain	TCGA
TCGA-BH-A18R	PRKAR1A	cna	amp	TCGA
TCGA-C8-A12L	PRKAR1A	cna	gain	TCGA
TCGA-D8-A27M	PRKAR1A	cna	gain	TCGA
TCGA-EW-A1OX	PRKAR1A	cna	amp	TCGA
TCGA-LL-A5YP	PRKAR1A	cna	amp	TCGA
MB-2827	PRKAR1A	cna	hetloss	METABRIC
MB-5070	PRKAR1A	cna	gain	METABRIC
MB-5107	PRKAR1A	cna	amp	METABRIC
MB-6098	PRKAR1A	cna	gain	METABRIC
MB-6271	PRKAR1A	cna	hetloss	METABRIC
MB-7048	PRKAR1A	cna	amp	METABRIC
MTS-T0064	PRKAR1A	cna	gain	METABRIC
P-0002023-T01-IM3	PRKAR1A	cna	amp	MSK-IMPACT
P-0002858-T01-IM3	PRKAR1A	cna	amp	MSK-IMPACT
PD11327	PRKAR1A	cna	amp	BASIS
PD11742	PRKAR1A	cna	gain	BASIS
PD13297	PRKAR1A	cna	gain	BASIS
PD23561	PRKAR1A	cna	gain	BASIS
PD23562	PRKAR1A	cna	gain	BASIS
PD23574	PRKAR1A	cna	gain	BASIS
PD24186	PRKAR1A	cna	gain	BASIS
PD24202	PRKAR1A	cna	gain	BASIS
PD24206	PRKAR1A	cna	hetloss	BASIS
PD24337	PRKAR1A	cna	hetloss	BASIS
PD3890	PRKAR1A	cna	gain	BASIS
PD3905	PRKAR1A	cna	gain	BASIS
PD4006	PRKAR1A	cna	gain	BASIS
PD4107	PRKAR1A	cna	gain	BASIS
PD4826	PRKAR1A	cna	amp	BASIS
PD4967	PRKAR1A	cna	hetloss	BASIS
PD5930	PRKAR1A	cna	gain	BASIS
PD5935	PRKAR1A	cna	gain	BASIS
PD5945	PRKAR1A	cna	amp	BASIS
PD5948	PRKAR1A	cna	gain	BASIS

PD6731	PRKAR1A	cna	hetloss	BASIS
PD7067	PRKAR1A	cna	gain	BASIS
PD7215	PRKAR1A	cna	gain	BASIS
PD8621	PRKAR1A	cna	gain	BASIS
PD8980	PRKAR1A	cna	gain	BASIS
PD9004	PRKAR1A	cna	gain	BASIS
PD9585	PRKAR1A	cna	gain	BASIS
PD9702	PRKAR1A	cna	gain	BASIS
TCGA-A2-A25B	RPTOR	cna	gain	TCGA
TCGA-AN-A0XU	RPTOR	cna	amp	TCGA
TCGA-AO-A0JL	RPTOR	cna	homdel	TCGA
TCGA-BH-A0AW	RPTOR	cna	amp	TCGA
TCGA-BH-A0C0	RPTOR	cna	gain	TCGA
TCGA-BH-A18R	RPTOR	cna	amp	TCGA
TCGA-C8-A12L	RPTOR	cna	gain	TCGA
TCGA-D8-A27M	RPTOR	cna	gain	TCGA
TCGA-E2-A1L7	RPTOR	cna	gain	TCGA
TCGA-EW-A1OX	RPTOR	cna	gain	TCGA
TCGA-LL-A5YP	RPTOR	cna	amp	TCGA
MB-0346	RPTOR	cna	gain	METABRIC
MB-2827	RPTOR	cna	hetloss	METABRIC
MB-5070	RPTOR	cna	amp	METABRIC
MB-5107	RPTOR	cna	amp	METABRIC
MB-5465	RPTOR	cna	gain	METABRIC
MB-6060	RPTOR	cna	hetloss	METABRIC
MB-6098	RPTOR	cna	gain	METABRIC
MB-6271	RPTOR	cna	hetloss	METABRIC
MTS-T0064	RPTOR	cna	gain	METABRIC
PD10014	RPTOR	cna	gain	BASIS
PD11327	RPTOR	cna	amp	BASIS
PD22355	RPTOR	cna	hetloss	BASIS
PD23561	RPTOR	cna	hetloss	BASIS
PD23574	RPTOR	cna	amp	BASIS
PD24186	RPTOR	cna	amp	BASIS
PD24202	RPTOR	cna	gain	BASIS
PD24206	RPTOR	cna	gain	BASIS
PD24337	RPTOR	cna	hetloss	BASIS
PD3890	RPTOR	cna	gain	BASIS
PD3905	RPTOR	cna	gain	BASIS
PD4006	RPTOR	cna	gain	BASIS
PD4107	RPTOR	cna	gain	BASIS
PD4826	RPTOR	cna	amp	BASIS
PD4967	RPTOR	cna	hetloss	BASIS
PD5930	RPTOR	cna	gain	BASIS
PD5935	RPTOR	cna	gain	BASIS
PD5945	RPTOR	cna	amp	BASIS
PD5948	RPTOR	cna	gain	BASIS

PD6413	RPTOR	cna	hetloss	BASIS
PD6731	RPTOR	cna	hetloss	BASIS
PD7067	RPTOR	cna	gain	BASIS
PD7215	RPTOR	cna	gain	BASIS
PD8621	RPTOR	cna	gain	BASIS
PD9004	RPTOR	cna	gain	BASIS
PD9585	RPTOR	cna	gain	BASIS
PD9702	RPTOR	cna	gain	BASIS
TCGA-A2-A25B	SPOP	cna	amp	TCGA
TCGA-AN-A0XU	SPOP	cna	hetloss	TCGA
TCGA-AO-A0JL	SPOP	cna	hetloss	TCGA
TCGA-BH-A0AW	SPOP	cna	amp	TCGA
TCGA-BH-A0C0	SPOP	cna	gain	TCGA
TCGA-BH-A18R	SPOP	cna	amp	TCGA
TCGA-BH-A1FU	SPOP	cna	homdel	TCGA
TCGA-C8-A12L	SPOP	cna	amp	TCGA
TCGA-D8-A27M	SPOP	cna	hetloss	TCGA
TCGA-E9-A1NC	SPOP	cna	hetloss	TCGA
TCGA-EW-A1OX	SPOP	cna	amp	TCGA
MB-0346	SPOP	cna	gain	METABRIC
MB-2827	SPOP	cna	hetloss	METABRIC
MB-5070	SPOP	cna	hetloss	METABRIC
MB-5107	SPOP	cna	amp	METABRIC
MB-6060	SPOP	cna	amp	METABRIC
MB-6098	SPOP	cna	hetloss	METABRIC
MB-6271	SPOP	cna	hetloss	METABRIC
MB-7048	SPOP	cna	gain	METABRIC
P-0002023-T01-IM3	SPOP	cna	amp	MSK-IMPACT
P-0002858-T01-IM3	SPOP	cna	amp	MSK-IMPACT
PD11327	SPOP	cna	gain	BASIS
PD11742	SPOP	cna	gain	BASIS
PD13296	SPOP	cna	gain	BASIS
PD13297	SPOP	cna	hetloss	BASIS
PD13299	SPOP	cna	gain	BASIS
PD13771	SPOP	cna	gain	BASIS
PD23561	SPOP	cna	amp	BASIS
PD23562	SPOP	cna	gain	BASIS
PD23574	SPOP	cna	gain	BASIS
PD24186	SPOP	cna	gain	BASIS
PD24206	SPOP	cna	hetloss	BASIS
PD24337	SPOP	cna	hetloss	BASIS
PD3890	SPOP	cna	hetloss	BASIS
PD3905	SPOP	cna	gain	BASIS
PD4005	SPOP	cna	gain	BASIS
PD4006	SPOP	cna	amp	BASIS
PD4826	SPOP	cna	gain	BASIS
PD4967	SPOP	cna	hetloss	BASIS

PD5945	SPOP	cna	amp	BASIS
PD5948	SPOP	cna	gain	BASIS
PD6406	SPOP	cna	hetloss	BASIS
PD6731	SPOP	cna	hetloss	BASIS
PD7067	SPOP	cna	gain	BASIS
PD7215	SPOP	cna	gain	BASIS
PD9004	SPOP	cna	gain	BASIS
PD9702	SPOP	cna	gain	BASIS
TCGA-A2-A25B	TPR	cna	amp	TCGA
TCGA-AN-A0XU	TPR	cna	hetloss	TCGA
TCGA-AO-A0JL	TPR	cna	gain	TCGA
TCGA-BH-A0AW	TPR	cna	gain	TCGA
TCGA-BH-A0C0	TPR	cna	gain	TCGA
TCGA-BH-A18R	TPR	cna	gain	TCGA
TCGA-BH-A1FU	TPR	cna	gain	TCGA
TCGA-C8-A12L	TPR	cna	amp	TCGA
TCGA-E2-A1L7	TPR	cna	gain	TCGA
TCGA-E9-A1NC	TPR	cna	hetloss	TCGA
TCGA-EW-A1OX	TPR	cna	gain	TCGA
TCGA-LL-A5YP	TPR	cna	amp	TCGA
MB-0346	TPR	cna	gain	METABRIC
MB-5107	TPR	cna	gain	METABRIC
MB-5465	TPR	cna	amp	METABRIC
MB-6098	TPR	cna	gain	METABRIC
MB-7032	TPR	cna	gain	METABRIC
MB-7038	TPR	cna	amp	METABRIC
MB-7048	TPR	cna	gain	METABRIC
PD10014	TPR	cna	gain	BASIS
PD11327	TPR	cna	gain	BASIS
PD13297	TPR	cna	gain	BASIS
PD13299	TPR	cna	gain	BASIS
PD14442	TPR	cna	gain	BASIS
PD22355	TPR	cna	gain	BASIS
PD23562	TPR	cna	gain	BASIS
PD23574	TPR	cna	gain	BASIS
PD24186	TPR	cna	amp	BASIS
PD24206	TPR	cna	gain	BASIS
PD24337	TPR	cna	gain	BASIS
PD3890	TPR	cna	gain	BASIS
PD3905	TPR	cna	gain	BASIS
PD4005	TPR	cna	gain	BASIS
PD4006	TPR	cna	gain	BASIS
PD4107	TPR	cna	gain	BASIS
PD4826	TPR	cna	gain	BASIS
PD5935	TPR	cna	gain	BASIS
PD5945	TPR	cna	amp	BASIS
PD5948	TPR	cna	gain	BASIS

PD6406	TPR	cna	gain	BASIS
PD6413	TPR	cna	gain	BASIS
PD7067	TPR	cna	amp	BASIS
PD7215	TPR	cna	amp	BASIS
PD8621	TPR	cna	gain	BASIS
PD8980	TPR	cna	gain	BASIS
PD9004	TPR	cna	gain	BASIS
PD9702	TPR	cna	gain	BASIS
TCGA-A2-A25B	ABL2	cna	amp	TCGA
TCGA-AN-A0XU	ABL2	cna	gain	TCGA
TCGA-AO-A0JL	ABL2	cna	gain	TCGA
TCGA-BH-A0AW	ABL2	cna	gain	TCGA
TCGA-BH-A0C0	ABL2	cna	gain	TCGA
TCGA-BH-A18R	ABL2	cna	gain	TCGA
TCGA-BH-A1FU	ABL2	cna	gain	TCGA
TCGA-C8-A12L	ABL2	cna	gain	TCGA
TCGA-E2-A1L7	ABL2	cna	gain	TCGA
TCGA-E9-A1NC	ABL2	cna	hetloss	TCGA
TCGA-EW-A1OX	ABL2	cna	gain	TCGA
TCGA-LL-A5YP	ABL2	cna	amp	TCGA
MB-0346	ABL2	cna	gain	METABRIC
MB-2827	ABL2	cna	hetloss	METABRIC
MB-5465	ABL2	cna	gain	METABRIC
MB-6098	ABL2	cna	gain	METABRIC
MB-7032	ABL2	cna	gain	METABRIC
MB-7048	ABL2	cna	gain	METABRIC
PD10014	ABL2	cna	gain	BASIS
PD13297	ABL2	cna	gain	BASIS
PD13299	ABL2	cna	gain	BASIS
PD13771	ABL2	cna	hetloss	BASIS
PD14442	ABL2	cna	gain	BASIS
PD22355	ABL2	cna	gain	BASIS
PD23562	ABL2	cna	gain	BASIS
PD23574	ABL2	cna	gain	BASIS
PD24186	ABL2	cna	amp	BASIS
PD24206	ABL2	cna	hetloss	BASIS
PD24337	ABL2	cna	gain	BASIS
PD3890	ABL2	cna	gain	BASIS
PD3905	ABL2	cna	gain	BASIS
PD4005	ABL2	cna	gain	BASIS
PD4006	ABL2	cna	gain	BASIS
PD4107	ABL2	cna	gain	BASIS
PD4826	ABL2	cna	gain	BASIS
PD5935	ABL2	cna	gain	BASIS
PD5945	ABL2	cna	amp	BASIS
PD5948	ABL2	cna	gain	BASIS
PD6406	ABL2	cna	gain	BASIS

PD6413	ABL2	cna	gain	BASIS
PD7067	ABL2	cna	amp	BASIS
PD7215	ABL2	cna	amp	BASIS
PD8621	ABL2	cna	gain	BASIS
PD8980	ABL2	cna	gain	BASIS
PD9004	ABL2	cna	gain	BASIS
PD9702	ABL2	cna	gain	BASIS
TCGA-A2-A25B	ASPSCR1	cna	gain	TCGA
TCGA-AN-A0XU	ASPSCR1	cna	amp	TCGA
TCGA-AO-A0JL	ASPSCR1	cna	hetloss	TCGA
TCGA-BH-A0AW	ASPSCR1	cna	amp	TCGA
TCGA-BH-A0C0	ASPSCR1	cna	amp	TCGA
TCGA-BH-A18R	ASPSCR1	cna	homdel	TCGA
TCGA-C8-A12L	ASPSCR1	cna	gain	TCGA
TCGA-D8-A27M	ASPSCR1	cna	gain	TCGA
TCGA-E2-A1L7	ASPSCR1	cna	gain	TCGA
TCGA-EW-A1OX	ASPSCR1	cna	gain	TCGA
TCGA-LL-A5YP	ASPSCR1	cna	amp	TCGA
MB-0346	ASPSCR1	cna	gain	METABRIC
MB-2827	ASPSCR1	cna	hetloss	METABRIC
MB-5070	ASPSCR1	cna	gain	METABRIC
MB-5107	ASPSCR1	cna	amp	METABRIC
MB-5465	ASPSCR1	cna	gain	METABRIC
MB-6060	ASPSCR1	cna	hetloss	METABRIC
MB-6098	ASPSCR1	cna	gain	METABRIC
MB-6271	ASPSCR1	cna	hetloss	METABRIC
MTS-T0064	ASPSCR1	cna	gain	METABRIC
PD10014	ASPSCR1	cna	gain	BASIS
PD11327	ASPSCR1	cna	amp	BASIS
PD22355	ASPSCR1	cna	hetloss	BASIS
PD23561	ASPSCR1	cna	hetloss	BASIS
PD23574	ASPSCR1	cna	amp	BASIS
PD24186	ASPSCR1	cna	amp	BASIS
PD24202	ASPSCR1	cna	gain	BASIS
PD24337	ASPSCR1	cna	hetloss	BASIS
PD3890	ASPSCR1	cna	gain	BASIS
PD3905	ASPSCR1	cna	gain	BASIS
PD4006	ASPSCR1	cna	gain	BASIS
PD4107	ASPSCR1	cna	gain	BASIS
PD4826	ASPSCR1	cna	amp	BASIS
PD4967	ASPSCR1	cna	hetloss	BASIS
PD5930	ASPSCR1	cna	gain	BASIS
PD5935	ASPSCR1	cna	gain	BASIS
PD5945	ASPSCR1	cna	amp	BASIS
PD5948	ASPSCR1	cna	gain	BASIS
PD6413	ASPSCR1	cna	hetloss	BASIS
PD6731	ASPSCR1	cna	hetloss	BASIS

PD7067	ASPSCR1	cna	gain	BASIS
PD7215	ASPSCR1	cna	gain	BASIS
PD8621	ASPSCR1	cna	gain	BASIS
PD9004	ASPSCR1	cna	gain	BASIS
PD9585	ASPSCR1	cna	gain	BASIS
PD9702	ASPSCR1	cna	gain	BASIS
TCGA-A2-A25B	CANT1	cna	amp	TCGA
TCGA-AN-A0XU	CANT1	cna	amp	TCGA
TCGA-AO-A0JL	CANT1	cna	hetloss	TCGA
TCGA-BH-A0AW	CANT1	cna	amp	TCGA
TCGA-BH-A0C0	CANT1	cna	gain	TCGA
TCGA-BH-A18R	CANT1	cna	amp	TCGA
TCGA-C8-A12L	CANT1	cna	gain	TCGA
TCGA-D8-A27M	CANT1	cna	gain	TCGA
TCGA-E2-A1L7	CANT1	cna	gain	TCGA
TCGA-EW-A1OX	CANT1	cna	amp	TCGA
TCGA-LL-A5YP	CANT1	cna	amp	TCGA
MB-2827	CANT1	cna	hetloss	METABRIC
MB-5070	CANT1	cna	amp	METABRIC
MB-5107	CANT1	cna	amp	METABRIC
MB-5465	CANT1	cna	gain	METABRIC
MB-6098	CANT1	cna	gain	METABRIC
MB-6271	CANT1	cna	hetloss	METABRIC
MB-7048	CANT1	cna	gain	METABRIC
MTS-T0064	CANT1	cna	gain	METABRIC
PD10014	CANT1	cna	gain	BASIS
PD11327	CANT1	cna	amp	BASIS
PD22355	CANT1	cna	hetloss	BASIS
PD23561	CANT1	cna	hetloss	BASIS
PD23574	CANT1	cna	amp	BASIS
PD24186	CANT1	cna	amp	BASIS
PD24202	CANT1	cna	gain	BASIS
PD24206	CANT1	cna	hetloss	BASIS
PD24337	CANT1	cna	gain	BASIS
PD3890	CANT1	cna	gain	BASIS
PD3905	CANT1	cna	gain	BASIS
PD4006	CANT1	cna	gain	BASIS
PD4107	CANT1	cna	gain	BASIS
PD4826	CANT1	cna	amp	BASIS
PD4967	CANT1	cna	hetloss	BASIS
PD5930	CANT1	cna	gain	BASIS
PD5935	CANT1	cna	gain	BASIS
PD5945	CANT1	cna	amp	BASIS
PD5948	CANT1	cna	gain	BASIS
PD6413	CANT1	cna	hetloss	BASIS
PD6731	CANT1	cna	hetloss	BASIS
PD7067	CANT1	cna	gain	BASIS

PD7215	CANT1	cna	gain	BASIS
PD8621	CANT1	cna	gain	BASIS
PD9004	CANT1	cna	gain	BASIS
PD9585	CANT1	cna	gain	BASIS
PD9702	CANT1	cna	gain	BASIS
TCGA-A2-A25B	CDK12	cna	hetloss	TCGA
TCGA-AN-A0XU	CDK12	cna	gain	TCGA
TCGA-AO-A0JL	CDK12	cna	amp	TCGA
TCGA-BH-A0AW	CDK12	cna	amp	TCGA
TCGA-BH-A0C0	CDK12	cna	gain	TCGA
TCGA-BH-A18R	CDK12	cna	amp	TCGA
TCGA-BH-A1FU	CDK12	cna	amp	TCGA
TCGA-C8-A12L	CDK12	cna	amp	TCGA
TCGA-D8-A27M	CDK12	cna	hetloss	TCGA
TCGA-E2-A1L7	CDK12	cna	hetloss	TCGA
TCGA-E9-A1NC	CDK12	cna	hetloss	TCGA
MB-0346	CDK12	cna	hetloss	METABRIC
MB-2827	CDK12	cna	hetloss	METABRIC
MB-5070	CDK12	cna	hetloss	METABRIC
MB-5107	CDK12	cna	gain	METABRIC
MB-5465	CDK12	cna	hetloss	METABRIC
MB-6060	CDK12	cna	amp	METABRIC
MB-6098	CDK12	cna	hetloss	METABRIC
MB-6271	CDK12	cna	hetloss	METABRIC
MB-7038	CDK12	cna	hetloss	METABRIC
MB-7048	CDK12	cna	gain	METABRIC
MB-0420	CDK12	cna	hetloss	METABRIC
P-0002023-T01-IM3	CDK12	cna	amp	MSK-IMPACT
P-0002858-T01-IM3	CDK12	cna	amp	MSK-IMPACT
P-0009557-T01-IM5	CDK12	cna	homdel	MSK-IMPACT
PD13297	CDK12	cna	hetloss	BASIS
PD13299	CDK12	cna	gain	BASIS
PD23561	CDK12	cna	amp	BASIS
PD23562	CDK12	cna	gain	BASIS
PD24186	CDK12	cna	gain	BASIS
PD24206	CDK12	cna	hetloss	BASIS
PD24337	CDK12	cna	hetloss	BASIS
PD3890	CDK12	cna	hetloss	BASIS
PD3905	CDK12	cna	gain	BASIS
PD4005	CDK12	cna	hetloss	BASIS
PD4826	CDK12	cna	amp	BASIS
PD4967	CDK12	cna	hetloss	BASIS
PD5945	CDK12	cna	amp	BASIS
PD5948	CDK12	cna	gain	BASIS
PD6406	CDK12	cna	hetloss	BASIS
PD6731	CDK12	cna	hetloss	BASIS
PD7067	CDK12	cna	gain	BASIS

PD7215	CDK12	cna	gain	BASIS
PD9004	CDK12	cna	gain	BASIS
PD9585	CDK12	cna	hetloss	BASIS
PD9702	CDK12	cna	gain	BASIS
TCGA-A2-A25B	CHD1	cna	hetloss	TCGA
TCGA-AN-A0XU	CHD1	cna	hetloss	TCGA
TCGA-AO-A0JL	CHD1	cna	hetloss	TCGA
TCGA-BH-A0C0	CHD1	cna	hetloss	TCGA
TCGA-BH-A1FU	CHD1	cna	hetloss	TCGA
TCGA-C8-A12L	CHD1	cna	hetloss	TCGA
TCGA-D8-A27M	CHD1	cna	hetloss	TCGA
TCGA-E2-A1L7	CHD1	cna	hetloss	TCGA
TCGA-E9-A1NC	CHD1	cna	hetloss	TCGA
TCGA-LL-A5YP	CHD1	cna	hetloss	TCGA
MB-0346	CHD1	cna	gain	METABRIC
MB-2827	CHD1	cna	hetloss	METABRIC
MB-5070	CHD1	cna	hetloss	METABRIC
MB-5107	CHD1	cna	hetloss	METABRIC
MB-5465	CHD1	cna	hetloss	METABRIC
MB-6060	CHD1	cna	hetloss	METABRIC
MB-6098	CHD1	cna	hetloss	METABRIC
MB-7038	CHD1	cna	hetloss	METABRIC
MB-0420	CHD1	cna	hetloss	METABRIC
PD10014	CHD1	cna	hetloss	BASIS
PD13296	CHD1	cna	hetloss	BASIS
PD13297	CHD1	cna	hetloss	BASIS
PD13299	CHD1	cna	hetloss	BASIS
PD13771	CHD1	cna	gain	BASIS
PD22355	CHD1	cna	hetloss	BASIS
PD23562	CHD1	cna	hetloss	BASIS
PD23578	CHD1	cna	hetloss	BASIS
PD24202	CHD1	cna	hetloss	BASIS
PD24206	CHD1	cna	gain	BASIS
PD24337	CHD1	cna	hetloss	BASIS
PD3890	CHD1	cna	hetloss	BASIS
PD3905	CHD1	cna	gain	BASIS
PD4005	CHD1	cna	hetloss	BASIS
PD4006	CHD1	cna	hetloss	BASIS
PD4107	CHD1	cna	homdel	BASIS
PD5930	CHD1	cna	hetloss	BASIS
PD5935	CHD1	cna	hetloss	BASIS
PD5948	CHD1	cna	hetloss	BASIS
PD6406	CHD1	cna	hetloss	BASIS
PD6413	CHD1	cna	hetloss	BASIS
PD6731	CHD1	cna	hetloss	BASIS
PD7067	CHD1	cna	gain	BASIS
PD7215	CHD1	cna	hetloss	BASIS

PD8621	CHD1	cna	hetloss	BASIS
PD9004	CHD1	cna	gain	BASIS
PD9585	CHD1	cna	hetloss	BASIS
TCGA-A2-A25B	ELK4	cna	amp	TCGA
TCGA-AN-A0XU	ELK4	cna	hetloss	TCGA
TCGA-AO-A0JL	ELK4	cna	gain	TCGA
TCGA-BH-A0AW	ELK4	cna	gain	TCGA
TCGA-BH-A0C0	ELK4	cna	gain	TCGA
TCGA-BH-A18R	ELK4	cna	amp	TCGA
TCGA-BH-A1FU	ELK4	cna	gain	TCGA
TCGA-C8-A12L	ELK4	cna	gain	TCGA
TCGA-E2-A1L7	ELK4	cna	gain	TCGA
TCGA-E9-A1NC	ELK4	cna	gain	TCGA
TCGA-EW-A1OX	ELK4	cna	gain	TCGA
TCGA-LL-A5YP	ELK4	cna	hetloss	TCGA
MB-0346	ELK4	cna	gain	METABRIC
MB-5107	ELK4	cna	gain	METABRIC
MB-5465	ELK4	cna	gain	METABRIC
MB-6060	ELK4	cna	gain	METABRIC
MB-6098	ELK4	cna	gain	METABRIC
MB-7032	ELK4	cna	gain	METABRIC
MB-7038	ELK4	cna	gain	METABRIC
MB-7048	ELK4	cna	gain	METABRIC
PD10014	ELK4	cna	gain	BASIS
PD13296	ELK4	cna	hetloss	BASIS
PD13299	ELK4	cna	gain	BASIS
PD14442	ELK4	cna	gain	BASIS
PD23562	ELK4	cna	gain	BASIS
PD23574	ELK4	cna	gain	BASIS
PD24186	ELK4	cna	amp	BASIS
PD24206	ELK4	cna	gain	BASIS
PD24337	ELK4	cna	gain	BASIS
PD3890	ELK4	cna	gain	BASIS
PD3905	ELK4	cna	gain	BASIS
PD4005	ELK4	cna	gain	BASIS
PD4006	ELK4	cna	amp	BASIS
PD4107	ELK4	cna	gain	BASIS
PD4826	ELK4	cna	gain	BASIS
PD5935	ELK4	cna	gain	BASIS
PD5945	ELK4	cna	amp	BASIS
PD5948	ELK4	cna	amp	BASIS
PD6413	ELK4	cna	gain	BASIS
PD6731	ELK4	cna	hetloss	BASIS
PD7067	ELK4	cna	gain	BASIS
PD7215	ELK4	cna	amp	BASIS
PD8621	ELK4	cna	gain	BASIS
PD8980	ELK4	cna	amp	BASIS

PD9004	ELK4	cna	gain	BASIS
PD9702	ELK4	cna	amp	BASIS
TCGA-A2-A25B	ERBB2	cna	hetloss	TCGA
TCGA-AN-A0XU	ERBB2	cna	gain	TCGA
TCGA-AO-A0JL	ERBB2	cna	amp	TCGA
TCGA-BH-A0AW	ERBB2	cna	amp	TCGA
TCGA-BH-A0C0	ERBB2	cna	gain	TCGA
TCGA-BH-A18R	ERBB2	cna	amp	TCGA
TCGA-BH-A1FU	ERBB2	cna	amp	TCGA
TCGA-C8-A12L	ERBB2	cna	amp	TCGA
TCGA-D8-A27M	ERBB2	cna	hetloss	TCGA
TCGA-E2-A1L7	ERBB2	cna	hetloss	TCGA
TCGA-E9-A1NC	ERBB2	cna	hetloss	TCGA
MB-0346	ERBB2	cna	amp	METABRIC
MB-2827	ERBB2	cna	hetloss	METABRIC
MB-5070	ERBB2	cna	hetloss	METABRIC
MB-5107	ERBB2	cna	gain	METABRIC
MB-5465	ERBB2	cna	hetloss	METABRIC
MB-6060	ERBB2	cna	amp	METABRIC
MB-6098	ERBB2	cna	hetloss	METABRIC
MB-6271	ERBB2	cna	hetloss	METABRIC
MB-7038	ERBB2	cna	hetloss	METABRIC
MB-7048	ERBB2	cna	gain	METABRIC
MB-0420	ERBB2	cna	hetloss	METABRIC
P-0002023-T01-IM3	ERBB2	cna	amp	MSK-IMPACT
P-0002858-T01-IM3	ERBB2	cna	amp	MSK-IMPACT
P-0009557-T01-IM5	ERBB2	cna	amp	MSK-IMPACT
PD13297	ERBB2	cna	hetloss	BASIS
PD13299	ERBB2	cna	gain	BASIS
PD23561	ERBB2	cna	amp	BASIS
PD23562	ERBB2	cna	gain	BASIS
PD24186	ERBB2	cna	gain	BASIS
PD24206	ERBB2	cna	hetloss	BASIS
PD24337	ERBB2	cna	hetloss	BASIS
PD3890	ERBB2	cna	hetloss	BASIS
PD3905	ERBB2	cna	gain	BASIS
PD4005	ERBB2	cna	hetloss	BASIS
PD4826	ERBB2	cna	amp	BASIS
PD4967	ERBB2	cna	hetloss	BASIS
PD5945	ERBB2	cna	amp	BASIS
PD5948	ERBB2	cna	gain	BASIS
PD6406	ERBB2	cna	hetloss	BASIS
PD6731	ERBB2	cna	hetloss	BASIS
PD7067	ERBB2	cna	gain	BASIS
PD7215	ERBB2	cna	gain	BASIS
PD9004	ERBB2	cna	gain	BASIS
PD9585	ERBB2	cna	hetloss	BASIS

PD9702	ERBB2	cna	amp	BASIS
TCGA-A2-A25B	FCRL4	cna	gain	TCGA
TCGA-AN-A0XU	FCRL4	cna	gain	TCGA
TCGA-AO-A0JL	FCRL4	cna	gain	TCGA
TCGA-BH-A0AW	FCRL4	cna	gain	TCGA
TCGA-BH-A0C0	FCRL4	cna	hetloss	TCGA
TCGA-BH-A18R	FCRL4	cna	gain	TCGA
TCGA-BH-A1FU	FCRL4	cna	gain	TCGA
TCGA-C8-A12L	FCRL4	cna	gain	TCGA
TCGA-E2-A1L7	FCRL4	cna	gain	TCGA
TCGA-EW-A1OX	FCRL4	cna	gain	TCGA
TCGA-LL-A5YP	FCRL4	cna	amp	TCGA
MB-2827	FCRL4	cna	amp	METABRIC
MB-5070	FCRL4	cna	gain	METABRIC
MB-5465	FCRL4	cna	gain	METABRIC
MB-6060	FCRL4	cna	gain	METABRIC
MB-6098	FCRL4	cna	amp	METABRIC
MB-7032	FCRL4	cna	gain	METABRIC
MB-7038	FCRL4	cna	gain	METABRIC
PD10014	FCRL4	cna	gain	BASIS
PD11327	FCRL4	cna	amp	BASIS
PD13297	FCRL4	cna	gain	BASIS
PD13299	FCRL4	cna	gain	BASIS
PD14442	FCRL4	cna	gain	BASIS
PD22355	FCRL4	cna	gain	BASIS
PD23562	FCRL4	cna	amp	BASIS
PD23574	FCRL4	cna	amp	BASIS
PD24186	FCRL4	cna	gain	BASIS
PD24206	FCRL4	cna	gain	BASIS
PD3890	FCRL4	cna	gain	BASIS
PD3905	FCRL4	cna	gain	BASIS
PD4005	FCRL4	cna	gain	BASIS
PD4006	FCRL4	cna	amp	BASIS
PD4107	FCRL4	cna	gain	BASIS
PD4826	FCRL4	cna	gain	BASIS
PD5930	FCRL4	cna	gain	BASIS
PD5935	FCRL4	cna	gain	BASIS
PD5945	FCRL4	cna	amp	BASIS
PD5948	FCRL4	cna	gain	BASIS
PD6406	FCRL4	cna	gain	BASIS
PD6413	FCRL4	cna	gain	BASIS
PD7067	FCRL4	cna	gain	BASIS
PD7215	FCRL4	cna	gain	BASIS
PD8621	FCRL4	cna	amp	BASIS
PD8980	FCRL4	cna	gain	BASIS
PD9004	FCRL4	cna	gain	BASIS
PD9702	FCRL4	cna	gain	BASIS

TCGA-A2-A25B	MAP3K14	cna	hetloss	TCGA
TCGA-AN-A0XU	MAP3K14	cna	hetloss	TCGA
TCGA-AO-A0JL	MAP3K14	cna	hetloss	TCGA
TCGA-BH-A0AW	MAP3K14	cna	homdel	TCGA
TCGA-BH-A0C0	MAP3K14	cna	gain	TCGA
TCGA-BH-A18R	MAP3K14	cna	homdel	TCGA
TCGA-BH-A1FU	MAP3K14	cna	homdel	TCGA
TCGA-C8-A12L	MAP3K14	cna	hetloss	TCGA
TCGA-D8-A27M	MAP3K14	cna	hetloss	TCGA
TCGA-E2-A1L7	MAP3K14	cna	hetloss	TCGA
TCGA-E9-A1NC	MAP3K14	cna	hetloss	TCGA
TCGA-EW-A1OX	MAP3K14	cna	homdel	TCGA
MB-0346	MAP3K14	cna	hetloss	METABRIC
MB-2827	MAP3K14	cna	hetloss	METABRIC
MB-5070	MAP3K14	cna	hetloss	METABRIC
MB-5107	MAP3K14	cna	amp	METABRIC
MB-5465	MAP3K14	cna	hetloss	METABRIC
MB-6060	MAP3K14	cna	hetloss	METABRIC
MB-6098	MAP3K14	cna	hetloss	METABRIC
MB-6271	MAP3K14	cna	hetloss	METABRIC
MB-7038	MAP3K14	cna	hetloss	METABRIC
MB-7048	MAP3K14	cna	hetloss	METABRIC
P-0009557-T01-IM5	MAP3K14	cna	homdel	MSK-IMPACT
PD13296	MAP3K14	cna	gain	BASIS
PD13297	MAP3K14	cna	hetloss	BASIS
PD13299	MAP3K14	cna	gain	BASIS
PD13771	MAP3K14	cna	homdel	BASIS
PD23561	MAP3K14	cna	hetloss	BASIS
PD23562	MAP3K14	cna	gain	BASIS
PD23574	MAP3K14	cna	gain	BASIS
PD24186	MAP3K14	cna	gain	BASIS
PD24206	MAP3K14	cna	hetloss	BASIS
PD24337	MAP3K14	cna	hetloss	BASIS
PD3890	MAP3K14	cna	hetloss	BASIS
PD3905	MAP3K14	cna	gain	BASIS
PD4006	MAP3K14	cna	gain	BASIS
PD4826	MAP3K14	cna	hetloss	BASIS
PD4967	MAP3K14	cna	hetloss	BASIS
PD5945	MAP3K14	cna	amp	BASIS
PD6406	MAP3K14	cna	hetloss	BASIS
PD6731	MAP3K14	cna	hetloss	BASIS
PD7067	MAP3K14	cna	gain	BASIS
PD7215	MAP3K14	cna	gain	BASIS
PD9004	MAP3K14	cna	gain	BASIS
PD9585	MAP3K14	cna	hetloss	BASIS
PD9702	MAP3K14	cna	amp	BASIS
TCGA-A2-A25B	MDM4	cna	amp	TCGA

TCGA-AN-A0XU	MDM4	cna	hetloss	TCGA
TCGA-AO-A0JL	MDM4	cna	gain	TCGA
TCGA-BH-A0AW	MDM4	cna	gain	TCGA
TCGA-BH-A0C0	MDM4	cna	gain	TCGA
TCGA-BH-A18R	MDM4	cna	gain	TCGA
TCGA-BH-A1FU	MDM4	cna	gain	TCGA
TCGA-C8-A12L	MDM4	cna	gain	TCGA
TCGA-E2-A1L7	MDM4	cna	gain	TCGA
TCGA-E9-A1NC	MDM4	cna	gain	TCGA
TCGA-EW-A1OX	MDM4	cna	gain	TCGA
TCGA-LL-A5YP	MDM4	cna	gain	TCGA
MB-0346	MDM4	cna	gain	METABRIC
MB-5107	MDM4	cna	gain	METABRIC
MB-5465	MDM4	cna	gain	METABRIC
MB-6060	MDM4	cna	gain	METABRIC
MB-6098	MDM4	cna	gain	METABRIC
MB-7032	MDM4	cna	gain	METABRIC
MB-7038	MDM4	cna	gain	METABRIC
MB-7048	MDM4	cna	gain	METABRIC
PD10014	MDM4	cna	gain	BASIS
PD13296	MDM4	cna	hetloss	BASIS
PD13299	MDM4	cna	gain	BASIS
PD14442	MDM4	cna	gain	BASIS
PD23562	MDM4	cna	gain	BASIS
PD23574	MDM4	cna	gain	BASIS
PD24186	MDM4	cna	amp	BASIS
PD24206	MDM4	cna	gain	BASIS
PD24337	MDM4	cna	gain	BASIS
PD3890	MDM4	cna	gain	BASIS
PD3905	MDM4	cna	gain	BASIS
PD4005	MDM4	cna	gain	BASIS
PD4006	MDM4	cna	amp	BASIS
PD4107	MDM4	cna	gain	BASIS
PD4826	MDM4	cna	gain	BASIS
PD5935	MDM4	cna	gain	BASIS
PD5945	MDM4	cna	amp	BASIS
PD5948	MDM4	cna	amp	BASIS
PD6413	MDM4	cna	gain	BASIS
PD6731	MDM4	cna	hetloss	BASIS
PD7067	MDM4	cna	gain	BASIS
PD7215	MDM4	cna	amp	BASIS
PD8621	MDM4	cna	gain	BASIS
PD8980	MDM4	cna	amp	BASIS
PD9004	MDM4	cna	gain	BASIS
PD9702	MDM4	cna	amp	BASIS
TCGA-A2-A25B	MEF2C	cna	hetloss	TCGA
TCGA-AN-A0XU	MEF2C	cna	hetloss	TCGA

TCGA-AO-A0JL	MEF2C	cna	hetloss	TCGA
TCGA-BH-A0C0	MEF2C	cna	hetloss	TCGA
TCGA-BH-A1FU	MEF2C	cna	hetloss	TCGA
TCGA-C8-A12L	MEF2C	cna	hetloss	TCGA
TCGA-D8-A27M	MEF2C	cna	hetloss	TCGA
TCGA-E2-A1L7	MEF2C	cna	hetloss	TCGA
TCGA-E9-A1NC	MEF2C	cna	hetloss	TCGA
TCGA-LL-A5YP	MEF2C	cna	hetloss	TCGA
MB-0346	MEF2C	cna	gain	METABRIC
MB-2827	MEF2C	cna	hetloss	METABRIC
MB-5070	MEF2C	cna	hetloss	METABRIC
MB-5107	MEF2C	cna	hetloss	METABRIC
MB-5465	MEF2C	cna	hetloss	METABRIC
MB-6060	MEF2C	cna	hetloss	METABRIC
MB-6098	MEF2C	cna	hetloss	METABRIC
MB-0420	MEF2C	cna	hetloss	METABRIC
MTS-T0064	MEF2C	cna	hetloss	METABRIC
PD10014	MEF2C	cna	hetloss	BASIS
PD11742	MEF2C	cna	hetloss	BASIS
PD13296	MEF2C	cna	hetloss	BASIS
PD13297	MEF2C	cna	hetloss	BASIS
PD13299	MEF2C	cna	hetloss	BASIS
PD13771	MEF2C	cna	gain	BASIS
PD22355	MEF2C	cna	hetloss	BASIS
PD23562	MEF2C	cna	hetloss	BASIS
PD24202	MEF2C	cna	hetloss	BASIS
PD24206	MEF2C	cna	gain	BASIS
PD24337	MEF2C	cna	hetloss	BASIS
PD3890	MEF2C	cna	hetloss	BASIS
PD3905	MEF2C	cna	gain	BASIS
PD4005	MEF2C	cna	homdel	BASIS
PD4006	MEF2C	cna	hetloss	BASIS
PD4107	MEF2C	cna	homdel	BASIS
PD5930	MEF2C	cna	hetloss	BASIS
PD5935	MEF2C	cna	hetloss	BASIS
PD5948	MEF2C	cna	hetloss	BASIS
PD6406	MEF2C	cna	hetloss	BASIS
PD6413	MEF2C	cna	hetloss	BASIS
PD6731	MEF2C	cna	hetloss	BASIS
PD7067	MEF2C	cna	gain	BASIS
PD7215	MEF2C	cna	hetloss	BASIS
PD8621	MEF2C	cna	hetloss	BASIS
PD8980	MEF2C	cna	hetloss	BASIS
PD9585	MEF2C	cna	hetloss	BASIS
TCGA-AO-A0JL	PDS5B	cna	hetloss	TCGA
TCGA-BH-A0AW	PDS5B	cna	hetloss	TCGA
TCGA-BH-A0C0	PDS5B	cna	gain	TCGA

TCGA-BH-A1FU	PDS5B	cna	hetloss	TCGA
TCGA-C8-A12L	PDS5B	cna	hetloss	TCGA
TCGA-D8-A27M	PDS5B	cna	hetloss	TCGA
TCGA-E9-A1NC	PDS5B	cna	hetloss	TCGA
TCGA-EW-A1OX	PDS5B	cna	homdel	TCGA
TCGA-LL-A5YP	PDS5B	cna	hetloss	TCGA
MB-0346	PDS5B	cna	hetloss	METABRIC
MB-2827	PDS5B	cna	hetloss	METABRIC
MB-5107	PDS5B	cna	hetloss	METABRIC
MB-6060	PDS5B	cna	gain	METABRIC
MB-6098	PDS5B	cna	hetloss	METABRIC
MB-7032	PDS5B	cna	hetloss	METABRIC
MB-0420	PDS5B	cna	hetloss	METABRIC
MTS-T0064	PDS5B	cna	hetloss	METABRIC
PD10014	PDS5B	cna	hetloss	BASIS
PD11742	PDS5B	cna	hetloss	BASIS
PD13296	PDS5B	cna	hetloss	BASIS
PD13297	PDS5B	cna	hetloss	BASIS
PD13771	PDS5B	cna	hetloss	BASIS
PD14442	PDS5B	cna	hetloss	BASIS
PD22355	PDS5B	cna	hetloss	BASIS
PD23562	PDS5B	cna	gain	BASIS
PD23578	PDS5B	cna	hetloss	BASIS
PD24202	PDS5B	cna	hetloss	BASIS
PD24206	PDS5B	cna	gain	BASIS
PD3890	PDS5B	cna	hetloss	BASIS
PD3905	PDS5B	cna	gain	BASIS
PD4005	PDS5B	cna	homdel	BASIS
PD4006	PDS5B	cna	hetloss	BASIS
PD4107	PDS5B	cna	gain	BASIS
PD4826	PDS5B	cna	hetloss	BASIS
PD4967	PDS5B	cna	hetloss	BASIS
PD5945	PDS5B	cna	amp	BASIS
PD5948	PDS5B	cna	gain	BASIS
PD6406	PDS5B	cna	hetloss	BASIS
PD6413	PDS5B	cna	hetloss	BASIS
PD6731	PDS5B	cna	hetloss	BASIS
PD7067	PDS5B	cna	gain	BASIS
PD7215	PDS5B	cna	gain	BASIS
PD8621	PDS5B	cna	hetloss	BASIS
PD8980	PDS5B	cna	hetloss	BASIS
PD9585	PDS5B	cna	gain	BASIS
PD9702	PDS5B	cna	gain	BASIS
TCGA-A2-A25B	PRCC	cna	gain	TCGA
TCGA-AN-A0XU	PRCC	cna	gain	TCGA
TCGA-AO-A0JL	PRCC	cna	gain	TCGA
TCGA-BH-A0AW	PRCC	cna	gain	TCGA

TCGA-BH-A0C0	PRCC	cna	hetloss	TCGA
TCGA-BH-A18R	PRCC	cna	gain	TCGA
TCGA-BH-A1FU	PRCC	cna	gain	TCGA
TCGA-C8-A12L	PRCC	cna	gain	TCGA
TCGA-E2-A1L7	PRCC	cna	gain	TCGA
TCGA-EW-A1OX	PRCC	cna	gain	TCGA
TCGA-LL-A5YP	PRCC	cna	amp	TCGA
MB-2827	PRCC	cna	amp	METABRIC
MB-5070	PRCC	cna	gain	METABRIC
MB-5465	PRCC	cna	gain	METABRIC
MB-6060	PRCC	cna	gain	METABRIC
MB-6098	PRCC	cna	amp	METABRIC
MB-7032	PRCC	cna	gain	METABRIC
MB-7038	PRCC	cna	gain	METABRIC
PD10014	PRCC	cna	gain	BASIS
PD11327	PRCC	cna	gain	BASIS
PD13297	PRCC	cna	gain	BASIS
PD13299	PRCC	cna	gain	BASIS
PD14442	PRCC	cna	gain	BASIS
PD22355	PRCC	cna	gain	BASIS
PD23562	PRCC	cna	amp	BASIS
PD23574	PRCC	cna	amp	BASIS
PD24186	PRCC	cna	gain	BASIS
PD24206	PRCC	cna	gain	BASIS
PD3890	PRCC	cna	gain	BASIS
PD3905	PRCC	cna	gain	BASIS
PD4005	PRCC	cna	gain	BASIS
PD4006	PRCC	cna	amp	BASIS
PD4107	PRCC	cna	gain	BASIS
PD4826	PRCC	cna	gain	BASIS
PD5930	PRCC	cna	gain	BASIS
PD5935	PRCC	cna	gain	BASIS
PD5945	PRCC	cna	amp	BASIS
PD5948	PRCC	cna	gain	BASIS
PD6406	PRCC	cna	gain	BASIS
PD6413	PRCC	cna	gain	BASIS
PD7067	PRCC	cna	gain	BASIS
PD7215	PRCC	cna	gain	BASIS
PD8621	PRCC	cna	amp	BASIS
PD8980	PRCC	cna	gain	BASIS
PD9004	PRCC	cna	amp	BASIS
PD9702	PRCC	cna	amp	BASIS
TCGA-A2-A25B	RASA1	cna	hetloss	TCGA
TCGA-AN-A0XU	RASA1	cna	hetloss	TCGA
TCGA-AO-A0JL	RASA1	cna	hetloss	TCGA
TCGA-BH-A0C0	RASA1	cna	hetloss	TCGA
TCGA-BH-A1FU	RASA1	cna	hetloss	TCGA

TCGA-C8-A12L	RASA1	cna	hetloss	TCGA
TCGA-D8-A27M	RASA1	cna	hetloss	TCGA
TCGA-E2-A1L7	RASA1	cna	hetloss	TCGA
TCGA-E9-A1NC	RASA1	cna	hetloss	TCGA
TCGA-LL-A5YP	RASA1	cna	hetloss	TCGA
MB-0346	RASA1	cna	gain	METABRIC
MB-2827	RASA1	cna	hetloss	METABRIC
MB-5070	RASA1	cna	hetloss	METABRIC
MB-5107	RASA1	cna	hetloss	METABRIC
MB-5465	RASA1	cna	hetloss	METABRIC
MB-6060	RASA1	cna	hetloss	METABRIC
MB-6098	RASA1	cna	hetloss	METABRIC
MB-0420	RASA1	cna	hetloss	METABRIC
MTS-T0064	RASA1	cna	hetloss	METABRIC
PD10014	RASA1	cna	hetloss	BASIS
PD11742	RASA1	cna	hetloss	BASIS
PD13296	RASA1	cna	hetloss	BASIS
PD13297	RASA1	cna	hetloss	BASIS
PD13299	RASA1	cna	hetloss	BASIS
PD13771	RASA1	cna	gain	BASIS
PD22355	RASA1	cna	hetloss	BASIS
PD23562	RASA1	cna	hetloss	BASIS
PD24202	RASA1	cna	hetloss	BASIS
PD24206	RASA1	cna	gain	BASIS
PD24337	RASA1	cna	hetloss	BASIS
PD3890	RASA1	cna	hetloss	BASIS
PD3905	RASA1	cna	gain	BASIS
PD4005	RASA1	cna	hetloss	BASIS
PD4006	RASA1	cna	hetloss	BASIS
PD4107	RASA1	cna	homdel	BASIS
PD5930	RASA1	cna	hetloss	BASIS
PD5935	RASA1	cna	hetloss	BASIS
PD5948	RASA1	cna	hetloss	BASIS
PD6406	RASA1	cna	hetloss	BASIS
PD6413	RASA1	cna	hetloss	BASIS
PD6731	RASA1	cna	hetloss	BASIS
PD7067	RASA1	cna	gain	BASIS
PD7215	RASA1	cna	hetloss	BASIS
PD8621	RASA1	cna	hetloss	BASIS
PD8980	RASA1	cna	gain	BASIS
PD9585	RASA1	cna	hetloss	BASIS
TCGA-A2-A25B	RFWD2	cna	amp	TCGA
TCGA-AN-A0XU	RFWD2	cna	gain	TCGA
TCGA-AO-A0JL	RFWD2	cna	gain	TCGA
TCGA-BH-A0AW	RFWD2	cna	gain	TCGA
TCGA-BH-A0C0	RFWD2	cna	gain	TCGA
TCGA-BH-A18R	RFWD2	cna	gain	TCGA

TCGA-BH-A1FU	RFWD2	cna	gain	TCGA
TCGA-C8-A12L	RFWD2	cna	gain	TCGA
TCGA-E2-A1L7	RFWD2	cna	gain	TCGA
TCGA-E9-A1NC	RFWD2	cna	hetloss	TCGA
TCGA-EW-A1OX	RFWD2	cna	gain	TCGA
TCGA-LL-A5YP	RFWD2	cna	amp	TCGA
MB-0346	RFWD2	cna	gain	METABRIC
MB-5465	RFWD2	cna	amp	METABRIC
MB-6098	RFWD2	cna	amp	METABRIC
MB-7032	RFWD2	cna	gain	METABRIC
MB-7038	RFWD2	cna	gain	METABRIC
PD10014	RFWD2	cna	gain	BASIS
PD11327	RFWD2	cna	gain	BASIS
PD13297	RFWD2	cna	gain	BASIS
PD13299	RFWD2	cna	gain	BASIS
PD13771	RFWD2	cna	hetloss	BASIS
PD14442	RFWD2	cna	gain	BASIS
PD22355	RFWD2	cna	gain	BASIS
PD23562	RFWD2	cna	gain	BASIS
PD23574	RFWD2	cna	gain	BASIS
PD24186	RFWD2	cna	amp	BASIS
PD24206	RFWD2	cna	gain	BASIS
PD24337	RFWD2	cna	hetloss	BASIS
PD3890	RFWD2	cna	gain	BASIS
PD3905	RFWD2	cna	gain	BASIS
PD4005	RFWD2	cna	gain	BASIS
PD4006	RFWD2	cna	gain	BASIS
PD4107	RFWD2	cna	gain	BASIS
PD4826	RFWD2	cna	gain	BASIS
PD5935	RFWD2	cna	gain	BASIS
PD5945	RFWD2	cna	amp	BASIS
PD5948	RFWD2	cna	gain	BASIS
PD6406	RFWD2	cna	gain	BASIS
PD6413	RFWD2	cna	gain	BASIS
PD7067	RFWD2	cna	gain	BASIS
PD7215	RFWD2	cna	amp	BASIS
PD8621	RFWD2	cna	gain	BASIS
PD8980	RFWD2	cna	gain	BASIS
PD9004	RFWD2	cna	gain	BASIS
PD9702	RFWD2	cna	gain	BASIS
TCGA-A2-A25B	RIT1	cna	gain	TCGA
TCGA-AN-A0XU	RIT1	cna	gain	TCGA
TCGA-AO-A0JL	RIT1	cna	gain	TCGA
TCGA-BH-A0AW	RIT1	cna	gain	TCGA
TCGA-BH-A0C0	RIT1	cna	hetloss	TCGA
TCGA-BH-A18R	RIT1	cna	gain	TCGA
TCGA-BH-A1FU	RIT1	cna	gain	TCGA

TCGA-C8-A12L	RIT1	cna	gain	TCGA
TCGA-E2-A1L7	RIT1	cna	gain	TCGA
TCGA-EW-A1OX	RIT1	cna	gain	TCGA
TCGA-LL-A5YP	RIT1	cna	amp	TCGA
MB-2827	RIT1	cna	amp	METABRIC
MB-5070	RIT1	cna	gain	METABRIC
MB-6060	RIT1	cna	gain	METABRIC
MB-6098	RIT1	cna	amp	METABRIC
MB-7032	RIT1	cna	gain	METABRIC
MB-7038	RIT1	cna	gain	METABRIC
PD10014	RIT1	cna	gain	BASIS
PD11327	RIT1	cna	gain	BASIS
PD13297	RIT1	cna	gain	BASIS
PD13299	RIT1	cna	gain	BASIS
PD13771	RIT1	cna	gain	BASIS
PD14442	RIT1	cna	gain	BASIS
PD22355	RIT1	cna	gain	BASIS
PD23562	RIT1	cna	amp	BASIS
PD23574	RIT1	cna	amp	BASIS
PD24186	RIT1	cna	gain	BASIS
PD24206	RIT1	cna	gain	BASIS
PD3890	RIT1	cna	gain	BASIS
PD3905	RIT1	cna	gain	BASIS
PD4005	RIT1	cna	gain	BASIS
PD4006	RIT1	cna	amp	BASIS
PD4107	RIT1	cna	gain	BASIS
PD4826	RIT1	cna	gain	BASIS
PD5930	RIT1	cna	gain	BASIS
PD5935	RIT1	cna	gain	BASIS
PD5945	RIT1	cna	amp	BASIS
PD5948	RIT1	cna	gain	BASIS
PD6406	RIT1	cna	gain	BASIS
PD6413	RIT1	cna	gain	BASIS
PD7067	RIT1	cna	gain	BASIS
PD7215	RIT1	cna	gain	BASIS
PD8621	RIT1	cna	amp	BASIS
PD8980	RIT1	cna	gain	BASIS
PD9004	RIT1	cna	gain	BASIS
PD9702	RIT1	cna	amp	BASIS
TCGA-A2-A25B	RNF213	cna	gain	TCGA
TCGA-AN-A0XU	RNF213	cna	amp	TCGA
TCGA-AO-A0JL	RNF213	cna	hetloss	TCGA
TCGA-BH-A0AW	RNF213	cna	amp	TCGA
TCGA-BH-A0C0	RNF213	cna	gain	TCGA
TCGA-BH-A18R	RNF213	cna	amp	TCGA
TCGA-C8-A12L	RNF213	cna	gain	TCGA
TCGA-D8-A27M	RNF213	cna	gain	TCGA

TCGA-E2-A1L7	RNF213	cna	gain	TCGA
TCGA-EW-A1OX	RNF213	cna	amp	TCGA
TCGA-LL-A5YP	RNF213	cna	amp	TCGA
MB-0346	RNF213	cna	gain	METABRIC
MB-2827	RNF213	cna	hetloss	METABRIC
MB-5070	RNF213	cna	amp	METABRIC
MB-5107	RNF213	cna	amp	METABRIC
MB-5465	RNF213	cna	gain	METABRIC
MB-6060	RNF213	cna	hetloss	METABRIC
MB-6098	RNF213	cna	gain	METABRIC
MB-6271	RNF213	cna	hetloss	METABRIC
MTS-T0064	RNF213	cna	gain	METABRIC
PD10014	RNF213	cna	gain	BASIS
PD11327	RNF213	cna	amp	BASIS
PD22355	RNF213	cna	hetloss	BASIS
PD23561	RNF213	cna	hetloss	BASIS
PD23574	RNF213	cna	amp	BASIS
PD24186	RNF213	cna	amp	BASIS
PD24202	RNF213	cna	gain	BASIS
PD24206	RNF213	cna	hetloss	BASIS
PD3890	RNF213	cna	gain	BASIS
PD3905	RNF213	cna	gain	BASIS
PD4006	RNF213	cna	gain	BASIS
PD4107	RNF213	cna	gain	BASIS
PD4826	RNF213	cna	amp	BASIS
PD4967	RNF213	cna	hetloss	BASIS
PD5930	RNF213	cna	gain	BASIS
PD5935	RNF213	cna	gain	BASIS
PD5945	RNF213	cna	amp	BASIS
PD5948	RNF213	cna	gain	BASIS
PD6413	RNF213	cna	hetloss	BASIS
PD6731	RNF213	cna	hetloss	BASIS
PD7067	RNF213	cna	gain	BASIS
PD7215	RNF213	cna	gain	BASIS
PD8621	RNF213	cna	gain	BASIS
PD9004	RNF213	cna	gain	BASIS
PD9585	RNF213	cna	gain	BASIS
PD9702	RNF213	cna	gain	BASIS
TCGA-A2-A25B	SLC45A3	cna	amp	TCGA
TCGA-AN-A0XU	SLC45A3	cna	hetloss	TCGA
TCGA-AO-A0JL	SLC45A3	cna	gain	TCGA
TCGA-BH-A0AW	SLC45A3	cna	gain	TCGA
TCGA-BH-A0C0	SLC45A3	cna	gain	TCGA
TCGA-BH-A18R	SLC45A3	cna	amp	TCGA
TCGA-BH-A1FU	SLC45A3	cna	gain	TCGA
TCGA-C8-A12L	SLC45A3	cna	gain	TCGA
TCGA-E2-A1L7	SLC45A3	cna	gain	TCGA

TCGA-E9-A1NC	SLC45A3	cna	gain	TCGA
TCGA-EW-A1OX	SLC45A3	cna	gain	TCGA
TCGA-LL-A5YP	SLC45A3	cna	hetloss	TCGA
MB-0346	SLC45A3	cna	gain	METABRIC
MB-5107	SLC45A3	cna	gain	METABRIC
MB-5465	SLC45A3	cna	gain	METABRIC
MB-6060	SLC45A3	cna	gain	METABRIC
MB-6098	SLC45A3	cna	gain	METABRIC
MB-7032	SLC45A3	cna	gain	METABRIC
MB-7038	SLC45A3	cna	gain	METABRIC
MB-7048	SLC45A3	cna	gain	METABRIC
PD10014	SLC45A3	cna	gain	BASIS
PD13296	SLC45A3	cna	hetloss	BASIS
PD13299	SLC45A3	cna	gain	BASIS
PD14442	SLC45A3	cna	gain	BASIS
PD23562	SLC45A3	cna	gain	BASIS
PD23574	SLC45A3	cna	gain	BASIS
PD24186	SLC45A3	cna	amp	BASIS
PD24206	SLC45A3	cna	gain	BASIS
PD24337	SLC45A3	cna	gain	BASIS
PD3890	SLC45A3	cna	gain	BASIS
PD3905	SLC45A3	cna	gain	BASIS
PD4005	SLC45A3	cna	gain	BASIS
PD4006	SLC45A3	cna	amp	BASIS
PD4107	SLC45A3	cna	gain	BASIS
PD4826	SLC45A3	cna	gain	BASIS
PD5935	SLC45A3	cna	gain	BASIS
PD5945	SLC45A3	cna	amp	BASIS
PD5948	SLC45A3	cna	amp	BASIS
PD6413	SLC45A3	cna	gain	BASIS
PD6731	SLC45A3	cna	hetloss	BASIS
PD7067	SLC45A3	cna	gain	BASIS
PD7215	SLC45A3	cna	amp	BASIS
PD8621	SLC45A3	cna	gain	BASIS
PD8980	SLC45A3	cna	amp	BASIS
PD9004	SLC45A3	cna	gain	BASIS
PD9702	SLC45A3	cna	amp	BASIS
TCGA-A2-A25B	TPM3	cna	gain	TCGA
TCGA-AN-A0XU	TPM3	cna	gain	TCGA
TCGA-AO-A0JL	TPM3	cna	gain	TCGA
TCGA-BH-A0AW	TPM3	cna	gain	TCGA
TCGA-BH-A0C0	TPM3	cna	hetloss	TCGA
TCGA-BH-A18R	TPM3	cna	gain	TCGA
TCGA-BH-A1FU	TPM3	cna	gain	TCGA
TCGA-C8-A12L	TPM3	cna	gain	TCGA
TCGA-E2-A1L7	TPM3	cna	gain	TCGA
TCGA-EW-A1OX	TPM3	cna	gain	TCGA

TCGA-LL-A5YP	TPM3	cna	amp	TCGA
MB-2827	TPM3	cna	amp	METABRIC
MB-5070	TPM3	cna	gain	METABRIC
MB-6060	TPM3	cna	gain	METABRIC
MB-6098	TPM3	cna	amp	METABRIC
MB-7032	TPM3	cna	gain	METABRIC
MB-7048	TPM3	cna	hetloss	METABRIC
PD10014	TPM3	cna	gain	BASIS
PD11327	TPM3	cna	gain	BASIS
PD13297	TPM3	cna	gain	BASIS
PD13299	TPM3	cna	gain	BASIS
PD14442	TPM3	cna	gain	BASIS
PD22355	TPM3	cna	gain	BASIS
PD23562	TPM3	cna	amp	BASIS
PD23574	TPM3	cna	amp	BASIS
PD24186	TPM3	cna	gain	BASIS
PD24206	TPM3	cna	gain	BASIS
PD3890	TPM3	cna	gain	BASIS
PD3905	TPM3	cna	gain	BASIS
PD4005	TPM3	cna	gain	BASIS
PD4006	TPM3	cna	amp	BASIS
PD4107	TPM3	cna	gain	BASIS
PD4826	TPM3	cna	gain	BASIS
PD4967	TPM3	cna	hetloss	BASIS
PD5930	TPM3	cna	amp	BASIS
PD5935	TPM3	cna	gain	BASIS
PD5945	TPM3	cna	amp	BASIS
PD5948	TPM3	cna	gain	BASIS
PD6406	TPM3	cna	gain	BASIS
PD6413	TPM3	cna	gain	BASIS
PD7067	TPM3	cna	amp	BASIS
PD7215	TPM3	cna	gain	BASIS
PD8621	TPM3	cna	amp	BASIS
PD8980	TPM3	cna	gain	BASIS
PD9004	TPM3	cna	amp	BASIS
PD9702	TPM3	cna	amp	BASIS
TCGA-A2-A25B	YY1AP1	cna	gain	TCGA
TCGA-AN-A0XU	YY1AP1	cna	gain	TCGA
TCGA-AO-A0JL	YY1AP1	cna	gain	TCGA
TCGA-BH-A0AW	YY1AP1	cna	gain	TCGA
TCGA-BH-A0C0	YY1AP1	cna	hetloss	TCGA
TCGA-BH-A18R	YY1AP1	cna	gain	TCGA
TCGA-BH-A1FU	YY1AP1	cna	gain	TCGA
TCGA-C8-A12L	YY1AP1	cna	gain	TCGA
TCGA-E2-A1L7	YY1AP1	cna	gain	TCGA
TCGA-EW-A1OX	YY1AP1	cna	gain	TCGA
TCGA-LL-A5YP	YY1AP1	cna	amp	TCGA

MB-2827	YY1AP1	cna	amp	METABRIC
MB-5070	YY1AP1	cna	gain	METABRIC
MB-6060	YY1AP1	cna	gain	METABRIC
MB-6098	YY1AP1	cna	amp	METABRIC
MB-7032	YY1AP1	cna	gain	METABRIC
MB-7038	YY1AP1	cna	gain	METABRIC
PD10014	YY1AP1	cna	gain	BASIS
PD11327	YY1AP1	cna	gain	BASIS
PD13297	YY1AP1	cna	gain	BASIS
PD13299	YY1AP1	cna	gain	BASIS
PD13771	YY1AP1	cna	gain	BASIS
PD14442	YY1AP1	cna	gain	BASIS
PD22355	YY1AP1	cna	gain	BASIS
PD23562	YY1AP1	cna	amp	BASIS
PD23574	YY1AP1	cna	amp	BASIS
PD24186	YY1AP1	cna	gain	BASIS
PD24206	YY1AP1	cna	gain	BASIS
PD3890	YY1AP1	cna	gain	BASIS
PD3905	YY1AP1	cna	gain	BASIS
PD4005	YY1AP1	cna	gain	BASIS
PD4006	YY1AP1	cna	amp	BASIS
PD4107	YY1AP1	cna	gain	BASIS
PD4826	YY1AP1	cna	gain	BASIS
PD5930	YY1AP1	cna	gain	BASIS
PD5935	YY1AP1	cna	gain	BASIS
PD5945	YY1AP1	cna	amp	BASIS
PD5948	YY1AP1	cna	gain	BASIS
PD6406	YY1AP1	cna	gain	BASIS
PD6413	YY1AP1	cna	gain	BASIS
PD7067	YY1AP1	cna	gain	BASIS
PD7215	YY1AP1	cna	gain	BASIS
PD8621	YY1AP1	cna	amp	BASIS
PD8980	YY1AP1	cna	gain	BASIS
PD9004	YY1AP1	cna	gain	BASIS
PD9702	YY1AP1	cna	amp	BASIS
TCGA-A2-A25B	AXIN2	cna	amp	TCGA
TCGA-AN-A0XU	AXIN2	cna	gain	TCGA
TCGA-AO-A0JL	AXIN2	cna	hetloss	TCGA
TCGA-BH-A0AW	AXIN2	cna	amp	TCGA
TCGA-BH-A0C0	AXIN2	cna	gain	TCGA
TCGA-C8-A12L	AXIN2	cna	gain	TCGA
TCGA-D8-A27M	AXIN2	cna	hetloss	TCGA
TCGA-EW-A1OX	AXIN2	cna	amp	TCGA
TCGA-LL-A5YP	AXIN2	cna	amp	TCGA
MB-0346	AXIN2	cna	gain	METABRIC
MB-2827	AXIN2	cna	hetloss	METABRIC
MB-5070	AXIN2	cna	gain	METABRIC

MB-5107	AXIN2	cna	amp	METABRIC
MB-5323	AXIN2	cna	gain	METABRIC
MB-6060	AXIN2	cna	amp	METABRIC
MB-6098	AXIN2	cna	gain	METABRIC
MB-6271	AXIN2	cna	hetloss	METABRIC
MB-7048	AXIN2	cna	hetloss	METABRIC
MTS-T0064	AXIN2	cna	gain	METABRIC
P-0002858-T01-IM3	AXIN2	cna	amp	MSK-IMPACT
PD11327	AXIN2	cna	gain	BASIS
PD11742	AXIN2	cna	gain	BASIS
PD22355	AXIN2	cna	gain	BASIS
PD23562	AXIN2	cna	gain	BASIS
PD23574	AXIN2	cna	gain	BASIS
PD24186	AXIN2	cna	gain	BASIS
PD24202	AXIN2	cna	gain	BASIS
PD24206	AXIN2	cna	hetloss	BASIS
PD24337	AXIN2	cna	hetloss	BASIS
PD3890	AXIN2	cna	gain	BASIS
PD3905	AXIN2	cna	gain	BASIS
PD4006	AXIN2	cna	gain	BASIS
PD4107	AXIN2	cna	gain	BASIS
PD4967	AXIN2	cna	homdel	BASIS
PD5930	AXIN2	cna	gain	BASIS
PD5935	AXIN2	cna	gain	BASIS
PD5945	AXIN2	cna	amp	BASIS
PD5948	AXIN2	cna	gain	BASIS
PD6731	AXIN2	cna	hetloss	BASIS
PD7067	AXIN2	cna	gain	BASIS
PD7215	AXIN2	cna	gain	BASIS
PD8621	AXIN2	cna	gain	BASIS
PD8980	AXIN2	cna	gain	BASIS
PD9004	AXIN2	cna	gain	BASIS
PD9702	AXIN2	cna	gain	BASIS
TCGA-AO-A0JL	BRCA2	cna	hetloss	TCGA
TCGA-BH-A0AW	BRCA2	cna	hetloss	TCGA
TCGA-BH-A0C0	BRCA2	cna	gain	TCGA
TCGA-BH-A1FU	BRCA2	cna	hetloss	TCGA
TCGA-C8-A12L	BRCA2	cna	hetloss	TCGA
TCGA-D8-A27M	BRCA2	cna	hetloss	TCGA
TCGA-E9-A1NC	BRCA2	cna	hetloss	TCGA
TCGA-EW-A1OX	BRCA2	cna	homdel	TCGA
TCGA-LL-A5YP	BRCA2	cna	hetloss	TCGA
MB-0346	BRCA2	cna	hetloss	METABRIC
MB-2827	BRCA2	cna	hetloss	METABRIC
MB-5465	BRCA2	cna	gain	METABRIC
MB-6060	BRCA2	cna	gain	METABRIC
MB-6098	BRCA2	cna	hetloss	METABRIC

MB-7032	BRCA2	cna	hetloss	METABRIC
MB-0420	BRCA2	cna	hetloss	METABRIC
MTS-T0064	BRCA2	cna	hetloss	METABRIC
PD10014	BRCA2	cna	hetloss	BASIS
PD11742	BRCA2	cna	hetloss	BASIS
PD13296	BRCA2	cna	hetloss	BASIS
PD13297	BRCA2	cna	hetloss	BASIS
PD13771	BRCA2	cna	hetloss	BASIS
PD14442	BRCA2	cna	hetloss	BASIS
PD22355	BRCA2	cna	hetloss	BASIS
PD23562	BRCA2	cna	gain	BASIS
PD23578	BRCA2	cna	hetloss	BASIS
PD24202	BRCA2	cna	hetloss	BASIS
PD24206	BRCA2	cna	gain	BASIS
PD3890	BRCA2	cna	hetloss	BASIS
PD3905	BRCA2	cna	gain	BASIS
PD4005	BRCA2	cna	homdel	BASIS
PD4006	BRCA2	cna	hetloss	BASIS
PD4107	BRCA2	cna	gain	BASIS
PD4826	BRCA2	cna	hetloss	BASIS
PD4967	BRCA2	cna	hetloss	BASIS
PD5945	BRCA2	cna	amp	BASIS
PD5948	BRCA2	cna	gain	BASIS
PD6406	BRCA2	cna	hetloss	BASIS
PD6413	BRCA2	cna	hetloss	BASIS
PD6731	BRCA2	cna	hetloss	BASIS
PD7067	BRCA2	cna	gain	BASIS
PD7215	BRCA2	cna	gain	BASIS
PD8980	BRCA2	cna	hetloss	BASIS
PD9585	BRCA2	cna	gain	BASIS
PD9702	BRCA2	cna	gain	BASIS
TCGA-A2-A25B	CD79B	cna	amp	TCGA
TCGA-AN-A0XU	CD79B	cna	gain	TCGA
TCGA-AO-A0JL	CD79B	cna	hetloss	TCGA
TCGA-BH-A0AW	CD79B	cna	amp	TCGA
TCGA-BH-A0C0	CD79B	cna	gain	TCGA
TCGA-C8-A12L	CD79B	cna	gain	TCGA
TCGA-D8-A27M	CD79B	cna	hetloss	TCGA
TCGA-EW-A1OX	CD79B	cna	amp	TCGA
TCGA-LL-A5YP	CD79B	cna	amp	TCGA
MB-0346	CD79B	cna	gain	METABRIC
MB-2827	CD79B	cna	hetloss	METABRIC
MB-5070	CD79B	cna	gain	METABRIC
MB-5107	CD79B	cna	amp	METABRIC
MB-5323	CD79B	cna	gain	METABRIC
MB-6060	CD79B	cna	gain	METABRIC
MB-6098	CD79B	cna	gain	METABRIC

MB-6271	CD79B	cna	hetloss	METABRIC
MB-7048	CD79B	cna	amp	METABRIC
MTS-T0064	CD79B	cna	gain	METABRIC
P-0002858-T01-IM3	CD79B	cna	amp	MSK-IMPACT
PD11327	CD79B	cna	gain	BASIS
PD11742	CD79B	cna	gain	BASIS
PD22355	CD79B	cna	gain	BASIS
PD23561	CD79B	cna	gain	BASIS
PD23562	CD79B	cna	gain	BASIS
PD23574	CD79B	cna	gain	BASIS
PD24186	CD79B	cna	gain	BASIS
PD24206	CD79B	cna	hetloss	BASIS
PD24337	CD79B	cna	hetloss	BASIS
PD3890	CD79B	cna	gain	BASIS
PD3905	CD79B	cna	gain	BASIS
PD4006	CD79B	cna	amp	BASIS
PD4826	CD79B	cna	gain	BASIS
PD4967	CD79B	cna	homdel	BASIS
PD5930	CD79B	cna	gain	BASIS
PD5935	CD79B	cna	gain	BASIS
PD5945	CD79B	cna	amp	BASIS
PD5948	CD79B	cna	gain	BASIS
PD6731	CD79B	cna	hetloss	BASIS
PD7067	CD79B	cna	gain	BASIS
PD7215	CD79B	cna	gain	BASIS
PD8621	CD79B	cna	gain	BASIS
PD8980	CD79B	cna	gain	BASIS
PD9004	CD79B	cna	gain	BASIS
PD9702	CD79B	cna	gain	BASIS
TCGA-A2-A25B	COL1A1	cna	amp	TCGA
TCGA-AN-A0XU	COL1A1	cna	hetloss	TCGA
TCGA-AO-A0JL	COL1A1	cna	hetloss	TCGA
TCGA-BH-A0AW	COL1A1	cna	amp	TCGA
TCGA-BH-A0C0	COL1A1	cna	gain	TCGA
TCGA-BH-A18R	COL1A1	cna	amp	TCGA
TCGA-BH-A1FU	COL1A1	cna	homdel	TCGA
TCGA-C8-A12L	COL1A1	cna	amp	TCGA
TCGA-D8-A27M	COL1A1	cna	hetloss	TCGA
TCGA-E9-A1NC	COL1A1	cna	hetloss	TCGA
TCGA-EW-A1OX	COL1A1	cna	amp	TCGA
MB-0346	COL1A1	cna	gain	METABRIC
MB-2827	COL1A1	cna	hetloss	METABRIC
MB-5070	COL1A1	cna	hetloss	METABRIC
MB-5107	COL1A1	cna	amp	METABRIC
MB-6060	COL1A1	cna	amp	METABRIC
MB-6098	COL1A1	cna	hetloss	METABRIC
MB-6271	COL1A1	cna	hetloss	METABRIC

MB-7048	COL1A1	cna	gain	METABRIC
PD11327	COL1A1	cna	gain	BASIS
PD11742	COL1A1	cna	gain	BASIS
PD13296	COL1A1	cna	gain	BASIS
PD13297	COL1A1	cna	hetloss	BASIS
PD13299	COL1A1	cna	gain	BASIS
PD13771	COL1A1	cna	gain	BASIS
PD23561	COL1A1	cna	amp	BASIS
PD23562	COL1A1	cna	gain	BASIS
PD23574	COL1A1	cna	gain	BASIS
PD24186	COL1A1	cna	gain	BASIS
PD24206	COL1A1	cna	hetloss	BASIS
PD24337	COL1A1	cna	hetloss	BASIS
PD3890	COL1A1	cna	hetloss	BASIS
PD3905	COL1A1	cna	gain	BASIS
PD4005	COL1A1	cna	gain	BASIS
PD4006	COL1A1	cna	amp	BASIS
PD4826	COL1A1	cna	gain	BASIS
PD4967	COL1A1	cna	hetloss	BASIS
PD5945	COL1A1	cna	amp	BASIS
PD5948	COL1A1	cna	gain	BASIS
PD6406	COL1A1	cna	hetloss	BASIS
PD6731	COL1A1	cna	hetloss	BASIS
PD7067	COL1A1	cna	gain	BASIS
PD7215	COL1A1	cna	gain	BASIS
PD9004	COL1A1	cna	gain	BASIS
PD9702	COL1A1	cna	gain	BASIS
TCGA-A2-A25B	ETV4	cna	hetloss	TCGA
TCGA-AN-A0XU	ETV4	cna	hetloss	TCGA
TCGA-AO-A0JL	ETV4	cna	homdel	TCGA
TCGA-BH-A0AW	ETV4	cna	homdel	TCGA
TCGA-BH-A0C0	ETV4	cna	gain	TCGA
TCGA-BH-A18R	ETV4	cna	homdel	TCGA
TCGA-BH-A1FU	ETV4	cna	homdel	TCGA
TCGA-C8-A12L	ETV4	cna	hetloss	TCGA
TCGA-D8-A27M	ETV4	cna	hetloss	TCGA
TCGA-E2-A1L7	ETV4	cna	hetloss	TCGA
TCGA-E9-A1NC	ETV4	cna	hetloss	TCGA
TCGA-EW-A1OX	ETV4	cna	homdel	TCGA
MB-0346	ETV4	cna	hetloss	METABRIC
MB-2827	ETV4	cna	hetloss	METABRIC
MB-5070	ETV4	cna	hetloss	METABRIC
MB-5465	ETV4	cna	hetloss	METABRIC
MB-6060	ETV4	cna	hetloss	METABRIC
MB-6098	ETV4	cna	hetloss	METABRIC
MB-6271	ETV4	cna	hetloss	METABRIC
MB-7038	ETV4	cna	hetloss	METABRIC

MB-7048	ETV4	cna	hetloss	METABRIC
PD13296	ETV4	cna	gain	BASIS
PD13297	ETV4	cna	hetloss	BASIS
PD13299	ETV4	cna	gain	BASIS
PD13771	ETV4	cna	hetloss	BASIS
PD23561	ETV4	cna	hetloss	BASIS
PD23562	ETV4	cna	gain	BASIS
PD23574	ETV4	cna	gain	BASIS
PD24186	ETV4	cna	gain	BASIS
PD24206	ETV4	cna	hetloss	BASIS
PD24337	ETV4	cna	hetloss	BASIS
PD3890	ETV4	cna	hetloss	BASIS
PD3905	ETV4	cna	gain	BASIS
PD4006	ETV4	cna	gain	BASIS
PD4826	ETV4	cna	hetloss	BASIS
PD4967	ETV4	cna	homdel	BASIS
PD5945	ETV4	cna	amp	BASIS
PD5948	ETV4	cna	gain	BASIS
PD6406	ETV4	cna	hetloss	BASIS
PD6731	ETV4	cna	hetloss	BASIS
PD7067	ETV4	cna	gain	BASIS
PD7215	ETV4	cna	gain	BASIS
PD9004	ETV4	cna	gain	BASIS
PD9585	ETV4	cna	hetloss	BASIS
PD9702	ETV4	cna	amp	BASIS
TCGA-A2-A25B	ETV6	cna	hetloss	TCGA
TCGA-AN-A0XU	ETV6	cna	hetloss	TCGA
TCGA-AO-A0JL	ETV6	cna	hetloss	TCGA
TCGA-BH-A0C0	ETV6	cna	hetloss	TCGA
TCGA-BH-A18R	ETV6	cna	gain	TCGA
TCGA-BH-A1FU	ETV6	cna	hetloss	TCGA
TCGA-C8-A12L	ETV6	cna	hetloss	TCGA
TCGA-D8-A27M	ETV6	cna	amp	TCGA
TCGA-LL-A5YP	ETV6	cna	hetloss	TCGA
MB-0346	ETV6	cna	hetloss	METABRIC
MB-5070	ETV6	cna	amp	METABRIC
MB-5107	ETV6	cna	hetloss	METABRIC
MB-6060	ETV6	cna	gain	METABRIC
MB-6098	ETV6	cna	gain	METABRIC
MB-7038	ETV6	cna	hetloss	METABRIC
MB-7048	ETV6	cna	gain	METABRIC
P-0009557-T01-IM5	ETV6	cna	homdel	MSK-IMPACT
P-0011369-T01-IM5	ETV6	cna	homdel	MSK-IMPACT
PD10014	ETV6	cna	hetloss	BASIS
PD11327	ETV6	cna	gain	BASIS
PD11742	ETV6	cna	gain	BASIS
PD13296	ETV6	cna	hetloss	BASIS

PD13297	ETV6	cna	hetloss	BASIS
PD13299	ETV6	cna	gain	BASIS
PD13771	ETV6	cna	hetloss	BASIS
PD23574	ETV6	cna	gain	BASIS
PD23578	ETV6	cna	gain	BASIS
PD24186	ETV6	cna	gain	BASIS
PD24202	ETV6	cna	hetloss	BASIS
PD3905	ETV6	cna	gain	BASIS
PD4005	ETV6	cna	hetloss	BASIS
PD4006	ETV6	cna	gain	BASIS
PD4107	ETV6	cna	gain	BASIS
PD4826	ETV6	cna	hetloss	BASIS
PD4967	ETV6	cna	hetloss	BASIS
PD5930	ETV6	cna	gain	BASIS
PD5935	ETV6	cna	gain	BASIS
PD5945	ETV6	cna	amp	BASIS
PD6406	ETV6	cna	amp	BASIS
PD6731	ETV6	cna	hetloss	BASIS
PD7215	ETV6	cna	gain	BASIS
PD8621	ETV6	cna	gain	BASIS
PD8980	ETV6	cna	hetloss	BASIS
PD9004	ETV6	cna	gain	BASIS
PD9702	ETV6	cna	gain	BASIS
TCGA-A2-A25B	GNAS	cna	gain	TCGA
TCGA-AN-A0XU	GNAS	cna	gain	TCGA
TCGA-AO-A0JL	GNAS	cna	gain	TCGA
TCGA-BH-A0AW	GNAS	cna	gain	TCGA
TCGA-BH-A0C0	GNAS	cna	gain	TCGA
TCGA-BH-A18R	GNAS	cna	gain	TCGA
TCGA-C8-A12L	GNAS	cna	gain	TCGA
TCGA-D8-A27M	GNAS	cna	hetloss	TCGA
TCGA-E2-A1L7	GNAS	cna	gain	TCGA
TCGA-E9-A1NC	GNAS	cna	gain	TCGA
TCGA-LL-A5YP	GNAS	cna	gain	TCGA
MB-0346	GNAS	cna	gain	METABRIC
MB-2827	GNAS	cna	amp	METABRIC
MB-5070	GNAS	cna	hetloss	METABRIC
MB-5107	GNAS	cna	gain	METABRIC
MB-6060	GNAS	cna	gain	METABRIC
MB-6098	GNAS	cna	hetloss	METABRIC
P-0002858-T01-IM3	GNAS	cna	amp	MSK-IMPACT
PD11327	GNAS	cna	gain	BASIS
PD13296	GNAS	cna	gain	BASIS
PD13297	GNAS	cna	hetloss	BASIS
PD13299	GNAS	cna	gain	BASIS
PD13771	GNAS	cna	gain	BASIS
PD22355	GNAS	cna	gain	BASIS

PD23562	GNAS	cna	gain	BASIS
PD23574	GNAS	cna	gain	BASIS
PD23578	GNAS	cna	hetloss	BASIS
PD24186	GNAS	cna	gain	BASIS
PD24202	GNAS	cna	hetloss	BASIS
PD24206	GNAS	cna	amp	BASIS
PD3905	GNAS	cna	gain	BASIS
PD4005	GNAS	cna	hetloss	BASIS
PD4006	GNAS	cna	gain	BASIS
PD4826	GNAS	cna	gain	BASIS
PD5935	GNAS	cna	gain	BASIS
PD5945	GNAS	cna	amp	BASIS
PD5948	GNAS	cna	gain	BASIS
PD6406	GNAS	cna	gain	BASIS
PD7067	GNAS	cna	gain	BASIS
PD7215	GNAS	cna	amp	BASIS
PD8621	GNAS	cna	gain	BASIS
PD8980	GNAS	cna	gain	BASIS
PD9004	GNAS	cna	gain	BASIS
PD9585	GNAS	cna	hetloss	BASIS
PD9702	GNAS	cna	gain	BASIS
TCGA-A2-A25B	HEY1	cna	gain	TCGA
TCGA-AN-A0XU	HEY1	cna	hetloss	TCGA
TCGA-AO-A0JL	HEY1	cna	amp	TCGA
TCGA-BH-A0AW	HEY1	cna	gain	TCGA
TCGA-BH-A0C0	HEY1	cna	amp	TCGA
TCGA-BH-A18R	HEY1	cna	homdel	TCGA
TCGA-BH-A1FU	HEY1	cna	gain	TCGA
TCGA-C8-A12L	HEY1	cna	hetloss	TCGA
TCGA-D8-A27M	HEY1	cna	amp	TCGA
TCGA-E2-A1L7	HEY1	cna	gain	TCGA
TCGA-LL-A5YP	HEY1	cna	amp	TCGA
MB-0346	HEY1	cna	gain	METABRIC
MB-2827	HEY1	cna	amp	METABRIC
MB-5107	HEY1	cna	gain	METABRIC
MB-5465	HEY1	cna	hetloss	METABRIC
MB-6060	HEY1	cna	amp	METABRIC
MB-6098	HEY1	cna	amp	METABRIC
MB-7038	HEY1	cna	gain	METABRIC
MB-7048	HEY1	cna	amp	METABRIC
MB-0420	HEY1	cna	gain	METABRIC
PD10014	HEY1	cna	amp	BASIS
PD11327	HEY1	cna	amp	BASIS
PD13296	HEY1	cna	hetloss	BASIS
PD13299	HEY1	cna	gain	BASIS
PD13771	HEY1	cna	gain	BASIS
PD23562	HEY1	cna	gain	BASIS

PD23574	HEY1	cna	gain	BASIS
PD24186	HEY1	cna	gain	BASIS
PD24206	HEY1	cna	amp	BASIS
PD3890	HEY1	cna	gain	BASIS
PD3905	HEY1	cna	gain	BASIS
PD4005	HEY1	cna	hetloss	BASIS
PD4006	HEY1	cna	gain	BASIS
PD4826	HEY1	cna	amp	BASIS
PD5935	HEY1	cna	gain	BASIS
PD5945	HEY1	cna	gain	BASIS
PD5948	HEY1	cna	amp	BASIS
PD6406	HEY1	cna	gain	BASIS
PD6413	HEY1	cna	gain	BASIS
PD7067	HEY1	cna	amp	BASIS
PD7215	HEY1	cna	amp	BASIS
PD8621	HEY1	cna	gain	BASIS
PD8980	HEY1	cna	gain	BASIS
PD9004	HEY1	cna	gain	BASIS
PD9702	HEY1	cna	gain	BASIS
TCGA-A2-A25B	HLF	cna	amp	TCGA
TCGA-AN-A0XU	HLF	cna	hetloss	TCGA
TCGA-AO-A0JL	HLF	cna	hetloss	TCGA
TCGA-BH-A0AW	HLF	cna	amp	TCGA
TCGA-BH-A0C0	HLF	cna	gain	TCGA
TCGA-BH-A18R	HLF	cna	amp	TCGA
TCGA-C8-A12L	HLF	cna	gain	TCGA
TCGA-D8-A27M	HLF	cna	gain	TCGA
TCGA-EW-A1OX	HLF	cna	amp	TCGA
MB-0346	HLF	cna	gain	METABRIC
MB-2827	HLF	cna	hetloss	METABRIC
MB-5070	HLF	cna	hetloss	METABRIC
MB-5107	HLF	cna	amp	METABRIC
MB-5323	HLF	cna	gain	METABRIC
MB-6060	HLF	cna	hetloss	METABRIC
MB-6098	HLF	cna	hetloss	METABRIC
MB-6271	HLF	cna	hetloss	METABRIC
MB-7048	HLF	cna	hetloss	METABRIC
PD11327	HLF	cna	amp	BASIS
PD11742	HLF	cna	gain	BASIS
PD13296	HLF	cna	gain	BASIS
PD13297	HLF	cna	hetloss	BASIS
PD13299	HLF	cna	gain	BASIS
PD22355	HLF	cna	gain	BASIS
PD23561	HLF	cna	gain	BASIS
PD23562	HLF	cna	gain	BASIS
PD23574	HLF	cna	gain	BASIS
PD24186	HLF	cna	gain	BASIS

PD24337	HLF	cna	hetloss	BASIS
PD3890	HLF	cna	hetloss	BASIS
PD3905	HLF	cna	gain	BASIS
PD4006	HLF	cna	gain	BASIS
PD4107	HLF	cna	hetloss	BASIS
PD4826	HLF	cna	gain	BASIS
PD4967	HLF	cna	hetloss	BASIS
PD5930	HLF	cna	gain	BASIS
PD5945	HLF	cna	amp	BASIS
PD5948	HLF	cna	gain	BASIS
PD6406	HLF	cna	hetloss	BASIS
PD6731	HLF	cna	hetloss	BASIS
PD7067	HLF	cna	gain	BASIS
PD7215	HLF	cna	gain	BASIS
PD8980	HLF	cna	gain	BASIS
PD9004	HLF	cna	gain	BASIS
PD9702	HLF	cna	gain	BASIS
TCGA-A2-A25B	IKBKE	cna	amp	TCGA
TCGA-AN-A0XU	IKBKE	cna	hetloss	TCGA
TCGA-AO-A0JL	IKBKE	cna	gain	TCGA
TCGA-BH-A0AW	IKBKE	cna	gain	TCGA
TCGA-BH-A0C0	IKBKE	cna	gain	TCGA
TCGA-BH-A18R	IKBKE	cna	amp	TCGA
TCGA-BH-A1FU	IKBKE	cna	gain	TCGA
TCGA-C8-A12L	IKBKE	cna	gain	TCGA
TCGA-E2-A1L7	IKBKE	cna	gain	TCGA
TCGA-E9-A1NC	IKBKE	cna	gain	TCGA
TCGA-EW-A1OX	IKBKE	cna	gain	TCGA
TCGA-LL-A5YP	IKBKE	cna	hetloss	TCGA
MB-0346	IKBKE	cna	gain	METABRIC
MB-5107	IKBKE	cna	gain	METABRIC
MB-5465	IKBKE	cna	gain	METABRIC
MB-6098	IKBKE	cna	gain	METABRIC
MB-7032	IKBKE	cna	gain	METABRIC
MB-7048	IKBKE	cna	gain	METABRIC
MB-0420	IKBKE	cna	gain	METABRIC
PD10014	IKBKE	cna	gain	BASIS
PD11742	IKBKE	cna	gain	BASIS
PD13296	IKBKE	cna	hetloss	BASIS
PD13299	IKBKE	cna	gain	BASIS
PD14442	IKBKE	cna	gain	BASIS
PD23562	IKBKE	cna	gain	BASIS
PD23574	IKBKE	cna	gain	BASIS
PD24186	IKBKE	cna	amp	BASIS
PD24206	IKBKE	cna	gain	BASIS
PD24337	IKBKE	cna	gain	BASIS
PD3890	IKBKE	cna	gain	BASIS

PD3905	IKBKE	cna	gain	BASIS
PD4005	IKBKE	cna	gain	BASIS
PD4006	IKBKE	cna	amp	BASIS
PD4107	IKBKE	cna	gain	BASIS
PD4826	IKBKE	cna	gain	BASIS
PD5935	IKBKE	cna	gain	BASIS
PD5945	IKBKE	cna	amp	BASIS
PD5948	IKBKE	cna	amp	BASIS
PD6413	IKBKE	cna	gain	BASIS
PD7067	IKBKE	cna	gain	BASIS
PD7215	IKBKE	cna	amp	BASIS
PD8621	IKBKE	cna	gain	BASIS
PD8980	IKBKE	cna	amp	BASIS
PD9004	IKBKE	cna	gain	BASIS
PD9702	IKBKE	cna	amp	BASIS
TCGA-A2-A25B	IL10	cna	amp	TCGA
TCGA-AN-A0XU	IL10	cna	hetloss	TCGA
TCGA-AO-A0JL	IL10	cna	gain	TCGA
TCGA-BH-A0AW	IL10	cna	gain	TCGA
TCGA-BH-A0C0	IL10	cna	gain	TCGA
TCGA-BH-A18R	IL10	cna	amp	TCGA
TCGA-BH-A1FU	IL10	cna	gain	TCGA
TCGA-C8-A12L	IL10	cna	hetloss	TCGA
TCGA-E2-A1L7	IL10	cna	gain	TCGA
TCGA-E9-A1NC	IL10	cna	gain	TCGA
TCGA-EW-A1OX	IL10	cna	gain	TCGA
TCGA-LL-A5YP	IL10	cna	hetloss	TCGA
MB-0346	IL10	cna	gain	METABRIC
MB-5107	IL10	cna	gain	METABRIC
MB-5465	IL10	cna	gain	METABRIC
MB-6098	IL10	cna	gain	METABRIC
MB-7032	IL10	cna	gain	METABRIC
MB-7048	IL10	cna	gain	METABRIC
MB-0420	IL10	cna	gain	METABRIC
PD10014	IL10	cna	gain	BASIS
PD11742	IL10	cna	gain	BASIS
PD13296	IL10	cna	hetloss	BASIS
PD13299	IL10	cna	gain	BASIS
PD14442	IL10	cna	gain	BASIS
PD23562	IL10	cna	gain	BASIS
PD23574	IL10	cna	gain	BASIS
PD24186	IL10	cna	amp	BASIS
PD24206	IL10	cna	gain	BASIS
PD24337	IL10	cna	gain	BASIS
PD3890	IL10	cna	gain	BASIS
PD3905	IL10	cna	gain	BASIS
PD4005	IL10	cna	gain	BASIS

PD4006	IL10	cna	amp	BASIS
PD4107	IL10	cna	gain	BASIS
PD4826	IL10	cna	gain	BASIS
PD5935	IL10	cna	gain	BASIS
PD5945	IL10	cna	amp	BASIS
PD5948	IL10	cna	amp	BASIS
PD6413	IL10	cna	gain	BASIS
PD7067	IL10	cna	gain	BASIS
PD7215	IL10	cna	amp	BASIS
PD8621	IL10	cna	gain	BASIS
PD8980	IL10	cna	amp	BASIS
PD9004	IL10	cna	gain	BASIS
PD9702	IL10	cna	amp	BASIS
TCGA-A2-A25B	MSH3	cna	hetloss	TCGA
TCGA-AN-A0XU	MSH3	cna	hetloss	TCGA
TCGA-AO-A0JL	MSH3	cna	hetloss	TCGA
TCGA-BH-A0C0	MSH3	cna	hetloss	TCGA
TCGA-BH-A1FU	MSH3	cna	hetloss	TCGA
TCGA-C8-A12L	MSH3	cna	hetloss	TCGA
TCGA-D8-A27M	MSH3	cna	hetloss	TCGA
TCGA-E2-A1L7	MSH3	cna	hetloss	TCGA
TCGA-E9-A1NC	MSH3	cna	hetloss	TCGA
TCGA-LL-A5YP	MSH3	cna	hetloss	TCGA
MB-0346	MSH3	cna	gain	METABRIC
MB-2827	MSH3	cna	hetloss	METABRIC
MB-5070	MSH3	cna	hetloss	METABRIC
MB-5107	MSH3	cna	hetloss	METABRIC
MB-5465	MSH3	cna	hetloss	METABRIC
MB-6098	MSH3	cna	hetloss	METABRIC
MB-0420	MSH3	cna	hetloss	METABRIC
MTS-T0064	MSH3	cna	hetloss	METABRIC
PD10014	MSH3	cna	hetloss	BASIS
PD11327	MSH3	cna	hetloss	BASIS
PD11742	MSH3	cna	hetloss	BASIS
PD13296	MSH3	cna	hetloss	BASIS
PD13297	MSH3	cna	hetloss	BASIS
PD13299	MSH3	cna	hetloss	BASIS
PD13771	MSH3	cna	gain	BASIS
PD22355	MSH3	cna	hetloss	BASIS
PD23562	MSH3	cna	hetloss	BASIS
PD24202	MSH3	cna	hetloss	BASIS
PD24206	MSH3	cna	gain	BASIS
PD24337	MSH3	cna	hetloss	BASIS
PD3890	MSH3	cna	hetloss	BASIS
PD3905	MSH3	cna	gain	BASIS
PD4005	MSH3	cna	hetloss	BASIS
PD4107	MSH3	cna	hetloss	BASIS

PD4967	MSH3	cna	hetloss	BASIS
PD5930	MSH3	cna	hetloss	BASIS
PD5935	MSH3	cna	hetloss	BASIS
PD5948	MSH3	cna	hetloss	BASIS
PD6406	MSH3	cna	hetloss	BASIS
PD6413	MSH3	cna	hetloss	BASIS
PD6731	MSH3	cna	hetloss	BASIS
PD7067	MSH3	cna	gain	BASIS
PD7215	MSH3	cna	hetloss	BASIS
PD8621	MSH3	cna	hetloss	BASIS
PD9585	MSH3	cna	hetloss	BASIS
TCGA-A2-A25B	MUC1	cna	gain	TCGA
TCGA-AN-A0XU	MUC1	cna	gain	TCGA
TCGA-AO-A0JL	MUC1	cna	gain	TCGA
TCGA-BH-A0AW	MUC1	cna	gain	TCGA
TCGA-BH-A0C0	MUC1	cna	hetloss	TCGA
TCGA-BH-A18R	MUC1	cna	gain	TCGA
TCGA-BH-A1FU	MUC1	cna	gain	TCGA
TCGA-C8-A12L	MUC1	cna	gain	TCGA
TCGA-E2-A1L7	MUC1	cna	gain	TCGA
TCGA-EW-A1OX	MUC1	cna	gain	TCGA
TCGA-LL-A5YP	MUC1	cna	amp	TCGA
MB-2827	MUC1	cna	amp	METABRIC
MB-5070	MUC1	cna	gain	METABRIC
MB-6060	MUC1	cna	gain	METABRIC
MB-6098	MUC1	cna	amp	METABRIC
MB-7032	MUC1	cna	gain	METABRIC
PD10014	MUC1	cna	gain	BASIS
PD11327	MUC1	cna	gain	BASIS
PD13297	MUC1	cna	gain	BASIS
PD13299	MUC1	cna	gain	BASIS
PD13771	MUC1	cna	gain	BASIS
PD14442	MUC1	cna	gain	BASIS
PD22355	MUC1	cna	gain	BASIS
PD23562	MUC1	cna	amp	BASIS
PD23574	MUC1	cna	amp	BASIS
PD24186	MUC1	cna	gain	BASIS
PD24206	MUC1	cna	gain	BASIS
PD3890	MUC1	cna	gain	BASIS
PD3905	MUC1	cna	gain	BASIS
PD4005	MUC1	cna	gain	BASIS
PD4006	MUC1	cna	amp	BASIS
PD4107	MUC1	cna	gain	BASIS
PD4826	MUC1	cna	gain	BASIS
PD5930	MUC1	cna	amp	BASIS
PD5935	MUC1	cna	gain	BASIS
PD5945	MUC1	cna	amp	BASIS

PD5948	MUC1	cna	gain	BASIS
PD6406	MUC1	cna	gain	BASIS
PD6413	MUC1	cna	gain	BASIS
PD7067	MUC1	cna	amp	BASIS
PD7215	MUC1	cna	gain	BASIS
PD8621	MUC1	cna	amp	BASIS
PD8980	MUC1	cna	gain	BASIS
PD9004	MUC1	cna	amp	BASIS
PD9702	MUC1	cna	amp	BASIS
TCGA-A2-A25B	PAG1	cna	gain	TCGA
TCGA-AN-A0XU	PAG1	cna	hetloss	TCGA
TCGA-AO-A0JL	PAG1	cna	amp	TCGA
TCGA-BH-A0AW	PAG1	cna	gain	TCGA
TCGA-BH-A0C0	PAG1	cna	amp	TCGA
TCGA-BH-A18R	PAG1	cna	homdel	TCGA
TCGA-BH-A1FU	PAG1	cna	gain	TCGA
TCGA-C8-A12L	PAG1	cna	hetloss	TCGA
TCGA-D8-A27M	PAG1	cna	amp	TCGA
TCGA-E2-A1L7	PAG1	cna	gain	TCGA
TCGA-LL-A5YP	PAG1	cna	amp	TCGA
MB-0346	PAG1	cna	gain	METABRIC
MB-2827	PAG1	cna	amp	METABRIC
MB-5107	PAG1	cna	gain	METABRIC
MB-5465	PAG1	cna	hetloss	METABRIC
MB-6098	PAG1	cna	amp	METABRIC
MB-7038	PAG1	cna	gain	METABRIC
MB-7048	PAG1	cna	gain	METABRIC
MB-0420	PAG1	cna	gain	METABRIC
PD10014	PAG1	cna	amp	BASIS
PD11327	PAG1	cna	amp	BASIS
PD13296	PAG1	cna	hetloss	BASIS
PD13299	PAG1	cna	gain	BASIS
PD13771	PAG1	cna	gain	BASIS
PD23562	PAG1	cna	amp	BASIS
PD23574	PAG1	cna	gain	BASIS
PD24186	PAG1	cna	gain	BASIS
PD24206	PAG1	cna	amp	BASIS
PD3890	PAG1	cna	gain	BASIS
PD3905	PAG1	cna	gain	BASIS
PD4005	PAG1	cna	hetloss	BASIS
PD4006	PAG1	cna	gain	BASIS
PD4826	PAG1	cna	amp	BASIS
PD5935	PAG1	cna	gain	BASIS
PD5945	PAG1	cna	gain	BASIS
PD5948	PAG1	cna	amp	BASIS
PD6406	PAG1	cna	gain	BASIS
PD6413	PAG1	cna	gain	BASIS

PD7067	PAG1	cna	amp	BASIS
PD7215	PAG1	cna	amp	BASIS
PD8621	PAG1	cna	gain	BASIS
PD8980	PAG1	cna	gain	BASIS
PD9004	PAG1	cna	gain	BASIS
PD9585	PAG1	cna	gain	BASIS
PD9702	PAG1	cna	gain	BASIS
TCGA-A2-A25B	PRKCI	cna	gain	TCGA
TCGA-AN-A0XU	PRKCI	cna	gain	TCGA
TCGA-BH-A0AW	PRKCI	cna	gain	TCGA
TCGA-BH-A0C0	PRKCI	cna	gain	TCGA
TCGA-BH-A18R	PRKCI	cna	amp	TCGA
TCGA-BH-A1FU	PRKCI	cna	hetloss	TCGA
TCGA-C8-A12L	PRKCI	cna	gain	TCGA
TCGA-E2-A1L7	PRKCI	cna	gain	TCGA
TCGA-LL-A5YP	PRKCI	cna	gain	TCGA
MB-0346	PRKCI	cna	gain	METABRIC
MB-2827	PRKCI	cna	hetloss	METABRIC
MB-5070	PRKCI	cna	amp	METABRIC
MB-5107	PRKCI	cna	amp	METABRIC
MB-6060	PRKCI	cna	gain	METABRIC
MB-7038	PRKCI	cna	hetloss	METABRIC
MB-7048	PRKCI	cna	hetloss	METABRIC
MB-0420	PRKCI	cna	amp	METABRIC
MTS-T0064	PRKCI	cna	amp	METABRIC
PD11327	PRKCI	cna	amp	BASIS
PD13296	PRKCI	cna	amp	BASIS
PD13299	PRKCI	cna	gain	BASIS
PD13771	PRKCI	cna	hetloss	BASIS
PD22355	PRKCI	cna	gain	BASIS
PD23561	PRKCI	cna	hetloss	BASIS
PD23562	PRKCI	cna	amp	BASIS
PD23574	PRKCI	cna	gain	BASIS
PD23578	PRKCI	cna	gain	BASIS
PD24186	PRKCI	cna	amp	BASIS
PD24202	PRKCI	cna	gain	BASIS
PD24206	PRKCI	cna	gain	BASIS
PD24337	PRKCI	cna	gain	BASIS
PD3905	PRKCI	cna	gain	BASIS
PD4005	PRKCI	cna	gain	BASIS
PD4107	PRKCI	cna	gain	BASIS
PD4826	PRKCI	cna	gain	BASIS
PD5930	PRKCI	cna	gain	BASIS
PD5935	PRKCI	cna	amp	BASIS
PD5945	PRKCI	cna	amp	BASIS
PD5948	PRKCI	cna	amp	BASIS
PD6406	PRKCI	cna	gain	BASIS

PD7067	PRKCI	cna	gain	BASIS
PD7215	PRKCI	cna	gain	BASIS
PD8621	PRKCI	cna	gain	BASIS
PD8980	PRKCI	cna	gain	BASIS
PD9702	PRKCI	cna	amp	BASIS
TCGA-A2-A25B	PRRX1	cna	gain	TCGA
TCGA-AN-A0XU	PRRX1	cna	gain	TCGA
TCGA-AO-A0JL	PRRX1	cna	gain	TCGA
TCGA-BH-A0AW	PRRX1	cna	gain	TCGA
TCGA-BH-A0C0	PRRX1	cna	gain	TCGA
TCGA-BH-A18R	PRRX1	cna	gain	TCGA
TCGA-BH-A1FU	PRRX1	cna	gain	TCGA
TCGA-C8-A12L	PRRX1	cna	gain	TCGA
TCGA-E2-A1L7	PRRX1	cna	gain	TCGA
TCGA-EW-A1OX	PRRX1	cna	gain	TCGA
TCGA-LL-A5YP	PRRX1	cna	amp	TCGA
MB-0346	PRRX1	cna	amp	METABRIC
MB-5070	PRRX1	cna	gain	METABRIC
MB-5465	PRRX1	cna	amp	METABRIC
MB-6098	PRRX1	cna	amp	METABRIC
MB-7032	PRRX1	cna	gain	METABRIC
MB-7038	PRRX1	cna	gain	METABRIC
MB-0420	PRRX1	cna	gain	METABRIC
PD10014	PRRX1	cna	gain	BASIS
PD11327	PRRX1	cna	gain	BASIS
PD13297	PRRX1	cna	gain	BASIS
PD13299	PRRX1	cna	gain	BASIS
PD13771	PRRX1	cna	hetloss	BASIS
PD14442	PRRX1	cna	gain	BASIS
PD22355	PRRX1	cna	gain	BASIS
PD23562	PRRX1	cna	gain	BASIS
PD23574	PRRX1	cna	gain	BASIS
PD24186	PRRX1	cna	amp	BASIS
PD24206	PRRX1	cna	gain	BASIS
PD24337	PRRX1	cna	hetloss	BASIS
PD3905	PRRX1	cna	gain	BASIS
PD4005	PRRX1	cna	gain	BASIS
PD4006	PRRX1	cna	gain	BASIS
PD4107	PRRX1	cna	gain	BASIS
PD4826	PRRX1	cna	gain	BASIS
PD5935	PRRX1	cna	gain	BASIS
PD5945	PRRX1	cna	amp	BASIS
PD5948	PRRX1	cna	gain	BASIS
PD6413	PRRX1	cna	gain	BASIS
PD7067	PRRX1	cna	gain	BASIS
PD7215	PRRX1	cna	gain	BASIS
PD8621	PRRX1	cna	gain	BASIS

PD8980	PRRX1	cna	gain	BASIS
PD9004	PRRX1	cna	gain	BASIS
PD9702	PRRX1	cna	gain	BASIS
TCGA-A2-A25B	SOCS3	cna	gain	TCGA
TCGA-AN-A0XU	SOCS3	cna	amp	TCGA
TCGA-AO-A0JL	SOCS3	cna	hetloss	TCGA
TCGA-BH-A0AW	SOCS3	cna	amp	TCGA
TCGA-BH-A0C0	SOCS3	cna	gain	TCGA
TCGA-BH-A18R	SOCS3	cna	amp	TCGA
TCGA-C8-A12L	SOCS3	cna	gain	TCGA
TCGA-D8-A27M	SOCS3	cna	gain	TCGA
TCGA-E2-A1L7	SOCS3	cna	gain	TCGA
TCGA-EW-A1OX	SOCS3	cna	amp	TCGA
TCGA-LL-A5YP	SOCS3	cna	amp	TCGA
MB-2827	SOCS3	cna	hetloss	METABRIC
MB-5070	SOCS3	cna	amp	METABRIC
MB-5107	SOCS3	cna	amp	METABRIC
MB-6098	SOCS3	cna	gain	METABRIC
MB-6271	SOCS3	cna	hetloss	METABRIC
MB-7048	SOCS3	cna	gain	METABRIC
MTS-T0064	SOCS3	cna	gain	METABRIC
PD10014	SOCS3	cna	gain	BASIS
PD11327	SOCS3	cna	amp	BASIS
PD22355	SOCS3	cna	hetloss	BASIS
PD23561	SOCS3	cna	gain	BASIS
PD23574	SOCS3	cna	amp	BASIS
PD24186	SOCS3	cna	amp	BASIS
PD24202	SOCS3	cna	gain	BASIS
PD24206	SOCS3	cna	hetloss	BASIS
PD24337	SOCS3	cna	gain	BASIS
PD3890	SOCS3	cna	gain	BASIS
PD3905	SOCS3	cna	gain	BASIS
PD4006	SOCS3	cna	gain	BASIS
PD4107	SOCS3	cna	gain	BASIS
PD4826	SOCS3	cna	amp	BASIS
PD4967	SOCS3	cna	hetloss	BASIS
PD5930	SOCS3	cna	gain	BASIS
PD5935	SOCS3	cna	gain	BASIS
PD5945	SOCS3	cna	amp	BASIS
PD5948	SOCS3	cna	gain	BASIS
PD6413	SOCS3	cna	hetloss	BASIS
PD6731	SOCS3	cna	hetloss	BASIS
PD7067	SOCS3	cna	gain	BASIS
PD7215	SOCS3	cna	gain	BASIS
PD8621	SOCS3	cna	gain	BASIS
PD9004	SOCS3	cna	gain	BASIS
PD9585	SOCS3	cna	gain	BASIS

PD9702	SOCS3	cna	gain	BASIS
TCGA-A2-A25B	TRAF5	cna	amp	TCGA
TCGA-AN-A0XU	TRAF5	cna	hetloss	TCGA
TCGA-AO-A0JL	TRAF5	cna	gain	TCGA
TCGA-BH-A0AW	TRAF5	cna	gain	TCGA
TCGA-BH-A0C0	TRAF5	cna	gain	TCGA
TCGA-BH-A18R	TRAF5	cna	amp	TCGA
TCGA-BH-A1FU	TRAF5	cna	gain	TCGA
TCGA-E2-A1L7	TRAF5	cna	gain	TCGA
TCGA-E9-A1NC	TRAF5	cna	gain	TCGA
TCGA-EW-A1OX	TRAF5	cna	gain	TCGA
TCGA-LL-A5YP	TRAF5	cna	hetloss	TCGA
MB-0346	TRAF5	cna	gain	METABRIC
MB-5107	TRAF5	cna	gain	METABRIC
MB-5465	TRAF5	cna	gain	METABRIC
MB-6098	TRAF5	cna	gain	METABRIC
MB-7032	TRAF5	cna	gain	METABRIC
MB-7048	TRAF5	cna	gain	METABRIC
MB-0420	TRAF5	cna	hetloss	METABRIC
PD10014	TRAF5	cna	gain	BASIS
PD11327	TRAF5	cna	homdel	BASIS
PD11742	TRAF5	cna	gain	BASIS
PD13296	TRAF5	cna	hetloss	BASIS
PD13299	TRAF5	cna	gain	BASIS
PD14442	TRAF5	cna	gain	BASIS
PD23562	TRAF5	cna	gain	BASIS
PD23574	TRAF5	cna	gain	BASIS
PD24186	TRAF5	cna	amp	BASIS
PD24206	TRAF5	cna	gain	BASIS
PD24337	TRAF5	cna	gain	BASIS
PD3890	TRAF5	cna	gain	BASIS
PD3905	TRAF5	cna	gain	BASIS
PD4005	TRAF5	cna	gain	BASIS
PD4006	TRAF5	cna	gain	BASIS
PD4107	TRAF5	cna	gain	BASIS
PD4826	TRAF5	cna	gain	BASIS
PD5935	TRAF5	cna	gain	BASIS
PD5945	TRAF5	cna	amp	BASIS
PD5948	TRAF5	cna	amp	BASIS
PD6413	TRAF5	cna	gain	BASIS
PD7067	TRAF5	cna	gain	BASIS
PD7215	TRAF5	cna	amp	BASIS
PD8621	TRAF5	cna	gain	BASIS
PD8980	TRAF5	cna	amp	BASIS
PD9004	TRAF5	cna	gain	BASIS
PD9702	TRAF5	cna	amp	BASIS
TCGA-A2-A25B	ARFRP1	cna	gain	TCGA

TCGA-AN-A0XU	ARFRP1	cna	gain	TCGA
TCGA-AO-A0JL	ARFRP1	cna	gain	TCGA
TCGA-BH-A0AW	ARFRP1	cna	gain	TCGA
TCGA-BH-A18R	ARFRP1	cna	gain	TCGA
TCGA-C8-A12L	ARFRP1	cna	gain	TCGA
TCGA-D8-A27M	ARFRP1	cna	hetloss	TCGA
TCGA-E2-A1L7	ARFRP1	cna	gain	TCGA
TCGA-E9-A1NC	ARFRP1	cna	gain	TCGA
TCGA-LL-A5YP	ARFRP1	cna	gain	TCGA
MB-0346	ARFRP1	cna	gain	METABRIC
MB-2827	ARFRP1	cna	hetloss	METABRIC
MB-5070	ARFRP1	cna	hetloss	METABRIC
MB-5107	ARFRP1	cna	gain	METABRIC
MB-6060	ARFRP1	cna	amp	METABRIC
MB-6098	ARFRP1	cna	hetloss	METABRIC
MTS-T0064	ARFRP1	cna	hetloss	METABRIC
PD11327	ARFRP1	cna	gain	BASIS
PD11742	ARFRP1	cna	gain	BASIS
PD13296	ARFRP1	cna	gain	BASIS
PD13299	ARFRP1	cna	gain	BASIS
PD13771	ARFRP1	cna	gain	BASIS
PD14442	ARFRP1	cna	hetloss	BASIS
PD22355	ARFRP1	cna	gain	BASIS
PD23562	ARFRP1	cna	gain	BASIS
PD23574	ARFRP1	cna	gain	BASIS
PD24186	ARFRP1	cna	gain	BASIS
PD24202	ARFRP1	cna	hetloss	BASIS
PD24206	ARFRP1	cna	amp	BASIS
PD3905	ARFRP1	cna	gain	BASIS
PD4005	ARFRP1	cna	gain	BASIS
PD4006	ARFRP1	cna	gain	BASIS
PD4107	ARFRP1	cna	gain	BASIS
PD4826	ARFRP1	cna	gain	BASIS
PD5935	ARFRP1	cna	gain	BASIS
PD5945	ARFRP1	cna	amp	BASIS
PD5948	ARFRP1	cna	gain	BASIS
PD6406	ARFRP1	cna	gain	BASIS
PD7067	ARFRP1	cna	gain	BASIS
PD7215	ARFRP1	cna	amp	BASIS
PD8980	ARFRP1	cna	gain	BASIS
PD9004	ARFRP1	cna	gain	BASIS
PD9585	ARFRP1	cna	hetloss	BASIS
PD9702	ARFRP1	cna	gain	BASIS
TCGA-A2-A25B	CCNL1	cna	gain	TCGA
TCGA-AN-A0XU	CCNL1	cna	gain	TCGA
TCGA-BH-A0AW	CCNL1	cna	gain	TCGA
TCGA-BH-A0C0	CCNL1	cna	gain	TCGA

TCGA-BH-A18R	CCNL1	cna	gain	TCGA
TCGA-BH-A1FU	CCNL1	cna	hetloss	TCGA
TCGA-C8-A12L	CCNL1	cna	amp	TCGA
TCGA-E2-A1L7	CCNL1	cna	gain	TCGA
TCGA-E9-A1NC	CCNL1	cna	gain	TCGA
TCGA-LL-A5YP	CCNL1	cna	gain	TCGA
MB-0346	CCNL1	cna	gain	METABRIC
MB-5070	CCNL1	cna	amp	METABRIC
MB-5107	CCNL1	cna	amp	METABRIC
MB-5465	CCNL1	cna	amp	METABRIC
MB-6060	CCNL1	cna	gain	METABRIC
MB-6098	CCNL1	cna	gain	METABRIC
MB-7038	CCNL1	cna	hetloss	METABRIC
MB-7048	CCNL1	cna	hetloss	METABRIC
MB-0420	CCNL1	cna	amp	METABRIC
PD11327	CCNL1	cna	gain	BASIS
PD13296	CCNL1	cna	gain	BASIS
PD13297	CCNL1	cna	gain	BASIS
PD13299	CCNL1	cna	gain	BASIS
PD13771	CCNL1	cna	hetloss	BASIS
PD22355	CCNL1	cna	gain	BASIS
PD23561	CCNL1	cna	hetloss	BASIS
PD23562	CCNL1	cna	amp	BASIS
PD23574	CCNL1	cna	gain	BASIS
PD23578	CCNL1	cna	gain	BASIS
PD24186	CCNL1	cna	gain	BASIS
PD24202	CCNL1	cna	gain	BASIS
PD24206	CCNL1	cna	gain	BASIS
PD3905	CCNL1	cna	gain	BASIS
PD4107	CCNL1	cna	gain	BASIS
PD5930	CCNL1	cna	gain	BASIS
PD5935	CCNL1	cna	gain	BASIS
PD5945	CCNL1	cna	amp	BASIS
PD5948	CCNL1	cna	gain	BASIS
PD6731	CCNL1	cna	gain	BASIS
PD7067	CCNL1	cna	gain	BASIS
PD7215	CCNL1	cna	gain	BASIS
PD8621	CCNL1	cna	gain	BASIS
PD8980	CCNL1	cna	gain	BASIS
PD9702	CCNL1	cna	amp	BASIS
TCGA-A2-A25B	DDX5	cna	amp	TCGA
TCGA-AN-A0XU	DDX5	cna	gain	TCGA
TCGA-AO-A0JL	DDX5	cna	hetloss	TCGA
TCGA-BH-A0AW	DDX5	cna	amp	TCGA
TCGA-BH-A0C0	DDX5	cna	gain	TCGA
TCGA-C8-A12L	DDX5	cna	gain	TCGA
TCGA-D8-A27M	DDX5	cna	hetloss	TCGA

TCGA-EW-A1OX	DDX5	cna	amp	TCGA
TCGA-LL-A5YP	DDX5	cna	amp	TCGA
MB-0346	DDX5	cna	gain	METABRIC
MB-2827	DDX5	cna	hetloss	METABRIC
MB-5070	DDX5	cna	gain	METABRIC
MB-5107	DDX5	cna	amp	METABRIC
MB-5323	DDX5	cna	gain	METABRIC
MB-6060	DDX5	cna	amp	METABRIC
MB-6098	DDX5	cna	gain	METABRIC
MB-6271	DDX5	cna	hetloss	METABRIC
MB-7048	DDX5	cna	gain	METABRIC
MTS-T0064	DDX5	cna	gain	METABRIC
PD11327	DDX5	cna	gain	BASIS
PD11742	DDX5	cna	gain	BASIS
PD22355	DDX5	cna	gain	BASIS
PD23561	DDX5	cna	gain	BASIS
PD23562	DDX5	cna	gain	BASIS
PD23574	DDX5	cna	gain	BASIS
PD24186	DDX5	cna	gain	BASIS
PD24206	DDX5	cna	hetloss	BASIS
PD24337	DDX5	cna	hetloss	BASIS
PD3890	DDX5	cna	gain	BASIS
PD3905	DDX5	cna	gain	BASIS
PD4006	DDX5	cna	amp	BASIS
PD4826	DDX5	cna	gain	BASIS
PD4967	DDX5	cna	homdel	BASIS
PD5930	DDX5	cna	gain	BASIS
PD5935	DDX5	cna	gain	BASIS
PD5945	DDX5	cna	amp	BASIS
PD5948	DDX5	cna	gain	BASIS
PD6731	DDX5	cna	hetloss	BASIS
PD7067	DDX5	cna	gain	BASIS
PD7215	DDX5	cna	gain	BASIS
PD8621	DDX5	cna	gain	BASIS
PD8980	DDX5	cna	gain	BASIS
PD9004	DDX5	cna	gain	BASIS
PD9702	DDX5	cna	gain	BASIS
TCGA-A2-A25B	FGFR1OP	cna	hetloss	TCGA
TCGA-AN-A0XU	FGFR1OP	cna	gain	TCGA
TCGA-AO-A0JL	FGFR1OP	cna	hetloss	TCGA
TCGA-BH-A0AW	FGFR1OP	cna	amp	TCGA
TCGA-BH-A0C0	FGFR1OP	cna	hetloss	TCGA
TCGA-BH-A18R	FGFR1OP	cna	homdel	TCGA
TCGA-C8-A12L	FGFR1OP	cna	hetloss	TCGA
TCGA-D8-A27M	FGFR1OP	cna	hetloss	TCGA
TCGA-E9-A1NC	FGFR1OP	cna	hetloss	TCGA
TCGA-LL-A5YP	FGFR1OP	cna	gain	TCGA

MB-2827	FGFR1OP	cna	hetloss	METABRIC
MB-5107	FGFR1OP	cna	hetloss	METABRIC
MB-6060	FGFR1OP	cna	hetloss	METABRIC
MB-6098	FGFR1OP	cna	hetloss	METABRIC
MB-7032	FGFR1OP	cna	gain	METABRIC
MB-7048	FGFR1OP	cna	hetloss	METABRIC
MTS-T0064	FGFR1OP	cna	hetloss	METABRIC
PD10014	FGFR1OP	cna	hetloss	BASIS
PD11327	FGFR1OP	cna	gain	BASIS
PD13296	FGFR1OP	cna	homdel	BASIS
PD13297	FGFR1OP	cna	hetloss	BASIS
PD13299	FGFR1OP	cna	gain	BASIS
PD23562	FGFR1OP	cna	gain	BASIS
PD23574	FGFR1OP	cna	amp	BASIS
PD24186	FGFR1OP	cna	gain	BASIS
PD24206	FGFR1OP	cna	gain	BASIS
PD24337	FGFR1OP	cna	hetloss	BASIS
PD3890	FGFR1OP	cna	hetloss	BASIS
PD3905	FGFR1OP	cna	gain	BASIS
PD4005	FGFR1OP	cna	hetloss	BASIS
PD4006	FGFR1OP	cna	gain	BASIS
PD4826	FGFR1OP	cna	gain	BASIS
PD4967	FGFR1OP	cna	hetloss	BASIS
PD5935	FGFR1OP	cna	gain	BASIS
PD5945	FGFR1OP	cna	gain	BASIS
PD5948	FGFR1OP	cna	hetloss	BASIS
PD6406	FGFR1OP	cna	hetloss	BASIS
PD6413	FGFR1OP	cna	hetloss	BASIS
PD6731	FGFR1OP	cna	hetloss	BASIS
PD7067	FGFR1OP	cna	amp	BASIS
PD8980	FGFR1OP	cna	gain	BASIS
PD9004	FGFR1OP	cna	gain	BASIS
PD9585	FGFR1OP	cna	hetloss	BASIS
PD9702	FGFR1OP	cna	gain	BASIS
TCGA-A2-A25B	IKZF3	cna	hetloss	TCGA
TCGA-AN-A0XU	IKZF3	cna	gain	TCGA
TCGA-AO-A0JL	IKZF3	cna	amp	TCGA
TCGA-BH-A0AW	IKZF3	cna	amp	TCGA
TCGA-BH-A0C0	IKZF3	cna	gain	TCGA
TCGA-BH-A18R	IKZF3	cna	amp	TCGA
TCGA-BH-A1FU	IKZF3	cna	amp	TCGA
TCGA-C8-A12L	IKZF3	cna	amp	TCGA
TCGA-D8-A27M	IKZF3	cna	hetloss	TCGA
TCGA-E2-A1L7	IKZF3	cna	hetloss	TCGA
TCGA-E9-A1NC	IKZF3	cna	hetloss	TCGA
MB-0346	IKZF3	cna	amp	METABRIC
MB-2827	IKZF3	cna	hetloss	METABRIC

MB-5070	IKZF3	cna	hetloss	METABRIC
MB-5107	IKZF3	cna	gain	METABRIC
MB-5465	IKZF3	cna	hetloss	METABRIC
MB-6060	IKZF3	cna	amp	METABRIC
MB-6098	IKZF3	cna	hetloss	METABRIC
MB-6271	IKZF3	cna	hetloss	METABRIC
MB-7038	IKZF3	cna	hetloss	METABRIC
MB-7048	IKZF3	cna	gain	METABRIC
MB-0420	IKZF3	cna	hetloss	METABRIC
PD13297	IKZF3	cna	hetloss	BASIS
PD13299	IKZF3	cna	gain	BASIS
PD13771	IKZF3	cna	hetloss	BASIS
PD23561	IKZF3	cna	amp	BASIS
PD23562	IKZF3	cna	gain	BASIS
PD24186	IKZF3	cna	gain	BASIS
PD24206	IKZF3	cna	hetloss	BASIS
PD24337	IKZF3	cna	hetloss	BASIS
PD3890	IKZF3	cna	hetloss	BASIS
PD3905	IKZF3	cna	gain	BASIS
PD4005	IKZF3	cna	hetloss	BASIS
PD4826	IKZF3	cna	amp	BASIS
PD4967	IKZF3	cna	hetloss	BASIS
PD5945	IKZF3	cna	amp	BASIS
PD5948	IKZF3	cna	gain	BASIS
PD6406	IKZF3	cna	hetloss	BASIS
PD6731	IKZF3	cna	hetloss	BASIS
PD7067	IKZF3	cna	gain	BASIS
PD7215	IKZF3	cna	gain	BASIS
PD9004	IKZF3	cna	gain	BASIS
PD9585	IKZF3	cna	hetloss	BASIS
PD9702	IKZF3	cna	amp	BASIS
TCGA-A2-A25B	KIAA1549	cna	gain	TCGA
TCGA-AO-A0JL	KIAA1549	cna	gain	TCGA
TCGA-BH-A0AW	KIAA1549	cna	gain	TCGA
TCGA-D8-A27M	KIAA1549	cna	gain	TCGA
TCGA-E2-A1L7	KIAA1549	cna	hetloss	TCGA
TCGA-E9-A1NC	KIAA1549	cna	gain	TCGA
TCGA-LL-A5YP	KIAA1549	cna	gain	TCGA
MB-0346	KIAA1549	cna	hetloss	METABRIC
MB-2827	KIAA1549	cna	hetloss	METABRIC
MB-5070	KIAA1549	cna	hetloss	METABRIC
MB-5107	KIAA1549	cna	gain	METABRIC
MB-6098	KIAA1549	cna	gain	METABRIC
MB-7038	KIAA1549	cna	gain	METABRIC
MB-7048	KIAA1549	cna	gain	METABRIC
PD10014	KIAA1549	cna	gain	BASIS
PD13296	KIAA1549	cna	hetloss	BASIS

PD13297	KIAA1549	cna	gain	BASIS
PD13299	KIAA1549	cna	gain	BASIS
PD22355	KIAA1549	cna	hetloss	BASIS
PD23562	KIAA1549	cna	gain	BASIS
PD23574	KIAA1549	cna	gain	BASIS
PD23578	KIAA1549	cna	hetloss	BASIS
PD24186	KIAA1549	cna	amp	BASIS
PD24202	KIAA1549	cna	gain	BASIS
PD24206	KIAA1549	cna	hetloss	BASIS
PD24337	KIAA1549	cna	gain	BASIS
PD3890	KIAA1549	cna	hetloss	BASIS
PD3905	KIAA1549	cna	gain	BASIS
PD4006	KIAA1549	cna	gain	BASIS
PD4107	KIAA1549	cna	amp	BASIS
PD4826	KIAA1549	cna	gain	BASIS
PD4967	KIAA1549	cna	hetloss	BASIS
PD5930	KIAA1549	cna	gain	BASIS
PD5935	KIAA1549	cna	gain	BASIS
PD5945	KIAA1549	cna	amp	BASIS
PD5948	KIAA1549	cna	gain	BASIS
PD6406	KIAA1549	cna	gain	BASIS
PD6731	KIAA1549	cna	hetloss	BASIS
PD7067	KIAA1549	cna	gain	BASIS
PD7215	KIAA1549	cna	gain	BASIS
PD8621	KIAA1549	cna	gain	BASIS
PD9004	KIAA1549	cna	gain	BASIS
PD9585	KIAA1549	cna	gain	BASIS
PD9702	KIAA1549	cna	gain	BASIS
TCGA-A2-A25B	LATS1	cna	hetloss	TCGA
TCGA-AN-A0XU	LATS1	cna	gain	TCGA
TCGA-AO-A0JL	LATS1	cna	hetloss	TCGA
TCGA-BH-A0AW	LATS1	cna	amp	TCGA
TCGA-BH-A0C0	LATS1	cna	gain	TCGA
TCGA-BH-A18R	LATS1	cna	gain	TCGA
TCGA-C8-A12L	LATS1	cna	gain	TCGA
TCGA-D8-A27M	LATS1	cna	hetloss	TCGA
TCGA-E2-A1L7	LATS1	cna	gain	TCGA
MB-2827	LATS1	cna	hetloss	METABRIC
MB-5070	LATS1	cna	amp	METABRIC
MB-5107	LATS1	cna	hetloss	METABRIC
MB-6098	LATS1	cna	hetloss	METABRIC
MB-7032	LATS1	cna	hetloss	METABRIC
MB-7038	LATS1	cna	amp	METABRIC
MB-7048	LATS1	cna	hetloss	METABRIC
MB-0420	LATS1	cna	hetloss	METABRIC
PD11327	LATS1	cna	gain	BASIS
PD13296	LATS1	cna	hetloss	BASIS

PD13297	LATS1	cna	hetloss	BASIS
PD13299	LATS1	cna	gain	BASIS
PD23562	LATS1	cna	gain	BASIS
PD23574	LATS1	cna	gain	BASIS
PD24186	LATS1	cna	gain	BASIS
PD24202	LATS1	cna	gain	BASIS
PD24206	LATS1	cna	gain	BASIS
PD24337	LATS1	cna	hetloss	BASIS
PD3890	LATS1	cna	hetloss	BASIS
PD3905	LATS1	cna	gain	BASIS
PD4005	LATS1	cna	hetloss	BASIS
PD4006	LATS1	cna	gain	BASIS
PD4826	LATS1	cna	gain	BASIS
PD4967	LATS1	cna	hetloss	BASIS
PD5935	LATS1	cna	gain	BASIS
PD5945	LATS1	cna	amp	BASIS
PD6406	LATS1	cna	hetloss	BASIS
PD6413	LATS1	cna	hetloss	BASIS
PD6731	LATS1	cna	hetloss	BASIS
PD7067	LATS1	cna	amp	BASIS
PD7215	LATS1	cna	gain	BASIS
PD8980	LATS1	cna	gain	BASIS
PD9004	LATS1	cna	homdel	BASIS
PD9585	LATS1	cna	hetloss	BASIS
PD9702	LATS1	cna	gain	BASIS
TCGA-A2-A25B	MCL1	cna	gain	TCGA
TCGA-AN-A0XU	MCL1	cna	gain	TCGA
TCGA-AO-A0JL	MCL1	cna	gain	TCGA
TCGA-BH-A0AW	MCL1	cna	gain	TCGA
TCGA-BH-A0C0	MCL1	cna	hetloss	TCGA
TCGA-BH-A18R	MCL1	cna	gain	TCGA
TCGA-BH-A1FU	MCL1	cna	gain	TCGA
TCGA-C8-A12L	MCL1	cna	gain	TCGA
TCGA-E2-A1L7	MCL1	cna	gain	TCGA
TCGA-EW-A1OX	MCL1	cna	gain	TCGA
TCGA-LL-A5YP	MCL1	cna	amp	TCGA
MB-2827	MCL1	cna	amp	METABRIC
MB-5070	MCL1	cna	gain	METABRIC
MB-6098	MCL1	cna	amp	METABRIC
MB-7032	MCL1	cna	gain	METABRIC
MB-7048	MCL1	cna	hetloss	METABRIC
MB-0420	MCL1	cna	gain	METABRIC
P-0002858-T01-IM3	MCL1	cna	amp	MSK-IMPACT
PD10014	MCL1	cna	gain	BASIS
PD11327	MCL1	cna	gain	BASIS
PD13297	MCL1	cna	gain	BASIS
PD13299	MCL1	cna	gain	BASIS

PD14442	MCL1	cna	gain	BASIS
PD22355	MCL1	cna	gain	BASIS
PD23562	MCL1	cna	amp	BASIS
PD23574	MCL1	cna	gain	BASIS
PD24186	MCL1	cna	gain	BASIS
PD24206	MCL1	cna	gain	BASIS
PD3905	MCL1	cna	gain	BASIS
PD4005	MCL1	cna	gain	BASIS
PD4006	MCL1	cna	amp	BASIS
PD4107	MCL1	cna	gain	BASIS
PD4826	MCL1	cna	gain	BASIS
PD4967	MCL1	cna	hetloss	BASIS
PD5930	MCL1	cna	amp	BASIS
PD5935	MCL1	cna	gain	BASIS
PD5945	MCL1	cna	amp	BASIS
PD5948	MCL1	cna	gain	BASIS
PD6413	MCL1	cna	gain	BASIS
PD7067	MCL1	cna	amp	BASIS
PD7215	MCL1	cna	gain	BASIS
PD8621	MCL1	cna	amp	BASIS
PD9004	MCL1	cna	amp	BASIS
PD9702	MCL1	cna	amp	BASIS
TCGA-A2-A25B	MECOM	cna	gain	TCGA
TCGA-AN-A0XU	MECOM	cna	gain	TCGA
TCGA-BH-A0AW	MECOM	cna	gain	TCGA
TCGA-BH-A0C0	MECOM	cna	gain	TCGA
TCGA-BH-A18R	MECOM	cna	amp	TCGA
TCGA-BH-A1FU	MECOM	cna	hetloss	TCGA
TCGA-C8-A12L	MECOM	cna	gain	TCGA
TCGA-E2-A1L7	MECOM	cna	gain	TCGA
TCGA-LL-A5YP	MECOM	cna	gain	TCGA
MB-0346	MECOM	cna	gain	METABRIC
MB-2827	MECOM	cna	hetloss	METABRIC
MB-5070	MECOM	cna	amp	METABRIC
MB-5107	MECOM	cna	amp	METABRIC
MB-6060	MECOM	cna	gain	METABRIC
MB-7038	MECOM	cna	hetloss	METABRIC
MB-0420	MECOM	cna	amp	METABRIC
MTS-T0064	MECOM	cna	amp	METABRIC
PD11327	MECOM	cna	amp	BASIS
PD13296	MECOM	cna	amp	BASIS
PD13299	MECOM	cna	gain	BASIS
PD13771	MECOM	cna	hetloss	BASIS
PD22355	MECOM	cna	gain	BASIS
PD23561	MECOM	cna	hetloss	BASIS
PD23562	MECOM	cna	amp	BASIS
PD23574	MECOM	cna	gain	BASIS

PD23578	MECOM	cna	gain	BASIS
PD24186	MECOM	cna	amp	BASIS
PD24202	MECOM	cna	gain	BASIS
PD24206	MECOM	cna	gain	BASIS
PD24337	MECOM	cna	gain	BASIS
PD3905	MECOM	cna	gain	BASIS
PD4005	MECOM	cna	gain	BASIS
PD4107	MECOM	cna	gain	BASIS
PD4826	MECOM	cna	gain	BASIS
PD5930	MECOM	cna	gain	BASIS
PD5935	MECOM	cna	amp	BASIS
PD5945	MECOM	cna	amp	BASIS
PD5948	MECOM	cna	amp	BASIS
PD6406	MECOM	cna	gain	BASIS
PD7067	MECOM	cna	gain	BASIS
PD7215	MECOM	cna	gain	BASIS
PD8621	MECOM	cna	gain	BASIS
PD8980	MECOM	cna	gain	BASIS
PD9702	MECOM	cna	amp	BASIS
TCGA-A2-A25B	MLLT4	cna	hetloss	TCGA
TCGA-AN-A0XU	MLLT4	cna	gain	TCGA
TCGA-AO-A0JL	MLLT4	cna	hetloss	TCGA
TCGA-BH-A0AW	MLLT4	cna	amp	TCGA
TCGA-BH-A0C0	MLLT4	cna	hetloss	TCGA
TCGA-BH-A18R	MLLT4	cna	homdel	TCGA
TCGA-C8-A12L	MLLT4	cna	hetloss	TCGA
TCGA-D8-A27M	MLLT4	cna	hetloss	TCGA
TCGA-E9-A1NC	MLLT4	cna	hetloss	TCGA
TCGA-LL-A5YP	MLLT4	cna	gain	TCGA
MB-2827	MLLT4	cna	hetloss	METABRIC
MB-5107	MLLT4	cna	hetloss	METABRIC
MB-6060	MLLT4	cna	hetloss	METABRIC
MB-6098	MLLT4	cna	hetloss	METABRIC
MB-7032	MLLT4	cna	gain	METABRIC
MB-7048	MLLT4	cna	hetloss	METABRIC
MTS-T0064	MLLT4	cna	hetloss	METABRIC
PD10014	MLLT4	cna	hetloss	BASIS
PD11327	MLLT4	cna	gain	BASIS
PD13296	MLLT4	cna	homdel	BASIS
PD13297	MLLT4	cna	hetloss	BASIS
PD13299	MLLT4	cna	gain	BASIS
PD22355	MLLT4	cna	gain	BASIS
PD23562	MLLT4	cna	gain	BASIS
PD23574	MLLT4	cna	amp	BASIS
PD24186	MLLT4	cna	gain	BASIS
PD24206	MLLT4	cna	amp	BASIS
PD24337	MLLT4	cna	hetloss	BASIS

PD3890	MLLT4	cna	hetloss	BASIS
PD3905	MLLT4	cna	gain	BASIS
PD4006	MLLT4	cna	gain	BASIS
PD4826	MLLT4	cna	gain	BASIS
PD4967	MLLT4	cna	hetloss	BASIS
PD5935	MLLT4	cna	amp	BASIS
PD5945	MLLT4	cna	gain	BASIS
PD5948	MLLT4	cna	hetloss	BASIS
PD6406	MLLT4	cna	hetloss	BASIS
PD6413	MLLT4	cna	hetloss	BASIS
PD6731	MLLT4	cna	hetloss	BASIS
PD7067	MLLT4	cna	amp	BASIS
PD8980	MLLT4	cna	gain	BASIS
PD9004	MLLT4	cna	gain	BASIS
PD9585	MLLT4	cna	hetloss	BASIS
PD9702	MLLT4	cna	gain	BASIS
TCGA-A2-A25B	PTPRO	cna	hetloss	TCGA
TCGA-AN-A0XU	PTPRO	cna	hetloss	TCGA
TCGA-AO-A0JL	PTPRO	cna	hetloss	TCGA
TCGA-BH-A0C0	PTPRO	cna	hetloss	TCGA
TCGA-BH-A18R	PTPRO	cna	gain	TCGA
TCGA-BH-A1FU	PTPRO	cna	hetloss	TCGA
TCGA-C8-A12L	PTPRO	cna	gain	TCGA
TCGA-D8-A27M	PTPRO	cna	gain	TCGA
TCGA-E2-A1L7	PTPRO	cna	hetloss	TCGA
TCGA-LL-A5YP	PTPRO	cna	hetloss	TCGA
MB-0346	PTPRO	cna	hetloss	METABRIC
MB-2827	PTPRO	cna	hetloss	METABRIC
MB-5070	PTPRO	cna	hetloss	METABRIC
MB-6060	PTPRO	cna	gain	METABRIC
MB-6098	PTPRO	cna	gain	METABRIC
MB-7038	PTPRO	cna	hetloss	METABRIC
MB-7048	PTPRO	cna	gain	METABRIC
PD10014	PTPRO	cna	gain	BASIS
PD11327	PTPRO	cna	gain	BASIS
PD11742	PTPRO	cna	gain	BASIS
PD13296	PTPRO	cna	hetloss	BASIS
PD13297	PTPRO	cna	hetloss	BASIS
PD13299	PTPRO	cna	gain	BASIS
PD13771	PTPRO	cna	hetloss	BASIS
PD23574	PTPRO	cna	gain	BASIS
PD24186	PTPRO	cna	gain	BASIS
PD24202	PTPRO	cna	hetloss	BASIS
PD3890	PTPRO	cna	hetloss	BASIS
PD3905	PTPRO	cna	gain	BASIS
PD4005	PTPRO	cna	hetloss	BASIS
PD4006	PTPRO	cna	gain	BASIS

PD4107	PTPRO	cna	gain	BASIS
PD4826	PTPRO	cna	hetloss	BASIS
PD5930	PTPRO	cna	gain	BASIS
PD5935	PTPRO	cna	gain	BASIS
PD5945	PTPRO	cna	amp	BASIS
PD5948	PTPRO	cna	hetloss	BASIS
PD6406	PTPRO	cna	amp	BASIS
PD6731	PTPRO	cna	hetloss	BASIS
PD7215	PTPRO	cna	gain	BASIS
PD8621	PTPRO	cna	gain	BASIS
PD8980	PTPRO	cna	hetloss	BASIS
PD9004	PTPRO	cna	gain	BASIS
PD9702	PTPRO	cna	gain	BASIS
TCGA-A2-A25B	RAD51C	cna	amp	TCGA
TCGA-AN-A0XU	RAD51C	cna	gain	TCGA
TCGA-AO-A0JL	RAD51C	cna	hetloss	TCGA
TCGA-BH-A0AW	RAD51C	cna	amp	TCGA
TCGA-BH-A0C0	RAD51C	cna	gain	TCGA
TCGA-BH-A18R	RAD51C	cna	amp	TCGA
TCGA-C8-A12L	RAD51C	cna	gain	TCGA
TCGA-D8-A27M	RAD51C	cna	hetloss	TCGA
TCGA-EW-A1OX	RAD51C	cna	amp	TCGA
MB-0346	RAD51C	cna	gain	METABRIC
MB-2827	RAD51C	cna	hetloss	METABRIC
MB-5070	RAD51C	cna	hetloss	METABRIC
MB-5107	RAD51C	cna	amp	METABRIC
MB-5323	RAD51C	cna	gain	METABRIC
MB-6060	RAD51C	cna	hetloss	METABRIC
MB-6098	RAD51C	cna	hetloss	METABRIC
MB-6271	RAD51C	cna	hetloss	METABRIC
MB-7048	RAD51C	cna	hetloss	METABRIC
P-0002023-T01-IM3	RAD51C	cna	amp	MSK-IMPACT
P-0002858-T01-IM3	RAD51C	cna	amp	MSK-IMPACT
PD11327	RAD51C	cna	gain	BASIS
PD11742	RAD51C	cna	gain	BASIS
PD13299	RAD51C	cna	gain	BASIS
PD22355	RAD51C	cna	gain	BASIS
PD23561	RAD51C	cna	hetloss	BASIS
PD23562	RAD51C	cna	gain	BASIS
PD23574	RAD51C	cna	gain	BASIS
PD24186	RAD51C	cna	gain	BASIS
PD24337	RAD51C	cna	hetloss	BASIS
PD3890	RAD51C	cna	hetloss	BASIS
PD3905	RAD51C	cna	gain	BASIS
PD4006	RAD51C	cna	amp	BASIS
PD4967	RAD51C	cna	homdel	BASIS
PD5930	RAD51C	cna	gain	BASIS

PD5945	RAD51C	cna	amp	BASIS
PD5948	RAD51C	cna	gain	BASIS
PD6406	RAD51C	cna	hetloss	BASIS
PD6731	RAD51C	cna	hetloss	BASIS
PD7067	RAD51C	cna	gain	BASIS
PD7215	RAD51C	cna	gain	BASIS
PD8621	RAD51C	cna	gain	BASIS
PD8980	RAD51C	cna	gain	BASIS
PD9004	RAD51C	cna	gain	BASIS
PD9702	RAD51C	cna	gain	BASIS
TCGA-A2-A25B	SS18L1	cna	gain	TCGA
TCGA-AN-A0XU	SS18L1	cna	gain	TCGA
TCGA-AO-A0JL	SS18L1	cna	gain	TCGA
TCGA-BH-A0AW	SS18L1	cna	gain	TCGA
TCGA-BH-A18R	SS18L1	cna	gain	TCGA
TCGA-C8-A12L	SS18L1	cna	gain	TCGA
TCGA-D8-A27M	SS18L1	cna	hetloss	TCGA
TCGA-E2-A1L7	SS18L1	cna	gain	TCGA
TCGA-E9-A1NC	SS18L1	cna	gain	TCGA
TCGA-LL-A5YP	SS18L1	cna	gain	TCGA
MB-0346	SS18L1	cna	gain	METABRIC
MB-2827	SS18L1	cna	hetloss	METABRIC
MB-5070	SS18L1	cna	hetloss	METABRIC
MB-5107	SS18L1	cna	gain	METABRIC
MB-6060	SS18L1	cna	gain	METABRIC
MB-6098	SS18L1	cna	hetloss	METABRIC
PD11327	SS18L1	cna	gain	BASIS
PD11742	SS18L1	cna	gain	BASIS
PD13296	SS18L1	cna	gain	BASIS
PD13297	SS18L1	cna	hetloss	BASIS
PD13299	SS18L1	cna	gain	BASIS
PD13771	SS18L1	cna	gain	BASIS
PD22355	SS18L1	cna	gain	BASIS
PD23562	SS18L1	cna	gain	BASIS
PD23574	SS18L1	cna	gain	BASIS
PD23578	SS18L1	cna	gain	BASIS
PD24186	SS18L1	cna	gain	BASIS
PD24202	SS18L1	cna	hetloss	BASIS
PD24206	SS18L1	cna	gain	BASIS
PD3905	SS18L1	cna	gain	BASIS
PD4005	SS18L1	cna	hetloss	BASIS
PD4006	SS18L1	cna	gain	BASIS
PD4107	SS18L1	cna	gain	BASIS
PD4826	SS18L1	cna	gain	BASIS
PD5935	SS18L1	cna	gain	BASIS
PD5945	SS18L1	cna	amp	BASIS
PD5948	SS18L1	cna	gain	BASIS

PD6406	SS18L1	cna	gain	BASIS
PD7067	SS18L1	cna	gain	BASIS
PD7215	SS18L1	cna	amp	BASIS
PD8980	SS18L1	cna	gain	BASIS
PD9004	SS18L1	cna	gain	BASIS
PD9585	SS18L1	cna	hetloss	BASIS
PD9702	SS18L1	cna	gain	BASIS
TCGA-A2-A25B	TIPARP	cna	gain	TCGA
TCGA-AN-A0XU	TIPARP	cna	gain	TCGA
TCGA-BH-A0C0	TIPARP	cna	gain	TCGA
TCGA-BH-A18R	TIPARP	cna	gain	TCGA
TCGA-BH-A1FU	TIPARP	cna	hetloss	TCGA
TCGA-C8-A12L	TIPARP	cna	gain	TCGA
TCGA-E2-A1L7	TIPARP	cna	gain	TCGA
TCGA-E9-A1NC	TIPARP	cna	gain	TCGA
TCGA-LL-A5YP	TIPARP	cna	gain	TCGA
MB-0346	TIPARP	cna	hetloss	METABRIC
MB-5070	TIPARP	cna	amp	METABRIC
MB-5107	TIPARP	cna	amp	METABRIC
MB-5465	TIPARP	cna	amp	METABRIC
MB-6060	TIPARP	cna	gain	METABRIC
MB-6098	TIPARP	cna	gain	METABRIC
MB-7038	TIPARP	cna	hetloss	METABRIC
MB-7048	TIPARP	cna	hetloss	METABRIC
MB-0420	TIPARP	cna	amp	METABRIC
PD11327	TIPARP	cna	gain	BASIS
PD13296	TIPARP	cna	gain	BASIS
PD13297	TIPARP	cna	gain	BASIS
PD13299	TIPARP	cna	gain	BASIS
PD13771	TIPARP	cna	hetloss	BASIS
PD22355	TIPARP	cna	gain	BASIS
PD23561	TIPARP	cna	hetloss	BASIS
PD23562	TIPARP	cna	amp	BASIS
PD23574	TIPARP	cna	gain	BASIS
PD23578	TIPARP	cna	gain	BASIS
PD24186	TIPARP	cna	gain	BASIS
PD24202	TIPARP	cna	gain	BASIS
PD24206	TIPARP	cna	gain	BASIS
PD3905	TIPARP	cna	gain	BASIS
PD4107	TIPARP	cna	gain	BASIS
PD5930	TIPARP	cna	gain	BASIS
PD5935	TIPARP	cna	gain	BASIS
PD5945	TIPARP	cna	amp	BASIS
PD5948	TIPARP	cna	amp	BASIS
PD6731	TIPARP	cna	gain	BASIS
PD7067	TIPARP	cna	gain	BASIS
PD7215	TIPARP	cna	amp	BASIS

PD8621	TIPARP	cna	gain	BASIS
PD8980	TIPARP	cna	gain	BASIS
PD9585	TIPARP	cna	hetloss	BASIS
PD9702	TIPARP	cna	amp	BASIS
TCGA-A2-A25B	ZNF217	cna	gain	TCGA
TCGA-AO-A0JL	ZNF217	cna	gain	TCGA
TCGA-BH-A0AW	ZNF217	cna	gain	TCGA
TCGA-BH-A0C0	ZNF217	cna	gain	TCGA
TCGA-BH-A18R	ZNF217	cna	gain	TCGA
TCGA-C8-A12L	ZNF217	cna	gain	TCGA
TCGA-D8-A27M	ZNF217	cna	hetloss	TCGA
TCGA-E2-A1L7	ZNF217	cna	gain	TCGA
TCGA-LL-A5YP	ZNF217	cna	gain	TCGA
MB-0346	ZNF217	cna	gain	METABRIC
MB-5070	ZNF217	cna	hetloss	METABRIC
MB-5107	ZNF217	cna	gain	METABRIC
MB-6060	ZNF217	cna	gain	METABRIC
MB-6098	ZNF217	cna	hetloss	METABRIC
MB-7038	ZNF217	cna	hetloss	METABRIC
MB-7048	ZNF217	cna	hetloss	METABRIC
PD11327	ZNF217	cna	hetloss	BASIS
PD11742	ZNF217	cna	amp	BASIS
PD13296	ZNF217	cna	gain	BASIS
PD13297	ZNF217	cna	hetloss	BASIS
PD13299	ZNF217	cna	gain	BASIS
PD13771	ZNF217	cna	gain	BASIS
PD22355	ZNF217	cna	gain	BASIS
PD23562	ZNF217	cna	gain	BASIS
PD23574	ZNF217	cna	gain	BASIS
PD24186	ZNF217	cna	gain	BASIS
PD24202	ZNF217	cna	hetloss	BASIS
PD24206	ZNF217	cna	amp	BASIS
PD3905	ZNF217	cna	gain	BASIS
PD4005	ZNF217	cna	homdel	BASIS
PD4006	ZNF217	cna	gain	BASIS
PD4826	ZNF217	cna	gain	BASIS
PD5935	ZNF217	cna	gain	BASIS
PD5945	ZNF217	cna	amp	BASIS
PD5948	ZNF217	cna	gain	BASIS
PD6406	ZNF217	cna	gain	BASIS
PD6731	ZNF217	cna	hetloss	BASIS
PD7067	ZNF217	cna	gain	BASIS
PD7215	ZNF217	cna	amp	BASIS
PD8621	ZNF217	cna	gain	BASIS
PD8980	ZNF217	cna	gain	BASIS
PD9004	ZNF217	cna	gain	BASIS
PD9585	ZNF217	cna	hetloss	BASIS

PD9702	ZNF217	cna	gain	BASIS
TCGA-A2-A25B	ZNF750	cna	gain	TCGA
TCGA-AN-A0XU	ZNF750	cna	amp	TCGA
TCGA-AO-A0JL	ZNF750	cna	hetloss	TCGA
TCGA-BH-A0AW	ZNF750	cna	amp	TCGA
TCGA-BH-A0C0	ZNF750	cna	gain	TCGA
TCGA-BH-A18R	ZNF750	cna	homdel	TCGA
TCGA-C8-A12L	ZNF750	cna	gain	TCGA
TCGA-D8-A27M	ZNF750	cna	gain	TCGA
TCGA-E2-A1L7	ZNF750	cna	gain	TCGA
TCGA-EW-A1OX	ZNF750	cna	amp	TCGA
TCGA-LL-A5YP	ZNF750	cna	amp	TCGA
MB-0346	ZNF750	cna	gain	METABRIC
MB-2827	ZNF750	cna	hetloss	METABRIC
MB-5107	ZNF750	cna	amp	METABRIC
MB-5465	ZNF750	cna	gain	METABRIC
MB-6060	ZNF750	cna	hetloss	METABRIC
MB-6098	ZNF750	cna	gain	METABRIC
MB-6271	ZNF750	cna	hetloss	METABRIC
MTS-T0064	ZNF750	cna	gain	METABRIC
PD10014	ZNF750	cna	gain	BASIS
PD11327	ZNF750	cna	amp	BASIS
PD22355	ZNF750	cna	hetloss	BASIS
PD23561	ZNF750	cna	hetloss	BASIS
PD23574	ZNF750	cna	amp	BASIS
PD24186	ZNF750	cna	amp	BASIS
PD24202	ZNF750	cna	gain	BASIS
PD24337	ZNF750	cna	hetloss	BASIS
PD3890	ZNF750	cna	gain	BASIS
PD3905	ZNF750	cna	gain	BASIS
PD4006	ZNF750	cna	gain	BASIS
PD4107	ZNF750	cna	gain	BASIS
PD4967	ZNF750	cna	hetloss	BASIS
PD5930	ZNF750	cna	gain	BASIS
PD5935	ZNF750	cna	gain	BASIS
PD5945	ZNF750	cna	amp	BASIS
PD5948	ZNF750	cna	gain	BASIS
PD6413	ZNF750	cna	hetloss	BASIS
PD6731	ZNF750	cna	hetloss	BASIS
PD7067	ZNF750	cna	gain	BASIS
PD7215	ZNF750	cna	gain	BASIS
PD8621	ZNF750	cna	gain	BASIS
PD9004	ZNF750	cna	gain	BASIS
PD9585	ZNF750	cna	gain	BASIS
PD9702	ZNF750	cna	gain	BASIS
TCGA-A2-A25B	A2ML1	cna	hetloss	TCGA
TCGA-AN-A0XU	A2ML1	cna	hetloss	TCGA

TCGA-AO-A0JL	A2ML1	cna	hetloss	TCGA
TCGA-BH-A0C0	A2ML1	cna	hetloss	TCGA
TCGA-BH-A18R	A2ML1	cna	gain	TCGA
TCGA-BH-A1FU	A2ML1	cna	hetloss	TCGA
TCGA-C8-A12L	A2ML1	cna	gain	TCGA
TCGA-D8-A27M	A2ML1	cna	gain	TCGA
TCGA-E9-A1NC	A2ML1	cna	gain	TCGA
TCGA-LL-A5YP	A2ML1	cna	hetloss	TCGA
MB-0346	A2ML1	cna	hetloss	METABRIC
MB-5070	A2ML1	cna	amp	METABRIC
MB-5107	A2ML1	cna	hetloss	METABRIC
MB-6098	A2ML1	cna	gain	METABRIC
MB-7048	A2ML1	cna	gain	METABRIC
MTS-T0064	A2ML1	cna	amp	METABRIC
PD10014	A2ML1	cna	hetloss	BASIS
PD11742	A2ML1	cna	gain	BASIS
PD13297	A2ML1	cna	hetloss	BASIS
PD13299	A2ML1	cna	gain	BASIS
PD13771	A2ML1	cna	hetloss	BASIS
PD23574	A2ML1	cna	gain	BASIS
PD23578	A2ML1	cna	gain	BASIS
PD24186	A2ML1	cna	gain	BASIS
PD24206	A2ML1	cna	homdel	BASIS
PD24337	A2ML1	cna	gain	BASIS
PD3890	A2ML1	cna	gain	BASIS
PD3905	A2ML1	cna	gain	BASIS
PD4005	A2ML1	cna	hetloss	BASIS
PD4006	A2ML1	cna	amp	BASIS
PD4107	A2ML1	cna	gain	BASIS
PD4826	A2ML1	cna	hetloss	BASIS
PD4967	A2ML1	cna	hetloss	BASIS
PD5930	A2ML1	cna	gain	BASIS
PD5935	A2ML1	cna	gain	BASIS
PD5945	A2ML1	cna	amp	BASIS
PD6406	A2ML1	cna	gain	BASIS
PD6731	A2ML1	cna	hetloss	BASIS
PD7215	A2ML1	cna	gain	BASIS
PD8621	A2ML1	cna	gain	BASIS
PD8980	A2ML1	cna	hetloss	BASIS
PD9004	A2ML1	cna	gain	BASIS
PD9702	A2ML1	cna	gain	BASIS
TCGA-A2-A25B	ABCA9	cna	gain	TCGA
TCGA-AN-A0XU	ABCA9	cna	gain	TCGA
TCGA-AO-A0JL	ABCA9	cna	hetloss	TCGA
TCGA-BH-A0AW	ABCA9	cna	amp	TCGA
TCGA-BH-A0C0	ABCA9	cna	gain	TCGA
TCGA-BH-A18R	ABCA9	cna	homdel	TCGA

TCGA-C8-A12L	ABCA9	cna	gain	TCGA
TCGA-D8-A27M	ABCA9	cna	gain	TCGA
TCGA-EW-A1OX	ABCA9	cna	amp	TCGA
TCGA-LL-A5YP	ABCA9	cna	amp	TCGA
MB-0346	ABCA9	cna	gain	METABRIC
MB-5070	ABCA9	cna	gain	METABRIC
MB-5107	ABCA9	cna	amp	METABRIC
MB-6098	ABCA9	cna	gain	METABRIC
MB-6271	ABCA9	cna	hetloss	METABRIC
MB-7048	ABCA9	cna	hetloss	METABRIC
MTS-T0064	ABCA9	cna	gain	METABRIC
PD11327	ABCA9	cna	amp	BASIS
PD11742	ABCA9	cna	gain	BASIS
PD23561	ABCA9	cna	gain	BASIS
PD23562	ABCA9	cna	gain	BASIS
PD23574	ABCA9	cna	gain	BASIS
PD24186	ABCA9	cna	gain	BASIS
PD24202	ABCA9	cna	gain	BASIS
PD24206	ABCA9	cna	hetloss	BASIS
PD24337	ABCA9	cna	hetloss	BASIS
PD3890	ABCA9	cna	gain	BASIS
PD3905	ABCA9	cna	gain	BASIS
PD4006	ABCA9	cna	gain	BASIS
PD4107	ABCA9	cna	gain	BASIS
PD4826	ABCA9	cna	amp	BASIS
PD4967	ABCA9	cna	hetloss	BASIS
PD5930	ABCA9	cna	gain	BASIS
PD5935	ABCA9	cna	gain	BASIS
PD5945	ABCA9	cna	amp	BASIS
PD5948	ABCA9	cna	gain	BASIS
PD6731	ABCA9	cna	hetloss	BASIS
PD7067	ABCA9	cna	gain	BASIS
PD7215	ABCA9	cna	gain	BASIS
PD8980	ABCA9	cna	gain	BASIS
PD9004	ABCA9	cna	gain	BASIS
PD9585	ABCA9	cna	gain	BASIS
PD9702	ABCA9	cna	gain	BASIS
TCGA-AN-A0XU	APC	cna	hetloss	TCGA
TCGA-AO-A0JL	APC	cna	hetloss	TCGA
TCGA-BH-A0C0	APC	cna	hetloss	TCGA
TCGA-BH-A1FU	APC	cna	hetloss	TCGA
TCGA-C8-A12L	APC	cna	hetloss	TCGA
TCGA-D8-A27M	APC	cna	hetloss	TCGA
TCGA-E2-A1L7	APC	cna	gain	TCGA
TCGA-E9-A1NC	APC	cna	hetloss	TCGA
TCGA-LL-A5YP	APC	cna	hetloss	TCGA
MB-0346	APC	cna	gain	METABRIC

MB-2827	APC	cna	hetloss	METABRIC
MB-5070	APC	cna	hetloss	METABRIC
MB-5107	APC	cna	hetloss	METABRIC
MB-5465	APC	cna	hetloss	METABRIC
MB-6060	APC	cna	hetloss	METABRIC
MB-6098	APC	cna	hetloss	METABRIC
MB-7038	APC	cna	hetloss	METABRIC
MB-0420	APC	cna	hetloss	METABRIC
PD10014	APC	cna	hetloss	BASIS
PD13296	APC	cna	hetloss	BASIS
PD13297	APC	cna	hetloss	BASIS
PD13771	APC	cna	gain	BASIS
PD22355	APC	cna	hetloss	BASIS
PD23562	APC	cna	hetloss	BASIS
PD24202	APC	cna	hetloss	BASIS
PD24206	APC	cna	gain	BASIS
PD24337	APC	cna	hetloss	BASIS
PD3890	APC	cna	hetloss	BASIS
PD3905	APC	cna	gain	BASIS
PD4005	APC	cna	hetloss	BASIS
PD4006	APC	cna	hetloss	BASIS
PD4107	APC	cna	hetloss	BASIS
PD5930	APC	cna	hetloss	BASIS
PD5935	APC	cna	hetloss	BASIS
PD5945	APC	cna	hetloss	BASIS
PD5948	APC	cna	hetloss	BASIS
PD6406	APC	cna	hetloss	BASIS
PD6413	APC	cna	hetloss	BASIS
PD7067	APC	cna	gain	BASIS
PD8621	APC	cna	hetloss	BASIS
PD8980	APC	cna	hetloss	BASIS
PD9004	APC	cna	gain	BASIS
PD9585	APC	cna	hetloss	BASIS
TCGA-A2-A25B	APH1A	cna	gain	TCGA
TCGA-AN-A0XU	APH1A	cna	gain	TCGA
TCGA-AO-A0JL	APH1A	cna	gain	TCGA
TCGA-BH-A0AW	APH1A	cna	gain	TCGA
TCGA-BH-A0C0	APH1A	cna	hetloss	TCGA
TCGA-BH-A18R	APH1A	cna	gain	TCGA
TCGA-BH-A1FU	APH1A	cna	gain	TCGA
TCGA-C8-A12L	APH1A	cna	gain	TCGA
TCGA-E2-A1L7	APH1A	cna	gain	TCGA
TCGA-EW-A1OX	APH1A	cna	gain	TCGA
TCGA-LL-A5YP	APH1A	cna	amp	TCGA
MB-2827	APH1A	cna	amp	METABRIC
MB-5070	APH1A	cna	gain	METABRIC
MB-6098	APH1A	cna	amp	METABRIC

MB-7032	APH1A	cna	gain	METABRIC
MB-7048	APH1A	cna	hetloss	METABRIC
MB-0420	APH1A	cna	gain	METABRIC
PD10014	APH1A	cna	gain	BASIS
PD11327	APH1A	cna	gain	BASIS
PD13297	APH1A	cna	gain	BASIS
PD13299	APH1A	cna	gain	BASIS
PD14442	APH1A	cna	gain	BASIS
PD22355	APH1A	cna	gain	BASIS
PD23562	APH1A	cna	amp	BASIS
PD23574	APH1A	cna	gain	BASIS
PD24186	APH1A	cna	gain	BASIS
PD24206	APH1A	cna	gain	BASIS
PD3905	APH1A	cna	gain	BASIS
PD4005	APH1A	cna	gain	BASIS
PD4006	APH1A	cna	amp	BASIS
PD4107	APH1A	cna	gain	BASIS
PD4826	APH1A	cna	gain	BASIS
PD4967	APH1A	cna	hetloss	BASIS
PD5930	APH1A	cna	amp	BASIS
PD5935	APH1A	cna	gain	BASIS
PD5945	APH1A	cna	amp	BASIS
PD5948	APH1A	cna	gain	BASIS
PD6413	APH1A	cna	gain	BASIS
PD7067	APH1A	cna	amp	BASIS
PD7215	APH1A	cna	gain	BASIS
PD8621	APH1A	cna	amp	BASIS
PD9004	APH1A	cna	amp	BASIS
PD9702	APH1A	cna	amp	BASIS
TCGA-A2-A25B	ARNT	cna	gain	TCGA
TCGA-AN-A0XU	ARNT	cna	gain	TCGA
TCGA-AO-A0JL	ARNT	cna	gain	TCGA
TCGA-BH-A0AW	ARNT	cna	gain	TCGA
TCGA-BH-A0C0	ARNT	cna	hetloss	TCGA
TCGA-BH-A18R	ARNT	cna	gain	TCGA
TCGA-BH-A1FU	ARNT	cna	gain	TCGA
TCGA-C8-A12L	ARNT	cna	gain	TCGA
TCGA-E2-A1L7	ARNT	cna	gain	TCGA
TCGA-EW-A1OX	ARNT	cna	gain	TCGA
TCGA-LL-A5YP	ARNT	cna	amp	TCGA
MB-2827	ARNT	cna	amp	METABRIC
MB-5070	ARNT	cna	gain	METABRIC
MB-6098	ARNT	cna	amp	METABRIC
MB-7032	ARNT	cna	gain	METABRIC
MB-7048	ARNT	cna	hetloss	METABRIC
MB-0420	ARNT	cna	gain	METABRIC
PD10014	ARNT	cna	gain	BASIS

PD11327	ARNT	cna	gain	BASIS
PD13297	ARNT	cna	gain	BASIS
PD13299	ARNT	cna	gain	BASIS
PD14442	ARNT	cna	gain	BASIS
PD22355	ARNT	cna	gain	BASIS
PD23562	ARNT	cna	amp	BASIS
PD23574	ARNT	cna	gain	BASIS
PD24186	ARNT	cna	gain	BASIS
PD24206	ARNT	cna	gain	BASIS
PD3905	ARNT	cna	gain	BASIS
PD4005	ARNT	cna	gain	BASIS
PD4006	ARNT	cna	amp	BASIS
PD4107	ARNT	cna	gain	BASIS
PD4826	ARNT	cna	gain	BASIS
PD4967	ARNT	cna	hetloss	BASIS
PD5930	ARNT	cna	amp	BASIS
PD5935	ARNT	cna	gain	BASIS
PD5945	ARNT	cna	amp	BASIS
PD5948	ARNT	cna	gain	BASIS
PD6413	ARNT	cna	gain	BASIS
PD7067	ARNT	cna	amp	BASIS
PD7215	ARNT	cna	gain	BASIS
PD8621	ARNT	cna	amp	BASIS
PD9004	ARNT	cna	amp	BASIS
PD9702	ARNT	cna	amp	BASIS
TCGA-A2-A25B	ATM	cna	hetloss	TCGA
TCGA-AN-A0XU	ATM	cna	gain	TCGA
TCGA-AO-A0JL	ATM	cna	hetloss	TCGA
TCGA-BH-A0C0	ATM	cna	gain	TCGA
TCGA-BH-A18R	ATM	cna	homdel	TCGA
TCGA-D8-A27M	ATM	cna	hetloss	TCGA
TCGA-E2-A1L7	ATM	cna	hetloss	TCGA
TCGA-EW-A1OX	ATM	cna	homdel	TCGA
TCGA-LL-A5YP	ATM	cna	hetloss	TCGA
MB-0346	ATM	cna	hetloss	METABRIC
MB-5070	ATM	cna	hetloss	METABRIC
MB-5107	ATM	cna	hetloss	METABRIC
MB-6271	ATM	cna	hetloss	METABRIC
MB-7032	ATM	cna	hetloss	METABRIC
MB-7048	ATM	cna	hetloss	METABRIC
PD11327	ATM	cna	hetloss	BASIS
PD11742	ATM	cna	hetloss	BASIS
PD13296	ATM	cna	hetloss	BASIS
PD13299	ATM	cna	gain	BASIS
PD13771	ATM	cna	hetloss	BASIS
PD22355	ATM	cna	hetloss	BASIS
PD23574	ATM	cna	gain	BASIS

PD23578	ATM	cna	hetloss	BASIS
PD24186	ATM	cna	gain	BASIS
PD24202	ATM	cna	gain	BASIS
PD24206	ATM	cna	gain	BASIS
PD24337	ATM	cna	hetloss	BASIS
PD3905	ATM	cna	gain	BASIS
PD4005	ATM	cna	homdel	BASIS
PD4006	ATM	cna	hetloss	BASIS
PD4107	ATM	cna	hetloss	BASIS
PD4826	ATM	cna	hetloss	BASIS
PD4967	ATM	cna	hetloss	BASIS
PD5935	ATM	cna	gain	BASIS
PD5945	ATM	cna	gain	BASIS
PD5948	ATM	cna	gain	BASIS
PD6413	ATM	cna	hetloss	BASIS
PD6731	ATM	cna	hetloss	BASIS
PD7067	ATM	cna	gain	BASIS
PD7215	ATM	cna	gain	BASIS
PD9004	ATM	cna	hetloss	BASIS
PD9585	ATM	cna	gain	BASIS
PD9702	ATM	cna	gain	BASIS
TCGA-A2-A25B	AURKA	cna	gain	TCGA
TCGA-AO-A0JL	AURKA	cna	gain	TCGA
TCGA-BH-A0AW	AURKA	cna	gain	TCGA
TCGA-BH-A0C0	AURKA	cna	gain	TCGA
TCGA-BH-A18R	AURKA	cna	gain	TCGA
TCGA-C8-A12L	AURKA	cna	gain	TCGA
TCGA-D8-A27M	AURKA	cna	hetloss	TCGA
TCGA-E2-A1L7	AURKA	cna	gain	TCGA
TCGA-LL-A5YP	AURKA	cna	gain	TCGA
MB-0346	AURKA	cna	gain	METABRIC
MB-5070	AURKA	cna	hetloss	METABRIC
MB-5107	AURKA	cna	gain	METABRIC
MB-6060	AURKA	cna	gain	METABRIC
MB-6098	AURKA	cna	gain	METABRIC
P-0002858-T01-IM3	AURKA	cna	amp	MSK-IMPACT
PD11327	AURKA	cna	hetloss	BASIS
PD13296	AURKA	cna	gain	BASIS
PD13297	AURKA	cna	hetloss	BASIS
PD13299	AURKA	cna	gain	BASIS
PD13771	AURKA	cna	gain	BASIS
PD22355	AURKA	cna	gain	BASIS
PD23562	AURKA	cna	gain	BASIS
PD23574	AURKA	cna	gain	BASIS
PD23578	AURKA	cna	gain	BASIS
PD24186	AURKA	cna	gain	BASIS
PD24202	AURKA	cna	hetloss	BASIS

PD24206	AURKA	cna	gain	BASIS
PD3905	AURKA	cna	gain	BASIS
PD4005	AURKA	cna	hetloss	BASIS
PD4006	AURKA	cna	gain	BASIS
PD4107	AURKA	cna	hetloss	BASIS
PD4826	AURKA	cna	gain	BASIS
PD5935	AURKA	cna	gain	BASIS
PD5945	AURKA	cna	amp	BASIS
PD5948	AURKA	cna	gain	BASIS
PD6406	AURKA	cna	gain	BASIS
PD7067	AURKA	cna	gain	BASIS
PD7215	AURKA	cna	amp	BASIS
PD8621	AURKA	cna	gain	BASIS
PD8980	AURKA	cna	gain	BASIS
PD9004	AURKA	cna	gain	BASIS
PD9585	AURKA	cna	hetloss	BASIS
PD9702	AURKA	cna	gain	BASIS
TCGA-AN-A0XU	AXIN1	cna	hetloss	TCGA
TCGA-AO-A0JL	AXIN1	cna	gain	TCGA
TCGA-BH-A0AW	AXIN1	cna	gain	TCGA
TCGA-BH-A0C0	AXIN1	cna	gain	TCGA
TCGA-C8-A12L	AXIN1	cna	gain	TCGA
TCGA-E2-A1L7	AXIN1	cna	hetloss	TCGA
TCGA-E9-A1NC	AXIN1	cna	hetloss	TCGA
TCGA-LL-A5YP	AXIN1	cna	gain	TCGA
MB-0346	AXIN1	cna	hetloss	METABRIC
MB-5465	AXIN1	cna	hetloss	METABRIC
MB-6271	AXIN1	cna	gain	METABRIC
MB-7032	AXIN1	cna	gain	METABRIC
MB-0420	AXIN1	cna	hetloss	METABRIC
PD10014	AXIN1	cna	hetloss	BASIS
PD11327	AXIN1	cna	gain	BASIS
PD11742	AXIN1	cna	gain	BASIS
PD13296	AXIN1	cna	gain	BASIS
PD13297	AXIN1	cna	hetloss	BASIS
PD13299	AXIN1	cna	gain	BASIS
PD22355	AXIN1	cna	hetloss	BASIS
PD23578	AXIN1	cna	hetloss	BASIS
PD24202	AXIN1	cna	hetloss	BASIS
PD24206	AXIN1	cna	gain	BASIS
PD24337	AXIN1	cna	gain	BASIS
PD3890	AXIN1	cna	hetloss	BASIS
PD3905	AXIN1	cna	gain	BASIS
PD4005	AXIN1	cna	gain	BASIS
PD4006	AXIN1	cna	gain	BASIS
PD4107	AXIN1	cna	gain	BASIS
PD4826	AXIN1	cna	gain	BASIS

PD4967	AXIN1	cna	gain	BASIS
PD5930	AXIN1	cna	gain	BASIS
PD5935	AXIN1	cna	gain	BASIS
PD5945	AXIN1	cna	gain	BASIS
PD5948	AXIN1	cna	gain	BASIS
PD6406	AXIN1	cna	gain	BASIS
PD6413	AXIN1	cna	gain	BASIS
PD7067	AXIN1	cna	gain	BASIS
PD7215	AXIN1	cna	amp	BASIS
PD8621	AXIN1	cna	gain	BASIS
PD9004	AXIN1	cna	gain	BASIS
PD9585	AXIN1	cna	hetloss	BASIS
PD9702	AXIN1	cna	gain	BASIS
TCGA-A2-A25B	CDKN1B	cna	hetloss	TCGA
TCGA-AN-A0XU	CDKN1B	cna	hetloss	TCGA
TCGA-AO-A0JL	CDKN1B	cna	hetloss	TCGA
TCGA-BH-A0C0	CDKN1B	cna	hetloss	TCGA
TCGA-BH-A18R	CDKN1B	cna	gain	TCGA
TCGA-BH-A1FU	CDKN1B	cna	hetloss	TCGA
TCGA-C8-A12L	CDKN1B	cna	gain	TCGA
TCGA-D8-A27M	CDKN1B	cna	gain	TCGA
TCGA-LL-A5YP	CDKN1B	cna	hetloss	TCGA
MB-0346	CDKN1B	cna	hetloss	METABRIC
MB-5070	CDKN1B	cna	amp	METABRIC
MB-5107	CDKN1B	cna	hetloss	METABRIC
MB-6060	CDKN1B	cna	gain	METABRIC
MB-6098	CDKN1B	cna	gain	METABRIC
MB-7038	CDKN1B	cna	hetloss	METABRIC
MB-7048	CDKN1B	cna	gain	METABRIC
P-0009557-T01-IM5	CDKN1B	cna	homdel	MSK-IMPACT
PD10014	CDKN1B	cna	hetloss	BASIS
PD11327	CDKN1B	cna	gain	BASIS
PD11742	CDKN1B	cna	gain	BASIS
PD13296	CDKN1B	cna	hetloss	BASIS
PD13297	CDKN1B	cna	hetloss	BASIS
PD13299	CDKN1B	cna	gain	BASIS
PD13771	CDKN1B	cna	hetloss	BASIS
PD23574	CDKN1B	cna	gain	BASIS
PD24186	CDKN1B	cna	gain	BASIS
PD24202	CDKN1B	cna	hetloss	BASIS
PD3905	CDKN1B	cna	gain	BASIS
PD4005	CDKN1B	cna	hetloss	BASIS
PD4006	CDKN1B	cna	gain	BASIS
PD4107	CDKN1B	cna	gain	BASIS
PD4826	CDKN1B	cna	hetloss	BASIS
PD4967	CDKN1B	cna	hetloss	BASIS
PD5930	CDKN1B	cna	gain	BASIS

PD5935	CDKN1B	cna	gain	BASIS
PD5945	CDKN1B	cna	amp	BASIS
PD6406	CDKN1B	cna	amp	BASIS
PD6731	CDKN1B	cna	hetloss	BASIS
PD7215	CDKN1B	cna	gain	BASIS
PD8621	CDKN1B	cna	gain	BASIS
PD8980	CDKN1B	cna	hetloss	BASIS
PD9004	CDKN1B	cna	gain	BASIS
PD9702	CDKN1B	cna	gain	BASIS
TCGA-A2-A25B	CHD4	cna	hetloss	TCGA
TCGA-AN-A0XU	CHD4	cna	gain	TCGA
TCGA-AO-A0JL	CHD4	cna	gain	TCGA
TCGA-BH-A0C0	CHD4	cna	hetloss	TCGA
TCGA-BH-A18R	CHD4	cna	gain	TCGA
TCGA-BH-A1FU	CHD4	cna	hetloss	TCGA
TCGA-C8-A12L	CHD4	cna	gain	TCGA
TCGA-D8-A27M	CHD4	cna	gain	TCGA
TCGA-E9-A1NC	CHD4	cna	gain	TCGA
TCGA-LL-A5YP	CHD4	cna	gain	TCGA
MB-0346	CHD4	cna	hetloss	METABRIC
MB-2827	CHD4	cna	amp	METABRIC
MB-5070	CHD4	cna	amp	METABRIC
MB-5107	CHD4	cna	hetloss	METABRIC
MB-6098	CHD4	cna	gain	METABRIC
MB-7048	CHD4	cna	gain	METABRIC
MTS-T0064	CHD4	cna	amp	METABRIC
PD10014	CHD4	cna	hetloss	BASIS
PD11742	CHD4	cna	gain	BASIS
PD13299	CHD4	cna	gain	BASIS
PD13771	CHD4	cna	hetloss	BASIS
PD23574	CHD4	cna	gain	BASIS
PD23578	CHD4	cna	gain	BASIS
PD24186	CHD4	cna	gain	BASIS
PD24206	CHD4	cna	hetloss	BASIS
PD24337	CHD4	cna	amp	BASIS
PD3890	CHD4	cna	gain	BASIS
PD3905	CHD4	cna	gain	BASIS
PD4005	CHD4	cna	gain	BASIS
PD4006	CHD4	cna	amp	BASIS
PD4107	CHD4	cna	gain	BASIS
PD4826	CHD4	cna	hetloss	BASIS
PD4967	CHD4	cna	hetloss	BASIS
PD5930	CHD4	cna	gain	BASIS
PD5935	CHD4	cna	gain	BASIS
PD5945	CHD4	cna	amp	BASIS
PD6406	CHD4	cna	gain	BASIS
PD6731	CHD4	cna	hetloss	BASIS

PD7215	CHD4	cna	gain	BASIS
PD8621	CHD4	cna	gain	BASIS
PD8980	CHD4	cna	hetloss	BASIS
PD9004	CHD4	cna	gain	BASIS
PD9702	CHD4	cna	gain	BASIS
TCGA-AN-A0XU	DKC1	cna	hetloss	TCGA
TCGA-BH-A0AW	DKC1	cna	gain	TCGA
TCGA-BH-A0C0	DKC1	cna	gain	TCGA
TCGA-BH-A1FU	DKC1	cna	hetloss	TCGA
TCGA-C8-A12L	DKC1	cna	gain	TCGA
TCGA-E2-A1L7	DKC1	cna	hetloss	TCGA
TCGA-E9-A1NC	DKC1	cna	gain	TCGA
TCGA-LL-A5YP	DKC1	cna	gain	TCGA
MB-0346	DKC1	cna	hetloss	METABRIC
MB-2827	DKC1	cna	hetloss	METABRIC
MB-5465	DKC1	cna	hetloss	METABRIC
MB-6060	DKC1	cna	gain	METABRIC
MB-6098	DKC1	cna	hetloss	METABRIC
MB-7038	DKC1	cna	gain	METABRIC
MTS-T0064	DKC1	cna	hetloss	METABRIC
PD10014	DKC1	cna	hetloss	BASIS
PD13296	DKC1	cna	gain	BASIS
PD13299	DKC1	cna	amp	BASIS
PD13771	DKC1	cna	hetloss	BASIS
PD14442	DKC1	cna	gain	BASIS
PD23562	DKC1	cna	hetloss	BASIS
PD23574	DKC1	cna	gain	BASIS
PD23578	DKC1	cna	hetloss	BASIS
PD24202	DKC1	cna	hetloss	BASIS
PD24206	DKC1	cna	gain	BASIS
PD24337	DKC1	cna	gain	BASIS
PD3890	DKC1	cna	hetloss	BASIS
PD3905	DKC1	cna	gain	BASIS
PD4005	DKC1	cna	gain	BASIS
PD4006	DKC1	cna	gain	BASIS
PD4107	DKC1	cna	gain	BASIS
PD4826	DKC1	cna	gain	BASIS
PD4967	DKC1	cna	gain	BASIS
PD5930	DKC1	cna	gain	BASIS
PD5935	DKC1	cna	gain	BASIS
PD5945	DKC1	cna	amp	BASIS
PD5948	DKC1	cna	gain	BASIS
PD6406	DKC1	cna	gain	BASIS
PD6413	DKC1	cna	gain	BASIS
PD7215	DKC1	cna	amp	BASIS
PD8621	DKC1	cna	amp	BASIS
PD8980	DKC1	cna	gain	BASIS

PD9004	DKC1	cna	gain	BASIS
TCGA-AN-A0XU	DNAH12	cna	hetloss	TCGA
TCGA-BH-A0C0	DNAH12	cna	hetloss	TCGA
TCGA-BH-A18R	DNAH12	cna	homdel	TCGA
TCGA-BH-A1FU	DNAH12	cna	hetloss	TCGA
TCGA-C8-A12L	DNAH12	cna	hetloss	TCGA
TCGA-D8-A27M	DNAH12	cna	hetloss	TCGA
TCGA-E2-A1L7	DNAH12	cna	hetloss	TCGA
TCGA-E9-A1NC	DNAH12	cna	hetloss	TCGA
TCGA-LL-A5YP	DNAH12	cna	hetloss	TCGA
MB-0346	DNAH12	cna	hetloss	METABRIC
MB-2827	DNAH12	cna	hetloss	METABRIC
MB-5465	DNAH12	cna	hetloss	METABRIC
MB-6098	DNAH12	cna	hetloss	METABRIC
MB-7048	DNAH12	cna	hetloss	METABRIC
MB-0420	DNAH12	cna	hetloss	METABRIC
MTS-T0064	DNAH12	cna	hetloss	METABRIC
PD10014	DNAH12	cna	hetloss	BASIS
PD11327	DNAH12	cna	homdel	BASIS
PD13296	DNAH12	cna	hetloss	BASIS
PD13297	DNAH12	cna	hetloss	BASIS
PD13299	DNAH12	cna	gain	BASIS
PD13771	DNAH12	cna	hetloss	BASIS
PD22355	DNAH12	cna	hetloss	BASIS
PD23561	DNAH12	cna	hetloss	BASIS
PD23578	DNAH12	cna	hetloss	BASIS
PD24202	DNAH12	cna	hetloss	BASIS
PD24206	DNAH12	cna	hetloss	BASIS
PD24337	DNAH12	cna	hetloss	BASIS
PD3890	DNAH12	cna	hetloss	BASIS
PD3905	DNAH12	cna	gain	BASIS
PD4005	DNAH12	cna	hetloss	BASIS
PD4107	DNAH12	cna	gain	BASIS
PD4826	DNAH12	cna	gain	BASIS
PD5945	DNAH12	cna	gain	BASIS
PD5948	DNAH12	cna	hetloss	BASIS
PD6406	DNAH12	cna	hetloss	BASIS
PD6413	DNAH12	cna	hetloss	BASIS
PD6731	DNAH12	cna	hetloss	BASIS
PD7067	DNAH12	cna	gain	BASIS
PD8980	DNAH12	cna	hetloss	BASIS
PD9004	DNAH12	cna	gain	BASIS
PD9585	DNAH12	cna	hetloss	BASIS
PD9702	DNAH12	cna	amp	BASIS
TCGA-A2-A25B	FGF23	cna	hetloss	TCGA
TCGA-AN-A0XU	FGF23	cna	gain	TCGA
TCGA-AO-A0JL	FGF23	cna	gain	TCGA

TCGA-BH-A0C0	FGF23	cna	hetloss	TCGA
TCGA-BH-A18R	FGF23	cna	gain	TCGA
TCGA-BH-A1FU	FGF23	cna	hetloss	TCGA
TCGA-C8-A12L	FGF23	cna	gain	TCGA
TCGA-D8-A27M	FGF23	cna	gain	TCGA
TCGA-E9-A1NC	FGF23	cna	gain	TCGA
TCGA-LL-A5YP	FGF23	cna	gain	TCGA
MB-0346	FGF23	cna	hetloss	METABRIC
MB-2827	FGF23	cna	amp	METABRIC
MB-5070	FGF23	cna	amp	METABRIC
MB-6098	FGF23	cna	gain	METABRIC
MB-7048	FGF23	cna	gain	METABRIC
MB-0420	FGF23	cna	gain	METABRIC
MTS-T0064	FGF23	cna	amp	METABRIC
PD10014	FGF23	cna	hetloss	BASIS
PD11327	FGF23	cna	gain	BASIS
PD11742	FGF23	cna	gain	BASIS
PD13299	FGF23	cna	gain	BASIS
PD13771	FGF23	cna	hetloss	BASIS
PD23562	FGF23	cna	gain	BASIS
PD23574	FGF23	cna	gain	BASIS
PD23578	FGF23	cna	gain	BASIS
PD24186	FGF23	cna	gain	BASIS
PD24206	FGF23	cna	gain	BASIS
PD24337	FGF23	cna	gain	BASIS
PD3890	FGF23	cna	gain	BASIS
PD3905	FGF23	cna	gain	BASIS
PD4005	FGF23	cna	gain	BASIS
PD4006	FGF23	cna	amp	BASIS
PD4107	FGF23	cna	gain	BASIS
PD4826	FGF23	cna	hetloss	BASIS
PD5930	FGF23	cna	gain	BASIS
PD5935	FGF23	cna	gain	BASIS
PD5945	FGF23	cna	amp	BASIS
PD6406	FGF23	cna	gain	BASIS
PD6731	FGF23	cna	hetloss	BASIS
PD7215	FGF23	cna	gain	BASIS
PD8621	FGF23	cna	gain	BASIS
PD9004	FGF23	cna	gain	BASIS
PD9702	FGF23	cna	gain	BASIS
TCGA-A2-A25B	FGF6	cna	hetloss	TCGA
TCGA-AN-A0XU	FGF6	cna	gain	TCGA
TCGA-AO-A0JL	FGF6	cna	gain	TCGA
TCGA-BH-A0C0	FGF6	cna	hetloss	TCGA
TCGA-BH-A18R	FGF6	cna	gain	TCGA
TCGA-BH-A1FU	FGF6	cna	hetloss	TCGA
TCGA-C8-A12L	FGF6	cna	gain	TCGA

TCGA-D8-A27M	FGF6	cna	gain	TCGA
TCGA-E9-A1NC	FGF6	cna	gain	TCGA
TCGA-LL-A5YP	FGF6	cna	gain	TCGA
MB-0346	FGF6	cna	hetloss	METABRIC
MB-2827	FGF6	cna	amp	METABRIC
MB-5070	FGF6	cna	amp	METABRIC
MB-6098	FGF6	cna	gain	METABRIC
MB-7048	FGF6	cna	gain	METABRIC
MB-0420	FGF6	cna	gain	METABRIC
MTS-T0064	FGF6	cna	amp	METABRIC
PD10014	FGF6	cna	hetloss	BASIS
PD11327	FGF6	cna	gain	BASIS
PD11742	FGF6	cna	gain	BASIS
PD13299	FGF6	cna	gain	BASIS
PD13771	FGF6	cna	hetloss	BASIS
PD23562	FGF6	cna	gain	BASIS
PD23574	FGF6	cna	gain	BASIS
PD23578	FGF6	cna	gain	BASIS
PD24186	FGF6	cna	gain	BASIS
PD24206	FGF6	cna	gain	BASIS
PD24337	FGF6	cna	gain	BASIS
PD3890	FGF6	cna	gain	BASIS
PD3905	FGF6	cna	gain	BASIS
PD4005	FGF6	cna	gain	BASIS
PD4006	FGF6	cna	amp	BASIS
PD4107	FGF6	cna	gain	BASIS
PD4826	FGF6	cna	hetloss	BASIS
PD5930	FGF6	cna	gain	BASIS
PD5935	FGF6	cna	gain	BASIS
PD5945	FGF6	cna	amp	BASIS
PD6406	FGF6	cna	gain	BASIS
PD6731	FGF6	cna	hetloss	BASIS
PD7215	FGF6	cna	gain	BASIS
PD8621	FGF6	cna	gain	BASIS
PD9004	FGF6	cna	gain	BASIS
PD9702	FGF6	cna	gain	BASIS
TCGA-A2-A25B	FYN	cna	hetloss	TCGA
TCGA-AN-A0XU	FYN	cna	amp	TCGA
TCGA-AO-A0JL	FYN	cna	hetloss	TCGA
TCGA-BH-A0AW	FYN	cna	amp	TCGA
TCGA-BH-A18R	FYN	cna	gain	TCGA
TCGA-C8-A12L	FYN	cna	gain	TCGA
TCGA-D8-A27M	FYN	cna	hetloss	TCGA
TCGA-E2-A1L7	FYN	cna	gain	TCGA
TCGA-LL-A5YP	FYN	cna	gain	TCGA
MB-2827	FYN	cna	amp	METABRIC
MB-5107	FYN	cna	hetloss	METABRIC

MB-6098	FYN	cna	hetloss	METABRIC
MB-7032	FYN	cna	hetloss	METABRIC
MB-7038	FYN	cna	gain	METABRIC
MB-7048	FYN	cna	hetloss	METABRIC
MB-0420	FYN	cna	gain	METABRIC
MTS-T0064	FYN	cna	amp	METABRIC
PD11327	FYN	cna	gain	BASIS
PD13296	FYN	cna	gain	BASIS
PD13299	FYN	cna	gain	BASIS
PD13771	FYN	cna	hetloss	BASIS
PD22355	FYN	cna	gain	BASIS
PD23562	FYN	cna	gain	BASIS
PD23574	FYN	cna	gain	BASIS
PD24186	FYN	cna	gain	BASIS
PD24206	FYN	cna	gain	BASIS
PD24337	FYN	cna	hetloss	BASIS
PD3905	FYN	cna	gain	BASIS
PD4006	FYN	cna	gain	BASIS
PD4107	FYN	cna	hetloss	BASIS
PD4826	FYN	cna	gain	BASIS
PD5930	FYN	cna	hetloss	BASIS
PD5935	FYN	cna	gain	BASIS
PD5945	FYN	cna	amp	BASIS
PD6406	FYN	cna	hetloss	BASIS
PD6731	FYN	cna	hetloss	BASIS
PD7067	FYN	cna	gain	BASIS
PD7215	FYN	cna	gain	BASIS
PD8621	FYN	cna	gain	BASIS
PD8980	FYN	cna	gain	BASIS
PD9004	FYN	cna	gain	BASIS
PD9585	FYN	cna	hetloss	BASIS
PD9702	FYN	cna	gain	BASIS
TCGA-A2-A25B	GNA13	cna	amp	TCGA
TCGA-AN-A0XU	GNA13	cna	gain	TCGA
TCGA-AO-A0JL	GNA13	cna	hetloss	TCGA
TCGA-BH-A0AW	GNA13	cna	amp	TCGA
TCGA-BH-A0C0	GNA13	cna	gain	TCGA
TCGA-C8-A12L	GNA13	cna	gain	TCGA
TCGA-D8-A27M	GNA13	cna	hetloss	TCGA
TCGA-EW-A1OX	GNA13	cna	amp	TCGA
TCGA-LL-A5YP	GNA13	cna	amp	TCGA
MB-0346	GNA13	cna	gain	METABRIC
MB-2827	GNA13	cna	hetloss	METABRIC
MB-5070	GNA13	cna	gain	METABRIC
MB-5107	GNA13	cna	amp	METABRIC
MB-5323	GNA13	cna	gain	METABRIC
MB-6060	GNA13	cna	amp	METABRIC

MB-6098	GNA13	cna	gain	METABRIC
MB-6271	GNA13	cna	hetloss	METABRIC
MB-7048	GNA13	cna	gain	METABRIC
MTS-T0064	GNA13	cna	gain	METABRIC
PD11327	GNA13	cna	gain	BASIS
PD11742	GNA13	cna	gain	BASIS
PD22355	GNA13	cna	gain	BASIS
PD23562	GNA13	cna	gain	BASIS
PD23574	GNA13	cna	gain	BASIS
PD24186	GNA13	cna	gain	BASIS
PD24206	GNA13	cna	hetloss	BASIS
PD24337	GNA13	cna	hetloss	BASIS
PD3890	GNA13	cna	gain	BASIS
PD3905	GNA13	cna	gain	BASIS
PD4006	GNA13	cna	amp	BASIS
PD4107	GNA13	cna	gain	BASIS
PD4967	GNA13	cna	homdel	BASIS
PD5930	GNA13	cna	gain	BASIS
PD5935	GNA13	cna	gain	BASIS
PD5945	GNA13	cna	amp	BASIS
PD5948	GNA13	cna	gain	BASIS
PD6731	GNA13	cna	hetloss	BASIS
PD7067	GNA13	cna	gain	BASIS
PD7215	GNA13	cna	gain	BASIS
PD8621	GNA13	cna	gain	BASIS
PD8980	GNA13	cna	gain	BASIS
PD9004	GNA13	cna	gain	BASIS
PD9702	GNA13	cna	gain	BASIS
TCGA-A2-A25B	MLF1	cna	gain	TCGA
TCGA-AN-A0XU	MLF1	cna	gain	TCGA
TCGA-BH-A0AW	MLF1	cna	gain	TCGA
TCGA-BH-A0C0	MLF1	cna	gain	TCGA
TCGA-BH-A18R	MLF1	cna	gain	TCGA
TCGA-BH-A1FU	MLF1	cna	hetloss	TCGA
TCGA-E2-A1L7	MLF1	cna	gain	TCGA
TCGA-E9-A1NC	MLF1	cna	gain	TCGA
TCGA-LL-A5YP	MLF1	cna	gain	TCGA
MB-0346	MLF1	cna	amp	METABRIC
MB-5070	MLF1	cna	amp	METABRIC
MB-5107	MLF1	cna	amp	METABRIC
MB-5465	MLF1	cna	amp	METABRIC
MB-6060	MLF1	cna	gain	METABRIC
MB-6098	MLF1	cna	gain	METABRIC
MB-7038	MLF1	cna	hetloss	METABRIC
MB-7048	MLF1	cna	hetloss	METABRIC
MB-0420	MLF1	cna	amp	METABRIC
MTS-T0064	MLF1	cna	gain	METABRIC

PD11327	MLF1	cna	gain	BASIS
PD13296	MLF1	cna	gain	BASIS
PD13299	MLF1	cna	gain	BASIS
PD13771	MLF1	cna	hetloss	BASIS
PD22355	MLF1	cna	gain	BASIS
PD23561	MLF1	cna	hetloss	BASIS
PD23562	MLF1	cna	amp	BASIS
PD23574	MLF1	cna	gain	BASIS
PD23578	MLF1	cna	gain	BASIS
PD24186	MLF1	cna	gain	BASIS
PD24202	MLF1	cna	gain	BASIS
PD24206	MLF1	cna	gain	BASIS
PD3905	MLF1	cna	gain	BASIS
PD4107	MLF1	cna	gain	BASIS
PD5930	MLF1	cna	gain	BASIS
PD5935	MLF1	cna	gain	BASIS
PD5945	MLF1	cna	amp	BASIS
PD5948	MLF1	cna	gain	BASIS
PD6731	MLF1	cna	gain	BASIS
PD7067	MLF1	cna	gain	BASIS
PD7215	MLF1	cna	gain	BASIS
PD8621	MLF1	cna	gain	BASIS
PD8980	MLF1	cna	gain	BASIS
PD9702	MLF1	cna	amp	BASIS
TCGA-A2-A25B	MLLT11	cna	gain	TCGA
TCGA-AN-A0XU	MLLT11	cna	gain	TCGA
TCGA-AO-A0JL	MLLT11	cna	gain	TCGA
TCGA-BH-A0AW	MLLT11	cna	gain	TCGA
TCGA-BH-A0C0	MLLT11	cna	hetloss	TCGA
TCGA-BH-A18R	MLLT11	cna	gain	TCGA
TCGA-BH-A1FU	MLLT11	cna	gain	TCGA
TCGA-C8-A12L	MLLT11	cna	amp	TCGA
TCGA-E2-A1L7	MLLT11	cna	gain	TCGA
TCGA-EW-A1OX	MLLT11	cna	gain	TCGA
TCGA-LL-A5YP	MLLT11	cna	amp	TCGA
MB-2827	MLLT11	cna	amp	METABRIC
MB-5070	MLLT11	cna	gain	METABRIC
MB-6098	MLLT11	cna	amp	METABRIC
MB-7032	MLLT11	cna	gain	METABRIC
MB-7048	MLLT11	cna	hetloss	METABRIC
MB-0420	MLLT11	cna	gain	METABRIC
PD10014	MLLT11	cna	gain	BASIS
PD11327	MLLT11	cna	gain	BASIS
PD13297	MLLT11	cna	gain	BASIS
PD13299	MLLT11	cna	gain	BASIS
PD14442	MLLT11	cna	gain	BASIS
PD22355	MLLT11	cna	gain	BASIS

PD23562	MLLT11	cna	amp	BASIS
PD23574	MLLT11	cna	gain	BASIS
PD24186	MLLT11	cna	gain	BASIS
PD24206	MLLT11	cna	gain	BASIS
PD3905	MLLT11	cna	gain	BASIS
PD4005	MLLT11	cna	gain	BASIS
PD4006	MLLT11	cna	amp	BASIS
PD4107	MLLT11	cna	gain	BASIS
PD4826	MLLT11	cna	gain	BASIS
PD4967	MLLT11	cna	hetloss	BASIS
PD5930	MLLT11	cna	amp	BASIS
PD5935	MLLT11	cna	gain	BASIS
PD5945	MLLT11	cna	amp	BASIS
PD5948	MLLT11	cna	gain	BASIS
PD6413	MLLT11	cna	gain	BASIS
PD7067	MLLT11	cna	amp	BASIS
PD7215	MLLT11	cna	gain	BASIS
PD8621	MLLT11	cna	amp	BASIS
PD9004	MLLT11	cna	amp	BASIS
PD9702	MLLT11	cna	amp	BASIS
TCGA-A2-A25B	MYB	cna	hetloss	TCGA
TCGA-AN-A0XU	MYB	cna	amp	TCGA
TCGA-AO-A0JL	MYB	cna	hetloss	TCGA
TCGA-BH-A0AW	MYB	cna	amp	TCGA
TCGA-BH-A0C0	MYB	cna	gain	TCGA
TCGA-BH-A18R	MYB	cna	gain	TCGA
TCGA-C8-A12L	MYB	cna	gain	TCGA
TCGA-D8-A27M	MYB	cna	hetloss	TCGA
MB-5070	MYB	cna	gain	METABRIC
MB-6098	MYB	cna	hetloss	METABRIC
MB-7032	MYB	cna	hetloss	METABRIC
MB-7038	MYB	cna	amp	METABRIC
MB-7048	MYB	cna	hetloss	METABRIC
MB-0420	MYB	cna	hetloss	METABRIC
PD11327	MYB	cna	gain	BASIS
PD13296	MYB	cna	gain	BASIS
PD13297	MYB	cna	gain	BASIS
PD13299	MYB	cna	gain	BASIS
PD13771	MYB	cna	hetloss	BASIS
PD22355	MYB	cna	gain	BASIS
PD23562	MYB	cna	gain	BASIS
PD23574	MYB	cna	gain	BASIS
PD23578	MYB	cna	gain	BASIS
PD24186	MYB	cna	gain	BASIS
PD24202	MYB	cna	gain	BASIS
PD3890	MYB	cna	gain	BASIS
PD3905	MYB	cna	gain	BASIS

PD4005	MYB	cna	hetloss	BASIS
PD4006	MYB	cna	gain	BASIS
PD4107	MYB	cna	amp	BASIS
PD4826	MYB	cna	gain	BASIS
PD5935	MYB	cna	gain	BASIS
PD5945	MYB	cna	amp	BASIS
PD5948	MYB	cna	gain	BASIS
PD6406	MYB	cna	hetloss	BASIS
PD6731	MYB	cna	hetloss	BASIS
PD7067	MYB	cna	amp	BASIS
PD7215	MYB	cna	gain	BASIS
PD8621	MYB	cna	gain	BASIS
PD8980	MYB	cna	gain	BASIS
PD9004	MYB	cna	gain	BASIS
PD9585	MYB	cna	hetloss	BASIS
PD9702	MYB	cna	gain	BASIS
TCGA-AN-A0XU	NR3C1	cna	hetloss	TCGA
TCGA-AO-A0JL	NR3C1	cna	hetloss	TCGA
TCGA-BH-A0C0	NR3C1	cna	hetloss	TCGA
TCGA-BH-A1FU	NR3C1	cna	hetloss	TCGA
TCGA-C8-A12L	NR3C1	cna	hetloss	TCGA
TCGA-D8-A27M	NR3C1	cna	hetloss	TCGA
TCGA-E2-A1L7	NR3C1	cna	hetloss	TCGA
TCGA-E9-A1NC	NR3C1	cna	hetloss	TCGA
TCGA-LL-A5YP	NR3C1	cna	hetloss	TCGA
MB-0346	NR3C1	cna	gain	METABRIC
MB-2827	NR3C1	cna	hetloss	METABRIC
MB-5070	NR3C1	cna	hetloss	METABRIC
MB-5465	NR3C1	cna	hetloss	METABRIC
MB-6098	NR3C1	cna	hetloss	METABRIC
MB-7038	NR3C1	cna	hetloss	METABRIC
MB-0420	NR3C1	cna	hetloss	METABRIC
PD10014	NR3C1	cna	hetloss	BASIS
PD11327	NR3C1	cna	gain	BASIS
PD13296	NR3C1	cna	hetloss	BASIS
PD13297	NR3C1	cna	hetloss	BASIS
PD13771	NR3C1	cna	gain	BASIS
PD22355	NR3C1	cna	hetloss	BASIS
PD23562	NR3C1	cna	gain	BASIS
PD23578	NR3C1	cna	hetloss	BASIS
PD24186	NR3C1	cna	gain	BASIS
PD24202	NR3C1	cna	hetloss	BASIS
PD24206	NR3C1	cna	gain	BASIS
PD24337	NR3C1	cna	hetloss	BASIS
PD3890	NR3C1	cna	hetloss	BASIS
PD3905	NR3C1	cna	gain	BASIS
PD4005	NR3C1	cna	hetloss	BASIS

PD4006	NR3C1	cna	hetloss	BASIS
PD4107	NR3C1	cna	hetloss	BASIS
PD4826	NR3C1	cna	gain	BASIS
PD5935	NR3C1	cna	gain	BASIS
PD5945	NR3C1	cna	gain	BASIS
PD5948	NR3C1	cna	hetloss	BASIS
PD6406	NR3C1	cna	hetloss	BASIS
PD6413	NR3C1	cna	hetloss	BASIS
PD7067	NR3C1	cna	gain	BASIS
PD8980	NR3C1	cna	hetloss	BASIS
PD9004	NR3C1	cna	gain	BASIS
PD9585	NR3C1	cna	hetloss	BASIS
TCGA-A2-A25B	PIK3C2G	cna	hetloss	TCGA
TCGA-AN-A0XU	PIK3C2G	cna	gain	TCGA
TCGA-AO-A0JL	PIK3C2G	cna	hetloss	TCGA
TCGA-BH-A0C0	PIK3C2G	cna	hetloss	TCGA
TCGA-BH-A18R	PIK3C2G	cna	gain	TCGA
TCGA-BH-A1FU	PIK3C2G	cna	hetloss	TCGA
TCGA-C8-A12L	PIK3C2G	cna	gain	TCGA
TCGA-D8-A27M	PIK3C2G	cna	gain	TCGA
TCGA-E2-A1L7	PIK3C2G	cna	hetloss	TCGA
TCGA-LL-A5YP	PIK3C2G	cna	hetloss	TCGA
MB-0346	PIK3C2G	cna	hetloss	METABRIC
MB-2827	PIK3C2G	cna	hetloss	METABRIC
MB-5070	PIK3C2G	cna	gain	METABRIC
MB-5465	PIK3C2G	cna	gain	METABRIC
MB-6098	PIK3C2G	cna	gain	METABRIC
MB-7048	PIK3C2G	cna	gain	METABRIC
P-0009557-T01-IM5	PIK3C2G	cna	homdel	MSK-IMPACT
PD10014	PIK3C2G	cna	gain	BASIS
PD11327	PIK3C2G	cna	gain	BASIS
PD11742	PIK3C2G	cna	gain	BASIS
PD13296	PIK3C2G	cna	hetloss	BASIS
PD13297	PIK3C2G	cna	hetloss	BASIS
PD13299	PIK3C2G	cna	gain	BASIS
PD13771	PIK3C2G	cna	hetloss	BASIS
PD23574	PIK3C2G	cna	gain	BASIS
PD24186	PIK3C2G	cna	gain	BASIS
PD3890	PIK3C2G	cna	hetloss	BASIS
PD3905	PIK3C2G	cna	gain	BASIS
PD4005	PIK3C2G	cna	hetloss	BASIS
PD4006	PIK3C2G	cna	gain	BASIS
PD4107	PIK3C2G	cna	gain	BASIS
PD4826	PIK3C2G	cna	hetloss	BASIS
PD5930	PIK3C2G	cna	gain	BASIS
PD5935	PIK3C2G	cna	gain	BASIS
PD5945	PIK3C2G	cna	amp	BASIS

PD5948	PIK3C2G	cna	hetloss	BASIS
PD6406	PIK3C2G	cna	gain	BASIS
PD6731	PIK3C2G	cna	hetloss	BASIS
PD7215	PIK3C2G	cna	gain	BASIS
PD8621	PIK3C2G	cna	gain	BASIS
PD8980	PIK3C2G	cna	hetloss	BASIS
PD9004	PIK3C2G	cna	amp	BASIS
PD9702	PIK3C2G	cna	gain	BASIS
TCGA-AN-A0XU	RB1	cna	amp	TCGA
TCGA-AO-A0JL	RB1	cna	hetloss	TCGA
TCGA-BH-A0AW	RB1	cna	hetloss	TCGA
TCGA-BH-A0C0	RB1	cna	hetloss	TCGA
TCGA-BH-A1FU	RB1	cna	hetloss	TCGA
TCGA-C8-A12L	RB1	cna	gain	TCGA
TCGA-LL-A5YP	RB1	cna	homdel	TCGA
MB-0346	RB1	cna	hetloss	METABRIC
MB-5107	RB1	cna	hetloss	METABRIC
MB-6098	RB1	cna	hetloss	METABRIC
MB-7032	RB1	cna	hetloss	METABRIC
MB-7038	RB1	cna	hetloss	METABRIC
MB-7048	RB1	cna	hetloss	METABRIC
MB-0420	RB1	cna	hetloss	METABRIC
MTS-T0064	RB1	cna	hetloss	METABRIC
PD10014	RB1	cna	hetloss	BASIS
PD11327	RB1	cna	hetloss	BASIS
PD11742	RB1	cna	hetloss	BASIS
PD13296	RB1	cna	hetloss	BASIS
PD13297	RB1	cna	hetloss	BASIS
PD13771	RB1	cna	hetloss	BASIS
PD14442	RB1	cna	hetloss	BASIS
PD22355	RB1	cna	hetloss	BASIS
PD23574	RB1	cna	hetloss	BASIS
PD24202	RB1	cna	hetloss	BASIS
PD24206	RB1	cna	gain	BASIS
PD3890	RB1	cna	hetloss	BASIS
PD3905	RB1	cna	gain	BASIS
PD4005	RB1	cna	homdel	BASIS
PD4006	RB1	cna	hetloss	BASIS
PD4826	RB1	cna	hetloss	BASIS
PD4967	RB1	cna	homdel	BASIS
PD5945	RB1	cna	gain	BASIS
PD5948	RB1	cna	gain	BASIS
PD6406	RB1	cna	hetloss	BASIS
PD6413	RB1	cna	hetloss	BASIS
PD6731	RB1	cna	hetloss	BASIS
PD7215	RB1	cna	gain	BASIS
PD8621	RB1	cna	hetloss	BASIS

PD8980	RB1	cna	hetloss	BASIS
PD9004	RB1	cna	hetloss	BASIS
PD9585	RB1	cna	hetloss	BASIS
PD9702	RB1	cna	gain	BASIS
TCGA-A2-A25B	RPS6KB1	cna	amp	TCGA
TCGA-AN-A0XU	RPS6KB1	cna	gain	TCGA
TCGA-AO-A0JL	RPS6KB1	cna	hetloss	TCGA
TCGA-BH-A0AW	RPS6KB1	cna	amp	TCGA
TCGA-BH-A0C0	RPS6KB1	cna	gain	TCGA
TCGA-BH-A18R	RPS6KB1	cna	amp	TCGA
TCGA-C8-A12L	RPS6KB1	cna	gain	TCGA
TCGA-D8-A27M	RPS6KB1	cna	hetloss	TCGA
TCGA-EW-A1OX	RPS6KB1	cna	amp	TCGA
TCGA-LL-A5YP	RPS6KB1	cna	amp	TCGA
MB-0346	RPS6KB1	cna	gain	METABRIC
MB-2827	RPS6KB1	cna	hetloss	METABRIC
MB-5070	RPS6KB1	cna	hetloss	METABRIC
MB-5107	RPS6KB1	cna	amp	METABRIC
MB-5323	RPS6KB1	cna	gain	METABRIC
MB-6060	RPS6KB1	cna	amp	METABRIC
MB-6098	RPS6KB1	cna	hetloss	METABRIC
MB-6271	RPS6KB1	cna	hetloss	METABRIC
MB-7048	RPS6KB1	cna	gain	METABRIC
MTS-T0064	RPS6KB1	cna	gain	METABRIC
PD11327	RPS6KB1	cna	gain	BASIS
PD11742	RPS6KB1	cna	gain	BASIS
PD13299	RPS6KB1	cna	gain	BASIS
PD22355	RPS6KB1	cna	gain	BASIS
PD23561	RPS6KB1	cna	gain	BASIS
PD23562	RPS6KB1	cna	gain	BASIS
PD23574	RPS6KB1	cna	gain	BASIS
PD24186	RPS6KB1	cna	gain	BASIS
PD24206	RPS6KB1	cna	hetloss	BASIS
PD24337	RPS6KB1	cna	hetloss	BASIS
PD3905	RPS6KB1	cna	gain	BASIS
PD4006	RPS6KB1	cna	amp	BASIS
PD4967	RPS6KB1	cna	homdel	BASIS
PD5930	RPS6KB1	cna	gain	BASIS
PD5935	RPS6KB1	cna	amp	BASIS
PD5945	RPS6KB1	cna	amp	BASIS
PD5948	RPS6KB1	cna	gain	BASIS
PD6731	RPS6KB1	cna	hetloss	BASIS
PD7067	RPS6KB1	cna	gain	BASIS
PD7215	RPS6KB1	cna	gain	BASIS
PD8980	RPS6KB1	cna	gain	BASIS
PD9004	RPS6KB1	cna	gain	BASIS
PD9702	RPS6KB1	cna	gain	BASIS

TCGA-A2-A25B	SKP2	cna	amp	TCGA
TCGA-AN-A0XU	SKP2	cna	gain	TCGA
TCGA-AO-A0JL	SKP2	cna	gain	TCGA
TCGA-BH-A0AW	SKP2	cna	gain	TCGA
TCGA-BH-A0C0	SKP2	cna	gain	TCGA
TCGA-BH-A1FU	SKP2	cna	amp	TCGA
TCGA-E9-A1NC	SKP2	cna	hetloss	TCGA
TCGA-LL-A5YP	SKP2	cna	amp	TCGA
MB-0346	SKP2	cna	gain	METABRIC
MB-5070	SKP2	cna	gain	METABRIC
MB-5107	SKP2	cna	gain	METABRIC
MB-6060	SKP2	cna	gain	METABRIC
MB-6098	SKP2	cna	amp	METABRIC
MB-7038	SKP2	cna	gain	METABRIC
MB-7048	SKP2	cna	hetloss	METABRIC
MB-0420	SKP2	cna	gain	METABRIC
PD11327	SKP2	cna	gain	BASIS
PD13296	SKP2	cna	hetloss	BASIS
PD13299	SKP2	cna	gain	BASIS
PD13771	SKP2	cna	hetloss	BASIS
PD23562	SKP2	cna	gain	BASIS
PD23574	SKP2	cna	gain	BASIS
PD24186	SKP2	cna	gain	BASIS
PD24206	SKP2	cna	gain	BASIS
PD24337	SKP2	cna	hetloss	BASIS
PD3890	SKP2	cna	gain	BASIS
PD3905	SKP2	cna	gain	BASIS
PD4005	SKP2	cna	hetloss	BASIS
PD4006	SKP2	cna	gain	BASIS
PD4107	SKP2	cna	gain	BASIS
PD4826	SKP2	cna	gain	BASIS
PD5930	SKP2	cna	gain	BASIS
PD5935	SKP2	cna	hetloss	BASIS
PD5945	SKP2	cna	amp	BASIS
PD5948	SKP2	cna	gain	BASIS
PD6406	SKP2	cna	gain	BASIS
PD6413	SKP2	cna	gain	BASIS
PD6731	SKP2	cna	gain	BASIS
PD7067	SKP2	cna	gain	BASIS
PD7215	SKP2	cna	amp	BASIS
PD8980	SKP2	cna	gain	BASIS
PD9004	SKP2	cna	gain	BASIS
PD9585	SKP2	cna	gain	BASIS
TCGA-A2-A25B	SMC3	cna	hetloss	TCGA
TCGA-AN-A0XU	SMC3	cna	hetloss	TCGA
TCGA-AO-A0JL	SMC3	cna	hetloss	TCGA
TCGA-BH-A18R	SMC3	cna	gain	TCGA

TCGA-C8-A12L	SMC3	cna	hetloss	TCGA
TCGA-D8-A27M	SMC3	cna	hetloss	TCGA
TCGA-E2-A1L7	SMC3	cna	hetloss	TCGA
TCGA-E9-A1NC	SMC3	cna	hetloss	TCGA
TCGA-LL-A5YP	SMC3	cna	gain	TCGA
MB-0346	SMC3	cna	hetloss	METABRIC
MB-2827	SMC3	cna	hetloss	METABRIC
MB-5070	SMC3	cna	hetloss	METABRIC
MB-5465	SMC3	cna	amp	METABRIC
MB-6098	SMC3	cna	hetloss	METABRIC
MB-7038	SMC3	cna	hetloss	METABRIC
MB-7048	SMC3	cna	hetloss	METABRIC
MB-0420	SMC3	cna	hetloss	METABRIC
MTS-T0064	SMC3	cna	gain	METABRIC
PD10014	SMC3	cna	hetloss	BASIS
PD11327	SMC3	cna	gain	BASIS
PD11742	SMC3	cna	hetloss	BASIS
PD13296	SMC3	cna	hetloss	BASIS
PD22355	SMC3	cna	hetloss	BASIS
PD23562	SMC3	cna	gain	BASIS
PD23574	SMC3	cna	gain	BASIS
PD23578	SMC3	cna	hetloss	BASIS
PD24206	SMC3	cna	hetloss	BASIS
PD24337	SMC3	cna	hetloss	BASIS
PD3905	SMC3	cna	gain	BASIS
PD4005	SMC3	cna	hetloss	BASIS
PD4006	SMC3	cna	gain	BASIS
PD4107	SMC3	cna	gain	BASIS
PD5935	SMC3	cna	gain	BASIS
PD5945	SMC3	cna	gain	BASIS
PD5948	SMC3	cna	gain	BASIS
PD6406	SMC3	cna	hetloss	BASIS
PD6731	SMC3	cna	hetloss	BASIS
PD7067	SMC3	cna	gain	BASIS
PD7215	SMC3	cna	gain	BASIS
PD8621	SMC3	cna	gain	BASIS
PD8980	SMC3	cna	hetloss	BASIS
PD9585	SMC3	cna	hetloss	BASIS
PD9702	SMC3	cna	gain	BASIS
TCGA-A2-A25B	TRIM24	cna	gain	TCGA
TCGA-AO-A0JL	TRIM24	cna	gain	TCGA
TCGA-BH-A0AW	TRIM24	cna	gain	TCGA
TCGA-D8-A27M	TRIM24	cna	gain	TCGA
TCGA-E2-A1L7	TRIM24	cna	hetloss	TCGA
TCGA-E9-A1NC	TRIM24	cna	gain	TCGA
TCGA-LL-A5YP	TRIM24	cna	gain	TCGA
MB-0346	TRIM24	cna	gain	METABRIC

MB-2827	TRIM24	cna	hetloss	METABRIC
MB-5070	TRIM24	cna	hetloss	METABRIC
MB-5107	TRIM24	cna	gain	METABRIC
MB-7038	TRIM24	cna	gain	METABRIC
MB-7048	TRIM24	cna	gain	METABRIC
PD10014	TRIM24	cna	gain	BASIS
PD13296	TRIM24	cna	hetloss	BASIS
PD13297	TRIM24	cna	gain	BASIS
PD13299	TRIM24	cna	gain	BASIS
PD22355	TRIM24	cna	hetloss	BASIS
PD23562	TRIM24	cna	gain	BASIS
PD23574	TRIM24	cna	gain	BASIS
PD23578	TRIM24	cna	hetloss	BASIS
PD24186	TRIM24	cna	amp	BASIS
PD24202	TRIM24	cna	gain	BASIS
PD24206	TRIM24	cna	hetloss	BASIS
PD24337	TRIM24	cna	gain	BASIS
PD3890	TRIM24	cna	hetloss	BASIS
PD3905	TRIM24	cna	gain	BASIS
PD4006	TRIM24	cna	gain	BASIS
PD4107	TRIM24	cna	gain	BASIS
PD4826	TRIM24	cna	gain	BASIS
PD4967	TRIM24	cna	hetloss	BASIS
PD5930	TRIM24	cna	gain	BASIS
PD5935	TRIM24	cna	gain	BASIS
PD5945	TRIM24	cna	amp	BASIS
PD5948	TRIM24	cna	gain	BASIS
PD6406	TRIM24	cna	gain	BASIS
PD6731	TRIM24	cna	hetloss	BASIS
PD7067	TRIM24	cna	gain	BASIS
PD7215	TRIM24	cna	gain	BASIS
PD8621	TRIM24	cna	gain	BASIS
PD9004	TRIM24	cna	gain	BASIS
PD9585	TRIM24	cna	gain	BASIS
PD9702	TRIM24	cna	gain	BASIS
TCGA-AN-A0XU	WDR90	cna	hetloss	TCGA
TCGA-AO-A0JL	WDR90	cna	gain	TCGA
TCGA-BH-A0AW	WDR90	cna	gain	TCGA
TCGA-BH-A0C0	WDR90	cna	gain	TCGA
TCGA-C8-A12L	WDR90	cna	gain	TCGA
TCGA-E2-A1L7	WDR90	cna	hetloss	TCGA
TCGA-E9-A1NC	WDR90	cna	hetloss	TCGA
TCGA-LL-A5YP	WDR90	cna	gain	TCGA
MB-0346	WDR90	cna	hetloss	METABRIC
MB-5465	WDR90	cna	hetloss	METABRIC
MB-6271	WDR90	cna	gain	METABRIC
MB-7032	WDR90	cna	gain	METABRIC

MB-0420	WDR90	cna	hetloss	METABRIC
PD10014	WDR90	cna	hetloss	BASIS
PD11327	WDR90	cna	gain	BASIS
PD11742	WDR90	cna	gain	BASIS
PD13296	WDR90	cna	gain	BASIS
PD13297	WDR90	cna	hetloss	BASIS
PD13299	WDR90	cna	gain	BASIS
PD22355	WDR90	cna	hetloss	BASIS
PD23578	WDR90	cna	hetloss	BASIS
PD24202	WDR90	cna	hetloss	BASIS
PD24206	WDR90	cna	gain	BASIS
PD24337	WDR90	cna	gain	BASIS
PD3890	WDR90	cna	hetloss	BASIS
PD3905	WDR90	cna	gain	BASIS
PD4005	WDR90	cna	gain	BASIS
PD4006	WDR90	cna	gain	BASIS
PD4107	WDR90	cna	gain	BASIS
PD4826	WDR90	cna	gain	BASIS
PD4967	WDR90	cna	gain	BASIS
PD5930	WDR90	cna	gain	BASIS
PD5935	WDR90	cna	gain	BASIS
PD5945	WDR90	cna	gain	BASIS
PD5948	WDR90	cna	gain	BASIS
PD6406	WDR90	cna	gain	BASIS
PD6413	WDR90	cna	gain	BASIS
PD7067	WDR90	cna	gain	BASIS
PD7215	WDR90	cna	amp	BASIS
PD8621	WDR90	cna	gain	BASIS
PD9004	WDR90	cna	gain	BASIS
PD9585	WDR90	cna	hetloss	BASIS
PD9702	WDR90	cna	gain	BASIS
TCGA-A2-A25B	ZNF384	cna	hetloss	TCGA
TCGA-AN-A0XU	ZNF384	cna	gain	TCGA
TCGA-AO-A0JL	ZNF384	cna	gain	TCGA
TCGA-BH-A0C0	ZNF384	cna	hetloss	TCGA
TCGA-BH-A18R	ZNF384	cna	gain	TCGA
TCGA-BH-A1FU	ZNF384	cna	hetloss	TCGA
TCGA-C8-A12L	ZNF384	cna	gain	TCGA
TCGA-D8-A27M	ZNF384	cna	gain	TCGA
TCGA-E9-A1NC	ZNF384	cna	gain	TCGA
TCGA-LL-A5YP	ZNF384	cna	gain	TCGA
MB-0346	ZNF384	cna	hetloss	METABRIC
MB-2827	ZNF384	cna	amp	METABRIC
MB-5070	ZNF384	cna	amp	METABRIC
MB-5107	ZNF384	cna	hetloss	METABRIC
MB-6098	ZNF384	cna	gain	METABRIC
MB-7048	ZNF384	cna	gain	METABRIC

MTS-T0064	ZNF384	cna	amp	METABRIC
PD10014	ZNF384	cna	hetloss	BASIS
PD11742	ZNF384	cna	gain	BASIS
PD13299	ZNF384	cna	gain	BASIS
PD13771	ZNF384	cna	hetloss	BASIS
PD23574	ZNF384	cna	gain	BASIS
PD23578	ZNF384	cna	gain	BASIS
PD24186	ZNF384	cna	gain	BASIS
PD24206	ZNF384	cna	hetloss	BASIS
PD24337	ZNF384	cna	amp	BASIS
PD3890	ZNF384	cna	gain	BASIS
PD3905	ZNF384	cna	gain	BASIS
PD4005	ZNF384	cna	gain	BASIS
PD4006	ZNF384	cna	amp	BASIS
PD4107	ZNF384	cna	gain	BASIS
PD4826	ZNF384	cna	hetloss	BASIS
PD4967	ZNF384	cna	hetloss	BASIS
PD5930	ZNF384	cna	gain	BASIS
PD5935	ZNF384	cna	gain	BASIS
PD5945	ZNF384	cna	amp	BASIS
PD6406	ZNF384	cna	gain	BASIS
PD6731	ZNF384	cna	hetloss	BASIS
PD7215	ZNF384	cna	gain	BASIS
PD8621	ZNF384	cna	gain	BASIS
PD8980	ZNF384	cna	hetloss	BASIS
PD9004	ZNF384	cna	gain	BASIS
PD9702	ZNF384	cna	gain	BASIS
TCGA-A2-A25B	AHNAK2	cna	gain	TCGA
TCGA-AN-A0XU	AHNAK2	cna	gain	TCGA
TCGA-AO-A0JL	AHNAK2	cna	hetloss	TCGA
TCGA-BH-A0AW	AHNAK2	cna	hetloss	TCGA
TCGA-BH-A1FU	AHNAK2	cna	gain	TCGA
TCGA-C8-A12L	AHNAK2	cna	gain	TCGA
TCGA-D8-A27M	AHNAK2	cna	hetloss	TCGA
TCGA-E2-A1L7	AHNAK2	cna	gain	TCGA
TCGA-E9-A1NC	AHNAK2	cna	hetloss	TCGA
TCGA-LL-A5YP	AHNAK2	cna	hetloss	TCGA
MB-0346	AHNAK2	cna	gain	METABRIC
MB-2827	AHNAK2	cna	hetloss	METABRIC
MB-5070	AHNAK2	cna	hetloss	METABRIC
MB-5107	AHNAK2	cna	hetloss	METABRIC
MB-6060	AHNAK2	cna	gain	METABRIC
MB-6098	AHNAK2	cna	gain	METABRIC
MB-7048	AHNAK2	cna	gain	METABRIC
MTS-T0064	AHNAK2	cna	hetloss	METABRIC
PD10014	AHNAK2	cna	hetloss	BASIS
PD11742	AHNAK2	cna	hetloss	BASIS

PD13296	AHNAK2	cna	gain	BASIS
PD13297	AHNAK2	cna	hetloss	BASIS
PD13771	AHNAK2	cna	hetloss	BASIS
PD22355	AHNAK2	cna	hetloss	BASIS
PD23574	AHNAK2	cna	gain	BASIS
PD23578	AHNAK2	cna	hetloss	BASIS
PD24202	AHNAK2	cna	hetloss	BASIS
PD24206	AHNAK2	cna	gain	BASIS
PD24337	AHNAK2	cna	hetloss	BASIS
PD3890	AHNAK2	cna	hetloss	BASIS
PD3905	AHNAK2	cna	gain	BASIS
PD4006	AHNAK2	cna	gain	BASIS
PD4826	AHNAK2	cna	gain	BASIS
PD4967	AHNAK2	cna	hetloss	BASIS
PD5945	AHNAK2	cna	gain	BASIS
PD5948	AHNAK2	cna	gain	BASIS
PD6406	AHNAK2	cna	hetloss	BASIS
PD6413	AHNAK2	cna	hetloss	BASIS
PD7067	AHNAK2	cna	gain	BASIS
PD8621	AHNAK2	cna	gain	BASIS
PD8980	AHNAK2	cna	hetloss	BASIS
PD9702	AHNAK2	cna	gain	BASIS
TCGA-A2-A25B	AK9	cna	hetloss	TCGA
TCGA-AN-A0XU	AK9	cna	amp	TCGA
TCGA-AO-A0JL	AK9	cna	hetloss	TCGA
TCGA-BH-A0AW	AK9	cna	gain	TCGA
TCGA-BH-A18R	AK9	cna	gain	TCGA
TCGA-C8-A12L	AK9	cna	gain	TCGA
TCGA-D8-A27M	AK9	cna	hetloss	TCGA
TCGA-E2-A1L7	AK9	cna	gain	TCGA
TCGA-LL-A5YP	AK9	cna	gain	TCGA
MB-2827	AK9	cna	amp	METABRIC
MB-5107	AK9	cna	hetloss	METABRIC
MB-6098	AK9	cna	hetloss	METABRIC
MB-7032	AK9	cna	hetloss	METABRIC
MB-7038	AK9	cna	gain	METABRIC
MB-7048	AK9	cna	hetloss	METABRIC
MTS-T0064	AK9	cna	amp	METABRIC
PD11327	AK9	cna	gain	BASIS
PD13296	AK9	cna	gain	BASIS
PD13299	AK9	cna	gain	BASIS
PD13771	AK9	cna	hetloss	BASIS
PD22355	AK9	cna	gain	BASIS
PD23562	AK9	cna	gain	BASIS
PD23574	AK9	cna	gain	BASIS
PD24186	AK9	cna	gain	BASIS
PD24206	AK9	cna	gain	BASIS

PD24337	AK9	cna	hetloss	BASIS
PD3905	AK9	cna	gain	BASIS
PD4006	AK9	cna	gain	BASIS
PD4107	AK9	cna	hetloss	BASIS
PD4826	AK9	cna	gain	BASIS
PD5930	AK9	cna	hetloss	BASIS
PD5935	AK9	cna	gain	BASIS
PD5945	AK9	cna	amp	BASIS
PD6406	AK9	cna	hetloss	BASIS
PD6731	AK9	cna	hetloss	BASIS
PD7067	AK9	cna	gain	BASIS
PD7215	AK9	cna	gain	BASIS
PD8621	AK9	cna	gain	BASIS
PD8980	AK9	cna	gain	BASIS
PD9004	AK9	cna	gain	BASIS
PD9585	AK9	cna	hetloss	BASIS
PD9702	AK9	cna	gain	BASIS
TCGA-A2-A25B	AKT1	cna	gain	TCGA
TCGA-AN-A0XU	AKT1	cna	gain	TCGA
TCGA-AO-A0JL	AKT1	cna	hetloss	TCGA
TCGA-BH-A0AW	AKT1	cna	hetloss	TCGA
TCGA-BH-A1FU	AKT1	cna	gain	TCGA
TCGA-C8-A12L	AKT1	cna	gain	TCGA
TCGA-D8-A27M	AKT1	cna	hetloss	TCGA
TCGA-E2-A1L7	AKT1	cna	gain	TCGA
TCGA-E9-A1NC	AKT1	cna	hetloss	TCGA
TCGA-LL-A5YP	AKT1	cna	hetloss	TCGA
MB-0346	AKT1	cna	gain	METABRIC
MB-2827	AKT1	cna	hetloss	METABRIC
MB-5070	AKT1	cna	hetloss	METABRIC
MB-5107	AKT1	cna	hetloss	METABRIC
MB-6060	AKT1	cna	gain	METABRIC
MB-6098	AKT1	cna	gain	METABRIC
MB-7048	AKT1	cna	gain	METABRIC
MTS-T0064	AKT1	cna	hetloss	METABRIC
PD10014	AKT1	cna	hetloss	BASIS
PD11742	AKT1	cna	hetloss	BASIS
PD13296	AKT1	cna	gain	BASIS
PD13297	AKT1	cna	hetloss	BASIS
PD13771	AKT1	cna	hetloss	BASIS
PD22355	AKT1	cna	hetloss	BASIS
PD23574	AKT1	cna	gain	BASIS
PD23578	AKT1	cna	hetloss	BASIS
PD24202	AKT1	cna	hetloss	BASIS
PD24206	AKT1	cna	gain	BASIS
PD24337	AKT1	cna	hetloss	BASIS
PD3890	AKT1	cna	hetloss	BASIS

PD3905	AKT1	cna	gain	BASIS
PD4006	AKT1	cna	gain	BASIS
PD4826	AKT1	cna	gain	BASIS
PD4967	AKT1	cna	hetloss	BASIS
PD5945	AKT1	cna	gain	BASIS
PD5948	AKT1	cna	gain	BASIS
PD6406	AKT1	cna	hetloss	BASIS
PD6413	AKT1	cna	hetloss	BASIS
PD7067	AKT1	cna	gain	BASIS
PD8621	AKT1	cna	gain	BASIS
PD8980	AKT1	cna	hetloss	BASIS
PD9702	AKT1	cna	gain	BASIS
TCGA-A2-A25B	ARID1B	cna	hetloss	TCGA
TCGA-AN-A0XU	ARID1B	cna	gain	TCGA
TCGA-AO-A0JL	ARID1B	cna	hetloss	TCGA
TCGA-BH-A0AW	ARID1B	cna	amp	TCGA
TCGA-BH-A0C0	ARID1B	cna	gain	TCGA
TCGA-C8-A12L	ARID1B	cna	gain	TCGA
TCGA-D8-A27M	ARID1B	cna	hetloss	TCGA
TCGA-E9-A1NC	ARID1B	cna	hetloss	TCGA
MB-2827	ARID1B	cna	hetloss	METABRIC
MB-5070	ARID1B	cna	gain	METABRIC
MB-5107	ARID1B	cna	hetloss	METABRIC
MB-6098	ARID1B	cna	hetloss	METABRIC
MB-7048	ARID1B	cna	hetloss	METABRIC
MB-0420	ARID1B	cna	hetloss	METABRIC
MTS-T0064	ARID1B	cna	hetloss	METABRIC
PD10014	ARID1B	cna	hetloss	BASIS
PD11327	ARID1B	cna	gain	BASIS
PD13296	ARID1B	cna	homdel	BASIS
PD13297	ARID1B	cna	hetloss	BASIS
PD13299	ARID1B	cna	gain	BASIS
PD22355	ARID1B	cna	gain	BASIS
PD23562	ARID1B	cna	gain	BASIS
PD23574	ARID1B	cna	gain	BASIS
PD24186	ARID1B	cna	gain	BASIS
PD3890	ARID1B	cna	hetloss	BASIS
PD3905	ARID1B	cna	gain	BASIS
PD4005	ARID1B	cna	hetloss	BASIS
PD4006	ARID1B	cna	gain	BASIS
PD4107	ARID1B	cna	gain	BASIS
PD4826	ARID1B	cna	gain	BASIS
PD4967	ARID1B	cna	hetloss	BASIS
PD5935	ARID1B	cna	amp	BASIS
PD5945	ARID1B	cna	gain	BASIS
PD6406	ARID1B	cna	hetloss	BASIS
PD6413	ARID1B	cna	hetloss	BASIS

PD6731	ARID1B	cna	hetloss	BASIS
PD7067	ARID1B	cna	amp	BASIS
PD7215	ARID1B	cna	gain	BASIS
PD8980	ARID1B	cna	gain	BASIS
PD9004	ARID1B	cna	gain	BASIS
PD9585	ARID1B	cna	hetloss	BASIS
PD9702	ARID1B	cna	gain	BASIS
TCGA-A2-A25B	ATN1	cna	hetloss	TCGA
TCGA-AN-A0XU	ATN1	cna	gain	TCGA
TCGA-AO-A0JL	ATN1	cna	gain	TCGA
TCGA-BH-A0C0	ATN1	cna	hetloss	TCGA
TCGA-BH-A18R	ATN1	cna	gain	TCGA
TCGA-BH-A1FU	ATN1	cna	hetloss	TCGA
TCGA-C8-A12L	ATN1	cna	gain	TCGA
TCGA-D8-A27M	ATN1	cna	gain	TCGA
TCGA-E9-A1NC	ATN1	cna	gain	TCGA
TCGA-LL-A5YP	ATN1	cna	gain	TCGA
MB-0346	ATN1	cna	hetloss	METABRIC
MB-5070	ATN1	cna	amp	METABRIC
MB-5107	ATN1	cna	hetloss	METABRIC
MB-6098	ATN1	cna	gain	METABRIC
MB-7048	ATN1	cna	gain	METABRIC
MTS-T0064	ATN1	cna	amp	METABRIC
PD10014	ATN1	cna	hetloss	BASIS
PD11742	ATN1	cna	gain	BASIS
PD13299	ATN1	cna	gain	BASIS
PD13771	ATN1	cna	hetloss	BASIS
PD23574	ATN1	cna	gain	BASIS
PD23578	ATN1	cna	gain	BASIS
PD24186	ATN1	cna	gain	BASIS
PD24206	ATN1	cna	hetloss	BASIS
PD24337	ATN1	cna	amp	BASIS
PD3890	ATN1	cna	gain	BASIS
PD3905	ATN1	cna	gain	BASIS
PD4005	ATN1	cna	hetloss	BASIS
PD4006	ATN1	cna	amp	BASIS
PD4107	ATN1	cna	gain	BASIS
PD4826	ATN1	cna	hetloss	BASIS
PD4967	ATN1	cna	hetloss	BASIS
PD5930	ATN1	cna	gain	BASIS
PD5935	ATN1	cna	gain	BASIS
PD5945	ATN1	cna	amp	BASIS
PD6406	ATN1	cna	gain	BASIS
PD6731	ATN1	cna	hetloss	BASIS
PD7215	ATN1	cna	gain	BASIS
PD8621	ATN1	cna	gain	BASIS
PD8980	ATN1	cna	hetloss	BASIS

PD9004	ATN1	cna	gain	BASIS
PD9702	ATN1	cna	gain	BASIS
TCGA-AN-A0XU	B2M	cna	hetloss	TCGA
TCGA-AO-A0JL	B2M	cna	hetloss	TCGA
TCGA-BH-A0AW	B2M	cna	hetloss	TCGA
TCGA-BH-A0C0	B2M	cna	hetloss	TCGA
TCGA-BH-A18R	B2M	cna	gain	TCGA
TCGA-C8-A12L	B2M	cna	hetloss	TCGA
TCGA-D8-A27M	B2M	cna	hetloss	TCGA
TCGA-E2-A1L7	B2M	cna	hetloss	TCGA
TCGA-E9-A1NC	B2M	cna	hetloss	TCGA
TCGA-LL-A5YP	B2M	cna	hetloss	TCGA
MB-0346	B2M	cna	gain	METABRIC
MB-2827	B2M	cna	hetloss	METABRIC
MB-5107	B2M	cna	gain	METABRIC
MB-5465	B2M	cna	hetloss	METABRIC
MB-6060	B2M	cna	hetloss	METABRIC
MB-6098	B2M	cna	hetloss	METABRIC
MB-7048	B2M	cna	hetloss	METABRIC
MB-0420	B2M	cna	hetloss	METABRIC
PD10014	B2M	cna	hetloss	BASIS
PD11327	B2M	cna	hetloss	BASIS
PD11742	B2M	cna	hetloss	BASIS
PD13296	B2M	cna	homdel	BASIS
PD13297	B2M	cna	hetloss	BASIS
PD13299	B2M	cna	hetloss	BASIS
PD22355	B2M	cna	hetloss	BASIS
PD23574	B2M	cna	hetloss	BASIS
PD23578	B2M	cna	hetloss	BASIS
PD24202	B2M	cna	hetloss	BASIS
PD24337	B2M	cna	hetloss	BASIS
PD3890	B2M	cna	hetloss	BASIS
PD3905	B2M	cna	gain	BASIS
PD4005	B2M	cna	homdel	BASIS
PD4967	B2M	cna	hetloss	BASIS
PD5930	B2M	cna	hetloss	BASIS
PD5948	B2M	cna	hetloss	BASIS
PD6406	B2M	cna	hetloss	BASIS
PD6413	B2M	cna	hetloss	BASIS
PD7067	B2M	cna	gain	BASIS
PD7215	B2M	cna	gain	BASIS
PD8980	B2M	cna	hetloss	BASIS
PD9585	B2M	cna	hetloss	BASIS
PD9702	B2M	cna	gain	BASIS
TCGA-AN-A0XU	BAP1	cna	hetloss	TCGA
TCGA-BH-A0C0	BAP1	cna	hetloss	TCGA
TCGA-BH-A18R	BAP1	cna	homdel	TCGA

TCGA-BH-A1FU	BAP1	cna	hetloss	TCGA
TCGA-C8-A12L	BAP1	cna	hetloss	TCGA
TCGA-D8-A27M	BAP1	cna	hetloss	TCGA
TCGA-E2-A1L7	BAP1	cna	hetloss	TCGA
TCGA-E9-A1NC	BAP1	cna	hetloss	TCGA
TCGA-LL-A5YP	BAP1	cna	hetloss	TCGA
MB-0346	BAP1	cna	hetloss	METABRIC
MB-2827	BAP1	cna	hetloss	METABRIC
MB-5465	BAP1	cna	hetloss	METABRIC
MB-6098	BAP1	cna	hetloss	METABRIC
MB-7038	BAP1	cna	hetloss	METABRIC
MB-7048	BAP1	cna	hetloss	METABRIC
MB-0420	BAP1	cna	hetloss	METABRIC
MTS-T0064	BAP1	cna	hetloss	METABRIC
PD10014	BAP1	cna	hetloss	BASIS
PD11327	BAP1	cna	hetloss	BASIS
PD13296	BAP1	cna	hetloss	BASIS
PD13297	BAP1	cna	hetloss	BASIS
PD13299	BAP1	cna	gain	BASIS
PD13771	BAP1	cna	hetloss	BASIS
PD14442	BAP1	cna	hetloss	BASIS
PD22355	BAP1	cna	hetloss	BASIS
PD23561	BAP1	cna	hetloss	BASIS
PD23578	BAP1	cna	hetloss	BASIS
PD24202	BAP1	cna	hetloss	BASIS
PD24206	BAP1	cna	hetloss	BASIS
PD24337	BAP1	cna	hetloss	BASIS
PD3890	BAP1	cna	hetloss	BASIS
PD3905	BAP1	cna	gain	BASIS
PD4005	BAP1	cna	hetloss	BASIS
PD4826	BAP1	cna	gain	BASIS
PD4967	BAP1	cna	hetloss	BASIS
PD5945	BAP1	cna	gain	BASIS
PD6406	BAP1	cna	hetloss	BASIS
PD6413	BAP1	cna	hetloss	BASIS
PD6731	BAP1	cna	hetloss	BASIS
PD7067	BAP1	cna	gain	BASIS
PD8980	BAP1	cna	hetloss	BASIS
PD9585	BAP1	cna	hetloss	BASIS
TCGA-A2-A25B	BCL11B	cna	hetloss	TCGA
TCGA-AN-A0XU	BCL11B	cna	hetloss	TCGA
TCGA-AO-A0JL	BCL11B	cna	hetloss	TCGA
TCGA-BH-A0AW	BCL11B	cna	hetloss	TCGA
TCGA-BH-A1FU	BCL11B	cna	gain	TCGA
TCGA-C8-A12L	BCL11B	cna	gain	TCGA
TCGA-D8-A27M	BCL11B	cna	hetloss	TCGA
TCGA-E2-A1L7	BCL11B	cna	gain	TCGA

TCGA-E9-A1NC	BCL11B	cna	hetloss	TCGA
TCGA-LL-A5YP	BCL11B	cna	hetloss	TCGA
MB-0346	BCL11B	cna	gain	METABRIC
MB-2827	BCL11B	cna	hetloss	METABRIC
MB-5070	BCL11B	cna	hetloss	METABRIC
MB-5107	BCL11B	cna	hetloss	METABRIC
MB-6098	BCL11B	cna	hetloss	METABRIC
MTS-T0064	BCL11B	cna	hetloss	METABRIC
PD10014	BCL11B	cna	hetloss	BASIS
PD11327	BCL11B	cna	amp	BASIS
PD11742	BCL11B	cna	hetloss	BASIS
PD13296	BCL11B	cna	gain	BASIS
PD13297	BCL11B	cna	hetloss	BASIS
PD13771	BCL11B	cna	hetloss	BASIS
PD22355	BCL11B	cna	hetloss	BASIS
PD23562	BCL11B	cna	gain	BASIS
PD23574	BCL11B	cna	gain	BASIS
PD23578	BCL11B	cna	hetloss	BASIS
PD24202	BCL11B	cna	hetloss	BASIS
PD24206	BCL11B	cna	gain	BASIS
PD24337	BCL11B	cna	hetloss	BASIS
PD3890	BCL11B	cna	hetloss	BASIS
PD3905	BCL11B	cna	gain	BASIS
PD4005	BCL11B	cna	hetloss	BASIS
PD4006	BCL11B	cna	gain	BASIS
PD4826	BCL11B	cna	gain	BASIS
PD5945	BCL11B	cna	gain	BASIS
PD5948	BCL11B	cna	gain	BASIS
PD6406	BCL11B	cna	hetloss	BASIS
PD6413	BCL11B	cna	hetloss	BASIS
PD7067	BCL11B	cna	gain	BASIS
PD8621	BCL11B	cna	gain	BASIS
PD8980	BCL11B	cna	hetloss	BASIS
PD9702	BCL11B	cna	gain	BASIS
TCGA-A2-A25B	BTK	cna	hetloss	TCGA
TCGA-AN-A0XU	BTK	cna	hetloss	TCGA
TCGA-AO-A0JL	BTK	cna	hetloss	TCGA
TCGA-BH-A0AW	BTK	cna	gain	TCGA
TCGA-C8-A12L	BTK	cna	hetloss	TCGA
TCGA-E2-A1L7	BTK	cna	hetloss	TCGA
TCGA-E9-A1NC	BTK	cna	hetloss	TCGA
MB-0346	BTK	cna	hetloss	METABRIC
MB-2827	BTK	cna	hetloss	METABRIC
MB-5465	BTK	cna	gain	METABRIC
MB-6060	BTK	cna	hetloss	METABRIC
MB-6098	BTK	cna	hetloss	METABRIC
MB-0420	BTK	cna	hetloss	METABRIC

PD10014	BTK	cna	hetloss	BASIS
PD11327	BTK	cna	hetloss	BASIS
PD13296	BTK	cna	hetloss	BASIS
PD13299	BTK	cna	amp	BASIS
PD13771	BTK	cna	gain	BASIS
PD14442	BTK	cna	gain	BASIS
PD22355	BTK	cna	gain	BASIS
PD23562	BTK	cna	hetloss	BASIS
PD23574	BTK	cna	gain	BASIS
PD23578	BTK	cna	hetloss	BASIS
PD24202	BTK	cna	hetloss	BASIS
PD24206	BTK	cna	gain	BASIS
PD3905	BTK	cna	amp	BASIS
PD4005	BTK	cna	hetloss	BASIS
PD4006	BTK	cna	gain	BASIS
PD4107	BTK	cna	gain	BASIS
PD4826	BTK	cna	gain	BASIS
PD4967	BTK	cna	gain	BASIS
PD5930	BTK	cna	gain	BASIS
PD5935	BTK	cna	gain	BASIS
PD5945	BTK	cna	gain	BASIS
PD5948	BTK	cna	gain	BASIS
PD6406	BTK	cna	gain	BASIS
PD6413	BTK	cna	gain	BASIS
PD7067	BTK	cna	gain	BASIS
PD7215	BTK	cna	gain	BASIS
PD8621	BTK	cna	amp	BASIS
PD8980	BTK	cna	hetloss	BASIS
PD9004	BTK	cna	gain	BASIS
TCGA-A2-A25B	CCND2	cna	hetloss	TCGA
TCGA-AN-A0XU	CCND2	cna	gain	TCGA
TCGA-AO-A0JL	CCND2	cna	gain	TCGA
TCGA-BH-A0C0	CCND2	cna	hetloss	TCGA
TCGA-BH-A18R	CCND2	cna	gain	TCGA
TCGA-BH-A1FU	CCND2	cna	hetloss	TCGA
TCGA-C8-A12L	CCND2	cna	gain	TCGA
TCGA-D8-A27M	CCND2	cna	gain	TCGA
TCGA-E9-A1NC	CCND2	cna	gain	TCGA
TCGA-LL-A5YP	CCND2	cna	gain	TCGA
MB-0346	CCND2	cna	hetloss	METABRIC
MB-2827	CCND2	cna	amp	METABRIC
MB-5070	CCND2	cna	amp	METABRIC
MB-6098	CCND2	cna	gain	METABRIC
MB-7048	CCND2	cna	gain	METABRIC
MB-0420	CCND2	cna	gain	METABRIC
MTS-T0064	CCND2	cna	amp	METABRIC
PD10014	CCND2	cna	hetloss	BASIS

PD11327	CCND2	cna	gain	BASIS
PD11742	CCND2	cna	gain	BASIS
PD13299	CCND2	cna	gain	BASIS
PD13771	CCND2	cna	hetloss	BASIS
PD23562	CCND2	cna	gain	BASIS
PD23574	CCND2	cna	gain	BASIS
PD23578	CCND2	cna	gain	BASIS
PD24186	CCND2	cna	gain	BASIS
PD24337	CCND2	cna	gain	BASIS
PD3890	CCND2	cna	gain	BASIS
PD3905	CCND2	cna	gain	BASIS
PD4005	CCND2	cna	gain	BASIS
PD4006	CCND2	cna	amp	BASIS
PD4107	CCND2	cna	gain	BASIS
PD4826	CCND2	cna	hetloss	BASIS
PD5930	CCND2	cna	gain	BASIS
PD5935	CCND2	cna	gain	BASIS
PD5945	CCND2	cna	amp	BASIS
PD6406	CCND2	cna	gain	BASIS
PD6731	CCND2	cna	hetloss	BASIS
PD7215	CCND2	cna	gain	BASIS
PD8621	CCND2	cna	gain	BASIS
PD9004	CCND2	cna	gain	BASIS
PD9702	CCND2	cna	gain	BASIS
TCGA-A2-A25B	CLTC	cna	amp	TCGA
TCGA-AN-A0XU	CLTC	cna	gain	TCGA
TCGA-AO-A0JL	CLTC	cna	hetloss	TCGA
TCGA-BH-A0AW	CLTC	cna	amp	TCGA
TCGA-BH-A0C0	CLTC	cna	gain	TCGA
TCGA-BH-A18R	CLTC	cna	amp	TCGA
TCGA-C8-A12L	CLTC	cna	gain	TCGA
TCGA-D8-A27M	CLTC	cna	hetloss	TCGA
TCGA-EW-A1OX	CLTC	cna	amp	TCGA
MB-0346	CLTC	cna	gain	METABRIC
MB-2827	CLTC	cna	hetloss	METABRIC
MB-5070	CLTC	cna	hetloss	METABRIC
MB-5107	CLTC	cna	amp	METABRIC
MB-5323	CLTC	cna	gain	METABRIC
MB-6060	CLTC	cna	amp	METABRIC
MB-6098	CLTC	cna	hetloss	METABRIC
MB-6271	CLTC	cna	hetloss	METABRIC
MB-7048	CLTC	cna	gain	METABRIC
MTS-T0064	CLTC	cna	gain	METABRIC
PD11327	CLTC	cna	gain	BASIS
PD11742	CLTC	cna	gain	BASIS
PD13299	CLTC	cna	gain	BASIS
PD22355	CLTC	cna	gain	BASIS

PD23561	CLTC	cna	gain	BASIS
PD23562	CLTC	cna	gain	BASIS
PD23574	CLTC	cna	gain	BASIS
PD24186	CLTC	cna	gain	BASIS
PD24337	CLTC	cna	hetloss	BASIS
PD3905	CLTC	cna	gain	BASIS
PD4006	CLTC	cna	amp	BASIS
PD4967	CLTC	cna	homdel	BASIS
PD5930	CLTC	cna	gain	BASIS
PD5935	CLTC	cna	amp	BASIS
PD5945	CLTC	cna	amp	BASIS
PD5948	CLTC	cna	gain	BASIS
PD6406	CLTC	cna	hetloss	BASIS
PD6731	CLTC	cna	hetloss	BASIS
PD7067	CLTC	cna	gain	BASIS
PD7215	CLTC	cna	gain	BASIS
PD8980	CLTC	cna	gain	BASIS
PD9004	CLTC	cna	gain	BASIS
PD9702	CLTC	cna	gain	BASIS
TCGA-A2-A25B	CREB3L2	cna	gain	TCGA
TCGA-AO-A0JL	CREB3L2	cna	gain	TCGA
TCGA-BH-A0AW	CREB3L2	cna	gain	TCGA
TCGA-D8-A27M	CREB3L2	cna	gain	TCGA
TCGA-E2-A1L7	CREB3L2	cna	hetloss	TCGA
TCGA-E9-A1NC	CREB3L2	cna	gain	TCGA
TCGA-LL-A5YP	CREB3L2	cna	gain	TCGA
MB-0346	CREB3L2	cna	gain	METABRIC
MB-2827	CREB3L2	cna	amp	METABRIC
MB-5107	CREB3L2	cna	gain	METABRIC
MB-7038	CREB3L2	cna	gain	METABRIC
MB-7048	CREB3L2	cna	gain	METABRIC
PD10014	CREB3L2	cna	gain	BASIS
PD11327	CREB3L2	cna	gain	BASIS
PD13296	CREB3L2	cna	hetloss	BASIS
PD13297	CREB3L2	cna	gain	BASIS
PD13299	CREB3L2	cna	gain	BASIS
PD22355	CREB3L2	cna	hetloss	BASIS
PD23562	CREB3L2	cna	gain	BASIS
PD23574	CREB3L2	cna	gain	BASIS
PD23578	CREB3L2	cna	hetloss	BASIS
PD24186	CREB3L2	cna	amp	BASIS
PD24202	CREB3L2	cna	gain	BASIS
PD24206	CREB3L2	cna	hetloss	BASIS
PD24337	CREB3L2	cna	gain	BASIS
PD3890	CREB3L2	cna	hetloss	BASIS
PD3905	CREB3L2	cna	gain	BASIS
PD4006	CREB3L2	cna	gain	BASIS

PD4107	CREB3L2	cna	gain	BASIS
PD4826	CREB3L2	cna	gain	BASIS
PD5930	CREB3L2	cna	gain	BASIS
PD5935	CREB3L2	cna	gain	BASIS
PD5945	CREB3L2	cna	amp	BASIS
PD5948	CREB3L2	cna	gain	BASIS
PD6406	CREB3L2	cna	gain	BASIS
PD6731	CREB3L2	cna	hetloss	BASIS
PD7067	CREB3L2	cna	gain	BASIS
PD7215	CREB3L2	cna	gain	BASIS
PD8621	CREB3L2	cna	gain	BASIS
PD9004	CREB3L2	cna	gain	BASIS
PD9585	CREB3L2	cna	gain	BASIS
PD9702	CREB3L2	cna	gain	BASIS
TCGA-A2-A25B	DDX10	cna	hetloss	TCGA
TCGA-AN-A0XU	DDX10	cna	gain	TCGA
TCGA-AO-A0JL	DDX10	cna	hetloss	TCGA
TCGA-BH-A0C0	DDX10	cna	gain	TCGA
TCGA-BH-A18R	DDX10	cna	homdel	TCGA
TCGA-D8-A27M	DDX10	cna	hetloss	TCGA
TCGA-E2-A1L7	DDX10	cna	hetloss	TCGA
TCGA-E9-A1NC	DDX10	cna	hetloss	TCGA
TCGA-EW-A1OX	DDX10	cna	homdel	TCGA
TCGA-LL-A5YP	DDX10	cna	hetloss	TCGA
MB-0346	DDX10	cna	hetloss	METABRIC
MB-5070	DDX10	cna	hetloss	METABRIC
MB-5107	DDX10	cna	hetloss	METABRIC
MB-6271	DDX10	cna	hetloss	METABRIC
MB-7032	DDX10	cna	hetloss	METABRIC
MB-7048	DDX10	cna	hetloss	METABRIC
PD11327	DDX10	cna	gain	BASIS
PD11742	DDX10	cna	hetloss	BASIS
PD13296	DDX10	cna	hetloss	BASIS
PD13299	DDX10	cna	gain	BASIS
PD13771	DDX10	cna	hetloss	BASIS
PD22355	DDX10	cna	hetloss	BASIS
PD23574	DDX10	cna	gain	BASIS
PD23578	DDX10	cna	hetloss	BASIS
PD24186	DDX10	cna	gain	BASIS
PD24206	DDX10	cna	gain	BASIS
PD24337	DDX10	cna	hetloss	BASIS
PD3905	DDX10	cna	gain	BASIS
PD4005	DDX10	cna	homdel	BASIS
PD4006	DDX10	cna	hetloss	BASIS
PD4107	DDX10	cna	hetloss	BASIS
PD4826	DDX10	cna	hetloss	BASIS
PD5935	DDX10	cna	gain	BASIS

PD5945	DDX10	cna	gain	BASIS
PD5948	DDX10	cna	gain	BASIS
PD6413	DDX10	cna	hetloss	BASIS
PD6731	DDX10	cna	hetloss	BASIS
PD7067	DDX10	cna	gain	BASIS
PD7215	DDX10	cna	gain	BASIS
PD9004	DDX10	cna	hetloss	BASIS
PD9585	DDX10	cna	gain	BASIS
PD9702	DDX10	cna	gain	BASIS
TCGA-A2-A25B	EGFL7	cna	gain	TCGA
TCGA-AN-A0XU	EGFL7	cna	hetloss	TCGA
TCGA-AO-A0JL	EGFL7	cna	hetloss	TCGA
TCGA-BH-A0C0	EGFL7	cna	gain	TCGA
TCGA-BH-A18R	EGFL7	cna	amp	TCGA
TCGA-C8-A12L	EGFL7	cna	gain	TCGA
TCGA-E2-A1L7	EGFL7	cna	hetloss	TCGA
TCGA-E9-A1NC	EGFL7	cna	hetloss	TCGA
TCGA-LL-A5YP	EGFL7	cna	hetloss	TCGA
MB-0346	EGFL7	cna	hetloss	METABRIC
MB-2827	EGFL7	cna	amp	METABRIC
MB-5070	EGFL7	cna	hetloss	METABRIC
MB-7038	EGFL7	cna	hetloss	METABRIC
MB-7048	EGFL7	cna	hetloss	METABRIC
MB-0420	EGFL7	cna	hetloss	METABRIC
P-0009557-T01-IM5	EGFL7	cna	homdel	MSK-IMPACT
PD10014	EGFL7	cna	gain	BASIS
PD11327	EGFL7	cna	gain	BASIS
PD11742	EGFL7	cna	gain	BASIS
PD13297	EGFL7	cna	hetloss	BASIS
PD13299	EGFL7	cna	gain	BASIS
PD22355	EGFL7	cna	gain	BASIS
PD24186	EGFL7	cna	gain	BASIS
PD24202	EGFL7	cna	hetloss	BASIS
PD24206	EGFL7	cna	gain	BASIS
PD24337	EGFL7	cna	hetloss	BASIS
PD3890	EGFL7	cna	hetloss	BASIS
PD3905	EGFL7	cna	gain	BASIS
PD4005	EGFL7	cna	hetloss	BASIS
PD4006	EGFL7	cna	gain	BASIS
PD4107	EGFL7	cna	hetloss	BASIS
PD4826	EGFL7	cna	gain	BASIS
PD5935	EGFL7	cna	hetloss	BASIS
PD5945	EGFL7	cna	gain	BASIS
PD5948	EGFL7	cna	gain	BASIS
PD6406	EGFL7	cna	hetloss	BASIS
PD6731	EGFL7	cna	hetloss	BASIS
PD7067	EGFL7	cna	gain	BASIS

PD7215	EGFL7	cna	gain	BASIS
PD8621	EGFL7	cna	gain	BASIS
PD8980	EGFL7	cna	hetloss	BASIS
PD9004	EGFL7	cna	hetloss	BASIS
TCGA-A2-A25B	EGR3	cna	hetloss	TCGA
TCGA-AN-A0XU	EGR3	cna	hetloss	TCGA
TCGA-AO-A0JL	EGR3	cna	hetloss	TCGA
TCGA-BH-A0C0	EGR3	cna	hetloss	TCGA
TCGA-BH-A1FU	EGR3	cna	homdel	TCGA
TCGA-C8-A12L	EGR3	cna	hetloss	TCGA
TCGA-D8-A27M	EGR3	cna	hetloss	TCGA
TCGA-E2-A1L7	EGR3	cna	hetloss	TCGA
TCGA-E9-A1NC	EGR3	cna	hetloss	TCGA
TCGA-LL-A5YP	EGR3	cna	hetloss	TCGA
MB-0346	EGR3	cna	hetloss	METABRIC
MB-2827	EGR3	cna	hetloss	METABRIC
MB-5070	EGR3	cna	hetloss	METABRIC
MB-5465	EGR3	cna	hetloss	METABRIC
MB-6060	EGR3	cna	hetloss	METABRIC
MB-6098	EGR3	cna	hetloss	METABRIC
MB-0420	EGR3	cna	hetloss	METABRIC
MTS-T0064	EGR3	cna	hetloss	METABRIC
PD10014	EGR3	cna	hetloss	BASIS
PD11327	EGR3	cna	homdel	BASIS
PD13296	EGR3	cna	hetloss	BASIS
PD13299	EGR3	cna	hetloss	BASIS
PD13771	EGR3	cna	hetloss	BASIS
PD14442	EGR3	cna	hetloss	BASIS
PD22355	EGR3	cna	hetloss	BASIS
PD23562	EGR3	cna	hetloss	BASIS
PD23574	EGR3	cna	hetloss	BASIS
PD23578	EGR3	cna	hetloss	BASIS
PD24186	EGR3	cna	gain	BASIS
PD24202	EGR3	cna	hetloss	BASIS
PD24337	EGR3	cna	hetloss	BASIS
PD3905	EGR3	cna	gain	BASIS
PD4005	EGR3	cna	hetloss	BASIS
PD4006	EGR3	cna	gain	BASIS
PD4826	EGR3	cna	hetloss	BASIS
PD5935	EGR3	cna	gain	BASIS
PD5945	EGR3	cna	gain	BASIS
PD6406	EGR3	cna	hetloss	BASIS
PD6413	EGR3	cna	hetloss	BASIS
PD7067	EGR3	cna	hetloss	BASIS
PD8980	EGR3	cna	hetloss	BASIS
PD9004	EGR3	cna	homdel	BASIS
TCGA-AN-A0XU	FLYWCH1	cna	gain	TCGA

TCGA-AO-A0JL	FLYWCH1	cna	gain	TCGA
TCGA-BH-A0AW	FLYWCH1	cna	gain	TCGA
TCGA-BH-A0C0	FLYWCH1	cna	gain	TCGA
TCGA-C8-A12L	FLYWCH1	cna	gain	TCGA
TCGA-E2-A1L7	FLYWCH1	cna	hetloss	TCGA
TCGA-E9-A1NC	FLYWCH1	cna	hetloss	TCGA
TCGA-LL-A5YP	FLYWCH1	cna	hetloss	TCGA
MB-0346	FLYWCH1	cna	hetloss	METABRIC
MB-5465	FLYWCH1	cna	hetloss	METABRIC
MB-6098	FLYWCH1	cna	hetloss	METABRIC
MB-6271	FLYWCH1	cna	gain	METABRIC
MB-7032	FLYWCH1	cna	gain	METABRIC
MB-7038	FLYWCH1	cna	hetloss	METABRIC
MB-0420	FLYWCH1	cna	hetloss	METABRIC
PD10014	FLYWCH1	cna	gain	BASIS
PD11327	FLYWCH1	cna	gain	BASIS
PD11742	FLYWCH1	cna	gain	BASIS
PD13296	FLYWCH1	cna	gain	BASIS
PD13297	FLYWCH1	cna	hetloss	BASIS
PD13299	FLYWCH1	cna	gain	BASIS
PD22355	FLYWCH1	cna	hetloss	BASIS
PD23578	FLYWCH1	cna	hetloss	BASIS
PD24202	FLYWCH1	cna	hetloss	BASIS
PD24337	FLYWCH1	cna	hetloss	BASIS
PD3905	FLYWCH1	cna	gain	BASIS
PD4005	FLYWCH1	cna	gain	BASIS
PD4006	FLYWCH1	cna	gain	BASIS
PD4107	FLYWCH1	cna	gain	BASIS
PD4826	FLYWCH1	cna	gain	BASIS
PD4967	FLYWCH1	cna	gain	BASIS
PD5930	FLYWCH1	cna	gain	BASIS
PD5935	FLYWCH1	cna	gain	BASIS
PD5945	FLYWCH1	cna	gain	BASIS
PD5948	FLYWCH1	cna	gain	BASIS
PD6413	FLYWCH1	cna	gain	BASIS
PD7067	FLYWCH1	cna	gain	BASIS
PD7215	FLYWCH1	cna	amp	BASIS
PD8621	FLYWCH1	cna	gain	BASIS
PD9004	FLYWCH1	cna	gain	BASIS
PD9585	FLYWCH1	cna	hetloss	BASIS
PD9702	FLYWCH1	cna	gain	BASIS
TCGA-AN-A0XU	FOXO1	cna	hetloss	TCGA
TCGA-AO-A0JL	FOXO1	cna	hetloss	TCGA
TCGA-BH-A0AW	FOXO1	cna	hetloss	TCGA
TCGA-BH-A0C0	FOXO1	cna	hetloss	TCGA
TCGA-BH-A1FU	FOXO1	cna	hetloss	TCGA
TCGA-C8-A12L	FOXO1	cna	hetloss	TCGA

TCGA-D8-A27M	FOXO1	cna	amp	TCGA
TCGA-EW-A1OX	FOXO1	cna	homdel	TCGA
TCGA-LL-A5YP	FOXO1	cna	homdel	TCGA
MB-0346	FOXO1	cna	hetloss	METABRIC
MB-2827	FOXO1	cna	hetloss	METABRIC
MB-5107	FOXO1	cna	hetloss	METABRIC
MB-6098	FOXO1	cna	hetloss	METABRIC
MB-7032	FOXO1	cna	hetloss	METABRIC
MB-7048	FOXO1	cna	hetloss	METABRIC
MB-0420	FOXO1	cna	hetloss	METABRIC
MTS-T0064	FOXO1	cna	hetloss	METABRIC
PD10014	FOXO1	cna	hetloss	BASIS
PD11742	FOXO1	cna	hetloss	BASIS
PD13296	FOXO1	cna	hetloss	BASIS
PD13297	FOXO1	cna	hetloss	BASIS
PD13771	FOXO1	cna	hetloss	BASIS
PD14442	FOXO1	cna	hetloss	BASIS
PD22355	FOXO1	cna	hetloss	BASIS
PD23574	FOXO1	cna	hetloss	BASIS
PD24202	FOXO1	cna	hetloss	BASIS
PD24206	FOXO1	cna	gain	BASIS
PD3890	FOXO1	cna	hetloss	BASIS
PD3905	FOXO1	cna	gain	BASIS
PD4005	FOXO1	cna	homdel	BASIS
PD4006	FOXO1	cna	hetloss	BASIS
PD4826	FOXO1	cna	hetloss	BASIS
PD4967	FOXO1	cna	hetloss	BASIS
PD5945	FOXO1	cna	amp	BASIS
PD6406	FOXO1	cna	hetloss	BASIS
PD6731	FOXO1	cna	hetloss	BASIS
PD7215	FOXO1	cna	gain	BASIS
PD8621	FOXO1	cna	hetloss	BASIS
PD8980	FOXO1	cna	hetloss	BASIS
PD9004	FOXO1	cna	hetloss	BASIS
PD9585	FOXO1	cna	gain	BASIS
PD9702	FOXO1	cna	gain	BASIS
TCGA-A2-A25B	GMPS	cna	gain	TCGA
TCGA-AN-A0XU	GMPS	cna	gain	TCGA
TCGA-BH-A0C0	GMPS	cna	gain	TCGA
TCGA-BH-A18R	GMPS	cna	gain	TCGA
TCGA-BH-A1FU	GMPS	cna	hetloss	TCGA
TCGA-C8-A12L	GMPS	cna	gain	TCGA
TCGA-E2-A1L7	GMPS	cna	gain	TCGA
TCGA-E9-A1NC	GMPS	cna	gain	TCGA
TCGA-LL-A5YP	GMPS	cna	gain	TCGA
MB-0346	GMPS	cna	hetloss	METABRIC
MB-5070	GMPS	cna	amp	METABRIC

MB-5107	GMPS	cna	amp	METABRIC
MB-5465	GMPS	cna	amp	METABRIC
MB-6060	GMPS	cna	gain	METABRIC
MB-6098	GMPS	cna	gain	METABRIC
MB-7048	GMPS	cna	hetloss	METABRIC
MB-0420	GMPS	cna	amp	METABRIC
PD11327	GMPS	cna	gain	BASIS
PD13296	GMPS	cna	gain	BASIS
PD13299	GMPS	cna	gain	BASIS
PD13771	GMPS	cna	hetloss	BASIS
PD22355	GMPS	cna	gain	BASIS
PD23561	GMPS	cna	hetloss	BASIS
PD23562	GMPS	cna	amp	BASIS
PD23574	GMPS	cna	gain	BASIS
PD23578	GMPS	cna	gain	BASIS
PD24186	GMPS	cna	gain	BASIS
PD24202	GMPS	cna	gain	BASIS
PD24206	GMPS	cna	gain	BASIS
PD3905	GMPS	cna	gain	BASIS
PD4107	GMPS	cna	gain	BASIS
PD5930	GMPS	cna	gain	BASIS
PD5935	GMPS	cna	gain	BASIS
PD5945	GMPS	cna	amp	BASIS
PD5948	GMPS	cna	amp	BASIS
PD6731	GMPS	cna	gain	BASIS
PD7067	GMPS	cna	gain	BASIS
PD7215	GMPS	cna	gain	BASIS
PD8621	GMPS	cna	gain	BASIS
PD8980	GMPS	cna	gain	BASIS
PD9585	GMPS	cna	hetloss	BASIS
PD9702	GMPS	cna	amp	BASIS
TCGA-A2-A25B	GOLPH3	cna	amp	TCGA
TCGA-AN-A0XU	GOLPH3	cna	gain	TCGA
TCGA-AO-A0JL	GOLPH3	cna	gain	TCGA
TCGA-BH-A0AW	GOLPH3	cna	gain	TCGA
TCGA-BH-A0C0	GOLPH3	cna	gain	TCGA
TCGA-BH-A1FU	GOLPH3	cna	amp	TCGA
TCGA-C8-A12L	GOLPH3	cna	gain	TCGA
TCGA-E9-A1NC	GOLPH3	cna	hetloss	TCGA
TCGA-LL-A5YP	GOLPH3	cna	amp	TCGA
MB-0346	GOLPH3	cna	gain	METABRIC
MB-5107	GOLPH3	cna	gain	METABRIC
MB-6060	GOLPH3	cna	gain	METABRIC
MB-6098	GOLPH3	cna	amp	METABRIC
MB-7048	GOLPH3	cna	hetloss	METABRIC
MB-0420	GOLPH3	cna	gain	METABRIC
PD10014	GOLPH3	cna	hetloss	BASIS

PD11327	GOLPH3	cna	homdel	BASIS
PD13296	GOLPH3	cna	hetloss	BASIS
PD13299	GOLPH3	cna	gain	BASIS
PD13771	GOLPH3	cna	hetloss	BASIS
PD23562	GOLPH3	cna	gain	BASIS
PD23574	GOLPH3	cna	gain	BASIS
PD24186	GOLPH3	cna	gain	BASIS
PD24206	GOLPH3	cna	gain	BASIS
PD24337	GOLPH3	cna	hetloss	BASIS
PD3905	GOLPH3	cna	gain	BASIS
PD4005	GOLPH3	cna	hetloss	BASIS
PD4006	GOLPH3	cna	gain	BASIS
PD4107	GOLPH3	cna	gain	BASIS
PD4826	GOLPH3	cna	gain	BASIS
PD5930	GOLPH3	cna	gain	BASIS
PD5935	GOLPH3	cna	gain	BASIS
PD5945	GOLPH3	cna	amp	BASIS
PD5948	GOLPH3	cna	gain	BASIS
PD6406	GOLPH3	cna	gain	BASIS
PD6413	GOLPH3	cna	gain	BASIS
PD6731	GOLPH3	cna	gain	BASIS
PD7067	GOLPH3	cna	gain	BASIS
PD7215	GOLPH3	cna	amp	BASIS
PD8980	GOLPH3	cna	gain	BASIS
PD9004	GOLPH3	cna	gain	BASIS
PD9585	GOLPH3	cna	gain	BASIS
TCGA-AN-A0XU	HMGN2P46	cna	hetloss	TCGA
TCGA-AO-A0JL	HMGN2P46	cna	hetloss	TCGA
TCGA-BH-A0AW	HMGN2P46	cna	hetloss	TCGA
TCGA-BH-A0C0	HMGN2P46	cna	hetloss	TCGA
TCGA-BH-A18R	HMGN2P46	cna	gain	TCGA
TCGA-C8-A12L	HMGN2P46	cna	hetloss	TCGA
TCGA-D8-A27M	HMGN2P46	cna	hetloss	TCGA
TCGA-E2-A1L7	HMGN2P46	cna	hetloss	TCGA
TCGA-E9-A1NC	HMGN2P46	cna	hetloss	TCGA
TCGA-LL-A5YP	HMGN2P46	cna	gain	TCGA
MB-0346	HMGN2P46	cna	gain	METABRIC
MB-2827	HMGN2P46	cna	hetloss	METABRIC
MB-5107	HMGN2P46	cna	gain	METABRIC
MB-5465	HMGN2P46	cna	hetloss	METABRIC
MB-6060	HMGN2P46	cna	hetloss	METABRIC
MB-6098	HMGN2P46	cna	hetloss	METABRIC
MB-7048	HMGN2P46	cna	hetloss	METABRIC
MB-0420	HMGN2P46	cna	hetloss	METABRIC
PD10014	HMGN2P46	cna	hetloss	BASIS
PD11327	HMGN2P46	cna	hetloss	BASIS
PD11742	HMGN2P46	cna	hetloss	BASIS

PD13296	HMGN2P46	cna	homdel	BASIS
PD13297	HMGN2P46	cna	hetloss	BASIS
PD13299	HMGN2P46	cna	hetloss	BASIS
PD22355	HMGN2P46	cna	hetloss	BASIS
PD23574	HMGN2P46	cna	hetloss	BASIS
PD23578	HMGN2P46	cna	hetloss	BASIS
PD24202	HMGN2P46	cna	hetloss	BASIS
PD24337	HMGN2P46	cna	hetloss	BASIS
PD3890	HMGN2P46	cna	hetloss	BASIS
PD3905	HMGN2P46	cna	gain	BASIS
PD4005	HMGN2P46	cna	homdel	BASIS
PD4967	HMGN2P46	cna	hetloss	BASIS
PD5930	HMGN2P46	cna	homdel	BASIS
PD5948	HMGN2P46	cna	hetloss	BASIS
PD6406	HMGN2P46	cna	hetloss	BASIS
PD6413	HMGN2P46	cna	hetloss	BASIS
PD7067	HMGN2P46	cna	gain	BASIS
PD7215	HMGN2P46	cna	gain	BASIS
PD8980	HMGN2P46	cna	hetloss	BASIS
PD9585	HMGN2P46	cna	hetloss	BASIS
PD9702	HMGN2P46	cna	gain	BASIS
TCGA-A2-A25B	IL7R	cna	amp	TCGA
TCGA-AN-A0XU	IL7R	cna	gain	TCGA
TCGA-AO-A0JL	IL7R	cna	gain	TCGA
TCGA-BH-A0AW	IL7R	cna	gain	TCGA
TCGA-BH-A0C0	IL7R	cna	gain	TCGA
TCGA-BH-A1FU	IL7R	cna	amp	TCGA
TCGA-E9-A1NC	IL7R	cna	hetloss	TCGA
TCGA-LL-A5YP	IL7R	cna	amp	TCGA
MB-0346	IL7R	cna	gain	METABRIC
MB-5070	IL7R	cna	gain	METABRIC
MB-5107	IL7R	cna	gain	METABRIC
MB-6060	IL7R	cna	gain	METABRIC
MB-6098	IL7R	cna	amp	METABRIC
MB-7048	IL7R	cna	hetloss	METABRIC
MB-0420	IL7R	cna	gain	METABRIC
PD11327	IL7R	cna	gain	BASIS
PD13296	IL7R	cna	hetloss	BASIS
PD13299	IL7R	cna	gain	BASIS
PD13771	IL7R	cna	hetloss	BASIS
PD23562	IL7R	cna	gain	BASIS
PD23574	IL7R	cna	gain	BASIS
PD24186	IL7R	cna	gain	BASIS
PD24206	IL7R	cna	gain	BASIS
PD24337	IL7R	cna	hetloss	BASIS
PD3890	IL7R	cna	gain	BASIS
PD3905	IL7R	cna	gain	BASIS

PD4005	IL7R	cna	hetloss	BASIS
PD4006	IL7R	cna	gain	BASIS
PD4107	IL7R	cna	gain	BASIS
PD4826	IL7R	cna	gain	BASIS
PD5930	IL7R	cna	gain	BASIS
PD5935	IL7R	cna	hetloss	BASIS
PD5945	IL7R	cna	amp	BASIS
PD5948	IL7R	cna	gain	BASIS
PD6406	IL7R	cna	gain	BASIS
PD6413	IL7R	cna	gain	BASIS
PD6731	IL7R	cna	gain	BASIS
PD7067	IL7R	cna	gain	BASIS
PD7215	IL7R	cna	amp	BASIS
PD8980	IL7R	cna	gain	BASIS
PD9004	IL7R	cna	gain	BASIS
PD9585	IL7R	cna	gain	BASIS
TCGA-A2-A25B	KRAS	cna	hetloss	TCGA
TCGA-AN-A0XU	KRAS	cna	gain	TCGA
TCGA-AO-A0JL	KRAS	cna	gain	TCGA
TCGA-BH-A0C0	KRAS	cna	hetloss	TCGA
TCGA-BH-A18R	KRAS	cna	gain	TCGA
TCGA-BH-A1FU	KRAS	cna	hetloss	TCGA
TCGA-C8-A12L	KRAS	cna	gain	TCGA
TCGA-D8-A27M	KRAS	cna	gain	TCGA
TCGA-E2-A1L7	KRAS	cna	hetloss	TCGA
TCGA-LL-A5YP	KRAS	cna	hetloss	TCGA
MB-0346	KRAS	cna	gain	METABRIC
MB-2827	KRAS	cna	hetloss	METABRIC
MB-6098	KRAS	cna	gain	METABRIC
MB-7038	KRAS	cna	hetloss	METABRIC
MB-7048	KRAS	cna	hetloss	METABRIC
MTS-T0064	KRAS	cna	amp	METABRIC
P-0009557-T01-IM5	KRAS	cna	homdel	MSK-IMPACT
PD10014	KRAS	cna	gain	BASIS
PD11327	KRAS	cna	amp	BASIS
PD11742	KRAS	cna	gain	BASIS
PD13296	KRAS	cna	hetloss	BASIS
PD13297	KRAS	cna	hetloss	BASIS
PD13299	KRAS	cna	gain	BASIS
PD13771	KRAS	cna	hetloss	BASIS
PD23574	KRAS	cna	gain	BASIS
PD24186	KRAS	cna	gain	BASIS
PD3905	KRAS	cna	gain	BASIS
PD4005	KRAS	cna	hetloss	BASIS
PD4006	KRAS	cna	gain	BASIS
PD4107	KRAS	cna	gain	BASIS
PD4826	KRAS	cna	hetloss	BASIS

PD5930	KRAS	cna	gain	BASIS
PD5935	KRAS	cna	gain	BASIS
PD5945	KRAS	cna	amp	BASIS
PD5948	KRAS	cna	homdel	BASIS
PD6406	KRAS	cna	gain	BASIS
PD6731	KRAS	cna	hetloss	BASIS
PD7215	KRAS	cna	gain	BASIS
PD8621	KRAS	cna	gain	BASIS
PD8980	KRAS	cna	hetloss	BASIS
PD9004	KRAS	cna	amp	BASIS
PD9702	KRAS	cna	amp	BASIS
TCGA-AN-A0XU	LCP1	cna	hetloss	TCGA
TCGA-AO-A0JL	LCP1	cna	hetloss	TCGA
TCGA-BH-A0AW	LCP1	cna	hetloss	TCGA
TCGA-BH-A0C0	LCP1	cna	hetloss	TCGA
TCGA-BH-A1FU	LCP1	cna	hetloss	TCGA
TCGA-C8-A12L	LCP1	cna	gain	TCGA
TCGA-LL-A5YP	LCP1	cna	hetloss	TCGA
MB-0346	LCP1	cna	hetloss	METABRIC
MB-2827	LCP1	cna	hetloss	METABRIC
MB-5107	LCP1	cna	hetloss	METABRIC
MB-6098	LCP1	cna	hetloss	METABRIC
MB-7032	LCP1	cna	hetloss	METABRIC
MB-7038	LCP1	cna	hetloss	METABRIC
MB-7048	LCP1	cna	hetloss	METABRIC
MB-0420	LCP1	cna	hetloss	METABRIC
MTS-T0064	LCP1	cna	hetloss	METABRIC
PD10014	LCP1	cna	hetloss	BASIS
PD11327	LCP1	cna	gain	BASIS
PD11742	LCP1	cna	hetloss	BASIS
PD13296	LCP1	cna	hetloss	BASIS
PD13297	LCP1	cna	hetloss	BASIS
PD13771	LCP1	cna	hetloss	BASIS
PD14442	LCP1	cna	hetloss	BASIS
PD22355	LCP1	cna	hetloss	BASIS
PD23574	LCP1	cna	hetloss	BASIS
PD24202	LCP1	cna	hetloss	BASIS
PD24206	LCP1	cna	gain	BASIS
PD3890	LCP1	cna	hetloss	BASIS
PD3905	LCP1	cna	gain	BASIS
PD4005	LCP1	cna	homdel	BASIS
PD4006	LCP1	cna	hetloss	BASIS
PD4826	LCP1	cna	hetloss	BASIS
PD4967	LCP1	cna	hetloss	BASIS
PD5945	LCP1	cna	gain	BASIS
PD6406	LCP1	cna	hetloss	BASIS
PD6731	LCP1	cna	hetloss	BASIS

PD7215	LCP1	cna	gain	BASIS
PD8621	LCP1	cna	hetloss	BASIS
PD8980	LCP1	cna	hetloss	BASIS
PD9004	LCP1	cna	hetloss	BASIS
PD9585	LCP1	cna	hetloss	BASIS
PD9702	LCP1	cna	gain	BASIS
TCGA-A2-A25B	MSI2	cna	amp	TCGA
TCGA-AN-A0XU	MSI2	cna	gain	TCGA
TCGA-AO-A0JL	MSI2	cna	hetloss	TCGA
TCGA-BH-A0AW	MSI2	cna	amp	TCGA
TCGA-BH-A0C0	MSI2	cna	gain	TCGA
TCGA-BH-A18R	MSI2	cna	amp	TCGA
TCGA-C8-A12L	MSI2	cna	gain	TCGA
TCGA-D8-A27M	MSI2	cna	hetloss	TCGA
TCGA-EW-A1OX	MSI2	cna	amp	TCGA
MB-0346	MSI2	cna	gain	METABRIC
MB-5070	MSI2	cna	hetloss	METABRIC
MB-5107	MSI2	cna	amp	METABRIC
MB-5323	MSI2	cna	gain	METABRIC
MB-6060	MSI2	cna	hetloss	METABRIC
MB-6098	MSI2	cna	hetloss	METABRIC
MB-6271	MSI2	cna	hetloss	METABRIC
MB-7048	MSI2	cna	gain	METABRIC
PD11327	MSI2	cna	gain	BASIS
PD11742	MSI2	cna	gain	BASIS
PD13297	MSI2	cna	hetloss	BASIS
PD13299	MSI2	cna	gain	BASIS
PD22355	MSI2	cna	gain	BASIS
PD23561	MSI2	cna	gain	BASIS
PD23562	MSI2	cna	gain	BASIS
PD23574	MSI2	cna	gain	BASIS
PD24186	MSI2	cna	gain	BASIS
PD24337	MSI2	cna	hetloss	BASIS
PD3890	MSI2	cna	hetloss	BASIS
PD3905	MSI2	cna	gain	BASIS
PD4006	MSI2	cna	amp	BASIS
PD4967	MSI2	cna	hetloss	BASIS
PD5930	MSI2	cna	gain	BASIS
PD5945	MSI2	cna	amp	BASIS
PD5948	MSI2	cna	gain	BASIS
PD6406	MSI2	cna	hetloss	BASIS
PD6731	MSI2	cna	hetloss	BASIS
PD7067	MSI2	cna	gain	BASIS
PD7215	MSI2	cna	gain	BASIS
PD8621	MSI2	cna	gain	BASIS
PD8980	MSI2	cna	gain	BASIS
PD9004	MSI2	cna	gain	BASIS

PD9702	MSI2	cna	gain	BASIS
TCGA-AN-A0XU	MTCP1	cna	hetloss	TCGA
TCGA-BH-A0AW	MTCP1	cna	gain	TCGA
TCGA-BH-A0C0	MTCP1	cna	gain	TCGA
TCGA-BH-A1FU	MTCP1	cna	hetloss	TCGA
TCGA-C8-A12L	MTCP1	cna	gain	TCGA
TCGA-E2-A1L7	MTCP1	cna	hetloss	TCGA
TCGA-E9-A1NC	MTCP1	cna	gain	TCGA
TCGA-LL-A5YP	MTCP1	cna	gain	TCGA
MB-0346	MTCP1	cna	hetloss	METABRIC
MB-2827	MTCP1	cna	hetloss	METABRIC
MB-5465	MTCP1	cna	hetloss	METABRIC
MB-6060	MTCP1	cna	gain	METABRIC
MB-6098	MTCP1	cna	hetloss	METABRIC
MB-7038	MTCP1	cna	gain	METABRIC
MTS-T0064	MTCP1	cna	hetloss	METABRIC
PD10014	MTCP1	cna	hetloss	BASIS
PD13296	MTCP1	cna	gain	BASIS
PD13299	MTCP1	cna	amp	BASIS
PD13771	MTCP1	cna	hetloss	BASIS
PD14442	MTCP1	cna	gain	BASIS
PD23562	MTCP1	cna	hetloss	BASIS
PD23574	MTCP1	cna	gain	BASIS
PD23578	MTCP1	cna	hetloss	BASIS
PD24202	MTCP1	cna	hetloss	BASIS
PD24206	MTCP1	cna	gain	BASIS
PD3890	MTCP1	cna	hetloss	BASIS
PD3905	MTCP1	cna	gain	BASIS
PD4005	MTCP1	cna	gain	BASIS
PD4006	MTCP1	cna	gain	BASIS
PD4107	MTCP1	cna	gain	BASIS
PD4826	MTCP1	cna	gain	BASIS
PD4967	MTCP1	cna	gain	BASIS
PD5930	MTCP1	cna	gain	BASIS
PD5935	MTCP1	cna	gain	BASIS
PD5945	MTCP1	cna	amp	BASIS
PD5948	MTCP1	cna	gain	BASIS
PD6406	MTCP1	cna	gain	BASIS
PD6413	MTCP1	cna	gain	BASIS
PD7215	MTCP1	cna	amp	BASIS
PD8621	MTCP1	cna	amp	BASIS
PD8980	MTCP1	cna	gain	BASIS
PD9004	MTCP1	cna	gain	BASIS
TCGA-A2-A25B	NFKB1	cna	hetloss	TCGA
TCGA-AN-A0XU	NFKB1	cna	hetloss	TCGA
TCGA-BH-A0AW	NFKB1	cna	hetloss	TCGA
TCGA-BH-A0C0	NFKB1	cna	hetloss	TCGA

TCGA-BH-A1FU	NFKB1	cna	gain	TCGA
TCGA-C8-A12L	NFKB1	cna	hetloss	TCGA
TCGA-D8-A27M	NFKB1	cna	gain	TCGA
TCGA-E2-A1L7	NFKB1	cna	hetloss	TCGA
TCGA-E9-A1NC	NFKB1	cna	hetloss	TCGA
TCGA-LL-A5YP	NFKB1	cna	gain	TCGA
MB-2827	NFKB1	cna	hetloss	METABRIC
MB-5070	NFKB1	cna	hetloss	METABRIC
MB-5465	NFKB1	cna	hetloss	METABRIC
MB-7048	NFKB1	cna	hetloss	METABRIC
MB-0420	NFKB1	cna	hetloss	METABRIC
PD10014	NFKB1	cna	gain	BASIS
PD11742	NFKB1	cna	hetloss	BASIS
PD13296	NFKB1	cna	hetloss	BASIS
PD13297	NFKB1	cna	hetloss	BASIS
PD13771	NFKB1	cna	gain	BASIS
PD22355	NFKB1	cna	hetloss	BASIS
PD23562	NFKB1	cna	gain	BASIS
PD23574	NFKB1	cna	gain	BASIS
PD23578	NFKB1	cna	hetloss	BASIS
PD24202	NFKB1	cna	hetloss	BASIS
PD24206	NFKB1	cna	gain	BASIS
PD24337	NFKB1	cna	hetloss	BASIS
PD3890	NFKB1	cna	gain	BASIS
PD3905	NFKB1	cna	gain	BASIS
PD4006	NFKB1	cna	hetloss	BASIS
PD4107	NFKB1	cna	gain	BASIS
PD4826	NFKB1	cna	gain	BASIS
PD5930	NFKB1	cna	hetloss	BASIS
PD5935	NFKB1	cna	gain	BASIS
PD5945	NFKB1	cna	gain	BASIS
PD5948	NFKB1	cna	gain	BASIS
PD6406	NFKB1	cna	hetloss	BASIS
PD7067	NFKB1	cna	gain	BASIS
PD7215	NFKB1	cna	gain	BASIS
PD8621	NFKB1	cna	hetloss	BASIS
PD8980	NFKB1	cna	hetloss	BASIS
PD9585	NFKB1	cna	hetloss	BASIS
TCGA-A2-A25B	NOTCH1	cna	gain	TCGA
TCGA-AN-A0XU	NOTCH1	cna	hetloss	TCGA
TCGA-AO-A0JL	NOTCH1	cna	hetloss	TCGA
TCGA-BH-A0C0	NOTCH1	cna	gain	TCGA
TCGA-BH-A18R	NOTCH1	cna	amp	TCGA
TCGA-C8-A12L	NOTCH1	cna	gain	TCGA
TCGA-E2-A1L7	NOTCH1	cna	hetloss	TCGA
TCGA-E9-A1NC	NOTCH1	cna	hetloss	TCGA
TCGA-LL-A5YP	NOTCH1	cna	hetloss	TCGA

MB-0346	NOTCH1	cna	hetloss	METABRIC
MB-2827	NOTCH1	cna	amp	METABRIC
MB-5070	NOTCH1	cna	hetloss	METABRIC
MB-7038	NOTCH1	cna	hetloss	METABRIC
MB-7048	NOTCH1	cna	hetloss	METABRIC
MB-0420	NOTCH1	cna	hetloss	METABRIC
P-0009557-T01-IM5	NOTCH1	cna	homdel	MSK-IMPACT
PD10014	NOTCH1	cna	gain	BASIS
PD11327	NOTCH1	cna	gain	BASIS
PD11742	NOTCH1	cna	gain	BASIS
PD13297	NOTCH1	cna	hetloss	BASIS
PD13299	NOTCH1	cna	gain	BASIS
PD22355	NOTCH1	cna	gain	BASIS
PD24186	NOTCH1	cna	gain	BASIS
PD24202	NOTCH1	cna	hetloss	BASIS
PD24206	NOTCH1	cna	gain	BASIS
PD24337	NOTCH1	cna	hetloss	BASIS
PD3890	NOTCH1	cna	hetloss	BASIS
PD3905	NOTCH1	cna	gain	BASIS
PD4005	NOTCH1	cna	hetloss	BASIS
PD4006	NOTCH1	cna	gain	BASIS
PD4107	NOTCH1	cna	hetloss	BASIS
PD4826	NOTCH1	cna	gain	BASIS
PD5935	NOTCH1	cna	hetloss	BASIS
PD5945	NOTCH1	cna	gain	BASIS
PD5948	NOTCH1	cna	gain	BASIS
PD6406	NOTCH1	cna	hetloss	BASIS
PD6731	NOTCH1	cna	hetloss	BASIS
PD7067	NOTCH1	cna	gain	BASIS
PD7215	NOTCH1	cna	gain	BASIS
PD8621	NOTCH1	cna	gain	BASIS
PD8980	NOTCH1	cna	hetloss	BASIS
PD9004	NOTCH1	cna	hetloss	BASIS
TCGA-AN-A0XU	PBRM1	cna	hetloss	TCGA
TCGA-BH-A0C0	PBRM1	cna	hetloss	TCGA
TCGA-BH-A18R	PBRM1	cna	homdel	TCGA
TCGA-BH-A1FU	PBRM1	cna	hetloss	TCGA
TCGA-C8-A12L	PBRM1	cna	hetloss	TCGA
TCGA-D8-A27M	PBRM1	cna	hetloss	TCGA
TCGA-E2-A1L7	PBRM1	cna	hetloss	TCGA
TCGA-E9-A1NC	PBRM1	cna	hetloss	TCGA
TCGA-LL-A5YP	PBRM1	cna	hetloss	TCGA
MB-0346	PBRM1	cna	hetloss	METABRIC
MB-2827	PBRM1	cna	hetloss	METABRIC
MB-5465	PBRM1	cna	hetloss	METABRIC
MB-6098	PBRM1	cna	hetloss	METABRIC
MB-7038	PBRM1	cna	hetloss	METABRIC

MB-7048	PBRM1	cna	hetloss	METABRIC
MB-0420	PBRM1	cna	hetloss	METABRIC
MTS-T0064	PBRM1	cna	hetloss	METABRIC
PD10014	PBRM1	cna	hetloss	BASIS
PD11327	PBRM1	cna	hetloss	BASIS
PD13296	PBRM1	cna	hetloss	BASIS
PD13297	PBRM1	cna	hetloss	BASIS
PD13299	PBRM1	cna	gain	BASIS
PD13771	PBRM1	cna	hetloss	BASIS
PD14442	PBRM1	cna	hetloss	BASIS
PD22355	PBRM1	cna	hetloss	BASIS
PD23561	PBRM1	cna	hetloss	BASIS
PD23578	PBRM1	cna	hetloss	BASIS
PD24202	PBRM1	cna	hetloss	BASIS
PD24206	PBRM1	cna	hetloss	BASIS
PD24337	PBRM1	cna	hetloss	BASIS
PD3890	PBRM1	cna	hetloss	BASIS
PD3905	PBRM1	cna	gain	BASIS
PD4005	PBRM1	cna	hetloss	BASIS
PD4826	PBRM1	cna	gain	BASIS
PD4967	PBRM1	cna	hetloss	BASIS
PD5945	PBRM1	cna	gain	BASIS
PD6406	PBRM1	cna	hetloss	BASIS
PD6413	PBRM1	cna	hetloss	BASIS
PD6731	PBRM1	cna	hetloss	BASIS
PD7067	PBRM1	cna	gain	BASIS
PD8980	PBRM1	cna	hetloss	BASIS
PD9585	PBRM1	cna	hetloss	BASIS
TCGA-AN-A0XU	PIK3R1	cna	hetloss	TCGA
TCGA-AO-A0JL	PIK3R1	cna	hetloss	TCGA
TCGA-BH-A0C0	PIK3R1	cna	hetloss	TCGA
TCGA-BH-A1FU	PIK3R1	cna	hetloss	TCGA
TCGA-C8-A12L	PIK3R1	cna	hetloss	TCGA
TCGA-D8-A27M	PIK3R1	cna	hetloss	TCGA
TCGA-E2-A1L7	PIK3R1	cna	hetloss	TCGA
TCGA-E9-A1NC	PIK3R1	cna	hetloss	TCGA
TCGA-LL-A5YP	PIK3R1	cna	hetloss	TCGA
MB-0346	PIK3R1	cna	gain	METABRIC
MB-2827	PIK3R1	cna	hetloss	METABRIC
MB-5070	PIK3R1	cna	homdel	METABRIC
MB-5107	PIK3R1	cna	hetloss	METABRIC
MB-5465	PIK3R1	cna	hetloss	METABRIC
MB-6098	PIK3R1	cna	hetloss	METABRIC
MB-7038	PIK3R1	cna	hetloss	METABRIC
MB-0420	PIK3R1	cna	hetloss	METABRIC
PD10014	PIK3R1	cna	hetloss	BASIS
PD11742	PIK3R1	cna	hetloss	BASIS

PD13296	PIK3R1	cna	hetloss	BASIS
PD13297	PIK3R1	cna	hetloss	BASIS
PD22355	PIK3R1	cna	hetloss	BASIS
PD23562	PIK3R1	cna	hetloss	BASIS
PD23578	PIK3R1	cna	hetloss	BASIS
PD24202	PIK3R1	cna	hetloss	BASIS
PD24206	PIK3R1	cna	gain	BASIS
PD24337	PIK3R1	cna	hetloss	BASIS
PD3890	PIK3R1	cna	hetloss	BASIS
PD3905	PIK3R1	cna	gain	BASIS
PD4005	PIK3R1	cna	hetloss	BASIS
PD4006	PIK3R1	cna	homdel	BASIS
PD4107	PIK3R1	cna	hetloss	BASIS
PD5930	PIK3R1	cna	gain	BASIS
PD5935	PIK3R1	cna	hetloss	BASIS
PD5948	PIK3R1	cna	hetloss	BASIS
PD6406	PIK3R1	cna	hetloss	BASIS
PD6413	PIK3R1	cna	hetloss	BASIS
PD6731	PIK3R1	cna	hetloss	BASIS
PD7215	PIK3R1	cna	hetloss	BASIS
PD8621	PIK3R1	cna	hetloss	BASIS
PD8980	PIK3R1	cna	hetloss	BASIS
PD9585	PIK3R1	cna	hetloss	BASIS
TCGA-A2-A25B	PTPN6	cna	hetloss	TCGA
TCGA-AN-A0XU	PTPN6	cna	gain	TCGA
TCGA-AO-A0JL	PTPN6	cna	gain	TCGA
TCGA-BH-A0C0	PTPN6	cna	hetloss	TCGA
TCGA-BH-A18R	PTPN6	cna	gain	TCGA
TCGA-BH-A1FU	PTPN6	cna	hetloss	TCGA
TCGA-C8-A12L	PTPN6	cna	gain	TCGA
TCGA-D8-A27M	PTPN6	cna	gain	TCGA
TCGA-E9-A1NC	PTPN6	cna	gain	TCGA
TCGA-LL-A5YP	PTPN6	cna	gain	TCGA
MB-0346	PTPN6	cna	hetloss	METABRIC
MB-5070	PTPN6	cna	amp	METABRIC
MB-5107	PTPN6	cna	hetloss	METABRIC
MB-6098	PTPN6	cna	gain	METABRIC
MB-7048	PTPN6	cna	gain	METABRIC
MTS-T0064	PTPN6	cna	amp	METABRIC
PD10014	PTPN6	cna	hetloss	BASIS
PD11742	PTPN6	cna	gain	BASIS
PD13299	PTPN6	cna	gain	BASIS
PD13771	PTPN6	cna	hetloss	BASIS
PD23574	PTPN6	cna	gain	BASIS
PD23578	PTPN6	cna	gain	BASIS
PD24186	PTPN6	cna	gain	BASIS
PD24206	PTPN6	cna	hetloss	BASIS

PD24337	PTPN6	cna	amp	BASIS
PD3890	PTPN6	cna	gain	BASIS
PD3905	PTPN6	cna	gain	BASIS
PD4005	PTPN6	cna	hetloss	BASIS
PD4006	PTPN6	cna	amp	BASIS
PD4107	PTPN6	cna	gain	BASIS
PD4826	PTPN6	cna	hetloss	BASIS
PD4967	PTPN6	cna	hetloss	BASIS
PD5930	PTPN6	cna	gain	BASIS
PD5935	PTPN6	cna	gain	BASIS
PD5945	PTPN6	cna	amp	BASIS
PD6406	PTPN6	cna	gain	BASIS
PD6731	PTPN6	cna	hetloss	BASIS
PD7215	PTPN6	cna	gain	BASIS
PD8621	PTPN6	cna	gain	BASIS
PD8980	PTPN6	cna	hetloss	BASIS
PD9004	PTPN6	cna	gain	BASIS
PD9702	PTPN6	cna	gain	BASIS
TCGA-A2-A25B	SDHA	cna	gain	TCGA
TCGA-AN-A0XU	SDHA	cna	gain	TCGA
TCGA-AO-A0JL	SDHA	cna	gain	TCGA
TCGA-BH-A0AW	SDHA	cna	gain	TCGA
TCGA-BH-A1FU	SDHA	cna	hetloss	TCGA
TCGA-C8-A12L	SDHA	cna	gain	TCGA
TCGA-E2-A1L7	SDHA	cna	gain	TCGA
TCGA-LL-A5YP	SDHA	cna	gain	TCGA
MB-0346	SDHA	cna	gain	METABRIC
MB-2827	SDHA	cna	hetloss	METABRIC
MB-5107	SDHA	cna	gain	METABRIC
MB-6098	SDHA	cna	amp	METABRIC
MB-7038	SDHA	cna	amp	METABRIC
MB-0420	SDHA	cna	gain	METABRIC
MTS-T0064	SDHA	cna	gain	METABRIC
PD10014	SDHA	cna	hetloss	BASIS
PD11327	SDHA	cna	gain	BASIS
PD13771	SDHA	cna	hetloss	BASIS
PD22355	SDHA	cna	hetloss	BASIS
PD23562	SDHA	cna	gain	BASIS
PD23574	SDHA	cna	gain	BASIS
PD23578	SDHA	cna	hetloss	BASIS
PD24186	SDHA	cna	gain	BASIS
PD24206	SDHA	cna	amp	BASIS
PD24337	SDHA	cna	hetloss	BASIS
PD3890	SDHA	cna	gain	BASIS
PD3905	SDHA	cna	gain	BASIS
PD4005	SDHA	cna	gain	BASIS
PD4006	SDHA	cna	amp	BASIS

PD4107	SDHA	cna	gain	BASIS
PD4826	SDHA	cna	gain	BASIS
PD5930	SDHA	cna	amp	BASIS
PD5935	SDHA	cna	gain	BASIS
PD5945	SDHA	cna	amp	BASIS
PD5948	SDHA	cna	gain	BASIS
PD6413	SDHA	cna	gain	BASIS
PD6731	SDHA	cna	gain	BASIS
PD7067	SDHA	cna	gain	BASIS
PD7215	SDHA	cna	amp	BASIS
PD8980	SDHA	cna	gain	BASIS
PD9004	SDHA	cna	gain	BASIS
PD9585	SDHA	cna	gain	BASIS
TCGA-A2-A25B	SRSF2	cna	gain	TCGA
TCGA-AN-A0XU	SRSF2	cna	gain	TCGA
TCGA-AO-A0JL	SRSF2	cna	hetloss	TCGA
TCGA-BH-A0AW	SRSF2	cna	amp	TCGA
TCGA-BH-A0C0	SRSF2	cna	gain	TCGA
TCGA-BH-A18R	SRSF2	cna	amp	TCGA
TCGA-C8-A12L	SRSF2	cna	gain	TCGA
TCGA-D8-A27M	SRSF2	cna	gain	TCGA
TCGA-E2-A1L7	SRSF2	cna	gain	TCGA
TCGA-LL-A5YP	SRSF2	cna	amp	TCGA
MB-0346	SRSF2	cna	gain	METABRIC
MB-5070	SRSF2	cna	amp	METABRIC
MB-5107	SRSF2	cna	amp	METABRIC
MB-6098	SRSF2	cna	gain	METABRIC
MB-6271	SRSF2	cna	hetloss	METABRIC
MTS-T0064	SRSF2	cna	gain	METABRIC
PD10014	SRSF2	cna	gain	BASIS
PD11327	SRSF2	cna	gain	BASIS
PD22355	SRSF2	cna	hetloss	BASIS
PD23561	SRSF2	cna	hetloss	BASIS
PD23574	SRSF2	cna	gain	BASIS
PD24186	SRSF2	cna	amp	BASIS
PD24202	SRSF2	cna	gain	BASIS
PD24206	SRSF2	cna	hetloss	BASIS
PD24337	SRSF2	cna	gain	BASIS
PD3890	SRSF2	cna	gain	BASIS
PD3905	SRSF2	cna	gain	BASIS
PD4006	SRSF2	cna	gain	BASIS
PD4107	SRSF2	cna	gain	BASIS
PD4826	SRSF2	cna	amp	BASIS
PD4967	SRSF2	cna	homdel	BASIS
PD5930	SRSF2	cna	gain	BASIS
PD5935	SRSF2	cna	gain	BASIS
PD5945	SRSF2	cna	amp	BASIS

PD5948	SRSF2	cna	gain	BASIS
PD6731	SRSF2	cna	hetloss	BASIS
PD7067	SRSF2	cna	gain	BASIS
PD7215	SRSF2	cna	gain	BASIS
PD8621	SRSF2	cna	gain	BASIS
PD9004	SRSF2	cna	gain	BASIS
PD9585	SRSF2	cna	gain	BASIS
PD9702	SRSF2	cna	gain	BASIS
TCGA-A2-A25B	TERT	cna	gain	TCGA
TCGA-AN-A0XU	TERT	cna	gain	TCGA
TCGA-AO-A0JL	TERT	cna	gain	TCGA
TCGA-BH-A0AW	TERT	cna	gain	TCGA
TCGA-BH-A1FU	TERT	cna	hetloss	TCGA
TCGA-C8-A12L	TERT	cna	gain	TCGA
TCGA-E2-A1L7	TERT	cna	gain	TCGA
TCGA-LL-A5YP	TERT	cna	gain	TCGA
MB-0346	TERT	cna	gain	METABRIC
MB-2827	TERT	cna	hetloss	METABRIC
MB-5107	TERT	cna	gain	METABRIC
MB-6098	TERT	cna	amp	METABRIC
MB-7038	TERT	cna	amp	METABRIC
MB-0420	TERT	cna	gain	METABRIC
MTS-T0064	TERT	cna	gain	METABRIC
PD10014	TERT	cna	hetloss	BASIS
PD11327	TERT	cna	gain	BASIS
PD13771	TERT	cna	hetloss	BASIS
PD22355	TERT	cna	hetloss	BASIS
PD23562	TERT	cna	gain	BASIS
PD23574	TERT	cna	gain	BASIS
PD23578	TERT	cna	hetloss	BASIS
PD24186	TERT	cna	gain	BASIS
PD24206	TERT	cna	amp	BASIS
PD24337	TERT	cna	hetloss	BASIS
PD3890	TERT	cna	gain	BASIS
PD3905	TERT	cna	gain	BASIS
PD4005	TERT	cna	gain	BASIS
PD4006	TERT	cna	amp	BASIS
PD4107	TERT	cna	gain	BASIS
PD4826	TERT	cna	gain	BASIS
PD5930	TERT	cna	amp	BASIS
PD5935	TERT	cna	gain	BASIS
PD5945	TERT	cna	amp	BASIS
PD5948	TERT	cna	gain	BASIS
PD6413	TERT	cna	gain	BASIS
PD6731	TERT	cna	gain	BASIS
PD7067	TERT	cna	gain	BASIS
PD7215	TERT	cna	amp	BASIS

PD8980	TERT	cna	gain	BASIS
PD9004	TERT	cna	gain	BASIS
PD9585	TERT	cna	gain	BASIS
TCGA-A2-A25B	TLX1	cna	hetloss	TCGA
TCGA-AN-A0XU	TLX1	cna	hetloss	TCGA
TCGA-AO-A0JL	TLX1	cna	hetloss	TCGA
TCGA-BH-A18R	TLX1	cna	gain	TCGA
TCGA-C8-A12L	TLX1	cna	hetloss	TCGA
TCGA-D8-A27M	TLX1	cna	hetloss	TCGA
TCGA-E2-A1L7	TLX1	cna	hetloss	TCGA
TCGA-LL-A5YP	TLX1	cna	gain	TCGA
MB-0346	TLX1	cna	gain	METABRIC
MB-2827	TLX1	cna	hetloss	METABRIC
MB-5070	TLX1	cna	hetloss	METABRIC
MB-6060	TLX1	cna	hetloss	METABRIC
MB-6098	TLX1	cna	hetloss	METABRIC
MB-7038	TLX1	cna	hetloss	METABRIC
MB-7048	TLX1	cna	hetloss	METABRIC
MB-0420	TLX1	cna	hetloss	METABRIC
PD10014	TLX1	cna	hetloss	BASIS
PD11327	TLX1	cna	homdel	BASIS
PD11742	TLX1	cna	hetloss	BASIS
PD22355	TLX1	cna	hetloss	BASIS
PD23562	TLX1	cna	gain	BASIS
PD23574	TLX1	cna	gain	BASIS
PD23578	TLX1	cna	hetloss	BASIS
PD24202	TLX1	cna	hetloss	BASIS
PD24206	TLX1	cna	hetloss	BASIS
PD3890	TLX1	cna	hetloss	BASIS
PD3905	TLX1	cna	gain	BASIS
PD4005	TLX1	cna	hetloss	BASIS
PD4006	TLX1	cna	gain	BASIS
PD4107	TLX1	cna	gain	BASIS
PD5935	TLX1	cna	hetloss	BASIS
PD5945	TLX1	cna	gain	BASIS
PD5948	TLX1	cna	gain	BASIS
PD6406	TLX1	cna	hetloss	BASIS
PD6731	TLX1	cna	hetloss	BASIS
PD7067	TLX1	cna	gain	BASIS
PD7215	TLX1	cna	gain	BASIS
PD8621	TLX1	cna	gain	BASIS
PD8980	TLX1	cna	hetloss	BASIS
PD9004	TLX1	cna	gain	BASIS
PD9585	TLX1	cna	hetloss	BASIS
PD9702	TLX1	cna	gain	BASIS
TCGA-AN-A0XU	TNKS	cna	hetloss	TCGA
TCGA-AO-A0JL	TNKS	cna	gain	TCGA

TCGA-BH-A0C0	TNKS	cna	hetloss	TCGA
TCGA-BH-A1FU	TNKS	cna	homdel	TCGA
TCGA-C8-A12L	TNKS	cna	homdel	TCGA
TCGA-D8-A27M	TNKS	cna	hetloss	TCGA
TCGA-E9-A1NC	TNKS	cna	hetloss	TCGA
TCGA-LL-A5YP	TNKS	cna	hetloss	TCGA
MB-0346	TNKS	cna	hetloss	METABRIC
MB-2827	TNKS	cna	hetloss	METABRIC
MB-5070	TNKS	cna	hetloss	METABRIC
MB-5107	TNKS	cna	hetloss	METABRIC
MB-5465	TNKS	cna	hetloss	METABRIC
MB-6060	TNKS	cna	hetloss	METABRIC
MB-7038	TNKS	cna	gain	METABRIC
MB-0420	TNKS	cna	hetloss	METABRIC
MTS-T0064	TNKS	cna	hetloss	METABRIC
PD10014	TNKS	cna	hetloss	BASIS
PD13296	TNKS	cna	hetloss	BASIS
PD13297	TNKS	cna	gain	BASIS
PD13299	TNKS	cna	hetloss	BASIS
PD13771	TNKS	cna	hetloss	BASIS
PD14442	TNKS	cna	hetloss	BASIS
PD22355	TNKS	cna	hetloss	BASIS
PD23562	TNKS	cna	hetloss	BASIS
PD23574	TNKS	cna	hetloss	BASIS
PD23578	TNKS	cna	hetloss	BASIS
PD24186	TNKS	cna	gain	BASIS
PD24202	TNKS	cna	hetloss	BASIS
PD24337	TNKS	cna	hetloss	BASIS
PD3905	TNKS	cna	gain	BASIS
PD4005	TNKS	cna	hetloss	BASIS
PD4006	TNKS	cna	gain	BASIS
PD4107	TNKS	cna	hetloss	BASIS
PD4826	TNKS	cna	hetloss	BASIS
PD5930	TNKS	cna	gain	BASIS
PD5945	TNKS	cna	amp	BASIS
PD6406	TNKS	cna	hetloss	BASIS
PD6413	TNKS	cna	gain	BASIS
PD7067	TNKS	cna	gain	BASIS
PD8980	TNKS	cna	hetloss	BASIS
PD9004	TNKS	cna	gain	BASIS
TCGA-A2-A25B	TRAF2	cna	gain	TCGA
TCGA-AN-A0XU	TRAF2	cna	hetloss	TCGA
TCGA-AO-A0JL	TRAF2	cna	hetloss	TCGA
TCGA-BH-A0C0	TRAF2	cna	gain	TCGA
TCGA-BH-A18R	TRAF2	cna	amp	TCGA
TCGA-C8-A12L	TRAF2	cna	gain	TCGA
TCGA-E2-A1L7	TRAF2	cna	hetloss	TCGA

TCGA-E9-A1NC	TRAF2	cna	hetloss	TCGA
TCGA-LL-A5YP	TRAF2	cna	hetloss	TCGA
MB-0346	TRAF2	cna	hetloss	METABRIC
MB-2827	TRAF2	cna	amp	METABRIC
MB-5070	TRAF2	cna	hetloss	METABRIC
MB-7038	TRAF2	cna	hetloss	METABRIC
MB-7048	TRAF2	cna	hetloss	METABRIC
MB-0420	TRAF2	cna	hetloss	METABRIC
P-0009557-T01-IM5	TRAF2	cna	homdel	MSK-IMPACT
PD10014	TRAF2	cna	gain	BASIS
PD11327	TRAF2	cna	gain	BASIS
PD11742	TRAF2	cna	gain	BASIS
PD13297	TRAF2	cna	hetloss	BASIS
PD13299	TRAF2	cna	gain	BASIS
PD22355	TRAF2	cna	gain	BASIS
PD24186	TRAF2	cna	gain	BASIS
PD24202	TRAF2	cna	hetloss	BASIS
PD24206	TRAF2	cna	gain	BASIS
PD24337	TRAF2	cna	hetloss	BASIS
PD3890	TRAF2	cna	hetloss	BASIS
PD3905	TRAF2	cna	gain	BASIS
PD4005	TRAF2	cna	hetloss	BASIS
PD4006	TRAF2	cna	gain	BASIS
PD4107	TRAF2	cna	hetloss	BASIS
PD4826	TRAF2	cna	gain	BASIS
PD5935	TRAF2	cna	hetloss	BASIS
PD5945	TRAF2	cna	gain	BASIS
PD5948	TRAF2	cna	gain	BASIS
PD6406	TRAF2	cna	hetloss	BASIS
PD6731	TRAF2	cna	hetloss	BASIS
PD7067	TRAF2	cna	gain	BASIS
PD7215	TRAF2	cna	gain	BASIS
PD8621	TRAF2	cna	gain	BASIS
PD8980	TRAF2	cna	hetloss	BASIS
PD9004	TRAF2	cna	hetloss	BASIS
TCGA-A2-A25B	TSHZ2	cna	gain	TCGA
TCGA-AO-A0JL	TSHZ2	cna	gain	TCGA
TCGA-BH-A0AW	TSHZ2	cna	gain	TCGA
TCGA-BH-A0C0	TSHZ2	cna	gain	TCGA
TCGA-BH-A18R	TSHZ2	cna	gain	TCGA
TCGA-C8-A12L	TSHZ2	cna	gain	TCGA
TCGA-D8-A27M	TSHZ2	cna	hetloss	TCGA
TCGA-E2-A1L7	TSHZ2	cna	gain	TCGA
TCGA-LL-A5YP	TSHZ2	cna	gain	TCGA
MB-0346	TSHZ2	cna	gain	METABRIC
MB-5070	TSHZ2	cna	hetloss	METABRIC
MB-5107	TSHZ2	cna	gain	METABRIC

MB-6060	TSHZ2	cna	gain	METABRIC
MB-6098	TSHZ2	cna	hetloss	METABRIC
MB-7038	TSHZ2	cna	hetloss	METABRIC
MB-7048	TSHZ2	cna	hetloss	METABRIC
PD11742	TSHZ2	cna	gain	BASIS
PD13297	TSHZ2	cna	hetloss	BASIS
PD13299	TSHZ2	cna	gain	BASIS
PD13771	TSHZ2	cna	gain	BASIS
PD22355	TSHZ2	cna	gain	BASIS
PD23562	TSHZ2	cna	gain	BASIS
PD23574	TSHZ2	cna	gain	BASIS
PD24186	TSHZ2	cna	gain	BASIS
PD24202	TSHZ2	cna	hetloss	BASIS
PD24206	TSHZ2	cna	amp	BASIS
PD3905	TSHZ2	cna	gain	BASIS
PD4005	TSHZ2	cna	homdel	BASIS
PD4006	TSHZ2	cna	gain	BASIS
PD4826	TSHZ2	cna	gain	BASIS
PD5935	TSHZ2	cna	gain	BASIS
PD5945	TSHZ2	cna	amp	BASIS
PD5948	TSHZ2	cna	gain	BASIS
PD6406	TSHZ2	cna	gain	BASIS
PD6731	TSHZ2	cna	hetloss	BASIS
PD7067	TSHZ2	cna	gain	BASIS
PD7215	TSHZ2	cna	amp	BASIS
PD8621	TSHZ2	cna	gain	BASIS
PD8980	TSHZ2	cna	gain	BASIS
PD9004	TSHZ2	cna	gain	BASIS
PD9585	TSHZ2	cna	hetloss	BASIS
PD9702	TSHZ2	cna	gain	BASIS
TCGA-A2-A25B	BRAF	cna	gain	TCGA
TCGA-AO-A0JL	BRAF	cna	gain	TCGA
TCGA-BH-A0AW	BRAF	cna	gain	TCGA
TCGA-D8-A27M	BRAF	cna	gain	TCGA
TCGA-E2-A1L7	BRAF	cna	hetloss	TCGA
TCGA-E9-A1NC	BRAF	cna	gain	TCGA
TCGA-LL-A5YP	BRAF	cna	gain	TCGA
MB-0346	BRAF	cna	hetloss	METABRIC
MB-2827	BRAF	cna	hetloss	METABRIC
MB-5070	BRAF	cna	hetloss	METABRIC
MB-6098	BRAF	cna	gain	METABRIC
MB-7038	BRAF	cna	gain	METABRIC
MB-7048	BRAF	cna	gain	METABRIC
MB-0420	BRAF	cna	hetloss	METABRIC
PD10014	BRAF	cna	gain	BASIS
PD13296	BRAF	cna	hetloss	BASIS
PD13297	BRAF	cna	gain	BASIS

PD13299	BRAF	cna	gain	BASIS
PD22355	BRAF	cna	hetloss	BASIS
PD23562	BRAF	cna	gain	BASIS
PD23574	BRAF	cna	gain	BASIS
PD23578	BRAF	cna	hetloss	BASIS
PD24186	BRAF	cna	amp	BASIS
PD24202	BRAF	cna	gain	BASIS
PD24337	BRAF	cna	gain	BASIS
PD3890	BRAF	cna	hetloss	BASIS
PD3905	BRAF	cna	gain	BASIS
PD4006	BRAF	cna	gain	BASIS
PD4107	BRAF	cna	amp	BASIS
PD4826	BRAF	cna	gain	BASIS
PD5930	BRAF	cna	gain	BASIS
PD5945	BRAF	cna	amp	BASIS
PD5948	BRAF	cna	gain	BASIS
PD6406	BRAF	cna	gain	BASIS
PD6731	BRAF	cna	hetloss	BASIS
PD7067	BRAF	cna	gain	BASIS
PD7215	BRAF	cna	gain	BASIS
PD8621	BRAF	cna	gain	BASIS
PD9004	BRAF	cna	gain	BASIS
PD9585	BRAF	cna	gain	BASIS
PD9702	BRAF	cna	gain	BASIS
TCGA-AN-A0XU	CASC5	cna	hetloss	TCGA
TCGA-AO-A0JL	CASC5	cna	hetloss	TCGA
TCGA-BH-A0C0	CASC5	cna	hetloss	TCGA
TCGA-C8-A12L	CASC5	cna	hetloss	TCGA
TCGA-D8-A27M	CASC5	cna	hetloss	TCGA
TCGA-E2-A1L7	CASC5	cna	hetloss	TCGA
TCGA-E9-A1NC	CASC5	cna	hetloss	TCGA
TCGA-LL-A5YP	CASC5	cna	hetloss	TCGA
MB-2827	CASC5	cna	hetloss	METABRIC
MB-5070	CASC5	cna	hetloss	METABRIC
MB-5107	CASC5	cna	gain	METABRIC
MB-5465	CASC5	cna	hetloss	METABRIC
MB-6060	CASC5	cna	hetloss	METABRIC
MB-6098	CASC5	cna	hetloss	METABRIC
MB-7048	CASC5	cna	hetloss	METABRIC
MB-0420	CASC5	cna	hetloss	METABRIC
PD10014	CASC5	cna	hetloss	BASIS
PD11327	CASC5	cna	homdel	BASIS
PD11742	CASC5	cna	hetloss	BASIS
PD13297	CASC5	cna	hetloss	BASIS
PD13299	CASC5	cna	hetloss	BASIS
PD22355	CASC5	cna	hetloss	BASIS
PD23574	CASC5	cna	hetloss	BASIS

PD23578	CASC5	cna	hetloss	BASIS
PD24202	CASC5	cna	hetloss	BASIS
PD24206	CASC5	cna	gain	BASIS
PD24337	CASC5	cna	hetloss	BASIS
PD3890	CASC5	cna	hetloss	BASIS
PD3905	CASC5	cna	gain	BASIS
PD4005	CASC5	cna	homdel	BASIS
PD4967	CASC5	cna	hetloss	BASIS
PD5930	CASC5	cna	hetloss	BASIS
PD5945	CASC5	cna	gain	BASIS
PD5948	CASC5	cna	hetloss	BASIS
PD6413	CASC5	cna	hetloss	BASIS
PD7067	CASC5	cna	gain	BASIS
PD7215	CASC5	cna	gain	BASIS
PD8980	CASC5	cna	hetloss	BASIS
PD9004	CASC5	cna	hetloss	BASIS
PD9585	CASC5	cna	hetloss	BASIS
PD9702	CASC5	cna	gain	BASIS
TCGA-A2-A25B	DCUN1D1	cna	amp	TCGA
TCGA-AN-A0XU	DCUN1D1	cna	gain	TCGA
TCGA-BH-A0AW	DCUN1D1	cna	gain	TCGA
TCGA-BH-A0C0	DCUN1D1	cna	gain	TCGA
TCGA-BH-A1FU	DCUN1D1	cna	hetloss	TCGA
TCGA-C8-A12L	DCUN1D1	cna	gain	TCGA
TCGA-E2-A1L7	DCUN1D1	cna	gain	TCGA
TCGA-LL-A5YP	DCUN1D1	cna	gain	TCGA
MB-0346	DCUN1D1	cna	gain	METABRIC
MB-5070	DCUN1D1	cna	amp	METABRIC
MB-5107	DCUN1D1	cna	amp	METABRIC
MB-6060	DCUN1D1	cna	gain	METABRIC
MB-6098	DCUN1D1	cna	gain	METABRIC
MB-7038	DCUN1D1	cna	hetloss	METABRIC
MB-0420	DCUN1D1	cna	gain	METABRIC
MTS-T0064	DCUN1D1	cna	amp	METABRIC
P-0002858-T01-IM3	DCUN1D1	cna	amp	MSK-IMPACT
PD11327	DCUN1D1	cna	homdel	BASIS
PD13296	DCUN1D1	cna	gain	BASIS
PD13299	DCUN1D1	cna	gain	BASIS
PD13771	DCUN1D1	cna	hetloss	BASIS
PD22355	DCUN1D1	cna	gain	BASIS
PD23561	DCUN1D1	cna	hetloss	BASIS
PD23562	DCUN1D1	cna	gain	BASIS
PD23578	DCUN1D1	cna	gain	BASIS
PD24186	DCUN1D1	cna	amp	BASIS
PD24202	DCUN1D1	cna	gain	BASIS
PD24206	DCUN1D1	cna	gain	BASIS
PD3905	DCUN1D1	cna	gain	BASIS

PD4005	DCUN1D1	cna	gain	BASIS
PD4107	DCUN1D1	cna	gain	BASIS
PD5935	DCUN1D1	cna	gain	BASIS
PD5945	DCUN1D1	cna	amp	BASIS
PD5948	DCUN1D1	cna	gain	BASIS
PD6406	DCUN1D1	cna	gain	BASIS
PD6731	DCUN1D1	cna	gain	BASIS
PD7067	DCUN1D1	cna	gain	BASIS
PD7215	DCUN1D1	cna	gain	BASIS
PD8621	DCUN1D1	cna	gain	BASIS
PD8980	DCUN1D1	cna	gain	BASIS
PD9702	DCUN1D1	cna	gain	BASIS
TCGA-AN-A0XU	DUSP9	cna	hetloss	TCGA
TCGA-BH-A0AW	DUSP9	cna	gain	TCGA
TCGA-BH-A1FU	DUSP9	cna	hetloss	TCGA
TCGA-C8-A12L	DUSP9	cna	gain	TCGA
TCGA-E2-A1L7	DUSP9	cna	hetloss	TCGA
TCGA-E9-A1NC	DUSP9	cna	gain	TCGA
TCGA-LL-A5YP	DUSP9	cna	gain	TCGA
MB-0346	DUSP9	cna	hetloss	METABRIC
MB-2827	DUSP9	cna	hetloss	METABRIC
MB-5465	DUSP9	cna	hetloss	METABRIC
MB-6060	DUSP9	cna	gain	METABRIC
MB-6098	DUSP9	cna	hetloss	METABRIC
MB-7038	DUSP9	cna	gain	METABRIC
MTS-T0064	DUSP9	cna	hetloss	METABRIC
PD10014	DUSP9	cna	hetloss	BASIS
PD13296	DUSP9	cna	gain	BASIS
PD13299	DUSP9	cna	amp	BASIS
PD13771	DUSP9	cna	hetloss	BASIS
PD14442	DUSP9	cna	gain	BASIS
PD23562	DUSP9	cna	hetloss	BASIS
PD23574	DUSP9	cna	gain	BASIS
PD23578	DUSP9	cna	hetloss	BASIS
PD24202	DUSP9	cna	hetloss	BASIS
PD24206	DUSP9	cna	gain	BASIS
PD3890	DUSP9	cna	hetloss	BASIS
PD3905	DUSP9	cna	gain	BASIS
PD4005	DUSP9	cna	gain	BASIS
PD4006	DUSP9	cna	gain	BASIS
PD4107	DUSP9	cna	gain	BASIS
PD4826	DUSP9	cna	gain	BASIS
PD4967	DUSP9	cna	gain	BASIS
PD5930	DUSP9	cna	gain	BASIS
PD5935	DUSP9	cna	gain	BASIS
PD5945	DUSP9	cna	amp	BASIS
PD5948	DUSP9	cna	gain	BASIS

PD6406	DUSP9	cna	gain	BASIS
PD6413	DUSP9	cna	gain	BASIS
PD7215	DUSP9	cna	amp	BASIS
PD8621	DUSP9	cna	amp	BASIS
PD8980	DUSP9	cna	gain	BASIS
PD9004	DUSP9	cna	gain	BASIS
TCGA-A2-A25B	ECT2L	cna	hetloss	TCGA
TCGA-AN-A0XU	ECT2L	cna	amp	TCGA
TCGA-AO-A0JL	ECT2L	cna	hetloss	TCGA
TCGA-BH-A0AW	ECT2L	cna	amp	TCGA
TCGA-BH-A0C0	ECT2L	cna	gain	TCGA
TCGA-BH-A18R	ECT2L	cna	gain	TCGA
TCGA-C8-A12L	ECT2L	cna	gain	TCGA
TCGA-D8-A27M	ECT2L	cna	hetloss	TCGA
MB-5070	ECT2L	cna	amp	METABRIC
MB-6098	ECT2L	cna	hetloss	METABRIC
MB-7032	ECT2L	cna	hetloss	METABRIC
MB-7038	ECT2L	cna	amp	METABRIC
MB-7048	ECT2L	cna	hetloss	METABRIC
MB-0420	ECT2L	cna	hetloss	METABRIC
PD10014	ECT2L	cna	gain	BASIS
PD11327	ECT2L	cna	gain	BASIS
PD13296	ECT2L	cna	hetloss	BASIS
PD13299	ECT2L	cna	gain	BASIS
PD13771	ECT2L	cna	hetloss	BASIS
PD22355	ECT2L	cna	gain	BASIS
PD23562	ECT2L	cna	gain	BASIS
PD23574	ECT2L	cna	gain	BASIS
PD24186	ECT2L	cna	gain	BASIS
PD24202	ECT2L	cna	gain	BASIS
PD24206	ECT2L	cna	hetloss	BASIS
PD3890	ECT2L	cna	hetloss	BASIS
PD3905	ECT2L	cna	gain	BASIS
PD4006	ECT2L	cna	hetloss	BASIS
PD4107	ECT2L	cna	gain	BASIS
PD4826	ECT2L	cna	gain	BASIS
PD4967	ECT2L	cna	homdel	BASIS
PD5935	ECT2L	cna	gain	BASIS
PD5945	ECT2L	cna	amp	BASIS
PD6406	ECT2L	cna	hetloss	BASIS
PD6731	ECT2L	cna	hetloss	BASIS
PD7067	ECT2L	cna	amp	BASIS
PD7215	ECT2L	cna	gain	BASIS
PD8980	ECT2L	cna	gain	BASIS
PD9004	ECT2L	cna	homdel	BASIS
PD9585	ECT2L	cna	hetloss	BASIS
PD9702	ECT2L	cna	gain	BASIS

TCGA-A2-A25B	ESR1	cna	hetloss	TCGA
TCGA-AN-A0XU	ESR1	cna	gain	TCGA
TCGA-AO-A0JL	ESR1	cna	hetloss	TCGA
TCGA-BH-A0AW	ESR1	cna	amp	TCGA
TCGA-BH-A0C0	ESR1	cna	amp	TCGA
TCGA-BH-A18R	ESR1	cna	amp	TCGA
TCGA-C8-A12L	ESR1	cna	gain	TCGA
TCGA-D8-A27M	ESR1	cna	hetloss	TCGA
MB-2827	ESR1	cna	hetloss	METABRIC
MB-5070	ESR1	cna	gain	METABRIC
MB-5107	ESR1	cna	hetloss	METABRIC
MB-6098	ESR1	cna	hetloss	METABRIC
MB-7032	ESR1	cna	hetloss	METABRIC
MB-7038	ESR1	cna	amp	METABRIC
MB-7048	ESR1	cna	hetloss	METABRIC
MB-0420	ESR1	cna	hetloss	METABRIC
PD11327	ESR1	cna	gain	BASIS
PD13296	ESR1	cna	hetloss	BASIS
PD13297	ESR1	cna	hetloss	BASIS
PD13299	ESR1	cna	gain	BASIS
PD23562	ESR1	cna	gain	BASIS
PD23574	ESR1	cna	gain	BASIS
PD24186	ESR1	cna	gain	BASIS
PD24202	ESR1	cna	gain	BASIS
PD3890	ESR1	cna	hetloss	BASIS
PD3905	ESR1	cna	gain	BASIS
PD4005	ESR1	cna	hetloss	BASIS
PD4006	ESR1	cna	gain	BASIS
PD4826	ESR1	cna	gain	BASIS
PD4967	ESR1	cna	hetloss	BASIS
PD5935	ESR1	cna	gain	BASIS
PD5945	ESR1	cna	amp	BASIS
PD6406	ESR1	cna	hetloss	BASIS
PD6413	ESR1	cna	hetloss	BASIS
PD6731	ESR1	cna	hetloss	BASIS
PD7067	ESR1	cna	amp	BASIS
PD7215	ESR1	cna	gain	BASIS
PD8980	ESR1	cna	gain	BASIS
PD9004	ESR1	cna	gain	BASIS
PD9585	ESR1	cna	hetloss	BASIS
PD9702	ESR1	cna	gain	BASIS
TCGA-AN-A0XU	FBXO31	cna	amp	TCGA
TCGA-AO-A0JL	FBXO31	cna	gain	TCGA
TCGA-BH-A0AW	FBXO31	cna	hetloss	TCGA
TCGA-BH-A0C0	FBXO31	cna	hetloss	TCGA
TCGA-C8-A12L	FBXO31	cna	hetloss	TCGA
TCGA-D8-A27M	FBXO31	cna	gain	TCGA

TCGA-E2-A1L7	FBXO31	cna	hetloss	TCGA
TCGA-E9-A1NC	FBXO31	cna	hetloss	TCGA
TCGA-LL-A5YP	FBXO31	cna	gain	TCGA
MB-0346	FBXO31	cna	gain	METABRIC
MB-5107	FBXO31	cna	hetloss	METABRIC
MB-5465	FBXO31	cna	hetloss	METABRIC
MB-6271	FBXO31	cna	hetloss	METABRIC
MB-7032	FBXO31	cna	hetloss	METABRIC
MB-7038	FBXO31	cna	amp	METABRIC
MB-7048	FBXO31	cna	hetloss	METABRIC
MB-0420	FBXO31	cna	hetloss	METABRIC
PD10014	FBXO31	cna	hetloss	BASIS
PD11327	FBXO31	cna	gain	BASIS
PD11742	FBXO31	cna	hetloss	BASIS
PD13296	FBXO31	cna	hetloss	BASIS
PD13297	FBXO31	cna	hetloss	BASIS
PD13299	FBXO31	cna	gain	BASIS
PD14442	FBXO31	cna	hetloss	BASIS
PD23562	FBXO31	cna	gain	BASIS
PD23574	FBXO31	cna	gain	BASIS
PD24202	FBXO31	cna	hetloss	BASIS
PD3890	FBXO31	cna	gain	BASIS
PD3905	FBXO31	cna	gain	BASIS
PD4005	FBXO31	cna	hetloss	BASIS
PD4006	FBXO31	cna	gain	BASIS
PD4107	FBXO31	cna	amp	BASIS
PD4967	FBXO31	cna	hetloss	BASIS
PD5930	FBXO31	cna	gain	BASIS
PD5945	FBXO31	cna	gain	BASIS
PD5948	FBXO31	cna	gain	BASIS
PD7215	FBXO31	cna	gain	BASIS
PD8621	FBXO31	cna	gain	BASIS
PD9004	FBXO31	cna	gain	BASIS
PD9585	FBXO31	cna	hetloss	BASIS
PD9702	FBXO31	cna	gain	BASIS
TCGA-A2-A25B	FBXW7	cna	gain	TCGA
TCGA-AN-A0XU	FBXW7	cna	hetloss	TCGA
TCGA-BH-A0AW	FBXW7	cna	gain	TCGA
TCGA-BH-A0C0	FBXW7	cna	hetloss	TCGA
TCGA-BH-A18R	FBXW7	cna	amp	TCGA
TCGA-BH-A1FU	FBXW7	cna	gain	TCGA
TCGA-C8-A12L	FBXW7	cna	hetloss	TCGA
TCGA-D8-A27M	FBXW7	cna	hetloss	TCGA
TCGA-E2-A1L7	FBXW7	cna	hetloss	TCGA
TCGA-E9-A1NC	FBXW7	cna	hetloss	TCGA
MB-2827	FBXW7	cna	hetloss	METABRIC
MB-5070	FBXW7	cna	hetloss	METABRIC

MB-5107	FBXW7	cna	gain	METABRIC
MB-5465	FBXW7	cna	hetloss	METABRIC
MB-7038	FBXW7	cna	hetloss	METABRIC
MB-7048	FBXW7	cna	hetloss	METABRIC
MB-0420	FBXW7	cna	hetloss	METABRIC
MTS-T0064	FBXW7	cna	gain	METABRIC
PD10014	FBXW7	cna	hetloss	BASIS
PD11742	FBXW7	cna	hetloss	BASIS
PD13296	FBXW7	cna	hetloss	BASIS
PD13297	FBXW7	cna	hetloss	BASIS
PD22355	FBXW7	cna	hetloss	BASIS
PD23562	FBXW7	cna	gain	BASIS
PD23574	FBXW7	cna	hetloss	BASIS
PD23578	FBXW7	cna	hetloss	BASIS
PD24202	FBXW7	cna	hetloss	BASIS
PD24206	FBXW7	cna	gain	BASIS
PD3905	FBXW7	cna	gain	BASIS
PD4005	FBXW7	cna	hetloss	BASIS
PD4107	FBXW7	cna	hetloss	BASIS
PD4826	FBXW7	cna	gain	BASIS
PD5930	FBXW7	cna	gain	BASIS
PD5945	FBXW7	cna	gain	BASIS
PD5948	FBXW7	cna	hetloss	BASIS
PD6406	FBXW7	cna	hetloss	BASIS
PD6731	FBXW7	cna	hetloss	BASIS
PD7067	FBXW7	cna	gain	BASIS
PD7215	FBXW7	cna	amp	BASIS
PD8980	FBXW7	cna	hetloss	BASIS
PD9585	FBXW7	cna	hetloss	BASIS
TCGA-AN-A0XU	FHIT	cna	hetloss	TCGA
TCGA-BH-A0C0	FHIT	cna	hetloss	TCGA
TCGA-BH-A18R	FHIT	cna	homdel	TCGA
TCGA-BH-A1FU	FHIT	cna	hetloss	TCGA
TCGA-C8-A12L	FHIT	cna	hetloss	TCGA
TCGA-D8-A27M	FHIT	cna	hetloss	TCGA
TCGA-E2-A1L7	FHIT	cna	homdel	TCGA
TCGA-E9-A1NC	FHIT	cna	hetloss	TCGA
TCGA-LL-A5YP	FHIT	cna	hetloss	TCGA
MB-0346	FHIT	cna	hetloss	METABRIC
MB-2827	FHIT	cna	hetloss	METABRIC
MB-6098	FHIT	cna	hetloss	METABRIC
MB-7048	FHIT	cna	hetloss	METABRIC
MB-0420	FHIT	cna	hetloss	METABRIC
MTS-T0064	FHIT	cna	hetloss	METABRIC
PD10014	FHIT	cna	hetloss	BASIS
PD11327	FHIT	cna	gain	BASIS
PD11742	FHIT	cna	hetloss	BASIS

PD13296	FHIT	cna	hetloss	BASIS
PD13297	FHIT	cna	hetloss	BASIS
PD13299	FHIT	cna	gain	BASIS
PD13771	FHIT	cna	hetloss	BASIS
PD22355	FHIT	cna	hetloss	BASIS
PD23561	FHIT	cna	hetloss	BASIS
PD23578	FHIT	cna	hetloss	BASIS
PD24202	FHIT	cna	hetloss	BASIS
PD24337	FHIT	cna	hetloss	BASIS
PD3890	FHIT	cna	hetloss	BASIS
PD3905	FHIT	cna	gain	BASIS
PD4005	FHIT	cna	homdel	BASIS
PD4107	FHIT	cna	gain	BASIS
PD4826	FHIT	cna	gain	BASIS
PD5945	FHIT	cna	gain	BASIS
PD5948	FHIT	cna	hetloss	BASIS
PD6406	FHIT	cna	hetloss	BASIS
PD6413	FHIT	cna	hetloss	BASIS
PD6731	FHIT	cna	hetloss	BASIS
PD7067	FHIT	cna	hetloss	BASIS
PD8980	FHIT	cna	hetloss	BASIS
PD9004	FHIT	cna	hetloss	BASIS
PD9585	FHIT	cna	hetloss	BASIS
TCGA-AO-A0JL	FKBP1A	cna	gain	TCGA
TCGA-BH-A0AW	FKBP1A	cna	gain	TCGA
TCGA-BH-A0C0	FKBP1A	cna	hetloss	TCGA
TCGA-BH-A18R	FKBP1A	cna	homdel	TCGA
TCGA-C8-A12L	FKBP1A	cna	hetloss	TCGA
TCGA-D8-A27M	FKBP1A	cna	gain	TCGA
TCGA-E2-A1L7	FKBP1A	cna	gain	TCGA
TCGA-LL-A5YP	FKBP1A	cna	hetloss	TCGA
MB-0346	FKBP1A	cna	gain	METABRIC
MB-5070	FKBP1A	cna	amp	METABRIC
MB-6060	FKBP1A	cna	gain	METABRIC
MB-6098	FKBP1A	cna	hetloss	METABRIC
MB-6271	FKBP1A	cna	hetloss	METABRIC
MB-7032	FKBP1A	cna	gain	METABRIC
MB-7048	FKBP1A	cna	gain	METABRIC
MB-0420	FKBP1A	cna	hetloss	METABRIC
MTS-T0064	FKBP1A	cna	gain	METABRIC
PD10014	FKBP1A	cna	gain	BASIS
PD11327	FKBP1A	cna	amp	BASIS
PD11742	FKBP1A	cna	hetloss	BASIS
PD13296	FKBP1A	cna	hetloss	BASIS
PD13299	FKBP1A	cna	gain	BASIS
PD22355	FKBP1A	cna	gain	BASIS
PD23574	FKBP1A	cna	gain	BASIS

PD23578	FKBP1A	cna	hetloss	BASIS
PD24186	FKBP1A	cna	amp	BASIS
PD24202	FKBP1A	cna	hetloss	BASIS
PD24337	FKBP1A	cna	hetloss	BASIS
PD3890	FKBP1A	cna	hetloss	BASIS
PD3905	FKBP1A	cna	gain	BASIS
PD4005	FKBP1A	cna	hetloss	BASIS
PD4107	FKBP1A	cna	gain	BASIS
PD4826	FKBP1A	cna	hetloss	BASIS
PD5935	FKBP1A	cna	gain	BASIS
PD5945	FKBP1A	cna	amp	BASIS
PD5948	FKBP1A	cna	gain	BASIS
PD6406	FKBP1A	cna	hetloss	BASIS
PD7215	FKBP1A	cna	gain	BASIS
PD8621	FKBP1A	cna	gain	BASIS
PD8980	FKBP1A	cna	hetloss	BASIS
PD9702	FKBP1A	cna	gain	BASIS
TCGA-A2-A25B	FOXO3	cna	hetloss	TCGA
TCGA-AN-A0XU	FOXO3	cna	amp	TCGA
TCGA-AO-A0JL	FOXO3	cna	hetloss	TCGA
TCGA-BH-A0AW	FOXO3	cna	gain	TCGA
TCGA-BH-A18R	FOXO3	cna	amp	TCGA
TCGA-C8-A12L	FOXO3	cna	gain	TCGA
TCGA-D8-A27M	FOXO3	cna	hetloss	TCGA
TCGA-E2-A1L7	FOXO3	cna	gain	TCGA
MB-2827	FOXO3	cna	amp	METABRIC
MB-5107	FOXO3	cna	hetloss	METABRIC
MB-6098	FOXO3	cna	hetloss	METABRIC
MB-7032	FOXO3	cna	hetloss	METABRIC
MB-7038	FOXO3	cna	gain	METABRIC
MB-7048	FOXO3	cna	hetloss	METABRIC
MTS-T0064	FOXO3	cna	amp	METABRIC
PD11327	FOXO3	cna	gain	BASIS
PD13296	FOXO3	cna	gain	BASIS
PD13299	FOXO3	cna	gain	BASIS
PD13771	FOXO3	cna	hetloss	BASIS
PD22355	FOXO3	cna	gain	BASIS
PD23562	FOXO3	cna	gain	BASIS
PD23574	FOXO3	cna	gain	BASIS
PD24186	FOXO3	cna	gain	BASIS
PD24206	FOXO3	cna	gain	BASIS
PD24337	FOXO3	cna	hetloss	BASIS
PD3905	FOXO3	cna	gain	BASIS
PD4006	FOXO3	cna	gain	BASIS
PD4107	FOXO3	cna	hetloss	BASIS
PD4826	FOXO3	cna	gain	BASIS
PD5930	FOXO3	cna	hetloss	BASIS

PD5935	FOXO3	cna	gain	BASIS
PD5945	FOXO3	cna	amp	BASIS
PD6406	FOXO3	cna	hetloss	BASIS
PD6731	FOXO3	cna	hetloss	BASIS
PD7067	FOXO3	cna	gain	BASIS
PD7215	FOXO3	cna	gain	BASIS
PD8621	FOXO3	cna	gain	BASIS
PD8980	FOXO3	cna	gain	BASIS
PD9004	FOXO3	cna	gain	BASIS
PD9585	FOXO3	cna	hetloss	BASIS
PD9702	FOXO3	cna	gain	BASIS
TCGA-A2-A25B	GID4	cna	hetloss	TCGA
TCGA-AN-A0XU	GID4	cna	gain	TCGA
TCGA-AO-A0JL	GID4	cna	gain	TCGA
TCGA-BH-A0AW	GID4	cna	hetloss	TCGA
TCGA-BH-A0C0	GID4	cna	hetloss	TCGA
TCGA-C8-A12L	GID4	cna	hetloss	TCGA
TCGA-D8-A27M	GID4	cna	hetloss	TCGA
TCGA-E2-A1L7	GID4	cna	hetloss	TCGA
TCGA-E9-A1NC	GID4	cna	hetloss	TCGA
TCGA-LL-A5YP	GID4	cna	gain	TCGA
MB-0346	GID4	cna	gain	METABRIC
MB-2827	GID4	cna	hetloss	METABRIC
MB-5070	GID4	cna	hetloss	METABRIC
MB-5107	GID4	cna	hetloss	METABRIC
MB-5465	GID4	cna	hetloss	METABRIC
MB-6098	GID4	cna	hetloss	METABRIC
MB-6271	GID4	cna	hetloss	METABRIC
MB-7038	GID4	cna	hetloss	METABRIC
MB-7048	GID4	cna	hetloss	METABRIC
MB-0420	GID4	cna	hetloss	METABRIC
PD11327	GID4	cna	hetloss	BASIS
PD11742	GID4	cna	hetloss	BASIS
PD13297	GID4	cna	hetloss	BASIS
PD13771	GID4	cna	hetloss	BASIS
PD22355	GID4	cna	hetloss	BASIS
PD23561	GID4	cna	hetloss	BASIS
PD24202	GID4	cna	hetloss	BASIS
PD24206	GID4	cna	hetloss	BASIS
PD24337	GID4	cna	hetloss	BASIS
PD3890	GID4	cna	hetloss	BASIS
PD3905	GID4	cna	gain	BASIS
PD4005	GID4	cna	hetloss	BASIS
PD4107	GID4	cna	gain	BASIS
PD4826	GID4	cna	hetloss	BASIS
PD4967	GID4	cna	hetloss	BASIS
PD5945	GID4	cna	gain	BASIS

PD6406	GID4	cna	hetloss	BASIS
PD6413	GID4	cna	hetloss	BASIS
PD6731	GID4	cna	hetloss	BASIS
PD7067	GID4	cna	gain	BASIS
PD9585	GID4	cna	hetloss	BASIS
TCGA-A2-A25B	GOLGA5	cna	hetloss	TCGA
TCGA-AN-A0XU	GOLGA5	cna	gain	TCGA
TCGA-AO-A0JL	GOLGA5	cna	hetloss	TCGA
TCGA-BH-A0AW	GOLGA5	cna	hetloss	TCGA
TCGA-BH-A1FU	GOLGA5	cna	gain	TCGA
TCGA-C8-A12L	GOLGA5	cna	gain	TCGA
TCGA-D8-A27M	GOLGA5	cna	hetloss	TCGA
TCGA-E2-A1L7	GOLGA5	cna	gain	TCGA
TCGA-E9-A1NC	GOLGA5	cna	hetloss	TCGA
TCGA-LL-A5YP	GOLGA5	cna	hetloss	TCGA
MB-0346	GOLGA5	cna	gain	METABRIC
MB-2827	GOLGA5	cna	hetloss	METABRIC
MB-5070	GOLGA5	cna	hetloss	METABRIC
MB-5107	GOLGA5	cna	hetloss	METABRIC
MB-6098	GOLGA5	cna	hetloss	METABRIC
MTS-T0064	GOLGA5	cna	hetloss	METABRIC
PD10014	GOLGA5	cna	hetloss	BASIS
PD11327	GOLGA5	cna	hetloss	BASIS
PD11742	GOLGA5	cna	hetloss	BASIS
PD13296	GOLGA5	cna	gain	BASIS
PD13297	GOLGA5	cna	hetloss	BASIS
PD13771	GOLGA5	cna	hetloss	BASIS
PD23562	GOLGA5	cna	gain	BASIS
PD23574	GOLGA5	cna	gain	BASIS
PD23578	GOLGA5	cna	hetloss	BASIS
PD24202	GOLGA5	cna	hetloss	BASIS
PD24206	GOLGA5	cna	gain	BASIS
PD24337	GOLGA5	cna	hetloss	BASIS
PD3890	GOLGA5	cna	hetloss	BASIS
PD3905	GOLGA5	cna	gain	BASIS
PD4005	GOLGA5	cna	hetloss	BASIS
PD4006	GOLGA5	cna	gain	BASIS
PD4826	GOLGA5	cna	gain	BASIS
PD4967	GOLGA5	cna	hetloss	BASIS
PD5945	GOLGA5	cna	gain	BASIS
PD5948	GOLGA5	cna	gain	BASIS
PD6406	GOLGA5	cna	hetloss	BASIS
PD6413	GOLGA5	cna	hetloss	BASIS
PD7067	GOLGA5	cna	gain	BASIS
PD8980	GOLGA5	cna	hetloss	BASIS
PD9702	GOLGA5	cna	gain	BASIS
TCGA-A2-A25B	KLF6	cna	gain	TCGA

TCGA-AN-A0XU	KLF6	cna	amp	TCGA
TCGA-AO-A0JL	KLF6	cna	gain	TCGA
TCGA-BH-A0AW	KLF6	cna	gain	TCGA
TCGA-BH-A0C0	KLF6	cna	gain	TCGA
TCGA-C8-A12L	KLF6	cna	gain	TCGA
MB-5070	KLF6	cna	gain	METABRIC
MB-5465	KLF6	cna	amp	METABRIC
MB-6098	KLF6	cna	amp	METABRIC
MB-7038	KLF6	cna	hetloss	METABRIC
PD10014	KLF6	cna	amp	BASIS
PD11327	KLF6	cna	amp	BASIS
PD13296	KLF6	cna	gain	BASIS
PD13297	KLF6	cna	gain	BASIS
PD13299	KLF6	cna	gain	BASIS
PD13771	KLF6	cna	gain	BASIS
PD22355	KLF6	cna	gain	BASIS
PD23562	KLF6	cna	amp	BASIS
PD23574	KLF6	cna	gain	BASIS
PD24186	KLF6	cna	amp	BASIS
PD24202	KLF6	cna	gain	BASIS
PD24206	KLF6	cna	gain	BASIS
PD24337	KLF6	cna	gain	BASIS
PD3905	KLF6	cna	gain	BASIS
PD4005	KLF6	cna	gain	BASIS
PD4006	KLF6	cna	amp	BASIS
PD4107	KLF6	cna	gain	BASIS
PD4826	KLF6	cna	gain	BASIS
PD5930	KLF6	cna	gain	BASIS
PD5935	KLF6	cna	gain	BASIS
PD5945	KLF6	cna	amp	BASIS
PD5948	KLF6	cna	gain	BASIS
PD6406	KLF6	cna	hetloss	BASIS
PD6413	KLF6	cna	gain	BASIS
PD6731	KLF6	cna	hetloss	BASIS
PD7067	KLF6	cna	amp	BASIS
PD7215	KLF6	cna	gain	BASIS
PD8621	KLF6	cna	gain	BASIS
PD8980	KLF6	cna	hetloss	BASIS
PD9004	KLF6	cna	gain	BASIS
PD9702	KLF6	cna	amp	BASIS
TCGA-A2-A25B	MAP3K4	cna	hetloss	TCGA
TCGA-AN-A0XU	MAP3K4	cna	gain	TCGA
TCGA-AO-A0JL	MAP3K4	cna	hetloss	TCGA
TCGA-BH-A0AW	MAP3K4	cna	amp	TCGA
TCGA-BH-A0C0	MAP3K4	cna	hetloss	TCGA
TCGA-BH-A18R	MAP3K4	cna	homdel	TCGA
TCGA-C8-A12L	MAP3K4	cna	hetloss	TCGA

TCGA-D8-A27M	MAP3K4	cna	hetloss	TCGA
TCGA-E9-A1NC	MAP3K4	cna	hetloss	TCGA
MB-2827	MAP3K4	cna	hetloss	METABRIC
MB-5107	MAP3K4	cna	hetloss	METABRIC
MB-6060	MAP3K4	cna	hetloss	METABRIC
MB-6098	MAP3K4	cna	hetloss	METABRIC
MB-7048	MAP3K4	cna	hetloss	METABRIC
MB-0420	MAP3K4	cna	hetloss	METABRIC
MTS-T0064	MAP3K4	cna	hetloss	METABRIC
PD10014	MAP3K4	cna	hetloss	BASIS
PD13296	MAP3K4	cna	homdel	BASIS
PD13297	MAP3K4	cna	hetloss	BASIS
PD13299	MAP3K4	cna	gain	BASIS
PD23562	MAP3K4	cna	gain	BASIS
PD23574	MAP3K4	cna	amp	BASIS
PD24186	MAP3K4	cna	gain	BASIS
PD24206	MAP3K4	cna	gain	BASIS
PD3890	MAP3K4	cna	hetloss	BASIS
PD3905	MAP3K4	cna	gain	BASIS
PD4005	MAP3K4	cna	hetloss	BASIS
PD4006	MAP3K4	cna	gain	BASIS
PD4107	MAP3K4	cna	gain	BASIS
PD4826	MAP3K4	cna	gain	BASIS
PD4967	MAP3K4	cna	hetloss	BASIS
PD5935	MAP3K4	cna	gain	BASIS
PD5945	MAP3K4	cna	gain	BASIS
PD6406	MAP3K4	cna	hetloss	BASIS
PD6413	MAP3K4	cna	hetloss	BASIS
PD6731	MAP3K4	cna	hetloss	BASIS
PD7067	MAP3K4	cna	amp	BASIS
PD8980	MAP3K4	cna	gain	BASIS
PD9004	MAP3K4	cna	gain	BASIS
PD9585	MAP3K4	cna	hetloss	BASIS
PD9702	MAP3K4	cna	gain	BASIS
TCGA-A2-A25B	MIR142	cna	amp	TCGA
TCGA-AN-A0XU	MIR142	cna	gain	TCGA
TCGA-AO-A0JL	MIR142	cna	hetloss	TCGA
TCGA-BH-A0AW	MIR142	cna	amp	TCGA
TCGA-BH-A0C0	MIR142	cna	gain	TCGA
TCGA-BH-A18R	MIR142	cna	amp	TCGA
TCGA-C8-A12L	MIR142	cna	gain	TCGA
TCGA-D8-A27M	MIR142	cna	hetloss	TCGA
TCGA-EW-A1OX	MIR142	cna	amp	TCGA
MB-0346	MIR142	cna	gain	METABRIC
MB-2827	MIR142	cna	hetloss	METABRIC
MB-5070	MIR142	cna	hetloss	METABRIC
MB-5107	MIR142	cna	amp	METABRIC

MB-5323	MIR142	cna	gain	METABRIC
MB-6060	MIR142	cna	hetloss	METABRIC
MB-6098	MIR142	cna	hetloss	METABRIC
MB-6271	MIR142	cna	hetloss	METABRIC
MB-7048	MIR142	cna	hetloss	METABRIC
PD11327	MIR142	cna	gain	BASIS
PD11742	MIR142	cna	gain	BASIS
PD13299	MIR142	cna	gain	BASIS
PD22355	MIR142	cna	gain	BASIS
PD23562	MIR142	cna	gain	BASIS
PD23574	MIR142	cna	gain	BASIS
PD24186	MIR142	cna	gain	BASIS
PD24337	MIR142	cna	hetloss	BASIS
PD3890	MIR142	cna	hetloss	BASIS
PD3905	MIR142	cna	gain	BASIS
PD4006	MIR142	cna	amp	BASIS
PD4967	MIR142	cna	gain	BASIS
PD5930	MIR142	cna	gain	BASIS
PD5945	MIR142	cna	amp	BASIS
PD5948	MIR142	cna	gain	BASIS
PD6406	MIR142	cna	hetloss	BASIS
PD6731	MIR142	cna	hetloss	BASIS
PD7067	MIR142	cna	gain	BASIS
PD7215	MIR142	cna	gain	BASIS
PD8621	MIR142	cna	gain	BASIS
PD8980	MIR142	cna	gain	BASIS
PD9004	MIR142	cna	gain	BASIS
PD9702	MIR142	cna	gain	BASIS
TCGA-AN-A0XU	NCKIPSD	cna	hetloss	TCGA
TCGA-BH-A0C0	NCKIPSD	cna	hetloss	TCGA
TCGA-BH-A18R	NCKIPSD	cna	gain	TCGA
TCGA-BH-A1FU	NCKIPSD	cna	hetloss	TCGA
TCGA-C8-A12L	NCKIPSD	cna	hetloss	TCGA
TCGA-D8-A27M	NCKIPSD	cna	hetloss	TCGA
TCGA-E2-A1L7	NCKIPSD	cna	hetloss	TCGA
TCGA-E9-A1NC	NCKIPSD	cna	hetloss	TCGA
TCGA-LL-A5YP	NCKIPSD	cna	hetloss	TCGA
MB-0346	NCKIPSD	cna	hetloss	METABRIC
MB-2827	NCKIPSD	cna	hetloss	METABRIC
MB-5107	NCKIPSD	cna	hetloss	METABRIC
MB-5465	NCKIPSD	cna	hetloss	METABRIC
MB-7038	NCKIPSD	cna	hetloss	METABRIC
MB-7048	NCKIPSD	cna	hetloss	METABRIC
MB-0420	NCKIPSD	cna	hetloss	METABRIC
PD10014	NCKIPSD	cna	hetloss	BASIS
PD11327	NCKIPSD	cna	homdel	BASIS
PD11742	NCKIPSD	cna	hetloss	BASIS

PD13296	NCKIPSD	cna	hetloss	BASIS
PD13297	NCKIPSD	cna	hetloss	BASIS
PD13299	NCKIPSD	cna	gain	BASIS
PD13771	NCKIPSD	cna	hetloss	BASIS
PD14442	NCKIPSD	cna	hetloss	BASIS
PD22355	NCKIPSD	cna	hetloss	BASIS
PD23561	NCKIPSD	cna	hetloss	BASIS
PD23578	NCKIPSD	cna	hetloss	BASIS
PD24202	NCKIPSD	cna	hetloss	BASIS
PD24206	NCKIPSD	cna	hetloss	BASIS
PD24337	NCKIPSD	cna	hetloss	BASIS
PD3890	NCKIPSD	cna	hetloss	BASIS
PD3905	NCKIPSD	cna	gain	BASIS
PD4005	NCKIPSD	cna	hetloss	BASIS
PD4826	NCKIPSD	cna	gain	BASIS
PD5945	NCKIPSD	cna	gain	BASIS
PD6406	NCKIPSD	cna	hetloss	BASIS
PD6413	NCKIPSD	cna	hetloss	BASIS
PD6731	NCKIPSD	cna	hetloss	BASIS
PD7067	NCKIPSD	cna	gain	BASIS
PD8980	NCKIPSD	cna	hetloss	BASIS
PD9585	NCKIPSD	cna	hetloss	BASIS
TCGA-A2-A25B	NCOA2	cna	gain	TCGA
TCGA-AO-A0JL	NCOA2	cna	amp	TCGA
TCGA-BH-A0AW	NCOA2	cna	gain	TCGA
TCGA-BH-A0C0	NCOA2	cna	amp	TCGA
TCGA-BH-A18R	NCOA2	cna	homdel	TCGA
TCGA-BH-A1FU	NCOA2	cna	gain	TCGA
TCGA-D8-A27M	NCOA2	cna	amp	TCGA
TCGA-E2-A1L7	NCOA2	cna	gain	TCGA
TCGA-LL-A5YP	NCOA2	cna	amp	TCGA
MB-0346	NCOA2	cna	amp	METABRIC
MB-2827	NCOA2	cna	amp	METABRIC
MB-5465	NCOA2	cna	hetloss	METABRIC
MB-6060	NCOA2	cna	gain	METABRIC
MB-6098	NCOA2	cna	amp	METABRIC
MB-7038	NCOA2	cna	gain	METABRIC
MB-0420	NCOA2	cna	gain	METABRIC
PD10014	NCOA2	cna	amp	BASIS
PD11327	NCOA2	cna	amp	BASIS
PD13296	NCOA2	cna	homdel	BASIS
PD13299	NCOA2	cna	gain	BASIS
PD13771	NCOA2	cna	gain	BASIS
PD23562	NCOA2	cna	gain	BASIS
PD23574	NCOA2	cna	gain	BASIS
PD24186	NCOA2	cna	gain	BASIS
PD24206	NCOA2	cna	amp	BASIS

PD3890	NCOA2	cna	gain	BASIS
PD3905	NCOA2	cna	gain	BASIS
PD4005	NCOA2	cna	hetloss	BASIS
PD4006	NCOA2	cna	gain	BASIS
PD4826	NCOA2	cna	amp	BASIS
PD5935	NCOA2	cna	gain	BASIS
PD5945	NCOA2	cna	gain	BASIS
PD5948	NCOA2	cna	gain	BASIS
PD6406	NCOA2	cna	gain	BASIS
PD7067	NCOA2	cna	amp	BASIS
PD7215	NCOA2	cna	amp	BASIS
PD8621	NCOA2	cna	gain	BASIS
PD8980	NCOA2	cna	gain	BASIS
PD9004	NCOA2	cna	gain	BASIS
PD9585	NCOA2	cna	hetloss	BASIS
PD9702	NCOA2	cna	gain	BASIS
TCGA-A2-A25B	PARK2	cna	hetloss	TCGA
TCGA-AN-A0XU	PARK2	cna	gain	TCGA
TCGA-AO-A0JL	PARK2	cna	hetloss	TCGA
TCGA-BH-A0AW	PARK2	cna	amp	TCGA
TCGA-BH-A0C0	PARK2	cna	hetloss	TCGA
TCGA-BH-A18R	PARK2	cna	homdel	TCGA
TCGA-C8-A12L	PARK2	cna	hetloss	TCGA
TCGA-D8-A27M	PARK2	cna	hetloss	TCGA
TCGA-E9-A1NC	PARK2	cna	hetloss	TCGA
MB-2827	PARK2	cna	hetloss	METABRIC
MB-5107	PARK2	cna	hetloss	METABRIC
MB-6060	PARK2	cna	hetloss	METABRIC
MB-6098	PARK2	cna	hetloss	METABRIC
MB-7048	PARK2	cna	hetloss	METABRIC
MB-0420	PARK2	cna	hetloss	METABRIC
MTS-T0064	PARK2	cna	hetloss	METABRIC
PD10014	PARK2	cna	hetloss	BASIS
PD13296	PARK2	cna	homdel	BASIS
PD13297	PARK2	cna	hetloss	BASIS
PD13299	PARK2	cna	gain	BASIS
PD23562	PARK2	cna	gain	BASIS
PD23574	PARK2	cna	amp	BASIS
PD24186	PARK2	cna	gain	BASIS
PD24206	PARK2	cna	gain	BASIS
PD3890	PARK2	cna	hetloss	BASIS
PD3905	PARK2	cna	gain	BASIS
PD4005	PARK2	cna	hetloss	BASIS
PD4006	PARK2	cna	gain	BASIS
PD4107	PARK2	cna	gain	BASIS
PD4826	PARK2	cna	gain	BASIS
PD4967	PARK2	cna	hetloss	BASIS

PD5935	PARK2	cna	gain	BASIS
PD5945	PARK2	cna	gain	BASIS
PD6406	PARK2	cna	hetloss	BASIS
PD6413	PARK2	cna	hetloss	BASIS
PD6731	PARK2	cna	hetloss	BASIS
PD7067	PARK2	cna	amp	BASIS
PD8980	PARK2	cna	gain	BASIS
PD9004	PARK2	cna	gain	BASIS
PD9585	PARK2	cna	hetloss	BASIS
PD9702	PARK2	cna	gain	BASIS
TCGA-AN-A0XU	PARP3	cna	hetloss	TCGA
TCGA-BH-A0C0	PARP3	cna	hetloss	TCGA
TCGA-BH-A18R	PARP3	cna	homdel	TCGA
TCGA-BH-A1FU	PARP3	cna	hetloss	TCGA
TCGA-C8-A12L	PARP3	cna	hetloss	TCGA
TCGA-D8-A27M	PARP3	cna	hetloss	TCGA
TCGA-E2-A1L7	PARP3	cna	hetloss	TCGA
TCGA-E9-A1NC	PARP3	cna	hetloss	TCGA
TCGA-LL-A5YP	PARP3	cna	hetloss	TCGA
MB-0346	PARP3	cna	hetloss	METABRIC
MB-2827	PARP3	cna	hetloss	METABRIC
MB-5465	PARP3	cna	hetloss	METABRIC
MB-6098	PARP3	cna	hetloss	METABRIC
MB-7038	PARP3	cna	hetloss	METABRIC
MB-7048	PARP3	cna	hetloss	METABRIC
MB-0420	PARP3	cna	hetloss	METABRIC
PD10014	PARP3	cna	hetloss	BASIS
PD11327	PARP3	cna	hetloss	BASIS
PD13296	PARP3	cna	hetloss	BASIS
PD13297	PARP3	cna	hetloss	BASIS
PD13299	PARP3	cna	gain	BASIS
PD13771	PARP3	cna	hetloss	BASIS
PD14442	PARP3	cna	hetloss	BASIS
PD22355	PARP3	cna	hetloss	BASIS
PD23561	PARP3	cna	hetloss	BASIS
PD23578	PARP3	cna	hetloss	BASIS
PD24202	PARP3	cna	hetloss	BASIS
PD24206	PARP3	cna	hetloss	BASIS
PD24337	PARP3	cna	hetloss	BASIS
PD3890	PARP3	cna	hetloss	BASIS
PD3905	PARP3	cna	gain	BASIS
PD4005	PARP3	cna	hetloss	BASIS
PD4826	PARP3	cna	gain	BASIS
PD4967	PARP3	cna	hetloss	BASIS
PD5945	PARP3	cna	gain	BASIS
PD6406	PARP3	cna	hetloss	BASIS
PD6413	PARP3	cna	hetloss	BASIS

PD6731	PARP3	cna	hetloss	BASIS
PD7067	PARP3	cna	gain	BASIS
PD8980	PARP3	cna	hetloss	BASIS
PD9585	PARP3	cna	hetloss	BASIS
TCGA-A2-A25B	PIK3CA	cna	gain	TCGA
TCGA-AN-A0XU	PIK3CA	cna	gain	TCGA
TCGA-BH-A0AW	PIK3CA	cna	gain	TCGA
TCGA-BH-A0C0	PIK3CA	cna	gain	TCGA
TCGA-BH-A1FU	PIK3CA	cna	hetloss	TCGA
TCGA-C8-A12L	PIK3CA	cna	amp	TCGA
TCGA-E2-A1L7	PIK3CA	cna	gain	TCGA
TCGA-LL-A5YP	PIK3CA	cna	gain	TCGA
MB-0346	PIK3CA	cna	gain	METABRIC
MB-5070	PIK3CA	cna	amp	METABRIC
MB-5107	PIK3CA	cna	amp	METABRIC
MB-6060	PIK3CA	cna	gain	METABRIC
MB-7038	PIK3CA	cna	hetloss	METABRIC
MB-0420	PIK3CA	cna	gain	METABRIC
MTS-T0064	PIK3CA	cna	amp	METABRIC
P-0002858-T01-IM3	PIK3CA	cna	amp	MSK-IMPACT
PD11327	PIK3CA	cna	amp	BASIS
PD13296	PIK3CA	cna	gain	BASIS
PD13299	PIK3CA	cna	gain	BASIS
PD13771	PIK3CA	cna	hetloss	BASIS
PD22355	PIK3CA	cna	gain	BASIS
PD23561	PIK3CA	cna	hetloss	BASIS
PD23562	PIK3CA	cna	amp	BASIS
PD23574	PIK3CA	cna	gain	BASIS
PD23578	PIK3CA	cna	gain	BASIS
PD24186	PIK3CA	cna	amp	BASIS
PD24202	PIK3CA	cna	gain	BASIS
PD24206	PIK3CA	cna	gain	BASIS
PD3905	PIK3CA	cna	gain	BASIS
PD4005	PIK3CA	cna	gain	BASIS
PD4107	PIK3CA	cna	gain	BASIS
PD4826	PIK3CA	cna	gain	BASIS
PD5935	PIK3CA	cna	amp	BASIS
PD5945	PIK3CA	cna	amp	BASIS
PD5948	PIK3CA	cna	gain	BASIS
PD6406	PIK3CA	cna	gain	BASIS
PD7067	PIK3CA	cna	gain	BASIS
PD7215	PIK3CA	cna	gain	BASIS
PD8621	PIK3CA	cna	gain	BASIS
PD8980	PIK3CA	cna	gain	BASIS
PD9702	PIK3CA	cna	amp	BASIS
TCGA-A2-A25B	PPP6C	cna	gain	TCGA
TCGA-AN-A0XU	PPP6C	cna	hetloss	TCGA

TCGA-AO-A0JL	PPP6C	cna	hetloss	TCGA
TCGA-BH-A0C0	PPP6C	cna	gain	TCGA
TCGA-C8-A12L	PPP6C	cna	gain	TCGA
TCGA-E2-A1L7	PPP6C	cna	gain	TCGA
TCGA-E9-A1NC	PPP6C	cna	hetloss	TCGA
TCGA-LL-A5YP	PPP6C	cna	hetloss	TCGA
MB-0346	PPP6C	cna	hetloss	METABRIC
MB-2827	PPP6C	cna	hetloss	METABRIC
MB-5070	PPP6C	cna	hetloss	METABRIC
MB-5107	PPP6C	cna	gain	METABRIC
MB-7038	PPP6C	cna	hetloss	METABRIC
MB-0420	PPP6C	cna	hetloss	METABRIC
PD10014	PPP6C	cna	gain	BASIS
PD11327	PPP6C	cna	hetloss	BASIS
PD13297	PPP6C	cna	hetloss	BASIS
PD13299	PPP6C	cna	gain	BASIS
PD23562	PPP6C	cna	gain	BASIS
PD23578	PPP6C	cna	hetloss	BASIS
PD24186	PPP6C	cna	amp	BASIS
PD24202	PPP6C	cna	hetloss	BASIS
PD24206	PPP6C	cna	gain	BASIS
PD24337	PPP6C	cna	hetloss	BASIS
PD3890	PPP6C	cna	hetloss	BASIS
PD3905	PPP6C	cna	gain	BASIS
PD4005	PPP6C	cna	hetloss	BASIS
PD4006	PPP6C	cna	gain	BASIS
PD4107	PPP6C	cna	hetloss	BASIS
PD4967	PPP6C	cna	hetloss	BASIS
PD5930	PPP6C	cna	gain	BASIS
PD5945	PPP6C	cna	gain	BASIS
PD5948	PPP6C	cna	gain	BASIS
PD6406	PPP6C	cna	hetloss	BASIS
PD6731	PPP6C	cna	hetloss	BASIS
PD7067	PPP6C	cna	gain	BASIS
PD7215	PPP6C	cna	gain	BASIS
PD8621	PPP6C	cna	gain	BASIS
PD8980	PPP6C	cna	gain	BASIS
PD9004	PPP6C	cna	hetloss	BASIS
PD9585	PPP6C	cna	hetloss	BASIS
TCGA-A2-A25B	PRDM1	cna	hetloss	TCGA
TCGA-AN-A0XU	PRDM1	cna	amp	TCGA
TCGA-AO-A0JL	PRDM1	cna	hetloss	TCGA
TCGA-BH-A0AW	PRDM1	cna	gain	TCGA
TCGA-BH-A18R	PRDM1	cna	homdel	TCGA
TCGA-C8-A12L	PRDM1	cna	gain	TCGA
TCGA-D8-A27M	PRDM1	cna	hetloss	TCGA
TCGA-E2-A1L7	PRDM1	cna	gain	TCGA

MB-2827	PRDM1	cna	amp	METABRIC
MB-5070	PRDM1	cna	amp	METABRIC
MB-5107	PRDM1	cna	hetloss	METABRIC
MB-6098	PRDM1	cna	hetloss	METABRIC
MB-7032	PRDM1	cna	hetloss	METABRIC
MB-7038	PRDM1	cna	gain	METABRIC
MB-7048	PRDM1	cna	hetloss	METABRIC
MTS-T0064	PRDM1	cna	amp	METABRIC
PD11327	PRDM1	cna	gain	BASIS
PD13296	PRDM1	cna	gain	BASIS
PD13299	PRDM1	cna	gain	BASIS
PD13771	PRDM1	cna	hetloss	BASIS
PD22355	PRDM1	cna	gain	BASIS
PD23562	PRDM1	cna	gain	BASIS
PD23574	PRDM1	cna	gain	BASIS
PD24186	PRDM1	cna	gain	BASIS
PD24206	PRDM1	cna	gain	BASIS
PD24337	PRDM1	cna	hetloss	BASIS
PD3905	PRDM1	cna	gain	BASIS
PD4006	PRDM1	cna	gain	BASIS
PD4107	PRDM1	cna	hetloss	BASIS
PD4826	PRDM1	cna	gain	BASIS
PD5935	PRDM1	cna	gain	BASIS
PD5945	PRDM1	cna	amp	BASIS
PD6406	PRDM1	cna	hetloss	BASIS
PD6731	PRDM1	cna	hetloss	BASIS
PD7067	PRDM1	cna	gain	BASIS
PD7215	PRDM1	cna	gain	BASIS
PD8621	PRDM1	cna	gain	BASIS
PD8980	PRDM1	cna	gain	BASIS
PD9004	PRDM1	cna	gain	BASIS
PD9585	PRDM1	cna	hetloss	BASIS
PD9702	PRDM1	cna	gain	BASIS
TCGA-A2-A25B	PREX2	cna	gain	TCGA
TCGA-AO-A0JL	PREX2	cna	amp	TCGA
TCGA-BH-A0AW	PREX2	cna	gain	TCGA
TCGA-BH-A0C0	PREX2	cna	amp	TCGA
TCGA-BH-A18R	PREX2	cna	homdel	TCGA
TCGA-BH-A1FU	PREX2	cna	gain	TCGA
TCGA-D8-A27M	PREX2	cna	amp	TCGA
TCGA-E2-A1L7	PREX2	cna	gain	TCGA
TCGA-LL-A5YP	PREX2	cna	gain	TCGA
MB-0346	PREX2	cna	amp	METABRIC
MB-2827	PREX2	cna	amp	METABRIC
MB-5465	PREX2	cna	hetloss	METABRIC
MB-6060	PREX2	cna	amp	METABRIC
MB-6098	PREX2	cna	amp	METABRIC

MB-7038	PREX2	cna	gain	METABRIC
MB-0420	PREX2	cna	gain	METABRIC
PD10014	PREX2	cna	amp	BASIS
PD11327	PREX2	cna	amp	BASIS
PD13296	PREX2	cna	homdel	BASIS
PD13299	PREX2	cna	gain	BASIS
PD13771	PREX2	cna	gain	BASIS
PD23562	PREX2	cna	gain	BASIS
PD23574	PREX2	cna	gain	BASIS
PD24186	PREX2	cna	gain	BASIS
PD24206	PREX2	cna	amp	BASIS
PD3890	PREX2	cna	gain	BASIS
PD3905	PREX2	cna	gain	BASIS
PD4005	PREX2	cna	hetloss	BASIS
PD4006	PREX2	cna	gain	BASIS
PD4826	PREX2	cna	amp	BASIS
PD5935	PREX2	cna	gain	BASIS
PD5945	PREX2	cna	gain	BASIS
PD5948	PREX2	cna	gain	BASIS
PD6406	PREX2	cna	gain	BASIS
PD7067	PREX2	cna	amp	BASIS
PD7215	PREX2	cna	amp	BASIS
PD8621	PREX2	cna	gain	BASIS
PD8980	PREX2	cna	gain	BASIS
PD9004	PREX2	cna	gain	BASIS
PD9585	PREX2	cna	hetloss	BASIS
PD9702	PREX2	cna	gain	BASIS
TCGA-AN-A0XU	RAD51	cna	hetloss	TCGA
TCGA-AO-A0JL	RAD51	cna	hetloss	TCGA
TCGA-BH-A0C0	RAD51	cna	hetloss	TCGA
TCGA-C8-A12L	RAD51	cna	hetloss	TCGA
TCGA-D8-A27M	RAD51	cna	hetloss	TCGA
TCGA-E2-A1L7	RAD51	cna	hetloss	TCGA
TCGA-E9-A1NC	RAD51	cna	hetloss	TCGA
TCGA-LL-A5YP	RAD51	cna	hetloss	TCGA
MB-2827	RAD51	cna	hetloss	METABRIC
MB-5070	RAD51	cna	hetloss	METABRIC
MB-5107	RAD51	cna	gain	METABRIC
MB-5465	RAD51	cna	hetloss	METABRIC
MB-6060	RAD51	cna	hetloss	METABRIC
MB-6098	RAD51	cna	hetloss	METABRIC
MB-7048	RAD51	cna	hetloss	METABRIC
MB-0420	RAD51	cna	hetloss	METABRIC
PD10014	RAD51	cna	hetloss	BASIS
PD11327	RAD51	cna	homdel	BASIS
PD11742	RAD51	cna	hetloss	BASIS
PD13297	RAD51	cna	hetloss	BASIS

PD13299	RAD51	cna	hetloss	BASIS
PD22355	RAD51	cna	hetloss	BASIS
PD23574	RAD51	cna	hetloss	BASIS
PD23578	RAD51	cna	hetloss	BASIS
PD24202	RAD51	cna	hetloss	BASIS
PD24206	RAD51	cna	gain	BASIS
PD24337	RAD51	cna	hetloss	BASIS
PD3890	RAD51	cna	hetloss	BASIS
PD3905	RAD51	cna	gain	BASIS
PD4005	RAD51	cna	homdel	BASIS
PD4967	RAD51	cna	hetloss	BASIS
PD5930	RAD51	cna	hetloss	BASIS
PD5945	RAD51	cna	gain	BASIS
PD5948	RAD51	cna	hetloss	BASIS
PD6413	RAD51	cna	hetloss	BASIS
PD7067	RAD51	cna	gain	BASIS
PD7215	RAD51	cna	gain	BASIS
PD8980	RAD51	cna	hetloss	BASIS
PD9004	RAD51	cna	hetloss	BASIS
PD9585	RAD51	cna	hetloss	BASIS
PD9702	RAD51	cna	gain	BASIS
TCGA-A2-A25B	RAP1GDS1	cna	hetloss	TCGA
TCGA-AN-A0XU	RAP1GDS1	cna	hetloss	TCGA
TCGA-AO-A0JL	RAP1GDS1	cna	gain	TCGA
TCGA-BH-A0AW	RAP1GDS1	cna	hetloss	TCGA
TCGA-BH-A0C0	RAP1GDS1	cna	hetloss	TCGA
TCGA-BH-A1FU	RAP1GDS1	cna	gain	TCGA
TCGA-C8-A12L	RAP1GDS1	cna	hetloss	TCGA
TCGA-D8-A27M	RAP1GDS1	cna	gain	TCGA
TCGA-E2-A1L7	RAP1GDS1	cna	gain	TCGA
TCGA-E9-A1NC	RAP1GDS1	cna	hetloss	TCGA
TCGA-LL-A5YP	RAP1GDS1	cna	gain	TCGA
MB-2827	RAP1GDS1	cna	hetloss	METABRIC
MB-5070	RAP1GDS1	cna	hetloss	METABRIC
MB-5465	RAP1GDS1	cna	hetloss	METABRIC
MB-7048	RAP1GDS1	cna	hetloss	METABRIC
MB-0420	RAP1GDS1	cna	hetloss	METABRIC
PD10014	RAP1GDS1	cna	gain	BASIS
PD11742	RAP1GDS1	cna	hetloss	BASIS
PD13296	RAP1GDS1	cna	hetloss	BASIS
PD13297	RAP1GDS1	cna	hetloss	BASIS
PD13771	RAP1GDS1	cna	gain	BASIS
PD23562	RAP1GDS1	cna	gain	BASIS
PD23578	RAP1GDS1	cna	hetloss	BASIS
PD24206	RAP1GDS1	cna	gain	BASIS
PD24337	RAP1GDS1	cna	hetloss	BASIS
PD3890	RAP1GDS1	cna	gain	BASIS

PD3905	RAP1GDS1	cna	gain	BASIS
PD4006	RAP1GDS1	cna	hetloss	BASIS
PD4107	RAP1GDS1	cna	gain	BASIS
PD5930	RAP1GDS1	cna	hetloss	BASIS
PD5935	RAP1GDS1	cna	gain	BASIS
PD5945	RAP1GDS1	cna	gain	BASIS
PD5948	RAP1GDS1	cna	gain	BASIS
PD6406	RAP1GDS1	cna	hetloss	BASIS
PD6413	RAP1GDS1	cna	hetloss	BASIS
PD6731	RAP1GDS1	cna	hetloss	BASIS
PD7067	RAP1GDS1	cna	gain	BASIS
PD7215	RAP1GDS1	cna	gain	BASIS
PD8621	RAP1GDS1	cna	hetloss	BASIS
PD8980	RAP1GDS1	cna	hetloss	BASIS
PD9585	RAP1GDS1	cna	hetloss	BASIS
TCGA-AN-A0XU	RHOA	cna	hetloss	TCGA
TCGA-BH-A0C0	RHOA	cna	hetloss	TCGA
TCGA-BH-A18R	RHOA	cna	gain	TCGA
TCGA-BH-A1FU	RHOA	cna	hetloss	TCGA
TCGA-C8-A12L	RHOA	cna	hetloss	TCGA
TCGA-D8-A27M	RHOA	cna	hetloss	TCGA
TCGA-E2-A1L7	RHOA	cna	hetloss	TCGA
TCGA-E9-A1NC	RHOA	cna	hetloss	TCGA
TCGA-LL-A5YP	RHOA	cna	hetloss	TCGA
MB-0346	RHOA	cna	hetloss	METABRIC
MB-2827	RHOA	cna	hetloss	METABRIC
MB-5107	RHOA	cna	hetloss	METABRIC
MB-5465	RHOA	cna	hetloss	METABRIC
MB-7038	RHOA	cna	hetloss	METABRIC
MB-7048	RHOA	cna	hetloss	METABRIC
MB-0420	RHOA	cna	hetloss	METABRIC
P-0009557-T01-IM5	RHOA	cna	homdel	MSK-IMPACT
PD10014	RHOA	cna	hetloss	BASIS
PD11327	RHOA	cna	homdel	BASIS
PD13296	RHOA	cna	hetloss	BASIS
PD13297	RHOA	cna	hetloss	BASIS
PD13299	RHOA	cna	gain	BASIS
PD13771	RHOA	cna	hetloss	BASIS
PD14442	RHOA	cna	hetloss	BASIS
PD22355	RHOA	cna	hetloss	BASIS
PD23561	RHOA	cna	hetloss	BASIS
PD23578	RHOA	cna	hetloss	BASIS
PD24202	RHOA	cna	hetloss	BASIS
PD24206	RHOA	cna	hetloss	BASIS
PD24337	RHOA	cna	hetloss	BASIS
PD3890	RHOA	cna	hetloss	BASIS
PD3905	RHOA	cna	gain	BASIS

PD4005	RHOA	cna	hetloss	BASIS
PD4826	RHOA	cna	gain	BASIS
PD5945	RHOA	cna	gain	BASIS
PD6406	RHOA	cna	hetloss	BASIS
PD6413	RHOA	cna	hetloss	BASIS
PD6731	RHOA	cna	hetloss	BASIS
PD7067	RHOA	cna	gain	BASIS
PD8980	RHOA	cna	hetloss	BASIS
PD9585	RHOA	cna	hetloss	BASIS
TCGA-A2-A25B	RNF43	cna	amp	TCGA
TCGA-AN-A0XU	RNF43	cna	gain	TCGA
TCGA-AO-A0JL	RNF43	cna	hetloss	TCGA
TCGA-BH-A0AW	RNF43	cna	amp	TCGA
TCGA-BH-A0C0	RNF43	cna	gain	TCGA
TCGA-BH-A18R	RNF43	cna	amp	TCGA
TCGA-C8-A12L	RNF43	cna	gain	TCGA
TCGA-D8-A27M	RNF43	cna	hetloss	TCGA
TCGA-EW-A1OX	RNF43	cna	amp	TCGA
MB-0346	RNF43	cna	gain	METABRIC
MB-2827	RNF43	cna	hetloss	METABRIC
MB-5070	RNF43	cna	hetloss	METABRIC
MB-5107	RNF43	cna	amp	METABRIC
MB-5323	RNF43	cna	gain	METABRIC
MB-6060	RNF43	cna	hetloss	METABRIC
MB-6098	RNF43	cna	hetloss	METABRIC
MB-6271	RNF43	cna	hetloss	METABRIC
MB-7048	RNF43	cna	hetloss	METABRIC
P-0002858-T01-IM3	RNF43	cna	amp	MSK-IMPACT
PD11327	RNF43	cna	gain	BASIS
PD11742	RNF43	cna	gain	BASIS
PD13299	RNF43	cna	gain	BASIS
PD22355	RNF43	cna	gain	BASIS
PD23562	RNF43	cna	gain	BASIS
PD23574	RNF43	cna	gain	BASIS
PD24186	RNF43	cna	gain	BASIS
PD24337	RNF43	cna	hetloss	BASIS
PD3890	RNF43	cna	hetloss	BASIS
PD3905	RNF43	cna	gain	BASIS
PD4006	RNF43	cna	amp	BASIS
PD5930	RNF43	cna	gain	BASIS
PD5945	RNF43	cna	amp	BASIS
PD5948	RNF43	cna	gain	BASIS
PD6406	RNF43	cna	hetloss	BASIS
PD6731	RNF43	cna	hetloss	BASIS
PD7067	RNF43	cna	gain	BASIS
PD7215	RNF43	cna	gain	BASIS
PD8621	RNF43	cna	gain	BASIS

PD8980	RNF43	cna	gain	BASIS
PD9004	RNF43	cna	gain	BASIS
PD9702	RNF43	cna	gain	BASIS
TCGA-A2-A25B	RPL35A	cna	amp	TCGA
TCGA-AN-A0XU	RPL35A	cna	gain	TCGA
TCGA-BH-A0AW	RPL35A	cna	gain	TCGA
TCGA-BH-A0C0	RPL35A	cna	gain	TCGA
TCGA-BH-A1FU	RPL35A	cna	hetloss	TCGA
TCGA-C8-A12L	RPL35A	cna	gain	TCGA
TCGA-D8-A27M	RPL35A	cna	hetloss	TCGA
TCGA-LL-A5YP	RPL35A	cna	gain	TCGA
MB-0346	RPL35A	cna	gain	METABRIC
MB-2827	RPL35A	cna	hetloss	METABRIC
MB-5070	RPL35A	cna	hetloss	METABRIC
MB-5107	RPL35A	cna	amp	METABRIC
MB-5465	RPL35A	cna	gain	METABRIC
MB-6060	RPL35A	cna	gain	METABRIC
MB-6098	RPL35A	cna	gain	METABRIC
MB-7038	RPL35A	cna	hetloss	METABRIC
PD11327	RPL35A	cna	hetloss	BASIS
PD13296	RPL35A	cna	hetloss	BASIS
PD13299	RPL35A	cna	gain	BASIS
PD13771	RPL35A	cna	hetloss	BASIS
PD22355	RPL35A	cna	gain	BASIS
PD23561	RPL35A	cna	hetloss	BASIS
PD23562	RPL35A	cna	gain	BASIS
PD23578	RPL35A	cna	gain	BASIS
PD24186	RPL35A	cna	amp	BASIS
PD24202	RPL35A	cna	gain	BASIS
PD3890	RPL35A	cna	hetloss	BASIS
PD3905	RPL35A	cna	gain	BASIS
PD4005	RPL35A	cna	gain	BASIS
PD4107	RPL35A	cna	gain	BASIS
PD4967	RPL35A	cna	hetloss	BASIS
PD5930	RPL35A	cna	gain	BASIS
PD5945	RPL35A	cna	amp	BASIS
PD5948	RPL35A	cna	gain	BASIS
PD6406	RPL35A	cna	gain	BASIS
PD6731	RPL35A	cna	gain	BASIS
PD7067	RPL35A	cna	gain	BASIS
PD7215	RPL35A	cna	gain	BASIS
PD8980	RPL35A	cna	gain	BASIS
PD9004	RPL35A	cna	gain	BASIS
PD9702	RPL35A	cna	gain	BASIS
TCGA-AN-A0XU	SERP2	cna	hetloss	TCGA
TCGA-AO-A0JL	SERP2	cna	hetloss	TCGA
TCGA-BH-A0AW	SERP2	cna	hetloss	TCGA

TCGA-BH-A0C0	SERP2	cna	hetloss	TCGA
TCGA-BH-A1FU	SERP2	cna	hetloss	TCGA
TCGA-C8-A12L	SERP2	cna	gain	TCGA
TCGA-LL-A5YP	SERP2	cna	hetloss	TCGA
MB-0346	SERP2	cna	hetloss	METABRIC
MB-2827	SERP2	cna	hetloss	METABRIC
MB-5107	SERP2	cna	hetloss	METABRIC
MB-6098	SERP2	cna	hetloss	METABRIC
MB-7032	SERP2	cna	hetloss	METABRIC
MB-7038	SERP2	cna	hetloss	METABRIC
MB-7048	SERP2	cna	hetloss	METABRIC
MB-0420	SERP2	cna	hetloss	METABRIC
MTS-T0064	SERP2	cna	hetloss	METABRIC
PD10014	SERP2	cna	hetloss	BASIS
PD11742	SERP2	cna	hetloss	BASIS
PD13296	SERP2	cna	hetloss	BASIS
PD13297	SERP2	cna	hetloss	BASIS
PD13771	SERP2	cna	hetloss	BASIS
PD14442	SERP2	cna	hetloss	BASIS
PD22355	SERP2	cna	hetloss	BASIS
PD23574	SERP2	cna	hetloss	BASIS
PD24202	SERP2	cna	hetloss	BASIS
PD24206	SERP2	cna	gain	BASIS
PD3890	SERP2	cna	hetloss	BASIS
PD3905	SERP2	cna	gain	BASIS
PD4005	SERP2	cna	homdel	BASIS
PD4006	SERP2	cna	hetloss	BASIS
PD4826	SERP2	cna	hetloss	BASIS
PD4967	SERP2	cna	hetloss	BASIS
PD5945	SERP2	cna	gain	BASIS
PD6406	SERP2	cna	hetloss	BASIS
PD6731	SERP2	cna	hetloss	BASIS
PD7215	SERP2	cna	gain	BASIS
PD8621	SERP2	cna	hetloss	BASIS
PD8980	SERP2	cna	hetloss	BASIS
PD9004	SERP2	cna	hetloss	BASIS
PD9585	SERP2	cna	gain	BASIS
PD9702	SERP2	cna	gain	BASIS
TCGA-A2-A25B	SGK1	cna	hetloss	TCGA
TCGA-AN-A0XU	SGK1	cna	amp	TCGA
TCGA-AO-A0JL	SGK1	cna	hetloss	TCGA
TCGA-BH-A0AW	SGK1	cna	amp	TCGA
TCGA-BH-A0C0	SGK1	cna	gain	TCGA
TCGA-BH-A18R	SGK1	cna	gain	TCGA
TCGA-C8-A12L	SGK1	cna	gain	TCGA
TCGA-D8-A27M	SGK1	cna	hetloss	TCGA
MB-5070	SGK1	cna	gain	METABRIC

MB-6098	SGK1	cna	hetloss	METABRIC
MB-7032	SGK1	cna	hetloss	METABRIC
MB-7038	SGK1	cna	amp	METABRIC
MB-7048	SGK1	cna	hetloss	METABRIC
MB-0420	SGK1	cna	hetloss	METABRIC
PD11327	SGK1	cna	homdel	BASIS
PD13296	SGK1	cna	gain	BASIS
PD13299	SGK1	cna	gain	BASIS
PD13771	SGK1	cna	hetloss	BASIS
PD22355	SGK1	cna	gain	BASIS
PD23562	SGK1	cna	gain	BASIS
PD23574	SGK1	cna	gain	BASIS
PD23578	SGK1	cna	hetloss	BASIS
PD24186	SGK1	cna	gain	BASIS
PD24202	SGK1	cna	gain	BASIS
PD3890	SGK1	cna	gain	BASIS
PD3905	SGK1	cna	gain	BASIS
PD4005	SGK1	cna	hetloss	BASIS
PD4006	SGK1	cna	gain	BASIS
PD4107	SGK1	cna	amp	BASIS
PD4826	SGK1	cna	gain	BASIS
PD5935	SGK1	cna	gain	BASIS
PD5945	SGK1	cna	amp	BASIS
PD6406	SGK1	cna	hetloss	BASIS
PD6731	SGK1	cna	hetloss	BASIS
PD7067	SGK1	cna	amp	BASIS
PD7215	SGK1	cna	gain	BASIS
PD8621	SGK1	cna	gain	BASIS
PD8980	SGK1	cna	gain	BASIS
PD9004	SGK1	cna	gain	BASIS
PD9585	SGK1	cna	hetloss	BASIS
PD9702	SGK1	cna	gain	BASIS
TCGA-A2-A25B	SOX2	cna	amp	TCGA
TCGA-AN-A0XU	SOX2	cna	gain	TCGA
TCGA-BH-A0AW	SOX2	cna	gain	TCGA
TCGA-BH-A0C0	SOX2	cna	gain	TCGA
TCGA-BH-A1FU	SOX2	cna	hetloss	TCGA
TCGA-C8-A12L	SOX2	cna	gain	TCGA
TCGA-E2-A1L7	SOX2	cna	gain	TCGA
TCGA-LL-A5YP	SOX2	cna	gain	TCGA
MB-0346	SOX2	cna	gain	METABRIC
MB-5070	SOX2	cna	amp	METABRIC
MB-5107	SOX2	cna	amp	METABRIC
MB-6060	SOX2	cna	gain	METABRIC
MB-6098	SOX2	cna	gain	METABRIC
MB-7038	SOX2	cna	hetloss	METABRIC
MB-0420	SOX2	cna	gain	METABRIC

MTS-T0064	SOX2	cna	amp	METABRIC
P-0002858-T01-IM3	SOX2	cna	amp	MSK-IMPACT
PD13296	SOX2	cna	gain	BASIS
PD13299	SOX2	cna	gain	BASIS
PD13771	SOX2	cna	hetloss	BASIS
PD22355	SOX2	cna	gain	BASIS
PD23561	SOX2	cna	hetloss	BASIS
PD23562	SOX2	cna	gain	BASIS
PD23578	SOX2	cna	gain	BASIS
PD24186	SOX2	cna	amp	BASIS
PD24202	SOX2	cna	gain	BASIS
PD24206	SOX2	cna	gain	BASIS
PD3905	SOX2	cna	gain	BASIS
PD4005	SOX2	cna	gain	BASIS
PD4107	SOX2	cna	gain	BASIS
PD4826	SOX2	cna	gain	BASIS
PD5935	SOX2	cna	amp	BASIS
PD5945	SOX2	cna	amp	BASIS
PD5948	SOX2	cna	gain	BASIS
PD6406	SOX2	cna	gain	BASIS
PD6731	SOX2	cna	gain	BASIS
PD7067	SOX2	cna	gain	BASIS
PD7215	SOX2	cna	gain	BASIS
PD8621	SOX2	cna	gain	BASIS
PD8980	SOX2	cna	gain	BASIS
PD9702	SOX2	cna	gain	BASIS
TCGA-A2-A25B	SPECC1	cna	gain	TCGA
TCGA-AN-A0XU	SPECC1	cna	hetloss	TCGA
TCGA-AO-A0JL	SPECC1	cna	gain	TCGA
TCGA-BH-A0AW	SPECC1	cna	hetloss	TCGA
TCGA-BH-A0C0	SPECC1	cna	hetloss	TCGA
TCGA-C8-A12L	SPECC1	cna	hetloss	TCGA
TCGA-D8-A27M	SPECC1	cna	hetloss	TCGA
TCGA-E2-A1L7	SPECC1	cna	hetloss	TCGA
TCGA-E9-A1NC	SPECC1	cna	hetloss	TCGA
TCGA-LL-A5YP	SPECC1	cna	hetloss	TCGA
MB-0346	SPECC1	cna	gain	METABRIC
MB-2827	SPECC1	cna	hetloss	METABRIC
MB-5070	SPECC1	cna	hetloss	METABRIC
MB-5465	SPECC1	cna	hetloss	METABRIC
MB-6060	SPECC1	cna	amp	METABRIC
MB-6098	SPECC1	cna	hetloss	METABRIC
MB-6271	SPECC1	cna	hetloss	METABRIC
MB-7038	SPECC1	cna	hetloss	METABRIC
MB-7048	SPECC1	cna	amp	METABRIC
MB-0420	SPECC1	cna	hetloss	METABRIC
PD11327	SPECC1	cna	hetloss	BASIS

PD11742	SPECC1	cna	hetloss	BASIS
PD13297	SPECC1	cna	hetloss	BASIS
PD13771	SPECC1	cna	hetloss	BASIS
PD22355	SPECC1	cna	hetloss	BASIS
PD23561	SPECC1	cna	hetloss	BASIS
PD24202	SPECC1	cna	hetloss	BASIS
PD24206	SPECC1	cna	hetloss	BASIS
PD24337	SPECC1	cna	hetloss	BASIS
PD3890	SPECC1	cna	hetloss	BASIS
PD3905	SPECC1	cna	gain	BASIS
PD4005	SPECC1	cna	hetloss	BASIS
PD4107	SPECC1	cna	gain	BASIS
PD4826	SPECC1	cna	hetloss	BASIS
PD4967	SPECC1	cna	hetloss	BASIS
PD5945	SPECC1	cna	gain	BASIS
PD6406	SPECC1	cna	hetloss	BASIS
PD6413	SPECC1	cna	hetloss	BASIS
PD6731	SPECC1	cna	hetloss	BASIS
PD7067	SPECC1	cna	gain	BASIS
PD9585	SPECC1	cna	hetloss	BASIS
TCGA-AN-A0XU	STAG1	cna	gain	TCGA
TCGA-BH-A0C0	STAG1	cna	gain	TCGA
TCGA-BH-A18R	STAG1	cna	gain	TCGA
TCGA-BH-A1FU	STAG1	cna	hetloss	TCGA
TCGA-C8-A12L	STAG1	cna	gain	TCGA
TCGA-E2-A1L7	STAG1	cna	gain	TCGA
TCGA-E9-A1NC	STAG1	cna	gain	TCGA
TCGA-LL-A5YP	STAG1	cna	gain	TCGA
MB-0346	STAG1	cna	hetloss	METABRIC
MB-5070	STAG1	cna	gain	METABRIC
MB-5107	STAG1	cna	gain	METABRIC
MB-5465	STAG1	cna	gain	METABRIC
MB-7038	STAG1	cna	gain	METABRIC
MTS-T0064	STAG1	cna	gain	METABRIC
PD10014	STAG1	cna	gain	BASIS
PD11327	STAG1	cna	hetloss	BASIS
PD13297	STAG1	cna	hetloss	BASIS
PD13299	STAG1	cna	gain	BASIS
PD13771	STAG1	cna	hetloss	BASIS
PD22355	STAG1	cna	gain	BASIS
PD23561	STAG1	cna	hetloss	BASIS
PD23574	STAG1	cna	gain	BASIS
PD23578	STAG1	cna	gain	BASIS
PD24186	STAG1	cna	gain	BASIS
PD24202	STAG1	cna	gain	BASIS
PD24206	STAG1	cna	gain	BASIS
PD3905	STAG1	cna	gain	BASIS

PD4006	STAG1	cna	hetloss	BASIS
PD4107	STAG1	cna	gain	BASIS
PD4826	STAG1	cna	gain	BASIS
PD4967	STAG1	cna	hetloss	BASIS
PD5935	STAG1	cna	gain	BASIS
PD5945	STAG1	cna	gain	BASIS
PD5948	STAG1	cna	gain	BASIS
PD6731	STAG1	cna	gain	BASIS
PD7067	STAG1	cna	gain	BASIS
PD7215	STAG1	cna	gain	BASIS
PD8621	STAG1	cna	gain	BASIS
PD8980	STAG1	cna	gain	BASIS
PD9004	STAG1	cna	gain	BASIS
PD9702	STAG1	cna	gain	BASIS
TCGA-A2-A25B	TFRC	cna	amp	TCGA
TCGA-AN-A0XU	TFRC	cna	gain	TCGA
TCGA-BH-A0AW	TFRC	cna	gain	TCGA
TCGA-BH-A0C0	TFRC	cna	gain	TCGA
TCGA-BH-A1FU	TFRC	cna	hetloss	TCGA
TCGA-C8-A12L	TFRC	cna	gain	TCGA
TCGA-D8-A27M	TFRC	cna	hetloss	TCGA
TCGA-LL-A5YP	TFRC	cna	gain	TCGA
MB-0346	TFRC	cna	gain	METABRIC
MB-2827	TFRC	cna	hetloss	METABRIC
MB-5070	TFRC	cna	hetloss	METABRIC
MB-5107	TFRC	cna	amp	METABRIC
MB-5465	TFRC	cna	gain	METABRIC
MB-6060	TFRC	cna	gain	METABRIC
MB-6098	TFRC	cna	gain	METABRIC
MB-7038	TFRC	cna	hetloss	METABRIC
PD10014	TFRC	cna	gain	BASIS
PD11327	TFRC	cna	hetloss	BASIS
PD13296	TFRC	cna	hetloss	BASIS
PD13299	TFRC	cna	gain	BASIS
PD13771	TFRC	cna	hetloss	BASIS
PD22355	TFRC	cna	gain	BASIS
PD23561	TFRC	cna	hetloss	BASIS
PD23562	TFRC	cna	gain	BASIS
PD23578	TFRC	cna	gain	BASIS
PD24186	TFRC	cna	amp	BASIS
PD24202	TFRC	cna	gain	BASIS
PD3905	TFRC	cna	gain	BASIS
PD4005	TFRC	cna	gain	BASIS
PD4107	TFRC	cna	gain	BASIS
PD4967	TFRC	cna	hetloss	BASIS
PD5930	TFRC	cna	gain	BASIS
PD5945	TFRC	cna	amp	BASIS

PD5948	TFRC	cna	gain	BASIS
PD6406	TFRC	cna	gain	BASIS
PD6731	TFRC	cna	gain	BASIS
PD7067	TFRC	cna	gain	BASIS
PD7215	TFRC	cna	gain	BASIS
PD8980	TFRC	cna	gain	BASIS
PD9004	TFRC	cna	gain	BASIS
PD9702	TFRC	cna	gain	BASIS
TCGA-A2-A25B	WISP3	cna	hetloss	TCGA
TCGA-AN-A0XU	WISP3	cna	amp	TCGA
TCGA-AO-A0JL	WISP3	cna	hetloss	TCGA
TCGA-BH-A0AW	WISP3	cna	amp	TCGA
TCGA-BH-A18R	WISP3	cna	gain	TCGA
TCGA-C8-A12L	WISP3	cna	gain	TCGA
TCGA-D8-A27M	WISP3	cna	hetloss	TCGA
TCGA-LL-A5YP	WISP3	cna	gain	TCGA
MB-2827	WISP3	cna	amp	METABRIC
MB-5107	WISP3	cna	hetloss	METABRIC
MB-6098	WISP3	cna	hetloss	METABRIC
MB-7032	WISP3	cna	hetloss	METABRIC
MB-7038	WISP3	cna	gain	METABRIC
MB-7048	WISP3	cna	hetloss	METABRIC
MTS-T0064	WISP3	cna	amp	METABRIC
PD11327	WISP3	cna	gain	BASIS
PD13296	WISP3	cna	gain	BASIS
PD13299	WISP3	cna	gain	BASIS
PD13771	WISP3	cna	hetloss	BASIS
PD22355	WISP3	cna	gain	BASIS
PD23562	WISP3	cna	gain	BASIS
PD23574	WISP3	cna	gain	BASIS
PD24186	WISP3	cna	gain	BASIS
PD24206	WISP3	cna	gain	BASIS
PD24337	WISP3	cna	hetloss	BASIS
PD3905	WISP3	cna	gain	BASIS
PD4006	WISP3	cna	gain	BASIS
PD4107	WISP3	cna	hetloss	BASIS
PD4826	WISP3	cna	gain	BASIS
PD5930	WISP3	cna	hetloss	BASIS
PD5935	WISP3	cna	gain	BASIS
PD5945	WISP3	cna	amp	BASIS
PD6406	WISP3	cna	hetloss	BASIS
PD6731	WISP3	cna	hetloss	BASIS
PD7067	WISP3	cna	gain	BASIS
PD7215	WISP3	cna	gain	BASIS
PD8621	WISP3	cna	gain	BASIS
PD8980	WISP3	cna	gain	BASIS
PD9004	WISP3	cna	gain	BASIS

PD9585	WISP3	cna	hetloss	BASIS
PD9702	WISP3	cna	gain	BASIS
TCGA-A2-A25B	XRCC3	cna	gain	TCGA
TCGA-AN-A0XU	XRCC3	cna	gain	TCGA
TCGA-AO-A0JL	XRCC3	cna	hetloss	TCGA
TCGA-BH-A0AW	XRCC3	cna	hetloss	TCGA
TCGA-BH-A1FU	XRCC3	cna	gain	TCGA
TCGA-C8-A12L	XRCC3	cna	gain	TCGA
TCGA-D8-A27M	XRCC3	cna	hetloss	TCGA
TCGA-E2-A1L7	XRCC3	cna	gain	TCGA
TCGA-E9-A1NC	XRCC3	cna	hetloss	TCGA
TCGA-LL-A5YP	XRCC3	cna	hetloss	TCGA
MB-0346	XRCC3	cna	gain	METABRIC
MB-2827	XRCC3	cna	hetloss	METABRIC
MB-5070	XRCC3	cna	hetloss	METABRIC
MB-5107	XRCC3	cna	hetloss	METABRIC
MB-6060	XRCC3	cna	gain	METABRIC
MB-6098	XRCC3	cna	gain	METABRIC
MTS-T0064	XRCC3	cna	hetloss	METABRIC
PD10014	XRCC3	cna	hetloss	BASIS
PD11742	XRCC3	cna	hetloss	BASIS
PD13296	XRCC3	cna	gain	BASIS
PD13297	XRCC3	cna	hetloss	BASIS
PD13299	XRCC3	cna	hetloss	BASIS
PD13771	XRCC3	cna	hetloss	BASIS
PD22355	XRCC3	cna	hetloss	BASIS
PD23578	XRCC3	cna	hetloss	BASIS
PD24202	XRCC3	cna	hetloss	BASIS
PD24206	XRCC3	cna	gain	BASIS
PD24337	XRCC3	cna	hetloss	BASIS
PD3890	XRCC3	cna	hetloss	BASIS
PD3905	XRCC3	cna	gain	BASIS
PD4006	XRCC3	cna	gain	BASIS
PD4826	XRCC3	cna	gain	BASIS
PD4967	XRCC3	cna	hetloss	BASIS
PD5945	XRCC3	cna	gain	BASIS
PD5948	XRCC3	cna	gain	BASIS
PD6406	XRCC3	cna	hetloss	BASIS
PD6413	XRCC3	cna	hetloss	BASIS
PD7067	XRCC3	cna	gain	BASIS
PD8621	XRCC3	cna	gain	BASIS
PD8980	XRCC3	cna	hetloss	BASIS
PD9702	XRCC3	cna	gain	BASIS
TCGA-A2-A25B	AADACL2	cna	gain	TCGA
TCGA-AN-A0XU	AADACL2	cna	gain	TCGA
TCGA-BH-A0C0	AADACL2	cna	gain	TCGA
TCGA-BH-A18R	AADACL2	cna	gain	TCGA

TCGA-BH-A1FU	AADACL2	cna	hetloss	TCGA
TCGA-C8-A12L	AADACL2	cna	amp	TCGA
TCGA-E2-A1L7	AADACL2	cna	gain	TCGA
TCGA-E9-A1NC	AADACL2	cna	gain	TCGA
TCGA-LL-A5YP	AADACL2	cna	gain	TCGA
MB-0346	AADACL2	cna	hetloss	METABRIC
MB-5070	AADACL2	cna	amp	METABRIC
MB-5107	AADACL2	cna	gain	METABRIC
MB-5465	AADACL2	cna	amp	METABRIC
MB-0420	AADACL2	cna	gain	METABRIC
PD10014	AADACL2	cna	amp	BASIS
PD11327	AADACL2	cna	gain	BASIS
PD13296	AADACL2	cna	gain	BASIS
PD13299	AADACL2	cna	gain	BASIS
PD13771	AADACL2	cna	hetloss	BASIS
PD22355	AADACL2	cna	gain	BASIS
PD23561	AADACL2	cna	hetloss	BASIS
PD23562	AADACL2	cna	amp	BASIS
PD23574	AADACL2	cna	gain	BASIS
PD23578	AADACL2	cna	gain	BASIS
PD24186	AADACL2	cna	gain	BASIS
PD24202	AADACL2	cna	gain	BASIS
PD24206	AADACL2	cna	gain	BASIS
PD3905	AADACL2	cna	gain	BASIS
PD4107	AADACL2	cna	gain	BASIS
PD5930	AADACL2	cna	gain	BASIS
PD5935	AADACL2	cna	gain	BASIS
PD5945	AADACL2	cna	amp	BASIS
PD5948	AADACL2	cna	amp	BASIS
PD6731	AADACL2	cna	gain	BASIS
PD7067	AADACL2	cna	gain	BASIS
PD7215	AADACL2	cna	gain	BASIS
PD8621	AADACL2	cna	gain	BASIS
PD8980	AADACL2	cna	gain	BASIS
PD9004	AADACL2	cna	gain	BASIS
PD9702	AADACL2	cna	gain	BASIS
TCGA-AN-A0XU	ACSL6	cna	hetloss	TCGA
TCGA-AO-A0JL	ACSL6	cna	hetloss	TCGA
TCGA-BH-A0C0	ACSL6	cna	hetloss	TCGA
TCGA-BH-A1FU	ACSL6	cna	hetloss	TCGA
TCGA-C8-A12L	ACSL6	cna	hetloss	TCGA
TCGA-D8-A27M	ACSL6	cna	hetloss	TCGA
TCGA-E2-A1L7	ACSL6	cna	gain	TCGA
TCGA-E9-A1NC	ACSL6	cna	hetloss	TCGA
TCGA-LL-A5YP	ACSL6	cna	hetloss	TCGA
MB-0346	ACSL6	cna	gain	METABRIC
MB-2827	ACSL6	cna	hetloss	METABRIC

MB-5070	ACSL6	cna	hetloss	METABRIC
MB-5107	ACSL6	cna	hetloss	METABRIC
MB-5465	ACSL6	cna	hetloss	METABRIC
MB-6060	ACSL6	cna	hetloss	METABRIC
MB-6098	ACSL6	cna	hetloss	METABRIC
MB-7038	ACSL6	cna	hetloss	METABRIC
MB-0420	ACSL6	cna	hetloss	METABRIC
PD10014	ACSL6	cna	hetloss	BASIS
PD13296	ACSL6	cna	hetloss	BASIS
PD13297	ACSL6	cna	hetloss	BASIS
PD13771	ACSL6	cna	gain	BASIS
PD22355	ACSL6	cna	hetloss	BASIS
PD23562	ACSL6	cna	hetloss	BASIS
PD23578	ACSL6	cna	hetloss	BASIS
PD24202	ACSL6	cna	hetloss	BASIS
PD24206	ACSL6	cna	gain	BASIS
PD24337	ACSL6	cna	hetloss	BASIS
PD3890	ACSL6	cna	hetloss	BASIS
PD3905	ACSL6	cna	gain	BASIS
PD4005	ACSL6	cna	hetloss	BASIS
PD5935	ACSL6	cna	gain	BASIS
PD5945	ACSL6	cna	gain	BASIS
PD5948	ACSL6	cna	hetloss	BASIS
PD6406	ACSL6	cna	hetloss	BASIS
PD6413	ACSL6	cna	hetloss	BASIS
PD7067	ACSL6	cna	gain	BASIS
PD8621	ACSL6	cna	hetloss	BASIS
PD8980	ACSL6	cna	hetloss	BASIS
PD9585	ACSL6	cna	hetloss	BASIS
TCGA-A2-A25B	ALDH2	cna	gain	TCGA
TCGA-AN-A0XU	ALDH2	cna	hetloss	TCGA
TCGA-AO-A0JL	ALDH2	cna	hetloss	TCGA
TCGA-BH-A0C0	ALDH2	cna	gain	TCGA
TCGA-BH-A18R	ALDH2	cna	gain	TCGA
TCGA-BH-A1FU	ALDH2	cna	hetloss	TCGA
TCGA-C8-A12L	ALDH2	cna	gain	TCGA
TCGA-D8-A27M	ALDH2	cna	hetloss	TCGA
TCGA-E2-A1L7	ALDH2	cna	gain	TCGA
TCGA-LL-A5YP	ALDH2	cna	hetloss	TCGA
MB-0346	ALDH2	cna	gain	METABRIC
MB-2827	ALDH2	cna	hetloss	METABRIC
MB-5070	ALDH2	cna	hetloss	METABRIC
MB-5465	ALDH2	cna	hetloss	METABRIC
MB-6098	ALDH2	cna	hetloss	METABRIC
MB-7048	ALDH2	cna	hetloss	METABRIC
PD10014	ALDH2	cna	gain	BASIS
PD11327	ALDH2	cna	hetloss	BASIS

PD11742	ALDH2	cna	hetloss	BASIS
PD13296	ALDH2	cna	hetloss	BASIS
PD13297	ALDH2	cna	gain	BASIS
PD22355	ALDH2	cna	hetloss	BASIS
PD23574	ALDH2	cna	gain	BASIS
PD24337	ALDH2	cna	gain	BASIS
PD3890	ALDH2	cna	hetloss	BASIS
PD3905	ALDH2	cna	gain	BASIS
PD4005	ALDH2	cna	hetloss	BASIS
PD4006	ALDH2	cna	gain	BASIS
PD4107	ALDH2	cna	gain	BASIS
PD4826	ALDH2	cna	gain	BASIS
PD4967	ALDH2	cna	hetloss	BASIS
PD5945	ALDH2	cna	gain	BASIS
PD5948	ALDH2	cna	gain	BASIS
PD6406	ALDH2	cna	hetloss	BASIS
PD6413	ALDH2	cna	hetloss	BASIS
PD6731	ALDH2	cna	hetloss	BASIS
PD7067	ALDH2	cna	gain	BASIS
PD9004	ALDH2	cna	hetloss	BASIS
PD9585	ALDH2	cna	hetloss	BASIS
PD9702	ALDH2	cna	gain	BASIS
TCGA-AN-A0XU	BCL9	cna	gain	TCGA
TCGA-AO-A0JL	BCL9	cna	gain	TCGA
TCGA-BH-A0AW	BCL9	cna	gain	TCGA
TCGA-BH-A0C0	BCL9	cna	hetloss	TCGA
TCGA-BH-A18R	BCL9	cna	gain	TCGA
TCGA-BH-A1FU	BCL9	cna	gain	TCGA
TCGA-C8-A12L	BCL9	cna	gain	TCGA
TCGA-E2-A1L7	BCL9	cna	gain	TCGA
TCGA-EW-A1OX	BCL9	cna	gain	TCGA
TCGA-LL-A5YP	BCL9	cna	amp	TCGA
MB-2827	BCL9	cna	amp	METABRIC
MB-5070	BCL9	cna	gain	METABRIC
MB-6098	BCL9	cna	amp	METABRIC
MB-7032	BCL9	cna	gain	METABRIC
MB-7038	BCL9	cna	gain	METABRIC
MB-0420	BCL9	cna	gain	METABRIC
PD10014	BCL9	cna	gain	BASIS
PD11327	BCL9	cna	amp	BASIS
PD13296	BCL9	cna	gain	BASIS
PD13297	BCL9	cna	gain	BASIS
PD13299	BCL9	cna	gain	BASIS
PD14442	BCL9	cna	gain	BASIS
PD23562	BCL9	cna	amp	BASIS
PD23574	BCL9	cna	gain	BASIS
PD24186	BCL9	cna	gain	BASIS

PD24206	BCL9	cna	gain	BASIS
PD3905	BCL9	cna	gain	BASIS
PD4006	BCL9	cna	amp	BASIS
PD4107	BCL9	cna	gain	BASIS
PD4826	BCL9	cna	gain	BASIS
PD5930	BCL9	cna	gain	BASIS
PD5935	BCL9	cna	gain	BASIS
PD5945	BCL9	cna	gain	BASIS
PD5948	BCL9	cna	gain	BASIS
PD6413	BCL9	cna	gain	BASIS
PD7067	BCL9	cna	amp	BASIS
PD7215	BCL9	cna	gain	BASIS
PD8621	BCL9	cna	amp	BASIS
PD9004	BCL9	cna	amp	BASIS
PD9702	BCL9	cna	gain	BASIS
TCGA-AN-A0XU	BUB1B	cna	hetloss	TCGA
TCGA-AO-A0JL	BUB1B	cna	hetloss	TCGA
TCGA-BH-A0C0	BUB1B	cna	hetloss	TCGA
TCGA-C8-A12L	BUB1B	cna	hetloss	TCGA
TCGA-D8-A27M	BUB1B	cna	hetloss	TCGA
TCGA-E2-A1L7	BUB1B	cna	hetloss	TCGA
TCGA-E9-A1NC	BUB1B	cna	hetloss	TCGA
TCGA-LL-A5YP	BUB1B	cna	hetloss	TCGA
MB-2827	BUB1B	cna	hetloss	METABRIC
MB-5070	BUB1B	cna	hetloss	METABRIC
MB-5465	BUB1B	cna	hetloss	METABRIC
MB-6060	BUB1B	cna	hetloss	METABRIC
MB-6098	BUB1B	cna	hetloss	METABRIC
MB-7038	BUB1B	cna	hetloss	METABRIC
MB-0420	BUB1B	cna	hetloss	METABRIC
PD10014	BUB1B	cna	hetloss	BASIS
PD11327	BUB1B	cna	homdel	BASIS
PD11742	BUB1B	cna	hetloss	BASIS
PD13297	BUB1B	cna	hetloss	BASIS
PD13299	BUB1B	cna	hetloss	BASIS
PD22355	BUB1B	cna	hetloss	BASIS
PD23574	BUB1B	cna	hetloss	BASIS
PD23578	BUB1B	cna	hetloss	BASIS
PD24202	BUB1B	cna	hetloss	BASIS
PD24206	BUB1B	cna	gain	BASIS
PD24337	BUB1B	cna	hetloss	BASIS
PD3890	BUB1B	cna	hetloss	BASIS
PD3905	BUB1B	cna	gain	BASIS
PD4005	BUB1B	cna	homdel	BASIS
PD4967	BUB1B	cna	hetloss	BASIS
PD5930	BUB1B	cna	hetloss	BASIS
PD5945	BUB1B	cna	amp	BASIS

PD5948	BUB1B	cna	hetloss	BASIS
PD6413	BUB1B	cna	hetloss	BASIS
PD7067	BUB1B	cna	gain	BASIS
PD7215	BUB1B	cna	gain	BASIS
PD8980	BUB1B	cna	hetloss	BASIS
PD9004	BUB1B	cna	hetloss	BASIS
PD9585	BUB1B	cna	hetloss	BASIS
PD9702	BUB1B	cna	gain	BASIS
TCGA-AO-A0JL	CDX2	cna	hetloss	TCGA
TCGA-BH-A0AW	CDX2	cna	hetloss	TCGA
TCGA-BH-A0C0	CDX2	cna	gain	TCGA
TCGA-BH-A1FU	CDX2	cna	hetloss	TCGA
TCGA-C8-A12L	CDX2	cna	hetloss	TCGA
TCGA-D8-A27M	CDX2	cna	gain	TCGA
TCGA-E9-A1NC	CDX2	cna	hetloss	TCGA
TCGA-EW-A1OX	CDX2	cna	homdel	TCGA
TCGA-LL-A5YP	CDX2	cna	hetloss	TCGA
MB-0346	CDX2	cna	amp	METABRIC
MB-2827	CDX2	cna	hetloss	METABRIC
MB-6098	CDX2	cna	hetloss	METABRIC
MB-7032	CDX2	cna	gain	METABRIC
MB-0420	CDX2	cna	hetloss	METABRIC
PD10014	CDX2	cna	hetloss	BASIS
PD11327	CDX2	cna	hetloss	BASIS
PD13296	CDX2	cna	hetloss	BASIS
PD13297	CDX2	cna	hetloss	BASIS
PD13771	CDX2	cna	hetloss	BASIS
PD14442	CDX2	cna	hetloss	BASIS
PD22355	CDX2	cna	hetloss	BASIS
PD23562	CDX2	cna	gain	BASIS
PD23578	CDX2	cna	hetloss	BASIS
PD24202	CDX2	cna	hetloss	BASIS
PD24206	CDX2	cna	gain	BASIS
PD24337	CDX2	cna	hetloss	BASIS
PD3890	CDX2	cna	hetloss	BASIS
PD3905	CDX2	cna	gain	BASIS
PD4005	CDX2	cna	homdel	BASIS
PD4006	CDX2	cna	gain	BASIS
PD4107	CDX2	cna	gain	BASIS
PD4826	CDX2	cna	hetloss	BASIS
PD4967	CDX2	cna	hetloss	BASIS
PD5945	CDX2	cna	amp	BASIS
PD6406	CDX2	cna	hetloss	BASIS
PD6413	CDX2	cna	hetloss	BASIS
PD6731	CDX2	cna	hetloss	BASIS
PD7067	CDX2	cna	gain	BASIS
PD8980	CDX2	cna	hetloss	BASIS

PD9702	CDX2	cna	gain	BASIS
TCGA-A2-A25B	CHUK	cna	hetloss	TCGA
TCGA-AN-A0XU	CHUK	cna	hetloss	TCGA
TCGA-AO-A0JL	CHUK	cna	hetloss	TCGA
TCGA-BH-A18R	CHUK	cna	gain	TCGA
TCGA-C8-A12L	CHUK	cna	hetloss	TCGA
TCGA-D8-A27M	CHUK	cna	hetloss	TCGA
TCGA-E2-A1L7	CHUK	cna	hetloss	TCGA
MB-0346	CHUK	cna	gain	METABRIC
MB-2827	CHUK	cna	hetloss	METABRIC
MB-5070	CHUK	cna	hetloss	METABRIC
MB-6060	CHUK	cna	hetloss	METABRIC
MB-6098	CHUK	cna	hetloss	METABRIC
MB-7038	CHUK	cna	hetloss	METABRIC
MB-7048	CHUK	cna	hetloss	METABRIC
MB-0420	CHUK	cna	hetloss	METABRIC
PD10014	CHUK	cna	hetloss	BASIS
PD11327	CHUK	cna	homdel	BASIS
PD11742	CHUK	cna	hetloss	BASIS
PD22355	CHUK	cna	hetloss	BASIS
PD23562	CHUK	cna	gain	BASIS
PD23574	CHUK	cna	gain	BASIS
PD23578	CHUK	cna	hetloss	BASIS
PD24202	CHUK	cna	hetloss	BASIS
PD24206	CHUK	cna	hetloss	BASIS
PD3890	CHUK	cna	hetloss	BASIS
PD3905	CHUK	cna	gain	BASIS
PD4006	CHUK	cna	gain	BASIS
PD4107	CHUK	cna	gain	BASIS
PD5935	CHUK	cna	hetloss	BASIS
PD5945	CHUK	cna	gain	BASIS
PD5948	CHUK	cna	gain	BASIS
PD6406	CHUK	cna	hetloss	BASIS
PD6731	CHUK	cna	hetloss	BASIS
PD7067	CHUK	cna	gain	BASIS
PD7215	CHUK	cna	gain	BASIS
PD8621	CHUK	cna	gain	BASIS
PD8980	CHUK	cna	hetloss	BASIS
PD9004	CHUK	cna	gain	BASIS
PD9585	CHUK	cna	hetloss	BASIS
PD9702	CHUK	cna	gain	BASIS
TCGA-A2-A25B	CNTRL	cna	gain	TCGA
TCGA-AN-A0XU	CNTRL	cna	hetloss	TCGA
TCGA-AO-A0JL	CNTRL	cna	hetloss	TCGA
TCGA-BH-A0C0	CNTRL	cna	gain	TCGA
TCGA-C8-A12L	CNTRL	cna	hetloss	TCGA
TCGA-E2-A1L7	CNTRL	cna	gain	TCGA

TCGA-E9-A1NC	CNTRL	cna	hetloss	TCGA
TCGA-LL-A5YP	CNTRL	cna	hetloss	TCGA
MB-0346	CNTRL	cna	hetloss	METABRIC
MB-2827	CNTRL	cna	hetloss	METABRIC
MB-5070	CNTRL	cna	hetloss	METABRIC
MB-5107	CNTRL	cna	hetloss	METABRIC
MB-6098	CNTRL	cna	hetloss	METABRIC
MB-7038	CNTRL	cna	hetloss	METABRIC
MB-0420	CNTRL	cna	hetloss	METABRIC
PD10014	CNTRL	cna	gain	BASIS
PD11327	CNTRL	cna	hetloss	BASIS
PD13297	CNTRL	cna	hetloss	BASIS
PD13299	CNTRL	cna	gain	BASIS
PD23562	CNTRL	cna	gain	BASIS
PD23578	CNTRL	cna	hetloss	BASIS
PD24186	CNTRL	cna	amp	BASIS
PD24202	CNTRL	cna	hetloss	BASIS
PD24206	CNTRL	cna	gain	BASIS
PD24337	CNTRL	cna	hetloss	BASIS
PD3890	CNTRL	cna	hetloss	BASIS
PD3905	CNTRL	cna	gain	BASIS
PD4005	CNTRL	cna	hetloss	BASIS
PD4006	CNTRL	cna	gain	BASIS
PD4107	CNTRL	cna	hetloss	BASIS
PD5930	CNTRL	cna	gain	BASIS
PD5945	CNTRL	cna	gain	BASIS
PD6406	CNTRL	cna	hetloss	BASIS
PD6731	CNTRL	cna	hetloss	BASIS
PD7067	CNTRL	cna	gain	BASIS
PD7215	CNTRL	cna	gain	BASIS
PD8621	CNTRL	cna	gain	BASIS
PD8980	CNTRL	cna	gain	BASIS
PD9004	CNTRL	cna	hetloss	BASIS
PD9585	CNTRL	cna	hetloss	BASIS
TCGA-AN-A0XU	CREBBP	cna	gain	TCGA
TCGA-AO-A0JL	CREBBP	cna	gain	TCGA
TCGA-BH-A0AW	CREBBP	cna	gain	TCGA
TCGA-BH-A0C0	CREBBP	cna	gain	TCGA
TCGA-C8-A12L	CREBBP	cna	gain	TCGA
TCGA-E2-A1L7	CREBBP	cna	hetloss	TCGA
TCGA-E9-A1NC	CREBBP	cna	hetloss	TCGA
TCGA-LL-A5YP	CREBBP	cna	hetloss	TCGA
MB-0346	CREBBP	cna	hetloss	METABRIC
MB-5465	CREBBP	cna	hetloss	METABRIC
MB-6098	CREBBP	cna	hetloss	METABRIC
MB-6271	CREBBP	cna	gain	METABRIC
MB-7032	CREBBP	cna	gain	METABRIC

MB-7038	CREBBP	cna	hetloss	METABRIC
MB-0420	CREBBP	cna	hetloss	METABRIC
PD10014	CREBBP	cna	hetloss	BASIS
PD11327	CREBBP	cna	hetloss	BASIS
PD11742	CREBBP	cna	gain	BASIS
PD13296	CREBBP	cna	gain	BASIS
PD13297	CREBBP	cna	hetloss	BASIS
PD13299	CREBBP	cna	gain	BASIS
PD22355	CREBBP	cna	hetloss	BASIS
PD23562	CREBBP	cna	hetloss	BASIS
PD23578	CREBBP	cna	hetloss	BASIS
PD24202	CREBBP	cna	hetloss	BASIS
PD24337	CREBBP	cna	hetloss	BASIS
PD3905	CREBBP	cna	gain	BASIS
PD4005	CREBBP	cna	gain	BASIS
PD4006	CREBBP	cna	gain	BASIS
PD4107	CREBBP	cna	gain	BASIS
PD4826	CREBBP	cna	gain	BASIS
PD5930	CREBBP	cna	gain	BASIS
PD5935	CREBBP	cna	gain	BASIS
PD5945	CREBBP	cna	amp	BASIS
PD5948	CREBBP	cna	gain	BASIS
PD7067	CREBBP	cna	gain	BASIS
PD7215	CREBBP	cna	amp	BASIS
PD9004	CREBBP	cna	gain	BASIS
PD9585	CREBBP	cna	hetloss	BASIS
PD9702	CREBBP	cna	gain	BASIS
TCGA-AN-A0XU	CTNNA1	cna	hetloss	TCGA
TCGA-AO-A0JL	CTNNA1	cna	hetloss	TCGA
TCGA-BH-A0C0	CTNNA1	cna	hetloss	TCGA
TCGA-BH-A1FU	CTNNA1	cna	hetloss	TCGA
TCGA-C8-A12L	CTNNA1	cna	hetloss	TCGA
TCGA-D8-A27M	CTNNA1	cna	hetloss	TCGA
TCGA-E2-A1L7	CTNNA1	cna	hetloss	TCGA
TCGA-E9-A1NC	CTNNA1	cna	hetloss	TCGA
TCGA-LL-A5YP	CTNNA1	cna	hetloss	TCGA
MB-0346	CTNNA1	cna	gain	METABRIC
MB-2827	CTNNA1	cna	hetloss	METABRIC
MB-5070	CTNNA1	cna	hetloss	METABRIC
MB-5465	CTNNA1	cna	hetloss	METABRIC
MB-6098	CTNNA1	cna	hetloss	METABRIC
MB-7038	CTNNA1	cna	hetloss	METABRIC
MB-0420	CTNNA1	cna	hetloss	METABRIC
PD10014	CTNNA1	cna	hetloss	BASIS
PD11327	CTNNA1	cna	hetloss	BASIS
PD13296	CTNNA1	cna	hetloss	BASIS
PD13297	CTNNA1	cna	hetloss	BASIS

PD13771	CTNNA1	cna	gain	BASIS
PD22355	CTNNA1	cna	hetloss	BASIS
PD23562	CTNNA1	cna	gain	BASIS
PD23574	CTNNA1	cna	gain	BASIS
PD23578	CTNNA1	cna	hetloss	BASIS
PD24186	CTNNA1	cna	gain	BASIS
PD24202	CTNNA1	cna	hetloss	BASIS
PD24206	CTNNA1	cna	gain	BASIS
PD24337	CTNNA1	cna	hetloss	BASIS
PD3890	CTNNA1	cna	hetloss	BASIS
PD3905	CTNNA1	cna	gain	BASIS
PD4005	CTNNA1	cna	hetloss	BASIS
PD4826	CTNNA1	cna	gain	BASIS
PD5935	CTNNA1	cna	gain	BASIS
PD5945	CTNNA1	cna	gain	BASIS
PD6406	CTNNA1	cna	hetloss	BASIS
PD6413	CTNNA1	cna	hetloss	BASIS
PD7067	CTNNA1	cna	gain	BASIS
PD8980	CTNNA1	cna	hetloss	BASIS
PD9585	CTNNA1	cna	hetloss	BASIS
TCGA-A2-A25B	CYP17A1	cna	hetloss	TCGA
TCGA-AN-A0XU	CYP17A1	cna	hetloss	TCGA
TCGA-AO-A0JL	CYP17A1	cna	hetloss	TCGA
TCGA-BH-A18R	CYP17A1	cna	gain	TCGA
TCGA-C8-A12L	CYP17A1	cna	hetloss	TCGA
TCGA-D8-A27M	CYP17A1	cna	hetloss	TCGA
TCGA-E2-A1L7	CYP17A1	cna	hetloss	TCGA
TCGA-LL-A5YP	CYP17A1	cna	gain	TCGA
MB-0346	CYP17A1	cna	gain	METABRIC
MB-2827	CYP17A1	cna	hetloss	METABRIC
MB-5070	CYP17A1	cna	hetloss	METABRIC
MB-6060	CYP17A1	cna	hetloss	METABRIC
MB-6098	CYP17A1	cna	hetloss	METABRIC
MB-7038	CYP17A1	cna	hetloss	METABRIC
MB-7048	CYP17A1	cna	hetloss	METABRIC
MB-0420	CYP17A1	cna	hetloss	METABRIC
PD10014	CYP17A1	cna	hetloss	BASIS
PD11327	CYP17A1	cna	homdel	BASIS
PD11742	CYP17A1	cna	hetloss	BASIS
PD22355	CYP17A1	cna	hetloss	BASIS
PD23562	CYP17A1	cna	gain	BASIS
PD23574	CYP17A1	cna	gain	BASIS
PD23578	CYP17A1	cna	hetloss	BASIS
PD24206	CYP17A1	cna	homdel	BASIS
PD3890	CYP17A1	cna	hetloss	BASIS
PD3905	CYP17A1	cna	gain	BASIS
PD4005	CYP17A1	cna	hetloss	BASIS

PD4006	CYP17A1	cna	gain	BASIS
PD4107	CYP17A1	cna	gain	BASIS
PD5935	CYP17A1	cna	hetloss	BASIS
PD5945	CYP17A1	cna	gain	BASIS
PD5948	CYP17A1	cna	gain	BASIS
PD6406	CYP17A1	cna	hetloss	BASIS
PD6731	CYP17A1	cna	hetloss	BASIS
PD7067	CYP17A1	cna	gain	BASIS
PD7215	CYP17A1	cna	gain	BASIS
PD8621	CYP17A1	cna	gain	BASIS
PD8980	CYP17A1	cna	hetloss	BASIS
PD9585	CYP17A1	cna	hetloss	BASIS
PD9702	CYP17A1	cna	gain	BASIS
TCGA-A2-A25B	DICER1	cna	hetloss	TCGA
TCGA-AN-A0XU	DICER1	cna	gain	TCGA
TCGA-AO-A0JL	DICER1	cna	hetloss	TCGA
TCGA-BH-A0AW	DICER1	cna	hetloss	TCGA
TCGA-BH-A1FU	DICER1	cna	gain	TCGA
TCGA-C8-A12L	DICER1	cna	gain	TCGA
TCGA-D8-A27M	DICER1	cna	hetloss	TCGA
TCGA-E2-A1L7	DICER1	cna	gain	TCGA
TCGA-E9-A1NC	DICER1	cna	hetloss	TCGA
TCGA-LL-A5YP	DICER1	cna	hetloss	TCGA
MB-0346	DICER1	cna	gain	METABRIC
MB-2827	DICER1	cna	hetloss	METABRIC
MB-5070	DICER1	cna	hetloss	METABRIC
MB-5107	DICER1	cna	hetloss	METABRIC
MB-6098	DICER1	cna	hetloss	METABRIC
MTS-T0064	DICER1	cna	hetloss	METABRIC
PD10014	DICER1	cna	hetloss	BASIS
PD11327	DICER1	cna	gain	BASIS
PD11742	DICER1	cna	hetloss	BASIS
PD13296	DICER1	cna	gain	BASIS
PD13297	DICER1	cna	hetloss	BASIS
PD13771	DICER1	cna	hetloss	BASIS
PD23562	DICER1	cna	gain	BASIS
PD23574	DICER1	cna	gain	BASIS
PD23578	DICER1	cna	hetloss	BASIS
PD24202	DICER1	cna	hetloss	BASIS
PD24206	DICER1	cna	gain	BASIS
PD24337	DICER1	cna	hetloss	BASIS
PD3890	DICER1	cna	hetloss	BASIS
PD3905	DICER1	cna	gain	BASIS
PD4005	DICER1	cna	hetloss	BASIS
PD4006	DICER1	cna	gain	BASIS
PD4826	DICER1	cna	gain	BASIS
PD5945	DICER1	cna	gain	BASIS

PD5948	DICER1	cna	gain	BASIS
PD6406	DICER1	cna	hetloss	BASIS
PD6413	DICER1	cna	hetloss	BASIS
PD7067	DICER1	cna	gain	BASIS
PD8980	DICER1	cna	hetloss	BASIS
PD9702	DICER1	cna	gain	BASIS
TCGA-A2-A25B	DIS3	cna	hetloss	TCGA
TCGA-AN-A0XU	DIS3	cna	hetloss	TCGA
TCGA-AO-A0JL	DIS3	cna	hetloss	TCGA
TCGA-BH-A0AW	DIS3	cna	hetloss	TCGA
TCGA-BH-A0C0	DIS3	cna	hetloss	TCGA
TCGA-BH-A1FU	DIS3	cna	hetloss	TCGA
TCGA-C8-A12L	DIS3	cna	gain	TCGA
TCGA-EW-A1OX	DIS3	cna	homdel	TCGA
TCGA-LL-A5YP	DIS3	cna	hetloss	TCGA
MB-0346	DIS3	cna	hetloss	METABRIC
MB-5107	DIS3	cna	hetloss	METABRIC
MB-6060	DIS3	cna	hetloss	METABRIC
MB-6098	DIS3	cna	gain	METABRIC
MB-7038	DIS3	cna	hetloss	METABRIC
MB-7048	DIS3	cna	hetloss	METABRIC
MB-0420	DIS3	cna	gain	METABRIC
PD10014	DIS3	cna	hetloss	BASIS
PD11327	DIS3	cna	homdel	BASIS
PD13299	DIS3	cna	gain	BASIS
PD13771	DIS3	cna	hetloss	BASIS
PD14442	DIS3	cna	hetloss	BASIS
PD23562	DIS3	cna	gain	BASIS
PD23574	DIS3	cna	hetloss	BASIS
PD23578	DIS3	cna	gain	BASIS
PD24202	DIS3	cna	hetloss	BASIS
PD24206	DIS3	cna	hetloss	BASIS
PD24337	DIS3	cna	hetloss	BASIS
PD3905	DIS3	cna	gain	BASIS
PD4005	DIS3	cna	hetloss	BASIS
PD4006	DIS3	cna	hetloss	BASIS
PD4967	DIS3	cna	hetloss	BASIS
PD5945	DIS3	cna	gain	BASIS
PD5948	DIS3	cna	gain	BASIS
PD6406	DIS3	cna	hetloss	BASIS
PD6731	DIS3	cna	hetloss	BASIS
PD7215	DIS3	cna	gain	BASIS
PD8621	DIS3	cna	hetloss	BASIS
PD8980	DIS3	cna	hetloss	BASIS
PD9585	DIS3	cna	hetloss	BASIS
PD9702	DIS3	cna	gain	BASIS
TCGA-A2-A25B	DKK2	cna	hetloss	TCGA

TCGA-AN-A0XU	DKK2	cna	hetloss	TCGA
TCGA-BH-A0AW	DKK2	cna	hetloss	TCGA
TCGA-BH-A0C0	DKK2	cna	hetloss	TCGA
TCGA-BH-A1FU	DKK2	cna	gain	TCGA
TCGA-C8-A12L	DKK2	cna	hetloss	TCGA
TCGA-D8-A27M	DKK2	cna	hetloss	TCGA
TCGA-E2-A1L7	DKK2	cna	hetloss	TCGA
TCGA-E9-A1NC	DKK2	cna	hetloss	TCGA
TCGA-LL-A5YP	DKK2	cna	gain	TCGA
MB-2827	DKK2	cna	hetloss	METABRIC
MB-5070	DKK2	cna	hetloss	METABRIC
MB-5465	DKK2	cna	hetloss	METABRIC
MB-7048	DKK2	cna	hetloss	METABRIC
MB-0420	DKK2	cna	hetloss	METABRIC
PD10014	DKK2	cna	gain	BASIS
PD11742	DKK2	cna	hetloss	BASIS
PD13296	DKK2	cna	hetloss	BASIS
PD13297	DKK2	cna	hetloss	BASIS
PD22355	DKK2	cna	hetloss	BASIS
PD23562	DKK2	cna	gain	BASIS
PD23578	DKK2	cna	hetloss	BASIS
PD24202	DKK2	cna	hetloss	BASIS
PD24206	DKK2	cna	gain	BASIS
PD24337	DKK2	cna	hetloss	BASIS
PD3890	DKK2	cna	gain	BASIS
PD3905	DKK2	cna	gain	BASIS
PD4006	DKK2	cna	hetloss	BASIS
PD4107	DKK2	cna	gain	BASIS
PD4826	DKK2	cna	gain	BASIS
PD5930	DKK2	cna	hetloss	BASIS
PD5935	DKK2	cna	gain	BASIS
PD5945	DKK2	cna	gain	BASIS
PD5948	DKK2	cna	gain	BASIS
PD6406	DKK2	cna	hetloss	BASIS
PD7067	DKK2	cna	gain	BASIS
PD7215	DKK2	cna	gain	BASIS
PD8621	DKK2	cna	hetloss	BASIS
PD8980	DKK2	cna	hetloss	BASIS
PD9585	DKK2	cna	hetloss	BASIS
TCGA-AN-A0XU	EBF1	cna	hetloss	TCGA
TCGA-AO-A0JL	EBF1	cna	gain	TCGA
TCGA-BH-A0C0	EBF1	cna	hetloss	TCGA
TCGA-BH-A1FU	EBF1	cna	hetloss	TCGA
TCGA-C8-A12L	EBF1	cna	hetloss	TCGA
TCGA-D8-A27M	EBF1	cna	hetloss	TCGA
TCGA-E2-A1L7	EBF1	cna	hetloss	TCGA
TCGA-E9-A1NC	EBF1	cna	hetloss	TCGA

TCGA-LL-A5YP	EBF1	cna	hetloss	TCGA
MB-0346	EBF1	cna	gain	METABRIC
MB-5070	EBF1	cna	hetloss	METABRIC
MB-5465	EBF1	cna	hetloss	METABRIC
MB-6098	EBF1	cna	hetloss	METABRIC
MB-7038	EBF1	cna	hetloss	METABRIC
MB-0420	EBF1	cna	hetloss	METABRIC
PD10014	EBF1	cna	hetloss	BASIS
PD11327	EBF1	cna	gain	BASIS
PD13296	EBF1	cna	hetloss	BASIS
PD13297	EBF1	cna	hetloss	BASIS
PD13771	EBF1	cna	gain	BASIS
PD22355	EBF1	cna	hetloss	BASIS
PD23578	EBF1	cna	hetloss	BASIS
PD24186	EBF1	cna	gain	BASIS
PD24202	EBF1	cna	hetloss	BASIS
PD24206	EBF1	cna	gain	BASIS
PD24337	EBF1	cna	hetloss	BASIS
PD3890	EBF1	cna	hetloss	BASIS
PD3905	EBF1	cna	gain	BASIS
PD4005	EBF1	cna	hetloss	BASIS
PD4006	EBF1	cna	hetloss	BASIS
PD4107	EBF1	cna	hetloss	BASIS
PD4826	EBF1	cna	gain	BASIS
PD5935	EBF1	cna	gain	BASIS
PD5945	EBF1	cna	gain	BASIS
PD5948	EBF1	cna	hetloss	BASIS
PD6406	EBF1	cna	hetloss	BASIS
PD6413	EBF1	cna	hetloss	BASIS
PD8621	EBF1	cna	hetloss	BASIS
PD8980	EBF1	cna	hetloss	BASIS
PD9585	EBF1	cna	hetloss	BASIS
TCGA-A2-A25B	ESCO2	cna	hetloss	TCGA
TCGA-AN-A0XU	ESCO2	cna	hetloss	TCGA
TCGA-AO-A0JL	ESCO2	cna	hetloss	TCGA
TCGA-BH-A0C0	ESCO2	cna	hetloss	TCGA
TCGA-BH-A1FU	ESCO2	cna	homdel	TCGA
TCGA-C8-A12L	ESCO2	cna	hetloss	TCGA
TCGA-D8-A27M	ESCO2	cna	hetloss	TCGA
TCGA-E2-A1L7	ESCO2	cna	hetloss	TCGA
TCGA-LL-A5YP	ESCO2	cna	hetloss	TCGA
MB-0346	ESCO2	cna	hetloss	METABRIC
MB-2827	ESCO2	cna	hetloss	METABRIC
MB-5070	ESCO2	cna	hetloss	METABRIC
MB-5465	ESCO2	cna	hetloss	METABRIC
MB-6060	ESCO2	cna	hetloss	METABRIC
MB-7038	ESCO2	cna	hetloss	METABRIC

MB-0420	ESCO2	cna	hetloss	METABRIC
PD10014	ESCO2	cna	hetloss	BASIS
PD11327	ESCO2	cna	hetloss	BASIS
PD13296	ESCO2	cna	hetloss	BASIS
PD13299	ESCO2	cna	hetloss	BASIS
PD13771	ESCO2	cna	hetloss	BASIS
PD14442	ESCO2	cna	hetloss	BASIS
PD22355	ESCO2	cna	hetloss	BASIS
PD23562	ESCO2	cna	hetloss	BASIS
PD23574	ESCO2	cna	hetloss	BASIS
PD23578	ESCO2	cna	hetloss	BASIS
PD24186	ESCO2	cna	gain	BASIS
PD24202	ESCO2	cna	hetloss	BASIS
PD24206	ESCO2	cna	gain	BASIS
PD24337	ESCO2	cna	hetloss	BASIS
PD3905	ESCO2	cna	gain	BASIS
PD4005	ESCO2	cna	hetloss	BASIS
PD4006	ESCO2	cna	gain	BASIS
PD4826	ESCO2	cna	hetloss	BASIS
PD5935	ESCO2	cna	gain	BASIS
PD5945	ESCO2	cna	gain	BASIS
PD6406	ESCO2	cna	hetloss	BASIS
PD6413	ESCO2	cna	hetloss	BASIS
PD7067	ESCO2	cna	hetloss	BASIS
PD9004	ESCO2	cna	hetloss	BASIS
TCGA-A2-A25B	EZH2	cna	gain	TCGA
TCGA-AO-A0JL	EZH2	cna	gain	TCGA
TCGA-C8-A12L	EZH2	cna	hetloss	TCGA
TCGA-D8-A27M	EZH2	cna	gain	TCGA
TCGA-E2-A1L7	EZH2	cna	hetloss	TCGA
TCGA-E9-A1NC	EZH2	cna	hetloss	TCGA
TCGA-LL-A5YP	EZH2	cna	hetloss	TCGA
MB-0346	EZH2	cna	hetloss	METABRIC
MB-5070	EZH2	cna	hetloss	METABRIC
MB-6098	EZH2	cna	gain	METABRIC
MB-7038	EZH2	cna	gain	METABRIC
MB-7048	EZH2	cna	gain	METABRIC
MB-0420	EZH2	cna	hetloss	METABRIC
MTS-T0064	EZH2	cna	amp	METABRIC
P-0009557-T01-IM5	EZH2	cna	homdel	MSK-IMPACT
PD10014	EZH2	cna	gain	BASIS
PD13296	EZH2	cna	hetloss	BASIS
PD13297	EZH2	cna	gain	BASIS
PD22355	EZH2	cna	hetloss	BASIS
PD23562	EZH2	cna	gain	BASIS
PD23574	EZH2	cna	gain	BASIS
PD23578	EZH2	cna	hetloss	BASIS

PD24186	EZH2	cna	gain	BASIS
PD24202	EZH2	cna	gain	BASIS
PD24337	EZH2	cna	gain	BASIS
PD3890	EZH2	cna	hetloss	BASIS
PD3905	EZH2	cna	gain	BASIS
PD4006	EZH2	cna	amp	BASIS
PD4107	EZH2	cna	gain	BASIS
PD5930	EZH2	cna	gain	BASIS
PD5945	EZH2	cna	amp	BASIS
PD5948	EZH2	cna	gain	BASIS
PD6406	EZH2	cna	gain	BASIS
PD6731	EZH2	cna	hetloss	BASIS
PD7067	EZH2	cna	gain	BASIS
PD7215	EZH2	cna	gain	BASIS
PD8621	EZH2	cna	gain	BASIS
PD9004	EZH2	cna	gain	BASIS
PD9585	EZH2	cna	hetloss	BASIS
PD9702	EZH2	cna	gain	BASIS
TCGA-A2-A25B	EZR	cna	hetloss	TCGA
TCGA-AN-A0XU	EZR	cna	gain	TCGA
TCGA-AO-A0JL	EZR	cna	hetloss	TCGA
TCGA-BH-A0AW	EZR	cna	amp	TCGA
TCGA-BH-A0C0	EZR	cna	hetloss	TCGA
TCGA-C8-A12L	EZR	cna	gain	TCGA
TCGA-D8-A27M	EZR	cna	hetloss	TCGA
TCGA-E9-A1NC	EZR	cna	hetloss	TCGA
MB-2827	EZR	cna	hetloss	METABRIC
MB-5070	EZR	cna	gain	METABRIC
MB-5107	EZR	cna	hetloss	METABRIC
MB-6060	EZR	cna	hetloss	METABRIC
MB-6098	EZR	cna	hetloss	METABRIC
MB-7048	EZR	cna	hetloss	METABRIC
MB-0420	EZR	cna	hetloss	METABRIC
MTS-T0064	EZR	cna	hetloss	METABRIC
PD10014	EZR	cna	hetloss	BASIS
PD13296	EZR	cna	homdel	BASIS
PD13297	EZR	cna	hetloss	BASIS
PD13299	EZR	cna	gain	BASIS
PD23562	EZR	cna	gain	BASIS
PD23574	EZR	cna	gain	BASIS
PD24186	EZR	cna	gain	BASIS
PD3890	EZR	cna	hetloss	BASIS
PD3905	EZR	cna	gain	BASIS
PD4005	EZR	cna	hetloss	BASIS
PD4006	EZR	cna	gain	BASIS
PD4107	EZR	cna	gain	BASIS
PD4826	EZR	cna	gain	BASIS

PD4967	EZR	cna	hetloss	BASIS
PD5935	EZR	cna	gain	BASIS
PD5945	EZR	cna	gain	BASIS
PD6406	EZR	cna	hetloss	BASIS
PD6413	EZR	cna	hetloss	BASIS
PD6731	EZR	cna	hetloss	BASIS
PD7067	EZR	cna	amp	BASIS
PD8980	EZR	cna	gain	BASIS
PD9004	EZR	cna	gain	BASIS
PD9585	EZR	cna	hetloss	BASIS
PD9702	EZR	cna	gain	BASIS
TCGA-A2-A25B	FLCN	cna	hetloss	TCGA
TCGA-AN-A0XU	FLCN	cna	hetloss	TCGA
TCGA-AO-A0JL	FLCN	cna	gain	TCGA
TCGA-BH-A0AW	FLCN	cna	hetloss	TCGA
TCGA-BH-A0C0	FLCN	cna	hetloss	TCGA
TCGA-C8-A12L	FLCN	cna	hetloss	TCGA
TCGA-D8-A27M	FLCN	cna	hetloss	TCGA
TCGA-E2-A1L7	FLCN	cna	hetloss	TCGA
TCGA-E9-A1NC	FLCN	cna	hetloss	TCGA
TCGA-LL-A5YP	FLCN	cna	gain	TCGA
MB-0346	FLCN	cna	hetloss	METABRIC
MB-2827	FLCN	cna	hetloss	METABRIC
MB-5070	FLCN	cna	hetloss	METABRIC
MB-5107	FLCN	cna	hetloss	METABRIC
MB-5465	FLCN	cna	hetloss	METABRIC
MB-6098	FLCN	cna	hetloss	METABRIC
MB-6271	FLCN	cna	hetloss	METABRIC
MB-7038	FLCN	cna	hetloss	METABRIC
MB-7048	FLCN	cna	hetloss	METABRIC
MB-0420	FLCN	cna	hetloss	METABRIC
PD11327	FLCN	cna	hetloss	BASIS
PD11742	FLCN	cna	hetloss	BASIS
PD13297	FLCN	cna	hetloss	BASIS
PD13771	FLCN	cna	hetloss	BASIS
PD22355	FLCN	cna	hetloss	BASIS
PD23561	FLCN	cna	hetloss	BASIS
PD24202	FLCN	cna	hetloss	BASIS
PD24206	FLCN	cna	hetloss	BASIS
PD24337	FLCN	cna	hetloss	BASIS
PD3890	FLCN	cna	hetloss	BASIS
PD3905	FLCN	cna	gain	BASIS
PD4005	FLCN	cna	hetloss	BASIS
PD4826	FLCN	cna	hetloss	BASIS
PD4967	FLCN	cna	hetloss	BASIS
PD5945	FLCN	cna	gain	BASIS
PD6406	FLCN	cna	hetloss	BASIS

PD6413	FLCN	cna	hetloss	BASIS
PD6731	FLCN	cna	hetloss	BASIS
PD7067	FLCN	cna	gain	BASIS
PD9585	FLCN	cna	hetloss	BASIS
TCGA-AN-A0XU	FOXL2	cna	gain	TCGA
TCGA-BH-A0C0	FOXL2	cna	gain	TCGA
TCGA-BH-A18R	FOXL2	cna	gain	TCGA
TCGA-BH-A1FU	FOXL2	cna	hetloss	TCGA
TCGA-C8-A12L	FOXL2	cna	gain	TCGA
TCGA-E2-A1L7	FOXL2	cna	gain	TCGA
TCGA-E9-A1NC	FOXL2	cna	gain	TCGA
TCGA-LL-A5YP	FOXL2	cna	gain	TCGA
MB-0346	FOXL2	cna	hetloss	METABRIC
MB-5070	FOXL2	cna	gain	METABRIC
MB-5107	FOXL2	cna	gain	METABRIC
MB-5465	FOXL2	cna	gain	METABRIC
MB-7038	FOXL2	cna	gain	METABRIC
MB-7048	FOXL2	cna	hetloss	METABRIC
MTS-T0064	FOXL2	cna	amp	METABRIC
PD10014	FOXL2	cna	gain	BASIS
PD11327	FOXL2	cna	gain	BASIS
PD13299	FOXL2	cna	gain	BASIS
PD13771	FOXL2	cna	hetloss	BASIS
PD22355	FOXL2	cna	gain	BASIS
PD23561	FOXL2	cna	hetloss	BASIS
PD23574	FOXL2	cna	gain	BASIS
PD23578	FOXL2	cna	gain	BASIS
PD24186	FOXL2	cna	gain	BASIS
PD24202	FOXL2	cna	gain	BASIS
PD24206	FOXL2	cna	gain	BASIS
PD3905	FOXL2	cna	gain	BASIS
PD4005	FOXL2	cna	hetloss	BASIS
PD4107	FOXL2	cna	gain	BASIS
PD4826	FOXL2	cna	gain	BASIS
PD5935	FOXL2	cna	gain	BASIS
PD5945	FOXL2	cna	gain	BASIS
PD5948	FOXL2	cna	gain	BASIS
PD6731	FOXL2	cna	gain	BASIS
PD7067	FOXL2	cna	gain	BASIS
PD7215	FOXL2	cna	gain	BASIS
PD8621	FOXL2	cna	gain	BASIS
PD8980	FOXL2	cna	gain	BASIS
PD9004	FOXL2	cna	gain	BASIS
PD9702	FOXL2	cna	gain	BASIS
TCGA-A2-A25B	GATA3	cna	gain	TCGA
TCGA-AN-A0XU	GATA3	cna	amp	TCGA
TCGA-AO-A0JL	GATA3	cna	gain	TCGA

TCGA-BH-A0AW	GATA3	cna	gain	TCGA
TCGA-BH-A0C0	GATA3	cna	gain	TCGA
TCGA-C8-A12L	GATA3	cna	gain	TCGA
MB-5070	GATA3	cna	gain	METABRIC
MB-5465	GATA3	cna	amp	METABRIC
MB-6098	GATA3	cna	amp	METABRIC
MB-7038	GATA3	cna	hetloss	METABRIC
MTS-T0064	GATA3	cna	gain	METABRIC
PD10014	GATA3	cna	amp	BASIS
PD11327	GATA3	cna	amp	BASIS
PD13296	GATA3	cna	gain	BASIS
PD13299	GATA3	cna	gain	BASIS
PD13771	GATA3	cna	gain	BASIS
PD22355	GATA3	cna	gain	BASIS
PD23562	GATA3	cna	amp	BASIS
PD23574	GATA3	cna	gain	BASIS
PD24186	GATA3	cna	amp	BASIS
PD24202	GATA3	cna	gain	BASIS
PD24337	GATA3	cna	gain	BASIS
PD3905	GATA3	cna	gain	BASIS
PD4005	GATA3	cna	gain	BASIS
PD4006	GATA3	cna	amp	BASIS
PD4107	GATA3	cna	gain	BASIS
PD4826	GATA3	cna	gain	BASIS
PD5930	GATA3	cna	gain	BASIS
PD5935	GATA3	cna	gain	BASIS
PD5945	GATA3	cna	amp	BASIS
PD5948	GATA3	cna	gain	BASIS
PD6406	GATA3	cna	hetloss	BASIS
PD6413	GATA3	cna	gain	BASIS
PD6731	GATA3	cna	hetloss	BASIS
PD7067	GATA3	cna	amp	BASIS
PD7215	GATA3	cna	amp	BASIS
PD8621	GATA3	cna	gain	BASIS
PD8980	GATA3	cna	gain	BASIS
PD9004	GATA3	cna	gain	BASIS
PD9702	GATA3	cna	amp	BASIS
TCGA-A2-A25B	GTSE1	cna	hetloss	TCGA
TCGA-AN-A0XU	GTSE1	cna	hetloss	TCGA
TCGA-BH-A0AW	GTSE1	cna	hetloss	TCGA
TCGA-BH-A1FU	GTSE1	cna	gain	TCGA
TCGA-C8-A12L	GTSE1	cna	gain	TCGA
TCGA-D8-A27M	GTSE1	cna	gain	TCGA
TCGA-E9-A1NC	GTSE1	cna	gain	TCGA
MB-5070	GTSE1	cna	gain	METABRIC
MB-5465	GTSE1	cna	gain	METABRIC
MB-6098	GTSE1	cna	gain	METABRIC

MB-0420	GTSE1	cna	gain	METABRIC
MTS-T0064	GTSE1	cna	gain	METABRIC
PD11327	GTSE1	cna	amp	BASIS
PD11742	GTSE1	cna	hetloss	BASIS
PD13296	GTSE1	cna	hetloss	BASIS
PD13297	GTSE1	cna	hetloss	BASIS
PD13299	GTSE1	cna	gain	BASIS
PD14442	GTSE1	cna	hetloss	BASIS
PD22355	GTSE1	cna	gain	BASIS
PD23562	GTSE1	cna	gain	BASIS
PD23578	GTSE1	cna	gain	BASIS
PD24186	GTSE1	cna	gain	BASIS
PD24202	GTSE1	cna	hetloss	BASIS
PD3905	GTSE1	cna	gain	BASIS
PD4005	GTSE1	cna	gain	BASIS
PD4006	GTSE1	cna	gain	BASIS
PD4107	GTSE1	cna	gain	BASIS
PD4826	GTSE1	cna	gain	BASIS
PD5935	GTSE1	cna	amp	BASIS
PD5945	GTSE1	cna	amp	BASIS
PD5948	GTSE1	cna	hetloss	BASIS
PD6406	GTSE1	cna	gain	BASIS
PD6731	GTSE1	cna	hetloss	BASIS
PD7067	GTSE1	cna	gain	BASIS
PD7215	GTSE1	cna	gain	BASIS
PD8621	GTSE1	cna	gain	BASIS
PD8980	GTSE1	cna	hetloss	BASIS
PD9004	GTSE1	cna	gain	BASIS
PD9585	GTSE1	cna	hetloss	BASIS
PD9702	GTSE1	cna	gain	BASIS
TCGA-A2-A25B	H3F3C	cna	hetloss	TCGA
TCGA-AN-A0XU	H3F3C	cna	gain	TCGA
TCGA-AO-A0JL	H3F3C	cna	gain	TCGA
TCGA-BH-A0C0	H3F3C	cna	hetloss	TCGA
TCGA-BH-A18R	H3F3C	cna	gain	TCGA
TCGA-BH-A1FU	H3F3C	cna	hetloss	TCGA
TCGA-C8-A12L	H3F3C	cna	gain	TCGA
TCGA-D8-A27M	H3F3C	cna	amp	TCGA
TCGA-E2-A1L7	H3F3C	cna	hetloss	TCGA
TCGA-LL-A5YP	H3F3C	cna	hetloss	TCGA
MB-0346	H3F3C	cna	gain	METABRIC
MB-2827	H3F3C	cna	hetloss	METABRIC
MB-5465	H3F3C	cna	hetloss	METABRIC
MB-6098	H3F3C	cna	gain	METABRIC
MB-7038	H3F3C	cna	hetloss	METABRIC
MTS-T0064	H3F3C	cna	gain	METABRIC
PD10014	H3F3C	cna	gain	BASIS

PD11327	H3F3C	cna	gain	BASIS
PD13296	H3F3C	cna	gain	BASIS
PD13299	H3F3C	cna	gain	BASIS
PD13771	H3F3C	cna	hetloss	BASIS
PD23574	H3F3C	cna	gain	BASIS
PD23578	H3F3C	cna	gain	BASIS
PD24186	H3F3C	cna	gain	BASIS
PD3905	H3F3C	cna	gain	BASIS
PD4005	H3F3C	cna	hetloss	BASIS
PD4006	H3F3C	cna	gain	BASIS
PD4107	H3F3C	cna	gain	BASIS
PD4826	H3F3C	cna	hetloss	BASIS
PD5930	H3F3C	cna	amp	BASIS
PD5935	H3F3C	cna	gain	BASIS
PD5945	H3F3C	cna	gain	BASIS
PD5948	H3F3C	cna	hetloss	BASIS
PD6731	H3F3C	cna	hetloss	BASIS
PD7067	H3F3C	cna	gain	BASIS
PD7215	H3F3C	cna	gain	BASIS
PD8621	H3F3C	cna	gain	BASIS
PD8980	H3F3C	cna	hetloss	BASIS
PD9004	H3F3C	cna	amp	BASIS
PD9702	H3F3C	cna	gain	BASIS
TCGA-A2-A25B	HRAS	cna	hetloss	TCGA
TCGA-AN-A0XU	HRAS	cna	hetloss	TCGA
TCGA-AO-A0JL	HRAS	cna	hetloss	TCGA
TCGA-BH-A0AW	HRAS	cna	hetloss	TCGA
TCGA-BH-A0C0	HRAS	cna	hetloss	TCGA
TCGA-C8-A12L	HRAS	cna	hetloss	TCGA
TCGA-E2-A1L7	HRAS	cna	homdel	TCGA
TCGA-LL-A5YP	HRAS	cna	hetloss	TCGA
MB-0346	HRAS	cna	hetloss	METABRIC
MB-5070	HRAS	cna	hetloss	METABRIC
MB-6060	HRAS	cna	hetloss	METABRIC
MB-6098	HRAS	cna	hetloss	METABRIC
MB-6271	HRAS	cna	gain	METABRIC
MB-7032	HRAS	cna	gain	METABRIC
MB-7048	HRAS	cna	hetloss	METABRIC
MB-0420	HRAS	cna	hetloss	METABRIC
PD10014	HRAS	cna	hetloss	BASIS
PD11742	HRAS	cna	hetloss	BASIS
PD13296	HRAS	cna	hetloss	BASIS
PD13297	HRAS	cna	hetloss	BASIS
PD22355	HRAS	cna	hetloss	BASIS
PD23574	HRAS	cna	gain	BASIS
PD24202	HRAS	cna	hetloss	BASIS
PD24206	HRAS	cna	gain	BASIS

PD24337	HRAS	cna	gain	BASIS
PD3890	HRAS	cna	hetloss	BASIS
PD3905	HRAS	cna	gain	BASIS
PD4005	HRAS	cna	hetloss	BASIS
PD4006	HRAS	cna	gain	BASIS
PD4107	HRAS	cna	gain	BASIS
PD4826	HRAS	cna	gain	BASIS
PD4967	HRAS	cna	gain	BASIS
PD5935	HRAS	cna	gain	BASIS
PD5945	HRAS	cna	gain	BASIS
PD5948	HRAS	cna	gain	BASIS
PD6413	HRAS	cna	hetloss	BASIS
PD6731	HRAS	cna	hetloss	BASIS
PD8980	HRAS	cna	hetloss	BASIS
PD9004	HRAS	cna	hetloss	BASIS
PD9585	HRAS	cna	hetloss	BASIS
TCGA-A2-A25B	HSP90AA1	cna	hetloss	TCGA
TCGA-AN-A0XU	HSP90AA1	cna	gain	TCGA
TCGA-AO-A0JL	HSP90AA1	cna	hetloss	TCGA
TCGA-BH-A0AW	HSP90AA1	cna	hetloss	TCGA
TCGA-BH-A1FU	HSP90AA1	cna	gain	TCGA
TCGA-C8-A12L	HSP90AA1	cna	gain	TCGA
TCGA-D8-A27M	HSP90AA1	cna	hetloss	TCGA
TCGA-E2-A1L7	HSP90AA1	cna	gain	TCGA
TCGA-E9-A1NC	HSP90AA1	cna	hetloss	TCGA
TCGA-LL-A5YP	HSP90AA1	cna	hetloss	TCGA
MB-0346	HSP90AA1	cna	gain	METABRIC
MB-2827	HSP90AA1	cna	hetloss	METABRIC
MB-5070	HSP90AA1	cna	hetloss	METABRIC
MB-5107	HSP90AA1	cna	hetloss	METABRIC
MB-6060	HSP90AA1	cna	gain	METABRIC
MB-6098	HSP90AA1	cna	hetloss	METABRIC
MTS-T0064	HSP90AA1	cna	hetloss	METABRIC
PD10014	HSP90AA1	cna	hetloss	BASIS
PD11742	HSP90AA1	cna	hetloss	BASIS
PD13296	HSP90AA1	cna	gain	BASIS
PD13297	HSP90AA1	cna	hetloss	BASIS
PD13771	HSP90AA1	cna	hetloss	BASIS
PD22355	HSP90AA1	cna	hetloss	BASIS
PD23578	HSP90AA1	cna	hetloss	BASIS
PD24202	HSP90AA1	cna	hetloss	BASIS
PD24206	HSP90AA1	cna	gain	BASIS
PD24337	HSP90AA1	cna	hetloss	BASIS
PD3890	HSP90AA1	cna	hetloss	BASIS
PD3905	HSP90AA1	cna	gain	BASIS
PD4006	HSP90AA1	cna	gain	BASIS
PD4826	HSP90AA1	cna	gain	BASIS

PD4967	HSP90AA1	cna	hetloss	BASIS
PD5945	HSP90AA1	cna	gain	BASIS
PD5948	HSP90AA1	cna	gain	BASIS
PD6406	HSP90AA1	cna	hetloss	BASIS
PD6413	HSP90AA1	cna	hetloss	BASIS
PD7067	HSP90AA1	cna	gain	BASIS
PD8621	HSP90AA1	cna	gain	BASIS
PD8980	HSP90AA1	cna	hetloss	BASIS
PD9702	HSP90AA1	cna	gain	BASIS
TCGA-A2-A25B	IGF1R	cna	amp	TCGA
TCGA-AN-A0XU	IGF1R	cna	gain	TCGA
TCGA-AO-A0JL	IGF1R	cna	hetloss	TCGA
TCGA-BH-A0C0	IGF1R	cna	gain	TCGA
TCGA-D8-A27M	IGF1R	cna	hetloss	TCGA
TCGA-E2-A1L7	IGF1R	cna	amp	TCGA
TCGA-LL-A5YP	IGF1R	cna	amp	TCGA
MB-0346	IGF1R	cna	hetloss	METABRIC
MB-2827	IGF1R	cna	amp	METABRIC
MB-5070	IGF1R	cna	gain	METABRIC
MB-5465	IGF1R	cna	hetloss	METABRIC
MB-6060	IGF1R	cna	hetloss	METABRIC
MB-7038	IGF1R	cna	gain	METABRIC
MB-7048	IGF1R	cna	gain	METABRIC
MB-0420	IGF1R	cna	gain	METABRIC
MTS-T0064	IGF1R	cna	gain	METABRIC
PD10014	IGF1R	cna	gain	BASIS
PD11327	IGF1R	cna	gain	BASIS
PD11742	IGF1R	cna	hetloss	BASIS
PD13296	IGF1R	cna	gain	BASIS
PD13299	IGF1R	cna	amp	BASIS
PD23562	IGF1R	cna	gain	BASIS
PD23574	IGF1R	cna	gain	BASIS
PD23578	IGF1R	cna	gain	BASIS
PD24202	IGF1R	cna	hetloss	BASIS
PD24206	IGF1R	cna	gain	BASIS
PD24337	IGF1R	cna	gain	BASIS
PD3890	IGF1R	cna	hetloss	BASIS
PD3905	IGF1R	cna	gain	BASIS
PD4006	IGF1R	cna	amp	BASIS
PD4107	IGF1R	cna	gain	BASIS
PD5930	IGF1R	cna	gain	BASIS
PD5945	IGF1R	cna	amp	BASIS
PD5948	IGF1R	cna	gain	BASIS
PD6413	IGF1R	cna	hetloss	BASIS
PD7067	IGF1R	cna	gain	BASIS
PD8621	IGF1R	cna	gain	BASIS
PD8980	IGF1R	cna	gain	BASIS

PD9585	IGF1R	cna	hetloss	BASIS
PD9702	IGF1R	cna	gain	BASIS
TCGA-A2-A25B	IL2	cna	gain	TCGA
TCGA-AN-A0XU	IL2	cna	hetloss	TCGA
TCGA-BH-A0AW	IL2	cna	hetloss	TCGA
TCGA-BH-A0C0	IL2	cna	hetloss	TCGA
TCGA-BH-A1FU	IL2	cna	gain	TCGA
TCGA-C8-A12L	IL2	cna	homdel	TCGA
TCGA-D8-A27M	IL2	cna	hetloss	TCGA
TCGA-E2-A1L7	IL2	cna	hetloss	TCGA
TCGA-E9-A1NC	IL2	cna	hetloss	TCGA
TCGA-LL-A5YP	IL2	cna	homdel	TCGA
MB-5070	IL2	cna	hetloss	METABRIC
MB-5465	IL2	cna	gain	METABRIC
MB-7048	IL2	cna	hetloss	METABRIC
MB-0420	IL2	cna	hetloss	METABRIC
PD10014	IL2	cna	gain	BASIS
PD11742	IL2	cna	hetloss	BASIS
PD13296	IL2	cna	hetloss	BASIS
PD13297	IL2	cna	hetloss	BASIS
PD22355	IL2	cna	hetloss	BASIS
PD23562	IL2	cna	gain	BASIS
PD23574	IL2	cna	gain	BASIS
PD23578	IL2	cna	hetloss	BASIS
PD24202	IL2	cna	hetloss	BASIS
PD24206	IL2	cna	gain	BASIS
PD24337	IL2	cna	hetloss	BASIS
PD3905	IL2	cna	gain	BASIS
PD4005	IL2	cna	hetloss	BASIS
PD4006	IL2	cna	hetloss	BASIS
PD4107	IL2	cna	gain	BASIS
PD4826	IL2	cna	gain	BASIS
PD5935	IL2	cna	gain	BASIS
PD5945	IL2	cna	amp	BASIS
PD5948	IL2	cna	gain	BASIS
PD6406	IL2	cna	hetloss	BASIS
PD6731	IL2	cna	hetloss	BASIS
PD7067	IL2	cna	gain	BASIS
PD7215	IL2	cna	gain	BASIS
PD8621	IL2	cna	hetloss	BASIS
PD8980	IL2	cna	hetloss	BASIS
PD9585	IL2	cna	hetloss	BASIS
TCGA-AN-A0XU	ITK	cna	hetloss	TCGA
TCGA-AO-A0JL	ITK	cna	gain	TCGA
TCGA-BH-A0C0	ITK	cna	hetloss	TCGA
TCGA-BH-A1FU	ITK	cna	hetloss	TCGA
TCGA-C8-A12L	ITK	cna	hetloss	TCGA

TCGA-D8-A27M	ITK	cna	hetloss	TCGA
TCGA-E2-A1L7	ITK	cna	hetloss	TCGA
TCGA-E9-A1NC	ITK	cna	hetloss	TCGA
TCGA-LL-A5YP	ITK	cna	hetloss	TCGA
MB-0346	ITK	cna	gain	METABRIC
MB-5070	ITK	cna	hetloss	METABRIC
MB-5465	ITK	cna	hetloss	METABRIC
MB-6098	ITK	cna	hetloss	METABRIC
MB-7038	ITK	cna	hetloss	METABRIC
MB-0420	ITK	cna	hetloss	METABRIC
PD10014	ITK	cna	hetloss	BASIS
PD11327	ITK	cna	hetloss	BASIS
PD13296	ITK	cna	hetloss	BASIS
PD13297	ITK	cna	hetloss	BASIS
PD13771	ITK	cna	gain	BASIS
PD22355	ITK	cna	hetloss	BASIS
PD23578	ITK	cna	hetloss	BASIS
PD24186	ITK	cna	gain	BASIS
PD24202	ITK	cna	hetloss	BASIS
PD24206	ITK	cna	gain	BASIS
PD24337	ITK	cna	hetloss	BASIS
PD3890	ITK	cna	hetloss	BASIS
PD3905	ITK	cna	gain	BASIS
PD4005	ITK	cna	hetloss	BASIS
PD4006	ITK	cna	hetloss	BASIS
PD4107	ITK	cna	hetloss	BASIS
PD4826	ITK	cna	gain	BASIS
PD5935	ITK	cna	gain	BASIS
PD5945	ITK	cna	gain	BASIS
PD5948	ITK	cna	hetloss	BASIS
PD6406	ITK	cna	hetloss	BASIS
PD6413	ITK	cna	hetloss	BASIS
PD8621	ITK	cna	hetloss	BASIS
PD8980	ITK	cna	hetloss	BASIS
PD9585	ITK	cna	hetloss	BASIS
TCGA-A2-A25B	JARID2	cna	gain	TCGA
TCGA-AN-A0XU	JARID2	cna	hetloss	TCGA
TCGA-AO-A0JL	JARID2	cna	gain	TCGA
TCGA-BH-A0AW	JARID2	cna	gain	TCGA
TCGA-BH-A0C0	JARID2	cna	hetloss	TCGA
TCGA-BH-A18R	JARID2	cna	homdel	TCGA
TCGA-C8-A12L	JARID2	cna	hetloss	TCGA
TCGA-D8-A27M	JARID2	cna	hetloss	TCGA
TCGA-E2-A1L7	JARID2	cna	hetloss	TCGA
MB-0346	JARID2	cna	hetloss	METABRIC
MB-2827	JARID2	cna	amp	METABRIC
MB-5070	JARID2	cna	amp	METABRIC

MB-5465	JARID2	cna	gain	METABRIC
MB-6060	JARID2	cna	gain	METABRIC
MB-6098	JARID2	cna	hetloss	METABRIC
PD11327	JARID2	cna	amp	BASIS
PD13296	JARID2	cna	gain	BASIS
PD13297	JARID2	cna	gain	BASIS
PD13299	JARID2	cna	gain	BASIS
PD22355	JARID2	cna	gain	BASIS
PD23562	JARID2	cna	gain	BASIS
PD23574	JARID2	cna	gain	BASIS
PD24186	JARID2	cna	gain	BASIS
PD24202	JARID2	cna	gain	BASIS
PD24206	JARID2	cna	gain	BASIS
PD3905	JARID2	cna	amp	BASIS
PD4005	JARID2	cna	hetloss	BASIS
PD4006	JARID2	cna	gain	BASIS
PD4107	JARID2	cna	gain	BASIS
PD4826	JARID2	cna	gain	BASIS
PD5935	JARID2	cna	gain	BASIS
PD5945	JARID2	cna	amp	BASIS
PD5948	JARID2	cna	gain	BASIS
PD6731	JARID2	cna	hetloss	BASIS
PD7067	JARID2	cna	gain	BASIS
PD7215	JARID2	cna	gain	BASIS
PD8621	JARID2	cna	amp	BASIS
PD9004	JARID2	cna	gain	BASIS
PD9585	JARID2	cna	hetloss	BASIS
PD9702	JARID2	cna	gain	BASIS
TCGA-A2-A25B	KIAA1109	cna	gain	TCGA
TCGA-AN-A0XU	KIAA1109	cna	hetloss	TCGA
TCGA-BH-A0AW	KIAA1109	cna	hetloss	TCGA
TCGA-BH-A0C0	KIAA1109	cna	hetloss	TCGA
TCGA-BH-A1FU	KIAA1109	cna	gain	TCGA
TCGA-C8-A12L	KIAA1109	cna	homdel	TCGA
TCGA-D8-A27M	KIAA1109	cna	hetloss	TCGA
TCGA-E2-A1L7	KIAA1109	cna	hetloss	TCGA
TCGA-E9-A1NC	KIAA1109	cna	hetloss	TCGA
TCGA-LL-A5YP	KIAA1109	cna	gain	TCGA
MB-5070	KIAA1109	cna	hetloss	METABRIC
MB-5465	KIAA1109	cna	gain	METABRIC
MB-7048	KIAA1109	cna	hetloss	METABRIC
MB-0420	KIAA1109	cna	hetloss	METABRIC
PD10014	KIAA1109	cna	gain	BASIS
PD11742	KIAA1109	cna	hetloss	BASIS
PD13296	KIAA1109	cna	hetloss	BASIS
PD13297	KIAA1109	cna	hetloss	BASIS
PD22355	KIAA1109	cna	hetloss	BASIS

PD23562	KIAA1109	cna	gain	BASIS
PD23574	KIAA1109	cna	gain	BASIS
PD23578	KIAA1109	cna	hetloss	BASIS
PD24202	KIAA1109	cna	hetloss	BASIS
PD24206	KIAA1109	cna	gain	BASIS
PD24337	KIAA1109	cna	hetloss	BASIS
PD3905	KIAA1109	cna	gain	BASIS
PD4005	KIAA1109	cna	hetloss	BASIS
PD4006	KIAA1109	cna	hetloss	BASIS
PD4107	KIAA1109	cna	gain	BASIS
PD4826	KIAA1109	cna	gain	BASIS
PD5935	KIAA1109	cna	gain	BASIS
PD5945	KIAA1109	cna	amp	BASIS
PD5948	KIAA1109	cna	gain	BASIS
PD6406	KIAA1109	cna	hetloss	BASIS
PD6731	KIAA1109	cna	hetloss	BASIS
PD7067	KIAA1109	cna	gain	BASIS
PD7215	KIAA1109	cna	gain	BASIS
PD8621	KIAA1109	cna	hetloss	BASIS
PD8980	KIAA1109	cna	hetloss	BASIS
PD9585	KIAA1109	cna	hetloss	BASIS
TCGA-A2-A25B	LEF1	cna	hetloss	TCGA
TCGA-AN-A0XU	LEF1	cna	hetloss	TCGA
TCGA-BH-A0AW	LEF1	cna	hetloss	TCGA
TCGA-BH-A0C0	LEF1	cna	hetloss	TCGA
TCGA-BH-A1FU	LEF1	cna	gain	TCGA
TCGA-C8-A12L	LEF1	cna	hetloss	TCGA
TCGA-D8-A27M	LEF1	cna	hetloss	TCGA
TCGA-E2-A1L7	LEF1	cna	hetloss	TCGA
TCGA-E9-A1NC	LEF1	cna	hetloss	TCGA
TCGA-LL-A5YP	LEF1	cna	gain	TCGA
MB-2827	LEF1	cna	hetloss	METABRIC
MB-5070	LEF1	cna	hetloss	METABRIC
MB-5465	LEF1	cna	hetloss	METABRIC
MB-7048	LEF1	cna	hetloss	METABRIC
MB-0420	LEF1	cna	hetloss	METABRIC
PD10014	LEF1	cna	gain	BASIS
PD11742	LEF1	cna	hetloss	BASIS
PD13296	LEF1	cna	hetloss	BASIS
PD13297	LEF1	cna	hetloss	BASIS
PD22355	LEF1	cna	hetloss	BASIS
PD23562	LEF1	cna	gain	BASIS
PD23578	LEF1	cna	hetloss	BASIS
PD24202	LEF1	cna	hetloss	BASIS
PD24206	LEF1	cna	gain	BASIS
PD24337	LEF1	cna	hetloss	BASIS
PD3890	LEF1	cna	gain	BASIS

PD3905	LEF1	cna	gain	BASIS
PD4006	LEF1	cna	hetloss	BASIS
PD4107	LEF1	cna	gain	BASIS
PD4826	LEF1	cna	gain	BASIS
PD5930	LEF1	cna	hetloss	BASIS
PD5935	LEF1	cna	gain	BASIS
PD5945	LEF1	cna	gain	BASIS
PD5948	LEF1	cna	gain	BASIS
PD6406	LEF1	cna	hetloss	BASIS
PD7067	LEF1	cna	gain	BASIS
PD7215	LEF1	cna	gain	BASIS
PD8621	LEF1	cna	hetloss	BASIS
PD8980	LEF1	cna	hetloss	BASIS
PD9585	LEF1	cna	hetloss	BASIS
TCGA-A2-A25B	MED12	cna	gain	TCGA
TCGA-AN-A0XU	MED12	cna	hetloss	TCGA
TCGA-AO-A0JL	MED12	cna	gain	TCGA
TCGA-BH-A0AW	MED12	cna	gain	TCGA
TCGA-C8-A12L	MED12	cna	hetloss	TCGA
TCGA-E2-A1L7	MED12	cna	hetloss	TCGA
TCGA-E9-A1NC	MED12	cna	hetloss	TCGA
MB-0346	MED12	cna	hetloss	METABRIC
MB-2827	MED12	cna	hetloss	METABRIC
MB-5465	MED12	cna	gain	METABRIC
MB-6060	MED12	cna	hetloss	METABRIC
MB-0420	MED12	cna	hetloss	METABRIC
PD10014	MED12	cna	hetloss	BASIS
PD11327	MED12	cna	hetloss	BASIS
PD13296	MED12	cna	amp	BASIS
PD13297	MED12	cna	gain	BASIS
PD13299	MED12	cna	gain	BASIS
PD13771	MED12	cna	gain	BASIS
PD14442	MED12	cna	gain	BASIS
PD23562	MED12	cna	hetloss	BASIS
PD23574	MED12	cna	gain	BASIS
PD24202	MED12	cna	gain	BASIS
PD24206	MED12	cna	gain	BASIS
PD3890	MED12	cna	gain	BASIS
PD3905	MED12	cna	gain	BASIS
PD4005	MED12	cna	hetloss	BASIS
PD4006	MED12	cna	gain	BASIS
PD4107	MED12	cna	amp	BASIS
PD4826	MED12	cna	gain	BASIS
PD4967	MED12	cna	gain	BASIS
PD5930	MED12	cna	gain	BASIS
PD5935	MED12	cna	gain	BASIS
PD5948	MED12	cna	gain	BASIS

PD6406	MED12	cna	gain	BASIS
PD6413	MED12	cna	gain	BASIS
PD7067	MED12	cna	gain	BASIS
PD7215	MED12	cna	gain	BASIS
PD8621	MED12	cna	amp	BASIS
PD8980	MED12	cna	gain	BASIS
PD9004	MED12	cna	gain	BASIS
TCGA-A2-A25B	MNX1	cna	gain	TCGA
TCGA-AN-A0XU	MNX1	cna	hetloss	TCGA
TCGA-AO-A0JL	MNX1	cna	gain	TCGA
TCGA-C8-A12L	MNX1	cna	hetloss	TCGA
TCGA-D8-A27M	MNX1	cna	gain	TCGA
TCGA-E2-A1L7	MNX1	cna	hetloss	TCGA
TCGA-E9-A1NC	MNX1	cna	hetloss	TCGA
TCGA-LL-A5YP	MNX1	cna	hetloss	TCGA
MB-0346	MNX1	cna	hetloss	METABRIC
MB-5070	MNX1	cna	hetloss	METABRIC
MB-6098	MNX1	cna	gain	METABRIC
MB-7038	MNX1	cna	amp	METABRIC
MB-7048	MNX1	cna	gain	METABRIC
MB-0420	MNX1	cna	hetloss	METABRIC
PD11327	MNX1	cna	gain	BASIS
PD13296	MNX1	cna	homdel	BASIS
PD13297	MNX1	cna	gain	BASIS
PD22355	MNX1	cna	hetloss	BASIS
PD23562	MNX1	cna	gain	BASIS
PD23574	MNX1	cna	gain	BASIS
PD23578	MNX1	cna	hetloss	BASIS
PD24186	MNX1	cna	gain	BASIS
PD24202	MNX1	cna	gain	BASIS
PD24206	MNX1	cna	gain	BASIS
PD24337	MNX1	cna	hetloss	BASIS
PD3890	MNX1	cna	hetloss	BASIS
PD3905	MNX1	cna	gain	BASIS
PD4006	MNX1	cna	gain	BASIS
PD4107	MNX1	cna	gain	BASIS
PD5930	MNX1	cna	amp	BASIS
PD5945	MNX1	cna	amp	BASIS
PD5948	MNX1	cna	gain	BASIS
PD6406	MNX1	cna	gain	BASIS
PD6731	MNX1	cna	hetloss	BASIS
PD7215	MNX1	cna	gain	BASIS
PD8621	MNX1	cna	gain	BASIS
PD8980	MNX1	cna	gain	BASIS
PD9004	MNX1	cna	gain	BASIS
PD9585	MNX1	cna	hetloss	BASIS
PD9702	MNX1	cna	gain	BASIS

TCGA-A2-A25B	NCOR1	cna	hetloss	TCGA
TCGA-AN-A0XU	NCOR1	cna	hetloss	TCGA
TCGA-AO-A0JL	NCOR1	cna	gain	TCGA
TCGA-BH-A0AW	NCOR1	cna	hetloss	TCGA
TCGA-BH-A0C0	NCOR1	cna	hetloss	TCGA
TCGA-C8-A12L	NCOR1	cna	hetloss	TCGA
TCGA-D8-A27M	NCOR1	cna	hetloss	TCGA
TCGA-E2-A1L7	NCOR1	cna	hetloss	TCGA
TCGA-E9-A1NC	NCOR1	cna	hetloss	TCGA
TCGA-LL-A5YP	NCOR1	cna	gain	TCGA
MB-0346	NCOR1	cna	hetloss	METABRIC
MB-2827	NCOR1	cna	hetloss	METABRIC
MB-5070	NCOR1	cna	hetloss	METABRIC
MB-5107	NCOR1	cna	homdel	METABRIC
MB-5465	NCOR1	cna	hetloss	METABRIC
MB-6098	NCOR1	cna	hetloss	METABRIC
MB-6271	NCOR1	cna	hetloss	METABRIC
MB-7038	NCOR1	cna	hetloss	METABRIC
MB-7048	NCOR1	cna	hetloss	METABRIC
MB-0420	NCOR1	cna	hetloss	METABRIC
PD11742	NCOR1	cna	hetloss	BASIS
PD13296	NCOR1	cna	hetloss	BASIS
PD13297	NCOR1	cna	hetloss	BASIS
PD13771	NCOR1	cna	hetloss	BASIS
PD22355	NCOR1	cna	hetloss	BASIS
PD23561	NCOR1	cna	hetloss	BASIS
PD24202	NCOR1	cna	hetloss	BASIS
PD24206	NCOR1	cna	hetloss	BASIS
PD24337	NCOR1	cna	hetloss	BASIS
PD3890	NCOR1	cna	hetloss	BASIS
PD3905	NCOR1	cna	gain	BASIS
PD4005	NCOR1	cna	hetloss	BASIS
PD4826	NCOR1	cna	hetloss	BASIS
PD4967	NCOR1	cna	hetloss	BASIS
PD5945	NCOR1	cna	gain	BASIS
PD6406	NCOR1	cna	hetloss	BASIS
PD6413	NCOR1	cna	hetloss	BASIS
PD6731	NCOR1	cna	hetloss	BASIS
PD7067	NCOR1	cna	gain	BASIS
PD9585	NCOR1	cna	hetloss	BASIS
TCGA-A2-A25B	NFKB2	cna	hetloss	TCGA
TCGA-AN-A0XU	NFKB2	cna	hetloss	TCGA
TCGA-AO-A0JL	NFKB2	cna	hetloss	TCGA
TCGA-BH-A18R	NFKB2	cna	gain	TCGA
TCGA-C8-A12L	NFKB2	cna	hetloss	TCGA
TCGA-D8-A27M	NFKB2	cna	hetloss	TCGA
TCGA-E2-A1L7	NFKB2	cna	hetloss	TCGA

TCGA-LL-A5YP	NFKB2	cna	gain	TCGA
MB-0346	NFKB2	cna	gain	METABRIC
MB-2827	NFKB2	cna	hetloss	METABRIC
MB-5070	NFKB2	cna	hetloss	METABRIC
MB-6060	NFKB2	cna	hetloss	METABRIC
MB-6098	NFKB2	cna	hetloss	METABRIC
MB-7038	NFKB2	cna	hetloss	METABRIC
MB-7048	NFKB2	cna	hetloss	METABRIC
MB-0420	NFKB2	cna	hetloss	METABRIC
PD10014	NFKB2	cna	hetloss	BASIS
PD11327	NFKB2	cna	homdel	BASIS
PD11742	NFKB2	cna	hetloss	BASIS
PD22355	NFKB2	cna	hetloss	BASIS
PD23562	NFKB2	cna	gain	BASIS
PD23574	NFKB2	cna	gain	BASIS
PD23578	NFKB2	cna	hetloss	BASIS
PD24206	NFKB2	cna	homdel	BASIS
PD3890	NFKB2	cna	hetloss	BASIS
PD3905	NFKB2	cna	gain	BASIS
PD4005	NFKB2	cna	hetloss	BASIS
PD4006	NFKB2	cna	gain	BASIS
PD4107	NFKB2	cna	gain	BASIS
PD5935	NFKB2	cna	hetloss	BASIS
PD5945	NFKB2	cna	amp	BASIS
PD5948	NFKB2	cna	gain	BASIS
PD6406	NFKB2	cna	hetloss	BASIS
PD6731	NFKB2	cna	hetloss	BASIS
PD7067	NFKB2	cna	gain	BASIS
PD7215	NFKB2	cna	gain	BASIS
PD8621	NFKB2	cna	gain	BASIS
PD8980	NFKB2	cna	hetloss	BASIS
PD9585	NFKB2	cna	hetloss	BASIS
PD9702	NFKB2	cna	gain	BASIS
TCGA-A2-A25B	NONO	cna	gain	TCGA
TCGA-AN-A0XU	NONO	cna	hetloss	TCGA
TCGA-AO-A0JL	NONO	cna	gain	TCGA
TCGA-BH-A0AW	NONO	cna	gain	TCGA
TCGA-C8-A12L	NONO	cna	hetloss	TCGA
TCGA-E2-A1L7	NONO	cna	hetloss	TCGA
TCGA-E9-A1NC	NONO	cna	hetloss	TCGA
MB-0346	NONO	cna	hetloss	METABRIC
MB-2827	NONO	cna	hetloss	METABRIC
MB-5465	NONO	cna	gain	METABRIC
MB-6060	NONO	cna	hetloss	METABRIC
MB-0420	NONO	cna	hetloss	METABRIC
PD10014	NONO	cna	hetloss	BASIS
PD11327	NONO	cna	hetloss	BASIS

PD13296	NONO	cna	amp	BASIS
PD13297	NONO	cna	gain	BASIS
PD13299	NONO	cna	gain	BASIS
PD13771	NONO	cna	gain	BASIS
PD14442	NONO	cna	gain	BASIS
PD23562	NONO	cna	hetloss	BASIS
PD23574	NONO	cna	gain	BASIS
PD24202	NONO	cna	gain	BASIS
PD24206	NONO	cna	gain	BASIS
PD3890	NONO	cna	gain	BASIS
PD3905	NONO	cna	gain	BASIS
PD4005	NONO	cna	hetloss	BASIS
PD4006	NONO	cna	gain	BASIS
PD4107	NONO	cna	amp	BASIS
PD4826	NONO	cna	gain	BASIS
PD4967	NONO	cna	gain	BASIS
PD5930	NONO	cna	gain	BASIS
PD5935	NONO	cna	gain	BASIS
PD5948	NONO	cna	gain	BASIS
PD6406	NONO	cna	gain	BASIS
PD6413	NONO	cna	gain	BASIS
PD7067	NONO	cna	gain	BASIS
PD7215	NONO	cna	gain	BASIS
PD8621	NONO	cna	amp	BASIS
PD8980	NONO	cna	gain	BASIS
PD9004	NONO	cna	gain	BASIS
TCGA-A2-A25B	NT5C2	cna	hetloss	TCGA
TCGA-AN-A0XU	NT5C2	cna	hetloss	TCGA
TCGA-AO-A0JL	NT5C2	cna	hetloss	TCGA
TCGA-BH-A18R	NT5C2	cna	gain	TCGA
TCGA-C8-A12L	NT5C2	cna	hetloss	TCGA
TCGA-D8-A27M	NT5C2	cna	hetloss	TCGA
TCGA-E2-A1L7	NT5C2	cna	hetloss	TCGA
TCGA-LL-A5YP	NT5C2	cna	gain	TCGA
MB-0346	NT5C2	cna	gain	METABRIC
MB-2827	NT5C2	cna	hetloss	METABRIC
MB-5070	NT5C2	cna	hetloss	METABRIC
MB-6060	NT5C2	cna	hetloss	METABRIC
MB-6098	NT5C2	cna	hetloss	METABRIC
MB-7038	NT5C2	cna	hetloss	METABRIC
MB-7048	NT5C2	cna	hetloss	METABRIC
MB-0420	NT5C2	cna	hetloss	METABRIC
PD10014	NT5C2	cna	hetloss	BASIS
PD11327	NT5C2	cna	homdel	BASIS
PD11742	NT5C2	cna	hetloss	BASIS
PD22355	NT5C2	cna	hetloss	BASIS
PD23562	NT5C2	cna	gain	BASIS

PD23574	NT5C2	cna	gain	BASIS
PD23578	NT5C2	cna	hetloss	BASIS
PD24206	NT5C2	cna	homdel	BASIS
PD3890	NT5C2	cna	hetloss	BASIS
PD3905	NT5C2	cna	gain	BASIS
PD4005	NT5C2	cna	hetloss	BASIS
PD4006	NT5C2	cna	gain	BASIS
PD4107	NT5C2	cna	gain	BASIS
PD5935	NT5C2	cna	hetloss	BASIS
PD5945	NT5C2	cna	gain	BASIS
PD5948	NT5C2	cna	gain	BASIS
PD6406	NT5C2	cna	hetloss	BASIS
PD6731	NT5C2	cna	hetloss	BASIS
PD7067	NT5C2	cna	gain	BASIS
PD7215	NT5C2	cna	gain	BASIS
PD8621	NT5C2	cna	gain	BASIS
PD8980	NT5C2	cna	hetloss	BASIS
PD9585	NT5C2	cna	hetloss	BASIS
PD9702	NT5C2	cna	gain	BASIS
TCGA-AN-A0XU	NUP93	cna	hetloss	TCGA
TCGA-AO-A0JL	NUP93	cna	gain	TCGA
TCGA-BH-A0AW	NUP93	cna	hetloss	TCGA
TCGA-BH-A0C0	NUP93	cna	hetloss	TCGA
TCGA-C8-A12L	NUP93	cna	gain	TCGA
TCGA-D8-A27M	NUP93	cna	gain	TCGA
TCGA-E2-A1L7	NUP93	cna	hetloss	TCGA
TCGA-LL-A5YP	NUP93	cna	gain	TCGA
MB-0346	NUP93	cna	gain	METABRIC
MB-5070	NUP93	cna	hetloss	METABRIC
MB-5107	NUP93	cna	hetloss	METABRIC
MB-5465	NUP93	cna	hetloss	METABRIC
MB-6271	NUP93	cna	hetloss	METABRIC
MB-7032	NUP93	cna	gain	METABRIC
MB-7048	NUP93	cna	hetloss	METABRIC
MB-0420	NUP93	cna	hetloss	METABRIC
MTS-T0064	NUP93	cna	hetloss	METABRIC
PD10014	NUP93	cna	hetloss	BASIS
PD11327	NUP93	cna	hetloss	BASIS
PD11742	NUP93	cna	hetloss	BASIS
PD13296	NUP93	cna	hetloss	BASIS
PD13297	NUP93	cna	hetloss	BASIS
PD13299	NUP93	cna	gain	BASIS
PD14442	NUP93	cna	hetloss	BASIS
PD22355	NUP93	cna	hetloss	BASIS
PD23562	NUP93	cna	gain	BASIS
PD24186	NUP93	cna	gain	BASIS
PD24202	NUP93	cna	hetloss	BASIS

PD24206	NUP93	cna	gain	BASIS
PD24337	NUP93	cna	hetloss	BASIS
PD3905	NUP93	cna	gain	BASIS
PD4005	NUP93	cna	hetloss	BASIS
PD4967	NUP93	cna	hetloss	BASIS
PD5945	NUP93	cna	amp	BASIS
PD5948	NUP93	cna	hetloss	BASIS
PD7067	NUP93	cna	hetloss	BASIS
PD7215	NUP93	cna	gain	BASIS
PD8980	NUP93	cna	gain	BASIS
PD9585	NUP93	cna	hetloss	BASIS
PD9702	NUP93	cna	gain	BASIS
TCGA-AN-A0XU	PDE4DIP	cna	gain	TCGA
TCGA-AO-A0JL	PDE4DIP	cna	gain	TCGA
TCGA-BH-A0AW	PDE4DIP	cna	gain	TCGA
TCGA-BH-A0C0	PDE4DIP	cna	hetloss	TCGA
TCGA-BH-A18R	PDE4DIP	cna	gain	TCGA
TCGA-BH-A1FU	PDE4DIP	cna	gain	TCGA
TCGA-C8-A12L	PDE4DIP	cna	gain	TCGA
TCGA-E2-A1L7	PDE4DIP	cna	gain	TCGA
TCGA-EW-A1OX	PDE4DIP	cna	gain	TCGA
TCGA-LL-A5YP	PDE4DIP	cna	amp	TCGA
MB-2827	PDE4DIP	cna	amp	METABRIC
MB-6098	PDE4DIP	cna	amp	METABRIC
MB-7032	PDE4DIP	cna	gain	METABRIC
MB-7038	PDE4DIP	cna	gain	METABRIC
MB-0420	PDE4DIP	cna	hetloss	METABRIC
PD10014	PDE4DIP	cna	gain	BASIS
PD11327	PDE4DIP	cna	amp	BASIS
PD13296	PDE4DIP	cna	gain	BASIS
PD13297	PDE4DIP	cna	gain	BASIS
PD13299	PDE4DIP	cna	gain	BASIS
PD14442	PDE4DIP	cna	gain	BASIS
PD23562	PDE4DIP	cna	gain	BASIS
PD23574	PDE4DIP	cna	gain	BASIS
PD23578	PDE4DIP	cna	gain	BASIS
PD24186	PDE4DIP	cna	gain	BASIS
PD24206	PDE4DIP	cna	gain	BASIS
PD3905	PDE4DIP	cna	gain	BASIS
PD4006	PDE4DIP	cna	amp	BASIS
PD4107	PDE4DIP	cna	gain	BASIS
PD4826	PDE4DIP	cna	gain	BASIS
PD5930	PDE4DIP	cna	gain	BASIS
PD5935	PDE4DIP	cna	gain	BASIS
PD5945	PDE4DIP	cna	gain	BASIS
PD5948	PDE4DIP	cna	gain	BASIS
PD6413	PDE4DIP	cna	gain	BASIS

PD7067	PDE4DIP	cna	amp	BASIS
PD7215	PDE4DIP	cna	gain	BASIS
PD8621	PDE4DIP	cna	amp	BASIS
PD9004	PDE4DIP	cna	amp	BASIS
PD9702	PDE4DIP	cna	gain	BASIS
TCGA-AN-A0XU	PDPK1	cna	hetloss	TCGA
TCGA-AO-A0JL	PDPK1	cna	gain	TCGA
TCGA-BH-A0AW	PDPK1	cna	gain	TCGA
TCGA-BH-A0C0	PDPK1	cna	gain	TCGA
TCGA-C8-A12L	PDPK1	cna	gain	TCGA
TCGA-E2-A1L7	PDPK1	cna	hetloss	TCGA
TCGA-E9-A1NC	PDPK1	cna	hetloss	TCGA
TCGA-LL-A5YP	PDPK1	cna	gain	TCGA
MB-0346	PDPK1	cna	hetloss	METABRIC
MB-5465	PDPK1	cna	hetloss	METABRIC
MB-6271	PDPK1	cna	gain	METABRIC
MB-7032	PDPK1	cna	gain	METABRIC
MB-7038	PDPK1	cna	hetloss	METABRIC
PD10014	PDPK1	cna	gain	BASIS
PD11327	PDPK1	cna	gain	BASIS
PD11742	PDPK1	cna	gain	BASIS
PD13296	PDPK1	cna	gain	BASIS
PD13297	PDPK1	cna	hetloss	BASIS
PD13299	PDPK1	cna	gain	BASIS
PD22355	PDPK1	cna	hetloss	BASIS
PD23578	PDPK1	cna	hetloss	BASIS
PD24202	PDPK1	cna	hetloss	BASIS
PD24337	PDPK1	cna	hetloss	BASIS
PD3905	PDPK1	cna	gain	BASIS
PD4005	PDPK1	cna	gain	BASIS
PD4006	PDPK1	cna	gain	BASIS
PD4107	PDPK1	cna	gain	BASIS
PD4826	PDPK1	cna	gain	BASIS
PD4967	PDPK1	cna	gain	BASIS
PD5930	PDPK1	cna	gain	BASIS
PD5935	PDPK1	cna	gain	BASIS
PD5945	PDPK1	cna	gain	BASIS
PD5948	PDPK1	cna	gain	BASIS
PD6413	PDPK1	cna	gain	BASIS
PD7067	PDPK1	cna	gain	BASIS
PD7215	PDPK1	cna	amp	BASIS
PD8621	PDPK1	cna	gain	BASIS
PD9004	PDPK1	cna	gain	BASIS
PD9585	PDPK1	cna	hetloss	BASIS
PD9702	PDPK1	cna	gain	BASIS
TCGA-AO-A0JL	PDYN	cna	gain	TCGA
TCGA-BH-A0AW	PDYN	cna	gain	TCGA

TCGA-BH-A0C0	PDYN	cna	hetloss	TCGA
TCGA-BH-A18R	PDYN	cna	homdel	TCGA
TCGA-C8-A12L	PDYN	cna	hetloss	TCGA
TCGA-D8-A27M	PDYN	cna	gain	TCGA
TCGA-E2-A1L7	PDYN	cna	gain	TCGA
TCGA-LL-A5YP	PDYN	cna	hetloss	TCGA
MB-0346	PDYN	cna	gain	METABRIC
MB-5070	PDYN	cna	amp	METABRIC
MB-6060	PDYN	cna	amp	METABRIC
MB-6098	PDYN	cna	hetloss	METABRIC
MB-6271	PDYN	cna	hetloss	METABRIC
MB-7032	PDYN	cna	gain	METABRIC
MB-7048	PDYN	cna	gain	METABRIC
MB-0420	PDYN	cna	hetloss	METABRIC
MTS-T0064	PDYN	cna	gain	METABRIC
PD11327	PDYN	cna	amp	BASIS
PD11742	PDYN	cna	hetloss	BASIS
PD13296	PDYN	cna	hetloss	BASIS
PD13299	PDYN	cna	gain	BASIS
PD22355	PDYN	cna	hetloss	BASIS
PD23574	PDYN	cna	gain	BASIS
PD23578	PDYN	cna	hetloss	BASIS
PD24186	PDYN	cna	amp	BASIS
PD24202	PDYN	cna	hetloss	BASIS
PD24337	PDYN	cna	hetloss	BASIS
PD3890	PDYN	cna	hetloss	BASIS
PD3905	PDYN	cna	gain	BASIS
PD4005	PDYN	cna	hetloss	BASIS
PD4107	PDYN	cna	gain	BASIS
PD4826	PDYN	cna	hetloss	BASIS
PD5935	PDYN	cna	gain	BASIS
PD5945	PDYN	cna	amp	BASIS
PD5948	PDYN	cna	gain	BASIS
PD6406	PDYN	cna	hetloss	BASIS
PD7215	PDYN	cna	gain	BASIS
PD8621	PDYN	cna	gain	BASIS
PD8980	PDYN	cna	hetloss	BASIS
PD9702	PDYN	cna	gain	BASIS
TCGA-AN-A0XU	PIK3CB	cna	gain	TCGA
TCGA-BH-A0C0	PIK3CB	cna	gain	TCGA
TCGA-BH-A18R	PIK3CB	cna	gain	TCGA
TCGA-BH-A1FU	PIK3CB	cna	hetloss	TCGA
TCGA-C8-A12L	PIK3CB	cna	gain	TCGA
TCGA-E2-A1L7	PIK3CB	cna	gain	TCGA
TCGA-E9-A1NC	PIK3CB	cna	gain	TCGA
TCGA-LL-A5YP	PIK3CB	cna	gain	TCGA
MB-0346	PIK3CB	cna	hetloss	METABRIC

MB-5070	PIK3CB	cna	gain	METABRIC
MB-5107	PIK3CB	cna	gain	METABRIC
MB-5465	PIK3CB	cna	gain	METABRIC
MB-7038	PIK3CB	cna	gain	METABRIC
MB-7048	PIK3CB	cna	hetloss	METABRIC
MTS-T0064	PIK3CB	cna	amp	METABRIC
PD10014	PIK3CB	cna	gain	BASIS
PD11327	PIK3CB	cna	gain	BASIS
PD13299	PIK3CB	cna	gain	BASIS
PD13771	PIK3CB	cna	hetloss	BASIS
PD22355	PIK3CB	cna	gain	BASIS
PD23561	PIK3CB	cna	hetloss	BASIS
PD23574	PIK3CB	cna	gain	BASIS
PD23578	PIK3CB	cna	gain	BASIS
PD24186	PIK3CB	cna	gain	BASIS
PD24202	PIK3CB	cna	gain	BASIS
PD24206	PIK3CB	cna	gain	BASIS
PD3905	PIK3CB	cna	gain	BASIS
PD4005	PIK3CB	cna	hetloss	BASIS
PD4107	PIK3CB	cna	gain	BASIS
PD4826	PIK3CB	cna	gain	BASIS
PD5935	PIK3CB	cna	gain	BASIS
PD5945	PIK3CB	cna	gain	BASIS
PD5948	PIK3CB	cna	gain	BASIS
PD6731	PIK3CB	cna	gain	BASIS
PD7067	PIK3CB	cna	gain	BASIS
PD7215	PIK3CB	cna	gain	BASIS
PD8621	PIK3CB	cna	gain	BASIS
PD8980	PIK3CB	cna	gain	BASIS
PD9004	PIK3CB	cna	gain	BASIS
PD9702	PIK3CB	cna	gain	BASIS
TCGA-A2-A25B	POU2AF1	cna	hetloss	TCGA
TCGA-AN-A0XU	POU2AF1	cna	gain	TCGA
TCGA-AO-A0JL	POU2AF1	cna	hetloss	TCGA
TCGA-BH-A0C0	POU2AF1	cna	gain	TCGA
TCGA-BH-A18R	POU2AF1	cna	homdel	TCGA
TCGA-D8-A27M	POU2AF1	cna	hetloss	TCGA
TCGA-E2-A1L7	POU2AF1	cna	hetloss	TCGA
TCGA-E9-A1NC	POU2AF1	cna	hetloss	TCGA
TCGA-EW-A1OX	POU2AF1	cna	homdel	TCGA
TCGA-LL-A5YP	POU2AF1	cna	hetloss	TCGA
MB-0346	POU2AF1	cna	hetloss	METABRIC
MB-5070	POU2AF1	cna	hetloss	METABRIC
MB-5107	POU2AF1	cna	hetloss	METABRIC
MB-6271	POU2AF1	cna	hetloss	METABRIC
MB-7032	POU2AF1	cna	hetloss	METABRIC
MB-7048	POU2AF1	cna	hetloss	METABRIC

PD11327	POU2AF1	cna	gain	BASIS
PD11742	POU2AF1	cna	hetloss	BASIS
PD13296	POU2AF1	cna	hetloss	BASIS
PD13299	POU2AF1	cna	gain	BASIS
PD13771	POU2AF1	cna	hetloss	BASIS
PD22355	POU2AF1	cna	hetloss	BASIS
PD23574	POU2AF1	cna	gain	BASIS
PD23578	POU2AF1	cna	hetloss	BASIS
PD24186	POU2AF1	cna	gain	BASIS
PD24206	POU2AF1	cna	gain	BASIS
PD24337	POU2AF1	cna	hetloss	BASIS
PD3905	POU2AF1	cna	gain	BASIS
PD4005	POU2AF1	cna	hetloss	BASIS
PD4826	POU2AF1	cna	hetloss	BASIS
PD5935	POU2AF1	cna	gain	BASIS
PD5945	POU2AF1	cna	gain	BASIS
PD5948	POU2AF1	cna	gain	BASIS
PD6413	POU2AF1	cna	hetloss	BASIS
PD6731	POU2AF1	cna	hetloss	BASIS
PD7067	POU2AF1	cna	gain	BASIS
PD7215	POU2AF1	cna	gain	BASIS
PD9004	POU2AF1	cna	hetloss	BASIS
PD9585	POU2AF1	cna	gain	BASIS
PD9702	POU2AF1	cna	gain	BASIS
TCGA-A2-A25B	PTPN11	cna	gain	TCGA
TCGA-AN-A0XU	PTPN11	cna	hetloss	TCGA
TCGA-AO-A0JL	PTPN11	cna	hetloss	TCGA
TCGA-BH-A0C0	PTPN11	cna	gain	TCGA
TCGA-BH-A18R	PTPN11	cna	gain	TCGA
TCGA-BH-A1FU	PTPN11	cna	hetloss	TCGA
TCGA-C8-A12L	PTPN11	cna	gain	TCGA
TCGA-D8-A27M	PTPN11	cna	hetloss	TCGA
TCGA-E2-A1L7	PTPN11	cna	gain	TCGA
TCGA-LL-A5YP	PTPN11	cna	hetloss	TCGA
MB-0346	PTPN11	cna	gain	METABRIC
MB-2827	PTPN11	cna	hetloss	METABRIC
MB-5070	PTPN11	cna	hetloss	METABRIC
MB-5465	PTPN11	cna	hetloss	METABRIC
MB-6098	PTPN11	cna	hetloss	METABRIC
MB-7048	PTPN11	cna	hetloss	METABRIC
PD10014	PTPN11	cna	gain	BASIS
PD11327	PTPN11	cna	hetloss	BASIS
PD11742	PTPN11	cna	hetloss	BASIS
PD13296	PTPN11	cna	hetloss	BASIS
PD13297	PTPN11	cna	gain	BASIS
PD22355	PTPN11	cna	hetloss	BASIS
PD23574	PTPN11	cna	gain	BASIS

PD23578	PTPN11	cna	hetloss	BASIS
PD3890	PTPN11	cna	hetloss	BASIS
PD3905	PTPN11	cna	gain	BASIS
PD4005	PTPN11	cna	hetloss	BASIS
PD4006	PTPN11	cna	gain	BASIS
PD4107	PTPN11	cna	gain	BASIS
PD4826	PTPN11	cna	gain	BASIS
PD4967	PTPN11	cna	hetloss	BASIS
PD5945	PTPN11	cna	gain	BASIS
PD5948	PTPN11	cna	gain	BASIS
PD6406	PTPN11	cna	hetloss	BASIS
PD6413	PTPN11	cna	hetloss	BASIS
PD6731	PTPN11	cna	hetloss	BASIS
PD7067	PTPN11	cna	gain	BASIS
PD9004	PTPN11	cna	hetloss	BASIS
PD9585	PTPN11	cna	hetloss	BASIS
PD9702	PTPN11	cna	gain	BASIS
TCGA-A2-A25B	RHEB	cna	gain	TCGA
TCGA-AN-A0XU	RHEB	cna	hetloss	TCGA
TCGA-AO-A0JL	RHEB	cna	gain	TCGA
TCGA-C8-A12L	RHEB	cna	hetloss	TCGA
TCGA-D8-A27M	RHEB	cna	gain	TCGA
TCGA-E2-A1L7	RHEB	cna	hetloss	TCGA
TCGA-E9-A1NC	RHEB	cna	hetloss	TCGA
TCGA-LL-A5YP	RHEB	cna	hetloss	TCGA
MB-0346	RHEB	cna	hetloss	METABRIC
MB-5070	RHEB	cna	hetloss	METABRIC
MB-6098	RHEB	cna	gain	METABRIC
MB-7038	RHEB	cna	gain	METABRIC
MB-7048	RHEB	cna	gain	METABRIC
MB-0420	RHEB	cna	hetloss	METABRIC
MTS-T0064	RHEB	cna	amp	METABRIC
P-0009557-T01-IM5	RHEB	cna	homdel	MSK-IMPACT
PD10014	RHEB	cna	gain	BASIS
PD13296	RHEB	cna	hetloss	BASIS
PD13297	RHEB	cna	gain	BASIS
PD22355	RHEB	cna	hetloss	BASIS
PD23562	RHEB	cna	gain	BASIS
PD23574	RHEB	cna	gain	BASIS
PD23578	RHEB	cna	hetloss	BASIS
PD24186	RHEB	cna	gain	BASIS
PD24202	RHEB	cna	gain	BASIS
PD24337	RHEB	cna	gain	BASIS
PD3890	RHEB	cna	hetloss	BASIS
PD3905	RHEB	cna	gain	BASIS
PD4006	RHEB	cna	amp	BASIS
PD4107	RHEB	cna	gain	BASIS

PD5930	RHEB	cna	gain	BASIS
PD5945	RHEB	cna	amp	BASIS
PD5948	RHEB	cna	gain	BASIS
PD6406	RHEB	cna	gain	BASIS
PD6731	RHEB	cna	hetloss	BASIS
PD7067	RHEB	cna	gain	BASIS
PD7215	RHEB	cna	gain	BASIS
PD9004	RHEB	cna	gain	BASIS
PD9585	RHEB	cna	hetloss	BASIS
PD9702	RHEB	cna	gain	BASIS
TCGA-A2-A25B	RSPO3	cna	hetloss	TCGA
TCGA-AN-A0XU	RSPO3	cna	amp	TCGA
TCGA-AO-A0JL	RSPO3	cna	hetloss	TCGA
TCGA-BH-A0AW	RSPO3	cna	amp	TCGA
TCGA-BH-A18R	RSPO3	cna	gain	TCGA
TCGA-C8-A12L	RSPO3	cna	gain	TCGA
TCGA-D8-A27M	RSPO3	cna	gain	TCGA
MB-2827	RSPO3	cna	amp	METABRIC
MB-5070	RSPO3	cna	gain	METABRIC
MB-7032	RSPO3	cna	hetloss	METABRIC
MB-7038	RSPO3	cna	gain	METABRIC
MB-7048	RSPO3	cna	hetloss	METABRIC
MB-0420	RSPO3	cna	hetloss	METABRIC
MTS-T0064	RSPO3	cna	amp	METABRIC
PD11327	RSPO3	cna	gain	BASIS
PD13296	RSPO3	cna	gain	BASIS
PD13299	RSPO3	cna	gain	BASIS
PD13771	RSPO3	cna	hetloss	BASIS
PD22355	RSPO3	cna	gain	BASIS
PD23562	RSPO3	cna	gain	BASIS
PD23574	RSPO3	cna	gain	BASIS
PD23578	RSPO3	cna	hetloss	BASIS
PD24186	RSPO3	cna	gain	BASIS
PD24206	RSPO3	cna	gain	BASIS
PD24337	RSPO3	cna	hetloss	BASIS
PD3905	RSPO3	cna	gain	BASIS
PD4006	RSPO3	cna	gain	BASIS
PD4107	RSPO3	cna	gain	BASIS
PD4826	RSPO3	cna	gain	BASIS
PD5935	RSPO3	cna	gain	BASIS
PD5945	RSPO3	cna	amp	BASIS
PD6406	RSPO3	cna	hetloss	BASIS
PD6731	RSPO3	cna	hetloss	BASIS
PD7067	RSPO3	cna	amp	BASIS
PD7215	RSPO3	cna	gain	BASIS
PD8621	RSPO3	cna	gain	BASIS
PD8980	RSPO3	cna	gain	BASIS

PD9004	RSPO3	cna	gain	BASIS
PD9585	RSPO3	cna	hetloss	BASIS
PD9702	RSPO3	cna	gain	BASIS
TCGA-A2-A25B	SFPQ	cna	hetloss	TCGA
TCGA-AN-A0XU	SFPQ	cna	hetloss	TCGA
TCGA-AO-A0JL	SFPQ	cna	hetloss	TCGA
TCGA-BH-A0AW	SFPQ	cna	gain	TCGA
TCGA-BH-A0C0	SFPQ	cna	gain	TCGA
TCGA-BH-A18R	SFPQ	cna	homdel	TCGA
TCGA-BH-A1FU	SFPQ	cna	gain	TCGA
TCGA-C8-A12L	SFPQ	cna	hetloss	TCGA
TCGA-D8-A27M	SFPQ	cna	gain	TCGA
TCGA-E2-A1L7	SFPQ	cna	gain	TCGA
TCGA-LL-A5YP	SFPQ	cna	gain	TCGA
MB-0346	SFPQ	cna	hetloss	METABRIC
MB-2827	SFPQ	cna	hetloss	METABRIC
MB-5070	SFPQ	cna	hetloss	METABRIC
MB-5465	SFPQ	cna	hetloss	METABRIC
MB-6060	SFPQ	cna	hetloss	METABRIC
MB-6098	SFPQ	cna	hetloss	METABRIC
MB-7038	SFPQ	cna	gain	METABRIC
MB-0420	SFPQ	cna	hetloss	METABRIC
PD10014	SFPQ	cna	gain	BASIS
PD11742	SFPQ	cna	hetloss	BASIS
PD13296	SFPQ	cna	gain	BASIS
PD13297	SFPQ	cna	gain	BASIS
PD13771	SFPQ	cna	gain	BASIS
PD23562	SFPQ	cna	gain	BASIS
PD24186	SFPQ	cna	gain	BASIS
PD24206	SFPQ	cna	hetloss	BASIS
PD24337	SFPQ	cna	hetloss	BASIS
PD3890	SFPQ	cna	hetloss	BASIS
PD3905	SFPQ	cna	gain	BASIS
PD4006	SFPQ	cna	gain	BASIS
PD4826	SFPQ	cna	gain	BASIS
PD5935	SFPQ	cna	gain	BASIS
PD5945	SFPQ	cna	amp	BASIS
PD7067	SFPQ	cna	gain	BASIS
PD7215	SFPQ	cna	gain	BASIS
PD8980	SFPQ	cna	gain	BASIS
PD9004	SFPQ	cna	hetloss	BASIS
PD9585	SFPQ	cna	hetloss	BASIS
PD9702	SFPQ	cna	gain	BASIS
TCGA-A2-A25B	SLC34A2	cna	hetloss	TCGA
TCGA-AN-A0XU	SLC34A2	cna	hetloss	TCGA
TCGA-AO-A0JL	SLC34A2	cna	homdel	TCGA
TCGA-BH-A0AW	SLC34A2	cna	hetloss	TCGA

TCGA-BH-A0C0	SLC34A2	cna	hetloss	TCGA
TCGA-BH-A1FU	SLC34A2	cna	homdel	TCGA
TCGA-C8-A12L	SLC34A2	cna	hetloss	TCGA
TCGA-D8-A27M	SLC34A2	cna	gain	TCGA
TCGA-E2-A1L7	SLC34A2	cna	hetloss	TCGA
TCGA-E9-A1NC	SLC34A2	cna	hetloss	TCGA
TCGA-LL-A5YP	SLC34A2	cna	hetloss	TCGA
MB-0346	SLC34A2	cna	hetloss	METABRIC
MB-2827	SLC34A2	cna	hetloss	METABRIC
MB-5070	SLC34A2	cna	hetloss	METABRIC
MB-5107	SLC34A2	cna	hetloss	METABRIC
MB-5465	SLC34A2	cna	hetloss	METABRIC
MB-6060	SLC34A2	cna	hetloss	METABRIC
MB-6098	SLC34A2	cna	hetloss	METABRIC
MB-0420	SLC34A2	cna	hetloss	METABRIC
PD10014	SLC34A2	cna	hetloss	BASIS
PD11327	SLC34A2	cna	homdel	BASIS
PD13297	SLC34A2	cna	hetloss	BASIS
PD22355	SLC34A2	cna	hetloss	BASIS
PD24186	SLC34A2	cna	gain	BASIS
PD24202	SLC34A2	cna	hetloss	BASIS
PD24206	SLC34A2	cna	hetloss	BASIS
PD3890	SLC34A2	cna	hetloss	BASIS
PD3905	SLC34A2	cna	gain	BASIS
PD4005	SLC34A2	cna	hetloss	BASIS
PD4006	SLC34A2	cna	hetloss	BASIS
PD4107	SLC34A2	cna	hetloss	BASIS
PD4826	SLC34A2	cna	hetloss	BASIS
PD5930	SLC34A2	cna	hetloss	BASIS
PD5945	SLC34A2	cna	gain	BASIS
PD6406	SLC34A2	cna	hetloss	BASIS
PD6413	SLC34A2	cna	hetloss	BASIS
PD7067	SLC34A2	cna	gain	BASIS
PD8621	SLC34A2	cna	gain	BASIS
PD8980	SLC34A2	cna	homdel	BASIS
PD9585	SLC34A2	cna	hetloss	BASIS
TCGA-A2-A25B	SOX9	cna	gain	TCGA
TCGA-AN-A0XU	SOX9	cna	gain	TCGA
TCGA-AO-A0JL	SOX9	cna	hetloss	TCGA
TCGA-BH-A0AW	SOX9	cna	amp	TCGA
TCGA-BH-A0C0	SOX9	cna	gain	TCGA
TCGA-BH-A18R	SOX9	cna	amp	TCGA
TCGA-C8-A12L	SOX9	cna	gain	TCGA
TCGA-D8-A27M	SOX9	cna	gain	TCGA
TCGA-E2-A1L7	SOX9	cna	gain	TCGA
TCGA-LL-A5YP	SOX9	cna	amp	TCGA
MB-0346	SOX9	cna	gain	METABRIC

MB-5070	SOX9	cna	gain	METABRIC
MB-5107	SOX9	cna	amp	METABRIC
MB-6098	SOX9	cna	gain	METABRIC
MB-6271	SOX9	cna	hetloss	METABRIC
MTS-T0064	SOX9	cna	gain	METABRIC
PD11327	SOX9	cna	amp	BASIS
PD11742	SOX9	cna	gain	BASIS
PD23561	SOX9	cna	gain	BASIS
PD23562	SOX9	cna	gain	BASIS
PD23574	SOX9	cna	gain	BASIS
PD24186	SOX9	cna	gain	BASIS
PD24202	SOX9	cna	gain	BASIS
PD24206	SOX9	cna	hetloss	BASIS
PD3890	SOX9	cna	gain	BASIS
PD3905	SOX9	cna	gain	BASIS
PD4006	SOX9	cna	gain	BASIS
PD4107	SOX9	cna	gain	BASIS
PD4826	SOX9	cna	hetloss	BASIS
PD4967	SOX9	cna	hetloss	BASIS
PD5930	SOX9	cna	gain	BASIS
PD5935	SOX9	cna	gain	BASIS
PD5945	SOX9	cna	amp	BASIS
PD5948	SOX9	cna	gain	BASIS
PD6731	SOX9	cna	hetloss	BASIS
PD7067	SOX9	cna	gain	BASIS
PD7215	SOX9	cna	gain	BASIS
PD9004	SOX9	cna	gain	BASIS
PD9585	SOX9	cna	gain	BASIS
PD9702	SOX9	cna	gain	BASIS
TCGA-A2-A25B	SUFU	cna	hetloss	TCGA
TCGA-AN-A0XU	SUFU	cna	hetloss	TCGA
TCGA-AO-A0JL	SUFU	cna	hetloss	TCGA
TCGA-BH-A18R	SUFU	cna	gain	TCGA
TCGA-C8-A12L	SUFU	cna	hetloss	TCGA
TCGA-D8-A27M	SUFU	cna	hetloss	TCGA
TCGA-E2-A1L7	SUFU	cna	hetloss	TCGA
TCGA-LL-A5YP	SUFU	cna	gain	TCGA
MB-0346	SUFU	cna	gain	METABRIC
MB-2827	SUFU	cna	hetloss	METABRIC
MB-5070	SUFU	cna	hetloss	METABRIC
MB-6060	SUFU	cna	hetloss	METABRIC
MB-6098	SUFU	cna	hetloss	METABRIC
MB-7038	SUFU	cna	hetloss	METABRIC
MB-7048	SUFU	cna	hetloss	METABRIC
MB-0420	SUFU	cna	hetloss	METABRIC
PD10014	SUFU	cna	hetloss	BASIS
PD11327	SUFU	cna	homdel	BASIS

PD11742	SUFU	cna	hetloss	BASIS
PD22355	SUFU	cna	hetloss	BASIS
PD23562	SUFU	cna	gain	BASIS
PD23574	SUFU	cna	gain	BASIS
PD23578	SUFU	cna	hetloss	BASIS
PD24206	SUFU	cna	homdel	BASIS
PD3890	SUFU	cna	hetloss	BASIS
PD3905	SUFU	cna	gain	BASIS
PD4005	SUFU	cna	hetloss	BASIS
PD4006	SUFU	cna	gain	BASIS
PD4107	SUFU	cna	gain	BASIS
PD5935	SUFU	cna	hetloss	BASIS
PD5945	SUFU	cna	gain	BASIS
PD5948	SUFU	cna	gain	BASIS
PD6406	SUFU	cna	hetloss	BASIS
PD6731	SUFU	cna	hetloss	BASIS
PD7067	SUFU	cna	gain	BASIS
PD7215	SUFU	cna	gain	BASIS
PD8621	SUFU	cna	gain	BASIS
PD8980	SUFU	cna	hetloss	BASIS
PD9585	SUFU	cna	hetloss	BASIS
PD9702	SUFU	cna	gain	BASIS
TCGA-A2-A25B	TBL1XR1	cna	gain	TCGA
TCGA-AN-A0XU	TBL1XR1	cna	gain	TCGA
TCGA-BH-A0AW	TBL1XR1	cna	gain	TCGA
TCGA-BH-A0C0	TBL1XR1	cna	gain	TCGA
TCGA-BH-A1FU	TBL1XR1	cna	hetloss	TCGA
TCGA-C8-A12L	TBL1XR1	cna	amp	TCGA
TCGA-E2-A1L7	TBL1XR1	cna	gain	TCGA
TCGA-LL-A5YP	TBL1XR1	cna	gain	TCGA
MB-0346	TBL1XR1	cna	gain	METABRIC
MB-5070	TBL1XR1	cna	amp	METABRIC
MB-5107	TBL1XR1	cna	amp	METABRIC
MB-6060	TBL1XR1	cna	gain	METABRIC
MB-7038	TBL1XR1	cna	hetloss	METABRIC
MB-0420	TBL1XR1	cna	amp	METABRIC
MTS-T0064	TBL1XR1	cna	amp	METABRIC
PD11327	TBL1XR1	cna	amp	BASIS
PD13296	TBL1XR1	cna	amp	BASIS
PD13299	TBL1XR1	cna	gain	BASIS
PD13771	TBL1XR1	cna	hetloss	BASIS
PD22355	TBL1XR1	cna	gain	BASIS
PD23561	TBL1XR1	cna	hetloss	BASIS
PD23562	TBL1XR1	cna	amp	BASIS
PD23574	TBL1XR1	cna	gain	BASIS
PD23578	TBL1XR1	cna	gain	BASIS
PD24186	TBL1XR1	cna	amp	BASIS

PD24202	TBL1XR1	cna	gain	BASIS
PD24206	TBL1XR1	cna	gain	BASIS
PD3905	TBL1XR1	cna	gain	BASIS
PD4005	TBL1XR1	cna	amp	BASIS
PD4107	TBL1XR1	cna	gain	BASIS
PD4826	TBL1XR1	cna	gain	BASIS
PD5935	TBL1XR1	cna	amp	BASIS
PD5945	TBL1XR1	cna	amp	BASIS
PD5948	TBL1XR1	cna	gain	BASIS
PD6406	TBL1XR1	cna	gain	BASIS
PD7067	TBL1XR1	cna	gain	BASIS
PD7215	TBL1XR1	cna	gain	BASIS
PD8621	TBL1XR1	cna	gain	BASIS
PD8980	TBL1XR1	cna	gain	BASIS
PD9702	TBL1XR1	cna	amp	BASIS
TCGA-A2-A25B	TET2	cna	hetloss	TCGA
TCGA-AN-A0XU	TET2	cna	hetloss	TCGA
TCGA-BH-A0AW	TET2	cna	hetloss	TCGA
TCGA-BH-A0C0	TET2	cna	hetloss	TCGA
TCGA-BH-A1FU	TET2	cna	gain	TCGA
TCGA-C8-A12L	TET2	cna	hetloss	TCGA
TCGA-D8-A27M	TET2	cna	hetloss	TCGA
TCGA-E2-A1L7	TET2	cna	hetloss	TCGA
TCGA-E9-A1NC	TET2	cna	hetloss	TCGA
TCGA-LL-A5YP	TET2	cna	gain	TCGA
MB-2827	TET2	cna	hetloss	METABRIC
MB-5070	TET2	cna	hetloss	METABRIC
MB-5465	TET2	cna	hetloss	METABRIC
MB-7048	TET2	cna	hetloss	METABRIC
MB-0420	TET2	cna	hetloss	METABRIC
PD10014	TET2	cna	gain	BASIS
PD11742	TET2	cna	hetloss	BASIS
PD13296	TET2	cna	hetloss	BASIS
PD13297	TET2	cna	hetloss	BASIS
PD22355	TET2	cna	hetloss	BASIS
PD23562	TET2	cna	gain	BASIS
PD23578	TET2	cna	hetloss	BASIS
PD24202	TET2	cna	hetloss	BASIS
PD24206	TET2	cna	gain	BASIS
PD24337	TET2	cna	hetloss	BASIS
PD3890	TET2	cna	gain	BASIS
PD3905	TET2	cna	gain	BASIS
PD4006	TET2	cna	hetloss	BASIS
PD4107	TET2	cna	gain	BASIS
PD4826	TET2	cna	gain	BASIS
PD5930	TET2	cna	hetloss	BASIS
PD5935	TET2	cna	gain	BASIS

PD5945	TET2	cna	gain	BASIS
PD5948	TET2	cna	gain	BASIS
PD6406	TET2	cna	hetloss	BASIS
PD7067	TET2	cna	gain	BASIS
PD7215	TET2	cna	gain	BASIS
PD8621	TET2	cna	hetloss	BASIS
PD8980	TET2	cna	hetloss	BASIS
PD9585	TET2	cna	hetloss	BASIS
TCGA-A2-A25B	TNFRSF14	cna	hetloss	TCGA
TCGA-AN-A0XU	TNFRSF14	cna	hetloss	TCGA
TCGA-AO-A0JL	TNFRSF14	cna	hetloss	TCGA
TCGA-BH-A0AW	TNFRSF14	cna	gain	TCGA
TCGA-BH-A0C0	TNFRSF14	cna	gain	TCGA
TCGA-BH-A18R	TNFRSF14	cna	homdel	TCGA
TCGA-BH-A1FU	TNFRSF14	cna	gain	TCGA
TCGA-C8-A12L	TNFRSF14	cna	hetloss	TCGA
TCGA-D8-A27M	TNFRSF14	cna	gain	TCGA
TCGA-E2-A1L7	TNFRSF14	cna	hetloss	TCGA
TCGA-E9-A1NC	TNFRSF14	cna	hetloss	TCGA
TCGA-LL-A5YP	TNFRSF14	cna	amp	TCGA
MB-2827	TNFRSF14	cna	hetloss	METABRIC
MB-5070	TNFRSF14	cna	hetloss	METABRIC
MB-5465	TNFRSF14	cna	hetloss	METABRIC
MB-6098	TNFRSF14	cna	hetloss	METABRIC
MB-0420	TNFRSF14	cna	hetloss	METABRIC
PD10014	TNFRSF14	cna	gain	BASIS
PD11327	TNFRSF14	cna	gain	BASIS
PD11742	TNFRSF14	cna	hetloss	BASIS
PD13297	TNFRSF14	cna	gain	BASIS
PD22355	TNFRSF14	cna	hetloss	BASIS
PD23578	TNFRSF14	cna	hetloss	BASIS
PD24186	TNFRSF14	cna	gain	BASIS
PD24202	TNFRSF14	cna	hetloss	BASIS
PD24206	TNFRSF14	cna	gain	BASIS
PD24337	TNFRSF14	cna	hetloss	BASIS
PD3890	TNFRSF14	cna	hetloss	BASIS
PD3905	TNFRSF14	cna	gain	BASIS
PD4005	TNFRSF14	cna	gain	BASIS
PD4107	TNFRSF14	cna	gain	BASIS
PD4826	TNFRSF14	cna	gain	BASIS
PD5935	TNFRSF14	cna	gain	BASIS
PD5945	TNFRSF14	cna	gain	BASIS
PD6406	TNFRSF14	cna	hetloss	BASIS
PD7067	TNFRSF14	cna	gain	BASIS
PD7215	TNFRSF14	cna	gain	BASIS
PD8621	TNFRSF14	cna	gain	BASIS
PD8980	TNFRSF14	cna	hetloss	BASIS

PD9004	TNFRSF14	cna	hetloss	BASIS
TCGA-A2-A25B	TP63	cna	amp	TCGA
TCGA-AN-A0XU	TP63	cna	gain	TCGA
TCGA-BH-A0AW	TP63	cna	gain	TCGA
TCGA-BH-A0C0	TP63	cna	gain	TCGA
TCGA-BH-A1FU	TP63	cna	hetloss	TCGA
TCGA-C8-A12L	TP63	cna	gain	TCGA
TCGA-LL-A5YP	TP63	cna	gain	TCGA
MB-0346	TP63	cna	gain	METABRIC
MB-5070	TP63	cna	hetloss	METABRIC
MB-5107	TP63	cna	amp	METABRIC
MB-5465	TP63	cna	gain	METABRIC
MB-6060	TP63	cna	gain	METABRIC
MB-6098	TP63	cna	gain	METABRIC
MB-7038	TP63	cna	hetloss	METABRIC
MTS-T0064	TP63	cna	amp	METABRIC
P-0002858-T01-IM3	TP63	cna	amp	MSK-IMPACT
PD11327	TP63	cna	gain	BASIS
PD13299	TP63	cna	gain	BASIS
PD13771	TP63	cna	hetloss	BASIS
PD22355	TP63	cna	gain	BASIS
PD23561	TP63	cna	hetloss	BASIS
PD23562	TP63	cna	gain	BASIS
PD23578	TP63	cna	gain	BASIS
PD24186	TP63	cna	amp	BASIS
PD24202	TP63	cna	gain	BASIS
PD3905	TP63	cna	gain	BASIS
PD4005	TP63	cna	gain	BASIS
PD4107	TP63	cna	gain	BASIS
PD4826	TP63	cna	gain	BASIS
PD5930	TP63	cna	gain	BASIS
PD5945	TP63	cna	amp	BASIS
PD5948	TP63	cna	gain	BASIS
PD6406	TP63	cna	gain	BASIS
PD6731	TP63	cna	gain	BASIS
PD7067	TP63	cna	gain	BASIS
PD7215	TP63	cna	gain	BASIS
PD8621	TP63	cna	gain	BASIS
PD8980	TP63	cna	gain	BASIS
PD9004	TP63	cna	gain	BASIS
PD9702	TP63	cna	amp	BASIS
TCGA-A2-A25B	TRIP11	cna	hetloss	TCGA
TCGA-AO-A0JL	TRIP11	cna	hetloss	TCGA
TCGA-BH-A0AW	TRIP11	cna	hetloss	TCGA
TCGA-BH-A1FU	TRIP11	cna	gain	TCGA
TCGA-C8-A12L	TRIP11	cna	gain	TCGA
TCGA-D8-A27M	TRIP11	cna	hetloss	TCGA

TCGA-E2-A1L7	TRIP11	cna	gain	TCGA
TCGA-E9-A1NC	TRIP11	cna	hetloss	TCGA
TCGA-LL-A5YP	TRIP11	cna	hetloss	TCGA
MB-0346	TRIP11	cna	gain	METABRIC
MB-2827	TRIP11	cna	hetloss	METABRIC
MB-5070	TRIP11	cna	hetloss	METABRIC
MB-5107	TRIP11	cna	hetloss	METABRIC
MB-6098	TRIP11	cna	hetloss	METABRIC
MTS-T0064	TRIP11	cna	hetloss	METABRIC
PD10014	TRIP11	cna	hetloss	BASIS
PD11327	TRIP11	cna	hetloss	BASIS
PD11742	TRIP11	cna	hetloss	BASIS
PD13296	TRIP11	cna	gain	BASIS
PD13297	TRIP11	cna	hetloss	BASIS
PD13771	TRIP11	cna	hetloss	BASIS
PD23562	TRIP11	cna	gain	BASIS
PD23574	TRIP11	cna	gain	BASIS
PD23578	TRIP11	cna	hetloss	BASIS
PD24202	TRIP11	cna	hetloss	BASIS
PD24206	TRIP11	cna	gain	BASIS
PD24337	TRIP11	cna	hetloss	BASIS
PD3890	TRIP11	cna	hetloss	BASIS
PD3905	TRIP11	cna	gain	BASIS
PD4005	TRIP11	cna	hetloss	BASIS
PD4006	TRIP11	cna	gain	BASIS
PD4826	TRIP11	cna	gain	BASIS
PD4967	TRIP11	cna	hetloss	BASIS
PD5945	TRIP11	cna	gain	BASIS
PD5948	TRIP11	cna	gain	BASIS
PD6406	TRIP11	cna	hetloss	BASIS
PD6413	TRIP11	cna	hetloss	BASIS
PD7067	TRIP11	cna	gain	BASIS
PD8980	TRIP11	cna	hetloss	BASIS
PD9702	TRIP11	cna	gain	BASIS
TCGA-A2-A25B	USP9X	cna	gain	TCGA
TCGA-AN-A0XU	USP9X	cna	hetloss	TCGA
TCGA-AO-A0JL	USP9X	cna	gain	TCGA
TCGA-BH-A0AW	USP9X	cna	gain	TCGA
TCGA-E2-A1L7	USP9X	cna	hetloss	TCGA
TCGA-E9-A1NC	USP9X	cna	hetloss	TCGA
MB-0346	USP9X	cna	hetloss	METABRIC
MB-2827	USP9X	cna	hetloss	METABRIC
MB-5070	USP9X	cna	hetloss	METABRIC
MB-5465	USP9X	cna	gain	METABRIC
MB-6098	USP9X	cna	hetloss	METABRIC
MB-0420	USP9X	cna	hetloss	METABRIC
MTS-T0064	USP9X	cna	hetloss	METABRIC

PD10014	USP9X	cna	hetloss	BASIS
PD11327	USP9X	cna	homdel	BASIS
PD11742	USP9X	cna	hetloss	BASIS
PD13296	USP9X	cna	gain	BASIS
PD13299	USP9X	cna	amp	BASIS
PD13771	USP9X	cna	gain	BASIS
PD14442	USP9X	cna	gain	BASIS
PD23562	USP9X	cna	hetloss	BASIS
PD23574	USP9X	cna	gain	BASIS
PD23578	USP9X	cna	hetloss	BASIS
PD24206	USP9X	cna	homdel	BASIS
PD3905	USP9X	cna	gain	BASIS
PD4005	USP9X	cna	hetloss	BASIS
PD4006	USP9X	cna	hetloss	BASIS
PD4107	USP9X	cna	gain	BASIS
PD4826	USP9X	cna	gain	BASIS
PD4967	USP9X	cna	gain	BASIS
PD5935	USP9X	cna	gain	BASIS
PD5948	USP9X	cna	gain	BASIS
PD6406	USP9X	cna	gain	BASIS
PD6413	USP9X	cna	gain	BASIS
PD7215	USP9X	cna	gain	BASIS
PD8621	USP9X	cna	amp	BASIS
PD8980	USP9X	cna	hetloss	BASIS
PD9004	USP9X	cna	gain	BASIS
PD9585	USP9X	cna	hetloss	BASIS
PD9702	USP9X	cna	gain	BASIS
TCGA-A2-A25B	ZMYM3	cna	gain	TCGA
TCGA-AN-A0XU	ZMYM3	cna	hetloss	TCGA
TCGA-AO-A0JL	ZMYM3	cna	gain	TCGA
TCGA-BH-A0AW	ZMYM3	cna	gain	TCGA
TCGA-C8-A12L	ZMYM3	cna	hetloss	TCGA
TCGA-E2-A1L7	ZMYM3	cna	hetloss	TCGA
TCGA-E9-A1NC	ZMYM3	cna	hetloss	TCGA
MB-0346	ZMYM3	cna	hetloss	METABRIC
MB-2827	ZMYM3	cna	hetloss	METABRIC
MB-5465	ZMYM3	cna	gain	METABRIC
MB-6060	ZMYM3	cna	hetloss	METABRIC
MB-0420	ZMYM3	cna	hetloss	METABRIC
PD10014	ZMYM3	cna	hetloss	BASIS
PD11327	ZMYM3	cna	hetloss	BASIS
PD13296	ZMYM3	cna	amp	BASIS
PD13297	ZMYM3	cna	gain	BASIS
PD13299	ZMYM3	cna	gain	BASIS
PD13771	ZMYM3	cna	gain	BASIS
PD14442	ZMYM3	cna	gain	BASIS
PD23562	ZMYM3	cna	hetloss	BASIS

PD23574	ZMYM3	cna	gain	BASIS
PD24202	ZMYM3	cna	gain	BASIS
PD24206	ZMYM3	cna	gain	BASIS
PD3890	ZMYM3	cna	gain	BASIS
PD3905	ZMYM3	cna	gain	BASIS
PD4005	ZMYM3	cna	hetloss	BASIS
PD4006	ZMYM3	cna	gain	BASIS
PD4107	ZMYM3	cna	amp	BASIS
PD4826	ZMYM3	cna	gain	BASIS
PD4967	ZMYM3	cna	gain	BASIS
PD5930	ZMYM3	cna	gain	BASIS
PD5935	ZMYM3	cna	gain	BASIS
PD5948	ZMYM3	cna	gain	BASIS
PD6406	ZMYM3	cna	gain	BASIS
PD6413	ZMYM3	cna	gain	BASIS
PD7067	ZMYM3	cna	gain	BASIS
PD7215	ZMYM3	cna	gain	BASIS
PD8621	ZMYM3	cna	amp	BASIS
PD8980	ZMYM3	cna	gain	BASIS
PD9004	ZMYM3	cna	gain	BASIS
TCGA-AN-A0XU	AFF4	cna	hetloss	TCGA
TCGA-AO-A0JL	AFF4	cna	hetloss	TCGA
TCGA-BH-A0C0	AFF4	cna	hetloss	TCGA
TCGA-BH-A18R	AFF4	cna	gain	TCGA
TCGA-BH-A1FU	AFF4	cna	hetloss	TCGA
TCGA-C8-A12L	AFF4	cna	hetloss	TCGA
TCGA-D8-A27M	AFF4	cna	hetloss	TCGA
TCGA-E2-A1L7	AFF4	cna	gain	TCGA
TCGA-E9-A1NC	AFF4	cna	hetloss	TCGA
TCGA-LL-A5YP	AFF4	cna	hetloss	TCGA
MB-0346	AFF4	cna	gain	METABRIC
MB-2827	AFF4	cna	hetloss	METABRIC
MB-5070	AFF4	cna	hetloss	METABRIC
MB-5107	AFF4	cna	hetloss	METABRIC
MB-5465	AFF4	cna	hetloss	METABRIC
MB-6060	AFF4	cna	hetloss	METABRIC
MB-6098	AFF4	cna	hetloss	METABRIC
MB-7038	AFF4	cna	hetloss	METABRIC
MB-0420	AFF4	cna	hetloss	METABRIC
PD10014	AFF4	cna	hetloss	BASIS
PD13296	AFF4	cna	hetloss	BASIS
PD13297	AFF4	cna	hetloss	BASIS
PD13771	AFF4	cna	gain	BASIS
PD22355	AFF4	cna	hetloss	BASIS
PD23562	AFF4	cna	hetloss	BASIS
PD23578	AFF4	cna	hetloss	BASIS
PD24202	AFF4	cna	hetloss	BASIS

PD24206	AFF4	cna	gain	BASIS
PD24337	AFF4	cna	hetloss	BASIS
PD3890	AFF4	cna	hetloss	BASIS
PD3905	AFF4	cna	gain	BASIS
PD4005	AFF4	cna	hetloss	BASIS
PD5935	AFF4	cna	gain	BASIS
PD5945	AFF4	cna	gain	BASIS
PD6406	AFF4	cna	hetloss	BASIS
PD6413	AFF4	cna	hetloss	BASIS
PD7067	AFF4	cna	gain	BASIS
PD8980	AFF4	cna	hetloss	BASIS
PD9585	AFF4	cna	hetloss	BASIS
TCGA-AN-A0XU	ARHGAP26	cna	hetloss	TCGA
TCGA-AO-A0JL	ARHGAP26	cna	hetloss	TCGA
TCGA-BH-A0C0	ARHGAP26	cna	hetloss	TCGA
TCGA-BH-A1FU	ARHGAP26	cna	hetloss	TCGA
TCGA-C8-A12L	ARHGAP26	cna	hetloss	TCGA
TCGA-D8-A27M	ARHGAP26	cna	hetloss	TCGA
TCGA-E2-A1L7	ARHGAP26	cna	hetloss	TCGA
TCGA-E9-A1NC	ARHGAP26	cna	hetloss	TCGA
TCGA-LL-A5YP	ARHGAP26	cna	hetloss	TCGA
MB-0346	ARHGAP26	cna	gain	METABRIC
MB-2827	ARHGAP26	cna	hetloss	METABRIC
MB-5070	ARHGAP26	cna	hetloss	METABRIC
MB-5465	ARHGAP26	cna	hetloss	METABRIC
MB-6098	ARHGAP26	cna	hetloss	METABRIC
MB-7038	ARHGAP26	cna	hetloss	METABRIC
MB-0420	ARHGAP26	cna	hetloss	METABRIC
PD10014	ARHGAP26	cna	hetloss	BASIS
PD13296	ARHGAP26	cna	hetloss	BASIS
PD13297	ARHGAP26	cna	hetloss	BASIS
PD13771	ARHGAP26	cna	gain	BASIS
PD22355	ARHGAP26	cna	hetloss	BASIS
PD23562	ARHGAP26	cna	gain	BASIS
PD23578	ARHGAP26	cna	hetloss	BASIS
PD24186	ARHGAP26	cna	gain	BASIS
PD24202	ARHGAP26	cna	hetloss	BASIS
PD24206	ARHGAP26	cna	gain	BASIS
PD24337	ARHGAP26	cna	hetloss	BASIS
PD3890	ARHGAP26	cna	hetloss	BASIS
PD3905	ARHGAP26	cna	gain	BASIS
PD4005	ARHGAP26	cna	hetloss	BASIS
PD4826	ARHGAP26	cna	gain	BASIS
PD5935	ARHGAP26	cna	gain	BASIS
PD5945	ARHGAP26	cna	gain	BASIS
PD6406	ARHGAP26	cna	hetloss	BASIS
PD6413	ARHGAP26	cna	hetloss	BASIS

PD7067	ARHGAP26	cna	gain	BASIS
PD8980	ARHGAP26	cna	hetloss	BASIS
PD9004	ARHGAP26	cna	gain	BASIS
PD9585	ARHGAP26	cna	hetloss	BASIS
TCGA-A2-A25B	ARHGEF12	cna	hetloss	TCGA
TCGA-AO-A0JL	ARHGEF12	cna	hetloss	TCGA
TCGA-BH-A0C0	ARHGEF12	cna	hetloss	TCGA
TCGA-BH-A18R	ARHGEF12	cna	homdel	TCGA
TCGA-D8-A27M	ARHGEF12	cna	hetloss	TCGA
TCGA-E2-A1L7	ARHGEF12	cna	hetloss	TCGA
TCGA-E9-A1NC	ARHGEF12	cna	hetloss	TCGA
TCGA-EW-A1OX	ARHGEF12	cna	homdel	TCGA
TCGA-LL-A5YP	ARHGEF12	cna	hetloss	TCGA
MB-0346	ARHGEF12	cna	hetloss	METABRIC
MB-5070	ARHGEF12	cna	hetloss	METABRIC
MB-5107	ARHGEF12	cna	hetloss	METABRIC
MB-6271	ARHGEF12	cna	hetloss	METABRIC
MB-7032	ARHGEF12	cna	hetloss	METABRIC
PD10014	ARHGEF12	cna	gain	BASIS
PD11327	ARHGEF12	cna	gain	BASIS
PD11742	ARHGEF12	cna	hetloss	BASIS
PD13296	ARHGEF12	cna	hetloss	BASIS
PD13297	ARHGEF12	cna	hetloss	BASIS
PD13771	ARHGEF12	cna	hetloss	BASIS
PD22355	ARHGEF12	cna	hetloss	BASIS
PD23574	ARHGEF12	cna	gain	BASIS
PD23578	ARHGEF12	cna	hetloss	BASIS
PD24186	ARHGEF12	cna	gain	BASIS
PD24206	ARHGEF12	cna	gain	BASIS
PD24337	ARHGEF12	cna	hetloss	BASIS
PD3890	ARHGEF12	cna	gain	BASIS
PD3905	ARHGEF12	cna	gain	BASIS
PD4005	ARHGEF12	cna	hetloss	BASIS
PD4826	ARHGEF12	cna	hetloss	BASIS
PD5935	ARHGEF12	cna	gain	BASIS
PD5945	ARHGEF12	cna	gain	BASIS
PD5948	ARHGEF12	cna	gain	BASIS
PD6413	ARHGEF12	cna	hetloss	BASIS
PD6731	ARHGEF12	cna	hetloss	BASIS
PD7067	ARHGEF12	cna	gain	BASIS
PD7215	ARHGEF12	cna	gain	BASIS
PD9004	ARHGEF12	cna	hetloss	BASIS
PD9702	ARHGEF12	cna	gain	BASIS
TCGA-A2-A25B	ARID1A	cna	hetloss	TCGA
TCGA-AN-A0XU	ARID1A	cna	hetloss	TCGA
TCGA-AO-A0JL	ARID1A	cna	hetloss	TCGA
TCGA-BH-A0AW	ARID1A	cna	gain	TCGA

TCGA-BH-A0C0	ARID1A	cna	hetloss	TCGA
TCGA-BH-A18R	ARID1A	cna	homdel	TCGA
TCGA-BH-A1FU	ARID1A	cna	gain	TCGA
TCGA-C8-A12L	ARID1A	cna	hetloss	TCGA
TCGA-D8-A27M	ARID1A	cna	gain	TCGA
TCGA-E2-A1L7	ARID1A	cna	hetloss	TCGA
TCGA-E9-A1NC	ARID1A	cna	hetloss	TCGA
TCGA-EW-A1OX	ARID1A	cna	homdel	TCGA
TCGA-LL-A5YP	ARID1A	cna	gain	TCGA
MB-0346	ARID1A	cna	hetloss	METABRIC
MB-2827	ARID1A	cna	hetloss	METABRIC
MB-5070	ARID1A	cna	hetloss	METABRIC
MB-5465	ARID1A	cna	hetloss	METABRIC
MB-6060	ARID1A	cna	hetloss	METABRIC
MB-6098	ARID1A	cna	hetloss	METABRIC
PD10014	ARID1A	cna	gain	BASIS
PD11327	ARID1A	cna	hetloss	BASIS
PD11742	ARID1A	cna	hetloss	BASIS
PD13296	ARID1A	cna	hetloss	BASIS
PD13771	ARID1A	cna	hetloss	BASIS
PD22355	ARID1A	cna	hetloss	BASIS
PD24186	ARID1A	cna	gain	BASIS
PD24206	ARID1A	cna	hetloss	BASIS
PD24337	ARID1A	cna	hetloss	BASIS
PD3890	ARID1A	cna	hetloss	BASIS
PD3905	ARID1A	cna	gain	BASIS
PD4006	ARID1A	cna	gain	BASIS
PD4826	ARID1A	cna	gain	BASIS
PD5945	ARID1A	cna	amp	BASIS
PD5948	ARID1A	cna	gain	BASIS
PD7067	ARID1A	cna	gain	BASIS
PD7215	ARID1A	cna	gain	BASIS
PD9004	ARID1A	cna	hetloss	BASIS
PD9585	ARID1A	cna	hetloss	BASIS
PD9702	ARID1A	cna	gain	BASIS
TCGA-A2-A25B	ATP1B4	cna	hetloss	TCGA
TCGA-AN-A0XU	ATP1B4	cna	hetloss	TCGA
TCGA-BH-A0AW	ATP1B4	cna	gain	TCGA
TCGA-C8-A12L	ATP1B4	cna	gain	TCGA
TCGA-E2-A1L7	ATP1B4	cna	hetloss	TCGA
TCGA-E9-A1NC	ATP1B4	cna	hetloss	TCGA
MB-0346	ATP1B4	cna	hetloss	METABRIC
MB-2827	ATP1B4	cna	hetloss	METABRIC
MB-5465	ATP1B4	cna	hetloss	METABRIC
MB-6098	ATP1B4	cna	hetloss	METABRIC
MB-7038	ATP1B4	cna	gain	METABRIC
PD10014	ATP1B4	cna	hetloss	BASIS

PD11327	ATP1B4	cna	homdel	BASIS
PD13296	ATP1B4	cna	hetloss	BASIS
PD13299	ATP1B4	cna	amp	BASIS
PD13771	ATP1B4	cna	gain	BASIS
PD14442	ATP1B4	cna	gain	BASIS
PD23562	ATP1B4	cna	hetloss	BASIS
PD23574	ATP1B4	cna	gain	BASIS
PD23578	ATP1B4	cna	hetloss	BASIS
PD24202	ATP1B4	cna	hetloss	BASIS
PD24206	ATP1B4	cna	gain	BASIS
PD3905	ATP1B4	cna	gain	BASIS
PD4005	ATP1B4	cna	gain	BASIS
PD4006	ATP1B4	cna	gain	BASIS
PD4107	ATP1B4	cna	gain	BASIS
PD4826	ATP1B4	cna	gain	BASIS
PD4967	ATP1B4	cna	gain	BASIS
PD5930	ATP1B4	cna	gain	BASIS
PD5935	ATP1B4	cna	gain	BASIS
PD5945	ATP1B4	cna	gain	BASIS
PD5948	ATP1B4	cna	gain	BASIS
PD6406	ATP1B4	cna	gain	BASIS
PD6413	ATP1B4	cna	gain	BASIS
PD7067	ATP1B4	cna	gain	BASIS
PD7215	ATP1B4	cna	gain	BASIS
PD8621	ATP1B4	cna	amp	BASIS
PD8980	ATP1B4	cna	hetloss	BASIS
PD9004	ATP1B4	cna	gain	BASIS
TCGA-A2-A25B	BCL6	cna	amp	TCGA
TCGA-AN-A0XU	BCL6	cna	gain	TCGA
TCGA-BH-A0AW	BCL6	cna	gain	TCGA
TCGA-BH-A0C0	BCL6	cna	gain	TCGA
TCGA-BH-A1FU	BCL6	cna	hetloss	TCGA
TCGA-C8-A12L	BCL6	cna	gain	TCGA
TCGA-LL-A5YP	BCL6	cna	gain	TCGA
MB-0346	BCL6	cna	gain	METABRIC
MB-5070	BCL6	cna	hetloss	METABRIC
MB-5107	BCL6	cna	amp	METABRIC
MB-5465	BCL6	cna	gain	METABRIC
MB-6060	BCL6	cna	gain	METABRIC
MB-6098	BCL6	cna	gain	METABRIC
MB-7038	BCL6	cna	hetloss	METABRIC
P-0002858-T01-IM3	BCL6	cna	amp	MSK-IMPACT
PD11327	BCL6	cna	gain	BASIS
PD13299	BCL6	cna	gain	BASIS
PD13771	BCL6	cna	hetloss	BASIS
PD22355	BCL6	cna	gain	BASIS
PD23561	BCL6	cna	hetloss	BASIS

PD23562	BCL6	cna	gain	BASIS
PD23578	BCL6	cna	gain	BASIS
PD24186	BCL6	cna	amp	BASIS
PD24202	BCL6	cna	gain	BASIS
PD3905	BCL6	cna	gain	BASIS
PD4005	BCL6	cna	gain	BASIS
PD4107	BCL6	cna	gain	BASIS
PD4826	BCL6	cna	gain	BASIS
PD5930	BCL6	cna	gain	BASIS
PD5945	BCL6	cna	amp	BASIS
PD5948	BCL6	cna	gain	BASIS
PD6406	BCL6	cna	gain	BASIS
PD6731	BCL6	cna	gain	BASIS
PD7067	BCL6	cna	gain	BASIS
PD7215	BCL6	cna	gain	BASIS
PD8621	BCL6	cna	gain	BASIS
PD8980	BCL6	cna	gain	BASIS
PD9004	BCL6	cna	gain	BASIS
PD9702	BCL6	cna	gain	BASIS
TCGA-A2-A25B	BCORL1	cna	hetloss	TCGA
TCGA-AN-A0XU	BCORL1	cna	hetloss	TCGA
TCGA-BH-A0AW	BCORL1	cna	gain	TCGA
TCGA-C8-A12L	BCORL1	cna	gain	TCGA
TCGA-E2-A1L7	BCORL1	cna	hetloss	TCGA
TCGA-E9-A1NC	BCORL1	cna	hetloss	TCGA
TCGA-LL-A5YP	BCORL1	cna	gain	TCGA
MB-0346	BCORL1	cna	hetloss	METABRIC
MB-2827	BCORL1	cna	hetloss	METABRIC
MB-5465	BCORL1	cna	hetloss	METABRIC
MB-6060	BCORL1	cna	gain	METABRIC
MB-6098	BCORL1	cna	hetloss	METABRIC
MB-7038	BCORL1	cna	gain	METABRIC
MB-0420	BCORL1	cna	hetloss	METABRIC
PD10014	BCORL1	cna	hetloss	BASIS
PD13296	BCORL1	cna	gain	BASIS
PD13299	BCORL1	cna	amp	BASIS
PD13771	BCORL1	cna	gain	BASIS
PD14442	BCORL1	cna	gain	BASIS
PD23562	BCORL1	cna	hetloss	BASIS
PD23574	BCORL1	cna	gain	BASIS
PD23578	BCORL1	cna	hetloss	BASIS
PD24202	BCORL1	cna	hetloss	BASIS
PD24206	BCORL1	cna	gain	BASIS
PD3890	BCORL1	cna	hetloss	BASIS
PD3905	BCORL1	cna	gain	BASIS
PD4107	BCORL1	cna	gain	BASIS
PD4826	BCORL1	cna	gain	BASIS

PD4967	BCORL1	cna	gain	BASIS
PD5930	BCORL1	cna	gain	BASIS
PD5935	BCORL1	cna	gain	BASIS
PD5945	BCORL1	cna	gain	BASIS
PD5948	BCORL1	cna	gain	BASIS
PD6406	BCORL1	cna	gain	BASIS
PD6413	BCORL1	cna	gain	BASIS
PD7215	BCORL1	cna	gain	BASIS
PD8621	BCORL1	cna	amp	BASIS
PD8980	BCORL1	cna	hetloss	BASIS
PD9004	BCORL1	cna	gain	BASIS
TCGA-A2-A25B	BIRC2	cna	hetloss	TCGA
TCGA-AN-A0XU	BIRC2	cna	gain	TCGA
TCGA-AO-A0JL	BIRC2	cna	hetloss	TCGA
TCGA-BH-A18R	BIRC2	cna	gain	TCGA
TCGA-D8-A27M	BIRC2	cna	hetloss	TCGA
TCGA-EW-A1OX	BIRC2	cna	homdel	TCGA
TCGA-LL-A5YP	BIRC2	cna	hetloss	TCGA
MB-0346	BIRC2	cna	hetloss	METABRIC
MB-5070	BIRC2	cna	hetloss	METABRIC
MB-5107	BIRC2	cna	hetloss	METABRIC
MB-6271	BIRC2	cna	hetloss	METABRIC
MB-7032	BIRC2	cna	hetloss	METABRIC
MB-7048	BIRC2	cna	hetloss	METABRIC
MTS-T0064	BIRC2	cna	amp	METABRIC
PD10014	BIRC2	cna	gain	BASIS
PD11327	BIRC2	cna	gain	BASIS
PD11742	BIRC2	cna	hetloss	BASIS
PD13296	BIRC2	cna	hetloss	BASIS
PD13299	BIRC2	cna	gain	BASIS
PD13771	BIRC2	cna	hetloss	BASIS
PD22355	BIRC2	cna	hetloss	BASIS
PD23574	BIRC2	cna	gain	BASIS
PD23578	BIRC2	cna	hetloss	BASIS
PD24186	BIRC2	cna	gain	BASIS
PD24206	BIRC2	cna	gain	BASIS
PD24337	BIRC2	cna	hetloss	BASIS
PD3905	BIRC2	cna	gain	BASIS
PD4005	BIRC2	cna	hetloss	BASIS
PD4006	BIRC2	cna	hetloss	BASIS
PD4826	BIRC2	cna	hetloss	BASIS
PD5935	BIRC2	cna	gain	BASIS
PD5945	BIRC2	cna	gain	BASIS
PD5948	BIRC2	cna	gain	BASIS
PD6413	BIRC2	cna	hetloss	BASIS
PD6731	BIRC2	cna	hetloss	BASIS
PD7067	BIRC2	cna	amp	BASIS

PD7215	BIRC2	cna	gain	BASIS
PD9585	BIRC2	cna	gain	BASIS
PD9702	BIRC2	cna	gain	BASIS
TCGA-A2-A25B	BIRC3	cna	hetloss	TCGA
TCGA-AN-A0XU	BIRC3	cna	gain	TCGA
TCGA-AO-A0JL	BIRC3	cna	hetloss	TCGA
TCGA-BH-A18R	BIRC3	cna	gain	TCGA
TCGA-D8-A27M	BIRC3	cna	hetloss	TCGA
TCGA-EW-A1OX	BIRC3	cna	homdel	TCGA
TCGA-LL-A5YP	BIRC3	cna	hetloss	TCGA
MB-0346	BIRC3	cna	hetloss	METABRIC
MB-5070	BIRC3	cna	hetloss	METABRIC
MB-5107	BIRC3	cna	hetloss	METABRIC
MB-6271	BIRC3	cna	hetloss	METABRIC
MB-7032	BIRC3	cna	hetloss	METABRIC
MB-7048	BIRC3	cna	hetloss	METABRIC
MTS-T0064	BIRC3	cna	amp	METABRIC
PD10014	BIRC3	cna	gain	BASIS
PD11327	BIRC3	cna	gain	BASIS
PD11742	BIRC3	cna	hetloss	BASIS
PD13296	BIRC3	cna	hetloss	BASIS
PD13299	BIRC3	cna	gain	BASIS
PD13771	BIRC3	cna	hetloss	BASIS
PD22355	BIRC3	cna	hetloss	BASIS
PD23574	BIRC3	cna	gain	BASIS
PD23578	BIRC3	cna	hetloss	BASIS
PD24186	BIRC3	cna	gain	BASIS
PD24206	BIRC3	cna	gain	BASIS
PD24337	BIRC3	cna	hetloss	BASIS
PD3905	BIRC3	cna	gain	BASIS
PD4005	BIRC3	cna	hetloss	BASIS
PD4006	BIRC3	cna	hetloss	BASIS
PD4826	BIRC3	cna	hetloss	BASIS
PD5935	BIRC3	cna	gain	BASIS
PD5945	BIRC3	cna	gain	BASIS
PD5948	BIRC3	cna	gain	BASIS
PD6413	BIRC3	cna	hetloss	BASIS
PD6731	BIRC3	cna	hetloss	BASIS
PD7067	BIRC3	cna	amp	BASIS
PD7215	BIRC3	cna	gain	BASIS
PD9585	BIRC3	cna	gain	BASIS
PD9702	BIRC3	cna	gain	BASIS
TCGA-A2-A25B	CARS	cna	hetloss	TCGA
TCGA-AN-A0XU	CARS	cna	hetloss	TCGA
TCGA-AO-A0JL	CARS	cna	hetloss	TCGA
TCGA-BH-A0C0	CARS	cna	hetloss	TCGA
TCGA-C8-A12L	CARS	cna	hetloss	TCGA

TCGA-E2-A1L7	CARS	cna	homdel	TCGA
TCGA-E9-A1NC	CARS	cna	hetloss	TCGA
TCGA-LL-A5YP	CARS	cna	hetloss	TCGA
MB-0346	CARS	cna	hetloss	METABRIC
MB-5070	CARS	cna	hetloss	METABRIC
MB-6060	CARS	cna	hetloss	METABRIC
MB-6098	CARS	cna	hetloss	METABRIC
MB-6271	CARS	cna	gain	METABRIC
MB-7032	CARS	cna	hetloss	METABRIC
MB-7048	CARS	cna	hetloss	METABRIC
MB-0420	CARS	cna	hetloss	METABRIC
PD10014	CARS	cna	hetloss	BASIS
PD11742	CARS	cna	hetloss	BASIS
PD13296	CARS	cna	hetloss	BASIS
PD13297	CARS	cna	hetloss	BASIS
PD22355	CARS	cna	hetloss	BASIS
PD24202	CARS	cna	hetloss	BASIS
PD24206	CARS	cna	gain	BASIS
PD3890	CARS	cna	hetloss	BASIS
PD3905	CARS	cna	gain	BASIS
PD4005	CARS	cna	hetloss	BASIS
PD4006	CARS	cna	gain	BASIS
PD4107	CARS	cna	gain	BASIS
PD4826	CARS	cna	gain	BASIS
PD4967	CARS	cna	gain	BASIS
PD5935	CARS	cna	gain	BASIS
PD5945	CARS	cna	gain	BASIS
PD5948	CARS	cna	gain	BASIS
PD6406	CARS	cna	hetloss	BASIS
PD6413	CARS	cna	hetloss	BASIS
PD6731	CARS	cna	hetloss	BASIS
PD8980	CARS	cna	hetloss	BASIS
PD9004	CARS	cna	hetloss	BASIS
PD9585	CARS	cna	hetloss	BASIS
TCGA-AN-A0XU	CBFA2T3	cna	gain	TCGA
TCGA-AO-A0JL	CBFA2T3	cna	gain	TCGA
TCGA-BH-A0AW	CBFA2T3	cna	hetloss	TCGA
TCGA-BH-A0C0	CBFA2T3	cna	gain	TCGA
TCGA-C8-A12L	CBFA2T3	cna	hetloss	TCGA
TCGA-E2-A1L7	CBFA2T3	cna	hetloss	TCGA
TCGA-E9-A1NC	CBFA2T3	cna	hetloss	TCGA
TCGA-LL-A5YP	CBFA2T3	cna	gain	TCGA
MB-0346	CBFA2T3	cna	gain	METABRIC
MB-5107	CBFA2T3	cna	hetloss	METABRIC
MB-6271	CBFA2T3	cna	hetloss	METABRIC
MB-7032	CBFA2T3	cna	hetloss	METABRIC
MB-7038	CBFA2T3	cna	gain	METABRIC

MB-7048	CBFA2T3	cna	hetloss	METABRIC
MB-0420	CBFA2T3	cna	hetloss	METABRIC
PD10014	CBFA2T3	cna	hetloss	BASIS
PD11327	CBFA2T3	cna	gain	BASIS
PD11742	CBFA2T3	cna	hetloss	BASIS
PD13296	CBFA2T3	cna	hetloss	BASIS
PD13297	CBFA2T3	cna	hetloss	BASIS
PD13299	CBFA2T3	cna	gain	BASIS
PD14442	CBFA2T3	cna	hetloss	BASIS
PD23562	CBFA2T3	cna	gain	BASIS
PD23574	CBFA2T3	cna	gain	BASIS
PD24202	CBFA2T3	cna	hetloss	BASIS
PD3890	CBFA2T3	cna	gain	BASIS
PD3905	CBFA2T3	cna	gain	BASIS
PD4005	CBFA2T3	cna	hetloss	BASIS
PD4006	CBFA2T3	cna	gain	BASIS
PD4107	CBFA2T3	cna	amp	BASIS
PD4967	CBFA2T3	cna	hetloss	BASIS
PD5930	CBFA2T3	cna	hetloss	BASIS
PD5945	CBFA2T3	cna	amp	BASIS
PD5948	CBFA2T3	cna	gain	BASIS
PD7215	CBFA2T3	cna	gain	BASIS
PD8621	CBFA2T3	cna	gain	BASIS
PD9004	CBFA2T3	cna	gain	BASIS
PD9585	CBFA2T3	cna	hetloss	BASIS
PD9702	CBFA2T3	cna	gain	BASIS
TCGA-A2-A25B	CCT6B	cna	gain	TCGA
TCGA-AO-A0JL	CCT6B	cna	gain	TCGA
TCGA-BH-A0AW	CCT6B	cna	gain	TCGA
TCGA-BH-A0C0	CCT6B	cna	gain	TCGA
TCGA-BH-A18R	CCT6B	cna	amp	TCGA
TCGA-C8-A12L	CCT6B	cna	gain	TCGA
TCGA-D8-A27M	CCT6B	cna	hetloss	TCGA
TCGA-E2-A1L7	CCT6B	cna	hetloss	TCGA
TCGA-E9-A1NC	CCT6B	cna	hetloss	TCGA
MB-0346	CCT6B	cna	hetloss	METABRIC
MB-2827	CCT6B	cna	hetloss	METABRIC
MB-5070	CCT6B	cna	gain	METABRIC
MB-5465	CCT6B	cna	hetloss	METABRIC
MB-6060	CCT6B	cna	hetloss	METABRIC
MB-6098	CCT6B	cna	hetloss	METABRIC
MB-6271	CCT6B	cna	hetloss	METABRIC
MB-7038	CCT6B	cna	hetloss	METABRIC
MB-7048	CCT6B	cna	hetloss	METABRIC
MB-0420	CCT6B	cna	hetloss	METABRIC
PD11327	CCT6B	cna	gain	BASIS
PD11742	CCT6B	cna	hetloss	BASIS

PD13297	CCT6B	cna	hetloss	BASIS
PD13299	CCT6B	cna	gain	BASIS
PD23561	CCT6B	cna	amp	BASIS
PD24206	CCT6B	cna	hetloss	BASIS
PD24337	CCT6B	cna	hetloss	BASIS
PD3890	CCT6B	cna	hetloss	BASIS
PD4005	CCT6B	cna	hetloss	BASIS
PD4967	CCT6B	cna	hetloss	BASIS
PD5945	CCT6B	cna	gain	BASIS
PD5948	CCT6B	cna	gain	BASIS
PD6406	CCT6B	cna	hetloss	BASIS
PD6413	CCT6B	cna	hetloss	BASIS
PD6731	CCT6B	cna	hetloss	BASIS
PD7067	CCT6B	cna	gain	BASIS
PD7215	CCT6B	cna	gain	BASIS
PD9004	CCT6B	cna	gain	BASIS
PD9585	CCT6B	cna	hetloss	BASIS
PD9702	CCT6B	cna	gain	BASIS
TCGA-AN-A0XU	CDH11	cna	hetloss	TCGA
TCGA-AO-A0JL	CDH11	cna	gain	TCGA
TCGA-BH-A0AW	CDH11	cna	hetloss	TCGA
TCGA-BH-A0C0	CDH11	cna	hetloss	TCGA
TCGA-C8-A12L	CDH11	cna	gain	TCGA
TCGA-D8-A27M	CDH11	cna	hetloss	TCGA
TCGA-E2-A1L7	CDH11	cna	hetloss	TCGA
TCGA-E9-A1NC	CDH11	cna	hetloss	TCGA
TCGA-LL-A5YP	CDH11	cna	gain	TCGA
MB-0346	CDH11	cna	gain	METABRIC
MB-5107	CDH11	cna	hetloss	METABRIC
MB-6271	CDH11	cna	hetloss	METABRIC
MB-7032	CDH11	cna	gain	METABRIC
MB-7048	CDH11	cna	hetloss	METABRIC
MB-0420	CDH11	cna	hetloss	METABRIC
PD10014	CDH11	cna	hetloss	BASIS
PD11327	CDH11	cna	gain	BASIS
PD11742	CDH11	cna	hetloss	BASIS
PD13296	CDH11	cna	hetloss	BASIS
PD13297	CDH11	cna	hetloss	BASIS
PD13299	CDH11	cna	gain	BASIS
PD14442	CDH11	cna	hetloss	BASIS
PD23562	CDH11	cna	gain	BASIS
PD24186	CDH11	cna	gain	BASIS
PD24202	CDH11	cna	hetloss	BASIS
PD24206	CDH11	cna	gain	BASIS
PD24337	CDH11	cna	hetloss	BASIS
PD3905	CDH11	cna	amp	BASIS
PD4005	CDH11	cna	hetloss	BASIS

PD4006	CDH11	cna	hetloss	BASIS
PD4107	CDH11	cna	hetloss	BASIS
PD4967	CDH11	cna	hetloss	BASIS
PD5945	CDH11	cna	gain	BASIS
PD5948	CDH11	cna	hetloss	BASIS
PD7215	CDH11	cna	gain	BASIS
PD8621	CDH11	cna	hetloss	BASIS
PD8980	CDH11	cna	hetloss	BASIS
PD9585	CDH11	cna	hetloss	BASIS
PD9702	CDH11	cna	gain	BASIS
TCGA-A2-A25B	CUL4B	cna	hetloss	TCGA
TCGA-AN-A0XU	CUL4B	cna	hetloss	TCGA
TCGA-BH-A0AW	CUL4B	cna	gain	TCGA
TCGA-C8-A12L	CUL4B	cna	gain	TCGA
TCGA-E2-A1L7	CUL4B	cna	hetloss	TCGA
TCGA-E9-A1NC	CUL4B	cna	hetloss	TCGA
MB-0346	CUL4B	cna	hetloss	METABRIC
MB-2827	CUL4B	cna	hetloss	METABRIC
MB-5465	CUL4B	cna	hetloss	METABRIC
MB-6098	CUL4B	cna	hetloss	METABRIC
MB-7038	CUL4B	cna	gain	METABRIC
PD10014	CUL4B	cna	hetloss	BASIS
PD11327	CUL4B	cna	homdel	BASIS
PD13296	CUL4B	cna	hetloss	BASIS
PD13299	CUL4B	cna	amp	BASIS
PD13771	CUL4B	cna	gain	BASIS
PD14442	CUL4B	cna	gain	BASIS
PD23562	CUL4B	cna	hetloss	BASIS
PD23574	CUL4B	cna	gain	BASIS
PD23578	CUL4B	cna	hetloss	BASIS
PD24202	CUL4B	cna	hetloss	BASIS
PD24206	CUL4B	cna	gain	BASIS
PD3905	CUL4B	cna	gain	BASIS
PD4005	CUL4B	cna	gain	BASIS
PD4006	CUL4B	cna	gain	BASIS
PD4107	CUL4B	cna	gain	BASIS
PD4826	CUL4B	cna	gain	BASIS
PD4967	CUL4B	cna	gain	BASIS
PD5930	CUL4B	cna	gain	BASIS
PD5935	CUL4B	cna	gain	BASIS
PD5945	CUL4B	cna	gain	BASIS
PD5948	CUL4B	cna	gain	BASIS
PD6406	CUL4B	cna	gain	BASIS
PD6413	CUL4B	cna	gain	BASIS
PD7067	CUL4B	cna	gain	BASIS
PD7215	CUL4B	cna	gain	BASIS
PD8621	CUL4B	cna	amp	BASIS

PD8980	CUL4B	cna	hetloss	BASIS
PD9004	CUL4B	cna	gain	BASIS
TCGA-A2-A25B	DDX3X	cna	gain	TCGA
TCGA-AN-A0XU	DDX3X	cna	hetloss	TCGA
TCGA-AO-A0JL	DDX3X	cna	gain	TCGA
TCGA-BH-A0AW	DDX3X	cna	gain	TCGA
TCGA-E2-A1L7	DDX3X	cna	hetloss	TCGA
TCGA-E9-A1NC	DDX3X	cna	hetloss	TCGA
MB-0346	DDX3X	cna	hetloss	METABRIC
MB-2827	DDX3X	cna	hetloss	METABRIC
MB-5070	DDX3X	cna	hetloss	METABRIC
MB-5465	DDX3X	cna	gain	METABRIC
MB-6098	DDX3X	cna	hetloss	METABRIC
MB-0420	DDX3X	cna	hetloss	METABRIC
MTS-T0064	DDX3X	cna	hetloss	METABRIC
PD10014	DDX3X	cna	hetloss	BASIS
PD11327	DDX3X	cna	homdel	BASIS
PD11742	DDX3X	cna	hetloss	BASIS
PD13296	DDX3X	cna	gain	BASIS
PD13299	DDX3X	cna	amp	BASIS
PD13771	DDX3X	cna	gain	BASIS
PD14442	DDX3X	cna	gain	BASIS
PD23562	DDX3X	cna	hetloss	BASIS
PD23574	DDX3X	cna	gain	BASIS
PD23578	DDX3X	cna	hetloss	BASIS
PD24206	DDX3X	cna	homdel	BASIS
PD3905	DDX3X	cna	gain	BASIS
PD4005	DDX3X	cna	hetloss	BASIS
PD4006	DDX3X	cna	hetloss	BASIS
PD4107	DDX3X	cna	gain	BASIS
PD4826	DDX3X	cna	gain	BASIS
PD4967	DDX3X	cna	gain	BASIS
PD5935	DDX3X	cna	gain	BASIS
PD5948	DDX3X	cna	gain	BASIS
PD6406	DDX3X	cna	gain	BASIS
PD6413	DDX3X	cna	gain	BASIS
PD7215	DDX3X	cna	gain	BASIS
PD8621	DDX3X	cna	amp	BASIS
PD8980	DDX3X	cna	hetloss	BASIS
PD9004	DDX3X	cna	gain	BASIS
PD9702	DDX3X	cna	gain	BASIS
TCGA-A2-A25B	ELF4	cna	hetloss	TCGA
TCGA-AN-A0XU	ELF4	cna	hetloss	TCGA
TCGA-BH-A0AW	ELF4	cna	gain	TCGA
TCGA-C8-A12L	ELF4	cna	gain	TCGA
TCGA-E2-A1L7	ELF4	cna	hetloss	TCGA
TCGA-E9-A1NC	ELF4	cna	hetloss	TCGA

TCGA-LL-A5YP	ELF4	cna	gain	TCGA
MB-0346	ELF4	cna	hetloss	METABRIC
MB-2827	ELF4	cna	hetloss	METABRIC
MB-5465	ELF4	cna	hetloss	METABRIC
MB-6060	ELF4	cna	gain	METABRIC
MB-6098	ELF4	cna	hetloss	METABRIC
MB-7038	ELF4	cna	gain	METABRIC
MB-0420	ELF4	cna	hetloss	METABRIC
PD10014	ELF4	cna	hetloss	BASIS
PD13296	ELF4	cna	gain	BASIS
PD13299	ELF4	cna	amp	BASIS
PD13771	ELF4	cna	gain	BASIS
PD14442	ELF4	cna	gain	BASIS
PD23562	ELF4	cna	hetloss	BASIS
PD23574	ELF4	cna	gain	BASIS
PD23578	ELF4	cna	hetloss	BASIS
PD24202	ELF4	cna	hetloss	BASIS
PD24206	ELF4	cna	gain	BASIS
PD3890	ELF4	cna	hetloss	BASIS
PD3905	ELF4	cna	gain	BASIS
PD4107	ELF4	cna	gain	BASIS
PD4826	ELF4	cna	gain	BASIS
PD4967	ELF4	cna	gain	BASIS
PD5930	ELF4	cna	gain	BASIS
PD5935	ELF4	cna	gain	BASIS
PD5945	ELF4	cna	gain	BASIS
PD5948	ELF4	cna	gain	BASIS
PD6406	ELF4	cna	gain	BASIS
PD6413	ELF4	cna	gain	BASIS
PD7215	ELF4	cna	gain	BASIS
PD8621	ELF4	cna	amp	BASIS
PD8980	ELF4	cna	hetloss	BASIS
PD9004	ELF4	cna	gain	BASIS
TCGA-A2-A25B	EPHA8	cna	hetloss	TCGA
TCGA-AN-A0XU	EPHA8	cna	hetloss	TCGA
TCGA-AO-A0JL	EPHA8	cna	hetloss	TCGA
TCGA-BH-A0AW	EPHA8	cna	gain	TCGA
TCGA-BH-A0C0	EPHA8	cna	hetloss	TCGA
TCGA-BH-A18R	EPHA8	cna	homdel	TCGA
TCGA-BH-A1FU	EPHA8	cna	gain	TCGA
TCGA-C8-A12L	EPHA8	cna	hetloss	TCGA
TCGA-D8-A27M	EPHA8	cna	gain	TCGA
TCGA-E2-A1L7	EPHA8	cna	hetloss	TCGA
TCGA-E9-A1NC	EPHA8	cna	hetloss	TCGA
TCGA-LL-A5YP	EPHA8	cna	hetloss	TCGA
MB-2827	EPHA8	cna	hetloss	METABRIC
MB-5070	EPHA8	cna	hetloss	METABRIC

MB-5465	EPHA8	cna	hetloss	METABRIC
MB-6060	EPHA8	cna	hetloss	METABRIC
MB-6098	EPHA8	cna	hetloss	METABRIC
MB-6271	EPHA8	cna	hetloss	METABRIC
MB-0420	EPHA8	cna	hetloss	METABRIC
PD10014	EPHA8	cna	gain	BASIS
PD11742	EPHA8	cna	hetloss	BASIS
PD13296	EPHA8	cna	hetloss	BASIS
PD13771	EPHA8	cna	hetloss	BASIS
PD22355	EPHA8	cna	hetloss	BASIS
PD23578	EPHA8	cna	gain	BASIS
PD24186	EPHA8	cna	gain	BASIS
PD24202	EPHA8	cna	hetloss	BASIS
PD24206	EPHA8	cna	gain	BASIS
PD24337	EPHA8	cna	hetloss	BASIS
PD3890	EPHA8	cna	hetloss	BASIS
PD3905	EPHA8	cna	gain	BASIS
PD4006	EPHA8	cna	gain	BASIS
PD4826	EPHA8	cna	gain	BASIS
PD5945	EPHA8	cna	amp	BASIS
PD6406	EPHA8	cna	hetloss	BASIS
PD7067	EPHA8	cna	gain	BASIS
PD7215	EPHA8	cna	gain	BASIS
PD9004	EPHA8	cna	hetloss	BASIS
PD9585	EPHA8	cna	hetloss	BASIS
TCGA-A2-A25B	EPHB6	cna	gain	TCGA
TCGA-AO-A0JL	EPHB6	cna	gain	TCGA
TCGA-BH-A0AW	EPHB6	cna	gain	TCGA
TCGA-D8-A27M	EPHB6	cna	gain	TCGA
TCGA-E2-A1L7	EPHB6	cna	hetloss	TCGA
TCGA-E9-A1NC	EPHB6	cna	hetloss	TCGA
TCGA-LL-A5YP	EPHB6	cna	gain	TCGA
MB-0346	EPHB6	cna	hetloss	METABRIC
MB-5070	EPHB6	cna	hetloss	METABRIC
MB-6098	EPHB6	cna	gain	METABRIC
MB-7038	EPHB6	cna	gain	METABRIC
MB-7048	EPHB6	cna	gain	METABRIC
MB-0420	EPHB6	cna	hetloss	METABRIC
PD10014	EPHB6	cna	gain	BASIS
PD13296	EPHB6	cna	hetloss	BASIS
PD13297	EPHB6	cna	gain	BASIS
PD13299	EPHB6	cna	gain	BASIS
PD22355	EPHB6	cna	hetloss	BASIS
PD23562	EPHB6	cna	gain	BASIS
PD23574	EPHB6	cna	gain	BASIS
PD23578	EPHB6	cna	hetloss	BASIS
PD24186	EPHB6	cna	amp	BASIS

PD24202	EPHB6	cna	gain	BASIS
PD24337	EPHB6	cna	gain	BASIS
PD3890	EPHB6	cna	hetloss	BASIS
PD3905	EPHB6	cna	gain	BASIS
PD4006	EPHB6	cna	gain	BASIS
PD4107	EPHB6	cna	gain	BASIS
PD5930	EPHB6	cna	gain	BASIS
PD5945	EPHB6	cna	amp	BASIS
PD5948	EPHB6	cna	gain	BASIS
PD6406	EPHB6	cna	gain	BASIS
PD6731	EPHB6	cna	hetloss	BASIS
PD7067	EPHB6	cna	gain	BASIS
PD7215	EPHB6	cna	gain	BASIS
PD8621	EPHB6	cna	gain	BASIS
PD9004	EPHB6	cna	gain	BASIS
PD9585	EPHB6	cna	gain	BASIS
PD9702	EPHB6	cna	gain	BASIS
TCGA-AN-A0XU	EXOSC6	cna	gain	TCGA
TCGA-AO-A0JL	EXOSC6	cna	gain	TCGA
TCGA-BH-A0C0	EXOSC6	cna	hetloss	TCGA
TCGA-BH-A18R	EXOSC6	cna	gain	TCGA
TCGA-C8-A12L	EXOSC6	cna	hetloss	TCGA
TCGA-D8-A27M	EXOSC6	cna	gain	TCGA
TCGA-E2-A1L7	EXOSC6	cna	hetloss	TCGA
TCGA-E9-A1NC	EXOSC6	cna	hetloss	TCGA
TCGA-LL-A5YP	EXOSC6	cna	gain	TCGA
MB-0346	EXOSC6	cna	gain	METABRIC
MB-2827	EXOSC6	cna	hetloss	METABRIC
MB-5107	EXOSC6	cna	hetloss	METABRIC
MB-6271	EXOSC6	cna	hetloss	METABRIC
MB-7032	EXOSC6	cna	gain	METABRIC
MB-7038	EXOSC6	cna	gain	METABRIC
MB-7048	EXOSC6	cna	hetloss	METABRIC
MB-0420	EXOSC6	cna	hetloss	METABRIC
MTS-T0064	EXOSC6	cna	hetloss	METABRIC
PD10014	EXOSC6	cna	hetloss	BASIS
PD11327	EXOSC6	cna	homdel	BASIS
PD11742	EXOSC6	cna	hetloss	BASIS
PD13297	EXOSC6	cna	hetloss	BASIS
PD13299	EXOSC6	cna	gain	BASIS
PD14442	EXOSC6	cna	homdel	BASIS
PD23562	EXOSC6	cna	gain	BASIS
PD24186	EXOSC6	cna	gain	BASIS
PD24202	EXOSC6	cna	hetloss	BASIS
PD24206	EXOSC6	cna	gain	BASIS
PD3890	EXOSC6	cna	gain	BASIS
PD3905	EXOSC6	cna	gain	BASIS

PD4005	EXOSC6	cna	hetloss	BASIS
PD4967	EXOSC6	cna	homdel	BASIS
PD5945	EXOSC6	cna	gain	BASIS
PD5948	EXOSC6	cna	hetloss	BASIS
PD6413	EXOSC6	cna	hetloss	BASIS
PD7215	EXOSC6	cna	gain	BASIS
PD8980	EXOSC6	cna	hetloss	BASIS
PD9585	EXOSC6	cna	hetloss	BASIS
PD9702	EXOSC6	cna	gain	BASIS
TCGA-A2-A25B	FAS	cna	hetloss	TCGA
TCGA-AN-A0XU	FAS	cna	hetloss	TCGA
TCGA-AO-A0JL	FAS	cna	hetloss	TCGA
TCGA-BH-A0AW	FAS	cna	hetloss	TCGA
TCGA-BH-A18R	FAS	cna	gain	TCGA
TCGA-C8-A12L	FAS	cna	hetloss	TCGA
TCGA-D8-A27M	FAS	cna	hetloss	TCGA
TCGA-E2-A1L7	FAS	cna	hetloss	TCGA
MB-0346	FAS	cna	gain	METABRIC
MB-2827	FAS	cna	hetloss	METABRIC
MB-5070	FAS	cna	hetloss	METABRIC
MB-6060	FAS	cna	hetloss	METABRIC
MB-6098	FAS	cna	hetloss	METABRIC
MB-7038	FAS	cna	hetloss	METABRIC
MB-7048	FAS	cna	homdel	METABRIC
MTS-T0064	FAS	cna	amp	METABRIC
PD10014	FAS	cna	hetloss	BASIS
PD11327	FAS	cna	gain	BASIS
PD11742	FAS	cna	hetloss	BASIS
PD13296	FAS	cna	hetloss	BASIS
PD13299	FAS	cna	gain	BASIS
PD22355	FAS	cna	hetloss	BASIS
PD23562	FAS	cna	gain	BASIS
PD23574	FAS	cna	gain	BASIS
PD23578	FAS	cna	hetloss	BASIS
PD24202	FAS	cna	hetloss	BASIS
PD3890	FAS	cna	hetloss	BASIS
PD3905	FAS	cna	gain	BASIS
PD4006	FAS	cna	gain	BASIS
PD4107	FAS	cna	gain	BASIS
PD5935	FAS	cna	homdel	BASIS
PD5948	FAS	cna	hetloss	BASIS
PD6406	FAS	cna	hetloss	BASIS
PD6413	FAS	cna	hetloss	BASIS
PD6731	FAS	cna	hetloss	BASIS
PD7215	FAS	cna	gain	BASIS
PD8621	FAS	cna	hetloss	BASIS
PD8980	FAS	cna	hetloss	BASIS

PD9585	FAS	cna	hetloss	BASIS
TCGA-A2-A25B	FAT1	cna	gain	TCGA
TCGA-AN-A0XU	FAT1	cna	hetloss	TCGA
TCGA-BH-A0AW	FAT1	cna	gain	TCGA
TCGA-BH-A0C0	FAT1	cna	hetloss	TCGA
TCGA-BH-A18R	FAT1	cna	homdel	TCGA
TCGA-C8-A12L	FAT1	cna	hetloss	TCGA
TCGA-D8-A27M	FAT1	cna	hetloss	TCGA
TCGA-E2-A1L7	FAT1	cna	hetloss	TCGA
TCGA-E9-A1NC	FAT1	cna	hetloss	TCGA
TCGA-LL-A5YP	FAT1	cna	hetloss	TCGA
MB-2827	FAT1	cna	hetloss	METABRIC
MB-5070	FAT1	cna	hetloss	METABRIC
MB-5107	FAT1	cna	hetloss	METABRIC
MB-5465	FAT1	cna	hetloss	METABRIC
MB-7038	FAT1	cna	hetloss	METABRIC
MB-7048	FAT1	cna	hetloss	METABRIC
MB-0420	FAT1	cna	hetloss	METABRIC
PD10014	FAT1	cna	hetloss	BASIS
PD11742	FAT1	cna	hetloss	BASIS
PD13296	FAT1	cna	hetloss	BASIS
PD13297	FAT1	cna	hetloss	BASIS
PD22355	FAT1	cna	hetloss	BASIS
PD23562	FAT1	cna	gain	BASIS
PD23578	FAT1	cna	hetloss	BASIS
PD24202	FAT1	cna	hetloss	BASIS
PD24206	FAT1	cna	gain	BASIS
PD24337	FAT1	cna	hetloss	BASIS
PD3905	FAT1	cna	gain	BASIS
PD4005	FAT1	cna	hetloss	BASIS
PD4107	FAT1	cna	hetloss	BASIS
PD5930	FAT1	cna	gain	BASIS
PD5945	FAT1	cna	gain	BASIS
PD5948	FAT1	cna	hetloss	BASIS
PD6406	FAT1	cna	hetloss	BASIS
PD6731	FAT1	cna	hetloss	BASIS
PD7067	FAT1	cna	gain	BASIS
PD7215	FAT1	cna	amp	BASIS
PD8980	FAT1	cna	hetloss	BASIS
PD9585	FAT1	cna	hetloss	BASIS
TCGA-A2-A25B	FGF12	cna	amp	TCGA
TCGA-AN-A0XU	FGF12	cna	gain	TCGA
TCGA-BH-A0AW	FGF12	cna	gain	TCGA
TCGA-BH-A0C0	FGF12	cna	gain	TCGA
TCGA-BH-A1FU	FGF12	cna	hetloss	TCGA
TCGA-C8-A12L	FGF12	cna	gain	TCGA
TCGA-D8-A27M	FGF12	cna	hetloss	TCGA

TCGA-LL-A5YP	FGF12	cna	gain	TCGA
MB-0346	FGF12	cna	gain	METABRIC
MB-5070	FGF12	cna	hetloss	METABRIC
MB-5107	FGF12	cna	amp	METABRIC
MB-5465	FGF12	cna	gain	METABRIC
MB-6060	FGF12	cna	gain	METABRIC
MB-6098	FGF12	cna	gain	METABRIC
MB-7038	FGF12	cna	hetloss	METABRIC
PD11327	FGF12	cna	gain	BASIS
PD13299	FGF12	cna	gain	BASIS
PD13771	FGF12	cna	hetloss	BASIS
PD22355	FGF12	cna	gain	BASIS
PD23561	FGF12	cna	hetloss	BASIS
PD23562	FGF12	cna	gain	BASIS
PD23578	FGF12	cna	gain	BASIS
PD24186	FGF12	cna	amp	BASIS
PD24202	FGF12	cna	gain	BASIS
PD3905	FGF12	cna	gain	BASIS
PD4005	FGF12	cna	gain	BASIS
PD4107	FGF12	cna	gain	BASIS
PD4826	FGF12	cna	gain	BASIS
PD5930	FGF12	cna	gain	BASIS
PD5945	FGF12	cna	amp	BASIS
PD5948	FGF12	cna	gain	BASIS
PD6406	FGF12	cna	gain	BASIS
PD6731	FGF12	cna	gain	BASIS
PD7067	FGF12	cna	gain	BASIS
PD7215	FGF12	cna	gain	BASIS
PD8621	FGF12	cna	gain	BASIS
PD8980	FGF12	cna	gain	BASIS
PD9004	FGF12	cna	gain	BASIS
PD9702	FGF12	cna	gain	BASIS
TCGA-A2-A25B	FGF7	cna	amp	TCGA
TCGA-AN-A0XU	FGF7	cna	hetloss	TCGA
TCGA-AO-A0JL	FGF7	cna	hetloss	TCGA
TCGA-BH-A0C0	FGF7	cna	hetloss	TCGA
TCGA-D8-A27M	FGF7	cna	hetloss	TCGA
TCGA-E2-A1L7	FGF7	cna	hetloss	TCGA
TCGA-E9-A1NC	FGF7	cna	hetloss	TCGA
TCGA-LL-A5YP	FGF7	cna	hetloss	TCGA
MB-0346	FGF7	cna	gain	METABRIC
MB-5107	FGF7	cna	gain	METABRIC
MB-5465	FGF7	cna	hetloss	METABRIC
MB-6060	FGF7	cna	hetloss	METABRIC
MB-6098	FGF7	cna	hetloss	METABRIC
MB-7048	FGF7	cna	hetloss	METABRIC
PD10014	FGF7	cna	hetloss	BASIS

PD11327	FGF7	cna	gain	BASIS
PD11742	FGF7	cna	hetloss	BASIS
PD13296	FGF7	cna	homdel	BASIS
PD13297	FGF7	cna	hetloss	BASIS
PD22355	FGF7	cna	hetloss	BASIS
PD24202	FGF7	cna	hetloss	BASIS
PD24206	FGF7	cna	gain	BASIS
PD24337	FGF7	cna	hetloss	BASIS
PD3890	FGF7	cna	hetloss	BASIS
PD3905	FGF7	cna	gain	BASIS
PD4005	FGF7	cna	hetloss	BASIS
PD4006	FGF7	cna	hetloss	BASIS
PD4107	FGF7	cna	hetloss	BASIS
PD5930	FGF7	cna	hetloss	BASIS
PD5935	FGF7	cna	gain	BASIS
PD5948	FGF7	cna	hetloss	BASIS
PD6406	FGF7	cna	hetloss	BASIS
PD6413	FGF7	cna	hetloss	BASIS
PD7067	FGF7	cna	gain	BASIS
PD7215	FGF7	cna	gain	BASIS
PD8621	FGF7	cna	hetloss	BASIS
PD8980	FGF7	cna	hetloss	BASIS
PD9585	FGF7	cna	hetloss	BASIS
PD9702	FGF7	cna	gain	BASIS
TCGA-AO-A0JL	FLT3	cna	hetloss	TCGA
TCGA-BH-A0AW	FLT3	cna	hetloss	TCGA
TCGA-BH-A0C0	FLT3	cna	gain	TCGA
TCGA-BH-A1FU	FLT3	cna	hetloss	TCGA
TCGA-C8-A12L	FLT3	cna	hetloss	TCGA
TCGA-D8-A27M	FLT3	cna	gain	TCGA
TCGA-E9-A1NC	FLT3	cna	hetloss	TCGA
TCGA-EW-A1OX	FLT3	cna	homdel	TCGA
TCGA-LL-A5YP	FLT3	cna	hetloss	TCGA
MB-0346	FLT3	cna	amp	METABRIC
MB-2827	FLT3	cna	hetloss	METABRIC
MB-6098	FLT3	cna	hetloss	METABRIC
MB-7032	FLT3	cna	gain	METABRIC
MB-0420	FLT3	cna	hetloss	METABRIC
PD10014	FLT3	cna	hetloss	BASIS
PD11327	FLT3	cna	hetloss	BASIS
PD13296	FLT3	cna	hetloss	BASIS
PD13297	FLT3	cna	hetloss	BASIS
PD13771	FLT3	cna	hetloss	BASIS
PD14442	FLT3	cna	hetloss	BASIS
PD22355	FLT3	cna	hetloss	BASIS
PD23562	FLT3	cna	gain	BASIS
PD23578	FLT3	cna	hetloss	BASIS

PD24202	FLT3	cna	hetloss	BASIS
PD24337	FLT3	cna	hetloss	BASIS
PD3890	FLT3	cna	hetloss	BASIS
PD3905	FLT3	cna	gain	BASIS
PD4005	FLT3	cna	homdel	BASIS
PD4006	FLT3	cna	gain	BASIS
PD4107	FLT3	cna	gain	BASIS
PD4826	FLT3	cna	hetloss	BASIS
PD4967	FLT3	cna	hetloss	BASIS
PD5945	FLT3	cna	amp	BASIS
PD6406	FLT3	cna	hetloss	BASIS
PD6413	FLT3	cna	hetloss	BASIS
PD6731	FLT3	cna	hetloss	BASIS
PD7067	FLT3	cna	gain	BASIS
PD8980	FLT3	cna	hetloss	BASIS
PD9702	FLT3	cna	gain	BASIS
TCGA-A2-A25B	FOXA2	cna	gain	TCGA
TCGA-AO-A0JL	FOXA2	cna	gain	TCGA
TCGA-BH-A0AW	FOXA2	cna	gain	TCGA
TCGA-BH-A0C0	FOXA2	cna	gain	TCGA
TCGA-C8-A12L	FOXA2	cna	gain	TCGA
TCGA-D8-A27M	FOXA2	cna	gain	TCGA
TCGA-E2-A1L7	FOXA2	cna	gain	TCGA
TCGA-E9-A1NC	FOXA2	cna	hetloss	TCGA
TCGA-LL-A5YP	FOXA2	cna	gain	TCGA
MB-0346	FOXA2	cna	gain	METABRIC
MB-5107	FOXA2	cna	hetloss	METABRIC
MB-6060	FOXA2	cna	gain	METABRIC
MB-6098	FOXA2	cna	gain	METABRIC
MB-7032	FOXA2	cna	gain	METABRIC
MB-7048	FOXA2	cna	gain	METABRIC
PD11327	FOXA2	cna	gain	BASIS
PD11742	FOXA2	cna	gain	BASIS
PD13296	FOXA2	cna	hetloss	BASIS
PD13299	FOXA2	cna	gain	BASIS
PD23574	FOXA2	cna	gain	BASIS
PD23578	FOXA2	cna	hetloss	BASIS
PD24186	FOXA2	cna	gain	BASIS
PD24202	FOXA2	cna	hetloss	BASIS
PD24206	FOXA2	cna	gain	BASIS
PD24337	FOXA2	cna	gain	BASIS
PD3905	FOXA2	cna	gain	BASIS
PD4005	FOXA2	cna	hetloss	BASIS
PD4107	FOXA2	cna	gain	BASIS
PD4826	FOXA2	cna	gain	BASIS
PD5930	FOXA2	cna	gain	BASIS
PD5935	FOXA2	cna	hetloss	BASIS

PD5945	FOXA2	cna	amp	BASIS
PD5948	FOXA2	cna	gain	BASIS
PD7067	FOXA2	cna	gain	BASIS
PD7215	FOXA2	cna	gain	BASIS
PD8621	FOXA2	cna	gain	BASIS
PD8980	FOXA2	cna	hetloss	BASIS
PD9004	FOXA2	cna	gain	BASIS
PD9702	FOXA2	cna	gain	BASIS
TCGA-A2-A25B	FOXO4	cna	gain	TCGA
TCGA-AN-A0XU	FOXO4	cna	hetloss	TCGA
TCGA-AO-A0JL	FOXO4	cna	gain	TCGA
TCGA-BH-A0AW	FOXO4	cna	gain	TCGA
TCGA-C8-A12L	FOXO4	cna	hetloss	TCGA
TCGA-E2-A1L7	FOXO4	cna	hetloss	TCGA
TCGA-E9-A1NC	FOXO4	cna	hetloss	TCGA
MB-0346	FOXO4	cna	hetloss	METABRIC
MB-2827	FOXO4	cna	hetloss	METABRIC
MB-5465	FOXO4	cna	gain	METABRIC
MB-6060	FOXO4	cna	hetloss	METABRIC
MB-0420	FOXO4	cna	hetloss	METABRIC
PD10014	FOXO4	cna	hetloss	BASIS
PD11327	FOXO4	cna	hetloss	BASIS
PD13296	FOXO4	cna	amp	BASIS
PD13297	FOXO4	cna	gain	BASIS
PD13299	FOXO4	cna	gain	BASIS
PD13771	FOXO4	cna	gain	BASIS
PD14442	FOXO4	cna	gain	BASIS
PD23562	FOXO4	cna	hetloss	BASIS
PD23574	FOXO4	cna	gain	BASIS
PD24202	FOXO4	cna	gain	BASIS
PD24206	FOXO4	cna	gain	BASIS
PD3890	FOXO4	cna	gain	BASIS
PD3905	FOXO4	cna	gain	BASIS
PD4005	FOXO4	cna	hetloss	BASIS
PD4006	FOXO4	cna	gain	BASIS
PD4107	FOXO4	cna	amp	BASIS
PD4826	FOXO4	cna	gain	BASIS
PD4967	FOXO4	cna	gain	BASIS
PD5930	FOXO4	cna	gain	BASIS
PD5935	FOXO4	cna	gain	BASIS
PD5948	FOXO4	cna	gain	BASIS
PD6413	FOXO4	cna	gain	BASIS
PD7067	FOXO4	cna	gain	BASIS
PD7215	FOXO4	cna	amp	BASIS
PD8621	FOXO4	cna	amp	BASIS
PD8980	FOXO4	cna	gain	BASIS
PD9004	FOXO4	cna	gain	BASIS

TCGA-A2-A25B	GOPC	cna	hetloss	TCGA
TCGA-AN-A0XU	GOPC	cna	amp	TCGA
TCGA-AO-A0JL	GOPC	cna	hetloss	TCGA
TCGA-BH-A0AW	GOPC	cna	amp	TCGA
TCGA-BH-A18R	GOPC	cna	homdel	TCGA
TCGA-C8-A12L	GOPC	cna	gain	TCGA
TCGA-D8-A27M	GOPC	cna	hetloss	TCGA
MB-2827	GOPC	cna	amp	METABRIC
MB-5070	GOPC	cna	gain	METABRIC
MB-6098	GOPC	cna	hetloss	METABRIC
MB-7032	GOPC	cna	hetloss	METABRIC
MB-7038	GOPC	cna	gain	METABRIC
MB-7048	GOPC	cna	hetloss	METABRIC
MTS-T0064	GOPC	cna	amp	METABRIC
PD11327	GOPC	cna	gain	BASIS
PD13299	GOPC	cna	gain	BASIS
PD13771	GOPC	cna	hetloss	BASIS
PD22355	GOPC	cna	gain	BASIS
PD23562	GOPC	cna	gain	BASIS
PD23574	GOPC	cna	gain	BASIS
PD24186	GOPC	cna	gain	BASIS
PD24206	GOPC	cna	gain	BASIS
PD24337	GOPC	cna	hetloss	BASIS
PD3905	GOPC	cna	gain	BASIS
PD4006	GOPC	cna	gain	BASIS
PD4107	GOPC	cna	hetloss	BASIS
PD4826	GOPC	cna	gain	BASIS
PD5930	GOPC	cna	hetloss	BASIS
PD5935	GOPC	cna	gain	BASIS
PD5945	GOPC	cna	amp	BASIS
PD6406	GOPC	cna	hetloss	BASIS
PD6731	GOPC	cna	hetloss	BASIS
PD7067	GOPC	cna	gain	BASIS
PD7215	GOPC	cna	gain	BASIS
PD8621	GOPC	cna	gain	BASIS
PD8980	GOPC	cna	gain	BASIS
PD9004	GOPC	cna	gain	BASIS
PD9585	GOPC	cna	hetloss	BASIS
PD9702	GOPC	cna	gain	BASIS
TCGA-AN-A0XU	GPC3	cna	hetloss	TCGA
TCGA-BH-A0AW	GPC3	cna	gain	TCGA
TCGA-C8-A12L	GPC3	cna	gain	TCGA
TCGA-E2-A1L7	GPC3	cna	hetloss	TCGA
TCGA-E9-A1NC	GPC3	cna	hetloss	TCGA
TCGA-LL-A5YP	GPC3	cna	gain	TCGA
MB-0346	GPC3	cna	hetloss	METABRIC
MB-2827	GPC3	cna	hetloss	METABRIC

MB-5465	GPC3	cna	hetloss	METABRIC
MB-6060	GPC3	cna	gain	METABRIC
MB-6098	GPC3	cna	hetloss	METABRIC
MB-7038	GPC3	cna	gain	METABRIC
MB-0420	GPC3	cna	hetloss	METABRIC
PD10014	GPC3	cna	hetloss	BASIS
PD13296	GPC3	cna	amp	BASIS
PD13299	GPC3	cna	amp	BASIS
PD13771	GPC3	cna	gain	BASIS
PD14442	GPC3	cna	gain	BASIS
PD23562	GPC3	cna	hetloss	BASIS
PD23574	GPC3	cna	gain	BASIS
PD23578	GPC3	cna	hetloss	BASIS
PD24202	GPC3	cna	hetloss	BASIS
PD24206	GPC3	cna	gain	BASIS
PD3890	GPC3	cna	hetloss	BASIS
PD3905	GPC3	cna	gain	BASIS
PD4006	GPC3	cna	gain	BASIS
PD4107	GPC3	cna	gain	BASIS
PD4826	GPC3	cna	gain	BASIS
PD4967	GPC3	cna	gain	BASIS
PD5930	GPC3	cna	gain	BASIS
PD5935	GPC3	cna	gain	BASIS
PD5945	GPC3	cna	gain	BASIS
PD5948	GPC3	cna	gain	BASIS
PD6406	GPC3	cna	gain	BASIS
PD6413	GPC3	cna	gain	BASIS
PD7215	GPC3	cna	gain	BASIS
PD8621	GPC3	cna	amp	BASIS
PD8980	GPC3	cna	hetloss	BASIS
PD9004	GPC3	cna	gain	BASIS
TCGA-A2-A25B	HDAC2	cna	hetloss	TCGA
TCGA-AN-A0XU	HDAC2	cna	amp	TCGA
TCGA-AO-A0JL	HDAC2	cna	hetloss	TCGA
TCGA-BH-A0AW	HDAC2	cna	amp	TCGA
TCGA-BH-A18R	HDAC2	cna	gain	TCGA
TCGA-C8-A12L	HDAC2	cna	gain	TCGA
TCGA-D8-A27M	HDAC2	cna	hetloss	TCGA
MB-2827	HDAC2	cna	amp	METABRIC
MB-6098	HDAC2	cna	hetloss	METABRIC
MB-7032	HDAC2	cna	hetloss	METABRIC
MB-7038	HDAC2	cna	gain	METABRIC
MB-7048	HDAC2	cna	hetloss	METABRIC
MTS-T0064	HDAC2	cna	amp	METABRIC
PD11327	HDAC2	cna	gain	BASIS
PD13296	HDAC2	cna	gain	BASIS
PD13299	HDAC2	cna	gain	BASIS

PD13771	HDAC2	cna	hetloss	BASIS
PD22355	HDAC2	cna	gain	BASIS
PD23562	HDAC2	cna	gain	BASIS
PD23574	HDAC2	cna	gain	BASIS
PD24186	HDAC2	cna	gain	BASIS
PD24206	HDAC2	cna	gain	BASIS
PD24337	HDAC2	cna	hetloss	BASIS
PD3905	HDAC2	cna	gain	BASIS
PD4006	HDAC2	cna	gain	BASIS
PD4107	HDAC2	cna	hetloss	BASIS
PD4826	HDAC2	cna	gain	BASIS
PD5930	HDAC2	cna	hetloss	BASIS
PD5935	HDAC2	cna	gain	BASIS
PD5945	HDAC2	cna	amp	BASIS
PD6406	HDAC2	cna	hetloss	BASIS
PD6731	HDAC2	cna	hetloss	BASIS
PD7067	HDAC2	cna	gain	BASIS
PD7215	HDAC2	cna	gain	BASIS
PD8621	HDAC2	cna	gain	BASIS
PD8980	HDAC2	cna	gain	BASIS
PD9004	HDAC2	cna	gain	BASIS
PD9585	HDAC2	cna	hetloss	BASIS
PD9702	HDAC2	cna	gain	BASIS
TCGA-AN-A0XU	HERPUD1	cna	hetloss	TCGA
TCGA-AO-A0JL	HERPUD1	cna	gain	TCGA
TCGA-BH-A0AW	HERPUD1	cna	hetloss	TCGA
TCGA-BH-A0C0	HERPUD1	cna	hetloss	TCGA
TCGA-C8-A12L	HERPUD1	cna	gain	TCGA
TCGA-D8-A27M	HERPUD1	cna	gain	TCGA
TCGA-E2-A1L7	HERPUD1	cna	hetloss	TCGA
TCGA-LL-A5YP	HERPUD1	cna	gain	TCGA
MB-0346	HERPUD1	cna	gain	METABRIC
MB-5070	HERPUD1	cna	hetloss	METABRIC
MB-5107	HERPUD1	cna	hetloss	METABRIC
MB-6271	HERPUD1	cna	hetloss	METABRIC
MB-7032	HERPUD1	cna	gain	METABRIC
MB-7048	HERPUD1	cna	hetloss	METABRIC
MB-0420	HERPUD1	cna	hetloss	METABRIC
MTS-T0064	HERPUD1	cna	hetloss	METABRIC
PD10014	HERPUD1	cna	hetloss	BASIS
PD11327	HERPUD1	cna	hetloss	BASIS
PD11742	HERPUD1	cna	hetloss	BASIS
PD13296	HERPUD1	cna	hetloss	BASIS
PD13297	HERPUD1	cna	hetloss	BASIS
PD13299	HERPUD1	cna	gain	BASIS
PD14442	HERPUD1	cna	hetloss	BASIS
PD22355	HERPUD1	cna	hetloss	BASIS

PD23562	HERPUD1	cna	gain	BASIS
PD24186	HERPUD1	cna	gain	BASIS
PD24202	HERPUD1	cna	hetloss	BASIS
PD24206	HERPUD1	cna	gain	BASIS
PD24337	HERPUD1	cna	hetloss	BASIS
PD3905	HERPUD1	cna	gain	BASIS
PD4005	HERPUD1	cna	hetloss	BASIS
PD4967	HERPUD1	cna	hetloss	BASIS
PD5945	HERPUD1	cna	amp	BASIS
PD5948	HERPUD1	cna	hetloss	BASIS
PD7067	HERPUD1	cna	gain	BASIS
PD7215	HERPUD1	cna	gain	BASIS
PD8980	HERPUD1	cna	gain	BASIS
PD9585	HERPUD1	cna	hetloss	BASIS
PD9702	HERPUD1	cna	gain	BASIS
TCGA-A2-A25B	IFNGR1	cna	hetloss	TCGA
TCGA-AN-A0XU	IFNGR1	cna	amp	TCGA
TCGA-AO-A0JL	IFNGR1	cna	hetloss	TCGA
TCGA-BH-A0AW	IFNGR1	cna	amp	TCGA
TCGA-BH-A0C0	IFNGR1	cna	gain	TCGA
TCGA-BH-A18R	IFNGR1	cna	amp	TCGA
TCGA-C8-A12L	IFNGR1	cna	gain	TCGA
TCGA-D8-A27M	IFNGR1	cna	hetloss	TCGA
MB-5070	IFNGR1	cna	amp	METABRIC
MB-6098	IFNGR1	cna	hetloss	METABRIC
MB-7032	IFNGR1	cna	hetloss	METABRIC
MB-7038	IFNGR1	cna	amp	METABRIC
MB-7048	IFNGR1	cna	hetloss	METABRIC
MB-0420	IFNGR1	cna	hetloss	METABRIC
PD11327	IFNGR1	cna	gain	BASIS
PD13299	IFNGR1	cna	gain	BASIS
PD13771	IFNGR1	cna	hetloss	BASIS
PD22355	IFNGR1	cna	gain	BASIS
PD23562	IFNGR1	cna	gain	BASIS
PD23574	IFNGR1	cna	gain	BASIS
PD24186	IFNGR1	cna	gain	BASIS
PD24202	IFNGR1	cna	gain	BASIS
PD24206	IFNGR1	cna	gain	BASIS
PD3890	IFNGR1	cna	gain	BASIS
PD3905	IFNGR1	cna	gain	BASIS
PD4006	IFNGR1	cna	hetloss	BASIS
PD4107	IFNGR1	cna	amp	BASIS
PD4826	IFNGR1	cna	gain	BASIS
PD5935	IFNGR1	cna	gain	BASIS
PD5945	IFNGR1	cna	amp	BASIS
PD6406	IFNGR1	cna	hetloss	BASIS
PD6731	IFNGR1	cna	hetloss	BASIS

PD7067	IFNGR1	cna	amp	BASIS
PD7215	IFNGR1	cna	gain	BASIS
PD8621	IFNGR1	cna	gain	BASIS
PD8980	IFNGR1	cna	gain	BASIS
PD9004	IFNGR1	cna	homdel	BASIS
PD9585	IFNGR1	cna	hetloss	BASIS
PD9702	IFNGR1	cna	gain	BASIS
TCGA-AN-A0XU	IRF8	cna	amp	TCGA
TCGA-AO-A0JL	IRF8	cna	gain	TCGA
TCGA-BH-A0AW	IRF8	cna	hetloss	TCGA
TCGA-BH-A0C0	IRF8	cna	hetloss	TCGA
TCGA-C8-A12L	IRF8	cna	hetloss	TCGA
TCGA-D8-A27M	IRF8	cna	gain	TCGA
TCGA-E2-A1L7	IRF8	cna	hetloss	TCGA
TCGA-E9-A1NC	IRF8	cna	hetloss	TCGA
TCGA-LL-A5YP	IRF8	cna	gain	TCGA
MB-0346	IRF8	cna	amp	METABRIC
MB-5107	IRF8	cna	hetloss	METABRIC
MB-5465	IRF8	cna	hetloss	METABRIC
MB-6271	IRF8	cna	hetloss	METABRIC
MB-7032	IRF8	cna	hetloss	METABRIC
MB-7038	IRF8	cna	amp	METABRIC
MB-7048	IRF8	cna	hetloss	METABRIC
MB-0420	IRF8	cna	hetloss	METABRIC
PD10014	IRF8	cna	hetloss	BASIS
PD11327	IRF8	cna	gain	BASIS
PD11742	IRF8	cna	hetloss	BASIS
PD13297	IRF8	cna	hetloss	BASIS
PD13299	IRF8	cna	gain	BASIS
PD14442	IRF8	cna	hetloss	BASIS
PD23562	IRF8	cna	gain	BASIS
PD23574	IRF8	cna	gain	BASIS
PD24202	IRF8	cna	hetloss	BASIS
PD3890	IRF8	cna	gain	BASIS
PD3905	IRF8	cna	gain	BASIS
PD4005	IRF8	cna	hetloss	BASIS
PD4006	IRF8	cna	hetloss	BASIS
PD4107	IRF8	cna	gain	BASIS
PD4967	IRF8	cna	hetloss	BASIS
PD5945	IRF8	cna	gain	BASIS
PD5948	IRF8	cna	gain	BASIS
PD7215	IRF8	cna	gain	BASIS
PD8621	IRF8	cna	gain	BASIS
PD9004	IRF8	cna	gain	BASIS
PD9585	IRF8	cna	hetloss	BASIS
PD9702	IRF8	cna	gain	BASIS
TCGA-AN-A0XU	LASP1	cna	hetloss	TCGA

TCGA-AO-A0JL	LASP1	cna	amp	TCGA
TCGA-BH-A0AW	LASP1	cna	amp	TCGA
TCGA-BH-A0C0	LASP1	cna	gain	TCGA
TCGA-BH-A18R	LASP1	cna	homdel	TCGA
TCGA-C8-A12L	LASP1	cna	hetloss	TCGA
TCGA-D8-A27M	LASP1	cna	hetloss	TCGA
TCGA-E2-A1L7	LASP1	cna	hetloss	TCGA
TCGA-E9-A1NC	LASP1	cna	hetloss	TCGA
MB-0346	LASP1	cna	hetloss	METABRIC
MB-2827	LASP1	cna	hetloss	METABRIC
MB-5070	LASP1	cna	gain	METABRIC
MB-5465	LASP1	cna	hetloss	METABRIC
MB-6060	LASP1	cna	amp	METABRIC
MB-6098	LASP1	cna	hetloss	METABRIC
MB-6271	LASP1	cna	hetloss	METABRIC
MB-7038	LASP1	cna	hetloss	METABRIC
MB-0420	LASP1	cna	hetloss	METABRIC
PD13297	LASP1	cna	hetloss	BASIS
PD13299	LASP1	cna	gain	BASIS
PD23561	LASP1	cna	amp	BASIS
PD23562	LASP1	cna	gain	BASIS
PD24186	LASP1	cna	gain	BASIS
PD24206	LASP1	cna	homdel	BASIS
PD24337	LASP1	cna	hetloss	BASIS
PD3890	LASP1	cna	hetloss	BASIS
PD3905	LASP1	cna	gain	BASIS
PD4005	LASP1	cna	hetloss	BASIS
PD4826	LASP1	cna	amp	BASIS
PD4967	LASP1	cna	hetloss	BASIS
PD5945	LASP1	cna	amp	BASIS
PD5948	LASP1	cna	gain	BASIS
PD6406	LASP1	cna	hetloss	BASIS
PD6731	LASP1	cna	hetloss	BASIS
PD7067	LASP1	cna	gain	BASIS
PD7215	LASP1	cna	gain	BASIS
PD9004	LASP1	cna	gain	BASIS
PD9585	LASP1	cna	hetloss	BASIS
PD9702	LASP1	cna	gain	BASIS
TCGA-AN-A0XU	LHFP	cna	hetloss	TCGA
TCGA-AO-A0JL	LHFP	cna	hetloss	TCGA
TCGA-BH-A0AW	LHFP	cna	hetloss	TCGA
TCGA-BH-A0C0	LHFP	cna	hetloss	TCGA
TCGA-BH-A1FU	LHFP	cna	hetloss	TCGA
TCGA-C8-A12L	LHFP	cna	hetloss	TCGA
TCGA-EW-A1OX	LHFP	cna	homdel	TCGA
TCGA-LL-A5YP	LHFP	cna	homdel	TCGA
MB-0346	LHFP	cna	hetloss	METABRIC

MB-2827	LHFP	cna	hetloss	METABRIC
MB-5107	LHFP	cna	hetloss	METABRIC
MB-6098	LHFP	cna	hetloss	METABRIC
MB-7032	LHFP	cna	hetloss	METABRIC
MB-7048	LHFP	cna	hetloss	METABRIC
MB-0420	LHFP	cna	hetloss	METABRIC
MTS-T0064	LHFP	cna	hetloss	METABRIC
PD10014	LHFP	cna	hetloss	BASIS
PD13296	LHFP	cna	hetloss	BASIS
PD13297	LHFP	cna	hetloss	BASIS
PD13771	LHFP	cna	hetloss	BASIS
PD14442	LHFP	cna	hetloss	BASIS
PD22355	LHFP	cna	hetloss	BASIS
PD23574	LHFP	cna	hetloss	BASIS
PD24202	LHFP	cna	hetloss	BASIS
PD24206	LHFP	cna	gain	BASIS
PD3890	LHFP	cna	hetloss	BASIS
PD3905	LHFP	cna	gain	BASIS
PD4005	LHFP	cna	homdel	BASIS
PD4006	LHFP	cna	hetloss	BASIS
PD4826	LHFP	cna	hetloss	BASIS
PD4967	LHFP	cna	hetloss	BASIS
PD5945	LHFP	cna	amp	BASIS
PD6406	LHFP	cna	hetloss	BASIS
PD6731	LHFP	cna	hetloss	BASIS
PD7215	LHFP	cna	gain	BASIS
PD8621	LHFP	cna	hetloss	BASIS
PD8980	LHFP	cna	hetloss	BASIS
PD9585	LHFP	cna	gain	BASIS
PD9702	LHFP	cna	gain	BASIS
TCGA-AN-A0XU	MAP3K1	cna	hetloss	TCGA
TCGA-AO-A0JL	MAP3K1	cna	hetloss	TCGA
TCGA-BH-A0C0	MAP3K1	cna	hetloss	TCGA
TCGA-BH-A1FU	MAP3K1	cna	hetloss	TCGA
TCGA-C8-A12L	MAP3K1	cna	hetloss	TCGA
TCGA-E2-A1L7	MAP3K1	cna	hetloss	TCGA
TCGA-E9-A1NC	MAP3K1	cna	hetloss	TCGA
TCGA-LL-A5YP	MAP3K1	cna	hetloss	TCGA
MB-0346	MAP3K1	cna	gain	METABRIC
MB-2827	MAP3K1	cna	hetloss	METABRIC
MB-5107	MAP3K1	cna	hetloss	METABRIC
MB-5465	MAP3K1	cna	hetloss	METABRIC
MB-6060	MAP3K1	cna	hetloss	METABRIC
MB-6098	MAP3K1	cna	gain	METABRIC
MB-0420	MAP3K1	cna	hetloss	METABRIC
PD10014	MAP3K1	cna	hetloss	BASIS
PD11327	MAP3K1	cna	hetloss	BASIS

PD11742	MAP3K1	cna	hetloss	BASIS
PD13296	MAP3K1	cna	hetloss	BASIS
PD13297	MAP3K1	cna	hetloss	BASIS
PD13771	MAP3K1	cna	hetloss	BASIS
PD23562	MAP3K1	cna	hetloss	BASIS
PD23578	MAP3K1	cna	hetloss	BASIS
PD24186	MAP3K1	cna	gain	BASIS
PD24202	MAP3K1	cna	hetloss	BASIS
PD24206	MAP3K1	cna	gain	BASIS
PD3890	MAP3K1	cna	hetloss	BASIS
PD3905	MAP3K1	cna	gain	BASIS
PD4005	MAP3K1	cna	hetloss	BASIS
PD4006	MAP3K1	cna	hetloss	BASIS
PD4107	MAP3K1	cna	hetloss	BASIS
PD5930	MAP3K1	cna	hetloss	BASIS
PD5935	MAP3K1	cna	hetloss	BASIS
PD5948	MAP3K1	cna	hetloss	BASIS
PD6413	MAP3K1	cna	hetloss	BASIS
PD7215	MAP3K1	cna	hetloss	BASIS
PD8621	MAP3K1	cna	hetloss	BASIS
PD8980	MAP3K1	cna	hetloss	BASIS
PD9004	MAP3K1	cna	gain	BASIS
TCGA-A2-A25B	MEF2A	cna	amp	TCGA
TCGA-AN-A0XU	MEF2A	cna	gain	TCGA
TCGA-AO-A0JL	MEF2A	cna	hetloss	TCGA
TCGA-BH-A0C0	MEF2A	cna	gain	TCGA
TCGA-D8-A27M	MEF2A	cna	hetloss	TCGA
TCGA-E2-A1L7	MEF2A	cna	gain	TCGA
TCGA-LL-A5YP	MEF2A	cna	gain	TCGA
MB-0346	MEF2A	cna	hetloss	METABRIC
MB-2827	MEF2A	cna	amp	METABRIC
MB-5070	MEF2A	cna	gain	METABRIC
MB-5465	MEF2A	cna	hetloss	METABRIC
MB-6060	MEF2A	cna	hetloss	METABRIC
MB-7038	MEF2A	cna	gain	METABRIC
MB-7048	MEF2A	cna	gain	METABRIC
MB-0420	MEF2A	cna	gain	METABRIC
MTS-T0064	MEF2A	cna	gain	METABRIC
PD10014	MEF2A	cna	gain	BASIS
PD11327	MEF2A	cna	gain	BASIS
PD11742	MEF2A	cna	hetloss	BASIS
PD13296	MEF2A	cna	gain	BASIS
PD13299	MEF2A	cna	amp	BASIS
PD23562	MEF2A	cna	gain	BASIS
PD23574	MEF2A	cna	gain	BASIS
PD23578	MEF2A	cna	gain	BASIS
PD24202	MEF2A	cna	hetloss	BASIS

PD24206	MEF2A	cna	gain	BASIS
PD24337	MEF2A	cna	gain	BASIS
PD3890	MEF2A	cna	hetloss	BASIS
PD3905	MEF2A	cna	gain	BASIS
PD4006	MEF2A	cna	amp	BASIS
PD4107	MEF2A	cna	gain	BASIS
PD5930	MEF2A	cna	gain	BASIS
PD5945	MEF2A	cna	amp	BASIS
PD5948	MEF2A	cna	gain	BASIS
PD6413	MEF2A	cna	hetloss	BASIS
PD7067	MEF2A	cna	gain	BASIS
PD8621	MEF2A	cna	gain	BASIS
PD8980	MEF2A	cna	gain	BASIS
PD9585	MEF2A	cna	hetloss	BASIS
TCGA-AN-A0XU	MLLT6	cna	hetloss	TCGA
TCGA-AO-A0JL	MLLT6	cna	amp	TCGA
TCGA-BH-A0AW	MLLT6	cna	amp	TCGA
TCGA-BH-A0C0	MLLT6	cna	gain	TCGA
TCGA-BH-A18R	MLLT6	cna	homdel	TCGA
TCGA-C8-A12L	MLLT6	cna	hetloss	TCGA
TCGA-D8-A27M	MLLT6	cna	hetloss	TCGA
TCGA-E2-A1L7	MLLT6	cna	hetloss	TCGA
TCGA-E9-A1NC	MLLT6	cna	hetloss	TCGA
MB-0346	MLLT6	cna	hetloss	METABRIC
MB-2827	MLLT6	cna	hetloss	METABRIC
MB-5070	MLLT6	cna	gain	METABRIC
MB-5465	MLLT6	cna	hetloss	METABRIC
MB-6060	MLLT6	cna	amp	METABRIC
MB-6098	MLLT6	cna	hetloss	METABRIC
MB-6271	MLLT6	cna	hetloss	METABRIC
MB-7038	MLLT6	cna	hetloss	METABRIC
MB-0420	MLLT6	cna	hetloss	METABRIC
PD13297	MLLT6	cna	hetloss	BASIS
PD13299	MLLT6	cna	gain	BASIS
PD23561	MLLT6	cna	amp	BASIS
PD23562	MLLT6	cna	gain	BASIS
PD24186	MLLT6	cna	gain	BASIS
PD24206	MLLT6	cna	hetloss	BASIS
PD24337	MLLT6	cna	hetloss	BASIS
PD3890	MLLT6	cna	hetloss	BASIS
PD3905	MLLT6	cna	gain	BASIS
PD4005	MLLT6	cna	hetloss	BASIS
PD4826	MLLT6	cna	amp	BASIS
PD4967	MLLT6	cna	hetloss	BASIS
PD5945	MLLT6	cna	amp	BASIS
PD5948	MLLT6	cna	gain	BASIS
PD6406	MLLT6	cna	hetloss	BASIS

PD6731	MLLT6	cna	hetloss	BASIS
PD7067	MLLT6	cna	gain	BASIS
PD7215	MLLT6	cna	gain	BASIS
PD9004	MLLT6	cna	gain	BASIS
PD9585	MLLT6	cna	hetloss	BASIS
PD9702	MLLT6	cna	gain	BASIS
TCGA-AN-A0XU	MLST8	cna	hetloss	TCGA
TCGA-AO-A0JL	MLST8	cna	gain	TCGA
TCGA-BH-A0AW	MLST8	cna	gain	TCGA
TCGA-BH-A0C0	MLST8	cna	gain	TCGA
TCGA-C8-A12L	MLST8	cna	gain	TCGA
TCGA-E2-A1L7	MLST8	cna	gain	TCGA
TCGA-E9-A1NC	MLST8	cna	hetloss	TCGA
TCGA-LL-A5YP	MLST8	cna	gain	TCGA
MB-0346	MLST8	cna	hetloss	METABRIC
MB-5465	MLST8	cna	hetloss	METABRIC
MB-6271	MLST8	cna	gain	METABRIC
MB-7032	MLST8	cna	gain	METABRIC
PD10014	MLST8	cna	gain	BASIS
PD11327	MLST8	cna	gain	BASIS
PD11742	MLST8	cna	gain	BASIS
PD13296	MLST8	cna	gain	BASIS
PD13297	MLST8	cna	hetloss	BASIS
PD13299	MLST8	cna	gain	BASIS
PD22355	MLST8	cna	hetloss	BASIS
PD23578	MLST8	cna	hetloss	BASIS
PD24202	MLST8	cna	hetloss	BASIS
PD24337	MLST8	cna	hetloss	BASIS
PD3905	MLST8	cna	gain	BASIS
PD4005	MLST8	cna	gain	BASIS
PD4006	MLST8	cna	gain	BASIS
PD4107	MLST8	cna	gain	BASIS
PD4826	MLST8	cna	gain	BASIS
PD4967	MLST8	cna	gain	BASIS
PD5930	MLST8	cna	gain	BASIS
PD5935	MLST8	cna	gain	BASIS
PD5945	MLST8	cna	gain	BASIS
PD5948	MLST8	cna	gain	BASIS
PD6413	MLST8	cna	gain	BASIS
PD7067	MLST8	cna	gain	BASIS
PD7215	MLST8	cna	amp	BASIS
PD8621	MLST8	cna	gain	BASIS
PD9004	MLST8	cna	gain	BASIS
PD9585	MLST8	cna	hetloss	BASIS
PD9702	MLST8	cna	gain	BASIS
TCGA-A2-A25B	NFKBIA	cna	amp	TCGA
TCGA-AN-A0XU	NFKBIA	cna	hetloss	TCGA

TCGA-AO-A0JL	NFKBIA	cna	hetloss	TCGA
TCGA-BH-A0AW	NFKBIA	cna	hetloss	TCGA
TCGA-BH-A1FU	NFKBIA	cna	gain	TCGA
TCGA-D8-A27M	NFKBIA	cna	hetloss	TCGA
TCGA-LL-A5YP	NFKBIA	cna	hetloss	TCGA
MB-0346	NFKBIA	cna	gain	METABRIC
MB-2827	NFKBIA	cna	hetloss	METABRIC
MB-5070	NFKBIA	cna	hetloss	METABRIC
MB-5465	NFKBIA	cna	hetloss	METABRIC
MB-6060	NFKBIA	cna	gain	METABRIC
MB-7038	NFKBIA	cna	gain	METABRIC
PD10014	NFKBIA	cna	hetloss	BASIS
PD11327	NFKBIA	cna	homdel	BASIS
PD22355	NFKBIA	cna	hetloss	BASIS
PD23578	NFKBIA	cna	hetloss	BASIS
PD24186	NFKBIA	cna	gain	BASIS
PD24202	NFKBIA	cna	hetloss	BASIS
PD24206	NFKBIA	cna	amp	BASIS
PD24337	NFKBIA	cna	hetloss	BASIS
PD3890	NFKBIA	cna	hetloss	BASIS
PD3905	NFKBIA	cna	gain	BASIS
PD4005	NFKBIA	cna	hetloss	BASIS
PD4006	NFKBIA	cna	hetloss	BASIS
PD4107	NFKBIA	cna	hetloss	BASIS
PD4826	NFKBIA	cna	gain	BASIS
PD4967	NFKBIA	cna	hetloss	BASIS
PD5930	NFKBIA	cna	hetloss	BASIS
PD5945	NFKBIA	cna	gain	BASIS
PD5948	NFKBIA	cna	gain	BASIS
PD6406	NFKBIA	cna	hetloss	BASIS
PD7067	NFKBIA	cna	gain	BASIS
PD7215	NFKBIA	cna	gain	BASIS
PD8621	NFKBIA	cna	gain	BASIS
PD8980	NFKBIA	cna	gain	BASIS
PD9004	NFKBIA	cna	gain	BASIS
PD9585	NFKBIA	cna	gain	BASIS
PD9702	NFKBIA	cna	gain	BASIS
TCGA-A2-A25B	NIN	cna	hetloss	TCGA
TCGA-AN-A0XU	NIN	cna	gain	TCGA
TCGA-AO-A0JL	NIN	cna	hetloss	TCGA
TCGA-BH-A0AW	NIN	cna	hetloss	TCGA
TCGA-BH-A1FU	NIN	cna	gain	TCGA
TCGA-C8-A12L	NIN	cna	hetloss	TCGA
TCGA-D8-A27M	NIN	cna	hetloss	TCGA
TCGA-LL-A5YP	NIN	cna	hetloss	TCGA
MB-2827	NIN	cna	hetloss	METABRIC
MB-5070	NIN	cna	hetloss	METABRIC

MB-5465	NIN	cna	hetloss	METABRIC
MB-6060	NIN	cna	gain	METABRIC
PD10014	NIN	cna	hetloss	BASIS
PD11327	NIN	cna	homdel	BASIS
PD13296	NIN	cna	hetloss	BASIS
PD13297	NIN	cna	hetloss	BASIS
PD13299	NIN	cna	hetloss	BASIS
PD22355	NIN	cna	hetloss	BASIS
PD23578	NIN	cna	hetloss	BASIS
PD24186	NIN	cna	gain	BASIS
PD24202	NIN	cna	hetloss	BASIS
PD24206	NIN	cna	gain	BASIS
PD24337	NIN	cna	hetloss	BASIS
PD3890	NIN	cna	hetloss	BASIS
PD3905	NIN	cna	gain	BASIS
PD4005	NIN	cna	hetloss	BASIS
PD4006	NIN	cna	hetloss	BASIS
PD4107	NIN	cna	hetloss	BASIS
PD4967	NIN	cna	hetloss	BASIS
PD5930	NIN	cna	hetloss	BASIS
PD5945	NIN	cna	gain	BASIS
PD6406	NIN	cna	hetloss	BASIS
PD7067	NIN	cna	gain	BASIS
PD7215	NIN	cna	gain	BASIS
PD8621	NIN	cna	hetloss	BASIS
PD8980	NIN	cna	gain	BASIS
PD9004	NIN	cna	gain	BASIS
PD9585	NIN	cna	gain	BASIS
PD9702	NIN	cna	gain	BASIS
TCGA-A2-A25B	OTX2	cna	hetloss	TCGA
TCGA-AO-A0JL	OTX2	cna	hetloss	TCGA
TCGA-BH-A0AW	OTX2	cna	hetloss	TCGA
TCGA-BH-A1FU	OTX2	cna	gain	TCGA
TCGA-C8-A12L	OTX2	cna	hetloss	TCGA
TCGA-D8-A27M	OTX2	cna	hetloss	TCGA
TCGA-LL-A5YP	OTX2	cna	hetloss	TCGA
MB-2827	OTX2	cna	hetloss	METABRIC
MB-5070	OTX2	cna	hetloss	METABRIC
MB-5465	OTX2	cna	hetloss	METABRIC
MB-6060	OTX2	cna	gain	METABRIC
MB-6098	OTX2	cna	hetloss	METABRIC
MB-7038	OTX2	cna	hetloss	METABRIC
PD10014	OTX2	cna	hetloss	BASIS
PD11327	OTX2	cna	gain	BASIS
PD13296	OTX2	cna	hetloss	BASIS
PD13297	OTX2	cna	hetloss	BASIS
PD22355	OTX2	cna	hetloss	BASIS

PD23574	OTX2	cna	hetloss	BASIS
PD23578	OTX2	cna	hetloss	BASIS
PD24202	OTX2	cna	hetloss	BASIS
PD24206	OTX2	cna	hetloss	BASIS
PD24337	OTX2	cna	hetloss	BASIS
PD3890	OTX2	cna	hetloss	BASIS
PD3905	OTX2	cna	gain	BASIS
PD4005	OTX2	cna	hetloss	BASIS
PD4006	OTX2	cna	hetloss	BASIS
PD4107	OTX2	cna	hetloss	BASIS
PD5930	OTX2	cna	hetloss	BASIS
PD5945	OTX2	cna	gain	BASIS
PD5948	OTX2	cna	hetloss	BASIS
PD6406	OTX2	cna	hetloss	BASIS
PD7067	OTX2	cna	gain	BASIS
PD7215	OTX2	cna	hetloss	BASIS
PD8621	OTX2	cna	hetloss	BASIS
PD8980	OTX2	cna	gain	BASIS
PD9004	OTX2	cna	gain	BASIS
PD9585	OTX2	cna	gain	BASIS
PD9702	OTX2	cna	gain	BASIS
TCGA-A2-A25B	PAFAH1B2	cna	hetloss	TCGA
TCGA-AN-A0XU	PAFAH1B2	cna	gain	TCGA
TCGA-AO-A0JL	PAFAH1B2	cna	hetloss	TCGA
TCGA-BH-A0C0	PAFAH1B2	cna	hetloss	TCGA
TCGA-BH-A18R	PAFAH1B2	cna	homdel	TCGA
TCGA-D8-A27M	PAFAH1B2	cna	hetloss	TCGA
TCGA-E2-A1L7	PAFAH1B2	cna	hetloss	TCGA
TCGA-E9-A1NC	PAFAH1B2	cna	hetloss	TCGA
TCGA-EW-A1OX	PAFAH1B2	cna	homdel	TCGA
TCGA-LL-A5YP	PAFAH1B2	cna	hetloss	TCGA
MB-0346	PAFAH1B2	cna	hetloss	METABRIC
MB-5070	PAFAH1B2	cna	hetloss	METABRIC
MB-5107	PAFAH1B2	cna	hetloss	METABRIC
MB-6271	PAFAH1B2	cna	hetloss	METABRIC
MB-7032	PAFAH1B2	cna	hetloss	METABRIC
MB-7048	PAFAH1B2	cna	hetloss	METABRIC
PD11327	PAFAH1B2	cna	hetloss	BASIS
PD11742	PAFAH1B2	cna	hetloss	BASIS
PD13296	PAFAH1B2	cna	hetloss	BASIS
PD13297	PAFAH1B2	cna	hetloss	BASIS
PD13771	PAFAH1B2	cna	hetloss	BASIS
PD22355	PAFAH1B2	cna	hetloss	BASIS
PD23574	PAFAH1B2	cna	gain	BASIS
PD23578	PAFAH1B2	cna	hetloss	BASIS
PD24186	PAFAH1B2	cna	gain	BASIS
PD24206	PAFAH1B2	cna	gain	BASIS

PD24337	PAFAH1B2	cna	hetloss	BASIS
PD3905	PAFAH1B2	cna	gain	BASIS
PD4005	PAFAH1B2	cna	hetloss	BASIS
PD4826	PAFAH1B2	cna	hetloss	BASIS
PD5935	PAFAH1B2	cna	gain	BASIS
PD5945	PAFAH1B2	cna	gain	BASIS
PD5948	PAFAH1B2	cna	gain	BASIS
PD6413	PAFAH1B2	cna	hetloss	BASIS
PD6731	PAFAH1B2	cna	hetloss	BASIS
PD7067	PAFAH1B2	cna	gain	BASIS
PD7215	PAFAH1B2	cna	gain	BASIS
PD9004	PAFAH1B2	cna	hetloss	BASIS
PD9702	PAFAH1B2	cna	gain	BASIS
TCGA-A2-A25B	PAK7	cna	gain	TCGA
TCGA-AO-A0JL	PAK7	cna	gain	TCGA
TCGA-BH-A0AW	PAK7	cna	gain	TCGA
TCGA-BH-A0C0	PAK7	cna	hetloss	TCGA
TCGA-BH-A18R	PAK7	cna	homdel	TCGA
TCGA-C8-A12L	PAK7	cna	gain	TCGA
TCGA-D8-A27M	PAK7	cna	gain	TCGA
TCGA-E2-A1L7	PAK7	cna	gain	TCGA
TCGA-LL-A5YP	PAK7	cna	hetloss	TCGA
MB-0346	PAK7	cna	gain	METABRIC
MB-5070	PAK7	cna	amp	METABRIC
MB-6060	PAK7	cna	amp	METABRIC
MB-6098	PAK7	cna	hetloss	METABRIC
MB-7032	PAK7	cna	gain	METABRIC
MB-7048	PAK7	cna	gain	METABRIC
PD11327	PAK7	cna	gain	BASIS
PD13296	PAK7	cna	hetloss	BASIS
PD13299	PAK7	cna	gain	BASIS
PD23574	PAK7	cna	gain	BASIS
PD23578	PAK7	cna	hetloss	BASIS
PD24186	PAK7	cna	amp	BASIS
PD24202	PAK7	cna	hetloss	BASIS
PD24337	PAK7	cna	amp	BASIS
PD3905	PAK7	cna	gain	BASIS
PD4005	PAK7	cna	hetloss	BASIS
PD4006	PAK7	cna	hetloss	BASIS
PD4107	PAK7	cna	gain	BASIS
PD4826	PAK7	cna	hetloss	BASIS
PD5930	PAK7	cna	gain	BASIS
PD5935	PAK7	cna	gain	BASIS
PD5945	PAK7	cna	amp	BASIS
PD5948	PAK7	cna	gain	BASIS
PD7067	PAK7	cna	hetloss	BASIS
PD7215	PAK7	cna	gain	BASIS

PD8621	PAK7	cna	gain	BASIS
PD8980	PAK7	cna	hetloss	BASIS
PD9004	PAK7	cna	gain	BASIS
PD9585	PAK7	cna	gain	BASIS
PD9702	PAK7	cna	gain	BASIS
TCGA-A2-A25B	PCSK7	cna	hetloss	TCGA
TCGA-AN-A0XU	PCSK7	cna	gain	TCGA
TCGA-AO-A0JL	PCSK7	cna	hetloss	TCGA
TCGA-BH-A0C0	PCSK7	cna	hetloss	TCGA
TCGA-BH-A18R	PCSK7	cna	homdel	TCGA
TCGA-D8-A27M	PCSK7	cna	hetloss	TCGA
TCGA-E2-A1L7	PCSK7	cna	hetloss	TCGA
TCGA-E9-A1NC	PCSK7	cna	hetloss	TCGA
TCGA-EW-A1OX	PCSK7	cna	homdel	TCGA
TCGA-LL-A5YP	PCSK7	cna	hetloss	TCGA
MB-0346	PCSK7	cna	hetloss	METABRIC
MB-5070	PCSK7	cna	hetloss	METABRIC
MB-5107	PCSK7	cna	hetloss	METABRIC
MB-6271	PCSK7	cna	hetloss	METABRIC
MB-7032	PCSK7	cna	hetloss	METABRIC
MB-7048	PCSK7	cna	hetloss	METABRIC
PD11327	PCSK7	cna	hetloss	BASIS
PD11742	PCSK7	cna	hetloss	BASIS
PD13296	PCSK7	cna	hetloss	BASIS
PD13297	PCSK7	cna	hetloss	BASIS
PD13771	PCSK7	cna	hetloss	BASIS
PD22355	PCSK7	cna	hetloss	BASIS
PD23574	PCSK7	cna	gain	BASIS
PD23578	PCSK7	cna	hetloss	BASIS
PD24186	PCSK7	cna	gain	BASIS
PD24206	PCSK7	cna	gain	BASIS
PD24337	PCSK7	cna	hetloss	BASIS
PD3905	PCSK7	cna	gain	BASIS
PD4005	PCSK7	cna	hetloss	BASIS
PD4826	PCSK7	cna	hetloss	BASIS
PD5935	PCSK7	cna	gain	BASIS
PD5945	PCSK7	cna	gain	BASIS
PD5948	PCSK7	cna	gain	BASIS
PD6413	PCSK7	cna	hetloss	BASIS
PD6731	PCSK7	cna	hetloss	BASIS
PD7067	PCSK7	cna	gain	BASIS
PD7215	PCSK7	cna	gain	BASIS
PD9004	PCSK7	cna	hetloss	BASIS
PD9702	PCSK7	cna	gain	BASIS
TCGA-A2-A25B	PDCD11	cna	hetloss	TCGA
TCGA-AN-A0XU	PDCD11	cna	hetloss	TCGA
TCGA-AO-A0JL	PDCD11	cna	hetloss	TCGA

TCGA-BH-A18R	PDCD11	cna	gain	TCGA
TCGA-C8-A12L	PDCD11	cna	hetloss	TCGA
TCGA-D8-A27M	PDCD11	cna	hetloss	TCGA
TCGA-E2-A1L7	PDCD11	cna	hetloss	TCGA
TCGA-LL-A5YP	PDCD11	cna	hetloss	TCGA
MB-0346	PDCD11	cna	gain	METABRIC
MB-2827	PDCD11	cna	hetloss	METABRIC
MB-5070	PDCD11	cna	hetloss	METABRIC
MB-6098	PDCD11	cna	hetloss	METABRIC
MB-7038	PDCD11	cna	hetloss	METABRIC
MB-7048	PDCD11	cna	hetloss	METABRIC
MB-0420	PDCD11	cna	hetloss	METABRIC
PD10014	PDCD11	cna	hetloss	BASIS
PD11327	PDCD11	cna	homdel	BASIS
PD11742	PDCD11	cna	hetloss	BASIS
PD22355	PDCD11	cna	hetloss	BASIS
PD23562	PDCD11	cna	gain	BASIS
PD23574	PDCD11	cna	gain	BASIS
PD23578	PDCD11	cna	hetloss	BASIS
PD24206	PDCD11	cna	homdel	BASIS
PD3890	PDCD11	cna	hetloss	BASIS
PD3905	PDCD11	cna	gain	BASIS
PD4005	PDCD11	cna	hetloss	BASIS
PD4006	PDCD11	cna	gain	BASIS
PD4107	PDCD11	cna	gain	BASIS
PD5935	PDCD11	cna	hetloss	BASIS
PD5945	PDCD11	cna	gain	BASIS
PD5948	PDCD11	cna	gain	BASIS
PD6406	PDCD11	cna	hetloss	BASIS
PD6731	PDCD11	cna	hetloss	BASIS
PD7067	PDCD11	cna	gain	BASIS
PD7215	PDCD11	cna	gain	BASIS
PD8621	PDCD11	cna	gain	BASIS
PD8980	PDCD11	cna	hetloss	BASIS
PD9585	PDCD11	cna	hetloss	BASIS
PD9702	PDCD11	cna	gain	BASIS
TCGA-AN-A0XU	PLK2	cna	hetloss	TCGA
TCGA-AO-A0JL	PLK2	cna	hetloss	TCGA
TCGA-BH-A0C0	PLK2	cna	hetloss	TCGA
TCGA-BH-A1FU	PLK2	cna	hetloss	TCGA
TCGA-C8-A12L	PLK2	cna	hetloss	TCGA
TCGA-E2-A1L7	PLK2	cna	hetloss	TCGA
TCGA-E9-A1NC	PLK2	cna	hetloss	TCGA
TCGA-LL-A5YP	PLK2	cna	hetloss	TCGA
MB-0346	PLK2	cna	gain	METABRIC
MB-2827	PLK2	cna	hetloss	METABRIC
MB-5107	PLK2	cna	hetloss	METABRIC

MB-5465	PLK2	cna	hetloss	METABRIC
MB-6098	PLK2	cna	gain	METABRIC
MB-7038	PLK2	cna	hetloss	METABRIC
MB-0420	PLK2	cna	hetloss	METABRIC
PD10014	PLK2	cna	hetloss	BASIS
PD11742	PLK2	cna	hetloss	BASIS
PD13296	PLK2	cna	hetloss	BASIS
PD13297	PLK2	cna	hetloss	BASIS
PD13771	PLK2	cna	hetloss	BASIS
PD23562	PLK2	cna	hetloss	BASIS
PD23578	PLK2	cna	hetloss	BASIS
PD24186	PLK2	cna	gain	BASIS
PD24202	PLK2	cna	hetloss	BASIS
PD24206	PLK2	cna	gain	BASIS
PD24337	PLK2	cna	hetloss	BASIS
PD3890	PLK2	cna	hetloss	BASIS
PD3905	PLK2	cna	gain	BASIS
PD4005	PLK2	cna	hetloss	BASIS
PD4006	PLK2	cna	hetloss	BASIS
PD4107	PLK2	cna	hetloss	BASIS
PD5930	PLK2	cna	hetloss	BASIS
PD5935	PLK2	cna	hetloss	BASIS
PD5948	PLK2	cna	homdel	BASIS
PD6413	PLK2	cna	hetloss	BASIS
PD7215	PLK2	cna	hetloss	BASIS
PD8621	PLK2	cna	hetloss	BASIS
PD8980	PLK2	cna	hetloss	BASIS
PD9004	PLK2	cna	gain	BASIS
TCGA-A2-A25B	PRDM16	cna	hetloss	TCGA
TCGA-AN-A0XU	PRDM16	cna	hetloss	TCGA
TCGA-AO-A0JL	PRDM16	cna	hetloss	TCGA
TCGA-BH-A0AW	PRDM16	cna	gain	TCGA
TCGA-BH-A0C0	PRDM16	cna	gain	TCGA
TCGA-BH-A18R	PRDM16	cna	homdel	TCGA
TCGA-BH-A1FU	PRDM16	cna	gain	TCGA
TCGA-C8-A12L	PRDM16	cna	hetloss	TCGA
TCGA-D8-A27M	PRDM16	cna	gain	TCGA
TCGA-E2-A1L7	PRDM16	cna	hetloss	TCGA
TCGA-E9-A1NC	PRDM16	cna	hetloss	TCGA
TCGA-LL-A5YP	PRDM16	cna	amp	TCGA
MB-2827	PRDM16	cna	hetloss	METABRIC
MB-5070	PRDM16	cna	hetloss	METABRIC
MB-5465	PRDM16	cna	hetloss	METABRIC
MB-6098	PRDM16	cna	hetloss	METABRIC
MB-0420	PRDM16	cna	hetloss	METABRIC
PD10014	PRDM16	cna	gain	BASIS
PD11327	PRDM16	cna	gain	BASIS

PD11742	PRDM16	cna	hetloss	BASIS
PD13297	PRDM16	cna	gain	BASIS
PD22355	PRDM16	cna	hetloss	BASIS
PD23578	PRDM16	cna	hetloss	BASIS
PD24186	PRDM16	cna	gain	BASIS
PD24202	PRDM16	cna	hetloss	BASIS
PD24206	PRDM16	cna	amp	BASIS
PD24337	PRDM16	cna	hetloss	BASIS
PD3890	PRDM16	cna	hetloss	BASIS
PD3905	PRDM16	cna	gain	BASIS
PD4005	PRDM16	cna	gain	BASIS
PD4107	PRDM16	cna	gain	BASIS
PD4826	PRDM16	cna	gain	BASIS
PD5945	PRDM16	cna	gain	BASIS
PD6406	PRDM16	cna	hetloss	BASIS
PD7067	PRDM16	cna	gain	BASIS
PD7215	PRDM16	cna	gain	BASIS
PD8621	PRDM16	cna	gain	BASIS
PD8980	PRDM16	cna	hetloss	BASIS
PD9004	PRDM16	cna	hetloss	BASIS
TCGA-A2-A25B	ROS1	cna	hetloss	TCGA
TCGA-AN-A0XU	ROS1	cna	amp	TCGA
TCGA-AO-A0JL	ROS1	cna	hetloss	TCGA
TCGA-BH-A0AW	ROS1	cna	amp	TCGA
TCGA-BH-A18R	ROS1	cna	homdel	TCGA
TCGA-C8-A12L	ROS1	cna	gain	TCGA
TCGA-D8-A27M	ROS1	cna	hetloss	TCGA
MB-2827	ROS1	cna	amp	METABRIC
MB-5070	ROS1	cna	gain	METABRIC
MB-6098	ROS1	cna	hetloss	METABRIC
MB-7032	ROS1	cna	hetloss	METABRIC
MB-7038	ROS1	cna	gain	METABRIC
MB-7048	ROS1	cna	hetloss	METABRIC
MTS-T0064	ROS1	cna	amp	METABRIC
PD11327	ROS1	cna	gain	BASIS
PD13299	ROS1	cna	gain	BASIS
PD13771	ROS1	cna	hetloss	BASIS
PD22355	ROS1	cna	gain	BASIS
PD23562	ROS1	cna	gain	BASIS
PD23574	ROS1	cna	gain	BASIS
PD24186	ROS1	cna	gain	BASIS
PD24206	ROS1	cna	gain	BASIS
PD24337	ROS1	cna	hetloss	BASIS
PD3905	ROS1	cna	gain	BASIS
PD4006	ROS1	cna	gain	BASIS
PD4107	ROS1	cna	hetloss	BASIS
PD4826	ROS1	cna	gain	BASIS

PD5930	ROS1	cna	hetloss	BASIS
PD5935	ROS1	cna	gain	BASIS
PD5945	ROS1	cna	amp	BASIS
PD6406	ROS1	cna	hetloss	BASIS
PD6731	ROS1	cna	hetloss	BASIS
PD7067	ROS1	cna	gain	BASIS
PD7215	ROS1	cna	gain	BASIS
PD8621	ROS1	cna	gain	BASIS
PD8980	ROS1	cna	gain	BASIS
PD9004	ROS1	cna	gain	BASIS
PD9585	ROS1	cna	hetloss	BASIS
PD9702	ROS1	cna	gain	BASIS
TCGA-AN-A0XU	RPL13	cna	gain	TCGA
TCGA-AO-A0JL	RPL13	cna	gain	TCGA
TCGA-BH-A0AW	RPL13	cna	hetloss	TCGA
TCGA-BH-A0C0	RPL13	cna	gain	TCGA
TCGA-C8-A12L	RPL13	cna	hetloss	TCGA
TCGA-E2-A1L7	RPL13	cna	hetloss	TCGA
TCGA-E9-A1NC	RPL13	cna	hetloss	TCGA
TCGA-LL-A5YP	RPL13	cna	gain	TCGA
MB-0346	RPL13	cna	gain	METABRIC
MB-5107	RPL13	cna	hetloss	METABRIC
MB-6271	RPL13	cna	hetloss	METABRIC
MB-7032	RPL13	cna	hetloss	METABRIC
MB-7038	RPL13	cna	gain	METABRIC
MB-7048	RPL13	cna	hetloss	METABRIC
MB-0420	RPL13	cna	hetloss	METABRIC
PD10014	RPL13	cna	hetloss	BASIS
PD11327	RPL13	cna	gain	BASIS
PD11742	RPL13	cna	hetloss	BASIS
PD13296	RPL13	cna	hetloss	BASIS
PD13297	RPL13	cna	hetloss	BASIS
PD13299	RPL13	cna	gain	BASIS
PD14442	RPL13	cna	hetloss	BASIS
PD23562	RPL13	cna	gain	BASIS
PD23574	RPL13	cna	gain	BASIS
PD24202	RPL13	cna	hetloss	BASIS
PD3890	RPL13	cna	gain	BASIS
PD3905	RPL13	cna	gain	BASIS
PD4005	RPL13	cna	hetloss	BASIS
PD4006	RPL13	cna	gain	BASIS
PD4107	RPL13	cna	amp	BASIS
PD4967	RPL13	cna	hetloss	BASIS
PD5930	RPL13	cna	hetloss	BASIS
PD5945	RPL13	cna	amp	BASIS
PD5948	RPL13	cna	gain	BASIS
PD7215	RPL13	cna	gain	BASIS

PD8621	RPL13	cna	gain	BASIS
PD9004	RPL13	cna	gain	BASIS
PD9585	RPL13	cna	hetloss	BASIS
PD9702	RPL13	cna	gain	BASIS
TCGA-A2-A25B	SH2B3	cna	gain	TCGA
TCGA-AO-A0JL	SH2B3	cna	hetloss	TCGA
TCGA-BH-A0C0	SH2B3	cna	gain	TCGA
TCGA-BH-A18R	SH2B3	cna	gain	TCGA
TCGA-BH-A1FU	SH2B3	cna	hetloss	TCGA
TCGA-C8-A12L	SH2B3	cna	gain	TCGA
TCGA-D8-A27M	SH2B3	cna	hetloss	TCGA
TCGA-E2-A1L7	SH2B3	cna	gain	TCGA
TCGA-LL-A5YP	SH2B3	cna	hetloss	TCGA
MB-0346	SH2B3	cna	gain	METABRIC
MB-2827	SH2B3	cna	hetloss	METABRIC
MB-5070	SH2B3	cna	hetloss	METABRIC
MB-5465	SH2B3	cna	hetloss	METABRIC
MB-6098	SH2B3	cna	hetloss	METABRIC
MB-7048	SH2B3	cna	hetloss	METABRIC
PD10014	SH2B3	cna	gain	BASIS
PD11327	SH2B3	cna	hetloss	BASIS
PD11742	SH2B3	cna	hetloss	BASIS
PD13296	SH2B3	cna	hetloss	BASIS
PD13297	SH2B3	cna	gain	BASIS
PD22355	SH2B3	cna	hetloss	BASIS
PD23574	SH2B3	cna	gain	BASIS
PD24337	SH2B3	cna	gain	BASIS
PD3890	SH2B3	cna	hetloss	BASIS
PD3905	SH2B3	cna	gain	BASIS
PD4005	SH2B3	cna	hetloss	BASIS
PD4006	SH2B3	cna	gain	BASIS
PD4107	SH2B3	cna	gain	BASIS
PD4826	SH2B3	cna	gain	BASIS
PD4967	SH2B3	cna	hetloss	BASIS
PD5945	SH2B3	cna	gain	BASIS
PD5948	SH2B3	cna	gain	BASIS
PD6406	SH2B3	cna	hetloss	BASIS
PD6413	SH2B3	cna	hetloss	BASIS
PD6731	SH2B3	cna	hetloss	BASIS
PD7067	SH2B3	cna	gain	BASIS
PD9004	SH2B3	cna	hetloss	BASIS
PD9585	SH2B3	cna	hetloss	BASIS
PD9702	SH2B3	cna	gain	BASIS
TCGA-A2-A25B	SMARCA1	cna	hetloss	TCGA
TCGA-AN-A0XU	SMARCA1	cna	hetloss	TCGA
TCGA-BH-A0AW	SMARCA1	cna	gain	TCGA
TCGA-C8-A12L	SMARCA1	cna	gain	TCGA

TCGA-E2-A1L7	SMARCA1	cna	hetloss	TCGA
TCGA-E9-A1NC	SMARCA1	cna	hetloss	TCGA
TCGA-LL-A5YP	SMARCA1	cna	gain	TCGA
MB-0346	SMARCA1	cna	hetloss	METABRIC
MB-2827	SMARCA1	cna	hetloss	METABRIC
MB-5465	SMARCA1	cna	hetloss	METABRIC
MB-6060	SMARCA1	cna	gain	METABRIC
MB-6098	SMARCA1	cna	hetloss	METABRIC
MB-7038	SMARCA1	cna	gain	METABRIC
MB-0420	SMARCA1	cna	hetloss	METABRIC
PD10014	SMARCA1	cna	hetloss	BASIS
PD13296	SMARCA1	cna	gain	BASIS
PD13299	SMARCA1	cna	amp	BASIS
PD13771	SMARCA1	cna	gain	BASIS
PD14442	SMARCA1	cna	gain	BASIS
PD23562	SMARCA1	cna	hetloss	BASIS
PD23574	SMARCA1	cna	gain	BASIS
PD23578	SMARCA1	cna	hetloss	BASIS
PD24202	SMARCA1	cna	hetloss	BASIS
PD24206	SMARCA1	cna	gain	BASIS
PD3890	SMARCA1	cna	hetloss	BASIS
PD3905	SMARCA1	cna	gain	BASIS
PD4107	SMARCA1	cna	amp	BASIS
PD4826	SMARCA1	cna	gain	BASIS
PD4967	SMARCA1	cna	gain	BASIS
PD5930	SMARCA1	cna	gain	BASIS
PD5935	SMARCA1	cna	gain	BASIS
PD5945	SMARCA1	cna	gain	BASIS
PD5948	SMARCA1	cna	gain	BASIS
PD6406	SMARCA1	cna	gain	BASIS
PD6413	SMARCA1	cna	gain	BASIS
PD7215	SMARCA1	cna	gain	BASIS
PD8621	SMARCA1	cna	amp	BASIS
PD8980	SMARCA1	cna	hetloss	BASIS
PD9004	SMARCA1	cna	gain	BASIS
TCGA-A2-A25B	SNAP25	cna	gain	TCGA
TCGA-AO-A0JL	SNAP25	cna	gain	TCGA
TCGA-BH-A0AW	SNAP25	cna	gain	TCGA
TCGA-BH-A0C0	SNAP25	cna	hetloss	TCGA
TCGA-BH-A18R	SNAP25	cna	homdel	TCGA
TCGA-C8-A12L	SNAP25	cna	gain	TCGA
TCGA-D8-A27M	SNAP25	cna	gain	TCGA
TCGA-E2-A1L7	SNAP25	cna	gain	TCGA
TCGA-LL-A5YP	SNAP25	cna	hetloss	TCGA
MB-0346	SNAP25	cna	gain	METABRIC
MB-5070	SNAP25	cna	amp	METABRIC
MB-6060	SNAP25	cna	amp	METABRIC

MB-6098	SNAP25	cna	hetloss	METABRIC
MB-7032	SNAP25	cna	gain	METABRIC
MB-7048	SNAP25	cna	gain	METABRIC
PD11327	SNAP25	cna	gain	BASIS
PD13296	SNAP25	cna	hetloss	BASIS
PD13299	SNAP25	cna	gain	BASIS
PD23574	SNAP25	cna	gain	BASIS
PD23578	SNAP25	cna	hetloss	BASIS
PD24186	SNAP25	cna	amp	BASIS
PD24202	SNAP25	cna	hetloss	BASIS
PD24337	SNAP25	cna	amp	BASIS
PD3905	SNAP25	cna	gain	BASIS
PD4005	SNAP25	cna	hetloss	BASIS
PD4006	SNAP25	cna	hetloss	BASIS
PD4107	SNAP25	cna	gain	BASIS
PD4826	SNAP25	cna	hetloss	BASIS
PD5930	SNAP25	cna	gain	BASIS
PD5935	SNAP25	cna	gain	BASIS
PD5945	SNAP25	cna	amp	BASIS
PD5948	SNAP25	cna	gain	BASIS
PD7067	SNAP25	cna	hetloss	BASIS
PD7215	SNAP25	cna	gain	BASIS
PD8621	SNAP25	cna	gain	BASIS
PD8980	SNAP25	cna	hetloss	BASIS
PD9004	SNAP25	cna	gain	BASIS
PD9585	SNAP25	cna	gain	BASIS
PD9702	SNAP25	cna	gain	BASIS
TCGA-A2-A25B	TAF1	cna	gain	TCGA
TCGA-AN-A0XU	TAF1	cna	hetloss	TCGA
TCGA-AO-A0JL	TAF1	cna	gain	TCGA
TCGA-BH-A0AW	TAF1	cna	gain	TCGA
TCGA-C8-A12L	TAF1	cna	hetloss	TCGA
TCGA-E2-A1L7	TAF1	cna	hetloss	TCGA
TCGA-E9-A1NC	TAF1	cna	hetloss	TCGA
MB-0346	TAF1	cna	hetloss	METABRIC
MB-2827	TAF1	cna	hetloss	METABRIC
MB-5465	TAF1	cna	gain	METABRIC
MB-6060	TAF1	cna	hetloss	METABRIC
MB-0420	TAF1	cna	hetloss	METABRIC
PD10014	TAF1	cna	hetloss	BASIS
PD11327	TAF1	cna	hetloss	BASIS
PD13296	TAF1	cna	amp	BASIS
PD13297	TAF1	cna	gain	BASIS
PD13299	TAF1	cna	gain	BASIS
PD13771	TAF1	cna	gain	BASIS
PD14442	TAF1	cna	gain	BASIS
PD23562	TAF1	cna	hetloss	BASIS

PD23574	TAF1	cna	gain	BASIS
PD24206	TAF1	cna	gain	BASIS
PD3890	TAF1	cna	gain	BASIS
PD3905	TAF1	cna	gain	BASIS
PD4005	TAF1	cna	hetloss	BASIS
PD4006	TAF1	cna	gain	BASIS
PD4107	TAF1	cna	amp	BASIS
PD4826	TAF1	cna	gain	BASIS
PD4967	TAF1	cna	gain	BASIS
PD5930	TAF1	cna	gain	BASIS
PD5935	TAF1	cna	gain	BASIS
PD5948	TAF1	cna	gain	BASIS
PD6406	TAF1	cna	gain	BASIS
PD6413	TAF1	cna	gain	BASIS
PD7067	TAF1	cna	gain	BASIS
PD7215	TAF1	cna	gain	BASIS
PD8621	TAF1	cna	amp	BASIS
PD8980	TAF1	cna	gain	BASIS
PD9004	TAF1	cna	gain	BASIS
TCGA-A2-A25B	TRAF3	cna	gain	TCGA
TCGA-AN-A0XU	TRAF3	cna	hetloss	TCGA
TCGA-AO-A0JL	TRAF3	cna	hetloss	TCGA
TCGA-BH-A0AW	TRAF3	cna	hetloss	TCGA
TCGA-BH-A1FU	TRAF3	cna	gain	TCGA
TCGA-C8-A12L	TRAF3	cna	gain	TCGA
TCGA-D8-A27M	TRAF3	cna	hetloss	TCGA
TCGA-E2-A1L7	TRAF3	cna	gain	TCGA
TCGA-E9-A1NC	TRAF3	cna	hetloss	TCGA
TCGA-LL-A5YP	TRAF3	cna	hetloss	TCGA
MB-0346	TRAF3	cna	gain	METABRIC
MB-2827	TRAF3	cna	hetloss	METABRIC
MB-5070	TRAF3	cna	hetloss	METABRIC
MB-5107	TRAF3	cna	hetloss	METABRIC
MB-6060	TRAF3	cna	gain	METABRIC
MB-6098	TRAF3	cna	gain	METABRIC
MTS-T0064	TRAF3	cna	hetloss	METABRIC
PD10014	TRAF3	cna	hetloss	BASIS
PD11742	TRAF3	cna	hetloss	BASIS
PD13296	TRAF3	cna	gain	BASIS
PD13297	TRAF3	cna	hetloss	BASIS
PD13771	TRAF3	cna	hetloss	BASIS
PD22355	TRAF3	cna	hetloss	BASIS
PD23578	TRAF3	cna	hetloss	BASIS
PD24202	TRAF3	cna	hetloss	BASIS
PD24337	TRAF3	cna	hetloss	BASIS
PD3890	TRAF3	cna	hetloss	BASIS
PD3905	TRAF3	cna	gain	BASIS

PD4006	TRAF3	cna	gain	BASIS
PD4826	TRAF3	cna	gain	BASIS
PD4967	TRAF3	cna	hetloss	BASIS
PD5945	TRAF3	cna	gain	BASIS
PD5948	TRAF3	cna	gain	BASIS
PD6406	TRAF3	cna	hetloss	BASIS
PD6413	TRAF3	cna	hetloss	BASIS
PD7067	TRAF3	cna	gain	BASIS
PD8621	TRAF3	cna	gain	BASIS
PD8980	TRAF3	cna	hetloss	BASIS
PD9702	TRAF3	cna	gain	BASIS
TCGA-AN-A0XU	TRAF7	cna	hetloss	TCGA
TCGA-AO-A0JL	TRAF7	cna	gain	TCGA
TCGA-BH-A0AW	TRAF7	cna	gain	TCGA
TCGA-BH-A0C0	TRAF7	cna	gain	TCGA
TCGA-C8-A12L	TRAF7	cna	gain	TCGA
TCGA-E2-A1L7	TRAF7	cna	gain	TCGA
TCGA-E9-A1NC	TRAF7	cna	hetloss	TCGA
TCGA-LL-A5YP	TRAF7	cna	gain	TCGA
MB-0346	TRAF7	cna	hetloss	METABRIC
MB-5465	TRAF7	cna	hetloss	METABRIC
MB-6271	TRAF7	cna	gain	METABRIC
MB-7032	TRAF7	cna	gain	METABRIC
PD10014	TRAF7	cna	gain	BASIS
PD11327	TRAF7	cna	gain	BASIS
PD11742	TRAF7	cna	gain	BASIS
PD13296	TRAF7	cna	gain	BASIS
PD13297	TRAF7	cna	hetloss	BASIS
PD13299	TRAF7	cna	gain	BASIS
PD22355	TRAF7	cna	hetloss	BASIS
PD23578	TRAF7	cna	hetloss	BASIS
PD24202	TRAF7	cna	hetloss	BASIS
PD24337	TRAF7	cna	hetloss	BASIS
PD3905	TRAF7	cna	gain	BASIS
PD4005	TRAF7	cna	gain	BASIS
PD4006	TRAF7	cna	gain	BASIS
PD4107	TRAF7	cna	gain	BASIS
PD4826	TRAF7	cna	gain	BASIS
PD4967	TRAF7	cna	gain	BASIS
PD5930	TRAF7	cna	gain	BASIS
PD5935	TRAF7	cna	gain	BASIS
PD5945	TRAF7	cna	gain	BASIS
PD5948	TRAF7	cna	gain	BASIS
PD6413	TRAF7	cna	gain	BASIS
PD7067	TRAF7	cna	gain	BASIS
PD7215	TRAF7	cna	amp	BASIS
PD8621	TRAF7	cna	gain	BASIS

PD9004	TRAF7	cna	gain	BASIS
PD9585	TRAF7	cna	hetloss	BASIS
PD9702	TRAF7	cna	gain	BASIS
TCGA-A2-A25B	WWTR1	cna	gain	TCGA
TCGA-AN-A0XU	WWTR1	cna	gain	TCGA
TCGA-BH-A0C0	WWTR1	cna	gain	TCGA
TCGA-BH-A18R	WWTR1	cna	gain	TCGA
TCGA-BH-A1FU	WWTR1	cna	hetloss	TCGA
TCGA-C8-A12L	WWTR1	cna	gain	TCGA
TCGA-E2-A1L7	WWTR1	cna	gain	TCGA
TCGA-E9-A1NC	WWTR1	cna	gain	TCGA
TCGA-LL-A5YP	WWTR1	cna	gain	TCGA
MB-0346	WWTR1	cna	gain	METABRIC
MB-5070	WWTR1	cna	amp	METABRIC
MB-5107	WWTR1	cna	gain	METABRIC
MB-5465	WWTR1	cna	amp	METABRIC
MB-0420	WWTR1	cna	gain	METABRIC
PD10014	WWTR1	cna	gain	BASIS
PD11327	WWTR1	cna	gain	BASIS
PD13296	WWTR1	cna	gain	BASIS
PD13299	WWTR1	cna	gain	BASIS
PD13771	WWTR1	cna	hetloss	BASIS
PD22355	WWTR1	cna	gain	BASIS
PD23561	WWTR1	cna	hetloss	BASIS
PD23562	WWTR1	cna	gain	BASIS
PD23574	WWTR1	cna	gain	BASIS
PD23578	WWTR1	cna	gain	BASIS
PD24186	WWTR1	cna	gain	BASIS
PD24206	WWTR1	cna	gain	BASIS
PD3905	WWTR1	cna	gain	BASIS
PD4107	WWTR1	cna	gain	BASIS
PD5930	WWTR1	cna	gain	BASIS
PD5935	WWTR1	cna	amp	BASIS
PD5945	WWTR1	cna	amp	BASIS
PD5948	WWTR1	cna	amp	BASIS
PD6731	WWTR1	cna	gain	BASIS
PD7067	WWTR1	cna	gain	BASIS
PD7215	WWTR1	cna	gain	BASIS
PD8621	WWTR1	cna	gain	BASIS
PD8980	WWTR1	cna	gain	BASIS
PD9004	WWTR1	cna	gain	BASIS
PD9702	WWTR1	cna	gain	BASIS
TCGA-A2-A25B	YAP1	cna	hetloss	TCGA
TCGA-AN-A0XU	YAP1	cna	gain	TCGA
TCGA-AO-A0JL	YAP1	cna	hetloss	TCGA
TCGA-BH-A18R	YAP1	cna	gain	TCGA
TCGA-D8-A27M	YAP1	cna	hetloss	TCGA

TCGA-EW-A1OX	YAP1	cna	homdel	TCGA
TCGA-LL-A5YP	YAP1	cna	hetloss	TCGA
MB-0346	YAP1	cna	hetloss	METABRIC
MB-5070	YAP1	cna	hetloss	METABRIC
MB-5107	YAP1	cna	hetloss	METABRIC
MB-6271	YAP1	cna	hetloss	METABRIC
MB-7032	YAP1	cna	hetloss	METABRIC
MB-7048	YAP1	cna	hetloss	METABRIC
MTS-T0064	YAP1	cna	amp	METABRIC
PD10014	YAP1	cna	gain	BASIS
PD11327	YAP1	cna	gain	BASIS
PD11742	YAP1	cna	hetloss	BASIS
PD13296	YAP1	cna	hetloss	BASIS
PD13299	YAP1	cna	gain	BASIS
PD13771	YAP1	cna	hetloss	BASIS
PD22355	YAP1	cna	hetloss	BASIS
PD23574	YAP1	cna	gain	BASIS
PD23578	YAP1	cna	hetloss	BASIS
PD24186	YAP1	cna	gain	BASIS
PD24206	YAP1	cna	gain	BASIS
PD24337	YAP1	cna	hetloss	BASIS
PD3905	YAP1	cna	gain	BASIS
PD4005	YAP1	cna	hetloss	BASIS
PD4006	YAP1	cna	hetloss	BASIS
PD4826	YAP1	cna	hetloss	BASIS
PD5935	YAP1	cna	gain	BASIS
PD5945	YAP1	cna	gain	BASIS
PD5948	YAP1	cna	gain	BASIS
PD6413	YAP1	cna	hetloss	BASIS
PD6731	YAP1	cna	hetloss	BASIS
PD7067	YAP1	cna	amp	BASIS
PD7215	YAP1	cna	gain	BASIS
PD9585	YAP1	cna	gain	BASIS
PD9702	YAP1	cna	gain	BASIS
TCGA-A2-A25B	ZBTB16	cna	hetloss	TCGA
TCGA-AN-A0XU	ZBTB16	cna	gain	TCGA
TCGA-AO-A0JL	ZBTB16	cna	hetloss	TCGA
TCGA-BH-A0C0	ZBTB16	cna	hetloss	TCGA
TCGA-BH-A18R	ZBTB16	cna	homdel	TCGA
TCGA-D8-A27M	ZBTB16	cna	hetloss	TCGA
TCGA-E2-A1L7	ZBTB16	cna	hetloss	TCGA
TCGA-E9-A1NC	ZBTB16	cna	hetloss	TCGA
TCGA-EW-A1OX	ZBTB16	cna	homdel	TCGA
TCGA-LL-A5YP	ZBTB16	cna	hetloss	TCGA
MB-0346	ZBTB16	cna	hetloss	METABRIC
MB-5070	ZBTB16	cna	hetloss	METABRIC
MB-5107	ZBTB16	cna	hetloss	METABRIC

MB-6271	ZBTB16	cna	hetloss	METABRIC
MB-7032	ZBTB16	cna	hetloss	METABRIC
MB-7048	ZBTB16	cna	hetloss	METABRIC
PD11327	ZBTB16	cna	gain	BASIS
PD11742	ZBTB16	cna	hetloss	BASIS
PD13296	ZBTB16	cna	hetloss	BASIS
PD13771	ZBTB16	cna	hetloss	BASIS
PD22355	ZBTB16	cna	hetloss	BASIS
PD23574	ZBTB16	cna	gain	BASIS
PD23578	ZBTB16	cna	hetloss	BASIS
PD24186	ZBTB16	cna	gain	BASIS
PD24206	ZBTB16	cna	gain	BASIS
PD24337	ZBTB16	cna	hetloss	BASIS
PD3905	ZBTB16	cna	gain	BASIS
PD4005	ZBTB16	cna	hetloss	BASIS
PD4826	ZBTB16	cna	hetloss	BASIS
PD5935	ZBTB16	cna	gain	BASIS
PD5945	ZBTB16	cna	gain	BASIS
PD5948	ZBTB16	cna	gain	BASIS
PD6413	ZBTB16	cna	hetloss	BASIS
PD6731	ZBTB16	cna	hetloss	BASIS
PD7067	ZBTB16	cna	gain	BASIS
PD7215	ZBTB16	cna	amp	BASIS
PD9004	ZBTB16	cna	hetloss	BASIS
PD9585	ZBTB16	cna	gain	BASIS
PD9702	ZBTB16	cna	gain	BASIS
TCGA-A2-A25B	AFF1	cna	hetloss	TCGA
TCGA-AN-A0XU	AFF1	cna	hetloss	TCGA
TCGA-AO-A0JL	AFF1	cna	gain	TCGA
TCGA-BH-A0AW	AFF1	cna	hetloss	TCGA
TCGA-BH-A0C0	AFF1	cna	hetloss	TCGA
TCGA-BH-A1FU	AFF1	cna	gain	TCGA
TCGA-D8-A27M	AFF1	cna	gain	TCGA
TCGA-E2-A1L7	AFF1	cna	hetloss	TCGA
TCGA-E9-A1NC	AFF1	cna	hetloss	TCGA
TCGA-LL-A5YP	AFF1	cna	gain	TCGA
MB-5070	AFF1	cna	hetloss	METABRIC
MB-5465	AFF1	cna	hetloss	METABRIC
MB-7038	AFF1	cna	gain	METABRIC
MB-7048	AFF1	cna	hetloss	METABRIC
MB-0420	AFF1	cna	hetloss	METABRIC
PD13296	AFF1	cna	hetloss	BASIS
PD13297	AFF1	cna	hetloss	BASIS
PD13771	AFF1	cna	gain	BASIS
PD22355	AFF1	cna	hetloss	BASIS
PD23562	AFF1	cna	gain	BASIS
PD23578	AFF1	cna	hetloss	BASIS

PD24202	AFF1	cna	hetloss	BASIS
PD24206	AFF1	cna	gain	BASIS
PD3890	AFF1	cna	gain	BASIS
PD3905	AFF1	cna	gain	BASIS
PD4006	AFF1	cna	hetloss	BASIS
PD4107	AFF1	cna	gain	BASIS
PD5930	AFF1	cna	hetloss	BASIS
PD5935	AFF1	cna	gain	BASIS
PD5945	AFF1	cna	gain	BASIS
PD5948	AFF1	cna	gain	BASIS
PD6731	AFF1	cna	hetloss	BASIS
PD7067	AFF1	cna	gain	BASIS
PD7215	AFF1	cna	gain	BASIS
PD8621	AFF1	cna	hetloss	BASIS
PD8980	AFF1	cna	hetloss	BASIS
PD9585	AFF1	cna	hetloss	BASIS
PD9702	AFF1	cna	hetloss	BASIS
TCGA-AN-A0XU	AFF2	cna	hetloss	TCGA
TCGA-BH-A0AW	AFF2	cna	gain	TCGA
TCGA-BH-A1FU	AFF2	cna	hetloss	TCGA
TCGA-C8-A12L	AFF2	cna	gain	TCGA
TCGA-E2-A1L7	AFF2	cna	hetloss	TCGA
TCGA-E9-A1NC	AFF2	cna	hetloss	TCGA
TCGA-LL-A5YP	AFF2	cna	gain	TCGA
MB-0346	AFF2	cna	hetloss	METABRIC
MB-2827	AFF2	cna	hetloss	METABRIC
MB-5465	AFF2	cna	hetloss	METABRIC
MB-6060	AFF2	cna	gain	METABRIC
MB-6098	AFF2	cna	hetloss	METABRIC
MB-7038	AFF2	cna	gain	METABRIC
PD10014	AFF2	cna	hetloss	BASIS
PD13296	AFF2	cna	gain	BASIS
PD13297	AFF2	cna	gain	BASIS
PD13299	AFF2	cna	amp	BASIS
PD13771	AFF2	cna	gain	BASIS
PD14442	AFF2	cna	gain	BASIS
PD23562	AFF2	cna	hetloss	BASIS
PD23574	AFF2	cna	gain	BASIS
PD23578	AFF2	cna	hetloss	BASIS
PD24202	AFF2	cna	hetloss	BASIS
PD24206	AFF2	cna	gain	BASIS
PD3890	AFF2	cna	hetloss	BASIS
PD3905	AFF2	cna	gain	BASIS
PD4107	AFF2	cna	gain	BASIS
PD4826	AFF2	cna	gain	BASIS
PD4967	AFF2	cna	gain	BASIS
PD5930	AFF2	cna	gain	BASIS

PD5935	AFF2	cna	gain	BASIS
PD5945	AFF2	cna	amp	BASIS
PD6406	AFF2	cna	gain	BASIS
PD6413	AFF2	cna	gain	BASIS
PD7215	AFF2	cna	amp	BASIS
PD8621	AFF2	cna	amp	BASIS
PD8980	AFF2	cna	hetloss	BASIS
PD9004	AFF2	cna	gain	BASIS
TCGA-A2-A25B	AMER1	cna	gain	TCGA
TCGA-AN-A0XU	AMER1	cna	hetloss	TCGA
TCGA-AO-A0JL	AMER1	cna	gain	TCGA
TCGA-BH-A0AW	AMER1	cna	gain	TCGA
TCGA-BH-A0C0	AMER1	cna	gain	TCGA
TCGA-E2-A1L7	AMER1	cna	hetloss	TCGA
TCGA-E9-A1NC	AMER1	cna	hetloss	TCGA
MB-0346	AMER1	cna	hetloss	METABRIC
MB-2827	AMER1	cna	hetloss	METABRIC
MB-5465	AMER1	cna	gain	METABRIC
MB-6060	AMER1	cna	hetloss	METABRIC
MB-6098	AMER1	cna	hetloss	METABRIC
MB-0420	AMER1	cna	hetloss	METABRIC
PD10014	AMER1	cna	hetloss	BASIS
PD11327	AMER1	cna	hetloss	BASIS
PD13296	AMER1	cna	amp	BASIS
PD13299	AMER1	cna	gain	BASIS
PD13771	AMER1	cna	gain	BASIS
PD14442	AMER1	cna	gain	BASIS
PD23574	AMER1	cna	gain	BASIS
PD24206	AMER1	cna	amp	BASIS
PD3890	AMER1	cna	gain	BASIS
PD3905	AMER1	cna	gain	BASIS
PD4005	AMER1	cna	hetloss	BASIS
PD4107	AMER1	cna	gain	BASIS
PD4826	AMER1	cna	gain	BASIS
PD4967	AMER1	cna	gain	BASIS
PD5930	AMER1	cna	gain	BASIS
PD5935	AMER1	cna	gain	BASIS
PD5945	AMER1	cna	gain	BASIS
PD5948	AMER1	cna	gain	BASIS
PD6406	AMER1	cna	gain	BASIS
PD6413	AMER1	cna	gain	BASIS
PD7067	AMER1	cna	gain	BASIS
PD7215	AMER1	cna	gain	BASIS
PD8621	AMER1	cna	gain	BASIS
PD8980	AMER1	cna	gain	BASIS
PD9004	AMER1	cna	gain	BASIS
TCGA-A2-A25B	ASCL4	cna	gain	TCGA

TCGA-AO-A0JL	ASCL4	cna	hetloss	TCGA
TCGA-BH-A0C0	ASCL4	cna	gain	TCGA
TCGA-BH-A18R	ASCL4	cna	gain	TCGA
TCGA-BH-A1FU	ASCL4	cna	hetloss	TCGA
TCGA-C8-A12L	ASCL4	cna	gain	TCGA
TCGA-D8-A27M	ASCL4	cna	hetloss	TCGA
TCGA-E2-A1L7	ASCL4	cna	gain	TCGA
TCGA-LL-A5YP	ASCL4	cna	hetloss	TCGA
MB-0346	ASCL4	cna	gain	METABRIC
MB-2827	ASCL4	cna	hetloss	METABRIC
MB-5070	ASCL4	cna	hetloss	METABRIC
MB-5465	ASCL4	cna	hetloss	METABRIC
MB-6098	ASCL4	cna	hetloss	METABRIC
MB-7048	ASCL4	cna	hetloss	METABRIC
PD10014	ASCL4	cna	gain	BASIS
PD11327	ASCL4	cna	gain	BASIS
PD11742	ASCL4	cna	hetloss	BASIS
PD13296	ASCL4	cna	hetloss	BASIS
PD13297	ASCL4	cna	gain	BASIS
PD22355	ASCL4	cna	hetloss	BASIS
PD23574	ASCL4	cna	gain	BASIS
PD3890	ASCL4	cna	hetloss	BASIS
PD3905	ASCL4	cna	gain	BASIS
PD4005	ASCL4	cna	hetloss	BASIS
PD4006	ASCL4	cna	gain	BASIS
PD4107	ASCL4	cna	gain	BASIS
PD4826	ASCL4	cna	gain	BASIS
PD5948	ASCL4	cna	gain	BASIS
PD6406	ASCL4	cna	hetloss	BASIS
PD6413	ASCL4	cna	hetloss	BASIS
PD6731	ASCL4	cna	hetloss	BASIS
PD7067	ASCL4	cna	gain	BASIS
PD7215	ASCL4	cna	hetloss	BASIS
PD8621	ASCL4	cna	hetloss	BASIS
PD9004	ASCL4	cna	hetloss	BASIS
PD9585	ASCL4	cna	hetloss	BASIS
PD9702	ASCL4	cna	gain	BASIS
TCGA-AN-A0XU	ATR	cna	gain	TCGA
TCGA-BH-A0C0	ATR	cna	gain	TCGA
TCGA-BH-A18R	ATR	cna	gain	TCGA
TCGA-BH-A1FU	ATR	cna	hetloss	TCGA
TCGA-C8-A12L	ATR	cna	gain	TCGA
TCGA-E2-A1L7	ATR	cna	gain	TCGA
TCGA-E9-A1NC	ATR	cna	gain	TCGA
TCGA-LL-A5YP	ATR	cna	gain	TCGA
MB-0346	ATR	cna	hetloss	METABRIC
MB-2827	ATR	cna	hetloss	METABRIC

MB-5070	ATR	cna	amp	METABRIC
MB-5107	ATR	cna	gain	METABRIC
MB-5465	ATR	cna	gain	METABRIC
MB-7038	ATR	cna	gain	METABRIC
MB-0420	ATR	cna	gain	METABRIC
P-0002858-T01-IM3	ATR	cna	amp	MSK-IMPACT
PD10014	ATR	cna	gain	BASIS
PD11327	ATR	cna	gain	BASIS
PD13299	ATR	cna	gain	BASIS
PD13771	ATR	cna	hetloss	BASIS
PD22355	ATR	cna	gain	BASIS
PD23561	ATR	cna	hetloss	BASIS
PD23574	ATR	cna	gain	BASIS
PD23578	ATR	cna	gain	BASIS
PD24186	ATR	cna	gain	BASIS
PD24206	ATR	cna	hetloss	BASIS
PD3905	ATR	cna	gain	BASIS
PD4107	ATR	cna	gain	BASIS
PD5935	ATR	cna	gain	BASIS
PD5945	ATR	cna	gain	BASIS
PD5948	ATR	cna	gain	BASIS
PD6731	ATR	cna	gain	BASIS
PD7067	ATR	cna	gain	BASIS
PD7215	ATR	cna	gain	BASIS
PD8621	ATR	cna	gain	BASIS
PD8980	ATR	cna	gain	BASIS
PD9004	ATR	cna	gain	BASIS
PD9702	ATR	cna	gain	BASIS
TCGA-A2-A25B	ATRX	cna	gain	TCGA
TCGA-AN-A0XU	ATRX	cna	hetloss	TCGA
TCGA-AO-A0JL	ATRX	cna	gain	TCGA
TCGA-BH-A0AW	ATRX	cna	gain	TCGA
TCGA-C8-A12L	ATRX	cna	hetloss	TCGA
TCGA-E2-A1L7	ATRX	cna	hetloss	TCGA
TCGA-E9-A1NC	ATRX	cna	hetloss	TCGA
MB-0346	ATRX	cna	hetloss	METABRIC
MB-2827	ATRX	cna	hetloss	METABRIC
MB-5465	ATRX	cna	gain	METABRIC
MB-6060	ATRX	cna	hetloss	METABRIC
MB-0420	ATRX	cna	hetloss	METABRIC
PD10014	ATRX	cna	hetloss	BASIS
PD11327	ATRX	cna	hetloss	BASIS
PD13296	ATRX	cna	amp	BASIS
PD13299	ATRX	cna	gain	BASIS
PD13771	ATRX	cna	gain	BASIS
PD14442	ATRX	cna	gain	BASIS
PD23562	ATRX	cna	hetloss	BASIS

PD23574	ATRX	cna	gain	BASIS
PD24186	ATRX	cna	gain	BASIS
PD24202	ATRX	cna	hetloss	BASIS
PD24206	ATRX	cna	gain	BASIS
PD3905	ATRX	cna	gain	BASIS
PD4005	ATRX	cna	hetloss	BASIS
PD4006	ATRX	cna	gain	BASIS
PD4107	ATRX	cna	gain	BASIS
PD4826	ATRX	cna	gain	BASIS
PD4967	ATRX	cna	gain	BASIS
PD5930	ATRX	cna	gain	BASIS
PD5935	ATRX	cna	gain	BASIS
PD5948	ATRX	cna	gain	BASIS
PD6406	ATRX	cna	gain	BASIS
PD6413	ATRX	cna	gain	BASIS
PD7067	ATRX	cna	gain	BASIS
PD7215	ATRX	cna	gain	BASIS
PD8621	ATRX	cna	gain	BASIS
PD9004	ATRX	cna	gain	BASIS
TCGA-A2-A25B	BCL7A	cna	gain	TCGA
TCGA-AN-A0XU	BCL7A	cna	hetloss	TCGA
TCGA-AO-A0JL	BCL7A	cna	hetloss	TCGA
TCGA-BH-A0C0	BCL7A	cna	hetloss	TCGA
TCGA-BH-A18R	BCL7A	cna	gain	TCGA
TCGA-BH-A1FU	BCL7A	cna	hetloss	TCGA
TCGA-D8-A27M	BCL7A	cna	hetloss	TCGA
TCGA-E2-A1L7	BCL7A	cna	hetloss	TCGA
TCGA-LL-A5YP	BCL7A	cna	hetloss	TCGA
MB-0346	BCL7A	cna	gain	METABRIC
MB-2827	BCL7A	cna	hetloss	METABRIC
MB-5070	BCL7A	cna	hetloss	METABRIC
MB-7048	BCL7A	cna	hetloss	METABRIC
MTS-T0064	BCL7A	cna	gain	METABRIC
PD11327	BCL7A	cna	homdel	BASIS
PD13296	BCL7A	cna	hetloss	BASIS
PD13297	BCL7A	cna	gain	BASIS
PD13771	BCL7A	cna	gain	BASIS
PD22355	BCL7A	cna	hetloss	BASIS
PD23574	BCL7A	cna	gain	BASIS
PD23578	BCL7A	cna	hetloss	BASIS
PD24206	BCL7A	cna	hetloss	BASIS
PD3890	BCL7A	cna	hetloss	BASIS
PD3905	BCL7A	cna	gain	BASIS
PD4005	BCL7A	cna	hetloss	BASIS
PD4006	BCL7A	cna	amp	BASIS
PD4107	BCL7A	cna	gain	BASIS
PD4826	BCL7A	cna	gain	BASIS

PD4967	BCL7A	cna	hetloss	BASIS
PD5935	BCL7A	cna	gain	BASIS
PD5945	BCL7A	cna	gain	BASIS
PD5948	BCL7A	cna	gain	BASIS
PD6406	BCL7A	cna	hetloss	BASIS
PD6731	BCL7A	cna	hetloss	BASIS
PD7067	BCL7A	cna	gain	BASIS
PD9004	BCL7A	cna	hetloss	BASIS
PD9585	BCL7A	cna	hetloss	BASIS
PD9702	BCL7A	cna	gain	BASIS
TCGA-AN-A0XU	CCDC160	cna	hetloss	TCGA
TCGA-BH-A0AW	CCDC160	cna	gain	TCGA
TCGA-C8-A12L	CCDC160	cna	gain	TCGA
TCGA-E2-A1L7	CCDC160	cna	hetloss	TCGA
TCGA-E9-A1NC	CCDC160	cna	hetloss	TCGA
TCGA-LL-A5YP	CCDC160	cna	gain	TCGA
MB-0346	CCDC160	cna	hetloss	METABRIC
MB-2827	CCDC160	cna	hetloss	METABRIC
MB-5465	CCDC160	cna	hetloss	METABRIC
MB-6060	CCDC160	cna	gain	METABRIC
MB-6098	CCDC160	cna	hetloss	METABRIC
MB-7038	CCDC160	cna	gain	METABRIC
PD10014	CCDC160	cna	hetloss	BASIS
PD13296	CCDC160	cna	amp	BASIS
PD13299	CCDC160	cna	amp	BASIS
PD13771	CCDC160	cna	gain	BASIS
PD14442	CCDC160	cna	gain	BASIS
PD23562	CCDC160	cna	hetloss	BASIS
PD23574	CCDC160	cna	gain	BASIS
PD23578	CCDC160	cna	hetloss	BASIS
PD24202	CCDC160	cna	hetloss	BASIS
PD24206	CCDC160	cna	gain	BASIS
PD3890	CCDC160	cna	hetloss	BASIS
PD3905	CCDC160	cna	gain	BASIS
PD4006	CCDC160	cna	gain	BASIS
PD4107	CCDC160	cna	gain	BASIS
PD4826	CCDC160	cna	gain	BASIS
PD4967	CCDC160	cna	gain	BASIS
PD5930	CCDC160	cna	gain	BASIS
PD5935	CCDC160	cna	gain	BASIS
PD5945	CCDC160	cna	gain	BASIS
PD5948	CCDC160	cna	gain	BASIS
PD6406	CCDC160	cna	gain	BASIS
PD6413	CCDC160	cna	gain	BASIS
PD7215	CCDC160	cna	gain	BASIS
PD8621	CCDC160	cna	amp	BASIS
PD8980	CCDC160	cna	hetloss	BASIS

PD9004	CCDC160	cna	gain	BASIS
TCGA-A2-A25B	CD274	cna	hetloss	TCGA
TCGA-AO-A0JL	CD274	cna	gain	TCGA
TCGA-BH-A0AW	CD274	cna	hetloss	TCGA
TCGA-BH-A0C0	CD274	cna	hetloss	TCGA
TCGA-BH-A1FU	CD274	cna	hetloss	TCGA
TCGA-C8-A12L	CD274	cna	hetloss	TCGA
TCGA-E2-A1L7	CD274	cna	hetloss	TCGA
MB-0346	CD274	cna	gain	METABRIC
MB-2827	CD274	cna	hetloss	METABRIC
MB-6060	CD274	cna	gain	METABRIC
MB-0420	CD274	cna	hetloss	METABRIC
MTS-T0064	CD274	cna	amp	METABRIC
P-0009557-T01-IM5	CD274	cna	amp	MSK-IMPACT
PD11327	CD274	cna	homdel	BASIS
PD13296	CD274	cna	gain	BASIS
PD13297	CD274	cna	hetloss	BASIS
PD13299	CD274	cna	amp	BASIS
PD22355	CD274	cna	gain	BASIS
PD23574	CD274	cna	amp	BASIS
PD24186	CD274	cna	gain	BASIS
PD24337	CD274	cna	gain	BASIS
PD3905	CD274	cna	gain	BASIS
PD4006	CD274	cna	gain	BASIS
PD4107	CD274	cna	gain	BASIS
PD4826	CD274	cna	gain	BASIS
PD4967	CD274	cna	hetloss	BASIS
PD5945	CD274	cna	gain	BASIS
PD5948	CD274	cna	hetloss	BASIS
PD6406	CD274	cna	homdel	BASIS
PD6413	CD274	cna	gain	BASIS
PD6731	CD274	cna	gain	BASIS
PD7067	CD274	cna	amp	BASIS
PD7215	CD274	cna	hetloss	BASIS
PD8621	CD274	cna	gain	BASIS
PD8980	CD274	cna	gain	BASIS
PD9004	CD274	cna	gain	BASIS
PD9585	CD274	cna	hetloss	BASIS
PD9702	CD274	cna	gain	BASIS
TCGA-AN-A0XU	CDK8	cna	hetloss	TCGA
TCGA-AO-A0JL	CDK8	cna	hetloss	TCGA
TCGA-BH-A0AW	CDK8	cna	hetloss	TCGA
TCGA-BH-A0C0	CDK8	cna	gain	TCGA
TCGA-BH-A1FU	CDK8	cna	hetloss	TCGA
TCGA-C8-A12L	CDK8	cna	hetloss	TCGA
TCGA-D8-A27M	CDK8	cna	gain	TCGA
TCGA-E9-A1NC	CDK8	cna	hetloss	TCGA

TCGA-EW-A1OX	CDK8	cna	homdel	TCGA
TCGA-LL-A5YP	CDK8	cna	hetloss	TCGA
MB-0346	CDK8	cna	amp	METABRIC
MB-2827	CDK8	cna	hetloss	METABRIC
MB-6098	CDK8	cna	hetloss	METABRIC
MB-7032	CDK8	cna	gain	METABRIC
PD10014	CDK8	cna	hetloss	BASIS
PD13296	CDK8	cna	hetloss	BASIS
PD13297	CDK8	cna	hetloss	BASIS
PD13771	CDK8	cna	hetloss	BASIS
PD14442	CDK8	cna	hetloss	BASIS
PD22355	CDK8	cna	hetloss	BASIS
PD23562	CDK8	cna	gain	BASIS
PD24202	CDK8	cna	hetloss	BASIS
PD24206	CDK8	cna	gain	BASIS
PD24337	CDK8	cna	hetloss	BASIS
PD3890	CDK8	cna	hetloss	BASIS
PD3905	CDK8	cna	gain	BASIS
PD4005	CDK8	cna	hetloss	BASIS
PD4006	CDK8	cna	gain	BASIS
PD4107	CDK8	cna	gain	BASIS
PD4826	CDK8	cna	hetloss	BASIS
PD4967	CDK8	cna	hetloss	BASIS
PD5945	CDK8	cna	amp	BASIS
PD6406	CDK8	cna	hetloss	BASIS
PD6413	CDK8	cna	hetloss	BASIS
PD6731	CDK8	cna	hetloss	BASIS
PD7067	CDK8	cna	gain	BASIS
PD8980	CDK8	cna	hetloss	BASIS
PD9702	CDK8	cna	gain	BASIS
TCGA-A2-A25B	CRBN	cna	gain	TCGA
TCGA-AN-A0XU	CRBN	cna	gain	TCGA
TCGA-BH-A0C0	CRBN	cna	gain	TCGA
TCGA-BH-A18R	CRBN	cna	gain	TCGA
TCGA-BH-A1FU	CRBN	cna	hetloss	TCGA
TCGA-C8-A12L	CRBN	cna	amp	TCGA
TCGA-D8-A27M	CRBN	cna	hetloss	TCGA
TCGA-E9-A1NC	CRBN	cna	hetloss	TCGA
TCGA-LL-A5YP	CRBN	cna	amp	TCGA
MB-7038	CRBN	cna	hetloss	METABRIC
MB-7048	CRBN	cna	hetloss	METABRIC
MB-0420	CRBN	cna	hetloss	METABRIC
PD10014	CRBN	cna	hetloss	BASIS
PD11327	CRBN	cna	gain	BASIS
PD11742	CRBN	cna	gain	BASIS
PD13296	CRBN	cna	gain	BASIS
PD13297	CRBN	cna	gain	BASIS

PD13299	CRBN	cna	gain	BASIS
PD23561	CRBN	cna	hetloss	BASIS
PD23562	CRBN	cna	gain	BASIS
PD23574	CRBN	cna	gain	BASIS
PD23578	CRBN	cna	hetloss	BASIS
PD24186	CRBN	cna	gain	BASIS
PD24206	CRBN	cna	gain	BASIS
PD24337	CRBN	cna	gain	BASIS
PD3905	CRBN	cna	gain	BASIS
PD4005	CRBN	cna	hetloss	BASIS
PD4107	CRBN	cna	hetloss	BASIS
PD4826	CRBN	cna	gain	BASIS
PD5945	CRBN	cna	amp	BASIS
PD5948	CRBN	cna	gain	BASIS
PD6406	CRBN	cna	hetloss	BASIS
PD6731	CRBN	cna	hetloss	BASIS
PD7215	CRBN	cna	gain	BASIS
PD8980	CRBN	cna	gain	BASIS
PD9004	CRBN	cna	gain	BASIS
PD9585	CRBN	cna	hetloss	BASIS
PD9702	CRBN	cna	gain	BASIS
TCGA-A2-A25B	CUL4A	cna	hetloss	TCGA
TCGA-AN-A0XU	CUL4A	cna	gain	TCGA
TCGA-AO-A0JL	CUL4A	cna	hetloss	TCGA
TCGA-BH-A0C0	CUL4A	cna	hetloss	TCGA
TCGA-BH-A1FU	CUL4A	cna	hetloss	TCGA
TCGA-C8-A12L	CUL4A	cna	gain	TCGA
TCGA-D8-A27M	CUL4A	cna	gain	TCGA
TCGA-LL-A5YP	CUL4A	cna	hetloss	TCGA
MB-0346	CUL4A	cna	hetloss	METABRIC
MB-5107	CUL4A	cna	hetloss	METABRIC
MB-5465	CUL4A	cna	gain	METABRIC
MB-6060	CUL4A	cna	hetloss	METABRIC
MB-7032	CUL4A	cna	hetloss	METABRIC
MB-0420	CUL4A	cna	amp	METABRIC
MTS-T0064	CUL4A	cna	hetloss	METABRIC
PD10014	CUL4A	cna	hetloss	BASIS
PD11327	CUL4A	cna	amp	BASIS
PD13299	CUL4A	cna	gain	BASIS
PD14442	CUL4A	cna	hetloss	BASIS
PD23562	CUL4A	cna	gain	BASIS
PD23574	CUL4A	cna	gain	BASIS
PD23578	CUL4A	cna	gain	BASIS
PD24186	CUL4A	cna	gain	BASIS
PD24202	CUL4A	cna	gain	BASIS
PD24206	CUL4A	cna	amp	BASIS
PD24337	CUL4A	cna	hetloss	BASIS

PD3905	CUL4A	cna	gain	BASIS
PD4107	CUL4A	cna	hetloss	BASIS
PD4826	CUL4A	cna	hetloss	BASIS
PD4967	CUL4A	cna	hetloss	BASIS
PD5945	CUL4A	cna	amp	BASIS
PD5948	CUL4A	cna	hetloss	BASIS
PD6731	CUL4A	cna	hetloss	BASIS
PD7215	CUL4A	cna	gain	BASIS
PD8621	CUL4A	cna	amp	BASIS
PD8980	CUL4A	cna	hetloss	BASIS
PD9585	CUL4A	cna	gain	BASIS
PD9702	CUL4A	cna	gain	BASIS
TCGA-A2-A25B	DEK	cna	gain	TCGA
TCGA-AN-A0XU	DEK	cna	amp	TCGA
TCGA-AO-A0JL	DEK	cna	gain	TCGA
TCGA-BH-A0AW	DEK	cna	gain	TCGA
TCGA-BH-A0C0	DEK	cna	hetloss	TCGA
TCGA-C8-A12L	DEK	cna	hetloss	TCGA
TCGA-D8-A27M	DEK	cna	hetloss	TCGA
TCGA-E2-A1L7	DEK	cna	hetloss	TCGA
MB-5070	DEK	cna	amp	METABRIC
MB-5465	DEK	cna	gain	METABRIC
MB-6060	DEK	cna	gain	METABRIC
MB-6098	DEK	cna	hetloss	METABRIC
MB-7032	DEK	cna	gain	METABRIC
PD11327	DEK	cna	gain	BASIS
PD13296	DEK	cna	gain	BASIS
PD13297	DEK	cna	gain	BASIS
PD13299	DEK	cna	amp	BASIS
PD22355	DEK	cna	gain	BASIS
PD23562	DEK	cna	gain	BASIS
PD23574	DEK	cna	gain	BASIS
PD24186	DEK	cna	gain	BASIS
PD24202	DEK	cna	gain	BASIS
PD24206	DEK	cna	gain	BASIS
PD3905	DEK	cna	amp	BASIS
PD4005	DEK	cna	hetloss	BASIS
PD4006	DEK	cna	gain	BASIS
PD4107	DEK	cna	gain	BASIS
PD4826	DEK	cna	gain	BASIS
PD5935	DEK	cna	gain	BASIS
PD5945	DEK	cna	amp	BASIS
PD5948	DEK	cna	gain	BASIS
PD6731	DEK	cna	hetloss	BASIS
PD7067	DEK	cna	gain	BASIS
PD7215	DEK	cna	gain	BASIS
PD8621	DEK	cna	amp	BASIS

PD9004	DEK	cna	gain	BASIS
PD9585	DEK	cna	hetloss	BASIS
PD9702	DEK	cna	gain	BASIS
TCGA-A2-A25B	EIF4A2	cna	amp	TCGA
TCGA-AN-A0XU	EIF4A2	cna	gain	TCGA
TCGA-BH-A0AW	EIF4A2	cna	gain	TCGA
TCGA-BH-A0C0	EIF4A2	cna	gain	TCGA
TCGA-BH-A1FU	EIF4A2	cna	hetloss	TCGA
TCGA-C8-A12L	EIF4A2	cna	gain	TCGA
TCGA-LL-A5YP	EIF4A2	cna	gain	TCGA
MB-0346	EIF4A2	cna	gain	METABRIC
MB-5070	EIF4A2	cna	hetloss	METABRIC
MB-5107	EIF4A2	cna	amp	METABRIC
MB-5465	EIF4A2	cna	gain	METABRIC
MB-6060	EIF4A2	cna	gain	METABRIC
MB-6098	EIF4A2	cna	gain	METABRIC
MB-7038	EIF4A2	cna	hetloss	METABRIC
PD11327	EIF4A2	cna	homdel	BASIS
PD13299	EIF4A2	cna	gain	BASIS
PD13771	EIF4A2	cna	hetloss	BASIS
PD22355	EIF4A2	cna	gain	BASIS
PD23561	EIF4A2	cna	hetloss	BASIS
PD23562	EIF4A2	cna	gain	BASIS
PD23578	EIF4A2	cna	gain	BASIS
PD24186	EIF4A2	cna	amp	BASIS
PD24202	EIF4A2	cna	gain	BASIS
PD3905	EIF4A2	cna	gain	BASIS
PD4005	EIF4A2	cna	gain	BASIS
PD4107	EIF4A2	cna	gain	BASIS
PD4826	EIF4A2	cna	gain	BASIS
PD5930	EIF4A2	cna	gain	BASIS
PD5945	EIF4A2	cna	amp	BASIS
PD5948	EIF4A2	cna	gain	BASIS
PD6406	EIF4A2	cna	gain	BASIS
PD6731	EIF4A2	cna	gain	BASIS
PD7067	EIF4A2	cna	gain	BASIS
PD7215	EIF4A2	cna	gain	BASIS
PD8621	EIF4A2	cna	gain	BASIS
PD8980	EIF4A2	cna	gain	BASIS
PD9004	EIF4A2	cna	gain	BASIS
PD9702	EIF4A2	cna	gain	BASIS
TCGA-AN-A0XU	EPHB1	cna	gain	TCGA
TCGA-BH-A0C0	EPHB1	cna	gain	TCGA
TCGA-BH-A18R	EPHB1	cna	gain	TCGA
TCGA-BH-A1FU	EPHB1	cna	hetloss	TCGA
TCGA-C8-A12L	EPHB1	cna	gain	TCGA
TCGA-E2-A1L7	EPHB1	cna	gain	TCGA

TCGA-E9-A1NC	EPHB1	cna	gain	TCGA
TCGA-LL-A5YP	EPHB1	cna	gain	TCGA
MB-0346	EPHB1	cna	hetloss	METABRIC
MB-5070	EPHB1	cna	gain	METABRIC
MB-5107	EPHB1	cna	gain	METABRIC
MB-5465	EPHB1	cna	gain	METABRIC
MB-7038	EPHB1	cna	gain	METABRIC
MTS-T0064	EPHB1	cna	gain	METABRIC
PD10014	EPHB1	cna	gain	BASIS
PD11327	EPHB1	cna	gain	BASIS
PD13297	EPHB1	cna	hetloss	BASIS
PD13299	EPHB1	cna	gain	BASIS
PD13771	EPHB1	cna	hetloss	BASIS
PD23561	EPHB1	cna	hetloss	BASIS
PD23574	EPHB1	cna	gain	BASIS
PD23578	EPHB1	cna	gain	BASIS
PD24186	EPHB1	cna	gain	BASIS
PD24202	EPHB1	cna	gain	BASIS
PD24206	EPHB1	cna	gain	BASIS
PD3905	EPHB1	cna	gain	BASIS
PD4107	EPHB1	cna	gain	BASIS
PD4826	EPHB1	cna	gain	BASIS
PD5935	EPHB1	cna	gain	BASIS
PD5945	EPHB1	cna	gain	BASIS
PD5948	EPHB1	cna	gain	BASIS
PD6731	EPHB1	cna	gain	BASIS
PD7067	EPHB1	cna	gain	BASIS
PD7215	EPHB1	cna	gain	BASIS
PD8621	EPHB1	cna	gain	BASIS
PD8980	EPHB1	cna	gain	BASIS
PD9004	EPHB1	cna	gain	BASIS
PD9702	EPHB1	cna	gain	BASIS
TCGA-AN-A0XU	FANCA	cna	gain	TCGA
TCGA-AO-A0JL	FANCA	cna	gain	TCGA
TCGA-BH-A0AW	FANCA	cna	hetloss	TCGA
TCGA-BH-A0C0	FANCA	cna	gain	TCGA
TCGA-C8-A12L	FANCA	cna	hetloss	TCGA
TCGA-E2-A1L7	FANCA	cna	hetloss	TCGA
TCGA-E9-A1NC	FANCA	cna	hetloss	TCGA
TCGA-LL-A5YP	FANCA	cna	gain	TCGA
MB-0346	FANCA	cna	gain	METABRIC
MB-5107	FANCA	cna	hetloss	METABRIC
MB-6271	FANCA	cna	hetloss	METABRIC
MB-7032	FANCA	cna	hetloss	METABRIC
MB-7038	FANCA	cna	gain	METABRIC
MB-7048	FANCA	cna	hetloss	METABRIC
MB-0420	FANCA	cna	hetloss	METABRIC

PD10014	FANCA	cna	hetloss	BASIS
PD11327	FANCA	cna	gain	BASIS
PD11742	FANCA	cna	hetloss	BASIS
PD13296	FANCA	cna	hetloss	BASIS
PD13297	FANCA	cna	hetloss	BASIS
PD13299	FANCA	cna	gain	BASIS
PD14442	FANCA	cna	hetloss	BASIS
PD23562	FANCA	cna	gain	BASIS
PD23574	FANCA	cna	gain	BASIS
PD24202	FANCA	cna	hetloss	BASIS
PD3890	FANCA	cna	gain	BASIS
PD3905	FANCA	cna	gain	BASIS
PD4006	FANCA	cna	gain	BASIS
PD4107	FANCA	cna	amp	BASIS
PD4967	FANCA	cna	hetloss	BASIS
PD5930	FANCA	cna	hetloss	BASIS
PD5945	FANCA	cna	amp	BASIS
PD5948	FANCA	cna	gain	BASIS
PD7215	FANCA	cna	gain	BASIS
PD8621	FANCA	cna	gain	BASIS
PD9004	FANCA	cna	gain	BASIS
PD9585	FANCA	cna	hetloss	BASIS
PD9702	FANCA	cna	gain	BASIS
TCGA-A2-A25B	FANCM	cna	hetloss	TCGA
TCGA-AN-A0XU	FANCM	cna	hetloss	TCGA
TCGA-AO-A0JL	FANCM	cna	hetloss	TCGA
TCGA-BH-A0AW	FANCM	cna	hetloss	TCGA
TCGA-BH-A1FU	FANCM	cna	gain	TCGA
TCGA-C8-A12L	FANCM	cna	hetloss	TCGA
TCGA-D8-A27M	FANCM	cna	hetloss	TCGA
TCGA-LL-A5YP	FANCM	cna	hetloss	TCGA
MB-5070	FANCM	cna	hetloss	METABRIC
MB-5465	FANCM	cna	hetloss	METABRIC
MB-6060	FANCM	cna	gain	METABRIC
PD10014	FANCM	cna	hetloss	BASIS
PD11327	FANCM	cna	gain	BASIS
PD13297	FANCM	cna	hetloss	BASIS
PD13771	FANCM	cna	gain	BASIS
PD22355	FANCM	cna	hetloss	BASIS
PD23578	FANCM	cna	hetloss	BASIS
PD24186	FANCM	cna	gain	BASIS
PD24202	FANCM	cna	hetloss	BASIS
PD24206	FANCM	cna	gain	BASIS
PD24337	FANCM	cna	hetloss	BASIS
PD3890	FANCM	cna	hetloss	BASIS
PD3905	FANCM	cna	gain	BASIS
PD4005	FANCM	cna	hetloss	BASIS

PD4006	FANCM	cna	hetloss	BASIS
PD4107	FANCM	cna	hetloss	BASIS
PD4826	FANCM	cna	gain	BASIS
PD5930	FANCM	cna	hetloss	BASIS
PD5945	FANCM	cna	gain	BASIS
PD5948	FANCM	cna	gain	BASIS
PD6406	FANCM	cna	hetloss	BASIS
PD7067	FANCM	cna	gain	BASIS
PD7215	FANCM	cna	gain	BASIS
PD8621	FANCM	cna	gain	BASIS
PD8980	FANCM	cna	gain	BASIS
PD9004	FANCM	cna	gain	BASIS
PD9585	FANCM	cna	gain	BASIS
PD9702	FANCM	cna	gain	BASIS
TCGA-A2-A25B	FAT4	cna	gain	TCGA
TCGA-AN-A0XU	FAT4	cna	gain	TCGA
TCGA-BH-A0AW	FAT4	cna	hetloss	TCGA
TCGA-BH-A0C0	FAT4	cna	hetloss	TCGA
TCGA-BH-A1FU	FAT4	cna	gain	TCGA
TCGA-C8-A12L	FAT4	cna	hetloss	TCGA
TCGA-D8-A27M	FAT4	cna	hetloss	TCGA
TCGA-E2-A1L7	FAT4	cna	hetloss	TCGA
TCGA-E9-A1NC	FAT4	cna	hetloss	TCGA
TCGA-LL-A5YP	FAT4	cna	gain	TCGA
MB-5070	FAT4	cna	hetloss	METABRIC
MB-5465	FAT4	cna	gain	METABRIC
MB-7048	FAT4	cna	hetloss	METABRIC
MB-0420	FAT4	cna	hetloss	METABRIC
PD11742	FAT4	cna	hetloss	BASIS
PD13296	FAT4	cna	hetloss	BASIS
PD13297	FAT4	cna	hetloss	BASIS
PD22355	FAT4	cna	hetloss	BASIS
PD23562	FAT4	cna	gain	BASIS
PD23574	FAT4	cna	gain	BASIS
PD23578	FAT4	cna	hetloss	BASIS
PD24202	FAT4	cna	hetloss	BASIS
PD24206	FAT4	cna	gain	BASIS
PD24337	FAT4	cna	hetloss	BASIS
PD3905	FAT4	cna	gain	BASIS
PD4006	FAT4	cna	hetloss	BASIS
PD4107	FAT4	cna	gain	BASIS
PD4826	FAT4	cna	gain	BASIS
PD5935	FAT4	cna	gain	BASIS
PD5945	FAT4	cna	amp	BASIS
PD5948	FAT4	cna	hetloss	BASIS
PD6406	FAT4	cna	hetloss	BASIS
PD6731	FAT4	cna	hetloss	BASIS

PD7067	FAT4	cna	gain	BASIS
PD7215	FAT4	cna	gain	BASIS
PD8621	FAT4	cna	hetloss	BASIS
PD8980	FAT4	cna	hetloss	BASIS
PD9585	FAT4	cna	hetloss	BASIS
TCGA-A2-A25B	FGF14	cna	hetloss	TCGA
TCGA-AN-A0XU	FGF14	cna	hetloss	TCGA
TCGA-AO-A0JL	FGF14	cna	hetloss	TCGA
TCGA-BH-A0C0	FGF14	cna	hetloss	TCGA
TCGA-BH-A1FU	FGF14	cna	hetloss	TCGA
TCGA-C8-A12L	FGF14	cna	gain	TCGA
TCGA-D8-A27M	FGF14	cna	amp	TCGA
TCGA-LL-A5YP	FGF14	cna	hetloss	TCGA
MB-5107	FGF14	cna	hetloss	METABRIC
MB-5465	FGF14	cna	gain	METABRIC
MB-6060	FGF14	cna	hetloss	METABRIC
MB-6098	FGF14	cna	gain	METABRIC
MB-0420	FGF14	cna	amp	METABRIC
PD10014	FGF14	cna	hetloss	BASIS
PD11327	FGF14	cna	amp	BASIS
PD13296	FGF14	cna	hetloss	BASIS
PD13299	FGF14	cna	gain	BASIS
PD14442	FGF14	cna	hetloss	BASIS
PD23562	FGF14	cna	gain	BASIS
PD23574	FGF14	cna	gain	BASIS
PD23578	FGF14	cna	gain	BASIS
PD24186	FGF14	cna	gain	BASIS
PD24202	FGF14	cna	gain	BASIS
PD24206	FGF14	cna	gain	BASIS
PD24337	FGF14	cna	hetloss	BASIS
PD3905	FGF14	cna	amp	BASIS
PD4005	FGF14	cna	hetloss	BASIS
PD4006	FGF14	cna	hetloss	BASIS
PD4107	FGF14	cna	hetloss	BASIS
PD4967	FGF14	cna	hetloss	BASIS
PD5945	FGF14	cna	amp	BASIS
PD5948	FGF14	cna	hetloss	BASIS
PD6731	FGF14	cna	hetloss	BASIS
PD7215	FGF14	cna	gain	BASIS
PD8621	FGF14	cna	amp	BASIS
PD8980	FGF14	cna	hetloss	BASIS
PD9585	FGF14	cna	gain	BASIS
PD9702	FGF14	cna	gain	BASIS
TCGA-A2-A25B	FGFR1	cna	gain	TCGA
TCGA-AN-A0XU	FGFR1	cna	hetloss	TCGA
TCGA-AO-A0JL	FGFR1	cna	gain	TCGA
TCGA-BH-A0C0	FGFR1	cna	hetloss	TCGA

TCGA-BH-A1FU	FGFR1	cna	homdel	TCGA
TCGA-D8-A27M	FGFR1	cna	hetloss	TCGA
TCGA-E2-A1L7	FGFR1	cna	hetloss	TCGA
TCGA-LL-A5YP	FGFR1	cna	hetloss	TCGA
MB-0346	FGFR1	cna	hetloss	METABRIC
MB-5465	FGFR1	cna	hetloss	METABRIC
MB-6060	FGFR1	cna	gain	METABRIC
MB-7038	FGFR1	cna	hetloss	METABRIC
MB-0420	FGFR1	cna	hetloss	METABRIC
P-0002237-T01-IM3	FGFR1	cna	amp	MSK-IMPACT
PD10014	FGFR1	cna	hetloss	BASIS
PD11327	FGFR1	cna	homdel	BASIS
PD13296	FGFR1	cna	hetloss	BASIS
PD13299	FGFR1	cna	gain	BASIS
PD13771	FGFR1	cna	hetloss	BASIS
PD23562	FGFR1	cna	gain	BASIS
PD23578	FGFR1	cna	hetloss	BASIS
PD24186	FGFR1	cna	gain	BASIS
PD24202	FGFR1	cna	hetloss	BASIS
PD24206	FGFR1	cna	amp	BASIS
PD24337	FGFR1	cna	hetloss	BASIS
PD3905	FGFR1	cna	gain	BASIS
PD4006	FGFR1	cna	gain	BASIS
PD4107	FGFR1	cna	gain	BASIS
PD4826	FGFR1	cna	hetloss	BASIS
PD5930	FGFR1	cna	gain	BASIS
PD5935	FGFR1	cna	gain	BASIS
PD5945	FGFR1	cna	gain	BASIS
PD7067	FGFR1	cna	gain	BASIS
PD7215	FGFR1	cna	amp	BASIS
PD8980	FGFR1	cna	amp	BASIS
PD9004	FGFR1	cna	gain	BASIS
PD9585	FGFR1	cna	hetloss	BASIS
PD9702	FGFR1	cna	gain	BASIS
TCGA-A2-A25B	HNF1A	cna	gain	TCGA
TCGA-AN-A0XU	HNF1A	cna	hetloss	TCGA
TCGA-AO-A0JL	HNF1A	cna	hetloss	TCGA
TCGA-BH-A0C0	HNF1A	cna	hetloss	TCGA
TCGA-BH-A18R	HNF1A	cna	gain	TCGA
TCGA-BH-A1FU	HNF1A	cna	hetloss	TCGA
TCGA-D8-A27M	HNF1A	cna	hetloss	TCGA
TCGA-E2-A1L7	HNF1A	cna	hetloss	TCGA
TCGA-LL-A5YP	HNF1A	cna	hetloss	TCGA
MB-0346	HNF1A	cna	gain	METABRIC
MB-2827	HNF1A	cna	hetloss	METABRIC
MB-5070	HNF1A	cna	hetloss	METABRIC
MB-7048	HNF1A	cna	hetloss	METABRIC

MTS-T0064	HNF1A	cna	gain	METABRIC
PD11327	HNF1A	cna	homdel	BASIS
PD13296	HNF1A	cna	hetloss	BASIS
PD13297	HNF1A	cna	gain	BASIS
PD13771	HNF1A	cna	gain	BASIS
PD22355	HNF1A	cna	hetloss	BASIS
PD23574	HNF1A	cna	gain	BASIS
PD23578	HNF1A	cna	hetloss	BASIS
PD24206	HNF1A	cna	homdel	BASIS
PD3890	HNF1A	cna	hetloss	BASIS
PD3905	HNF1A	cna	gain	BASIS
PD4005	HNF1A	cna	hetloss	BASIS
PD4006	HNF1A	cna	amp	BASIS
PD4107	HNF1A	cna	gain	BASIS
PD4826	HNF1A	cna	gain	BASIS
PD4967	HNF1A	cna	hetloss	BASIS
PD5935	HNF1A	cna	gain	BASIS
PD5945	HNF1A	cna	gain	BASIS
PD5948	HNF1A	cna	gain	BASIS
PD6406	HNF1A	cna	hetloss	BASIS
PD6731	HNF1A	cna	hetloss	BASIS
PD7067	HNF1A	cna	gain	BASIS
PD9004	HNF1A	cna	hetloss	BASIS
PD9585	HNF1A	cna	hetloss	BASIS
PD9702	HNF1A	cna	gain	BASIS
TCGA-AN-A0XU	IRF1	cna	hetloss	TCGA
TCGA-AO-A0JL	IRF1	cna	hetloss	TCGA
TCGA-BH-A0C0	IRF1	cna	hetloss	TCGA
TCGA-BH-A1FU	IRF1	cna	hetloss	TCGA
TCGA-C8-A12L	IRF1	cna	hetloss	TCGA
TCGA-D8-A27M	IRF1	cna	hetloss	TCGA
TCGA-E2-A1L7	IRF1	cna	gain	TCGA
TCGA-E9-A1NC	IRF1	cna	hetloss	TCGA
TCGA-LL-A5YP	IRF1	cna	hetloss	TCGA
MB-0346	IRF1	cna	gain	METABRIC
MB-2827	IRF1	cna	hetloss	METABRIC
MB-5070	IRF1	cna	hetloss	METABRIC
MB-5107	IRF1	cna	hetloss	METABRIC
MB-5465	IRF1	cna	hetloss	METABRIC
MB-6060	IRF1	cna	hetloss	METABRIC
MB-6098	IRF1	cna	hetloss	METABRIC
MB-7038	IRF1	cna	hetloss	METABRIC
MB-0420	IRF1	cna	hetloss	METABRIC
PD10014	IRF1	cna	hetloss	BASIS
PD13296	IRF1	cna	hetloss	BASIS
PD13297	IRF1	cna	hetloss	BASIS
PD13771	IRF1	cna	gain	BASIS

PD22355	IRF1	cna	hetloss	BASIS
PD23562	IRF1	cna	hetloss	BASIS
PD23578	IRF1	cna	hetloss	BASIS
PD24202	IRF1	cna	hetloss	BASIS
PD24206	IRF1	cna	gain	BASIS
PD24337	IRF1	cna	hetloss	BASIS
PD3890	IRF1	cna	hetloss	BASIS
PD3905	IRF1	cna	gain	BASIS
PD4005	IRF1	cna	hetloss	BASIS
PD5935	IRF1	cna	gain	BASIS
PD5945	IRF1	cna	gain	BASIS
PD6406	IRF1	cna	hetloss	BASIS
PD6413	IRF1	cna	hetloss	BASIS
PD7067	IRF1	cna	gain	BASIS
PD8980	IRF1	cna	hetloss	BASIS
PD9585	IRF1	cna	hetloss	BASIS
TCGA-A2-A25B	JAK2	cna	hetloss	TCGA
TCGA-AO-A0JL	JAK2	cna	gain	TCGA
TCGA-BH-A0AW	JAK2	cna	hetloss	TCGA
TCGA-BH-A0C0	JAK2	cna	hetloss	TCGA
TCGA-BH-A1FU	JAK2	cna	hetloss	TCGA
TCGA-C8-A12L	JAK2	cna	hetloss	TCGA
TCGA-E2-A1L7	JAK2	cna	hetloss	TCGA
MB-0346	JAK2	cna	gain	METABRIC
MB-2827	JAK2	cna	hetloss	METABRIC
MB-6060	JAK2	cna	gain	METABRIC
MB-0420	JAK2	cna	hetloss	METABRIC
MTS-T0064	JAK2	cna	amp	METABRIC
P-0009557-T01-IM5	JAK2	cna	amp	MSK-IMPACT
PD11327	JAK2	cna	homdel	BASIS
PD13296	JAK2	cna	gain	BASIS
PD13297	JAK2	cna	hetloss	BASIS
PD13299	JAK2	cna	amp	BASIS
PD22355	JAK2	cna	gain	BASIS
PD23574	JAK2	cna	amp	BASIS
PD24186	JAK2	cna	gain	BASIS
PD24337	JAK2	cna	gain	BASIS
PD3905	JAK2	cna	gain	BASIS
PD4006	JAK2	cna	gain	BASIS
PD4107	JAK2	cna	gain	BASIS
PD4826	JAK2	cna	gain	BASIS
PD4967	JAK2	cna	hetloss	BASIS
PD5945	JAK2	cna	gain	BASIS
PD5948	JAK2	cna	hetloss	BASIS
PD6406	JAK2	cna	homdel	BASIS
PD6413	JAK2	cna	gain	BASIS
PD6731	JAK2	cna	gain	BASIS

PD7067	JAK2	cna	amp	BASIS
PD7215	JAK2	cna	hetloss	BASIS
PD8621	JAK2	cna	gain	BASIS
PD8980	JAK2	cna	gain	BASIS
PD9004	JAK2	cna	gain	BASIS
PD9585	JAK2	cna	hetloss	BASIS
PD9702	JAK2	cna	gain	BASIS
TCGA-A2-A25B	KAT6B	cna	hetloss	TCGA
TCGA-AN-A0XU	KAT6B	cna	hetloss	TCGA
TCGA-AO-A0JL	KAT6B	cna	gain	TCGA
TCGA-BH-A0AW	KAT6B	cna	gain	TCGA
TCGA-BH-A0C0	KAT6B	cna	gain	TCGA
TCGA-C8-A12L	KAT6B	cna	hetloss	TCGA
TCGA-D8-A27M	KAT6B	cna	hetloss	TCGA
TCGA-E2-A1L7	KAT6B	cna	gain	TCGA
TCGA-LL-A5YP	KAT6B	cna	hetloss	TCGA
MB-2827	KAT6B	cna	hetloss	METABRIC
MB-5070	KAT6B	cna	gain	METABRIC
MB-6098	KAT6B	cna	hetloss	METABRIC
MB-7048	KAT6B	cna	hetloss	METABRIC
MTS-T0064	KAT6B	cna	gain	METABRIC
PD10014	KAT6B	cna	gain	BASIS
PD11742	KAT6B	cna	hetloss	BASIS
PD13296	KAT6B	cna	hetloss	BASIS
PD13299	KAT6B	cna	gain	BASIS
PD23574	KAT6B	cna	gain	BASIS
PD24186	KAT6B	cna	gain	BASIS
PD24202	KAT6B	cna	hetloss	BASIS
PD24206	KAT6B	cna	gain	BASIS
PD4006	KAT6B	cna	gain	BASIS
PD4107	KAT6B	cna	gain	BASIS
PD4826	KAT6B	cna	gain	BASIS
PD4967	KAT6B	cna	hetloss	BASIS
PD5930	KAT6B	cna	gain	BASIS
PD5935	KAT6B	cna	gain	BASIS
PD5945	KAT6B	cna	hetloss	BASIS
PD6406	KAT6B	cna	hetloss	BASIS
PD6731	KAT6B	cna	hetloss	BASIS
PD7067	KAT6B	cna	gain	BASIS
PD7215	KAT6B	cna	gain	BASIS
PD8621	KAT6B	cna	gain	BASIS
PD8980	KAT6B	cna	hetloss	BASIS
PD9004	KAT6B	cna	gain	BASIS
PD9585	KAT6B	cna	hetloss	BASIS
PD9702	KAT6B	cna	gain	BASIS
TCGA-A2-A25B	KDM2B	cna	gain	TCGA
TCGA-AN-A0XU	KDM2B	cna	hetloss	TCGA

TCGA-AO-A0JL	KDM2B	cna	hetloss	TCGA
TCGA-BH-A0C0	KDM2B	cna	hetloss	TCGA
TCGA-BH-A18R	KDM2B	cna	gain	TCGA
TCGA-BH-A1FU	KDM2B	cna	hetloss	TCGA
TCGA-D8-A27M	KDM2B	cna	hetloss	TCGA
TCGA-E2-A1L7	KDM2B	cna	hetloss	TCGA
TCGA-LL-A5YP	KDM2B	cna	hetloss	TCGA
MB-0346	KDM2B	cna	gain	METABRIC
MB-2827	KDM2B	cna	hetloss	METABRIC
MB-5070	KDM2B	cna	hetloss	METABRIC
MB-7048	KDM2B	cna	hetloss	METABRIC
MTS-T0064	KDM2B	cna	gain	METABRIC
PD11327	KDM2B	cna	homdel	BASIS
PD13296	KDM2B	cna	hetloss	BASIS
PD13297	KDM2B	cna	gain	BASIS
PD13771	KDM2B	cna	gain	BASIS
PD22355	KDM2B	cna	hetloss	BASIS
PD23574	KDM2B	cna	gain	BASIS
PD23578	KDM2B	cna	hetloss	BASIS
PD24206	KDM2B	cna	homdel	BASIS
PD3890	KDM2B	cna	hetloss	BASIS
PD3905	KDM2B	cna	gain	BASIS
PD4005	KDM2B	cna	hetloss	BASIS
PD4006	KDM2B	cna	amp	BASIS
PD4107	KDM2B	cna	gain	BASIS
PD4826	KDM2B	cna	gain	BASIS
PD4967	KDM2B	cna	hetloss	BASIS
PD5935	KDM2B	cna	gain	BASIS
PD5945	KDM2B	cna	gain	BASIS
PD5948	KDM2B	cna	gain	BASIS
PD6406	KDM2B	cna	hetloss	BASIS
PD6731	KDM2B	cna	hetloss	BASIS
PD7067	KDM2B	cna	gain	BASIS
PD9004	KDM2B	cna	hetloss	BASIS
PD9585	KDM2B	cna	hetloss	BASIS
PD9702	KDM2B	cna	gain	BASIS
TCGA-A2-A25B	KDSR	cna	hetloss	TCGA
TCGA-AO-A0JL	KDSR	cna	gain	TCGA
TCGA-BH-A0AW	KDSR	cna	hetloss	TCGA
TCGA-BH-A0C0	KDSR	cna	gain	TCGA
TCGA-C8-A12L	KDSR	cna	gain	TCGA
TCGA-D8-A27M	KDSR	cna	hetloss	TCGA
TCGA-E2-A1L7	KDSR	cna	gain	TCGA
TCGA-LL-A5YP	KDSR	cna	hetloss	TCGA
MB-0346	KDSR	cna	hetloss	METABRIC
MB-0420	KDSR	cna	hetloss	METABRIC
MTS-T0064	KDSR	cna	hetloss	METABRIC

PD10014	KDSR	cna	hetloss	BASIS
PD11742	KDSR	cna	hetloss	BASIS
PD13296	KDSR	cna	hetloss	BASIS
PD13297	KDSR	cna	gain	BASIS
PD13299	KDSR	cna	gain	BASIS
PD22355	KDSR	cna	hetloss	BASIS
PD23574	KDSR	cna	hetloss	BASIS
PD23578	KDSR	cna	hetloss	BASIS
PD24186	KDSR	cna	gain	BASIS
PD24202	KDSR	cna	hetloss	BASIS
PD24206	KDSR	cna	hetloss	BASIS
PD24337	KDSR	cna	gain	BASIS
PD3905	KDSR	cna	gain	BASIS
PD4006	KDSR	cna	gain	BASIS
PD4107	KDSR	cna	gain	BASIS
PD4826	KDSR	cna	hetloss	BASIS
PD4967	KDSR	cna	hetloss	BASIS
PD5930	KDSR	cna	hetloss	BASIS
PD5945	KDSR	cna	gain	BASIS
PD5948	KDSR	cna	gain	BASIS
PD6406	KDSR	cna	gain	BASIS
PD7067	KDSR	cna	gain	BASIS
PD8621	KDSR	cna	hetloss	BASIS
PD8980	KDSR	cna	hetloss	BASIS
PD9004	KDSR	cna	hetloss	BASIS
PD9585	KDSR	cna	hetloss	BASIS
PD9702	KDSR	cna	gain	BASIS
TCGA-A2-A25B	KLHL6	cna	amp	TCGA
TCGA-AN-A0XU	KLHL6	cna	gain	TCGA
TCGA-BH-A0AW	KLHL6	cna	gain	TCGA
TCGA-BH-A0C0	KLHL6	cna	gain	TCGA
TCGA-BH-A1FU	KLHL6	cna	hetloss	TCGA
TCGA-C8-A12L	KLHL6	cna	gain	TCGA
TCGA-E2-A1L7	KLHL6	cna	gain	TCGA
TCGA-LL-A5YP	KLHL6	cna	gain	TCGA
MB-0346	KLHL6	cna	gain	METABRIC
MB-5070	KLHL6	cna	amp	METABRIC
MB-5107	KLHL6	cna	amp	METABRIC
MB-6098	KLHL6	cna	gain	METABRIC
MB-0420	KLHL6	cna	gain	METABRIC
MTS-T0064	KLHL6	cna	amp	METABRIC
PD11327	KLHL6	cna	homdel	BASIS
PD13296	KLHL6	cna	gain	BASIS
PD13299	KLHL6	cna	gain	BASIS
PD13771	KLHL6	cna	hetloss	BASIS
PD22355	KLHL6	cna	gain	BASIS
PD23561	KLHL6	cna	hetloss	BASIS

PD23562	KLHL6	cna	gain	BASIS
PD23578	KLHL6	cna	gain	BASIS
PD24186	KLHL6	cna	amp	BASIS
PD24202	KLHL6	cna	gain	BASIS
PD24206	KLHL6	cna	gain	BASIS
PD3905	KLHL6	cna	gain	BASIS
PD4005	KLHL6	cna	gain	BASIS
PD4107	KLHL6	cna	gain	BASIS
PD5935	KLHL6	cna	gain	BASIS
PD5945	KLHL6	cna	amp	BASIS
PD5948	KLHL6	cna	gain	BASIS
PD6406	KLHL6	cna	gain	BASIS
PD6731	KLHL6	cna	gain	BASIS
PD7067	KLHL6	cna	gain	BASIS
PD7215	KLHL6	cna	gain	BASIS
PD8621	KLHL6	cna	gain	BASIS
PD8980	KLHL6	cna	gain	BASIS
PD9702	KLHL6	cna	gain	BASIS
TCGA-A2-A25B	KMT2A	cna	hetloss	TCGA
TCGA-AN-A0XU	KMT2A	cna	gain	TCGA
TCGA-AO-A0JL	KMT2A	cna	hetloss	TCGA
TCGA-BH-A0C0	KMT2A	cna	hetloss	TCGA
TCGA-BH-A18R	KMT2A	cna	homdel	TCGA
TCGA-D8-A27M	KMT2A	cna	hetloss	TCGA
TCGA-E2-A1L7	KMT2A	cna	hetloss	TCGA
TCGA-E9-A1NC	KMT2A	cna	hetloss	TCGA
TCGA-EW-A1OX	KMT2A	cna	homdel	TCGA
TCGA-LL-A5YP	KMT2A	cna	hetloss	TCGA
MB-0346	KMT2A	cna	hetloss	METABRIC
MB-5070	KMT2A	cna	hetloss	METABRIC
MB-5107	KMT2A	cna	hetloss	METABRIC
MB-6271	KMT2A	cna	hetloss	METABRIC
MB-7032	KMT2A	cna	hetloss	METABRIC
MB-7048	KMT2A	cna	hetloss	METABRIC
PD11742	KMT2A	cna	hetloss	BASIS
PD13296	KMT2A	cna	hetloss	BASIS
PD13297	KMT2A	cna	hetloss	BASIS
PD13771	KMT2A	cna	hetloss	BASIS
PD22355	KMT2A	cna	hetloss	BASIS
PD23574	KMT2A	cna	gain	BASIS
PD23578	KMT2A	cna	hetloss	BASIS
PD24186	KMT2A	cna	gain	BASIS
PD24206	KMT2A	cna	gain	BASIS
PD24337	KMT2A	cna	hetloss	BASIS
PD3905	KMT2A	cna	gain	BASIS
PD4005	KMT2A	cna	hetloss	BASIS
PD4826	KMT2A	cna	hetloss	BASIS

PD5935	KMT2A	cna	gain	BASIS
PD5945	KMT2A	cna	gain	BASIS
PD5948	KMT2A	cna	gain	BASIS
PD6413	KMT2A	cna	hetloss	BASIS
PD6731	KMT2A	cna	hetloss	BASIS
PD7067	KMT2A	cna	gain	BASIS
PD7215	KMT2A	cna	gain	BASIS
PD9004	KMT2A	cna	hetloss	BASIS
PD9702	KMT2A	cna	gain	BASIS
TCGA-AN-A0XU	LATS2	cna	hetloss	TCGA
TCGA-AO-A0JL	LATS2	cna	hetloss	TCGA
TCGA-BH-A0AW	LATS2	cna	hetloss	TCGA
TCGA-BH-A0C0	LATS2	cna	hetloss	TCGA
TCGA-BH-A1FU	LATS2	cna	hetloss	TCGA
TCGA-C8-A12L	LATS2	cna	hetloss	TCGA
TCGA-D8-A27M	LATS2	cna	hetloss	TCGA
TCGA-E2-A1L7	LATS2	cna	hetloss	TCGA
TCGA-E9-A1NC	LATS2	cna	hetloss	TCGA
TCGA-EW-A1OX	LATS2	cna	homdel	TCGA
TCGA-LL-A5YP	LATS2	cna	gain	TCGA
MB-0346	LATS2	cna	hetloss	METABRIC
MB-2827	LATS2	cna	hetloss	METABRIC
MB-6098	LATS2	cna	hetloss	METABRIC
MTS-T0064	LATS2	cna	hetloss	METABRIC
PD10014	LATS2	cna	hetloss	BASIS
PD11327	LATS2	cna	homdel	BASIS
PD13296	LATS2	cna	hetloss	BASIS
PD13297	LATS2	cna	hetloss	BASIS
PD13771	LATS2	cna	hetloss	BASIS
PD14442	LATS2	cna	hetloss	BASIS
PD22355	LATS2	cna	hetloss	BASIS
PD24202	LATS2	cna	hetloss	BASIS
PD24206	LATS2	cna	gain	BASIS
PD24337	LATS2	cna	hetloss	BASIS
PD3890	LATS2	cna	hetloss	BASIS
PD3905	LATS2	cna	gain	BASIS
PD4005	LATS2	cna	hetloss	BASIS
PD4006	LATS2	cna	gain	BASIS
PD4107	LATS2	cna	gain	BASIS
PD4826	LATS2	cna	hetloss	BASIS
PD4967	LATS2	cna	hetloss	BASIS
PD5945	LATS2	cna	amp	BASIS
PD6413	LATS2	cna	hetloss	BASIS
PD6731	LATS2	cna	hetloss	BASIS
PD7067	LATS2	cna	gain	BASIS
PD8980	LATS2	cna	hetloss	BASIS
PD9702	LATS2	cna	gain	BASIS

TCGA-A2-A25B	LIFR	cna	amp	TCGA
TCGA-AN-A0XU	LIFR	cna	gain	TCGA
TCGA-AO-A0JL	LIFR	cna	gain	TCGA
TCGA-BH-A0AW	LIFR	cna	gain	TCGA
TCGA-BH-A0C0	LIFR	cna	gain	TCGA
TCGA-BH-A1FU	LIFR	cna	amp	TCGA
TCGA-E9-A1NC	LIFR	cna	hetloss	TCGA
TCGA-LL-A5YP	LIFR	cna	amp	TCGA
MB-0346	LIFR	cna	gain	METABRIC
MB-5070	LIFR	cna	gain	METABRIC
MB-6060	LIFR	cna	gain	METABRIC
MB-6098	LIFR	cna	amp	METABRIC
MB-0420	LIFR	cna	gain	METABRIC
PD10014	LIFR	cna	hetloss	BASIS
PD11327	LIFR	cna	gain	BASIS
PD13299	LIFR	cna	gain	BASIS
PD23562	LIFR	cna	gain	BASIS
PD23574	LIFR	cna	gain	BASIS
PD24186	LIFR	cna	gain	BASIS
PD24206	LIFR	cna	gain	BASIS
PD24337	LIFR	cna	hetloss	BASIS
PD3890	LIFR	cna	gain	BASIS
PD3905	LIFR	cna	gain	BASIS
PD4005	LIFR	cna	hetloss	BASIS
PD4006	LIFR	cna	gain	BASIS
PD4107	LIFR	cna	gain	BASIS
PD4826	LIFR	cna	gain	BASIS
PD5930	LIFR	cna	gain	BASIS
PD5935	LIFR	cna	hetloss	BASIS
PD5945	LIFR	cna	amp	BASIS
PD5948	LIFR	cna	gain	BASIS
PD6406	LIFR	cna	hetloss	BASIS
PD6413	LIFR	cna	gain	BASIS
PD6731	LIFR	cna	gain	BASIS
PD7215	LIFR	cna	amp	BASIS
PD8980	LIFR	cna	gain	BASIS
PD9004	LIFR	cna	gain	BASIS
PD9585	LIFR	cna	gain	BASIS
TCGA-A2-A25B	MAGEE1	cna	gain	TCGA
TCGA-AN-A0XU	MAGEE1	cna	hetloss	TCGA
TCGA-AO-A0JL	MAGEE1	cna	gain	TCGA
TCGA-BH-A0AW	MAGEE1	cna	gain	TCGA
TCGA-C8-A12L	MAGEE1	cna	hetloss	TCGA
TCGA-E2-A1L7	MAGEE1	cna	hetloss	TCGA
TCGA-E9-A1NC	MAGEE1	cna	hetloss	TCGA
MB-0346	MAGEE1	cna	hetloss	METABRIC
MB-2827	MAGEE1	cna	hetloss	METABRIC

MB-5465	MAGEE1	cna	gain	METABRIC
MB-6060	MAGEE1	cna	hetloss	METABRIC
MB-0420	MAGEE1	cna	hetloss	METABRIC
PD10014	MAGEE1	cna	hetloss	BASIS
PD11327	MAGEE1	cna	hetloss	BASIS
PD13296	MAGEE1	cna	amp	BASIS
PD13299	MAGEE1	cna	gain	BASIS
PD13771	MAGEE1	cna	gain	BASIS
PD14442	MAGEE1	cna	gain	BASIS
PD23562	MAGEE1	cna	hetloss	BASIS
PD23574	MAGEE1	cna	gain	BASIS
PD24186	MAGEE1	cna	gain	BASIS
PD24206	MAGEE1	cna	gain	BASIS
PD3905	MAGEE1	cna	gain	BASIS
PD4005	MAGEE1	cna	hetloss	BASIS
PD4006	MAGEE1	cna	gain	BASIS
PD4107	MAGEE1	cna	gain	BASIS
PD4826	MAGEE1	cna	gain	BASIS
PD4967	MAGEE1	cna	gain	BASIS
PD5930	MAGEE1	cna	gain	BASIS
PD5935	MAGEE1	cna	gain	BASIS
PD5948	MAGEE1	cna	gain	BASIS
PD6406	MAGEE1	cna	gain	BASIS
PD6413	MAGEE1	cna	gain	BASIS
PD7067	MAGEE1	cna	gain	BASIS
PD7215	MAGEE1	cna	gain	BASIS
PD8621	MAGEE1	cna	gain	BASIS
PD8980	MAGEE1	cna	gain	BASIS
PD9004	MAGEE1	cna	gain	BASIS
TCGA-A2-A25B	MAML2	cna	hetloss	TCGA
TCGA-AN-A0XU	MAML2	cna	gain	TCGA
TCGA-AO-A0JL	MAML2	cna	hetloss	TCGA
TCGA-D8-A27M	MAML2	cna	amp	TCGA
TCGA-EW-A1OX	MAML2	cna	homdel	TCGA
TCGA-LL-A5YP	MAML2	cna	hetloss	TCGA
MB-0346	MAML2	cna	hetloss	METABRIC
MB-5070	MAML2	cna	hetloss	METABRIC
MB-6271	MAML2	cna	hetloss	METABRIC
MB-7032	MAML2	cna	hetloss	METABRIC
MB-7048	MAML2	cna	hetloss	METABRIC
MB-0420	MAML2	cna	hetloss	METABRIC
MTS-T0064	MAML2	cna	gain	METABRIC
PD10014	MAML2	cna	gain	BASIS
PD11327	MAML2	cna	gain	BASIS
PD11742	MAML2	cna	hetloss	BASIS
PD13296	MAML2	cna	hetloss	BASIS
PD13299	MAML2	cna	gain	BASIS

PD13771	MAML2	cna	hetloss	BASIS
PD22355	MAML2	cna	hetloss	BASIS
PD23574	MAML2	cna	gain	BASIS
PD23578	MAML2	cna	hetloss	BASIS
PD24186	MAML2	cna	gain	BASIS
PD24206	MAML2	cna	gain	BASIS
PD24337	MAML2	cna	hetloss	BASIS
PD3890	MAML2	cna	hetloss	BASIS
PD3905	MAML2	cna	gain	BASIS
PD4006	MAML2	cna	hetloss	BASIS
PD4826	MAML2	cna	hetloss	BASIS
PD5935	MAML2	cna	gain	BASIS
PD5945	MAML2	cna	gain	BASIS
PD5948	MAML2	cna	gain	BASIS
PD6413	MAML2	cna	hetloss	BASIS
PD7067	MAML2	cna	amp	BASIS
PD7215	MAML2	cna	gain	BASIS
PD8980	MAML2	cna	gain	BASIS
PD9585	MAML2	cna	gain	BASIS
PD9702	MAML2	cna	amp	BASIS
TCGA-A2-A25B	MAP3K13	cna	amp	TCGA
TCGA-AN-A0XU	MAP3K13	cna	gain	TCGA
TCGA-BH-A0AW	MAP3K13	cna	gain	TCGA
TCGA-BH-A0C0	MAP3K13	cna	gain	TCGA
TCGA-BH-A1FU	MAP3K13	cna	hetloss	TCGA
TCGA-C8-A12L	MAP3K13	cna	gain	TCGA
TCGA-LL-A5YP	MAP3K13	cna	gain	TCGA
MB-0346	MAP3K13	cna	gain	METABRIC
MB-5070	MAP3K13	cna	hetloss	METABRIC
MB-5107	MAP3K13	cna	amp	METABRIC
MB-6098	MAP3K13	cna	gain	METABRIC
MB-7038	MAP3K13	cna	hetloss	METABRIC
MB-0420	MAP3K13	cna	gain	METABRIC
P-0002858-T01-IM3	MAP3K13	cna	amp	MSK-IMPACT
PD11327	MAP3K13	cna	homdel	BASIS
PD13299	MAP3K13	cna	gain	BASIS
PD13771	MAP3K13	cna	hetloss	BASIS
PD22355	MAP3K13	cna	gain	BASIS
PD23561	MAP3K13	cna	hetloss	BASIS
PD23562	MAP3K13	cna	gain	BASIS
PD23578	MAP3K13	cna	gain	BASIS
PD24186	MAP3K13	cna	amp	BASIS
PD24202	MAP3K13	cna	gain	BASIS
PD24206	MAP3K13	cna	homdel	BASIS
PD3905	MAP3K13	cna	gain	BASIS
PD4005	MAP3K13	cna	gain	BASIS
PD4107	MAP3K13	cna	gain	BASIS

PD4826	MAP3K13	cna	gain	BASIS
PD5945	MAP3K13	cna	amp	BASIS
PD5948	MAP3K13	cna	gain	BASIS
PD6406	MAP3K13	cna	gain	BASIS
PD6731	MAP3K13	cna	gain	BASIS
PD7067	MAP3K13	cna	gain	BASIS
PD7215	MAP3K13	cna	gain	BASIS
PD8621	MAP3K13	cna	gain	BASIS
PD8980	MAP3K13	cna	gain	BASIS
PD9004	MAP3K13	cna	gain	BASIS
PD9702	MAP3K13	cna	gain	BASIS
TCGA-A2-A25B	MAP3K6	cna	hetloss	TCGA
TCGA-AN-A0XU	MAP3K6	cna	hetloss	TCGA
TCGA-AO-A0JL	MAP3K6	cna	hetloss	TCGA
TCGA-BH-A0AW	MAP3K6	cna	gain	TCGA
TCGA-BH-A0C0	MAP3K6	cna	hetloss	TCGA
TCGA-BH-A18R	MAP3K6	cna	homdel	TCGA
TCGA-BH-A1FU	MAP3K6	cna	gain	TCGA
TCGA-C8-A12L	MAP3K6	cna	hetloss	TCGA
TCGA-D8-A27M	MAP3K6	cna	gain	TCGA
TCGA-E2-A1L7	MAP3K6	cna	hetloss	TCGA
TCGA-EW-A1OX	MAP3K6	cna	homdel	TCGA
TCGA-LL-A5YP	MAP3K6	cna	gain	TCGA
MB-0346	MAP3K6	cna	hetloss	METABRIC
MB-2827	MAP3K6	cna	hetloss	METABRIC
MB-5070	MAP3K6	cna	hetloss	METABRIC
MB-5465	MAP3K6	cna	hetloss	METABRIC
MB-6060	MAP3K6	cna	hetloss	METABRIC
MB-6098	MAP3K6	cna	hetloss	METABRIC
PD10014	MAP3K6	cna	gain	BASIS
PD11327	MAP3K6	cna	hetloss	BASIS
PD11742	MAP3K6	cna	hetloss	BASIS
PD13296	MAP3K6	cna	hetloss	BASIS
PD13771	MAP3K6	cna	hetloss	BASIS
PD22355	MAP3K6	cna	hetloss	BASIS
PD24186	MAP3K6	cna	gain	BASIS
PD24206	MAP3K6	cna	hetloss	BASIS
PD24337	MAP3K6	cna	hetloss	BASIS
PD3890	MAP3K6	cna	hetloss	BASIS
PD3905	MAP3K6	cna	gain	BASIS
PD4006	MAP3K6	cna	gain	BASIS
PD4826	MAP3K6	cna	gain	BASIS
PD5945	MAP3K6	cna	amp	BASIS
PD5948	MAP3K6	cna	gain	BASIS
PD7067	MAP3K6	cna	gain	BASIS
PD7215	MAP3K6	cna	gain	BASIS
PD9004	MAP3K6	cna	hetloss	BASIS

PD9585	MAP3K6	cna	hetloss	BASIS
PD9702	MAP3K6	cna	gain	BASIS
TCGA-A2-A25B	MSI1	cna	gain	TCGA
TCGA-AN-A0XU	MSI1	cna	hetloss	TCGA
TCGA-AO-A0JL	MSI1	cna	hetloss	TCGA
TCGA-BH-A0C0	MSI1	cna	hetloss	TCGA
TCGA-BH-A18R	MSI1	cna	gain	TCGA
TCGA-BH-A1FU	MSI1	cna	hetloss	TCGA
TCGA-D8-A27M	MSI1	cna	hetloss	TCGA
TCGA-E2-A1L7	MSI1	cna	hetloss	TCGA
TCGA-LL-A5YP	MSI1	cna	hetloss	TCGA
MB-0346	MSI1	cna	gain	METABRIC
MB-2827	MSI1	cna	hetloss	METABRIC
MB-5070	MSI1	cna	hetloss	METABRIC
MB-7048	MSI1	cna	hetloss	METABRIC
MTS-T0064	MSI1	cna	gain	METABRIC
PD11327	MSI1	cna	homdel	BASIS
PD13296	MSI1	cna	hetloss	BASIS
PD13297	MSI1	cna	gain	BASIS
PD13771	MSI1	cna	gain	BASIS
PD22355	MSI1	cna	hetloss	BASIS
PD23574	MSI1	cna	gain	BASIS
PD23578	MSI1	cna	hetloss	BASIS
PD24206	MSI1	cna	homdel	BASIS
PD3890	MSI1	cna	hetloss	BASIS
PD3905	MSI1	cna	gain	BASIS
PD4005	MSI1	cna	hetloss	BASIS
PD4006	MSI1	cna	amp	BASIS
PD4107	MSI1	cna	gain	BASIS
PD4826	MSI1	cna	gain	BASIS
PD4967	MSI1	cna	hetloss	BASIS
PD5935	MSI1	cna	gain	BASIS
PD5945	MSI1	cna	gain	BASIS
PD5948	MSI1	cna	gain	BASIS
PD6406	MSI1	cna	hetloss	BASIS
PD6731	MSI1	cna	hetloss	BASIS
PD7067	MSI1	cna	gain	BASIS
PD9004	MSI1	cna	hetloss	BASIS
PD9585	MSI1	cna	hetloss	BASIS
PD9702	MSI1	cna	gain	BASIS
TCGA-A2-A25B	NTRK3	cna	gain	TCGA
TCGA-AN-A0XU	NTRK3	cna	gain	TCGA
TCGA-AO-A0JL	NTRK3	cna	hetloss	TCGA
TCGA-BH-A0AW	NTRK3	cna	hetloss	TCGA
TCGA-BH-A0C0	NTRK3	cna	hetloss	TCGA
TCGA-D8-A27M	NTRK3	cna	hetloss	TCGA
TCGA-E2-A1L7	NTRK3	cna	gain	TCGA

TCGA-LL-A5YP	NTRK3	cna	gain	TCGA
MB-0346	NTRK3	cna	hetloss	METABRIC
MB-5070	NTRK3	cna	gain	METABRIC
MB-5465	NTRK3	cna	hetloss	METABRIC
MB-7038	NTRK3	cna	gain	METABRIC
MB-7048	NTRK3	cna	gain	METABRIC
MB-0420	NTRK3	cna	gain	METABRIC
MTS-T0064	NTRK3	cna	amp	METABRIC
PD11327	NTRK3	cna	gain	BASIS
PD11742	NTRK3	cna	hetloss	BASIS
PD13296	NTRK3	cna	gain	BASIS
PD13297	NTRK3	cna	hetloss	BASIS
PD13299	NTRK3	cna	gain	BASIS
PD23562	NTRK3	cna	gain	BASIS
PD23574	NTRK3	cna	gain	BASIS
PD23578	NTRK3	cna	gain	BASIS
PD24202	NTRK3	cna	hetloss	BASIS
PD24206	NTRK3	cna	gain	BASIS
PD24337	NTRK3	cna	gain	BASIS
PD3890	NTRK3	cna	hetloss	BASIS
PD3905	NTRK3	cna	gain	BASIS
PD4006	NTRK3	cna	gain	BASIS
PD4107	NTRK3	cna	gain	BASIS
PD5930	NTRK3	cna	gain	BASIS
PD5945	NTRK3	cna	gain	BASIS
PD6413	NTRK3	cna	hetloss	BASIS
PD7067	NTRK3	cna	gain	BASIS
PD8621	NTRK3	cna	gain	BASIS
PD8980	NTRK3	cna	gain	BASIS
PD9585	NTRK3	cna	hetloss	BASIS
PD9702	NTRK3	cna	gain	BASIS
TCGA-A2-A25B	NUP98	cna	hetloss	TCGA
TCGA-AN-A0XU	NUP98	cna	hetloss	TCGA
TCGA-AO-A0JL	NUP98	cna	hetloss	TCGA
TCGA-BH-A0C0	NUP98	cna	hetloss	TCGA
TCGA-C8-A12L	NUP98	cna	hetloss	TCGA
TCGA-E2-A1L7	NUP98	cna	homdel	TCGA
TCGA-LL-A5YP	NUP98	cna	hetloss	TCGA
MB-0346	NUP98	cna	hetloss	METABRIC
MB-5070	NUP98	cna	hetloss	METABRIC
MB-6060	NUP98	cna	hetloss	METABRIC
MB-6098	NUP98	cna	hetloss	METABRIC
MB-6271	NUP98	cna	gain	METABRIC
MB-7032	NUP98	cna	hetloss	METABRIC
MB-7048	NUP98	cna	hetloss	METABRIC
MB-0420	NUP98	cna	hetloss	METABRIC
PD10014	NUP98	cna	hetloss	BASIS

PD11327	NUP98	cna	homdel	BASIS
PD11742	NUP98	cna	hetloss	BASIS
PD13296	NUP98	cna	hetloss	BASIS
PD13297	NUP98	cna	hetloss	BASIS
PD22355	NUP98	cna	hetloss	BASIS
PD24202	NUP98	cna	hetloss	BASIS
PD24206	NUP98	cna	gain	BASIS
PD3890	NUP98	cna	hetloss	BASIS
PD3905	NUP98	cna	gain	BASIS
PD4005	NUP98	cna	hetloss	BASIS
PD4006	NUP98	cna	gain	BASIS
PD4107	NUP98	cna	gain	BASIS
PD4826	NUP98	cna	gain	BASIS
PD5935	NUP98	cna	gain	BASIS
PD5945	NUP98	cna	gain	BASIS
PD6406	NUP98	cna	hetloss	BASIS
PD6413	NUP98	cna	hetloss	BASIS
PD6731	NUP98	cna	hetloss	BASIS
PD8621	NUP98	cna	hetloss	BASIS
PD8980	NUP98	cna	hetloss	BASIS
PD9004	NUP98	cna	hetloss	BASIS
PD9585	NUP98	cna	hetloss	BASIS
TCGA-A2-A25B	PAK1	cna	hetloss	TCGA
TCGA-AN-A0XU	PAK1	cna	gain	TCGA
TCGA-AO-A0JL	PAK1	cna	hetloss	TCGA
TCGA-BH-A18R	PAK1	cna	amp	TCGA
TCGA-C8-A12L	PAK1	cna	gain	TCGA
TCGA-D8-A27M	PAK1	cna	gain	TCGA
TCGA-EW-A1OX	PAK1	cna	homdel	TCGA
TCGA-LL-A5YP	PAK1	cna	hetloss	TCGA
MB-0346	PAK1	cna	gain	METABRIC
MB-5107	PAK1	cna	amp	METABRIC
MB-6060	PAK1	cna	gain	METABRIC
MB-6271	PAK1	cna	gain	METABRIC
MB-7032	PAK1	cna	gain	METABRIC
MTS-T0064	PAK1	cna	gain	METABRIC
P-0002591-T01-IM3	PAK1	cna	amp	MSK-IMPACT
PD10014	PAK1	cna	gain	BASIS
PD11327	PAK1	cna	gain	BASIS
PD13771	PAK1	cna	hetloss	BASIS
PD22355	PAK1	cna	hetloss	BASIS
PD23574	PAK1	cna	gain	BASIS
PD24186	PAK1	cna	amp	BASIS
PD24206	PAK1	cna	gain	BASIS
PD3905	PAK1	cna	gain	BASIS
PD4005	PAK1	cna	gain	BASIS
PD4006	PAK1	cna	amp	BASIS

PD4107	PAK1	cna	gain	BASIS
PD4826	PAK1	cna	gain	BASIS
PD5930	PAK1	cna	gain	BASIS
PD5935	PAK1	cna	gain	BASIS
PD5945	PAK1	cna	amp	BASIS
PD5948	PAK1	cna	gain	BASIS
PD6413	PAK1	cna	gain	BASIS
PD7067	PAK1	cna	gain	BASIS
PD7215	PAK1	cna	gain	BASIS
PD8980	PAK1	cna	gain	BASIS
PD9004	PAK1	cna	gain	BASIS
PD9585	PAK1	cna	hetloss	BASIS
PD9702	PAK1	cna	gain	BASIS
TCGA-A2-A25B	PCM1	cna	hetloss	TCGA
TCGA-AN-A0XU	PCM1	cna	hetloss	TCGA
TCGA-AO-A0JL	PCM1	cna	hetloss	TCGA
TCGA-BH-A0C0	PCM1	cna	hetloss	TCGA
TCGA-BH-A1FU	PCM1	cna	homdel	TCGA
TCGA-C8-A12L	PCM1	cna	gain	TCGA
TCGA-D8-A27M	PCM1	cna	hetloss	TCGA
TCGA-E2-A1L7	PCM1	cna	hetloss	TCGA
TCGA-E9-A1NC	PCM1	cna	hetloss	TCGA
TCGA-LL-A5YP	PCM1	cna	hetloss	TCGA
MB-0346	PCM1	cna	gain	METABRIC
MB-2827	PCM1	cna	hetloss	METABRIC
MB-5070	PCM1	cna	hetloss	METABRIC
MB-5465	PCM1	cna	hetloss	METABRIC
MB-6060	PCM1	cna	hetloss	METABRIC
MB-6098	PCM1	cna	hetloss	METABRIC
MB-7038	PCM1	cna	amp	METABRIC
MB-0420	PCM1	cna	hetloss	METABRIC
MTS-T0064	PCM1	cna	hetloss	METABRIC
PD10014	PCM1	cna	hetloss	BASIS
PD13296	PCM1	cna	hetloss	BASIS
PD13299	PCM1	cna	hetloss	BASIS
PD13771	PCM1	cna	hetloss	BASIS
PD14442	PCM1	cna	hetloss	BASIS
PD22355	PCM1	cna	hetloss	BASIS
PD23562	PCM1	cna	hetloss	BASIS
PD23578	PCM1	cna	hetloss	BASIS
PD24186	PCM1	cna	gain	BASIS
PD24202	PCM1	cna	hetloss	BASIS
PD24337	PCM1	cna	hetloss	BASIS
PD3905	PCM1	cna	gain	BASIS
PD4005	PCM1	cna	hetloss	BASIS
PD4006	PCM1	cna	gain	BASIS
PD4826	PCM1	cna	hetloss	BASIS

PD6406	PCM1	cna	hetloss	BASIS
PD7067	PCM1	cna	hetloss	BASIS
PD8980	PCM1	cna	homdel	BASIS
PD9004	PCM1	cna	gain	BASIS
TCGA-AN-A0XU	PHF6	cna	hetloss	TCGA
TCGA-BH-A0AW	PHF6	cna	gain	TCGA
TCGA-C8-A12L	PHF6	cna	gain	TCGA
TCGA-E2-A1L7	PHF6	cna	hetloss	TCGA
TCGA-E9-A1NC	PHF6	cna	hetloss	TCGA
TCGA-LL-A5YP	PHF6	cna	gain	TCGA
MB-0346	PHF6	cna	hetloss	METABRIC
MB-2827	PHF6	cna	hetloss	METABRIC
MB-5465	PHF6	cna	hetloss	METABRIC
MB-6060	PHF6	cna	gain	METABRIC
MB-6098	PHF6	cna	hetloss	METABRIC
MB-7038	PHF6	cna	gain	METABRIC
PD10014	PHF6	cna	hetloss	BASIS
PD13296	PHF6	cna	amp	BASIS
PD13299	PHF6	cna	amp	BASIS
PD13771	PHF6	cna	gain	BASIS
PD14442	PHF6	cna	gain	BASIS
PD23562	PHF6	cna	hetloss	BASIS
PD23574	PHF6	cna	gain	BASIS
PD23578	PHF6	cna	hetloss	BASIS
PD24202	PHF6	cna	hetloss	BASIS
PD24206	PHF6	cna	gain	BASIS
PD3890	PHF6	cna	hetloss	BASIS
PD3905	PHF6	cna	gain	BASIS
PD4006	PHF6	cna	gain	BASIS
PD4107	PHF6	cna	gain	BASIS
PD4826	PHF6	cna	gain	BASIS
PD4967	PHF6	cna	gain	BASIS
PD5930	PHF6	cna	gain	BASIS
PD5935	PHF6	cna	gain	BASIS
PD5945	PHF6	cna	gain	BASIS
PD5948	PHF6	cna	gain	BASIS
PD6406	PHF6	cna	gain	BASIS
PD6413	PHF6	cna	gain	BASIS
PD7215	PHF6	cna	gain	BASIS
PD8621	PHF6	cna	amp	BASIS
PD8980	PHF6	cna	hetloss	BASIS
PD9004	PHF6	cna	gain	BASIS
TCGA-A2-A25B	POT1	cna	gain	TCGA
TCGA-AO-A0JL	POT1	cna	gain	TCGA
TCGA-BH-A0AW	POT1	cna	gain	TCGA
TCGA-C8-A12L	POT1	cna	hetloss	TCGA
TCGA-D8-A27M	POT1	cna	gain	TCGA

TCGA-E2-A1L7	POT1	cna	hetloss	TCGA
TCGA-E9-A1NC	POT1	cna	gain	TCGA
TCGA-LL-A5YP	POT1	cna	gain	TCGA
MB-0346	POT1	cna	hetloss	METABRIC
MB-5070	POT1	cna	hetloss	METABRIC
MB-5465	POT1	cna	gain	METABRIC
MB-7038	POT1	cna	gain	METABRIC
MB-7048	POT1	cna	gain	METABRIC
MTS-T0064	POT1	cna	hetloss	METABRIC
PD11327	POT1	cna	gain	BASIS
PD13296	POT1	cna	hetloss	BASIS
PD13297	POT1	cna	gain	BASIS
PD13299	POT1	cna	gain	BASIS
PD22355	POT1	cna	hetloss	BASIS
PD23574	POT1	cna	gain	BASIS
PD23578	POT1	cna	gain	BASIS
PD24186	POT1	cna	amp	BASIS
PD24337	POT1	cna	hetloss	BASIS
PD3890	POT1	cna	hetloss	BASIS
PD3905	POT1	cna	gain	BASIS
PD4107	POT1	cna	gain	BASIS
PD4826	POT1	cna	gain	BASIS
PD5930	POT1	cna	gain	BASIS
PD5935	POT1	cna	gain	BASIS
PD5945	POT1	cna	amp	BASIS
PD5948	POT1	cna	gain	BASIS
PD6406	POT1	cna	gain	BASIS
PD7067	POT1	cna	gain	BASIS
PD7215	POT1	cna	gain	BASIS
PD8621	POT1	cna	gain	BASIS
PD9004	POT1	cna	gain	BASIS
PD9585	POT1	cna	gain	BASIS
PD9702	POT1	cna	amp	BASIS
TCGA-A2-A25B	PSIP1	cna	hetloss	TCGA
TCGA-AO-A0JL	PSIP1	cna	gain	TCGA
TCGA-BH-A0AW	PSIP1	cna	hetloss	TCGA
TCGA-BH-A1FU	PSIP1	cna	hetloss	TCGA
TCGA-C8-A12L	PSIP1	cna	hetloss	TCGA
TCGA-E2-A1L7	PSIP1	cna	hetloss	TCGA
TCGA-E9-A1NC	PSIP1	cna	amp	TCGA
MB-0346	PSIP1	cna	hetloss	METABRIC
MB-5070	PSIP1	cna	hetloss	METABRIC
MB-6098	PSIP1	cna	hetloss	METABRIC
MB-0420	PSIP1	cna	hetloss	METABRIC
MTS-T0064	PSIP1	cna	amp	METABRIC
PD11327	PSIP1	cna	homdel	BASIS
PD13296	PSIP1	cna	gain	BASIS

PD13297	PSIP1	cna	hetloss	BASIS
PD13299	PSIP1	cna	amp	BASIS
PD22355	PSIP1	cna	gain	BASIS
PD23574	PSIP1	cna	amp	BASIS
PD24186	PSIP1	cna	gain	BASIS
PD24202	PSIP1	cna	hetloss	BASIS
PD24337	PSIP1	cna	hetloss	BASIS
PD3905	PSIP1	cna	gain	BASIS
PD4005	PSIP1	cna	hetloss	BASIS
PD4006	PSIP1	cna	gain	BASIS
PD4107	PSIP1	cna	gain	BASIS
PD4826	PSIP1	cna	gain	BASIS
PD4967	PSIP1	cna	hetloss	BASIS
PD5945	PSIP1	cna	amp	BASIS
PD5948	PSIP1	cna	hetloss	BASIS
PD6406	PSIP1	cna	homdel	BASIS
PD6413	PSIP1	cna	gain	BASIS
PD7067	PSIP1	cna	amp	BASIS
PD7215	PSIP1	cna	hetloss	BASIS
PD8621	PSIP1	cna	gain	BASIS
PD8980	PSIP1	cna	hetloss	BASIS
PD9004	PSIP1	cna	gain	BASIS
PD9585	PSIP1	cna	hetloss	BASIS
PD9702	PSIP1	cna	gain	BASIS
TCGA-AN-A0XU	RAD50	cna	hetloss	TCGA
TCGA-AO-A0JL	RAD50	cna	hetloss	TCGA
TCGA-BH-A0C0	RAD50	cna	hetloss	TCGA
TCGA-BH-A1FU	RAD50	cna	hetloss	TCGA
TCGA-C8-A12L	RAD50	cna	hetloss	TCGA
TCGA-D8-A27M	RAD50	cna	hetloss	TCGA
TCGA-E2-A1L7	RAD50	cna	gain	TCGA
TCGA-E9-A1NC	RAD50	cna	hetloss	TCGA
TCGA-LL-A5YP	RAD50	cna	hetloss	TCGA
MB-0346	RAD50	cna	gain	METABRIC
MB-2827	RAD50	cna	hetloss	METABRIC
MB-5070	RAD50	cna	hetloss	METABRIC
MB-5107	RAD50	cna	hetloss	METABRIC
MB-5465	RAD50	cna	hetloss	METABRIC
MB-6060	RAD50	cna	hetloss	METABRIC
MB-6098	RAD50	cna	hetloss	METABRIC
MB-7038	RAD50	cna	hetloss	METABRIC
MB-0420	RAD50	cna	hetloss	METABRIC
PD10014	RAD50	cna	hetloss	BASIS
PD13296	RAD50	cna	hetloss	BASIS
PD13297	RAD50	cna	hetloss	BASIS
PD13771	RAD50	cna	gain	BASIS
PD22355	RAD50	cna	hetloss	BASIS

PD23562	RAD50	cna	hetloss	BASIS
PD23578	RAD50	cna	hetloss	BASIS
PD24202	RAD50	cna	hetloss	BASIS
PD24206	RAD50	cna	gain	BASIS
PD24337	RAD50	cna	hetloss	BASIS
PD3890	RAD50	cna	hetloss	BASIS
PD3905	RAD50	cna	gain	BASIS
PD4005	RAD50	cna	hetloss	BASIS
PD5935	RAD50	cna	gain	BASIS
PD5945	RAD50	cna	gain	BASIS
PD6406	RAD50	cna	hetloss	BASIS
PD6413	RAD50	cna	hetloss	BASIS
PD7067	RAD50	cna	gain	BASIS
PD8980	RAD50	cna	hetloss	BASIS
PD9585	RAD50	cna	hetloss	BASIS
TCGA-A2-A25B	RAD51D	cna	amp	TCGA
TCGA-AO-A0JL	RAD51D	cna	gain	TCGA
TCGA-BH-A0AW	RAD51D	cna	gain	TCGA
TCGA-BH-A0C0	RAD51D	cna	gain	TCGA
TCGA-BH-A18R	RAD51D	cna	homdel	TCGA
TCGA-C8-A12L	RAD51D	cna	gain	TCGA
TCGA-D8-A27M	RAD51D	cna	hetloss	TCGA
TCGA-E2-A1L7	RAD51D	cna	hetloss	TCGA
TCGA-E9-A1NC	RAD51D	cna	hetloss	TCGA
MB-0346	RAD51D	cna	hetloss	METABRIC
MB-2827	RAD51D	cna	hetloss	METABRIC
MB-5070	RAD51D	cna	gain	METABRIC
MB-5465	RAD51D	cna	hetloss	METABRIC
MB-6060	RAD51D	cna	hetloss	METABRIC
MB-6098	RAD51D	cna	hetloss	METABRIC
MB-6271	RAD51D	cna	hetloss	METABRIC
MB-7038	RAD51D	cna	hetloss	METABRIC
MB-7048	RAD51D	cna	hetloss	METABRIC
MB-0420	RAD51D	cna	hetloss	METABRIC
PD11327	RAD51D	cna	gain	BASIS
PD13297	RAD51D	cna	hetloss	BASIS
PD13299	RAD51D	cna	gain	BASIS
PD23561	RAD51D	cna	amp	BASIS
PD24206	RAD51D	cna	hetloss	BASIS
PD24337	RAD51D	cna	hetloss	BASIS
PD3890	RAD51D	cna	hetloss	BASIS
PD4005	RAD51D	cna	hetloss	BASIS
PD4967	RAD51D	cna	hetloss	BASIS
PD5945	RAD51D	cna	gain	BASIS
PD5948	RAD51D	cna	gain	BASIS
PD6406	RAD51D	cna	hetloss	BASIS
PD6413	RAD51D	cna	hetloss	BASIS

PD6731	RAD51D	cna	hetloss	BASIS
PD7067	RAD51D	cna	gain	BASIS
PD7215	RAD51D	cna	gain	BASIS
PD9004	RAD51D	cna	gain	BASIS
PD9585	RAD51D	cna	hetloss	BASIS
PD9702	RAD51D	cna	gain	BASIS
TCGA-AN-A0XU	RBMX	cna	hetloss	TCGA
TCGA-BH-A0AW	RBMX	cna	gain	TCGA
TCGA-C8-A12L	RBMX	cna	gain	TCGA
TCGA-E2-A1L7	RBMX	cna	hetloss	TCGA
TCGA-E9-A1NC	RBMX	cna	hetloss	TCGA
TCGA-LL-A5YP	RBMX	cna	gain	TCGA
MB-0346	RBMX	cna	hetloss	METABRIC
MB-2827	RBMX	cna	hetloss	METABRIC
MB-5465	RBMX	cna	hetloss	METABRIC
MB-6060	RBMX	cna	gain	METABRIC
MB-6098	RBMX	cna	hetloss	METABRIC
MB-7038	RBMX	cna	gain	METABRIC
PD10014	RBMX	cna	hetloss	BASIS
PD13296	RBMX	cna	gain	BASIS
PD13299	RBMX	cna	amp	BASIS
PD13771	RBMX	cna	gain	BASIS
PD14442	RBMX	cna	gain	BASIS
PD23562	RBMX	cna	hetloss	BASIS
PD23574	RBMX	cna	gain	BASIS
PD23578	RBMX	cna	hetloss	BASIS
PD24202	RBMX	cna	hetloss	BASIS
PD24206	RBMX	cna	gain	BASIS
PD3890	RBMX	cna	hetloss	BASIS
PD3905	RBMX	cna	gain	BASIS
PD4107	RBMX	cna	gain	BASIS
PD4826	RBMX	cna	gain	BASIS
PD4967	RBMX	cna	gain	BASIS
PD5930	RBMX	cna	gain	BASIS
PD5935	RBMX	cna	gain	BASIS
PD5945	RBMX	cna	amp	BASIS
PD5948	RBMX	cna	gain	BASIS
PD6406	RBMX	cna	gain	BASIS
PD6413	RBMX	cna	gain	BASIS
PD7215	RBMX	cna	gain	BASIS
PD8621	RBMX	cna	amp	BASIS
PD8980	RBMX	cna	hetloss	BASIS
PD9004	RBMX	cna	gain	BASIS
PD9702	RBMX	cna	gain	BASIS
TCGA-A2-A25B	REL	cna	hetloss	TCGA
TCGA-AN-A0XU	REL	cna	gain	TCGA
TCGA-AO-A0JL	REL	cna	gain	TCGA

TCGA-BH-A0AW	REL	cna	gain	TCGA
TCGA-BH-A0C0	REL	cna	gain	TCGA
TCGA-D8-A27M	REL	cna	gain	TCGA
TCGA-E9-A1NC	REL	cna	gain	TCGA
TCGA-LL-A5YP	REL	cna	gain	TCGA
MB-2827	REL	cna	hetloss	METABRIC
MB-5465	REL	cna	amp	METABRIC
MB-6271	REL	cna	hetloss	METABRIC
MB-7038	REL	cna	gain	METABRIC
MB-7048	REL	cna	hetloss	METABRIC
PD10014	REL	cna	gain	BASIS
PD11327	REL	cna	hetloss	BASIS
PD13296	REL	cna	gain	BASIS
PD13297	REL	cna	gain	BASIS
PD13299	REL	cna	gain	BASIS
PD22355	REL	cna	gain	BASIS
PD23562	REL	cna	gain	BASIS
PD23578	REL	cna	gain	BASIS
PD24186	REL	cna	gain	BASIS
PD24202	REL	cna	gain	BASIS
PD24206	REL	cna	hetloss	BASIS
PD3890	REL	cna	hetloss	BASIS
PD3905	REL	cna	gain	BASIS
PD4006	REL	cna	amp	BASIS
PD4107	REL	cna	gain	BASIS
PD4967	REL	cna	hetloss	BASIS
PD5930	REL	cna	gain	BASIS
PD5935	REL	cna	gain	BASIS
PD5945	REL	cna	amp	BASIS
PD5948	REL	cna	gain	BASIS
PD7067	REL	cna	gain	BASIS
PD7215	REL	cna	gain	BASIS
PD8621	REL	cna	gain	BASIS
PD9004	REL	cna	gain	BASIS
PD9702	REL	cna	gain	BASIS
TCGA-A2-A25B	RELN	cna	gain	TCGA
TCGA-AN-A0XU	RELN	cna	hetloss	TCGA
TCGA-AO-A0JL	RELN	cna	gain	TCGA
TCGA-BH-A0AW	RELN	cna	gain	TCGA
TCGA-C8-A12L	RELN	cna	hetloss	TCGA
TCGA-D8-A27M	RELN	cna	gain	TCGA
TCGA-E2-A1L7	RELN	cna	hetloss	TCGA
TCGA-E9-A1NC	RELN	cna	gain	TCGA
TCGA-LL-A5YP	RELN	cna	gain	TCGA
MB-0346	RELN	cna	hetloss	METABRIC
MB-5465	RELN	cna	hetloss	METABRIC
MB-6098	RELN	cna	hetloss	METABRIC

MB-7038	RELN	cna	gain	METABRIC
MB-7048	RELN	cna	gain	METABRIC
PD11327	RELN	cna	amp	BASIS
PD13296	RELN	cna	hetloss	BASIS
PD22355	RELN	cna	hetloss	BASIS
PD23574	RELN	cna	gain	BASIS
PD23578	RELN	cna	hetloss	BASIS
PD24186	RELN	cna	amp	BASIS
PD24337	RELN	cna	hetloss	BASIS
PD3890	RELN	cna	hetloss	BASIS
PD3905	RELN	cna	gain	BASIS
PD4005	RELN	cna	hetloss	BASIS
PD4006	RELN	cna	amp	BASIS
PD4107	RELN	cna	gain	BASIS
PD4826	RELN	cna	gain	BASIS
PD5930	RELN	cna	gain	BASIS
PD5935	RELN	cna	gain	BASIS
PD5945	RELN	cna	amp	BASIS
PD5948	RELN	cna	gain	BASIS
PD6406	RELN	cna	gain	BASIS
PD7067	RELN	cna	gain	BASIS
PD7215	RELN	cna	gain	BASIS
PD8621	RELN	cna	gain	BASIS
PD9004	RELN	cna	gain	BASIS
PD9585	RELN	cna	hetloss	BASIS
PD9702	RELN	cna	gain	BASIS
TCGA-A2-A25B	RPA1	cna	hetloss	TCGA
TCGA-AN-A0XU	RPA1	cna	hetloss	TCGA
TCGA-AO-A0JL	RPA1	cna	hetloss	TCGA
TCGA-BH-A0AW	RPA1	cna	hetloss	TCGA
TCGA-BH-A0C0	RPA1	cna	hetloss	TCGA
TCGA-C8-A12L	RPA1	cna	hetloss	TCGA
TCGA-D8-A27M	RPA1	cna	hetloss	TCGA
TCGA-E2-A1L7	RPA1	cna	hetloss	TCGA
TCGA-E9-A1NC	RPA1	cna	hetloss	TCGA
TCGA-EW-A1OX	RPA1	cna	hetloss	TCGA
TCGA-LL-A5YP	RPA1	cna	hetloss	TCGA
MB-0346	RPA1	cna	hetloss	METABRIC
MB-2827	RPA1	cna	hetloss	METABRIC
MB-5107	RPA1	cna	hetloss	METABRIC
MB-6098	RPA1	cna	gain	METABRIC
MB-6271	RPA1	cna	hetloss	METABRIC
MB-7032	RPA1	cna	gain	METABRIC
MB-7048	RPA1	cna	hetloss	METABRIC
MB-0420	RPA1	cna	hetloss	METABRIC
PD10014	RPA1	cna	hetloss	BASIS
PD11742	RPA1	cna	hetloss	BASIS

PD13296	RPA1	cna	hetloss	BASIS
PD13771	RPA1	cna	hetloss	BASIS
PD24202	RPA1	cna	hetloss	BASIS
PD24206	RPA1	cna	hetloss	BASIS
PD24337	RPA1	cna	hetloss	BASIS
PD3890	RPA1	cna	hetloss	BASIS
PD3905	RPA1	cna	gain	BASIS
PD4005	RPA1	cna	hetloss	BASIS
PD4826	RPA1	cna	hetloss	BASIS
PD4967	RPA1	cna	hetloss	BASIS
PD5945	RPA1	cna	gain	BASIS
PD6406	RPA1	cna	hetloss	BASIS
PD6413	RPA1	cna	hetloss	BASIS
PD6731	RPA1	cna	hetloss	BASIS
PD7067	RPA1	cna	gain	BASIS
PD9585	RPA1	cna	hetloss	BASIS
PD9702	RPA1	cna	gain	BASIS
TCGA-A2-A25B	SET	cna	gain	TCGA
TCGA-AN-A0XU	SET	cna	hetloss	TCGA
TCGA-AO-A0JL	SET	cna	hetloss	TCGA
TCGA-BH-A0C0	SET	cna	gain	TCGA
TCGA-C8-A12L	SET	cna	gain	TCGA
TCGA-E2-A1L7	SET	cna	gain	TCGA
TCGA-E9-A1NC	SET	cna	hetloss	TCGA
TCGA-LL-A5YP	SET	cna	hetloss	TCGA
MB-0346	SET	cna	hetloss	METABRIC
MB-2827	SET	cna	hetloss	METABRIC
MB-5070	SET	cna	hetloss	METABRIC
MB-5107	SET	cna	gain	METABRIC
MB-7038	SET	cna	hetloss	METABRIC
MB-0420	SET	cna	hetloss	METABRIC
PD10014	SET	cna	gain	BASIS
PD11327	SET	cna	hetloss	BASIS
PD13297	SET	cna	hetloss	BASIS
PD13299	SET	cna	gain	BASIS
PD22355	SET	cna	gain	BASIS
PD23578	SET	cna	hetloss	BASIS
PD24186	SET	cna	amp	BASIS
PD24202	SET	cna	hetloss	BASIS
PD24337	SET	cna	hetloss	BASIS
PD3890	SET	cna	hetloss	BASIS
PD3905	SET	cna	gain	BASIS
PD4005	SET	cna	hetloss	BASIS
PD4006	SET	cna	gain	BASIS
PD4107	SET	cna	hetloss	BASIS
PD5930	SET	cna	hetloss	BASIS
PD5945	SET	cna	gain	BASIS

PD5948	SET	cna	gain	BASIS
PD6406	SET	cna	hetloss	BASIS
PD6731	SET	cna	hetloss	BASIS
PD7067	SET	cna	gain	BASIS
PD7215	SET	cna	gain	BASIS
PD8621	SET	cna	gain	BASIS
PD8980	SET	cna	gain	BASIS
PD9004	SET	cna	hetloss	BASIS
TCGA-A2-A25B	SYNE1	cna	hetloss	TCGA
TCGA-AN-A0XU	SYNE1	cna	gain	TCGA
TCGA-AO-A0JL	SYNE1	cna	hetloss	TCGA
TCGA-BH-A0AW	SYNE1	cna	amp	TCGA
TCGA-BH-A0C0	SYNE1	cna	gain	TCGA
TCGA-C8-A12L	SYNE1	cna	gain	TCGA
TCGA-D8-A27M	SYNE1	cna	hetloss	TCGA
MB-2827	SYNE1	cna	hetloss	METABRIC
MB-5070	SYNE1	cna	gain	METABRIC
MB-5107	SYNE1	cna	hetloss	METABRIC
MB-6098	SYNE1	cna	hetloss	METABRIC
MB-7038	SYNE1	cna	amp	METABRIC
MB-7048	SYNE1	cna	hetloss	METABRIC
MB-0420	SYNE1	cna	hetloss	METABRIC
PD11327	SYNE1	cna	gain	BASIS
PD13296	SYNE1	cna	homdel	BASIS
PD13297	SYNE1	cna	hetloss	BASIS
PD13299	SYNE1	cna	gain	BASIS
PD23562	SYNE1	cna	gain	BASIS
PD23574	SYNE1	cna	gain	BASIS
PD24186	SYNE1	cna	gain	BASIS
PD3890	SYNE1	cna	hetloss	BASIS
PD3905	SYNE1	cna	gain	BASIS
PD4005	SYNE1	cna	hetloss	BASIS
PD4006	SYNE1	cna	gain	BASIS
PD4826	SYNE1	cna	gain	BASIS
PD4967	SYNE1	cna	hetloss	BASIS
PD5935	SYNE1	cna	gain	BASIS
PD5945	SYNE1	cna	amp	BASIS
PD6406	SYNE1	cna	hetloss	BASIS
PD6413	SYNE1	cna	hetloss	BASIS
PD6731	SYNE1	cna	hetloss	BASIS
PD7067	SYNE1	cna	amp	BASIS
PD7215	SYNE1	cna	gain	BASIS
PD8980	SYNE1	cna	gain	BASIS
PD9004	SYNE1	cna	gain	BASIS
PD9585	SYNE1	cna	hetloss	BASIS
PD9702	SYNE1	cna	gain	BASIS
TCGA-AN-A0XU	TAF15	cna	gain	TCGA

TCGA-AO-A0JL	TAF15	cna	gain	TCGA
TCGA-BH-A0AW	TAF15	cna	gain	TCGA
TCGA-BH-A0C0	TAF15	cna	gain	TCGA
TCGA-BH-A18R	TAF15	cna	homdel	TCGA
TCGA-C8-A12L	TAF15	cna	hetloss	TCGA
TCGA-D8-A27M	TAF15	cna	hetloss	TCGA
TCGA-E2-A1L7	TAF15	cna	hetloss	TCGA
TCGA-E9-A1NC	TAF15	cna	hetloss	TCGA
MB-0346	TAF15	cna	hetloss	METABRIC
MB-2827	TAF15	cna	hetloss	METABRIC
MB-5070	TAF15	cna	gain	METABRIC
MB-5465	TAF15	cna	hetloss	METABRIC
MB-6060	TAF15	cna	hetloss	METABRIC
MB-6098	TAF15	cna	hetloss	METABRIC
MB-6271	TAF15	cna	hetloss	METABRIC
MB-7038	TAF15	cna	hetloss	METABRIC
MB-7048	TAF15	cna	hetloss	METABRIC
MB-0420	TAF15	cna	hetloss	METABRIC
PD13297	TAF15	cna	hetloss	BASIS
PD13299	TAF15	cna	gain	BASIS
PD13771	TAF15	cna	hetloss	BASIS
PD23561	TAF15	cna	gain	BASIS
PD24206	TAF15	cna	hetloss	BASIS
PD24337	TAF15	cna	hetloss	BASIS
PD3890	TAF15	cna	hetloss	BASIS
PD4005	TAF15	cna	hetloss	BASIS
PD4967	TAF15	cna	hetloss	BASIS
PD5945	TAF15	cna	gain	BASIS
PD5948	TAF15	cna	gain	BASIS
PD6406	TAF15	cna	hetloss	BASIS
PD6413	TAF15	cna	hetloss	BASIS
PD6731	TAF15	cna	hetloss	BASIS
PD7067	TAF15	cna	gain	BASIS
PD7215	TAF15	cna	gain	BASIS
PD9004	TAF15	cna	gain	BASIS
PD9585	TAF15	cna	hetloss	BASIS
PD9702	TAF15	cna	gain	BASIS
TCGA-AN-A0XU	TCF12	cna	hetloss	TCGA
TCGA-AO-A0JL	TCF12	cna	hetloss	TCGA
TCGA-BH-A0AW	TCF12	cna	hetloss	TCGA
TCGA-BH-A0C0	TCF12	cna	hetloss	TCGA
TCGA-C8-A12L	TCF12	cna	gain	TCGA
TCGA-D8-A27M	TCF12	cna	hetloss	TCGA
TCGA-E2-A1L7	TCF12	cna	hetloss	TCGA
TCGA-E9-A1NC	TCF12	cna	hetloss	TCGA
TCGA-LL-A5YP	TCF12	cna	hetloss	TCGA
MB-0346	TCF12	cna	gain	METABRIC

MB-5070	TCF12	cna	hetloss	METABRIC
MB-5107	TCF12	cna	gain	METABRIC
MB-6060	TCF12	cna	gain	METABRIC
MB-6098	TCF12	cna	hetloss	METABRIC
MB-7048	TCF12	cna	hetloss	METABRIC
PD10014	TCF12	cna	hetloss	BASIS
PD11327	TCF12	cna	gain	BASIS
PD11742	TCF12	cna	hetloss	BASIS
PD13296	TCF12	cna	homdel	BASIS
PD13297	TCF12	cna	hetloss	BASIS
PD22355	TCF12	cna	hetloss	BASIS
PD24202	TCF12	cna	hetloss	BASIS
PD3890	TCF12	cna	hetloss	BASIS
PD3905	TCF12	cna	gain	BASIS
PD4005	TCF12	cna	hetloss	BASIS
PD4006	TCF12	cna	gain	BASIS
PD4107	TCF12	cna	hetloss	BASIS
PD5930	TCF12	cna	hetloss	BASIS
PD5935	TCF12	cna	gain	BASIS
PD5945	TCF12	cna	gain	BASIS
PD5948	TCF12	cna	hetloss	BASIS
PD6406	TCF12	cna	hetloss	BASIS
PD6413	TCF12	cna	hetloss	BASIS
PD7067	TCF12	cna	gain	BASIS
PD7215	TCF12	cna	gain	BASIS
PD8980	TCF12	cna	hetloss	BASIS
PD9585	TCF12	cna	hetloss	BASIS
PD9702	TCF12	cna	gain	BASIS
TCGA-A2-A25B	TCF7L2	cna	hetloss	TCGA
TCGA-AN-A0XU	TCF7L2	cna	hetloss	TCGA
TCGA-AO-A0JL	TCF7L2	cna	hetloss	TCGA
TCGA-BH-A18R	TCF7L2	cna	gain	TCGA
TCGA-C8-A12L	TCF7L2	cna	hetloss	TCGA
TCGA-D8-A27M	TCF7L2	cna	hetloss	TCGA
TCGA-E2-A1L7	TCF7L2	cna	hetloss	TCGA
TCGA-E9-A1NC	TCF7L2	cna	hetloss	TCGA
TCGA-LL-A5YP	TCF7L2	cna	gain	TCGA
MB-0346	TCF7L2	cna	hetloss	METABRIC
MB-2827	TCF7L2	cna	hetloss	METABRIC
MB-5070	TCF7L2	cna	hetloss	METABRIC
MB-5107	TCF7L2	cna	hetloss	METABRIC
MB-6098	TCF7L2	cna	hetloss	METABRIC
MB-7038	TCF7L2	cna	hetloss	METABRIC
MB-0420	TCF7L2	cna	hetloss	METABRIC
PD10014	TCF7L2	cna	hetloss	BASIS
PD11742	TCF7L2	cna	hetloss	BASIS
PD13296	TCF7L2	cna	hetloss	BASIS

PD23562	TCF7L2	cna	gain	BASIS
PD23574	TCF7L2	cna	gain	BASIS
PD23578	TCF7L2	cna	hetloss	BASIS
PD24206	TCF7L2	cna	gain	BASIS
PD24337	TCF7L2	cna	hetloss	BASIS
PD3905	TCF7L2	cna	gain	BASIS
PD4005	TCF7L2	cna	hetloss	BASIS
PD4006	TCF7L2	cna	gain	BASIS
PD4107	TCF7L2	cna	gain	BASIS
PD5935	TCF7L2	cna	gain	BASIS
PD5945	TCF7L2	cna	gain	BASIS
PD5948	TCF7L2	cna	gain	BASIS
PD6731	TCF7L2	cna	hetloss	BASIS
PD7067	TCF7L2	cna	gain	BASIS
PD7215	TCF7L2	cna	gain	BASIS
PD8621	TCF7L2	cna	gain	BASIS
PD8980	TCF7L2	cna	hetloss	BASIS
PD9585	TCF7L2	cna	hetloss	BASIS
PD9702	TCF7L2	cna	gain	BASIS
TCGA-A2-A25B	TCL1A	cna	hetloss	TCGA
TCGA-AN-A0XU	TCL1A	cna	gain	TCGA
TCGA-AO-A0JL	TCL1A	cna	hetloss	TCGA
TCGA-BH-A0AW	TCL1A	cna	hetloss	TCGA
TCGA-BH-A1FU	TCL1A	cna	gain	TCGA
TCGA-C8-A12L	TCL1A	cna	gain	TCGA
TCGA-D8-A27M	TCL1A	cna	hetloss	TCGA
TCGA-E2-A1L7	TCL1A	cna	gain	TCGA
TCGA-E9-A1NC	TCL1A	cna	hetloss	TCGA
TCGA-LL-A5YP	TCL1A	cna	hetloss	TCGA
MB-0346	TCL1A	cna	gain	METABRIC
MB-2827	TCL1A	cna	hetloss	METABRIC
MB-5070	TCL1A	cna	hetloss	METABRIC
MB-5107	TCL1A	cna	hetloss	METABRIC
MB-6098	TCL1A	cna	hetloss	METABRIC
MTS-T0064	TCL1A	cna	hetloss	METABRIC
PD10014	TCL1A	cna	hetloss	BASIS
PD11742	TCL1A	cna	hetloss	BASIS
PD13296	TCL1A	cna	gain	BASIS
PD13297	TCL1A	cna	hetloss	BASIS
PD13771	TCL1A	cna	hetloss	BASIS
PD23562	TCL1A	cna	gain	BASIS
PD23578	TCL1A	cna	hetloss	BASIS
PD24202	TCL1A	cna	hetloss	BASIS
PD24206	TCL1A	cna	gain	BASIS
PD24337	TCL1A	cna	hetloss	BASIS
PD3890	TCL1A	cna	hetloss	BASIS
PD3905	TCL1A	cna	gain	BASIS

PD4005	TCL1A	cna	hetloss	BASIS
PD4006	TCL1A	cna	gain	BASIS
PD4826	TCL1A	cna	gain	BASIS
PD5945	TCL1A	cna	gain	BASIS
PD5948	TCL1A	cna	gain	BASIS
PD6406	TCL1A	cna	hetloss	BASIS
PD6413	TCL1A	cna	hetloss	BASIS
PD7067	TCL1A	cna	gain	BASIS
PD8980	TCL1A	cna	hetloss	BASIS
PD9702	TCL1A	cna	gain	BASIS
TCGA-A2-A25B	TCL6	cna	hetloss	TCGA
TCGA-AN-A0XU	TCL6	cna	gain	TCGA
TCGA-AO-A0JL	TCL6	cna	hetloss	TCGA
TCGA-BH-A0AW	TCL6	cna	hetloss	TCGA
TCGA-BH-A1FU	TCL6	cna	gain	TCGA
TCGA-C8-A12L	TCL6	cna	gain	TCGA
TCGA-D8-A27M	TCL6	cna	hetloss	TCGA
TCGA-E2-A1L7	TCL6	cna	gain	TCGA
TCGA-E9-A1NC	TCL6	cna	hetloss	TCGA
TCGA-LL-A5YP	TCL6	cna	hetloss	TCGA
MB-0346	TCL6	cna	gain	METABRIC
MB-2827	TCL6	cna	hetloss	METABRIC
MB-5070	TCL6	cna	hetloss	METABRIC
MB-5107	TCL6	cna	hetloss	METABRIC
MB-6098	TCL6	cna	hetloss	METABRIC
MTS-T0064	TCL6	cna	hetloss	METABRIC
PD10014	TCL6	cna	hetloss	BASIS
PD11742	TCL6	cna	hetloss	BASIS
PD13296	TCL6	cna	gain	BASIS
PD13297	TCL6	cna	hetloss	BASIS
PD13771	TCL6	cna	hetloss	BASIS
PD23562	TCL6	cna	gain	BASIS
PD23578	TCL6	cna	hetloss	BASIS
PD24202	TCL6	cna	hetloss	BASIS
PD24206	TCL6	cna	gain	BASIS
PD24337	TCL6	cna	hetloss	BASIS
PD3890	TCL6	cna	hetloss	BASIS
PD3905	TCL6	cna	gain	BASIS
PD4005	TCL6	cna	hetloss	BASIS
PD4006	TCL6	cna	gain	BASIS
PD4826	TCL6	cna	gain	BASIS
PD5945	TCL6	cna	gain	BASIS
PD5948	TCL6	cna	gain	BASIS
PD6406	TCL6	cna	hetloss	BASIS
PD6413	TCL6	cna	hetloss	BASIS
PD7067	TCL6	cna	gain	BASIS
PD8980	TCL6	cna	hetloss	BASIS

PD9702	TCL6	cna	gain	BASIS
TCGA-A2-A25B	TLR4	cna	gain	TCGA
TCGA-AN-A0XU	TLR4	cna	hetloss	TCGA
TCGA-AO-A0JL	TLR4	cna	hetloss	TCGA
TCGA-BH-A0C0	TLR4	cna	gain	TCGA
TCGA-C8-A12L	TLR4	cna	hetloss	TCGA
TCGA-E2-A1L7	TLR4	cna	gain	TCGA
TCGA-E9-A1NC	TLR4	cna	hetloss	TCGA
TCGA-LL-A5YP	TLR4	cna	hetloss	TCGA
MB-0346	TLR4	cna	hetloss	METABRIC
MB-5070	TLR4	cna	hetloss	METABRIC
MB-5107	TLR4	cna	hetloss	METABRIC
MB-6098	TLR4	cna	hetloss	METABRIC
MB-7038	TLR4	cna	hetloss	METABRIC
MB-0420	TLR4	cna	hetloss	METABRIC
PD10014	TLR4	cna	gain	BASIS
PD13296	TLR4	cna	hetloss	BASIS
PD13297	TLR4	cna	hetloss	BASIS
PD23562	TLR4	cna	amp	BASIS
PD23578	TLR4	cna	hetloss	BASIS
PD24186	TLR4	cna	amp	BASIS
PD24202	TLR4	cna	hetloss	BASIS
PD24206	TLR4	cna	gain	BASIS
PD24337	TLR4	cna	hetloss	BASIS
PD3890	TLR4	cna	hetloss	BASIS
PD3905	TLR4	cna	gain	BASIS
PD4005	TLR4	cna	hetloss	BASIS
PD4006	TLR4	cna	hetloss	BASIS
PD4107	TLR4	cna	hetloss	BASIS
PD5930	TLR4	cna	gain	BASIS
PD5945	TLR4	cna	gain	BASIS
PD6406	TLR4	cna	hetloss	BASIS
PD6731	TLR4	cna	hetloss	BASIS
PD7067	TLR4	cna	hetloss	BASIS
PD7215	TLR4	cna	gain	BASIS
PD8621	TLR4	cna	gain	BASIS
PD8980	TLR4	cna	gain	BASIS
PD9004	TLR4	cna	hetloss	BASIS
PD9585	TLR4	cna	hetloss	BASIS
TCGA-A2-A25B	TNFAIP3	cna	hetloss	TCGA
TCGA-AN-A0XU	TNFAIP3	cna	amp	TCGA
TCGA-AO-A0JL	TNFAIP3	cna	hetloss	TCGA
TCGA-BH-A0AW	TNFAIP3	cna	amp	TCGA
TCGA-BH-A0C0	TNFAIP3	cna	gain	TCGA
TCGA-BH-A18R	TNFAIP3	cna	amp	TCGA
TCGA-C8-A12L	TNFAIP3	cna	gain	TCGA
TCGA-D8-A27M	TNFAIP3	cna	hetloss	TCGA

MB-5070	TNFAIP3	cna	amp	METABRIC
MB-6098	TNFAIP3	cna	hetloss	METABRIC
MB-7032	TNFAIP3	cna	hetloss	METABRIC
MB-7038	TNFAIP3	cna	amp	METABRIC
MB-7048	TNFAIP3	cna	hetloss	METABRIC
MB-0420	TNFAIP3	cna	hetloss	METABRIC
PD11327	TNFAIP3	cna	gain	BASIS
PD13299	TNFAIP3	cna	gain	BASIS
PD13771	TNFAIP3	cna	hetloss	BASIS
PD22355	TNFAIP3	cna	gain	BASIS
PD23562	TNFAIP3	cna	gain	BASIS
PD23574	TNFAIP3	cna	gain	BASIS
PD24186	TNFAIP3	cna	gain	BASIS
PD24202	TNFAIP3	cna	gain	BASIS
PD24206	TNFAIP3	cna	gain	BASIS
PD3890	TNFAIP3	cna	gain	BASIS
PD3905	TNFAIP3	cna	gain	BASIS
PD4006	TNFAIP3	cna	hetloss	BASIS
PD4107	TNFAIP3	cna	amp	BASIS
PD4826	TNFAIP3	cna	gain	BASIS
PD5935	TNFAIP3	cna	gain	BASIS
PD5945	TNFAIP3	cna	amp	BASIS
PD6406	TNFAIP3	cna	hetloss	BASIS
PD6731	TNFAIP3	cna	hetloss	BASIS
PD7067	TNFAIP3	cna	amp	BASIS
PD7215	TNFAIP3	cna	gain	BASIS
PD8980	TNFAIP3	cna	gain	BASIS
PD9004	TNFAIP3	cna	homdel	BASIS
PD9585	TNFAIP3	cna	hetloss	BASIS
PD9702	TNFAIP3	cna	gain	BASIS
TCGA-A2-A25B	TOX	cna	gain	TCGA
TCGA-AO-A0JL	TOX	cna	amp	TCGA
TCGA-BH-A0AW	TOX	cna	gain	TCGA
TCGA-BH-A0C0	TOX	cna	gain	TCGA
TCGA-BH-A18R	TOX	cna	homdel	TCGA
TCGA-BH-A1FU	TOX	cna	gain	TCGA
TCGA-D8-A27M	TOX	cna	amp	TCGA
TCGA-E2-A1L7	TOX	cna	gain	TCGA
TCGA-E9-A1NC	TOX	cna	hetloss	TCGA
TCGA-LL-A5YP	TOX	cna	gain	TCGA
MB-0346	TOX	cna	gain	METABRIC
MB-5465	TOX	cna	hetloss	METABRIC
MB-6060	TOX	cna	amp	METABRIC
MB-6098	TOX	cna	gain	METABRIC
MB-7038	TOX	cna	gain	METABRIC
PD10014	TOX	cna	amp	BASIS
PD11327	TOX	cna	gain	BASIS

PD13296	TOX	cna	hetloss	BASIS
PD13299	TOX	cna	gain	BASIS
PD23562	TOX	cna	gain	BASIS
PD23574	TOX	cna	gain	BASIS
PD23578	TOX	cna	hetloss	BASIS
PD24186	TOX	cna	gain	BASIS
PD24202	TOX	cna	hetloss	BASIS
PD24206	TOX	cna	amp	BASIS
PD3905	TOX	cna	gain	BASIS
PD4005	TOX	cna	hetloss	BASIS
PD4006	TOX	cna	gain	BASIS
PD4826	TOX	cna	amp	BASIS
PD5935	TOX	cna	gain	BASIS
PD5945	TOX	cna	gain	BASIS
PD7067	TOX	cna	amp	BASIS
PD7215	TOX	cna	amp	BASIS
PD8621	TOX	cna	gain	BASIS
PD8980	TOX	cna	gain	BASIS
PD9004	TOX	cna	gain	BASIS
PD9585	TOX	cna	hetloss	BASIS
PD9702	TOX	cna	gain	BASIS
TCGA-AN-A0XU	TSC2	cna	hetloss	TCGA
TCGA-AO-A0JL	TSC2	cna	gain	TCGA
TCGA-BH-A0AW	TSC2	cna	gain	TCGA
TCGA-BH-A0C0	TSC2	cna	gain	TCGA
TCGA-C8-A12L	TSC2	cna	gain	TCGA
TCGA-E2-A1L7	TSC2	cna	hetloss	TCGA
TCGA-E9-A1NC	TSC2	cna	hetloss	TCGA
TCGA-LL-A5YP	TSC2	cna	gain	TCGA
MB-0346	TSC2	cna	hetloss	METABRIC
MB-5465	TSC2	cna	hetloss	METABRIC
MB-6271	TSC2	cna	gain	METABRIC
MB-7032	TSC2	cna	gain	METABRIC
PD11327	TSC2	cna	gain	BASIS
PD11742	TSC2	cna	gain	BASIS
PD13296	TSC2	cna	gain	BASIS
PD13297	TSC2	cna	hetloss	BASIS
PD13299	TSC2	cna	gain	BASIS
PD22355	TSC2	cna	hetloss	BASIS
PD23578	TSC2	cna	hetloss	BASIS
PD24202	TSC2	cna	hetloss	BASIS
PD24337	TSC2	cna	hetloss	BASIS
PD3905	TSC2	cna	gain	BASIS
PD4005	TSC2	cna	gain	BASIS
PD4006	TSC2	cna	gain	BASIS
PD4107	TSC2	cna	gain	BASIS
PD4826	TSC2	cna	gain	BASIS

PD4967	TSC2	cna	gain	BASIS
PD5930	TSC2	cna	gain	BASIS
PD5935	TSC2	cna	gain	BASIS
PD5945	TSC2	cna	gain	BASIS
PD5948	TSC2	cna	gain	BASIS
PD6413	TSC2	cna	gain	BASIS
PD7067	TSC2	cna	gain	BASIS
PD7215	TSC2	cna	amp	BASIS
PD8621	TSC2	cna	gain	BASIS
PD9004	TSC2	cna	gain	BASIS
PD9585	TSC2	cna	hetloss	BASIS
PD9702	TSC2	cna	gain	BASIS
TCGA-AN-A0XU	TUSC3	cna	hetloss	TCGA
TCGA-AO-A0JL	TUSC3	cna	hetloss	TCGA
TCGA-BH-A0C0	TUSC3	cna	hetloss	TCGA
TCGA-BH-A1FU	TUSC3	cna	homdel	TCGA
TCGA-C8-A12L	TUSC3	cna	gain	TCGA
TCGA-D8-A27M	TUSC3	cna	hetloss	TCGA
TCGA-E2-A1L7	TUSC3	cna	hetloss	TCGA
TCGA-E9-A1NC	TUSC3	cna	hetloss	TCGA
TCGA-LL-A5YP	TUSC3	cna	hetloss	TCGA
MB-0346	TUSC3	cna	gain	METABRIC
MB-2827	TUSC3	cna	hetloss	METABRIC
MB-5070	TUSC3	cna	hetloss	METABRIC
MB-5465	TUSC3	cna	hetloss	METABRIC
MB-6060	TUSC3	cna	hetloss	METABRIC
MB-6098	TUSC3	cna	hetloss	METABRIC
MB-7038	TUSC3	cna	gain	METABRIC
MB-0420	TUSC3	cna	hetloss	METABRIC
MTS-T0064	TUSC3	cna	hetloss	METABRIC
PD10014	TUSC3	cna	hetloss	BASIS
PD13296	TUSC3	cna	hetloss	BASIS
PD13299	TUSC3	cna	hetloss	BASIS
PD13771	TUSC3	cna	hetloss	BASIS
PD14442	TUSC3	cna	hetloss	BASIS
PD22355	TUSC3	cna	hetloss	BASIS
PD23562	TUSC3	cna	hetloss	BASIS
PD23578	TUSC3	cna	hetloss	BASIS
PD24186	TUSC3	cna	gain	BASIS
PD24202	TUSC3	cna	hetloss	BASIS
PD24206	TUSC3	cna	hetloss	BASIS
PD24337	TUSC3	cna	hetloss	BASIS
PD3905	TUSC3	cna	gain	BASIS
PD4005	TUSC3	cna	hetloss	BASIS
PD4006	TUSC3	cna	gain	BASIS
PD4107	TUSC3	cna	hetloss	BASIS
PD4826	TUSC3	cna	hetloss	BASIS

PD6406	TUSC3	cna	hetloss	BASIS
PD8980	TUSC3	cna	hetloss	BASIS
PD9004	TUSC3	cna	gain	BASIS
TCGA-A2-A25B	VTI1A	cna	hetloss	TCGA
TCGA-AN-A0XU	VTI1A	cna	hetloss	TCGA
TCGA-AO-A0JL	VTI1A	cna	hetloss	TCGA
TCGA-BH-A18R	VTI1A	cna	gain	TCGA
TCGA-C8-A12L	VTI1A	cna	hetloss	TCGA
TCGA-D8-A27M	VTI1A	cna	hetloss	TCGA
TCGA-E2-A1L7	VTI1A	cna	hetloss	TCGA
TCGA-E9-A1NC	VTI1A	cna	hetloss	TCGA
TCGA-LL-A5YP	VTI1A	cna	gain	TCGA
MB-0346	VTI1A	cna	hetloss	METABRIC
MB-2827	VTI1A	cna	hetloss	METABRIC
MB-5070	VTI1A	cna	hetloss	METABRIC
MB-5107	VTI1A	cna	hetloss	METABRIC
MB-6098	VTI1A	cna	hetloss	METABRIC
MB-7038	VTI1A	cna	hetloss	METABRIC
MB-0420	VTI1A	cna	hetloss	METABRIC
PD10014	VTI1A	cna	hetloss	BASIS
PD11742	VTI1A	cna	hetloss	BASIS
PD13296	VTI1A	cna	hetloss	BASIS
PD23562	VTI1A	cna	gain	BASIS
PD23574	VTI1A	cna	gain	BASIS
PD23578	VTI1A	cna	hetloss	BASIS
PD24206	VTI1A	cna	gain	BASIS
PD24337	VTI1A	cna	hetloss	BASIS
PD3905	VTI1A	cna	gain	BASIS
PD4005	VTI1A	cna	hetloss	BASIS
PD4006	VTI1A	cna	gain	BASIS
PD4107	VTI1A	cna	gain	BASIS
PD5935	VTI1A	cna	gain	BASIS
PD5945	VTI1A	cna	gain	BASIS
PD5948	VTI1A	cna	gain	BASIS
PD6731	VTI1A	cna	hetloss	BASIS
PD7067	VTI1A	cna	gain	BASIS
PD7215	VTI1A	cna	gain	BASIS
PD8621	VTI1A	cna	gain	BASIS
PD8980	VTI1A	cna	hetloss	BASIS
PD9585	VTI1A	cna	hetloss	BASIS
PD9702	VTI1A	cna	gain	BASIS
TCGA-A2-A25B	WHSC1L1	cna	gain	TCGA
TCGA-AN-A0XU	WHSC1L1	cna	hetloss	TCGA
TCGA-AO-A0JL	WHSC1L1	cna	gain	TCGA
TCGA-BH-A0C0	WHSC1L1	cna	hetloss	TCGA
TCGA-BH-A1FU	WHSC1L1	cna	homdel	TCGA
TCGA-D8-A27M	WHSC1L1	cna	hetloss	TCGA

TCGA-E2-A1L7	WHSC1L1	cna	hetloss	TCGA
TCGA-LL-A5YP	WHSC1L1	cna	hetloss	TCGA
MB-0346	WHSC1L1	cna	hetloss	METABRIC
MB-5465	WHSC1L1	cna	hetloss	METABRIC
MB-6060	WHSC1L1	cna	gain	METABRIC
MB-7038	WHSC1L1	cna	hetloss	METABRIC
MB-0420	WHSC1L1	cna	hetloss	METABRIC
PD10014	WHSC1L1	cna	hetloss	BASIS
PD11327	WHSC1L1	cna	homdel	BASIS
PD13296	WHSC1L1	cna	hetloss	BASIS
PD13299	WHSC1L1	cna	gain	BASIS
PD13771	WHSC1L1	cna	hetloss	BASIS
PD14442	WHSC1L1	cna	hetloss	BASIS
PD23562	WHSC1L1	cna	gain	BASIS
PD23578	WHSC1L1	cna	hetloss	BASIS
PD24186	WHSC1L1	cna	gain	BASIS
PD24202	WHSC1L1	cna	hetloss	BASIS
PD24206	WHSC1L1	cna	amp	BASIS
PD24337	WHSC1L1	cna	hetloss	BASIS
PD3905	WHSC1L1	cna	gain	BASIS
PD4006	WHSC1L1	cna	gain	BASIS
PD4107	WHSC1L1	cna	gain	BASIS
PD4826	WHSC1L1	cna	hetloss	BASIS
PD5930	WHSC1L1	cna	gain	BASIS
PD5935	WHSC1L1	cna	gain	BASIS
PD5945	WHSC1L1	cna	gain	BASIS
PD7067	WHSC1L1	cna	gain	BASIS
PD7215	WHSC1L1	cna	amp	BASIS
PD8980	WHSC1L1	cna	amp	BASIS
PD9004	WHSC1L1	cna	gain	BASIS
PD9585	WHSC1L1	cna	hetloss	BASIS
PD9702	WHSC1L1	cna	gain	BASIS
TCGA-A2-A25B	WRN	cna	hetloss	TCGA
TCGA-AN-A0XU	WRN	cna	hetloss	TCGA
TCGA-AO-A0JL	WRN	cna	hetloss	TCGA
TCGA-BH-A0C0	WRN	cna	hetloss	TCGA
TCGA-BH-A1FU	WRN	cna	homdel	TCGA
TCGA-C8-A12L	WRN	cna	hetloss	TCGA
TCGA-D8-A27M	WRN	cna	hetloss	TCGA
TCGA-E2-A1L7	WRN	cna	hetloss	TCGA
TCGA-LL-A5YP	WRN	cna	hetloss	TCGA
MB-0346	WRN	cna	hetloss	METABRIC
MB-2827	WRN	cna	hetloss	METABRIC
MB-5465	WRN	cna	hetloss	METABRIC
MB-6060	WRN	cna	hetloss	METABRIC
MB-7038	WRN	cna	hetloss	METABRIC
MB-0420	WRN	cna	hetloss	METABRIC

PD10014	WRN	cna	hetloss	BASIS
PD11327	WRN	cna	homdel	BASIS
PD13296	WRN	cna	hetloss	BASIS
PD13299	WRN	cna	gain	BASIS
PD13771	WRN	cna	hetloss	BASIS
PD14442	WRN	cna	hetloss	BASIS
PD22355	WRN	cna	hetloss	BASIS
PD23562	WRN	cna	hetloss	BASIS
PD23574	WRN	cna	hetloss	BASIS
PD23578	WRN	cna	hetloss	BASIS
PD24186	WRN	cna	gain	BASIS
PD24202	WRN	cna	hetloss	BASIS
PD24206	WRN	cna	gain	BASIS
PD24337	WRN	cna	hetloss	BASIS
PD3905	WRN	cna	gain	BASIS
PD4005	WRN	cna	hetloss	BASIS
PD4826	WRN	cna	hetloss	BASIS
PD5935	WRN	cna	gain	BASIS
PD5945	WRN	cna	gain	BASIS
PD6413	WRN	cna	gain	BASIS
PD7067	WRN	cna	hetloss	BASIS
PD9004	WRN	cna	gain	BASIS
PD9585	WRN	cna	hetloss	BASIS
TCGA-A2-A25B	XPO1	cna	hetloss	TCGA
TCGA-AN-A0XU	XPO1	cna	gain	TCGA
TCGA-AO-A0JL	XPO1	cna	gain	TCGA
TCGA-BH-A0AW	XPO1	cna	gain	TCGA
TCGA-BH-A0C0	XPO1	cna	gain	TCGA
TCGA-D8-A27M	XPO1	cna	gain	TCGA
TCGA-E9-A1NC	XPO1	cna	gain	TCGA
TCGA-LL-A5YP	XPO1	cna	amp	TCGA
MB-2827	XPO1	cna	hetloss	METABRIC
MB-5465	XPO1	cna	amp	METABRIC
MB-6271	XPO1	cna	hetloss	METABRIC
MB-7038	XPO1	cna	gain	METABRIC
MB-7048	XPO1	cna	hetloss	METABRIC
PD10014	XPO1	cna	gain	BASIS
PD11327	XPO1	cna	hetloss	BASIS
PD13296	XPO1	cna	gain	BASIS
PD13297	XPO1	cna	gain	BASIS
PD13299	XPO1	cna	gain	BASIS
PD22355	XPO1	cna	gain	BASIS
PD23562	XPO1	cna	gain	BASIS
PD23578	XPO1	cna	gain	BASIS
PD24186	XPO1	cna	gain	BASIS
PD24202	XPO1	cna	gain	BASIS
PD24206	XPO1	cna	hetloss	BASIS

PD3890	XPO1	cna	hetloss	BASIS
PD3905	XPO1	cna	gain	BASIS
PD4006	XPO1	cna	amp	BASIS
PD4107	XPO1	cna	gain	BASIS
PD4967	XPO1	cna	hetloss	BASIS
PD5930	XPO1	cna	gain	BASIS
PD5935	XPO1	cna	gain	BASIS
PD5945	XPO1	cna	amp	BASIS
PD5948	XPO1	cna	gain	BASIS
PD7067	XPO1	cna	gain	BASIS
PD7215	XPO1	cna	gain	BASIS
PD8621	XPO1	cna	gain	BASIS
PD9004	XPO1	cna	gain	BASIS
PD9702	XPO1	cna	gain	BASIS
TCGA-A2-A25B	YWHAE	cna	hetloss	TCGA
TCGA-AN-A0XU	YWHAE	cna	hetloss	TCGA
TCGA-AO-A0JL	YWHAE	cna	hetloss	TCGA
TCGA-BH-A0AW	YWHAE	cna	hetloss	TCGA
TCGA-BH-A0C0	YWHAE	cna	hetloss	TCGA
TCGA-C8-A12L	YWHAE	cna	hetloss	TCGA
TCGA-D8-A27M	YWHAE	cna	hetloss	TCGA
TCGA-E2-A1L7	YWHAE	cna	hetloss	TCGA
TCGA-E9-A1NC	YWHAE	cna	hetloss	TCGA
TCGA-EW-A1OX	YWHAE	cna	hetloss	TCGA
TCGA-LL-A5YP	YWHAE	cna	hetloss	TCGA
MB-0346	YWHAE	cna	hetloss	METABRIC
MB-2827	YWHAE	cna	hetloss	METABRIC
MB-5107	YWHAE	cna	hetloss	METABRIC
MB-6098	YWHAE	cna	gain	METABRIC
MB-6271	YWHAE	cna	hetloss	METABRIC
MB-7032	YWHAE	cna	gain	METABRIC
MB-7048	YWHAE	cna	hetloss	METABRIC
MB-0420	YWHAE	cna	hetloss	METABRIC
PD10014	YWHAE	cna	hetloss	BASIS
PD11742	YWHAE	cna	hetloss	BASIS
PD13296	YWHAE	cna	hetloss	BASIS
PD13771	YWHAE	cna	hetloss	BASIS
PD24202	YWHAE	cna	hetloss	BASIS
PD24206	YWHAE	cna	hetloss	BASIS
PD24337	YWHAE	cna	hetloss	BASIS
PD3890	YWHAE	cna	hetloss	BASIS
PD3905	YWHAE	cna	gain	BASIS
PD4005	YWHAE	cna	hetloss	BASIS
PD4826	YWHAE	cna	hetloss	BASIS
PD4967	YWHAE	cna	hetloss	BASIS
PD5945	YWHAE	cna	gain	BASIS
PD6406	YWHAE	cna	hetloss	BASIS

PD6413	YWHAE	cna	hetloss	BASIS
PD6731	YWHAE	cna	hetloss	BASIS
PD7067	YWHAE	cna	gain	BASIS
PD9585	YWHAE	cna	hetloss	BASIS
PD9702	YWHAE	cna	gain	BASIS
TCGA-AN-A0XU	ZMYM2	cna	hetloss	TCGA
TCGA-AO-A0JL	ZMYM2	cna	hetloss	TCGA
TCGA-BH-A0AW	ZMYM2	cna	hetloss	TCGA
TCGA-BH-A0C0	ZMYM2	cna	hetloss	TCGA
TCGA-BH-A1FU	ZMYM2	cna	hetloss	TCGA
TCGA-C8-A12L	ZMYM2	cna	hetloss	TCGA
TCGA-D8-A27M	ZMYM2	cna	hetloss	TCGA
TCGA-E2-A1L7	ZMYM2	cna	hetloss	TCGA
TCGA-E9-A1NC	ZMYM2	cna	hetloss	TCGA
TCGA-EW-A1OX	ZMYM2	cna	homdel	TCGA
TCGA-LL-A5YP	ZMYM2	cna	gain	TCGA
MB-0346	ZMYM2	cna	hetloss	METABRIC
MB-2827	ZMYM2	cna	hetloss	METABRIC
MB-6098	ZMYM2	cna	hetloss	METABRIC
MTS-T0064	ZMYM2	cna	hetloss	METABRIC
PD10014	ZMYM2	cna	hetloss	BASIS
PD11327	ZMYM2	cna	hetloss	BASIS
PD13296	ZMYM2	cna	hetloss	BASIS
PD13297	ZMYM2	cna	hetloss	BASIS
PD13771	ZMYM2	cna	hetloss	BASIS
PD14442	ZMYM2	cna	hetloss	BASIS
PD22355	ZMYM2	cna	hetloss	BASIS
PD24202	ZMYM2	cna	hetloss	BASIS
PD24206	ZMYM2	cna	gain	BASIS
PD24337	ZMYM2	cna	hetloss	BASIS
PD3890	ZMYM2	cna	hetloss	BASIS
PD3905	ZMYM2	cna	gain	BASIS
PD4005	ZMYM2	cna	hetloss	BASIS
PD4006	ZMYM2	cna	gain	BASIS
PD4107	ZMYM2	cna	gain	BASIS
PD4826	ZMYM2	cna	hetloss	BASIS
PD4967	ZMYM2	cna	hetloss	BASIS
PD5945	ZMYM2	cna	amp	BASIS
PD6413	ZMYM2	cna	hetloss	BASIS
PD7067	ZMYM2	cna	gain	BASIS
PD8980	ZMYM2	cna	hetloss	BASIS
PD9585	ZMYM2	cna	gain	BASIS
PD9702	ZMYM2	cna	gain	BASIS
TCGA-A2-A25B	ZNF703	cna	hetloss	TCGA
TCGA-AN-A0XU	ZNF703	cna	hetloss	TCGA
TCGA-AO-A0JL	ZNF703	cna	gain	TCGA
TCGA-BH-A0C0	ZNF703	cna	hetloss	TCGA

TCGA-BH-A1FU	ZNF703	cna	homdel	TCGA
TCGA-D8-A27M	ZNF703	cna	hetloss	TCGA
TCGA-E2-A1L7	ZNF703	cna	hetloss	TCGA
TCGA-LL-A5YP	ZNF703	cna	hetloss	TCGA
MB-0346	ZNF703	cna	gain	METABRIC
MB-5465	ZNF703	cna	hetloss	METABRIC
MB-6060	ZNF703	cna	gain	METABRIC
MB-7038	ZNF703	cna	hetloss	METABRIC
MB-0420	ZNF703	cna	hetloss	METABRIC
PD10014	ZNF703	cna	hetloss	BASIS
PD11327	ZNF703	cna	homdel	BASIS
PD13296	ZNF703	cna	hetloss	BASIS
PD13299	ZNF703	cna	gain	BASIS
PD13771	ZNF703	cna	hetloss	BASIS
PD14442	ZNF703	cna	hetloss	BASIS
PD22355	ZNF703	cna	hetloss	BASIS
PD23562	ZNF703	cna	gain	BASIS
PD23578	ZNF703	cna	hetloss	BASIS
PD24186	ZNF703	cna	gain	BASIS
PD24202	ZNF703	cna	hetloss	BASIS
PD24206	ZNF703	cna	amp	BASIS
PD24337	ZNF703	cna	hetloss	BASIS
PD3905	ZNF703	cna	gain	BASIS
PD4006	ZNF703	cna	gain	BASIS
PD4107	ZNF703	cna	gain	BASIS
PD4826	ZNF703	cna	hetloss	BASIS
PD5935	ZNF703	cna	gain	BASIS
PD5945	ZNF703	cna	gain	BASIS
PD7067	ZNF703	cna	gain	BASIS
PD7215	ZNF703	cna	amp	BASIS
PD8980	ZNF703	cna	amp	BASIS
PD9004	ZNF703	cna	gain	BASIS
PD9585	ZNF703	cna	hetloss	BASIS
PD9702	ZNF703	cna	gain	BASIS
TCGA-A2-A25B	SEPT9	cna	gain	TCGA
TCGA-AN-A0XU	SEPT9	cna	amp	TCGA
TCGA-AO-A0JL	SEPT9	cna	hetloss	TCGA
TCGA-BH-A0AW	SEPT9	cna	amp	TCGA
TCGA-BH-A0C0	SEPT9	cna	gain	TCGA
TCGA-BH-A18R	SEPT9	cna	amp	TCGA
TCGA-C8-A12L	SEPT9	cna	gain	TCGA
TCGA-D8-A27M	SEPT9	cna	gain	TCGA
TCGA-E2-A1L7	SEPT9	cna	gain	TCGA
TCGA-EW-A1OX	SEPT9	cna	amp	TCGA
TCGA-LL-A5YP	SEPT9	cna	amp	TCGA
PD10014	SEPT9	cna	gain	BASIS
PD11327	SEPT9	cna	amp	BASIS

PD22355	SEPT9	cna	hetloss	BASIS
PD23561	SEPT9	cna	hetloss	BASIS
PD23574	SEPT9	cna	gain	BASIS
PD24186	SEPT9	cna	amp	BASIS
PD24202	SEPT9	cna	gain	BASIS
PD24206	SEPT9	cna	hetloss	BASIS
PD24337	SEPT9	cna	gain	BASIS
PD3890	SEPT9	cna	gain	BASIS
PD3905	SEPT9	cna	gain	BASIS
PD4006	SEPT9	cna	gain	BASIS
PD4107	SEPT9	cna	gain	BASIS
PD4826	SEPT9	cna	amp	BASIS
PD4967	SEPT9	cna	hetloss	BASIS
PD5930	SEPT9	cna	gain	BASIS
PD5935	SEPT9	cna	gain	BASIS
PD5945	SEPT9	cna	amp	BASIS
PD5948	SEPT9	cna	gain	BASIS
PD6731	SEPT9	cna	hetloss	BASIS
PD7067	SEPT9	cna	gain	BASIS
PD7215	SEPT9	cna	gain	BASIS
PD8621	SEPT9	cna	gain	BASIS
PD9004	SEPT9	cna	gain	BASIS
PD9585	SEPT9	cna	gain	BASIS
PD9702	SEPT9	cna	gain	BASIS
TCGA-A2-A25B	ABCG4	cna	hetloss	TCGA
TCGA-AO-A0JL	ABCG4	cna	hetloss	TCGA
TCGA-BH-A0C0	ABCG4	cna	hetloss	TCGA
TCGA-BH-A18R	ABCG4	cna	homdel	TCGA
TCGA-D8-A27M	ABCG4	cna	hetloss	TCGA
TCGA-E2-A1L7	ABCG4	cna	hetloss	TCGA
TCGA-E9-A1NC	ABCG4	cna	hetloss	TCGA
TCGA-EW-A1OX	ABCG4	cna	homdel	TCGA
TCGA-LL-A5YP	ABCG4	cna	hetloss	TCGA
MB-0346	ABCG4	cna	hetloss	METABRIC
MB-5070	ABCG4	cna	hetloss	METABRIC
MB-5107	ABCG4	cna	hetloss	METABRIC
MB-6271	ABCG4	cna	hetloss	METABRIC
MB-7032	ABCG4	cna	hetloss	METABRIC
MB-7048	ABCG4	cna	hetloss	METABRIC
PD11742	ABCG4	cna	hetloss	BASIS
PD13296	ABCG4	cna	hetloss	BASIS
PD13297	ABCG4	cna	hetloss	BASIS
PD13771	ABCG4	cna	hetloss	BASIS
PD22355	ABCG4	cna	hetloss	BASIS
PD23574	ABCG4	cna	gain	BASIS
PD23578	ABCG4	cna	hetloss	BASIS
PD24186	ABCG4	cna	gain	BASIS

PD24206	ABCG4	cna	gain	BASIS
PD24337	ABCG4	cna	hetloss	BASIS
PD3905	ABCG4	cna	gain	BASIS
PD4005	ABCG4	cna	hetloss	BASIS
PD4826	ABCG4	cna	hetloss	BASIS
PD5935	ABCG4	cna	gain	BASIS
PD5945	ABCG4	cna	gain	BASIS
PD5948	ABCG4	cna	gain	BASIS
PD6413	ABCG4	cna	hetloss	BASIS
PD6731	ABCG4	cna	hetloss	BASIS
PD7067	ABCG4	cna	gain	BASIS
PD7215	ABCG4	cna	gain	BASIS
PD9004	ABCG4	cna	hetloss	BASIS
PD9702	ABCG4	cna	gain	BASIS
TCGA-AN-A0XU	ANKRD12	cna	hetloss	TCGA
TCGA-AO-A0JL	ANKRD12	cna	gain	TCGA
TCGA-BH-A0AW	ANKRD12	cna	amp	TCGA
TCGA-BH-A0C0	ANKRD12	cna	hetloss	TCGA
TCGA-BH-A1FU	ANKRD12	cna	gain	TCGA
TCGA-C8-A12L	ANKRD12	cna	gain	TCGA
TCGA-D8-A27M	ANKRD12	cna	hetloss	TCGA
TCGA-LL-A5YP	ANKRD12	cna	gain	TCGA
MB-0346	ANKRD12	cna	gain	METABRIC
MB-5107	ANKRD12	cna	hetloss	METABRIC
MB-5465	ANKRD12	cna	hetloss	METABRIC
MB-6098	ANKRD12	cna	hetloss	METABRIC
MB-0420	ANKRD12	cna	gain	METABRIC
PD11742	ANKRD12	cna	hetloss	BASIS
PD13296	ANKRD12	cna	hetloss	BASIS
PD13299	ANKRD12	cna	gain	BASIS
PD23574	ANKRD12	cna	gain	BASIS
PD24186	ANKRD12	cna	gain	BASIS
PD24206	ANKRD12	cna	gain	BASIS
PD24337	ANKRD12	cna	hetloss	BASIS
PD3890	ANKRD12	cna	hetloss	BASIS
PD3905	ANKRD12	cna	gain	BASIS
PD4005	ANKRD12	cna	hetloss	BASIS
PD4006	ANKRD12	cna	gain	BASIS
PD4107	ANKRD12	cna	gain	BASIS
PD4967	ANKRD12	cna	hetloss	BASIS
PD5930	ANKRD12	cna	gain	BASIS
PD5935	ANKRD12	cna	gain	BASIS
PD5945	ANKRD12	cna	amp	BASIS
PD5948	ANKRD12	cna	gain	BASIS
PD7067	ANKRD12	cna	gain	BASIS
PD7215	ANKRD12	cna	gain	BASIS
PD8621	ANKRD12	cna	gain	BASIS

PD8980	ANKRD12	cna	hetloss	BASIS
PD9004	ANKRD12	cna	gain	BASIS
PD9585	ANKRD12	cna	hetloss	BASIS
PD9702	ANKRD12	cna	gain	BASIS
TCGA-A2-A25B	AR	cna	gain	TCGA
TCGA-AN-A0XU	AR	cna	hetloss	TCGA
TCGA-AO-A0JL	AR	cna	gain	TCGA
TCGA-BH-A0AW	AR	cna	gain	TCGA
TCGA-C8-A12L	AR	cna	amp	TCGA
TCGA-E2-A1L7	AR	cna	hetloss	TCGA
TCGA-E9-A1NC	AR	cna	hetloss	TCGA
MB-0346	AR	cna	amp	METABRIC
MB-5465	AR	cna	gain	METABRIC
MB-6060	AR	cna	hetloss	METABRIC
MB-0420	AR	cna	hetloss	METABRIC
PD10014	AR	cna	hetloss	BASIS
PD11327	AR	cna	hetloss	BASIS
PD13296	AR	cna	gain	BASIS
PD13299	AR	cna	gain	BASIS
PD13771	AR	cna	gain	BASIS
PD14442	AR	cna	gain	BASIS
PD23562	AR	cna	hetloss	BASIS
PD23574	AR	cna	gain	BASIS
PD24206	AR	cna	gain	BASIS
PD3890	AR	cna	gain	BASIS
PD3905	AR	cna	gain	BASIS
PD4005	AR	cna	hetloss	BASIS
PD4107	AR	cna	gain	BASIS
PD4826	AR	cna	gain	BASIS
PD4967	AR	cna	gain	BASIS
PD5930	AR	cna	gain	BASIS
PD5935	AR	cna	gain	BASIS
PD5945	AR	cna	gain	BASIS
PD5948	AR	cna	gain	BASIS
PD6406	AR	cna	gain	BASIS
PD6413	AR	cna	gain	BASIS
PD7067	AR	cna	homdel	BASIS
PD7215	AR	cna	gain	BASIS
PD8621	AR	cna	gain	BASIS
PD8980	AR	cna	gain	BASIS
PD9004	AR	cna	gain	BASIS
TCGA-A2-A25B	ARID2	cna	gain	TCGA
TCGA-AN-A0XU	ARID2	cna	gain	TCGA
TCGA-AO-A0JL	ARID2	cna	hetloss	TCGA
TCGA-BH-A0C0	ARID2	cna	hetloss	TCGA
TCGA-BH-A18R	ARID2	cna	gain	TCGA
TCGA-BH-A1FU	ARID2	cna	hetloss	TCGA

TCGA-C8-A12L	ARID2	cna	gain	TCGA
TCGA-D8-A27M	ARID2	cna	hetloss	TCGA
TCGA-E2-A1L7	ARID2	cna	hetloss	TCGA
TCGA-E9-A1NC	ARID2	cna	hetloss	TCGA
TCGA-LL-A5YP	ARID2	cna	hetloss	TCGA
MB-0346	ARID2	cna	gain	METABRIC
MB-2827	ARID2	cna	hetloss	METABRIC
MB-5070	ARID2	cna	hetloss	METABRIC
MB-6098	ARID2	cna	hetloss	METABRIC
MB-0420	ARID2	cna	hetloss	METABRIC
PD11327	ARID2	cna	amp	BASIS
PD13296	ARID2	cna	hetloss	BASIS
PD13297	ARID2	cna	hetloss	BASIS
PD23574	ARID2	cna	gain	BASIS
PD23578	ARID2	cna	gain	BASIS
PD24186	ARID2	cna	gain	BASIS
PD24202	ARID2	cna	hetloss	BASIS
PD24206	ARID2	cna	gain	BASIS
PD24337	ARID2	cna	hetloss	BASIS
PD3890	ARID2	cna	hetloss	BASIS
PD3905	ARID2	cna	gain	BASIS
PD4005	ARID2	cna	hetloss	BASIS
PD4006	ARID2	cna	gain	BASIS
PD4107	ARID2	cna	hetloss	BASIS
PD4826	ARID2	cna	gain	BASIS
PD5945	ARID2	cna	gain	BASIS
PD5948	ARID2	cna	hetloss	BASIS
PD6731	ARID2	cna	hetloss	BASIS
PD7067	ARID2	cna	gain	BASIS
PD9585	ARID2	cna	hetloss	BASIS
PD9702	ARID2	cna	gain	BASIS
TCGA-A2-A25B	ASH2L	cna	hetloss	TCGA
TCGA-AN-A0XU	ASH2L	cna	hetloss	TCGA
TCGA-AO-A0JL	ASH2L	cna	gain	TCGA
TCGA-BH-A0C0	ASH2L	cna	hetloss	TCGA
TCGA-BH-A1FU	ASH2L	cna	homdel	TCGA
TCGA-D8-A27M	ASH2L	cna	hetloss	TCGA
TCGA-E2-A1L7	ASH2L	cna	hetloss	TCGA
TCGA-LL-A5YP	ASH2L	cna	hetloss	TCGA
MB-0346	ASH2L	cna	hetloss	METABRIC
MB-5465	ASH2L	cna	hetloss	METABRIC
MB-6060	ASH2L	cna	gain	METABRIC
MB-7038	ASH2L	cna	hetloss	METABRIC
MB-0420	ASH2L	cna	hetloss	METABRIC
PD10014	ASH2L	cna	hetloss	BASIS
PD11327	ASH2L	cna	homdel	BASIS
PD13296	ASH2L	cna	hetloss	BASIS

PD13299	ASH2L	cna	gain	BASIS
PD13771	ASH2L	cna	hetloss	BASIS
PD14442	ASH2L	cna	hetloss	BASIS
PD23562	ASH2L	cna	gain	BASIS
PD23578	ASH2L	cna	hetloss	BASIS
PD24186	ASH2L	cna	gain	BASIS
PD24202	ASH2L	cna	hetloss	BASIS
PD24206	ASH2L	cna	amp	BASIS
PD24337	ASH2L	cna	hetloss	BASIS
PD3905	ASH2L	cna	gain	BASIS
PD4006	ASH2L	cna	gain	BASIS
PD4107	ASH2L	cna	gain	BASIS
PD4826	ASH2L	cna	hetloss	BASIS
PD5935	ASH2L	cna	gain	BASIS
PD5945	ASH2L	cna	gain	BASIS
PD7067	ASH2L	cna	gain	BASIS
PD7215	ASH2L	cna	amp	BASIS
PD8980	ASH2L	cna	amp	BASIS
PD9004	ASH2L	cna	gain	BASIS
PD9585	ASH2L	cna	hetloss	BASIS
PD9702	ASH2L	cna	gain	BASIS
TCGA-A2-A25B	ATF1	cna	gain	TCGA
TCGA-AN-A0XU	ATF1	cna	gain	TCGA
TCGA-AO-A0JL	ATF1	cna	hetloss	TCGA
TCGA-BH-A18R	ATF1	cna	gain	TCGA
TCGA-BH-A1FU	ATF1	cna	hetloss	TCGA
TCGA-C8-A12L	ATF1	cna	gain	TCGA
TCGA-D8-A27M	ATF1	cna	hetloss	TCGA
TCGA-E2-A1L7	ATF1	cna	hetloss	TCGA
TCGA-E9-A1NC	ATF1	cna	hetloss	TCGA
TCGA-LL-A5YP	ATF1	cna	hetloss	TCGA
MB-0346	ATF1	cna	gain	METABRIC
MB-2827	ATF1	cna	hetloss	METABRIC
MB-5070	ATF1	cna	hetloss	METABRIC
MB-5465	ATF1	cna	gain	METABRIC
MB-6098	ATF1	cna	hetloss	METABRIC
MB-7038	ATF1	cna	hetloss	METABRIC
MB-7048	ATF1	cna	hetloss	METABRIC
MB-0420	ATF1	cna	homdel	METABRIC
PD11327	ATF1	cna	hetloss	BASIS
PD13296	ATF1	cna	hetloss	BASIS
PD13297	ATF1	cna	hetloss	BASIS
PD13299	ATF1	cna	gain	BASIS
PD23578	ATF1	cna	hetloss	BASIS
PD24202	ATF1	cna	hetloss	BASIS
PD24206	ATF1	cna	gain	BASIS
PD24337	ATF1	cna	hetloss	BASIS

PD3890	ATF1	cna	hetloss	BASIS
PD3905	ATF1	cna	gain	BASIS
PD4107	ATF1	cna	hetloss	BASIS
PD4826	ATF1	cna	gain	BASIS
PD5945	ATF1	cna	gain	BASIS
PD6413	ATF1	cna	hetloss	BASIS
PD6731	ATF1	cna	hetloss	BASIS
PD7067	ATF1	cna	gain	BASIS
PD8621	ATF1	cna	gain	BASIS
PD9585	ATF1	cna	hetloss	BASIS
PD9702	ATF1	cna	gain	BASIS
TCGA-A2-A25B	BCL2	cna	hetloss	TCGA
TCGA-AO-A0JL	BCL2	cna	gain	TCGA
TCGA-BH-A0AW	BCL2	cna	hetloss	TCGA
TCGA-BH-A0C0	BCL2	cna	gain	TCGA
TCGA-C8-A12L	BCL2	cna	gain	TCGA
TCGA-D8-A27M	BCL2	cna	hetloss	TCGA
TCGA-E2-A1L7	BCL2	cna	gain	TCGA
TCGA-LL-A5YP	BCL2	cna	hetloss	TCGA
MB-0346	BCL2	cna	hetloss	METABRIC
MB-0420	BCL2	cna	hetloss	METABRIC
MTS-T0064	BCL2	cna	hetloss	METABRIC
PD10014	BCL2	cna	hetloss	BASIS
PD11742	BCL2	cna	hetloss	BASIS
PD13296	BCL2	cna	hetloss	BASIS
PD13297	BCL2	cna	gain	BASIS
PD13299	BCL2	cna	gain	BASIS
PD22355	BCL2	cna	hetloss	BASIS
PD23574	BCL2	cna	hetloss	BASIS
PD23578	BCL2	cna	hetloss	BASIS
PD24186	BCL2	cna	gain	BASIS
PD24202	BCL2	cna	hetloss	BASIS
PD24206	BCL2	cna	hetloss	BASIS
PD24337	BCL2	cna	gain	BASIS
PD3905	BCL2	cna	gain	BASIS
PD4006	BCL2	cna	gain	BASIS
PD4107	BCL2	cna	gain	BASIS
PD4826	BCL2	cna	hetloss	BASIS
PD4967	BCL2	cna	hetloss	BASIS
PD5930	BCL2	cna	hetloss	BASIS
PD5945	BCL2	cna	gain	BASIS
PD5948	BCL2	cna	gain	BASIS
PD6406	BCL2	cna	gain	BASIS
PD7067	BCL2	cna	gain	BASIS
PD8980	BCL2	cna	hetloss	BASIS
PD9004	BCL2	cna	hetloss	BASIS
PD9585	BCL2	cna	hetloss	BASIS

PD9702	BCL2	cna	gain	BASIS
TCGA-A2-A25B	BCOR	cna	gain	TCGA
TCGA-AN-A0XU	BCOR	cna	hetloss	TCGA
TCGA-AO-A0JL	BCOR	cna	gain	TCGA
TCGA-BH-A0AW	BCOR	cna	gain	TCGA
TCGA-E2-A1L7	BCOR	cna	hetloss	TCGA
TCGA-E9-A1NC	BCOR	cna	hetloss	TCGA
MB-0346	BCOR	cna	hetloss	METABRIC
MB-2827	BCOR	cna	hetloss	METABRIC
MB-5070	BCOR	cna	hetloss	METABRIC
MB-5465	BCOR	cna	gain	METABRIC
MB-6098	BCOR	cna	hetloss	METABRIC
MB-0420	BCOR	cna	hetloss	METABRIC
MTS-T0064	BCOR	cna	hetloss	METABRIC
PD10014	BCOR	cna	hetloss	BASIS
PD11327	BCOR	cna	homdel	BASIS
PD13296	BCOR	cna	gain	BASIS
PD13299	BCOR	cna	amp	BASIS
PD13771	BCOR	cna	gain	BASIS
PD14442	BCOR	cna	gain	BASIS
PD23562	BCOR	cna	hetloss	BASIS
PD23574	BCOR	cna	gain	BASIS
PD23578	BCOR	cna	hetloss	BASIS
PD3905	BCOR	cna	gain	BASIS
PD4005	BCOR	cna	hetloss	BASIS
PD4006	BCOR	cna	hetloss	BASIS
PD4107	BCOR	cna	gain	BASIS
PD4826	BCOR	cna	gain	BASIS
PD4967	BCOR	cna	gain	BASIS
PD5935	BCOR	cna	gain	BASIS
PD5948	BCOR	cna	gain	BASIS
PD6406	BCOR	cna	gain	BASIS
PD6413	BCOR	cna	gain	BASIS
PD7215	BCOR	cna	gain	BASIS
PD8621	BCOR	cna	amp	BASIS
PD8980	BCOR	cna	hetloss	BASIS
PD9004	BCOR	cna	gain	BASIS
PD9585	BCOR	cna	hetloss	BASIS
TCGA-A2-A25B	BMPR1A	cna	hetloss	TCGA
TCGA-AN-A0XU	BMPR1A	cna	hetloss	TCGA
TCGA-AO-A0JL	BMPR1A	cna	gain	TCGA
TCGA-BH-A0AW	BMPR1A	cna	gain	TCGA
TCGA-BH-A18R	BMPR1A	cna	gain	TCGA
TCGA-C8-A12L	BMPR1A	cna	hetloss	TCGA
TCGA-D8-A27M	BMPR1A	cna	hetloss	TCGA
TCGA-E2-A1L7	BMPR1A	cna	hetloss	TCGA
MB-2827	BMPR1A	cna	hetloss	METABRIC

MB-5070	BMPR1A	cna	hetloss	METABRIC
MB-6060	BMPR1A	cna	hetloss	METABRIC
MB-6098	BMPR1A	cna	hetloss	METABRIC
MB-7048	BMPR1A	cna	hetloss	METABRIC
MTS-T0064	BMPR1A	cna	gain	METABRIC
PD10014	BMPR1A	cna	hetloss	BASIS
PD11327	BMPR1A	cna	hetloss	BASIS
PD11742	BMPR1A	cna	hetloss	BASIS
PD13296	BMPR1A	cna	hetloss	BASIS
PD13299	BMPR1A	cna	gain	BASIS
PD23562	BMPR1A	cna	gain	BASIS
PD23574	BMPR1A	cna	gain	BASIS
PD23578	BMPR1A	cna	hetloss	BASIS
PD24202	BMPR1A	cna	hetloss	BASIS
PD24206	BMPR1A	cna	gain	BASIS
PD3890	BMPR1A	cna	hetloss	BASIS
PD3905	BMPR1A	cna	gain	BASIS
PD4006	BMPR1A	cna	gain	BASIS
PD4107	BMPR1A	cna	gain	BASIS
PD5930	BMPR1A	cna	gain	BASIS
PD5935	BMPR1A	cna	homdel	BASIS
PD6406	BMPR1A	cna	hetloss	BASIS
PD6731	BMPR1A	cna	hetloss	BASIS
PD7215	BMPR1A	cna	gain	BASIS
PD8980	BMPR1A	cna	hetloss	BASIS
PD9004	BMPR1A	cna	gain	BASIS
PD9585	BMPR1A	cna	hetloss	BASIS
PD9702	BMPR1A	cna	gain	BASIS
TCGA-A2-A25B	CBL	cna	hetloss	TCGA
TCGA-AO-A0JL	CBL	cna	hetloss	TCGA
TCGA-BH-A0C0	CBL	cna	hetloss	TCGA
TCGA-BH-A18R	CBL	cna	homdel	TCGA
TCGA-D8-A27M	CBL	cna	hetloss	TCGA
TCGA-E2-A1L7	CBL	cna	hetloss	TCGA
TCGA-E9-A1NC	CBL	cna	hetloss	TCGA
TCGA-EW-A1OX	CBL	cna	homdel	TCGA
TCGA-LL-A5YP	CBL	cna	hetloss	TCGA
MB-0346	CBL	cna	hetloss	METABRIC
MB-5070	CBL	cna	hetloss	METABRIC
MB-5107	CBL	cna	hetloss	METABRIC
MB-6271	CBL	cna	hetloss	METABRIC
MB-7032	CBL	cna	hetloss	METABRIC
MB-7048	CBL	cna	hetloss	METABRIC
PD11742	CBL	cna	hetloss	BASIS
PD13296	CBL	cna	hetloss	BASIS
PD13297	CBL	cna	hetloss	BASIS
PD13771	CBL	cna	hetloss	BASIS

PD22355	CBL	cna	hetloss	BASIS
PD23574	CBL	cna	gain	BASIS
PD23578	CBL	cna	hetloss	BASIS
PD24186	CBL	cna	gain	BASIS
PD24206	CBL	cna	gain	BASIS
PD24337	CBL	cna	hetloss	BASIS
PD3905	CBL	cna	gain	BASIS
PD4005	CBL	cna	hetloss	BASIS
PD4826	CBL	cna	hetloss	BASIS
PD5935	CBL	cna	gain	BASIS
PD5945	CBL	cna	gain	BASIS
PD5948	CBL	cna	gain	BASIS
PD6413	CBL	cna	hetloss	BASIS
PD6731	CBL	cna	hetloss	BASIS
PD7067	CBL	cna	gain	BASIS
PD7215	CBL	cna	gain	BASIS
PD9004	CBL	cna	hetloss	BASIS
PD9702	CBL	cna	gain	BASIS
TCGA-AN-A0XU	CD74	cna	hetloss	TCGA
TCGA-AO-A0JL	CD74	cna	hetloss	TCGA
TCGA-BH-A0C0	CD74	cna	hetloss	TCGA
TCGA-BH-A18R	CD74	cna	gain	TCGA
TCGA-BH-A1FU	CD74	cna	hetloss	TCGA
TCGA-C8-A12L	CD74	cna	hetloss	TCGA
TCGA-D8-A27M	CD74	cna	hetloss	TCGA
TCGA-E2-A1L7	CD74	cna	hetloss	TCGA
TCGA-E9-A1NC	CD74	cna	hetloss	TCGA
TCGA-LL-A5YP	CD74	cna	hetloss	TCGA
MB-0346	CD74	cna	gain	METABRIC
MB-5070	CD74	cna	hetloss	METABRIC
MB-5465	CD74	cna	hetloss	METABRIC
MB-6098	CD74	cna	hetloss	METABRIC
MB-7038	CD74	cna	hetloss	METABRIC
MB-0420	CD74	cna	hetloss	METABRIC
PD10014	CD74	cna	hetloss	BASIS
PD13296	CD74	cna	hetloss	BASIS
PD13297	CD74	cna	hetloss	BASIS
PD13771	CD74	cna	gain	BASIS
PD22355	CD74	cna	hetloss	BASIS
PD23578	CD74	cna	hetloss	BASIS
PD24186	CD74	cna	gain	BASIS
PD24202	CD74	cna	hetloss	BASIS
PD24206	CD74	cna	gain	BASIS
PD24337	CD74	cna	hetloss	BASIS
PD3890	CD74	cna	hetloss	BASIS
PD3905	CD74	cna	gain	BASIS
PD4005	CD74	cna	hetloss	BASIS

PD4826	CD74	cna	gain	BASIS
PD5935	CD74	cna	gain	BASIS
PD5945	CD74	cna	gain	BASIS
PD6406	CD74	cna	hetloss	BASIS
PD6413	CD74	cna	hetloss	BASIS
PD7067	CD74	cna	gain	BASIS
PD8980	CD74	cna	hetloss	BASIS
PD9585	CD74	cna	hetloss	BASIS
TCGA-A2-A25B	CHCHD7	cna	gain	TCGA
TCGA-AO-A0JL	CHCHD7	cna	amp	TCGA
TCGA-BH-A0AW	CHCHD7	cna	gain	TCGA
TCGA-BH-A0C0	CHCHD7	cna	gain	TCGA
TCGA-BH-A18R	CHCHD7	cna	homdel	TCGA
TCGA-BH-A1FU	CHCHD7	cna	gain	TCGA
TCGA-D8-A27M	CHCHD7	cna	amp	TCGA
TCGA-E9-A1NC	CHCHD7	cna	amp	TCGA
TCGA-LL-A5YP	CHCHD7	cna	gain	TCGA
MB-0346	CHCHD7	cna	gain	METABRIC
MB-5465	CHCHD7	cna	hetloss	METABRIC
MB-6060	CHCHD7	cna	amp	METABRIC
MB-6098	CHCHD7	cna	gain	METABRIC
MB-7038	CHCHD7	cna	gain	METABRIC
PD10014	CHCHD7	cna	amp	BASIS
PD11327	CHCHD7	cna	hetloss	BASIS
PD13296	CHCHD7	cna	homdel	BASIS
PD13299	CHCHD7	cna	gain	BASIS
PD23562	CHCHD7	cna	gain	BASIS
PD23574	CHCHD7	cna	gain	BASIS
PD23578	CHCHD7	cna	hetloss	BASIS
PD24186	CHCHD7	cna	gain	BASIS
PD24202	CHCHD7	cna	hetloss	BASIS
PD3905	CHCHD7	cna	gain	BASIS
PD4005	CHCHD7	cna	hetloss	BASIS
PD4006	CHCHD7	cna	gain	BASIS
PD4107	CHCHD7	cna	gain	BASIS
PD4826	CHCHD7	cna	amp	BASIS
PD5935	CHCHD7	cna	gain	BASIS
PD5945	CHCHD7	cna	gain	BASIS
PD7067	CHCHD7	cna	amp	BASIS
PD7215	CHCHD7	cna	amp	BASIS
PD8621	CHCHD7	cna	gain	BASIS
PD8980	CHCHD7	cna	gain	BASIS
PD9004	CHCHD7	cna	gain	BASIS
PD9585	CHCHD7	cna	hetloss	BASIS
PD9702	CHCHD7	cna	gain	BASIS
TCGA-A2-A25B	CHD2	cna	amp	TCGA
TCGA-AO-A0JL	CHD2	cna	hetloss	TCGA

TCGA-BH-A0AW	CHD2	cna	hetloss	TCGA
TCGA-BH-A0C0	CHD2	cna	gain	TCGA
TCGA-D8-A27M	CHD2	cna	hetloss	TCGA
TCGA-E2-A1L7	CHD2	cna	gain	TCGA
TCGA-LL-A5YP	CHD2	cna	gain	TCGA
MB-0346	CHD2	cna	hetloss	METABRIC
MB-2827	CHD2	cna	amp	METABRIC
MB-5465	CHD2	cna	hetloss	METABRIC
MB-6060	CHD2	cna	gain	METABRIC
MB-7038	CHD2	cna	gain	METABRIC
MB-7048	CHD2	cna	amp	METABRIC
MB-0420	CHD2	cna	gain	METABRIC
MTS-T0064	CHD2	cna	amp	METABRIC
PD10014	CHD2	cna	gain	BASIS
PD11327	CHD2	cna	gain	BASIS
PD11742	CHD2	cna	hetloss	BASIS
PD13296	CHD2	cna	gain	BASIS
PD13299	CHD2	cna	gain	BASIS
PD23562	CHD2	cna	gain	BASIS
PD23574	CHD2	cna	gain	BASIS
PD23578	CHD2	cna	gain	BASIS
PD24202	CHD2	cna	hetloss	BASIS
PD24206	CHD2	cna	gain	BASIS
PD3890	CHD2	cna	hetloss	BASIS
PD3905	CHD2	cna	gain	BASIS
PD4006	CHD2	cna	gain	BASIS
PD4107	CHD2	cna	gain	BASIS
PD5930	CHD2	cna	gain	BASIS
PD5945	CHD2	cna	gain	BASIS
PD6413	CHD2	cna	hetloss	BASIS
PD7067	CHD2	cna	gain	BASIS
PD8621	CHD2	cna	gain	BASIS
PD8980	CHD2	cna	gain	BASIS
PD9585	CHD2	cna	hetloss	BASIS
PD9702	CHD2	cna	gain	BASIS
TCGA-A2-A25B	CHEK1	cna	hetloss	TCGA
TCGA-AO-A0JL	CHEK1	cna	hetloss	TCGA
TCGA-BH-A0C0	CHEK1	cna	hetloss	TCGA
TCGA-BH-A18R	CHEK1	cna	homdel	TCGA
TCGA-D8-A27M	CHEK1	cna	hetloss	TCGA
TCGA-E2-A1L7	CHEK1	cna	hetloss	TCGA
TCGA-E9-A1NC	CHEK1	cna	hetloss	TCGA
TCGA-EW-A1OX	CHEK1	cna	homdel	TCGA
TCGA-LL-A5YP	CHEK1	cna	gain	TCGA
MB-0346	CHEK1	cna	hetloss	METABRIC
MB-5107	CHEK1	cna	hetloss	METABRIC
MB-6271	CHEK1	cna	hetloss	METABRIC

PD11327	CHEK1	cna	gain	BASIS
PD11742	CHEK1	cna	hetloss	BASIS
PD13296	CHEK1	cna	hetloss	BASIS
PD13297	CHEK1	cna	hetloss	BASIS
PD13771	CHEK1	cna	hetloss	BASIS
PD22355	CHEK1	cna	hetloss	BASIS
PD23574	CHEK1	cna	gain	BASIS
PD23578	CHEK1	cna	hetloss	BASIS
PD24186	CHEK1	cna	gain	BASIS
PD24202	CHEK1	cna	gain	BASIS
PD24206	CHEK1	cna	gain	BASIS
PD24337	CHEK1	cna	hetloss	BASIS
PD3890	CHEK1	cna	gain	BASIS
PD3905	CHEK1	cna	gain	BASIS
PD4005	CHEK1	cna	hetloss	BASIS
PD4826	CHEK1	cna	hetloss	BASIS
PD5935	CHEK1	cna	gain	BASIS
PD5945	CHEK1	cna	gain	BASIS
PD5948	CHEK1	cna	gain	BASIS
PD6413	CHEK1	cna	hetloss	BASIS
PD6731	CHEK1	cna	hetloss	BASIS
PD7067	CHEK1	cna	gain	BASIS
PD7215	CHEK1	cna	gain	BASIS
PD9004	CHEK1	cna	hetloss	BASIS
PD9702	CHEK1	cna	gain	BASIS
TCGA-AN-A0XU	CIITA	cna	gain	TCGA
TCGA-AO-A0JL	CIITA	cna	amp	TCGA
TCGA-BH-A0AW	CIITA	cna	gain	TCGA
TCGA-BH-A0C0	CIITA	cna	gain	TCGA
TCGA-C8-A12L	CIITA	cna	gain	TCGA
TCGA-E2-A1L7	CIITA	cna	hetloss	TCGA
TCGA-LL-A5YP	CIITA	cna	gain	TCGA
MB-0346	CIITA	cna	gain	METABRIC
MB-6060	CIITA	cna	gain	METABRIC
MB-6098	CIITA	cna	hetloss	METABRIC
MB-6271	CIITA	cna	gain	METABRIC
MB-7032	CIITA	cna	gain	METABRIC
MB-7038	CIITA	cna	hetloss	METABRIC
MB-0420	CIITA	cna	hetloss	METABRIC
PD10014	CIITA	cna	hetloss	BASIS
PD11327	CIITA	cna	hetloss	BASIS
PD11742	CIITA	cna	gain	BASIS
PD13296	CIITA	cna	gain	BASIS
PD13297	CIITA	cna	hetloss	BASIS
PD13299	CIITA	cna	gain	BASIS
PD22355	CIITA	cna	hetloss	BASIS
PD23578	CIITA	cna	hetloss	BASIS

PD24186	CIITA	cna	gain	BASIS
PD24206	CIITA	cna	gain	BASIS
PD24337	CIITA	cna	hetloss	BASIS
PD3905	CIITA	cna	gain	BASIS
PD4005	CIITA	cna	gain	BASIS
PD4107	CIITA	cna	gain	BASIS
PD4826	CIITA	cna	gain	BASIS
PD5935	CIITA	cna	gain	BASIS
PD5945	CIITA	cna	gain	BASIS
PD5948	CIITA	cna	gain	BASIS
PD7067	CIITA	cna	gain	BASIS
PD7215	CIITA	cna	amp	BASIS
PD9004	CIITA	cna	gain	BASIS
PD9585	CIITA	cna	hetloss	BASIS
PD9702	CIITA	cna	gain	BASIS
TCGA-AN-A0XU	CSF1R	cna	hetloss	TCGA
TCGA-AO-A0JL	CSF1R	cna	hetloss	TCGA
TCGA-BH-A0C0	CSF1R	cna	hetloss	TCGA
TCGA-BH-A18R	CSF1R	cna	gain	TCGA
TCGA-BH-A1FU	CSF1R	cna	hetloss	TCGA
TCGA-C8-A12L	CSF1R	cna	hetloss	TCGA
TCGA-D8-A27M	CSF1R	cna	hetloss	TCGA
TCGA-E2-A1L7	CSF1R	cna	hetloss	TCGA
TCGA-E9-A1NC	CSF1R	cna	hetloss	TCGA
TCGA-LL-A5YP	CSF1R	cna	hetloss	TCGA
MB-0346	CSF1R	cna	gain	METABRIC
MB-5070	CSF1R	cna	hetloss	METABRIC
MB-5465	CSF1R	cna	hetloss	METABRIC
MB-6098	CSF1R	cna	hetloss	METABRIC
MB-7038	CSF1R	cna	hetloss	METABRIC
MB-0420	CSF1R	cna	hetloss	METABRIC
PD10014	CSF1R	cna	hetloss	BASIS
PD13296	CSF1R	cna	hetloss	BASIS
PD13297	CSF1R	cna	hetloss	BASIS
PD13771	CSF1R	cna	gain	BASIS
PD22355	CSF1R	cna	hetloss	BASIS
PD23578	CSF1R	cna	hetloss	BASIS
PD24186	CSF1R	cna	gain	BASIS
PD24202	CSF1R	cna	hetloss	BASIS
PD24206	CSF1R	cna	gain	BASIS
PD24337	CSF1R	cna	hetloss	BASIS
PD3890	CSF1R	cna	hetloss	BASIS
PD3905	CSF1R	cna	gain	BASIS
PD4005	CSF1R	cna	hetloss	BASIS
PD4826	CSF1R	cna	gain	BASIS
PD5935	CSF1R	cna	gain	BASIS
PD5945	CSF1R	cna	gain	BASIS

PD6406	CSF1R	cna	hetloss	BASIS
PD6413	CSF1R	cna	hetloss	BASIS
PD7067	CSF1R	cna	gain	BASIS
PD8980	CSF1R	cna	hetloss	BASIS
PD9585	CSF1R	cna	hetloss	BASIS
TCGA-A2-A25B	CUX1	cna	gain	TCGA
TCGA-AN-A0XU	CUX1	cna	hetloss	TCGA
TCGA-AO-A0JL	CUX1	cna	gain	TCGA
TCGA-BH-A0AW	CUX1	cna	gain	TCGA
TCGA-C8-A12L	CUX1	cna	hetloss	TCGA
TCGA-D8-A27M	CUX1	cna	gain	TCGA
TCGA-E2-A1L7	CUX1	cna	hetloss	TCGA
TCGA-E9-A1NC	CUX1	cna	gain	TCGA
TCGA-LL-A5YP	CUX1	cna	gain	TCGA
MB-0346	CUX1	cna	hetloss	METABRIC
MB-2827	CUX1	cna	hetloss	METABRIC
MB-5465	CUX1	cna	hetloss	METABRIC
MB-6098	CUX1	cna	hetloss	METABRIC
MB-7038	CUX1	cna	gain	METABRIC
PD11327	CUX1	cna	gain	BASIS
PD13296	CUX1	cna	hetloss	BASIS
PD22355	CUX1	cna	hetloss	BASIS
PD23574	CUX1	cna	gain	BASIS
PD23578	CUX1	cna	hetloss	BASIS
PD24186	CUX1	cna	amp	BASIS
PD24206	CUX1	cna	hetloss	BASIS
PD3890	CUX1	cna	hetloss	BASIS
PD3905	CUX1	cna	gain	BASIS
PD4006	CUX1	cna	amp	BASIS
PD4107	CUX1	cna	gain	BASIS
PD4826	CUX1	cna	gain	BASIS
PD5930	CUX1	cna	gain	BASIS
PD5935	CUX1	cna	gain	BASIS
PD5945	CUX1	cna	amp	BASIS
PD5948	CUX1	cna	gain	BASIS
PD6406	CUX1	cna	gain	BASIS
PD7067	CUX1	cna	gain	BASIS
PD7215	CUX1	cna	gain	BASIS
PD8621	CUX1	cna	gain	BASIS
PD9004	CUX1	cna	amp	BASIS
PD9585	CUX1	cna	hetloss	BASIS
PD9702	CUX1	cna	gain	BASIS
TCGA-A2-A25B	DCHS1	cna	hetloss	TCGA
TCGA-AN-A0XU	DCHS1	cna	hetloss	TCGA
TCGA-AO-A0JL	DCHS1	cna	hetloss	TCGA
TCGA-BH-A0C0	DCHS1	cna	hetloss	TCGA
TCGA-C8-A12L	DCHS1	cna	hetloss	TCGA

TCGA-E2-A1L7	DCHS1	cna	hetloss	TCGA
TCGA-E9-A1NC	DCHS1	cna	hetloss	TCGA
TCGA-LL-A5YP	DCHS1	cna	hetloss	TCGA
MB-0346	DCHS1	cna	hetloss	METABRIC
MB-5070	DCHS1	cna	hetloss	METABRIC
MB-6060	DCHS1	cna	hetloss	METABRIC
MB-6271	DCHS1	cna	gain	METABRIC
MB-7048	DCHS1	cna	hetloss	METABRIC
MB-0420	DCHS1	cna	hetloss	METABRIC
PD10014	DCHS1	cna	hetloss	BASIS
PD11742	DCHS1	cna	hetloss	BASIS
PD13296	DCHS1	cna	hetloss	BASIS
PD13297	DCHS1	cna	hetloss	BASIS
PD22355	DCHS1	cna	hetloss	BASIS
PD23574	DCHS1	cna	gain	BASIS
PD24202	DCHS1	cna	hetloss	BASIS
PD24206	DCHS1	cna	gain	BASIS
PD24337	DCHS1	cna	gain	BASIS
PD3890	DCHS1	cna	hetloss	BASIS
PD3905	DCHS1	cna	gain	BASIS
PD4005	DCHS1	cna	hetloss	BASIS
PD4107	DCHS1	cna	gain	BASIS
PD4826	DCHS1	cna	gain	BASIS
PD5935	DCHS1	cna	gain	BASIS
PD5945	DCHS1	cna	gain	BASIS
PD6406	DCHS1	cna	hetloss	BASIS
PD6413	DCHS1	cna	hetloss	BASIS
PD6731	DCHS1	cna	hetloss	BASIS
PD7215	DCHS1	cna	hetloss	BASIS
PD8621	DCHS1	cna	hetloss	BASIS
PD8980	DCHS1	cna	hetloss	BASIS
PD9585	DCHS1	cna	hetloss	BASIS
TCGA-A2-A25B	DMD	cna	gain	TCGA
TCGA-AN-A0XU	DMD	cna	hetloss	TCGA
TCGA-AO-A0JL	DMD	cna	hetloss	TCGA
TCGA-BH-A0AW	DMD	cna	gain	TCGA
TCGA-E2-A1L7	DMD	cna	hetloss	TCGA
TCGA-E9-A1NC	DMD	cna	hetloss	TCGA
TCGA-LL-A5YP	DMD	cna	hetloss	TCGA
MB-0346	DMD	cna	hetloss	METABRIC
MB-2827	DMD	cna	hetloss	METABRIC
MB-5465	DMD	cna	gain	METABRIC
MB-6098	DMD	cna	hetloss	METABRIC
MB-7038	DMD	cna	gain	METABRIC
MB-0420	DMD	cna	hetloss	METABRIC
PD10014	DMD	cna	hetloss	BASIS
PD13296	DMD	cna	amp	BASIS

PD13297	DMD	cna	gain	BASIS
PD13299	DMD	cna	gain	BASIS
PD13771	DMD	cna	gain	BASIS
PD14442	DMD	cna	gain	BASIS
PD23562	DMD	cna	hetloss	BASIS
PD23574	DMD	cna	gain	BASIS
PD23578	DMD	cna	hetloss	BASIS
PD3905	DMD	cna	amp	BASIS
PD4005	DMD	cna	hetloss	BASIS
PD4006	DMD	cna	homdel	BASIS
PD4107	DMD	cna	hetloss	BASIS
PD4826	DMD	cna	gain	BASIS
PD4967	DMD	cna	gain	BASIS
PD5935	DMD	cna	gain	BASIS
PD5948	DMD	cna	gain	BASIS
PD6406	DMD	cna	gain	BASIS
PD6413	DMD	cna	gain	BASIS
PD7215	DMD	cna	gain	BASIS
PD8621	DMD	cna	amp	BASIS
PD8980	DMD	cna	hetloss	BASIS
PD9004	DMD	cna	gain	BASIS
PD9702	DMD	cna	hetloss	BASIS
TCGA-A2-A25B	DTX1	cna	gain	TCGA
TCGA-AN-A0XU	DTX1	cna	hetloss	TCGA
TCGA-AO-A0JL	DTX1	cna	hetloss	TCGA
TCGA-BH-A0C0	DTX1	cna	gain	TCGA
TCGA-BH-A18R	DTX1	cna	gain	TCGA
TCGA-BH-A1FU	DTX1	cna	hetloss	TCGA
TCGA-D8-A27M	DTX1	cna	hetloss	TCGA
TCGA-E2-A1L7	DTX1	cna	gain	TCGA
TCGA-LL-A5YP	DTX1	cna	hetloss	TCGA
MB-0346	DTX1	cna	gain	METABRIC
MB-2827	DTX1	cna	hetloss	METABRIC
MB-5070	DTX1	cna	hetloss	METABRIC
MB-6098	DTX1	cna	hetloss	METABRIC
MB-7048	DTX1	cna	hetloss	METABRIC
PD10014	DTX1	cna	gain	BASIS
PD11327	DTX1	cna	hetloss	BASIS
PD11742	DTX1	cna	hetloss	BASIS
PD13296	DTX1	cna	hetloss	BASIS
PD13297	DTX1	cna	gain	BASIS
PD22355	DTX1	cna	hetloss	BASIS
PD23574	DTX1	cna	gain	BASIS
PD23578	DTX1	cna	hetloss	BASIS
PD3890	DTX1	cna	hetloss	BASIS
PD3905	DTX1	cna	gain	BASIS
PD4005	DTX1	cna	hetloss	BASIS

PD4006	DTX1	cna	gain	BASIS
PD4107	DTX1	cna	gain	BASIS
PD4826	DTX1	cna	gain	BASIS
PD5945	DTX1	cna	gain	BASIS
PD5948	DTX1	cna	gain	BASIS
PD6406	DTX1	cna	hetloss	BASIS
PD6413	DTX1	cna	hetloss	BASIS
PD6731	DTX1	cna	hetloss	BASIS
PD7067	DTX1	cna	gain	BASIS
PD9004	DTX1	cna	hetloss	BASIS
PD9585	DTX1	cna	hetloss	BASIS
PD9702	DTX1	cna	gain	BASIS
TCGA-A2-A25B	E2F3	cna	gain	TCGA
TCGA-AN-A0XU	E2F3	cna	amp	TCGA
TCGA-AO-A0JL	E2F3	cna	gain	TCGA
TCGA-BH-A0AW	E2F3	cna	gain	TCGA
TCGA-BH-A0C0	E2F3	cna	hetloss	TCGA
TCGA-BH-A18R	E2F3	cna	homdel	TCGA
TCGA-C8-A12L	E2F3	cna	hetloss	TCGA
TCGA-E2-A1L7	E2F3	cna	gain	TCGA
MB-5070	E2F3	cna	amp	METABRIC
MB-5465	E2F3	cna	gain	METABRIC
MB-6060	E2F3	cna	gain	METABRIC
MB-7032	E2F3	cna	gain	METABRIC
PD11327	E2F3	cna	amp	BASIS
PD13296	E2F3	cna	gain	BASIS
PD13297	E2F3	cna	gain	BASIS
PD13299	E2F3	cna	amp	BASIS
PD22355	E2F3	cna	gain	BASIS
PD23562	E2F3	cna	amp	BASIS
PD23574	E2F3	cna	gain	BASIS
PD24186	E2F3	cna	amp	BASIS
PD24202	E2F3	cna	gain	BASIS
PD24206	E2F3	cna	gain	BASIS
PD3905	E2F3	cna	amp	BASIS
PD4005	E2F3	cna	gain	BASIS
PD4006	E2F3	cna	gain	BASIS
PD4107	E2F3	cna	gain	BASIS
PD4826	E2F3	cna	gain	BASIS
PD5935	E2F3	cna	gain	BASIS
PD5945	E2F3	cna	amp	BASIS
PD5948	E2F3	cna	amp	BASIS
PD6731	E2F3	cna	hetloss	BASIS
PD7067	E2F3	cna	amp	BASIS
PD7215	E2F3	cna	gain	BASIS
PD8621	E2F3	cna	amp	BASIS
PD8980	E2F3	cna	gain	BASIS

PD9004	E2F3	cna	amp	BASIS
PD9702	E2F3	cna	gain	BASIS
TCGA-A2-A25B	EED	cna	hetloss	TCGA
TCGA-AN-A0XU	EED	cna	gain	TCGA
TCGA-AO-A0JL	EED	cna	hetloss	TCGA
TCGA-C8-A12L	EED	cna	gain	TCGA
TCGA-D8-A27M	EED	cna	gain	TCGA
TCGA-EW-A1OX	EED	cna	homdel	TCGA
TCGA-LL-A5YP	EED	cna	hetloss	TCGA
MB-0346	EED	cna	gain	METABRIC
MB-5070	EED	cna	hetloss	METABRIC
MB-5107	EED	cna	amp	METABRIC
MB-6271	EED	cna	hetloss	METABRIC
MB-7032	EED	cna	hetloss	METABRIC
MTS-T0064	EED	cna	gain	METABRIC
PD10014	EED	cna	gain	BASIS
PD11327	EED	cna	gain	BASIS
PD11742	EED	cna	hetloss	BASIS
PD13771	EED	cna	hetloss	BASIS
PD22355	EED	cna	hetloss	BASIS
PD23574	EED	cna	gain	BASIS
PD23578	EED	cna	hetloss	BASIS
PD24186	EED	cna	gain	BASIS
PD24206	EED	cna	gain	BASIS
PD24337	EED	cna	hetloss	BASIS
PD3905	EED	cna	gain	BASIS
PD4005	EED	cna	gain	BASIS
PD4006	EED	cna	hetloss	BASIS
PD4826	EED	cna	gain	BASIS
PD5930	EED	cna	gain	BASIS
PD5935	EED	cna	gain	BASIS
PD5945	EED	cna	gain	BASIS
PD5948	EED	cna	gain	BASIS
PD6413	EED	cna	hetloss	BASIS
PD7215	EED	cna	gain	BASIS
PD8980	EED	cna	gain	BASIS
PD9004	EED	cna	gain	BASIS
PD9585	EED	cna	hetloss	BASIS
PD9702	EED	cna	gain	BASIS
TCGA-A2-A25B	EIF4EBP1	cna	hetloss	TCGA
TCGA-AN-A0XU	EIF4EBP1	cna	hetloss	TCGA
TCGA-AO-A0JL	EIF4EBP1	cna	gain	TCGA
TCGA-BH-A0C0	EIF4EBP1	cna	hetloss	TCGA
TCGA-BH-A1FU	EIF4EBP1	cna	homdel	TCGA
TCGA-D8-A27M	EIF4EBP1	cna	hetloss	TCGA
TCGA-E2-A1L7	EIF4EBP1	cna	hetloss	TCGA
TCGA-LL-A5YP	EIF4EBP1	cna	hetloss	TCGA

MB-0346	EIF4EBP1	cna	hetloss	METABRIC
MB-5465	EIF4EBP1	cna	hetloss	METABRIC
MB-6060	EIF4EBP1	cna	gain	METABRIC
MB-7038	EIF4EBP1	cna	hetloss	METABRIC
MB-0420	EIF4EBP1	cna	hetloss	METABRIC
PD10014	EIF4EBP1	cna	hetloss	BASIS
PD11327	EIF4EBP1	cna	homdel	BASIS
PD13296	EIF4EBP1	cna	hetloss	BASIS
PD13299	EIF4EBP1	cna	gain	BASIS
PD13771	EIF4EBP1	cna	hetloss	BASIS
PD14442	EIF4EBP1	cna	hetloss	BASIS
PD23562	EIF4EBP1	cna	gain	BASIS
PD23578	EIF4EBP1	cna	hetloss	BASIS
PD24186	EIF4EBP1	cna	gain	BASIS
PD24202	EIF4EBP1	cna	hetloss	BASIS
PD24206	EIF4EBP1	cna	amp	BASIS
PD24337	EIF4EBP1	cna	hetloss	BASIS
PD3905	EIF4EBP1	cna	gain	BASIS
PD4006	EIF4EBP1	cna	gain	BASIS
PD4107	EIF4EBP1	cna	gain	BASIS
PD4826	EIF4EBP1	cna	hetloss	BASIS
PD5935	EIF4EBP1	cna	gain	BASIS
PD5945	EIF4EBP1	cna	gain	BASIS
PD7067	EIF4EBP1	cna	gain	BASIS
PD7215	EIF4EBP1	cna	amp	BASIS
PD8980	EIF4EBP1	cna	amp	BASIS
PD9004	EIF4EBP1	cna	gain	BASIS
PD9585	EIF4EBP1	cna	hetloss	BASIS
PD9702	EIF4EBP1	cna	gain	BASIS
TCGA-A2-A25B	EPHB3	cna	amp	TCGA
TCGA-AN-A0XU	EPHB3	cna	gain	TCGA
TCGA-BH-A0AW	EPHB3	cna	gain	TCGA
TCGA-BH-A0C0	EPHB3	cna	gain	TCGA
TCGA-BH-A1FU	EPHB3	cna	hetloss	TCGA
TCGA-C8-A12L	EPHB3	cna	gain	TCGA
TCGA-E2-A1L7	EPHB3	cna	gain	TCGA
TCGA-LL-A5YP	EPHB3	cna	gain	TCGA
MB-0346	EPHB3	cna	gain	METABRIC
MB-5107	EPHB3	cna	amp	METABRIC
MB-6098	EPHB3	cna	gain	METABRIC
MB-0420	EPHB3	cna	gain	METABRIC
MTS-T0064	EPHB3	cna	amp	METABRIC
PD11327	EPHB3	cna	homdel	BASIS
PD11742	EPHB3	cna	gain	BASIS
PD13299	EPHB3	cna	gain	BASIS
PD13771	EPHB3	cna	hetloss	BASIS
PD22355	EPHB3	cna	gain	BASIS

PD23561	EPHB3	cna	hetloss	BASIS
PD23562	EPHB3	cna	gain	BASIS
PD23578	EPHB3	cna	gain	BASIS
PD24186	EPHB3	cna	amp	BASIS
PD24202	EPHB3	cna	gain	BASIS
PD24206	EPHB3	cna	gain	BASIS
PD3905	EPHB3	cna	gain	BASIS
PD4005	EPHB3	cna	gain	BASIS
PD4107	EPHB3	cna	gain	BASIS
PD4826	EPHB3	cna	gain	BASIS
PD5945	EPHB3	cna	amp	BASIS
PD5948	EPHB3	cna	gain	BASIS
PD6406	EPHB3	cna	gain	BASIS
PD6731	EPHB3	cna	gain	BASIS
PD7067	EPHB3	cna	gain	BASIS
PD7215	EPHB3	cna	gain	BASIS
PD8621	EPHB3	cna	gain	BASIS
PD8980	EPHB3	cna	gain	BASIS
PD9702	EPHB3	cna	gain	BASIS
TCGA-A2-A25B	EPHB4	cna	gain	TCGA
TCGA-AN-A0XU	EPHB4	cna	hetloss	TCGA
TCGA-AO-A0JL	EPHB4	cna	gain	TCGA
TCGA-BH-A0AW	EPHB4	cna	gain	TCGA
TCGA-C8-A12L	EPHB4	cna	hetloss	TCGA
TCGA-D8-A27M	EPHB4	cna	gain	TCGA
TCGA-E2-A1L7	EPHB4	cna	hetloss	TCGA
TCGA-E9-A1NC	EPHB4	cna	gain	TCGA
TCGA-LL-A5YP	EPHB4	cna	gain	TCGA
MB-0346	EPHB4	cna	hetloss	METABRIC
MB-2827	EPHB4	cna	hetloss	METABRIC
MB-5465	EPHB4	cna	hetloss	METABRIC
MB-7038	EPHB4	cna	gain	METABRIC
PD11327	EPHB4	cna	gain	BASIS
PD13296	EPHB4	cna	hetloss	BASIS
PD13299	EPHB4	cna	hetloss	BASIS
PD22355	EPHB4	cna	hetloss	BASIS
PD23574	EPHB4	cna	gain	BASIS
PD23578	EPHB4	cna	hetloss	BASIS
PD24186	EPHB4	cna	amp	BASIS
PD24206	EPHB4	cna	homdel	BASIS
PD3890	EPHB4	cna	hetloss	BASIS
PD3905	EPHB4	cna	gain	BASIS
PD4006	EPHB4	cna	amp	BASIS
PD4107	EPHB4	cna	gain	BASIS
PD4826	EPHB4	cna	gain	BASIS
PD5930	EPHB4	cna	gain	BASIS
PD5935	EPHB4	cna	gain	BASIS

PD5945	EPHB4	cna	amp	BASIS
PD5948	EPHB4	cna	gain	BASIS
PD6406	EPHB4	cna	gain	BASIS
PD7067	EPHB4	cna	amp	BASIS
PD7215	EPHB4	cna	gain	BASIS
PD8621	EPHB4	cna	gain	BASIS
PD9004	EPHB4	cna	amp	BASIS
PD9585	EPHB4	cna	hetloss	BASIS
PD9702	EPHB4	cna	gain	BASIS
TCGA-A2-A25B	ERCC5	cna	hetloss	TCGA
TCGA-AN-A0XU	ERCC5	cna	hetloss	TCGA
TCGA-AO-A0JL	ERCC5	cna	hetloss	TCGA
TCGA-BH-A0C0	ERCC5	cna	hetloss	TCGA
TCGA-BH-A1FU	ERCC5	cna	hetloss	TCGA
TCGA-C8-A12L	ERCC5	cna	gain	TCGA
TCGA-D8-A27M	ERCC5	cna	gain	TCGA
TCGA-LL-A5YP	ERCC5	cna	hetloss	TCGA
MB-5107	ERCC5	cna	hetloss	METABRIC
MB-5465	ERCC5	cna	gain	METABRIC
MB-6060	ERCC5	cna	hetloss	METABRIC
MB-0420	ERCC5	cna	amp	METABRIC
PD10014	ERCC5	cna	hetloss	BASIS
PD11327	ERCC5	cna	amp	BASIS
PD13296	ERCC5	cna	hetloss	BASIS
PD13299	ERCC5	cna	gain	BASIS
PD14442	ERCC5	cna	hetloss	BASIS
PD23562	ERCC5	cna	gain	BASIS
PD23574	ERCC5	cna	gain	BASIS
PD23578	ERCC5	cna	gain	BASIS
PD24186	ERCC5	cna	gain	BASIS
PD24202	ERCC5	cna	gain	BASIS
PD24206	ERCC5	cna	gain	BASIS
PD24337	ERCC5	cna	hetloss	BASIS
PD3905	ERCC5	cna	amp	BASIS
PD4005	ERCC5	cna	hetloss	BASIS
PD4006	ERCC5	cna	hetloss	BASIS
PD4107	ERCC5	cna	hetloss	BASIS
PD4967	ERCC5	cna	hetloss	BASIS
PD5945	ERCC5	cna	amp	BASIS
PD5948	ERCC5	cna	hetloss	BASIS
PD6731	ERCC5	cna	hetloss	BASIS
PD7215	ERCC5	cna	gain	BASIS
PD8621	ERCC5	cna	amp	BASIS
PD8980	ERCC5	cna	hetloss	BASIS
PD9585	ERCC5	cna	gain	BASIS
PD9702	ERCC5	cna	gain	BASIS
TCGA-A2-A25B	ETS1	cna	hetloss	TCGA

TCGA-AO-A0JL	ETS1	cna	hetloss	TCGA
TCGA-BH-A0C0	ETS1	cna	hetloss	TCGA
TCGA-BH-A18R	ETS1	cna	homdel	TCGA
TCGA-D8-A27M	ETS1	cna	hetloss	TCGA
TCGA-E2-A1L7	ETS1	cna	hetloss	TCGA
TCGA-E9-A1NC	ETS1	cna	hetloss	TCGA
TCGA-EW-A1OX	ETS1	cna	homdel	TCGA
TCGA-LL-A5YP	ETS1	cna	gain	TCGA
MB-0346	ETS1	cna	hetloss	METABRIC
MB-5107	ETS1	cna	hetloss	METABRIC
MB-6271	ETS1	cna	hetloss	METABRIC
PD11327	ETS1	cna	gain	BASIS
PD11742	ETS1	cna	hetloss	BASIS
PD13296	ETS1	cna	hetloss	BASIS
PD13297	ETS1	cna	hetloss	BASIS
PD13771	ETS1	cna	hetloss	BASIS
PD22355	ETS1	cna	hetloss	BASIS
PD23574	ETS1	cna	gain	BASIS
PD23578	ETS1	cna	hetloss	BASIS
PD24186	ETS1	cna	gain	BASIS
PD24202	ETS1	cna	gain	BASIS
PD24206	ETS1	cna	gain	BASIS
PD24337	ETS1	cna	hetloss	BASIS
PD3890	ETS1	cna	hetloss	BASIS
PD3905	ETS1	cna	gain	BASIS
PD4005	ETS1	cna	homdel	BASIS
PD4826	ETS1	cna	gain	BASIS
PD5935	ETS1	cna	gain	BASIS
PD5945	ETS1	cna	amp	BASIS
PD5948	ETS1	cna	gain	BASIS
PD6413	ETS1	cna	hetloss	BASIS
PD6731	ETS1	cna	hetloss	BASIS
PD7067	ETS1	cna	gain	BASIS
PD7215	ETS1	cna	gain	BASIS
PD9004	ETS1	cna	hetloss	BASIS
PD9702	ETS1	cna	gain	BASIS
TCGA-A2-A25B	ETV5	cna	amp	TCGA
TCGA-AN-A0XU	ETV5	cna	gain	TCGA
TCGA-BH-A0AW	ETV5	cna	gain	TCGA
TCGA-BH-A0C0	ETV5	cna	gain	TCGA
TCGA-BH-A1FU	ETV5	cna	hetloss	TCGA
TCGA-C8-A12L	ETV5	cna	gain	TCGA
TCGA-LL-A5YP	ETV5	cna	gain	TCGA
MB-0346	ETV5	cna	gain	METABRIC
MB-5070	ETV5	cna	hetloss	METABRIC
MB-5107	ETV5	cna	amp	METABRIC
MB-6098	ETV5	cna	gain	METABRIC

MB-7038	ETV5	cna	hetloss	METABRIC
MB-0420	ETV5	cna	gain	METABRIC
PD11327	ETV5	cna	homdel	BASIS
PD13299	ETV5	cna	gain	BASIS
PD13771	ETV5	cna	hetloss	BASIS
PD22355	ETV5	cna	gain	BASIS
PD23561	ETV5	cna	hetloss	BASIS
PD23562	ETV5	cna	gain	BASIS
PD23578	ETV5	cna	gain	BASIS
PD24186	ETV5	cna	amp	BASIS
PD24202	ETV5	cna	gain	BASIS
PD24206	ETV5	cna	hetloss	BASIS
PD3905	ETV5	cna	gain	BASIS
PD4005	ETV5	cna	gain	BASIS
PD4107	ETV5	cna	gain	BASIS
PD4826	ETV5	cna	gain	BASIS
PD5945	ETV5	cna	amp	BASIS
PD5948	ETV5	cna	gain	BASIS
PD6406	ETV5	cna	gain	BASIS
PD6731	ETV5	cna	gain	BASIS
PD7067	ETV5	cna	gain	BASIS
PD7215	ETV5	cna	gain	BASIS
PD8621	ETV5	cna	gain	BASIS
PD8980	ETV5	cna	gain	BASIS
PD9004	ETV5	cna	gain	BASIS
PD9702	ETV5	cna	gain	BASIS
TCGA-A2-A25B	FAM175A	cna	hetloss	TCGA
TCGA-AN-A0XU	FAM175A	cna	hetloss	TCGA
TCGA-AO-A0JL	FAM175A	cna	gain	TCGA
TCGA-BH-A0AW	FAM175A	cna	hetloss	TCGA
TCGA-BH-A0C0	FAM175A	cna	gain	TCGA
TCGA-BH-A1FU	FAM175A	cna	gain	TCGA
TCGA-D8-A27M	FAM175A	cna	gain	TCGA
TCGA-E2-A1L7	FAM175A	cna	hetloss	TCGA
TCGA-E9-A1NC	FAM175A	cna	hetloss	TCGA
TCGA-LL-A5YP	FAM175A	cna	gain	TCGA
MB-5070	FAM175A	cna	hetloss	METABRIC
MB-5465	FAM175A	cna	hetloss	METABRIC
MB-6060	FAM175A	cna	hetloss	METABRIC
MB-0420	FAM175A	cna	hetloss	METABRIC
PD11327	FAM175A	cna	homdel	BASIS
PD11742	FAM175A	cna	hetloss	BASIS
PD13296	FAM175A	cna	hetloss	BASIS
PD13297	FAM175A	cna	hetloss	BASIS
PD13771	FAM175A	cna	gain	BASIS
PD22355	FAM175A	cna	hetloss	BASIS
PD23562	FAM175A	cna	gain	BASIS

PD23578	FAM175A	cna	hetloss	BASIS
PD24202	FAM175A	cna	hetloss	BASIS
PD24206	FAM175A	cna	gain	BASIS
PD3890	FAM175A	cna	gain	BASIS
PD3905	FAM175A	cna	gain	BASIS
PD4107	FAM175A	cna	gain	BASIS
PD4826	FAM175A	cna	gain	BASIS
PD5930	FAM175A	cna	hetloss	BASIS
PD5935	FAM175A	cna	gain	BASIS
PD5948	FAM175A	cna	gain	BASIS
PD6731	FAM175A	cna	hetloss	BASIS
PD7215	FAM175A	cna	gain	BASIS
PD8621	FAM175A	cna	hetloss	BASIS
PD8980	FAM175A	cna	hetloss	BASIS
PD9585	FAM175A	cna	hetloss	BASIS
PD9702	FAM175A	cna	hetloss	BASIS
TCGA-A2-A25B	FLI1	cna	hetloss	TCGA
TCGA-AO-A0JL	FLI1	cna	hetloss	TCGA
TCGA-BH-A0C0	FLI1	cna	hetloss	TCGA
TCGA-BH-A18R	FLI1	cna	homdel	TCGA
TCGA-D8-A27M	FLI1	cna	hetloss	TCGA
TCGA-E2-A1L7	FLI1	cna	hetloss	TCGA
TCGA-E9-A1NC	FLI1	cna	hetloss	TCGA
TCGA-EW-A1OX	FLI1	cna	homdel	TCGA
TCGA-LL-A5YP	FLI1	cna	gain	TCGA
MB-0346	FLI1	cna	hetloss	METABRIC
MB-5107	FLI1	cna	hetloss	METABRIC
MB-6271	FLI1	cna	hetloss	METABRIC
PD11327	FLI1	cna	gain	BASIS
PD11742	FLI1	cna	hetloss	BASIS
PD13296	FLI1	cna	hetloss	BASIS
PD13297	FLI1	cna	hetloss	BASIS
PD13771	FLI1	cna	hetloss	BASIS
PD22355	FLI1	cna	hetloss	BASIS
PD23574	FLI1	cna	gain	BASIS
PD23578	FLI1	cna	hetloss	BASIS
PD24186	FLI1	cna	gain	BASIS
PD24202	FLI1	cna	gain	BASIS
PD24206	FLI1	cna	gain	BASIS
PD24337	FLI1	cna	hetloss	BASIS
PD3890	FLI1	cna	hetloss	BASIS
PD3905	FLI1	cna	gain	BASIS
PD4005	FLI1	cna	homdel	BASIS
PD4826	FLI1	cna	gain	BASIS
PD5935	FLI1	cna	gain	BASIS
PD5945	FLI1	cna	amp	BASIS
PD5948	FLI1	cna	gain	BASIS

PD6413	FLI1	cna	hetloss	BASIS
PD6731	FLI1	cna	hetloss	BASIS
PD7067	FLI1	cna	gain	BASIS
PD7215	FLI1	cna	gain	BASIS
PD9004	FLI1	cna	hetloss	BASIS
PD9702	FLI1	cna	gain	BASIS
TCGA-AO-A0JL	FLT1	cna	hetloss	TCGA
TCGA-BH-A0AW	FLT1	cna	hetloss	TCGA
TCGA-BH-A0C0	FLT1	cna	gain	TCGA
TCGA-BH-A1FU	FLT1	cna	hetloss	TCGA
TCGA-C8-A12L	FLT1	cna	hetloss	TCGA
TCGA-D8-A27M	FLT1	cna	gain	TCGA
TCGA-E9-A1NC	FLT1	cna	hetloss	TCGA
TCGA-EW-A1OX	FLT1	cna	homdel	TCGA
TCGA-LL-A5YP	FLT1	cna	hetloss	TCGA
MB-0346	FLT1	cna	amp	METABRIC
MB-2827	FLT1	cna	hetloss	METABRIC
MB-6098	FLT1	cna	hetloss	METABRIC
MB-7032	FLT1	cna	gain	METABRIC
MB-0420	FLT1	cna	hetloss	METABRIC
PD10014	FLT1	cna	hetloss	BASIS
PD13296	FLT1	cna	hetloss	BASIS
PD13297	FLT1	cna	hetloss	BASIS
PD13771	FLT1	cna	hetloss	BASIS
PD14442	FLT1	cna	hetloss	BASIS
PD22355	FLT1	cna	hetloss	BASIS
PD23562	FLT1	cna	gain	BASIS
PD23578	FLT1	cna	hetloss	BASIS
PD24202	FLT1	cna	hetloss	BASIS
PD3890	FLT1	cna	hetloss	BASIS
PD3905	FLT1	cna	gain	BASIS
PD4005	FLT1	cna	homdel	BASIS
PD4006	FLT1	cna	gain	BASIS
PD4107	FLT1	cna	gain	BASIS
PD4826	FLT1	cna	hetloss	BASIS
PD4967	FLT1	cna	hetloss	BASIS
PD5945	FLT1	cna	amp	BASIS
PD6406	FLT1	cna	hetloss	BASIS
PD6413	FLT1	cna	hetloss	BASIS
PD6731	FLT1	cna	hetloss	BASIS
PD7067	FLT1	cna	gain	BASIS
PD8980	FLT1	cna	hetloss	BASIS
PD9702	FLT1	cna	gain	BASIS
TCGA-A2-A25B	GAS7	cna	hetloss	TCGA
TCGA-AN-A0XU	GAS7	cna	hetloss	TCGA
TCGA-AO-A0JL	GAS7	cna	gain	TCGA
TCGA-BH-A0AW	GAS7	cna	hetloss	TCGA

TCGA-BH-A0C0	GAS7	cna	hetloss	TCGA
TCGA-C8-A12L	GAS7	cna	hetloss	TCGA
TCGA-D8-A27M	GAS7	cna	hetloss	TCGA
TCGA-E2-A1L7	GAS7	cna	hetloss	TCGA
TCGA-E9-A1NC	GAS7	cna	hetloss	TCGA
TCGA-LL-A5YP	GAS7	cna	gain	TCGA
MB-0346	GAS7	cna	hetloss	METABRIC
MB-2827	GAS7	cna	hetloss	METABRIC
MB-5070	GAS7	cna	hetloss	METABRIC
MB-5107	GAS7	cna	hetloss	METABRIC
MB-6098	GAS7	cna	hetloss	METABRIC
MB-6271	GAS7	cna	hetloss	METABRIC
MB-7048	GAS7	cna	hetloss	METABRIC
MB-0420	GAS7	cna	hetloss	METABRIC
PD11742	GAS7	cna	hetloss	BASIS
PD13296	GAS7	cna	hetloss	BASIS
PD13771	GAS7	cna	hetloss	BASIS
PD22355	GAS7	cna	hetloss	BASIS
PD23561	GAS7	cna	hetloss	BASIS
PD24202	GAS7	cna	hetloss	BASIS
PD24206	GAS7	cna	hetloss	BASIS
PD24337	GAS7	cna	hetloss	BASIS
PD3890	GAS7	cna	hetloss	BASIS
PD3905	GAS7	cna	gain	BASIS
PD4005	GAS7	cna	homdel	BASIS
PD4826	GAS7	cna	hetloss	BASIS
PD4967	GAS7	cna	hetloss	BASIS
PD5945	GAS7	cna	gain	BASIS
PD6406	GAS7	cna	hetloss	BASIS
PD6413	GAS7	cna	hetloss	BASIS
PD6731	GAS7	cna	hetloss	BASIS
PD7067	GAS7	cna	gain	BASIS
PD9585	GAS7	cna	hetloss	BASIS
TCGA-A2-A25B	GATA1	cna	gain	TCGA
TCGA-AN-A0XU	GATA1	cna	hetloss	TCGA
TCGA-AO-A0JL	GATA1	cna	gain	TCGA
TCGA-BH-A0AW	GATA1	cna	gain	TCGA
TCGA-E2-A1L7	GATA1	cna	hetloss	TCGA
TCGA-E9-A1NC	GATA1	cna	hetloss	TCGA
MB-0346	GATA1	cna	gain	METABRIC
MB-2827	GATA1	cna	hetloss	METABRIC
MB-5465	GATA1	cna	gain	METABRIC
MB-6098	GATA1	cna	hetloss	METABRIC
MB-0420	GATA1	cna	hetloss	METABRIC
PD10014	GATA1	cna	hetloss	BASIS
PD11327	GATA1	cna	homdel	BASIS
PD13296	GATA1	cna	gain	BASIS

PD13297	GATA1	cna	gain	BASIS
PD13299	GATA1	cna	amp	BASIS
PD13771	GATA1	cna	gain	BASIS
PD14442	GATA1	cna	gain	BASIS
PD23562	GATA1	cna	hetloss	BASIS
PD23574	GATA1	cna	gain	BASIS
PD24206	GATA1	cna	hetloss	BASIS
PD3905	GATA1	cna	gain	BASIS
PD4005	GATA1	cna	hetloss	BASIS
PD4006	GATA1	cna	hetloss	BASIS
PD4107	GATA1	cna	gain	BASIS
PD4826	GATA1	cna	gain	BASIS
PD4967	GATA1	cna	gain	BASIS
PD5935	GATA1	cna	gain	BASIS
PD5945	GATA1	cna	gain	BASIS
PD5948	GATA1	cna	gain	BASIS
PD6406	GATA1	cna	gain	BASIS
PD6413	GATA1	cna	gain	BASIS
PD7067	GATA1	cna	gain	BASIS
PD7215	GATA1	cna	amp	BASIS
PD8621	GATA1	cna	amp	BASIS
PD8980	GATA1	cna	hetloss	BASIS
PD9004	GATA1	cna	gain	BASIS
TCGA-AN-A0XU	GATA2	cna	gain	TCGA
TCGA-BH-A0C0	GATA2	cna	gain	TCGA
TCGA-BH-A18R	GATA2	cna	gain	TCGA
TCGA-BH-A1FU	GATA2	cna	hetloss	TCGA
TCGA-C8-A12L	GATA2	cna	gain	TCGA
TCGA-E2-A1L7	GATA2	cna	hetloss	TCGA
TCGA-LL-A5YP	GATA2	cna	gain	TCGA
MB-0346	GATA2	cna	hetloss	METABRIC
MB-5070	GATA2	cna	hetloss	METABRIC
MB-5107	GATA2	cna	gain	METABRIC
MB-5465	GATA2	cna	gain	METABRIC
MB-6098	GATA2	cna	hetloss	METABRIC
MB-7038	GATA2	cna	gain	METABRIC
PD10014	GATA2	cna	gain	BASIS
PD11327	GATA2	cna	gain	BASIS
PD13297	GATA2	cna	hetloss	BASIS
PD13299	GATA2	cna	gain	BASIS
PD13771	GATA2	cna	hetloss	BASIS
PD22355	GATA2	cna	hetloss	BASIS
PD23561	GATA2	cna	hetloss	BASIS
PD23574	GATA2	cna	gain	BASIS
PD24186	GATA2	cna	gain	BASIS
PD24206	GATA2	cna	gain	BASIS
PD3905	GATA2	cna	gain	BASIS

PD4005	GATA2	cna	hetloss	BASIS
PD4107	GATA2	cna	gain	BASIS
PD4826	GATA2	cna	gain	BASIS
PD5935	GATA2	cna	gain	BASIS
PD5945	GATA2	cna	gain	BASIS
PD5948	GATA2	cna	gain	BASIS
PD6731	GATA2	cna	gain	BASIS
PD7067	GATA2	cna	gain	BASIS
PD7215	GATA2	cna	gain	BASIS
PD8621	GATA2	cna	gain	BASIS
PD8980	GATA2	cna	gain	BASIS
PD9004	GATA2	cna	gain	BASIS
PD9702	GATA2	cna	gain	BASIS
TCGA-A2-A25B	HOXC11	cna	gain	TCGA
TCGA-AN-A0XU	HOXC11	cna	gain	TCGA
TCGA-AO-A0JL	HOXC11	cna	hetloss	TCGA
TCGA-BH-A18R	HOXC11	cna	gain	TCGA
TCGA-BH-A1FU	HOXC11	cna	hetloss	TCGA
TCGA-C8-A12L	HOXC11	cna	gain	TCGA
TCGA-D8-A27M	HOXC11	cna	hetloss	TCGA
TCGA-E2-A1L7	HOXC11	cna	hetloss	TCGA
TCGA-E9-A1NC	HOXC11	cna	hetloss	TCGA
TCGA-LL-A5YP	HOXC11	cna	hetloss	TCGA
MB-0346	HOXC11	cna	gain	METABRIC
MB-2827	HOXC11	cna	hetloss	METABRIC
MB-5070	HOXC11	cna	hetloss	METABRIC
MB-6060	HOXC11	cna	gain	METABRIC
MB-6098	HOXC11	cna	hetloss	METABRIC
MB-7038	HOXC11	cna	hetloss	METABRIC
MB-0420	HOXC11	cna	hetloss	METABRIC
PD11327	HOXC11	cna	hetloss	BASIS
PD13296	HOXC11	cna	hetloss	BASIS
PD13297	HOXC11	cna	hetloss	BASIS
PD23578	HOXC11	cna	hetloss	BASIS
PD24202	HOXC11	cna	hetloss	BASIS
PD24206	HOXC11	cna	gain	BASIS
PD24337	HOXC11	cna	hetloss	BASIS
PD3890	HOXC11	cna	hetloss	BASIS
PD3905	HOXC11	cna	gain	BASIS
PD4107	HOXC11	cna	hetloss	BASIS
PD4826	HOXC11	cna	gain	BASIS
PD5945	HOXC11	cna	gain	BASIS
PD5948	HOXC11	cna	gain	BASIS
PD6406	HOXC11	cna	hetloss	BASIS
PD6413	HOXC11	cna	hetloss	BASIS
PD6731	HOXC11	cna	hetloss	BASIS
PD7067	HOXC11	cna	gain	BASIS

PD8621	HOXC11	cna	hetloss	BASIS
PD9585	HOXC11	cna	hetloss	BASIS
PD9702	HOXC11	cna	gain	BASIS
TCGA-A2-A25B	HOXC13	cna	gain	TCGA
TCGA-AN-A0XU	HOXC13	cna	gain	TCGA
TCGA-AO-A0JL	HOXC13	cna	hetloss	TCGA
TCGA-BH-A18R	HOXC13	cna	gain	TCGA
TCGA-BH-A1FU	HOXC13	cna	hetloss	TCGA
TCGA-C8-A12L	HOXC13	cna	gain	TCGA
TCGA-D8-A27M	HOXC13	cna	hetloss	TCGA
TCGA-E2-A1L7	HOXC13	cna	hetloss	TCGA
TCGA-E9-A1NC	HOXC13	cna	hetloss	TCGA
TCGA-LL-A5YP	HOXC13	cna	hetloss	TCGA
MB-0346	HOXC13	cna	gain	METABRIC
MB-2827	HOXC13	cna	hetloss	METABRIC
MB-5070	HOXC13	cna	hetloss	METABRIC
MB-6060	HOXC13	cna	gain	METABRIC
MB-6098	HOXC13	cna	hetloss	METABRIC
MB-7038	HOXC13	cna	hetloss	METABRIC
MB-0420	HOXC13	cna	hetloss	METABRIC
PD11327	HOXC13	cna	hetloss	BASIS
PD13296	HOXC13	cna	hetloss	BASIS
PD13297	HOXC13	cna	hetloss	BASIS
PD23578	HOXC13	cna	hetloss	BASIS
PD24202	HOXC13	cna	hetloss	BASIS
PD24206	HOXC13	cna	gain	BASIS
PD24337	HOXC13	cna	hetloss	BASIS
PD3890	HOXC13	cna	hetloss	BASIS
PD3905	HOXC13	cna	gain	BASIS
PD4107	HOXC13	cna	hetloss	BASIS
PD4826	HOXC13	cna	gain	BASIS
PD5945	HOXC13	cna	gain	BASIS
PD5948	HOXC13	cna	gain	BASIS
PD6406	HOXC13	cna	hetloss	BASIS
PD6413	HOXC13	cna	hetloss	BASIS
PD6731	HOXC13	cna	hetloss	BASIS
PD7067	HOXC13	cna	gain	BASIS
PD8621	HOXC13	cna	hetloss	BASIS
PD9585	HOXC13	cna	hetloss	BASIS
PD9702	HOXC13	cna	gain	BASIS
TCGA-A2-A25B	IGF2	cna	hetloss	TCGA
TCGA-AN-A0XU	IGF2	cna	hetloss	TCGA
TCGA-AO-A0JL	IGF2	cna	hetloss	TCGA
TCGA-BH-A0C0	IGF2	cna	hetloss	TCGA
TCGA-C8-A12L	IGF2	cna	hetloss	TCGA
TCGA-E2-A1L7	IGF2	cna	homdel	TCGA
TCGA-LL-A5YP	IGF2	cna	hetloss	TCGA

MB-0346	IGF2	cna	hetloss	METABRIC
MB-5070	IGF2	cna	hetloss	METABRIC
MB-6060	IGF2	cna	hetloss	METABRIC
MB-6098	IGF2	cna	hetloss	METABRIC
MB-6271	IGF2	cna	gain	METABRIC
MB-7032	IGF2	cna	gain	METABRIC
MB-7048	IGF2	cna	hetloss	METABRIC
MB-0420	IGF2	cna	hetloss	METABRIC
PD10014	IGF2	cna	hetloss	BASIS
PD11742	IGF2	cna	hetloss	BASIS
PD13296	IGF2	cna	hetloss	BASIS
PD13297	IGF2	cna	hetloss	BASIS
PD22355	IGF2	cna	hetloss	BASIS
PD24202	IGF2	cna	hetloss	BASIS
PD24206	IGF2	cna	gain	BASIS
PD3890	IGF2	cna	hetloss	BASIS
PD3905	IGF2	cna	gain	BASIS
PD4005	IGF2	cna	hetloss	BASIS
PD4006	IGF2	cna	gain	BASIS
PD4107	IGF2	cna	gain	BASIS
PD4826	IGF2	cna	gain	BASIS
PD4967	IGF2	cna	gain	BASIS
PD5935	IGF2	cna	gain	BASIS
PD5945	IGF2	cna	gain	BASIS
PD5948	IGF2	cna	gain	BASIS
PD6413	IGF2	cna	hetloss	BASIS
PD6731	IGF2	cna	hetloss	BASIS
PD8980	IGF2	cna	hetloss	BASIS
PD9004	IGF2	cna	hetloss	BASIS
PD9585	IGF2	cna	hetloss	BASIS
TCGA-AN-A0XU	IL6ST	cna	hetloss	TCGA
TCGA-AO-A0JL	IL6ST	cna	hetloss	TCGA
TCGA-BH-A0C0	IL6ST	cna	hetloss	TCGA
TCGA-BH-A18R	IL6ST	cna	gain	TCGA
TCGA-BH-A1FU	IL6ST	cna	hetloss	TCGA
TCGA-C8-A12L	IL6ST	cna	hetloss	TCGA
TCGA-E2-A1L7	IL6ST	cna	hetloss	TCGA
TCGA-E9-A1NC	IL6ST	cna	hetloss	TCGA
TCGA-LL-A5YP	IL6ST	cna	amp	TCGA
MB-0346	IL6ST	cna	gain	METABRIC
MB-2827	IL6ST	cna	hetloss	METABRIC
MB-5107	IL6ST	cna	hetloss	METABRIC
MB-5465	IL6ST	cna	hetloss	METABRIC
MB-6098	IL6ST	cna	gain	METABRIC
MB-0420	IL6ST	cna	hetloss	METABRIC
PD10014	IL6ST	cna	hetloss	BASIS
PD11327	IL6ST	cna	hetloss	BASIS

PD11742	IL6ST	cna	hetloss	BASIS
PD13296	IL6ST	cna	hetloss	BASIS
PD13297	IL6ST	cna	hetloss	BASIS
PD13771	IL6ST	cna	hetloss	BASIS
PD23562	IL6ST	cna	hetloss	BASIS
PD23578	IL6ST	cna	hetloss	BASIS
PD24186	IL6ST	cna	gain	BASIS
PD24202	IL6ST	cna	hetloss	BASIS
PD24206	IL6ST	cna	gain	BASIS
PD3890	IL6ST	cna	hetloss	BASIS
PD3905	IL6ST	cna	gain	BASIS
PD4005	IL6ST	cna	hetloss	BASIS
PD4006	IL6ST	cna	hetloss	BASIS
PD5930	IL6ST	cna	hetloss	BASIS
PD5935	IL6ST	cna	hetloss	BASIS
PD5948	IL6ST	cna	hetloss	BASIS
PD7215	IL6ST	cna	hetloss	BASIS
PD8621	IL6ST	cna	hetloss	BASIS
PD8980	IL6ST	cna	hetloss	BASIS
PD9004	IL6ST	cna	gain	BASIS
TCGA-A2-A25B	JAK1	cna	gain	TCGA
TCGA-AN-A0XU	JAK1	cna	hetloss	TCGA
TCGA-BH-A0AW	JAK1	cna	gain	TCGA
TCGA-BH-A0C0	JAK1	cna	hetloss	TCGA
TCGA-BH-A1FU	JAK1	cna	gain	TCGA
TCGA-D8-A27M	JAK1	cna	hetloss	TCGA
TCGA-E2-A1L7	JAK1	cna	amp	TCGA
TCGA-LL-A5YP	JAK1	cna	hetloss	TCGA
MB-0346	JAK1	cna	hetloss	METABRIC
MB-6098	JAK1	cna	hetloss	METABRIC
MB-7038	JAK1	cna	gain	METABRIC
PD11327	JAK1	cna	gain	BASIS
PD11742	JAK1	cna	hetloss	BASIS
PD13296	JAK1	cna	hetloss	BASIS
PD13297	JAK1	cna	hetloss	BASIS
PD13299	JAK1	cna	gain	BASIS
PD22355	JAK1	cna	hetloss	BASIS
PD23562	JAK1	cna	gain	BASIS
PD23574	JAK1	cna	gain	BASIS
PD24186	JAK1	cna	amp	BASIS
PD24202	JAK1	cna	gain	BASIS
PD24206	JAK1	cna	gain	BASIS
PD3890	JAK1	cna	gain	BASIS
PD3905	JAK1	cna	gain	BASIS
PD4006	JAK1	cna	gain	BASIS
PD4107	JAK1	cna	gain	BASIS
PD4826	JAK1	cna	gain	BASIS

PD5930	JAK1	cna	gain	BASIS
PD5935	JAK1	cna	gain	BASIS
PD5945	JAK1	cna	gain	BASIS
PD5948	JAK1	cna	gain	BASIS
PD7067	JAK1	cna	gain	BASIS
PD7215	JAK1	cna	gain	BASIS
PD8621	JAK1	cna	gain	BASIS
PD9004	JAK1	cna	gain	BASIS
PD9585	JAK1	cna	hetloss	BASIS
PD9702	JAK1	cna	gain	BASIS
TCGA-A2-A25B	KCNJ5	cna	hetloss	TCGA
TCGA-AO-A0JL	KCNJ5	cna	hetloss	TCGA
TCGA-BH-A0C0	KCNJ5	cna	hetloss	TCGA
TCGA-BH-A18R	KCNJ5	cna	homdel	TCGA
TCGA-D8-A27M	KCNJ5	cna	hetloss	TCGA
TCGA-E2-A1L7	KCNJ5	cna	hetloss	TCGA
TCGA-E9-A1NC	KCNJ5	cna	hetloss	TCGA
TCGA-EW-A1OX	KCNJ5	cna	homdel	TCGA
TCGA-LL-A5YP	KCNJ5	cna	gain	TCGA
MB-0346	KCNJ5	cna	hetloss	METABRIC
MB-5107	KCNJ5	cna	hetloss	METABRIC
MB-6271	KCNJ5	cna	hetloss	METABRIC
PD11327	KCNJ5	cna	gain	BASIS
PD11742	KCNJ5	cna	hetloss	BASIS
PD13296	KCNJ5	cna	hetloss	BASIS
PD13297	KCNJ5	cna	hetloss	BASIS
PD13771	KCNJ5	cna	hetloss	BASIS
PD22355	KCNJ5	cna	hetloss	BASIS
PD23574	KCNJ5	cna	gain	BASIS
PD23578	KCNJ5	cna	hetloss	BASIS
PD24186	KCNJ5	cna	gain	BASIS
PD24202	KCNJ5	cna	gain	BASIS
PD24206	KCNJ5	cna	gain	BASIS
PD24337	KCNJ5	cna	hetloss	BASIS
PD3890	KCNJ5	cna	hetloss	BASIS
PD3905	KCNJ5	cna	gain	BASIS
PD4005	KCNJ5	cna	hetloss	BASIS
PD4826	KCNJ5	cna	gain	BASIS
PD5935	KCNJ5	cna	gain	BASIS
PD5945	KCNJ5	cna	amp	BASIS
PD5948	KCNJ5	cna	gain	BASIS
PD6413	KCNJ5	cna	hetloss	BASIS
PD6731	KCNJ5	cna	hetloss	BASIS
PD7067	KCNJ5	cna	gain	BASIS
PD7215	KCNJ5	cna	gain	BASIS
PD9004	KCNJ5	cna	hetloss	BASIS
PD9702	KCNJ5	cna	gain	BASIS

TCGA-A2-A25B	KDM5C	cna	gain	TCGA
TCGA-AN-A0XU	KDM5C	cna	hetloss	TCGA
TCGA-AO-A0JL	KDM5C	cna	gain	TCGA
TCGA-BH-A0AW	KDM5C	cna	gain	TCGA
TCGA-E2-A1L7	KDM5C	cna	hetloss	TCGA
TCGA-E9-A1NC	KDM5C	cna	hetloss	TCGA
MB-0346	KDM5C	cna	gain	METABRIC
MB-2827	KDM5C	cna	hetloss	METABRIC
MB-5465	KDM5C	cna	gain	METABRIC
MB-6098	KDM5C	cna	hetloss	METABRIC
MB-0420	KDM5C	cna	hetloss	METABRIC
PD10014	KDM5C	cna	hetloss	BASIS
PD11327	KDM5C	cna	homdel	BASIS
PD13296	KDM5C	cna	gain	BASIS
PD13297	KDM5C	cna	gain	BASIS
PD13299	KDM5C	cna	gain	BASIS
PD13771	KDM5C	cna	gain	BASIS
PD14442	KDM5C	cna	gain	BASIS
PD23562	KDM5C	cna	hetloss	BASIS
PD23574	KDM5C	cna	gain	BASIS
PD24206	KDM5C	cna	amp	BASIS
PD3905	KDM5C	cna	gain	BASIS
PD4005	KDM5C	cna	hetloss	BASIS
PD4006	KDM5C	cna	hetloss	BASIS
PD4107	KDM5C	cna	gain	BASIS
PD4826	KDM5C	cna	gain	BASIS
PD4967	KDM5C	cna	gain	BASIS
PD5935	KDM5C	cna	gain	BASIS
PD5945	KDM5C	cna	gain	BASIS
PD5948	KDM5C	cna	gain	BASIS
PD6406	KDM5C	cna	gain	BASIS
PD6413	KDM5C	cna	gain	BASIS
PD7067	KDM5C	cna	gain	BASIS
PD7215	KDM5C	cna	amp	BASIS
PD8621	KDM5C	cna	amp	BASIS
PD8980	KDM5C	cna	hetloss	BASIS
PD9004	KDM5C	cna	gain	BASIS
TCGA-A2-A25B	KMT2C	cna	gain	TCGA
TCGA-AN-A0XU	KMT2C	cna	gain	TCGA
TCGA-AO-A0JL	KMT2C	cna	gain	TCGA
TCGA-C8-A12L	KMT2C	cna	hetloss	TCGA
TCGA-D8-A27M	KMT2C	cna	gain	TCGA
TCGA-E2-A1L7	KMT2C	cna	hetloss	TCGA
TCGA-E9-A1NC	KMT2C	cna	hetloss	TCGA
TCGA-LL-A5YP	KMT2C	cna	hetloss	TCGA
MB-0346	KMT2C	cna	hetloss	METABRIC
MB-5070	KMT2C	cna	hetloss	METABRIC

MB-6098	KMT2C	cna	gain	METABRIC
MB-7038	KMT2C	cna	gain	METABRIC
MB-0420	KMT2C	cna	hetloss	METABRIC
PD10014	KMT2C	cna	gain	BASIS
PD13296	KMT2C	cna	hetloss	BASIS
PD13297	KMT2C	cna	gain	BASIS
PD22355	KMT2C	cna	hetloss	BASIS
PD23562	KMT2C	cna	gain	BASIS
PD23574	KMT2C	cna	gain	BASIS
PD23578	KMT2C	cna	hetloss	BASIS
PD24186	KMT2C	cna	gain	BASIS
PD24202	KMT2C	cna	gain	BASIS
PD24337	KMT2C	cna	hetloss	BASIS
PD3890	KMT2C	cna	hetloss	BASIS
PD3905	KMT2C	cna	gain	BASIS
PD4006	KMT2C	cna	amp	BASIS
PD4107	KMT2C	cna	gain	BASIS
PD5930	KMT2C	cna	gain	BASIS
PD5945	KMT2C	cna	amp	BASIS
PD5948	KMT2C	cna	gain	BASIS
PD6406	KMT2C	cna	gain	BASIS
PD6731	KMT2C	cna	hetloss	BASIS
PD7067	KMT2C	cna	gain	BASIS
PD7215	KMT2C	cna	gain	BASIS
PD9004	KMT2C	cna	gain	BASIS
PD9585	KMT2C	cna	hetloss	BASIS
PD9702	KMT2C	cna	gain	BASIS
TCGA-A2-A25B	KTN1	cna	hetloss	TCGA
TCGA-AO-A0JL	KTN1	cna	hetloss	TCGA
TCGA-BH-A0AW	KTN1	cna	hetloss	TCGA
TCGA-BH-A1FU	KTN1	cna	gain	TCGA
TCGA-C8-A12L	KTN1	cna	hetloss	TCGA
TCGA-D8-A27M	KTN1	cna	hetloss	TCGA
TCGA-LL-A5YP	KTN1	cna	hetloss	TCGA
MB-2827	KTN1	cna	hetloss	METABRIC
MB-5070	KTN1	cna	hetloss	METABRIC
MB-5465	KTN1	cna	hetloss	METABRIC
MB-6060	KTN1	cna	gain	METABRIC
MB-7038	KTN1	cna	hetloss	METABRIC
PD10014	KTN1	cna	hetloss	BASIS
PD11327	KTN1	cna	gain	BASIS
PD13296	KTN1	cna	hetloss	BASIS
PD13297	KTN1	cna	hetloss	BASIS
PD22355	KTN1	cna	hetloss	BASIS
PD23574	KTN1	cna	hetloss	BASIS
PD23578	KTN1	cna	hetloss	BASIS
PD24202	KTN1	cna	hetloss	BASIS

PD24206	KTN1	cna	gain	BASIS
PD24337	KTN1	cna	hetloss	BASIS
PD3890	KTN1	cna	hetloss	BASIS
PD3905	KTN1	cna	gain	BASIS
PD4005	KTN1	cna	hetloss	BASIS
PD4006	KTN1	cna	hetloss	BASIS
PD4107	KTN1	cna	hetloss	BASIS
PD5930	KTN1	cna	hetloss	BASIS
PD5945	KTN1	cna	gain	BASIS
PD5948	KTN1	cna	hetloss	BASIS
PD6406	KTN1	cna	hetloss	BASIS
PD7067	KTN1	cna	gain	BASIS
PD7215	KTN1	cna	gain	BASIS
PD8621	KTN1	cna	hetloss	BASIS
PD9004	KTN1	cna	gain	BASIS
PD9585	KTN1	cna	gain	BASIS
PD9702	KTN1	cna	gain	BASIS
TCGA-A2-A25B	LPP	cna	amp	TCGA
TCGA-AN-A0XU	LPP	cna	gain	TCGA
TCGA-BH-A0AW	LPP	cna	gain	TCGA
TCGA-BH-A0C0	LPP	cna	gain	TCGA
TCGA-BH-A1FU	LPP	cna	hetloss	TCGA
TCGA-C8-A12L	LPP	cna	gain	TCGA
TCGA-LL-A5YP	LPP	cna	gain	TCGA
MB-0346	LPP	cna	gain	METABRIC
MB-5070	LPP	cna	hetloss	METABRIC
MB-5107	LPP	cna	amp	METABRIC
MB-5465	LPP	cna	gain	METABRIC
MB-6060	LPP	cna	gain	METABRIC
MB-6098	LPP	cna	gain	METABRIC
MB-7038	LPP	cna	hetloss	METABRIC
PD11327	LPP	cna	gain	BASIS
PD13299	LPP	cna	gain	BASIS
PD22355	LPP	cna	gain	BASIS
PD23561	LPP	cna	hetloss	BASIS
PD23562	LPP	cna	gain	BASIS
PD23578	LPP	cna	gain	BASIS
PD24186	LPP	cna	amp	BASIS
PD24202	LPP	cna	gain	BASIS
PD3905	LPP	cna	gain	BASIS
PD4005	LPP	cna	gain	BASIS
PD4107	LPP	cna	gain	BASIS
PD4826	LPP	cna	gain	BASIS
PD5930	LPP	cna	gain	BASIS
PD5945	LPP	cna	amp	BASIS
PD5948	LPP	cna	gain	BASIS
PD6406	LPP	cna	gain	BASIS

PD6731	LPP	cna	gain	BASIS
PD7067	LPP	cna	gain	BASIS
PD7215	LPP	cna	gain	BASIS
PD8621	LPP	cna	gain	BASIS
PD8980	LPP	cna	gain	BASIS
PD9004	LPP	cna	gain	BASIS
PD9702	LPP	cna	amp	BASIS
TCGA-AN-A0XU	MAP2K2	cna	hetloss	TCGA
TCGA-AO-A0JL	MAP2K2	cna	gain	TCGA
TCGA-BH-A0C0	MAP2K2	cna	gain	TCGA
TCGA-BH-A1FU	MAP2K2	cna	hetloss	TCGA
TCGA-C8-A12L	MAP2K2	cna	amp	TCGA
TCGA-D8-A27M	MAP2K2	cna	hetloss	TCGA
TCGA-E9-A1NC	MAP2K2	cna	hetloss	TCGA
TCGA-EW-A1OX	MAP2K2	cna	hetloss	TCGA
TCGA-LL-A5YP	MAP2K2	cna	hetloss	TCGA
MB-0346	MAP2K2	cna	gain	METABRIC
MB-2827	MAP2K2	cna	hetloss	METABRIC
MB-5070	MAP2K2	cna	hetloss	METABRIC
MB-5465	MAP2K2	cna	gain	METABRIC
MB-6060	MAP2K2	cna	gain	METABRIC
MB-6098	MAP2K2	cna	hetloss	METABRIC
MB-7038	MAP2K2	cna	hetloss	METABRIC
MB-0420	MAP2K2	cna	hetloss	METABRIC
PD10014	MAP2K2	cna	hetloss	BASIS
PD11742	MAP2K2	cna	hetloss	BASIS
PD14442	MAP2K2	cna	hetloss	BASIS
PD22355	MAP2K2	cna	hetloss	BASIS
PD23574	MAP2K2	cna	gain	BASIS
PD23578	MAP2K2	cna	hetloss	BASIS
PD24202	MAP2K2	cna	gain	BASIS
PD24206	MAP2K2	cna	hetloss	BASIS
PD3905	MAP2K2	cna	gain	BASIS
PD4006	MAP2K2	cna	gain	BASIS
PD4826	MAP2K2	cna	gain	BASIS
PD4967	MAP2K2	cna	hetloss	BASIS
PD5930	MAP2K2	cna	gain	BASIS
PD5945	MAP2K2	cna	gain	BASIS
PD5948	MAP2K2	cna	gain	BASIS
PD7067	MAP2K2	cna	gain	BASIS
PD8621	MAP2K2	cna	gain	BASIS
PD8980	MAP2K2	cna	gain	BASIS
PD9004	MAP2K2	cna	hetloss	BASIS
PD9702	MAP2K2	cna	gain	BASIS
TCGA-A2-A25B	MAP2K4	cna	hetloss	TCGA
TCGA-AN-A0XU	MAP2K4	cna	hetloss	TCGA
TCGA-AO-A0JL	MAP2K4	cna	gain	TCGA

TCGA-BH-A0AW	MAP2K4	cna	hetloss	TCGA
TCGA-BH-A0C0	MAP2K4	cna	hetloss	TCGA
TCGA-C8-A12L	MAP2K4	cna	hetloss	TCGA
TCGA-D8-A27M	MAP2K4	cna	hetloss	TCGA
TCGA-E2-A1L7	MAP2K4	cna	hetloss	TCGA
TCGA-E9-A1NC	MAP2K4	cna	hetloss	TCGA
TCGA-LL-A5YP	MAP2K4	cna	gain	TCGA
MB-0346	MAP2K4	cna	hetloss	METABRIC
MB-2827	MAP2K4	cna	hetloss	METABRIC
MB-5070	MAP2K4	cna	hetloss	METABRIC
MB-5107	MAP2K4	cna	hetloss	METABRIC
MB-6098	MAP2K4	cna	hetloss	METABRIC
MB-6271	MAP2K4	cna	hetloss	METABRIC
MB-7048	MAP2K4	cna	hetloss	METABRIC
MB-0420	MAP2K4	cna	hetloss	METABRIC
PD11742	MAP2K4	cna	hetloss	BASIS
PD13296	MAP2K4	cna	hetloss	BASIS
PD13771	MAP2K4	cna	hetloss	BASIS
PD22355	MAP2K4	cna	hetloss	BASIS
PD23561	MAP2K4	cna	hetloss	BASIS
PD24202	MAP2K4	cna	hetloss	BASIS
PD24206	MAP2K4	cna	homdel	BASIS
PD24337	MAP2K4	cna	hetloss	BASIS
PD3890	MAP2K4	cna	hetloss	BASIS
PD3905	MAP2K4	cna	gain	BASIS
PD4005	MAP2K4	cna	homdel	BASIS
PD4826	MAP2K4	cna	hetloss	BASIS
PD4967	MAP2K4	cna	hetloss	BASIS
PD5945	MAP2K4	cna	gain	BASIS
PD6406	MAP2K4	cna	hetloss	BASIS
PD6413	MAP2K4	cna	hetloss	BASIS
PD6731	MAP2K4	cna	hetloss	BASIS
PD7067	MAP2K4	cna	gain	BASIS
PD9585	MAP2K4	cna	hetloss	BASIS
TCGA-A2-A25B	MET	cna	gain	TCGA
TCGA-AO-A0JL	MET	cna	gain	TCGA
TCGA-BH-A0AW	MET	cna	gain	TCGA
TCGA-C8-A12L	MET	cna	hetloss	TCGA
TCGA-D8-A27M	MET	cna	gain	TCGA
TCGA-E2-A1L7	MET	cna	hetloss	TCGA
TCGA-E9-A1NC	MET	cna	gain	TCGA
TCGA-LL-A5YP	MET	cna	gain	TCGA
MB-0346	MET	cna	hetloss	METABRIC
MB-5465	MET	cna	gain	METABRIC
MB-7038	MET	cna	gain	METABRIC
MB-7048	MET	cna	gain	METABRIC
PD11327	MET	cna	gain	BASIS

PD13296	MET	cna	hetloss	BASIS
PD13297	MET	cna	gain	BASIS
PD13299	MET	cna	gain	BASIS
PD22355	MET	cna	hetloss	BASIS
PD23574	MET	cna	gain	BASIS
PD23578	MET	cna	gain	BASIS
PD24186	MET	cna	amp	BASIS
PD24337	MET	cna	hetloss	BASIS
PD3890	MET	cna	hetloss	BASIS
PD3905	MET	cna	gain	BASIS
PD4005	MET	cna	hetloss	BASIS
PD4107	MET	cna	gain	BASIS
PD4826	MET	cna	gain	BASIS
PD5930	MET	cna	gain	BASIS
PD5935	MET	cna	gain	BASIS
PD5945	MET	cna	amp	BASIS
PD5948	MET	cna	gain	BASIS
PD6406	MET	cna	gain	BASIS
PD7067	MET	cna	gain	BASIS
PD7215	MET	cna	gain	BASIS
PD8621	MET	cna	gain	BASIS
PD9004	MET	cna	gain	BASIS
PD9585	MET	cna	hetloss	BASIS
PD9702	MET	cna	amp	BASIS
TCGA-A2-A25B	MYCN	cna	hetloss	TCGA
TCGA-AN-A0XU	MYCN	cna	hetloss	TCGA
TCGA-BH-A0AW	MYCN	cna	gain	TCGA
TCGA-BH-A0C0	MYCN	cna	gain	TCGA
TCGA-C8-A12L	MYCN	cna	gain	TCGA
TCGA-D8-A27M	MYCN	cna	hetloss	TCGA
TCGA-E9-A1NC	MYCN	cna	hetloss	TCGA
TCGA-LL-A5YP	MYCN	cna	gain	TCGA
MB-0346	MYCN	cna	amp	METABRIC
MB-5070	MYCN	cna	gain	METABRIC
MB-5465	MYCN	cna	amp	METABRIC
MB-6098	MYCN	cna	hetloss	METABRIC
MB-6271	MYCN	cna	hetloss	METABRIC
MB-7038	MYCN	cna	gain	METABRIC
MB-0420	MYCN	cna	hetloss	METABRIC
MTS-T0064	MYCN	cna	hetloss	METABRIC
PD10014	MYCN	cna	gain	BASIS
PD11327	MYCN	cna	gain	BASIS
PD13297	MYCN	cna	hetloss	BASIS
PD13299	MYCN	cna	gain	BASIS
PD13771	MYCN	cna	hetloss	BASIS
PD22355	MYCN	cna	gain	BASIS
PD24186	MYCN	cna	amp	BASIS

PD24206	MYCN	cna	gain	BASIS
PD3890	MYCN	cna	hetloss	BASIS
PD3905	MYCN	cna	gain	BASIS
PD4005	MYCN	cna	hetloss	BASIS
PD4006	MYCN	cna	hetloss	BASIS
PD4826	MYCN	cna	hetloss	BASIS
PD5930	MYCN	cna	gain	BASIS
PD5935	MYCN	cna	gain	BASIS
PD5945	MYCN	cna	gain	BASIS
PD5948	MYCN	cna	gain	BASIS
PD6406	MYCN	cna	hetloss	BASIS
PD7067	MYCN	cna	gain	BASIS
PD9004	MYCN	cna	gain	BASIS
PD9702	MYCN	cna	gain	BASIS
TCGA-A2-A25B	NUMA1	cna	hetloss	TCGA
TCGA-AN-A0XU	NUMA1	cna	hetloss	TCGA
TCGA-AO-A0JL	NUMA1	cna	hetloss	TCGA
TCGA-BH-A18R	NUMA1	cna	homdel	TCGA
TCGA-C8-A12L	NUMA1	cna	amp	TCGA
TCGA-D8-A27M	NUMA1	cna	gain	TCGA
TCGA-EW-A1OX	NUMA1	cna	amp	TCGA
TCGA-LL-A5YP	NUMA1	cna	hetloss	TCGA
MB-0346	NUMA1	cna	gain	METABRIC
MB-5107	NUMA1	cna	gain	METABRIC
MB-6060	NUMA1	cna	gain	METABRIC
MB-6271	NUMA1	cna	hetloss	METABRIC
MB-7032	NUMA1	cna	hetloss	METABRIC
MTS-T0064	NUMA1	cna	gain	METABRIC
PD10014	NUMA1	cna	gain	BASIS
PD11327	NUMA1	cna	gain	BASIS
PD11742	NUMA1	cna	gain	BASIS
PD22355	NUMA1	cna	hetloss	BASIS
PD23574	NUMA1	cna	gain	BASIS
PD24186	NUMA1	cna	amp	BASIS
PD24206	NUMA1	cna	gain	BASIS
PD3905	NUMA1	cna	gain	BASIS
PD4005	NUMA1	cna	gain	BASIS
PD4006	NUMA1	cna	amp	BASIS
PD4107	NUMA1	cna	gain	BASIS
PD4826	NUMA1	cna	gain	BASIS
PD5930	NUMA1	cna	gain	BASIS
PD5935	NUMA1	cna	gain	BASIS
PD5945	NUMA1	cna	amp	BASIS
PD5948	NUMA1	cna	gain	BASIS
PD6413	NUMA1	cna	gain	BASIS
PD7067	NUMA1	cna	gain	BASIS
PD7215	NUMA1	cna	gain	BASIS

PD8980	NUMA1	cna	gain	BASIS
PD9004	NUMA1	cna	gain	BASIS
PD9585	NUMA1	cna	hetloss	BASIS
PD9702	NUMA1	cna	gain	BASIS
TCGA-AO-A0JL	NUTM1	cna	hetloss	TCGA
TCGA-BH-A0AW	NUTM1	cna	gain	TCGA
TCGA-BH-A0C0	NUTM1	cna	hetloss	TCGA
TCGA-C8-A12L	NUTM1	cna	hetloss	TCGA
TCGA-D8-A27M	NUTM1	cna	hetloss	TCGA
TCGA-E2-A1L7	NUTM1	cna	hetloss	TCGA
TCGA-E9-A1NC	NUTM1	cna	hetloss	TCGA
TCGA-LL-A5YP	NUTM1	cna	hetloss	TCGA
MB-2827	NUTM1	cna	hetloss	METABRIC
MB-5070	NUTM1	cna	hetloss	METABRIC
MB-5465	NUTM1	cna	hetloss	METABRIC
MB-6060	NUTM1	cna	hetloss	METABRIC
MB-6098	NUTM1	cna	hetloss	METABRIC
MB-7038	NUTM1	cna	hetloss	METABRIC
MB-0420	NUTM1	cna	hetloss	METABRIC
PD10014	NUTM1	cna	hetloss	BASIS
PD11327	NUTM1	cna	hetloss	BASIS
PD11742	NUTM1	cna	hetloss	BASIS
PD13297	NUTM1	cna	hetloss	BASIS
PD13299	NUTM1	cna	hetloss	BASIS
PD23574	NUTM1	cna	hetloss	BASIS
PD23578	NUTM1	cna	hetloss	BASIS
PD24202	NUTM1	cna	hetloss	BASIS
PD24206	NUTM1	cna	gain	BASIS
PD3890	NUTM1	cna	hetloss	BASIS
PD3905	NUTM1	cna	gain	BASIS
PD4005	NUTM1	cna	homdel	BASIS
PD4107	NUTM1	cna	hetloss	BASIS
PD5930	NUTM1	cna	hetloss	BASIS
PD5935	NUTM1	cna	gain	BASIS
PD5945	NUTM1	cna	amp	BASIS
PD6413	NUTM1	cna	hetloss	BASIS
PD7067	NUTM1	cna	gain	BASIS
PD7215	NUTM1	cna	gain	BASIS
PD9004	NUTM1	cna	hetloss	BASIS
PD9585	NUTM1	cna	hetloss	BASIS
PD9702	NUTM1	cna	gain	BASIS
TCGA-A2-A25B	NUTM2A	cna	hetloss	TCGA
TCGA-AN-A0XU	NUTM2A	cna	hetloss	TCGA
TCGA-AO-A0JL	NUTM2A	cna	hetloss	TCGA
TCGA-BH-A0AW	NUTM2A	cna	gain	TCGA
TCGA-BH-A18R	NUTM2A	cna	gain	TCGA
TCGA-C8-A12L	NUTM2A	cna	hetloss	TCGA

TCGA-D8-A27M	NUTM2A	cna	hetloss	TCGA
TCGA-E2-A1L7	NUTM2A	cna	hetloss	TCGA
MB-2827	NUTM2A	cna	hetloss	METABRIC
MB-5070	NUTM2A	cna	hetloss	METABRIC
MB-6060	NUTM2A	cna	hetloss	METABRIC
MB-6098	NUTM2A	cna	hetloss	METABRIC
MB-7048	NUTM2A	cna	hetloss	METABRIC
MTS-T0064	NUTM2A	cna	gain	METABRIC
PD10014	NUTM2A	cna	hetloss	BASIS
PD11327	NUTM2A	cna	hetloss	BASIS
PD11742	NUTM2A	cna	hetloss	BASIS
PD13296	NUTM2A	cna	hetloss	BASIS
PD13299	NUTM2A	cna	gain	BASIS
PD23562	NUTM2A	cna	gain	BASIS
PD23574	NUTM2A	cna	gain	BASIS
PD23578	NUTM2A	cna	hetloss	BASIS
PD24202	NUTM2A	cna	hetloss	BASIS
PD24206	NUTM2A	cna	gain	BASIS
PD3890	NUTM2A	cna	hetloss	BASIS
PD3905	NUTM2A	cna	gain	BASIS
PD4006	NUTM2A	cna	gain	BASIS
PD4107	NUTM2A	cna	gain	BASIS
PD5935	NUTM2A	cna	homdel	BASIS
PD6406	NUTM2A	cna	hetloss	BASIS
PD6731	NUTM2A	cna	hetloss	BASIS
PD7215	NUTM2A	cna	gain	BASIS
PD8621	NUTM2A	cna	hetloss	BASIS
PD8980	NUTM2A	cna	hetloss	BASIS
PD9004	NUTM2A	cna	gain	BASIS
PD9585	NUTM2A	cna	hetloss	BASIS
PD9702	NUTM2A	cna	gain	BASIS
TCGA-A2-A25B	PAK3	cna	hetloss	TCGA
TCGA-AN-A0XU	PAK3	cna	hetloss	TCGA
TCGA-BH-A0AW	PAK3	cna	gain	TCGA
TCGA-C8-A12L	PAK3	cna	hetloss	TCGA
TCGA-E2-A1L7	PAK3	cna	hetloss	TCGA
TCGA-E9-A1NC	PAK3	cna	hetloss	TCGA
MB-0346	PAK3	cna	hetloss	METABRIC
MB-2827	PAK3	cna	hetloss	METABRIC
MB-5465	PAK3	cna	gain	METABRIC
MB-6098	PAK3	cna	hetloss	METABRIC
PD10014	PAK3	cna	hetloss	BASIS
PD13296	PAK3	cna	hetloss	BASIS
PD13297	PAK3	cna	gain	BASIS
PD13299	PAK3	cna	amp	BASIS
PD13771	PAK3	cna	gain	BASIS
PD14442	PAK3	cna	gain	BASIS

PD22355	PAK3	cna	gain	BASIS
PD23562	PAK3	cna	hetloss	BASIS
PD23574	PAK3	cna	gain	BASIS
PD23578	PAK3	cna	hetloss	BASIS
PD24202	PAK3	cna	hetloss	BASIS
PD24206	PAK3	cna	gain	BASIS
PD3905	PAK3	cna	gain	BASIS
PD4005	PAK3	cna	gain	BASIS
PD4006	PAK3	cna	gain	BASIS
PD4107	PAK3	cna	gain	BASIS
PD4826	PAK3	cna	gain	BASIS
PD4967	PAK3	cna	gain	BASIS
PD5930	PAK3	cna	gain	BASIS
PD5945	PAK3	cna	gain	BASIS
PD6406	PAK3	cna	gain	BASIS
PD6413	PAK3	cna	gain	BASIS
PD7067	PAK3	cna	gain	BASIS
PD7215	PAK3	cna	amp	BASIS
PD8621	PAK3	cna	amp	BASIS
PD8980	PAK3	cna	hetloss	BASIS
PD9004	PAK3	cna	gain	BASIS
TCGA-AN-A0XU	PARP4	cna	hetloss	TCGA
TCGA-AO-A0JL	PARP4	cna	hetloss	TCGA
TCGA-BH-A0AW	PARP4	cna	hetloss	TCGA
TCGA-BH-A0C0	PARP4	cna	gain	TCGA
TCGA-BH-A1FU	PARP4	cna	hetloss	TCGA
TCGA-C8-A12L	PARP4	cna	hetloss	TCGA
TCGA-D8-A27M	PARP4	cna	gain	TCGA
TCGA-E9-A1NC	PARP4	cna	hetloss	TCGA
TCGA-EW-A1OX	PARP4	cna	homdel	TCGA
TCGA-LL-A5YP	PARP4	cna	hetloss	TCGA
MB-0346	PARP4	cna	hetloss	METABRIC
MB-2827	PARP4	cna	hetloss	METABRIC
MB-6098	PARP4	cna	hetloss	METABRIC
MB-7032	PARP4	cna	gain	METABRIC
PD10014	PARP4	cna	hetloss	BASIS
PD13296	PARP4	cna	hetloss	BASIS
PD13297	PARP4	cna	hetloss	BASIS
PD13771	PARP4	cna	hetloss	BASIS
PD14442	PARP4	cna	hetloss	BASIS
PD22355	PARP4	cna	hetloss	BASIS
PD24202	PARP4	cna	hetloss	BASIS
PD24206	PARP4	cna	gain	BASIS
PD24337	PARP4	cna	hetloss	BASIS
PD3890	PARP4	cna	hetloss	BASIS
PD3905	PARP4	cna	gain	BASIS
PD4005	PARP4	cna	hetloss	BASIS

PD4006	PARP4	cna	gain	BASIS
PD4107	PARP4	cna	gain	BASIS
PD4826	PARP4	cna	hetloss	BASIS
PD4967	PARP4	cna	hetloss	BASIS
PD5945	PARP4	cna	amp	BASIS
PD6406	PARP4	cna	hetloss	BASIS
PD6413	PARP4	cna	hetloss	BASIS
PD6731	PARP4	cna	hetloss	BASIS
PD7067	PARP4	cna	gain	BASIS
PD8980	PARP4	cna	hetloss	BASIS
PD9702	PARP4	cna	gain	BASIS
TCGA-A2-A25B	PAX7	cna	hetloss	TCGA
TCGA-AN-A0XU	PAX7	cna	hetloss	TCGA
TCGA-AO-A0JL	PAX7	cna	hetloss	TCGA
TCGA-BH-A0AW	PAX7	cna	gain	TCGA
TCGA-BH-A0C0	PAX7	cna	hetloss	TCGA
TCGA-BH-A18R	PAX7	cna	homdel	TCGA
TCGA-BH-A1FU	PAX7	cna	gain	TCGA
TCGA-C8-A12L	PAX7	cna	hetloss	TCGA
TCGA-D8-A27M	PAX7	cna	gain	TCGA
TCGA-E2-A1L7	PAX7	cna	hetloss	TCGA
TCGA-E9-A1NC	PAX7	cna	hetloss	TCGA
TCGA-LL-A5YP	PAX7	cna	hetloss	TCGA
MB-2827	PAX7	cna	hetloss	METABRIC
MB-5070	PAX7	cna	hetloss	METABRIC
MB-5465	PAX7	cna	hetloss	METABRIC
MB-6060	PAX7	cna	hetloss	METABRIC
MB-6098	PAX7	cna	hetloss	METABRIC
MB-6271	PAX7	cna	hetloss	METABRIC
MB-0420	PAX7	cna	hetloss	METABRIC
PD10014	PAX7	cna	gain	BASIS
PD11742	PAX7	cna	hetloss	BASIS
PD13296	PAX7	cna	hetloss	BASIS
PD13771	PAX7	cna	hetloss	BASIS
PD22355	PAX7	cna	hetloss	BASIS
PD24202	PAX7	cna	hetloss	BASIS
PD24206	PAX7	cna	gain	BASIS
PD24337	PAX7	cna	hetloss	BASIS
PD3890	PAX7	cna	hetloss	BASIS
PD3905	PAX7	cna	gain	BASIS
PD4826	PAX7	cna	gain	BASIS
PD5945	PAX7	cna	gain	BASIS
PD6406	PAX7	cna	hetloss	BASIS
PD6413	PAX7	cna	hetloss	BASIS
PD7067	PAX7	cna	gain	BASIS
PD7215	PAX7	cna	gain	BASIS
PD9004	PAX7	cna	hetloss	BASIS

PD9585	PAX7	cna	hetloss	BASIS
TCGA-A2-A25B	PDCD1LG2	cna	hetloss	TCGA
TCGA-AO-A0JL	PDCD1LG2	cna	gain	TCGA
TCGA-BH-A0AW	PDCD1LG2	cna	hetloss	TCGA
TCGA-BH-A0C0	PDCD1LG2	cna	hetloss	TCGA
TCGA-BH-A1FU	PDCD1LG2	cna	hetloss	TCGA
TCGA-C8-A12L	PDCD1LG2	cna	hetloss	TCGA
TCGA-E2-A1L7	PDCD1LG2	cna	hetloss	TCGA
MB-0346	PDCD1LG2	cna	gain	METABRIC
MB-2827	PDCD1LG2	cna	hetloss	METABRIC
MB-6060	PDCD1LG2	cna	gain	METABRIC
MB-0420	PDCD1LG2	cna	hetloss	METABRIC
MTS-T0064	PDCD1LG2	cna	amp	METABRIC
PD11327	PDCD1LG2	cna	homdel	BASIS
PD13296	PDCD1LG2	cna	gain	BASIS
PD13297	PDCD1LG2	cna	hetloss	BASIS
PD13299	PDCD1LG2	cna	amp	BASIS
PD22355	PDCD1LG2	cna	gain	BASIS
PD23574	PDCD1LG2	cna	amp	BASIS
PD24186	PDCD1LG2	cna	gain	BASIS
PD24337	PDCD1LG2	cna	gain	BASIS
PD3905	PDCD1LG2	cna	gain	BASIS
PD4006	PDCD1LG2	cna	gain	BASIS
PD4107	PDCD1LG2	cna	gain	BASIS
PD4826	PDCD1LG2	cna	gain	BASIS
PD4967	PDCD1LG2	cna	hetloss	BASIS
PD5945	PDCD1LG2	cna	gain	BASIS
PD5948	PDCD1LG2	cna	hetloss	BASIS
PD6406	PDCD1LG2	cna	homdel	BASIS
PD6413	PDCD1LG2	cna	gain	BASIS
PD6731	PDCD1LG2	cna	gain	BASIS
PD7067	PDCD1LG2	cna	amp	BASIS
PD7215	PDCD1LG2	cna	hetloss	BASIS
PD8621	PDCD1LG2	cna	gain	BASIS
PD8980	PDCD1LG2	cna	gain	BASIS
PD9004	PDCD1LG2	cna	gain	BASIS
PD9585	PDCD1LG2	cna	hetloss	BASIS
PD9702	PDCD1LG2	cna	gain	BASIS
TCGA-AN-A0XU	PDGFRB	cna	hetloss	TCGA
TCGA-AO-A0JL	PDGFRB	cna	hetloss	TCGA
TCGA-BH-A0C0	PDGFRB	cna	hetloss	TCGA
TCGA-BH-A18R	PDGFRB	cna	gain	TCGA
TCGA-BH-A1FU	PDGFRB	cna	hetloss	TCGA
TCGA-C8-A12L	PDGFRB	cna	hetloss	TCGA
TCGA-D8-A27M	PDGFRB	cna	hetloss	TCGA
TCGA-E2-A1L7	PDGFRB	cna	hetloss	TCGA
TCGA-E9-A1NC	PDGFRB	cna	hetloss	TCGA

TCGA-LL-A5YP	PDGFRB	cna	hetloss	TCGA
MB-0346	PDGFRB	cna	gain	METABRIC
MB-5070	PDGFRB	cna	hetloss	METABRIC
MB-5465	PDGFRB	cna	hetloss	METABRIC
MB-6098	PDGFRB	cna	hetloss	METABRIC
MB-7038	PDGFRB	cna	hetloss	METABRIC
MB-0420	PDGFRB	cna	hetloss	METABRIC
PD10014	PDGFRB	cna	hetloss	BASIS
PD13296	PDGFRB	cna	hetloss	BASIS
PD13297	PDGFRB	cna	hetloss	BASIS
PD13771	PDGFRB	cna	gain	BASIS
PD22355	PDGFRB	cna	hetloss	BASIS
PD23578	PDGFRB	cna	hetloss	BASIS
PD24186	PDGFRB	cna	gain	BASIS
PD24202	PDGFRB	cna	hetloss	BASIS
PD24206	PDGFRB	cna	gain	BASIS
PD24337	PDGFRB	cna	hetloss	BASIS
PD3890	PDGFRB	cna	hetloss	BASIS
PD3905	PDGFRB	cna	gain	BASIS
PD4005	PDGFRB	cna	hetloss	BASIS
PD4826	PDGFRB	cna	gain	BASIS
PD5935	PDGFRB	cna	gain	BASIS
PD5945	PDGFRB	cna	gain	BASIS
PD6406	PDGFRB	cna	hetloss	BASIS
PD6413	PDGFRB	cna	hetloss	BASIS
PD7067	PDGFRB	cna	gain	BASIS
PD8980	PDGFRB	cna	hetloss	BASIS
PD9585	PDGFRB	cna	hetloss	BASIS
TCGA-AN-A0XU	PHOX2B	cna	hetloss	TCGA
TCGA-AO-A0JL	PHOX2B	cna	homdel	TCGA
TCGA-BH-A0AW	PHOX2B	cna	hetloss	TCGA
TCGA-BH-A0C0	PHOX2B	cna	gain	TCGA
TCGA-BH-A1FU	PHOX2B	cna	homdel	TCGA
TCGA-C8-A12L	PHOX2B	cna	hetloss	TCGA
TCGA-D8-A27M	PHOX2B	cna	gain	TCGA
TCGA-E9-A1NC	PHOX2B	cna	hetloss	TCGA
TCGA-LL-A5YP	PHOX2B	cna	hetloss	TCGA
MB-0346	PHOX2B	cna	hetloss	METABRIC
MB-2827	PHOX2B	cna	hetloss	METABRIC
MB-5465	PHOX2B	cna	hetloss	METABRIC
MB-6060	PHOX2B	cna	hetloss	METABRIC
MB-6098	PHOX2B	cna	hetloss	METABRIC
MB-7038	PHOX2B	cna	gain	METABRIC
MB-0420	PHOX2B	cna	hetloss	METABRIC
P-0010758-T01-IM5	PHOX2B	cna	homdel	MSK-IMPACT
PD11327	PHOX2B	cna	hetloss	BASIS
PD11742	PHOX2B	cna	hetloss	BASIS

PD13296	PHOX2B	cna	hetloss	BASIS
PD13297	PHOX2B	cna	hetloss	BASIS
PD13299	PHOX2B	cna	hetloss	BASIS
PD22355	PHOX2B	cna	hetloss	BASIS
PD24186	PHOX2B	cna	gain	BASIS
PD24202	PHOX2B	cna	hetloss	BASIS
PD24206	PHOX2B	cna	gain	BASIS
PD24337	PHOX2B	cna	hetloss	BASIS
PD4005	PHOX2B	cna	hetloss	BASIS
PD4006	PHOX2B	cna	hetloss	BASIS
PD4107	PHOX2B	cna	hetloss	BASIS
PD5930	PHOX2B	cna	hetloss	BASIS
PD5945	PHOX2B	cna	gain	BASIS
PD6413	PHOX2B	cna	hetloss	BASIS
PD7067	PHOX2B	cna	gain	BASIS
PD8621	PHOX2B	cna	gain	BASIS
PD8980	PHOX2B	cna	hetloss	BASIS
PD9702	PHOX2B	cna	gain	BASIS
TCGA-A2-A25B	PICALM	cna	hetloss	TCGA
TCGA-AN-A0XU	PICALM	cna	gain	TCGA
TCGA-AO-A0JL	PICALM	cna	hetloss	TCGA
TCGA-C8-A12L	PICALM	cna	gain	TCGA
TCGA-D8-A27M	PICALM	cna	gain	TCGA
TCGA-EW-A1OX	PICALM	cna	homdel	TCGA
TCGA-LL-A5YP	PICALM	cna	hetloss	TCGA
MB-0346	PICALM	cna	gain	METABRIC
MB-5070	PICALM	cna	hetloss	METABRIC
MB-5107	PICALM	cna	amp	METABRIC
MB-6271	PICALM	cna	hetloss	METABRIC
MB-7032	PICALM	cna	hetloss	METABRIC
MTS-T0064	PICALM	cna	gain	METABRIC
PD10014	PICALM	cna	gain	BASIS
PD11327	PICALM	cna	gain	BASIS
PD11742	PICALM	cna	hetloss	BASIS
PD13771	PICALM	cna	hetloss	BASIS
PD22355	PICALM	cna	hetloss	BASIS
PD23574	PICALM	cna	gain	BASIS
PD23578	PICALM	cna	hetloss	BASIS
PD24186	PICALM	cna	gain	BASIS
PD24206	PICALM	cna	gain	BASIS
PD24337	PICALM	cna	hetloss	BASIS
PD3905	PICALM	cna	gain	BASIS
PD4005	PICALM	cna	gain	BASIS
PD4006	PICALM	cna	hetloss	BASIS
PD4826	PICALM	cna	gain	BASIS
PD5930	PICALM	cna	gain	BASIS
PD5935	PICALM	cna	gain	BASIS

PD5945	PICALM	cna	gain	BASIS
PD5948	PICALM	cna	gain	BASIS
PD6413	PICALM	cna	hetloss	BASIS
PD7215	PICALM	cna	gain	BASIS
PD8980	PICALM	cna	gain	BASIS
PD9004	PICALM	cna	gain	BASIS
PD9585	PICALM	cna	hetloss	BASIS
PD9702	PICALM	cna	gain	BASIS
TCGA-A2-A25B	PLAG1	cna	gain	TCGA
TCGA-AO-A0JL	PLAG1	cna	amp	TCGA
TCGA-BH-A0AW	PLAG1	cna	gain	TCGA
TCGA-BH-A0C0	PLAG1	cna	gain	TCGA
TCGA-BH-A18R	PLAG1	cna	homdel	TCGA
TCGA-BH-A1FU	PLAG1	cna	gain	TCGA
TCGA-D8-A27M	PLAG1	cna	amp	TCGA
TCGA-E9-A1NC	PLAG1	cna	amp	TCGA
TCGA-LL-A5YP	PLAG1	cna	gain	TCGA
MB-0346	PLAG1	cna	gain	METABRIC
MB-5465	PLAG1	cna	hetloss	METABRIC
MB-6060	PLAG1	cna	amp	METABRIC
MB-6098	PLAG1	cna	gain	METABRIC
MB-7038	PLAG1	cna	gain	METABRIC
PD10014	PLAG1	cna	amp	BASIS
PD11327	PLAG1	cna	hetloss	BASIS
PD13296	PLAG1	cna	homdel	BASIS
PD13299	PLAG1	cna	gain	BASIS
PD23562	PLAG1	cna	gain	BASIS
PD23574	PLAG1	cna	gain	BASIS
PD23578	PLAG1	cna	hetloss	BASIS
PD24186	PLAG1	cna	gain	BASIS
PD24202	PLAG1	cna	hetloss	BASIS
PD3905	PLAG1	cna	gain	BASIS
PD4005	PLAG1	cna	hetloss	BASIS
PD4006	PLAG1	cna	gain	BASIS
PD4107	PLAG1	cna	gain	BASIS
PD4826	PLAG1	cna	amp	BASIS
PD5935	PLAG1	cna	gain	BASIS
PD5945	PLAG1	cna	gain	BASIS
PD7067	PLAG1	cna	amp	BASIS
PD7215	PLAG1	cna	amp	BASIS
PD8621	PLAG1	cna	gain	BASIS
PD8980	PLAG1	cna	gain	BASIS
PD9004	PLAG1	cna	gain	BASIS
PD9585	PLAG1	cna	hetloss	BASIS
PD9702	PLAG1	cna	gain	BASIS
TCGA-A2-A25B	PRDM2	cna	hetloss	TCGA
TCGA-AN-A0XU	PRDM2	cna	hetloss	TCGA

TCGA-AO-A0JL	PRDM2	cna	hetloss	TCGA
TCGA-BH-A0AW	PRDM2	cna	gain	TCGA
TCGA-BH-A0C0	PRDM2	cna	hetloss	TCGA
TCGA-BH-A18R	PRDM2	cna	homdel	TCGA
TCGA-BH-A1FU	PRDM2	cna	gain	TCGA
TCGA-C8-A12L	PRDM2	cna	hetloss	TCGA
TCGA-D8-A27M	PRDM2	cna	gain	TCGA
TCGA-E2-A1L7	PRDM2	cna	hetloss	TCGA
TCGA-E9-A1NC	PRDM2	cna	hetloss	TCGA
TCGA-LL-A5YP	PRDM2	cna	hetloss	TCGA
MB-2827	PRDM2	cna	hetloss	METABRIC
MB-5070	PRDM2	cna	hetloss	METABRIC
MB-5465	PRDM2	cna	hetloss	METABRIC
MB-6060	PRDM2	cna	hetloss	METABRIC
MB-6098	PRDM2	cna	hetloss	METABRIC
MB-0420	PRDM2	cna	hetloss	METABRIC
PD10014	PRDM2	cna	gain	BASIS
PD11327	PRDM2	cna	gain	BASIS
PD11742	PRDM2	cna	hetloss	BASIS
PD13296	PRDM2	cna	hetloss	BASIS
PD13771	PRDM2	cna	hetloss	BASIS
PD22355	PRDM2	cna	hetloss	BASIS
PD24202	PRDM2	cna	hetloss	BASIS
PD24206	PRDM2	cna	gain	BASIS
PD24337	PRDM2	cna	hetloss	BASIS
PD3890	PRDM2	cna	hetloss	BASIS
PD3905	PRDM2	cna	gain	BASIS
PD4107	PRDM2	cna	hetloss	BASIS
PD4826	PRDM2	cna	gain	BASIS
PD5945	PRDM2	cna	gain	BASIS
PD6406	PRDM2	cna	hetloss	BASIS
PD7067	PRDM2	cna	gain	BASIS
PD7215	PRDM2	cna	gain	BASIS
PD8980	PRDM2	cna	hetloss	BASIS
PD9004	PRDM2	cna	hetloss	BASIS
TCGA-A2-A25B	PTEN	cna	hetloss	TCGA
TCGA-AN-A0XU	PTEN	cna	homdel	TCGA
TCGA-AO-A0JL	PTEN	cna	hetloss	TCGA
TCGA-BH-A0AW	PTEN	cna	hetloss	TCGA
TCGA-BH-A18R	PTEN	cna	gain	TCGA
TCGA-C8-A12L	PTEN	cna	hetloss	TCGA
TCGA-D8-A27M	PTEN	cna	hetloss	TCGA
TCGA-E2-A1L7	PTEN	cna	hetloss	TCGA
MB-2827	PTEN	cna	hetloss	METABRIC
MB-5070	PTEN	cna	hetloss	METABRIC
MB-6060	PTEN	cna	hetloss	METABRIC
MB-6098	PTEN	cna	hetloss	METABRIC

MB-7038	PTEN	cna	hetloss	METABRIC
MB-7048	PTEN	cna	homdel	METABRIC
MTS-T0064	PTEN	cna	gain	METABRIC
P-0002237-T01-IM3	PTEN	cna	homdel	MSK-IMPACT
PD10014	PTEN	cna	hetloss	BASIS
PD11742	PTEN	cna	hetloss	BASIS
PD13296	PTEN	cna	hetloss	BASIS
PD13299	PTEN	cna	gain	BASIS
PD23562	PTEN	cna	homdel	BASIS
PD23574	PTEN	cna	gain	BASIS
PD23578	PTEN	cna	hetloss	BASIS
PD24202	PTEN	cna	hetloss	BASIS
PD3890	PTEN	cna	hetloss	BASIS
PD3905	PTEN	cna	gain	BASIS
PD4006	PTEN	cna	gain	BASIS
PD4107	PTEN	cna	gain	BASIS
PD5935	PTEN	cna	homdel	BASIS
PD5945	PTEN	cna	homdel	BASIS
PD6406	PTEN	cna	hetloss	BASIS
PD6413	PTEN	cna	hetloss	BASIS
PD6731	PTEN	cna	hetloss	BASIS
PD7215	PTEN	cna	gain	BASIS
PD8621	PTEN	cna	hetloss	BASIS
PD8980	PTEN	cna	hetloss	BASIS
PD9585	PTEN	cna	hetloss	BASIS
TCGA-AN-A0XU	RMI2	cna	gain	TCGA
TCGA-AO-A0JL	RMI2	cna	amp	TCGA
TCGA-BH-A0AW	RMI2	cna	gain	TCGA
TCGA-BH-A0C0	RMI2	cna	gain	TCGA
TCGA-C8-A12L	RMI2	cna	gain	TCGA
TCGA-E2-A1L7	RMI2	cna	hetloss	TCGA
TCGA-LL-A5YP	RMI2	cna	gain	TCGA
MB-0346	RMI2	cna	gain	METABRIC
MB-6060	RMI2	cna	gain	METABRIC
MB-6098	RMI2	cna	hetloss	METABRIC
MB-6271	RMI2	cna	gain	METABRIC
MB-7032	RMI2	cna	gain	METABRIC
MB-7038	RMI2	cna	hetloss	METABRIC
MB-0420	RMI2	cna	hetloss	METABRIC
PD10014	RMI2	cna	hetloss	BASIS
PD11327	RMI2	cna	hetloss	BASIS
PD11742	RMI2	cna	gain	BASIS
PD13296	RMI2	cna	gain	BASIS
PD13297	RMI2	cna	hetloss	BASIS
PD13299	RMI2	cna	gain	BASIS
PD22355	RMI2	cna	hetloss	BASIS
PD23578	RMI2	cna	hetloss	BASIS

PD24202	RMI2	cna	hetloss	BASIS
PD24206	RMI2	cna	gain	BASIS
PD24337	RMI2	cna	hetloss	BASIS
PD3905	RMI2	cna	gain	BASIS
PD4005	RMI2	cna	gain	BASIS
PD4107	RMI2	cna	gain	BASIS
PD4826	RMI2	cna	gain	BASIS
PD5935	RMI2	cna	gain	BASIS
PD5945	RMI2	cna	gain	BASIS
PD5948	RMI2	cna	gain	BASIS
PD7067	RMI2	cna	gain	BASIS
PD7215	RMI2	cna	amp	BASIS
PD9004	RMI2	cna	gain	BASIS
PD9585	RMI2	cna	hetloss	BASIS
PD9702	RMI2	cna	gain	BASIS
TCGA-A2-A25B	RPGR	cna	gain	TCGA
TCGA-AN-A0XU	RPGR	cna	hetloss	TCGA
TCGA-AO-A0JL	RPGR	cna	gain	TCGA
TCGA-BH-A0AW	RPGR	cna	gain	TCGA
TCGA-E2-A1L7	RPGR	cna	hetloss	TCGA
TCGA-E9-A1NC	RPGR	cna	hetloss	TCGA
MB-0346	RPGR	cna	hetloss	METABRIC
MB-2827	RPGR	cna	hetloss	METABRIC
MB-5070	RPGR	cna	hetloss	METABRIC
MB-5465	RPGR	cna	gain	METABRIC
MB-6098	RPGR	cna	hetloss	METABRIC
MB-0420	RPGR	cna	hetloss	METABRIC
MTS-T0064	RPGR	cna	hetloss	METABRIC
PD10014	RPGR	cna	hetloss	BASIS
PD13296	RPGR	cna	gain	BASIS
PD13299	RPGR	cna	gain	BASIS
PD13771	RPGR	cna	gain	BASIS
PD14442	RPGR	cna	gain	BASIS
PD23562	RPGR	cna	hetloss	BASIS
PD23574	RPGR	cna	gain	BASIS
PD23578	RPGR	cna	hetloss	BASIS
PD3905	RPGR	cna	gain	BASIS
PD4005	RPGR	cna	hetloss	BASIS
PD4006	RPGR	cna	hetloss	BASIS
PD4107	RPGR	cna	gain	BASIS
PD4826	RPGR	cna	gain	BASIS
PD4967	RPGR	cna	gain	BASIS
PD5935	RPGR	cna	gain	BASIS
PD5948	RPGR	cna	gain	BASIS
PD6406	RPGR	cna	gain	BASIS
PD6413	RPGR	cna	gain	BASIS
PD7215	RPGR	cna	gain	BASIS

PD8621	RPGR	cna	amp	BASIS
PD8980	RPGR	cna	hetloss	BASIS
PD9004	RPGR	cna	gain	BASIS
PD9585	RPGR	cna	hetloss	BASIS
PD9702	RPGR	cna	hetloss	BASIS
TCGA-AN-A0XU	RPS14	cna	hetloss	TCGA
TCGA-AO-A0JL	RPS14	cna	hetloss	TCGA
TCGA-BH-A0C0	RPS14	cna	hetloss	TCGA
TCGA-BH-A18R	RPS14	cna	gain	TCGA
TCGA-BH-A1FU	RPS14	cna	hetloss	TCGA
TCGA-C8-A12L	RPS14	cna	hetloss	TCGA
TCGA-D8-A27M	RPS14	cna	hetloss	TCGA
TCGA-E2-A1L7	RPS14	cna	hetloss	TCGA
TCGA-E9-A1NC	RPS14	cna	hetloss	TCGA
TCGA-LL-A5YP	RPS14	cna	hetloss	TCGA
MB-0346	RPS14	cna	gain	METABRIC
MB-5070	RPS14	cna	hetloss	METABRIC
MB-5465	RPS14	cna	hetloss	METABRIC
MB-6098	RPS14	cna	hetloss	METABRIC
MB-7038	RPS14	cna	hetloss	METABRIC
MB-0420	RPS14	cna	hetloss	METABRIC
PD10014	RPS14	cna	hetloss	BASIS
PD13296	RPS14	cna	hetloss	BASIS
PD13297	RPS14	cna	hetloss	BASIS
PD13771	RPS14	cna	gain	BASIS
PD22355	RPS14	cna	hetloss	BASIS
PD23578	RPS14	cna	hetloss	BASIS
PD24186	RPS14	cna	gain	BASIS
PD24202	RPS14	cna	hetloss	BASIS
PD24206	RPS14	cna	gain	BASIS
PD24337	RPS14	cna	hetloss	BASIS
PD3890	RPS14	cna	hetloss	BASIS
PD3905	RPS14	cna	gain	BASIS
PD4005	RPS14	cna	hetloss	BASIS
PD4826	RPS14	cna	gain	BASIS
PD5935	RPS14	cna	gain	BASIS
PD5945	RPS14	cna	gain	BASIS
PD6406	RPS14	cna	hetloss	BASIS
PD6413	RPS14	cna	hetloss	BASIS
PD7067	RPS14	cna	gain	BASIS
PD8980	RPS14	cna	hetloss	BASIS
PD9585	RPS14	cna	hetloss	BASIS
TCGA-A2-A25B	SDHB	cna	hetloss	TCGA
TCGA-AN-A0XU	SDHB	cna	hetloss	TCGA
TCGA-AO-A0JL	SDHB	cna	hetloss	TCGA
TCGA-BH-A0AW	SDHB	cna	gain	TCGA
TCGA-BH-A0C0	SDHB	cna	hetloss	TCGA

TCGA-BH-A18R	SDHB	cna	homdel	TCGA
TCGA-BH-A1FU	SDHB	cna	gain	TCGA
TCGA-C8-A12L	SDHB	cna	hetloss	TCGA
TCGA-D8-A27M	SDHB	cna	gain	TCGA
TCGA-E2-A1L7	SDHB	cna	hetloss	TCGA
TCGA-E9-A1NC	SDHB	cna	hetloss	TCGA
TCGA-LL-A5YP	SDHB	cna	hetloss	TCGA
MB-2827	SDHB	cna	hetloss	METABRIC
MB-5070	SDHB	cna	hetloss	METABRIC
MB-5465	SDHB	cna	hetloss	METABRIC
MB-6060	SDHB	cna	hetloss	METABRIC
MB-6098	SDHB	cna	hetloss	METABRIC
MB-6271	SDHB	cna	hetloss	METABRIC
MB-0420	SDHB	cna	hetloss	METABRIC
PD10014	SDHB	cna	gain	BASIS
PD11327	SDHB	cna	hetloss	BASIS
PD11742	SDHB	cna	hetloss	BASIS
PD13296	SDHB	cna	hetloss	BASIS
PD13771	SDHB	cna	hetloss	BASIS
PD22355	SDHB	cna	hetloss	BASIS
PD24202	SDHB	cna	hetloss	BASIS
PD24206	SDHB	cna	gain	BASIS
PD24337	SDHB	cna	hetloss	BASIS
PD3890	SDHB	cna	hetloss	BASIS
PD3905	SDHB	cna	gain	BASIS
PD4826	SDHB	cna	gain	BASIS
PD5945	SDHB	cna	gain	BASIS
PD6406	SDHB	cna	hetloss	BASIS
PD7067	SDHB	cna	gain	BASIS
PD7215	SDHB	cna	gain	BASIS
PD9004	SDHB	cna	hetloss	BASIS
PD9585	SDHB	cna	hetloss	BASIS
TCGA-A2-A25B	SMAD4	cna	hetloss	TCGA
TCGA-AN-A0XU	SMAD4	cna	hetloss	TCGA
TCGA-AO-A0JL	SMAD4	cna	gain	TCGA
TCGA-BH-A0AW	SMAD4	cna	hetloss	TCGA
TCGA-BH-A0C0	SMAD4	cna	gain	TCGA
TCGA-C8-A12L	SMAD4	cna	gain	TCGA
TCGA-D8-A27M	SMAD4	cna	hetloss	TCGA
TCGA-E2-A1L7	SMAD4	cna	gain	TCGA
TCGA-LL-A5YP	SMAD4	cna	hetloss	TCGA
MB-0346	SMAD4	cna	hetloss	METABRIC
MB-0420	SMAD4	cna	homdel	METABRIC
PD10014	SMAD4	cna	hetloss	BASIS
PD11327	SMAD4	cna	gain	BASIS
PD11742	SMAD4	cna	hetloss	BASIS
PD13296	SMAD4	cna	hetloss	BASIS

PD13297	SMAD4	cna	gain	BASIS
PD13299	SMAD4	cna	gain	BASIS
PD22355	SMAD4	cna	hetloss	BASIS
PD23574	SMAD4	cna	hetloss	BASIS
PD23578	SMAD4	cna	hetloss	BASIS
PD24186	SMAD4	cna	gain	BASIS
PD24202	SMAD4	cna	hetloss	BASIS
PD24337	SMAD4	cna	gain	BASIS
PD3905	SMAD4	cna	gain	BASIS
PD4005	SMAD4	cna	hetloss	BASIS
PD4006	SMAD4	cna	gain	BASIS
PD4107	SMAD4	cna	gain	BASIS
PD4826	SMAD4	cna	hetloss	BASIS
PD4967	SMAD4	cna	hetloss	BASIS
PD5930	SMAD4	cna	gain	BASIS
PD5945	SMAD4	cna	gain	BASIS
PD5948	SMAD4	cna	hetloss	BASIS
PD6406	SMAD4	cna	gain	BASIS
PD8980	SMAD4	cna	hetloss	BASIS
PD9004	SMAD4	cna	hetloss	BASIS
PD9585	SMAD4	cna	hetloss	BASIS
PD9702	SMAD4	cna	gain	BASIS
TCGA-A2-A25B	SMARCD1	cna	gain	TCGA
TCGA-AN-A0XU	SMARCD1	cna	gain	TCGA
TCGA-AO-A0JL	SMARCD1	cna	hetloss	TCGA
TCGA-BH-A18R	SMARCD1	cna	gain	TCGA
TCGA-BH-A1FU	SMARCD1	cna	hetloss	TCGA
TCGA-C8-A12L	SMARCD1	cna	gain	TCGA
TCGA-D8-A27M	SMARCD1	cna	hetloss	TCGA
TCGA-E2-A1L7	SMARCD1	cna	hetloss	TCGA
TCGA-E9-A1NC	SMARCD1	cna	hetloss	TCGA
TCGA-LL-A5YP	SMARCD1	cna	hetloss	TCGA
MB-0346	SMARCD1	cna	gain	METABRIC
MB-2827	SMARCD1	cna	hetloss	METABRIC
MB-5070	SMARCD1	cna	hetloss	METABRIC
MB-6060	SMARCD1	cna	gain	METABRIC
MB-6098	SMARCD1	cna	hetloss	METABRIC
MB-7038	SMARCD1	cna	hetloss	METABRIC
MB-7048	SMARCD1	cna	hetloss	METABRIC
MB-0420	SMARCD1	cna	hetloss	METABRIC
PD11327	SMARCD1	cna	hetloss	BASIS
PD13296	SMARCD1	cna	hetloss	BASIS
PD13297	SMARCD1	cna	hetloss	BASIS
PD13299	SMARCD1	cna	gain	BASIS
PD23578	SMARCD1	cna	hetloss	BASIS
PD24202	SMARCD1	cna	hetloss	BASIS
PD24206	SMARCD1	cna	gain	BASIS

PD24337	SMARCD1	cna	hetloss	BASIS
PD3890	SMARCD1	cna	hetloss	BASIS
PD3905	SMARCD1	cna	gain	BASIS
PD4107	SMARCD1	cna	hetloss	BASIS
PD4826	SMARCD1	cna	gain	BASIS
PD5945	SMARCD1	cna	gain	BASIS
PD6413	SMARCD1	cna	hetloss	BASIS
PD6731	SMARCD1	cna	hetloss	BASIS
PD7067	SMARCD1	cna	gain	BASIS
PD8621	SMARCD1	cna	gain	BASIS
PD9585	SMARCD1	cna	hetloss	BASIS
PD9702	SMARCD1	cna	gain	BASIS
TCGA-A2-A25B	SMO	cna	gain	TCGA
TCGA-AO-A0JL	SMO	cna	gain	TCGA
TCGA-BH-A0AW	SMO	cna	gain	TCGA
TCGA-C8-A12L	SMO	cna	hetloss	TCGA
TCGA-D8-A27M	SMO	cna	gain	TCGA
TCGA-E2-A1L7	SMO	cna	hetloss	TCGA
TCGA-E9-A1NC	SMO	cna	gain	TCGA
TCGA-LL-A5YP	SMO	cna	gain	TCGA
MB-0346	SMO	cna	hetloss	METABRIC
MB-2827	SMO	cna	amp	METABRIC
MB-7038	SMO	cna	gain	METABRIC
MB-7048	SMO	cna	gain	METABRIC
MTS-T0064	SMO	cna	hetloss	METABRIC
PD13296	SMO	cna	hetloss	BASIS
PD13297	SMO	cna	gain	BASIS
PD13299	SMO	cna	gain	BASIS
PD22355	SMO	cna	hetloss	BASIS
PD23574	SMO	cna	gain	BASIS
PD23578	SMO	cna	gain	BASIS
PD24186	SMO	cna	amp	BASIS
PD3890	SMO	cna	hetloss	BASIS
PD3905	SMO	cna	gain	BASIS
PD4006	SMO	cna	gain	BASIS
PD4107	SMO	cna	gain	BASIS
PD4826	SMO	cna	gain	BASIS
PD4967	SMO	cna	hetloss	BASIS
PD5930	SMO	cna	gain	BASIS
PD5935	SMO	cna	gain	BASIS
PD5945	SMO	cna	amp	BASIS
PD5948	SMO	cna	gain	BASIS
PD6406	SMO	cna	gain	BASIS
PD7067	SMO	cna	gain	BASIS
PD7215	SMO	cna	gain	BASIS
PD8621	SMO	cna	gain	BASIS
PD9004	SMO	cna	gain	BASIS

PD9585	SMO	cna	gain	BASIS
PD9702	SMO	cna	amp	BASIS
TCGA-AN-A0XU	SOCS1	cna	gain	TCGA
TCGA-AO-A0JL	SOCS1	cna	amp	TCGA
TCGA-BH-A0AW	SOCS1	cna	gain	TCGA
TCGA-BH-A0C0	SOCS1	cna	gain	TCGA
TCGA-C8-A12L	SOCS1	cna	gain	TCGA
TCGA-E2-A1L7	SOCS1	cna	hetloss	TCGA
TCGA-LL-A5YP	SOCS1	cna	gain	TCGA
MB-0346	SOCS1	cna	gain	METABRIC
MB-6060	SOCS1	cna	gain	METABRIC
MB-6098	SOCS1	cna	hetloss	METABRIC
MB-6271	SOCS1	cna	gain	METABRIC
MB-7032	SOCS1	cna	gain	METABRIC
MB-7038	SOCS1	cna	hetloss	METABRIC
MB-0420	SOCS1	cna	hetloss	METABRIC
PD10014	SOCS1	cna	hetloss	BASIS
PD11327	SOCS1	cna	hetloss	BASIS
PD11742	SOCS1	cna	gain	BASIS
PD13296	SOCS1	cna	gain	BASIS
PD13297	SOCS1	cna	hetloss	BASIS
PD13299	SOCS1	cna	gain	BASIS
PD22355	SOCS1	cna	hetloss	BASIS
PD23578	SOCS1	cna	hetloss	BASIS
PD24202	SOCS1	cna	hetloss	BASIS
PD24206	SOCS1	cna	gain	BASIS
PD24337	SOCS1	cna	hetloss	BASIS
PD3905	SOCS1	cna	gain	BASIS
PD4005	SOCS1	cna	gain	BASIS
PD4107	SOCS1	cna	gain	BASIS
PD4826	SOCS1	cna	gain	BASIS
PD5935	SOCS1	cna	gain	BASIS
PD5945	SOCS1	cna	gain	BASIS
PD5948	SOCS1	cna	gain	BASIS
PD7067	SOCS1	cna	gain	BASIS
PD7215	SOCS1	cna	amp	BASIS
PD9004	SOCS1	cna	gain	BASIS
PD9585	SOCS1	cna	hetloss	BASIS
PD9702	SOCS1	cna	gain	BASIS
TCGA-A2-A25B	SSX1	cna	gain	TCGA
TCGA-AN-A0XU	SSX1	cna	hetloss	TCGA
TCGA-AO-A0JL	SSX1	cna	gain	TCGA
TCGA-BH-A0AW	SSX1	cna	gain	TCGA
TCGA-E2-A1L7	SSX1	cna	hetloss	TCGA
TCGA-E9-A1NC	SSX1	cna	hetloss	TCGA
MB-0346	SSX1	cna	hetloss	METABRIC
MB-2827	SSX1	cna	hetloss	METABRIC

MB-5465	SSX1	cna	gain	METABRIC
MB-6098	SSX1	cna	hetloss	METABRIC
MB-0420	SSX1	cna	hetloss	METABRIC
PD10014	SSX1	cna	hetloss	BASIS
PD11327	SSX1	cna	homdel	BASIS
PD13296	SSX1	cna	gain	BASIS
PD13297	SSX1	cna	gain	BASIS
PD13299	SSX1	cna	amp	BASIS
PD13771	SSX1	cna	gain	BASIS
PD14442	SSX1	cna	gain	BASIS
PD23562	SSX1	cna	hetloss	BASIS
PD23574	SSX1	cna	gain	BASIS
PD24206	SSX1	cna	hetloss	BASIS
PD3905	SSX1	cna	gain	BASIS
PD4005	SSX1	cna	hetloss	BASIS
PD4006	SSX1	cna	hetloss	BASIS
PD4107	SSX1	cna	gain	BASIS
PD4826	SSX1	cna	gain	BASIS
PD4967	SSX1	cna	gain	BASIS
PD5935	SSX1	cna	gain	BASIS
PD5945	SSX1	cna	gain	BASIS
PD5948	SSX1	cna	gain	BASIS
PD6406	SSX1	cna	gain	BASIS
PD6413	SSX1	cna	gain	BASIS
PD7067	SSX1	cna	amp	BASIS
PD7215	SSX1	cna	amp	BASIS
PD8621	SSX1	cna	amp	BASIS
PD8980	SSX1	cna	hetloss	BASIS
PD9004	SSX1	cna	gain	BASIS
TCGA-A2-A25B	SSX2	cna	gain	TCGA
TCGA-AN-A0XU	SSX2	cna	hetloss	TCGA
TCGA-AO-A0JL	SSX2	cna	gain	TCGA
TCGA-BH-A0AW	SSX2	cna	gain	TCGA
TCGA-E2-A1L7	SSX2	cna	hetloss	TCGA
TCGA-E9-A1NC	SSX2	cna	hetloss	TCGA
MB-0346	SSX2	cna	gain	METABRIC
MB-2827	SSX2	cna	hetloss	METABRIC
MB-5465	SSX2	cna	gain	METABRIC
MB-6098	SSX2	cna	hetloss	METABRIC
MB-0420	SSX2	cna	hetloss	METABRIC
PD10014	SSX2	cna	hetloss	BASIS
PD11327	SSX2	cna	homdel	BASIS
PD13296	SSX2	cna	gain	BASIS
PD13297	SSX2	cna	gain	BASIS
PD13299	SSX2	cna	gain	BASIS
PD13771	SSX2	cna	gain	BASIS
PD14442	SSX2	cna	gain	BASIS

PD23562	SSX2	cna	hetloss	BASIS
PD23574	SSX2	cna	gain	BASIS
PD24206	SSX2	cna	amp	BASIS
PD3905	SSX2	cna	gain	BASIS
PD4005	SSX2	cna	hetloss	BASIS
PD4006	SSX2	cna	hetloss	BASIS
PD4107	SSX2	cna	gain	BASIS
PD4826	SSX2	cna	gain	BASIS
PD4967	SSX2	cna	gain	BASIS
PD5935	SSX2	cna	gain	BASIS
PD5945	SSX2	cna	gain	BASIS
PD5948	SSX2	cna	gain	BASIS
PD6406	SSX2	cna	gain	BASIS
PD6413	SSX2	cna	gain	BASIS
PD7067	SSX2	cna	gain	BASIS
PD7215	SSX2	cna	amp	BASIS
PD8621	SSX2	cna	amp	BASIS
PD8980	SSX2	cna	hetloss	BASIS
PD9004	SSX2	cna	gain	BASIS
TCGA-A2-A25B	SSX4	cna	gain	TCGA
TCGA-AN-A0XU	SSX4	cna	hetloss	TCGA
TCGA-AO-A0JL	SSX4	cna	gain	TCGA
TCGA-BH-A0AW	SSX4	cna	gain	TCGA
TCGA-E2-A1L7	SSX4	cna	hetloss	TCGA
TCGA-E9-A1NC	SSX4	cna	hetloss	TCGA
MB-0346	SSX4	cna	hetloss	METABRIC
MB-2827	SSX4	cna	hetloss	METABRIC
MB-5465	SSX4	cna	gain	METABRIC
MB-6098	SSX4	cna	hetloss	METABRIC
MB-0420	SSX4	cna	hetloss	METABRIC
PD10014	SSX4	cna	hetloss	BASIS
PD11327	SSX4	cna	homdel	BASIS
PD13296	SSX4	cna	gain	BASIS
PD13297	SSX4	cna	gain	BASIS
PD13299	SSX4	cna	amp	BASIS
PD13771	SSX4	cna	gain	BASIS
PD14442	SSX4	cna	gain	BASIS
PD23562	SSX4	cna	hetloss	BASIS
PD23574	SSX4	cna	gain	BASIS
PD24206	SSX4	cna	hetloss	BASIS
PD3905	SSX4	cna	gain	BASIS
PD4005	SSX4	cna	hetloss	BASIS
PD4006	SSX4	cna	hetloss	BASIS
PD4107	SSX4	cna	gain	BASIS
PD4826	SSX4	cna	gain	BASIS
PD4967	SSX4	cna	gain	BASIS
PD5935	SSX4	cna	gain	BASIS

PD5945	SSX4	cna	gain	BASIS
PD5948	SSX4	cna	gain	BASIS
PD6406	SSX4	cna	gain	BASIS
PD6413	SSX4	cna	gain	BASIS
PD7067	SSX4	cna	amp	BASIS
PD7215	SSX4	cna	amp	BASIS
PD8621	SSX4	cna	amp	BASIS
PD8980	SSX4	cna	hetloss	BASIS
PD9004	SSX4	cna	gain	BASIS
TCGA-A2-A25B	STAG2	cna	hetloss	TCGA
TCGA-AN-A0XU	STAG2	cna	hetloss	TCGA
TCGA-BH-A0AW	STAG2	cna	gain	TCGA
TCGA-C8-A12L	STAG2	cna	gain	TCGA
TCGA-E2-A1L7	STAG2	cna	hetloss	TCGA
TCGA-E9-A1NC	STAG2	cna	hetloss	TCGA
MB-0346	STAG2	cna	hetloss	METABRIC
MB-2827	STAG2	cna	hetloss	METABRIC
MB-5465	STAG2	cna	hetloss	METABRIC
MB-6098	STAG2	cna	hetloss	METABRIC
MB-7038	STAG2	cna	gain	METABRIC
MB-7048	STAG2	cna	hetloss	METABRIC
PD10014	STAG2	cna	hetloss	BASIS
PD11327	STAG2	cna	hetloss	BASIS
PD13296	STAG2	cna	hetloss	BASIS
PD13299	STAG2	cna	amp	BASIS
PD13771	STAG2	cna	gain	BASIS
PD14442	STAG2	cna	gain	BASIS
PD23562	STAG2	cna	hetloss	BASIS
PD23574	STAG2	cna	gain	BASIS
PD23578	STAG2	cna	hetloss	BASIS
PD24202	STAG2	cna	hetloss	BASIS
PD24206	STAG2	cna	gain	BASIS
PD3905	STAG2	cna	gain	BASIS
PD4107	STAG2	cna	gain	BASIS
PD4826	STAG2	cna	gain	BASIS
PD4967	STAG2	cna	gain	BASIS
PD5930	STAG2	cna	gain	BASIS
PD5935	STAG2	cna	gain	BASIS
PD5945	STAG2	cna	gain	BASIS
PD5948	STAG2	cna	gain	BASIS
PD6406	STAG2	cna	gain	BASIS
PD6413	STAG2	cna	gain	BASIS
PD7215	STAG2	cna	gain	BASIS
PD8621	STAG2	cna	amp	BASIS
PD8980	STAG2	cna	hetloss	BASIS
PD9004	STAG2	cna	gain	BASIS
TCGA-AN-A0XU	STK11	cna	hetloss	TCGA

TCGA-AO-A0JL	STK11	cna	gain	TCGA
TCGA-BH-A0C0	STK11	cna	gain	TCGA
TCGA-BH-A1FU	STK11	cna	hetloss	TCGA
TCGA-C8-A12L	STK11	cna	hetloss	TCGA
TCGA-D8-A27M	STK11	cna	hetloss	TCGA
TCGA-E9-A1NC	STK11	cna	hetloss	TCGA
TCGA-EW-A1OX	STK11	cna	hetloss	TCGA
TCGA-LL-A5YP	STK11	cna	hetloss	TCGA
MB-0346	STK11	cna	gain	METABRIC
MB-2827	STK11	cna	hetloss	METABRIC
MB-5070	STK11	cna	hetloss	METABRIC
MB-6098	STK11	cna	hetloss	METABRIC
MB-7038	STK11	cna	hetloss	METABRIC
MB-0420	STK11	cna	hetloss	METABRIC
P-0009557-T01-IM5	STK11	cna	homdel	MSK-IMPACT
PD11327	STK11	cna	amp	BASIS
PD11742	STK11	cna	hetloss	BASIS
PD14442	STK11	cna	hetloss	BASIS
PD22355	STK11	cna	hetloss	BASIS
PD23574	STK11	cna	gain	BASIS
PD23578	STK11	cna	hetloss	BASIS
PD24206	STK11	cna	hetloss	BASIS
PD3890	STK11	cna	hetloss	BASIS
PD3905	STK11	cna	gain	BASIS
PD4005	STK11	cna	gain	BASIS
PD4006	STK11	cna	gain	BASIS
PD4826	STK11	cna	hetloss	BASIS
PD4967	STK11	cna	hetloss	BASIS
PD5930	STK11	cna	gain	BASIS
PD5945	STK11	cna	gain	BASIS
PD5948	STK11	cna	gain	BASIS
PD7067	STK11	cna	gain	BASIS
PD8621	STK11	cna	gain	BASIS
PD8980	STK11	cna	gain	BASIS
PD9004	STK11	cna	hetloss	BASIS
PD9585	STK11	cna	hetloss	BASIS
TCGA-A2-A25B	TFE3	cna	gain	TCGA
TCGA-AN-A0XU	TFE3	cna	hetloss	TCGA
TCGA-AO-A0JL	TFE3	cna	gain	TCGA
TCGA-BH-A0AW	TFE3	cna	gain	TCGA
TCGA-E2-A1L7	TFE3	cna	hetloss	TCGA
TCGA-E9-A1NC	TFE3	cna	hetloss	TCGA
MB-0346	TFE3	cna	gain	METABRIC
MB-2827	TFE3	cna	hetloss	METABRIC
MB-5465	TFE3	cna	gain	METABRIC
MB-6098	TFE3	cna	hetloss	METABRIC
MB-0420	TFE3	cna	hetloss	METABRIC

PD10014	TFE3	cna	hetloss	BASIS
PD11327	TFE3	cna	homdel	BASIS
PD13296	TFE3	cna	gain	BASIS
PD13297	TFE3	cna	gain	BASIS
PD13299	TFE3	cna	amp	BASIS
PD13771	TFE3	cna	gain	BASIS
PD14442	TFE3	cna	gain	BASIS
PD23562	TFE3	cna	hetloss	BASIS
PD23574	TFE3	cna	gain	BASIS
PD24206	TFE3	cna	hetloss	BASIS
PD3905	TFE3	cna	gain	BASIS
PD4005	TFE3	cna	hetloss	BASIS
PD4006	TFE3	cna	hetloss	BASIS
PD4107	TFE3	cna	gain	BASIS
PD4826	TFE3	cna	gain	BASIS
PD4967	TFE3	cna	gain	BASIS
PD5935	TFE3	cna	gain	BASIS
PD5945	TFE3	cna	gain	BASIS
PD5948	TFE3	cna	gain	BASIS
PD6406	TFE3	cna	gain	BASIS
PD6413	TFE3	cna	gain	BASIS
PD7067	TFE3	cna	gain	BASIS
PD7215	TFE3	cna	amp	BASIS
PD8621	TFE3	cna	amp	BASIS
PD8980	TFE3	cna	hetloss	BASIS
PD9004	TFE3	cna	gain	BASIS
TCGA-A2-A25B	TNFRSF11A	cna	hetloss	TCGA
TCGA-AO-A0JL	TNFRSF11A	cna	gain	TCGA
TCGA-BH-A0AW	TNFRSF11A	cna	hetloss	TCGA
TCGA-BH-A0C0	TNFRSF11A	cna	gain	TCGA
TCGA-C8-A12L	TNFRSF11A	cna	gain	TCGA
TCGA-D8-A27M	TNFRSF11A	cna	hetloss	TCGA
TCGA-E2-A1L7	TNFRSF11A	cna	gain	TCGA
TCGA-LL-A5YP	TNFRSF11A	cna	hetloss	TCGA
MB-0346	TNFRSF11A	cna	hetloss	METABRIC
MB-7048	TNFRSF11A	cna	hetloss	METABRIC
MB-0420	TNFRSF11A	cna	hetloss	METABRIC
MTS-T0064	TNFRSF11A	cna	hetloss	METABRIC
PD10014	TNFRSF11A	cna	hetloss	BASIS
PD11742	TNFRSF11A	cna	hetloss	BASIS
PD13296	TNFRSF11A	cna	hetloss	BASIS
PD13297	TNFRSF11A	cna	gain	BASIS
PD13299	TNFRSF11A	cna	gain	BASIS
PD22355	TNFRSF11A	cna	hetloss	BASIS
PD23574	TNFRSF11A	cna	hetloss	BASIS
PD23578	TNFRSF11A	cna	hetloss	BASIS
PD24186	TNFRSF11A	cna	gain	BASIS

PD24202	TNFRSF11A	cna	hetloss	BASIS
PD24337	TNFRSF11A	cna	gain	BASIS
PD3905	TNFRSF11A	cna	gain	BASIS
PD4006	TNFRSF11A	cna	gain	BASIS
PD4107	TNFRSF11A	cna	gain	BASIS
PD4826	TNFRSF11A	cna	hetloss	BASIS
PD4967	TNFRSF11A	cna	hetloss	BASIS
PD5930	TNFRSF11A	cna	hetloss	BASIS
PD5945	TNFRSF11A	cna	gain	BASIS
PD5948	TNFRSF11A	cna	gain	BASIS
PD6406	TNFRSF11A	cna	gain	BASIS
PD7067	TNFRSF11A	cna	gain	BASIS
PD8980	TNFRSF11A	cna	hetloss	BASIS
PD9004	TNFRSF11A	cna	hetloss	BASIS
PD9585	TNFRSF11A	cna	hetloss	BASIS
PD9702	TNFRSF11A	cna	gain	BASIS
TCGA-A2-A25B	TSHR	cna	hetloss	TCGA
TCGA-AO-A0JL	TSHR	cna	hetloss	TCGA
TCGA-BH-A0AW	TSHR	cna	hetloss	TCGA
TCGA-BH-A1FU	TSHR	cna	gain	TCGA
TCGA-C8-A12L	TSHR	cna	hetloss	TCGA
TCGA-D8-A27M	TSHR	cna	hetloss	TCGA
TCGA-E2-A1L7	TSHR	cna	hetloss	TCGA
TCGA-LL-A5YP	TSHR	cna	hetloss	TCGA
MB-0346	TSHR	cna	gain	METABRIC
MB-2827	TSHR	cna	hetloss	METABRIC
MB-5070	TSHR	cna	hetloss	METABRIC
MB-5107	TSHR	cna	hetloss	METABRIC
MTS-T0064	TSHR	cna	amp	METABRIC
PD10014	TSHR	cna	hetloss	BASIS
PD11327	TSHR	cna	gain	BASIS
PD11742	TSHR	cna	hetloss	BASIS
PD13296	TSHR	cna	gain	BASIS
PD13297	TSHR	cna	hetloss	BASIS
PD13771	TSHR	cna	hetloss	BASIS
PD23574	TSHR	cna	gain	BASIS
PD23578	TSHR	cna	hetloss	BASIS
PD24202	TSHR	cna	hetloss	BASIS
PD24206	TSHR	cna	gain	BASIS
PD24337	TSHR	cna	hetloss	BASIS
PD3890	TSHR	cna	hetloss	BASIS
PD3905	TSHR	cna	gain	BASIS
PD4005	TSHR	cna	hetloss	BASIS
PD4107	TSHR	cna	hetloss	BASIS
PD4826	TSHR	cna	gain	BASIS
PD5945	TSHR	cna	gain	BASIS
PD6406	TSHR	cna	hetloss	BASIS

PD6413	TSHR	cna	hetloss	BASIS
PD7067	TSHR	cna	gain	BASIS
PD7215	TSHR	cna	hetloss	BASIS
PD8621	TSHR	cna	hetloss	BASIS
PD8980	TSHR	cna	hetloss	BASIS
PD9702	TSHR	cna	gain	BASIS
TCGA-A2-A25B	U2AF1	cna	hetloss	TCGA
TCGA-BH-A0AW	U2AF1	cna	gain	TCGA
TCGA-BH-A0C0	U2AF1	cna	hetloss	TCGA
TCGA-BH-A1FU	U2AF1	cna	gain	TCGA
TCGA-C8-A12L	U2AF1	cna	hetloss	TCGA
TCGA-D8-A27M	U2AF1	cna	hetloss	TCGA
TCGA-LL-A5YP	U2AF1	cna	gain	TCGA
MB-2827	U2AF1	cna	amp	METABRIC
MB-6098	U2AF1	cna	hetloss	METABRIC
MB-7038	U2AF1	cna	gain	METABRIC
MB-7048	U2AF1	cna	hetloss	METABRIC
PD10014	U2AF1	cna	hetloss	BASIS
PD11327	U2AF1	cna	gain	BASIS
PD13296	U2AF1	cna	gain	BASIS
PD13297	U2AF1	cna	hetloss	BASIS
PD13299	U2AF1	cna	gain	BASIS
PD13771	U2AF1	cna	gain	BASIS
PD23562	U2AF1	cna	gain	BASIS
PD23574	U2AF1	cna	gain	BASIS
PD23578	U2AF1	cna	gain	BASIS
PD24186	U2AF1	cna	gain	BASIS
PD24206	U2AF1	cna	gain	BASIS
PD3890	U2AF1	cna	hetloss	BASIS
PD3905	U2AF1	cna	gain	BASIS
PD4006	U2AF1	cna	amp	BASIS
PD4107	U2AF1	cna	gain	BASIS
PD4826	U2AF1	cna	gain	BASIS
PD5930	U2AF1	cna	gain	BASIS
PD5945	U2AF1	cna	gain	BASIS
PD5948	U2AF1	cna	gain	BASIS
PD6731	U2AF1	cna	gain	BASIS
PD7067	U2AF1	cna	gain	BASIS
PD7215	U2AF1	cna	gain	BASIS
PD8621	U2AF1	cna	gain	BASIS
PD8980	U2AF1	cna	hetloss	BASIS
PD9004	U2AF1	cna	gain	BASIS
PD9702	U2AF1	cna	gain	BASIS
TCGA-A2-A25B	WAS	cna	gain	TCGA
TCGA-AN-A0XU	WAS	cna	hetloss	TCGA
TCGA-AO-A0JL	WAS	cna	gain	TCGA
TCGA-BH-A0AW	WAS	cna	gain	TCGA

TCGA-E2-A1L7	WAS	cna	hetloss	TCGA
TCGA-E9-A1NC	WAS	cna	hetloss	TCGA
MB-0346	WAS	cna	gain	METABRIC
MB-2827	WAS	cna	hetloss	METABRIC
MB-5465	WAS	cna	gain	METABRIC
MB-6098	WAS	cna	hetloss	METABRIC
MB-0420	WAS	cna	hetloss	METABRIC
PD10014	WAS	cna	hetloss	BASIS
PD11327	WAS	cna	homdel	BASIS
PD13296	WAS	cna	gain	BASIS
PD13297	WAS	cna	gain	BASIS
PD13299	WAS	cna	amp	BASIS
PD13771	WAS	cna	gain	BASIS
PD14442	WAS	cna	gain	BASIS
PD23562	WAS	cna	hetloss	BASIS
PD23574	WAS	cna	gain	BASIS
PD24206	WAS	cna	hetloss	BASIS
PD3905	WAS	cna	gain	BASIS
PD4005	WAS	cna	hetloss	BASIS
PD4006	WAS	cna	hetloss	BASIS
PD4107	WAS	cna	gain	BASIS
PD4826	WAS	cna	gain	BASIS
PD4967	WAS	cna	gain	BASIS
PD5935	WAS	cna	gain	BASIS
PD5945	WAS	cna	gain	BASIS
PD5948	WAS	cna	gain	BASIS
PD6406	WAS	cna	gain	BASIS
PD6413	WAS	cna	gain	BASIS
PD7067	WAS	cna	gain	BASIS
PD7215	WAS	cna	amp	BASIS
PD8621	WAS	cna	amp	BASIS
PD8980	WAS	cna	hetloss	BASIS
PD9004	WAS	cna	gain	BASIS
TCGA-A2-A25B	XIAP	cna	hetloss	TCGA
TCGA-AN-A0XU	XIAP	cna	hetloss	TCGA
TCGA-BH-A0AW	XIAP	cna	gain	TCGA
TCGA-C8-A12L	XIAP	cna	gain	TCGA
TCGA-E2-A1L7	XIAP	cna	hetloss	TCGA
TCGA-E9-A1NC	XIAP	cna	hetloss	TCGA
MB-0346	XIAP	cna	hetloss	METABRIC
MB-2827	XIAP	cna	hetloss	METABRIC
MB-5465	XIAP	cna	hetloss	METABRIC
MB-6098	XIAP	cna	hetloss	METABRIC
MB-7038	XIAP	cna	gain	METABRIC
MB-7048	XIAP	cna	hetloss	METABRIC
PD10014	XIAP	cna	hetloss	BASIS
PD11327	XIAP	cna	homdel	BASIS

PD13296	XIAP	cna	hetloss	BASIS
PD13299	XIAP	cna	amp	BASIS
PD13771	XIAP	cna	gain	BASIS
PD14442	XIAP	cna	gain	BASIS
PD23562	XIAP	cna	hetloss	BASIS
PD23574	XIAP	cna	gain	BASIS
PD23578	XIAP	cna	hetloss	BASIS
PD24202	XIAP	cna	hetloss	BASIS
PD24206	XIAP	cna	gain	BASIS
PD3905	XIAP	cna	gain	BASIS
PD4107	XIAP	cna	gain	BASIS
PD4826	XIAP	cna	gain	BASIS
PD4967	XIAP	cna	gain	BASIS
PD5930	XIAP	cna	gain	BASIS
PD5935	XIAP	cna	gain	BASIS
PD5945	XIAP	cna	gain	BASIS
PD5948	XIAP	cna	gain	BASIS
PD6406	XIAP	cna	gain	BASIS
PD6413	XIAP	cna	gain	BASIS
PD7215	XIAP	cna	gain	BASIS
PD8621	XIAP	cna	amp	BASIS
PD8980	XIAP	cna	hetloss	BASIS
PD9004	XIAP	cna	gain	BASIS
TCGA-AN-A0XU	ZNF300	cna	hetloss	TCGA
TCGA-AO-A0JL	ZNF300	cna	gain	TCGA
TCGA-BH-A0C0	ZNF300	cna	hetloss	TCGA
TCGA-BH-A1FU	ZNF300	cna	hetloss	TCGA
TCGA-C8-A12L	ZNF300	cna	hetloss	TCGA
TCGA-D8-A27M	ZNF300	cna	hetloss	TCGA
TCGA-E2-A1L7	ZNF300	cna	hetloss	TCGA
TCGA-E9-A1NC	ZNF300	cna	hetloss	TCGA
TCGA-LL-A5YP	ZNF300	cna	hetloss	TCGA
MB-0346	ZNF300	cna	gain	METABRIC
MB-5070	ZNF300	cna	hetloss	METABRIC
MB-5465	ZNF300	cna	hetloss	METABRIC
MB-6098	ZNF300	cna	hetloss	METABRIC
MB-7038	ZNF300	cna	hetloss	METABRIC
MB-0420	ZNF300	cna	hetloss	METABRIC
PD10014	ZNF300	cna	hetloss	BASIS
PD13296	ZNF300	cna	hetloss	BASIS
PD13297	ZNF300	cna	hetloss	BASIS
PD13771	ZNF300	cna	gain	BASIS
PD22355	ZNF300	cna	hetloss	BASIS
PD23578	ZNF300	cna	hetloss	BASIS
PD24186	ZNF300	cna	gain	BASIS
PD24202	ZNF300	cna	hetloss	BASIS
PD24206	ZNF300	cna	gain	BASIS

PD24337	ZNF300	cna	hetloss	BASIS
PD3890	ZNF300	cna	hetloss	BASIS
PD3905	ZNF300	cna	gain	BASIS
PD4005	ZNF300	cna	hetloss	BASIS
PD4826	ZNF300	cna	gain	BASIS
PD5935	ZNF300	cna	gain	BASIS
PD5945	ZNF300	cna	gain	BASIS
PD6406	ZNF300	cna	hetloss	BASIS
PD6413	ZNF300	cna	hetloss	BASIS
PD7067	ZNF300	cna	gain	BASIS
PD8621	ZNF300	cna	hetloss	BASIS
PD8980	ZNF300	cna	hetloss	BASIS
PD9585	ZNF300	cna	hetloss	BASIS
TCGA-A2-A25B	ABI1	cna	gain	TCGA
TCGA-AN-A0XU	ABI1	cna	hetloss	TCGA
TCGA-AO-A0JL	ABI1	cna	gain	TCGA
TCGA-BH-A0AW	ABI1	cna	gain	TCGA
TCGA-BH-A0C0	ABI1	cna	gain	TCGA
TCGA-C8-A12L	ABI1	cna	gain	TCGA
TCGA-LL-A5YP	ABI1	cna	gain	TCGA
MB-6098	ABI1	cna	gain	METABRIC
MB-7038	ABI1	cna	hetloss	METABRIC
MB-7048	ABI1	cna	hetloss	METABRIC
MTS-T0064	ABI1	cna	gain	METABRIC
PD11327	ABI1	cna	gain	BASIS
PD13296	ABI1	cna	gain	BASIS
PD13299	ABI1	cna	gain	BASIS
PD13771	ABI1	cna	gain	BASIS
PD23562	ABI1	cna	amp	BASIS
PD23574	ABI1	cna	gain	BASIS
PD24186	ABI1	cna	gain	BASIS
PD24202	ABI1	cna	hetloss	BASIS
PD3905	ABI1	cna	gain	BASIS
PD4005	ABI1	cna	gain	BASIS
PD4006	ABI1	cna	gain	BASIS
PD4107	ABI1	cna	gain	BASIS
PD4826	ABI1	cna	gain	BASIS
PD4967	ABI1	cna	hetloss	BASIS
PD5930	ABI1	cna	gain	BASIS
PD5945	ABI1	cna	amp	BASIS
PD5948	ABI1	cna	gain	BASIS
PD6406	ABI1	cna	hetloss	BASIS
PD6413	ABI1	cna	gain	BASIS
PD7067	ABI1	cna	amp	BASIS
PD7215	ABI1	cna	gain	BASIS
PD8621	ABI1	cna	gain	BASIS
PD8980	ABI1	cna	gain	BASIS

PD9004	ABI1	cna	gain	BASIS
PD9702	ABI1	cna	gain	BASIS
TCGA-AN-A0XU	AJUBA	cna	hetloss	TCGA
TCGA-AO-A0JL	AJUBA	cna	hetloss	TCGA
TCGA-BH-A0AW	AJUBA	cna	hetloss	TCGA
TCGA-BH-A1FU	AJUBA	cna	gain	TCGA
TCGA-D8-A27M	AJUBA	cna	hetloss	TCGA
TCGA-LL-A5YP	AJUBA	cna	hetloss	TCGA
MB-2827	AJUBA	cna	amp	METABRIC
MB-5070	AJUBA	cna	hetloss	METABRIC
MB-6060	AJUBA	cna	gain	METABRIC
MB-7048	AJUBA	cna	hetloss	METABRIC
PD10014	AJUBA	cna	gain	BASIS
PD11327	AJUBA	cna	homdel	BASIS
PD13299	AJUBA	cna	gain	BASIS
PD14442	AJUBA	cna	hetloss	BASIS
PD22355	AJUBA	cna	hetloss	BASIS
PD23574	AJUBA	cna	gain	BASIS
PD23578	AJUBA	cna	hetloss	BASIS
PD24186	AJUBA	cna	gain	BASIS
PD24202	AJUBA	cna	hetloss	BASIS
PD24206	AJUBA	cna	gain	BASIS
PD3890	AJUBA	cna	hetloss	BASIS
PD3905	AJUBA	cna	gain	BASIS
PD4005	AJUBA	cna	hetloss	BASIS
PD4826	AJUBA	cna	gain	BASIS
PD4967	AJUBA	cna	hetloss	BASIS
PD5930	AJUBA	cna	hetloss	BASIS
PD5935	AJUBA	cna	gain	BASIS
PD5945	AJUBA	cna	amp	BASIS
PD5948	AJUBA	cna	gain	BASIS
PD6406	AJUBA	cna	hetloss	BASIS
PD6731	AJUBA	cna	hetloss	BASIS
PD7215	AJUBA	cna	gain	BASIS
PD8621	AJUBA	cna	gain	BASIS
PD8980	AJUBA	cna	amp	BASIS
PD9004	AJUBA	cna	gain	BASIS
PD9702	AJUBA	cna	gain	BASIS
TCGA-A2-A25B	ALOX12B	cna	hetloss	TCGA
TCGA-AN-A0XU	ALOX12B	cna	hetloss	TCGA
TCGA-AO-A0JL	ALOX12B	cna	gain	TCGA
TCGA-BH-A0AW	ALOX12B	cna	hetloss	TCGA
TCGA-BH-A0C0	ALOX12B	cna	hetloss	TCGA
TCGA-C8-A12L	ALOX12B	cna	hetloss	TCGA
TCGA-D8-A27M	ALOX12B	cna	hetloss	TCGA
TCGA-E2-A1L7	ALOX12B	cna	hetloss	TCGA
TCGA-E9-A1NC	ALOX12B	cna	hetloss	TCGA

TCGA-LL-A5YP	ALOX12B	cna	gain	TCGA
MB-0346	ALOX12B	cna	hetloss	METABRIC
MB-2827	ALOX12B	cna	hetloss	METABRIC
MB-5070	ALOX12B	cna	hetloss	METABRIC
MB-5107	ALOX12B	cna	hetloss	METABRIC
MB-6098	ALOX12B	cna	hetloss	METABRIC
MB-6271	ALOX12B	cna	hetloss	METABRIC
MB-7048	ALOX12B	cna	hetloss	METABRIC
MB-0420	ALOX12B	cna	hetloss	METABRIC
PD11742	ALOX12B	cna	hetloss	BASIS
PD13296	ALOX12B	cna	hetloss	BASIS
PD13771	ALOX12B	cna	hetloss	BASIS
PD22355	ALOX12B	cna	hetloss	BASIS
PD24202	ALOX12B	cna	hetloss	BASIS
PD24206	ALOX12B	cna	hetloss	BASIS
PD24337	ALOX12B	cna	hetloss	BASIS
PD3890	ALOX12B	cna	hetloss	BASIS
PD3905	ALOX12B	cna	gain	BASIS
PD4005	ALOX12B	cna	homdel	BASIS
PD4826	ALOX12B	cna	hetloss	BASIS
PD4967	ALOX12B	cna	hetloss	BASIS
PD5945	ALOX12B	cna	gain	BASIS
PD6406	ALOX12B	cna	hetloss	BASIS
PD6413	ALOX12B	cna	hetloss	BASIS
PD6731	ALOX12B	cna	hetloss	BASIS
PD7067	ALOX12B	cna	gain	BASIS
PD9585	ALOX12B	cna	hetloss	BASIS
TCGA-AN-A0XU	APCDD1	cna	hetloss	TCGA
TCGA-AO-A0JL	APCDD1	cna	gain	TCGA
TCGA-BH-A0AW	APCDD1	cna	gain	TCGA
TCGA-BH-A0C0	APCDD1	cna	hetloss	TCGA
TCGA-BH-A1FU	APCDD1	cna	gain	TCGA
TCGA-C8-A12L	APCDD1	cna	gain	TCGA
TCGA-D8-A27M	APCDD1	cna	hetloss	TCGA
TCGA-LL-A5YP	APCDD1	cna	gain	TCGA
MB-0346	APCDD1	cna	gain	METABRIC
MB-5107	APCDD1	cna	hetloss	METABRIC
MB-5465	APCDD1	cna	hetloss	METABRIC
MB-6098	APCDD1	cna	hetloss	METABRIC
MB-0420	APCDD1	cna	gain	METABRIC
PD13296	APCDD1	cna	hetloss	BASIS
PD13299	APCDD1	cna	gain	BASIS
PD23574	APCDD1	cna	gain	BASIS
PD24186	APCDD1	cna	gain	BASIS
PD24206	APCDD1	cna	gain	BASIS
PD24337	APCDD1	cna	hetloss	BASIS
PD3890	APCDD1	cna	hetloss	BASIS

PD3905	APCDD1	cna	gain	BASIS
PD4005	APCDD1	cna	hetloss	BASIS
PD4006	APCDD1	cna	gain	BASIS
PD4107	APCDD1	cna	gain	BASIS
PD4967	APCDD1	cna	hetloss	BASIS
PD5930	APCDD1	cna	gain	BASIS
PD5935	APCDD1	cna	gain	BASIS
PD5945	APCDD1	cna	amp	BASIS
PD5948	APCDD1	cna	gain	BASIS
PD7067	APCDD1	cna	gain	BASIS
PD7215	APCDD1	cna	gain	BASIS
PD8621	APCDD1	cna	gain	BASIS
PD8980	APCDD1	cna	hetloss	BASIS
PD9004	APCDD1	cna	gain	BASIS
PD9585	APCDD1	cna	hetloss	BASIS
PD9702	APCDD1	cna	gain	BASIS
TCGA-A2-A25B	ARAF	cna	gain	TCGA
TCGA-AN-A0XU	ARAF	cna	hetloss	TCGA
TCGA-AO-A0JL	ARAF	cna	gain	TCGA
TCGA-BH-A0AW	ARAF	cna	gain	TCGA
TCGA-E2-A1L7	ARAF	cna	hetloss	TCGA
TCGA-E9-A1NC	ARAF	cna	hetloss	TCGA
MB-0346	ARAF	cna	hetloss	METABRIC
MB-2827	ARAF	cna	hetloss	METABRIC
MB-5465	ARAF	cna	gain	METABRIC
MB-6098	ARAF	cna	hetloss	METABRIC
MB-0420	ARAF	cna	hetloss	METABRIC
PD10014	ARAF	cna	hetloss	BASIS
PD11327	ARAF	cna	homdel	BASIS
PD13296	ARAF	cna	gain	BASIS
PD13299	ARAF	cna	amp	BASIS
PD13771	ARAF	cna	gain	BASIS
PD14442	ARAF	cna	gain	BASIS
PD23562	ARAF	cna	hetloss	BASIS
PD23574	ARAF	cna	gain	BASIS
PD24206	ARAF	cna	homdel	BASIS
PD3905	ARAF	cna	gain	BASIS
PD4005	ARAF	cna	hetloss	BASIS
PD4006	ARAF	cna	hetloss	BASIS
PD4107	ARAF	cna	gain	BASIS
PD4826	ARAF	cna	gain	BASIS
PD4967	ARAF	cna	gain	BASIS
PD5935	ARAF	cna	gain	BASIS
PD5945	ARAF	cna	gain	BASIS
PD5948	ARAF	cna	gain	BASIS
PD6406	ARAF	cna	gain	BASIS
PD6413	ARAF	cna	gain	BASIS

PD7067	ARAF	cna	amp	BASIS
PD7215	ARAF	cna	gain	BASIS
PD8621	ARAF	cna	amp	BASIS
PD8980	ARAF	cna	hetloss	BASIS
PD9004	ARAF	cna	gain	BASIS
TCGA-A2-A25B	AURKB	cna	hetloss	TCGA
TCGA-AN-A0XU	AURKB	cna	hetloss	TCGA
TCGA-AO-A0JL	AURKB	cna	gain	TCGA
TCGA-BH-A0AW	AURKB	cna	hetloss	TCGA
TCGA-BH-A0C0	AURKB	cna	hetloss	TCGA
TCGA-C8-A12L	AURKB	cna	hetloss	TCGA
TCGA-D8-A27M	AURKB	cna	hetloss	TCGA
TCGA-E2-A1L7	AURKB	cna	hetloss	TCGA
TCGA-E9-A1NC	AURKB	cna	hetloss	TCGA
TCGA-LL-A5YP	AURKB	cna	gain	TCGA
MB-0346	AURKB	cna	hetloss	METABRIC
MB-2827	AURKB	cna	hetloss	METABRIC
MB-5070	AURKB	cna	hetloss	METABRIC
MB-5107	AURKB	cna	hetloss	METABRIC
MB-6098	AURKB	cna	hetloss	METABRIC
MB-6271	AURKB	cna	hetloss	METABRIC
MB-7048	AURKB	cna	hetloss	METABRIC
MB-0420	AURKB	cna	hetloss	METABRIC
PD11742	AURKB	cna	hetloss	BASIS
PD13296	AURKB	cna	hetloss	BASIS
PD13771	AURKB	cna	hetloss	BASIS
PD22355	AURKB	cna	hetloss	BASIS
PD24202	AURKB	cna	hetloss	BASIS
PD24206	AURKB	cna	hetloss	BASIS
PD24337	AURKB	cna	hetloss	BASIS
PD3890	AURKB	cna	hetloss	BASIS
PD3905	AURKB	cna	gain	BASIS
PD4005	AURKB	cna	homdel	BASIS
PD4826	AURKB	cna	hetloss	BASIS
PD4967	AURKB	cna	hetloss	BASIS
PD5945	AURKB	cna	gain	BASIS
PD6406	AURKB	cna	hetloss	BASIS
PD6413	AURKB	cna	hetloss	BASIS
PD6731	AURKB	cna	hetloss	BASIS
PD7067	AURKB	cna	gain	BASIS
PD9585	AURKB	cna	hetloss	BASIS
TCGA-A2-A25B	BCL11A	cna	hetloss	TCGA
TCGA-AN-A0XU	BCL11A	cna	gain	TCGA
TCGA-AO-A0JL	BCL11A	cna	gain	TCGA
TCGA-BH-A0AW	BCL11A	cna	gain	TCGA
TCGA-BH-A0C0	BCL11A	cna	gain	TCGA
TCGA-D8-A27M	BCL11A	cna	gain	TCGA

TCGA-E9-A1NC	BCL11A	cna	gain	TCGA
TCGA-LL-A5YP	BCL11A	cna	gain	TCGA
MB-2827	BCL11A	cna	hetloss	METABRIC
MB-5465	BCL11A	cna	amp	METABRIC
MB-6271	BCL11A	cna	hetloss	METABRIC
MB-7038	BCL11A	cna	gain	METABRIC
PD10014	BCL11A	cna	gain	BASIS
PD11327	BCL11A	cna	gain	BASIS
PD13296	BCL11A	cna	gain	BASIS
PD13297	BCL11A	cna	gain	BASIS
PD13299	BCL11A	cna	gain	BASIS
PD22355	BCL11A	cna	gain	BASIS
PD23562	BCL11A	cna	gain	BASIS
PD23578	BCL11A	cna	gain	BASIS
PD24186	BCL11A	cna	gain	BASIS
PD24202	BCL11A	cna	gain	BASIS
PD24206	BCL11A	cna	gain	BASIS
PD3890	BCL11A	cna	hetloss	BASIS
PD3905	BCL11A	cna	gain	BASIS
PD4006	BCL11A	cna	amp	BASIS
PD4107	BCL11A	cna	gain	BASIS
PD5930	BCL11A	cna	gain	BASIS
PD5935	BCL11A	cna	gain	BASIS
PD5945	BCL11A	cna	amp	BASIS
PD5948	BCL11A	cna	gain	BASIS
PD7067	BCL11A	cna	gain	BASIS
PD7215	BCL11A	cna	gain	BASIS
PD8621	BCL11A	cna	gain	BASIS
PD9004	BCL11A	cna	gain	BASIS
PD9702	BCL11A	cna	gain	BASIS
TCGA-A2-A25B	BLM	cna	gain	TCGA
TCGA-AN-A0XU	BLM	cna	gain	TCGA
TCGA-AO-A0JL	BLM	cna	hetloss	TCGA
TCGA-BH-A0AW	BLM	cna	hetloss	TCGA
TCGA-BH-A0C0	BLM	cna	gain	TCGA
TCGA-D8-A27M	BLM	cna	hetloss	TCGA
TCGA-E2-A1L7	BLM	cna	gain	TCGA
TCGA-LL-A5YP	BLM	cna	gain	TCGA
MB-0346	BLM	cna	hetloss	METABRIC
MB-5465	BLM	cna	hetloss	METABRIC
MB-7038	BLM	cna	gain	METABRIC
MB-7048	BLM	cna	gain	METABRIC
MB-0420	BLM	cna	gain	METABRIC
MTS-T0064	BLM	cna	amp	METABRIC
PD10014	BLM	cna	gain	BASIS
PD11327	BLM	cna	hetloss	BASIS
PD11742	BLM	cna	hetloss	BASIS

PD13299	BLM	cna	gain	BASIS
PD23562	BLM	cna	gain	BASIS
PD23574	BLM	cna	gain	BASIS
PD23578	BLM	cna	gain	BASIS
PD24202	BLM	cna	hetloss	BASIS
PD24206	BLM	cna	gain	BASIS
PD3890	BLM	cna	hetloss	BASIS
PD3905	BLM	cna	gain	BASIS
PD4006	BLM	cna	gain	BASIS
PD4107	BLM	cna	gain	BASIS
PD4967	BLM	cna	hetloss	BASIS
PD5930	BLM	cna	gain	BASIS
PD5945	BLM	cna	gain	BASIS
PD6413	BLM	cna	hetloss	BASIS
PD7067	BLM	cna	gain	BASIS
PD8621	BLM	cna	gain	BASIS
PD8980	BLM	cna	gain	BASIS
PD9585	BLM	cna	hetloss	BASIS
PD9702	BLM	cna	gain	BASIS
TCGA-A2-A25B	CARD11	cna	gain	TCGA
TCGA-AO-A0JL	CARD11	cna	gain	TCGA
TCGA-BH-A0AW	CARD11	cna	gain	TCGA
TCGA-BH-A0C0	CARD11	cna	hetloss	TCGA
TCGA-C8-A12L	CARD11	cna	gain	TCGA
TCGA-D8-A27M	CARD11	cna	hetloss	TCGA
TCGA-E2-A1L7	CARD11	cna	hetloss	TCGA
TCGA-LL-A5YP	CARD11	cna	homdel	TCGA
MB-0346	CARD11	cna	gain	METABRIC
MB-5107	CARD11	cna	hetloss	METABRIC
MB-5465	CARD11	cna	gain	METABRIC
MB-6098	CARD11	cna	hetloss	METABRIC
MB-7038	CARD11	cna	hetloss	METABRIC
MB-0420	CARD11	cna	hetloss	METABRIC
PD13296	CARD11	cna	gain	BASIS
PD13297	CARD11	cna	hetloss	BASIS
PD13771	CARD11	cna	gain	BASIS
PD22355	CARD11	cna	hetloss	BASIS
PD23574	CARD11	cna	gain	BASIS
PD24202	CARD11	cna	gain	BASIS
PD24206	CARD11	cna	gain	BASIS
PD24337	CARD11	cna	hetloss	BASIS
PD3905	CARD11	cna	gain	BASIS
PD4006	CARD11	cna	gain	BASIS
PD4107	CARD11	cna	amp	BASIS
PD4826	CARD11	cna	gain	BASIS
PD5935	CARD11	cna	amp	BASIS
PD5945	CARD11	cna	amp	BASIS

PD6406	CARD11	cna	hetloss	BASIS
PD6731	CARD11	cna	gain	BASIS
PD7067	CARD11	cna	amp	BASIS
PD7215	CARD11	cna	gain	BASIS
PD8621	CARD11	cna	gain	BASIS
PD8980	CARD11	cna	hetloss	BASIS
PD9004	CARD11	cna	gain	BASIS
PD9702	CARD11	cna	gain	BASIS
TCGA-A2-A25B	CCND1	cna	hetloss	TCGA
TCGA-AN-A0XU	CCND1	cna	hetloss	TCGA
TCGA-AO-A0JL	CCND1	cna	hetloss	TCGA
TCGA-BH-A18R	CCND1	cna	amp	TCGA
TCGA-C8-A12L	CCND1	cna	amp	TCGA
TCGA-EW-A1OX	CCND1	cna	amp	TCGA
TCGA-LL-A5YP	CCND1	cna	hetloss	TCGA
MB-0346	CCND1	cna	gain	METABRIC
MB-5107	CCND1	cna	gain	METABRIC
MB-6060	CCND1	cna	amp	METABRIC
MB-6271	CCND1	cna	amp	METABRIC
MTS-T0064	CCND1	cna	gain	METABRIC
P-0002591-T01-IM3	CCND1	cna	amp	MSK-IMPACT
P-0002858-T01-IM3	CCND1	cna	amp	MSK-IMPACT
PD10014	CCND1	cna	gain	BASIS
PD11327	CCND1	cna	gain	BASIS
PD11742	CCND1	cna	gain	BASIS
PD13771	CCND1	cna	amp	BASIS
PD23574	CCND1	cna	gain	BASIS
PD24186	CCND1	cna	amp	BASIS
PD24206	CCND1	cna	gain	BASIS
PD3905	CCND1	cna	gain	BASIS
PD4005	CCND1	cna	gain	BASIS
PD4006	CCND1	cna	amp	BASIS
PD4107	CCND1	cna	gain	BASIS
PD4826	CCND1	cna	gain	BASIS
PD5930	CCND1	cna	gain	BASIS
PD5935	CCND1	cna	gain	BASIS
PD5945	CCND1	cna	amp	BASIS
PD5948	CCND1	cna	amp	BASIS
PD6413	CCND1	cna	gain	BASIS
PD7067	CCND1	cna	gain	BASIS
PD7215	CCND1	cna	gain	BASIS
PD8980	CCND1	cna	gain	BASIS
PD9004	CCND1	cna	gain	BASIS
PD9702	CCND1	cna	gain	BASIS
TCGA-AN-A0XU	CDH1	cna	hetloss	TCGA
TCGA-AO-A0JL	CDH1	cna	gain	TCGA
TCGA-BH-A0C0	CDH1	cna	hetloss	TCGA

TCGA-C8-A12L	CDH1	cna	gain	TCGA
TCGA-D8-A27M	CDH1	cna	hetloss	TCGA
TCGA-E2-A1L7	CDH1	cna	hetloss	TCGA
TCGA-E9-A1NC	CDH1	cna	hetloss	TCGA
TCGA-LL-A5YP	CDH1	cna	gain	TCGA
MB-0346	CDH1	cna	gain	METABRIC
MB-2827	CDH1	cna	hetloss	METABRIC
MB-5107	CDH1	cna	hetloss	METABRIC
MB-6271	CDH1	cna	hetloss	METABRIC
MB-7032	CDH1	cna	gain	METABRIC
MB-7048	CDH1	cna	homdel	METABRIC
MB-0420	CDH1	cna	hetloss	METABRIC
MTS-T0064	CDH1	cna	hetloss	METABRIC
PD10014	CDH1	cna	hetloss	BASIS
PD11327	CDH1	cna	homdel	BASIS
PD11742	CDH1	cna	hetloss	BASIS
PD13297	CDH1	cna	hetloss	BASIS
PD13299	CDH1	cna	gain	BASIS
PD14442	CDH1	cna	homdel	BASIS
PD23562	CDH1	cna	gain	BASIS
PD24186	CDH1	cna	gain	BASIS
PD24202	CDH1	cna	hetloss	BASIS
PD24206	CDH1	cna	hetloss	BASIS
PD3890	CDH1	cna	gain	BASIS
PD3905	CDH1	cna	gain	BASIS
PD4005	CDH1	cna	hetloss	BASIS
PD4967	CDH1	cna	homdel	BASIS
PD5945	CDH1	cna	gain	BASIS
PD5948	CDH1	cna	hetloss	BASIS
PD7215	CDH1	cna	gain	BASIS
PD8980	CDH1	cna	hetloss	BASIS
PD9585	CDH1	cna	hetloss	BASIS
PD9702	CDH1	cna	gain	BASIS
TCGA-AN-A0XU	CNBP	cna	gain	TCGA
TCGA-BH-A0C0	CNBP	cna	gain	TCGA
TCGA-BH-A18R	CNBP	cna	gain	TCGA
TCGA-BH-A1FU	CNBP	cna	hetloss	TCGA
TCGA-C8-A12L	CNBP	cna	gain	TCGA
TCGA-E2-A1L7	CNBP	cna	hetloss	TCGA
TCGA-LL-A5YP	CNBP	cna	gain	TCGA
MB-0346	CNBP	cna	hetloss	METABRIC
MB-5070	CNBP	cna	gain	METABRIC
MB-5107	CNBP	cna	gain	METABRIC
MB-5465	CNBP	cna	gain	METABRIC
MB-6098	CNBP	cna	hetloss	METABRIC
MB-7038	CNBP	cna	gain	METABRIC
PD10014	CNBP	cna	gain	BASIS

PD11327	CNBP	cna	gain	BASIS
PD13297	CNBP	cna	hetloss	BASIS
PD13299	CNBP	cna	gain	BASIS
PD13771	CNBP	cna	hetloss	BASIS
PD23561	CNBP	cna	hetloss	BASIS
PD23574	CNBP	cna	gain	BASIS
PD24186	CNBP	cna	gain	BASIS
PD24206	CNBP	cna	gain	BASIS
PD3905	CNBP	cna	gain	BASIS
PD4005	CNBP	cna	hetloss	BASIS
PD4107	CNBP	cna	gain	BASIS
PD4826	CNBP	cna	gain	BASIS
PD5935	CNBP	cna	gain	BASIS
PD5945	CNBP	cna	gain	BASIS
PD5948	CNBP	cna	gain	BASIS
PD6731	CNBP	cna	gain	BASIS
PD7067	CNBP	cna	gain	BASIS
PD7215	CNBP	cna	gain	BASIS
PD8621	CNBP	cna	gain	BASIS
PD8980	CNBP	cna	gain	BASIS
PD9004	CNBP	cna	gain	BASIS
PD9702	CNBP	cna	gain	BASIS
TCGA-AN-A0XU	CRIPAK	cna	hetloss	TCGA
TCGA-BH-A0AW	CRIPAK	cna	hetloss	TCGA
TCGA-BH-A0C0	CRIPAK	cna	hetloss	TCGA
TCGA-BH-A1FU	CRIPAK	cna	hetloss	TCGA
TCGA-C8-A12L	CRIPAK	cna	gain	TCGA
TCGA-D8-A27M	CRIPAK	cna	amp	TCGA
TCGA-E9-A1NC	CRIPAK	cna	hetloss	TCGA
TCGA-LL-A5YP	CRIPAK	cna	hetloss	TCGA
MB-0346	CRIPAK	cna	hetloss	METABRIC
MB-2827	CRIPAK	cna	hetloss	METABRIC
MB-5070	CRIPAK	cna	gain	METABRIC
MB-5465	CRIPAK	cna	hetloss	METABRIC
MB-6060	CRIPAK	cna	hetloss	METABRIC
MB-6098	CRIPAK	cna	hetloss	METABRIC
MB-7038	CRIPAK	cna	gain	METABRIC
MTS-T0064	CRIPAK	cna	homdel	METABRIC
PD10014	CRIPAK	cna	hetloss	BASIS
PD11327	CRIPAK	cna	gain	BASIS
PD13296	CRIPAK	cna	hetloss	BASIS
PD13297	CRIPAK	cna	hetloss	BASIS
PD22355	CRIPAK	cna	hetloss	BASIS
PD24186	CRIPAK	cna	gain	BASIS
PD24202	CRIPAK	cna	hetloss	BASIS
PD24206	CRIPAK	cna	gain	BASIS
PD3890	CRIPAK	cna	hetloss	BASIS

PD3905	CRIPAK	cna	gain	BASIS
PD4005	CRIPAK	cna	hetloss	BASIS
PD4006	CRIPAK	cna	gain	BASIS
PD4826	CRIPAK	cna	hetloss	BASIS
PD5945	CRIPAK	cna	gain	BASIS
PD6406	CRIPAK	cna	hetloss	BASIS
PD6413	CRIPAK	cna	hetloss	BASIS
PD7067	CRIPAK	cna	gain	BASIS
PD8621	CRIPAK	cna	gain	BASIS
PD8980	CRIPAK	cna	hetloss	BASIS
PD9585	CRIPAK	cna	hetloss	BASIS
TCGA-A2-A25B	CRTC3	cna	gain	TCGA
TCGA-AN-A0XU	CRTC3	cna	gain	TCGA
TCGA-AO-A0JL	CRTC3	cna	hetloss	TCGA
TCGA-BH-A0AW	CRTC3	cna	hetloss	TCGA
TCGA-BH-A0C0	CRTC3	cna	gain	TCGA
TCGA-D8-A27M	CRTC3	cna	hetloss	TCGA
TCGA-E2-A1L7	CRTC3	cna	gain	TCGA
TCGA-LL-A5YP	CRTC3	cna	gain	TCGA
MB-0346	CRTC3	cna	hetloss	METABRIC
MB-5465	CRTC3	cna	hetloss	METABRIC
MB-7038	CRTC3	cna	gain	METABRIC
MB-7048	CRTC3	cna	gain	METABRIC
MB-0420	CRTC3	cna	gain	METABRIC
MTS-T0064	CRTC3	cna	amp	METABRIC
PD10014	CRTC3	cna	gain	BASIS
PD11327	CRTC3	cna	hetloss	BASIS
PD11742	CRTC3	cna	hetloss	BASIS
PD13299	CRTC3	cna	gain	BASIS
PD23562	CRTC3	cna	gain	BASIS
PD23574	CRTC3	cna	gain	BASIS
PD23578	CRTC3	cna	gain	BASIS
PD24202	CRTC3	cna	hetloss	BASIS
PD24206	CRTC3	cna	gain	BASIS
PD3890	CRTC3	cna	hetloss	BASIS
PD3905	CRTC3	cna	gain	BASIS
PD4006	CRTC3	cna	gain	BASIS
PD4107	CRTC3	cna	gain	BASIS
PD4967	CRTC3	cna	hetloss	BASIS
PD5930	CRTC3	cna	gain	BASIS
PD5945	CRTC3	cna	gain	BASIS
PD6413	CRTC3	cna	hetloss	BASIS
PD7067	CRTC3	cna	gain	BASIS
PD8621	CRTC3	cna	gain	BASIS
PD8980	CRTC3	cna	gain	BASIS
PD9585	CRTC3	cna	hetloss	BASIS
PD9702	CRTC3	cna	gain	BASIS

TCGA-AN-A0XU	CYLD	cna	hetloss	TCGA
TCGA-AO-A0JL	CYLD	cna	gain	TCGA
TCGA-BH-A0AW	CYLD	cna	hetloss	TCGA
TCGA-BH-A0C0	CYLD	cna	hetloss	TCGA
TCGA-C8-A12L	CYLD	cna	gain	TCGA
TCGA-D8-A27M	CYLD	cna	gain	TCGA
TCGA-E2-A1L7	CYLD	cna	hetloss	TCGA
TCGA-LL-A5YP	CYLD	cna	gain	TCGA
MB-0346	CYLD	cna	gain	METABRIC
MB-5070	CYLD	cna	hetloss	METABRIC
MB-5465	CYLD	cna	hetloss	METABRIC
MB-6271	CYLD	cna	hetloss	METABRIC
MB-7032	CYLD	cna	gain	METABRIC
MB-7048	CYLD	cna	hetloss	METABRIC
MB-0420	CYLD	cna	hetloss	METABRIC
MTS-T0064	CYLD	cna	hetloss	METABRIC
PD10014	CYLD	cna	hetloss	BASIS
PD11327	CYLD	cna	gain	BASIS
PD11742	CYLD	cna	hetloss	BASIS
PD13297	CYLD	cna	hetloss	BASIS
PD13299	CYLD	cna	gain	BASIS
PD14442	CYLD	cna	hetloss	BASIS
PD22355	CYLD	cna	hetloss	BASIS
PD23562	CYLD	cna	gain	BASIS
PD24186	CYLD	cna	gain	BASIS
PD24202	CYLD	cna	hetloss	BASIS
PD24206	CYLD	cna	gain	BASIS
PD24337	CYLD	cna	hetloss	BASIS
PD3905	CYLD	cna	gain	BASIS
PD4005	CYLD	cna	hetloss	BASIS
PD4967	CYLD	cna	hetloss	BASIS
PD5945	CYLD	cna	amp	BASIS
PD5948	CYLD	cna	hetloss	BASIS
PD7067	CYLD	cna	hetloss	BASIS
PD7215	CYLD	cna	gain	BASIS
PD9585	CYLD	cna	hetloss	BASIS
TCGA-AO-A0JL	D2HGDH	cna	hetloss	TCGA
TCGA-BH-A0AW	D2HGDH	cna	hetloss	TCGA
TCGA-C8-A12L	D2HGDH	cna	hetloss	TCGA
TCGA-D8-A27M	D2HGDH	cna	gain	TCGA
TCGA-E2-A1L7	D2HGDH	cna	hetloss	TCGA
TCGA-E9-A1NC	D2HGDH	cna	hetloss	TCGA
MB-5070	D2HGDH	cna	hetloss	METABRIC
MB-5107	D2HGDH	cna	hetloss	METABRIC
MB-6060	D2HGDH	cna	hetloss	METABRIC
MB-6098	D2HGDH	cna	hetloss	METABRIC
MB-6271	D2HGDH	cna	hetloss	METABRIC

MB-7048	D2HGDH	cna	hetloss	METABRIC
PD10014	D2HGDH	cna	gain	BASIS
PD11327	D2HGDH	cna	gain	BASIS
PD11742	D2HGDH	cna	hetloss	BASIS
PD13296	D2HGDH	cna	homdel	BASIS
PD13297	D2HGDH	cna	hetloss	BASIS
PD23578	D2HGDH	cna	hetloss	BASIS
PD24186	D2HGDH	cna	gain	BASIS
PD24202	D2HGDH	cna	hetloss	BASIS
PD24206	D2HGDH	cna	gain	BASIS
PD3890	D2HGDH	cna	hetloss	BASIS
PD3905	D2HGDH	cna	gain	BASIS
PD4005	D2HGDH	cna	hetloss	BASIS
PD4006	D2HGDH	cna	gain	BASIS
PD4967	D2HGDH	cna	hetloss	BASIS
PD5935	D2HGDH	cna	gain	BASIS
PD5945	D2HGDH	cna	gain	BASIS
PD5948	D2HGDH	cna	gain	BASIS
PD6406	D2HGDH	cna	hetloss	BASIS
PD6413	D2HGDH	cna	hetloss	BASIS
PD6731	D2HGDH	cna	hetloss	BASIS
PD7067	D2HGDH	cna	gain	BASIS
PD7215	D2HGDH	cna	gain	BASIS
PD8980	D2HGDH	cna	hetloss	BASIS
PD9585	D2HGDH	cna	hetloss	BASIS
TCGA-A2-A25B	DDX6	cna	hetloss	TCGA
TCGA-AO-A0JL	DDX6	cna	hetloss	TCGA
TCGA-BH-A0C0	DDX6	cna	hetloss	TCGA
TCGA-BH-A18R	DDX6	cna	homdel	TCGA
TCGA-D8-A27M	DDX6	cna	hetloss	TCGA
TCGA-E2-A1L7	DDX6	cna	hetloss	TCGA
TCGA-EW-A1OX	DDX6	cna	homdel	TCGA
TCGA-LL-A5YP	DDX6	cna	hetloss	TCGA
MB-0346	DDX6	cna	hetloss	METABRIC
MB-5070	DDX6	cna	hetloss	METABRIC
MB-5107	DDX6	cna	hetloss	METABRIC
MB-6271	DDX6	cna	hetloss	METABRIC
MB-7032	DDX6	cna	hetloss	METABRIC
MB-7048	DDX6	cna	hetloss	METABRIC
PD11742	DDX6	cna	hetloss	BASIS
PD13296	DDX6	cna	hetloss	BASIS
PD13297	DDX6	cna	hetloss	BASIS
PD13771	DDX6	cna	hetloss	BASIS
PD22355	DDX6	cna	hetloss	BASIS
PD23574	DDX6	cna	gain	BASIS
PD23578	DDX6	cna	hetloss	BASIS
PD24186	DDX6	cna	gain	BASIS

PD24206	DDX6	cna	gain	BASIS
PD24337	DDX6	cna	hetloss	BASIS
PD3905	DDX6	cna	gain	BASIS
PD4005	DDX6	cna	hetloss	BASIS
PD4826	DDX6	cna	hetloss	BASIS
PD5935	DDX6	cna	gain	BASIS
PD5945	DDX6	cna	gain	BASIS
PD5948	DDX6	cna	gain	BASIS
PD6413	DDX6	cna	hetloss	BASIS
PD6731	DDX6	cna	hetloss	BASIS
PD7067	DDX6	cna	gain	BASIS
PD7215	DDX6	cna	gain	BASIS
PD9004	DDX6	cna	hetloss	BASIS
PD9702	DDX6	cna	gain	BASIS
TCGA-AN-A0XU	DOT1L	cna	hetloss	TCGA
TCGA-AO-A0JL	DOT1L	cna	gain	TCGA
TCGA-BH-A0C0	DOT1L	cna	gain	TCGA
TCGA-BH-A1FU	DOT1L	cna	hetloss	TCGA
TCGA-C8-A12L	DOT1L	cna	hetloss	TCGA
TCGA-D8-A27M	DOT1L	cna	hetloss	TCGA
TCGA-E9-A1NC	DOT1L	cna	hetloss	TCGA
TCGA-EW-A1OX	DOT1L	cna	hetloss	TCGA
TCGA-LL-A5YP	DOT1L	cna	hetloss	TCGA
MB-0346	DOT1L	cna	gain	METABRIC
MB-2827	DOT1L	cna	hetloss	METABRIC
MB-5070	DOT1L	cna	hetloss	METABRIC
MB-6098	DOT1L	cna	hetloss	METABRIC
MB-7038	DOT1L	cna	hetloss	METABRIC
MB-0420	DOT1L	cna	hetloss	METABRIC
PD11742	DOT1L	cna	hetloss	BASIS
PD14442	DOT1L	cna	hetloss	BASIS
PD22355	DOT1L	cna	hetloss	BASIS
PD23574	DOT1L	cna	gain	BASIS
PD23578	DOT1L	cna	hetloss	BASIS
PD24202	DOT1L	cna	hetloss	BASIS
PD24206	DOT1L	cna	hetloss	BASIS
PD3890	DOT1L	cna	hetloss	BASIS
PD3905	DOT1L	cna	gain	BASIS
PD4006	DOT1L	cna	gain	BASIS
PD4826	DOT1L	cna	hetloss	BASIS
PD4967	DOT1L	cna	hetloss	BASIS
PD5930	DOT1L	cna	gain	BASIS
PD5945	DOT1L	cna	gain	BASIS
PD5948	DOT1L	cna	gain	BASIS
PD7067	DOT1L	cna	gain	BASIS
PD7215	DOT1L	cna	gain	BASIS
PD8621	DOT1L	cna	gain	BASIS

PD8980	DOT1L	cna	gain	BASIS
PD9004	DOT1L	cna	hetloss	BASIS
PD9585	DOT1L	cna	hetloss	BASIS
TCGA-A2-A25B	EIF1AX	cna	gain	TCGA
TCGA-AN-A0XU	EIF1AX	cna	hetloss	TCGA
TCGA-AO-A0JL	EIF1AX	cna	gain	TCGA
TCGA-BH-A0AW	EIF1AX	cna	gain	TCGA
TCGA-E2-A1L7	EIF1AX	cna	hetloss	TCGA
TCGA-E9-A1NC	EIF1AX	cna	hetloss	TCGA
TCGA-LL-A5YP	EIF1AX	cna	hetloss	TCGA
MB-0346	EIF1AX	cna	hetloss	METABRIC
MB-2827	EIF1AX	cna	hetloss	METABRIC
MB-5465	EIF1AX	cna	gain	METABRIC
MB-6098	EIF1AX	cna	hetloss	METABRIC
MB-0420	EIF1AX	cna	hetloss	METABRIC
PD10014	EIF1AX	cna	hetloss	BASIS
PD11327	EIF1AX	cna	hetloss	BASIS
PD11742	EIF1AX	cna	gain	BASIS
PD13296	EIF1AX	cna	amp	BASIS
PD13297	EIF1AX	cna	gain	BASIS
PD13299	EIF1AX	cna	gain	BASIS
PD13771	EIF1AX	cna	gain	BASIS
PD14442	EIF1AX	cna	gain	BASIS
PD23562	EIF1AX	cna	hetloss	BASIS
PD23574	EIF1AX	cna	gain	BASIS
PD24206	EIF1AX	cna	homdel	BASIS
PD3905	EIF1AX	cna	gain	BASIS
PD4005	EIF1AX	cna	hetloss	BASIS
PD4006	EIF1AX	cna	hetloss	BASIS
PD4826	EIF1AX	cna	gain	BASIS
PD4967	EIF1AX	cna	gain	BASIS
PD5935	EIF1AX	cna	gain	BASIS
PD6406	EIF1AX	cna	gain	BASIS
PD6413	EIF1AX	cna	gain	BASIS
PD7215	EIF1AX	cna	gain	BASIS
PD8621	EIF1AX	cna	amp	BASIS
PD8980	EIF1AX	cna	hetloss	BASIS
PD9004	EIF1AX	cna	gain	BASIS
PD9702	EIF1AX	cna	hetloss	BASIS
TCGA-A2-A25B	EIF2B1	cna	gain	TCGA
TCGA-AN-A0XU	EIF2B1	cna	hetloss	TCGA
TCGA-AO-A0JL	EIF2B1	cna	hetloss	TCGA
TCGA-BH-A0C0	EIF2B1	cna	hetloss	TCGA
TCGA-BH-A18R	EIF2B1	cna	gain	TCGA
TCGA-BH-A1FU	EIF2B1	cna	hetloss	TCGA
TCGA-D8-A27M	EIF2B1	cna	hetloss	TCGA
TCGA-E2-A1L7	EIF2B1	cna	hetloss	TCGA

TCGA-LL-A5YP	EIF2B1	cna	hetloss	TCGA
MB-0346	EIF2B1	cna	gain	METABRIC
MB-2827	EIF2B1	cna	hetloss	METABRIC
MB-5070	EIF2B1	cna	hetloss	METABRIC
MB-7048	EIF2B1	cna	hetloss	METABRIC
MTS-T0064	EIF2B1	cna	gain	METABRIC
PD11327	EIF2B1	cna	homdel	BASIS
PD13296	EIF2B1	cna	hetloss	BASIS
PD13297	EIF2B1	cna	gain	BASIS
PD13771	EIF2B1	cna	gain	BASIS
PD22355	EIF2B1	cna	hetloss	BASIS
PD23574	EIF2B1	cna	gain	BASIS
PD23578	EIF2B1	cna	hetloss	BASIS
PD3890	EIF2B1	cna	hetloss	BASIS
PD3905	EIF2B1	cna	gain	BASIS
PD4005	EIF2B1	cna	hetloss	BASIS
PD4006	EIF2B1	cna	amp	BASIS
PD4107	EIF2B1	cna	gain	BASIS
PD4826	EIF2B1	cna	gain	BASIS
PD4967	EIF2B1	cna	hetloss	BASIS
PD5935	EIF2B1	cna	gain	BASIS
PD5945	EIF2B1	cna	gain	BASIS
PD5948	EIF2B1	cna	gain	BASIS
PD6406	EIF2B1	cna	hetloss	BASIS
PD6731	EIF2B1	cna	hetloss	BASIS
PD7067	EIF2B1	cna	gain	BASIS
PD9585	EIF2B1	cna	hetloss	BASIS
PD9702	EIF2B1	cna	gain	BASIS
TCGA-A2-A25B	EP300	cna	hetloss	TCGA
TCGA-AN-A0XU	EP300	cna	hetloss	TCGA
TCGA-BH-A0AW	EP300	cna	hetloss	TCGA
TCGA-BH-A1FU	EP300	cna	gain	TCGA
TCGA-C8-A12L	EP300	cna	gain	TCGA
TCGA-D8-A27M	EP300	cna	gain	TCGA
TCGA-E9-A1NC	EP300	cna	gain	TCGA
MB-2827	EP300	cna	hetloss	METABRIC
MB-5465	EP300	cna	gain	METABRIC
MB-6098	EP300	cna	gain	METABRIC
PD11327	EP300	cna	gain	BASIS
PD11742	EP300	cna	hetloss	BASIS
PD13296	EP300	cna	gain	BASIS
PD13297	EP300	cna	hetloss	BASIS
PD13299	EP300	cna	gain	BASIS
PD14442	EP300	cna	hetloss	BASIS
PD22355	EP300	cna	gain	BASIS
PD23562	EP300	cna	gain	BASIS
PD23578	EP300	cna	gain	BASIS

PD24202	EP300	cna	hetloss	BASIS
PD24206	EP300	cna	homdel	BASIS
PD3905	EP300	cna	gain	BASIS
PD4005	EP300	cna	gain	BASIS
PD4006	EP300	cna	gain	BASIS
PD4107	EP300	cna	gain	BASIS
PD4826	EP300	cna	gain	BASIS
PD4967	EP300	cna	hetloss	BASIS
PD5935	EP300	cna	gain	BASIS
PD5945	EP300	cna	amp	BASIS
PD5948	EP300	cna	gain	BASIS
PD6731	EP300	cna	hetloss	BASIS
PD7067	EP300	cna	gain	BASIS
PD7215	EP300	cna	gain	BASIS
PD8621	EP300	cna	gain	BASIS
PD9004	EP300	cna	gain	BASIS
PD9702	EP300	cna	gain	BASIS
TCGA-A2-A25B	EPHA2	cna	hetloss	TCGA
TCGA-AN-A0XU	EPHA2	cna	hetloss	TCGA
TCGA-AO-A0JL	EPHA2	cna	hetloss	TCGA
TCGA-BH-A0AW	EPHA2	cna	gain	TCGA
TCGA-BH-A0C0	EPHA2	cna	hetloss	TCGA
TCGA-BH-A18R	EPHA2	cna	homdel	TCGA
TCGA-BH-A1FU	EPHA2	cna	gain	TCGA
TCGA-C8-A12L	EPHA2	cna	hetloss	TCGA
TCGA-D8-A27M	EPHA2	cna	gain	TCGA
TCGA-E2-A1L7	EPHA2	cna	hetloss	TCGA
TCGA-E9-A1NC	EPHA2	cna	hetloss	TCGA
TCGA-LL-A5YP	EPHA2	cna	hetloss	TCGA
MB-2827	EPHA2	cna	hetloss	METABRIC
MB-5070	EPHA2	cna	hetloss	METABRIC
MB-5465	EPHA2	cna	hetloss	METABRIC
MB-6060	EPHA2	cna	hetloss	METABRIC
MB-6098	EPHA2	cna	hetloss	METABRIC
MB-0420	EPHA2	cna	hetloss	METABRIC
PD10014	EPHA2	cna	gain	BASIS
PD11327	EPHA2	cna	hetloss	BASIS
PD11742	EPHA2	cna	hetloss	BASIS
PD13296	EPHA2	cna	hetloss	BASIS
PD13771	EPHA2	cna	hetloss	BASIS
PD22355	EPHA2	cna	hetloss	BASIS
PD24202	EPHA2	cna	hetloss	BASIS
PD24206	EPHA2	cna	gain	BASIS
PD24337	EPHA2	cna	hetloss	BASIS
PD3890	EPHA2	cna	hetloss	BASIS
PD3905	EPHA2	cna	gain	BASIS
PD4826	EPHA2	cna	gain	BASIS

PD5945	EPHA2	cna	gain	BASIS
PD6406	EPHA2	cna	hetloss	BASIS
PD7067	EPHA2	cna	gain	BASIS
PD7215	EPHA2	cna	gain	BASIS
PD9004	EPHA2	cna	hetloss	BASIS
PD9585	EPHA2	cna	hetloss	BASIS
TCGA-A2-A25B	EPHB2	cna	hetloss	TCGA
TCGA-AN-A0XU	EPHB2	cna	hetloss	TCGA
TCGA-AO-A0JL	EPHB2	cna	hetloss	TCGA
TCGA-BH-A0AW	EPHB2	cna	gain	TCGA
TCGA-BH-A0C0	EPHB2	cna	hetloss	TCGA
TCGA-BH-A18R	EPHB2	cna	homdel	TCGA
TCGA-BH-A1FU	EPHB2	cna	gain	TCGA
TCGA-C8-A12L	EPHB2	cna	hetloss	TCGA
TCGA-D8-A27M	EPHB2	cna	gain	TCGA
TCGA-E2-A1L7	EPHB2	cna	hetloss	TCGA
TCGA-E9-A1NC	EPHB2	cna	hetloss	TCGA
TCGA-LL-A5YP	EPHB2	cna	hetloss	TCGA
MB-2827	EPHB2	cna	hetloss	METABRIC
MB-5070	EPHB2	cna	hetloss	METABRIC
MB-5465	EPHB2	cna	hetloss	METABRIC
MB-6060	EPHB2	cna	hetloss	METABRIC
MB-6098	EPHB2	cna	hetloss	METABRIC
MB-6271	EPHB2	cna	hetloss	METABRIC
PD10014	EPHB2	cna	gain	BASIS
PD11742	EPHB2	cna	hetloss	BASIS
PD13296	EPHB2	cna	hetloss	BASIS
PD13771	EPHB2	cna	hetloss	BASIS
PD22355	EPHB2	cna	hetloss	BASIS
PD24186	EPHB2	cna	gain	BASIS
PD24202	EPHB2	cna	hetloss	BASIS
PD24206	EPHB2	cna	gain	BASIS
PD24337	EPHB2	cna	hetloss	BASIS
PD3890	EPHB2	cna	hetloss	BASIS
PD3905	EPHB2	cna	gain	BASIS
PD4006	EPHB2	cna	gain	BASIS
PD4826	EPHB2	cna	gain	BASIS
PD5945	EPHB2	cna	amp	BASIS
PD7067	EPHB2	cna	gain	BASIS
PD7215	EPHB2	cna	gain	BASIS
PD9004	EPHB2	cna	hetloss	BASIS
PD9585	EPHB2	cna	hetloss	BASIS
TCGA-A2-A25B	FANCI	cna	gain	TCGA
TCGA-AN-A0XU	FANCI	cna	gain	TCGA
TCGA-AO-A0JL	FANCI	cna	hetloss	TCGA
TCGA-BH-A0AW	FANCI	cna	hetloss	TCGA
TCGA-BH-A0C0	FANCI	cna	gain	TCGA

TCGA-D8-A27M	FANCI	cna	hetloss	TCGA
TCGA-E2-A1L7	FANCI	cna	gain	TCGA
TCGA-LL-A5YP	FANCI	cna	gain	TCGA
MB-0346	FANCI	cna	hetloss	METABRIC
MB-5465	FANCI	cna	hetloss	METABRIC
MB-7038	FANCI	cna	gain	METABRIC
MB-7048	FANCI	cna	amp	METABRIC
MB-0420	FANCI	cna	gain	METABRIC
MTS-T0064	FANCI	cna	amp	METABRIC
PD10014	FANCI	cna	gain	BASIS
PD11742	FANCI	cna	hetloss	BASIS
PD13299	FANCI	cna	gain	BASIS
PD23562	FANCI	cna	gain	BASIS
PD23574	FANCI	cna	gain	BASIS
PD23578	FANCI	cna	gain	BASIS
PD24202	FANCI	cna	hetloss	BASIS
PD24206	FANCI	cna	gain	BASIS
PD24337	FANCI	cna	gain	BASIS
PD3890	FANCI	cna	hetloss	BASIS
PD3905	FANCI	cna	gain	BASIS
PD4006	FANCI	cna	gain	BASIS
PD4107	FANCI	cna	gain	BASIS
PD4967	FANCI	cna	hetloss	BASIS
PD5930	FANCI	cna	gain	BASIS
PD5945	FANCI	cna	gain	BASIS
PD6413	FANCI	cna	hetloss	BASIS
PD7067	FANCI	cna	gain	BASIS
PD8621	FANCI	cna	gain	BASIS
PD8980	FANCI	cna	gain	BASIS
PD9585	FANCI	cna	hetloss	BASIS
PD9702	FANCI	cna	gain	BASIS
TCGA-A2-A25B	FGF19	cna	hetloss	TCGA
TCGA-AN-A0XU	FGF19	cna	hetloss	TCGA
TCGA-AO-A0JL	FGF19	cna	hetloss	TCGA
TCGA-BH-A18R	FGF19	cna	amp	TCGA
TCGA-C8-A12L	FGF19	cna	amp	TCGA
TCGA-EW-A1OX	FGF19	cna	amp	TCGA
TCGA-LL-A5YP	FGF19	cna	hetloss	TCGA
MB-0346	FGF19	cna	gain	METABRIC
MB-5107	FGF19	cna	gain	METABRIC
MB-6060	FGF19	cna	amp	METABRIC
MB-6271	FGF19	cna	amp	METABRIC
MTS-T0064	FGF19	cna	gain	METABRIC
P-0002591-T01-IM3	FGF19	cna	amp	MSK-IMPACT
P-0002858-T01-IM3	FGF19	cna	amp	MSK-IMPACT
PD10014	FGF19	cna	gain	BASIS
PD11327	FGF19	cna	gain	BASIS

PD11742	FGF19	cna	gain	BASIS
PD13771	FGF19	cna	amp	BASIS
PD23574	FGF19	cna	gain	BASIS
PD24186	FGF19	cna	amp	BASIS
PD24206	FGF19	cna	gain	BASIS
PD3905	FGF19	cna	gain	BASIS
PD4005	FGF19	cna	gain	BASIS
PD4006	FGF19	cna	amp	BASIS
PD4107	FGF19	cna	gain	BASIS
PD4826	FGF19	cna	gain	BASIS
PD5930	FGF19	cna	gain	BASIS
PD5935	FGF19	cna	gain	BASIS
PD5945	FGF19	cna	amp	BASIS
PD5948	FGF19	cna	amp	BASIS
PD6413	FGF19	cna	gain	BASIS
PD7067	FGF19	cna	gain	BASIS
PD7215	FGF19	cna	gain	BASIS
PD8980	FGF19	cna	gain	BASIS
PD9004	FGF19	cna	gain	BASIS
PD9702	FGF19	cna	gain	BASIS
TCGA-A2-A25B	FGF3	cna	hetloss	TCGA
TCGA-AN-A0XU	FGF3	cna	hetloss	TCGA
TCGA-AO-A0JL	FGF3	cna	hetloss	TCGA
TCGA-BH-A18R	FGF3	cna	amp	TCGA
TCGA-C8-A12L	FGF3	cna	amp	TCGA
TCGA-EW-A1OX	FGF3	cna	amp	TCGA
TCGA-LL-A5YP	FGF3	cna	hetloss	TCGA
MB-0346	FGF3	cna	gain	METABRIC
MB-5107	FGF3	cna	gain	METABRIC
MB-6060	FGF3	cna	amp	METABRIC
MB-6271	FGF3	cna	amp	METABRIC
MTS-T0064	FGF3	cna	gain	METABRIC
P-0002591-T01-IM3	FGF3	cna	amp	MSK-IMPACT
P-0002858-T01-IM3	FGF3	cna	amp	MSK-IMPACT
PD10014	FGF3	cna	gain	BASIS
PD11327	FGF3	cna	gain	BASIS
PD11742	FGF3	cna	gain	BASIS
PD13771	FGF3	cna	amp	BASIS
PD23574	FGF3	cna	gain	BASIS
PD24186	FGF3	cna	amp	BASIS
PD24206	FGF3	cna	gain	BASIS
PD3905	FGF3	cna	gain	BASIS
PD4005	FGF3	cna	gain	BASIS
PD4006	FGF3	cna	amp	BASIS
PD4107	FGF3	cna	gain	BASIS
PD4826	FGF3	cna	gain	BASIS
PD5930	FGF3	cna	gain	BASIS

PD5935	FGF3	cna	gain	BASIS
PD5945	FGF3	cna	amp	BASIS
PD5948	FGF3	cna	amp	BASIS
PD6413	FGF3	cna	gain	BASIS
PD7067	FGF3	cna	gain	BASIS
PD7215	FGF3	cna	gain	BASIS
PD8980	FGF3	cna	gain	BASIS
PD9004	FGF3	cna	gain	BASIS
PD9702	FGF3	cna	gain	BASIS
TCGA-A2-A25B	FGF4	cna	hetloss	TCGA
TCGA-AN-A0XU	FGF4	cna	hetloss	TCGA
TCGA-AO-A0JL	FGF4	cna	hetloss	TCGA
TCGA-BH-A18R	FGF4	cna	amp	TCGA
TCGA-C8-A12L	FGF4	cna	amp	TCGA
TCGA-EW-A1OX	FGF4	cna	amp	TCGA
TCGA-LL-A5YP	FGF4	cna	hetloss	TCGA
MB-0346	FGF4	cna	gain	METABRIC
MB-5107	FGF4	cna	gain	METABRIC
MB-6060	FGF4	cna	amp	METABRIC
MB-6271	FGF4	cna	amp	METABRIC
MTS-T0064	FGF4	cna	gain	METABRIC
P-0002591-T01-IM3	FGF4	cna	amp	MSK-IMPACT
P-0002858-T01-IM3	FGF4	cna	amp	MSK-IMPACT
PD10014	FGF4	cna	gain	BASIS
PD11327	FGF4	cna	gain	BASIS
PD11742	FGF4	cna	gain	BASIS
PD13771	FGF4	cna	amp	BASIS
PD23574	FGF4	cna	gain	BASIS
PD24186	FGF4	cna	amp	BASIS
PD24206	FGF4	cna	gain	BASIS
PD3905	FGF4	cna	gain	BASIS
PD4005	FGF4	cna	gain	BASIS
PD4006	FGF4	cna	amp	BASIS
PD4107	FGF4	cna	gain	BASIS
PD4826	FGF4	cna	gain	BASIS
PD5930	FGF4	cna	gain	BASIS
PD5935	FGF4	cna	gain	BASIS
PD5945	FGF4	cna	amp	BASIS
PD5948	FGF4	cna	amp	BASIS
PD6413	FGF4	cna	gain	BASIS
PD7067	FGF4	cna	gain	BASIS
PD7215	FGF4	cna	gain	BASIS
PD8980	FGF4	cna	gain	BASIS
PD9004	FGF4	cna	gain	BASIS
PD9702	FGF4	cna	gain	BASIS
TCGA-AN-A0XU	FGFR3	cna	hetloss	TCGA
TCGA-BH-A0AW	FGFR3	cna	hetloss	TCGA

TCGA-BH-A0C0	FGFR3	cna	hetloss	TCGA
TCGA-BH-A1FU	FGFR3	cna	hetloss	TCGA
TCGA-C8-A12L	FGFR3	cna	gain	TCGA
TCGA-D8-A27M	FGFR3	cna	amp	TCGA
TCGA-E9-A1NC	FGFR3	cna	hetloss	TCGA
TCGA-LL-A5YP	FGFR3	cna	hetloss	TCGA
MB-0346	FGFR3	cna	hetloss	METABRIC
MB-2827	FGFR3	cna	hetloss	METABRIC
MB-5070	FGFR3	cna	gain	METABRIC
MB-5465	FGFR3	cna	hetloss	METABRIC
MB-6060	FGFR3	cna	hetloss	METABRIC
MB-6098	FGFR3	cna	hetloss	METABRIC
MB-7038	FGFR3	cna	gain	METABRIC
MTS-T0064	FGFR3	cna	homdel	METABRIC
P-0009557-T01-IM5	FGFR3	cna	homdel	MSK-IMPACT
PD10014	FGFR3	cna	hetloss	BASIS
PD11327	FGFR3	cna	gain	BASIS
PD13296	FGFR3	cna	hetloss	BASIS
PD13297	FGFR3	cna	hetloss	BASIS
PD24186	FGFR3	cna	gain	BASIS
PD24202	FGFR3	cna	hetloss	BASIS
PD24206	FGFR3	cna	gain	BASIS
PD3890	FGFR3	cna	hetloss	BASIS
PD3905	FGFR3	cna	gain	BASIS
PD4005	FGFR3	cna	hetloss	BASIS
PD4006	FGFR3	cna	gain	BASIS
PD4826	FGFR3	cna	hetloss	BASIS
PD5945	FGFR3	cna	gain	BASIS
PD6406	FGFR3	cna	hetloss	BASIS
PD6413	FGFR3	cna	hetloss	BASIS
PD7067	FGFR3	cna	gain	BASIS
PD8621	FGFR3	cna	gain	BASIS
PD8980	FGFR3	cna	hetloss	BASIS
PD9585	FGFR3	cna	hetloss	BASIS
TCGA-A2-A25B	FNBP1	cna	gain	TCGA
TCGA-AN-A0XU	FNBP1	cna	hetloss	TCGA
TCGA-AO-A0JL	FNBP1	cna	hetloss	TCGA
TCGA-BH-A0C0	FNBP1	cna	gain	TCGA
TCGA-C8-A12L	FNBP1	cna	gain	TCGA
TCGA-E2-A1L7	FNBP1	cna	gain	TCGA
TCGA-E9-A1NC	FNBP1	cna	hetloss	TCGA
TCGA-LL-A5YP	FNBP1	cna	hetloss	TCGA
MB-0346	FNBP1	cna	hetloss	METABRIC
MB-2827	FNBP1	cna	hetloss	METABRIC
MB-5070	FNBP1	cna	hetloss	METABRIC
MB-5107	FNBP1	cna	gain	METABRIC
MB-7038	FNBP1	cna	hetloss	METABRIC

MB-0420	FNBP1	cna	hetloss	METABRIC
PD10014	FNBP1	cna	gain	BASIS
PD11327	FNBP1	cna	hetloss	BASIS
PD13299	FNBP1	cna	gain	BASIS
PD22355	FNBP1	cna	gain	BASIS
PD23578	FNBP1	cna	hetloss	BASIS
PD24186	FNBP1	cna	gain	BASIS
PD24202	FNBP1	cna	hetloss	BASIS
PD24337	FNBP1	cna	hetloss	BASIS
PD3890	FNBP1	cna	hetloss	BASIS
PD3905	FNBP1	cna	gain	BASIS
PD4005	FNBP1	cna	hetloss	BASIS
PD4006	FNBP1	cna	gain	BASIS
PD4107	FNBP1	cna	hetloss	BASIS
PD5930	FNBP1	cna	hetloss	BASIS
PD5945	FNBP1	cna	gain	BASIS
PD5948	FNBP1	cna	gain	BASIS
PD6406	FNBP1	cna	hetloss	BASIS
PD6731	FNBP1	cna	hetloss	BASIS
PD7067	FNBP1	cna	gain	BASIS
PD7215	FNBP1	cna	gain	BASIS
PD8621	FNBP1	cna	gain	BASIS
PD9004	FNBP1	cna	hetloss	BASIS
TCGA-A2-A25B	FUBP1	cna	gain	TCGA
TCGA-AN-A0XU	FUBP1	cna	hetloss	TCGA
TCGA-BH-A0AW	FUBP1	cna	gain	TCGA
TCGA-BH-A0C0	FUBP1	cna	hetloss	TCGA
TCGA-BH-A1FU	FUBP1	cna	gain	TCGA
TCGA-C8-A12L	FUBP1	cna	hetloss	TCGA
TCGA-D8-A27M	FUBP1	cna	hetloss	TCGA
TCGA-E2-A1L7	FUBP1	cna	amp	TCGA
TCGA-LL-A5YP	FUBP1	cna	hetloss	TCGA
MB-0346	FUBP1	cna	hetloss	METABRIC
MB-6060	FUBP1	cna	hetloss	METABRIC
MB-6098	FUBP1	cna	hetloss	METABRIC
MB-7038	FUBP1	cna	gain	METABRIC
PD11327	FUBP1	cna	gain	BASIS
PD11742	FUBP1	cna	hetloss	BASIS
PD13296	FUBP1	cna	hetloss	BASIS
PD13299	FUBP1	cna	gain	BASIS
PD22355	FUBP1	cna	hetloss	BASIS
PD23574	FUBP1	cna	gain	BASIS
PD24186	FUBP1	cna	gain	BASIS
PD24206	FUBP1	cna	gain	BASIS
PD3890	FUBP1	cna	gain	BASIS
PD3905	FUBP1	cna	gain	BASIS
PD4006	FUBP1	cna	hetloss	BASIS

PD4107	FUBP1	cna	gain	BASIS
PD4826	FUBP1	cna	gain	BASIS
PD5930	FUBP1	cna	gain	BASIS
PD5935	FUBP1	cna	gain	BASIS
PD5945	FUBP1	cna	gain	BASIS
PD5948	FUBP1	cna	gain	BASIS
PD7067	FUBP1	cna	gain	BASIS
PD7215	FUBP1	cna	gain	BASIS
PD8621	FUBP1	cna	gain	BASIS
PD9004	FUBP1	cna	gain	BASIS
PD9585	FUBP1	cna	hetloss	BASIS
PD9702	FUBP1	cna	gain	BASIS
TCGA-A2-A25B	FZD10	cna	gain	TCGA
TCGA-AN-A0XU	FZD10	cna	hetloss	TCGA
TCGA-AO-A0JL	FZD10	cna	hetloss	TCGA
TCGA-BH-A0C0	FZD10	cna	hetloss	TCGA
TCGA-BH-A18R	FZD10	cna	gain	TCGA
TCGA-BH-A1FU	FZD10	cna	hetloss	TCGA
TCGA-D8-A27M	FZD10	cna	hetloss	TCGA
TCGA-E2-A1L7	FZD10	cna	hetloss	TCGA
TCGA-E9-A1NC	FZD10	cna	hetloss	TCGA
TCGA-LL-A5YP	FZD10	cna	hetloss	TCGA
MB-0346	FZD10	cna	gain	METABRIC
MB-2827	FZD10	cna	hetloss	METABRIC
MB-5070	FZD10	cna	hetloss	METABRIC
MB-7048	FZD10	cna	hetloss	METABRIC
MTS-T0064	FZD10	cna	gain	METABRIC
PD11327	FZD10	cna	gain	BASIS
PD13296	FZD10	cna	hetloss	BASIS
PD13297	FZD10	cna	hetloss	BASIS
PD13771	FZD10	cna	gain	BASIS
PD22355	FZD10	cna	hetloss	BASIS
PD23574	FZD10	cna	gain	BASIS
PD23578	FZD10	cna	hetloss	BASIS
PD24186	FZD10	cna	gain	BASIS
PD3890	FZD10	cna	hetloss	BASIS
PD3905	FZD10	cna	gain	BASIS
PD4005	FZD10	cna	hetloss	BASIS
PD4006	FZD10	cna	amp	BASIS
PD4107	FZD10	cna	gain	BASIS
PD4826	FZD10	cna	gain	BASIS
PD5935	FZD10	cna	gain	BASIS
PD5945	FZD10	cna	homdel	BASIS
PD5948	FZD10	cna	gain	BASIS
PD6731	FZD10	cna	hetloss	BASIS
PD7067	FZD10	cna	gain	BASIS
PD9585	FZD10	cna	hetloss	BASIS

PD9702	FZD10	cna	gain	BASIS
TCGA-AN-A0XU	GADD45B	cna	hetloss	TCGA
TCGA-AO-A0JL	GADD45B	cna	gain	TCGA
TCGA-BH-A0C0	GADD45B	cna	gain	TCGA
TCGA-BH-A1FU	GADD45B	cna	hetloss	TCGA
TCGA-C8-A12L	GADD45B	cna	gain	TCGA
TCGA-D8-A27M	GADD45B	cna	hetloss	TCGA
TCGA-E9-A1NC	GADD45B	cna	hetloss	TCGA
TCGA-EW-A1OX	GADD45B	cna	hetloss	TCGA
TCGA-LL-A5YP	GADD45B	cna	hetloss	TCGA
MB-0346	GADD45B	cna	gain	METABRIC
MB-2827	GADD45B	cna	hetloss	METABRIC
MB-5070	GADD45B	cna	hetloss	METABRIC
MB-6098	GADD45B	cna	hetloss	METABRIC
MB-7038	GADD45B	cna	hetloss	METABRIC
MB-0420	GADD45B	cna	hetloss	METABRIC
PD11742	GADD45B	cna	hetloss	BASIS
PD14442	GADD45B	cna	hetloss	BASIS
PD22355	GADD45B	cna	hetloss	BASIS
PD23574	GADD45B	cna	gain	BASIS
PD23578	GADD45B	cna	hetloss	BASIS
PD24202	GADD45B	cna	hetloss	BASIS
PD24206	GADD45B	cna	hetloss	BASIS
PD3890	GADD45B	cna	hetloss	BASIS
PD3905	GADD45B	cna	gain	BASIS
PD4006	GADD45B	cna	gain	BASIS
PD4826	GADD45B	cna	hetloss	BASIS
PD4967	GADD45B	cna	hetloss	BASIS
PD5930	GADD45B	cna	gain	BASIS
PD5945	GADD45B	cna	gain	BASIS
PD5948	GADD45B	cna	gain	BASIS
PD7067	GADD45B	cna	gain	BASIS
PD7215	GADD45B	cna	gain	BASIS
PD8621	GADD45B	cna	gain	BASIS
PD8980	GADD45B	cna	gain	BASIS
PD9004	GADD45B	cna	hetloss	BASIS
PD9585	GADD45B	cna	hetloss	BASIS
TCGA-AN-A0XU	GNA11	cna	hetloss	TCGA
TCGA-AO-A0JL	GNA11	cna	gain	TCGA
TCGA-BH-A0C0	GNA11	cna	gain	TCGA
TCGA-BH-A1FU	GNA11	cna	hetloss	TCGA
TCGA-C8-A12L	GNA11	cna	amp	TCGA
TCGA-D8-A27M	GNA11	cna	hetloss	TCGA
TCGA-E9-A1NC	GNA11	cna	hetloss	TCGA
TCGA-EW-A1OX	GNA11	cna	hetloss	TCGA
TCGA-LL-A5YP	GNA11	cna	hetloss	TCGA
MB-0346	GNA11	cna	gain	METABRIC

MB-2827	GNA11	cna	hetloss	METABRIC
MB-5070	GNA11	cna	hetloss	METABRIC
MB-6098	GNA11	cna	hetloss	METABRIC
MB-7038	GNA11	cna	hetloss	METABRIC
MB-0420	GNA11	cna	hetloss	METABRIC
PD11742	GNA11	cna	hetloss	BASIS
PD13296	GNA11	cna	gain	BASIS
PD13299	GNA11	cna	gain	BASIS
PD14442	GNA11	cna	hetloss	BASIS
PD22355	GNA11	cna	hetloss	BASIS
PD23574	GNA11	cna	gain	BASIS
PD23578	GNA11	cna	hetloss	BASIS
PD24202	GNA11	cna	hetloss	BASIS
PD24206	GNA11	cna	hetloss	BASIS
PD3905	GNA11	cna	gain	BASIS
PD4006	GNA11	cna	gain	BASIS
PD4826	GNA11	cna	gain	BASIS
PD4967	GNA11	cna	hetloss	BASIS
PD5930	GNA11	cna	gain	BASIS
PD5945	GNA11	cna	gain	BASIS
PD5948	GNA11	cna	gain	BASIS
PD7067	GNA11	cna	gain	BASIS
PD8621	GNA11	cna	gain	BASIS
PD8980	GNA11	cna	gain	BASIS
PD9004	GNA11	cna	hetloss	BASIS
PD9585	GNA11	cna	hetloss	BASIS
TCGA-A2-A25B	GPHN	cna	hetloss	TCGA
TCGA-AO-A0JL	GPHN	cna	hetloss	TCGA
TCGA-BH-A0AW	GPHN	cna	hetloss	TCGA
TCGA-BH-A1FU	GPHN	cna	gain	TCGA
TCGA-C8-A12L	GPHN	cna	hetloss	TCGA
TCGA-D8-A27M	GPHN	cna	hetloss	TCGA
TCGA-E2-A1L7	GPHN	cna	hetloss	TCGA
TCGA-LL-A5YP	GPHN	cna	hetloss	TCGA
MB-2827	GPHN	cna	hetloss	METABRIC
MB-5070	GPHN	cna	hetloss	METABRIC
MB-5465	GPHN	cna	gain	METABRIC
MB-6098	GPHN	cna	hetloss	METABRIC
MB-7038	GPHN	cna	hetloss	METABRIC
PD10014	GPHN	cna	hetloss	BASIS
PD11327	GPHN	cna	amp	BASIS
PD11742	GPHN	cna	hetloss	BASIS
PD13296	GPHN	cna	gain	BASIS
PD13299	GPHN	cna	hetloss	BASIS
PD13771	GPHN	cna	hetloss	BASIS
PD14442	GPHN	cna	hetloss	BASIS
PD22355	GPHN	cna	hetloss	BASIS

PD23578	GPHN	cna	hetloss	BASIS
PD24202	GPHN	cna	hetloss	BASIS
PD24206	GPHN	cna	gain	BASIS
PD24337	GPHN	cna	hetloss	BASIS
PD3890	GPHN	cna	hetloss	BASIS
PD3905	GPHN	cna	gain	BASIS
PD4005	GPHN	cna	hetloss	BASIS
PD4006	GPHN	cna	gain	BASIS
PD5945	GPHN	cna	gain	BASIS
PD6406	GPHN	cna	hetloss	BASIS
PD7067	GPHN	cna	gain	BASIS
PD7215	GPHN	cna	hetloss	BASIS
PD8980	GPHN	cna	gain	BASIS
PD9004	GPHN	cna	hetloss	BASIS
PD9702	GPHN	cna	gain	BASIS
TCGA-A2-A25B	GPS2	cna	hetloss	TCGA
TCGA-AN-A0XU	GPS2	cna	hetloss	TCGA
TCGA-AO-A0JL	GPS2	cna	gain	TCGA
TCGA-BH-A0AW	GPS2	cna	hetloss	TCGA
TCGA-BH-A0C0	GPS2	cna	hetloss	TCGA
TCGA-C8-A12L	GPS2	cna	hetloss	TCGA
TCGA-D8-A27M	GPS2	cna	hetloss	TCGA
TCGA-E2-A1L7	GPS2	cna	hetloss	TCGA
TCGA-E9-A1NC	GPS2	cna	hetloss	TCGA
TCGA-LL-A5YP	GPS2	cna	gain	TCGA
MB-0346	GPS2	cna	hetloss	METABRIC
MB-2827	GPS2	cna	hetloss	METABRIC
MB-5070	GPS2	cna	hetloss	METABRIC
MB-5107	GPS2	cna	hetloss	METABRIC
MB-6271	GPS2	cna	hetloss	METABRIC
MB-7048	GPS2	cna	hetloss	METABRIC
MB-0420	GPS2	cna	hetloss	METABRIC
PD10014	GPS2	cna	hetloss	BASIS
PD11742	GPS2	cna	hetloss	BASIS
PD13296	GPS2	cna	hetloss	BASIS
PD13771	GPS2	cna	hetloss	BASIS
PD22355	GPS2	cna	hetloss	BASIS
PD24202	GPS2	cna	hetloss	BASIS
PD24206	GPS2	cna	hetloss	BASIS
PD24337	GPS2	cna	hetloss	BASIS
PD3890	GPS2	cna	hetloss	BASIS
PD3905	GPS2	cna	gain	BASIS
PD4005	GPS2	cna	gain	BASIS
PD4826	GPS2	cna	hetloss	BASIS
PD4967	GPS2	cna	hetloss	BASIS
PD5945	GPS2	cna	gain	BASIS
PD6406	GPS2	cna	hetloss	BASIS

PD6413	GPS2	cna	hetloss	BASIS
PD6731	GPS2	cna	hetloss	BASIS
PD7067	GPS2	cna	gain	BASIS
PD9585	GPS2	cna	hetloss	BASIS
TCGA-A2-A25B	HDAC1	cna	hetloss	TCGA
TCGA-AN-A0XU	HDAC1	cna	hetloss	TCGA
TCGA-AO-A0JL	HDAC1	cna	hetloss	TCGA
TCGA-BH-A0AW	HDAC1	cna	gain	TCGA
TCGA-BH-A0C0	HDAC1	cna	hetloss	TCGA
TCGA-BH-A18R	HDAC1	cna	homdel	TCGA
TCGA-BH-A1FU	HDAC1	cna	gain	TCGA
TCGA-C8-A12L	HDAC1	cna	hetloss	TCGA
TCGA-D8-A27M	HDAC1	cna	gain	TCGA
TCGA-E2-A1L7	HDAC1	cna	gain	TCGA
TCGA-LL-A5YP	HDAC1	cna	gain	TCGA
MB-0346	HDAC1	cna	hetloss	METABRIC
MB-2827	HDAC1	cna	hetloss	METABRIC
MB-5070	HDAC1	cna	hetloss	METABRIC
MB-5465	HDAC1	cna	hetloss	METABRIC
MB-6060	HDAC1	cna	hetloss	METABRIC
MB-6098	HDAC1	cna	hetloss	METABRIC
MB-0420	HDAC1	cna	hetloss	METABRIC
PD10014	HDAC1	cna	gain	BASIS
PD11327	HDAC1	cna	hetloss	BASIS
PD11742	HDAC1	cna	hetloss	BASIS
PD13296	HDAC1	cna	hetloss	BASIS
PD24206	HDAC1	cna	gain	BASIS
PD24337	HDAC1	cna	hetloss	BASIS
PD3890	HDAC1	cna	hetloss	BASIS
PD3905	HDAC1	cna	gain	BASIS
PD4006	HDAC1	cna	gain	BASIS
PD4826	HDAC1	cna	gain	BASIS
PD5945	HDAC1	cna	amp	BASIS
PD5948	HDAC1	cna	hetloss	BASIS
PD7067	HDAC1	cna	gain	BASIS
PD7215	HDAC1	cna	gain	BASIS
PD8980	HDAC1	cna	gain	BASIS
PD9004	HDAC1	cna	hetloss	BASIS
PD9585	HDAC1	cna	hetloss	BASIS
PD9702	HDAC1	cna	gain	BASIS
TCGA-A2-A25B	HDAC7	cna	gain	TCGA
TCGA-AN-A0XU	HDAC7	cna	gain	TCGA
TCGA-AO-A0JL	HDAC7	cna	hetloss	TCGA
TCGA-BH-A0C0	HDAC7	cna	hetloss	TCGA
TCGA-BH-A18R	HDAC7	cna	gain	TCGA
TCGA-BH-A1FU	HDAC7	cna	hetloss	TCGA
TCGA-C8-A12L	HDAC7	cna	gain	TCGA

TCGA-D8-A27M	HDAC7	cna	hetloss	TCGA
TCGA-E2-A1L7	HDAC7	cna	hetloss	TCGA
TCGA-E9-A1NC	HDAC7	cna	hetloss	TCGA
TCGA-LL-A5YP	HDAC7	cna	hetloss	TCGA
MB-0346	HDAC7	cna	gain	METABRIC
MB-2827	HDAC7	cna	hetloss	METABRIC
MB-5070	HDAC7	cna	hetloss	METABRIC
MB-6060	HDAC7	cna	gain	METABRIC
MB-6098	HDAC7	cna	hetloss	METABRIC
MB-7038	HDAC7	cna	hetloss	METABRIC
MB-0420	HDAC7	cna	hetloss	METABRIC
PD11327	HDAC7	cna	amp	BASIS
PD13296	HDAC7	cna	hetloss	BASIS
PD13297	HDAC7	cna	hetloss	BASIS
PD23574	HDAC7	cna	gain	BASIS
PD23578	HDAC7	cna	hetloss	BASIS
PD24202	HDAC7	cna	hetloss	BASIS
PD24206	HDAC7	cna	gain	BASIS
PD24337	HDAC7	cna	hetloss	BASIS
PD3890	HDAC7	cna	hetloss	BASIS
PD3905	HDAC7	cna	gain	BASIS
PD4107	HDAC7	cna	hetloss	BASIS
PD4826	HDAC7	cna	gain	BASIS
PD5945	HDAC7	cna	gain	BASIS
PD6731	HDAC7	cna	hetloss	BASIS
PD7067	HDAC7	cna	gain	BASIS
PD8621	HDAC7	cna	hetloss	BASIS
PD9585	HDAC7	cna	hetloss	BASIS
PD9702	HDAC7	cna	gain	BASIS
TCGA-A2-A25B	ICOSLG	cna	hetloss	TCGA
TCGA-AN-A0XU	ICOSLG	cna	hetloss	TCGA
TCGA-BH-A0AW	ICOSLG	cna	gain	TCGA
TCGA-BH-A0C0	ICOSLG	cna	hetloss	TCGA
TCGA-BH-A1FU	ICOSLG	cna	gain	TCGA
TCGA-C8-A12L	ICOSLG	cna	hetloss	TCGA
TCGA-D8-A27M	ICOSLG	cna	hetloss	TCGA
TCGA-LL-A5YP	ICOSLG	cna	gain	TCGA
MB-2827	ICOSLG	cna	amp	METABRIC
MB-6098	ICOSLG	cna	hetloss	METABRIC
MB-7048	ICOSLG	cna	hetloss	METABRIC
PD10014	ICOSLG	cna	hetloss	BASIS
PD11327	ICOSLG	cna	gain	BASIS
PD13296	ICOSLG	cna	gain	BASIS
PD13297	ICOSLG	cna	hetloss	BASIS
PD13299	ICOSLG	cna	gain	BASIS
PD13771	ICOSLG	cna	gain	BASIS
PD23562	ICOSLG	cna	gain	BASIS

PD23574	ICOSLG	cna	gain	BASIS
PD23578	ICOSLG	cna	gain	BASIS
PD24186	ICOSLG	cna	amp	BASIS
PD24206	ICOSLG	cna	gain	BASIS
PD3905	ICOSLG	cna	gain	BASIS
PD4006	ICOSLG	cna	amp	BASIS
PD4107	ICOSLG	cna	gain	BASIS
PD4826	ICOSLG	cna	gain	BASIS
PD5930	ICOSLG	cna	gain	BASIS
PD5945	ICOSLG	cna	gain	BASIS
PD5948	ICOSLG	cna	gain	BASIS
PD6731	ICOSLG	cna	gain	BASIS
PD7067	ICOSLG	cna	gain	BASIS
PD7215	ICOSLG	cna	gain	BASIS
PD8621	ICOSLG	cna	gain	BASIS
PD8980	ICOSLG	cna	hetloss	BASIS
PD9004	ICOSLG	cna	gain	BASIS
PD9702	ICOSLG	cna	gain	BASIS
TCGA-A2-A25B	ID3	cna	hetloss	TCGA
TCGA-AN-A0XU	ID3	cna	hetloss	TCGA
TCGA-AO-A0JL	ID3	cna	hetloss	TCGA
TCGA-BH-A0AW	ID3	cna	gain	TCGA
TCGA-BH-A0C0	ID3	cna	hetloss	TCGA
TCGA-BH-A18R	ID3	cna	homdel	TCGA
TCGA-BH-A1FU	ID3	cna	gain	TCGA
TCGA-C8-A12L	ID3	cna	hetloss	TCGA
TCGA-D8-A27M	ID3	cna	gain	TCGA
TCGA-E2-A1L7	ID3	cna	hetloss	TCGA
TCGA-E9-A1NC	ID3	cna	hetloss	TCGA
TCGA-LL-A5YP	ID3	cna	hetloss	TCGA
MB-2827	ID3	cna	hetloss	METABRIC
MB-5070	ID3	cna	hetloss	METABRIC
MB-5465	ID3	cna	hetloss	METABRIC
MB-6060	ID3	cna	hetloss	METABRIC
MB-6098	ID3	cna	hetloss	METABRIC
MB-6271	ID3	cna	hetloss	METABRIC
MB-0420	ID3	cna	hetloss	METABRIC
PD10014	ID3	cna	gain	BASIS
PD11742	ID3	cna	hetloss	BASIS
PD13296	ID3	cna	hetloss	BASIS
PD13771	ID3	cna	hetloss	BASIS
PD22355	ID3	cna	hetloss	BASIS
PD24186	ID3	cna	gain	BASIS
PD24206	ID3	cna	hetloss	BASIS
PD24337	ID3	cna	hetloss	BASIS
PD3890	ID3	cna	hetloss	BASIS
PD3905	ID3	cna	gain	BASIS

PD4006	ID3	cna	gain	BASIS
PD4826	ID3	cna	gain	BASIS
PD5945	ID3	cna	amp	BASIS
PD7067	ID3	cna	gain	BASIS
PD7215	ID3	cna	gain	BASIS
PD9004	ID3	cna	hetloss	BASIS
PD9585	ID3	cna	hetloss	BASIS
TCGA-A2-A25B	IGF1	cna	gain	TCGA
TCGA-AO-A0JL	IGF1	cna	hetloss	TCGA
TCGA-BH-A0C0	IGF1	cna	hetloss	TCGA
TCGA-BH-A18R	IGF1	cna	gain	TCGA
TCGA-BH-A1FU	IGF1	cna	hetloss	TCGA
TCGA-C8-A12L	IGF1	cna	gain	TCGA
TCGA-D8-A27M	IGF1	cna	hetloss	TCGA
TCGA-E2-A1L7	IGF1	cna	hetloss	TCGA
TCGA-LL-A5YP	IGF1	cna	hetloss	TCGA
MB-0346	IGF1	cna	gain	METABRIC
MB-2827	IGF1	cna	hetloss	METABRIC
MB-5070	IGF1	cna	hetloss	METABRIC
MB-5107	IGF1	cna	gain	METABRIC
MB-5465	IGF1	cna	gain	METABRIC
MB-6098	IGF1	cna	hetloss	METABRIC
PD10014	IGF1	cna	gain	BASIS
PD11327	IGF1	cna	gain	BASIS
PD11742	IGF1	cna	hetloss	BASIS
PD13296	IGF1	cna	hetloss	BASIS
PD13297	IGF1	cna	gain	BASIS
PD22355	IGF1	cna	hetloss	BASIS
PD3890	IGF1	cna	hetloss	BASIS
PD4005	IGF1	cna	hetloss	BASIS
PD4006	IGF1	cna	gain	BASIS
PD4107	IGF1	cna	gain	BASIS
PD4826	IGF1	cna	gain	BASIS
PD5945	IGF1	cna	gain	BASIS
PD6406	IGF1	cna	hetloss	BASIS
PD6413	IGF1	cna	hetloss	BASIS
PD6731	IGF1	cna	hetloss	BASIS
PD7067	IGF1	cna	gain	BASIS
PD7215	IGF1	cna	hetloss	BASIS
PD8621	IGF1	cna	hetloss	BASIS
PD9004	IGF1	cna	hetloss	BASIS
PD9585	IGF1	cna	hetloss	BASIS
PD9702	IGF1	cna	gain	BASIS
TCGA-A2-A25B	INPP4B	cna	gain	TCGA
TCGA-AN-A0XU	INPP4B	cna	gain	TCGA
TCGA-BH-A0AW	INPP4B	cna	gain	TCGA
TCGA-BH-A0C0	INPP4B	cna	hetloss	TCGA

TCGA-BH-A18R	INPP4B	cna	amp	TCGA
TCGA-BH-A1FU	INPP4B	cna	gain	TCGA
TCGA-C8-A12L	INPP4B	cna	hetloss	TCGA
TCGA-D8-A27M	INPP4B	cna	hetloss	TCGA
TCGA-E2-A1L7	INPP4B	cna	hetloss	TCGA
TCGA-E9-A1NC	INPP4B	cna	hetloss	TCGA
MB-5070	INPP4B	cna	hetloss	METABRIC
MB-5465	INPP4B	cna	hetloss	METABRIC
MB-7048	INPP4B	cna	hetloss	METABRIC
MB-0420	INPP4B	cna	hetloss	METABRIC
PD10014	INPP4B	cna	hetloss	BASIS
PD11742	INPP4B	cna	hetloss	BASIS
PD13296	INPP4B	cna	hetloss	BASIS
PD13297	INPP4B	cna	hetloss	BASIS
PD22355	INPP4B	cna	hetloss	BASIS
PD23562	INPP4B	cna	gain	BASIS
PD23574	INPP4B	cna	gain	BASIS
PD23578	INPP4B	cna	hetloss	BASIS
PD24202	INPP4B	cna	hetloss	BASIS
PD24206	INPP4B	cna	gain	BASIS
PD3905	INPP4B	cna	gain	BASIS
PD4005	INPP4B	cna	hetloss	BASIS
PD4006	INPP4B	cna	hetloss	BASIS
PD4107	INPP4B	cna	hetloss	BASIS
PD4826	INPP4B	cna	gain	BASIS
PD5930	INPP4B	cna	gain	BASIS
PD5945	INPP4B	cna	gain	BASIS
PD5948	INPP4B	cna	hetloss	BASIS
PD6406	INPP4B	cna	hetloss	BASIS
PD6731	INPP4B	cna	hetloss	BASIS
PD7215	INPP4B	cna	amp	BASIS
PD8980	INPP4B	cna	hetloss	BASIS
TCGA-A2-A25B	IRS2	cna	hetloss	TCGA
TCGA-AN-A0XU	IRS2	cna	hetloss	TCGA
TCGA-AO-A0JL	IRS2	cna	hetloss	TCGA
TCGA-BH-A0C0	IRS2	cna	hetloss	TCGA
TCGA-BH-A1FU	IRS2	cna	hetloss	TCGA
TCGA-C8-A12L	IRS2	cna	gain	TCGA
TCGA-D8-A27M	IRS2	cna	gain	TCGA
TCGA-LL-A5YP	IRS2	cna	hetloss	TCGA
MB-0346	IRS2	cna	hetloss	METABRIC
MB-5107	IRS2	cna	hetloss	METABRIC
MB-5465	IRS2	cna	gain	METABRIC
MB-6060	IRS2	cna	hetloss	METABRIC
MB-0420	IRS2	cna	amp	METABRIC
PD10014	IRS2	cna	hetloss	BASIS
PD11327	IRS2	cna	amp	BASIS

PD13299	IRS2	cna	gain	BASIS
PD14442	IRS2	cna	hetloss	BASIS
PD22355	IRS2	cna	gain	BASIS
PD23562	IRS2	cna	gain	BASIS
PD23574	IRS2	cna	gain	BASIS
PD23578	IRS2	cna	gain	BASIS
PD24186	IRS2	cna	gain	BASIS
PD24202	IRS2	cna	gain	BASIS
PD24206	IRS2	cna	gain	BASIS
PD24337	IRS2	cna	hetloss	BASIS
PD3905	IRS2	cna	amp	BASIS
PD4006	IRS2	cna	hetloss	BASIS
PD4107	IRS2	cna	hetloss	BASIS
PD4967	IRS2	cna	hetloss	BASIS
PD5945	IRS2	cna	amp	BASIS
PD6731	IRS2	cna	hetloss	BASIS
PD7215	IRS2	cna	gain	BASIS
PD8621	IRS2	cna	amp	BASIS
PD8980	IRS2	cna	hetloss	BASIS
PD9585	IRS2	cna	gain	BASIS
PD9702	IRS2	cna	gain	BASIS
TCGA-A2-A25B	JUN	cna	gain	TCGA
TCGA-AN-A0XU	JUN	cna	gain	TCGA
TCGA-AO-A0JL	JUN	cna	hetloss	TCGA
TCGA-BH-A0AW	JUN	cna	gain	TCGA
TCGA-BH-A0C0	JUN	cna	hetloss	TCGA
TCGA-BH-A1FU	JUN	cna	gain	TCGA
TCGA-D8-A27M	JUN	cna	gain	TCGA
TCGA-E2-A1L7	JUN	cna	amp	TCGA
TCGA-LL-A5YP	JUN	cna	hetloss	TCGA
MB-0346	JUN	cna	hetloss	METABRIC
MB-6060	JUN	cna	amp	METABRIC
MB-6098	JUN	cna	hetloss	METABRIC
MB-7038	JUN	cna	gain	METABRIC
MB-0420	JUN	cna	hetloss	METABRIC
PD11327	JUN	cna	gain	BASIS
PD11742	JUN	cna	hetloss	BASIS
PD13296	JUN	cna	gain	BASIS
PD13299	JUN	cna	gain	BASIS
PD22355	JUN	cna	hetloss	BASIS
PD23574	JUN	cna	gain	BASIS
PD24186	JUN	cna	amp	BASIS
PD24206	JUN	cna	gain	BASIS
PD3890	JUN	cna	gain	BASIS
PD3905	JUN	cna	gain	BASIS
PD4006	JUN	cna	gain	BASIS
PD4107	JUN	cna	gain	BASIS

PD4826	JUN	cna	gain	BASIS
PD5930	JUN	cna	gain	BASIS
PD5935	JUN	cna	gain	BASIS
PD5945	JUN	cna	gain	BASIS
PD5948	JUN	cna	gain	BASIS
PD7067	JUN	cna	gain	BASIS
PD7215	JUN	cna	gain	BASIS
PD8621	JUN	cna	gain	BASIS
PD9004	JUN	cna	gain	BASIS
PD9702	JUN	cna	gain	BASIS
TCGA-A2-A25B	KDM4C	cna	hetloss	TCGA
TCGA-AO-A0JL	KDM4C	cna	gain	TCGA
TCGA-BH-A0AW	KDM4C	cna	hetloss	TCGA
TCGA-BH-A1FU	KDM4C	cna	hetloss	TCGA
TCGA-C8-A12L	KDM4C	cna	gain	TCGA
TCGA-E2-A1L7	KDM4C	cna	hetloss	TCGA
TCGA-E9-A1NC	KDM4C	cna	amp	TCGA
MB-0346	KDM4C	cna	gain	METABRIC
MB-2827	KDM4C	cna	hetloss	METABRIC
MB-6060	KDM4C	cna	gain	METABRIC
MB-0420	KDM4C	cna	hetloss	METABRIC
MTS-T0064	KDM4C	cna	amp	METABRIC
PD11327	KDM4C	cna	homdel	BASIS
PD13296	KDM4C	cna	gain	BASIS
PD13297	KDM4C	cna	hetloss	BASIS
PD13299	KDM4C	cna	amp	BASIS
PD22355	KDM4C	cna	gain	BASIS
PD23574	KDM4C	cna	amp	BASIS
PD24186	KDM4C	cna	gain	BASIS
PD24337	KDM4C	cna	gain	BASIS
PD3905	KDM4C	cna	gain	BASIS
PD4006	KDM4C	cna	gain	BASIS
PD4107	KDM4C	cna	gain	BASIS
PD4826	KDM4C	cna	gain	BASIS
PD5945	KDM4C	cna	gain	BASIS
PD5948	KDM4C	cna	hetloss	BASIS
PD6406	KDM4C	cna	homdel	BASIS
PD6413	KDM4C	cna	gain	BASIS
PD6731	KDM4C	cna	gain	BASIS
PD7067	KDM4C	cna	amp	BASIS
PD7215	KDM4C	cna	hetloss	BASIS
PD8621	KDM4C	cna	gain	BASIS
PD8980	KDM4C	cna	gain	BASIS
PD9004	KDM4C	cna	gain	BASIS
PD9585	KDM4C	cna	hetloss	BASIS
PD9702	KDM4C	cna	gain	BASIS
TCGA-A2-A25B	KMT2D	cna	gain	TCGA

TCGA-AN-A0XU	KMT2D	cna	gain	TCGA
TCGA-AO-A0JL	KMT2D	cna	hetloss	TCGA
TCGA-BH-A18R	KMT2D	cna	gain	TCGA
TCGA-BH-A1FU	KMT2D	cna	hetloss	TCGA
TCGA-C8-A12L	KMT2D	cna	gain	TCGA
TCGA-D8-A27M	KMT2D	cna	hetloss	TCGA
TCGA-E2-A1L7	KMT2D	cna	hetloss	TCGA
TCGA-E9-A1NC	KMT2D	cna	hetloss	TCGA
TCGA-LL-A5YP	KMT2D	cna	hetloss	TCGA
MB-0346	KMT2D	cna	gain	METABRIC
MB-2827	KMT2D	cna	hetloss	METABRIC
MB-5070	KMT2D	cna	hetloss	METABRIC
MB-6060	KMT2D	cna	gain	METABRIC
MB-6098	KMT2D	cna	hetloss	METABRIC
MB-7038	KMT2D	cna	hetloss	METABRIC
MB-7048	KMT2D	cna	hetloss	METABRIC
MB-0420	KMT2D	cna	hetloss	METABRIC
PD11327	KMT2D	cna	hetloss	BASIS
PD13296	KMT2D	cna	hetloss	BASIS
PD13297	KMT2D	cna	hetloss	BASIS
PD23578	KMT2D	cna	hetloss	BASIS
PD24202	KMT2D	cna	hetloss	BASIS
PD24206	KMT2D	cna	gain	BASIS
PD24337	KMT2D	cna	hetloss	BASIS
PD3890	KMT2D	cna	hetloss	BASIS
PD3905	KMT2D	cna	gain	BASIS
PD4107	KMT2D	cna	hetloss	BASIS
PD4826	KMT2D	cna	gain	BASIS
PD5945	KMT2D	cna	gain	BASIS
PD6413	KMT2D	cna	hetloss	BASIS
PD6731	KMT2D	cna	hetloss	BASIS
PD7067	KMT2D	cna	gain	BASIS
PD8621	KMT2D	cna	gain	BASIS
PD9585	KMT2D	cna	hetloss	BASIS
PD9702	KMT2D	cna	gain	BASIS
TCGA-A2-A25B	LCK	cna	hetloss	TCGA
TCGA-AN-A0XU	LCK	cna	hetloss	TCGA
TCGA-AO-A0JL	LCK	cna	hetloss	TCGA
TCGA-BH-A0AW	LCK	cna	gain	TCGA
TCGA-BH-A0C0	LCK	cna	hetloss	TCGA
TCGA-BH-A18R	LCK	cna	homdel	TCGA
TCGA-BH-A1FU	LCK	cna	gain	TCGA
TCGA-C8-A12L	LCK	cna	hetloss	TCGA
TCGA-D8-A27M	LCK	cna	gain	TCGA
TCGA-E2-A1L7	LCK	cna	gain	TCGA
TCGA-LL-A5YP	LCK	cna	gain	TCGA
MB-0346	LCK	cna	hetloss	METABRIC

MB-2827	LCK	cna	hetloss	METABRIC
MB-5070	LCK	cna	hetloss	METABRIC
MB-5465	LCK	cna	hetloss	METABRIC
MB-6060	LCK	cna	hetloss	METABRIC
MB-6098	LCK	cna	hetloss	METABRIC
MB-0420	LCK	cna	hetloss	METABRIC
PD10014	LCK	cna	gain	BASIS
PD11327	LCK	cna	hetloss	BASIS
PD11742	LCK	cna	hetloss	BASIS
PD13296	LCK	cna	hetloss	BASIS
PD24206	LCK	cna	gain	BASIS
PD24337	LCK	cna	hetloss	BASIS
PD3890	LCK	cna	hetloss	BASIS
PD3905	LCK	cna	gain	BASIS
PD4006	LCK	cna	gain	BASIS
PD4826	LCK	cna	gain	BASIS
PD5945	LCK	cna	amp	BASIS
PD5948	LCK	cna	gain	BASIS
PD7067	LCK	cna	gain	BASIS
PD7215	LCK	cna	gain	BASIS
PD8980	LCK	cna	gain	BASIS
PD9004	LCK	cna	hetloss	BASIS
PD9585	LCK	cna	hetloss	BASIS
PD9702	LCK	cna	gain	BASIS
TCGA-AN-A0XU	MAF	cna	gain	TCGA
TCGA-AO-A0JL	MAF	cna	gain	TCGA
TCGA-BH-A0AW	MAF	cna	hetloss	TCGA
TCGA-BH-A0C0	MAF	cna	hetloss	TCGA
TCGA-C8-A12L	MAF	cna	hetloss	TCGA
TCGA-D8-A27M	MAF	cna	gain	TCGA
TCGA-E2-A1L7	MAF	cna	hetloss	TCGA
TCGA-E9-A1NC	MAF	cna	hetloss	TCGA
TCGA-LL-A5YP	MAF	cna	gain	TCGA
MB-0346	MAF	cna	gain	METABRIC
MB-5107	MAF	cna	hetloss	METABRIC
MB-6271	MAF	cna	hetloss	METABRIC
MB-7032	MAF	cna	gain	METABRIC
MB-7038	MAF	cna	gain	METABRIC
MB-7048	MAF	cna	hetloss	METABRIC
MB-0420	MAF	cna	hetloss	METABRIC
PD10014	MAF	cna	hetloss	BASIS
PD11327	MAF	cna	gain	BASIS
PD11742	MAF	cna	hetloss	BASIS
PD13297	MAF	cna	hetloss	BASIS
PD13299	MAF	cna	gain	BASIS
PD14442	MAF	cna	hetloss	BASIS
PD23562	MAF	cna	gain	BASIS

PD24202	MAF	cna	hetloss	BASIS
PD24337	MAF	cna	hetloss	BASIS
PD3890	MAF	cna	gain	BASIS
PD3905	MAF	cna	gain	BASIS
PD4005	MAF	cna	hetloss	BASIS
PD4006	MAF	cna	hetloss	BASIS
PD4107	MAF	cna	gain	BASIS
PD4967	MAF	cna	hetloss	BASIS
PD5945	MAF	cna	amp	BASIS
PD5948	MAF	cna	gain	BASIS
PD7215	MAF	cna	gain	BASIS
PD9585	MAF	cna	hetloss	BASIS
PD9702	MAF	cna	gain	BASIS
TCGA-A2-A25B	MDS2	cna	hetloss	TCGA
TCGA-AN-A0XU	MDS2	cna	hetloss	TCGA
TCGA-AO-A0JL	MDS2	cna	hetloss	TCGA
TCGA-BH-A0AW	MDS2	cna	gain	TCGA
TCGA-BH-A0C0	MDS2	cna	hetloss	TCGA
TCGA-BH-A18R	MDS2	cna	homdel	TCGA
TCGA-BH-A1FU	MDS2	cna	gain	TCGA
TCGA-C8-A12L	MDS2	cna	hetloss	TCGA
TCGA-D8-A27M	MDS2	cna	gain	TCGA
TCGA-E2-A1L7	MDS2	cna	hetloss	TCGA
TCGA-E9-A1NC	MDS2	cna	hetloss	TCGA
TCGA-LL-A5YP	MDS2	cna	hetloss	TCGA
MB-2827	MDS2	cna	hetloss	METABRIC
MB-5070	MDS2	cna	hetloss	METABRIC
MB-5465	MDS2	cna	hetloss	METABRIC
MB-6060	MDS2	cna	hetloss	METABRIC
MB-6098	MDS2	cna	hetloss	METABRIC
MB-6271	MDS2	cna	hetloss	METABRIC
MB-0420	MDS2	cna	hetloss	METABRIC
PD10014	MDS2	cna	gain	BASIS
PD11742	MDS2	cna	hetloss	BASIS
PD13296	MDS2	cna	hetloss	BASIS
PD13771	MDS2	cna	hetloss	BASIS
PD22355	MDS2	cna	hetloss	BASIS
PD24186	MDS2	cna	gain	BASIS
PD24206	MDS2	cna	hetloss	BASIS
PD24337	MDS2	cna	hetloss	BASIS
PD3890	MDS2	cna	hetloss	BASIS
PD3905	MDS2	cna	gain	BASIS
PD4006	MDS2	cna	gain	BASIS
PD4826	MDS2	cna	gain	BASIS
PD5945	MDS2	cna	amp	BASIS
PD7067	MDS2	cna	gain	BASIS
PD7215	MDS2	cna	gain	BASIS

PD9004	MDS2	cna	hetloss	BASIS
PD9585	MDS2	cna	hetloss	BASIS
TCGA-A2-A25B	MIR17HG	cna	hetloss	TCGA
TCGA-AN-A0XU	MIR17HG	cna	hetloss	TCGA
TCGA-AO-A0JL	MIR17HG	cna	hetloss	TCGA
TCGA-BH-A0AW	MIR17HG	cna	amp	TCGA
TCGA-BH-A0C0	MIR17HG	cna	hetloss	TCGA
TCGA-BH-A1FU	MIR17HG	cna	hetloss	TCGA
TCGA-C8-A12L	MIR17HG	cna	gain	TCGA
TCGA-LL-A5YP	MIR17HG	cna	hetloss	TCGA
MB-0346	MIR17HG	cna	hetloss	METABRIC
MB-5070	MIR17HG	cna	hetloss	METABRIC
MB-6060	MIR17HG	cna	hetloss	METABRIC
MB-6098	MIR17HG	cna	gain	METABRIC
MB-0420	MIR17HG	cna	amp	METABRIC
PD10014	MIR17HG	cna	hetloss	BASIS
PD11327	MIR17HG	cna	gain	BASIS
PD13296	MIR17HG	cna	hetloss	BASIS
PD13299	MIR17HG	cna	gain	BASIS
PD14442	MIR17HG	cna	hetloss	BASIS
PD23562	MIR17HG	cna	gain	BASIS
PD23578	MIR17HG	cna	gain	BASIS
PD24202	MIR17HG	cna	hetloss	BASIS
PD24206	MIR17HG	cna	gain	BASIS
PD24337	MIR17HG	cna	hetloss	BASIS
PD3905	MIR17HG	cna	amp	BASIS
PD4005	MIR17HG	cna	hetloss	BASIS
PD4006	MIR17HG	cna	hetloss	BASIS
PD4107	MIR17HG	cna	hetloss	BASIS
PD5945	MIR17HG	cna	amp	BASIS
PD5948	MIR17HG	cna	hetloss	BASIS
PD6731	MIR17HG	cna	hetloss	BASIS
PD7067	MIR17HG	cna	gain	BASIS
PD7215	MIR17HG	cna	gain	BASIS
PD8621	MIR17HG	cna	amp	BASIS
PD8980	MIR17HG	cna	hetloss	BASIS
PD9585	MIR17HG	cna	gain	BASIS
PD9702	MIR17HG	cna	gain	BASIS
TCGA-A2-A25B	MKL1	cna	hetloss	TCGA
TCGA-AN-A0XU	MKL1	cna	hetloss	TCGA
TCGA-BH-A0AW	MKL1	cna	hetloss	TCGA
TCGA-BH-A1FU	MKL1	cna	gain	TCGA
TCGA-C8-A12L	MKL1	cna	gain	TCGA
TCGA-D8-A27M	MKL1	cna	gain	TCGA
TCGA-E9-A1NC	MKL1	cna	gain	TCGA
MB-2827	MKL1	cna	hetloss	METABRIC
MB-5465	MKL1	cna	gain	METABRIC

MB-6098	MKL1	cna	gain	METABRIC
MTS-T0064	MKL1	cna	gain	METABRIC
PD11327	MKL1	cna	gain	BASIS
PD11742	MKL1	cna	hetloss	BASIS
PD13296	MKL1	cna	gain	BASIS
PD13297	MKL1	cna	hetloss	BASIS
PD13299	MKL1	cna	gain	BASIS
PD14442	MKL1	cna	hetloss	BASIS
PD22355	MKL1	cna	gain	BASIS
PD23562	MKL1	cna	gain	BASIS
PD23578	MKL1	cna	gain	BASIS
PD24206	MKL1	cna	homdel	BASIS
PD3905	MKL1	cna	gain	BASIS
PD4005	MKL1	cna	gain	BASIS
PD4006	MKL1	cna	gain	BASIS
PD4107	MKL1	cna	gain	BASIS
PD4826	MKL1	cna	gain	BASIS
PD4967	MKL1	cna	hetloss	BASIS
PD5935	MKL1	cna	gain	BASIS
PD5945	MKL1	cna	amp	BASIS
PD5948	MKL1	cna	gain	BASIS
PD6731	MKL1	cna	hetloss	BASIS
PD7067	MKL1	cna	gain	BASIS
PD7215	MKL1	cna	gain	BASIS
PD8621	MKL1	cna	gain	BASIS
PD9004	MKL1	cna	gain	BASIS
PD9702	MKL1	cna	gain	BASIS
TCGA-A2-A25B	MLLT10	cna	gain	TCGA
TCGA-AN-A0XU	MLLT10	cna	amp	TCGA
TCGA-AO-A0JL	MLLT10	cna	gain	TCGA
TCGA-BH-A0AW	MLLT10	cna	gain	TCGA
TCGA-BH-A0C0	MLLT10	cna	gain	TCGA
TCGA-C8-A12L	MLLT10	cna	gain	TCGA
TCGA-LL-A5YP	MLLT10	cna	gain	TCGA
MB-6098	MLLT10	cna	gain	METABRIC
MB-7038	MLLT10	cna	hetloss	METABRIC
MTS-T0064	MLLT10	cna	gain	METABRIC
PD10014	MLLT10	cna	amp	BASIS
PD11327	MLLT10	cna	amp	BASIS
PD13296	MLLT10	cna	gain	BASIS
PD13299	MLLT10	cna	gain	BASIS
PD13771	MLLT10	cna	gain	BASIS
PD22355	MLLT10	cna	gain	BASIS
PD23562	MLLT10	cna	amp	BASIS
PD23574	MLLT10	cna	gain	BASIS
PD24186	MLLT10	cna	gain	BASIS
PD24202	MLLT10	cna	hetloss	BASIS

PD3890	MLLT10	cna	hetloss	BASIS
PD3905	MLLT10	cna	gain	BASIS
PD4005	MLLT10	cna	gain	BASIS
PD4006	MLLT10	cna	gain	BASIS
PD4107	MLLT10	cna	gain	BASIS
PD5930	MLLT10	cna	gain	BASIS
PD5945	MLLT10	cna	amp	BASIS
PD5948	MLLT10	cna	gain	BASIS
PD6406	MLLT10	cna	hetloss	BASIS
PD6413	MLLT10	cna	gain	BASIS
PD7067	MLLT10	cna	gain	BASIS
PD7215	MLLT10	cna	gain	BASIS
PD8621	MLLT10	cna	gain	BASIS
PD8980	MLLT10	cna	gain	BASIS
PD9004	MLLT10	cna	gain	BASIS
PD9702	MLLT10	cna	gain	BASIS
TCGA-A2-A25B	MSN	cna	gain	TCGA
TCGA-AN-A0XU	MSN	cna	hetloss	TCGA
TCGA-AO-A0JL	MSN	cna	gain	TCGA
TCGA-BH-A0AW	MSN	cna	gain	TCGA
TCGA-E2-A1L7	MSN	cna	hetloss	TCGA
TCGA-E9-A1NC	MSN	cna	hetloss	TCGA
MB-0346	MSN	cna	amp	METABRIC
MB-5465	MSN	cna	gain	METABRIC
MB-6060	MSN	cna	hetloss	METABRIC
MB-6098	MSN	cna	hetloss	METABRIC
MB-0420	MSN	cna	hetloss	METABRIC
PD10014	MSN	cna	hetloss	BASIS
PD11327	MSN	cna	hetloss	BASIS
PD13296	MSN	cna	amp	BASIS
PD13299	MSN	cna	gain	BASIS
PD13771	MSN	cna	gain	BASIS
PD14442	MSN	cna	gain	BASIS
PD23562	MSN	cna	hetloss	BASIS
PD23574	MSN	cna	gain	BASIS
PD24206	MSN	cna	amp	BASIS
PD3890	MSN	cna	gain	BASIS
PD3905	MSN	cna	gain	BASIS
PD4005	MSN	cna	hetloss	BASIS
PD4107	MSN	cna	gain	BASIS
PD4826	MSN	cna	gain	BASIS
PD4967	MSN	cna	gain	BASIS
PD5930	MSN	cna	gain	BASIS
PD5935	MSN	cna	gain	BASIS
PD5945	MSN	cna	gain	BASIS
PD5948	MSN	cna	gain	BASIS
PD6406	MSN	cna	gain	BASIS

PD7067	MSN	cna	gain	BASIS
PD7215	MSN	cna	gain	BASIS
PD8621	MSN	cna	gain	BASIS
PD8980	MSN	cna	gain	BASIS
PD9004	MSN	cna	gain	BASIS
TCGA-A2-A25B	MTOR	cna	hetloss	TCGA
TCGA-AN-A0XU	MTOR	cna	hetloss	TCGA
TCGA-AO-A0JL	MTOR	cna	hetloss	TCGA
TCGA-BH-A0AW	MTOR	cna	gain	TCGA
TCGA-BH-A0C0	MTOR	cna	hetloss	TCGA
TCGA-BH-A18R	MTOR	cna	homdel	TCGA
TCGA-BH-A1FU	MTOR	cna	gain	TCGA
TCGA-C8-A12L	MTOR	cna	hetloss	TCGA
TCGA-D8-A27M	MTOR	cna	gain	TCGA
TCGA-E2-A1L7	MTOR	cna	hetloss	TCGA
TCGA-E9-A1NC	MTOR	cna	hetloss	TCGA
TCGA-LL-A5YP	MTOR	cna	amp	TCGA
MB-2827	MTOR	cna	hetloss	METABRIC
MB-5070	MTOR	cna	hetloss	METABRIC
MB-5465	MTOR	cna	hetloss	METABRIC
MB-6060	MTOR	cna	hetloss	METABRIC
MB-6098	MTOR	cna	hetloss	METABRIC
MB-0420	MTOR	cna	hetloss	METABRIC
P-0010758-T01-IM5	MTOR	cna	homdel	MSK-IMPACT
PD10014	MTOR	cna	gain	BASIS
PD11742	MTOR	cna	hetloss	BASIS
PD13296	MTOR	cna	hetloss	BASIS
PD22355	MTOR	cna	hetloss	BASIS
PD24202	MTOR	cna	hetloss	BASIS
PD24206	MTOR	cna	gain	BASIS
PD24337	MTOR	cna	hetloss	BASIS
PD3890	MTOR	cna	hetloss	BASIS
PD3905	MTOR	cna	gain	BASIS
PD4107	MTOR	cna	hetloss	BASIS
PD4826	MTOR	cna	gain	BASIS
PD5945	MTOR	cna	gain	BASIS
PD6406	MTOR	cna	hetloss	BASIS
PD7067	MTOR	cna	gain	BASIS
PD7215	MTOR	cna	gain	BASIS
PD8980	MTOR	cna	hetloss	BASIS
PD9004	MTOR	cna	hetloss	BASIS
TCGA-A2-A25B	MYH9	cna	hetloss	TCGA
TCGA-AO-A0JL	MYH9	cna	hetloss	TCGA
TCGA-BH-A0AW	MYH9	cna	hetloss	TCGA
TCGA-BH-A1FU	MYH9	cna	gain	TCGA
TCGA-C8-A12L	MYH9	cna	gain	TCGA
TCGA-D8-A27M	MYH9	cna	gain	TCGA

TCGA-E9-A1NC	MYH9	cna	gain	TCGA
TCGA-LL-A5YP	MYH9	cna	gain	TCGA
MB-0346	MYH9	cna	gain	METABRIC
MB-5465	MYH9	cna	gain	METABRIC
MB-6098	MYH9	cna	gain	METABRIC
PD11742	MYH9	cna	hetloss	BASIS
PD13296	MYH9	cna	gain	BASIS
PD13297	MYH9	cna	gain	BASIS
PD13299	MYH9	cna	gain	BASIS
PD14442	MYH9	cna	hetloss	BASIS
PD22355	MYH9	cna	gain	BASIS
PD23562	MYH9	cna	gain	BASIS
PD23574	MYH9	cna	gain	BASIS
PD23578	MYH9	cna	gain	BASIS
PD24206	MYH9	cna	hetloss	BASIS
PD3905	MYH9	cna	gain	BASIS
PD4006	MYH9	cna	gain	BASIS
PD4107	MYH9	cna	gain	BASIS
PD4826	MYH9	cna	gain	BASIS
PD4967	MYH9	cna	hetloss	BASIS
PD5935	MYH9	cna	gain	BASIS
PD5945	MYH9	cna	amp	BASIS
PD5948	MYH9	cna	gain	BASIS
PD6406	MYH9	cna	gain	BASIS
PD6731	MYH9	cna	hetloss	BASIS
PD7067	MYH9	cna	gain	BASIS
PD7215	MYH9	cna	gain	BASIS
PD8621	MYH9	cna	gain	BASIS
PD9004	MYH9	cna	gain	BASIS
PD9702	MYH9	cna	gain	BASIS
TCGA-A2-A25B	MYO18A	cna	amp	TCGA
TCGA-AN-A0XU	MYO18A	cna	gain	TCGA
TCGA-AO-A0JL	MYO18A	cna	gain	TCGA
TCGA-BH-A0AW	MYO18A	cna	amp	TCGA
TCGA-BH-A0C0	MYO18A	cna	gain	TCGA
TCGA-BH-A18R	MYO18A	cna	gain	TCGA
TCGA-C8-A12L	MYO18A	cna	gain	TCGA
TCGA-D8-A27M	MYO18A	cna	hetloss	TCGA
TCGA-E2-A1L7	MYO18A	cna	hetloss	TCGA
TCGA-E9-A1NC	MYO18A	cna	hetloss	TCGA
TCGA-LL-A5YP	MYO18A	cna	hetloss	TCGA
MB-0346	MYO18A	cna	hetloss	METABRIC
MB-2827	MYO18A	cna	homdel	METABRIC
MB-5070	MYO18A	cna	gain	METABRIC
MB-5465	MYO18A	cna	hetloss	METABRIC
MB-6060	MYO18A	cna	gain	METABRIC
MB-6098	MYO18A	cna	hetloss	METABRIC

MB-6271	MYO18A	cna	hetloss	METABRIC
MB-0420	MYO18A	cna	hetloss	METABRIC
PD11742	MYO18A	cna	hetloss	BASIS
PD13297	MYO18A	cna	hetloss	BASIS
PD22355	MYO18A	cna	hetloss	BASIS
PD23561	MYO18A	cna	gain	BASIS
PD23562	MYO18A	cna	gain	BASIS
PD24337	MYO18A	cna	hetloss	BASIS
PD4005	MYO18A	cna	hetloss	BASIS
PD4967	MYO18A	cna	hetloss	BASIS
PD5945	MYO18A	cna	gain	BASIS
PD6406	MYO18A	cna	hetloss	BASIS
PD6413	MYO18A	cna	hetloss	BASIS
PD6731	MYO18A	cna	hetloss	BASIS
PD7067	MYO18A	cna	gain	BASIS
PD7215	MYO18A	cna	gain	BASIS
PD9004	MYO18A	cna	hetloss	BASIS
PD9585	MYO18A	cna	hetloss	BASIS
PD9702	MYO18A	cna	gain	BASIS
TCGA-A2-A25B	NCOA3	cna	gain	TCGA
TCGA-AO-A0JL	NCOA3	cna	gain	TCGA
TCGA-BH-A0AW	NCOA3	cna	gain	TCGA
TCGA-BH-A0C0	NCOA3	cna	gain	TCGA
TCGA-C8-A12L	NCOA3	cna	gain	TCGA
TCGA-D8-A27M	NCOA3	cna	hetloss	TCGA
TCGA-E2-A1L7	NCOA3	cna	gain	TCGA
TCGA-E9-A1NC	NCOA3	cna	hetloss	TCGA
TCGA-LL-A5YP	NCOA3	cna	hetloss	TCGA
MB-0346	NCOA3	cna	gain	METABRIC
MB-5070	NCOA3	cna	hetloss	METABRIC
MB-5107	NCOA3	cna	gain	METABRIC
MB-6060	NCOA3	cna	gain	METABRIC
MB-6098	NCOA3	cna	hetloss	METABRIC
PD11327	NCOA3	cna	homdel	BASIS
PD13297	NCOA3	cna	hetloss	BASIS
PD13299	NCOA3	cna	gain	BASIS
PD13771	NCOA3	cna	gain	BASIS
PD22355	NCOA3	cna	hetloss	BASIS
PD23562	NCOA3	cna	gain	BASIS
PD24186	NCOA3	cna	gain	BASIS
PD24202	NCOA3	cna	hetloss	BASIS
PD3905	NCOA3	cna	gain	BASIS
PD4005	NCOA3	cna	hetloss	BASIS
PD4006	NCOA3	cna	gain	BASIS
PD4826	NCOA3	cna	gain	BASIS
PD5935	NCOA3	cna	gain	BASIS
PD5945	NCOA3	cna	amp	BASIS

PD5948	NCOA3	cna	gain	BASIS
PD6406	NCOA3	cna	gain	BASIS
PD6731	NCOA3	cna	hetloss	BASIS
PD7067	NCOA3	cna	gain	BASIS
PD7215	NCOA3	cna	amp	BASIS
PD8980	NCOA3	cna	gain	BASIS
PD9004	NCOA3	cna	gain	BASIS
PD9585	NCOA3	cna	hetloss	BASIS
TCGA-A2-A25B	NFIB	cna	hetloss	TCGA
TCGA-AO-A0JL	NFIB	cna	gain	TCGA
TCGA-BH-A0AW	NFIB	cna	hetloss	TCGA
TCGA-BH-A1FU	NFIB	cna	hetloss	TCGA
TCGA-C8-A12L	NFIB	cna	gain	TCGA
TCGA-E2-A1L7	NFIB	cna	hetloss	TCGA
TCGA-E9-A1NC	NFIB	cna	amp	TCGA
MB-0346	NFIB	cna	hetloss	METABRIC
MB-5070	NFIB	cna	hetloss	METABRIC
MB-5465	NFIB	cna	gain	METABRIC
MB-6098	NFIB	cna	hetloss	METABRIC
MB-0420	NFIB	cna	hetloss	METABRIC
MTS-T0064	NFIB	cna	amp	METABRIC
PD11327	NFIB	cna	hetloss	BASIS
PD13296	NFIB	cna	gain	BASIS
PD13297	NFIB	cna	hetloss	BASIS
PD13299	NFIB	cna	amp	BASIS
PD22355	NFIB	cna	gain	BASIS
PD23574	NFIB	cna	amp	BASIS
PD24186	NFIB	cna	gain	BASIS
PD24202	NFIB	cna	hetloss	BASIS
PD24337	NFIB	cna	gain	BASIS
PD3905	NFIB	cna	amp	BASIS
PD4005	NFIB	cna	homdel	BASIS
PD4006	NFIB	cna	gain	BASIS
PD4107	NFIB	cna	gain	BASIS
PD4826	NFIB	cna	gain	BASIS
PD5945	NFIB	cna	amp	BASIS
PD6406	NFIB	cna	homdel	BASIS
PD6413	NFIB	cna	gain	BASIS
PD7067	NFIB	cna	amp	BASIS
PD8621	NFIB	cna	gain	BASIS
PD8980	NFIB	cna	hetloss	BASIS
PD9004	NFIB	cna	gain	BASIS
PD9585	NFIB	cna	hetloss	BASIS
PD9702	NFIB	cna	gain	BASIS
TCGA-AO-A0JL	PDCD1	cna	hetloss	TCGA
TCGA-BH-A0AW	PDCD1	cna	hetloss	TCGA
TCGA-C8-A12L	PDCD1	cna	hetloss	TCGA

TCGA-D8-A27M	PDCD1	cna	gain	TCGA
TCGA-E2-A1L7	PDCD1	cna	hetloss	TCGA
TCGA-E9-A1NC	PDCD1	cna	hetloss	TCGA
MB-5070	PDCD1	cna	hetloss	METABRIC
MB-5107	PDCD1	cna	hetloss	METABRIC
MB-6060	PDCD1	cna	hetloss	METABRIC
MB-6098	PDCD1	cna	hetloss	METABRIC
MB-6271	PDCD1	cna	hetloss	METABRIC
MB-7048	PDCD1	cna	hetloss	METABRIC
PD10014	PDCD1	cna	gain	BASIS
PD11327	PDCD1	cna	gain	BASIS
PD11742	PDCD1	cna	hetloss	BASIS
PD13296	PDCD1	cna	homdel	BASIS
PD13297	PDCD1	cna	hetloss	BASIS
PD23578	PDCD1	cna	hetloss	BASIS
PD24186	PDCD1	cna	gain	BASIS
PD24202	PDCD1	cna	hetloss	BASIS
PD24206	PDCD1	cna	gain	BASIS
PD3890	PDCD1	cna	hetloss	BASIS
PD3905	PDCD1	cna	gain	BASIS
PD4005	PDCD1	cna	hetloss	BASIS
PD4006	PDCD1	cna	gain	BASIS
PD4967	PDCD1	cna	hetloss	BASIS
PD5935	PDCD1	cna	gain	BASIS
PD5945	PDCD1	cna	gain	BASIS
PD5948	PDCD1	cna	gain	BASIS
PD6406	PDCD1	cna	hetloss	BASIS
PD6413	PDCD1	cna	hetloss	BASIS
PD6731	PDCD1	cna	hetloss	BASIS
PD7067	PDCD1	cna	gain	BASIS
PD7215	PDCD1	cna	gain	BASIS
PD8980	PDCD1	cna	gain	BASIS
PD9585	PDCD1	cna	hetloss	BASIS
TCGA-A2-A25B	PER1	cna	hetloss	TCGA
TCGA-AN-A0XU	PER1	cna	hetloss	TCGA
TCGA-AO-A0JL	PER1	cna	gain	TCGA
TCGA-BH-A0AW	PER1	cna	hetloss	TCGA
TCGA-BH-A0C0	PER1	cna	hetloss	TCGA
TCGA-C8-A12L	PER1	cna	hetloss	TCGA
TCGA-D8-A27M	PER1	cna	hetloss	TCGA
TCGA-E2-A1L7	PER1	cna	hetloss	TCGA
TCGA-E9-A1NC	PER1	cna	hetloss	TCGA
TCGA-LL-A5YP	PER1	cna	gain	TCGA
MB-0346	PER1	cna	hetloss	METABRIC
MB-2827	PER1	cna	hetloss	METABRIC
MB-5070	PER1	cna	hetloss	METABRIC
MB-5107	PER1	cna	hetloss	METABRIC

MB-6098	PER1	cna	hetloss	METABRIC
MB-6271	PER1	cna	hetloss	METABRIC
MB-7048	PER1	cna	hetloss	METABRIC
MB-0420	PER1	cna	hetloss	METABRIC
PD11742	PER1	cna	hetloss	BASIS
PD13296	PER1	cna	hetloss	BASIS
PD13771	PER1	cna	hetloss	BASIS
PD22355	PER1	cna	hetloss	BASIS
PD24202	PER1	cna	hetloss	BASIS
PD24206	PER1	cna	hetloss	BASIS
PD24337	PER1	cna	hetloss	BASIS
PD3890	PER1	cna	hetloss	BASIS
PD3905	PER1	cna	gain	BASIS
PD4005	PER1	cna	homdel	BASIS
PD4826	PER1	cna	hetloss	BASIS
PD4967	PER1	cna	hetloss	BASIS
PD5945	PER1	cna	gain	BASIS
PD6406	PER1	cna	hetloss	BASIS
PD6413	PER1	cna	hetloss	BASIS
PD6731	PER1	cna	hetloss	BASIS
PD7067	PER1	cna	gain	BASIS
PD9585	PER1	cna	hetloss	BASIS
TCGA-A2-A25B	PIK3CD	cna	hetloss	TCGA
TCGA-AN-A0XU	PIK3CD	cna	hetloss	TCGA
TCGA-AO-A0JL	PIK3CD	cna	hetloss	TCGA
TCGA-BH-A0AW	PIK3CD	cna	gain	TCGA
TCGA-BH-A0C0	PIK3CD	cna	hetloss	TCGA
TCGA-BH-A18R	PIK3CD	cna	homdel	TCGA
TCGA-BH-A1FU	PIK3CD	cna	gain	TCGA
TCGA-C8-A12L	PIK3CD	cna	hetloss	TCGA
TCGA-D8-A27M	PIK3CD	cna	gain	TCGA
TCGA-E2-A1L7	PIK3CD	cna	hetloss	TCGA
TCGA-LL-A5YP	PIK3CD	cna	amp	TCGA
MB-2827	PIK3CD	cna	hetloss	METABRIC
MB-5070	PIK3CD	cna	hetloss	METABRIC
MB-5465	PIK3CD	cna	hetloss	METABRIC
MB-6060	PIK3CD	cna	hetloss	METABRIC
MB-6098	PIK3CD	cna	gain	METABRIC
MB-0420	PIK3CD	cna	hetloss	METABRIC
P-0010758-T01-IM5	PIK3CD	cna	homdel	MSK-IMPACT
PD10014	PIK3CD	cna	gain	BASIS
PD11742	PIK3CD	cna	hetloss	BASIS
PD13296	PIK3CD	cna	hetloss	BASIS
PD22355	PIK3CD	cna	hetloss	BASIS
PD24202	PIK3CD	cna	hetloss	BASIS
PD24206	PIK3CD	cna	hetloss	BASIS
PD24337	PIK3CD	cna	hetloss	BASIS

PD3890	PIK3CD	cna	hetloss	BASIS
PD3905	PIK3CD	cna	gain	BASIS
PD4107	PIK3CD	cna	hetloss	BASIS
PD4826	PIK3CD	cna	gain	BASIS
PD5945	PIK3CD	cna	gain	BASIS
PD6406	PIK3CD	cna	hetloss	BASIS
PD7067	PIK3CD	cna	gain	BASIS
PD7215	PIK3CD	cna	gain	BASIS
PD8621	PIK3CD	cna	gain	BASIS
PD8980	PIK3CD	cna	hetloss	BASIS
PD9004	PIK3CD	cna	hetloss	BASIS
TCGA-A2-A25B	PIK3CG	cna	gain	TCGA
TCGA-AN-A0XU	PIK3CG	cna	hetloss	TCGA
TCGA-AO-A0JL	PIK3CG	cna	gain	TCGA
TCGA-BH-A0AW	PIK3CG	cna	gain	TCGA
TCGA-C8-A12L	PIK3CG	cna	hetloss	TCGA
TCGA-D8-A27M	PIK3CG	cna	gain	TCGA
TCGA-E2-A1L7	PIK3CG	cna	hetloss	TCGA
TCGA-E9-A1NC	PIK3CG	cna	gain	TCGA
TCGA-LL-A5YP	PIK3CG	cna	gain	TCGA
MB-0346	PIK3CG	cna	hetloss	METABRIC
MB-5465	PIK3CG	cna	hetloss	METABRIC
MB-6098	PIK3CG	cna	hetloss	METABRIC
MB-7038	PIK3CG	cna	gain	METABRIC
MB-7048	PIK3CG	cna	gain	METABRIC
PD11327	PIK3CG	cna	amp	BASIS
PD13296	PIK3CG	cna	hetloss	BASIS
PD22355	PIK3CG	cna	hetloss	BASIS
PD23574	PIK3CG	cna	gain	BASIS
PD24186	PIK3CG	cna	amp	BASIS
PD24337	PIK3CG	cna	hetloss	BASIS
PD3890	PIK3CG	cna	hetloss	BASIS
PD3905	PIK3CG	cna	gain	BASIS
PD4006	PIK3CG	cna	gain	BASIS
PD4107	PIK3CG	cna	gain	BASIS
PD4826	PIK3CG	cna	gain	BASIS
PD5930	PIK3CG	cna	gain	BASIS
PD5935	PIK3CG	cna	gain	BASIS
PD5945	PIK3CG	cna	amp	BASIS
PD5948	PIK3CG	cna	gain	BASIS
PD6406	PIK3CG	cna	gain	BASIS
PD7067	PIK3CG	cna	gain	BASIS
PD7215	PIK3CG	cna	gain	BASIS
PD8621	PIK3CG	cna	gain	BASIS
PD9004	PIK3CG	cna	gain	BASIS
PD9585	PIK3CG	cna	hetloss	BASIS
PD9702	PIK3CG	cna	amp	BASIS

TCGA-AN-A0XU	PLCG2	cna	gain	TCGA
TCGA-AO-A0JL	PLCG2	cna	gain	TCGA
TCGA-BH-A0AW	PLCG2	cna	hetloss	TCGA
TCGA-BH-A0C0	PLCG2	cna	hetloss	TCGA
TCGA-C8-A12L	PLCG2	cna	hetloss	TCGA
TCGA-D8-A27M	PLCG2	cna	gain	TCGA
TCGA-E2-A1L7	PLCG2	cna	hetloss	TCGA
TCGA-E9-A1NC	PLCG2	cna	hetloss	TCGA
TCGA-LL-A5YP	PLCG2	cna	gain	TCGA
MB-0346	PLCG2	cna	gain	METABRIC
MB-5107	PLCG2	cna	hetloss	METABRIC
MB-6271	PLCG2	cna	hetloss	METABRIC
MB-7032	PLCG2	cna	hetloss	METABRIC
MB-7038	PLCG2	cna	gain	METABRIC
MB-7048	PLCG2	cna	hetloss	METABRIC
MB-0420	PLCG2	cna	hetloss	METABRIC
PD10014	PLCG2	cna	hetloss	BASIS
PD11327	PLCG2	cna	gain	BASIS
PD11742	PLCG2	cna	hetloss	BASIS
PD13299	PLCG2	cna	gain	BASIS
PD14442	PLCG2	cna	hetloss	BASIS
PD23562	PLCG2	cna	gain	BASIS
PD24202	PLCG2	cna	hetloss	BASIS
PD3890	PLCG2	cna	gain	BASIS
PD3905	PLCG2	cna	gain	BASIS
PD4005	PLCG2	cna	homdel	BASIS
PD4006	PLCG2	cna	hetloss	BASIS
PD4107	PLCG2	cna	gain	BASIS
PD4826	PLCG2	cna	hetloss	BASIS
PD4967	PLCG2	cna	hetloss	BASIS
PD5945	PLCG2	cna	amp	BASIS
PD5948	PLCG2	cna	gain	BASIS
PD6413	PLCG2	cna	gain	BASIS
PD7215	PLCG2	cna	gain	BASIS
PD9585	PLCG2	cna	hetloss	BASIS
PD9702	PLCG2	cna	gain	BASIS
TCGA-A2-A25B	PRF1	cna	hetloss	TCGA
TCGA-AN-A0XU	PRF1	cna	hetloss	TCGA
TCGA-AO-A0JL	PRF1	cna	gain	TCGA
TCGA-BH-A0C0	PRF1	cna	gain	TCGA
TCGA-BH-A18R	PRF1	cna	homdel	TCGA
TCGA-C8-A12L	PRF1	cna	hetloss	TCGA
TCGA-D8-A27M	PRF1	cna	hetloss	TCGA
TCGA-E2-A1L7	PRF1	cna	gain	TCGA
TCGA-LL-A5YP	PRF1	cna	hetloss	TCGA
MB-0346	PRF1	cna	gain	METABRIC
MB-5070	PRF1	cna	gain	METABRIC

MB-6098	PRF1	cna	hetloss	METABRIC
MB-7038	PRF1	cna	hetloss	METABRIC
MTS-T0064	PRF1	cna	gain	METABRIC
PD10014	PRF1	cna	gain	BASIS
PD11327	PRF1	cna	gain	BASIS
PD11742	PRF1	cna	hetloss	BASIS
PD13296	PRF1	cna	hetloss	BASIS
PD13299	PRF1	cna	gain	BASIS
PD23574	PRF1	cna	gain	BASIS
PD24186	PRF1	cna	gain	BASIS
PD24202	PRF1	cna	hetloss	BASIS
PD24206	PRF1	cna	gain	BASIS
PD3905	PRF1	cna	gain	BASIS
PD4006	PRF1	cna	gain	BASIS
PD4107	PRF1	cna	gain	BASIS
PD4826	PRF1	cna	gain	BASIS
PD5930	PRF1	cna	gain	BASIS
PD6406	PRF1	cna	hetloss	BASIS
PD7067	PRF1	cna	gain	BASIS
PD7215	PRF1	cna	gain	BASIS
PD8621	PRF1	cna	gain	BASIS
PD8980	PRF1	cna	hetloss	BASIS
PD9004	PRF1	cna	gain	BASIS
PD9585	PRF1	cna	hetloss	BASIS
PD9702	PRF1	cna	gain	BASIS
TCGA-A2-A25B	PRKDC	cna	gain	TCGA
TCGA-AO-A0JL	PRKDC	cna	amp	TCGA
TCGA-BH-A0AW	PRKDC	cna	gain	TCGA
TCGA-BH-A0C0	PRKDC	cna	gain	TCGA
TCGA-BH-A1FU	PRKDC	cna	gain	TCGA
TCGA-C8-A12L	PRKDC	cna	gain	TCGA
TCGA-D8-A27M	PRKDC	cna	amp	TCGA
MB-0346	PRKDC	cna	gain	METABRIC
MB-6098	PRKDC	cna	gain	METABRIC
MB-0420	PRKDC	cna	gain	METABRIC
PD10014	PRKDC	cna	amp	BASIS
PD11327	PRKDC	cna	gain	BASIS
PD13296	PRKDC	cna	hetloss	BASIS
PD13299	PRKDC	cna	gain	BASIS
PD23562	PRKDC	cna	gain	BASIS
PD23574	PRKDC	cna	gain	BASIS
PD23578	PRKDC	cna	hetloss	BASIS
PD24186	PRKDC	cna	gain	BASIS
PD24202	PRKDC	cna	hetloss	BASIS
PD24206	PRKDC	cna	amp	BASIS
PD3905	PRKDC	cna	gain	BASIS
PD4005	PRKDC	cna	hetloss	BASIS

PD4006	PRKDC	cna	gain	BASIS
PD4107	PRKDC	cna	gain	BASIS
PD4826	PRKDC	cna	amp	BASIS
PD5930	PRKDC	cna	gain	BASIS
PD5935	PRKDC	cna	gain	BASIS
PD5945	PRKDC	cna	gain	BASIS
PD5948	PRKDC	cna	gain	BASIS
PD7067	PRKDC	cna	amp	BASIS
PD7215	PRKDC	cna	amp	BASIS
PD8621	PRKDC	cna	gain	BASIS
PD8980	PRKDC	cna	gain	BASIS
PD9004	PRKDC	cna	gain	BASIS
PD9585	PRKDC	cna	gain	BASIS
PD9702	PRKDC	cna	gain	BASIS
TCGA-AN-A0XU	PTPN2	cna	hetloss	TCGA
TCGA-AO-A0JL	PTPN2	cna	gain	TCGA
TCGA-BH-A0AW	PTPN2	cna	amp	TCGA
TCGA-BH-A0C0	PTPN2	cna	hetloss	TCGA
TCGA-BH-A1FU	PTPN2	cna	gain	TCGA
TCGA-C8-A12L	PTPN2	cna	gain	TCGA
TCGA-D8-A27M	PTPN2	cna	hetloss	TCGA
TCGA-LL-A5YP	PTPN2	cna	gain	TCGA
MB-0346	PTPN2	cna	gain	METABRIC
MB-5465	PTPN2	cna	hetloss	METABRIC
MB-7038	PTPN2	cna	gain	METABRIC
MB-0420	PTPN2	cna	gain	METABRIC
PD10014	PTPN2	cna	gain	BASIS
PD11327	PTPN2	cna	homdel	BASIS
PD13296	PTPN2	cna	hetloss	BASIS
PD13299	PTPN2	cna	gain	BASIS
PD23562	PTPN2	cna	gain	BASIS
PD23574	PTPN2	cna	gain	BASIS
PD24186	PTPN2	cna	gain	BASIS
PD24206	PTPN2	cna	gain	BASIS
PD24337	PTPN2	cna	hetloss	BASIS
PD3890	PTPN2	cna	hetloss	BASIS
PD3905	PTPN2	cna	gain	BASIS
PD4006	PTPN2	cna	gain	BASIS
PD4107	PTPN2	cna	gain	BASIS
PD4967	PTPN2	cna	hetloss	BASIS
PD5930	PTPN2	cna	gain	BASIS
PD5935	PTPN2	cna	gain	BASIS
PD5945	PTPN2	cna	amp	BASIS
PD5948	PTPN2	cna	gain	BASIS
PD7067	PTPN2	cna	gain	BASIS
PD7215	PTPN2	cna	gain	BASIS
PD8621	PTPN2	cna	gain	BASIS

PD8980	PTPN2	cna	hetloss	BASIS
PD9004	PTPN2	cna	gain	BASIS
PD9702	PTPN2	cna	gain	BASIS
TCGA-A2-A25B	PTPRT	cna	gain	TCGA
TCGA-AO-A0JL	PTPRT	cna	gain	TCGA
TCGA-BH-A0AW	PTPRT	cna	gain	TCGA
TCGA-BH-A0C0	PTPRT	cna	gain	TCGA
TCGA-C8-A12L	PTPRT	cna	gain	TCGA
TCGA-D8-A27M	PTPRT	cna	gain	TCGA
TCGA-E2-A1L7	PTPRT	cna	gain	TCGA
TCGA-E9-A1NC	PTPRT	cna	hetloss	TCGA
TCGA-LL-A5YP	PTPRT	cna	hetloss	TCGA
MB-0346	PTPRT	cna	gain	METABRIC
MB-2827	PTPRT	cna	amp	METABRIC
MB-5107	PTPRT	cna	gain	METABRIC
MB-6060	PTPRT	cna	amp	METABRIC
MB-6098	PTPRT	cna	hetloss	METABRIC
P-0002858-T01-IM3	PTPRT	cna	amp	MSK-IMPACT
PD11327	PTPRT	cna	hetloss	BASIS
PD13296	PTPRT	cna	hetloss	BASIS
PD13297	PTPRT	cna	hetloss	BASIS
PD13771	PTPRT	cna	gain	BASIS
PD22355	PTPRT	cna	hetloss	BASIS
PD23578	PTPRT	cna	hetloss	BASIS
PD24186	PTPRT	cna	gain	BASIS
PD24202	PTPRT	cna	hetloss	BASIS
PD24206	PTPRT	cna	amp	BASIS
PD3905	PTPRT	cna	gain	BASIS
PD4005	PTPRT	cna	hetloss	BASIS
PD4826	PTPRT	cna	gain	BASIS
PD5935	PTPRT	cna	gain	BASIS
PD5945	PTPRT	cna	amp	BASIS
PD5948	PTPRT	cna	gain	BASIS
PD6731	PTPRT	cna	hetloss	BASIS
PD7067	PTPRT	cna	gain	BASIS
PD7215	PTPRT	cna	amp	BASIS
PD8980	PTPRT	cna	gain	BASIS
PD9004	PTPRT	cna	gain	BASIS
PD9585	PTPRT	cna	hetloss	BASIS
TCGA-A2-A25B	RBM10	cna	gain	TCGA
TCGA-AN-A0XU	RBM10	cna	hetloss	TCGA
TCGA-AO-A0JL	RBM10	cna	gain	TCGA
TCGA-BH-A0AW	RBM10	cna	gain	TCGA
TCGA-E2-A1L7	RBM10	cna	hetloss	TCGA
TCGA-E9-A1NC	RBM10	cna	hetloss	TCGA
MB-0346	RBM10	cna	hetloss	METABRIC
MB-2827	RBM10	cna	hetloss	METABRIC

MB-5465	RBM10	cna	gain	METABRIC
MB-6098	RBM10	cna	hetloss	METABRIC
MB-0420	RBM10	cna	hetloss	METABRIC
PD10014	RBM10	cna	hetloss	BASIS
PD11327	RBM10	cna	homdel	BASIS
PD13296	RBM10	cna	gain	BASIS
PD13299	RBM10	cna	amp	BASIS
PD13771	RBM10	cna	gain	BASIS
PD14442	RBM10	cna	gain	BASIS
PD23562	RBM10	cna	hetloss	BASIS
PD23574	RBM10	cna	gain	BASIS
PD24206	RBM10	cna	homdel	BASIS
PD3905	RBM10	cna	gain	BASIS
PD4005	RBM10	cna	hetloss	BASIS
PD4006	RBM10	cna	hetloss	BASIS
PD4107	RBM10	cna	gain	BASIS
PD4826	RBM10	cna	gain	BASIS
PD4967	RBM10	cna	gain	BASIS
PD5935	RBM10	cna	gain	BASIS
PD5945	RBM10	cna	gain	BASIS
PD5948	RBM10	cna	gain	BASIS
PD6406	RBM10	cna	gain	BASIS
PD6413	RBM10	cna	gain	BASIS
PD7067	RBM10	cna	amp	BASIS
PD7215	RBM10	cna	gain	BASIS
PD8621	RBM10	cna	amp	BASIS
PD8980	RBM10	cna	hetloss	BASIS
PD9004	RBM10	cna	gain	BASIS
TCGA-AN-A0XU	RHOH	cna	hetloss	TCGA
TCGA-AO-A0JL	RHOH	cna	gain	TCGA
TCGA-BH-A0AW	RHOH	cna	hetloss	TCGA
TCGA-BH-A0C0	RHOH	cna	gain	TCGA
TCGA-BH-A1FU	RHOH	cna	homdel	TCGA
TCGA-C8-A12L	RHOH	cna	hetloss	TCGA
TCGA-D8-A27M	RHOH	cna	gain	TCGA
TCGA-E9-A1NC	RHOH	cna	hetloss	TCGA
TCGA-LL-A5YP	RHOH	cna	hetloss	TCGA
MB-0346	RHOH	cna	hetloss	METABRIC
MB-2827	RHOH	cna	homdel	METABRIC
MB-5107	RHOH	cna	hetloss	METABRIC
MB-5465	RHOH	cna	hetloss	METABRIC
MB-6060	RHOH	cna	hetloss	METABRIC
MB-6098	RHOH	cna	hetloss	METABRIC
MB-7048	RHOH	cna	hetloss	METABRIC
MB-0420	RHOH	cna	hetloss	METABRIC
PD11327	RHOH	cna	homdel	BASIS
PD13296	RHOH	cna	hetloss	BASIS

PD13297	RHOH	cna	hetloss	BASIS
PD13299	RHOH	cna	hetloss	BASIS
PD22355	RHOH	cna	hetloss	BASIS
PD24186	RHOH	cna	gain	BASIS
PD24202	RHOH	cna	hetloss	BASIS
PD24206	RHOH	cna	hetloss	BASIS
PD24337	RHOH	cna	hetloss	BASIS
PD4005	RHOH	cna	hetloss	BASIS
PD4006	RHOH	cna	hetloss	BASIS
PD4107	RHOH	cna	hetloss	BASIS
PD4967	RHOH	cna	hetloss	BASIS
PD5930	RHOH	cna	hetloss	BASIS
PD5945	RHOH	cna	gain	BASIS
PD6413	RHOH	cna	hetloss	BASIS
PD7067	RHOH	cna	gain	BASIS
PD8621	RHOH	cna	gain	BASIS
PD8980	RHOH	cna	hetloss	BASIS
TCGA-A2-A25B	RICTOR	cna	amp	TCGA
TCGA-AN-A0XU	RICTOR	cna	gain	TCGA
TCGA-AO-A0JL	RICTOR	cna	gain	TCGA
TCGA-BH-A0AW	RICTOR	cna	gain	TCGA
TCGA-BH-A0C0	RICTOR	cna	gain	TCGA
TCGA-BH-A1FU	RICTOR	cna	amp	TCGA
TCGA-E9-A1NC	RICTOR	cna	hetloss	TCGA
TCGA-LL-A5YP	RICTOR	cna	amp	TCGA
MB-0346	RICTOR	cna	gain	METABRIC
MB-5070	RICTOR	cna	gain	METABRIC
MB-6060	RICTOR	cna	gain	METABRIC
MB-6098	RICTOR	cna	amp	METABRIC
MB-0420	RICTOR	cna	gain	METABRIC
PD10014	RICTOR	cna	hetloss	BASIS
PD11327	RICTOR	cna	gain	BASIS
PD13299	RICTOR	cna	gain	BASIS
PD23562	RICTOR	cna	gain	BASIS
PD23574	RICTOR	cna	gain	BASIS
PD24186	RICTOR	cna	gain	BASIS
PD24206	RICTOR	cna	gain	BASIS
PD24337	RICTOR	cna	hetloss	BASIS
PD3890	RICTOR	cna	gain	BASIS
PD3905	RICTOR	cna	gain	BASIS
PD4005	RICTOR	cna	hetloss	BASIS
PD4006	RICTOR	cna	gain	BASIS
PD4107	RICTOR	cna	gain	BASIS
PD4826	RICTOR	cna	gain	BASIS
PD5930	RICTOR	cna	gain	BASIS
PD5945	RICTOR	cna	amp	BASIS
PD5948	RICTOR	cna	gain	BASIS

PD6406	RICTOR	cna	hetloss	BASIS
PD6413	RICTOR	cna	gain	BASIS
PD7215	RICTOR	cna	amp	BASIS
PD8980	RICTOR	cna	gain	BASIS
PD9004	RICTOR	cna	gain	BASIS
PD9585	RICTOR	cna	gain	BASIS
TCGA-A2-A25B	RPL11	cna	hetloss	TCGA
TCGA-AN-A0XU	RPL11	cna	hetloss	TCGA
TCGA-AO-A0JL	RPL11	cna	hetloss	TCGA
TCGA-BH-A0AW	RPL11	cna	gain	TCGA
TCGA-BH-A0C0	RPL11	cna	hetloss	TCGA
TCGA-BH-A18R	RPL11	cna	homdel	TCGA
TCGA-BH-A1FU	RPL11	cna	gain	TCGA
TCGA-C8-A12L	RPL11	cna	hetloss	TCGA
TCGA-D8-A27M	RPL11	cna	gain	TCGA
TCGA-E2-A1L7	RPL11	cna	hetloss	TCGA
TCGA-E9-A1NC	RPL11	cna	hetloss	TCGA
TCGA-LL-A5YP	RPL11	cna	hetloss	TCGA
MB-2827	RPL11	cna	hetloss	METABRIC
MB-5070	RPL11	cna	hetloss	METABRIC
MB-5465	RPL11	cna	hetloss	METABRIC
MB-6060	RPL11	cna	hetloss	METABRIC
MB-6098	RPL11	cna	hetloss	METABRIC
MB-6271	RPL11	cna	hetloss	METABRIC
MB-0420	RPL11	cna	hetloss	METABRIC
PD10014	RPL11	cna	gain	BASIS
PD11742	RPL11	cna	hetloss	BASIS
PD13296	RPL11	cna	hetloss	BASIS
PD13771	RPL11	cna	hetloss	BASIS
PD22355	RPL11	cna	hetloss	BASIS
PD24186	RPL11	cna	gain	BASIS
PD24206	RPL11	cna	hetloss	BASIS
PD24337	RPL11	cna	hetloss	BASIS
PD3890	RPL11	cna	hetloss	BASIS
PD3905	RPL11	cna	gain	BASIS
PD4006	RPL11	cna	gain	BASIS
PD4826	RPL11	cna	gain	BASIS
PD5945	RPL11	cna	amp	BASIS
PD7067	RPL11	cna	gain	BASIS
PD7215	RPL11	cna	gain	BASIS
PD9004	RPL11	cna	hetloss	BASIS
PD9585	RPL11	cna	hetloss	BASIS
TCGA-A2-A25B	RPL22	cna	hetloss	TCGA
TCGA-AN-A0XU	RPL22	cna	hetloss	TCGA
TCGA-AO-A0JL	RPL22	cna	hetloss	TCGA
TCGA-BH-A0AW	RPL22	cna	gain	TCGA
TCGA-BH-A0C0	RPL22	cna	gain	TCGA

TCGA-BH-A18R	RPL22	cna	homdel	TCGA
TCGA-BH-A1FU	RPL22	cna	gain	TCGA
TCGA-C8-A12L	RPL22	cna	hetloss	TCGA
TCGA-D8-A27M	RPL22	cna	gain	TCGA
TCGA-E2-A1L7	RPL22	cna	hetloss	TCGA
TCGA-E9-A1NC	RPL22	cna	hetloss	TCGA
TCGA-LL-A5YP	RPL22	cna	amp	TCGA
MB-2827	RPL22	cna	hetloss	METABRIC
MB-5070	RPL22	cna	hetloss	METABRIC
MB-5465	RPL22	cna	hetloss	METABRIC
MB-6060	RPL22	cna	hetloss	METABRIC
MB-6098	RPL22	cna	hetloss	METABRIC
MB-0420	RPL22	cna	hetloss	METABRIC
PD10014	RPL22	cna	gain	BASIS
PD11327	RPL22	cna	gain	BASIS
PD11742	RPL22	cna	hetloss	BASIS
PD22355	RPL22	cna	hetloss	BASIS
PD24202	RPL22	cna	hetloss	BASIS
PD24206	RPL22	cna	gain	BASIS
PD24337	RPL22	cna	hetloss	BASIS
PD3890	RPL22	cna	hetloss	BASIS
PD3905	RPL22	cna	gain	BASIS
PD4107	RPL22	cna	gain	BASIS
PD4826	RPL22	cna	gain	BASIS
PD5945	RPL22	cna	gain	BASIS
PD6406	RPL22	cna	hetloss	BASIS
PD7067	RPL22	cna	gain	BASIS
PD7215	RPL22	cna	gain	BASIS
PD8621	RPL22	cna	gain	BASIS
PD8980	RPL22	cna	hetloss	BASIS
PD9004	RPL22	cna	hetloss	BASIS
TCGA-AN-A0XU	RPN1	cna	gain	TCGA
TCGA-BH-A0C0	RPN1	cna	gain	TCGA
TCGA-BH-A18R	RPN1	cna	gain	TCGA
TCGA-BH-A1FU	RPN1	cna	hetloss	TCGA
TCGA-C8-A12L	RPN1	cna	gain	TCGA
TCGA-E2-A1L7	RPN1	cna	hetloss	TCGA
TCGA-LL-A5YP	RPN1	cna	gain	TCGA
MB-0346	RPN1	cna	hetloss	METABRIC
MB-5070	RPN1	cna	gain	METABRIC
MB-5107	RPN1	cna	gain	METABRIC
MB-5465	RPN1	cna	gain	METABRIC
MB-6098	RPN1	cna	hetloss	METABRIC
MB-7038	RPN1	cna	gain	METABRIC
PD10014	RPN1	cna	gain	BASIS
PD11327	RPN1	cna	gain	BASIS
PD13297	RPN1	cna	hetloss	BASIS

PD13299	RPN1	cna	gain	BASIS
PD13771	RPN1	cna	hetloss	BASIS
PD23561	RPN1	cna	hetloss	BASIS
PD23574	RPN1	cna	gain	BASIS
PD24186	RPN1	cna	gain	BASIS
PD24206	RPN1	cna	gain	BASIS
PD3905	RPN1	cna	gain	BASIS
PD4005	RPN1	cna	hetloss	BASIS
PD4107	RPN1	cna	gain	BASIS
PD4826	RPN1	cna	gain	BASIS
PD5935	RPN1	cna	gain	BASIS
PD5945	RPN1	cna	gain	BASIS
PD5948	RPN1	cna	gain	BASIS
PD6731	RPN1	cna	gain	BASIS
PD7067	RPN1	cna	gain	BASIS
PD7215	RPN1	cna	gain	BASIS
PD8621	RPN1	cna	gain	BASIS
PD8980	RPN1	cna	gain	BASIS
PD9004	RPN1	cna	gain	BASIS
PD9702	RPN1	cna	gain	BASIS
TCGA-AN-A0XU	SH3GL1	cna	hetloss	TCGA
TCGA-AO-A0JL	SH3GL1	cna	gain	TCGA
TCGA-BH-A0C0	SH3GL1	cna	gain	TCGA
TCGA-BH-A1FU	SH3GL1	cna	hetloss	TCGA
TCGA-C8-A12L	SH3GL1	cna	amp	TCGA
TCGA-D8-A27M	SH3GL1	cna	hetloss	TCGA
TCGA-E9-A1NC	SH3GL1	cna	hetloss	TCGA
TCGA-EW-A1OX	SH3GL1	cna	hetloss	TCGA
TCGA-LL-A5YP	SH3GL1	cna	hetloss	TCGA
MB-0346	SH3GL1	cna	gain	METABRIC
MB-2827	SH3GL1	cna	hetloss	METABRIC
MB-5070	SH3GL1	cna	hetloss	METABRIC
MB-5465	SH3GL1	cna	gain	METABRIC
MB-6060	SH3GL1	cna	gain	METABRIC
MB-6098	SH3GL1	cna	hetloss	METABRIC
MB-7038	SH3GL1	cna	hetloss	METABRIC
MB-0420	SH3GL1	cna	hetloss	METABRIC
PD10014	SH3GL1	cna	hetloss	BASIS
PD11742	SH3GL1	cna	hetloss	BASIS
PD14442	SH3GL1	cna	hetloss	BASIS
PD22355	SH3GL1	cna	hetloss	BASIS
PD23574	SH3GL1	cna	gain	BASIS
PD24202	SH3GL1	cna	gain	BASIS
PD24206	SH3GL1	cna	hetloss	BASIS
PD3905	SH3GL1	cna	gain	BASIS
PD4006	SH3GL1	cna	gain	BASIS
PD4826	SH3GL1	cna	gain	BASIS

PD4967	SH3GL1	cna	hetloss	BASIS
PD5930	SH3GL1	cna	gain	BASIS
PD5945	SH3GL1	cna	gain	BASIS
PD5948	SH3GL1	cna	gain	BASIS
PD7067	SH3GL1	cna	gain	BASIS
PD8621	SH3GL1	cna	gain	BASIS
PD8980	SH3GL1	cna	gain	BASIS
PD9004	SH3GL1	cna	hetloss	BASIS
PD9702	SH3GL1	cna	gain	BASIS
TCGA-A2-A25B	SMC1A	cna	gain	TCGA
TCGA-AN-A0XU	SMC1A	cna	hetloss	TCGA
TCGA-AO-A0JL	SMC1A	cna	gain	TCGA
TCGA-BH-A0AW	SMC1A	cna	gain	TCGA
TCGA-E2-A1L7	SMC1A	cna	hetloss	TCGA
TCGA-E9-A1NC	SMC1A	cna	hetloss	TCGA
MB-0346	SMC1A	cna	gain	METABRIC
MB-2827	SMC1A	cna	hetloss	METABRIC
MB-5465	SMC1A	cna	gain	METABRIC
MB-6098	SMC1A	cna	hetloss	METABRIC
MB-0420	SMC1A	cna	hetloss	METABRIC
PD10014	SMC1A	cna	hetloss	BASIS
PD11327	SMC1A	cna	homdel	BASIS
PD13296	SMC1A	cna	gain	BASIS
PD13297	SMC1A	cna	gain	BASIS
PD13299	SMC1A	cna	gain	BASIS
PD13771	SMC1A	cna	gain	BASIS
PD14442	SMC1A	cna	gain	BASIS
PD23562	SMC1A	cna	hetloss	BASIS
PD23574	SMC1A	cna	gain	BASIS
PD24206	SMC1A	cna	amp	BASIS
PD3905	SMC1A	cna	gain	BASIS
PD4005	SMC1A	cna	hetloss	BASIS
PD4006	SMC1A	cna	hetloss	BASIS
PD4107	SMC1A	cna	gain	BASIS
PD4826	SMC1A	cna	gain	BASIS
PD4967	SMC1A	cna	gain	BASIS
PD5935	SMC1A	cna	gain	BASIS
PD5945	SMC1A	cna	gain	BASIS
PD5948	SMC1A	cna	gain	BASIS
PD6406	SMC1A	cna	gain	BASIS
PD6413	SMC1A	cna	gain	BASIS
PD7067	SMC1A	cna	gain	BASIS
PD7215	SMC1A	cna	amp	BASIS
PD8621	SMC1A	cna	amp	BASIS
PD9004	SMC1A	cna	gain	BASIS
TCGA-A2-A25B	SOX10	cna	hetloss	TCGA
TCGA-BH-A0AW	SOX10	cna	hetloss	TCGA

TCGA-BH-A1FU	SOX10	cna	gain	TCGA
TCGA-C8-A12L	SOX10	cna	gain	TCGA
TCGA-D8-A27M	SOX10	cna	gain	TCGA
TCGA-E9-A1NC	SOX10	cna	gain	TCGA
TCGA-LL-A5YP	SOX10	cna	amp	TCGA
MB-0346	SOX10	cna	gain	METABRIC
MB-5465	SOX10	cna	gain	METABRIC
MB-6098	SOX10	cna	gain	METABRIC
MTS-T0064	SOX10	cna	gain	METABRIC
PD11742	SOX10	cna	hetloss	BASIS
PD13296	SOX10	cna	gain	BASIS
PD13297	SOX10	cna	gain	BASIS
PD13299	SOX10	cna	gain	BASIS
PD14442	SOX10	cna	hetloss	BASIS
PD22355	SOX10	cna	gain	BASIS
PD23562	SOX10	cna	gain	BASIS
PD23574	SOX10	cna	gain	BASIS
PD23578	SOX10	cna	gain	BASIS
PD24206	SOX10	cna	hetloss	BASIS
PD3905	SOX10	cna	gain	BASIS
PD4005	SOX10	cna	gain	BASIS
PD4006	SOX10	cna	gain	BASIS
PD4107	SOX10	cna	gain	BASIS
PD4826	SOX10	cna	gain	BASIS
PD4967	SOX10	cna	hetloss	BASIS
PD5935	SOX10	cna	gain	BASIS
PD5945	SOX10	cna	amp	BASIS
PD5948	SOX10	cna	gain	BASIS
PD6731	SOX10	cna	hetloss	BASIS
PD7067	SOX10	cna	gain	BASIS
PD7215	SOX10	cna	gain	BASIS
PD8621	SOX10	cna	gain	BASIS
PD9004	SOX10	cna	gain	BASIS
PD9702	SOX10	cna	gain	BASIS
TCGA-AN-A0XU	SRGAP3	cna	gain	TCGA
TCGA-BH-A0C0	SRGAP3	cna	gain	TCGA
TCGA-BH-A18R	SRGAP3	cna	gain	TCGA
TCGA-BH-A1FU	SRGAP3	cna	hetloss	TCGA
TCGA-C8-A12L	SRGAP3	cna	gain	TCGA
TCGA-D8-A27M	SRGAP3	cna	hetloss	TCGA
TCGA-E9-A1NC	SRGAP3	cna	hetloss	TCGA
TCGA-LL-A5YP	SRGAP3	cna	amp	TCGA
MB-6060	SRGAP3	cna	amp	METABRIC
MB-7038	SRGAP3	cna	hetloss	METABRIC
MB-7048	SRGAP3	cna	hetloss	METABRIC
MB-0420	SRGAP3	cna	hetloss	METABRIC
PD10014	SRGAP3	cna	hetloss	BASIS

PD11327	SRGAP3	cna	gain	BASIS
PD11742	SRGAP3	cna	gain	BASIS
PD13296	SRGAP3	cna	gain	BASIS
PD13299	SRGAP3	cna	gain	BASIS
PD13771	SRGAP3	cna	gain	BASIS
PD23561	SRGAP3	cna	hetloss	BASIS
PD23562	SRGAP3	cna	gain	BASIS
PD23574	SRGAP3	cna	gain	BASIS
PD24186	SRGAP3	cna	amp	BASIS
PD24206	SRGAP3	cna	gain	BASIS
PD3905	SRGAP3	cna	gain	BASIS
PD4006	SRGAP3	cna	gain	BASIS
PD4826	SRGAP3	cna	gain	BASIS
PD5945	SRGAP3	cna	amp	BASIS
PD5948	SRGAP3	cna	gain	BASIS
PD6406	SRGAP3	cna	hetloss	BASIS
PD6731	SRGAP3	cna	hetloss	BASIS
PD7067	SRGAP3	cna	gain	BASIS
PD7215	SRGAP3	cna	gain	BASIS
PD8980	SRGAP3	cna	gain	BASIS
PD9004	SRGAP3	cna	gain	BASIS
PD9585	SRGAP3	cna	hetloss	BASIS
PD9702	SRGAP3	cna	gain	BASIS
TCGA-A2-A25B	STK40	cna	hetloss	TCGA
TCGA-AN-A0XU	STK40	cna	hetloss	TCGA
TCGA-AO-A0JL	STK40	cna	hetloss	TCGA
TCGA-BH-A0AW	STK40	cna	gain	TCGA
TCGA-BH-A0C0	STK40	cna	gain	TCGA
TCGA-BH-A1FU	STK40	cna	gain	TCGA
TCGA-C8-A12L	STK40	cna	hetloss	TCGA
TCGA-D8-A27M	STK40	cna	gain	TCGA
TCGA-E2-A1L7	STK40	cna	gain	TCGA
TCGA-LL-A5YP	STK40	cna	gain	TCGA
MB-0346	STK40	cna	hetloss	METABRIC
MB-5465	STK40	cna	hetloss	METABRIC
MB-6060	STK40	cna	hetloss	METABRIC
MB-6098	STK40	cna	hetloss	METABRIC
MB-7038	STK40	cna	gain	METABRIC
MB-0420	STK40	cna	hetloss	METABRIC
PD10014	STK40	cna	gain	BASIS
PD13296	STK40	cna	gain	BASIS
PD13299	STK40	cna	gain	BASIS
PD13771	STK40	cna	gain	BASIS
PD24186	STK40	cna	gain	BASIS
PD24206	STK40	cna	gain	BASIS
PD24337	STK40	cna	hetloss	BASIS
PD3905	STK40	cna	gain	BASIS

PD4006	STK40	cna	gain	BASIS
PD4107	STK40	cna	gain	BASIS
PD4826	STK40	cna	gain	BASIS
PD5935	STK40	cna	gain	BASIS
PD5945	STK40	cna	amp	BASIS
PD7067	STK40	cna	gain	BASIS
PD7215	STK40	cna	gain	BASIS
PD8621	STK40	cna	gain	BASIS
PD8980	STK40	cna	gain	BASIS
PD9004	STK40	cna	hetloss	BASIS
PD9585	STK40	cna	hetloss	BASIS
PD9702	STK40	cna	gain	BASIS
TCGA-A2-A25B	TBX3	cna	gain	TCGA
TCGA-AN-A0XU	TBX3	cna	hetloss	TCGA
TCGA-AO-A0JL	TBX3	cna	hetloss	TCGA
TCGA-BH-A0C0	TBX3	cna	gain	TCGA
TCGA-BH-A18R	TBX3	cna	gain	TCGA
TCGA-BH-A1FU	TBX3	cna	hetloss	TCGA
TCGA-D8-A27M	TBX3	cna	hetloss	TCGA
TCGA-E2-A1L7	TBX3	cna	hetloss	TCGA
TCGA-LL-A5YP	TBX3	cna	hetloss	TCGA
MB-0346	TBX3	cna	gain	METABRIC
MB-2827	TBX3	cna	hetloss	METABRIC
MB-5070	TBX3	cna	hetloss	METABRIC
MB-7048	TBX3	cna	hetloss	METABRIC
PD10014	TBX3	cna	gain	BASIS
PD11327	TBX3	cna	gain	BASIS
PD11742	TBX3	cna	hetloss	BASIS
PD13296	TBX3	cna	hetloss	BASIS
PD22355	TBX3	cna	hetloss	BASIS
PD23574	TBX3	cna	gain	BASIS
PD23578	TBX3	cna	hetloss	BASIS
PD24206	TBX3	cna	homdel	BASIS
PD3890	TBX3	cna	hetloss	BASIS
PD3905	TBX3	cna	gain	BASIS
PD4005	TBX3	cna	hetloss	BASIS
PD4006	TBX3	cna	gain	BASIS
PD4107	TBX3	cna	gain	BASIS
PD4826	TBX3	cna	gain	BASIS
PD5945	TBX3	cna	gain	BASIS
PD5948	TBX3	cna	gain	BASIS
PD6406	TBX3	cna	hetloss	BASIS
PD6413	TBX3	cna	hetloss	BASIS
PD6731	TBX3	cna	hetloss	BASIS
PD7067	TBX3	cna	gain	BASIS
PD9004	TBX3	cna	hetloss	BASIS
PD9585	TBX3	cna	hetloss	BASIS

PD9702	TBX3	cna	gain	BASIS
TCGA-AN-A0XU	TCF3	cna	hetloss	TCGA
TCGA-AO-A0JL	TCF3	cna	gain	TCGA
TCGA-BH-A0C0	TCF3	cna	gain	TCGA
TCGA-BH-A1FU	TCF3	cna	hetloss	TCGA
TCGA-C8-A12L	TCF3	cna	hetloss	TCGA
TCGA-D8-A27M	TCF3	cna	hetloss	TCGA
TCGA-E9-A1NC	TCF3	cna	hetloss	TCGA
TCGA-EW-A1OX	TCF3	cna	hetloss	TCGA
TCGA-LL-A5YP	TCF3	cna	hetloss	TCGA
MB-0346	TCF3	cna	gain	METABRIC
MB-2827	TCF3	cna	hetloss	METABRIC
MB-5070	TCF3	cna	hetloss	METABRIC
MB-6098	TCF3	cna	hetloss	METABRIC
MB-7038	TCF3	cna	hetloss	METABRIC
MB-0420	TCF3	cna	hetloss	METABRIC
P-0009557-T01-IM5	TCF3	cna	homdel	MSK-IMPACT
PD11742	TCF3	cna	hetloss	BASIS
PD14442	TCF3	cna	hetloss	BASIS
PD22355	TCF3	cna	hetloss	BASIS
PD23574	TCF3	cna	gain	BASIS
PD23578	TCF3	cna	hetloss	BASIS
PD24202	TCF3	cna	hetloss	BASIS
PD24206	TCF3	cna	hetloss	BASIS
PD3890	TCF3	cna	hetloss	BASIS
PD3905	TCF3	cna	gain	BASIS
PD4006	TCF3	cna	gain	BASIS
PD4826	TCF3	cna	hetloss	BASIS
PD4967	TCF3	cna	hetloss	BASIS
PD5930	TCF3	cna	gain	BASIS
PD5945	TCF3	cna	gain	BASIS
PD5948	TCF3	cna	gain	BASIS
PD7067	TCF3	cna	gain	BASIS
PD8621	TCF3	cna	gain	BASIS
PD8980	TCF3	cna	gain	BASIS
PD9004	TCF3	cna	hetloss	BASIS
PD9585	TCF3	cna	hetloss	BASIS
TCGA-A2-A25B	TET1	cna	hetloss	TCGA
TCGA-AN-A0XU	TET1	cna	hetloss	TCGA
TCGA-AO-A0JL	TET1	cna	gain	TCGA
TCGA-BH-A0C0	TET1	cna	gain	TCGA
TCGA-C8-A12L	TET1	cna	hetloss	TCGA
TCGA-D8-A27M	TET1	cna	hetloss	TCGA
TCGA-E2-A1L7	TET1	cna	gain	TCGA
TCGA-LL-A5YP	TET1	cna	gain	TCGA
MB-0346	TET1	cna	hetloss	METABRIC
MB-2827	TET1	cna	hetloss	METABRIC

MB-6098	TET1	cna	hetloss	METABRIC
MB-7038	TET1	cna	hetloss	METABRIC
MB-7048	TET1	cna	hetloss	METABRIC
MTS-T0064	TET1	cna	gain	METABRIC
PD10014	TET1	cna	gain	BASIS
PD11327	TET1	cna	hetloss	BASIS
PD11742	TET1	cna	hetloss	BASIS
PD13296	TET1	cna	hetloss	BASIS
PD13299	TET1	cna	gain	BASIS
PD23574	TET1	cna	gain	BASIS
PD24186	TET1	cna	gain	BASIS
PD24202	TET1	cna	hetloss	BASIS
PD24206	TET1	cna	hetloss	BASIS
PD3905	TET1	cna	gain	BASIS
PD4006	TET1	cna	gain	BASIS
PD4107	TET1	cna	gain	BASIS
PD4826	TET1	cna	gain	BASIS
PD5930	TET1	cna	gain	BASIS
PD6406	TET1	cna	hetloss	BASIS
PD7067	TET1	cna	gain	BASIS
PD7215	TET1	cna	gain	BASIS
PD8621	TET1	cna	gain	BASIS
PD8980	TET1	cna	hetloss	BASIS
PD9004	TET1	cna	gain	BASIS
PD9585	TET1	cna	hetloss	BASIS
PD9702	TET1	cna	gain	BASIS
TCGA-A2-A25B	THRAP3	cna	hetloss	TCGA
TCGA-AN-A0XU	THRAP3	cna	hetloss	TCGA
TCGA-AO-A0JL	THRAP3	cna	hetloss	TCGA
TCGA-BH-A0AW	THRAP3	cna	gain	TCGA
TCGA-BH-A0C0	THRAP3	cna	gain	TCGA
TCGA-BH-A1FU	THRAP3	cna	gain	TCGA
TCGA-C8-A12L	THRAP3	cna	hetloss	TCGA
TCGA-D8-A27M	THRAP3	cna	gain	TCGA
TCGA-E2-A1L7	THRAP3	cna	gain	TCGA
TCGA-LL-A5YP	THRAP3	cna	gain	TCGA
MB-0346	THRAP3	cna	hetloss	METABRIC
MB-5465	THRAP3	cna	hetloss	METABRIC
MB-6060	THRAP3	cna	hetloss	METABRIC
MB-6098	THRAP3	cna	hetloss	METABRIC
MB-7038	THRAP3	cna	gain	METABRIC
MB-0420	THRAP3	cna	hetloss	METABRIC
PD10014	THRAP3	cna	gain	BASIS
PD13296	THRAP3	cna	gain	BASIS
PD13299	THRAP3	cna	gain	BASIS
PD13771	THRAP3	cna	gain	BASIS
PD24186	THRAP3	cna	gain	BASIS

PD24206	THRAP3	cna	gain	BASIS
PD24337	THRAP3	cna	hetloss	BASIS
PD3905	THRAP3	cna	gain	BASIS
PD4006	THRAP3	cna	gain	BASIS
PD4107	THRAP3	cna	gain	BASIS
PD4826	THRAP3	cna	gain	BASIS
PD5935	THRAP3	cna	gain	BASIS
PD5945	THRAP3	cna	amp	BASIS
PD7067	THRAP3	cna	gain	BASIS
PD7215	THRAP3	cna	gain	BASIS
PD8621	THRAP3	cna	gain	BASIS
PD8980	THRAP3	cna	gain	BASIS
PD9004	THRAP3	cna	hetloss	BASIS
PD9585	THRAP3	cna	hetloss	BASIS
PD9702	THRAP3	cna	gain	BASIS
TCGA-AN-A0XU	TICAM1	cna	hetloss	TCGA
TCGA-AO-A0JL	TICAM1	cna	gain	TCGA
TCGA-BH-A0C0	TICAM1	cna	gain	TCGA
TCGA-BH-A1FU	TICAM1	cna	hetloss	TCGA
TCGA-C8-A12L	TICAM1	cna	gain	TCGA
TCGA-D8-A27M	TICAM1	cna	hetloss	TCGA
TCGA-E9-A1NC	TICAM1	cna	hetloss	TCGA
TCGA-EW-A1OX	TICAM1	cna	hetloss	TCGA
TCGA-LL-A5YP	TICAM1	cna	hetloss	TCGA
MB-0346	TICAM1	cna	gain	METABRIC
MB-2827	TICAM1	cna	hetloss	METABRIC
MB-5070	TICAM1	cna	hetloss	METABRIC
MB-5465	TICAM1	cna	gain	METABRIC
MB-6060	TICAM1	cna	gain	METABRIC
MB-6098	TICAM1	cna	hetloss	METABRIC
MB-7038	TICAM1	cna	hetloss	METABRIC
MB-0420	TICAM1	cna	hetloss	METABRIC
PD10014	TICAM1	cna	hetloss	BASIS
PD11742	TICAM1	cna	hetloss	BASIS
PD14442	TICAM1	cna	hetloss	BASIS
PD23574	TICAM1	cna	gain	BASIS
PD24202	TICAM1	cna	gain	BASIS
PD24206	TICAM1	cna	hetloss	BASIS
PD3905	TICAM1	cna	gain	BASIS
PD4006	TICAM1	cna	gain	BASIS
PD4107	TICAM1	cna	gain	BASIS
PD4826	TICAM1	cna	gain	BASIS
PD4967	TICAM1	cna	hetloss	BASIS
PD5930	TICAM1	cna	gain	BASIS
PD5945	TICAM1	cna	gain	BASIS
PD5948	TICAM1	cna	gain	BASIS
PD7067	TICAM1	cna	gain	BASIS

PD8621	TICAM1	cna	gain	BASIS
PD8980	TICAM1	cna	gain	BASIS
PD9004	TICAM1	cna	hetloss	BASIS
PD9702	TICAM1	cna	gain	BASIS
TCGA-AN-A0XU	TNFRSF17	cna	gain	TCGA
TCGA-AO-A0JL	TNFRSF17	cna	gain	TCGA
TCGA-BH-A0AW	TNFRSF17	cna	gain	TCGA
TCGA-BH-A0C0	TNFRSF17	cna	gain	TCGA
TCGA-C8-A12L	TNFRSF17	cna	gain	TCGA
TCGA-E2-A1L7	TNFRSF17	cna	hetloss	TCGA
TCGA-LL-A5YP	TNFRSF17	cna	gain	TCGA
MB-0346	TNFRSF17	cna	gain	METABRIC
MB-6060	TNFRSF17	cna	gain	METABRIC
MB-6098	TNFRSF17	cna	hetloss	METABRIC
MB-6271	TNFRSF17	cna	gain	METABRIC
MB-7032	TNFRSF17	cna	gain	METABRIC
MB-7038	TNFRSF17	cna	hetloss	METABRIC
MB-0420	TNFRSF17	cna	hetloss	METABRIC
PD10014	TNFRSF17	cna	hetloss	BASIS
PD11327	TNFRSF17	cna	hetloss	BASIS
PD11742	TNFRSF17	cna	gain	BASIS
PD13296	TNFRSF17	cna	gain	BASIS
PD13297	TNFRSF17	cna	hetloss	BASIS
PD13299	TNFRSF17	cna	gain	BASIS
PD22355	TNFRSF17	cna	hetloss	BASIS
PD24202	TNFRSF17	cna	hetloss	BASIS
PD24206	TNFRSF17	cna	gain	BASIS
PD24337	TNFRSF17	cna	hetloss	BASIS
PD3905	TNFRSF17	cna	gain	BASIS
PD4005	TNFRSF17	cna	gain	BASIS
PD4107	TNFRSF17	cna	gain	BASIS
PD4826	TNFRSF17	cna	gain	BASIS
PD5935	TNFRSF17	cna	gain	BASIS
PD5945	TNFRSF17	cna	gain	BASIS
PD5948	TNFRSF17	cna	gain	BASIS
PD7067	TNFRSF17	cna	gain	BASIS
PD7215	TNFRSF17	cna	amp	BASIS
PD9004	TNFRSF17	cna	gain	BASIS
PD9585	TNFRSF17	cna	hetloss	BASIS
PD9702	TNFRSF17	cna	gain	BASIS
TCGA-A2-A25B	TP53	cna	hetloss	TCGA
TCGA-AN-A0XU	TP53	cna	hetloss	TCGA
TCGA-AO-A0JL	TP53	cna	gain	TCGA
TCGA-BH-A0AW	TP53	cna	hetloss	TCGA
TCGA-BH-A0C0	TP53	cna	hetloss	TCGA
TCGA-C8-A12L	TP53	cna	hetloss	TCGA
TCGA-D8-A27M	TP53	cna	hetloss	TCGA

TCGA-E2-A1L7	TP53	cna	hetloss	TCGA
TCGA-E9-A1NC	TP53	cna	hetloss	TCGA
TCGA-LL-A5YP	TP53	cna	gain	TCGA
MB-0346	TP53	cna	hetloss	METABRIC
MB-2827	TP53	cna	hetloss	METABRIC
MB-5070	TP53	cna	hetloss	METABRIC
MB-5107	TP53	cna	hetloss	METABRIC
MB-6271	TP53	cna	hetloss	METABRIC
MB-7048	TP53	cna	hetloss	METABRIC
MB-0420	TP53	cna	hetloss	METABRIC
P-0002591-T01-IM3	TP53	cna	homdel	MSK-IMPACT
PD11742	TP53	cna	hetloss	BASIS
PD13296	TP53	cna	hetloss	BASIS
PD13771	TP53	cna	hetloss	BASIS
PD22355	TP53	cna	hetloss	BASIS
PD24202	TP53	cna	hetloss	BASIS
PD24206	TP53	cna	hetloss	BASIS
PD24337	TP53	cna	hetloss	BASIS
PD3890	TP53	cna	hetloss	BASIS
PD3905	TP53	cna	gain	BASIS
PD4005	TP53	cna	homdel	BASIS
PD4826	TP53	cna	hetloss	BASIS
PD4967	TP53	cna	hetloss	BASIS
PD5945	TP53	cna	gain	BASIS
PD6406	TP53	cna	hetloss	BASIS
PD6413	TP53	cna	hetloss	BASIS
PD6731	TP53	cna	hetloss	BASIS
PD7067	TP53	cna	gain	BASIS
PD9585	TP53	cna	hetloss	BASIS
TCGA-A2-A25B	TRIOBP	cna	hetloss	TCGA
TCGA-BH-A0AW	TRIOBP	cna	hetloss	TCGA
TCGA-BH-A1FU	TRIOBP	cna	gain	TCGA
TCGA-C8-A12L	TRIOBP	cna	gain	TCGA
TCGA-D8-A27M	TRIOBP	cna	gain	TCGA
TCGA-E9-A1NC	TRIOBP	cna	gain	TCGA
TCGA-LL-A5YP	TRIOBP	cna	gain	TCGA
MB-0346	TRIOBP	cna	gain	METABRIC
MB-5465	TRIOBP	cna	gain	METABRIC
MB-6098	TRIOBP	cna	gain	METABRIC
MTS-T0064	TRIOBP	cna	gain	METABRIC
PD11742	TRIOBP	cna	hetloss	BASIS
PD13296	TRIOBP	cna	gain	BASIS
PD13297	TRIOBP	cna	gain	BASIS
PD13299	TRIOBP	cna	gain	BASIS
PD14442	TRIOBP	cna	hetloss	BASIS
PD22355	TRIOBP	cna	gain	BASIS
PD23562	TRIOBP	cna	gain	BASIS

PD23574	TRIOBP	cna	gain	BASIS
PD23578	TRIOBP	cna	gain	BASIS
PD24206	TRIOBP	cna	hetloss	BASIS
PD3905	TRIOBP	cna	gain	BASIS
PD4005	TRIOBP	cna	gain	BASIS
PD4006	TRIOBP	cna	gain	BASIS
PD4107	TRIOBP	cna	gain	BASIS
PD4826	TRIOBP	cna	gain	BASIS
PD4967	TRIOBP	cna	hetloss	BASIS
PD5935	TRIOBP	cna	gain	BASIS
PD5945	TRIOBP	cna	amp	BASIS
PD5948	TRIOBP	cna	gain	BASIS
PD6731	TRIOBP	cna	hetloss	BASIS
PD7067	TRIOBP	cna	gain	BASIS
PD7215	TRIOBP	cna	gain	BASIS
PD8621	TRIOBP	cna	gain	BASIS
PD9004	TRIOBP	cna	gain	BASIS
PD9702	TRIOBP	cna	gain	BASIS
TCGA-AN-A0XU	TYMS	cna	hetloss	TCGA
TCGA-AO-A0JL	TYMS	cna	gain	TCGA
TCGA-BH-A0AW	TYMS	cna	hetloss	TCGA
TCGA-BH-A0C0	TYMS	cna	hetloss	TCGA
TCGA-BH-A1FU	TYMS	cna	gain	TCGA
TCGA-C8-A12L	TYMS	cna	gain	TCGA
TCGA-D8-A27M	TYMS	cna	hetloss	TCGA
TCGA-LL-A5YP	TYMS	cna	gain	TCGA
MB-0346	TYMS	cna	gain	METABRIC
MB-5107	TYMS	cna	hetloss	METABRIC
MB-5465	TYMS	cna	hetloss	METABRIC
MB-6098	TYMS	cna	hetloss	METABRIC
MB-0420	TYMS	cna	gain	METABRIC
MTS-T0064	TYMS	cna	hetloss	METABRIC
PD11327	TYMS	cna	gain	BASIS
PD11742	TYMS	cna	hetloss	BASIS
PD13296	TYMS	cna	hetloss	BASIS
PD13299	TYMS	cna	gain	BASIS
PD23574	TYMS	cna	gain	BASIS
PD24206	TYMS	cna	gain	BASIS
PD24337	TYMS	cna	hetloss	BASIS
PD3890	TYMS	cna	hetloss	BASIS
PD3905	TYMS	cna	gain	BASIS
PD4005	TYMS	cna	hetloss	BASIS
PD4006	TYMS	cna	gain	BASIS
PD4107	TYMS	cna	gain	BASIS
PD4826	TYMS	cna	gain	BASIS
PD4967	TYMS	cna	hetloss	BASIS
PD5935	TYMS	cna	gain	BASIS

PD5945	TYMS	cna	amp	BASIS
PD5948	TYMS	cna	gain	BASIS
PD7215	TYMS	cna	gain	BASIS
PD8621	TYMS	cna	gain	BASIS
PD9004	TYMS	cna	gain	BASIS
PD9585	TYMS	cna	hetloss	BASIS
PD9702	TYMS	cna	gain	BASIS
TCGA-AN-A0XU	YES1	cna	hetloss	TCGA
TCGA-AO-A0JL	YES1	cna	gain	TCGA
TCGA-BH-A0AW	YES1	cna	hetloss	TCGA
TCGA-BH-A0C0	YES1	cna	hetloss	TCGA
TCGA-BH-A1FU	YES1	cna	gain	TCGA
TCGA-C8-A12L	YES1	cna	gain	TCGA
TCGA-D8-A27M	YES1	cna	hetloss	TCGA
TCGA-LL-A5YP	YES1	cna	gain	TCGA
MB-0346	YES1	cna	gain	METABRIC
MB-5107	YES1	cna	hetloss	METABRIC
MB-5465	YES1	cna	hetloss	METABRIC
MB-6098	YES1	cna	hetloss	METABRIC
MB-0420	YES1	cna	gain	METABRIC
MTS-T0064	YES1	cna	hetloss	METABRIC
PD11327	YES1	cna	gain	BASIS
PD11742	YES1	cna	hetloss	BASIS
PD13296	YES1	cna	hetloss	BASIS
PD13299	YES1	cna	gain	BASIS
PD23574	YES1	cna	gain	BASIS
PD24202	YES1	cna	gain	BASIS
PD24206	YES1	cna	gain	BASIS
PD24337	YES1	cna	hetloss	BASIS
PD3890	YES1	cna	hetloss	BASIS
PD3905	YES1	cna	gain	BASIS
PD4005	YES1	cna	hetloss	BASIS
PD4006	YES1	cna	gain	BASIS
PD4107	YES1	cna	gain	BASIS
PD4967	YES1	cna	hetloss	BASIS
PD5935	YES1	cna	gain	BASIS
PD5945	YES1	cna	amp	BASIS
PD5948	YES1	cna	gain	BASIS
PD7215	YES1	cna	gain	BASIS
PD8621	YES1	cna	gain	BASIS
PD9004	YES1	cna	gain	BASIS
PD9585	YES1	cna	hetloss	BASIS
PD9702	YES1	cna	gain	BASIS
TCGA-AN-A0XU	ZNF521	cna	hetloss	TCGA
TCGA-AO-A0JL	ZNF521	cna	gain	TCGA
TCGA-BH-A0AW	ZNF521	cna	hetloss	TCGA
TCGA-BH-A0C0	ZNF521	cna	gain	TCGA

TCGA-C8-A12L	ZNF521	cna	gain	TCGA
TCGA-D8-A27M	ZNF521	cna	hetloss	TCGA
TCGA-E2-A1L7	ZNF521	cna	gain	TCGA
TCGA-LL-A5YP	ZNF521	cna	amp	TCGA
MB-0346	ZNF521	cna	hetloss	METABRIC
MB-5465	ZNF521	cna	hetloss	METABRIC
MB-0420	ZNF521	cna	gain	METABRIC
PD10014	ZNF521	cna	gain	BASIS
PD11327	ZNF521	cna	amp	BASIS
PD13297	ZNF521	cna	gain	BASIS
PD13299	ZNF521	cna	gain	BASIS
PD22355	ZNF521	cna	hetloss	BASIS
PD23562	ZNF521	cna	gain	BASIS
PD23574	ZNF521	cna	gain	BASIS
PD24186	ZNF521	cna	gain	BASIS
PD24202	ZNF521	cna	hetloss	BASIS
PD24206	ZNF521	cna	gain	BASIS
PD24337	ZNF521	cna	gain	BASIS
PD3905	ZNF521	cna	gain	BASIS
PD4005	ZNF521	cna	hetloss	BASIS
PD4006	ZNF521	cna	gain	BASIS
PD4107	ZNF521	cna	gain	BASIS
PD4967	ZNF521	cna	hetloss	BASIS
PD5930	ZNF521	cna	gain	BASIS
PD5935	ZNF521	cna	gain	BASIS
PD5945	ZNF521	cna	gain	BASIS
PD5948	ZNF521	cna	amp	BASIS
PD7067	ZNF521	cna	gain	BASIS
PD7215	ZNF521	cna	gain	BASIS
PD8980	ZNF521	cna	hetloss	BASIS
PD9004	ZNF521	cna	gain	BASIS
PD9702	ZNF521	cna	gain	BASIS
TCGA-A2-A25B	ACVR1B	cna	gain	TCGA
TCGA-AN-A0XU	ACVR1B	cna	gain	TCGA
TCGA-AO-A0JL	ACVR1B	cna	hetloss	TCGA
TCGA-BH-A18R	ACVR1B	cna	gain	TCGA
TCGA-BH-A1FU	ACVR1B	cna	hetloss	TCGA
TCGA-C8-A12L	ACVR1B	cna	gain	TCGA
TCGA-D8-A27M	ACVR1B	cna	hetloss	TCGA
TCGA-E2-A1L7	ACVR1B	cna	hetloss	TCGA
TCGA-E9-A1NC	ACVR1B	cna	hetloss	TCGA
TCGA-LL-A5YP	ACVR1B	cna	hetloss	TCGA
MB-0346	ACVR1B	cna	gain	METABRIC
MB-2827	ACVR1B	cna	hetloss	METABRIC
MB-5070	ACVR1B	cna	hetloss	METABRIC
MB-6098	ACVR1B	cna	hetloss	METABRIC
MB-7038	ACVR1B	cna	hetloss	METABRIC

MB-0420	ACVR1B	cna	hetloss	METABRIC
PD11327	ACVR1B	cna	hetloss	BASIS
PD13296	ACVR1B	cna	hetloss	BASIS
PD13297	ACVR1B	cna	hetloss	BASIS
PD23578	ACVR1B	cna	hetloss	BASIS
PD24202	ACVR1B	cna	hetloss	BASIS
PD24206	ACVR1B	cna	gain	BASIS
PD24337	ACVR1B	cna	hetloss	BASIS
PD3890	ACVR1B	cna	hetloss	BASIS
PD3905	ACVR1B	cna	gain	BASIS
PD4107	ACVR1B	cna	hetloss	BASIS
PD4826	ACVR1B	cna	gain	BASIS
PD5945	ACVR1B	cna	gain	BASIS
PD6406	ACVR1B	cna	hetloss	BASIS
PD6413	ACVR1B	cna	hetloss	BASIS
PD6731	ACVR1B	cna	hetloss	BASIS
PD7067	ACVR1B	cna	gain	BASIS
PD8621	ACVR1B	cna	hetloss	BASIS
PD9585	ACVR1B	cna	hetloss	BASIS
PD9702	ACVR1B	cna	amp	BASIS
TCGA-A2-A25B	BCL10	cna	gain	TCGA
TCGA-AN-A0XU	BCL10	cna	hetloss	TCGA
TCGA-BH-A0AW	BCL10	cna	gain	TCGA
TCGA-BH-A0C0	BCL10	cna	hetloss	TCGA
TCGA-BH-A1FU	BCL10	cna	gain	TCGA
TCGA-C8-A12L	BCL10	cna	hetloss	TCGA
TCGA-D8-A27M	BCL10	cna	hetloss	TCGA
TCGA-E2-A1L7	BCL10	cna	gain	TCGA
TCGA-LL-A5YP	BCL10	cna	hetloss	TCGA
MB-0346	BCL10	cna	hetloss	METABRIC
MB-2827	BCL10	cna	hetloss	METABRIC
MB-6060	BCL10	cna	hetloss	METABRIC
MB-6098	BCL10	cna	hetloss	METABRIC
MB-7038	BCL10	cna	gain	METABRIC
PD11742	BCL10	cna	hetloss	BASIS
PD13299	BCL10	cna	gain	BASIS
PD22355	BCL10	cna	hetloss	BASIS
PD23574	BCL10	cna	gain	BASIS
PD23578	BCL10	cna	gain	BASIS
PD24186	BCL10	cna	gain	BASIS
PD24206	BCL10	cna	gain	BASIS
PD3890	BCL10	cna	gain	BASIS
PD3905	BCL10	cna	gain	BASIS
PD4005	BCL10	cna	gain	BASIS
PD4107	BCL10	cna	gain	BASIS
PD4826	BCL10	cna	gain	BASIS
PD5930	BCL10	cna	gain	BASIS

PD5945	BCL10	cna	gain	BASIS
PD5948	BCL10	cna	gain	BASIS
PD7067	BCL10	cna	gain	BASIS
PD7215	BCL10	cna	gain	BASIS
PD8621	BCL10	cna	gain	BASIS
PD9004	BCL10	cna	gain	BASIS
PD9585	BCL10	cna	hetloss	BASIS
PD9702	BCL10	cna	gain	BASIS
TCGA-A2-A25B	BRD3	cna	gain	TCGA
TCGA-AN-A0XU	BRD3	cna	hetloss	TCGA
TCGA-AO-A0JL	BRD3	cna	hetloss	TCGA
TCGA-BH-A0C0	BRD3	cna	gain	TCGA
TCGA-C8-A12L	BRD3	cna	gain	TCGA
TCGA-E2-A1L7	BRD3	cna	hetloss	TCGA
TCGA-E9-A1NC	BRD3	cna	hetloss	TCGA
TCGA-LL-A5YP	BRD3	cna	hetloss	TCGA
MB-0346	BRD3	cna	hetloss	METABRIC
MB-2827	BRD3	cna	amp	METABRIC
MB-5070	BRD3	cna	hetloss	METABRIC
MB-7038	BRD3	cna	hetloss	METABRIC
MB-0420	BRD3	cna	hetloss	METABRIC
PD10014	BRD3	cna	gain	BASIS
PD11327	BRD3	cna	gain	BASIS
PD13299	BRD3	cna	gain	BASIS
PD22355	BRD3	cna	gain	BASIS
PD24186	BRD3	cna	gain	BASIS
PD24202	BRD3	cna	hetloss	BASIS
PD24206	BRD3	cna	gain	BASIS
PD24337	BRD3	cna	hetloss	BASIS
PD3890	BRD3	cna	hetloss	BASIS
PD3905	BRD3	cna	gain	BASIS
PD4005	BRD3	cna	hetloss	BASIS
PD4006	BRD3	cna	gain	BASIS
PD4107	BRD3	cna	hetloss	BASIS
PD4826	BRD3	cna	gain	BASIS
PD5945	BRD3	cna	gain	BASIS
PD5948	BRD3	cna	gain	BASIS
PD6406	BRD3	cna	hetloss	BASIS
PD6731	BRD3	cna	hetloss	BASIS
PD7067	BRD3	cna	gain	BASIS
PD7215	BRD3	cna	gain	BASIS
PD8621	BRD3	cna	gain	BASIS
PD9004	BRD3	cna	hetloss	BASIS
TCGA-AO-A0JL	BRD4	cna	gain	TCGA
TCGA-BH-A0C0	BRD4	cna	gain	TCGA
TCGA-BH-A1FU	BRD4	cna	hetloss	TCGA
TCGA-C8-A12L	BRD4	cna	gain	TCGA

TCGA-D8-A27M	BRD4	cna	hetloss	TCGA
TCGA-EW-A1OX	BRD4	cna	hetloss	TCGA
TCGA-LL-A5YP	BRD4	cna	hetloss	TCGA
MB-0346	BRD4	cna	hetloss	METABRIC
MB-2827	BRD4	cna	hetloss	METABRIC
MB-5465	BRD4	cna	gain	METABRIC
MB-6098	BRD4	cna	hetloss	METABRIC
MB-7038	BRD4	cna	gain	METABRIC
MB-0420	BRD4	cna	hetloss	METABRIC
MTS-T0064	BRD4	cna	amp	METABRIC
P-0009557-T01-IM5	BRD4	cna	homdel	MSK-IMPACT
PD14442	BRD4	cna	hetloss	BASIS
PD22355	BRD4	cna	gain	BASIS
PD23562	BRD4	cna	gain	BASIS
PD23574	BRD4	cna	gain	BASIS
PD24186	BRD4	cna	gain	BASIS
PD24206	BRD4	cna	hetloss	BASIS
PD3905	BRD4	cna	gain	BASIS
PD4006	BRD4	cna	amp	BASIS
PD4107	BRD4	cna	gain	BASIS
PD4826	BRD4	cna	gain	BASIS
PD4967	BRD4	cna	hetloss	BASIS
PD5930	BRD4	cna	gain	BASIS
PD5935	BRD4	cna	gain	BASIS
PD5945	BRD4	cna	amp	BASIS
PD5948	BRD4	cna	gain	BASIS
PD6413	BRD4	cna	gain	BASIS
PD7067	BRD4	cna	gain	BASIS
PD7215	BRD4	cna	amp	BASIS
PD8621	BRD4	cna	gain	BASIS
PD9702	BRD4	cna	gain	BASIS
TCGA-AO-A0JL	BRSK1	cna	hetloss	TCGA
TCGA-BH-A0AW	BRSK1	cna	gain	TCGA
TCGA-BH-A0C0	BRSK1	cna	gain	TCGA
TCGA-BH-A1FU	BRSK1	cna	hetloss	TCGA
TCGA-C8-A12L	BRSK1	cna	gain	TCGA
TCGA-D8-A27M	BRSK1	cna	hetloss	TCGA
TCGA-EW-A1OX	BRSK1	cna	hetloss	TCGA
TCGA-LL-A5YP	BRSK1	cna	gain	TCGA
MB-0346	BRSK1	cna	hetloss	METABRIC
MB-2827	BRSK1	cna	hetloss	METABRIC
MB-6098	BRSK1	cna	hetloss	METABRIC
MB-7048	BRSK1	cna	gain	METABRIC
PD11327	BRSK1	cna	gain	BASIS
PD13296	BRSK1	cna	gain	BASIS
PD13299	BRSK1	cna	gain	BASIS
PD13771	BRSK1	cna	hetloss	BASIS

PD23562	BRSK1	cna	gain	BASIS
PD23574	BRSK1	cna	gain	BASIS
PD24186	BRSK1	cna	gain	BASIS
PD24206	BRSK1	cna	hetloss	BASIS
PD3890	BRSK1	cna	hetloss	BASIS
PD3905	BRSK1	cna	gain	BASIS
PD4006	BRSK1	cna	gain	BASIS
PD4107	BRSK1	cna	gain	BASIS
PD4826	BRSK1	cna	gain	BASIS
PD5935	BRSK1	cna	gain	BASIS
PD5945	BRSK1	cna	gain	BASIS
PD5948	BRSK1	cna	gain	BASIS
PD6406	BRSK1	cna	gain	BASIS
PD7067	BRSK1	cna	gain	BASIS
PD7215	BRSK1	cna	gain	BASIS
PD8621	BRSK1	cna	gain	BASIS
PD8980	BRSK1	cna	hetloss	BASIS
PD9585	BRSK1	cna	gain	BASIS
PD9702	BRSK1	cna	gain	BASIS
TCGA-AO-A0JL	CALR	cna	gain	TCGA
TCGA-BH-A0C0	CALR	cna	gain	TCGA
TCGA-BH-A1FU	CALR	cna	hetloss	TCGA
TCGA-C8-A12L	CALR	cna	gain	TCGA
TCGA-D8-A27M	CALR	cna	hetloss	TCGA
TCGA-EW-A1OX	CALR	cna	hetloss	TCGA
TCGA-LL-A5YP	CALR	cna	hetloss	TCGA
MB-0346	CALR	cna	hetloss	METABRIC
MB-2827	CALR	cna	hetloss	METABRIC
MB-5465	CALR	cna	gain	METABRIC
MB-6098	CALR	cna	hetloss	METABRIC
MB-7038	CALR	cna	hetloss	METABRIC
MB-0420	CALR	cna	hetloss	METABRIC
P-0009557-T01-IM5	CALR	cna	homdel	MSK-IMPACT
PD11742	CALR	cna	hetloss	BASIS
PD13297	CALR	cna	gain	BASIS
PD14442	CALR	cna	hetloss	BASIS
PD23562	CALR	cna	gain	BASIS
PD23574	CALR	cna	gain	BASIS
PD24186	CALR	cna	amp	BASIS
PD24206	CALR	cna	homdel	BASIS
PD3905	CALR	cna	gain	BASIS
PD4006	CALR	cna	amp	BASIS
PD4107	CALR	cna	gain	BASIS
PD4826	CALR	cna	gain	BASIS
PD4967	CALR	cna	hetloss	BASIS
PD5930	CALR	cna	gain	BASIS
PD5935	CALR	cna	gain	BASIS

PD5945	CALR	cna	amp	BASIS
PD5948	CALR	cna	gain	BASIS
PD6413	CALR	cna	gain	BASIS
PD7067	CALR	cna	gain	BASIS
PD7215	CALR	cna	amp	BASIS
PD8621	CALR	cna	gain	BASIS
PD9702	CALR	cna	gain	BASIS
TCGA-A2-A25B	CAMTA1	cna	hetloss	TCGA
TCGA-AN-A0XU	CAMTA1	cna	hetloss	TCGA
TCGA-AO-A0JL	CAMTA1	cna	hetloss	TCGA
TCGA-BH-A0AW	CAMTA1	cna	gain	TCGA
TCGA-BH-A0C0	CAMTA1	cna	gain	TCGA
TCGA-BH-A18R	CAMTA1	cna	homdel	TCGA
TCGA-BH-A1FU	CAMTA1	cna	gain	TCGA
TCGA-C8-A12L	CAMTA1	cna	hetloss	TCGA
TCGA-D8-A27M	CAMTA1	cna	gain	TCGA
TCGA-E2-A1L7	CAMTA1	cna	hetloss	TCGA
TCGA-E9-A1NC	CAMTA1	cna	hetloss	TCGA
TCGA-LL-A5YP	CAMTA1	cna	amp	TCGA
MB-2827	CAMTA1	cna	hetloss	METABRIC
MB-5070	CAMTA1	cna	hetloss	METABRIC
MB-5465	CAMTA1	cna	hetloss	METABRIC
MB-6060	CAMTA1	cna	hetloss	METABRIC
MB-6098	CAMTA1	cna	gain	METABRIC
MB-0420	CAMTA1	cna	hetloss	METABRIC
PD10014	CAMTA1	cna	gain	BASIS
PD11742	CAMTA1	cna	hetloss	BASIS
PD22355	CAMTA1	cna	hetloss	BASIS
PD24202	CAMTA1	cna	hetloss	BASIS
PD24206	CAMTA1	cna	gain	BASIS
PD24337	CAMTA1	cna	hetloss	BASIS
PD3890	CAMTA1	cna	hetloss	BASIS
PD3905	CAMTA1	cna	gain	BASIS
PD4107	CAMTA1	cna	gain	BASIS
PD4826	CAMTA1	cna	gain	BASIS
PD5945	CAMTA1	cna	gain	BASIS
PD6406	CAMTA1	cna	hetloss	BASIS
PD7067	CAMTA1	cna	gain	BASIS
PD7215	CAMTA1	cna	gain	BASIS
PD8621	CAMTA1	cna	gain	BASIS
PD8980	CAMTA1	cna	hetloss	BASIS
PD9004	CAMTA1	cna	hetloss	BASIS
TCGA-AN-A0XU	CBFB	cna	hetloss	TCGA
TCGA-AO-A0JL	CBFB	cna	gain	TCGA
TCGA-BH-A0C0	CBFB	cna	hetloss	TCGA
TCGA-C8-A12L	CBFB	cna	gain	TCGA
TCGA-D8-A27M	CBFB	cna	hetloss	TCGA

TCGA-E2-A1L7	CBFB	cna	hetloss	TCGA
TCGA-E9-A1NC	CBFB	cna	hetloss	TCGA
TCGA-LL-A5YP	CBFB	cna	gain	TCGA
MB-0346	CBFB	cna	gain	METABRIC
MB-5107	CBFB	cna	hetloss	METABRIC
MB-6271	CBFB	cna	hetloss	METABRIC
MB-7032	CBFB	cna	gain	METABRIC
MB-7048	CBFB	cna	hetloss	METABRIC
MB-0420	CBFB	cna	hetloss	METABRIC
MTS-T0064	CBFB	cna	hetloss	METABRIC
PD10014	CBFB	cna	hetloss	BASIS
PD11327	CBFB	cna	homdel	BASIS
PD11742	CBFB	cna	hetloss	BASIS
PD13296	CBFB	cna	hetloss	BASIS
PD13297	CBFB	cna	hetloss	BASIS
PD13299	CBFB	cna	gain	BASIS
PD14442	CBFB	cna	hetloss	BASIS
PD23562	CBFB	cna	gain	BASIS
PD24186	CBFB	cna	gain	BASIS
PD24202	CBFB	cna	gain	BASIS
PD24206	CBFB	cna	gain	BASIS
PD3905	CBFB	cna	gain	BASIS
PD4005	CBFB	cna	hetloss	BASIS
PD4967	CBFB	cna	homdel	BASIS
PD5945	CBFB	cna	gain	BASIS
PD5948	CBFB	cna	hetloss	BASIS
PD7215	CBFB	cna	gain	BASIS
PD8980	CBFB	cna	hetloss	BASIS
PD9585	CBFB	cna	hetloss	BASIS
PD9702	CBFB	cna	gain	BASIS
TCGA-AN-A0XU	CCNB1IP1	cna	gain	TCGA
TCGA-AO-A0JL	CCNB1IP1	cna	amp	TCGA
TCGA-BH-A0AW	CCNB1IP1	cna	hetloss	TCGA
TCGA-BH-A1FU	CCNB1IP1	cna	gain	TCGA
TCGA-D8-A27M	CCNB1IP1	cna	gain	TCGA
TCGA-LL-A5YP	CCNB1IP1	cna	hetloss	TCGA
MB-5070	CCNB1IP1	cna	hetloss	METABRIC
MB-6060	CCNB1IP1	cna	gain	METABRIC
MB-7048	CCNB1IP1	cna	gain	METABRIC
PD11327	CCNB1IP1	cna	homdel	BASIS
PD13296	CCNB1IP1	cna	gain	BASIS
PD13299	CCNB1IP1	cna	gain	BASIS
PD22355	CCNB1IP1	cna	hetloss	BASIS
PD23574	CCNB1IP1	cna	gain	BASIS
PD23578	CCNB1IP1	cna	hetloss	BASIS
PD24186	CCNB1IP1	cna	gain	BASIS
PD24202	CCNB1IP1	cna	hetloss	BASIS

PD24206	CCNB1IP1	cna	gain	BASIS
PD24337	CCNB1IP1	cna	hetloss	BASIS
PD3890	CCNB1IP1	cna	hetloss	BASIS
PD3905	CCNB1IP1	cna	amp	BASIS
PD4005	CCNB1IP1	cna	hetloss	BASIS
PD4107	CCNB1IP1	cna	gain	BASIS
PD4967	CCNB1IP1	cna	hetloss	BASIS
PD5930	CCNB1IP1	cna	hetloss	BASIS
PD5935	CCNB1IP1	cna	gain	BASIS
PD5945	CCNB1IP1	cna	amp	BASIS
PD5948	CCNB1IP1	cna	gain	BASIS
PD6406	CCNB1IP1	cna	hetloss	BASIS
PD6731	CCNB1IP1	cna	hetloss	BASIS
PD7215	CCNB1IP1	cna	gain	BASIS
PD8621	CCNB1IP1	cna	gain	BASIS
PD8980	CCNB1IP1	cna	gain	BASIS
PD9004	CCNB1IP1	cna	gain	BASIS
PD9702	CCNB1IP1	cna	gain	BASIS
TCGA-A2-A25B	CNRIP1	cna	hetloss	TCGA
TCGA-AN-A0XU	CNRIP1	cna	gain	TCGA
TCGA-AO-A0JL	CNRIP1	cna	gain	TCGA
TCGA-BH-A0AW	CNRIP1	cna	gain	TCGA
TCGA-BH-A0C0	CNRIP1	cna	gain	TCGA
TCGA-D8-A27M	CNRIP1	cna	gain	TCGA
TCGA-E9-A1NC	CNRIP1	cna	gain	TCGA
TCGA-LL-A5YP	CNRIP1	cna	gain	TCGA
MB-5070	CNRIP1	cna	hetloss	METABRIC
MB-6098	CNRIP1	cna	gain	METABRIC
MB-6271	CNRIP1	cna	hetloss	METABRIC
MB-7038	CNRIP1	cna	gain	METABRIC
PD11327	CNRIP1	cna	gain	BASIS
PD13296	CNRIP1	cna	hetloss	BASIS
PD13297	CNRIP1	cna	gain	BASIS
PD13299	CNRIP1	cna	gain	BASIS
PD23562	CNRIP1	cna	gain	BASIS
PD23578	CNRIP1	cna	gain	BASIS
PD24186	CNRIP1	cna	gain	BASIS
PD24202	CNRIP1	cna	gain	BASIS
PD24206	CNRIP1	cna	gain	BASIS
PD3890	CNRIP1	cna	hetloss	BASIS
PD3905	CNRIP1	cna	gain	BASIS
PD4006	CNRIP1	cna	amp	BASIS
PD4107	CNRIP1	cna	hetloss	BASIS
PD5930	CNRIP1	cna	gain	BASIS
PD5935	CNRIP1	cna	gain	BASIS
PD5945	CNRIP1	cna	gain	BASIS
PD5948	CNRIP1	cna	gain	BASIS

PD7067	CNRIP1	cna	gain	BASIS
PD7215	CNRIP1	cna	gain	BASIS
PD8621	CNRIP1	cna	gain	BASIS
PD9004	CNRIP1	cna	gain	BASIS
PD9585	CNRIP1	cna	gain	BASIS
PD9702	CNRIP1	cna	gain	BASIS
TCGA-A2-A25B	CSF3R	cna	hetloss	TCGA
TCGA-AN-A0XU	CSF3R	cna	hetloss	TCGA
TCGA-AO-A0JL	CSF3R	cna	hetloss	TCGA
TCGA-BH-A0AW	CSF3R	cna	gain	TCGA
TCGA-BH-A0C0	CSF3R	cna	gain	TCGA
TCGA-BH-A1FU	CSF3R	cna	gain	TCGA
TCGA-C8-A12L	CSF3R	cna	hetloss	TCGA
TCGA-D8-A27M	CSF3R	cna	gain	TCGA
TCGA-E2-A1L7	CSF3R	cna	gain	TCGA
TCGA-LL-A5YP	CSF3R	cna	gain	TCGA
MB-0346	CSF3R	cna	hetloss	METABRIC
MB-5465	CSF3R	cna	hetloss	METABRIC
MB-6060	CSF3R	cna	hetloss	METABRIC
MB-6098	CSF3R	cna	hetloss	METABRIC
MB-7038	CSF3R	cna	gain	METABRIC
MB-0420	CSF3R	cna	hetloss	METABRIC
PD10014	CSF3R	cna	gain	BASIS
PD13296	CSF3R	cna	gain	BASIS
PD13299	CSF3R	cna	gain	BASIS
PD24186	CSF3R	cna	gain	BASIS
PD24206	CSF3R	cna	gain	BASIS
PD24337	CSF3R	cna	hetloss	BASIS
PD3905	CSF3R	cna	gain	BASIS
PD4006	CSF3R	cna	gain	BASIS
PD4107	CSF3R	cna	gain	BASIS
PD4826	CSF3R	cna	gain	BASIS
PD5935	CSF3R	cna	gain	BASIS
PD5945	CSF3R	cna	amp	BASIS
PD7067	CSF3R	cna	gain	BASIS
PD7215	CSF3R	cna	gain	BASIS
PD8621	CSF3R	cna	gain	BASIS
PD8980	CSF3R	cna	gain	BASIS
PD9004	CSF3R	cna	hetloss	BASIS
PD9585	CSF3R	cna	hetloss	BASIS
PD9702	CSF3R	cna	gain	BASIS
TCGA-AN-A0XU	CTCF	cna	hetloss	TCGA
TCGA-AO-A0JL	CTCF	cna	gain	TCGA
TCGA-BH-A0AW	CTCF	cna	hetloss	TCGA
TCGA-BH-A0C0	CTCF	cna	hetloss	TCGA
TCGA-C8-A12L	CTCF	cna	gain	TCGA
TCGA-D8-A27M	CTCF	cna	hetloss	TCGA

TCGA-E2-A1L7	CTCF	cna	hetloss	TCGA
TCGA-E9-A1NC	CTCF	cna	hetloss	TCGA
TCGA-LL-A5YP	CTCF	cna	gain	TCGA
MB-0346	CTCF	cna	gain	METABRIC
MB-5107	CTCF	cna	hetloss	METABRIC
MB-6271	CTCF	cna	hetloss	METABRIC
MB-7032	CTCF	cna	gain	METABRIC
MB-7048	CTCF	cna	hetloss	METABRIC
MB-0420	CTCF	cna	hetloss	METABRIC
MTS-T0064	CTCF	cna	hetloss	METABRIC
PD10014	CTCF	cna	hetloss	BASIS
PD11327	CTCF	cna	homdel	BASIS
PD11742	CTCF	cna	hetloss	BASIS
PD13297	CTCF	cna	hetloss	BASIS
PD13299	CTCF	cna	gain	BASIS
PD14442	CTCF	cna	hetloss	BASIS
PD23562	CTCF	cna	gain	BASIS
PD24186	CTCF	cna	gain	BASIS
PD24202	CTCF	cna	hetloss	BASIS
PD24206	CTCF	cna	hetloss	BASIS
PD3905	CTCF	cna	gain	BASIS
PD4005	CTCF	cna	hetloss	BASIS
PD4967	CTCF	cna	homdel	BASIS
PD5945	CTCF	cna	gain	BASIS
PD5948	CTCF	cna	hetloss	BASIS
PD7215	CTCF	cna	gain	BASIS
PD8980	CTCF	cna	hetloss	BASIS
PD9585	CTCF	cna	hetloss	BASIS
PD9702	CTCF	cna	gain	BASIS
TCGA-AN-A0XU	CXCR4	cna	gain	TCGA
TCGA-AO-A0JL	CXCR4	cna	gain	TCGA
TCGA-D8-A27M	CXCR4	cna	gain	TCGA
TCGA-E9-A1NC	CXCR4	cna	gain	TCGA
TCGA-LL-A5YP	CXCR4	cna	hetloss	TCGA
MB-2827	CXCR4	cna	hetloss	METABRIC
MB-5107	CXCR4	cna	hetloss	METABRIC
MB-5465	CXCR4	cna	gain	METABRIC
MB-6098	CXCR4	cna	hetloss	METABRIC
MB-6271	CXCR4	cna	hetloss	METABRIC
PD11327	CXCR4	cna	gain	BASIS
PD11742	CXCR4	cna	hetloss	BASIS
PD13296	CXCR4	cna	hetloss	BASIS
PD13297	CXCR4	cna	hetloss	BASIS
PD13299	CXCR4	cna	gain	BASIS
PD22355	CXCR4	cna	hetloss	BASIS
PD23574	CXCR4	cna	gain	BASIS
PD23578	CXCR4	cna	hetloss	BASIS

PD24186	CXCR4	cna	gain	BASIS
PD24202	CXCR4	cna	hetloss	BASIS
PD24206	CXCR4	cna	gain	BASIS
PD3890	CXCR4	cna	hetloss	BASIS
PD3905	CXCR4	cna	gain	BASIS
PD4005	CXCR4	cna	hetloss	BASIS
PD4006	CXCR4	cna	homdel	BASIS
PD4967	CXCR4	cna	hetloss	BASIS
PD5935	CXCR4	cna	gain	BASIS
PD5945	CXCR4	cna	gain	BASIS
PD5948	CXCR4	cna	gain	BASIS
PD6406	CXCR4	cna	hetloss	BASIS
PD6413	CXCR4	cna	hetloss	BASIS
PD7215	CXCR4	cna	gain	BASIS
PD8621	CXCR4	cna	hetloss	BASIS
PD9004	CXCR4	cna	hetloss	BASIS
PD9702	CXCR4	cna	gain	BASIS
TCGA-A2-A25B	DKK3	cna	hetloss	TCGA
TCGA-AN-A0XU	DKK3	cna	hetloss	TCGA
TCGA-AO-A0JL	DKK3	cna	hetloss	TCGA
TCGA-BH-A0C0	DKK3	cna	hetloss	TCGA
TCGA-C8-A12L	DKK3	cna	hetloss	TCGA
TCGA-E2-A1L7	DKK3	cna	hetloss	TCGA
TCGA-E9-A1NC	DKK3	cna	hetloss	TCGA
TCGA-LL-A5YP	DKK3	cna	hetloss	TCGA
MB-0346	DKK3	cna	hetloss	METABRIC
MB-6060	DKK3	cna	hetloss	METABRIC
MB-6271	DKK3	cna	gain	METABRIC
MB-7048	DKK3	cna	hetloss	METABRIC
PD10014	DKK3	cna	hetloss	BASIS
PD11327	DKK3	cna	gain	BASIS
PD11742	DKK3	cna	hetloss	BASIS
PD13296	DKK3	cna	hetloss	BASIS
PD13297	DKK3	cna	hetloss	BASIS
PD23562	DKK3	cna	gain	BASIS
PD23574	DKK3	cna	gain	BASIS
PD24206	DKK3	cna	gain	BASIS
PD3890	DKK3	cna	hetloss	BASIS
PD3905	DKK3	cna	gain	BASIS
PD4005	DKK3	cna	hetloss	BASIS
PD4107	DKK3	cna	gain	BASIS
PD4826	DKK3	cna	gain	BASIS
PD5935	DKK3	cna	gain	BASIS
PD5945	DKK3	cna	amp	BASIS
PD5948	DKK3	cna	gain	BASIS
PD6406	DKK3	cna	hetloss	BASIS
PD6413	DKK3	cna	hetloss	BASIS

PD6731	DKK3	cna	hetloss	BASIS
PD7215	DKK3	cna	hetloss	BASIS
PD8980	DKK3	cna	hetloss	BASIS
PD9585	DKK3	cna	hetloss	BASIS
PD9702	DKK3	cna	gain	BASIS
TCGA-A2-A25B	DKK4	cna	hetloss	TCGA
TCGA-AO-A0JL	DKK4	cna	amp	TCGA
TCGA-BH-A0C0	DKK4	cna	gain	TCGA
TCGA-BH-A1FU	DKK4	cna	homdel	TCGA
TCGA-D8-A27M	DKK4	cna	amp	TCGA
TCGA-E2-A1L7	DKK4	cna	hetloss	TCGA
TCGA-LL-A5YP	DKK4	cna	amp	TCGA
MB-0346	DKK4	cna	hetloss	METABRIC
MB-2827	DKK4	cna	hetloss	METABRIC
MB-6060	DKK4	cna	amp	METABRIC
MB-7038	DKK4	cna	hetloss	METABRIC
PD11327	DKK4	cna	homdel	BASIS
PD13296	DKK4	cna	hetloss	BASIS
PD13299	DKK4	cna	gain	BASIS
PD13771	DKK4	cna	amp	BASIS
PD23562	DKK4	cna	gain	BASIS
PD23574	DKK4	cna	gain	BASIS
PD23578	DKK4	cna	hetloss	BASIS
PD24186	DKK4	cna	gain	BASIS
PD24202	DKK4	cna	hetloss	BASIS
PD24206	DKK4	cna	amp	BASIS
PD3905	DKK4	cna	gain	BASIS
PD4006	DKK4	cna	gain	BASIS
PD4107	DKK4	cna	gain	BASIS
PD4826	DKK4	cna	hetloss	BASIS
PD5930	DKK4	cna	gain	BASIS
PD5935	DKK4	cna	gain	BASIS
PD5945	DKK4	cna	amp	BASIS
PD5948	DKK4	cna	hetloss	BASIS
PD7067	DKK4	cna	amp	BASIS
PD7215	DKK4	cna	amp	BASIS
PD8980	DKK4	cna	amp	BASIS
PD9004	DKK4	cna	amp	BASIS
PD9585	DKK4	cna	gain	BASIS
PD9702	DKK4	cna	gain	BASIS
TCGA-AO-A0JL	DNMT1	cna	gain	TCGA
TCGA-BH-A0C0	DNMT1	cna	gain	TCGA
TCGA-BH-A1FU	DNMT1	cna	hetloss	TCGA
TCGA-C8-A12L	DNMT1	cna	gain	TCGA
TCGA-D8-A27M	DNMT1	cna	hetloss	TCGA
TCGA-EW-A1OX	DNMT1	cna	hetloss	TCGA
TCGA-LL-A5YP	DNMT1	cna	hetloss	TCGA

MB-0346	DNMT1	cna	hetloss	METABRIC
MB-2827	DNMT1	cna	hetloss	METABRIC
MB-5070	DNMT1	cna	hetloss	METABRIC
MB-5465	DNMT1	cna	gain	METABRIC
MB-6098	DNMT1	cna	hetloss	METABRIC
MB-7038	DNMT1	cna	hetloss	METABRIC
MB-0420	DNMT1	cna	hetloss	METABRIC
P-0009557-T01-IM5	DNMT1	cna	amp	MSK-IMPACT
PD11327	DNMT1	cna	homdel	BASIS
PD13771	DNMT1	cna	gain	BASIS
PD14442	DNMT1	cna	hetloss	BASIS
PD23562	DNMT1	cna	gain	BASIS
PD23574	DNMT1	cna	gain	BASIS
PD24206	DNMT1	cna	homdel	BASIS
PD3905	DNMT1	cna	gain	BASIS
PD4006	DNMT1	cna	gain	BASIS
PD4107	DNMT1	cna	gain	BASIS
PD4826	DNMT1	cna	gain	BASIS
PD4967	DNMT1	cna	hetloss	BASIS
PD5930	DNMT1	cna	gain	BASIS
PD5935	DNMT1	cna	gain	BASIS
PD5945	DNMT1	cna	amp	BASIS
PD5948	DNMT1	cna	gain	BASIS
PD6406	DNMT1	cna	amp	BASIS
PD7067	DNMT1	cna	gain	BASIS
PD7215	DNMT1	cna	amp	BASIS
PD8621	DNMT1	cna	gain	BASIS
PD9702	DNMT1	cna	gain	BASIS
TCGA-A2-A25B	EPHA10	cna	hetloss	TCGA
TCGA-AN-A0XU	EPHA10	cna	hetloss	TCGA
TCGA-AO-A0JL	EPHA10	cna	hetloss	TCGA
TCGA-BH-A0AW	EPHA10	cna	gain	TCGA
TCGA-BH-A0C0	EPHA10	cna	gain	TCGA
TCGA-BH-A1FU	EPHA10	cna	gain	TCGA
TCGA-C8-A12L	EPHA10	cna	hetloss	TCGA
TCGA-D8-A27M	EPHA10	cna	gain	TCGA
TCGA-E2-A1L7	EPHA10	cna	gain	TCGA
TCGA-LL-A5YP	EPHA10	cna	gain	TCGA
MB-0346	EPHA10	cna	hetloss	METABRIC
MB-5465	EPHA10	cna	hetloss	METABRIC
MB-6060	EPHA10	cna	hetloss	METABRIC
MB-6098	EPHA10	cna	hetloss	METABRIC
MB-7038	EPHA10	cna	gain	METABRIC
MB-0420	EPHA10	cna	hetloss	METABRIC
PD10014	EPHA10	cna	gain	BASIS
PD13296	EPHA10	cna	gain	BASIS
PD13299	EPHA10	cna	gain	BASIS

PD24186	EPHA10	cna	gain	BASIS
PD24206	EPHA10	cna	gain	BASIS
PD24337	EPHA10	cna	hetloss	BASIS
PD3905	EPHA10	cna	gain	BASIS
PD4006	EPHA10	cna	gain	BASIS
PD4107	EPHA10	cna	gain	BASIS
PD4826	EPHA10	cna	gain	BASIS
PD5935	EPHA10	cna	gain	BASIS
PD5945	EPHA10	cna	amp	BASIS
PD5948	EPHA10	cna	gain	BASIS
PD7067	EPHA10	cna	gain	BASIS
PD7215	EPHA10	cna	gain	BASIS
PD8621	EPHA10	cna	gain	BASIS
PD8980	EPHA10	cna	gain	BASIS
PD9004	EPHA10	cna	hetloss	BASIS
PD9702	EPHA10	cna	gain	BASIS
TCGA-A2-A25B	EPHA7	cna	hetloss	TCGA
TCGA-AN-A0XU	EPHA7	cna	amp	TCGA
TCGA-AO-A0JL	EPHA7	cna	hetloss	TCGA
TCGA-BH-A0AW	EPHA7	cna	gain	TCGA
TCGA-BH-A18R	EPHA7	cna	homdel	TCGA
TCGA-C8-A12L	EPHA7	cna	gain	TCGA
MB-2827	EPHA7	cna	amp	METABRIC
MB-5107	EPHA7	cna	hetloss	METABRIC
MB-6060	EPHA7	cna	hetloss	METABRIC
MB-6098	EPHA7	cna	hetloss	METABRIC
MB-7032	EPHA7	cna	hetloss	METABRIC
MB-7038	EPHA7	cna	gain	METABRIC
MB-7048	EPHA7	cna	hetloss	METABRIC
PD11327	EPHA7	cna	gain	BASIS
PD13299	EPHA7	cna	gain	BASIS
PD13771	EPHA7	cna	hetloss	BASIS
PD23562	EPHA7	cna	gain	BASIS
PD23574	EPHA7	cna	gain	BASIS
PD24186	EPHA7	cna	gain	BASIS
PD24206	EPHA7	cna	gain	BASIS
PD24337	EPHA7	cna	hetloss	BASIS
PD3905	EPHA7	cna	amp	BASIS
PD4006	EPHA7	cna	gain	BASIS
PD4107	EPHA7	cna	hetloss	BASIS
PD4826	EPHA7	cna	gain	BASIS
PD5935	EPHA7	cna	gain	BASIS
PD5945	EPHA7	cna	amp	BASIS
PD6731	EPHA7	cna	hetloss	BASIS
PD7067	EPHA7	cna	amp	BASIS
PD7215	EPHA7	cna	gain	BASIS
PD8621	EPHA7	cna	gain	BASIS

PD8980	EPHA7	cna	gain	BASIS
PD9004	EPHA7	cna	gain	BASIS
PD9585	EPHA7	cna	hetloss	BASIS
PD9702	EPHA7	cna	gain	BASIS
TCGA-AN-A0XU	FANCD2	cna	gain	TCGA
TCGA-BH-A0C0	FANCD2	cna	gain	TCGA
TCGA-BH-A18R	FANCD2	cna	gain	TCGA
TCGA-BH-A1FU	FANCD2	cna	hetloss	TCGA
TCGA-D8-A27M	FANCD2	cna	hetloss	TCGA
TCGA-E9-A1NC	FANCD2	cna	hetloss	TCGA
TCGA-LL-A5YP	FANCD2	cna	amp	TCGA
MB-7038	FANCD2	cna	hetloss	METABRIC
MB-7048	FANCD2	cna	hetloss	METABRIC
MB-0420	FANCD2	cna	hetloss	METABRIC
PD10014	FANCD2	cna	hetloss	BASIS
PD11327	FANCD2	cna	gain	BASIS
PD11742	FANCD2	cna	gain	BASIS
PD13296	FANCD2	cna	gain	BASIS
PD13299	FANCD2	cna	gain	BASIS
PD13771	FANCD2	cna	gain	BASIS
PD23561	FANCD2	cna	hetloss	BASIS
PD23562	FANCD2	cna	gain	BASIS
PD23574	FANCD2	cna	gain	BASIS
PD23578	FANCD2	cna	gain	BASIS
PD24186	FANCD2	cna	amp	BASIS
PD24206	FANCD2	cna	gain	BASIS
PD3905	FANCD2	cna	gain	BASIS
PD4006	FANCD2	cna	gain	BASIS
PD4826	FANCD2	cna	gain	BASIS
PD5945	FANCD2	cna	amp	BASIS
PD5948	FANCD2	cna	gain	BASIS
PD6406	FANCD2	cna	hetloss	BASIS
PD6731	FANCD2	cna	hetloss	BASIS
PD7067	FANCD2	cna	gain	BASIS
PD7215	FANCD2	cna	gain	BASIS
PD8980	FANCD2	cna	gain	BASIS
PD9004	FANCD2	cna	gain	BASIS
PD9585	FANCD2	cna	hetloss	BASIS
PD9702	FANCD2	cna	gain	BASIS
TCGA-A2-A25B	FAT3	cna	hetloss	TCGA
TCGA-AN-A0XU	FAT3	cna	gain	TCGA
TCGA-AO-A0JL	FAT3	cna	hetloss	TCGA
TCGA-D8-A27M	FAT3	cna	gain	TCGA
TCGA-EW-A1OX	FAT3	cna	homdel	TCGA
TCGA-LL-A5YP	FAT3	cna	hetloss	TCGA
MB-0346	FAT3	cna	hetloss	METABRIC
MB-5070	FAT3	cna	hetloss	METABRIC

MB-5107	FAT3	cna	amp	METABRIC
MB-6271	FAT3	cna	hetloss	METABRIC
MB-7032	FAT3	cna	hetloss	METABRIC
MTS-T0064	FAT3	cna	gain	METABRIC
PD10014	FAT3	cna	gain	BASIS
PD11327	FAT3	cna	gain	BASIS
PD11742	FAT3	cna	hetloss	BASIS
PD13299	FAT3	cna	gain	BASIS
PD13771	FAT3	cna	hetloss	BASIS
PD22355	FAT3	cna	hetloss	BASIS
PD23574	FAT3	cna	gain	BASIS
PD23578	FAT3	cna	hetloss	BASIS
PD24186	FAT3	cna	gain	BASIS
PD24206	FAT3	cna	gain	BASIS
PD24337	FAT3	cna	hetloss	BASIS
PD3905	FAT3	cna	gain	BASIS
PD4006	FAT3	cna	hetloss	BASIS
PD4107	FAT3	cna	hetloss	BASIS
PD5935	FAT3	cna	gain	BASIS
PD5945	FAT3	cna	gain	BASIS
PD5948	FAT3	cna	gain	BASIS
PD6413	FAT3	cna	hetloss	BASIS
PD7067	FAT3	cna	amp	BASIS
PD7215	FAT3	cna	gain	BASIS
PD8980	FAT3	cna	gain	BASIS
PD9585	FAT3	cna	hetloss	BASIS
PD9702	FAT3	cna	gain	BASIS
TCGA-AN-A0XU	FGF10	cna	gain	TCGA
TCGA-AO-A0JL	FGF10	cna	gain	TCGA
TCGA-BH-A0C0	FGF10	cna	gain	TCGA
TCGA-BH-A1FU	FGF10	cna	amp	TCGA
TCGA-E9-A1NC	FGF10	cna	hetloss	TCGA
TCGA-LL-A5YP	FGF10	cna	amp	TCGA
MB-0346	FGF10	cna	gain	METABRIC
MB-5070	FGF10	cna	gain	METABRIC
MB-6060	FGF10	cna	amp	METABRIC
MB-6098	FGF10	cna	gain	METABRIC
PD10014	FGF10	cna	hetloss	BASIS
PD11327	FGF10	cna	gain	BASIS
PD13296	FGF10	cna	gain	BASIS
PD13299	FGF10	cna	gain	BASIS
PD13771	FGF10	cna	gain	BASIS
PD23562	FGF10	cna	gain	BASIS
PD23574	FGF10	cna	gain	BASIS
PD24186	FGF10	cna	gain	BASIS
PD24206	FGF10	cna	gain	BASIS
PD24337	FGF10	cna	hetloss	BASIS

PD3890	FGF10	cna	gain	BASIS
PD3905	FGF10	cna	gain	BASIS
PD4006	FGF10	cna	gain	BASIS
PD4107	FGF10	cna	gain	BASIS
PD4826	FGF10	cna	gain	BASIS
PD5930	FGF10	cna	gain	BASIS
PD5945	FGF10	cna	amp	BASIS
PD5948	FGF10	cna	gain	BASIS
PD6406	FGF10	cna	hetloss	BASIS
PD6413	FGF10	cna	gain	BASIS
PD7215	FGF10	cna	amp	BASIS
PD8621	FGF10	cna	hetloss	BASIS
PD8980	FGF10	cna	gain	BASIS
PD9004	FGF10	cna	gain	BASIS
PD9585	FGF10	cna	gain	BASIS
TCGA-A2-A25B	FOXA1	cna	amp	TCGA
TCGA-AN-A0XU	FOXA1	cna	hetloss	TCGA
TCGA-AO-A0JL	FOXA1	cna	hetloss	TCGA
TCGA-BH-A0AW	FOXA1	cna	hetloss	TCGA
TCGA-BH-A1FU	FOXA1	cna	gain	TCGA
TCGA-D8-A27M	FOXA1	cna	hetloss	TCGA
TCGA-LL-A5YP	FOXA1	cna	hetloss	TCGA
MB-5070	FOXA1	cna	hetloss	METABRIC
MB-5465	FOXA1	cna	hetloss	METABRIC
MB-6060	FOXA1	cna	gain	METABRIC
PD10014	FOXA1	cna	hetloss	BASIS
PD13297	FOXA1	cna	hetloss	BASIS
PD22355	FOXA1	cna	hetloss	BASIS
PD23578	FOXA1	cna	hetloss	BASIS
PD24186	FOXA1	cna	gain	BASIS
PD24202	FOXA1	cna	hetloss	BASIS
PD24206	FOXA1	cna	amp	BASIS
PD24337	FOXA1	cna	hetloss	BASIS
PD3890	FOXA1	cna	hetloss	BASIS
PD3905	FOXA1	cna	gain	BASIS
PD4005	FOXA1	cna	hetloss	BASIS
PD4006	FOXA1	cna	hetloss	BASIS
PD4107	FOXA1	cna	hetloss	BASIS
PD4826	FOXA1	cna	gain	BASIS
PD5930	FOXA1	cna	hetloss	BASIS
PD5945	FOXA1	cna	gain	BASIS
PD5948	FOXA1	cna	gain	BASIS
PD6406	FOXA1	cna	hetloss	BASIS
PD7067	FOXA1	cna	gain	BASIS
PD7215	FOXA1	cna	gain	BASIS
PD8621	FOXA1	cna	gain	BASIS
PD8980	FOXA1	cna	gain	BASIS

PD9004	FOXA1	cna	gain	BASIS
PD9585	FOXA1	cna	gain	BASIS
PD9702	FOXA1	cna	gain	BASIS
TCGA-AN-A0XU	FOXP1	cna	hetloss	TCGA
TCGA-BH-A0C0	FOXP1	cna	hetloss	TCGA
TCGA-BH-A18R	FOXP1	cna	homdel	TCGA
TCGA-BH-A1FU	FOXP1	cna	hetloss	TCGA
TCGA-C8-A12L	FOXP1	cna	hetloss	TCGA
TCGA-D8-A27M	FOXP1	cna	hetloss	TCGA
TCGA-E2-A1L7	FOXP1	cna	homdel	TCGA
TCGA-E9-A1NC	FOXP1	cna	hetloss	TCGA
TCGA-LL-A5YP	FOXP1	cna	gain	TCGA
MB-0346	FOXP1	cna	hetloss	METABRIC
MB-2827	FOXP1	cna	hetloss	METABRIC
MB-5465	FOXP1	cna	hetloss	METABRIC
MB-6098	FOXP1	cna	hetloss	METABRIC
MB-7048	FOXP1	cna	hetloss	METABRIC
MB-0420	FOXP1	cna	hetloss	METABRIC
MTS-T0064	FOXP1	cna	gain	METABRIC
PD10014	FOXP1	cna	hetloss	BASIS
PD11742	FOXP1	cna	hetloss	BASIS
PD13296	FOXP1	cna	hetloss	BASIS
PD13297	FOXP1	cna	hetloss	BASIS
PD13299	FOXP1	cna	gain	BASIS
PD13771	FOXP1	cna	hetloss	BASIS
PD23561	FOXP1	cna	hetloss	BASIS
PD23574	FOXP1	cna	gain	BASIS
PD23578	FOXP1	cna	hetloss	BASIS
PD24202	FOXP1	cna	hetloss	BASIS
PD24337	FOXP1	cna	hetloss	BASIS
PD3905	FOXP1	cna	gain	BASIS
PD4107	FOXP1	cna	gain	BASIS
PD5945	FOXP1	cna	gain	BASIS
PD6406	FOXP1	cna	hetloss	BASIS
PD6413	FOXP1	cna	hetloss	BASIS
PD6731	FOXP1	cna	hetloss	BASIS
PD7067	FOXP1	cna	gain	BASIS
PD9585	FOXP1	cna	hetloss	BASIS
TCGA-A2-A25B	FRS2	cna	gain	TCGA
TCGA-AO-A0JL	FRS2	cna	hetloss	TCGA
TCGA-BH-A18R	FRS2	cna	gain	TCGA
TCGA-BH-A1FU	FRS2	cna	hetloss	TCGA
TCGA-C8-A12L	FRS2	cna	gain	TCGA
TCGA-E2-A1L7	FRS2	cna	hetloss	TCGA
TCGA-LL-A5YP	FRS2	cna	hetloss	TCGA
MB-0346	FRS2	cna	gain	METABRIC
MB-2827	FRS2	cna	hetloss	METABRIC

MB-5070	FRS2	cna	gain	METABRIC
MB-5107	FRS2	cna	gain	METABRIC
MB-5465	FRS2	cna	gain	METABRIC
MB-6060	FRS2	cna	gain	METABRIC
MB-6098	FRS2	cna	gain	METABRIC
MTS-T0064	FRS2	cna	gain	METABRIC
PD11327	FRS2	cna	homdel	BASIS
PD11742	FRS2	cna	gain	BASIS
PD22355	FRS2	cna	hetloss	BASIS
PD23578	FRS2	cna	gain	BASIS
PD24186	FRS2	cna	gain	BASIS
PD24202	FRS2	cna	hetloss	BASIS
PD24206	FRS2	cna	gain	BASIS
PD3890	FRS2	cna	hetloss	BASIS
PD3905	FRS2	cna	gain	BASIS
PD4005	FRS2	cna	hetloss	BASIS
PD4006	FRS2	cna	gain	BASIS
PD4826	FRS2	cna	gain	BASIS
PD5945	FRS2	cna	gain	BASIS
PD6406	FRS2	cna	hetloss	BASIS
PD6413	FRS2	cna	hetloss	BASIS
PD6731	FRS2	cna	hetloss	BASIS
PD7067	FRS2	cna	gain	BASIS
PD8621	FRS2	cna	hetloss	BASIS
PD9585	FRS2	cna	hetloss	BASIS
PD9702	FRS2	cna	amp	BASIS
TCGA-AN-A0XU	FSTL3	cna	hetloss	TCGA
TCGA-AO-A0JL	FSTL3	cna	gain	TCGA
TCGA-BH-A0C0	FSTL3	cna	gain	TCGA
TCGA-BH-A1FU	FSTL3	cna	hetloss	TCGA
TCGA-C8-A12L	FSTL3	cna	hetloss	TCGA
TCGA-D8-A27M	FSTL3	cna	hetloss	TCGA
TCGA-E9-A1NC	FSTL3	cna	hetloss	TCGA
TCGA-EW-A1OX	FSTL3	cna	hetloss	TCGA
TCGA-LL-A5YP	FSTL3	cna	hetloss	TCGA
MB-0346	FSTL3	cna	gain	METABRIC
MB-2827	FSTL3	cna	hetloss	METABRIC
MB-5070	FSTL3	cna	hetloss	METABRIC
MB-6098	FSTL3	cna	hetloss	METABRIC
MB-7038	FSTL3	cna	hetloss	METABRIC
MB-0420	FSTL3	cna	hetloss	METABRIC
PD11327	FSTL3	cna	amp	BASIS
PD11742	FSTL3	cna	hetloss	BASIS
PD14442	FSTL3	cna	hetloss	BASIS
PD22355	FSTL3	cna	hetloss	BASIS
PD23578	FSTL3	cna	hetloss	BASIS
PD24206	FSTL3	cna	hetloss	BASIS

PD3890	FSTL3	cna	hetloss	BASIS
PD3905	FSTL3	cna	gain	BASIS
PD4005	FSTL3	cna	gain	BASIS
PD4006	FSTL3	cna	gain	BASIS
PD4826	FSTL3	cna	hetloss	BASIS
PD4967	FSTL3	cna	hetloss	BASIS
PD5930	FSTL3	cna	gain	BASIS
PD5945	FSTL3	cna	gain	BASIS
PD5948	FSTL3	cna	gain	BASIS
PD7067	FSTL3	cna	gain	BASIS
PD8621	FSTL3	cna	gain	BASIS
PD8980	FSTL3	cna	gain	BASIS
PD9004	FSTL3	cna	hetloss	BASIS
PD9585	FSTL3	cna	hetloss	BASIS
TCGA-A2-A25B	GNA12	cna	gain	TCGA
TCGA-AO-A0JL	GNA12	cna	gain	TCGA
TCGA-BH-A0AW	GNA12	cna	gain	TCGA
TCGA-BH-A0C0	GNA12	cna	hetloss	TCGA
TCGA-C8-A12L	GNA12	cna	gain	TCGA
TCGA-D8-A27M	GNA12	cna	hetloss	TCGA
TCGA-E2-A1L7	GNA12	cna	hetloss	TCGA
TCGA-LL-A5YP	GNA12	cna	homdel	TCGA
MB-0346	GNA12	cna	gain	METABRIC
MB-5107	GNA12	cna	hetloss	METABRIC
MB-5465	GNA12	cna	gain	METABRIC
MB-6098	GNA12	cna	hetloss	METABRIC
MB-7038	GNA12	cna	hetloss	METABRIC
MB-0420	GNA12	cna	hetloss	METABRIC
PD13296	GNA12	cna	gain	BASIS
PD13297	GNA12	cna	hetloss	BASIS
PD13771	GNA12	cna	gain	BASIS
PD23574	GNA12	cna	gain	BASIS
PD24202	GNA12	cna	gain	BASIS
PD24206	GNA12	cna	gain	BASIS
PD24337	GNA12	cna	hetloss	BASIS
PD3905	GNA12	cna	gain	BASIS
PD4006	GNA12	cna	gain	BASIS
PD4107	GNA12	cna	amp	BASIS
PD4826	GNA12	cna	gain	BASIS
PD5935	GNA12	cna	amp	BASIS
PD5945	GNA12	cna	amp	BASIS
PD6406	GNA12	cna	hetloss	BASIS
PD6731	GNA12	cna	gain	BASIS
PD7067	GNA12	cna	amp	BASIS
PD7215	GNA12	cna	gain	BASIS
PD8621	GNA12	cna	gain	BASIS
PD8980	GNA12	cna	hetloss	BASIS

PD9004	GNA12	cna	gain	BASIS
PD9702	GNA12	cna	gain	BASIS
TCGA-AO-A0JL	GREM1	cna	hetloss	TCGA
TCGA-BH-A0AW	GREM1	cna	gain	TCGA
TCGA-BH-A0C0	GREM1	cna	hetloss	TCGA
TCGA-C8-A12L	GREM1	cna	hetloss	TCGA
TCGA-D8-A27M	GREM1	cna	hetloss	TCGA
TCGA-E2-A1L7	GREM1	cna	hetloss	TCGA
TCGA-LL-A5YP	GREM1	cna	hetloss	TCGA
MB-2827	GREM1	cna	hetloss	METABRIC
MB-5070	GREM1	cna	hetloss	METABRIC
MB-5465	GREM1	cna	hetloss	METABRIC
MB-6060	GREM1	cna	hetloss	METABRIC
MB-6098	GREM1	cna	hetloss	METABRIC
MB-7038	GREM1	cna	hetloss	METABRIC
MB-0420	GREM1	cna	hetloss	METABRIC
P-0009557-T01-IM5	GREM1	cna	homdel	MSK-IMPACT
PD11327	GREM1	cna	hetloss	BASIS
PD11742	GREM1	cna	hetloss	BASIS
PD13297	GREM1	cna	hetloss	BASIS
PD13299	GREM1	cna	hetloss	BASIS
PD23574	GREM1	cna	hetloss	BASIS
PD23578	GREM1	cna	hetloss	BASIS
PD24202	GREM1	cna	hetloss	BASIS
PD24206	GREM1	cna	gain	BASIS
PD3890	GREM1	cna	hetloss	BASIS
PD3905	GREM1	cna	gain	BASIS
PD4005	GREM1	cna	homdel	BASIS
PD5930	GREM1	cna	hetloss	BASIS
PD5935	GREM1	cna	gain	BASIS
PD5945	GREM1	cna	amp	BASIS
PD6413	GREM1	cna	hetloss	BASIS
PD7067	GREM1	cna	gain	BASIS
PD7215	GREM1	cna	gain	BASIS
PD9004	GREM1	cna	hetloss	BASIS
PD9585	GREM1	cna	hetloss	BASIS
PD9702	GREM1	cna	gain	BASIS
TCGA-A2-A25B	IDH2	cna	amp	TCGA
TCGA-AN-A0XU	IDH2	cna	gain	TCGA
TCGA-AO-A0JL	IDH2	cna	hetloss	TCGA
TCGA-BH-A0AW	IDH2	cna	hetloss	TCGA
TCGA-BH-A0C0	IDH2	cna	gain	TCGA
TCGA-D8-A27M	IDH2	cna	hetloss	TCGA
TCGA-E2-A1L7	IDH2	cna	gain	TCGA
TCGA-LL-A5YP	IDH2	cna	gain	TCGA
MB-0346	IDH2	cna	hetloss	METABRIC
MB-5465	IDH2	cna	hetloss	METABRIC

MB-7038	IDH2	cna	gain	METABRIC
MB-7048	IDH2	cna	amp	METABRIC
MB-0420	IDH2	cna	gain	METABRIC
MTS-T0064	IDH2	cna	amp	METABRIC
PD10014	IDH2	cna	gain	BASIS
PD11742	IDH2	cna	hetloss	BASIS
PD13299	IDH2	cna	gain	BASIS
PD23562	IDH2	cna	gain	BASIS
PD23574	IDH2	cna	gain	BASIS
PD23578	IDH2	cna	gain	BASIS
PD24202	IDH2	cna	hetloss	BASIS
PD24206	IDH2	cna	hetloss	BASIS
PD3890	IDH2	cna	hetloss	BASIS
PD3905	IDH2	cna	gain	BASIS
PD4006	IDH2	cna	gain	BASIS
PD4107	IDH2	cna	gain	BASIS
PD4967	IDH2	cna	hetloss	BASIS
PD5930	IDH2	cna	gain	BASIS
PD5945	IDH2	cna	gain	BASIS
PD6413	IDH2	cna	hetloss	BASIS
PD7067	IDH2	cna	gain	BASIS
PD8621	IDH2	cna	gain	BASIS
PD8980	IDH2	cna	gain	BASIS
PD9585	IDH2	cna	hetloss	BASIS
PD9702	IDH2	cna	gain	BASIS
TCGA-A2-A25B	IRF4	cna	hetloss	TCGA
TCGA-AN-A0XU	IRF4	cna	hetloss	TCGA
TCGA-AO-A0JL	IRF4	cna	gain	TCGA
TCGA-C8-A12L	IRF4	cna	gain	TCGA
TCGA-D8-A27M	IRF4	cna	homdel	TCGA
TCGA-E2-A1L7	IRF4	cna	gain	TCGA
TCGA-E9-A1NC	IRF4	cna	hetloss	TCGA
TCGA-LL-A5YP	IRF4	cna	gain	TCGA
MB-0346	IRF4	cna	hetloss	METABRIC
MB-5070	IRF4	cna	gain	METABRIC
MB-6060	IRF4	cna	gain	METABRIC
MB-6098	IRF4	cna	gain	METABRIC
MB-6271	IRF4	cna	hetloss	METABRIC
MB-7038	IRF4	cna	hetloss	METABRIC
MB-0420	IRF4	cna	hetloss	METABRIC
MTS-T0064	IRF4	cna	homdel	METABRIC
PD10014	IRF4	cna	hetloss	BASIS
PD11327	IRF4	cna	amp	BASIS
PD13296	IRF4	cna	gain	BASIS
PD13297	IRF4	cna	gain	BASIS
PD13299	IRF4	cna	gain	BASIS
PD22355	IRF4	cna	gain	BASIS

PD23562	IRF4	cna	gain	BASIS
PD23578	IRF4	cna	gain	BASIS
PD24202	IRF4	cna	gain	BASIS
PD24206	IRF4	cna	gain	BASIS
PD3890	IRF4	cna	hetloss	BASIS
PD3905	IRF4	cna	amp	BASIS
PD4005	IRF4	cna	hetloss	BASIS
PD4107	IRF4	cna	gain	BASIS
PD4826	IRF4	cna	gain	BASIS
PD5935	IRF4	cna	gain	BASIS
PD5945	IRF4	cna	gain	BASIS
PD6731	IRF4	cna	hetloss	BASIS
PD9702	IRF4	cna	gain	BASIS
TCGA-A2-A25B	KAT6A	cna	hetloss	TCGA
TCGA-AO-A0JL	KAT6A	cna	amp	TCGA
TCGA-BH-A0C0	KAT6A	cna	gain	TCGA
TCGA-BH-A1FU	KAT6A	cna	homdel	TCGA
TCGA-D8-A27M	KAT6A	cna	amp	TCGA
TCGA-E2-A1L7	KAT6A	cna	hetloss	TCGA
TCGA-LL-A5YP	KAT6A	cna	hetloss	TCGA
MB-0346	KAT6A	cna	hetloss	METABRIC
MB-2827	KAT6A	cna	hetloss	METABRIC
MB-5465	KAT6A	cna	hetloss	METABRIC
MB-6060	KAT6A	cna	amp	METABRIC
MB-7038	KAT6A	cna	hetloss	METABRIC
PD11327	KAT6A	cna	homdel	BASIS
PD13296	KAT6A	cna	hetloss	BASIS
PD13299	KAT6A	cna	gain	BASIS
PD13771	KAT6A	cna	amp	BASIS
PD23562	KAT6A	cna	gain	BASIS
PD23574	KAT6A	cna	gain	BASIS
PD23578	KAT6A	cna	hetloss	BASIS
PD24186	KAT6A	cna	gain	BASIS
PD24202	KAT6A	cna	hetloss	BASIS
PD24206	KAT6A	cna	amp	BASIS
PD3905	KAT6A	cna	gain	BASIS
PD4006	KAT6A	cna	gain	BASIS
PD4107	KAT6A	cna	gain	BASIS
PD5930	KAT6A	cna	gain	BASIS
PD5935	KAT6A	cna	gain	BASIS
PD5945	KAT6A	cna	amp	BASIS
PD5948	KAT6A	cna	hetloss	BASIS
PD7067	KAT6A	cna	amp	BASIS
PD7215	KAT6A	cna	amp	BASIS
PD8980	KAT6A	cna	amp	BASIS
PD9004	KAT6A	cna	amp	BASIS
PD9585	KAT6A	cna	gain	BASIS

PD9702	KAT6A	cna	gain	BASIS
TCGA-A2-A25B	LFNG	cna	gain	TCGA
TCGA-AO-A0JL	LFNG	cna	gain	TCGA
TCGA-BH-A0AW	LFNG	cna	gain	TCGA
TCGA-BH-A0C0	LFNG	cna	hetloss	TCGA
TCGA-C8-A12L	LFNG	cna	gain	TCGA
TCGA-D8-A27M	LFNG	cna	hetloss	TCGA
TCGA-E2-A1L7	LFNG	cna	hetloss	TCGA
TCGA-LL-A5YP	LFNG	cna	homdel	TCGA
MB-0346	LFNG	cna	gain	METABRIC
MB-5107	LFNG	cna	hetloss	METABRIC
MB-5465	LFNG	cna	gain	METABRIC
MB-6098	LFNG	cna	hetloss	METABRIC
MB-7038	LFNG	cna	hetloss	METABRIC
MB-0420	LFNG	cna	hetloss	METABRIC
PD13296	LFNG	cna	gain	BASIS
PD13297	LFNG	cna	hetloss	BASIS
PD13771	LFNG	cna	gain	BASIS
PD23574	LFNG	cna	gain	BASIS
PD24202	LFNG	cna	gain	BASIS
PD24206	LFNG	cna	gain	BASIS
PD24337	LFNG	cna	hetloss	BASIS
PD3905	LFNG	cna	gain	BASIS
PD4006	LFNG	cna	gain	BASIS
PD4107	LFNG	cna	amp	BASIS
PD4826	LFNG	cna	gain	BASIS
PD5935	LFNG	cna	amp	BASIS
PD5945	LFNG	cna	amp	BASIS
PD6406	LFNG	cna	hetloss	BASIS
PD6731	LFNG	cna	gain	BASIS
PD7067	LFNG	cna	amp	BASIS
PD7215	LFNG	cna	gain	BASIS
PD8621	LFNG	cna	gain	BASIS
PD8980	LFNG	cna	hetloss	BASIS
PD9004	LFNG	cna	gain	BASIS
PD9702	LFNG	cna	gain	BASIS
TCGA-AO-A0JL	LYL1	cna	gain	TCGA
TCGA-BH-A0C0	LYL1	cna	gain	TCGA
TCGA-BH-A1FU	LYL1	cna	hetloss	TCGA
TCGA-C8-A12L	LYL1	cna	gain	TCGA
TCGA-D8-A27M	LYL1	cna	hetloss	TCGA
TCGA-EW-A1OX	LYL1	cna	hetloss	TCGA
TCGA-LL-A5YP	LYL1	cna	hetloss	TCGA
MB-0346	LYL1	cna	hetloss	METABRIC
MB-2827	LYL1	cna	hetloss	METABRIC
MB-5465	LYL1	cna	gain	METABRIC
MB-6098	LYL1	cna	hetloss	METABRIC

MB-7038	LYL1	cna	hetloss	METABRIC
MB-0420	LYL1	cna	hetloss	METABRIC
PD11742	LYL1	cna	hetloss	BASIS
PD13297	LYL1	cna	gain	BASIS
PD14442	LYL1	cna	hetloss	BASIS
PD23562	LYL1	cna	gain	BASIS
PD23574	LYL1	cna	gain	BASIS
PD24186	LYL1	cna	amp	BASIS
PD24206	LYL1	cna	homdel	BASIS
PD3905	LYL1	cna	gain	BASIS
PD4006	LYL1	cna	amp	BASIS
PD4107	LYL1	cna	gain	BASIS
PD4826	LYL1	cna	gain	BASIS
PD4967	LYL1	cna	hetloss	BASIS
PD5930	LYL1	cna	gain	BASIS
PD5935	LYL1	cna	gain	BASIS
PD5945	LYL1	cna	amp	BASIS
PD5948	LYL1	cna	gain	BASIS
PD6406	LYL1	cna	gain	BASIS
PD6413	LYL1	cna	gain	BASIS
PD7067	LYL1	cna	gain	BASIS
PD7215	LYL1	cna	amp	BASIS
PD8621	LYL1	cna	gain	BASIS
PD9702	LYL1	cna	gain	BASIS
TCGA-A2-A25B	MAGED1	cna	gain	TCGA
TCGA-AN-A0XU	MAGED1	cna	hetloss	TCGA
TCGA-AO-A0JL	MAGED1	cna	gain	TCGA
TCGA-BH-A0AW	MAGED1	cna	gain	TCGA
TCGA-E2-A1L7	MAGED1	cna	hetloss	TCGA
TCGA-E9-A1NC	MAGED1	cna	hetloss	TCGA
MB-0346	MAGED1	cna	gain	METABRIC
MB-2827	MAGED1	cna	hetloss	METABRIC
MB-5465	MAGED1	cna	gain	METABRIC
MB-6098	MAGED1	cna	hetloss	METABRIC
MB-0420	MAGED1	cna	hetloss	METABRIC
PD10014	MAGED1	cna	hetloss	BASIS
PD13296	MAGED1	cna	gain	BASIS
PD13299	MAGED1	cna	gain	BASIS
PD13771	MAGED1	cna	gain	BASIS
PD14442	MAGED1	cna	gain	BASIS
PD23562	MAGED1	cna	hetloss	BASIS
PD23574	MAGED1	cna	gain	BASIS
PD24206	MAGED1	cna	amp	BASIS
PD3905	MAGED1	cna	gain	BASIS
PD4005	MAGED1	cna	hetloss	BASIS
PD4006	MAGED1	cna	hetloss	BASIS
PD4107	MAGED1	cna	gain	BASIS

PD4826	MAGED1	cna	gain	BASIS
PD4967	MAGED1	cna	gain	BASIS
PD5935	MAGED1	cna	gain	BASIS
PD5945	MAGED1	cna	gain	BASIS
PD5948	MAGED1	cna	gain	BASIS
PD6406	MAGED1	cna	gain	BASIS
PD6413	MAGED1	cna	gain	BASIS
PD7067	MAGED1	cna	gain	BASIS
PD7215	MAGED1	cna	amp	BASIS
PD8621	MAGED1	cna	amp	BASIS
PD8980	MAGED1	cna	hetloss	BASIS
PD9004	MAGED1	cna	gain	BASIS
TCGA-A2-A25B	MALT1	cna	hetloss	TCGA
TCGA-AO-A0JL	MALT1	cna	gain	TCGA
TCGA-BH-A0AW	MALT1	cna	hetloss	TCGA
TCGA-BH-A0C0	MALT1	cna	gain	TCGA
TCGA-C8-A12L	MALT1	cna	gain	TCGA
TCGA-D8-A27M	MALT1	cna	hetloss	TCGA
TCGA-E2-A1L7	MALT1	cna	gain	TCGA
TCGA-LL-A5YP	MALT1	cna	homdel	TCGA
MB-0346	MALT1	cna	hetloss	METABRIC
MB-0420	MALT1	cna	hetloss	METABRIC
PD10014	MALT1	cna	hetloss	BASIS
PD11742	MALT1	cna	hetloss	BASIS
PD13296	MALT1	cna	hetloss	BASIS
PD13297	MALT1	cna	gain	BASIS
PD13299	MALT1	cna	gain	BASIS
PD22355	MALT1	cna	hetloss	BASIS
PD23574	MALT1	cna	hetloss	BASIS
PD23578	MALT1	cna	hetloss	BASIS
PD24186	MALT1	cna	gain	BASIS
PD24202	MALT1	cna	hetloss	BASIS
PD24337	MALT1	cna	gain	BASIS
PD4006	MALT1	cna	gain	BASIS
PD4107	MALT1	cna	gain	BASIS
PD4826	MALT1	cna	hetloss	BASIS
PD4967	MALT1	cna	hetloss	BASIS
PD5930	MALT1	cna	gain	BASIS
PD5935	MALT1	cna	hetloss	BASIS
PD5945	MALT1	cna	gain	BASIS
PD5948	MALT1	cna	gain	BASIS
PD6406	MALT1	cna	gain	BASIS
PD6413	MALT1	cna	gain	BASIS
PD8980	MALT1	cna	hetloss	BASIS
PD9004	MALT1	cna	hetloss	BASIS
PD9585	MALT1	cna	hetloss	BASIS
PD9702	MALT1	cna	gain	BASIS

TCGA-A2-A25B	MAX	cna	hetloss	TCGA
TCGA-AO-A0JL	MAX	cna	hetloss	TCGA
TCGA-BH-A0AW	MAX	cna	hetloss	TCGA
TCGA-BH-A1FU	MAX	cna	gain	TCGA
TCGA-C8-A12L	MAX	cna	hetloss	TCGA
TCGA-D8-A27M	MAX	cna	hetloss	TCGA
TCGA-E2-A1L7	MAX	cna	hetloss	TCGA
TCGA-LL-A5YP	MAX	cna	hetloss	TCGA
MB-2827	MAX	cna	hetloss	METABRIC
MB-5070	MAX	cna	hetloss	METABRIC
MB-6098	MAX	cna	hetloss	METABRIC
MB-7038	MAX	cna	hetloss	METABRIC
PD10014	MAX	cna	hetloss	BASIS
PD11742	MAX	cna	hetloss	BASIS
PD13296	MAX	cna	gain	BASIS
PD13297	MAX	cna	hetloss	BASIS
PD13771	MAX	cna	hetloss	BASIS
PD14442	MAX	cna	hetloss	BASIS
PD22355	MAX	cna	hetloss	BASIS
PD23578	MAX	cna	hetloss	BASIS
PD24202	MAX	cna	hetloss	BASIS
PD24206	MAX	cna	gain	BASIS
PD24337	MAX	cna	hetloss	BASIS
PD3890	MAX	cna	hetloss	BASIS
PD3905	MAX	cna	gain	BASIS
PD4005	MAX	cna	hetloss	BASIS
PD4006	MAX	cna	gain	BASIS
PD5945	MAX	cna	gain	BASIS
PD6406	MAX	cna	hetloss	BASIS
PD7067	MAX	cna	gain	BASIS
PD7215	MAX	cna	hetloss	BASIS
PD8980	MAX	cna	gain	BASIS
PD9004	MAX	cna	hetloss	BASIS
PD9585	MAX	cna	gain	BASIS
PD9702	MAX	cna	gain	BASIS
TCGA-AN-A0XU	MEF2B	cna	hetloss	TCGA
TCGA-AO-A0JL	MEF2B	cna	gain	TCGA
TCGA-BH-A0AW	MEF2B	cna	gain	TCGA
TCGA-BH-A0C0	MEF2B	cna	gain	TCGA
TCGA-BH-A1FU	MEF2B	cna	hetloss	TCGA
TCGA-C8-A12L	MEF2B	cna	gain	TCGA
TCGA-D8-A27M	MEF2B	cna	hetloss	TCGA
TCGA-EW-A1OX	MEF2B	cna	hetloss	TCGA
TCGA-LL-A5YP	MEF2B	cna	hetloss	TCGA
MB-0346	MEF2B	cna	hetloss	METABRIC
MB-2827	MEF2B	cna	hetloss	METABRIC
MB-6060	MEF2B	cna	gain	METABRIC

MB-6098	MEF2B	cna	hetloss	METABRIC
MB-0420	MEF2B	cna	hetloss	METABRIC
MTS-T0064	MEF2B	cna	amp	METABRIC
PD13299	MEF2B	cna	gain	BASIS
PD14442	MEF2B	cna	hetloss	BASIS
PD22355	MEF2B	cna	gain	BASIS
PD23562	MEF2B	cna	gain	BASIS
PD23574	MEF2B	cna	gain	BASIS
PD24186	MEF2B	cna	amp	BASIS
PD24206	MEF2B	cna	hetloss	BASIS
PD3890	MEF2B	cna	hetloss	BASIS
PD3905	MEF2B	cna	gain	BASIS
PD4006	MEF2B	cna	amp	BASIS
PD4107	MEF2B	cna	gain	BASIS
PD4826	MEF2B	cna	gain	BASIS
PD4967	MEF2B	cna	hetloss	BASIS
PD5930	MEF2B	cna	gain	BASIS
PD5935	MEF2B	cna	gain	BASIS
PD5945	MEF2B	cna	amp	BASIS
PD5948	MEF2B	cna	gain	BASIS
PD7067	MEF2B	cna	gain	BASIS
PD7215	MEF2B	cna	amp	BASIS
PD9702	MEF2B	cna	gain	BASIS
TCGA-AN-A0XU	MITF	cna	hetloss	TCGA
TCGA-BH-A0C0	MITF	cna	hetloss	TCGA
TCGA-BH-A18R	MITF	cna	homdel	TCGA
TCGA-BH-A1FU	MITF	cna	hetloss	TCGA
TCGA-C8-A12L	MITF	cna	hetloss	TCGA
TCGA-D8-A27M	MITF	cna	hetloss	TCGA
TCGA-E2-A1L7	MITF	cna	homdel	TCGA
TCGA-E9-A1NC	MITF	cna	hetloss	TCGA
TCGA-LL-A5YP	MITF	cna	gain	TCGA
MB-0346	MITF	cna	hetloss	METABRIC
MB-2827	MITF	cna	hetloss	METABRIC
MB-5465	MITF	cna	hetloss	METABRIC
MB-6098	MITF	cna	hetloss	METABRIC
MB-7048	MITF	cna	hetloss	METABRIC
MB-0420	MITF	cna	hetloss	METABRIC
MTS-T0064	MITF	cna	gain	METABRIC
PD10014	MITF	cna	hetloss	BASIS
PD11742	MITF	cna	hetloss	BASIS
PD13296	MITF	cna	hetloss	BASIS
PD13297	MITF	cna	hetloss	BASIS
PD13299	MITF	cna	gain	BASIS
PD13771	MITF	cna	hetloss	BASIS
PD23561	MITF	cna	hetloss	BASIS
PD23574	MITF	cna	gain	BASIS

PD23578	MITF	cna	hetloss	BASIS
PD24202	MITF	cna	hetloss	BASIS
PD24337	MITF	cna	hetloss	BASIS
PD3905	MITF	cna	gain	BASIS
PD4107	MITF	cna	gain	BASIS
PD5945	MITF	cna	gain	BASIS
PD6406	MITF	cna	hetloss	BASIS
PD6413	MITF	cna	hetloss	BASIS
PD6731	MITF	cna	hetloss	BASIS
PD8980	MITF	cna	hetloss	BASIS
PD9585	MITF	cna	hetloss	BASIS
TCGA-A2-A25B	MKI67	cna	hetloss	TCGA
TCGA-AN-A0XU	MKI67	cna	hetloss	TCGA
TCGA-AO-A0JL	MKI67	cna	hetloss	TCGA
TCGA-C8-A12L	MKI67	cna	hetloss	TCGA
TCGA-E2-A1L7	MKI67	cna	hetloss	TCGA
TCGA-E9-A1NC	MKI67	cna	hetloss	TCGA
TCGA-LL-A5YP	MKI67	cna	gain	TCGA
MB-0346	MKI67	cna	hetloss	METABRIC
MB-2827	MKI67	cna	hetloss	METABRIC
MB-5070	MKI67	cna	hetloss	METABRIC
MB-5465	MKI67	cna	hetloss	METABRIC
MB-6098	MKI67	cna	hetloss	METABRIC
MB-7038	MKI67	cna	hetloss	METABRIC
PD10014	MKI67	cna	gain	BASIS
PD11327	MKI67	cna	gain	BASIS
PD11742	MKI67	cna	hetloss	BASIS
PD23562	MKI67	cna	gain	BASIS
PD24186	MKI67	cna	gain	BASIS
PD24202	MKI67	cna	hetloss	BASIS
PD24206	MKI67	cna	gain	BASIS
PD24337	MKI67	cna	hetloss	BASIS
PD3890	MKI67	cna	hetloss	BASIS
PD3905	MKI67	cna	gain	BASIS
PD4005	MKI67	cna	hetloss	BASIS
PD4006	MKI67	cna	hetloss	BASIS
PD4107	MKI67	cna	gain	BASIS
PD5935	MKI67	cna	gain	BASIS
PD5945	MKI67	cna	gain	BASIS
PD5948	MKI67	cna	gain	BASIS
PD6406	MKI67	cna	hetloss	BASIS
PD6731	MKI67	cna	hetloss	BASIS
PD7067	MKI67	cna	gain	BASIS
PD7215	MKI67	cna	gain	BASIS
PD8621	MKI67	cna	gain	BASIS
PD8980	MKI67	cna	hetloss	BASIS
TCGA-A2-A25B	MRE11A	cna	hetloss	TCGA

TCGA-AN-A0XU	MRE11A	cna	gain	TCGA
TCGA-AO-A0JL	MRE11A	cna	hetloss	TCGA
TCGA-D8-A27M	MRE11A	cna	gain	TCGA
TCGA-EW-A1OX	MRE11A	cna	homdel	TCGA
TCGA-LL-A5YP	MRE11A	cna	hetloss	TCGA
MB-0346	MRE11A	cna	hetloss	METABRIC
MB-5070	MRE11A	cna	hetloss	METABRIC
MB-6271	MRE11A	cna	hetloss	METABRIC
MB-7032	MRE11A	cna	hetloss	METABRIC
MB-7048	MRE11A	cna	hetloss	METABRIC
MTS-T0064	MRE11A	cna	gain	METABRIC
PD10014	MRE11A	cna	gain	BASIS
PD11327	MRE11A	cna	gain	BASIS
PD11742	MRE11A	cna	hetloss	BASIS
PD13299	MRE11A	cna	gain	BASIS
PD13771	MRE11A	cna	hetloss	BASIS
PD22355	MRE11A	cna	hetloss	BASIS
PD23574	MRE11A	cna	gain	BASIS
PD23578	MRE11A	cna	hetloss	BASIS
PD24186	MRE11A	cna	gain	BASIS
PD24206	MRE11A	cna	gain	BASIS
PD24337	MRE11A	cna	hetloss	BASIS
PD3905	MRE11A	cna	gain	BASIS
PD4006	MRE11A	cna	hetloss	BASIS
PD4826	MRE11A	cna	hetloss	BASIS
PD5935	MRE11A	cna	gain	BASIS
PD5945	MRE11A	cna	gain	BASIS
PD5948	MRE11A	cna	gain	BASIS
PD6413	MRE11A	cna	hetloss	BASIS
PD7067	MRE11A	cna	amp	BASIS
PD7215	MRE11A	cna	gain	BASIS
PD8980	MRE11A	cna	gain	BASIS
PD9585	MRE11A	cna	gain	BASIS
PD9702	MRE11A	cna	gain	BASIS
TCGA-AN-A0XU	PARP2	cna	gain	TCGA
TCGA-AO-A0JL	PARP2	cna	amp	TCGA
TCGA-BH-A0AW	PARP2	cna	hetloss	TCGA
TCGA-BH-A1FU	PARP2	cna	gain	TCGA
TCGA-D8-A27M	PARP2	cna	gain	TCGA
TCGA-LL-A5YP	PARP2	cna	hetloss	TCGA
MB-5070	PARP2	cna	hetloss	METABRIC
MB-6060	PARP2	cna	gain	METABRIC
MB-7048	PARP2	cna	gain	METABRIC
PD11327	PARP2	cna	homdel	BASIS
PD13296	PARP2	cna	gain	BASIS
PD13299	PARP2	cna	gain	BASIS
PD22355	PARP2	cna	hetloss	BASIS

PD23574	PARP2	cna	gain	BASIS
PD23578	PARP2	cna	hetloss	BASIS
PD24186	PARP2	cna	gain	BASIS
PD24202	PARP2	cna	hetloss	BASIS
PD24206	PARP2	cna	gain	BASIS
PD24337	PARP2	cna	hetloss	BASIS
PD3890	PARP2	cna	hetloss	BASIS
PD3905	PARP2	cna	gain	BASIS
PD4005	PARP2	cna	hetloss	BASIS
PD4107	PARP2	cna	gain	BASIS
PD4967	PARP2	cna	hetloss	BASIS
PD5930	PARP2	cna	hetloss	BASIS
PD5935	PARP2	cna	gain	BASIS
PD5945	PARP2	cna	amp	BASIS
PD5948	PARP2	cna	gain	BASIS
PD6406	PARP2	cna	hetloss	BASIS
PD6731	PARP2	cna	hetloss	BASIS
PD7215	PARP2	cna	gain	BASIS
PD8621	PARP2	cna	gain	BASIS
PD8980	PARP2	cna	gain	BASIS
PD9004	PARP2	cna	gain	BASIS
PD9702	PARP2	cna	gain	BASIS
TCGA-AO-A0JL	PASK	cna	hetloss	TCGA
TCGA-BH-A0AW	PASK	cna	hetloss	TCGA
TCGA-C8-A12L	PASK	cna	hetloss	TCGA
TCGA-D8-A27M	PASK	cna	gain	TCGA
TCGA-E2-A1L7	PASK	cna	hetloss	TCGA
TCGA-E9-A1NC	PASK	cna	hetloss	TCGA
MB-5107	PASK	cna	hetloss	METABRIC
MB-6060	PASK	cna	hetloss	METABRIC
MB-6098	PASK	cna	hetloss	METABRIC
MB-6271	PASK	cna	hetloss	METABRIC
MB-7048	PASK	cna	hetloss	METABRIC
PD10014	PASK	cna	gain	BASIS
PD11327	PASK	cna	gain	BASIS
PD11742	PASK	cna	hetloss	BASIS
PD13296	PASK	cna	hetloss	BASIS
PD13297	PASK	cna	hetloss	BASIS
PD23578	PASK	cna	hetloss	BASIS
PD24186	PASK	cna	gain	BASIS
PD24202	PASK	cna	hetloss	BASIS
PD24206	PASK	cna	gain	BASIS
PD3890	PASK	cna	hetloss	BASIS
PD3905	PASK	cna	gain	BASIS
PD4005	PASK	cna	hetloss	BASIS
PD4006	PASK	cna	gain	BASIS
PD4967	PASK	cna	hetloss	BASIS

PD5935	PASK	cna	gain	BASIS
PD5945	PASK	cna	gain	BASIS
PD5948	PASK	cna	gain	BASIS
PD6406	PASK	cna	hetloss	BASIS
PD6413	PASK	cna	hetloss	BASIS
PD6731	PASK	cna	hetloss	BASIS
PD7067	PASK	cna	gain	BASIS
PD7215	PASK	cna	gain	BASIS
PD8980	PASK	cna	hetloss	BASIS
PD9585	PASK	cna	hetloss	BASIS
TCGA-A2-A25B	PMAIP1	cna	hetloss	TCGA
TCGA-AO-A0JL	PMAIP1	cna	gain	TCGA
TCGA-BH-A0AW	PMAIP1	cna	hetloss	TCGA
TCGA-BH-A0C0	PMAIP1	cna	gain	TCGA
TCGA-C8-A12L	PMAIP1	cna	gain	TCGA
TCGA-D8-A27M	PMAIP1	cna	hetloss	TCGA
TCGA-E2-A1L7	PMAIP1	cna	gain	TCGA
TCGA-LL-A5YP	PMAIP1	cna	homdel	TCGA
MB-0346	PMAIP1	cna	hetloss	METABRIC
MB-0420	PMAIP1	cna	hetloss	METABRIC
MTS-T0064	PMAIP1	cna	hetloss	METABRIC
PD10014	PMAIP1	cna	hetloss	BASIS
PD11742	PMAIP1	cna	hetloss	BASIS
PD13296	PMAIP1	cna	hetloss	BASIS
PD13297	PMAIP1	cna	gain	BASIS
PD13299	PMAIP1	cna	gain	BASIS
PD22355	PMAIP1	cna	hetloss	BASIS
PD23574	PMAIP1	cna	hetloss	BASIS
PD23578	PMAIP1	cna	hetloss	BASIS
PD24186	PMAIP1	cna	gain	BASIS
PD24202	PMAIP1	cna	hetloss	BASIS
PD24337	PMAIP1	cna	gain	BASIS
PD4006	PMAIP1	cna	gain	BASIS
PD4107	PMAIP1	cna	gain	BASIS
PD4826	PMAIP1	cna	hetloss	BASIS
PD4967	PMAIP1	cna	hetloss	BASIS
PD5930	PMAIP1	cna	gain	BASIS
PD5935	PMAIP1	cna	hetloss	BASIS
PD5945	PMAIP1	cna	gain	BASIS
PD5948	PMAIP1	cna	gain	BASIS
PD6406	PMAIP1	cna	gain	BASIS
PD8980	PMAIP1	cna	hetloss	BASIS
PD9004	PMAIP1	cna	hetloss	BASIS
PD9585	PMAIP1	cna	hetloss	BASIS
PD9702	PMAIP1	cna	gain	BASIS
TCGA-A2-A25B	PML	cna	gain	TCGA
TCGA-AN-A0XU	PML	cna	amp	TCGA

TCGA-AO-A0JL	PML	cna	hetloss	TCGA
TCGA-BH-A0AW	PML	cna	hetloss	TCGA
TCGA-BH-A0C0	PML	cna	hetloss	TCGA
TCGA-D8-A27M	PML	cna	hetloss	TCGA
TCGA-E2-A1L7	PML	cna	gain	TCGA
TCGA-LL-A5YP	PML	cna	hetloss	TCGA
MB-0346	PML	cna	hetloss	METABRIC
MB-5070	PML	cna	hetloss	METABRIC
MB-6060	PML	cna	hetloss	METABRIC
MB-7038	PML	cna	gain	METABRIC
PD11327	PML	cna	gain	BASIS
PD11742	PML	cna	hetloss	BASIS
PD13296	PML	cna	homdel	BASIS
PD13297	PML	cna	hetloss	BASIS
PD13299	PML	cna	gain	BASIS
PD22355	PML	cna	hetloss	BASIS
PD23562	PML	cna	gain	BASIS
PD23574	PML	cna	gain	BASIS
PD23578	PML	cna	gain	BASIS
PD24202	PML	cna	hetloss	BASIS
PD24206	PML	cna	gain	BASIS
PD3890	PML	cna	hetloss	BASIS
PD3905	PML	cna	gain	BASIS
PD4006	PML	cna	gain	BASIS
PD5945	PML	cna	gain	BASIS
PD5948	PML	cna	gain	BASIS
PD6406	PML	cna	hetloss	BASIS
PD7067	PML	cna	gain	BASIS
PD7215	PML	cna	gain	BASIS
PD8621	PML	cna	gain	BASIS
PD8980	PML	cna	gain	BASIS
PD9585	PML	cna	hetloss	BASIS
PD9702	PML	cna	gain	BASIS
TCGA-A2-A25B	POLE	cna	gain	TCGA
TCGA-AN-A0XU	POLE	cna	hetloss	TCGA
TCGA-AO-A0JL	POLE	cna	hetloss	TCGA
TCGA-BH-A0C0	POLE	cna	hetloss	TCGA
TCGA-BH-A18R	POLE	cna	gain	TCGA
TCGA-BH-A1FU	POLE	cna	hetloss	TCGA
TCGA-D8-A27M	POLE	cna	hetloss	TCGA
TCGA-E2-A1L7	POLE	cna	hetloss	TCGA
TCGA-LL-A5YP	POLE	cna	hetloss	TCGA
MB-0346	POLE	cna	gain	METABRIC
MB-2827	POLE	cna	hetloss	METABRIC
MB-5070	POLE	cna	hetloss	METABRIC
MB-5465	POLE	cna	hetloss	METABRIC
MB-7048	POLE	cna	hetloss	METABRIC

MTS-T0064	POLE	cna	gain	METABRIC
PD11327	POLE	cna	gain	BASIS
PD13296	POLE	cna	hetloss	BASIS
PD13771	POLE	cna	gain	BASIS
PD22355	POLE	cna	hetloss	BASIS
PD23574	POLE	cna	gain	BASIS
PD23578	POLE	cna	hetloss	BASIS
PD24186	POLE	cna	gain	BASIS
PD24206	POLE	cna	gain	BASIS
PD3905	POLE	cna	gain	BASIS
PD4005	POLE	cna	hetloss	BASIS
PD4006	POLE	cna	amp	BASIS
PD4107	POLE	cna	gain	BASIS
PD4826	POLE	cna	gain	BASIS
PD5935	POLE	cna	gain	BASIS
PD5945	POLE	cna	gain	BASIS
PD5948	POLE	cna	gain	BASIS
PD6731	POLE	cna	hetloss	BASIS
PD7067	POLE	cna	gain	BASIS
PD9585	POLE	cna	hetloss	BASIS
PD9702	POLE	cna	gain	BASIS
TCGA-AO-A0JL	PPP2R1A	cna	hetloss	TCGA
TCGA-BH-A0AW	PPP2R1A	cna	gain	TCGA
TCGA-BH-A0C0	PPP2R1A	cna	hetloss	TCGA
TCGA-BH-A1FU	PPP2R1A	cna	hetloss	TCGA
TCGA-D8-A27M	PPP2R1A	cna	hetloss	TCGA
TCGA-E2-A1L7	PPP2R1A	cna	hetloss	TCGA
TCGA-EW-A1OX	PPP2R1A	cna	hetloss	TCGA
TCGA-LL-A5YP	PPP2R1A	cna	gain	TCGA
MB-0346	PPP2R1A	cna	hetloss	METABRIC
MB-2827	PPP2R1A	cna	hetloss	METABRIC
MB-6098	PPP2R1A	cna	hetloss	METABRIC
MB-7048	PPP2R1A	cna	gain	METABRIC
PD11327	PPP2R1A	cna	gain	BASIS
PD13296	PPP2R1A	cna	gain	BASIS
PD13299	PPP2R1A	cna	gain	BASIS
PD13771	PPP2R1A	cna	hetloss	BASIS
PD23562	PPP2R1A	cna	gain	BASIS
PD23574	PPP2R1A	cna	gain	BASIS
PD24186	PPP2R1A	cna	gain	BASIS
PD24206	PPP2R1A	cna	hetloss	BASIS
PD24337	PPP2R1A	cna	hetloss	BASIS
PD3890	PPP2R1A	cna	hetloss	BASIS
PD3905	PPP2R1A	cna	gain	BASIS
PD4006	PPP2R1A	cna	gain	BASIS
PD4107	PPP2R1A	cna	gain	BASIS
PD4826	PPP2R1A	cna	gain	BASIS

PD5935	PPP2R1A	cna	gain	BASIS
PD5945	PPP2R1A	cna	gain	BASIS
PD5948	PPP2R1A	cna	gain	BASIS
PD6406	PPP2R1A	cna	gain	BASIS
PD7067	PPP2R1A	cna	gain	BASIS
PD7215	PPP2R1A	cna	gain	BASIS
PD8621	PPP2R1A	cna	gain	BASIS
PD8980	PPP2R1A	cna	hetloss	BASIS
PD9585	PPP2R1A	cna	gain	BASIS
TCGA-A2-A25B	PTCH1	cna	gain	TCGA
TCGA-AN-A0XU	PTCH1	cna	hetloss	TCGA
TCGA-AO-A0JL	PTCH1	cna	hetloss	TCGA
TCGA-BH-A1FU	PTCH1	cna	hetloss	TCGA
TCGA-C8-A12L	PTCH1	cna	hetloss	TCGA
TCGA-E2-A1L7	PTCH1	cna	hetloss	TCGA
TCGA-E9-A1NC	PTCH1	cna	hetloss	TCGA
TCGA-LL-A5YP	PTCH1	cna	hetloss	TCGA
MB-0346	PTCH1	cna	hetloss	METABRIC
MB-2827	PTCH1	cna	amp	METABRIC
MB-5070	PTCH1	cna	hetloss	METABRIC
MB-5107	PTCH1	cna	hetloss	METABRIC
MB-6098	PTCH1	cna	hetloss	METABRIC
MB-7038	PTCH1	cna	hetloss	METABRIC
PD11327	PTCH1	cna	gain	BASIS
PD13296	PTCH1	cna	gain	BASIS
PD13771	PTCH1	cna	hetloss	BASIS
PD23562	PTCH1	cna	gain	BASIS
PD23578	PTCH1	cna	hetloss	BASIS
PD24186	PTCH1	cna	amp	BASIS
PD24202	PTCH1	cna	hetloss	BASIS
PD24206	PTCH1	cna	gain	BASIS
PD3905	PTCH1	cna	gain	BASIS
PD4005	PTCH1	cna	gain	BASIS
PD4006	PTCH1	cna	gain	BASIS
PD4107	PTCH1	cna	gain	BASIS
PD5945	PTCH1	cna	amp	BASIS
PD5948	PTCH1	cna	gain	BASIS
PD6406	PTCH1	cna	hetloss	BASIS
PD6731	PTCH1	cna	hetloss	BASIS
PD7067	PTCH1	cna	gain	BASIS
PD7215	PTCH1	cna	gain	BASIS
PD8621	PTCH1	cna	gain	BASIS
PD9004	PTCH1	cna	gain	BASIS
PD9585	PTCH1	cna	hetloss	BASIS
TCGA-A2-A25B	PTPRD	cna	homdel	TCGA
TCGA-AO-A0JL	PTPRD	cna	gain	TCGA
TCGA-BH-A0AW	PTPRD	cna	homdel	TCGA

TCGA-BH-A1FU	PTPRD	cna	hetloss	TCGA
TCGA-C8-A12L	PTPRD	cna	gain	TCGA
TCGA-E2-A1L7	PTPRD	cna	hetloss	TCGA
TCGA-E9-A1NC	PTPRD	cna	amp	TCGA
MB-6060	PTPRD	cna	gain	METABRIC
MB-0420	PTPRD	cna	hetloss	METABRIC
MTS-T0064	PTPRD	cna	amp	METABRIC
P-0009557-T01-IM5	PTPRD	cna	amp	MSK-IMPACT
PD11327	PTPRD	cna	hetloss	BASIS
PD13296	PTPRD	cna	gain	BASIS
PD13297	PTPRD	cna	hetloss	BASIS
PD13299	PTPRD	cna	amp	BASIS
PD22355	PTPRD	cna	gain	BASIS
PD23574	PTPRD	cna	gain	BASIS
PD24186	PTPRD	cna	gain	BASIS
PD24337	PTPRD	cna	gain	BASIS
PD3905	PTPRD	cna	gain	BASIS
PD4005	PTPRD	cna	hetloss	BASIS
PD4006	PTPRD	cna	gain	BASIS
PD4107	PTPRD	cna	gain	BASIS
PD4826	PTPRD	cna	gain	BASIS
PD5945	PTPRD	cna	gain	BASIS
PD5948	PTPRD	cna	hetloss	BASIS
PD6406	PTPRD	cna	homdel	BASIS
PD6413	PTPRD	cna	gain	BASIS
PD6731	PTPRD	cna	gain	BASIS
PD7067	PTPRD	cna	amp	BASIS
PD8621	PTPRD	cna	gain	BASIS
PD8980	PTPRD	cna	gain	BASIS
PD9004	PTPRD	cna	gain	BASIS
PD9585	PTPRD	cna	hetloss	BASIS
PD9702	PTPRD	cna	gain	BASIS
TCGA-AN-A0XU	PTPRS	cna	hetloss	TCGA
TCGA-AO-A0JL	PTPRS	cna	gain	TCGA
TCGA-BH-A0C0	PTPRS	cna	gain	TCGA
TCGA-BH-A1FU	PTPRS	cna	hetloss	TCGA
TCGA-C8-A12L	PTPRS	cna	gain	TCGA
TCGA-D8-A27M	PTPRS	cna	hetloss	TCGA
TCGA-E9-A1NC	PTPRS	cna	hetloss	TCGA
TCGA-EW-A1OX	PTPRS	cna	hetloss	TCGA
TCGA-LL-A5YP	PTPRS	cna	hetloss	TCGA
MB-0346	PTPRS	cna	gain	METABRIC
MB-2827	PTPRS	cna	hetloss	METABRIC
MB-5070	PTPRS	cna	hetloss	METABRIC
MB-5465	PTPRS	cna	gain	METABRIC
MB-6060	PTPRS	cna	gain	METABRIC
MB-6098	PTPRS	cna	hetloss	METABRIC

MB-7038	PTPRS	cna	hetloss	METABRIC
MB-0420	PTPRS	cna	hetloss	METABRIC
PD10014	PTPRS	cna	hetloss	BASIS
PD11742	PTPRS	cna	hetloss	BASIS
PD14442	PTPRS	cna	hetloss	BASIS
PD23574	PTPRS	cna	gain	BASIS
PD24202	PTPRS	cna	gain	BASIS
PD24206	PTPRS	cna	hetloss	BASIS
PD3905	PTPRS	cna	gain	BASIS
PD4006	PTPRS	cna	gain	BASIS
PD4107	PTPRS	cna	gain	BASIS
PD4826	PTPRS	cna	gain	BASIS
PD5930	PTPRS	cna	gain	BASIS
PD5945	PTPRS	cna	gain	BASIS
PD5948	PTPRS	cna	gain	BASIS
PD7067	PTPRS	cna	gain	BASIS
PD8621	PTPRS	cna	gain	BASIS
PD8980	PTPRS	cna	gain	BASIS
PD9004	PTPRS	cna	hetloss	BASIS
PD9702	PTPRS	cna	gain	BASIS
TCGA-A2-A25B	RABEP1	cna	hetloss	TCGA
TCGA-AN-A0XU	RABEP1	cna	gain	TCGA
TCGA-AO-A0JL	RABEP1	cna	gain	TCGA
TCGA-BH-A0AW	RABEP1	cna	hetloss	TCGA
TCGA-BH-A0C0	RABEP1	cna	hetloss	TCGA
TCGA-C8-A12L	RABEP1	cna	hetloss	TCGA
TCGA-D8-A27M	RABEP1	cna	hetloss	TCGA
TCGA-E2-A1L7	RABEP1	cna	hetloss	TCGA
TCGA-E9-A1NC	RABEP1	cna	hetloss	TCGA
TCGA-LL-A5YP	RABEP1	cna	gain	TCGA
MB-0346	RABEP1	cna	hetloss	METABRIC
MB-2827	RABEP1	cna	hetloss	METABRIC
MB-5107	RABEP1	cna	hetloss	METABRIC
MB-6271	RABEP1	cna	hetloss	METABRIC
MB-7032	RABEP1	cna	gain	METABRIC
MB-7048	RABEP1	cna	hetloss	METABRIC
MB-0420	RABEP1	cna	hetloss	METABRIC
PD10014	RABEP1	cna	hetloss	BASIS
PD11742	RABEP1	cna	hetloss	BASIS
PD13296	RABEP1	cna	hetloss	BASIS
PD13771	RABEP1	cna	hetloss	BASIS
PD24202	RABEP1	cna	hetloss	BASIS
PD24206	RABEP1	cna	hetloss	BASIS
PD24337	RABEP1	cna	hetloss	BASIS
PD3890	RABEP1	cna	hetloss	BASIS
PD3905	RABEP1	cna	gain	BASIS
PD4005	RABEP1	cna	hetloss	BASIS

PD4826	RABEP1	cna	hetloss	BASIS
PD4967	RABEP1	cna	hetloss	BASIS
PD5945	RABEP1	cna	gain	BASIS
PD6406	RABEP1	cna	hetloss	BASIS
PD6413	RABEP1	cna	hetloss	BASIS
PD6731	RABEP1	cna	hetloss	BASIS
PD7067	RABEP1	cna	gain	BASIS
PD9585	RABEP1	cna	hetloss	BASIS
TCGA-A2-A25B	RAD51B	cna	hetloss	TCGA
TCGA-AO-A0JL	RAD51B	cna	hetloss	TCGA
TCGA-BH-A0AW	RAD51B	cna	hetloss	TCGA
TCGA-BH-A1FU	RAD51B	cna	gain	TCGA
TCGA-C8-A12L	RAD51B	cna	hetloss	TCGA
TCGA-D8-A27M	RAD51B	cna	hetloss	TCGA
TCGA-E2-A1L7	RAD51B	cna	hetloss	TCGA
TCGA-LL-A5YP	RAD51B	cna	hetloss	TCGA
MB-2827	RAD51B	cna	hetloss	METABRIC
MB-5070	RAD51B	cna	hetloss	METABRIC
MB-6060	RAD51B	cna	gain	METABRIC
MB-6098	RAD51B	cna	hetloss	METABRIC
MB-7038	RAD51B	cna	hetloss	METABRIC
MTS-T0064	RAD51B	cna	gain	METABRIC
PD11327	RAD51B	cna	amp	BASIS
PD11742	RAD51B	cna	hetloss	BASIS
PD13296	RAD51B	cna	gain	BASIS
PD13771	RAD51B	cna	hetloss	BASIS
PD14442	RAD51B	cna	hetloss	BASIS
PD23578	RAD51B	cna	hetloss	BASIS
PD24186	RAD51B	cna	gain	BASIS
PD24202	RAD51B	cna	hetloss	BASIS
PD24206	RAD51B	cna	gain	BASIS
PD24337	RAD51B	cna	hetloss	BASIS
PD3890	RAD51B	cna	hetloss	BASIS
PD3905	RAD51B	cna	gain	BASIS
PD4005	RAD51B	cna	hetloss	BASIS
PD4006	RAD51B	cna	gain	BASIS
PD5945	RAD51B	cna	gain	BASIS
PD6406	RAD51B	cna	hetloss	BASIS
PD7067	RAD51B	cna	gain	BASIS
PD7215	RAD51B	cna	hetloss	BASIS
PD8980	RAD51B	cna	gain	BASIS
PD9004	RAD51B	cna	hetloss	BASIS
PD9702	RAD51B	cna	gain	BASIS
TCGA-A2-A25B	RALGDS	cna	gain	TCGA
TCGA-AN-A0XU	RALGDS	cna	hetloss	TCGA
TCGA-AO-A0JL	RALGDS	cna	hetloss	TCGA
TCGA-BH-A0C0	RALGDS	cna	gain	TCGA

TCGA-C8-A12L	RALGDS	cna	gain	TCGA
TCGA-E2-A1L7	RALGDS	cna	hetloss	TCGA
TCGA-E9-A1NC	RALGDS	cna	hetloss	TCGA
TCGA-LL-A5YP	RALGDS	cna	hetloss	TCGA
MB-0346	RALGDS	cna	hetloss	METABRIC
MB-2827	RALGDS	cna	amp	METABRIC
MB-5070	RALGDS	cna	hetloss	METABRIC
MB-7038	RALGDS	cna	hetloss	METABRIC
MB-0420	RALGDS	cna	hetloss	METABRIC
PD10014	RALGDS	cna	gain	BASIS
PD11327	RALGDS	cna	gain	BASIS
PD13299	RALGDS	cna	gain	BASIS
PD22355	RALGDS	cna	gain	BASIS
PD24186	RALGDS	cna	gain	BASIS
PD24202	RALGDS	cna	hetloss	BASIS
PD24206	RALGDS	cna	gain	BASIS
PD24337	RALGDS	cna	hetloss	BASIS
PD3890	RALGDS	cna	hetloss	BASIS
PD3905	RALGDS	cna	gain	BASIS
PD4005	RALGDS	cna	hetloss	BASIS
PD4006	RALGDS	cna	gain	BASIS
PD4107	RALGDS	cna	hetloss	BASIS
PD4826	RALGDS	cna	gain	BASIS
PD5945	RALGDS	cna	gain	BASIS
PD5948	RALGDS	cna	gain	BASIS
PD6406	RALGDS	cna	hetloss	BASIS
PD6731	RALGDS	cna	hetloss	BASIS
PD7067	RALGDS	cna	gain	BASIS
PD7215	RALGDS	cna	gain	BASIS
PD8621	RALGDS	cna	gain	BASIS
PD9004	RALGDS	cna	hetloss	BASIS
TCGA-AN-A0XU	RPL15	cna	hetloss	TCGA
TCGA-BH-A0C0	RPL15	cna	hetloss	TCGA
TCGA-BH-A18R	RPL15	cna	gain	TCGA
TCGA-BH-A1FU	RPL15	cna	hetloss	TCGA
TCGA-D8-A27M	RPL15	cna	hetloss	TCGA
TCGA-E9-A1NC	RPL15	cna	hetloss	TCGA
TCGA-LL-A5YP	RPL15	cna	amp	TCGA
MB-2827	RPL15	cna	hetloss	METABRIC
MB-7038	RPL15	cna	hetloss	METABRIC
MB-7048	RPL15	cna	hetloss	METABRIC
MB-0420	RPL15	cna	hetloss	METABRIC
PD10014	RPL15	cna	hetloss	BASIS
PD11327	RPL15	cna	hetloss	BASIS
PD11742	RPL15	cna	gain	BASIS
PD13296	RPL15	cna	gain	BASIS
PD13297	RPL15	cna	hetloss	BASIS

PD13299	RPL15	cna	gain	BASIS
PD13771	RPL15	cna	gain	BASIS
PD22355	RPL15	cna	hetloss	BASIS
PD23561	RPL15	cna	hetloss	BASIS
PD23562	RPL15	cna	gain	BASIS
PD23574	RPL15	cna	gain	BASIS
PD24202	RPL15	cna	hetloss	BASIS
PD24206	RPL15	cna	gain	BASIS
PD24337	RPL15	cna	hetloss	BASIS
PD3905	RPL15	cna	gain	BASIS
PD4006	RPL15	cna	hetloss	BASIS
PD4826	RPL15	cna	gain	BASIS
PD5945	RPL15	cna	amp	BASIS
PD5948	RPL15	cna	gain	BASIS
PD6406	RPL15	cna	hetloss	BASIS
PD6731	RPL15	cna	hetloss	BASIS
PD8980	RPL15	cna	gain	BASIS
PD9585	RPL15	cna	hetloss	BASIS
PD9702	RPL15	cna	gain	BASIS
TCGA-A2-A25B	SETBP1	cna	hetloss	TCGA
TCGA-AN-A0XU	SETBP1	cna	hetloss	TCGA
TCGA-AO-A0JL	SETBP1	cna	gain	TCGA
TCGA-BH-A0AW	SETBP1	cna	hetloss	TCGA
TCGA-BH-A0C0	SETBP1	cna	gain	TCGA
TCGA-C8-A12L	SETBP1	cna	gain	TCGA
TCGA-D8-A27M	SETBP1	cna	hetloss	TCGA
TCGA-E2-A1L7	SETBP1	cna	gain	TCGA
TCGA-LL-A5YP	SETBP1	cna	hetloss	TCGA
MB-0346	SETBP1	cna	hetloss	METABRIC
MB-6060	SETBP1	cna	gain	METABRIC
PD10014	SETBP1	cna	hetloss	BASIS
PD11327	SETBP1	cna	gain	BASIS
PD11742	SETBP1	cna	hetloss	BASIS
PD13296	SETBP1	cna	hetloss	BASIS
PD13297	SETBP1	cna	gain	BASIS
PD13299	SETBP1	cna	gain	BASIS
PD23574	SETBP1	cna	hetloss	BASIS
PD24186	SETBP1	cna	gain	BASIS
PD24202	SETBP1	cna	hetloss	BASIS
PD24337	SETBP1	cna	gain	BASIS
PD3905	SETBP1	cna	gain	BASIS
PD4005	SETBP1	cna	hetloss	BASIS
PD4006	SETBP1	cna	gain	BASIS
PD4107	SETBP1	cna	gain	BASIS
PD4967	SETBP1	cna	hetloss	BASIS
PD5930	SETBP1	cna	gain	BASIS
PD5945	SETBP1	cna	gain	BASIS

PD5948	SETBP1	cna	hetloss	BASIS
PD6406	SETBP1	cna	gain	BASIS
PD7215	SETBP1	cna	gain	BASIS
PD8621	SETBP1	cna	hetloss	BASIS
PD8980	SETBP1	cna	hetloss	BASIS
PD9004	SETBP1	cna	gain	BASIS
PD9702	SETBP1	cna	gain	BASIS
TCGA-A2-A25B	SH2D1A	cna	hetloss	TCGA
TCGA-AN-A0XU	SH2D1A	cna	hetloss	TCGA
TCGA-BH-A0AW	SH2D1A	cna	gain	TCGA
TCGA-C8-A12L	SH2D1A	cna	gain	TCGA
TCGA-E2-A1L7	SH2D1A	cna	hetloss	TCGA
TCGA-E9-A1NC	SH2D1A	cna	hetloss	TCGA
MB-0346	SH2D1A	cna	hetloss	METABRIC
MB-2827	SH2D1A	cna	hetloss	METABRIC
MB-5465	SH2D1A	cna	hetloss	METABRIC
MB-6098	SH2D1A	cna	hetloss	METABRIC
MB-7038	SH2D1A	cna	gain	METABRIC
PD10014	SH2D1A	cna	hetloss	BASIS
PD13296	SH2D1A	cna	hetloss	BASIS
PD13299	SH2D1A	cna	amp	BASIS
PD13771	SH2D1A	cna	gain	BASIS
PD14442	SH2D1A	cna	gain	BASIS
PD23562	SH2D1A	cna	hetloss	BASIS
PD23574	SH2D1A	cna	gain	BASIS
PD23578	SH2D1A	cna	hetloss	BASIS
PD24202	SH2D1A	cna	hetloss	BASIS
PD24206	SH2D1A	cna	gain	BASIS
PD3905	SH2D1A	cna	gain	BASIS
PD4107	SH2D1A	cna	gain	BASIS
PD4826	SH2D1A	cna	gain	BASIS
PD4967	SH2D1A	cna	gain	BASIS
PD5930	SH2D1A	cna	gain	BASIS
PD5935	SH2D1A	cna	gain	BASIS
PD5945	SH2D1A	cna	gain	BASIS
PD5948	SH2D1A	cna	gain	BASIS
PD6406	SH2D1A	cna	gain	BASIS
PD6413	SH2D1A	cna	gain	BASIS
PD7215	SH2D1A	cna	gain	BASIS
PD8621	SH2D1A	cna	amp	BASIS
PD8980	SH2D1A	cna	hetloss	BASIS
PD9004	SH2D1A	cna	gain	BASIS
TCGA-AO-A0JL	SNED1	cna	hetloss	TCGA
TCGA-BH-A0AW	SNED1	cna	hetloss	TCGA
TCGA-C8-A12L	SNED1	cna	hetloss	TCGA
TCGA-D8-A27M	SNED1	cna	gain	TCGA
TCGA-E2-A1L7	SNED1	cna	hetloss	TCGA

TCGA-E9-A1NC	SNED1	cna	hetloss	TCGA
MB-5107	SNED1	cna	hetloss	METABRIC
MB-6060	SNED1	cna	hetloss	METABRIC
MB-6098	SNED1	cna	hetloss	METABRIC
MB-6271	SNED1	cna	hetloss	METABRIC
MB-7048	SNED1	cna	hetloss	METABRIC
PD10014	SNED1	cna	gain	BASIS
PD11327	SNED1	cna	gain	BASIS
PD11742	SNED1	cna	hetloss	BASIS
PD13296	SNED1	cna	hetloss	BASIS
PD13297	SNED1	cna	hetloss	BASIS
PD23578	SNED1	cna	hetloss	BASIS
PD24186	SNED1	cna	gain	BASIS
PD24202	SNED1	cna	hetloss	BASIS
PD24206	SNED1	cna	gain	BASIS
PD3890	SNED1	cna	hetloss	BASIS
PD3905	SNED1	cna	gain	BASIS
PD4005	SNED1	cna	hetloss	BASIS
PD4006	SNED1	cna	gain	BASIS
PD4967	SNED1	cna	hetloss	BASIS
PD5935	SNED1	cna	gain	BASIS
PD5945	SNED1	cna	gain	BASIS
PD5948	SNED1	cna	gain	BASIS
PD6406	SNED1	cna	hetloss	BASIS
PD6413	SNED1	cna	hetloss	BASIS
PD6731	SNED1	cna	hetloss	BASIS
PD7067	SNED1	cna	gain	BASIS
PD7215	SNED1	cna	gain	BASIS
PD8980	SNED1	cna	hetloss	BASIS
PD9585	SNED1	cna	hetloss	BASIS
TCGA-A2-A25B	SOX17	cna	gain	TCGA
TCGA-AO-A0JL	SOX17	cna	amp	TCGA
TCGA-BH-A0AW	SOX17	cna	gain	TCGA
TCGA-BH-A0C0	SOX17	cna	gain	TCGA
TCGA-BH-A18R	SOX17	cna	homdel	TCGA
TCGA-BH-A1FU	SOX17	cna	gain	TCGA
TCGA-D8-A27M	SOX17	cna	amp	TCGA
TCGA-E9-A1NC	SOX17	cna	amp	TCGA
MB-0346	SOX17	cna	gain	METABRIC
MB-5465	SOX17	cna	hetloss	METABRIC
MB-6060	SOX17	cna	amp	METABRIC
MB-6098	SOX17	cna	gain	METABRIC
PD10014	SOX17	cna	amp	BASIS
PD11327	SOX17	cna	gain	BASIS
PD13296	SOX17	cna	homdel	BASIS
PD13299	SOX17	cna	gain	BASIS
PD23562	SOX17	cna	gain	BASIS

PD23574	SOX17	cna	gain	BASIS
PD23578	SOX17	cna	hetloss	BASIS
PD24186	SOX17	cna	gain	BASIS
PD24202	SOX17	cna	hetloss	BASIS
PD24206	SOX17	cna	gain	BASIS
PD3905	SOX17	cna	gain	BASIS
PD4005	SOX17	cna	hetloss	BASIS
PD4006	SOX17	cna	gain	BASIS
PD4826	SOX17	cna	amp	BASIS
PD5935	SOX17	cna	gain	BASIS
PD5945	SOX17	cna	gain	BASIS
PD7067	SOX17	cna	amp	BASIS
PD7215	SOX17	cna	amp	BASIS
PD8621	SOX17	cna	gain	BASIS
PD8980	SOX17	cna	gain	BASIS
PD9004	SOX17	cna	gain	BASIS
PD9585	SOX17	cna	hetloss	BASIS
PD9702	SOX17	cna	gain	BASIS
TCGA-A2-A25B	SPEN	cna	hetloss	TCGA
TCGA-AN-A0XU	SPEN	cna	hetloss	TCGA
TCGA-AO-A0JL	SPEN	cna	hetloss	TCGA
TCGA-BH-A0AW	SPEN	cna	gain	TCGA
TCGA-BH-A0C0	SPEN	cna	hetloss	TCGA
TCGA-BH-A18R	SPEN	cna	homdel	TCGA
TCGA-BH-A1FU	SPEN	cna	gain	TCGA
TCGA-C8-A12L	SPEN	cna	hetloss	TCGA
TCGA-D8-A27M	SPEN	cna	gain	TCGA
TCGA-E2-A1L7	SPEN	cna	hetloss	TCGA
TCGA-E9-A1NC	SPEN	cna	hetloss	TCGA
TCGA-LL-A5YP	SPEN	cna	hetloss	TCGA
MB-2827	SPEN	cna	hetloss	METABRIC
MB-5070	SPEN	cna	hetloss	METABRIC
MB-5465	SPEN	cna	hetloss	METABRIC
MB-6060	SPEN	cna	hetloss	METABRIC
MB-6098	SPEN	cna	hetloss	METABRIC
MB-0420	SPEN	cna	hetloss	METABRIC
PD10014	SPEN	cna	gain	BASIS
PD11327	SPEN	cna	hetloss	BASIS
PD11742	SPEN	cna	hetloss	BASIS
PD13296	SPEN	cna	hetloss	BASIS
PD13771	SPEN	cna	hetloss	BASIS
PD22355	SPEN	cna	hetloss	BASIS
PD24202	SPEN	cna	hetloss	BASIS
PD24206	SPEN	cna	gain	BASIS
PD24337	SPEN	cna	hetloss	BASIS
PD3890	SPEN	cna	hetloss	BASIS
PD3905	SPEN	cna	gain	BASIS

PD4826	SPEN	cna	gain	BASIS
PD5945	SPEN	cna	gain	BASIS
PD6406	SPEN	cna	hetloss	BASIS
PD7067	SPEN	cna	gain	BASIS
PD7215	SPEN	cna	gain	BASIS
PD9004	SPEN	cna	hetloss	BASIS
TCGA-AN-A0XU	SS18	cna	hetloss	TCGA
TCGA-AO-A0JL	SS18	cna	gain	TCGA
TCGA-BH-A0AW	SS18	cna	hetloss	TCGA
TCGA-BH-A0C0	SS18	cna	gain	TCGA
TCGA-C8-A12L	SS18	cna	gain	TCGA
TCGA-D8-A27M	SS18	cna	hetloss	TCGA
TCGA-E2-A1L7	SS18	cna	gain	TCGA
TCGA-LL-A5YP	SS18	cna	amp	TCGA
MB-0346	SS18	cna	hetloss	METABRIC
MB-2827	SS18	cna	hetloss	METABRIC
MB-5465	SS18	cna	hetloss	METABRIC
PD10014	SS18	cna	gain	BASIS
PD11327	SS18	cna	amp	BASIS
PD13297	SS18	cna	gain	BASIS
PD13299	SS18	cna	gain	BASIS
PD22355	SS18	cna	hetloss	BASIS
PD23574	SS18	cna	gain	BASIS
PD24186	SS18	cna	gain	BASIS
PD24202	SS18	cna	hetloss	BASIS
PD24206	SS18	cna	gain	BASIS
PD24337	SS18	cna	gain	BASIS
PD3905	SS18	cna	gain	BASIS
PD4006	SS18	cna	gain	BASIS
PD4107	SS18	cna	gain	BASIS
PD4967	SS18	cna	hetloss	BASIS
PD5930	SS18	cna	gain	BASIS
PD5935	SS18	cna	hetloss	BASIS
PD5945	SS18	cna	gain	BASIS
PD5948	SS18	cna	amp	BASIS
PD6406	SS18	cna	gain	BASIS
PD7067	SS18	cna	gain	BASIS
PD7215	SS18	cna	gain	BASIS
PD8980	SS18	cna	hetloss	BASIS
PD9004	SS18	cna	gain	BASIS
PD9702	SS18	cna	gain	BASIS
TCGA-A2-A25B	SUZ12	cna	hetloss	TCGA
TCGA-AN-A0XU	SUZ12	cna	gain	TCGA
TCGA-AO-A0JL	SUZ12	cna	gain	TCGA
TCGA-BH-A0AW	SUZ12	cna	homdel	TCGA
TCGA-BH-A0C0	SUZ12	cna	gain	TCGA
TCGA-BH-A18R	SUZ12	cna	homdel	TCGA

TCGA-C8-A12L	SUZ12	cna	gain	TCGA
TCGA-D8-A27M	SUZ12	cna	hetloss	TCGA
TCGA-E2-A1L7	SUZ12	cna	hetloss	TCGA
TCGA-E9-A1NC	SUZ12	cna	hetloss	TCGA
MB-0346	SUZ12	cna	hetloss	METABRIC
MB-2827	SUZ12	cna	homdel	METABRIC
MB-5070	SUZ12	cna	gain	METABRIC
MB-5465	SUZ12	cna	hetloss	METABRIC
MB-6098	SUZ12	cna	hetloss	METABRIC
MB-6271	SUZ12	cna	hetloss	METABRIC
MB-7038	SUZ12	cna	hetloss	METABRIC
MB-0420	SUZ12	cna	hetloss	METABRIC
PD11742	SUZ12	cna	hetloss	BASIS
PD13297	SUZ12	cna	hetloss	BASIS
PD22355	SUZ12	cna	hetloss	BASIS
PD23561	SUZ12	cna	hetloss	BASIS
PD24337	SUZ12	cna	hetloss	BASIS
PD3890	SUZ12	cna	hetloss	BASIS
PD4005	SUZ12	cna	hetloss	BASIS
PD4967	SUZ12	cna	hetloss	BASIS
PD5945	SUZ12	cna	gain	BASIS
PD6406	SUZ12	cna	hetloss	BASIS
PD6413	SUZ12	cna	hetloss	BASIS
PD6731	SUZ12	cna	hetloss	BASIS
PD7067	SUZ12	cna	gain	BASIS
PD7215	SUZ12	cna	gain	BASIS
PD9004	SUZ12	cna	gain	BASIS
PD9585	SUZ12	cna	hetloss	BASIS
PD9702	SUZ12	cna	gain	BASIS
TCGA-A2-A25B	TMPRSS2	cna	hetloss	TCGA
TCGA-BH-A0AW	TMPRSS2	cna	gain	TCGA
TCGA-BH-A0C0	TMPRSS2	cna	hetloss	TCGA
TCGA-BH-A1FU	TMPRSS2	cna	gain	TCGA
TCGA-C8-A12L	TMPRSS2	cna	hetloss	TCGA
TCGA-D8-A27M	TMPRSS2	cna	hetloss	TCGA
TCGA-LL-A5YP	TMPRSS2	cna	gain	TCGA
MB-0346	TMPRSS2	cna	gain	METABRIC
MB-2827	TMPRSS2	cna	amp	METABRIC
MB-6098	TMPRSS2	cna	hetloss	METABRIC
MB-7048	TMPRSS2	cna	hetloss	METABRIC
PD10014	TMPRSS2	cna	hetloss	BASIS
PD11327	TMPRSS2	cna	gain	BASIS
PD13296	TMPRSS2	cna	gain	BASIS
PD13297	TMPRSS2	cna	hetloss	BASIS
PD13771	TMPRSS2	cna	gain	BASIS
PD23562	TMPRSS2	cna	gain	BASIS
PD23574	TMPRSS2	cna	gain	BASIS

PD23578	TMPRSS2	cna	gain	BASIS
PD24186	TMPRSS2	cna	gain	BASIS
PD24206	TMPRSS2	cna	gain	BASIS
PD3890	TMPRSS2	cna	hetloss	BASIS
PD3905	TMPRSS2	cna	gain	BASIS
PD4107	TMPRSS2	cna	gain	BASIS
PD4826	TMPRSS2	cna	gain	BASIS
PD5930	TMPRSS2	cna	gain	BASIS
PD5945	TMPRSS2	cna	gain	BASIS
PD5948	TMPRSS2	cna	gain	BASIS
PD6731	TMPRSS2	cna	gain	BASIS
PD7067	TMPRSS2	cna	gain	BASIS
PD7215	TMPRSS2	cna	gain	BASIS
PD8621	TMPRSS2	cna	gain	BASIS
PD8980	TMPRSS2	cna	homdel	BASIS
PD9004	TMPRSS2	cna	gain	BASIS
PD9702	TMPRSS2	cna	gain	BASIS
TCGA-A2-A25B	TOP1	cna	gain	TCGA
TCGA-AO-A0JL	TOP1	cna	gain	TCGA
TCGA-BH-A0AW	TOP1	cna	gain	TCGA
TCGA-BH-A0C0	TOP1	cna	gain	TCGA
TCGA-C8-A12L	TOP1	cna	gain	TCGA
TCGA-D8-A27M	TOP1	cna	gain	TCGA
TCGA-E2-A1L7	TOP1	cna	gain	TCGA
TCGA-E9-A1NC	TOP1	cna	hetloss	TCGA
TCGA-LL-A5YP	TOP1	cna	hetloss	TCGA
MB-0346	TOP1	cna	gain	METABRIC
MB-5107	TOP1	cna	gain	METABRIC
MB-6060	TOP1	cna	gain	METABRIC
MB-6098	TOP1	cna	hetloss	METABRIC
P-0002858-T01-IM3	TOP1	cna	amp	MSK-IMPACT
PD13296	TOP1	cna	hetloss	BASIS
PD13297	TOP1	cna	hetloss	BASIS
PD13771	TOP1	cna	gain	BASIS
PD22355	TOP1	cna	hetloss	BASIS
PD23578	TOP1	cna	hetloss	BASIS
PD24186	TOP1	cna	gain	BASIS
PD24202	TOP1	cna	hetloss	BASIS
PD24206	TOP1	cna	gain	BASIS
PD3905	TOP1	cna	gain	BASIS
PD4005	TOP1	cna	hetloss	BASIS
PD4826	TOP1	cna	gain	BASIS
PD5935	TOP1	cna	gain	BASIS
PD5945	TOP1	cna	amp	BASIS
PD5948	TOP1	cna	gain	BASIS
PD6731	TOP1	cna	hetloss	BASIS
PD7067	TOP1	cna	gain	BASIS

PD7215	TOP1	cna	gain	BASIS
PD8621	TOP1	cna	hetloss	BASIS
PD8980	TOP1	cna	gain	BASIS
PD9004	TOP1	cna	gain	BASIS
PD9585	TOP1	cna	hetloss	BASIS
TCGA-A2-A25B	UHRF1BP1L	cna	gain	TCGA
TCGA-AO-A0JL	UHRF1BP1L	cna	hetloss	TCGA
TCGA-BH-A0AW	UHRF1BP1L	cna	hetloss	TCGA
TCGA-BH-A0C0	UHRF1BP1L	cna	hetloss	TCGA
TCGA-BH-A18R	UHRF1BP1L	cna	gain	TCGA
TCGA-BH-A1FU	UHRF1BP1L	cna	hetloss	TCGA
TCGA-C8-A12L	UHRF1BP1L	cna	gain	TCGA
TCGA-D8-A27M	UHRF1BP1L	cna	hetloss	TCGA
TCGA-E2-A1L7	UHRF1BP1L	cna	hetloss	TCGA
TCGA-LL-A5YP	UHRF1BP1L	cna	hetloss	TCGA
MB-0346	UHRF1BP1L	cna	hetloss	METABRIC
MB-2827	UHRF1BP1L	cna	hetloss	METABRIC
MB-5070	UHRF1BP1L	cna	hetloss	METABRIC
MB-5107	UHRF1BP1L	cna	gain	METABRIC
MB-5465	UHRF1BP1L	cna	gain	METABRIC
MB-6098	UHRF1BP1L	cna	hetloss	METABRIC
PD10014	UHRF1BP1L	cna	gain	BASIS
PD11742	UHRF1BP1L	cna	hetloss	BASIS
PD13296	UHRF1BP1L	cna	hetloss	BASIS
PD13297	UHRF1BP1L	cna	gain	BASIS
PD22355	UHRF1BP1L	cna	hetloss	BASIS
PD3890	UHRF1BP1L	cna	hetloss	BASIS
PD4005	UHRF1BP1L	cna	hetloss	BASIS
PD4006	UHRF1BP1L	cna	gain	BASIS
PD4107	UHRF1BP1L	cna	gain	BASIS
PD4826	UHRF1BP1L	cna	gain	BASIS
PD5945	UHRF1BP1L	cna	gain	BASIS
PD6406	UHRF1BP1L	cna	hetloss	BASIS
PD6413	UHRF1BP1L	cna	hetloss	BASIS
PD7067	UHRF1BP1L	cna	gain	BASIS
PD7215	UHRF1BP1L	cna	hetloss	BASIS
PD8621	UHRF1BP1L	cna	hetloss	BASIS
PD9004	UHRF1BP1L	cna	hetloss	BASIS
PD9585	UHRF1BP1L	cna	hetloss	BASIS
PD9702	UHRF1BP1L	cna	gain	BASIS
TCGA-A2-A25B	USP6	cna	hetloss	TCGA
TCGA-AN-A0XU	USP6	cna	hetloss	TCGA
TCGA-AO-A0JL	USP6	cna	gain	TCGA
TCGA-BH-A0AW	USP6	cna	hetloss	TCGA
TCGA-BH-A0C0	USP6	cna	hetloss	TCGA
TCGA-C8-A12L	USP6	cna	hetloss	TCGA
TCGA-D8-A27M	USP6	cna	hetloss	TCGA

TCGA-E2-A1L7	USP6	cna	hetloss	TCGA
TCGA-E9-A1NC	USP6	cna	hetloss	TCGA
TCGA-LL-A5YP	USP6	cna	gain	TCGA
MB-0346	USP6	cna	hetloss	METABRIC
MB-2827	USP6	cna	hetloss	METABRIC
MB-5107	USP6	cna	hetloss	METABRIC
MB-6271	USP6	cna	hetloss	METABRIC
MB-7032	USP6	cna	gain	METABRIC
MB-7048	USP6	cna	hetloss	METABRIC
MB-0420	USP6	cna	hetloss	METABRIC
PD10014	USP6	cna	hetloss	BASIS
PD11742	USP6	cna	hetloss	BASIS
PD13296	USP6	cna	hetloss	BASIS
PD13771	USP6	cna	hetloss	BASIS
PD24202	USP6	cna	hetloss	BASIS
PD24206	USP6	cna	hetloss	BASIS
PD24337	USP6	cna	hetloss	BASIS
PD3890	USP6	cna	hetloss	BASIS
PD3905	USP6	cna	gain	BASIS
PD4005	USP6	cna	hetloss	BASIS
PD4826	USP6	cna	hetloss	BASIS
PD4967	USP6	cna	hetloss	BASIS
PD5945	USP6	cna	gain	BASIS
PD6406	USP6	cna	hetloss	BASIS
PD6413	USP6	cna	hetloss	BASIS
PD6731	USP6	cna	hetloss	BASIS
PD7067	USP6	cna	gain	BASIS
PD9585	USP6	cna	hetloss	BASIS
TCGA-AN-A0XU	VHL	cna	gain	TCGA
TCGA-BH-A0C0	VHL	cna	gain	TCGA
TCGA-BH-A18R	VHL	cna	gain	TCGA
TCGA-BH-A1FU	VHL	cna	hetloss	TCGA
TCGA-D8-A27M	VHL	cna	hetloss	TCGA
TCGA-E9-A1NC	VHL	cna	hetloss	TCGA
TCGA-LL-A5YP	VHL	cna	amp	TCGA
MB-7038	VHL	cna	hetloss	METABRIC
MB-7048	VHL	cna	hetloss	METABRIC
MB-0420	VHL	cna	hetloss	METABRIC
PD10014	VHL	cna	hetloss	BASIS
PD11327	VHL	cna	gain	BASIS
PD11742	VHL	cna	gain	BASIS
PD13296	VHL	cna	gain	BASIS
PD13299	VHL	cna	gain	BASIS
PD13771	VHL	cna	gain	BASIS
PD23561	VHL	cna	hetloss	BASIS
PD23562	VHL	cna	gain	BASIS
PD23574	VHL	cna	gain	BASIS

PD23578	VHL	cna	gain	BASIS
PD24186	VHL	cna	amp	BASIS
PD24206	VHL	cna	gain	BASIS
PD3905	VHL	cna	gain	BASIS
PD4006	VHL	cna	gain	BASIS
PD4826	VHL	cna	gain	BASIS
PD5945	VHL	cna	amp	BASIS
PD5948	VHL	cna	gain	BASIS
PD6406	VHL	cna	hetloss	BASIS
PD6731	VHL	cna	hetloss	BASIS
PD7067	VHL	cna	gain	BASIS
PD7215	VHL	cna	gain	BASIS
PD8980	VHL	cna	gain	BASIS
PD9004	VHL	cna	gain	BASIS
PD9585	VHL	cna	hetloss	BASIS
PD9702	VHL	cna	gain	BASIS
TCGA-AN-A0XU	WHSC1	cna	hetloss	TCGA
TCGA-BH-A0AW	WHSC1	cna	hetloss	TCGA
TCGA-BH-A0C0	WHSC1	cna	hetloss	TCGA
TCGA-BH-A1FU	WHSC1	cna	hetloss	TCGA
TCGA-C8-A12L	WHSC1	cna	gain	TCGA
TCGA-D8-A27M	WHSC1	cna	amp	TCGA
TCGA-E9-A1NC	WHSC1	cna	hetloss	TCGA
TCGA-LL-A5YP	WHSC1	cna	hetloss	TCGA
MB-0346	WHSC1	cna	hetloss	METABRIC
MB-2827	WHSC1	cna	hetloss	METABRIC
MB-5070	WHSC1	cna	gain	METABRIC
MB-5465	WHSC1	cna	hetloss	METABRIC
MB-6060	WHSC1	cna	hetloss	METABRIC
MB-6098	WHSC1	cna	hetloss	METABRIC
MB-7038	WHSC1	cna	gain	METABRIC
MTS-T0064	WHSC1	cna	homdel	METABRIC
PD10014	WHSC1	cna	hetloss	BASIS
PD11327	WHSC1	cna	gain	BASIS
PD13296	WHSC1	cna	hetloss	BASIS
PD13297	WHSC1	cna	hetloss	BASIS
PD24186	WHSC1	cna	gain	BASIS
PD24202	WHSC1	cna	hetloss	BASIS
PD24206	WHSC1	cna	gain	BASIS
PD3890	WHSC1	cna	hetloss	BASIS
PD3905	WHSC1	cna	gain	BASIS
PD4005	WHSC1	cna	hetloss	BASIS
PD4006	WHSC1	cna	gain	BASIS
PD4826	WHSC1	cna	hetloss	BASIS
PD5945	WHSC1	cna	gain	BASIS
PD6406	WHSC1	cna	hetloss	BASIS
PD6413	WHSC1	cna	hetloss	BASIS

PD7067	WHSC1	cna	gain	BASIS
PD8621	WHSC1	cna	gain	BASIS
PD8980	WHSC1	cna	hetloss	BASIS
PD9585	WHSC1	cna	hetloss	BASIS
TCGA-AN-A0XU	WWOX	cna	gain	TCGA
TCGA-AO-A0JL	WWOX	cna	gain	TCGA
TCGA-BH-A0AW	WWOX	cna	hetloss	TCGA
TCGA-BH-A0C0	WWOX	cna	hetloss	TCGA
TCGA-C8-A12L	WWOX	cna	hetloss	TCGA
TCGA-D8-A27M	WWOX	cna	gain	TCGA
TCGA-E2-A1L7	WWOX	cna	hetloss	TCGA
TCGA-E9-A1NC	WWOX	cna	hetloss	TCGA
TCGA-LL-A5YP	WWOX	cna	gain	TCGA
MB-0346	WWOX	cna	gain	METABRIC
MB-5070	WWOX	cna	amp	METABRIC
MB-5107	WWOX	cna	hetloss	METABRIC
MB-6271	WWOX	cna	hetloss	METABRIC
MB-7032	WWOX	cna	gain	METABRIC
MB-7038	WWOX	cna	gain	METABRIC
MB-0420	WWOX	cna	hetloss	METABRIC
PD10014	WWOX	cna	hetloss	BASIS
PD11327	WWOX	cna	gain	BASIS
PD11742	WWOX	cna	hetloss	BASIS
PD13297	WWOX	cna	hetloss	BASIS
PD13299	WWOX	cna	gain	BASIS
PD14442	WWOX	cna	hetloss	BASIS
PD23562	WWOX	cna	gain	BASIS
PD24186	WWOX	cna	gain	BASIS
PD24202	WWOX	cna	hetloss	BASIS
PD24206	WWOX	cna	hetloss	BASIS
PD3890	WWOX	cna	gain	BASIS
PD4005	WWOX	cna	hetloss	BASIS
PD4006	WWOX	cna	hetloss	BASIS
PD4107	WWOX	cna	gain	BASIS
PD4967	WWOX	cna	hetloss	BASIS
PD5945	WWOX	cna	amp	BASIS
PD7215	WWOX	cna	gain	BASIS
PD9585	WWOX	cna	hetloss	BASIS
PD9702	WWOX	cna	gain	BASIS
TCGA-AN-A0XU	ZFHX3	cna	gain	TCGA
TCGA-AO-A0JL	ZFHX3	cna	gain	TCGA
TCGA-BH-A0C0	ZFHX3	cna	hetloss	TCGA
TCGA-C8-A12L	ZFHX3	cna	hetloss	TCGA
TCGA-D8-A27M	ZFHX3	cna	gain	TCGA
TCGA-E2-A1L7	ZFHX3	cna	hetloss	TCGA
TCGA-E9-A1NC	ZFHX3	cna	hetloss	TCGA
TCGA-LL-A5YP	ZFHX3	cna	gain	TCGA

MB-0346	ZFHX3	cna	gain	METABRIC
MB-5107	ZFHX3	cna	hetloss	METABRIC
MB-6271	ZFHX3	cna	hetloss	METABRIC
MB-7032	ZFHX3	cna	gain	METABRIC
MB-7038	ZFHX3	cna	gain	METABRIC
MB-7048	ZFHX3	cna	hetloss	METABRIC
MB-0420	ZFHX3	cna	hetloss	METABRIC
MTS-T0064	ZFHX3	cna	hetloss	METABRIC
PD10014	ZFHX3	cna	hetloss	BASIS
PD11742	ZFHX3	cna	hetloss	BASIS
PD13297	ZFHX3	cna	hetloss	BASIS
PD13299	ZFHX3	cna	gain	BASIS
PD14442	ZFHX3	cna	hetloss	BASIS
PD23562	ZFHX3	cna	gain	BASIS
PD24186	ZFHX3	cna	gain	BASIS
PD24202	ZFHX3	cna	hetloss	BASIS
PD24206	ZFHX3	cna	gain	BASIS
PD3890	ZFHX3	cna	gain	BASIS
PD3905	ZFHX3	cna	gain	BASIS
PD4005	ZFHX3	cna	hetloss	BASIS
PD4967	ZFHX3	cna	homdel	BASIS
PD5945	ZFHX3	cna	gain	BASIS
PD5948	ZFHX3	cna	hetloss	BASIS
PD6413	ZFHX3	cna	hetloss	BASIS
PD7215	ZFHX3	cna	gain	BASIS
PD9585	ZFHX3	cna	hetloss	BASIS
PD9702	ZFHX3	cna	gain	BASIS
TCGA-A2-A25B	ZRSR2	cna	gain	TCGA
TCGA-AN-A0XU	ZRSR2	cna	hetloss	TCGA
TCGA-AO-A0JL	ZRSR2	cna	gain	TCGA
TCGA-BH-A0AW	ZRSR2	cna	gain	TCGA
TCGA-E2-A1L7	ZRSR2	cna	hetloss	TCGA
TCGA-E9-A1NC	ZRSR2	cna	hetloss	TCGA
TCGA-LL-A5YP	ZRSR2	cna	hetloss	TCGA
MB-0346	ZRSR2	cna	gain	METABRIC
MB-2827	ZRSR2	cna	hetloss	METABRIC
MB-5465	ZRSR2	cna	amp	METABRIC
MB-6098	ZRSR2	cna	hetloss	METABRIC
MB-0420	ZRSR2	cna	hetloss	METABRIC
PD10014	ZRSR2	cna	hetloss	BASIS
PD11327	ZRSR2	cna	hetloss	BASIS
PD11742	ZRSR2	cna	gain	BASIS
PD13296	ZRSR2	cna	amp	BASIS
PD13297	ZRSR2	cna	gain	BASIS
PD13299	ZRSR2	cna	gain	BASIS
PD13771	ZRSR2	cna	gain	BASIS
PD14442	ZRSR2	cna	gain	BASIS

PD23562	ZRSR2	cna	hetloss	BASIS
PD23574	ZRSR2	cna	gain	BASIS
PD3905	ZRSR2	cna	gain	BASIS
PD4005	ZRSR2	cna	hetloss	BASIS
PD4006	ZRSR2	cna	hetloss	BASIS
PD4107	ZRSR2	cna	hetloss	BASIS
PD4826	ZRSR2	cna	gain	BASIS
PD4967	ZRSR2	cna	gain	BASIS
PD5935	ZRSR2	cna	gain	BASIS
PD6406	ZRSR2	cna	gain	BASIS
PD6413	ZRSR2	cna	gain	BASIS
PD7215	ZRSR2	cna	amp	BASIS
PD8621	ZRSR2	cna	gain	BASIS
PD9004	ZRSR2	cna	gain	BASIS
PD9702	ZRSR2	cna	hetloss	BASIS
TCGA-A2-A25B	SEPT6	cna	hetloss	TCGA
TCGA-AN-A0XU	SEPT6	cna	hetloss	TCGA
TCGA-BH-A0AW	SEPT6	cna	gain	TCGA
TCGA-C8-A12L	SEPT6	cna	gain	TCGA
TCGA-E2-A1L7	SEPT6	cna	hetloss	TCGA
TCGA-E9-A1NC	SEPT6	cna	hetloss	TCGA
PD10014	SEPT6	cna	hetloss	BASIS
PD11327	SEPT6	cna	homdel	BASIS
PD13296	SEPT6	cna	hetloss	BASIS
PD13299	SEPT6	cna	amp	BASIS
PD13771	SEPT6	cna	gain	BASIS
PD14442	SEPT6	cna	gain	BASIS
PD23562	SEPT6	cna	hetloss	BASIS
PD23574	SEPT6	cna	gain	BASIS
PD23578	SEPT6	cna	hetloss	BASIS
PD24202	SEPT6	cna	hetloss	BASIS
PD24206	SEPT6	cna	homdel	BASIS
PD3905	SEPT6	cna	gain	BASIS
PD4005	SEPT6	cna	gain	BASIS
PD4006	SEPT6	cna	gain	BASIS
PD4107	SEPT6	cna	gain	BASIS
PD4826	SEPT6	cna	gain	BASIS
PD4967	SEPT6	cna	gain	BASIS
PD5930	SEPT6	cna	gain	BASIS
PD5935	SEPT6	cna	gain	BASIS
PD5945	SEPT6	cna	gain	BASIS
PD5948	SEPT6	cna	gain	BASIS
PD6406	SEPT6	cna	gain	BASIS
PD6413	SEPT6	cna	gain	BASIS
PD7067	SEPT6	cna	gain	BASIS
PD7215	SEPT6	cna	gain	BASIS
PD8621	SEPT6	cna	amp	BASIS

PD8980	SEPT6	cna	hetloss	BASIS
PD9004	SEPT6	cna	gain	BASIS
TCGA-AO-A0JL	ACVR2A	cna	gain	TCGA
TCGA-D8-A27M	ACVR2A	cna	gain	TCGA
TCGA-E9-A1NC	ACVR2A	cna	gain	TCGA
TCGA-LL-A5YP	ACVR2A	cna	hetloss	TCGA
MB-2827	ACVR2A	cna	hetloss	METABRIC
MB-5107	ACVR2A	cna	hetloss	METABRIC
MB-6098	ACVR2A	cna	hetloss	METABRIC
MB-6271	ACVR2A	cna	hetloss	METABRIC
PD11327	ACVR2A	cna	gain	BASIS
PD11742	ACVR2A	cna	hetloss	BASIS
PD13297	ACVR2A	cna	hetloss	BASIS
PD13299	ACVR2A	cna	gain	BASIS
PD22355	ACVR2A	cna	hetloss	BASIS
PD23574	ACVR2A	cna	gain	BASIS
PD23578	ACVR2A	cna	hetloss	BASIS
PD24186	ACVR2A	cna	gain	BASIS
PD24202	ACVR2A	cna	hetloss	BASIS
PD24206	ACVR2A	cna	gain	BASIS
PD24337	ACVR2A	cna	hetloss	BASIS
PD3890	ACVR2A	cna	hetloss	BASIS
PD3905	ACVR2A	cna	gain	BASIS
PD4005	ACVR2A	cna	hetloss	BASIS
PD4006	ACVR2A	cna	hetloss	BASIS
PD4107	ACVR2A	cna	gain	BASIS
PD4967	ACVR2A	cna	hetloss	BASIS
PD5935	ACVR2A	cna	gain	BASIS
PD5945	ACVR2A	cna	gain	BASIS
PD5948	ACVR2A	cna	gain	BASIS
PD6406	ACVR2A	cna	hetloss	BASIS
PD6413	ACVR2A	cna	hetloss	BASIS
PD7067	ACVR2A	cna	gain	BASIS
PD7215	ACVR2A	cna	gain	BASIS
PD8621	ACVR2A	cna	hetloss	BASIS
PD9702	ACVR2A	cna	gain	BASIS
TCGA-AN-A0XU	BCL2L2	cna	hetloss	TCGA
TCGA-AO-A0JL	BCL2L2	cna	hetloss	TCGA
TCGA-BH-A0AW	BCL2L2	cna	hetloss	TCGA
TCGA-BH-A1FU	BCL2L2	cna	gain	TCGA
TCGA-D8-A27M	BCL2L2	cna	hetloss	TCGA
TCGA-LL-A5YP	BCL2L2	cna	hetloss	TCGA
MB-2827	BCL2L2	cna	amp	METABRIC
MB-5070	BCL2L2	cna	hetloss	METABRIC
MB-6060	BCL2L2	cna	gain	METABRIC
PD10014	BCL2L2	cna	gain	BASIS
PD11327	BCL2L2	cna	homdel	BASIS

PD13299	BCL2L2	cna	gain	BASIS
PD22355	BCL2L2	cna	hetloss	BASIS
PD23574	BCL2L2	cna	gain	BASIS
PD23578	BCL2L2	cna	hetloss	BASIS
PD24186	BCL2L2	cna	gain	BASIS
PD24202	BCL2L2	cna	hetloss	BASIS
PD24206	BCL2L2	cna	gain	BASIS
PD3890	BCL2L2	cna	hetloss	BASIS
PD3905	BCL2L2	cna	gain	BASIS
PD4005	BCL2L2	cna	hetloss	BASIS
PD4826	BCL2L2	cna	gain	BASIS
PD4967	BCL2L2	cna	hetloss	BASIS
PD5930	BCL2L2	cna	hetloss	BASIS
PD5935	BCL2L2	cna	gain	BASIS
PD5945	BCL2L2	cna	amp	BASIS
PD5948	BCL2L2	cna	gain	BASIS
PD6406	BCL2L2	cna	hetloss	BASIS
PD6731	BCL2L2	cna	hetloss	BASIS
PD7215	BCL2L2	cna	gain	BASIS
PD8621	BCL2L2	cna	gain	BASIS
PD8980	BCL2L2	cna	amp	BASIS
PD9004	BCL2L2	cna	gain	BASIS
PD9702	BCL2L2	cna	gain	BASIS
TCGA-BH-A0C0	CBLB	cna	hetloss	TCGA
TCGA-BH-A18R	CBLB	cna	gain	TCGA
TCGA-BH-A1FU	CBLB	cna	hetloss	TCGA
TCGA-C8-A12L	CBLB	cna	hetloss	TCGA
TCGA-E2-A1L7	CBLB	cna	hetloss	TCGA
TCGA-LL-A5YP	CBLB	cna	gain	TCGA
MB-0346	CBLB	cna	hetloss	METABRIC
MB-5107	CBLB	cna	gain	METABRIC
MB-6060	CBLB	cna	gain	METABRIC
MTS-T0064	CBLB	cna	gain	METABRIC
PD11327	CBLB	cna	gain	BASIS
PD13296	CBLB	cna	hetloss	BASIS
PD13299	CBLB	cna	gain	BASIS
PD23561	CBLB	cna	hetloss	BASIS
PD23574	CBLB	cna	gain	BASIS
PD24186	CBLB	cna	gain	BASIS
PD24202	CBLB	cna	gain	BASIS
PD24206	CBLB	cna	gain	BASIS
PD24337	CBLB	cna	gain	BASIS
PD3905	CBLB	cna	amp	BASIS
PD4005	CBLB	cna	hetloss	BASIS
PD4006	CBLB	cna	hetloss	BASIS
PD4107	CBLB	cna	gain	BASIS
PD4826	CBLB	cna	gain	BASIS

PD5930	CBLB	cna	hetloss	BASIS
PD5935	CBLB	cna	gain	BASIS
PD5945	CBLB	cna	amp	BASIS
PD5948	CBLB	cna	gain	BASIS
PD7067	CBLB	cna	gain	BASIS
PD7215	CBLB	cna	gain	BASIS
PD8621	CBLB	cna	gain	BASIS
PD8980	CBLB	cna	gain	BASIS
PD9004	CBLB	cna	gain	BASIS
PD9702	CBLB	cna	gain	BASIS
TCGA-AN-A0XU	CD276	cna	amp	TCGA
TCGA-AO-A0JL	CD276	cna	hetloss	TCGA
TCGA-BH-A0AW	CD276	cna	hetloss	TCGA
TCGA-BH-A0C0	CD276	cna	hetloss	TCGA
TCGA-D8-A27M	CD276	cna	hetloss	TCGA
TCGA-E2-A1L7	CD276	cna	gain	TCGA
TCGA-LL-A5YP	CD276	cna	hetloss	TCGA
MB-0346	CD276	cna	hetloss	METABRIC
MB-5070	CD276	cna	hetloss	METABRIC
MB-6060	CD276	cna	hetloss	METABRIC
MB-7038	CD276	cna	gain	METABRIC
PD11327	CD276	cna	gain	BASIS
PD11742	CD276	cna	hetloss	BASIS
PD13296	CD276	cna	homdel	BASIS
PD13297	CD276	cna	hetloss	BASIS
PD13299	CD276	cna	gain	BASIS
PD22355	CD276	cna	hetloss	BASIS
PD23562	CD276	cna	gain	BASIS
PD23574	CD276	cna	gain	BASIS
PD23578	CD276	cna	gain	BASIS
PD24202	CD276	cna	hetloss	BASIS
PD24206	CD276	cna	gain	BASIS
PD3890	CD276	cna	hetloss	BASIS
PD3905	CD276	cna	gain	BASIS
PD4006	CD276	cna	gain	BASIS
PD5945	CD276	cna	gain	BASIS
PD5948	CD276	cna	gain	BASIS
PD6406	CD276	cna	hetloss	BASIS
PD7067	CD276	cna	gain	BASIS
PD7215	CD276	cna	gain	BASIS
PD8621	CD276	cna	gain	BASIS
PD8980	CD276	cna	gain	BASIS
PD9585	CD276	cna	hetloss	BASIS
PD9702	CD276	cna	gain	BASIS
TCGA-AN-A0XU	CD70	cna	hetloss	TCGA
TCGA-AO-A0JL	CD70	cna	gain	TCGA
TCGA-BH-A0C0	CD70	cna	gain	TCGA

TCGA-BH-A1FU	CD70	cna	hetloss	TCGA
TCGA-C8-A12L	CD70	cna	hetloss	TCGA
TCGA-D8-A27M	CD70	cna	hetloss	TCGA
TCGA-EW-A1OX	CD70	cna	hetloss	TCGA
TCGA-LL-A5YP	CD70	cna	hetloss	TCGA
MB-0346	CD70	cna	gain	METABRIC
MB-2827	CD70	cna	hetloss	METABRIC
MB-5070	CD70	cna	hetloss	METABRIC
MB-5465	CD70	cna	gain	METABRIC
MB-6098	CD70	cna	hetloss	METABRIC
MB-7038	CD70	cna	hetloss	METABRIC
MB-0420	CD70	cna	hetloss	METABRIC
PD10014	CD70	cna	hetloss	BASIS
PD11327	CD70	cna	homdel	BASIS
PD14442	CD70	cna	hetloss	BASIS
PD23574	CD70	cna	gain	BASIS
PD24202	CD70	cna	gain	BASIS
PD24206	CD70	cna	hetloss	BASIS
PD3905	CD70	cna	gain	BASIS
PD4006	CD70	cna	gain	BASIS
PD4107	CD70	cna	gain	BASIS
PD4826	CD70	cna	gain	BASIS
PD4967	CD70	cna	homdel	BASIS
PD5930	CD70	cna	gain	BASIS
PD5945	CD70	cna	gain	BASIS
PD5948	CD70	cna	gain	BASIS
PD7067	CD70	cna	gain	BASIS
PD7215	CD70	cna	amp	BASIS
PD8621	CD70	cna	gain	BASIS
PD8980	CD70	cna	gain	BASIS
PD9702	CD70	cna	gain	BASIS
TCGA-AO-A0JL	CRTC1	cna	gain	TCGA
TCGA-BH-A0AW	CRTC1	cna	gain	TCGA
TCGA-BH-A0C0	CRTC1	cna	gain	TCGA
TCGA-BH-A1FU	CRTC1	cna	hetloss	TCGA
TCGA-C8-A12L	CRTC1	cna	gain	TCGA
TCGA-D8-A27M	CRTC1	cna	hetloss	TCGA
TCGA-EW-A1OX	CRTC1	cna	hetloss	TCGA
TCGA-LL-A5YP	CRTC1	cna	hetloss	TCGA
MB-0346	CRTC1	cna	hetloss	METABRIC
MB-2827	CRTC1	cna	hetloss	METABRIC
MB-6060	CRTC1	cna	gain	METABRIC
MB-6098	CRTC1	cna	hetloss	METABRIC
MB-0420	CRTC1	cna	hetloss	METABRIC
MTS-T0064	CRTC1	cna	amp	METABRIC
PD13299	CRTC1	cna	gain	BASIS
PD14442	CRTC1	cna	hetloss	BASIS

PD22355	CRTC1	cna	gain	BASIS
PD23562	CRTC1	cna	gain	BASIS
PD23574	CRTC1	cna	gain	BASIS
PD24186	CRTC1	cna	amp	BASIS
PD24206	CRTC1	cna	hetloss	BASIS
PD3890	CRTC1	cna	hetloss	BASIS
PD3905	CRTC1	cna	gain	BASIS
PD4006	CRTC1	cna	amp	BASIS
PD4107	CRTC1	cna	gain	BASIS
PD4826	CRTC1	cna	gain	BASIS
PD4967	CRTC1	cna	hetloss	BASIS
PD5930	CRTC1	cna	gain	BASIS
PD5935	CRTC1	cna	gain	BASIS
PD5945	CRTC1	cna	amp	BASIS
PD5948	CRTC1	cna	gain	BASIS
PD7067	CRTC1	cna	gain	BASIS
PD7215	CRTC1	cna	amp	BASIS
PD9702	CRTC1	cna	gain	BASIS
TCGA-AN-A0XU	CTNNB1	cna	hetloss	TCGA
TCGA-BH-A0C0	CTNNB1	cna	hetloss	TCGA
TCGA-BH-A1FU	CTNNB1	cna	hetloss	TCGA
TCGA-C8-A12L	CTNNB1	cna	hetloss	TCGA
TCGA-D8-A27M	CTNNB1	cna	hetloss	TCGA
TCGA-E9-A1NC	CTNNB1	cna	hetloss	TCGA
TCGA-LL-A5YP	CTNNB1	cna	gain	TCGA
MB-2827	CTNNB1	cna	hetloss	METABRIC
MB-7038	CTNNB1	cna	hetloss	METABRIC
MB-7048	CTNNB1	cna	hetloss	METABRIC
MB-0420	CTNNB1	cna	hetloss	METABRIC
P-0009557-T01-IM5	CTNNB1	cna	homdel	MSK-IMPACT
PD10014	CTNNB1	cna	hetloss	BASIS
PD13296	CTNNB1	cna	hetloss	BASIS
PD13297	CTNNB1	cna	hetloss	BASIS
PD13299	CTNNB1	cna	gain	BASIS
PD13771	CTNNB1	cna	gain	BASIS
PD22355	CTNNB1	cna	hetloss	BASIS
PD23561	CTNNB1	cna	hetloss	BASIS
PD23574	CTNNB1	cna	gain	BASIS
PD23578	CTNNB1	cna	hetloss	BASIS
PD24202	CTNNB1	cna	hetloss	BASIS
PD24206	CTNNB1	cna	gain	BASIS
PD24337	CTNNB1	cna	hetloss	BASIS
PD3890	CTNNB1	cna	hetloss	BASIS
PD3905	CTNNB1	cna	gain	BASIS
PD4005	CTNNB1	cna	gain	BASIS
PD4826	CTNNB1	cna	gain	BASIS
PD5945	CTNNB1	cna	gain	BASIS

PD6406	CTNNB1	cna	hetloss	BASIS
PD6731	CTNNB1	cna	hetloss	BASIS
PD7067	CTNNB1	cna	gain	BASIS
PD8980	CTNNB1	cna	gain	BASIS
PD9585	CTNNB1	cna	hetloss	BASIS
TCGA-AN-A0XU	DOCK2	cna	hetloss	TCGA
TCGA-AO-A0JL	DOCK2	cna	gain	TCGA
TCGA-BH-A0C0	DOCK2	cna	gain	TCGA
TCGA-BH-A1FU	DOCK2	cna	hetloss	TCGA
TCGA-C8-A12L	DOCK2	cna	hetloss	TCGA
TCGA-D8-A27M	DOCK2	cna	hetloss	TCGA
TCGA-E2-A1L7	DOCK2	cna	hetloss	TCGA
TCGA-LL-A5YP	DOCK2	cna	hetloss	TCGA
MB-0346	DOCK2	cna	gain	METABRIC
MB-5070	DOCK2	cna	hetloss	METABRIC
MB-5465	DOCK2	cna	hetloss	METABRIC
MB-6098	DOCK2	cna	hetloss	METABRIC
MB-0420	DOCK2	cna	hetloss	METABRIC
PD10014	DOCK2	cna	hetloss	BASIS
PD11327	DOCK2	cna	gain	BASIS
PD13296	DOCK2	cna	hetloss	BASIS
PD13297	DOCK2	cna	hetloss	BASIS
PD13771	DOCK2	cna	gain	BASIS
PD22355	DOCK2	cna	hetloss	BASIS
PD23578	DOCK2	cna	hetloss	BASIS
PD24186	DOCK2	cna	gain	BASIS
PD24202	DOCK2	cna	hetloss	BASIS
PD24206	DOCK2	cna	gain	BASIS
PD24337	DOCK2	cna	hetloss	BASIS
PD3890	DOCK2	cna	hetloss	BASIS
PD3905	DOCK2	cna	gain	BASIS
PD4005	DOCK2	cna	hetloss	BASIS
PD4006	DOCK2	cna	hetloss	BASIS
PD4107	DOCK2	cna	hetloss	BASIS
PD4826	DOCK2	cna	gain	BASIS
PD5945	DOCK2	cna	gain	BASIS
PD6406	DOCK2	cna	hetloss	BASIS
PD6413	DOCK2	cna	hetloss	BASIS
PD8980	DOCK2	cna	gain	BASIS
TCGA-AO-A0JL	ELL	cna	gain	TCGA
TCGA-BH-A0AW	ELL	cna	gain	TCGA
TCGA-BH-A0C0	ELL	cna	gain	TCGA
TCGA-BH-A1FU	ELL	cna	hetloss	TCGA
TCGA-C8-A12L	ELL	cna	gain	TCGA
TCGA-D8-A27M	ELL	cna	hetloss	TCGA
TCGA-EW-A1OX	ELL	cna	hetloss	TCGA
TCGA-LL-A5YP	ELL	cna	hetloss	TCGA

MB-0346	ELL	cna	hetloss	METABRIC
MB-2827	ELL	cna	hetloss	METABRIC
MB-6060	ELL	cna	gain	METABRIC
MB-6098	ELL	cna	hetloss	METABRIC
MB-0420	ELL	cna	hetloss	METABRIC
MTS-T0064	ELL	cna	amp	METABRIC
PD13299	ELL	cna	gain	BASIS
PD14442	ELL	cna	hetloss	BASIS
PD22355	ELL	cna	gain	BASIS
PD23562	ELL	cna	gain	BASIS
PD23574	ELL	cna	gain	BASIS
PD24186	ELL	cna	gain	BASIS
PD24206	ELL	cna	hetloss	BASIS
PD3890	ELL	cna	hetloss	BASIS
PD3905	ELL	cna	gain	BASIS
PD4006	ELL	cna	amp	BASIS
PD4107	ELL	cna	gain	BASIS
PD4826	ELL	cna	gain	BASIS
PD4967	ELL	cna	hetloss	BASIS
PD5930	ELL	cna	gain	BASIS
PD5935	ELL	cna	gain	BASIS
PD5945	ELL	cna	amp	BASIS
PD5948	ELL	cna	gain	BASIS
PD7067	ELL	cna	gain	BASIS
PD7215	ELL	cna	amp	BASIS
PD9702	ELL	cna	gain	BASIS
TCGA-AN-A0XU	EPHA3	cna	hetloss	TCGA
TCGA-AO-A0JL	EPHA3	cna	gain	TCGA
TCGA-BH-A0C0	EPHA3	cna	gain	TCGA
TCGA-BH-A1FU	EPHA3	cna	hetloss	TCGA
TCGA-C8-A12L	EPHA3	cna	gain	TCGA
TCGA-D8-A27M	EPHA3	cna	hetloss	TCGA
TCGA-E2-A1L7	EPHA3	cna	hetloss	TCGA
TCGA-E9-A1NC	EPHA3	cna	hetloss	TCGA
TCGA-LL-A5YP	EPHA3	cna	gain	TCGA
MB-0346	EPHA3	cna	hetloss	METABRIC
MB-2827	EPHA3	cna	hetloss	METABRIC
PD10014	EPHA3	cna	gain	BASIS
PD13296	EPHA3	cna	gain	BASIS
PD13299	EPHA3	cna	gain	BASIS
PD13771	EPHA3	cna	hetloss	BASIS
PD23561	EPHA3	cna	hetloss	BASIS
PD23574	EPHA3	cna	gain	BASIS
PD24202	EPHA3	cna	hetloss	BASIS
PD24206	EPHA3	cna	gain	BASIS
PD24337	EPHA3	cna	hetloss	BASIS
PD3905	EPHA3	cna	amp	BASIS

PD4006	EPHA3	cna	hetloss	BASIS
PD4107	EPHA3	cna	gain	BASIS
PD4826	EPHA3	cna	gain	BASIS
PD5930	EPHA3	cna	hetloss	BASIS
PD5945	EPHA3	cna	amp	BASIS
PD5948	EPHA3	cna	gain	BASIS
PD6731	EPHA3	cna	hetloss	BASIS
PD7067	EPHA3	cna	gain	BASIS
PD7215	EPHA3	cna	gain	BASIS
PD8621	EPHA3	cna	hetloss	BASIS
PD8980	EPHA3	cna	hetloss	BASIS
PD9004	EPHA3	cna	gain	BASIS
PD9585	EPHA3	cna	hetloss	BASIS
TCGA-A2-A25B	EPHA5	cna	hetloss	TCGA
TCGA-AO-A0JL	EPHA5	cna	gain	TCGA
TCGA-BH-A0C0	EPHA5	cna	gain	TCGA
TCGA-BH-A1FU	EPHA5	cna	gain	TCGA
TCGA-D8-A27M	EPHA5	cna	gain	TCGA
TCGA-E2-A1L7	EPHA5	cna	hetloss	TCGA
TCGA-E9-A1NC	EPHA5	cna	hetloss	TCGA
TCGA-LL-A5YP	EPHA5	cna	gain	TCGA
MB-6060	EPHA5	cna	hetloss	METABRIC
MB-6098	EPHA5	cna	amp	METABRIC
MB-7038	EPHA5	cna	gain	METABRIC
MB-0420	EPHA5	cna	hetloss	METABRIC
PD11327	EPHA5	cna	amp	BASIS
PD11742	EPHA5	cna	hetloss	BASIS
PD13296	EPHA5	cna	hetloss	BASIS
PD13297	EPHA5	cna	hetloss	BASIS
PD22355	EPHA5	cna	hetloss	BASIS
PD23562	EPHA5	cna	gain	BASIS
PD23574	EPHA5	cna	gain	BASIS
PD24202	EPHA5	cna	hetloss	BASIS
PD24206	EPHA5	cna	gain	BASIS
PD3890	EPHA5	cna	gain	BASIS
PD3905	EPHA5	cna	gain	BASIS
PD4006	EPHA5	cna	hetloss	BASIS
PD4107	EPHA5	cna	gain	BASIS
PD4826	EPHA5	cna	gain	BASIS
PD5945	EPHA5	cna	gain	BASIS
PD5948	EPHA5	cna	gain	BASIS
PD6731	EPHA5	cna	hetloss	BASIS
PD7067	EPHA5	cna	gain	BASIS
PD7215	EPHA5	cna	gain	BASIS
PD8621	EPHA5	cna	hetloss	BASIS
PD8980	EPHA5	cna	hetloss	BASIS
PD9702	EPHA5	cna	gain	BASIS

TCGA-A2-A25B	EPS15	cna	gain	TCGA
TCGA-AN-A0XU	EPS15	cna	gain	TCGA
TCGA-AO-A0JL	EPS15	cna	hetloss	TCGA
TCGA-BH-A0AW	EPS15	cna	gain	TCGA
TCGA-BH-A0C0	EPS15	cna	gain	TCGA
TCGA-BH-A1FU	EPS15	cna	gain	TCGA
TCGA-D8-A27M	EPS15	cna	gain	TCGA
TCGA-E2-A1L7	EPS15	cna	gain	TCGA
TCGA-LL-A5YP	EPS15	cna	hetloss	TCGA
MB-0346	EPS15	cna	hetloss	METABRIC
MB-5465	EPS15	cna	gain	METABRIC
MB-6060	EPS15	cna	hetloss	METABRIC
MB-7038	EPS15	cna	gain	METABRIC
MB-0420	EPS15	cna	hetloss	METABRIC
PD11327	EPS15	cna	hetloss	BASIS
PD11742	EPS15	cna	hetloss	BASIS
PD13299	EPS15	cna	gain	BASIS
PD24186	EPS15	cna	amp	BASIS
PD24206	EPS15	cna	gain	BASIS
PD3905	EPS15	cna	gain	BASIS
PD4005	EPS15	cna	hetloss	BASIS
PD4006	EPS15	cna	gain	BASIS
PD4107	EPS15	cna	gain	BASIS
PD4826	EPS15	cna	gain	BASIS
PD5930	EPS15	cna	gain	BASIS
PD5935	EPS15	cna	gain	BASIS
PD5945	EPS15	cna	amp	BASIS
PD5948	EPS15	cna	gain	BASIS
PD6413	EPS15	cna	hetloss	BASIS
PD7067	EPS15	cna	gain	BASIS
PD7215	EPS15	cna	gain	BASIS
PD8621	EPS15	cna	gain	BASIS
PD9004	EPS15	cna	gain	BASIS
PD9702	EPS15	cna	gain	BASIS
TCGA-A2-A25B	ERBB3	cna	gain	TCGA
TCGA-AO-A0JL	ERBB3	cna	hetloss	TCGA
TCGA-BH-A18R	ERBB3	cna	gain	TCGA
TCGA-BH-A1FU	ERBB3	cna	hetloss	TCGA
TCGA-C8-A12L	ERBB3	cna	gain	TCGA
TCGA-D8-A27M	ERBB3	cna	hetloss	TCGA
TCGA-E2-A1L7	ERBB3	cna	hetloss	TCGA
TCGA-E9-A1NC	ERBB3	cna	hetloss	TCGA
TCGA-LL-A5YP	ERBB3	cna	hetloss	TCGA
MB-0346	ERBB3	cna	gain	METABRIC
MB-2827	ERBB3	cna	hetloss	METABRIC
MB-6098	ERBB3	cna	hetloss	METABRIC
MB-7038	ERBB3	cna	hetloss	METABRIC

MB-0420	ERBB3	cna	hetloss	METABRIC
PD11327	ERBB3	cna	homdel	BASIS
PD13296	ERBB3	cna	hetloss	BASIS
PD13297	ERBB3	cna	hetloss	BASIS
PD13771	ERBB3	cna	gain	BASIS
PD24186	ERBB3	cna	gain	BASIS
PD24202	ERBB3	cna	hetloss	BASIS
PD24206	ERBB3	cna	gain	BASIS
PD24337	ERBB3	cna	hetloss	BASIS
PD3890	ERBB3	cna	hetloss	BASIS
PD3905	ERBB3	cna	gain	BASIS
PD4826	ERBB3	cna	gain	BASIS
PD5945	ERBB3	cna	gain	BASIS
PD5948	ERBB3	cna	gain	BASIS
PD6406	ERBB3	cna	hetloss	BASIS
PD6413	ERBB3	cna	hetloss	BASIS
PD6731	ERBB3	cna	hetloss	BASIS
PD7067	ERBB3	cna	gain	BASIS
PD8621	ERBB3	cna	hetloss	BASIS
PD9585	ERBB3	cna	hetloss	BASIS
PD9702	ERBB3	cna	gain	BASIS
TCGA-A2-A25B	FANCC	cna	gain	TCGA
TCGA-AN-A0XU	FANCC	cna	hetloss	TCGA
TCGA-AO-A0JL	FANCC	cna	hetloss	TCGA
TCGA-BH-A1FU	FANCC	cna	hetloss	TCGA
TCGA-C8-A12L	FANCC	cna	hetloss	TCGA
TCGA-E9-A1NC	FANCC	cna	hetloss	TCGA
TCGA-LL-A5YP	FANCC	cna	hetloss	TCGA
MB-0346	FANCC	cna	hetloss	METABRIC
MB-2827	FANCC	cna	amp	METABRIC
MB-5070	FANCC	cna	hetloss	METABRIC
MB-5107	FANCC	cna	hetloss	METABRIC
MB-6098	FANCC	cna	hetloss	METABRIC
MB-7038	FANCC	cna	hetloss	METABRIC
PD11327	FANCC	cna	gain	BASIS
PD13296	FANCC	cna	gain	BASIS
PD13771	FANCC	cna	hetloss	BASIS
PD23562	FANCC	cna	gain	BASIS
PD23578	FANCC	cna	hetloss	BASIS
PD24186	FANCC	cna	amp	BASIS
PD24202	FANCC	cna	hetloss	BASIS
PD24206	FANCC	cna	gain	BASIS
PD3905	FANCC	cna	gain	BASIS
PD4005	FANCC	cna	gain	BASIS
PD4006	FANCC	cna	gain	BASIS
PD4107	FANCC	cna	gain	BASIS
PD5945	FANCC	cna	amp	BASIS

PD5948	FANCC	cna	gain	BASIS
PD6406	FANCC	cna	hetloss	BASIS
PD6731	FANCC	cna	hetloss	BASIS
PD7067	FANCC	cna	gain	BASIS
PD7215	FANCC	cna	gain	BASIS
PD8621	FANCC	cna	gain	BASIS
PD9004	FANCC	cna	gain	BASIS
PD9585	FANCC	cna	hetloss	BASIS
TCGA-A2-A25B	FANCL	cna	hetloss	TCGA
TCGA-AN-A0XU	FANCL	cna	gain	TCGA
TCGA-AO-A0JL	FANCL	cna	gain	TCGA
TCGA-BH-A0AW	FANCL	cna	gain	TCGA
TCGA-BH-A0C0	FANCL	cna	gain	TCGA
TCGA-D8-A27M	FANCL	cna	gain	TCGA
TCGA-LL-A5YP	FANCL	cna	gain	TCGA
MB-5465	FANCL	cna	amp	METABRIC
MB-6271	FANCL	cna	hetloss	METABRIC
MB-7038	FANCL	cna	amp	METABRIC
PD10014	FANCL	cna	gain	BASIS
PD11327	FANCL	cna	gain	BASIS
PD13296	FANCL	cna	gain	BASIS
PD13297	FANCL	cna	gain	BASIS
PD13299	FANCL	cna	gain	BASIS
PD22355	FANCL	cna	gain	BASIS
PD23562	FANCL	cna	gain	BASIS
PD23578	FANCL	cna	gain	BASIS
PD24186	FANCL	cna	gain	BASIS
PD24202	FANCL	cna	gain	BASIS
PD24206	FANCL	cna	gain	BASIS
PD3890	FANCL	cna	hetloss	BASIS
PD3905	FANCL	cna	gain	BASIS
PD4006	FANCL	cna	gain	BASIS
PD5930	FANCL	cna	gain	BASIS
PD5935	FANCL	cna	gain	BASIS
PD5945	FANCL	cna	amp	BASIS
PD5948	FANCL	cna	gain	BASIS
PD7067	FANCL	cna	gain	BASIS
PD7215	FANCL	cna	gain	BASIS
PD8621	FANCL	cna	gain	BASIS
PD8980	FANCL	cna	hetloss	BASIS
PD9004	FANCL	cna	gain	BASIS
PD9702	FANCL	cna	gain	BASIS
TCGA-A2-A25B	GNAQ	cna	gain	TCGA
TCGA-AN-A0XU	GNAQ	cna	hetloss	TCGA
TCGA-AO-A0JL	GNAQ	cna	hetloss	TCGA
TCGA-BH-A0C0	GNAQ	cna	gain	TCGA
TCGA-BH-A1FU	GNAQ	cna	hetloss	TCGA

TCGA-C8-A12L	GNAQ	cna	hetloss	TCGA
TCGA-E9-A1NC	GNAQ	cna	hetloss	TCGA
TCGA-LL-A5YP	GNAQ	cna	hetloss	TCGA
MB-0346	GNAQ	cna	hetloss	METABRIC
MB-5107	GNAQ	cna	hetloss	METABRIC
MB-5465	GNAQ	cna	gain	METABRIC
MB-6098	GNAQ	cna	hetloss	METABRIC
MB-7038	GNAQ	cna	hetloss	METABRIC
MB-0420	GNAQ	cna	hetloss	METABRIC
MTS-T0064	GNAQ	cna	gain	METABRIC
PD11327	GNAQ	cna	gain	BASIS
PD13296	GNAQ	cna	hetloss	BASIS
PD23562	GNAQ	cna	gain	BASIS
PD23578	GNAQ	cna	hetloss	BASIS
PD24186	GNAQ	cna	amp	BASIS
PD24202	GNAQ	cna	hetloss	BASIS
PD24206	GNAQ	cna	gain	BASIS
PD3890	GNAQ	cna	gain	BASIS
PD3905	GNAQ	cna	gain	BASIS
PD4006	GNAQ	cna	gain	BASIS
PD4107	GNAQ	cna	gain	BASIS
PD5945	GNAQ	cna	amp	BASIS
PD5948	GNAQ	cna	hetloss	BASIS
PD6731	GNAQ	cna	hetloss	BASIS
PD7067	GNAQ	cna	gain	BASIS
PD7215	GNAQ	cna	gain	BASIS
PD8621	GNAQ	cna	gain	BASIS
PD9004	GNAQ	cna	gain	BASIS
PD9585	GNAQ	cna	hetloss	BASIS
TCGA-AN-A0XU	GRIN2A	cna	gain	TCGA
TCGA-AO-A0JL	GRIN2A	cna	gain	TCGA
TCGA-BH-A0AW	GRIN2A	cna	gain	TCGA
TCGA-BH-A0C0	GRIN2A	cna	gain	TCGA
TCGA-C8-A12L	GRIN2A	cna	gain	TCGA
TCGA-E2-A1L7	GRIN2A	cna	hetloss	TCGA
TCGA-LL-A5YP	GRIN2A	cna	gain	TCGA
MB-0346	GRIN2A	cna	gain	METABRIC
MB-6060	GRIN2A	cna	gain	METABRIC
MB-6098	GRIN2A	cna	hetloss	METABRIC
MB-6271	GRIN2A	cna	gain	METABRIC
MB-7032	GRIN2A	cna	gain	METABRIC
MB-7038	GRIN2A	cna	hetloss	METABRIC
MB-0420	GRIN2A	cna	hetloss	METABRIC
PD10014	GRIN2A	cna	hetloss	BASIS
PD11742	GRIN2A	cna	gain	BASIS
PD13296	GRIN2A	cna	gain	BASIS
PD13297	GRIN2A	cna	hetloss	BASIS

PD13299	GRIN2A	cna	gain	BASIS
PD22355	GRIN2A	cna	hetloss	BASIS
PD23578	GRIN2A	cna	hetloss	BASIS
PD24186	GRIN2A	cna	gain	BASIS
PD24206	GRIN2A	cna	gain	BASIS
PD24337	GRIN2A	cna	hetloss	BASIS
PD3905	GRIN2A	cna	gain	BASIS
PD4005	GRIN2A	cna	gain	BASIS
PD4006	GRIN2A	cna	hetloss	BASIS
PD4107	GRIN2A	cna	gain	BASIS
PD4826	GRIN2A	cna	gain	BASIS
PD5935	GRIN2A	cna	gain	BASIS
PD5945	GRIN2A	cna	gain	BASIS
PD7215	GRIN2A	cna	amp	BASIS
PD9004	GRIN2A	cna	gain	BASIS
PD9585	GRIN2A	cna	hetloss	BASIS
TCGA-AO-A0JL	HDAC4	cna	hetloss	TCGA
TCGA-BH-A0AW	HDAC4	cna	hetloss	TCGA
TCGA-C8-A12L	HDAC4	cna	hetloss	TCGA
TCGA-D8-A27M	HDAC4	cna	gain	TCGA
TCGA-E2-A1L7	HDAC4	cna	hetloss	TCGA
TCGA-E9-A1NC	HDAC4	cna	hetloss	TCGA
MB-5107	HDAC4	cna	hetloss	METABRIC
MB-6060	HDAC4	cna	hetloss	METABRIC
MB-6098	HDAC4	cna	hetloss	METABRIC
MB-6271	HDAC4	cna	hetloss	METABRIC
MB-7048	HDAC4	cna	hetloss	METABRIC
PD10014	HDAC4	cna	gain	BASIS
PD11327	HDAC4	cna	gain	BASIS
PD11742	HDAC4	cna	hetloss	BASIS
PD13297	HDAC4	cna	hetloss	BASIS
PD23578	HDAC4	cna	hetloss	BASIS
PD24186	HDAC4	cna	gain	BASIS
PD24202	HDAC4	cna	hetloss	BASIS
PD24206	HDAC4	cna	gain	BASIS
PD3890	HDAC4	cna	hetloss	BASIS
PD3905	HDAC4	cna	gain	BASIS
PD4005	HDAC4	cna	hetloss	BASIS
PD4006	HDAC4	cna	gain	BASIS
PD4967	HDAC4	cna	hetloss	BASIS
PD5935	HDAC4	cna	gain	BASIS
PD5945	HDAC4	cna	gain	BASIS
PD5948	HDAC4	cna	gain	BASIS
PD6406	HDAC4	cna	hetloss	BASIS
PD6413	HDAC4	cna	hetloss	BASIS
PD6731	HDAC4	cna	hetloss	BASIS
PD7067	HDAC4	cna	gain	BASIS

PD7215	HDAC4	cna	gain	BASIS
PD8980	HDAC4	cna	hetloss	BASIS
PD9585	HDAC4	cna	hetloss	BASIS
TCGA-A2-A25B	HIF1A	cna	hetloss	TCGA
TCGA-AO-A0JL	HIF1A	cna	hetloss	TCGA
TCGA-BH-A0AW	HIF1A	cna	hetloss	TCGA
TCGA-BH-A1FU	HIF1A	cna	gain	TCGA
TCGA-C8-A12L	HIF1A	cna	hetloss	TCGA
TCGA-D8-A27M	HIF1A	cna	hetloss	TCGA
TCGA-E2-A1L7	HIF1A	cna	hetloss	TCGA
TCGA-LL-A5YP	HIF1A	cna	hetloss	TCGA
MB-2827	HIF1A	cna	hetloss	METABRIC
MB-5070	HIF1A	cna	hetloss	METABRIC
MB-6098	HIF1A	cna	hetloss	METABRIC
MB-7038	HIF1A	cna	hetloss	METABRIC
PD10014	HIF1A	cna	hetloss	BASIS
PD13296	HIF1A	cna	hetloss	BASIS
PD13297	HIF1A	cna	hetloss	BASIS
PD22355	HIF1A	cna	hetloss	BASIS
PD23574	HIF1A	cna	hetloss	BASIS
PD23578	HIF1A	cna	hetloss	BASIS
PD24202	HIF1A	cna	hetloss	BASIS
PD24206	HIF1A	cna	gain	BASIS
PD24337	HIF1A	cna	hetloss	BASIS
PD3890	HIF1A	cna	hetloss	BASIS
PD3905	HIF1A	cna	hetloss	BASIS
PD4005	HIF1A	cna	hetloss	BASIS
PD4006	HIF1A	cna	hetloss	BASIS
PD4107	HIF1A	cna	hetloss	BASIS
PD5930	HIF1A	cna	hetloss	BASIS
PD5945	HIF1A	cna	gain	BASIS
PD6406	HIF1A	cna	hetloss	BASIS
PD7067	HIF1A	cna	gain	BASIS
PD7215	HIF1A	cna	hetloss	BASIS
PD8980	HIF1A	cna	gain	BASIS
PD9585	HIF1A	cna	gain	BASIS
PD9702	HIF1A	cna	gain	BASIS
TCGA-A2-A25B	HOOK3	cna	hetloss	TCGA
TCGA-AO-A0JL	HOOK3	cna	amp	TCGA
TCGA-BH-A0C0	HOOK3	cna	gain	TCGA
TCGA-BH-A1FU	HOOK3	cna	homdel	TCGA
TCGA-D8-A27M	HOOK3	cna	amp	TCGA
TCGA-LL-A5YP	HOOK3	cna	amp	TCGA
MB-0346	HOOK3	cna	hetloss	METABRIC
MB-2827	HOOK3	cna	homdel	METABRIC
MB-6060	HOOK3	cna	hetloss	METABRIC
MB-7038	HOOK3	cna	hetloss	METABRIC

PD11327	HOOK3	cna	homdel	BASIS
PD13296	HOOK3	cna	hetloss	BASIS
PD13299	HOOK3	cna	gain	BASIS
PD13771	HOOK3	cna	amp	BASIS
PD23562	HOOK3	cna	gain	BASIS
PD23574	HOOK3	cna	gain	BASIS
PD23578	HOOK3	cna	hetloss	BASIS
PD24186	HOOK3	cna	gain	BASIS
PD24202	HOOK3	cna	hetloss	BASIS
PD24206	HOOK3	cna	amp	BASIS
PD3905	HOOK3	cna	gain	BASIS
PD4006	HOOK3	cna	gain	BASIS
PD4107	HOOK3	cna	gain	BASIS
PD4826	HOOK3	cna	hetloss	BASIS
PD5930	HOOK3	cna	gain	BASIS
PD5935	HOOK3	cna	gain	BASIS
PD5945	HOOK3	cna	amp	BASIS
PD5948	HOOK3	cna	hetloss	BASIS
PD7067	HOOK3	cna	amp	BASIS
PD7215	HOOK3	cna	amp	BASIS
PD8980	HOOK3	cna	amp	BASIS
PD9004	HOOK3	cna	amp	BASIS
PD9585	HOOK3	cna	gain	BASIS
PD9702	HOOK3	cna	gain	BASIS
TCGA-A2-A25B	IKBKB	cna	hetloss	TCGA
TCGA-AO-A0JL	IKBKB	cna	amp	TCGA
TCGA-BH-A0C0	IKBKB	cna	gain	TCGA
TCGA-BH-A1FU	IKBKB	cna	homdel	TCGA
TCGA-D8-A27M	IKBKB	cna	amp	TCGA
TCGA-E2-A1L7	IKBKB	cna	hetloss	TCGA
TCGA-LL-A5YP	IKBKB	cna	amp	TCGA
MB-0346	IKBKB	cna	hetloss	METABRIC
MB-2827	IKBKB	cna	hetloss	METABRIC
MB-6060	IKBKB	cna	amp	METABRIC
MB-7038	IKBKB	cna	hetloss	METABRIC
PD11327	IKBKB	cna	homdel	BASIS
PD13296	IKBKB	cna	hetloss	BASIS
PD13299	IKBKB	cna	gain	BASIS
PD13771	IKBKB	cna	amp	BASIS
PD23562	IKBKB	cna	gain	BASIS
PD23574	IKBKB	cna	gain	BASIS
PD23578	IKBKB	cna	hetloss	BASIS
PD24186	IKBKB	cna	gain	BASIS
PD24202	IKBKB	cna	hetloss	BASIS
PD24206	IKBKB	cna	amp	BASIS
PD3905	IKBKB	cna	gain	BASIS
PD4006	IKBKB	cna	gain	BASIS

PD4107	IKBKB	cna	gain	BASIS
PD5930	IKBKB	cna	gain	BASIS
PD5935	IKBKB	cna	gain	BASIS
PD5945	IKBKB	cna	amp	BASIS
PD5948	IKBKB	cna	hetloss	BASIS
PD7067	IKBKB	cna	amp	BASIS
PD7215	IKBKB	cna	amp	BASIS
PD8980	IKBKB	cna	amp	BASIS
PD9004	IKBKB	cna	amp	BASIS
PD9585	IKBKB	cna	gain	BASIS
PD9702	IKBKB	cna	gain	BASIS
TCGA-A2-A25B	KDM6A	cna	gain	TCGA
TCGA-AN-A0XU	KDM6A	cna	hetloss	TCGA
TCGA-AO-A0JL	KDM6A	cna	gain	TCGA
TCGA-BH-A0AW	KDM6A	cna	gain	TCGA
TCGA-E2-A1L7	KDM6A	cna	hetloss	TCGA
TCGA-E9-A1NC	KDM6A	cna	hetloss	TCGA
MB-0346	KDM6A	cna	hetloss	METABRIC
MB-2827	KDM6A	cna	hetloss	METABRIC
MB-5070	KDM6A	cna	hetloss	METABRIC
MB-5465	KDM6A	cna	gain	METABRIC
MB-6098	KDM6A	cna	hetloss	METABRIC
MB-0420	KDM6A	cna	hetloss	METABRIC
PD10014	KDM6A	cna	hetloss	BASIS
PD11327	KDM6A	cna	homdel	BASIS
PD13296	KDM6A	cna	gain	BASIS
PD13299	KDM6A	cna	amp	BASIS
PD13771	KDM6A	cna	gain	BASIS
PD14442	KDM6A	cna	gain	BASIS
PD23562	KDM6A	cna	hetloss	BASIS
PD23574	KDM6A	cna	gain	BASIS
PD3905	KDM6A	cna	gain	BASIS
PD4005	KDM6A	cna	hetloss	BASIS
PD4006	KDM6A	cna	hetloss	BASIS
PD4107	KDM6A	cna	gain	BASIS
PD4826	KDM6A	cna	gain	BASIS
PD4967	KDM6A	cna	gain	BASIS
PD5935	KDM6A	cna	gain	BASIS
PD5948	KDM6A	cna	gain	BASIS
PD6406	KDM6A	cna	gain	BASIS
PD6413	KDM6A	cna	gain	BASIS
PD7215	KDM6A	cna	gain	BASIS
PD8621	KDM6A	cna	amp	BASIS
PD8980	KDM6A	cna	hetloss	BASIS
PD9004	KDM6A	cna	gain	BASIS
TCGA-AO-A0JL	KEAP1	cna	gain	TCGA
TCGA-BH-A0C0	KEAP1	cna	gain	TCGA

TCGA-BH-A1FU	KEAP1	cna	hetloss	TCGA
TCGA-C8-A12L	KEAP1	cna	amp	TCGA
TCGA-D8-A27M	KEAP1	cna	hetloss	TCGA
TCGA-EW-A1OX	KEAP1	cna	hetloss	TCGA
TCGA-LL-A5YP	KEAP1	cna	hetloss	TCGA
MB-0346	KEAP1	cna	hetloss	METABRIC
MB-2827	KEAP1	cna	hetloss	METABRIC
MB-5070	KEAP1	cna	hetloss	METABRIC
MB-5465	KEAP1	cna	gain	METABRIC
MB-6098	KEAP1	cna	hetloss	METABRIC
MB-7038	KEAP1	cna	hetloss	METABRIC
MB-0420	KEAP1	cna	hetloss	METABRIC
PD11327	KEAP1	cna	homdel	BASIS
PD13771	KEAP1	cna	gain	BASIS
PD14442	KEAP1	cna	hetloss	BASIS
PD23562	KEAP1	cna	gain	BASIS
PD23574	KEAP1	cna	gain	BASIS
PD24206	KEAP1	cna	homdel	BASIS
PD3905	KEAP1	cna	gain	BASIS
PD4006	KEAP1	cna	gain	BASIS
PD4107	KEAP1	cna	gain	BASIS
PD4826	KEAP1	cna	gain	BASIS
PD4967	KEAP1	cna	hetloss	BASIS
PD5930	KEAP1	cna	gain	BASIS
PD5935	KEAP1	cna	gain	BASIS
PD5945	KEAP1	cna	amp	BASIS
PD5948	KEAP1	cna	gain	BASIS
PD6406	KEAP1	cna	amp	BASIS
PD7067	KEAP1	cna	gain	BASIS
PD7215	KEAP1	cna	amp	BASIS
PD8621	KEAP1	cna	gain	BASIS
PD9702	KEAP1	cna	gain	BASIS
TCGA-A2-A25B	KIF5B	cna	gain	TCGA
TCGA-AN-A0XU	KIF5B	cna	hetloss	TCGA
TCGA-AO-A0JL	KIF5B	cna	gain	TCGA
TCGA-BH-A0AW	KIF5B	cna	gain	TCGA
TCGA-BH-A0C0	KIF5B	cna	gain	TCGA
TCGA-C8-A12L	KIF5B	cna	gain	TCGA
TCGA-LL-A5YP	KIF5B	cna	gain	TCGA
MB-6098	KIF5B	cna	gain	METABRIC
MB-7038	KIF5B	cna	hetloss	METABRIC
MTS-T0064	KIF5B	cna	gain	METABRIC
PD11327	KIF5B	cna	gain	BASIS
PD13296	KIF5B	cna	gain	BASIS
PD13299	KIF5B	cna	gain	BASIS
PD13771	KIF5B	cna	gain	BASIS
PD22355	KIF5B	cna	gain	BASIS

PD23562	KIF5B	cna	gain	BASIS
PD23574	KIF5B	cna	gain	BASIS
PD24186	KIF5B	cna	gain	BASIS
PD24202	KIF5B	cna	hetloss	BASIS
PD24206	KIF5B	cna	gain	BASIS
PD3905	KIF5B	cna	gain	BASIS
PD4005	KIF5B	cna	gain	BASIS
PD4006	KIF5B	cna	gain	BASIS
PD4107	KIF5B	cna	gain	BASIS
PD4826	KIF5B	cna	gain	BASIS
PD5930	KIF5B	cna	gain	BASIS
PD5945	KIF5B	cna	amp	BASIS
PD5948	KIF5B	cna	gain	BASIS
PD7067	KIF5B	cna	amp	BASIS
PD7215	KIF5B	cna	gain	BASIS
PD8621	KIF5B	cna	gain	BASIS
PD8980	KIF5B	cna	hetloss	BASIS
PD9004	KIF5B	cna	gain	BASIS
PD9702	KIF5B	cna	gain	BASIS
TCGA-A2-A25B	LMO1	cna	hetloss	TCGA
TCGA-AN-A0XU	LMO1	cna	hetloss	TCGA
TCGA-AO-A0JL	LMO1	cna	hetloss	TCGA
TCGA-BH-A0C0	LMO1	cna	hetloss	TCGA
TCGA-C8-A12L	LMO1	cna	hetloss	TCGA
TCGA-E2-A1L7	LMO1	cna	hetloss	TCGA
TCGA-E9-A1NC	LMO1	cna	hetloss	TCGA
TCGA-LL-A5YP	LMO1	cna	hetloss	TCGA
MB-0346	LMO1	cna	hetloss	METABRIC
MB-5070	LMO1	cna	hetloss	METABRIC
MB-6060	LMO1	cna	hetloss	METABRIC
MB-6271	LMO1	cna	gain	METABRIC
MB-7048	LMO1	cna	hetloss	METABRIC
PD10014	LMO1	cna	hetloss	BASIS
PD11742	LMO1	cna	hetloss	BASIS
PD13297	LMO1	cna	hetloss	BASIS
PD22355	LMO1	cna	hetloss	BASIS
PD23574	LMO1	cna	gain	BASIS
PD24202	LMO1	cna	hetloss	BASIS
PD24206	LMO1	cna	amp	BASIS
PD3890	LMO1	cna	hetloss	BASIS
PD3905	LMO1	cna	gain	BASIS
PD4005	LMO1	cna	hetloss	BASIS
PD4006	LMO1	cna	gain	BASIS
PD4107	LMO1	cna	gain	BASIS
PD4826	LMO1	cna	gain	BASIS
PD5935	LMO1	cna	gain	BASIS
PD5945	LMO1	cna	gain	BASIS

PD6406	LMO1	cna	hetloss	BASIS
PD6413	LMO1	cna	hetloss	BASIS
PD6731	LMO1	cna	hetloss	BASIS
PD7215	LMO1	cna	hetloss	BASIS
PD8980	LMO1	cna	hetloss	BASIS
PD9585	LMO1	cna	hetloss	BASIS
TCGA-AN-A0XU	LMO2	cna	gain	TCGA
TCGA-AO-A0JL	LMO2	cna	hetloss	TCGA
TCGA-BH-A0C0	LMO2	cna	gain	TCGA
TCGA-C8-A12L	LMO2	cna	hetloss	TCGA
TCGA-E2-A1L7	LMO2	cna	gain	TCGA
TCGA-LL-A5YP	LMO2	cna	amp	TCGA
MB-0346	LMO2	cna	hetloss	METABRIC
MB-6060	LMO2	cna	gain	METABRIC
MB-6098	LMO2	cna	hetloss	METABRIC
MB-6271	LMO2	cna	gain	METABRIC
MB-7048	LMO2	cna	hetloss	METABRIC
PD10014	LMO2	cna	hetloss	BASIS
PD11327	LMO2	cna	homdel	BASIS
PD13296	LMO2	cna	gain	BASIS
PD13299	LMO2	cna	hetloss	BASIS
PD23562	LMO2	cna	gain	BASIS
PD23578	LMO2	cna	hetloss	BASIS
PD24186	LMO2	cna	gain	BASIS
PD24206	LMO2	cna	gain	BASIS
PD3905	LMO2	cna	gain	BASIS
PD4006	LMO2	cna	hetloss	BASIS
PD4107	LMO2	cna	gain	BASIS
PD4826	LMO2	cna	gain	BASIS
PD5930	LMO2	cna	gain	BASIS
PD5935	LMO2	cna	gain	BASIS
PD5945	LMO2	cna	gain	BASIS
PD5948	LMO2	cna	gain	BASIS
PD6413	LMO2	cna	hetloss	BASIS
PD6731	LMO2	cna	hetloss	BASIS
PD7215	LMO2	cna	gain	BASIS
PD8621	LMO2	cna	gain	BASIS
PD8980	LMO2	cna	homdel	BASIS
PD9585	LMO2	cna	hetloss	BASIS
PD9702	LMO2	cna	gain	BASIS
TCGA-AN-A0XU	LRP1B	cna	hetloss	TCGA
TCGA-AO-A0JL	LRP1B	cna	gain	TCGA
TCGA-D8-A27M	LRP1B	cna	gain	TCGA
TCGA-E9-A1NC	LRP1B	cna	gain	TCGA
TCGA-LL-A5YP	LRP1B	cna	hetloss	TCGA
MB-2827	LRP1B	cna	hetloss	METABRIC
MB-5107	LRP1B	cna	hetloss	METABRIC

MB-6098	LRP1B	cna	hetloss	METABRIC
MB-6271	LRP1B	cna	hetloss	METABRIC
PD11327	LRP1B	cna	gain	BASIS
PD11742	LRP1B	cna	hetloss	BASIS
PD13296	LRP1B	cna	hetloss	BASIS
PD13297	LRP1B	cna	hetloss	BASIS
PD13299	LRP1B	cna	gain	BASIS
PD22355	LRP1B	cna	hetloss	BASIS
PD23574	LRP1B	cna	amp	BASIS
PD23578	LRP1B	cna	hetloss	BASIS
PD24186	LRP1B	cna	gain	BASIS
PD24202	LRP1B	cna	hetloss	BASIS
PD24206	LRP1B	cna	gain	BASIS
PD3890	LRP1B	cna	hetloss	BASIS
PD3905	LRP1B	cna	gain	BASIS
PD4005	LRP1B	cna	hetloss	BASIS
PD4006	LRP1B	cna	homdel	BASIS
PD4107	LRP1B	cna	gain	BASIS
PD4967	LRP1B	cna	hetloss	BASIS
PD5935	LRP1B	cna	gain	BASIS
PD5945	LRP1B	cna	gain	BASIS
PD5948	LRP1B	cna	gain	BASIS
PD6406	LRP1B	cna	hetloss	BASIS
PD6413	LRP1B	cna	hetloss	BASIS
PD7215	LRP1B	cna	gain	BASIS
PD8621	LRP1B	cna	hetloss	BASIS
PD9702	LRP1B	cna	gain	BASIS
TCGA-A2-A25B	LRP5	cna	hetloss	TCGA
TCGA-AN-A0XU	LRP5	cna	hetloss	TCGA
TCGA-AO-A0JL	LRP5	cna	hetloss	TCGA
TCGA-BH-A18R	LRP5	cna	homdel	TCGA
TCGA-C8-A12L	LRP5	cna	gain	TCGA
TCGA-EW-A1OX	LRP5	cna	amp	TCGA
TCGA-LL-A5YP	LRP5	cna	hetloss	TCGA
MB-0346	LRP5	cna	gain	METABRIC
MB-5107	LRP5	cna	gain	METABRIC
MB-6271	LRP5	cna	gain	METABRIC
MB-7038	LRP5	cna	gain	METABRIC
PD11327	LRP5	cna	gain	BASIS
PD11742	LRP5	cna	gain	BASIS
PD13771	LRP5	cna	amp	BASIS
PD22355	LRP5	cna	hetloss	BASIS
PD23574	LRP5	cna	gain	BASIS
PD24186	LRP5	cna	amp	BASIS
PD24206	LRP5	cna	gain	BASIS
PD3905	LRP5	cna	gain	BASIS
PD4005	LRP5	cna	gain	BASIS

PD4006	LRP5	cna	amp	BASIS
PD4107	LRP5	cna	gain	BASIS
PD4826	LRP5	cna	gain	BASIS
PD5930	LRP5	cna	gain	BASIS
PD5935	LRP5	cna	gain	BASIS
PD5945	LRP5	cna	amp	BASIS
PD5948	LRP5	cna	amp	BASIS
PD6413	LRP5	cna	gain	BASIS
PD7067	LRP5	cna	gain	BASIS
PD7215	LRP5	cna	gain	BASIS
PD8621	LRP5	cna	gain	BASIS
PD8980	LRP5	cna	gain	BASIS
PD9004	LRP5	cna	gain	BASIS
PD9702	LRP5	cna	gain	BASIS
TCGA-A2-A25B	LRRK2	cna	gain	TCGA
TCGA-AN-A0XU	LRRK2	cna	gain	TCGA
TCGA-BH-A0C0	LRRK2	cna	hetloss	TCGA
TCGA-BH-A18R	LRRK2	cna	gain	TCGA
TCGA-BH-A1FU	LRRK2	cna	hetloss	TCGA
TCGA-C8-A12L	LRRK2	cna	gain	TCGA
TCGA-D8-A27M	LRRK2	cna	gain	TCGA
TCGA-E2-A1L7	LRRK2	cna	hetloss	TCGA
TCGA-LL-A5YP	LRRK2	cna	hetloss	TCGA
MB-0346	LRRK2	cna	gain	METABRIC
MB-2827	LRRK2	cna	hetloss	METABRIC
MB-5070	LRRK2	cna	hetloss	METABRIC
MB-6098	LRRK2	cna	gain	METABRIC
MTS-T0064	LRRK2	cna	gain	METABRIC
PD10014	LRRK2	cna	gain	BASIS
PD11327	LRRK2	cna	amp	BASIS
PD13296	LRRK2	cna	gain	BASIS
PD13297	LRRK2	cna	hetloss	BASIS
PD23574	LRRK2	cna	gain	BASIS
PD24186	LRRK2	cna	gain	BASIS
PD24202	LRRK2	cna	hetloss	BASIS
PD24206	LRRK2	cna	gain	BASIS
PD3905	LRRK2	cna	gain	BASIS
PD4005	LRRK2	cna	hetloss	BASIS
PD4107	LRRK2	cna	gain	BASIS
PD4826	LRRK2	cna	gain	BASIS
PD5935	LRRK2	cna	gain	BASIS
PD5945	LRRK2	cna	amp	BASIS
PD5948	LRRK2	cna	gain	BASIS
PD6731	LRRK2	cna	hetloss	BASIS
PD7067	LRRK2	cna	gain	BASIS
PD9004	LRRK2	cna	hetloss	BASIS
PD9585	LRRK2	cna	hetloss	BASIS

PD9702	LRRK2	cna	gain	BASIS
TCGA-A2-A25B	MAP3K8	cna	gain	TCGA
TCGA-AN-A0XU	MAP3K8	cna	hetloss	TCGA
TCGA-AO-A0JL	MAP3K8	cna	gain	TCGA
TCGA-BH-A0AW	MAP3K8	cna	gain	TCGA
TCGA-BH-A0C0	MAP3K8	cna	gain	TCGA
TCGA-C8-A12L	MAP3K8	cna	gain	TCGA
TCGA-LL-A5YP	MAP3K8	cna	gain	TCGA
MB-6098	MAP3K8	cna	gain	METABRIC
MB-7038	MAP3K8	cna	hetloss	METABRIC
MTS-T0064	MAP3K8	cna	gain	METABRIC
PD11327	MAP3K8	cna	gain	BASIS
PD13296	MAP3K8	cna	gain	BASIS
PD13299	MAP3K8	cna	gain	BASIS
PD13771	MAP3K8	cna	gain	BASIS
PD23562	MAP3K8	cna	gain	BASIS
PD23574	MAP3K8	cna	gain	BASIS
PD24186	MAP3K8	cna	gain	BASIS
PD24202	MAP3K8	cna	hetloss	BASIS
PD3905	MAP3K8	cna	gain	BASIS
PD4005	MAP3K8	cna	gain	BASIS
PD4006	MAP3K8	cna	gain	BASIS
PD4107	MAP3K8	cna	gain	BASIS
PD4826	MAP3K8	cna	gain	BASIS
PD5930	MAP3K8	cna	gain	BASIS
PD5935	MAP3K8	cna	gain	BASIS
PD5945	MAP3K8	cna	amp	BASIS
PD5948	MAP3K8	cna	gain	BASIS
PD6413	MAP3K8	cna	gain	BASIS
PD7067	MAP3K8	cna	amp	BASIS
PD7215	MAP3K8	cna	gain	BASIS
PD8621	MAP3K8	cna	gain	BASIS
PD8980	MAP3K8	cna	hetloss	BASIS
PD9004	MAP3K8	cna	gain	BASIS
PD9702	MAP3K8	cna	gain	BASIS
TCGA-A2-A25B	MDM2	cna	gain	TCGA
TCGA-AO-A0JL	MDM2	cna	hetloss	TCGA
TCGA-BH-A18R	MDM2	cna	gain	TCGA
TCGA-BH-A1FU	MDM2	cna	hetloss	TCGA
TCGA-C8-A12L	MDM2	cna	gain	TCGA
TCGA-E2-A1L7	MDM2	cna	hetloss	TCGA
TCGA-LL-A5YP	MDM2	cna	hetloss	TCGA
MB-0346	MDM2	cna	gain	METABRIC
MB-2827	MDM2	cna	hetloss	METABRIC
MB-5070	MDM2	cna	gain	METABRIC
MB-5107	MDM2	cna	gain	METABRIC
MB-5465	MDM2	cna	gain	METABRIC

MB-6098	MDM2	cna	gain	METABRIC
MTS-T0064	MDM2	cna	gain	METABRIC
PD11327	MDM2	cna	homdel	BASIS
PD11742	MDM2	cna	gain	BASIS
PD22355	MDM2	cna	hetloss	BASIS
PD23578	MDM2	cna	gain	BASIS
PD24186	MDM2	cna	gain	BASIS
PD24202	MDM2	cna	hetloss	BASIS
PD24206	MDM2	cna	gain	BASIS
PD3890	MDM2	cna	hetloss	BASIS
PD3905	MDM2	cna	gain	BASIS
PD4005	MDM2	cna	hetloss	BASIS
PD4006	MDM2	cna	gain	BASIS
PD4826	MDM2	cna	gain	BASIS
PD5945	MDM2	cna	gain	BASIS
PD6406	MDM2	cna	hetloss	BASIS
PD6413	MDM2	cna	hetloss	BASIS
PD6731	MDM2	cna	hetloss	BASIS
PD7067	MDM2	cna	gain	BASIS
PD8621	MDM2	cna	hetloss	BASIS
PD9585	MDM2	cna	hetloss	BASIS
PD9702	MDM2	cna	amp	BASIS
TCGA-AN-A0XU	MIB1	cna	hetloss	TCGA
TCGA-AO-A0JL	MIB1	cna	gain	TCGA
TCGA-BH-A0AW	MIB1	cna	gain	TCGA
TCGA-BH-A0C0	MIB1	cna	gain	TCGA
TCGA-C8-A12L	MIB1	cna	gain	TCGA
TCGA-D8-A27M	MIB1	cna	hetloss	TCGA
TCGA-E2-A1L7	MIB1	cna	gain	TCGA
TCGA-LL-A5YP	MIB1	cna	amp	TCGA
MB-5465	MIB1	cna	hetloss	METABRIC
PD10014	MIB1	cna	gain	BASIS
PD11327	MIB1	cna	gain	BASIS
PD13296	MIB1	cna	hetloss	BASIS
PD13297	MIB1	cna	gain	BASIS
PD13299	MIB1	cna	gain	BASIS
PD23562	MIB1	cna	gain	BASIS
PD23574	MIB1	cna	gain	BASIS
PD24186	MIB1	cna	gain	BASIS
PD24202	MIB1	cna	hetloss	BASIS
PD24206	MIB1	cna	gain	BASIS
PD24337	MIB1	cna	gain	BASIS
PD3890	MIB1	cna	gain	BASIS
PD3905	MIB1	cna	gain	BASIS
PD4006	MIB1	cna	gain	BASIS
PD4107	MIB1	cna	gain	BASIS
PD4967	MIB1	cna	hetloss	BASIS

PD5930	MIB1	cna	gain	BASIS
PD5935	MIB1	cna	gain	BASIS
PD5945	MIB1	cna	amp	BASIS
PD5948	MIB1	cna	gain	BASIS
PD7067	MIB1	cna	gain	BASIS
PD7215	MIB1	cna	gain	BASIS
PD8980	MIB1	cna	hetloss	BASIS
PD9004	MIB1	cna	gain	BASIS
PD9702	MIB1	cna	gain	BASIS
TCGA-AN-A0XU	MLLT1	cna	hetloss	TCGA
TCGA-AO-A0JL	MLLT1	cna	gain	TCGA
TCGA-BH-A0C0	MLLT1	cna	gain	TCGA
TCGA-BH-A1FU	MLLT1	cna	hetloss	TCGA
TCGA-C8-A12L	MLLT1	cna	hetloss	TCGA
TCGA-D8-A27M	MLLT1	cna	hetloss	TCGA
TCGA-E9-A1NC	MLLT1	cna	hetloss	TCGA
TCGA-EW-A1OX	MLLT1	cna	hetloss	TCGA
TCGA-LL-A5YP	MLLT1	cna	hetloss	TCGA
MB-0346	MLLT1	cna	gain	METABRIC
MB-2827	MLLT1	cna	hetloss	METABRIC
MB-5070	MLLT1	cna	hetloss	METABRIC
MB-5465	MLLT1	cna	gain	METABRIC
MB-6098	MLLT1	cna	hetloss	METABRIC
MB-7038	MLLT1	cna	hetloss	METABRIC
MB-0420	MLLT1	cna	hetloss	METABRIC
PD10014	MLLT1	cna	hetloss	BASIS
PD14442	MLLT1	cna	hetloss	BASIS
PD23574	MLLT1	cna	gain	BASIS
PD24202	MLLT1	cna	gain	BASIS
PD24206	MLLT1	cna	hetloss	BASIS
PD3905	MLLT1	cna	gain	BASIS
PD4006	MLLT1	cna	gain	BASIS
PD4107	MLLT1	cna	gain	BASIS
PD4826	MLLT1	cna	gain	BASIS
PD4967	MLLT1	cna	homdel	BASIS
PD5930	MLLT1	cna	gain	BASIS
PD5945	MLLT1	cna	gain	BASIS
PD5948	MLLT1	cna	gain	BASIS
PD7067	MLLT1	cna	gain	BASIS
PD7215	MLLT1	cna	amp	BASIS
PD8621	MLLT1	cna	gain	BASIS
PD8980	MLLT1	cna	gain	BASIS
PD9702	MLLT1	cna	gain	BASIS
TCGA-A2-A25B	MPL	cna	hetloss	TCGA
TCGA-AN-A0XU	MPL	cna	gain	TCGA
TCGA-AO-A0JL	MPL	cna	hetloss	TCGA
TCGA-BH-A0AW	MPL	cna	gain	TCGA

TCGA-BH-A0C0	MPL	cna	gain	TCGA
TCGA-BH-A1FU	MPL	cna	gain	TCGA
TCGA-C8-A12L	MPL	cna	hetloss	TCGA
TCGA-D8-A27M	MPL	cna	gain	TCGA
TCGA-E2-A1L7	MPL	cna	gain	TCGA
TCGA-LL-A5YP	MPL	cna	gain	TCGA
MB-0346	MPL	cna	hetloss	METABRIC
MB-6060	MPL	cna	hetloss	METABRIC
MB-6098	MPL	cna	gain	METABRIC
MB-7038	MPL	cna	gain	METABRIC
MB-0420	MPL	cna	hetloss	METABRIC
PD13296	MPL	cna	gain	BASIS
PD13299	MPL	cna	gain	BASIS
PD24186	MPL	cna	amp	BASIS
PD24202	MPL	cna	gain	BASIS
PD24206	MPL	cna	gain	BASIS
PD24337	MPL	cna	gain	BASIS
PD3905	MPL	cna	gain	BASIS
PD4006	MPL	cna	gain	BASIS
PD4107	MPL	cna	gain	BASIS
PD4826	MPL	cna	gain	BASIS
PD5935	MPL	cna	gain	BASIS
PD5945	MPL	cna	amp	BASIS
PD5948	MPL	cna	gain	BASIS
PD7067	MPL	cna	gain	BASIS
PD7215	MPL	cna	gain	BASIS
PD8621	MPL	cna	gain	BASIS
PD8980	MPL	cna	gain	BASIS
PD9004	MPL	cna	gain	BASIS
PD9702	MPL	cna	gain	BASIS
TCGA-AN-A0XU	MYH11	cna	gain	TCGA
TCGA-AO-A0JL	MYH11	cna	gain	TCGA
TCGA-BH-A0AW	MYH11	cna	gain	TCGA
TCGA-BH-A0C0	MYH11	cna	gain	TCGA
TCGA-C8-A12L	MYH11	cna	gain	TCGA
TCGA-E2-A1L7	MYH11	cna	hetloss	TCGA
TCGA-LL-A5YP	MYH11	cna	gain	TCGA
MB-6060	MYH11	cna	gain	METABRIC
MB-6271	MYH11	cna	gain	METABRIC
MB-7032	MYH11	cna	gain	METABRIC
MB-7038	MYH11	cna	hetloss	METABRIC
MB-0420	MYH11	cna	hetloss	METABRIC
PD10014	MYH11	cna	hetloss	BASIS
PD11327	MYH11	cna	hetloss	BASIS
PD11742	MYH11	cna	gain	BASIS
PD13296	MYH11	cna	gain	BASIS
PD13297	MYH11	cna	hetloss	BASIS

PD13299	MYH11	cna	gain	BASIS
PD22355	MYH11	cna	hetloss	BASIS
PD24186	MYH11	cna	gain	BASIS
PD24202	MYH11	cna	hetloss	BASIS
PD3905	MYH11	cna	gain	BASIS
PD4005	MYH11	cna	gain	BASIS
PD4006	MYH11	cna	gain	BASIS
PD4107	MYH11	cna	gain	BASIS
PD4826	MYH11	cna	gain	BASIS
PD5935	MYH11	cna	gain	BASIS
PD5945	MYH11	cna	gain	BASIS
PD5948	MYH11	cna	gain	BASIS
PD7067	MYH11	cna	gain	BASIS
PD7215	MYH11	cna	amp	BASIS
PD8980	MYH11	cna	hetloss	BASIS
PD9004	MYH11	cna	gain	BASIS
PD9585	MYH11	cna	hetloss	BASIS
TCGA-A2-A25B	NCOR2	cna	gain	TCGA
TCGA-AN-A0XU	NCOR2	cna	hetloss	TCGA
TCGA-AO-A0JL	NCOR2	cna	hetloss	TCGA
TCGA-BH-A0C0	NCOR2	cna	hetloss	TCGA
TCGA-BH-A18R	NCOR2	cna	gain	TCGA
TCGA-BH-A1FU	NCOR2	cna	hetloss	TCGA
TCGA-D8-A27M	NCOR2	cna	hetloss	TCGA
TCGA-E2-A1L7	NCOR2	cna	hetloss	TCGA
TCGA-LL-A5YP	NCOR2	cna	hetloss	TCGA
MB-0346	NCOR2	cna	amp	METABRIC
MB-2827	NCOR2	cna	hetloss	METABRIC
MB-5070	NCOR2	cna	hetloss	METABRIC
MB-7048	NCOR2	cna	hetloss	METABRIC
MTS-T0064	NCOR2	cna	amp	METABRIC
PD11327	NCOR2	cna	gain	BASIS
PD13296	NCOR2	cna	hetloss	BASIS
PD13297	NCOR2	cna	gain	BASIS
PD13771	NCOR2	cna	gain	BASIS
PD22355	NCOR2	cna	hetloss	BASIS
PD23574	NCOR2	cna	gain	BASIS
PD23578	NCOR2	cna	hetloss	BASIS
PD3890	NCOR2	cna	hetloss	BASIS
PD3905	NCOR2	cna	gain	BASIS
PD4005	NCOR2	cna	hetloss	BASIS
PD4006	NCOR2	cna	amp	BASIS
PD4107	NCOR2	cna	gain	BASIS
PD4826	NCOR2	cna	gain	BASIS
PD5935	NCOR2	cna	gain	BASIS
PD5945	NCOR2	cna	gain	BASIS
PD5948	NCOR2	cna	gain	BASIS

PD6731	NCOR2	cna	hetloss	BASIS
PD7067	NCOR2	cna	gain	BASIS
PD9585	NCOR2	cna	hetloss	BASIS
PD9702	NCOR2	cna	gain	BASIS
TCGA-A2-A25B	NEFH	cna	hetloss	TCGA
TCGA-BH-A0C0	NEFH	cna	gain	TCGA
TCGA-BH-A1FU	NEFH	cna	gain	TCGA
TCGA-C8-A12L	NEFH	cna	gain	TCGA
TCGA-D8-A27M	NEFH	cna	gain	TCGA
TCGA-E9-A1NC	NEFH	cna	gain	TCGA
TCGA-LL-A5YP	NEFH	cna	gain	TCGA
MB-0346	NEFH	cna	gain	METABRIC
MB-6060	NEFH	cna	gain	METABRIC
MB-6098	NEFH	cna	gain	METABRIC
PD11327	NEFH	cna	gain	BASIS
PD13296	NEFH	cna	gain	BASIS
PD13297	NEFH	cna	gain	BASIS
PD13299	NEFH	cna	gain	BASIS
PD13771	NEFH	cna	gain	BASIS
PD14442	NEFH	cna	hetloss	BASIS
PD23562	NEFH	cna	gain	BASIS
PD23574	NEFH	cna	gain	BASIS
PD24206	NEFH	cna	hetloss	BASIS
PD3905	NEFH	cna	gain	BASIS
PD4006	NEFH	cna	gain	BASIS
PD4107	NEFH	cna	amp	BASIS
PD4826	NEFH	cna	gain	BASIS
PD4967	NEFH	cna	hetloss	BASIS
PD5930	NEFH	cna	gain	BASIS
PD5935	NEFH	cna	gain	BASIS
PD5945	NEFH	cna	amp	BASIS
PD5948	NEFH	cna	gain	BASIS
PD6731	NEFH	cna	hetloss	BASIS
PD7067	NEFH	cna	gain	BASIS
PD7215	NEFH	cna	gain	BASIS
PD8621	NEFH	cna	gain	BASIS
PD9004	NEFH	cna	gain	BASIS
PD9702	NEFH	cna	gain	BASIS
MB-0346	NKX3-1	cna	hetloss	METABRIC
MB-2827	NKX3-1	cna	hetloss	METABRIC
MB-5070	NKX3-1	cna	hetloss	METABRIC
MB-5465	NKX3-1	cna	hetloss	METABRIC
MB-6060	NKX3-1	cna	hetloss	METABRIC
MB-6098	NKX3-1	cna	hetloss	METABRIC
MB-7038	NKX3-1	cna	hetloss	METABRIC
MB-0420	NKX3-1	cna	hetloss	METABRIC
MTS-T0064	NKX3-1	cna	hetloss	METABRIC

PD10014	NKX3-1	cna	hetloss	BASIS
PD11327	NKX3-1	cna	hetloss	BASIS
PD13296	NKX3-1	cna	hetloss	BASIS
PD13299	NKX3-1	cna	hetloss	BASIS
PD13771	NKX3-1	cna	hetloss	BASIS
PD14442	NKX3-1	cna	hetloss	BASIS
PD22355	NKX3-1	cna	hetloss	BASIS
PD23562	NKX3-1	cna	hetloss	BASIS
PD23574	NKX3-1	cna	hetloss	BASIS
PD23578	NKX3-1	cna	hetloss	BASIS
PD24186	NKX3-1	cna	gain	BASIS
PD24202	NKX3-1	cna	hetloss	BASIS
PD24206	NKX3-1	cna	hetloss	BASIS
PD24337	NKX3-1	cna	hetloss	BASIS
PD3905	NKX3-1	cna	gain	BASIS
PD4005	NKX3-1	cna	hetloss	BASIS
PD4006	NKX3-1	cna	gain	BASIS
PD4826	NKX3-1	cna	hetloss	BASIS
PD5935	NKX3-1	cna	gain	BASIS
PD5945	NKX3-1	cna	gain	BASIS
PD6406	NKX3-1	cna	hetloss	BASIS
PD6413	NKX3-1	cna	hetloss	BASIS
PD7067	NKX3-1	cna	homdel	BASIS
PD8980	NKX3-1	cna	hetloss	BASIS
PD9004	NKX3-1	cna	hetloss	BASIS
TCGA-AO-A0JL	NOTCH3	cna	gain	TCGA
TCGA-BH-A0C0	NOTCH3	cna	gain	TCGA
TCGA-BH-A1FU	NOTCH3	cna	hetloss	TCGA
TCGA-C8-A12L	NOTCH3	cna	gain	TCGA
TCGA-D8-A27M	NOTCH3	cna	hetloss	TCGA
TCGA-EW-A1OX	NOTCH3	cna	hetloss	TCGA
TCGA-LL-A5YP	NOTCH3	cna	hetloss	TCGA
MB-0346	NOTCH3	cna	hetloss	METABRIC
MB-2827	NOTCH3	cna	hetloss	METABRIC
MB-5465	NOTCH3	cna	gain	METABRIC
MB-6098	NOTCH3	cna	hetloss	METABRIC
MB-7038	NOTCH3	cna	gain	METABRIC
MB-0420	NOTCH3	cna	hetloss	METABRIC
P-0009557-T01-IM5	NOTCH3	cna	homdel	MSK-IMPACT
PD14442	NOTCH3	cna	hetloss	BASIS
PD22355	NOTCH3	cna	gain	BASIS
PD23562	NOTCH3	cna	gain	BASIS
PD23574	NOTCH3	cna	gain	BASIS
PD24186	NOTCH3	cna	gain	BASIS
PD24206	NOTCH3	cna	hetloss	BASIS
PD3905	NOTCH3	cna	gain	BASIS
PD4006	NOTCH3	cna	amp	BASIS

PD4107	NOTCH3	cna	gain	BASIS
PD4826	NOTCH3	cna	gain	BASIS
PD4967	NOTCH3	cna	hetloss	BASIS
PD5930	NOTCH3	cna	gain	BASIS
PD5935	NOTCH3	cna	gain	BASIS
PD5945	NOTCH3	cna	amp	BASIS
PD5948	NOTCH3	cna	gain	BASIS
PD6413	NOTCH3	cna	gain	BASIS
PD7067	NOTCH3	cna	gain	BASIS
PD7215	NOTCH3	cna	amp	BASIS
PD8621	NOTCH3	cna	gain	BASIS
PD9702	NOTCH3	cna	gain	BASIS
TCGA-A2-A25B	NR4A3	cna	gain	TCGA
TCGA-AN-A0XU	NR4A3	cna	hetloss	TCGA
TCGA-AO-A0JL	NR4A3	cna	hetloss	TCGA
TCGA-BH-A1FU	NR4A3	cna	hetloss	TCGA
TCGA-C8-A12L	NR4A3	cna	hetloss	TCGA
TCGA-E2-A1L7	NR4A3	cna	hetloss	TCGA
TCGA-E9-A1NC	NR4A3	cna	hetloss	TCGA
TCGA-LL-A5YP	NR4A3	cna	hetloss	TCGA
MB-0346	NR4A3	cna	hetloss	METABRIC
MB-2827	NR4A3	cna	hetloss	METABRIC
MB-5070	NR4A3	cna	hetloss	METABRIC
MB-5107	NR4A3	cna	hetloss	METABRIC
MB-6098	NR4A3	cna	hetloss	METABRIC
MB-7038	NR4A3	cna	hetloss	METABRIC
MB-0420	NR4A3	cna	hetloss	METABRIC
PD11327	NR4A3	cna	gain	BASIS
PD23562	NR4A3	cna	gain	BASIS
PD23578	NR4A3	cna	hetloss	BASIS
PD24186	NR4A3	cna	amp	BASIS
PD24202	NR4A3	cna	hetloss	BASIS
PD24206	NR4A3	cna	gain	BASIS
PD3905	NR4A3	cna	gain	BASIS
PD4005	NR4A3	cna	gain	BASIS
PD4006	NR4A3	cna	gain	BASIS
PD4107	NR4A3	cna	hetloss	BASIS
PD5945	NR4A3	cna	amp	BASIS
PD6406	NR4A3	cna	hetloss	BASIS
PD6731	NR4A3	cna	hetloss	BASIS
PD7067	NR4A3	cna	gain	BASIS
PD7215	NR4A3	cna	gain	BASIS
PD8621	NR4A3	cna	gain	BASIS
PD8980	NR4A3	cna	gain	BASIS
PD9004	NR4A3	cna	gain	BASIS
PD9585	NR4A3	cna	hetloss	BASIS
TCGA-A2-A25B	NUP214	cna	gain	TCGA

TCGA-AN-A0XU	NUP214	cna	hetloss	TCGA
TCGA-AO-A0JL	NUP214	cna	hetloss	TCGA
TCGA-BH-A0C0	NUP214	cna	gain	TCGA
TCGA-C8-A12L	NUP214	cna	gain	TCGA
TCGA-E2-A1L7	NUP214	cna	gain	TCGA
TCGA-E9-A1NC	NUP214	cna	hetloss	TCGA
TCGA-LL-A5YP	NUP214	cna	hetloss	TCGA
MB-0346	NUP214	cna	hetloss	METABRIC
MB-5070	NUP214	cna	hetloss	METABRIC
MB-5107	NUP214	cna	gain	METABRIC
MB-7038	NUP214	cna	hetloss	METABRIC
MB-0420	NUP214	cna	hetloss	METABRIC
PD10014	NUP214	cna	gain	BASIS
PD11327	NUP214	cna	hetloss	BASIS
PD13299	NUP214	cna	gain	BASIS
PD22355	NUP214	cna	gain	BASIS
PD24186	NUP214	cna	gain	BASIS
PD24202	NUP214	cna	hetloss	BASIS
PD24206	NUP214	cna	gain	BASIS
PD24337	NUP214	cna	hetloss	BASIS
PD3890	NUP214	cna	hetloss	BASIS
PD3905	NUP214	cna	gain	BASIS
PD4005	NUP214	cna	hetloss	BASIS
PD4006	NUP214	cna	gain	BASIS
PD4107	NUP214	cna	hetloss	BASIS
PD5945	NUP214	cna	gain	BASIS
PD5948	NUP214	cna	gain	BASIS
PD6406	NUP214	cna	hetloss	BASIS
PD6731	NUP214	cna	hetloss	BASIS
PD7067	NUP214	cna	gain	BASIS
PD7215	NUP214	cna	gain	BASIS
PD8621	NUP214	cna	gain	BASIS
PD9004	NUP214	cna	hetloss	BASIS
TCGA-A2-A25B	OMD	cna	gain	TCGA
TCGA-AN-A0XU	OMD	cna	hetloss	TCGA
TCGA-AO-A0JL	OMD	cna	hetloss	TCGA
TCGA-BH-A1FU	OMD	cna	hetloss	TCGA
TCGA-C8-A12L	OMD	cna	hetloss	TCGA
TCGA-E9-A1NC	OMD	cna	hetloss	TCGA
TCGA-LL-A5YP	OMD	cna	hetloss	TCGA
MB-0346	OMD	cna	hetloss	METABRIC
MB-2827	OMD	cna	amp	METABRIC
MB-5070	OMD	cna	hetloss	METABRIC
MB-5107	OMD	cna	hetloss	METABRIC
MB-6098	OMD	cna	hetloss	METABRIC
MB-7038	OMD	cna	hetloss	METABRIC
MB-0420	OMD	cna	hetloss	METABRIC

MTS-T0064	OMD	cna	gain	METABRIC
PD11327	OMD	cna	gain	BASIS
PD13771	OMD	cna	hetloss	BASIS
PD23562	OMD	cna	gain	BASIS
PD23578	OMD	cna	hetloss	BASIS
PD24186	OMD	cna	amp	BASIS
PD24202	OMD	cna	hetloss	BASIS
PD24206	OMD	cna	gain	BASIS
PD3905	OMD	cna	gain	BASIS
PD4005	OMD	cna	gain	BASIS
PD4006	OMD	cna	gain	BASIS
PD4107	OMD	cna	gain	BASIS
PD5945	OMD	cna	amp	BASIS
PD5948	OMD	cna	gain	BASIS
PD6731	OMD	cna	hetloss	BASIS
PD7067	OMD	cna	gain	BASIS
PD7215	OMD	cna	gain	BASIS
PD8621	OMD	cna	gain	BASIS
PD9004	OMD	cna	gain	BASIS
PD9585	OMD	cna	hetloss	BASIS
TCGA-A2-A25B	PDGFB	cna	hetloss	TCGA
TCGA-BH-A0AW	PDGFB	cna	hetloss	TCGA
TCGA-BH-A1FU	PDGFB	cna	gain	TCGA
TCGA-C8-A12L	PDGFB	cna	gain	TCGA
TCGA-D8-A27M	PDGFB	cna	gain	TCGA
TCGA-E9-A1NC	PDGFB	cna	gain	TCGA
TCGA-LL-A5YP	PDGFB	cna	gain	TCGA
MB-5465	PDGFB	cna	gain	METABRIC
MB-6098	PDGFB	cna	gain	METABRIC
MTS-T0064	PDGFB	cna	gain	METABRIC
PD11742	PDGFB	cna	hetloss	BASIS
PD13296	PDGFB	cna	gain	BASIS
PD13297	PDGFB	cna	gain	BASIS
PD13299	PDGFB	cna	gain	BASIS
PD14442	PDGFB	cna	hetloss	BASIS
PD22355	PDGFB	cna	gain	BASIS
PD23562	PDGFB	cna	gain	BASIS
PD23578	PDGFB	cna	gain	BASIS
PD24206	PDGFB	cna	homdel	BASIS
PD3905	PDGFB	cna	gain	BASIS
PD4005	PDGFB	cna	gain	BASIS
PD4006	PDGFB	cna	gain	BASIS
PD4107	PDGFB	cna	gain	BASIS
PD4826	PDGFB	cna	gain	BASIS
PD4967	PDGFB	cna	hetloss	BASIS
PD5935	PDGFB	cna	gain	BASIS
PD5945	PDGFB	cna	amp	BASIS

PD5948	PDGFB	cna	gain	BASIS
PD6731	PDGFB	cna	hetloss	BASIS
PD7067	PDGFB	cna	gain	BASIS
PD7215	PDGFB	cna	gain	BASIS
PD8621	PDGFB	cna	gain	BASIS
PD9004	PDGFB	cna	gain	BASIS
PD9702	PDGFB	cna	gain	BASIS
TCGA-AN-A0XU	PKM	cna	amp	TCGA
TCGA-AO-A0JL	PKM	cna	hetloss	TCGA
TCGA-BH-A0AW	PKM	cna	hetloss	TCGA
TCGA-BH-A0C0	PKM	cna	hetloss	TCGA
TCGA-D8-A27M	PKM	cna	hetloss	TCGA
TCGA-E2-A1L7	PKM	cna	gain	TCGA
TCGA-LL-A5YP	PKM	cna	amp	TCGA
MB-0346	PKM	cna	hetloss	METABRIC
MB-5070	PKM	cna	hetloss	METABRIC
MB-6060	PKM	cna	gain	METABRIC
MB-7038	PKM	cna	gain	METABRIC
PD11327	PKM	cna	gain	BASIS
PD11742	PKM	cna	hetloss	BASIS
PD13296	PKM	cna	homdel	BASIS
PD13297	PKM	cna	hetloss	BASIS
PD13299	PKM	cna	gain	BASIS
PD13771	PKM	cna	gain	BASIS
PD22355	PKM	cna	hetloss	BASIS
PD23562	PKM	cna	gain	BASIS
PD23574	PKM	cna	gain	BASIS
PD23578	PKM	cna	gain	BASIS
PD24202	PKM	cna	hetloss	BASIS
PD24206	PKM	cna	gain	BASIS
PD3890	PKM	cna	hetloss	BASIS
PD3905	PKM	cna	gain	BASIS
PD4006	PKM	cna	gain	BASIS
PD5945	PKM	cna	gain	BASIS
PD5948	PKM	cna	gain	BASIS
PD6406	PKM	cna	hetloss	BASIS
PD6413	PKM	cna	hetloss	BASIS
PD7067	PKM	cna	gain	BASIS
PD8980	PKM	cna	gain	BASIS
PD9585	PKM	cna	hetloss	BASIS
PD9702	PKM	cna	gain	BASIS
TCGA-A2-A25B	PMEL	cna	gain	TCGA
TCGA-AO-A0JL	PMEL	cna	hetloss	TCGA
TCGA-BH-A18R	PMEL	cna	gain	TCGA
TCGA-BH-A1FU	PMEL	cna	hetloss	TCGA
TCGA-C8-A12L	PMEL	cna	gain	TCGA
TCGA-D8-A27M	PMEL	cna	hetloss	TCGA

TCGA-E2-A1L7	PMEL	cna	hetloss	TCGA
TCGA-E9-A1NC	PMEL	cna	hetloss	TCGA
TCGA-LL-A5YP	PMEL	cna	hetloss	TCGA
MB-0346	PMEL	cna	gain	METABRIC
MB-2827	PMEL	cna	hetloss	METABRIC
MB-6098	PMEL	cna	hetloss	METABRIC
MB-7038	PMEL	cna	hetloss	METABRIC
MB-0420	PMEL	cna	hetloss	METABRIC
PD11327	PMEL	cna	homdel	BASIS
PD13296	PMEL	cna	hetloss	BASIS
PD13297	PMEL	cna	hetloss	BASIS
PD13771	PMEL	cna	gain	BASIS
PD24186	PMEL	cna	gain	BASIS
PD24202	PMEL	cna	hetloss	BASIS
PD24206	PMEL	cna	gain	BASIS
PD24337	PMEL	cna	hetloss	BASIS
PD3890	PMEL	cna	hetloss	BASIS
PD3905	PMEL	cna	gain	BASIS
PD4826	PMEL	cna	gain	BASIS
PD5945	PMEL	cna	gain	BASIS
PD5948	PMEL	cna	gain	BASIS
PD6406	PMEL	cna	hetloss	BASIS
PD6413	PMEL	cna	hetloss	BASIS
PD6731	PMEL	cna	hetloss	BASIS
PD7067	PMEL	cna	gain	BASIS
PD8621	PMEL	cna	hetloss	BASIS
PD9585	PMEL	cna	hetloss	BASIS
PD9702	PMEL	cna	gain	BASIS
TCGA-AO-A0JL	PPARG	cna	hetloss	TCGA
TCGA-BH-A0C0	PPARG	cna	gain	TCGA
TCGA-BH-A18R	PPARG	cna	gain	TCGA
TCGA-BH-A1FU	PPARG	cna	hetloss	TCGA
TCGA-D8-A27M	PPARG	cna	hetloss	TCGA
TCGA-E9-A1NC	PPARG	cna	hetloss	TCGA
TCGA-LL-A5YP	PPARG	cna	amp	TCGA
MB-5070	PPARG	cna	hetloss	METABRIC
MB-7038	PPARG	cna	hetloss	METABRIC
MB-7048	PPARG	cna	hetloss	METABRIC
MB-0420	PPARG	cna	hetloss	METABRIC
PD10014	PPARG	cna	hetloss	BASIS
PD11742	PPARG	cna	gain	BASIS
PD13296	PPARG	cna	gain	BASIS
PD13299	PPARG	cna	gain	BASIS
PD13771	PPARG	cna	gain	BASIS
PD23561	PPARG	cna	hetloss	BASIS
PD23562	PPARG	cna	gain	BASIS
PD23574	PPARG	cna	gain	BASIS

PD24206	PPARG	cna	gain	BASIS
PD24337	PPARG	cna	hetloss	BASIS
PD3905	PPARG	cna	gain	BASIS
PD4006	PPARG	cna	gain	BASIS
PD4826	PPARG	cna	gain	BASIS
PD5945	PPARG	cna	amp	BASIS
PD5948	PPARG	cna	gain	BASIS
PD6406	PPARG	cna	hetloss	BASIS
PD6731	PPARG	cna	hetloss	BASIS
PD7067	PPARG	cna	gain	BASIS
PD7215	PPARG	cna	gain	BASIS
PD8980	PPARG	cna	gain	BASIS
PD9004	PPARG	cna	gain	BASIS
PD9585	PPARG	cna	hetloss	BASIS
PD9702	PPARG	cna	gain	BASIS
TCGA-A2-A25B	PRKAA2	cna	gain	TCGA
TCGA-AN-A0XU	PRKAA2	cna	gain	TCGA
TCGA-AO-A0JL	PRKAA2	cna	hetloss	TCGA
TCGA-BH-A0AW	PRKAA2	cna	gain	TCGA
TCGA-BH-A0C0	PRKAA2	cna	hetloss	TCGA
TCGA-BH-A1FU	PRKAA2	cna	gain	TCGA
TCGA-D8-A27M	PRKAA2	cna	gain	TCGA
TCGA-E2-A1L7	PRKAA2	cna	amp	TCGA
TCGA-LL-A5YP	PRKAA2	cna	hetloss	TCGA
MB-0346	PRKAA2	cna	hetloss	METABRIC
MB-6060	PRKAA2	cna	gain	METABRIC
MB-7038	PRKAA2	cna	gain	METABRIC
MB-0420	PRKAA2	cna	hetloss	METABRIC
PD11327	PRKAA2	cna	gain	BASIS
PD11742	PRKAA2	cna	hetloss	BASIS
PD13296	PRKAA2	cna	gain	BASIS
PD13299	PRKAA2	cna	gain	BASIS
PD22355	PRKAA2	cna	hetloss	BASIS
PD23574	PRKAA2	cna	gain	BASIS
PD24186	PRKAA2	cna	amp	BASIS
PD24206	PRKAA2	cna	gain	BASIS
PD3890	PRKAA2	cna	gain	BASIS
PD3905	PRKAA2	cna	gain	BASIS
PD4006	PRKAA2	cna	gain	BASIS
PD4107	PRKAA2	cna	gain	BASIS
PD4826	PRKAA2	cna	gain	BASIS
PD5930	PRKAA2	cna	gain	BASIS
PD5945	PRKAA2	cna	amp	BASIS
PD5948	PRKAA2	cna	gain	BASIS
PD7067	PRKAA2	cna	gain	BASIS
PD7215	PRKAA2	cna	gain	BASIS
PD8621	PRKAA2	cna	gain	BASIS

PD9004	PRKAA2	cna	gain	BASIS
PD9702	PRKAA2	cna	gain	BASIS
TCGA-AO-A0JL	RAF1	cna	hetloss	TCGA
TCGA-BH-A0C0	RAF1	cna	gain	TCGA
TCGA-BH-A18R	RAF1	cna	gain	TCGA
TCGA-BH-A1FU	RAF1	cna	hetloss	TCGA
TCGA-D8-A27M	RAF1	cna	hetloss	TCGA
TCGA-E9-A1NC	RAF1	cna	hetloss	TCGA
TCGA-LL-A5YP	RAF1	cna	amp	TCGA
MB-5070	RAF1	cna	hetloss	METABRIC
MB-7038	RAF1	cna	hetloss	METABRIC
MB-7048	RAF1	cna	hetloss	METABRIC
MB-0420	RAF1	cna	hetloss	METABRIC
PD10014	RAF1	cna	hetloss	BASIS
PD11742	RAF1	cna	gain	BASIS
PD13296	RAF1	cna	gain	BASIS
PD13299	RAF1	cna	gain	BASIS
PD13771	RAF1	cna	gain	BASIS
PD23561	RAF1	cna	hetloss	BASIS
PD23562	RAF1	cna	gain	BASIS
PD23574	RAF1	cna	gain	BASIS
PD24206	RAF1	cna	gain	BASIS
PD24337	RAF1	cna	hetloss	BASIS
PD3905	RAF1	cna	gain	BASIS
PD4006	RAF1	cna	gain	BASIS
PD4826	RAF1	cna	gain	BASIS
PD5945	RAF1	cna	amp	BASIS
PD5948	RAF1	cna	gain	BASIS
PD6406	RAF1	cna	hetloss	BASIS
PD6731	RAF1	cna	hetloss	BASIS
PD7067	RAF1	cna	gain	BASIS
PD7215	RAF1	cna	gain	BASIS
PD8980	RAF1	cna	gain	BASIS
PD9004	RAF1	cna	gain	BASIS
PD9585	RAF1	cna	hetloss	BASIS
PD9702	RAF1	cna	gain	BASIS
TCGA-A2-A25B	ROR2	cna	gain	TCGA
TCGA-AN-A0XU	ROR2	cna	hetloss	TCGA
TCGA-AO-A0JL	ROR2	cna	hetloss	TCGA
TCGA-BH-A1FU	ROR2	cna	hetloss	TCGA
TCGA-C8-A12L	ROR2	cna	hetloss	TCGA
TCGA-E9-A1NC	ROR2	cna	hetloss	TCGA
TCGA-LL-A5YP	ROR2	cna	hetloss	TCGA
MB-0346	ROR2	cna	hetloss	METABRIC
MB-2827	ROR2	cna	amp	METABRIC
MB-5070	ROR2	cna	hetloss	METABRIC
MB-5107	ROR2	cna	hetloss	METABRIC

MB-6098	ROR2	cna	hetloss	METABRIC
MB-7038	ROR2	cna	hetloss	METABRIC
MB-0420	ROR2	cna	hetloss	METABRIC
MTS-T0064	ROR2	cna	gain	METABRIC
PD11327	ROR2	cna	gain	BASIS
PD13771	ROR2	cna	hetloss	BASIS
PD23562	ROR2	cna	gain	BASIS
PD23578	ROR2	cna	hetloss	BASIS
PD24186	ROR2	cna	amp	BASIS
PD24202	ROR2	cna	hetloss	BASIS
PD24206	ROR2	cna	gain	BASIS
PD3905	ROR2	cna	gain	BASIS
PD4005	ROR2	cna	gain	BASIS
PD4006	ROR2	cna	gain	BASIS
PD4107	ROR2	cna	gain	BASIS
PD5945	ROR2	cna	amp	BASIS
PD5948	ROR2	cna	gain	BASIS
PD6731	ROR2	cna	hetloss	BASIS
PD7067	ROR2	cna	gain	BASIS
PD7215	ROR2	cna	gain	BASIS
PD8621	ROR2	cna	gain	BASIS
PD9004	ROR2	cna	gain	BASIS
PD9585	ROR2	cna	hetloss	BASIS
TCGA-AN-A0XU	RPL5	cna	hetloss	TCGA
TCGA-BH-A0AW	RPL5	cna	gain	TCGA
TCGA-BH-A0C0	RPL5	cna	hetloss	TCGA
TCGA-BH-A1FU	RPL5	cna	gain	TCGA
TCGA-C8-A12L	RPL5	cna	hetloss	TCGA
TCGA-D8-A27M	RPL5	cna	hetloss	TCGA
TCGA-E2-A1L7	RPL5	cna	gain	TCGA
TCGA-LL-A5YP	RPL5	cna	hetloss	TCGA
MB-0346	RPL5	cna	hetloss	METABRIC
MB-2827	RPL5	cna	hetloss	METABRIC
MB-7048	RPL5	cna	hetloss	METABRIC
PD11327	RPL5	cna	hetloss	BASIS
PD11742	RPL5	cna	hetloss	BASIS
PD13299	RPL5	cna	gain	BASIS
PD22355	RPL5	cna	hetloss	BASIS
PD23574	RPL5	cna	gain	BASIS
PD23578	RPL5	cna	gain	BASIS
PD24186	RPL5	cna	gain	BASIS
PD24202	RPL5	cna	gain	BASIS
PD24206	RPL5	cna	hetloss	BASIS
PD3905	RPL5	cna	gain	BASIS
PD4005	RPL5	cna	hetloss	BASIS
PD4006	RPL5	cna	gain	BASIS
PD4107	RPL5	cna	gain	BASIS

PD4826	RPL5	cna	gain	BASIS
PD5930	RPL5	cna	gain	BASIS
PD5945	RPL5	cna	gain	BASIS
PD5948	RPL5	cna	gain	BASIS
PD7067	RPL5	cna	gain	BASIS
PD7215	RPL5	cna	gain	BASIS
PD8621	RPL5	cna	gain	BASIS
PD9004	RPL5	cna	gain	BASIS
PD9585	RPL5	cna	hetloss	BASIS
PD9702	RPL5	cna	gain	BASIS
TCGA-A2-A25B	RPS26	cna	gain	TCGA
TCGA-AO-A0JL	RPS26	cna	hetloss	TCGA
TCGA-BH-A18R	RPS26	cna	gain	TCGA
TCGA-BH-A1FU	RPS26	cna	hetloss	TCGA
TCGA-C8-A12L	RPS26	cna	gain	TCGA
TCGA-D8-A27M	RPS26	cna	hetloss	TCGA
TCGA-E2-A1L7	RPS26	cna	hetloss	TCGA
TCGA-E9-A1NC	RPS26	cna	hetloss	TCGA
TCGA-LL-A5YP	RPS26	cna	hetloss	TCGA
MB-0346	RPS26	cna	gain	METABRIC
MB-2827	RPS26	cna	hetloss	METABRIC
MB-6098	RPS26	cna	hetloss	METABRIC
MB-7038	RPS26	cna	hetloss	METABRIC
MB-0420	RPS26	cna	hetloss	METABRIC
PD11327	RPS26	cna	homdel	BASIS
PD13296	RPS26	cna	hetloss	BASIS
PD13297	RPS26	cna	hetloss	BASIS
PD13771	RPS26	cna	gain	BASIS
PD24186	RPS26	cna	gain	BASIS
PD24202	RPS26	cna	hetloss	BASIS
PD24206	RPS26	cna	gain	BASIS
PD24337	RPS26	cna	hetloss	BASIS
PD3890	RPS26	cna	hetloss	BASIS
PD3905	RPS26	cna	gain	BASIS
PD4826	RPS26	cna	gain	BASIS
PD5945	RPS26	cna	gain	BASIS
PD5948	RPS26	cna	gain	BASIS
PD6406	RPS26	cna	hetloss	BASIS
PD6413	RPS26	cna	hetloss	BASIS
PD6731	RPS26	cna	hetloss	BASIS
PD7067	RPS26	cna	gain	BASIS
PD8621	RPS26	cna	hetloss	BASIS
PD9585	RPS26	cna	hetloss	BASIS
PD9702	RPS26	cna	gain	BASIS
TCGA-AO-A0JL	S1PR2	cna	gain	TCGA
TCGA-BH-A0C0	S1PR2	cna	gain	TCGA
TCGA-BH-A1FU	S1PR2	cna	hetloss	TCGA

TCGA-C8-A12L	S1PR2	cna	gain	TCGA
TCGA-D8-A27M	S1PR2	cna	hetloss	TCGA
TCGA-EW-A1OX	S1PR2	cna	hetloss	TCGA
TCGA-LL-A5YP	S1PR2	cna	hetloss	TCGA
MB-0346	S1PR2	cna	hetloss	METABRIC
MB-2827	S1PR2	cna	hetloss	METABRIC
MB-5070	S1PR2	cna	hetloss	METABRIC
MB-5465	S1PR2	cna	gain	METABRIC
MB-6098	S1PR2	cna	hetloss	METABRIC
MB-7038	S1PR2	cna	hetloss	METABRIC
MB-0420	S1PR2	cna	hetloss	METABRIC
PD11327	S1PR2	cna	homdel	BASIS
PD13771	S1PR2	cna	gain	BASIS
PD14442	S1PR2	cna	hetloss	BASIS
PD23562	S1PR2	cna	gain	BASIS
PD23574	S1PR2	cna	gain	BASIS
PD24206	S1PR2	cna	homdel	BASIS
PD3905	S1PR2	cna	gain	BASIS
PD4006	S1PR2	cna	gain	BASIS
PD4107	S1PR2	cna	gain	BASIS
PD4826	S1PR2	cna	gain	BASIS
PD4967	S1PR2	cna	hetloss	BASIS
PD5930	S1PR2	cna	gain	BASIS
PD5935	S1PR2	cna	gain	BASIS
PD5945	S1PR2	cna	amp	BASIS
PD5948	S1PR2	cna	gain	BASIS
PD6406	S1PR2	cna	amp	BASIS
PD7067	S1PR2	cna	gain	BASIS
PD7215	S1PR2	cna	amp	BASIS
PD8621	S1PR2	cna	gain	BASIS
PD9702	S1PR2	cna	gain	BASIS
TCGA-A2-A25B	SDC4	cna	gain	TCGA
TCGA-AO-A0JL	SDC4	cna	gain	TCGA
TCGA-BH-A0AW	SDC4	cna	gain	TCGA
TCGA-BH-A0C0	SDC4	cna	gain	TCGA
TCGA-C8-A12L	SDC4	cna	gain	TCGA
TCGA-D8-A27M	SDC4	cna	gain	TCGA
TCGA-E2-A1L7	SDC4	cna	gain	TCGA
TCGA-E9-A1NC	SDC4	cna	hetloss	TCGA
TCGA-LL-A5YP	SDC4	cna	hetloss	TCGA
MB-0346	SDC4	cna	gain	METABRIC
MB-5107	SDC4	cna	gain	METABRIC
MB-6060	SDC4	cna	amp	METABRIC
MB-6098	SDC4	cna	hetloss	METABRIC
PD11327	SDC4	cna	homdel	BASIS
PD13297	SDC4	cna	hetloss	BASIS
PD13299	SDC4	cna	gain	BASIS

PD13771	SDC4	cna	gain	BASIS
PD22355	SDC4	cna	hetloss	BASIS
PD24186	SDC4	cna	gain	BASIS
PD24202	SDC4	cna	hetloss	BASIS
PD3905	SDC4	cna	gain	BASIS
PD4005	SDC4	cna	hetloss	BASIS
PD4006	SDC4	cna	gain	BASIS
PD4107	SDC4	cna	gain	BASIS
PD4826	SDC4	cna	gain	BASIS
PD5935	SDC4	cna	gain	BASIS
PD5945	SDC4	cna	amp	BASIS
PD5948	SDC4	cna	gain	BASIS
PD6731	SDC4	cna	hetloss	BASIS
PD7067	SDC4	cna	gain	BASIS
PD7215	SDC4	cna	amp	BASIS
PD8980	SDC4	cna	gain	BASIS
PD9004	SDC4	cna	gain	BASIS
PD9585	SDC4	cna	hetloss	BASIS
TCGA-A2-A25B	SEMG1	cna	gain	TCGA
TCGA-AO-A0JL	SEMG1	cna	gain	TCGA
TCGA-BH-A0AW	SEMG1	cna	gain	TCGA
TCGA-BH-A0C0	SEMG1	cna	gain	TCGA
TCGA-C8-A12L	SEMG1	cna	gain	TCGA
TCGA-D8-A27M	SEMG1	cna	gain	TCGA
TCGA-E2-A1L7	SEMG1	cna	gain	TCGA
TCGA-E9-A1NC	SEMG1	cna	hetloss	TCGA
TCGA-LL-A5YP	SEMG1	cna	hetloss	TCGA
MB-0346	SEMG1	cna	gain	METABRIC
MB-5107	SEMG1	cna	gain	METABRIC
MB-6060	SEMG1	cna	amp	METABRIC
MB-6098	SEMG1	cna	hetloss	METABRIC
PD11327	SEMG1	cna	homdel	BASIS
PD13297	SEMG1	cna	hetloss	BASIS
PD13299	SEMG1	cna	gain	BASIS
PD13771	SEMG1	cna	gain	BASIS
PD22355	SEMG1	cna	hetloss	BASIS
PD24186	SEMG1	cna	gain	BASIS
PD24202	SEMG1	cna	hetloss	BASIS
PD3905	SEMG1	cna	gain	BASIS
PD4005	SEMG1	cna	hetloss	BASIS
PD4006	SEMG1	cna	gain	BASIS
PD4107	SEMG1	cna	gain	BASIS
PD4826	SEMG1	cna	gain	BASIS
PD5935	SEMG1	cna	gain	BASIS
PD5945	SEMG1	cna	amp	BASIS
PD5948	SEMG1	cna	gain	BASIS
PD6731	SEMG1	cna	hetloss	BASIS

PD7067	SEMG1	cna	gain	BASIS
PD7215	SEMG1	cna	amp	BASIS
PD8980	SEMG1	cna	gain	BASIS
PD9004	SEMG1	cna	gain	BASIS
PD9585	SEMG1	cna	hetloss	BASIS
TCGA-AN-A0XU	SETD2	cna	hetloss	TCGA
TCGA-BH-A0C0	SETD2	cna	hetloss	TCGA
TCGA-BH-A1FU	SETD2	cna	hetloss	TCGA
TCGA-C8-A12L	SETD2	cna	hetloss	TCGA
TCGA-D8-A27M	SETD2	cna	hetloss	TCGA
TCGA-E9-A1NC	SETD2	cna	hetloss	TCGA
TCGA-LL-A5YP	SETD2	cna	hetloss	TCGA
MB-2827	SETD2	cna	hetloss	METABRIC
MB-5107	SETD2	cna	hetloss	METABRIC
MB-7038	SETD2	cna	hetloss	METABRIC
MB-7048	SETD2	cna	hetloss	METABRIC
MB-0420	SETD2	cna	hetloss	METABRIC
P-0009557-T01-IM5	SETD2	cna	homdel	MSK-IMPACT
PD10014	SETD2	cna	hetloss	BASIS
PD11327	SETD2	cna	homdel	BASIS
PD13296	SETD2	cna	homdel	BASIS
PD13297	SETD2	cna	hetloss	BASIS
PD13299	SETD2	cna	gain	BASIS
PD22355	SETD2	cna	hetloss	BASIS
PD23561	SETD2	cna	hetloss	BASIS
PD23578	SETD2	cna	hetloss	BASIS
PD24202	SETD2	cna	hetloss	BASIS
PD24206	SETD2	cna	gain	BASIS
PD24337	SETD2	cna	hetloss	BASIS
PD3890	SETD2	cna	hetloss	BASIS
PD3905	SETD2	cna	gain	BASIS
PD4005	SETD2	cna	hetloss	BASIS
PD4826	SETD2	cna	gain	BASIS
PD5945	SETD2	cna	gain	BASIS
PD6406	SETD2	cna	hetloss	BASIS
PD6413	SETD2	cna	hetloss	BASIS
PD6731	SETD2	cna	hetloss	BASIS
PD7067	SETD2	cna	gain	BASIS
PD9585	SETD2	cna	hetloss	BASIS
TCGA-AN-A0XU	SHQ1	cna	hetloss	TCGA
TCGA-BH-A0C0	SHQ1	cna	hetloss	TCGA
TCGA-BH-A18R	SHQ1	cna	homdel	TCGA
TCGA-BH-A1FU	SHQ1	cna	hetloss	TCGA
TCGA-C8-A12L	SHQ1	cna	gain	TCGA
TCGA-D8-A27M	SHQ1	cna	hetloss	TCGA
TCGA-E2-A1L7	SHQ1	cna	homdel	TCGA
TCGA-E9-A1NC	SHQ1	cna	hetloss	TCGA

TCGA-LL-A5YP	SHQ1	cna	gain	TCGA
MB-0346	SHQ1	cna	hetloss	METABRIC
MB-2827	SHQ1	cna	hetloss	METABRIC
MB-5465	SHQ1	cna	hetloss	METABRIC
MB-6098	SHQ1	cna	hetloss	METABRIC
MB-7048	SHQ1	cna	hetloss	METABRIC
MB-0420	SHQ1	cna	hetloss	METABRIC
MTS-T0064	SHQ1	cna	gain	METABRIC
PD10014	SHQ1	cna	hetloss	BASIS
PD11327	SHQ1	cna	homdel	BASIS
PD11742	SHQ1	cna	hetloss	BASIS
PD13296	SHQ1	cna	hetloss	BASIS
PD13299	SHQ1	cna	gain	BASIS
PD13771	SHQ1	cna	hetloss	BASIS
PD23561	SHQ1	cna	hetloss	BASIS
PD23574	SHQ1	cna	gain	BASIS
PD23578	SHQ1	cna	hetloss	BASIS
PD24202	SHQ1	cna	hetloss	BASIS
PD24206	SHQ1	cna	hetloss	BASIS
PD24337	SHQ1	cna	hetloss	BASIS
PD4107	SHQ1	cna	gain	BASIS
PD5945	SHQ1	cna	gain	BASIS
PD6406	SHQ1	cna	hetloss	BASIS
PD6731	SHQ1	cna	hetloss	BASIS
PD7067	SHQ1	cna	gain	BASIS
PD9585	SHQ1	cna	hetloss	BASIS
TCGA-A2-A25B	SMAD2	cna	hetloss	TCGA
TCGA-AN-A0XU	SMAD2	cna	hetloss	TCGA
TCGA-AO-A0JL	SMAD2	cna	gain	TCGA
TCGA-BH-A0AW	SMAD2	cna	hetloss	TCGA
TCGA-BH-A0C0	SMAD2	cna	gain	TCGA
TCGA-C8-A12L	SMAD2	cna	gain	TCGA
TCGA-D8-A27M	SMAD2	cna	hetloss	TCGA
TCGA-E2-A1L7	SMAD2	cna	gain	TCGA
TCGA-LL-A5YP	SMAD2	cna	hetloss	TCGA
MB-0346	SMAD2	cna	hetloss	METABRIC
MB-0420	SMAD2	cna	hetloss	METABRIC
PD10014	SMAD2	cna	hetloss	BASIS
PD11327	SMAD2	cna	gain	BASIS
PD11742	SMAD2	cna	hetloss	BASIS
PD13296	SMAD2	cna	hetloss	BASIS
PD13297	SMAD2	cna	gain	BASIS
PD13299	SMAD2	cna	gain	BASIS
PD23574	SMAD2	cna	hetloss	BASIS
PD24186	SMAD2	cna	gain	BASIS
PD24202	SMAD2	cna	hetloss	BASIS
PD24337	SMAD2	cna	gain	BASIS

PD3905	SMAD2	cna	gain	BASIS
PD4006	SMAD2	cna	gain	BASIS
PD4107	SMAD2	cna	gain	BASIS
PD4826	SMAD2	cna	gain	BASIS
PD4967	SMAD2	cna	hetloss	BASIS
PD5930	SMAD2	cna	gain	BASIS
PD5945	SMAD2	cna	gain	BASIS
PD5948	SMAD2	cna	hetloss	BASIS
PD6406	SMAD2	cna	gain	BASIS
PD7215	SMAD2	cna	gain	BASIS
PD8980	SMAD2	cna	hetloss	BASIS
PD9004	SMAD2	cna	hetloss	BASIS
PD9702	SMAD2	cna	gain	BASIS
TCGA-AN-A0XU	SNX29	cna	gain	TCGA
TCGA-AO-A0JL	SNX29	cna	gain	TCGA
TCGA-BH-A0AW	SNX29	cna	gain	TCGA
TCGA-BH-A0C0	SNX29	cna	gain	TCGA
TCGA-C8-A12L	SNX29	cna	gain	TCGA
TCGA-E2-A1L7	SNX29	cna	hetloss	TCGA
TCGA-LL-A5YP	SNX29	cna	gain	TCGA
MB-0346	SNX29	cna	gain	METABRIC
MB-6060	SNX29	cna	gain	METABRIC
MB-6098	SNX29	cna	hetloss	METABRIC
MB-6271	SNX29	cna	gain	METABRIC
MB-7032	SNX29	cna	gain	METABRIC
MB-7038	SNX29	cna	hetloss	METABRIC
MB-0420	SNX29	cna	hetloss	METABRIC
PD10014	SNX29	cna	hetloss	BASIS
PD11327	SNX29	cna	hetloss	BASIS
PD11742	SNX29	cna	gain	BASIS
PD13296	SNX29	cna	gain	BASIS
PD13297	SNX29	cna	hetloss	BASIS
PD13299	SNX29	cna	gain	BASIS
PD22355	SNX29	cna	homdel	BASIS
PD24202	SNX29	cna	hetloss	BASIS
PD24206	SNX29	cna	gain	BASIS
PD3905	SNX29	cna	gain	BASIS
PD4005	SNX29	cna	gain	BASIS
PD4107	SNX29	cna	gain	BASIS
PD4826	SNX29	cna	gain	BASIS
PD5935	SNX29	cna	gain	BASIS
PD5945	SNX29	cna	gain	BASIS
PD5948	SNX29	cna	gain	BASIS
PD7067	SNX29	cna	gain	BASIS
PD7215	SNX29	cna	amp	BASIS
PD9004	SNX29	cna	gain	BASIS
PD9585	SNX29	cna	hetloss	BASIS

TCGA-A2-A25B	SOCS2	cna	gain	TCGA
TCGA-AO-A0JL	SOCS2	cna	hetloss	TCGA
TCGA-BH-A0C0	SOCS2	cna	hetloss	TCGA
TCGA-BH-A18R	SOCS2	cna	gain	TCGA
TCGA-BH-A1FU	SOCS2	cna	hetloss	TCGA
TCGA-C8-A12L	SOCS2	cna	gain	TCGA
TCGA-E2-A1L7	SOCS2	cna	hetloss	TCGA
TCGA-LL-A5YP	SOCS2	cna	hetloss	TCGA
MB-0346	SOCS2	cna	gain	METABRIC
MB-2827	SOCS2	cna	hetloss	METABRIC
MB-5070	SOCS2	cna	hetloss	METABRIC
MB-5107	SOCS2	cna	gain	METABRIC
MB-5465	SOCS2	cna	gain	METABRIC
MB-6098	SOCS2	cna	hetloss	METABRIC
PD10014	SOCS2	cna	gain	BASIS
PD11327	SOCS2	cna	homdel	BASIS
PD13296	SOCS2	cna	hetloss	BASIS
PD22355	SOCS2	cna	hetloss	BASIS
PD23574	SOCS2	cna	hetloss	BASIS
PD24337	SOCS2	cna	gain	BASIS
PD3890	SOCS2	cna	hetloss	BASIS
PD4005	SOCS2	cna	hetloss	BASIS
PD4006	SOCS2	cna	gain	BASIS
PD4107	SOCS2	cna	gain	BASIS
PD4826	SOCS2	cna	gain	BASIS
PD5945	SOCS2	cna	gain	BASIS
PD6406	SOCS2	cna	hetloss	BASIS
PD6413	SOCS2	cna	hetloss	BASIS
PD6731	SOCS2	cna	hetloss	BASIS
PD7067	SOCS2	cna	gain	BASIS
PD7215	SOCS2	cna	hetloss	BASIS
PD8621	SOCS2	cna	hetloss	BASIS
PD8980	SOCS2	cna	hetloss	BASIS
PD9585	SOCS2	cna	hetloss	BASIS
TCGA-A2-A25B	SOX4	cna	gain	TCGA
TCGA-AN-A0XU	SOX4	cna	amp	TCGA
TCGA-AO-A0JL	SOX4	cna	gain	TCGA
TCGA-BH-A0AW	SOX4	cna	gain	TCGA
TCGA-BH-A0C0	SOX4	cna	hetloss	TCGA
TCGA-BH-A18R	SOX4	cna	homdel	TCGA
TCGA-C8-A12L	SOX4	cna	hetloss	TCGA
TCGA-E2-A1L7	SOX4	cna	gain	TCGA
MB-5070	SOX4	cna	amp	METABRIC
MB-5465	SOX4	cna	gain	METABRIC
MB-6060	SOX4	cna	gain	METABRIC
MB-7032	SOX4	cna	gain	METABRIC
PD11327	SOX4	cna	amp	BASIS

PD13296	SOX4	cna	gain	BASIS
PD13299	SOX4	cna	amp	BASIS
PD22355	SOX4	cna	gain	BASIS
PD23562	SOX4	cna	gain	BASIS
PD23574	SOX4	cna	gain	BASIS
PD24186	SOX4	cna	amp	BASIS
PD24202	SOX4	cna	gain	BASIS
PD24206	SOX4	cna	gain	BASIS
PD3905	SOX4	cna	amp	BASIS
PD4005	SOX4	cna	gain	BASIS
PD4006	SOX4	cna	gain	BASIS
PD4107	SOX4	cna	gain	BASIS
PD4826	SOX4	cna	gain	BASIS
PD5935	SOX4	cna	gain	BASIS
PD5945	SOX4	cna	amp	BASIS
PD5948	SOX4	cna	amp	BASIS
PD6731	SOX4	cna	hetloss	BASIS
PD7067	SOX4	cna	amp	BASIS
PD7215	SOX4	cna	gain	BASIS
PD8621	SOX4	cna	amp	BASIS
PD9702	SOX4	cna	gain	BASIS
TCGA-AN-A0XU	STAT4	cna	gain	TCGA
TCGA-AO-A0JL	STAT4	cna	gain	TCGA
TCGA-BH-A0AW	STAT4	cna	gain	TCGA
TCGA-C8-A12L	STAT4	cna	gain	TCGA
TCGA-D8-A27M	STAT4	cna	gain	TCGA
TCGA-LL-A5YP	STAT4	cna	gain	TCGA
MB-2827	STAT4	cna	hetloss	METABRIC
MB-5465	STAT4	cna	hetloss	METABRIC
MB-6060	STAT4	cna	gain	METABRIC
MB-6098	STAT4	cna	hetloss	METABRIC
MB-6271	STAT4	cna	hetloss	METABRIC
MB-7032	STAT4	cna	gain	METABRIC
MB-0420	STAT4	cna	gain	METABRIC
PD10014	STAT4	cna	gain	BASIS
PD11327	STAT4	cna	gain	BASIS
PD13297	STAT4	cna	hetloss	BASIS
PD13299	STAT4	cna	gain	BASIS
PD22355	STAT4	cna	hetloss	BASIS
PD23578	STAT4	cna	gain	BASIS
PD24186	STAT4	cna	gain	BASIS
PD24206	STAT4	cna	gain	BASIS
PD3890	STAT4	cna	hetloss	BASIS
PD3905	STAT4	cna	gain	BASIS
PD4107	STAT4	cna	gain	BASIS
PD4967	STAT4	cna	hetloss	BASIS
PD5935	STAT4	cna	gain	BASIS

PD5945	STAT4	cna	amp	BASIS
PD5948	STAT4	cna	gain	BASIS
PD6413	STAT4	cna	hetloss	BASIS
PD7067	STAT4	cna	amp	BASIS
PD7215	STAT4	cna	gain	BASIS
PD9004	STAT4	cna	gain	BASIS
PD9585	STAT4	cna	hetloss	BASIS
PD9702	STAT4	cna	gain	BASIS
TCGA-A2-A25B	TAL2	cna	gain	TCGA
TCGA-AN-A0XU	TAL2	cna	hetloss	TCGA
TCGA-AO-A0JL	TAL2	cna	hetloss	TCGA
TCGA-BH-A1FU	TAL2	cna	hetloss	TCGA
TCGA-C8-A12L	TAL2	cna	hetloss	TCGA
TCGA-E2-A1L7	TAL2	cna	hetloss	TCGA
TCGA-E9-A1NC	TAL2	cna	hetloss	TCGA
TCGA-LL-A5YP	TAL2	cna	hetloss	TCGA
MB-0346	TAL2	cna	hetloss	METABRIC
MB-2827	TAL2	cna	hetloss	METABRIC
MB-5070	TAL2	cna	hetloss	METABRIC
MB-5107	TAL2	cna	hetloss	METABRIC
MB-6098	TAL2	cna	hetloss	METABRIC
MB-7038	TAL2	cna	hetloss	METABRIC
MB-0420	TAL2	cna	hetloss	METABRIC
PD11327	TAL2	cna	gain	BASIS
PD13296	TAL2	cna	hetloss	BASIS
PD23562	TAL2	cna	gain	BASIS
PD23578	TAL2	cna	hetloss	BASIS
PD24186	TAL2	cna	amp	BASIS
PD24202	TAL2	cna	hetloss	BASIS
PD24206	TAL2	cna	gain	BASIS
PD3905	TAL2	cna	gain	BASIS
PD4005	TAL2	cna	hetloss	BASIS
PD4006	TAL2	cna	gain	BASIS
PD4107	TAL2	cna	hetloss	BASIS
PD5945	TAL2	cna	amp	BASIS
PD6406	TAL2	cna	hetloss	BASIS
PD6731	TAL2	cna	hetloss	BASIS
PD7215	TAL2	cna	gain	BASIS
PD8621	TAL2	cna	gain	BASIS
PD8980	TAL2	cna	gain	BASIS
PD9004	TAL2	cna	gain	BASIS
PD9585	TAL2	cna	hetloss	BASIS
TCGA-A2-A25B	TBK1	cna	gain	TCGA
TCGA-AO-A0JL	TBK1	cna	hetloss	TCGA
TCGA-BH-A18R	TBK1	cna	gain	TCGA
TCGA-BH-A1FU	TBK1	cna	hetloss	TCGA
TCGA-C8-A12L	TBK1	cna	gain	TCGA

TCGA-E2-A1L7	TBK1	cna	hetloss	TCGA
TCGA-LL-A5YP	TBK1	cna	hetloss	TCGA
MB-0346	TBK1	cna	gain	METABRIC
MB-2827	TBK1	cna	hetloss	METABRIC
MB-5107	TBK1	cna	gain	METABRIC
MB-6060	TBK1	cna	gain	METABRIC
MB-6098	TBK1	cna	gain	METABRIC
MB-0420	TBK1	cna	hetloss	METABRIC
MTS-T0064	TBK1	cna	gain	METABRIC
PD10014	TBK1	cna	gain	BASIS
PD11327	TBK1	cna	homdel	BASIS
PD22355	TBK1	cna	hetloss	BASIS
PD23578	TBK1	cna	gain	BASIS
PD24186	TBK1	cna	gain	BASIS
PD24202	TBK1	cna	hetloss	BASIS
PD24206	TBK1	cna	gain	BASIS
PD24337	TBK1	cna	gain	BASIS
PD3890	TBK1	cna	hetloss	BASIS
PD3905	TBK1	cna	gain	BASIS
PD4107	TBK1	cna	gain	BASIS
PD4826	TBK1	cna	gain	BASIS
PD5945	TBK1	cna	gain	BASIS
PD6406	TBK1	cna	hetloss	BASIS
PD6413	TBK1	cna	hetloss	BASIS
PD6731	TBK1	cna	hetloss	BASIS
PD7067	TBK1	cna	gain	BASIS
PD8621	TBK1	cna	hetloss	BASIS
PD9585	TBK1	cna	hetloss	BASIS
PD9702	TBK1	cna	gain	BASIS
TCGA-A2-A25B	TGFBR1	cna	gain	TCGA
TCGA-AN-A0XU	TGFBR1	cna	hetloss	TCGA
TCGA-AO-A0JL	TGFBR1	cna	hetloss	TCGA
TCGA-BH-A1FU	TGFBR1	cna	hetloss	TCGA
TCGA-C8-A12L	TGFBR1	cna	hetloss	TCGA
TCGA-E2-A1L7	TGFBR1	cna	hetloss	TCGA
TCGA-E9-A1NC	TGFBR1	cna	hetloss	TCGA
TCGA-LL-A5YP	TGFBR1	cna	hetloss	TCGA
MB-0346	TGFBR1	cna	hetloss	METABRIC
MB-2827	TGFBR1	cna	hetloss	METABRIC
MB-5070	TGFBR1	cna	hetloss	METABRIC
MB-5107	TGFBR1	cna	hetloss	METABRIC
MB-6098	TGFBR1	cna	hetloss	METABRIC
MB-7038	TGFBR1	cna	hetloss	METABRIC
MB-0420	TGFBR1	cna	hetloss	METABRIC
PD11327	TGFBR1	cna	gain	BASIS
PD23562	TGFBR1	cna	gain	BASIS
PD23578	TGFBR1	cna	hetloss	BASIS

PD24186	TGFBR1	cna	amp	BASIS
PD24202	TGFBR1	cna	hetloss	BASIS
PD24206	TGFBR1	cna	gain	BASIS
PD3905	TGFBR1	cna	gain	BASIS
PD4005	TGFBR1	cna	gain	BASIS
PD4006	TGFBR1	cna	gain	BASIS
PD4107	TGFBR1	cna	hetloss	BASIS
PD5945	TGFBR1	cna	amp	BASIS
PD6406	TGFBR1	cna	hetloss	BASIS
PD6731	TGFBR1	cna	hetloss	BASIS
PD7067	TGFBR1	cna	gain	BASIS
PD7215	TGFBR1	cna	gain	BASIS
PD8621	TGFBR1	cna	gain	BASIS
PD8980	TGFBR1	cna	gain	BASIS
PD9004	TGFBR1	cna	gain	BASIS
PD9585	TGFBR1	cna	hetloss	BASIS
TCGA-AN-A0XU	TGFBR2	cna	hetloss	TCGA
TCGA-BH-A0C0	TGFBR2	cna	hetloss	TCGA
TCGA-BH-A18R	TGFBR2	cna	gain	TCGA
TCGA-BH-A1FU	TGFBR2	cna	hetloss	TCGA
TCGA-C8-A12L	TGFBR2	cna	hetloss	TCGA
TCGA-D8-A27M	TGFBR2	cna	hetloss	TCGA
TCGA-E9-A1NC	TGFBR2	cna	hetloss	TCGA
TCGA-LL-A5YP	TGFBR2	cna	amp	TCGA
MB-2827	TGFBR2	cna	hetloss	METABRIC
MB-7038	TGFBR2	cna	hetloss	METABRIC
MB-7048	TGFBR2	cna	hetloss	METABRIC
MB-0420	TGFBR2	cna	hetloss	METABRIC
PD10014	TGFBR2	cna	hetloss	BASIS
PD11327	TGFBR2	cna	gain	BASIS
PD11742	TGFBR2	cna	gain	BASIS
PD13296	TGFBR2	cna	gain	BASIS
PD13297	TGFBR2	cna	hetloss	BASIS
PD13299	TGFBR2	cna	hetloss	BASIS
PD13771	TGFBR2	cna	gain	BASIS
PD22355	TGFBR2	cna	hetloss	BASIS
PD23561	TGFBR2	cna	hetloss	BASIS
PD23574	TGFBR2	cna	gain	BASIS
PD24202	TGFBR2	cna	hetloss	BASIS
PD24206	TGFBR2	cna	gain	BASIS
PD24337	TGFBR2	cna	hetloss	BASIS
PD3905	TGFBR2	cna	gain	BASIS
PD4006	TGFBR2	cna	hetloss	BASIS
PD4107	TGFBR2	cna	hetloss	BASIS
PD4826	TGFBR2	cna	gain	BASIS
PD5945	TGFBR2	cna	gain	BASIS
PD6406	TGFBR2	cna	hetloss	BASIS

PD6731	TGFBR2	cna	hetloss	BASIS
PD8980	TGFBR2	cna	gain	BASIS
PD9585	TGFBR2	cna	hetloss	BASIS
TCGA-A2-A25B	TMEM30A	cna	gain	TCGA
TCGA-AO-A0JL	TMEM30A	cna	hetloss	TCGA
TCGA-BH-A0C0	TMEM30A	cna	gain	TCGA
TCGA-C8-A12L	TMEM30A	cna	gain	TCGA
MB-0346	TMEM30A	cna	hetloss	METABRIC
MB-5070	TMEM30A	cna	hetloss	METABRIC
MB-5107	TMEM30A	cna	hetloss	METABRIC
MB-6098	TMEM30A	cna	hetloss	METABRIC
MB-7032	TMEM30A	cna	hetloss	METABRIC
MB-7038	TMEM30A	cna	amp	METABRIC
MB-7048	TMEM30A	cna	hetloss	METABRIC
MB-0420	TMEM30A	cna	gain	METABRIC
PD11327	TMEM30A	cna	gain	BASIS
PD13299	TMEM30A	cna	gain	BASIS
PD13771	TMEM30A	cna	hetloss	BASIS
PD23562	TMEM30A	cna	gain	BASIS
PD23574	TMEM30A	cna	gain	BASIS
PD24186	TMEM30A	cna	gain	BASIS
PD24206	TMEM30A	cna	gain	BASIS
PD24337	TMEM30A	cna	hetloss	BASIS
PD3905	TMEM30A	cna	gain	BASIS
PD4006	TMEM30A	cna	gain	BASIS
PD5930	TMEM30A	cna	hetloss	BASIS
PD5935	TMEM30A	cna	amp	BASIS
PD5945	TMEM30A	cna	amp	BASIS
PD5948	TMEM30A	cna	gain	BASIS
PD6731	TMEM30A	cna	hetloss	BASIS
PD7067	TMEM30A	cna	gain	BASIS
PD7215	TMEM30A	cna	gain	BASIS
PD8621	TMEM30A	cna	gain	BASIS
PD8980	TMEM30A	cna	hetloss	BASIS
PD9004	TMEM30A	cna	gain	BASIS
PD9585	TMEM30A	cna	hetloss	BASIS
PD9702	TMEM30A	cna	gain	BASIS
TCGA-AO-A0JL	TNFAIP6	cna	gain	TCGA
TCGA-D8-A27M	TNFAIP6	cna	gain	TCGA
TCGA-E9-A1NC	TNFAIP6	cna	gain	TCGA
TCGA-LL-A5YP	TNFAIP6	cna	gain	TCGA
MB-2827	TNFAIP6	cna	hetloss	METABRIC
MB-5107	TNFAIP6	cna	hetloss	METABRIC
MB-6098	TNFAIP6	cna	hetloss	METABRIC
MB-6271	TNFAIP6	cna	hetloss	METABRIC
MB-7032	TNFAIP6	cna	gain	METABRIC
PD11327	TNFAIP6	cna	gain	BASIS

PD11742	TNFAIP6	cna	hetloss	BASIS
PD13297	TNFAIP6	cna	hetloss	BASIS
PD13299	TNFAIP6	cna	gain	BASIS
PD22355	TNFAIP6	cna	hetloss	BASIS
PD23574	TNFAIP6	cna	gain	BASIS
PD23578	TNFAIP6	cna	hetloss	BASIS
PD24186	TNFAIP6	cna	gain	BASIS
PD24202	TNFAIP6	cna	hetloss	BASIS
PD24206	TNFAIP6	cna	gain	BASIS
PD3890	TNFAIP6	cna	hetloss	BASIS
PD3905	TNFAIP6	cna	gain	BASIS
PD4005	TNFAIP6	cna	hetloss	BASIS
PD4006	TNFAIP6	cna	hetloss	BASIS
PD4107	TNFAIP6	cna	gain	BASIS
PD4967	TNFAIP6	cna	hetloss	BASIS
PD5930	TNFAIP6	cna	hetloss	BASIS
PD5935	TNFAIP6	cna	gain	BASIS
PD5948	TNFAIP6	cna	gain	BASIS
PD6406	TNFAIP6	cna	hetloss	BASIS
PD6413	TNFAIP6	cna	hetloss	BASIS
PD7067	TNFAIP6	cna	gain	BASIS
PD7215	TNFAIP6	cna	gain	BASIS
PD8621	TNFAIP6	cna	hetloss	BASIS
PD9702	TNFAIP6	cna	gain	BASIS
TCGA-AN-A0XU	TNFSF9	cna	hetloss	TCGA
TCGA-AO-A0JL	TNFSF9	cna	gain	TCGA
TCGA-BH-A0C0	TNFSF9	cna	gain	TCGA
TCGA-BH-A1FU	TNFSF9	cna	hetloss	TCGA
TCGA-C8-A12L	TNFSF9	cna	hetloss	TCGA
TCGA-D8-A27M	TNFSF9	cna	hetloss	TCGA
TCGA-EW-A1OX	TNFSF9	cna	hetloss	TCGA
TCGA-LL-A5YP	TNFSF9	cna	hetloss	TCGA
MB-0346	TNFSF9	cna	gain	METABRIC
MB-2827	TNFSF9	cna	hetloss	METABRIC
MB-5070	TNFSF9	cna	hetloss	METABRIC
MB-5465	TNFSF9	cna	gain	METABRIC
MB-6098	TNFSF9	cna	hetloss	METABRIC
MB-7038	TNFSF9	cna	hetloss	METABRIC
MB-0420	TNFSF9	cna	hetloss	METABRIC
PD10014	TNFSF9	cna	hetloss	BASIS
PD11327	TNFSF9	cna	homdel	BASIS
PD14442	TNFSF9	cna	hetloss	BASIS
PD23574	TNFSF9	cna	gain	BASIS
PD24202	TNFSF9	cna	gain	BASIS
PD24206	TNFSF9	cna	hetloss	BASIS
PD3905	TNFSF9	cna	gain	BASIS
PD4006	TNFSF9	cna	gain	BASIS

PD4107	TNFSF9	cna	gain	BASIS
PD4826	TNFSF9	cna	gain	BASIS
PD4967	TNFSF9	cna	homdel	BASIS
PD5930	TNFSF9	cna	gain	BASIS
PD5945	TNFSF9	cna	gain	BASIS
PD5948	TNFSF9	cna	gain	BASIS
PD7067	TNFSF9	cna	gain	BASIS
PD7215	TNFSF9	cna	amp	BASIS
PD8621	TNFSF9	cna	gain	BASIS
PD8980	TNFSF9	cna	gain	BASIS
PD9702	TNFSF9	cna	gain	BASIS
TCGA-AO-A0JL	TPRX1	cna	hetloss	TCGA
TCGA-BH-A0AW	TPRX1	cna	gain	TCGA
TCGA-BH-A0C0	TPRX1	cna	hetloss	TCGA
TCGA-BH-A1FU	TPRX1	cna	hetloss	TCGA
TCGA-D8-A27M	TPRX1	cna	hetloss	TCGA
TCGA-E2-A1L7	TPRX1	cna	hetloss	TCGA
TCGA-EW-A1OX	TPRX1	cna	hetloss	TCGA
TCGA-LL-A5YP	TPRX1	cna	gain	TCGA
MB-0346	TPRX1	cna	hetloss	METABRIC
MB-6098	TPRX1	cna	hetloss	METABRIC
MB-7048	TPRX1	cna	gain	METABRIC
PD11327	TPRX1	cna	gain	BASIS
PD13296	TPRX1	cna	hetloss	BASIS
PD13299	TPRX1	cna	hetloss	BASIS
PD13771	TPRX1	cna	hetloss	BASIS
PD23562	TPRX1	cna	gain	BASIS
PD23574	TPRX1	cna	gain	BASIS
PD24186	TPRX1	cna	gain	BASIS
PD24206	TPRX1	cna	hetloss	BASIS
PD24337	TPRX1	cna	hetloss	BASIS
PD3890	TPRX1	cna	hetloss	BASIS
PD3905	TPRX1	cna	gain	BASIS
PD4005	TPRX1	cna	hetloss	BASIS
PD4006	TPRX1	cna	gain	BASIS
PD4826	TPRX1	cna	gain	BASIS
PD5935	TPRX1	cna	gain	BASIS
PD5945	TPRX1	cna	gain	BASIS
PD5948	TPRX1	cna	gain	BASIS
PD6406	TPRX1	cna	gain	BASIS
PD7067	TPRX1	cna	gain	BASIS
PD7215	TPRX1	cna	gain	BASIS
PD8621	TPRX1	cna	gain	BASIS
PD8980	TPRX1	cna	hetloss	BASIS
PD9585	TPRX1	cna	gain	BASIS
TCGA-AO-A0JL	TYK2	cna	gain	TCGA
TCGA-BH-A0C0	TYK2	cna	gain	TCGA

TCGA-BH-A1FU	TYK2	cna	hetloss	TCGA
TCGA-C8-A12L	TYK2	cna	amp	TCGA
TCGA-D8-A27M	TYK2	cna	hetloss	TCGA
TCGA-EW-A1OX	TYK2	cna	hetloss	TCGA
TCGA-LL-A5YP	TYK2	cna	hetloss	TCGA
MB-0346	TYK2	cna	hetloss	METABRIC
MB-2827	TYK2	cna	hetloss	METABRIC
MB-5070	TYK2	cna	hetloss	METABRIC
MB-5465	TYK2	cna	gain	METABRIC
MB-6098	TYK2	cna	hetloss	METABRIC
MB-7038	TYK2	cna	hetloss	METABRIC
MB-0420	TYK2	cna	hetloss	METABRIC
PD11327	TYK2	cna	homdel	BASIS
PD13771	TYK2	cna	gain	BASIS
PD14442	TYK2	cna	hetloss	BASIS
PD23562	TYK2	cna	gain	BASIS
PD23574	TYK2	cna	gain	BASIS
PD24206	TYK2	cna	homdel	BASIS
PD3905	TYK2	cna	gain	BASIS
PD4006	TYK2	cna	gain	BASIS
PD4107	TYK2	cna	gain	BASIS
PD4826	TYK2	cna	gain	BASIS
PD4967	TYK2	cna	hetloss	BASIS
PD5930	TYK2	cna	gain	BASIS
PD5935	TYK2	cna	gain	BASIS
PD5945	TYK2	cna	amp	BASIS
PD5948	TYK2	cna	gain	BASIS
PD6406	TYK2	cna	amp	BASIS
PD7067	TYK2	cna	gain	BASIS
PD7215	TYK2	cna	amp	BASIS
PD8621	TYK2	cna	gain	BASIS
PD9702	TYK2	cna	gain	BASIS
TCGA-AO-A0JL	U2AF2	cna	hetloss	TCGA
TCGA-BH-A0AW	U2AF2	cna	gain	TCGA
TCGA-BH-A0C0	U2AF2	cna	gain	TCGA
TCGA-BH-A1FU	U2AF2	cna	hetloss	TCGA
TCGA-C8-A12L	U2AF2	cna	gain	TCGA
TCGA-D8-A27M	U2AF2	cna	hetloss	TCGA
TCGA-EW-A1OX	U2AF2	cna	hetloss	TCGA
TCGA-LL-A5YP	U2AF2	cna	gain	TCGA
MB-0346	U2AF2	cna	hetloss	METABRIC
MB-2827	U2AF2	cna	hetloss	METABRIC
MB-6098	U2AF2	cna	hetloss	METABRIC
MB-7048	U2AF2	cna	gain	METABRIC
PD11327	U2AF2	cna	gain	BASIS
PD13296	U2AF2	cna	gain	BASIS
PD13299	U2AF2	cna	gain	BASIS

PD13771	U2AF2	cna	hetloss	BASIS
PD23562	U2AF2	cna	gain	BASIS
PD23574	U2AF2	cna	gain	BASIS
PD24186	U2AF2	cna	gain	BASIS
PD24206	U2AF2	cna	hetloss	BASIS
PD3890	U2AF2	cna	hetloss	BASIS
PD3905	U2AF2	cna	gain	BASIS
PD4006	U2AF2	cna	gain	BASIS
PD4107	U2AF2	cna	gain	BASIS
PD4826	U2AF2	cna	gain	BASIS
PD5935	U2AF2	cna	gain	BASIS
PD5945	U2AF2	cna	gain	BASIS
PD5948	U2AF2	cna	gain	BASIS
PD6406	U2AF2	cna	gain	BASIS
PD7067	U2AF2	cna	gain	BASIS
PD7215	U2AF2	cna	gain	BASIS
PD8621	U2AF2	cna	gain	BASIS
PD8980	U2AF2	cna	hetloss	BASIS
PD9585	U2AF2	cna	gain	BASIS
TCGA-A2-A25B	XPA	cna	gain	TCGA
TCGA-AN-A0XU	XPA	cna	hetloss	TCGA
TCGA-AO-A0JL	XPA	cna	hetloss	TCGA
TCGA-BH-A1FU	XPA	cna	hetloss	TCGA
TCGA-C8-A12L	XPA	cna	hetloss	TCGA
TCGA-E2-A1L7	XPA	cna	hetloss	TCGA
TCGA-E9-A1NC	XPA	cna	hetloss	TCGA
TCGA-LL-A5YP	XPA	cna	hetloss	TCGA
MB-0346	XPA	cna	hetloss	METABRIC
MB-2827	XPA	cna	hetloss	METABRIC
MB-5070	XPA	cna	hetloss	METABRIC
MB-5107	XPA	cna	hetloss	METABRIC
MB-6098	XPA	cna	hetloss	METABRIC
MB-7038	XPA	cna	hetloss	METABRIC
PD11327	XPA	cna	gain	BASIS
PD13296	XPA	cna	gain	BASIS
PD23562	XPA	cna	gain	BASIS
PD23578	XPA	cna	hetloss	BASIS
PD24186	XPA	cna	amp	BASIS
PD24202	XPA	cna	hetloss	BASIS
PD24206	XPA	cna	gain	BASIS
PD3905	XPA	cna	gain	BASIS
PD4005	XPA	cna	gain	BASIS
PD4006	XPA	cna	gain	BASIS
PD4107	XPA	cna	hetloss	BASIS
PD5945	XPA	cna	amp	BASIS
PD6406	XPA	cna	hetloss	BASIS
PD6731	XPA	cna	hetloss	BASIS

PD7067	XPA	cna	gain	BASIS
PD7215	XPA	cna	gain	BASIS
PD8621	XPA	cna	gain	BASIS
PD8980	XPA	cna	gain	BASIS
PD9004	XPA	cna	gain	BASIS
PD9585	XPA	cna	hetloss	BASIS
TCGA-BH-A0C0	XPC	cna	gain	TCGA
TCGA-BH-A18R	XPC	cna	gain	TCGA
TCGA-BH-A1FU	XPC	cna	hetloss	TCGA
TCGA-D8-A27M	XPC	cna	hetloss	TCGA
TCGA-E9-A1NC	XPC	cna	hetloss	TCGA
TCGA-LL-A5YP	XPC	cna	amp	TCGA
MB-0346	XPC	cna	gain	METABRIC
MB-5070	XPC	cna	hetloss	METABRIC
MB-7038	XPC	cna	hetloss	METABRIC
MB-7048	XPC	cna	hetloss	METABRIC
MB-0420	XPC	cna	hetloss	METABRIC
PD10014	XPC	cna	hetloss	BASIS
PD11742	XPC	cna	gain	BASIS
PD13296	XPC	cna	gain	BASIS
PD13299	XPC	cna	gain	BASIS
PD13771	XPC	cna	gain	BASIS
PD23561	XPC	cna	hetloss	BASIS
PD23562	XPC	cna	gain	BASIS
PD23574	XPC	cna	gain	BASIS
PD24206	XPC	cna	gain	BASIS
PD24337	XPC	cna	hetloss	BASIS
PD3905	XPC	cna	gain	BASIS
PD4006	XPC	cna	gain	BASIS
PD4826	XPC	cna	gain	BASIS
PD5945	XPC	cna	amp	BASIS
PD5948	XPC	cna	gain	BASIS
PD6406	XPC	cna	hetloss	BASIS
PD6731	XPC	cna	hetloss	BASIS
PD7067	XPC	cna	amp	BASIS
PD7215	XPC	cna	gain	BASIS
PD8980	XPC	cna	gain	BASIS
PD9004	XPC	cna	gain	BASIS
PD9585	XPC	cna	hetloss	BASIS
PD9702	XPC	cna	gain	BASIS
TCGA-A2-A25B	ZFP36L1	cna	hetloss	TCGA
TCGA-AO-A0JL	ZFP36L1	cna	hetloss	TCGA
TCGA-BH-A0AW	ZFP36L1	cna	hetloss	TCGA
TCGA-BH-A1FU	ZFP36L1	cna	gain	TCGA
TCGA-C8-A12L	ZFP36L1	cna	hetloss	TCGA
TCGA-D8-A27M	ZFP36L1	cna	hetloss	TCGA
TCGA-E2-A1L7	ZFP36L1	cna	hetloss	TCGA

TCGA-LL-A5YP	ZFP36L1	cna	hetloss	TCGA
MB-2827	ZFP36L1	cna	hetloss	METABRIC
MB-5070	ZFP36L1	cna	hetloss	METABRIC
MB-6098	ZFP36L1	cna	hetloss	METABRIC
MB-7038	ZFP36L1	cna	hetloss	METABRIC
MTS-T0064	ZFP36L1	cna	gain	METABRIC
PD11327	ZFP36L1	cna	amp	BASIS
PD11742	ZFP36L1	cna	hetloss	BASIS
PD13296	ZFP36L1	cna	gain	BASIS
PD13297	ZFP36L1	cna	hetloss	BASIS
PD13771	ZFP36L1	cna	hetloss	BASIS
PD14442	ZFP36L1	cna	hetloss	BASIS
PD23578	ZFP36L1	cna	hetloss	BASIS
PD24202	ZFP36L1	cna	hetloss	BASIS
PD24206	ZFP36L1	cna	gain	BASIS
PD24337	ZFP36L1	cna	hetloss	BASIS
PD3890	ZFP36L1	cna	hetloss	BASIS
PD3905	ZFP36L1	cna	gain	BASIS
PD4005	ZFP36L1	cna	hetloss	BASIS
PD4006	ZFP36L1	cna	gain	BASIS
PD5945	ZFP36L1	cna	gain	BASIS
PD6406	ZFP36L1	cna	hetloss	BASIS
PD7067	ZFP36L1	cna	gain	BASIS
PD7215	ZFP36L1	cna	hetloss	BASIS
PD8980	ZFP36L1	cna	gain	BASIS
PD9004	ZFP36L1	cna	hetloss	BASIS
PD9702	ZFP36L1	cna	gain	BASIS
TCGA-AO-A0JL	ZNF132	cna	hetloss	TCGA
TCGA-BH-A0AW	ZNF132	cna	gain	TCGA
TCGA-BH-A0C0	ZNF132	cna	gain	TCGA
TCGA-BH-A1FU	ZNF132	cna	hetloss	TCGA
TCGA-C8-A12L	ZNF132	cna	gain	TCGA
TCGA-D8-A27M	ZNF132	cna	gain	TCGA
TCGA-EW-A1OX	ZNF132	cna	hetloss	TCGA
TCGA-LL-A5YP	ZNF132	cna	gain	TCGA
MB-0346	ZNF132	cna	hetloss	METABRIC
MB-2827	ZNF132	cna	hetloss	METABRIC
MB-7048	ZNF132	cna	gain	METABRIC
MB-0420	ZNF132	cna	hetloss	METABRIC
MTS-T0064	ZNF132	cna	hetloss	METABRIC
PD11327	ZNF132	cna	hetloss	BASIS
PD13296	ZNF132	cna	gain	BASIS
PD13299	ZNF132	cna	gain	BASIS
PD13771	ZNF132	cna	hetloss	BASIS
PD23562	ZNF132	cna	hetloss	BASIS
PD23574	ZNF132	cna	gain	BASIS
PD24186	ZNF132	cna	gain	BASIS

PD24206	ZNF132	cna	hetloss	BASIS
PD3890	ZNF132	cna	hetloss	BASIS
PD3905	ZNF132	cna	gain	BASIS
PD4006	ZNF132	cna	gain	BASIS
PD4107	ZNF132	cna	gain	BASIS
PD4826	ZNF132	cna	gain	BASIS
PD5935	ZNF132	cna	gain	BASIS
PD5945	ZNF132	cna	gain	BASIS
PD5948	ZNF132	cna	gain	BASIS
PD6406	ZNF132	cna	gain	BASIS
PD7067	ZNF132	cna	gain	BASIS
PD7215	ZNF132	cna	gain	BASIS
PD8980	ZNF132	cna	hetloss	BASIS
PD9585	ZNF132	cna	gain	BASIS
TCGA-A2-A25B	ABL1	cna	gain	TCGA
TCGA-AN-A0XU	ABL1	cna	hetloss	TCGA
TCGA-AO-A0JL	ABL1	cna	hetloss	TCGA
TCGA-BH-A0C0	ABL1	cna	gain	TCGA
TCGA-C8-A12L	ABL1	cna	gain	TCGA
TCGA-E2-A1L7	ABL1	cna	gain	TCGA
TCGA-E9-A1NC	ABL1	cna	hetloss	TCGA
TCGA-LL-A5YP	ABL1	cna	hetloss	TCGA
MB-0346	ABL1	cna	hetloss	METABRIC
MB-5070	ABL1	cna	hetloss	METABRIC
MB-5107	ABL1	cna	gain	METABRIC
MB-7038	ABL1	cna	hetloss	METABRIC
MB-0420	ABL1	cna	hetloss	METABRIC
PD10014	ABL1	cna	gain	BASIS
PD11327	ABL1	cna	hetloss	BASIS
PD13299	ABL1	cna	gain	BASIS
PD22355	ABL1	cna	gain	BASIS
PD24186	ABL1	cna	gain	BASIS
PD24202	ABL1	cna	hetloss	BASIS
PD24337	ABL1	cna	hetloss	BASIS
PD3890	ABL1	cna	hetloss	BASIS
PD3905	ABL1	cna	gain	BASIS
PD4005	ABL1	cna	hetloss	BASIS
PD4006	ABL1	cna	gain	BASIS
PD4107	ABL1	cna	hetloss	BASIS
PD5945	ABL1	cna	gain	BASIS
PD5948	ABL1	cna	gain	BASIS
PD6406	ABL1	cna	hetloss	BASIS
PD6731	ABL1	cna	hetloss	BASIS
PD7067	ABL1	cna	gain	BASIS
PD7215	ABL1	cna	gain	BASIS
PD8621	ABL1	cna	gain	BASIS
PD9004	ABL1	cna	hetloss	BASIS

TCGA-AO-A0JL	ARHGAP35	cna	hetloss	TCGA
TCGA-BH-A0AW	ARHGAP35	cna	gain	TCGA
TCGA-BH-A0C0	ARHGAP35	cna	hetloss	TCGA
TCGA-BH-A1FU	ARHGAP35	cna	hetloss	TCGA
TCGA-D8-A27M	ARHGAP35	cna	hetloss	TCGA
TCGA-E2-A1L7	ARHGAP35	cna	hetloss	TCGA
TCGA-EW-A1OX	ARHGAP35	cna	hetloss	TCGA
TCGA-LL-A5YP	ARHGAP35	cna	gain	TCGA
MB-0346	ARHGAP35	cna	hetloss	METABRIC
MB-6098	ARHGAP35	cna	gain	METABRIC
PD11327	ARHGAP35	cna	gain	BASIS
PD13296	ARHGAP35	cna	hetloss	BASIS
PD13299	ARHGAP35	cna	hetloss	BASIS
PD13771	ARHGAP35	cna	hetloss	BASIS
PD23562	ARHGAP35	cna	gain	BASIS
PD23574	ARHGAP35	cna	gain	BASIS
PD24186	ARHGAP35	cna	gain	BASIS
PD24206	ARHGAP35	cna	hetloss	BASIS
PD24337	ARHGAP35	cna	hetloss	BASIS
PD3890	ARHGAP35	cna	hetloss	BASIS
PD3905	ARHGAP35	cna	gain	BASIS
PD4005	ARHGAP35	cna	hetloss	BASIS
PD4006	ARHGAP35	cna	gain	BASIS
PD4826	ARHGAP35	cna	gain	BASIS
PD5935	ARHGAP35	cna	gain	BASIS
PD5945	ARHGAP35	cna	gain	BASIS
PD5948	ARHGAP35	cna	gain	BASIS
PD6406	ARHGAP35	cna	gain	BASIS
PD7067	ARHGAP35	cna	gain	BASIS
PD7215	ARHGAP35	cna	gain	BASIS
PD8621	ARHGAP35	cna	gain	BASIS
PD8980	ARHGAP35	cna	hetloss	BASIS
PD9585	ARHGAP35	cna	gain	BASIS
TCGA-A2-A25B	ATP10A	cna	homdel	TCGA
TCGA-AO-A0JL	ATP10A	cna	hetloss	TCGA
TCGA-BH-A0C0	ATP10A	cna	hetloss	TCGA
TCGA-C8-A12L	ATP10A	cna	hetloss	TCGA
TCGA-D8-A27M	ATP10A	cna	hetloss	TCGA
TCGA-E2-A1L7	ATP10A	cna	hetloss	TCGA
MB-2827	ATP10A	cna	hetloss	METABRIC
MB-5070	ATP10A	cna	hetloss	METABRIC
MB-6098	ATP10A	cna	hetloss	METABRIC
MB-7038	ATP10A	cna	hetloss	METABRIC
MB-0420	ATP10A	cna	hetloss	METABRIC
PD11327	ATP10A	cna	hetloss	BASIS
PD11742	ATP10A	cna	hetloss	BASIS
PD13296	ATP10A	cna	gain	BASIS

PD13297	ATP10A	cna	hetloss	BASIS
PD13299	ATP10A	cna	gain	BASIS
PD23574	ATP10A	cna	hetloss	BASIS
PD23578	ATP10A	cna	hetloss	BASIS
PD24202	ATP10A	cna	hetloss	BASIS
PD24206	ATP10A	cna	gain	BASIS
PD3890	ATP10A	cna	hetloss	BASIS
PD3905	ATP10A	cna	gain	BASIS
PD4005	ATP10A	cna	hetloss	BASIS
PD5930	ATP10A	cna	hetloss	BASIS
PD5935	ATP10A	cna	gain	BASIS
PD5945	ATP10A	cna	amp	BASIS
PD5948	ATP10A	cna	hetloss	BASIS
PD6413	ATP10A	cna	hetloss	BASIS
PD7067	ATP10A	cna	gain	BASIS
PD7215	ATP10A	cna	gain	BASIS
PD8980	ATP10A	cna	hetloss	BASIS
PD9004	ATP10A	cna	gain	BASIS
PD9585	ATP10A	cna	hetloss	BASIS
TCGA-AO-A0JL	BBC3	cna	hetloss	TCGA
TCGA-BH-A0AW	BBC3	cna	gain	TCGA
TCGA-BH-A0C0	BBC3	cna	hetloss	TCGA
TCGA-BH-A1FU	BBC3	cna	hetloss	TCGA
TCGA-D8-A27M	BBC3	cna	hetloss	TCGA
TCGA-E2-A1L7	BBC3	cna	hetloss	TCGA
TCGA-EW-A1OX	BBC3	cna	hetloss	TCGA
TCGA-LL-A5YP	BBC3	cna	gain	TCGA
MB-0346	BBC3	cna	hetloss	METABRIC
MB-6098	BBC3	cna	gain	METABRIC
PD11327	BBC3	cna	gain	BASIS
PD13296	BBC3	cna	hetloss	BASIS
PD13299	BBC3	cna	hetloss	BASIS
PD13771	BBC3	cna	hetloss	BASIS
PD23562	BBC3	cna	gain	BASIS
PD23574	BBC3	cna	gain	BASIS
PD24186	BBC3	cna	gain	BASIS
PD24206	BBC3	cna	hetloss	BASIS
PD24337	BBC3	cna	hetloss	BASIS
PD3890	BBC3	cna	hetloss	BASIS
PD3905	BBC3	cna	gain	BASIS
PD4005	BBC3	cna	hetloss	BASIS
PD4006	BBC3	cna	gain	BASIS
PD4826	BBC3	cna	gain	BASIS
PD5935	BBC3	cna	gain	BASIS
PD5945	BBC3	cna	gain	BASIS
PD5948	BBC3	cna	gain	BASIS
PD6406	BBC3	cna	gain	BASIS

PD7067	BBC3	cna	gain	BASIS
PD7215	BBC3	cna	gain	BASIS
PD8621	BBC3	cna	gain	BASIS
PD8980	BBC3	cna	hetloss	BASIS
PD9585	BBC3	cna	gain	BASIS
TCGA-A2-A25B	BCL2L1	cna	gain	TCGA
TCGA-AO-A0JL	BCL2L1	cna	gain	TCGA
TCGA-BH-A0AW	BCL2L1	cna	gain	TCGA
TCGA-BH-A0C0	BCL2L1	cna	gain	TCGA
TCGA-C8-A12L	BCL2L1	cna	gain	TCGA
TCGA-D8-A27M	BCL2L1	cna	gain	TCGA
TCGA-E2-A1L7	BCL2L1	cna	gain	TCGA
TCGA-E9-A1NC	BCL2L1	cna	hetloss	TCGA
TCGA-LL-A5YP	BCL2L1	cna	gain	TCGA
MB-0346	BCL2L1	cna	gain	METABRIC
MB-5070	BCL2L1	cna	amp	METABRIC
MB-6060	BCL2L1	cna	gain	METABRIC
MB-7038	BCL2L1	cna	hetloss	METABRIC
P-0010758-T01-IM5	BCL2L1	cna	amp	MSK-IMPACT
PD11327	BCL2L1	cna	homdel	BASIS
PD13296	BCL2L1	cna	gain	BASIS
PD13297	BCL2L1	cna	gain	BASIS
PD13299	BCL2L1	cna	gain	BASIS
PD23574	BCL2L1	cna	gain	BASIS
PD23578	BCL2L1	cna	hetloss	BASIS
PD24186	BCL2L1	cna	gain	BASIS
PD24206	BCL2L1	cna	gain	BASIS
PD3905	BCL2L1	cna	gain	BASIS
PD4107	BCL2L1	cna	amp	BASIS
PD4826	BCL2L1	cna	gain	BASIS
PD5930	BCL2L1	cna	gain	BASIS
PD5935	BCL2L1	cna	gain	BASIS
PD5945	BCL2L1	cna	amp	BASIS
PD5948	BCL2L1	cna	gain	BASIS
PD7067	BCL2L1	cna	gain	BASIS
PD7215	BCL2L1	cna	gain	BASIS
PD9004	BCL2L1	cna	gain	BASIS
PD9702	BCL2L1	cna	gain	BASIS
TCGA-BH-A0C0	BTLA	cna	hetloss	TCGA
TCGA-BH-A18R	BTLA	cna	gain	TCGA
TCGA-BH-A1FU	BTLA	cna	hetloss	TCGA
TCGA-C8-A12L	BTLA	cna	hetloss	TCGA
TCGA-E2-A1L7	BTLA	cna	hetloss	TCGA
TCGA-LL-A5YP	BTLA	cna	gain	TCGA
MB-0346	BTLA	cna	hetloss	METABRIC
MB-5070	BTLA	cna	hetloss	METABRIC
MB-5107	BTLA	cna	gain	METABRIC

MB-5465	BTLA	cna	gain	METABRIC
MTS-T0064	BTLA	cna	hetloss	METABRIC
PD11327	BTLA	cna	gain	BASIS
PD13296	BTLA	cna	hetloss	BASIS
PD13299	BTLA	cna	gain	BASIS
PD23561	BTLA	cna	hetloss	BASIS
PD23574	BTLA	cna	gain	BASIS
PD23578	BTLA	cna	gain	BASIS
PD24186	BTLA	cna	gain	BASIS
PD24206	BTLA	cna	gain	BASIS
PD3905	BTLA	cna	gain	BASIS
PD4005	BTLA	cna	hetloss	BASIS
PD4006	BTLA	cna	hetloss	BASIS
PD4107	BTLA	cna	gain	BASIS
PD4826	BTLA	cna	gain	BASIS
PD5935	BTLA	cna	gain	BASIS
PD5945	BTLA	cna	gain	BASIS
PD5948	BTLA	cna	gain	BASIS
PD7067	BTLA	cna	gain	BASIS
PD7215	BTLA	cna	gain	BASIS
PD8621	BTLA	cna	gain	BASIS
PD8980	BTLA	cna	gain	BASIS
PD9004	BTLA	cna	gain	BASIS
PD9702	BTLA	cna	gain	BASIS
TCGA-AN-A0XU	CCNE1	cna	gain	TCGA
TCGA-AO-A0JL	CCNE1	cna	amp	TCGA
TCGA-BH-A0AW	CCNE1	cna	gain	TCGA
TCGA-BH-A0C0	CCNE1	cna	gain	TCGA
TCGA-BH-A1FU	CCNE1	cna	hetloss	TCGA
TCGA-C8-A12L	CCNE1	cna	gain	TCGA
TCGA-D8-A27M	CCNE1	cna	amp	TCGA
TCGA-E9-A1NC	CCNE1	cna	hetloss	TCGA
TCGA-EW-A1OX	CCNE1	cna	hetloss	TCGA
TCGA-LL-A5YP	CCNE1	cna	gain	TCGA
MB-0346	CCNE1	cna	hetloss	METABRIC
MB-6098	CCNE1	cna	gain	METABRIC
MB-7038	CCNE1	cna	gain	METABRIC
P-0009557-T01-IM5	CCNE1	cna	amp	MSK-IMPACT
PD10014	CCNE1	cna	hetloss	BASIS
PD11327	CCNE1	cna	gain	BASIS
PD13296	CCNE1	cna	gain	BASIS
PD13299	CCNE1	cna	gain	BASIS
PD23562	CCNE1	cna	gain	BASIS
PD23574	CCNE1	cna	amp	BASIS
PD24186	CCNE1	cna	gain	BASIS
PD3905	CCNE1	cna	gain	BASIS
PD4006	CCNE1	cna	gain	BASIS

PD4826	CCNE1	cna	gain	BASIS
PD4967	CCNE1	cna	gain	BASIS
PD5935	CCNE1	cna	gain	BASIS
PD5945	CCNE1	cna	amp	BASIS
PD5948	CCNE1	cna	gain	BASIS
PD7067	CCNE1	cna	gain	BASIS
PD7215	CCNE1	cna	gain	BASIS
PD8621	CCNE1	cna	gain	BASIS
PD8980	CCNE1	cna	hetloss	BASIS
PD9702	CCNE1	cna	gain	BASIS
TCGA-A2-A25B	CDKN2C	cna	gain	TCGA
TCGA-AN-A0XU	CDKN2C	cna	gain	TCGA
TCGA-AO-A0JL	CDKN2C	cna	hetloss	TCGA
TCGA-BH-A0AW	CDKN2C	cna	gain	TCGA
TCGA-BH-A0C0	CDKN2C	cna	gain	TCGA
TCGA-BH-A1FU	CDKN2C	cna	gain	TCGA
TCGA-D8-A27M	CDKN2C	cna	gain	TCGA
TCGA-E2-A1L7	CDKN2C	cna	gain	TCGA
TCGA-LL-A5YP	CDKN2C	cna	hetloss	TCGA
MB-0346	CDKN2C	cna	hetloss	METABRIC
MB-6060	CDKN2C	cna	hetloss	METABRIC
MB-7038	CDKN2C	cna	gain	METABRIC
MB-0420	CDKN2C	cna	hetloss	METABRIC
PD11327	CDKN2C	cna	hetloss	BASIS
PD11742	CDKN2C	cna	hetloss	BASIS
PD13299	CDKN2C	cna	gain	BASIS
PD24186	CDKN2C	cna	amp	BASIS
PD24206	CDKN2C	cna	gain	BASIS
PD3905	CDKN2C	cna	gain	BASIS
PD4005	CDKN2C	cna	hetloss	BASIS
PD4006	CDKN2C	cna	gain	BASIS
PD4107	CDKN2C	cna	gain	BASIS
PD4826	CDKN2C	cna	gain	BASIS
PD5930	CDKN2C	cna	gain	BASIS
PD5935	CDKN2C	cna	gain	BASIS
PD5945	CDKN2C	cna	amp	BASIS
PD5948	CDKN2C	cna	gain	BASIS
PD6413	CDKN2C	cna	hetloss	BASIS
PD7067	CDKN2C	cna	gain	BASIS
PD7215	CDKN2C	cna	gain	BASIS
PD8621	CDKN2C	cna	gain	BASIS
PD9004	CDKN2C	cna	gain	BASIS
PD9702	CDKN2C	cna	gain	BASIS
TCGA-AO-A0JL	CHN1	cna	gain	TCGA
TCGA-BH-A0AW	CHN1	cna	gain	TCGA
TCGA-D8-A27M	CHN1	cna	gain	TCGA
TCGA-LL-A5YP	CHN1	cna	gain	TCGA

MB-2827	CHN1	cna	hetloss	METABRIC
MB-5107	CHN1	cna	hetloss	METABRIC
MB-6098	CHN1	cna	hetloss	METABRIC
MB-6271	CHN1	cna	hetloss	METABRIC
MB-7032	CHN1	cna	gain	METABRIC
MB-7038	CHN1	cna	gain	METABRIC
MB-0420	CHN1	cna	gain	METABRIC
PD10014	CHN1	cna	gain	BASIS
PD11327	CHN1	cna	gain	BASIS
PD13299	CHN1	cna	gain	BASIS
PD22355	CHN1	cna	hetloss	BASIS
PD24186	CHN1	cna	gain	BASIS
PD24202	CHN1	cna	gain	BASIS
PD24206	CHN1	cna	gain	BASIS
PD3890	CHN1	cna	hetloss	BASIS
PD3905	CHN1	cna	gain	BASIS
PD4006	CHN1	cna	gain	BASIS
PD4107	CHN1	cna	gain	BASIS
PD4967	CHN1	cna	hetloss	BASIS
PD5930	CHN1	cna	hetloss	BASIS
PD5935	CHN1	cna	gain	BASIS
PD5945	CHN1	cna	amp	BASIS
PD5948	CHN1	cna	gain	BASIS
PD6413	CHN1	cna	hetloss	BASIS
PD7067	CHN1	cna	gain	BASIS
PD7215	CHN1	cna	gain	BASIS
PD9004	CHN1	cna	gain	BASIS
PD9585	CHN1	cna	hetloss	BASIS
PD9702	CHN1	cna	gain	BASIS
TCGA-AN-A0XU	CUL3	cna	hetloss	TCGA
TCGA-AO-A0JL	CUL3	cna	gain	TCGA
TCGA-BH-A0AW	CUL3	cna	hetloss	TCGA
TCGA-C8-A12L	CUL3	cna	hetloss	TCGA
TCGA-D8-A27M	CUL3	cna	gain	TCGA
TCGA-E2-A1L7	CUL3	cna	hetloss	TCGA
TCGA-E9-A1NC	CUL3	cna	hetloss	TCGA
MB-2827	CUL3	cna	hetloss	METABRIC
MB-6060	CUL3	cna	hetloss	METABRIC
MB-6098	CUL3	cna	hetloss	METABRIC
MB-6271	CUL3	cna	hetloss	METABRIC
MB-7038	CUL3	cna	gain	METABRIC
PD10014	CUL3	cna	gain	BASIS
PD11327	CUL3	cna	gain	BASIS
PD13296	CUL3	cna	hetloss	BASIS
PD13297	CUL3	cna	hetloss	BASIS
PD23578	CUL3	cna	hetloss	BASIS
PD24186	CUL3	cna	gain	BASIS

PD24337	CUL3	cna	hetloss	BASIS
PD3890	CUL3	cna	hetloss	BASIS
PD4005	CUL3	cna	gain	BASIS
PD4006	CUL3	cna	hetloss	BASIS
PD4967	CUL3	cna	hetloss	BASIS
PD5935	CUL3	cna	gain	BASIS
PD5945	CUL3	cna	gain	BASIS
PD5948	CUL3	cna	gain	BASIS
PD6406	CUL3	cna	hetloss	BASIS
PD6413	CUL3	cna	hetloss	BASIS
PD6731	CUL3	cna	hetloss	BASIS
PD7067	CUL3	cna	gain	BASIS
PD7215	CUL3	cna	gain	BASIS
PD8980	CUL3	cna	hetloss	BASIS
PD9585	CUL3	cna	hetloss	BASIS
TCGA-AO-A0JL	DNM2	cna	gain	TCGA
TCGA-BH-A0C0	DNM2	cna	gain	TCGA
TCGA-BH-A1FU	DNM2	cna	hetloss	TCGA
TCGA-C8-A12L	DNM2	cna	amp	TCGA
TCGA-D8-A27M	DNM2	cna	hetloss	TCGA
TCGA-EW-A1OX	DNM2	cna	hetloss	TCGA
TCGA-LL-A5YP	DNM2	cna	hetloss	TCGA
MB-0346	DNM2	cna	hetloss	METABRIC
MB-2827	DNM2	cna	hetloss	METABRIC
MB-5070	DNM2	cna	hetloss	METABRIC
MB-5465	DNM2	cna	gain	METABRIC
MB-6098	DNM2	cna	hetloss	METABRIC
MB-7038	DNM2	cna	hetloss	METABRIC
MB-0420	DNM2	cna	hetloss	METABRIC
PD11327	DNM2	cna	homdel	BASIS
PD13771	DNM2	cna	gain	BASIS
PD14442	DNM2	cna	hetloss	BASIS
PD23562	DNM2	cna	gain	BASIS
PD23574	DNM2	cna	gain	BASIS
PD24206	DNM2	cna	homdel	BASIS
PD3905	DNM2	cna	gain	BASIS
PD4006	DNM2	cna	gain	BASIS
PD4107	DNM2	cna	gain	BASIS
PD4826	DNM2	cna	gain	BASIS
PD4967	DNM2	cna	hetloss	BASIS
PD5930	DNM2	cna	gain	BASIS
PD5935	DNM2	cna	gain	BASIS
PD5945	DNM2	cna	amp	BASIS
PD5948	DNM2	cna	gain	BASIS
PD7067	DNM2	cna	gain	BASIS
PD7215	DNM2	cna	amp	BASIS
PD8621	DNM2	cna	gain	BASIS

PD9702	DNM2	cna	gain	BASIS
TCGA-AN-A0XU	ELP2	cna	hetloss	TCGA
TCGA-AO-A0JL	ELP2	cna	gain	TCGA
TCGA-BH-A0AW	ELP2	cna	hetloss	TCGA
TCGA-BH-A0C0	ELP2	cna	gain	TCGA
TCGA-C8-A12L	ELP2	cna	gain	TCGA
TCGA-D8-A27M	ELP2	cna	hetloss	TCGA
TCGA-E2-A1L7	ELP2	cna	gain	TCGA
TCGA-LL-A5YP	ELP2	cna	gain	TCGA
MB-0346	ELP2	cna	hetloss	METABRIC
PD10014	ELP2	cna	hetloss	BASIS
PD11327	ELP2	cna	gain	BASIS
PD13296	ELP2	cna	hetloss	BASIS
PD13297	ELP2	cna	gain	BASIS
PD13299	ELP2	cna	gain	BASIS
PD23574	ELP2	cna	hetloss	BASIS
PD23578	ELP2	cna	gain	BASIS
PD24186	ELP2	cna	gain	BASIS
PD24202	ELP2	cna	hetloss	BASIS
PD24206	ELP2	cna	gain	BASIS
PD24337	ELP2	cna	gain	BASIS
PD3905	ELP2	cna	gain	BASIS
PD4006	ELP2	cna	gain	BASIS
PD4107	ELP2	cna	gain	BASIS
PD4967	ELP2	cna	hetloss	BASIS
PD5930	ELP2	cna	gain	BASIS
PD5945	ELP2	cna	gain	BASIS
PD5948	ELP2	cna	gain	BASIS
PD6406	ELP2	cna	gain	BASIS
PD7067	ELP2	cna	gain	BASIS
PD7215	ELP2	cna	gain	BASIS
PD8980	ELP2	cna	hetloss	BASIS
PD9004	ELP2	cna	hetloss	BASIS
PD9702	ELP2	cna	gain	BASIS
TCGA-A2-A25B	EPCAM	cna	hetloss	TCGA
TCGA-AN-A0XU	EPCAM	cna	gain	TCGA
TCGA-AO-A0JL	EPCAM	cna	gain	TCGA
TCGA-BH-A0AW	EPCAM	cna	gain	TCGA
TCGA-BH-A0C0	EPCAM	cna	gain	TCGA
TCGA-C8-A12L	EPCAM	cna	gain	TCGA
TCGA-D8-A27M	EPCAM	cna	gain	TCGA
TCGA-LL-A5YP	EPCAM	cna	gain	TCGA
MB-5465	EPCAM	cna	gain	METABRIC
MB-6271	EPCAM	cna	hetloss	METABRIC
MB-7038	EPCAM	cna	gain	METABRIC
PD10014	EPCAM	cna	gain	BASIS
PD13297	EPCAM	cna	gain	BASIS

PD13299	EPCAM	cna	gain	BASIS
PD23562	EPCAM	cna	gain	BASIS
PD23578	EPCAM	cna	gain	BASIS
PD24186	EPCAM	cna	amp	BASIS
PD24206	EPCAM	cna	gain	BASIS
PD24337	EPCAM	cna	gain	BASIS
PD3890	EPCAM	cna	hetloss	BASIS
PD3905	EPCAM	cna	gain	BASIS
PD4006	EPCAM	cna	amp	BASIS
PD4107	EPCAM	cna	gain	BASIS
PD5930	EPCAM	cna	gain	BASIS
PD5935	EPCAM	cna	gain	BASIS
PD5945	EPCAM	cna	gain	BASIS
PD5948	EPCAM	cna	gain	BASIS
PD7067	EPCAM	cna	gain	BASIS
PD7215	EPCAM	cna	gain	BASIS
PD8621	EPCAM	cna	gain	BASIS
PD8980	EPCAM	cna	gain	BASIS
PD9004	EPCAM	cna	gain	BASIS
PD9702	EPCAM	cna	gain	BASIS
TCGA-AN-A0XU	ERCC4	cna	gain	TCGA
TCGA-AO-A0JL	ERCC4	cna	amp	TCGA
TCGA-BH-A0AW	ERCC4	cna	gain	TCGA
TCGA-BH-A0C0	ERCC4	cna	gain	TCGA
TCGA-C8-A12L	ERCC4	cna	gain	TCGA
TCGA-E2-A1L7	ERCC4	cna	hetloss	TCGA
TCGA-LL-A5YP	ERCC4	cna	gain	TCGA
MB-6060	ERCC4	cna	gain	METABRIC
MB-6098	ERCC4	cna	hetloss	METABRIC
MB-6271	ERCC4	cna	gain	METABRIC
MB-7032	ERCC4	cna	gain	METABRIC
MB-7038	ERCC4	cna	hetloss	METABRIC
MB-0420	ERCC4	cna	hetloss	METABRIC
PD10014	ERCC4	cna	hetloss	BASIS
PD11327	ERCC4	cna	hetloss	BASIS
PD11742	ERCC4	cna	gain	BASIS
PD13296	ERCC4	cna	gain	BASIS
PD13297	ERCC4	cna	hetloss	BASIS
PD13299	ERCC4	cna	gain	BASIS
PD22355	ERCC4	cna	hetloss	BASIS
PD24186	ERCC4	cna	gain	BASIS
PD24202	ERCC4	cna	hetloss	BASIS
PD3905	ERCC4	cna	gain	BASIS
PD4005	ERCC4	cna	gain	BASIS
PD4107	ERCC4	cna	gain	BASIS
PD4826	ERCC4	cna	gain	BASIS
PD5935	ERCC4	cna	gain	BASIS

PD5945	ERCC4	cna	gain	BASIS
PD5948	ERCC4	cna	gain	BASIS
PD7067	ERCC4	cna	gain	BASIS
PD7215	ERCC4	cna	amp	BASIS
PD9004	ERCC4	cna	gain	BASIS
PD9585	ERCC4	cna	hetloss	BASIS
TCGA-A2-A25B	ERG	cna	hetloss	TCGA
TCGA-BH-A0AW	ERG	cna	gain	TCGA
TCGA-BH-A0C0	ERG	cna	hetloss	TCGA
TCGA-BH-A1FU	ERG	cna	gain	TCGA
TCGA-C8-A12L	ERG	cna	hetloss	TCGA
TCGA-D8-A27M	ERG	cna	hetloss	TCGA
TCGA-LL-A5YP	ERG	cna	gain	TCGA
MB-0346	ERG	cna	gain	METABRIC
MB-2827	ERG	cna	amp	METABRIC
MB-6098	ERG	cna	hetloss	METABRIC
MB-7048	ERG	cna	hetloss	METABRIC
PD11327	ERG	cna	gain	BASIS
PD13296	ERG	cna	gain	BASIS
PD13297	ERG	cna	hetloss	BASIS
PD13771	ERG	cna	gain	BASIS
PD23562	ERG	cna	gain	BASIS
PD23578	ERG	cna	gain	BASIS
PD24186	ERG	cna	gain	BASIS
PD24206	ERG	cna	gain	BASIS
PD3890	ERG	cna	hetloss	BASIS
PD3905	ERG	cna	gain	BASIS
PD4107	ERG	cna	gain	BASIS
PD4826	ERG	cna	gain	BASIS
PD5930	ERG	cna	gain	BASIS
PD5945	ERG	cna	gain	BASIS
PD5948	ERG	cna	gain	BASIS
PD6731	ERG	cna	gain	BASIS
PD7067	ERG	cna	gain	BASIS
PD7215	ERG	cna	gain	BASIS
PD8621	ERG	cna	gain	BASIS
PD8980	ERG	cna	homdel	BASIS
PD9004	ERG	cna	gain	BASIS
PD9702	ERG	cna	gain	BASIS
TCGA-AN-A0XU	ESCO1	cna	hetloss	TCGA
TCGA-AO-A0JL	ESCO1	cna	gain	TCGA
TCGA-BH-A0AW	ESCO1	cna	gain	TCGA
TCGA-BH-A0C0	ESCO1	cna	gain	TCGA
TCGA-C8-A12L	ESCO1	cna	gain	TCGA
TCGA-D8-A27M	ESCO1	cna	hetloss	TCGA
TCGA-E2-A1L7	ESCO1	cna	gain	TCGA
TCGA-LL-A5YP	ESCO1	cna	amp	TCGA

MB-5465	ESCO1	cna	hetloss	METABRIC
PD10014	ESCO1	cna	gain	BASIS
PD13296	ESCO1	cna	hetloss	BASIS
PD13297	ESCO1	cna	gain	BASIS
PD13299	ESCO1	cna	gain	BASIS
PD23562	ESCO1	cna	gain	BASIS
PD23574	ESCO1	cna	gain	BASIS
PD24186	ESCO1	cna	gain	BASIS
PD24202	ESCO1	cna	hetloss	BASIS
PD24206	ESCO1	cna	gain	BASIS
PD24337	ESCO1	cna	gain	BASIS
PD3890	ESCO1	cna	gain	BASIS
PD3905	ESCO1	cna	gain	BASIS
PD4006	ESCO1	cna	gain	BASIS
PD4107	ESCO1	cna	gain	BASIS
PD4967	ESCO1	cna	hetloss	BASIS
PD5930	ESCO1	cna	gain	BASIS
PD5935	ESCO1	cna	gain	BASIS
PD5945	ESCO1	cna	amp	BASIS
PD5948	ESCO1	cna	gain	BASIS
PD7067	ESCO1	cna	gain	BASIS
PD7215	ESCO1	cna	gain	BASIS
PD8980	ESCO1	cna	hetloss	BASIS
PD9004	ESCO1	cna	gain	BASIS
PD9702	ESCO1	cna	gain	BASIS
TCGA-A2-A25B	ETV1	cna	gain	TCGA
TCGA-BH-A0AW	ETV1	cna	gain	TCGA
TCGA-BH-A0C0	ETV1	cna	hetloss	TCGA
TCGA-C8-A12L	ETV1	cna	gain	TCGA
TCGA-E2-A1L7	ETV1	cna	hetloss	TCGA
TCGA-LL-A5YP	ETV1	cna	hetloss	TCGA
MB-0346	ETV1	cna	hetloss	METABRIC
MB-5070	ETV1	cna	hetloss	METABRIC
MB-5107	ETV1	cna	gain	METABRIC
MB-6098	ETV1	cna	hetloss	METABRIC
MB-0420	ETV1	cna	hetloss	METABRIC
PD11327	ETV1	cna	gain	BASIS
PD13296	ETV1	cna	gain	BASIS
PD13297	ETV1	cna	hetloss	BASIS
PD13771	ETV1	cna	gain	BASIS
PD22355	ETV1	cna	hetloss	BASIS
PD23574	ETV1	cna	gain	BASIS
PD24206	ETV1	cna	gain	BASIS
PD24337	ETV1	cna	hetloss	BASIS
PD3905	ETV1	cna	gain	BASIS
PD4006	ETV1	cna	gain	BASIS
PD4107	ETV1	cna	amp	BASIS

PD4826	ETV1	cna	gain	BASIS
PD5935	ETV1	cna	gain	BASIS
PD5945	ETV1	cna	gain	BASIS
PD6406	ETV1	cna	hetloss	BASIS
PD6413	ETV1	cna	gain	BASIS
PD7067	ETV1	cna	gain	BASIS
PD7215	ETV1	cna	gain	BASIS
PD8621	ETV1	cna	gain	BASIS
PD8980	ETV1	cna	hetloss	BASIS
PD9004	ETV1	cna	gain	BASIS
PD9702	ETV1	cna	gain	BASIS
TCGA-A2-A25B	EWSR1	cna	hetloss	TCGA
TCGA-BH-A0C0	EWSR1	cna	gain	TCGA
TCGA-BH-A1FU	EWSR1	cna	gain	TCGA
TCGA-C8-A12L	EWSR1	cna	gain	TCGA
TCGA-D8-A27M	EWSR1	cna	gain	TCGA
TCGA-E9-A1NC	EWSR1	cna	gain	TCGA
TCGA-LL-A5YP	EWSR1	cna	gain	TCGA
MB-0346	EWSR1	cna	gain	METABRIC
MB-6060	EWSR1	cna	gain	METABRIC
MB-6098	EWSR1	cna	gain	METABRIC
PD11327	EWSR1	cna	gain	BASIS
PD13296	EWSR1	cna	gain	BASIS
PD13297	EWSR1	cna	gain	BASIS
PD13771	EWSR1	cna	gain	BASIS
PD14442	EWSR1	cna	hetloss	BASIS
PD23562	EWSR1	cna	gain	BASIS
PD23574	EWSR1	cna	gain	BASIS
PD24206	EWSR1	cna	hetloss	BASIS
PD3905	EWSR1	cna	gain	BASIS
PD4006	EWSR1	cna	gain	BASIS
PD4107	EWSR1	cna	gain	BASIS
PD4826	EWSR1	cna	gain	BASIS
PD4967	EWSR1	cna	hetloss	BASIS
PD5930	EWSR1	cna	gain	BASIS
PD5935	EWSR1	cna	gain	BASIS
PD5945	EWSR1	cna	amp	BASIS
PD5948	EWSR1	cna	gain	BASIS
PD6731	EWSR1	cna	hetloss	BASIS
PD7067	EWSR1	cna	gain	BASIS
PD7215	EWSR1	cna	gain	BASIS
PD8621	EWSR1	cna	gain	BASIS
PD9004	EWSR1	cna	gain	BASIS
PD9702	EWSR1	cna	gain	BASIS
TCGA-A2-A25B	FAF1	cna	gain	TCGA
TCGA-AN-A0XU	FAF1	cna	gain	TCGA
TCGA-AO-A0JL	FAF1	cna	hetloss	TCGA

TCGA-BH-A0AW	FAF1	cna	gain	TCGA
TCGA-BH-A0C0	FAF1	cna	hetloss	TCGA
TCGA-BH-A1FU	FAF1	cna	gain	TCGA
TCGA-D8-A27M	FAF1	cna	gain	TCGA
TCGA-E2-A1L7	FAF1	cna	gain	TCGA
TCGA-LL-A5YP	FAF1	cna	hetloss	TCGA
MB-0346	FAF1	cna	hetloss	METABRIC
MB-6060	FAF1	cna	hetloss	METABRIC
MB-6098	FAF1	cna	gain	METABRIC
MB-7038	FAF1	cna	gain	METABRIC
MB-0420	FAF1	cna	hetloss	METABRIC
PD11742	FAF1	cna	hetloss	BASIS
PD13299	FAF1	cna	gain	BASIS
PD24186	FAF1	cna	amp	BASIS
PD24206	FAF1	cna	gain	BASIS
PD3905	FAF1	cna	gain	BASIS
PD4005	FAF1	cna	hetloss	BASIS
PD4006	FAF1	cna	gain	BASIS
PD4107	FAF1	cna	gain	BASIS
PD4826	FAF1	cna	gain	BASIS
PD5930	FAF1	cna	gain	BASIS
PD5935	FAF1	cna	gain	BASIS
PD5945	FAF1	cna	amp	BASIS
PD5948	FAF1	cna	gain	BASIS
PD6413	FAF1	cna	hetloss	BASIS
PD7067	FAF1	cna	gain	BASIS
PD7215	FAF1	cna	gain	BASIS
PD8621	FAF1	cna	gain	BASIS
PD9004	FAF1	cna	gain	BASIS
PD9702	FAF1	cna	gain	BASIS
TCGA-A2-A25B	FBXO11	cna	hetloss	TCGA
TCGA-AN-A0XU	FBXO11	cna	gain	TCGA
TCGA-AO-A0JL	FBXO11	cna	gain	TCGA
TCGA-BH-A0AW	FBXO11	cna	gain	TCGA
TCGA-BH-A0C0	FBXO11	cna	gain	TCGA
TCGA-C8-A12L	FBXO11	cna	gain	TCGA
TCGA-D8-A27M	FBXO11	cna	gain	TCGA
TCGA-LL-A5YP	FBXO11	cna	gain	TCGA
MB-5465	FBXO11	cna	gain	METABRIC
MB-6271	FBXO11	cna	hetloss	METABRIC
MB-7038	FBXO11	cna	gain	METABRIC
PD10014	FBXO11	cna	gain	BASIS
PD13297	FBXO11	cna	gain	BASIS
PD13299	FBXO11	cna	gain	BASIS
PD23562	FBXO11	cna	gain	BASIS
PD23578	FBXO11	cna	gain	BASIS
PD24186	FBXO11	cna	amp	BASIS

PD24206	FBXO11	cna	hetloss	BASIS
PD24337	FBXO11	cna	gain	BASIS
PD3890	FBXO11	cna	hetloss	BASIS
PD3905	FBXO11	cna	gain	BASIS
PD4006	FBXO11	cna	amp	BASIS
PD4107	FBXO11	cna	gain	BASIS
PD5930	FBXO11	cna	gain	BASIS
PD5935	FBXO11	cna	gain	BASIS
PD5945	FBXO11	cna	gain	BASIS
PD5948	FBXO11	cna	gain	BASIS
PD7067	FBXO11	cna	gain	BASIS
PD7215	FBXO11	cna	gain	BASIS
PD8621	FBXO11	cna	gain	BASIS
PD8980	FBXO11	cna	gain	BASIS
PD9004	FBXO11	cna	gain	BASIS
PD9702	FBXO11	cna	gain	BASIS
TCGA-A2-A25B	FGFR2	cna	hetloss	TCGA
TCGA-AN-A0XU	FGFR2	cna	hetloss	TCGA
TCGA-AO-A0JL	FGFR2	cna	hetloss	TCGA
TCGA-C8-A12L	FGFR2	cna	hetloss	TCGA
TCGA-E2-A1L7	FGFR2	cna	hetloss	TCGA
TCGA-E9-A1NC	FGFR2	cna	hetloss	TCGA
TCGA-LL-A5YP	FGFR2	cna	gain	TCGA
MB-0346	FGFR2	cna	hetloss	METABRIC
MB-2827	FGFR2	cna	hetloss	METABRIC
MB-5070	FGFR2	cna	hetloss	METABRIC
MB-5465	FGFR2	cna	hetloss	METABRIC
MB-6098	FGFR2	cna	hetloss	METABRIC
MB-7038	FGFR2	cna	hetloss	METABRIC
MB-0420	FGFR2	cna	hetloss	METABRIC
PD10014	FGFR2	cna	gain	BASIS
PD11742	FGFR2	cna	hetloss	BASIS
PD23562	FGFR2	cna	gain	BASIS
PD23578	FGFR2	cna	hetloss	BASIS
PD24186	FGFR2	cna	gain	BASIS
PD24337	FGFR2	cna	hetloss	BASIS
PD3890	FGFR2	cna	hetloss	BASIS
PD3905	FGFR2	cna	gain	BASIS
PD4006	FGFR2	cna	gain	BASIS
PD4107	FGFR2	cna	gain	BASIS
PD5935	FGFR2	cna	gain	BASIS
PD5945	FGFR2	cna	gain	BASIS
PD5948	FGFR2	cna	amp	BASIS
PD6731	FGFR2	cna	hetloss	BASIS
PD7067	FGFR2	cna	gain	BASIS
PD7215	FGFR2	cna	gain	BASIS
PD8621	FGFR2	cna	gain	BASIS

PD8980	FGFR2	cna	hetloss	BASIS
PD9702	FGFR2	cna	gain	BASIS
TCGA-A2-A25B	FLT4	cna	hetloss	TCGA
TCGA-AN-A0XU	FLT4	cna	hetloss	TCGA
TCGA-AO-A0JL	FLT4	cna	hetloss	TCGA
TCGA-BH-A0C0	FLT4	cna	hetloss	TCGA
TCGA-BH-A1FU	FLT4	cna	amp	TCGA
TCGA-C8-A12L	FLT4	cna	hetloss	TCGA
TCGA-D8-A27M	FLT4	cna	hetloss	TCGA
TCGA-E2-A1L7	FLT4	cna	hetloss	TCGA
MB-5070	FLT4	cna	hetloss	METABRIC
MB-5465	FLT4	cna	gain	METABRIC
MB-6098	FLT4	cna	hetloss	METABRIC
MB-7048	FLT4	cna	gain	METABRIC
PD10014	FLT4	cna	hetloss	BASIS
PD13296	FLT4	cna	hetloss	BASIS
PD13297	FLT4	cna	hetloss	BASIS
PD13771	FLT4	cna	hetloss	BASIS
PD22355	FLT4	cna	hetloss	BASIS
PD23562	FLT4	cna	amp	BASIS
PD23574	FLT4	cna	gain	BASIS
PD23578	FLT4	cna	hetloss	BASIS
PD24186	FLT4	cna	gain	BASIS
PD24206	FLT4	cna	gain	BASIS
PD3905	FLT4	cna	gain	BASIS
PD4005	FLT4	cna	hetloss	BASIS
PD4006	FLT4	cna	gain	BASIS
PD4107	FLT4	cna	hetloss	BASIS
PD4826	FLT4	cna	gain	BASIS
PD5945	FLT4	cna	amp	BASIS
PD5948	FLT4	cna	hetloss	BASIS
PD7067	FLT4	cna	gain	BASIS
PD7215	FLT4	cna	amp	BASIS
PD8980	FLT4	cna	gain	BASIS
PD9702	FLT4	cna	gain	BASIS
TCGA-A2-A25B	GPR124	cna	hetloss	TCGA
TCGA-AN-A0XU	GPR124	cna	hetloss	TCGA
TCGA-AO-A0JL	GPR124	cna	gain	TCGA
TCGA-BH-A0C0	GPR124	cna	hetloss	TCGA
TCGA-BH-A1FU	GPR124	cna	homdel	TCGA
TCGA-D8-A27M	GPR124	cna	hetloss	TCGA
TCGA-E2-A1L7	GPR124	cna	hetloss	TCGA
TCGA-LL-A5YP	GPR124	cna	hetloss	TCGA
PD10014	GPR124	cna	hetloss	BASIS
PD11327	GPR124	cna	homdel	BASIS
PD13296	GPR124	cna	hetloss	BASIS
PD13299	GPR124	cna	gain	BASIS

PD13771	GPR124	cna	hetloss	BASIS
PD14442	GPR124	cna	hetloss	BASIS
PD22355	GPR124	cna	hetloss	BASIS
PD23562	GPR124	cna	gain	BASIS
PD23578	GPR124	cna	hetloss	BASIS
PD24186	GPR124	cna	gain	BASIS
PD24202	GPR124	cna	hetloss	BASIS
PD24206	GPR124	cna	amp	BASIS
PD24337	GPR124	cna	hetloss	BASIS
PD3905	GPR124	cna	gain	BASIS
PD4006	GPR124	cna	gain	BASIS
PD4107	GPR124	cna	gain	BASIS
PD4826	GPR124	cna	hetloss	BASIS
PD5935	GPR124	cna	gain	BASIS
PD5945	GPR124	cna	gain	BASIS
PD7067	GPR124	cna	gain	BASIS
PD7215	GPR124	cna	amp	BASIS
PD8980	GPR124	cna	amp	BASIS
PD9004	GPR124	cna	gain	BASIS
PD9585	GPR124	cna	hetloss	BASIS
PD9702	GPR124	cna	gain	BASIS
TCGA-A2-A25B	ICK	cna	gain	TCGA
TCGA-AO-A0JL	ICK	cna	hetloss	TCGA
TCGA-BH-A0AW	ICK	cna	gain	TCGA
TCGA-BH-A0C0	ICK	cna	hetloss	TCGA
TCGA-C8-A12L	ICK	cna	gain	TCGA
TCGA-D8-A27M	ICK	cna	gain	TCGA
MB-0346	ICK	cna	gain	METABRIC
MB-5070	ICK	cna	amp	METABRIC
MB-6098	ICK	cna	hetloss	METABRIC
MB-7032	ICK	cna	hetloss	METABRIC
MB-7038	ICK	cna	hetloss	METABRIC
PD11327	ICK	cna	amp	BASIS
PD13299	ICK	cna	gain	BASIS
PD22355	ICK	cna	gain	BASIS
PD23562	ICK	cna	gain	BASIS
PD23578	ICK	cna	gain	BASIS
PD24186	ICK	cna	gain	BASIS
PD24206	ICK	cna	gain	BASIS
PD3905	ICK	cna	gain	BASIS
PD4006	ICK	cna	gain	BASIS
PD4107	ICK	cna	gain	BASIS
PD4826	ICK	cna	gain	BASIS
PD5930	ICK	cna	gain	BASIS
PD5935	ICK	cna	gain	BASIS
PD5945	ICK	cna	amp	BASIS
PD5948	ICK	cna	gain	BASIS

PD6406	ICK	cna	hetloss	BASIS
PD7067	ICK	cna	amp	BASIS
PD7215	ICK	cna	gain	BASIS
PD8621	ICK	cna	gain	BASIS
PD9004	ICK	cna	gain	BASIS
PD9585	ICK	cna	gain	BASIS
PD9702	ICK	cna	gain	BASIS
TCGA-AO-A0JL	INPP5D	cna	gain	TCGA
TCGA-BH-A0AW	INPP5D	cna	hetloss	TCGA
TCGA-C8-A12L	INPP5D	cna	hetloss	TCGA
TCGA-D8-A27M	INPP5D	cna	gain	TCGA
TCGA-E2-A1L7	INPP5D	cna	hetloss	TCGA
TCGA-E9-A1NC	INPP5D	cna	hetloss	TCGA
MB-2827	INPP5D	cna	hetloss	METABRIC
MB-6060	INPP5D	cna	hetloss	METABRIC
MB-6098	INPP5D	cna	hetloss	METABRIC
MB-6271	INPP5D	cna	hetloss	METABRIC
MB-7038	INPP5D	cna	gain	METABRIC
MB-7048	INPP5D	cna	hetloss	METABRIC
PD10014	INPP5D	cna	gain	BASIS
PD11327	INPP5D	cna	gain	BASIS
PD11742	INPP5D	cna	hetloss	BASIS
PD13296	INPP5D	cna	hetloss	BASIS
PD13297	INPP5D	cna	hetloss	BASIS
PD23578	INPP5D	cna	hetloss	BASIS
PD24186	INPP5D	cna	gain	BASIS
PD24206	INPP5D	cna	gain	BASIS
PD3890	INPP5D	cna	hetloss	BASIS
PD4006	INPP5D	cna	gain	BASIS
PD4967	INPP5D	cna	hetloss	BASIS
PD5935	INPP5D	cna	gain	BASIS
PD5945	INPP5D	cna	gain	BASIS
PD5948	INPP5D	cna	gain	BASIS
PD6406	INPP5D	cna	hetloss	BASIS
PD6413	INPP5D	cna	hetloss	BASIS
PD6731	INPP5D	cna	hetloss	BASIS
PD7067	INPP5D	cna	gain	BASIS
PD7215	INPP5D	cna	gain	BASIS
PD8980	INPP5D	cna	hetloss	BASIS
PD9585	INPP5D	cna	hetloss	BASIS
TCGA-AO-A0JL	JAK3	cna	gain	TCGA
TCGA-BH-A0C0	JAK3	cna	gain	TCGA
TCGA-BH-A1FU	JAK3	cna	hetloss	TCGA
TCGA-C8-A12L	JAK3	cna	gain	TCGA
TCGA-D8-A27M	JAK3	cna	hetloss	TCGA
TCGA-EW-A1OX	JAK3	cna	hetloss	TCGA
TCGA-LL-A5YP	JAK3	cna	hetloss	TCGA

MB-0346	JAK3	cna	hetloss	METABRIC
MB-2827	JAK3	cna	hetloss	METABRIC
MB-6060	JAK3	cna	gain	METABRIC
MB-6098	JAK3	cna	hetloss	METABRIC
MB-0420	JAK3	cna	hetloss	METABRIC
MTS-T0064	JAK3	cna	amp	METABRIC
PD13299	JAK3	cna	gain	BASIS
PD14442	JAK3	cna	hetloss	BASIS
PD22355	JAK3	cna	gain	BASIS
PD23562	JAK3	cna	gain	BASIS
PD23574	JAK3	cna	gain	BASIS
PD24186	JAK3	cna	gain	BASIS
PD24206	JAK3	cna	hetloss	BASIS
PD3890	JAK3	cna	hetloss	BASIS
PD3905	JAK3	cna	gain	BASIS
PD4006	JAK3	cna	amp	BASIS
PD4107	JAK3	cna	gain	BASIS
PD4826	JAK3	cna	gain	BASIS
PD4967	JAK3	cna	hetloss	BASIS
PD5930	JAK3	cna	gain	BASIS
PD5935	JAK3	cna	gain	BASIS
PD5945	JAK3	cna	amp	BASIS
PD5948	JAK3	cna	gain	BASIS
PD7067	JAK3	cna	gain	BASIS
PD7215	JAK3	cna	amp	BASIS
PD9702	JAK3	cna	gain	BASIS
TCGA-AO-A0JL	KLK2	cna	hetloss	TCGA
TCGA-BH-A0AW	KLK2	cna	gain	TCGA
TCGA-BH-A0C0	KLK2	cna	hetloss	TCGA
TCGA-BH-A1FU	KLK2	cna	hetloss	TCGA
TCGA-D8-A27M	KLK2	cna	hetloss	TCGA
TCGA-E2-A1L7	KLK2	cna	hetloss	TCGA
TCGA-EW-A1OX	KLK2	cna	hetloss	TCGA
TCGA-LL-A5YP	KLK2	cna	gain	TCGA
MB-0346	KLK2	cna	hetloss	METABRIC
MB-6098	KLK2	cna	hetloss	METABRIC
MB-7048	KLK2	cna	gain	METABRIC
PD11327	KLK2	cna	gain	BASIS
PD13296	KLK2	cna	gain	BASIS
PD13299	KLK2	cna	gain	BASIS
PD13771	KLK2	cna	hetloss	BASIS
PD23562	KLK2	cna	gain	BASIS
PD23574	KLK2	cna	gain	BASIS
PD24186	KLK2	cna	gain	BASIS
PD24206	KLK2	cna	hetloss	BASIS
PD24337	KLK2	cna	hetloss	BASIS
PD3890	KLK2	cna	hetloss	BASIS

PD3905	KLK2	cna	gain	BASIS
PD4006	KLK2	cna	gain	BASIS
PD4826	KLK2	cna	gain	BASIS
PD5935	KLK2	cna	gain	BASIS
PD5945	KLK2	cna	gain	BASIS
PD5948	KLK2	cna	gain	BASIS
PD6406	KLK2	cna	gain	BASIS
PD7067	KLK2	cna	gain	BASIS
PD7215	KLK2	cna	gain	BASIS
PD8621	KLK2	cna	gain	BASIS
PD8980	KLK2	cna	hetloss	BASIS
PD9585	KLK2	cna	gain	BASIS
TCGA-A2-A25B	MAFB	cna	gain	TCGA
TCGA-AO-A0JL	MAFB	cna	gain	TCGA
TCGA-BH-A0AW	MAFB	cna	gain	TCGA
TCGA-BH-A0C0	MAFB	cna	gain	TCGA
TCGA-C8-A12L	MAFB	cna	gain	TCGA
TCGA-D8-A27M	MAFB	cna	gain	TCGA
TCGA-E2-A1L7	MAFB	cna	gain	TCGA
TCGA-E9-A1NC	MAFB	cna	hetloss	TCGA
TCGA-LL-A5YP	MAFB	cna	hetloss	TCGA
MB-0346	MAFB	cna	gain	METABRIC
MB-5107	MAFB	cna	gain	METABRIC
MB-6060	MAFB	cna	gain	METABRIC
MB-6098	MAFB	cna	hetloss	METABRIC
PD13296	MAFB	cna	hetloss	BASIS
PD13297	MAFB	cna	hetloss	BASIS
PD13771	MAFB	cna	gain	BASIS
PD22355	MAFB	cna	hetloss	BASIS
PD23578	MAFB	cna	hetloss	BASIS
PD24186	MAFB	cna	gain	BASIS
PD24202	MAFB	cna	hetloss	BASIS
PD24206	MAFB	cna	gain	BASIS
PD3905	MAFB	cna	gain	BASIS
PD4005	MAFB	cna	hetloss	BASIS
PD4826	MAFB	cna	gain	BASIS
PD5935	MAFB	cna	gain	BASIS
PD5945	MAFB	cna	amp	BASIS
PD5948	MAFB	cna	gain	BASIS
PD6731	MAFB	cna	hetloss	BASIS
PD7067	MAFB	cna	gain	BASIS
PD7215	MAFB	cna	gain	BASIS
PD8621	MAFB	cna	hetloss	BASIS
PD8980	MAFB	cna	gain	BASIS
PD9004	MAFB	cna	gain	BASIS
TCGA-AN-A0XU	MAP2K1	cna	hetloss	TCGA
TCGA-AO-A0JL	MAP2K1	cna	hetloss	TCGA

TCGA-BH-A0AW	MAP2K1	cna	hetloss	TCGA
TCGA-BH-A0C0	MAP2K1	cna	hetloss	TCGA
TCGA-D8-A27M	MAP2K1	cna	hetloss	TCGA
TCGA-E2-A1L7	MAP2K1	cna	gain	TCGA
TCGA-LL-A5YP	MAP2K1	cna	gain	TCGA
MB-0346	MAP2K1	cna	hetloss	METABRIC
MB-2827	MAP2K1	cna	hetloss	METABRIC
MB-6060	MAP2K1	cna	gain	METABRIC
MB-7038	MAP2K1	cna	gain	METABRIC
MB-7048	MAP2K1	cna	hetloss	METABRIC
PD11742	MAP2K1	cna	hetloss	BASIS
PD13296	MAP2K1	cna	hetloss	BASIS
PD13297	MAP2K1	cna	hetloss	BASIS
PD22355	MAP2K1	cna	hetloss	BASIS
PD23562	MAP2K1	cna	gain	BASIS
PD23574	MAP2K1	cna	gain	BASIS
PD23578	MAP2K1	cna	gain	BASIS
PD24202	MAP2K1	cna	hetloss	BASIS
PD24206	MAP2K1	cna	gain	BASIS
PD3890	MAP2K1	cna	hetloss	BASIS
PD3905	MAP2K1	cna	gain	BASIS
PD4006	MAP2K1	cna	gain	BASIS
PD5945	MAP2K1	cna	gain	BASIS
PD5948	MAP2K1	cna	gain	BASIS
PD6406	MAP2K1	cna	hetloss	BASIS
PD6413	MAP2K1	cna	gain	BASIS
PD7067	MAP2K1	cna	gain	BASIS
PD7215	MAP2K1	cna	gain	BASIS
PD8980	MAP2K1	cna	gain	BASIS
PD9585	MAP2K1	cna	hetloss	BASIS
PD9702	MAP2K1	cna	gain	BASIS
TCGA-AN-A0XU	MLH1	cna	hetloss	TCGA
TCGA-BH-A0C0	MLH1	cna	hetloss	TCGA
TCGA-BH-A18R	MLH1	cna	amp	TCGA
TCGA-BH-A1FU	MLH1	cna	hetloss	TCGA
TCGA-C8-A12L	MLH1	cna	hetloss	TCGA
TCGA-D8-A27M	MLH1	cna	hetloss	TCGA
TCGA-E9-A1NC	MLH1	cna	hetloss	TCGA
TCGA-LL-A5YP	MLH1	cna	amp	TCGA
MB-2827	MLH1	cna	hetloss	METABRIC
MB-7038	MLH1	cna	hetloss	METABRIC
MB-7048	MLH1	cna	hetloss	METABRIC
MB-0420	MLH1	cna	hetloss	METABRIC
PD10014	MLH1	cna	hetloss	BASIS
PD11742	MLH1	cna	gain	BASIS
PD13296	MLH1	cna	gain	BASIS
PD13299	MLH1	cna	gain	BASIS

PD13771	MLH1	cna	gain	BASIS
PD22355	MLH1	cna	hetloss	BASIS
PD23561	MLH1	cna	hetloss	BASIS
PD23574	MLH1	cna	gain	BASIS
PD23578	MLH1	cna	hetloss	BASIS
PD24202	MLH1	cna	hetloss	BASIS
PD24206	MLH1	cna	gain	BASIS
PD3890	MLH1	cna	hetloss	BASIS
PD3905	MLH1	cna	gain	BASIS
PD4826	MLH1	cna	gain	BASIS
PD5945	MLH1	cna	gain	BASIS
PD6406	MLH1	cna	hetloss	BASIS
PD6731	MLH1	cna	hetloss	BASIS
PD7067	MLH1	cna	gain	BASIS
PD8980	MLH1	cna	gain	BASIS
PD9585	MLH1	cna	hetloss	BASIS
PD9702	MLH1	cna	gain	BASIS
TCGA-A2-A25B	MSH2	cna	hetloss	TCGA
TCGA-AN-A0XU	MSH2	cna	gain	TCGA
TCGA-AO-A0JL	MSH2	cna	gain	TCGA
TCGA-BH-A0AW	MSH2	cna	gain	TCGA
TCGA-BH-A0C0	MSH2	cna	gain	TCGA
TCGA-C8-A12L	MSH2	cna	gain	TCGA
TCGA-D8-A27M	MSH2	cna	gain	TCGA
TCGA-LL-A5YP	MSH2	cna	gain	TCGA
MB-5465	MSH2	cna	gain	METABRIC
MB-6271	MSH2	cna	hetloss	METABRIC
MB-7038	MSH2	cna	gain	METABRIC
PD10014	MSH2	cna	gain	BASIS
PD13297	MSH2	cna	gain	BASIS
PD13299	MSH2	cna	gain	BASIS
PD23562	MSH2	cna	gain	BASIS
PD23578	MSH2	cna	gain	BASIS
PD24186	MSH2	cna	amp	BASIS
PD24206	MSH2	cna	gain	BASIS
PD24337	MSH2	cna	gain	BASIS
PD3890	MSH2	cna	hetloss	BASIS
PD3905	MSH2	cna	gain	BASIS
PD4006	MSH2	cna	amp	BASIS
PD4107	MSH2	cna	gain	BASIS
PD5930	MSH2	cna	gain	BASIS
PD5935	MSH2	cna	gain	BASIS
PD5945	MSH2	cna	gain	BASIS
PD5948	MSH2	cna	gain	BASIS
PD7067	MSH2	cna	gain	BASIS
PD7215	MSH2	cna	gain	BASIS
PD8621	MSH2	cna	gain	BASIS

PD8980	MSH2	cna	gain	BASIS
PD9004	MSH2	cna	gain	BASIS
PD9702	MSH2	cna	gain	BASIS
TCGA-A2-A25B	MSH6	cna	hetloss	TCGA
TCGA-AN-A0XU	MSH6	cna	gain	TCGA
TCGA-AO-A0JL	MSH6	cna	gain	TCGA
TCGA-BH-A0AW	MSH6	cna	gain	TCGA
TCGA-BH-A0C0	MSH6	cna	gain	TCGA
TCGA-C8-A12L	MSH6	cna	gain	TCGA
TCGA-D8-A27M	MSH6	cna	gain	TCGA
TCGA-LL-A5YP	MSH6	cna	gain	TCGA
MB-5465	MSH6	cna	gain	METABRIC
MB-6271	MSH6	cna	hetloss	METABRIC
MB-7038	MSH6	cna	gain	METABRIC
PD10014	MSH6	cna	gain	BASIS
PD13297	MSH6	cna	gain	BASIS
PD13299	MSH6	cna	gain	BASIS
PD23562	MSH6	cna	gain	BASIS
PD23578	MSH6	cna	gain	BASIS
PD24186	MSH6	cna	amp	BASIS
PD24206	MSH6	cna	hetloss	BASIS
PD24337	MSH6	cna	gain	BASIS
PD3890	MSH6	cna	hetloss	BASIS
PD3905	MSH6	cna	gain	BASIS
PD4006	MSH6	cna	amp	BASIS
PD4107	MSH6	cna	gain	BASIS
PD5930	MSH6	cna	gain	BASIS
PD5935	MSH6	cna	gain	BASIS
PD5945	MSH6	cna	gain	BASIS
PD5948	MSH6	cna	gain	BASIS
PD7067	MSH6	cna	gain	BASIS
PD7215	MSH6	cna	gain	BASIS
PD8621	MSH6	cna	gain	BASIS
PD8980	MSH6	cna	gain	BASIS
PD9004	MSH6	cna	gain	BASIS
PD9702	MSH6	cna	gain	BASIS
TCGA-A2-A25B	MYCL	cna	hetloss	TCGA
TCGA-AN-A0XU	MYCL	cna	hetloss	TCGA
TCGA-AO-A0JL	MYCL	cna	hetloss	TCGA
TCGA-BH-A0AW	MYCL	cna	gain	TCGA
TCGA-BH-A0C0	MYCL	cna	gain	TCGA
TCGA-BH-A1FU	MYCL	cna	gain	TCGA
TCGA-C8-A12L	MYCL	cna	hetloss	TCGA
TCGA-D8-A27M	MYCL	cna	gain	TCGA
TCGA-E2-A1L7	MYCL	cna	gain	TCGA
TCGA-LL-A5YP	MYCL	cna	gain	TCGA
MB-0346	MYCL	cna	hetloss	METABRIC

MB-6060	MYCL	cna	hetloss	METABRIC
MB-6098	MYCL	cna	hetloss	METABRIC
MB-7038	MYCL	cna	gain	METABRIC
MB-0420	MYCL	cna	hetloss	METABRIC
PD13296	MYCL	cna	gain	BASIS
PD13299	MYCL	cna	gain	BASIS
PD24186	MYCL	cna	amp	BASIS
PD24206	MYCL	cna	gain	BASIS
PD24337	MYCL	cna	gain	BASIS
PD3905	MYCL	cna	gain	BASIS
PD4006	MYCL	cna	gain	BASIS
PD4107	MYCL	cna	gain	BASIS
PD4826	MYCL	cna	gain	BASIS
PD5935	MYCL	cna	gain	BASIS
PD5945	MYCL	cna	amp	BASIS
PD5948	MYCL	cna	gain	BASIS
PD7067	MYCL	cna	gain	BASIS
PD7215	MYCL	cna	gain	BASIS
PD8621	MYCL	cna	gain	BASIS
PD8980	MYCL	cna	gain	BASIS
PD9004	MYCL	cna	hetloss	BASIS
PD9702	MYCL	cna	gain	BASIS
TCGA-A2-A25B	NF1	cna	hetloss	TCGA
TCGA-AN-A0XU	NF1	cna	gain	TCGA
TCGA-AO-A0JL	NF1	cna	gain	TCGA
TCGA-BH-A0AW	NF1	cna	homdel	TCGA
TCGA-BH-A0C0	NF1	cna	gain	TCGA
TCGA-BH-A18R	NF1	cna	amp	TCGA
TCGA-C8-A12L	NF1	cna	gain	TCGA
TCGA-D8-A27M	NF1	cna	hetloss	TCGA
TCGA-E2-A1L7	NF1	cna	hetloss	TCGA
TCGA-E9-A1NC	NF1	cna	hetloss	TCGA
MB-0346	NF1	cna	hetloss	METABRIC
MB-2827	NF1	cna	homdel	METABRIC
MB-5070	NF1	cna	gain	METABRIC
MB-5465	NF1	cna	hetloss	METABRIC
MB-6098	NF1	cna	hetloss	METABRIC
MB-6271	NF1	cna	hetloss	METABRIC
MB-7038	NF1	cna	hetloss	METABRIC
MB-0420	NF1	cna	hetloss	METABRIC
PD11742	NF1	cna	hetloss	BASIS
PD22355	NF1	cna	hetloss	BASIS
PD23561	NF1	cna	hetloss	BASIS
PD24337	NF1	cna	hetloss	BASIS
PD3890	NF1	cna	hetloss	BASIS
PD4005	NF1	cna	hetloss	BASIS
PD4967	NF1	cna	hetloss	BASIS

PD5945	NF1	cna	gain	BASIS
PD6406	NF1	cna	hetloss	BASIS
PD6413	NF1	cna	hetloss	BASIS
PD6731	NF1	cna	hetloss	BASIS
PD7067	NF1	cna	gain	BASIS
PD7215	NF1	cna	gain	BASIS
PD9585	NF1	cna	hetloss	BASIS
PD9702	NF1	cna	gain	BASIS
TCGA-A2-A25B	NF2	cna	hetloss	TCGA
TCGA-BH-A0C0	NF2	cna	gain	TCGA
TCGA-BH-A1FU	NF2	cna	gain	TCGA
TCGA-C8-A12L	NF2	cna	gain	TCGA
TCGA-D8-A27M	NF2	cna	gain	TCGA
TCGA-E9-A1NC	NF2	cna	gain	TCGA
TCGA-LL-A5YP	NF2	cna	amp	TCGA
MB-0346	NF2	cna	gain	METABRIC
MB-6060	NF2	cna	gain	METABRIC
MB-6098	NF2	cna	gain	METABRIC
PD11327	NF2	cna	gain	BASIS
PD13296	NF2	cna	gain	BASIS
PD13297	NF2	cna	gain	BASIS
PD13299	NF2	cna	gain	BASIS
PD14442	NF2	cna	hetloss	BASIS
PD23562	NF2	cna	gain	BASIS
PD23574	NF2	cna	gain	BASIS
PD24206	NF2	cna	hetloss	BASIS
PD3905	NF2	cna	gain	BASIS
PD4006	NF2	cna	gain	BASIS
PD4107	NF2	cna	amp	BASIS
PD4826	NF2	cna	gain	BASIS
PD4967	NF2	cna	hetloss	BASIS
PD5930	NF2	cna	gain	BASIS
PD5935	NF2	cna	gain	BASIS
PD5945	NF2	cna	amp	BASIS
PD5948	NF2	cna	gain	BASIS
PD6731	NF2	cna	hetloss	BASIS
PD7067	NF2	cna	gain	BASIS
PD7215	NF2	cna	gain	BASIS
PD8621	NF2	cna	gain	BASIS
PD9004	NF2	cna	gain	BASIS
PD9702	NF2	cna	gain	BASIS
TCGA-A2-A25B	NFE2L3	cna	gain	TCGA
TCGA-BH-A0AW	NFE2L3	cna	gain	TCGA
TCGA-BH-A0C0	NFE2L3	cna	hetloss	TCGA
TCGA-C8-A12L	NFE2L3	cna	gain	TCGA
TCGA-D8-A27M	NFE2L3	cna	hetloss	TCGA
TCGA-LL-A5YP	NFE2L3	cna	hetloss	TCGA

MB-0346	NFE2L3	cna	gain	METABRIC
MB-5070	NFE2L3	cna	hetloss	METABRIC
MB-6098	NFE2L3	cna	hetloss	METABRIC
MB-0420	NFE2L3	cna	hetloss	METABRIC
PD10014	NFE2L3	cna	hetloss	BASIS
PD11327	NFE2L3	cna	gain	BASIS
PD13296	NFE2L3	cna	gain	BASIS
PD13297	NFE2L3	cna	hetloss	BASIS
PD22355	NFE2L3	cna	hetloss	BASIS
PD23574	NFE2L3	cna	gain	BASIS
PD23578	NFE2L3	cna	hetloss	BASIS
PD24186	NFE2L3	cna	gain	BASIS
PD24206	NFE2L3	cna	gain	BASIS
PD24337	NFE2L3	cna	hetloss	BASIS
PD3905	NFE2L3	cna	gain	BASIS
PD4005	NFE2L3	cna	hetloss	BASIS
PD4006	NFE2L3	cna	gain	BASIS
PD4107	NFE2L3	cna	amp	BASIS
PD4826	NFE2L3	cna	gain	BASIS
PD5935	NFE2L3	cna	gain	BASIS
PD5945	NFE2L3	cna	amp	BASIS
PD6406	NFE2L3	cna	hetloss	BASIS
PD6413	NFE2L3	cna	gain	BASIS
PD7215	NFE2L3	cna	gain	BASIS
PD8621	NFE2L3	cna	gain	BASIS
PD9585	NFE2L3	cna	gain	BASIS
PD9702	NFE2L3	cna	gain	BASIS
TCGA-AN-A0XU	NPM1	cna	hetloss	TCGA
TCGA-AO-A0JL	NPM1	cna	gain	TCGA
TCGA-BH-A0C0	NPM1	cna	hetloss	TCGA
TCGA-BH-A1FU	NPM1	cna	hetloss	TCGA
TCGA-C8-A12L	NPM1	cna	hetloss	TCGA
TCGA-D8-A27M	NPM1	cna	hetloss	TCGA
TCGA-E2-A1L7	NPM1	cna	hetloss	TCGA
TCGA-LL-A5YP	NPM1	cna	hetloss	TCGA
MB-5070	NPM1	cna	hetloss	METABRIC
MB-5465	NPM1	cna	hetloss	METABRIC
MB-6098	NPM1	cna	hetloss	METABRIC
MB-0420	NPM1	cna	hetloss	METABRIC
PD10014	NPM1	cna	hetloss	BASIS
PD13296	NPM1	cna	hetloss	BASIS
PD13297	NPM1	cna	hetloss	BASIS
PD13771	NPM1	cna	gain	BASIS
PD22355	NPM1	cna	hetloss	BASIS
PD23578	NPM1	cna	hetloss	BASIS
PD24186	NPM1	cna	gain	BASIS
PD24202	NPM1	cna	hetloss	BASIS

PD24206	NPM1	cna	gain	BASIS
PD24337	NPM1	cna	hetloss	BASIS
PD3890	NPM1	cna	hetloss	BASIS
PD3905	NPM1	cna	gain	BASIS
PD4005	NPM1	cna	hetloss	BASIS
PD4006	NPM1	cna	gain	BASIS
PD4107	NPM1	cna	hetloss	BASIS
PD4826	NPM1	cna	gain	BASIS
PD5945	NPM1	cna	gain	BASIS
PD6406	NPM1	cna	hetloss	BASIS
PD6413	NPM1	cna	hetloss	BASIS
PD7067	NPM1	cna	gain	BASIS
PD8980	NPM1	cna	gain	BASIS
TCGA-AO-A0JL	NR1H2	cna	hetloss	TCGA
TCGA-BH-A0AW	NR1H2	cna	gain	TCGA
TCGA-BH-A0C0	NR1H2	cna	hetloss	TCGA
TCGA-BH-A1FU	NR1H2	cna	hetloss	TCGA
TCGA-D8-A27M	NR1H2	cna	hetloss	TCGA
TCGA-E2-A1L7	NR1H2	cna	hetloss	TCGA
TCGA-EW-A1OX	NR1H2	cna	gain	TCGA
TCGA-LL-A5YP	NR1H2	cna	gain	TCGA
MB-0346	NR1H2	cna	hetloss	METABRIC
MB-6098	NR1H2	cna	hetloss	METABRIC
MB-7048	NR1H2	cna	gain	METABRIC
PD11327	NR1H2	cna	gain	BASIS
PD13296	NR1H2	cna	gain	BASIS
PD13299	NR1H2	cna	gain	BASIS
PD13771	NR1H2	cna	hetloss	BASIS
PD23562	NR1H2	cna	gain	BASIS
PD23574	NR1H2	cna	gain	BASIS
PD24186	NR1H2	cna	gain	BASIS
PD24206	NR1H2	cna	hetloss	BASIS
PD24337	NR1H2	cna	hetloss	BASIS
PD3890	NR1H2	cna	hetloss	BASIS
PD3905	NR1H2	cna	gain	BASIS
PD4006	NR1H2	cna	gain	BASIS
PD4826	NR1H2	cna	gain	BASIS
PD5935	NR1H2	cna	gain	BASIS
PD5945	NR1H2	cna	gain	BASIS
PD5948	NR1H2	cna	gain	BASIS
PD6406	NR1H2	cna	gain	BASIS
PD7067	NR1H2	cna	gain	BASIS
PD7215	NR1H2	cna	gain	BASIS
PD8621	NR1H2	cna	gain	BASIS
PD8980	NR1H2	cna	hetloss	BASIS
PD9585	NR1H2	cna	gain	BASIS
TCGA-A2-A25B	NTRK2	cna	gain	TCGA

TCGA-AN-A0XU	NTRK2	cna	hetloss	TCGA
TCGA-AO-A0JL	NTRK2	cna	hetloss	TCGA
TCGA-BH-A0C0	NTRK2	cna	hetloss	TCGA
TCGA-BH-A1FU	NTRK2	cna	hetloss	TCGA
TCGA-C8-A12L	NTRK2	cna	hetloss	TCGA
TCGA-E9-A1NC	NTRK2	cna	hetloss	TCGA
TCGA-LL-A5YP	NTRK2	cna	hetloss	TCGA
MB-0346	NTRK2	cna	hetloss	METABRIC
MB-5107	NTRK2	cna	hetloss	METABRIC
MB-6098	NTRK2	cna	hetloss	METABRIC
MB-7038	NTRK2	cna	hetloss	METABRIC
MB-0420	NTRK2	cna	hetloss	METABRIC
MTS-T0064	NTRK2	cna	gain	METABRIC
PD11327	NTRK2	cna	gain	BASIS
PD23562	NTRK2	cna	gain	BASIS
PD23578	NTRK2	cna	hetloss	BASIS
PD24186	NTRK2	cna	amp	BASIS
PD24202	NTRK2	cna	hetloss	BASIS
PD24206	NTRK2	cna	gain	BASIS
PD3890	NTRK2	cna	gain	BASIS
PD3905	NTRK2	cna	gain	BASIS
PD4006	NTRK2	cna	gain	BASIS
PD4107	NTRK2	cna	gain	BASIS
PD5935	NTRK2	cna	gain	BASIS
PD5945	NTRK2	cna	amp	BASIS
PD5948	NTRK2	cna	gain	BASIS
PD6731	NTRK2	cna	hetloss	BASIS
PD7067	NTRK2	cna	gain	BASIS
PD7215	NTRK2	cna	gain	BASIS
PD8621	NTRK2	cna	gain	BASIS
PD9004	NTRK2	cna	gain	BASIS
PD9585	NTRK2	cna	hetloss	BASIS
TCGA-A2-A25B	NUTM2B	cna	hetloss	TCGA
TCGA-AN-A0XU	NUTM2B	cna	hetloss	TCGA
TCGA-AO-A0JL	NUTM2B	cna	gain	TCGA
TCGA-BH-A18R	NUTM2B	cna	gain	TCGA
TCGA-C8-A12L	NUTM2B	cna	gain	TCGA
TCGA-D8-A27M	NUTM2B	cna	hetloss	TCGA
MB-5070	NUTM2B	cna	amp	METABRIC
MB-6060	NUTM2B	cna	hetloss	METABRIC
MB-6098	NUTM2B	cna	hetloss	METABRIC
MTS-T0064	NUTM2B	cna	gain	METABRIC
PD10014	NUTM2B	cna	gain	BASIS
PD11327	NUTM2B	cna	amp	BASIS
PD13296	NUTM2B	cna	hetloss	BASIS
PD13299	NUTM2B	cna	gain	BASIS
PD23574	NUTM2B	cna	gain	BASIS

PD24186	NUTM2B	cna	gain	BASIS
PD24202	NUTM2B	cna	hetloss	BASIS
PD24206	NUTM2B	cna	gain	BASIS
PD3890	NUTM2B	cna	hetloss	BASIS
PD4006	NUTM2B	cna	gain	BASIS
PD4107	NUTM2B	cna	gain	BASIS
PD4826	NUTM2B	cna	gain	BASIS
PD5930	NUTM2B	cna	gain	BASIS
PD5945	NUTM2B	cna	gain	BASIS
PD6406	NUTM2B	cna	hetloss	BASIS
PD6731	NUTM2B	cna	hetloss	BASIS
PD7067	NUTM2B	cna	gain	BASIS
PD7215	NUTM2B	cna	amp	BASIS
PD8621	NUTM2B	cna	gain	BASIS
PD8980	NUTM2B	cna	hetloss	BASIS
PD9004	NUTM2B	cna	gain	BASIS
PD9585	NUTM2B	cna	hetloss	BASIS
PD9702	NUTM2B	cna	gain	BASIS
TCGA-A2-A25B	PCBP1	cna	hetloss	TCGA
TCGA-AN-A0XU	PCBP1	cna	gain	TCGA
TCGA-AO-A0JL	PCBP1	cna	gain	TCGA
TCGA-BH-A0AW	PCBP1	cna	gain	TCGA
TCGA-BH-A0C0	PCBP1	cna	gain	TCGA
TCGA-D8-A27M	PCBP1	cna	gain	TCGA
TCGA-E9-A1NC	PCBP1	cna	gain	TCGA
TCGA-LL-A5YP	PCBP1	cna	gain	TCGA
MB-5070	PCBP1	cna	hetloss	METABRIC
MB-6098	PCBP1	cna	gain	METABRIC
MB-6271	PCBP1	cna	hetloss	METABRIC
MB-7038	PCBP1	cna	gain	METABRIC
MB-7048	PCBP1	cna	hetloss	METABRIC
PD13296	PCBP1	cna	hetloss	BASIS
PD13297	PCBP1	cna	gain	BASIS
PD13299	PCBP1	cna	gain	BASIS
PD23562	PCBP1	cna	gain	BASIS
PD23578	PCBP1	cna	gain	BASIS
PD24186	PCBP1	cna	gain	BASIS
PD24202	PCBP1	cna	gain	BASIS
PD24206	PCBP1	cna	gain	BASIS
PD3890	PCBP1	cna	hetloss	BASIS
PD3905	PCBP1	cna	gain	BASIS
PD4006	PCBP1	cna	gain	BASIS
PD5930	PCBP1	cna	gain	BASIS
PD5935	PCBP1	cna	gain	BASIS
PD5945	PCBP1	cna	gain	BASIS
PD7067	PCBP1	cna	gain	BASIS
PD7215	PCBP1	cna	gain	BASIS

PD8621	PCBP1	cna	gain	BASIS
PD9004	PCBP1	cna	gain	BASIS
PD9585	PCBP1	cna	gain	BASIS
PD9702	PCBP1	cna	gain	BASIS
TCGA-A2-A25B	PDK1	cna	gain	TCGA
TCGA-AO-A0JL	PDK1	cna	gain	TCGA
TCGA-BH-A0AW	PDK1	cna	gain	TCGA
TCGA-D8-A27M	PDK1	cna	gain	TCGA
TCGA-LL-A5YP	PDK1	cna	gain	TCGA
MB-2827	PDK1	cna	hetloss	METABRIC
MB-5107	PDK1	cna	hetloss	METABRIC
MB-6098	PDK1	cna	hetloss	METABRIC
MB-6271	PDK1	cna	hetloss	METABRIC
MB-7032	PDK1	cna	gain	METABRIC
PD11327	PDK1	cna	gain	BASIS
PD13299	PDK1	cna	gain	BASIS
PD22355	PDK1	cna	hetloss	BASIS
PD23578	PDK1	cna	gain	BASIS
PD24186	PDK1	cna	gain	BASIS
PD24202	PDK1	cna	gain	BASIS
PD24206	PDK1	cna	gain	BASIS
PD3890	PDK1	cna	hetloss	BASIS
PD3905	PDK1	cna	gain	BASIS
PD4005	PDK1	cna	hetloss	BASIS
PD4006	PDK1	cna	gain	BASIS
PD4107	PDK1	cna	gain	BASIS
PD4967	PDK1	cna	hetloss	BASIS
PD5930	PDK1	cna	hetloss	BASIS
PD5935	PDK1	cna	gain	BASIS
PD5945	PDK1	cna	amp	BASIS
PD5948	PDK1	cna	gain	BASIS
PD6413	PDK1	cna	hetloss	BASIS
PD7067	PDK1	cna	gain	BASIS
PD7215	PDK1	cna	gain	BASIS
PD8621	PDK1	cna	hetloss	BASIS
PD9004	PDK1	cna	gain	BASIS
PD9702	PDK1	cna	gain	BASIS
TCGA-A2-A25B	PIK3C3	cna	hetloss	TCGA
TCGA-AN-A0XU	PIK3C3	cna	hetloss	TCGA
TCGA-AO-A0JL	PIK3C3	cna	gain	TCGA
TCGA-BH-A0AW	PIK3C3	cna	hetloss	TCGA
TCGA-BH-A0C0	PIK3C3	cna	gain	TCGA
TCGA-C8-A12L	PIK3C3	cna	gain	TCGA
TCGA-D8-A27M	PIK3C3	cna	hetloss	TCGA
TCGA-E2-A1L7	PIK3C3	cna	gain	TCGA
TCGA-LL-A5YP	PIK3C3	cna	hetloss	TCGA
MB-0346	PIK3C3	cna	hetloss	METABRIC

PD10014	PIK3C3	cna	hetloss	BASIS
PD11327	PIK3C3	cna	gain	BASIS
PD11742	PIK3C3	cna	hetloss	BASIS
PD13296	PIK3C3	cna	hetloss	BASIS
PD13297	PIK3C3	cna	gain	BASIS
PD13299	PIK3C3	cna	gain	BASIS
PD23574	PIK3C3	cna	hetloss	BASIS
PD24186	PIK3C3	cna	gain	BASIS
PD24202	PIK3C3	cna	hetloss	BASIS
PD24337	PIK3C3	cna	gain	BASIS
PD3905	PIK3C3	cna	gain	BASIS
PD4005	PIK3C3	cna	gain	BASIS
PD4107	PIK3C3	cna	gain	BASIS
PD4967	PIK3C3	cna	hetloss	BASIS
PD5930	PIK3C3	cna	gain	BASIS
PD5945	PIK3C3	cna	gain	BASIS
PD5948	PIK3C3	cna	hetloss	BASIS
PD6406	PIK3C3	cna	gain	BASIS
PD7215	PIK3C3	cna	gain	BASIS
PD8621	PIK3C3	cna	hetloss	BASIS
PD8980	PIK3C3	cna	hetloss	BASIS
PD9004	PIK3C3	cna	gain	BASIS
PD9702	PIK3C3	cna	gain	BASIS
TCGA-AN-A0XU	PMS1	cna	gain	TCGA
TCGA-AO-A0JL	PMS1	cna	gain	TCGA
TCGA-BH-A0AW	PMS1	cna	gain	TCGA
TCGA-C8-A12L	PMS1	cna	gain	TCGA
TCGA-D8-A27M	PMS1	cna	gain	TCGA
TCGA-LL-A5YP	PMS1	cna	gain	TCGA
MB-2827	PMS1	cna	hetloss	METABRIC
MB-5465	PMS1	cna	hetloss	METABRIC
MB-6060	PMS1	cna	gain	METABRIC
MB-6098	PMS1	cna	hetloss	METABRIC
MB-6271	PMS1	cna	hetloss	METABRIC
MB-7032	PMS1	cna	gain	METABRIC
MB-0420	PMS1	cna	gain	METABRIC
PD10014	PMS1	cna	gain	BASIS
PD11327	PMS1	cna	gain	BASIS
PD13297	PMS1	cna	hetloss	BASIS
PD13299	PMS1	cna	gain	BASIS
PD22355	PMS1	cna	hetloss	BASIS
PD24186	PMS1	cna	gain	BASIS
PD24206	PMS1	cna	gain	BASIS
PD3890	PMS1	cna	hetloss	BASIS
PD3905	PMS1	cna	gain	BASIS
PD4107	PMS1	cna	gain	BASIS
PD4967	PMS1	cna	hetloss	BASIS

PD5935	PMS1	cna	gain	BASIS
PD5945	PMS1	cna	amp	BASIS
PD5948	PMS1	cna	gain	BASIS
PD6413	PMS1	cna	hetloss	BASIS
PD7067	PMS1	cna	amp	BASIS
PD7215	PMS1	cna	gain	BASIS
PD9004	PMS1	cna	gain	BASIS
PD9585	PMS1	cna	hetloss	BASIS
PD9702	PMS1	cna	gain	BASIS
TCGA-BH-A0C0	POGLUT1	cna	hetloss	TCGA
TCGA-BH-A18R	POGLUT1	cna	gain	TCGA
TCGA-BH-A1FU	POGLUT1	cna	hetloss	TCGA
TCGA-C8-A12L	POGLUT1	cna	hetloss	TCGA
TCGA-E2-A1L7	POGLUT1	cna	hetloss	TCGA
TCGA-LL-A5YP	POGLUT1	cna	gain	TCGA
MB-0346	POGLUT1	cna	hetloss	METABRIC
MB-2827	POGLUT1	cna	hetloss	METABRIC
MB-5070	POGLUT1	cna	hetloss	METABRIC
MB-5107	POGLUT1	cna	gain	METABRIC
MB-5465	POGLUT1	cna	gain	METABRIC
MB-6098	POGLUT1	cna	hetloss	METABRIC
MTS-T0064	POGLUT1	cna	hetloss	METABRIC
PD13296	POGLUT1	cna	hetloss	BASIS
PD13299	POGLUT1	cna	gain	BASIS
PD23561	POGLUT1	cna	hetloss	BASIS
PD23574	POGLUT1	cna	gain	BASIS
PD24186	POGLUT1	cna	gain	BASIS
PD24206	POGLUT1	cna	gain	BASIS
PD3905	POGLUT1	cna	gain	BASIS
PD4005	POGLUT1	cna	hetloss	BASIS
PD4107	POGLUT1	cna	gain	BASIS
PD4826	POGLUT1	cna	gain	BASIS
PD5935	POGLUT1	cna	gain	BASIS
PD5945	POGLUT1	cna	gain	BASIS
PD5948	POGLUT1	cna	gain	BASIS
PD6731	POGLUT1	cna	gain	BASIS
PD7067	POGLUT1	cna	gain	BASIS
PD7215	POGLUT1	cna	gain	BASIS
PD8621	POGLUT1	cna	gain	BASIS
PD8980	POGLUT1	cna	gain	BASIS
PD9004	POGLUT1	cna	gain	BASIS
PD9702	POGLUT1	cna	gain	BASIS
TCGA-AN-A0XU	POLQ	cna	gain	TCGA
TCGA-BH-A0C0	POLQ	cna	hetloss	TCGA
TCGA-BH-A18R	POLQ	cna	gain	TCGA
TCGA-BH-A1FU	POLQ	cna	hetloss	TCGA
TCGA-C8-A12L	POLQ	cna	hetloss	TCGA

TCGA-E2-A1L7	POLQ	cna	hetloss	TCGA
TCGA-LL-A5YP	POLQ	cna	gain	TCGA
MB-0346	POLQ	cna	hetloss	METABRIC
MB-5070	POLQ	cna	hetloss	METABRIC
MB-5107	POLQ	cna	gain	METABRIC
MB-5465	POLQ	cna	gain	METABRIC
MB-6098	POLQ	cna	hetloss	METABRIC
MTS-T0064	POLQ	cna	hetloss	METABRIC
PD13296	POLQ	cna	hetloss	BASIS
PD13299	POLQ	cna	gain	BASIS
PD23561	POLQ	cna	hetloss	BASIS
PD23574	POLQ	cna	gain	BASIS
PD24186	POLQ	cna	gain	BASIS
PD24206	POLQ	cna	gain	BASIS
PD3905	POLQ	cna	gain	BASIS
PD4005	POLQ	cna	hetloss	BASIS
PD4107	POLQ	cna	gain	BASIS
PD4826	POLQ	cna	gain	BASIS
PD5935	POLQ	cna	gain	BASIS
PD5945	POLQ	cna	gain	BASIS
PD5948	POLQ	cna	gain	BASIS
PD6731	POLQ	cna	gain	BASIS
PD7067	POLQ	cna	gain	BASIS
PD7215	POLQ	cna	gain	BASIS
PD8621	POLQ	cna	gain	BASIS
PD8980	POLQ	cna	gain	BASIS
PD9004	POLQ	cna	gain	BASIS
PD9702	POLQ	cna	gain	BASIS
TCGA-A2-A25B	RAC1	cna	gain	TCGA
TCGA-BH-A0AW	RAC1	cna	gain	TCGA
TCGA-BH-A0C0	RAC1	cna	hetloss	TCGA
TCGA-C8-A12L	RAC1	cna	gain	TCGA
TCGA-E2-A1L7	RAC1	cna	hetloss	TCGA
TCGA-LL-A5YP	RAC1	cna	hetloss	TCGA
MB-5465	RAC1	cna	gain	METABRIC
MB-6098	RAC1	cna	hetloss	METABRIC
MB-7038	RAC1	cna	hetloss	METABRIC
MB-7048	RAC1	cna	hetloss	METABRIC
MB-0420	RAC1	cna	hetloss	METABRIC
PD11327	RAC1	cna	homdel	BASIS
PD13297	RAC1	cna	hetloss	BASIS
PD13771	RAC1	cna	gain	BASIS
PD22355	RAC1	cna	hetloss	BASIS
PD23574	RAC1	cna	gain	BASIS
PD23578	RAC1	cna	hetloss	BASIS
PD24202	RAC1	cna	gain	BASIS
PD24206	RAC1	cna	gain	BASIS

PD24337	RAC1	cna	hetloss	BASIS
PD3905	RAC1	cna	gain	BASIS
PD4006	RAC1	cna	gain	BASIS
PD4107	RAC1	cna	gain	BASIS
PD4826	RAC1	cna	gain	BASIS
PD5935	RAC1	cna	gain	BASIS
PD5945	RAC1	cna	gain	BASIS
PD6406	RAC1	cna	hetloss	BASIS
PD7067	RAC1	cna	gain	BASIS
PD7215	RAC1	cna	gain	BASIS
PD8621	RAC1	cna	gain	BASIS
PD8980	RAC1	cna	hetloss	BASIS
PD9004	RAC1	cna	gain	BASIS
PD9702	RAC1	cna	gain	BASIS
TCGA-AN-A0XU	RANBP17	cna	hetloss	TCGA
TCGA-AO-A0JL	RANBP17	cna	gain	TCGA
TCGA-BH-A0C0	RANBP17	cna	gain	TCGA
TCGA-BH-A1FU	RANBP17	cna	hetloss	TCGA
TCGA-C8-A12L	RANBP17	cna	hetloss	TCGA
TCGA-D8-A27M	RANBP17	cna	hetloss	TCGA
TCGA-E2-A1L7	RANBP17	cna	hetloss	TCGA
TCGA-LL-A5YP	RANBP17	cna	hetloss	TCGA
MB-5070	RANBP17	cna	hetloss	METABRIC
MB-5465	RANBP17	cna	hetloss	METABRIC
MB-6098	RANBP17	cna	hetloss	METABRIC
MB-0420	RANBP17	cna	hetloss	METABRIC
PD10014	RANBP17	cna	hetloss	BASIS
PD13296	RANBP17	cna	hetloss	BASIS
PD13297	RANBP17	cna	hetloss	BASIS
PD13771	RANBP17	cna	gain	BASIS
PD22355	RANBP17	cna	hetloss	BASIS
PD23578	RANBP17	cna	hetloss	BASIS
PD24186	RANBP17	cna	gain	BASIS
PD24202	RANBP17	cna	hetloss	BASIS
PD24206	RANBP17	cna	gain	BASIS
PD24337	RANBP17	cna	hetloss	BASIS
PD3890	RANBP17	cna	hetloss	BASIS
PD3905	RANBP17	cna	gain	BASIS
PD4005	RANBP17	cna	hetloss	BASIS
PD4107	RANBP17	cna	hetloss	BASIS
PD4826	RANBP17	cna	gain	BASIS
PD5945	RANBP17	cna	gain	BASIS
PD6406	RANBP17	cna	hetloss	BASIS
PD6413	RANBP17	cna	hetloss	BASIS
PD7067	RANBP17	cna	gain	BASIS
PD8980	RANBP17	cna	gain	BASIS
PD9702	RANBP17	cna	gain	BASIS

TCGA-A2-A25B	RPS6KB2	cna	hetloss	TCGA
TCGA-AN-A0XU	RPS6KB2	cna	hetloss	TCGA
TCGA-AO-A0JL	RPS6KB2	cna	hetloss	TCGA
TCGA-BH-A0C0	RPS6KB2	cna	gain	TCGA
TCGA-C8-A12L	RPS6KB2	cna	gain	TCGA
TCGA-EW-A1OX	RPS6KB2	cna	gain	TCGA
TCGA-LL-A5YP	RPS6KB2	cna	hetloss	TCGA
MB-0346	RPS6KB2	cna	gain	METABRIC
MB-5107	RPS6KB2	cna	gain	METABRIC
MB-6271	RPS6KB2	cna	amp	METABRIC
MB-7038	RPS6KB2	cna	gain	METABRIC
P-0002591-T01-IM3	RPS6KB2	cna	amp	MSK-IMPACT
P-0002858-T01-IM3	RPS6KB2	cna	amp	MSK-IMPACT
PD11327	RPS6KB2	cna	hetloss	BASIS
PD11742	RPS6KB2	cna	gain	BASIS
PD22355	RPS6KB2	cna	gain	BASIS
PD23574	RPS6KB2	cna	gain	BASIS
PD24186	RPS6KB2	cna	amp	BASIS
PD3905	RPS6KB2	cna	gain	BASIS
PD4006	RPS6KB2	cna	amp	BASIS
PD4107	RPS6KB2	cna	gain	BASIS
PD4826	RPS6KB2	cna	gain	BASIS
PD5930	RPS6KB2	cna	gain	BASIS
PD5935	RPS6KB2	cna	gain	BASIS
PD5945	RPS6KB2	cna	amp	BASIS
PD5948	RPS6KB2	cna	amp	BASIS
PD6413	RPS6KB2	cna	gain	BASIS
PD7067	RPS6KB2	cna	gain	BASIS
PD7215	RPS6KB2	cna	gain	BASIS
PD8621	RPS6KB2	cna	gain	BASIS
PD8980	RPS6KB2	cna	gain	BASIS
PD9004	RPS6KB2	cna	gain	BASIS
PD9702	RPS6KB2	cna	gain	BASIS
TCGA-A2-A25B	SBDS	cna	hetloss	TCGA
TCGA-AN-A0XU	SBDS	cna	hetloss	TCGA
TCGA-AO-A0JL	SBDS	cna	gain	TCGA
TCGA-BH-A0AW	SBDS	cna	gain	TCGA
TCGA-C8-A12L	SBDS	cna	gain	TCGA
TCGA-D8-A27M	SBDS	cna	gain	TCGA
TCGA-LL-A5YP	SBDS	cna	gain	TCGA
MB-0346	SBDS	cna	hetloss	METABRIC
MB-2827	SBDS	cna	hetloss	METABRIC
MB-6060	SBDS	cna	hetloss	METABRIC
MB-7048	SBDS	cna	hetloss	METABRIC
PD11327	SBDS	cna	homdel	BASIS
PD13296	SBDS	cna	hetloss	BASIS
PD23562	SBDS	cna	gain	BASIS

PD23574	SBDS	cna	gain	BASIS
PD24186	SBDS	cna	gain	BASIS
PD24202	SBDS	cna	hetloss	BASIS
PD24206	SBDS	cna	hetloss	BASIS
PD3890	SBDS	cna	hetloss	BASIS
PD3905	SBDS	cna	gain	BASIS
PD4006	SBDS	cna	gain	BASIS
PD4107	SBDS	cna	gain	BASIS
PD4826	SBDS	cna	gain	BASIS
PD4967	SBDS	cna	hetloss	BASIS
PD5930	SBDS	cna	gain	BASIS
PD5935	SBDS	cna	gain	BASIS
PD5945	SBDS	cna	amp	BASIS
PD5948	SBDS	cna	gain	BASIS
PD6406	SBDS	cna	hetloss	BASIS
PD7215	SBDS	cna	gain	BASIS
PD8621	SBDS	cna	gain	BASIS
PD9004	SBDS	cna	hetloss	BASIS
PD9702	SBDS	cna	gain	BASIS
TCGA-A2-A25B	SIN3A	cna	gain	TCGA
TCGA-AN-A0XU	SIN3A	cna	amp	TCGA
TCGA-AO-A0JL	SIN3A	cna	hetloss	TCGA
TCGA-BH-A0AW	SIN3A	cna	hetloss	TCGA
TCGA-BH-A0C0	SIN3A	cna	hetloss	TCGA
TCGA-D8-A27M	SIN3A	cna	hetloss	TCGA
TCGA-E2-A1L7	SIN3A	cna	gain	TCGA
TCGA-LL-A5YP	SIN3A	cna	hetloss	TCGA
MB-0346	SIN3A	cna	hetloss	METABRIC
MB-5070	SIN3A	cna	hetloss	METABRIC
MB-6060	SIN3A	cna	hetloss	METABRIC
MB-7038	SIN3A	cna	gain	METABRIC
PD10014	SIN3A	cna	gain	BASIS
PD11327	SIN3A	cna	gain	BASIS
PD13296	SIN3A	cna	homdel	BASIS
PD13299	SIN3A	cna	gain	BASIS
PD22355	SIN3A	cna	hetloss	BASIS
PD23562	SIN3A	cna	gain	BASIS
PD23574	SIN3A	cna	gain	BASIS
PD23578	SIN3A	cna	gain	BASIS
PD24202	SIN3A	cna	hetloss	BASIS
PD24206	SIN3A	cna	gain	BASIS
PD3890	SIN3A	cna	hetloss	BASIS
PD3905	SIN3A	cna	gain	BASIS
PD4006	SIN3A	cna	gain	BASIS
PD5945	SIN3A	cna	gain	BASIS
PD5948	SIN3A	cna	hetloss	BASIS
PD7067	SIN3A	cna	gain	BASIS

PD7215	SIN3A	cna	hetloss	BASIS
PD8621	SIN3A	cna	gain	BASIS
PD8980	SIN3A	cna	gain	BASIS
PD9585	SIN3A	cna	hetloss	BASIS
PD9702	SIN3A	cna	gain	BASIS
TCGA-AO-A0JL	SMARCA4	cna	gain	TCGA
TCGA-BH-A0C0	SMARCA4	cna	gain	TCGA
TCGA-BH-A1FU	SMARCA4	cna	hetloss	TCGA
TCGA-C8-A12L	SMARCA4	cna	amp	TCGA
TCGA-D8-A27M	SMARCA4	cna	hetloss	TCGA
TCGA-EW-A1OX	SMARCA4	cna	hetloss	TCGA
TCGA-LL-A5YP	SMARCA4	cna	hetloss	TCGA
MB-0346	SMARCA4	cna	hetloss	METABRIC
MB-2827	SMARCA4	cna	hetloss	METABRIC
MB-5070	SMARCA4	cna	hetloss	METABRIC
MB-5465	SMARCA4	cna	gain	METABRIC
MB-6098	SMARCA4	cna	hetloss	METABRIC
MB-7038	SMARCA4	cna	hetloss	METABRIC
MB-0420	SMARCA4	cna	hetloss	METABRIC
PD11327	SMARCA4	cna	homdel	BASIS
PD13771	SMARCA4	cna	gain	BASIS
PD14442	SMARCA4	cna	hetloss	BASIS
PD23562	SMARCA4	cna	gain	BASIS
PD23574	SMARCA4	cna	gain	BASIS
PD24206	SMARCA4	cna	homdel	BASIS
PD3905	SMARCA4	cna	gain	BASIS
PD4006	SMARCA4	cna	gain	BASIS
PD4107	SMARCA4	cna	gain	BASIS
PD4826	SMARCA4	cna	gain	BASIS
PD4967	SMARCA4	cna	hetloss	BASIS
PD5930	SMARCA4	cna	gain	BASIS
PD5935	SMARCA4	cna	gain	BASIS
PD5945	SMARCA4	cna	amp	BASIS
PD5948	SMARCA4	cna	gain	BASIS
PD7067	SMARCA4	cna	gain	BASIS
PD7215	SMARCA4	cna	amp	BASIS
PD8621	SMARCA4	cna	gain	BASIS
PD9702	SMARCA4	cna	gain	BASIS
TCGA-A2-A25B	SMARCB1	cna	hetloss	TCGA
TCGA-BH-A0C0	SMARCB1	cna	gain	TCGA
TCGA-BH-A1FU	SMARCB1	cna	gain	TCGA
TCGA-C8-A12L	SMARCB1	cna	hetloss	TCGA
TCGA-E9-A1NC	SMARCB1	cna	gain	TCGA
TCGA-LL-A5YP	SMARCB1	cna	gain	TCGA
MB-0346	SMARCB1	cna	gain	METABRIC
MB-6060	SMARCB1	cna	gain	METABRIC
MB-6098	SMARCB1	cna	hetloss	METABRIC

PD11327	SMARCB1	cna	gain	BASIS
PD11742	SMARCB1	cna	hetloss	BASIS
PD13296	SMARCB1	cna	gain	BASIS
PD13297	SMARCB1	cna	gain	BASIS
PD14442	SMARCB1	cna	hetloss	BASIS
PD22355	SMARCB1	cna	hetloss	BASIS
PD23562	SMARCB1	cna	gain	BASIS
PD23574	SMARCB1	cna	gain	BASIS
PD23578	SMARCB1	cna	hetloss	BASIS
PD24206	SMARCB1	cna	hetloss	BASIS
PD3905	SMARCB1	cna	gain	BASIS
PD4006	SMARCB1	cna	gain	BASIS
PD4107	SMARCB1	cna	gain	BASIS
PD4826	SMARCB1	cna	gain	BASIS
PD4967	SMARCB1	cna	hetloss	BASIS
PD5930	SMARCB1	cna	gain	BASIS
PD5935	SMARCB1	cna	gain	BASIS
PD5945	SMARCB1	cna	amp	BASIS
PD5948	SMARCB1	cna	gain	BASIS
PD7067	SMARCB1	cna	gain	BASIS
PD7215	SMARCB1	cna	gain	BASIS
PD8621	SMARCB1	cna	gain	BASIS
PD9004	SMARCB1	cna	gain	BASIS
PD9702	SMARCB1	cna	gain	BASIS
TCGA-A2-A25B	SYK	cna	gain	TCGA
TCGA-AN-A0XU	SYK	cna	hetloss	TCGA
TCGA-AO-A0JL	SYK	cna	hetloss	TCGA
TCGA-BH-A1FU	SYK	cna	hetloss	TCGA
TCGA-C8-A12L	SYK	cna	hetloss	TCGA
TCGA-E9-A1NC	SYK	cna	hetloss	TCGA
TCGA-LL-A5YP	SYK	cna	hetloss	TCGA
MB-0346	SYK	cna	hetloss	METABRIC
MB-5070	SYK	cna	hetloss	METABRIC
MB-5107	SYK	cna	hetloss	METABRIC
MB-6098	SYK	cna	hetloss	METABRIC
MB-7038	SYK	cna	hetloss	METABRIC
MB-0420	SYK	cna	hetloss	METABRIC
MTS-T0064	SYK	cna	gain	METABRIC
PD11327	SYK	cna	gain	BASIS
PD13771	SYK	cna	hetloss	BASIS
PD23562	SYK	cna	gain	BASIS
PD23578	SYK	cna	hetloss	BASIS
PD24186	SYK	cna	amp	BASIS
PD24202	SYK	cna	hetloss	BASIS
PD24206	SYK	cna	gain	BASIS
PD3905	SYK	cna	gain	BASIS
PD4005	SYK	cna	gain	BASIS

PD4006	SYK	cna	gain	BASIS
PD4107	SYK	cna	gain	BASIS
PD5945	SYK	cna	amp	BASIS
PD5948	SYK	cna	gain	BASIS
PD6731	SYK	cna	hetloss	BASIS
PD7067	SYK	cna	gain	BASIS
PD7215	SYK	cna	gain	BASIS
PD8621	SYK	cna	gain	BASIS
PD9004	SYK	cna	gain	BASIS
PD9585	SYK	cna	hetloss	BASIS
TCGA-A2-A25B	TCEA1	cna	gain	TCGA
TCGA-AO-A0JL	TCEA1	cna	amp	TCGA
TCGA-BH-A0AW	TCEA1	cna	gain	TCGA
TCGA-BH-A0C0	TCEA1	cna	gain	TCGA
TCGA-BH-A18R	TCEA1	cna	homdel	TCGA
TCGA-BH-A1FU	TCEA1	cna	gain	TCGA
TCGA-D8-A27M	TCEA1	cna	amp	TCGA
TCGA-E9-A1NC	TCEA1	cna	amp	TCGA
MB-0346	TCEA1	cna	gain	METABRIC
MB-5465	TCEA1	cna	hetloss	METABRIC
MB-6060	TCEA1	cna	amp	METABRIC
MB-6098	TCEA1	cna	gain	METABRIC
PD10014	TCEA1	cna	amp	BASIS
PD11327	TCEA1	cna	amp	BASIS
PD13296	TCEA1	cna	homdel	BASIS
PD13299	TCEA1	cna	gain	BASIS
PD23562	TCEA1	cna	gain	BASIS
PD23574	TCEA1	cna	gain	BASIS
PD23578	TCEA1	cna	hetloss	BASIS
PD24186	TCEA1	cna	gain	BASIS
PD3905	TCEA1	cna	gain	BASIS
PD4005	TCEA1	cna	hetloss	BASIS
PD4006	TCEA1	cna	gain	BASIS
PD4826	TCEA1	cna	amp	BASIS
PD5935	TCEA1	cna	gain	BASIS
PD5945	TCEA1	cna	gain	BASIS
PD7067	TCEA1	cna	amp	BASIS
PD7215	TCEA1	cna	amp	BASIS
PD8621	TCEA1	cna	gain	BASIS
PD8980	TCEA1	cna	gain	BASIS
PD9004	TCEA1	cna	gain	BASIS
PD9585	TCEA1	cna	hetloss	BASIS
PD9702	TCEA1	cna	gain	BASIS
TCGA-AO-A0JL	TFPT	cna	hetloss	TCGA
TCGA-BH-A0AW	TFPT	cna	gain	TCGA
TCGA-BH-A0C0	TFPT	cna	hetloss	TCGA
TCGA-BH-A1FU	TFPT	cna	hetloss	TCGA

TCGA-D8-A27M	TFPT	cna	hetloss	TCGA
TCGA-EW-A1OX	TFPT	cna	hetloss	TCGA
TCGA-LL-A5YP	TFPT	cna	gain	TCGA
MB-0346	TFPT	cna	hetloss	METABRIC
MB-2827	TFPT	cna	hetloss	METABRIC
MB-6098	TFPT	cna	hetloss	METABRIC
MB-7048	TFPT	cna	gain	METABRIC
PD11327	TFPT	cna	gain	BASIS
PD13296	TFPT	cna	gain	BASIS
PD13299	TFPT	cna	gain	BASIS
PD13771	TFPT	cna	hetloss	BASIS
PD23562	TFPT	cna	gain	BASIS
PD23574	TFPT	cna	gain	BASIS
PD24186	TFPT	cna	gain	BASIS
PD24206	TFPT	cna	hetloss	BASIS
PD3890	TFPT	cna	hetloss	BASIS
PD3905	TFPT	cna	gain	BASIS
PD4006	TFPT	cna	gain	BASIS
PD4107	TFPT	cna	gain	BASIS
PD4826	TFPT	cna	gain	BASIS
PD5935	TFPT	cna	gain	BASIS
PD5945	TFPT	cna	gain	BASIS
PD5948	TFPT	cna	gain	BASIS
PD6406	TFPT	cna	gain	BASIS
PD7067	TFPT	cna	gain	BASIS
PD7215	TFPT	cna	gain	BASIS
PD8621	TFPT	cna	gain	BASIS
PD8980	TFPT	cna	hetloss	BASIS
PD9585	TFPT	cna	gain	BASIS
TCGA-A2-A25B	TLL2	cna	hetloss	TCGA
TCGA-AN-A0XU	TLL2	cna	hetloss	TCGA
TCGA-AO-A0JL	TLL2	cna	hetloss	TCGA
TCGA-BH-A18R	TLL2	cna	gain	TCGA
TCGA-C8-A12L	TLL2	cna	gain	TCGA
TCGA-D8-A27M	TLL2	cna	hetloss	TCGA
TCGA-E2-A1L7	TLL2	cna	hetloss	TCGA
MB-2827	TLL2	cna	hetloss	METABRIC
MB-5070	TLL2	cna	hetloss	METABRIC
MB-6060	TLL2	cna	hetloss	METABRIC
MB-6098	TLL2	cna	hetloss	METABRIC
MB-7038	TLL2	cna	hetloss	METABRIC
MB-7048	TLL2	cna	hetloss	METABRIC
PD10014	TLL2	cna	hetloss	BASIS
PD11742	TLL2	cna	hetloss	BASIS
PD13296	TLL2	cna	hetloss	BASIS
PD22355	TLL2	cna	hetloss	BASIS
PD23562	TLL2	cna	gain	BASIS

PD23574	TLL2	cna	gain	BASIS
PD23578	TLL2	cna	hetloss	BASIS
PD24202	TLL2	cna	hetloss	BASIS
PD3905	TLL2	cna	gain	BASIS
PD4006	TLL2	cna	gain	BASIS
PD4107	TLL2	cna	gain	BASIS
PD5935	TLL2	cna	hetloss	BASIS
PD6406	TLL2	cna	hetloss	BASIS
PD6731	TLL2	cna	hetloss	BASIS
PD7067	TLL2	cna	gain	BASIS
PD7215	TLL2	cna	gain	BASIS
PD8621	TLL2	cna	gain	BASIS
PD8980	TLL2	cna	hetloss	BASIS
PD9004	TLL2	cna	gain	BASIS
PD9585	TLL2	cna	hetloss	BASIS
TCGA-AN-A0XU	TLX3	cna	hetloss	TCGA
TCGA-AO-A0JL	TLX3	cna	gain	TCGA
TCGA-BH-A0C0	TLX3	cna	hetloss	TCGA
TCGA-BH-A1FU	TLX3	cna	hetloss	TCGA
TCGA-C8-A12L	TLX3	cna	hetloss	TCGA
TCGA-D8-A27M	TLX3	cna	hetloss	TCGA
TCGA-E2-A1L7	TLX3	cna	hetloss	TCGA
TCGA-LL-A5YP	TLX3	cna	hetloss	TCGA
MB-5070	TLX3	cna	hetloss	METABRIC
MB-5465	TLX3	cna	hetloss	METABRIC
MB-6098	TLX3	cna	hetloss	METABRIC
MB-0420	TLX3	cna	hetloss	METABRIC
PD10014	TLX3	cna	hetloss	BASIS
PD13296	TLX3	cna	hetloss	BASIS
PD13297	TLX3	cna	hetloss	BASIS
PD13771	TLX3	cna	gain	BASIS
PD22355	TLX3	cna	hetloss	BASIS
PD23578	TLX3	cna	hetloss	BASIS
PD24186	TLX3	cna	gain	BASIS
PD24202	TLX3	cna	hetloss	BASIS
PD24206	TLX3	cna	gain	BASIS
PD24337	TLX3	cna	hetloss	BASIS
PD3890	TLX3	cna	hetloss	BASIS
PD3905	TLX3	cna	gain	BASIS
PD4005	TLX3	cna	hetloss	BASIS
PD4006	TLX3	cna	gain	BASIS
PD4107	TLX3	cna	hetloss	BASIS
PD4826	TLX3	cna	gain	BASIS
PD5945	TLX3	cna	gain	BASIS
PD6406	TLX3	cna	hetloss	BASIS
PD6413	TLX3	cna	hetloss	BASIS
PD7067	TLX3	cna	gain	BASIS

PD8980	TLX3	cna	gain	BASIS
TCGA-A2-A25B	TRIM7	cna	hetloss	TCGA
TCGA-AN-A0XU	TRIM7	cna	hetloss	TCGA
TCGA-AO-A0JL	TRIM7	cna	hetloss	TCGA
TCGA-BH-A0C0	TRIM7	cna	hetloss	TCGA
TCGA-BH-A1FU	TRIM7	cna	amp	TCGA
TCGA-C8-A12L	TRIM7	cna	hetloss	TCGA
TCGA-D8-A27M	TRIM7	cna	hetloss	TCGA
TCGA-E2-A1L7	TRIM7	cna	hetloss	TCGA
MB-5070	TRIM7	cna	hetloss	METABRIC
MB-5465	TRIM7	cna	gain	METABRIC
MB-6098	TRIM7	cna	hetloss	METABRIC
MB-7048	TRIM7	cna	gain	METABRIC
PD10014	TRIM7	cna	hetloss	BASIS
PD13296	TRIM7	cna	hetloss	BASIS
PD13297	TRIM7	cna	hetloss	BASIS
PD13771	TRIM7	cna	hetloss	BASIS
PD22355	TRIM7	cna	hetloss	BASIS
PD23562	TRIM7	cna	amp	BASIS
PD23574	TRIM7	cna	gain	BASIS
PD23578	TRIM7	cna	hetloss	BASIS
PD24186	TRIM7	cna	gain	BASIS
PD24206	TRIM7	cna	gain	BASIS
PD3905	TRIM7	cna	gain	BASIS
PD4005	TRIM7	cna	hetloss	BASIS
PD4006	TRIM7	cna	gain	BASIS
PD4107	TRIM7	cna	hetloss	BASIS
PD4826	TRIM7	cna	gain	BASIS
PD5945	TRIM7	cna	amp	BASIS
PD5948	TRIM7	cna	hetloss	BASIS
PD7067	TRIM7	cna	gain	BASIS
PD7215	TRIM7	cna	amp	BASIS
PD8980	TRIM7	cna	gain	BASIS
PD9702	TRIM7	cna	gain	BASIS
TCGA-A2-A25B	TRRAP	cna	gain	TCGA
TCGA-AN-A0XU	TRRAP	cna	hetloss	TCGA
TCGA-AO-A0JL	TRRAP	cna	gain	TCGA
TCGA-BH-A0AW	TRRAP	cna	gain	TCGA
TCGA-C8-A12L	TRRAP	cna	hetloss	TCGA
TCGA-D8-A27M	TRRAP	cna	gain	TCGA
TCGA-E2-A1L7	TRRAP	cna	hetloss	TCGA
TCGA-E9-A1NC	TRRAP	cna	gain	TCGA
TCGA-LL-A5YP	TRRAP	cna	gain	TCGA
MB-0346	TRRAP	cna	hetloss	METABRIC
MB-2827	TRRAP	cna	hetloss	METABRIC
PD11327	TRRAP	cna	gain	BASIS
PD13296	TRRAP	cna	hetloss	BASIS

PD22355	TRRAP	cna	hetloss	BASIS
PD23574	TRRAP	cna	gain	BASIS
PD23578	TRRAP	cna	gain	BASIS
PD24186	TRRAP	cna	amp	BASIS
PD24206	TRRAP	cna	homdel	BASIS
PD3890	TRRAP	cna	hetloss	BASIS
PD3905	TRRAP	cna	gain	BASIS
PD4006	TRRAP	cna	amp	BASIS
PD4107	TRRAP	cna	gain	BASIS
PD4826	TRRAP	cna	gain	BASIS
PD5930	TRRAP	cna	gain	BASIS
PD5935	TRRAP	cna	gain	BASIS
PD5945	TRRAP	cna	amp	BASIS
PD5948	TRRAP	cna	gain	BASIS
PD7067	TRRAP	cna	amp	BASIS
PD7215	TRRAP	cna	gain	BASIS
PD8621	TRRAP	cna	gain	BASIS
PD9004	TRRAP	cna	amp	BASIS
PD9585	TRRAP	cna	hetloss	BASIS
PD9702	TRRAP	cna	gain	BASIS
TCGA-A2-A25B	TSC1	cna	gain	TCGA
TCGA-AN-A0XU	TSC1	cna	hetloss	TCGA
TCGA-AO-A0JL	TSC1	cna	hetloss	TCGA
TCGA-BH-A0C0	TSC1	cna	gain	TCGA
TCGA-C8-A12L	TSC1	cna	gain	TCGA
TCGA-E2-A1L7	TSC1	cna	gain	TCGA
TCGA-E9-A1NC	TSC1	cna	hetloss	TCGA
TCGA-LL-A5YP	TSC1	cna	hetloss	TCGA
MB-0346	TSC1	cna	hetloss	METABRIC
MB-5070	TSC1	cna	hetloss	METABRIC
MB-7038	TSC1	cna	hetloss	METABRIC
MB-0420	TSC1	cna	hetloss	METABRIC
PD10014	TSC1	cna	gain	BASIS
PD11327	TSC1	cna	gain	BASIS
PD13299	TSC1	cna	gain	BASIS
PD22355	TSC1	cna	gain	BASIS
PD24186	TSC1	cna	gain	BASIS
PD24202	TSC1	cna	hetloss	BASIS
PD24206	TSC1	cna	gain	BASIS
PD24337	TSC1	cna	hetloss	BASIS
PD3890	TSC1	cna	hetloss	BASIS
PD3905	TSC1	cna	gain	BASIS
PD4005	TSC1	cna	hetloss	BASIS
PD4006	TSC1	cna	gain	BASIS
PD4107	TSC1	cna	hetloss	BASIS
PD5945	TSC1	cna	gain	BASIS
PD5948	TSC1	cna	gain	BASIS

PD6406	TSC1	cna	hetloss	BASIS
PD6731	TSC1	cna	hetloss	BASIS
PD7067	TSC1	cna	gain	BASIS
PD7215	TSC1	cna	gain	BASIS
PD8621	TSC1	cna	gain	BASIS
PD9004	TSC1	cna	hetloss	BASIS
TCGA-AN-A0XU	TSHZ3	cna	gain	TCGA
TCGA-AO-A0JL	TSHZ3	cna	gain	TCGA
TCGA-BH-A0AW	TSHZ3	cna	gain	TCGA
TCGA-BH-A0C0	TSHZ3	cna	hetloss	TCGA
TCGA-BH-A1FU	TSHZ3	cna	hetloss	TCGA
TCGA-C8-A12L	TSHZ3	cna	gain	TCGA
TCGA-D8-A27M	TSHZ3	cna	amp	TCGA
TCGA-E9-A1NC	TSHZ3	cna	hetloss	TCGA
TCGA-EW-A1OX	TSHZ3	cna	hetloss	TCGA
TCGA-LL-A5YP	TSHZ3	cna	gain	TCGA
MB-0346	TSHZ3	cna	hetloss	METABRIC
MB-6098	TSHZ3	cna	gain	METABRIC
MB-7038	TSHZ3	cna	gain	METABRIC
PD10014	TSHZ3	cna	hetloss	BASIS
PD11327	TSHZ3	cna	gain	BASIS
PD13296	TSHZ3	cna	gain	BASIS
PD13299	TSHZ3	cna	gain	BASIS
PD23562	TSHZ3	cna	gain	BASIS
PD23574	TSHZ3	cna	amp	BASIS
PD24186	TSHZ3	cna	gain	BASIS
PD24206	TSHZ3	cna	gain	BASIS
PD3905	TSHZ3	cna	gain	BASIS
PD4006	TSHZ3	cna	gain	BASIS
PD4826	TSHZ3	cna	gain	BASIS
PD4967	TSHZ3	cna	gain	BASIS
PD5935	TSHZ3	cna	gain	BASIS
PD5945	TSHZ3	cna	amp	BASIS
PD5948	TSHZ3	cna	gain	BASIS
PD7067	TSHZ3	cna	gain	BASIS
PD7215	TSHZ3	cna	gain	BASIS
PD8621	TSHZ3	cna	gain	BASIS
PD8980	TSHZ3	cna	hetloss	BASIS
PD9702	TSHZ3	cna	gain	BASIS
TCGA-A2-A25B	WIF1	cna	gain	TCGA
TCGA-AO-A0JL	WIF1	cna	hetloss	TCGA
TCGA-BH-A18R	WIF1	cna	gain	TCGA
TCGA-BH-A1FU	WIF1	cna	hetloss	TCGA
TCGA-C8-A12L	WIF1	cna	gain	TCGA
TCGA-E2-A1L7	WIF1	cna	hetloss	TCGA
TCGA-LL-A5YP	WIF1	cna	hetloss	TCGA
MB-0346	WIF1	cna	gain	METABRIC

MB-2827	WIF1	cna	hetloss	METABRIC
MB-5107	WIF1	cna	gain	METABRIC
MB-5465	WIF1	cna	gain	METABRIC
MB-6098	WIF1	cna	gain	METABRIC
MB-0420	WIF1	cna	hetloss	METABRIC
MTS-T0064	WIF1	cna	gain	METABRIC
PD10014	WIF1	cna	gain	BASIS
PD22355	WIF1	cna	hetloss	BASIS
PD23578	WIF1	cna	gain	BASIS
PD24186	WIF1	cna	gain	BASIS
PD24202	WIF1	cna	hetloss	BASIS
PD24206	WIF1	cna	gain	BASIS
PD24337	WIF1	cna	gain	BASIS
PD3890	WIF1	cna	hetloss	BASIS
PD3905	WIF1	cna	gain	BASIS
PD4107	WIF1	cna	gain	BASIS
PD4826	WIF1	cna	gain	BASIS
PD5945	WIF1	cna	gain	BASIS
PD6406	WIF1	cna	hetloss	BASIS
PD6413	WIF1	cna	hetloss	BASIS
PD6731	WIF1	cna	hetloss	BASIS
PD7067	WIF1	cna	gain	BASIS
PD8621	WIF1	cna	hetloss	BASIS
PD9585	WIF1	cna	hetloss	BASIS
PD9702	WIF1	cna	gain	BASIS
TCGA-AN-A0XU	ZNF24	cna	hetloss	TCGA
TCGA-AO-A0JL	ZNF24	cna	gain	TCGA
TCGA-BH-A0AW	ZNF24	cna	hetloss	TCGA
TCGA-BH-A0C0	ZNF24	cna	gain	TCGA
TCGA-C8-A12L	ZNF24	cna	gain	TCGA
TCGA-D8-A27M	ZNF24	cna	hetloss	TCGA
TCGA-E2-A1L7	ZNF24	cna	gain	TCGA
TCGA-LL-A5YP	ZNF24	cna	gain	TCGA
MB-0346	ZNF24	cna	hetloss	METABRIC
PD10014	ZNF24	cna	hetloss	BASIS
PD11327	ZNF24	cna	gain	BASIS
PD13296	ZNF24	cna	hetloss	BASIS
PD13297	ZNF24	cna	gain	BASIS
PD13299	ZNF24	cna	gain	BASIS
PD23574	ZNF24	cna	hetloss	BASIS
PD23578	ZNF24	cna	gain	BASIS
PD24186	ZNF24	cna	gain	BASIS
PD24202	ZNF24	cna	hetloss	BASIS
PD24206	ZNF24	cna	gain	BASIS
PD24337	ZNF24	cna	gain	BASIS
PD3905	ZNF24	cna	gain	BASIS
PD4006	ZNF24	cna	gain	BASIS

PD4107	ZNF24	cna	gain	BASIS
PD4967	ZNF24	cna	hetloss	BASIS
PD5930	ZNF24	cna	gain	BASIS
PD5945	ZNF24	cna	gain	BASIS
PD5948	ZNF24	cna	gain	BASIS
PD6406	ZNF24	cna	gain	BASIS
PD7067	ZNF24	cna	amp	BASIS
PD7215	ZNF24	cna	gain	BASIS
PD8980	ZNF24	cna	hetloss	BASIS
PD9004	ZNF24	cna	hetloss	BASIS
PD9702	ZNF24	cna	gain	BASIS
TCGA-AO-A0JL	ZNF331	cna	hetloss	TCGA
TCGA-BH-A0AW	ZNF331	cna	gain	TCGA
TCGA-BH-A0C0	ZNF331	cna	hetloss	TCGA
TCGA-BH-A1FU	ZNF331	cna	hetloss	TCGA
TCGA-D8-A27M	ZNF331	cna	hetloss	TCGA
TCGA-EW-A1OX	ZNF331	cna	hetloss	TCGA
TCGA-LL-A5YP	ZNF331	cna	gain	TCGA
MB-0346	ZNF331	cna	hetloss	METABRIC
MB-2827	ZNF331	cna	hetloss	METABRIC
MB-6098	ZNF331	cna	hetloss	METABRIC
MB-7048	ZNF331	cna	gain	METABRIC
PD11327	ZNF331	cna	gain	BASIS
PD13296	ZNF331	cna	gain	BASIS
PD13299	ZNF331	cna	gain	BASIS
PD13771	ZNF331	cna	hetloss	BASIS
PD23562	ZNF331	cna	gain	BASIS
PD23574	ZNF331	cna	gain	BASIS
PD24186	ZNF331	cna	gain	BASIS
PD24206	ZNF331	cna	hetloss	BASIS
PD3890	ZNF331	cna	hetloss	BASIS
PD3905	ZNF331	cna	gain	BASIS
PD4006	ZNF331	cna	gain	BASIS
PD4107	ZNF331	cna	gain	BASIS
PD4826	ZNF331	cna	gain	BASIS
PD5935	ZNF331	cna	gain	BASIS
PD5945	ZNF331	cna	gain	BASIS
PD5948	ZNF331	cna	gain	BASIS
PD6406	ZNF331	cna	gain	BASIS
PD7067	ZNF331	cna	gain	BASIS
PD7215	ZNF331	cna	gain	BASIS
PD8621	ZNF331	cna	gain	BASIS
PD8980	ZNF331	cna	hetloss	BASIS
PD9585	ZNF331	cna	gain	BASIS
TCGA-AO-A0JL	ACKR3	cna	gain	TCGA
TCGA-BH-A0AW	ACKR3	cna	hetloss	TCGA
TCGA-C8-A12L	ACKR3	cna	hetloss	TCGA

TCGA-D8-A27M	ACKR3	cna	gain	TCGA
TCGA-E2-A1L7	ACKR3	cna	hetloss	TCGA
TCGA-E9-A1NC	ACKR3	cna	hetloss	TCGA
MB-2827	ACKR3	cna	hetloss	METABRIC
MB-6098	ACKR3	cna	hetloss	METABRIC
MB-6271	ACKR3	cna	hetloss	METABRIC
MB-7048	ACKR3	cna	hetloss	METABRIC
PD10014	ACKR3	cna	gain	BASIS
PD11327	ACKR3	cna	gain	BASIS
PD11742	ACKR3	cna	hetloss	BASIS
PD13296	ACKR3	cna	hetloss	BASIS
PD13297	ACKR3	cna	hetloss	BASIS
PD23578	ACKR3	cna	hetloss	BASIS
PD24186	ACKR3	cna	gain	BASIS
PD24206	ACKR3	cna	gain	BASIS
PD3890	ACKR3	cna	hetloss	BASIS
PD4005	ACKR3	cna	hetloss	BASIS
PD4006	ACKR3	cna	gain	BASIS
PD4967	ACKR3	cna	hetloss	BASIS
PD5935	ACKR3	cna	gain	BASIS
PD5945	ACKR3	cna	gain	BASIS
PD5948	ACKR3	cna	gain	BASIS
PD6406	ACKR3	cna	hetloss	BASIS
PD6413	ACKR3	cna	hetloss	BASIS
PD6731	ACKR3	cna	hetloss	BASIS
PD7067	ACKR3	cna	gain	BASIS
PD7215	ACKR3	cna	gain	BASIS
PD8980	ACKR3	cna	hetloss	BASIS
PD9585	ACKR3	cna	hetloss	BASIS
TCGA-A2-A25B	ACTB	cna	gain	TCGA
TCGA-BH-A0AW	ACTB	cna	gain	TCGA
TCGA-BH-A0C0	ACTB	cna	hetloss	TCGA
TCGA-C8-A12L	ACTB	cna	gain	TCGA
TCGA-E2-A1L7	ACTB	cna	hetloss	TCGA
TCGA-LL-A5YP	ACTB	cna	homdel	TCGA
MB-5465	ACTB	cna	gain	METABRIC
MB-6098	ACTB	cna	hetloss	METABRIC
MB-7038	ACTB	cna	hetloss	METABRIC
MB-7048	ACTB	cna	hetloss	METABRIC
MB-0420	ACTB	cna	hetloss	METABRIC
PD11327	ACTB	cna	homdel	BASIS
PD13297	ACTB	cna	hetloss	BASIS
PD22355	ACTB	cna	hetloss	BASIS
PD23574	ACTB	cna	gain	BASIS
PD23578	ACTB	cna	hetloss	BASIS
PD24202	ACTB	cna	gain	BASIS
PD24206	ACTB	cna	homdel	BASIS

PD24337	ACTB	cna	hetloss	BASIS
PD3905	ACTB	cna	gain	BASIS
PD4006	ACTB	cna	gain	BASIS
PD4107	ACTB	cna	gain	BASIS
PD4826	ACTB	cna	gain	BASIS
PD5935	ACTB	cna	gain	BASIS
PD5945	ACTB	cna	gain	BASIS
PD6406	ACTB	cna	hetloss	BASIS
PD7067	ACTB	cna	gain	BASIS
PD7215	ACTB	cna	gain	BASIS
PD8621	ACTB	cna	gain	BASIS
PD8980	ACTB	cna	hetloss	BASIS
PD9004	ACTB	cna	gain	BASIS
PD9702	ACTB	cna	gain	BASIS
TCGA-A2-A25B	BCR	cna	hetloss	TCGA
TCGA-BH-A0C0	BCR	cna	gain	TCGA
TCGA-BH-A1FU	BCR	cna	gain	TCGA
TCGA-C8-A12L	BCR	cna	hetloss	TCGA
TCGA-E9-A1NC	BCR	cna	gain	TCGA
TCGA-LL-A5YP	BCR	cna	gain	TCGA
MB-0346	BCR	cna	gain	METABRIC
MB-6060	BCR	cna	gain	METABRIC
MB-6098	BCR	cna	hetloss	METABRIC
PD11327	BCR	cna	gain	BASIS
PD11742	BCR	cna	hetloss	BASIS
PD13296	BCR	cna	gain	BASIS
PD13297	BCR	cna	gain	BASIS
PD14442	BCR	cna	hetloss	BASIS
PD22355	BCR	cna	hetloss	BASIS
PD23562	BCR	cna	gain	BASIS
PD23574	BCR	cna	gain	BASIS
PD23578	BCR	cna	hetloss	BASIS
PD24206	BCR	cna	hetloss	BASIS
PD3905	BCR	cna	gain	BASIS
PD4006	BCR	cna	gain	BASIS
PD4107	BCR	cna	gain	BASIS
PD4826	BCR	cna	gain	BASIS
PD5930	BCR	cna	gain	BASIS
PD5935	BCR	cna	gain	BASIS
PD5945	BCR	cna	amp	BASIS
PD5948	BCR	cna	gain	BASIS
PD7067	BCR	cna	gain	BASIS
PD7215	BCR	cna	gain	BASIS
PD8621	BCR	cna	gain	BASIS
PD9004	BCR	cna	gain	BASIS
PD9702	BCR	cna	gain	BASIS
TCGA-AN-A0XU	COL11A1	cna	hetloss	TCGA

TCGA-BH-A0AW	COL11A1	cna	gain	TCGA
TCGA-BH-A0C0	COL11A1	cna	hetloss	TCGA
TCGA-BH-A1FU	COL11A1	cna	gain	TCGA
TCGA-E2-A1L7	COL11A1	cna	gain	TCGA
TCGA-LL-A5YP	COL11A1	cna	hetloss	TCGA
MB-0346	COL11A1	cna	hetloss	METABRIC
MB-2827	COL11A1	cna	hetloss	METABRIC
MB-7038	COL11A1	cna	gain	METABRIC
PD11327	COL11A1	cna	gain	BASIS
PD11742	COL11A1	cna	hetloss	BASIS
PD13296	COL11A1	cna	hetloss	BASIS
PD13299	COL11A1	cna	gain	BASIS
PD22355	COL11A1	cna	hetloss	BASIS
PD23574	COL11A1	cna	gain	BASIS
PD24186	COL11A1	cna	gain	BASIS
PD24202	COL11A1	cna	gain	BASIS
PD24206	COL11A1	cna	gain	BASIS
PD3890	COL11A1	cna	hetloss	BASIS
PD3905	COL11A1	cna	amp	BASIS
PD4005	COL11A1	cna	hetloss	BASIS
PD4006	COL11A1	cna	hetloss	BASIS
PD4107	COL11A1	cna	gain	BASIS
PD5930	COL11A1	cna	gain	BASIS
PD5945	COL11A1	cna	gain	BASIS
PD5948	COL11A1	cna	gain	BASIS
PD6413	COL11A1	cna	hetloss	BASIS
PD7067	COL11A1	cna	gain	BASIS
PD7215	COL11A1	cna	gain	BASIS
PD8621	COL11A1	cna	gain	BASIS
PD9585	COL11A1	cna	hetloss	BASIS
PD9702	COL11A1	cna	gain	BASIS
TCGA-AN-A0XU	CREB1	cna	gain	TCGA
TCGA-AO-A0JL	CREB1	cna	gain	TCGA
TCGA-BH-A0AW	CREB1	cna	hetloss	TCGA
TCGA-C8-A12L	CREB1	cna	gain	TCGA
TCGA-D8-A27M	CREB1	cna	gain	TCGA
TCGA-E9-A1NC	CREB1	cna	hetloss	TCGA
MB-2827	CREB1	cna	hetloss	METABRIC
MB-6098	CREB1	cna	hetloss	METABRIC
MB-6271	CREB1	cna	hetloss	METABRIC
MB-7032	CREB1	cna	gain	METABRIC
MB-0420	CREB1	cna	gain	METABRIC
PD10014	CREB1	cna	gain	BASIS
PD11327	CREB1	cna	hetloss	BASIS
PD13297	CREB1	cna	hetloss	BASIS
PD23578	CREB1	cna	hetloss	BASIS
PD24186	CREB1	cna	gain	BASIS

PD24206	CREB1	cna	gain	BASIS
PD3890	CREB1	cna	hetloss	BASIS
PD3905	CREB1	cna	gain	BASIS
PD4006	CREB1	cna	gain	BASIS
PD4107	CREB1	cna	gain	BASIS
PD4967	CREB1	cna	hetloss	BASIS
PD5935	CREB1	cna	gain	BASIS
PD5945	CREB1	cna	gain	BASIS
PD5948	CREB1	cna	gain	BASIS
PD6413	CREB1	cna	hetloss	BASIS
PD6731	CREB1	cna	hetloss	BASIS
PD7067	CREB1	cna	gain	BASIS
PD7215	CREB1	cna	gain	BASIS
PD9004	CREB1	cna	gain	BASIS
PD9585	CREB1	cna	hetloss	BASIS
PD9702	CREB1	cna	gain	BASIS
TCGA-A2-A25B	DNMT3A	cna	hetloss	TCGA
TCGA-AN-A0XU	DNMT3A	cna	gain	TCGA
TCGA-BH-A0AW	DNMT3A	cna	gain	TCGA
TCGA-BH-A0C0	DNMT3A	cna	gain	TCGA
TCGA-C8-A12L	DNMT3A	cna	gain	TCGA
TCGA-D8-A27M	DNMT3A	cna	hetloss	TCGA
TCGA-LL-A5YP	DNMT3A	cna	gain	TCGA
MB-0346	DNMT3A	cna	gain	METABRIC
MB-2827	DNMT3A	cna	hetloss	METABRIC
MB-5465	DNMT3A	cna	amp	METABRIC
MB-6271	DNMT3A	cna	hetloss	METABRIC
MB-7038	DNMT3A	cna	gain	METABRIC
PD10014	DNMT3A	cna	gain	BASIS
PD11327	DNMT3A	cna	gain	BASIS
PD13299	DNMT3A	cna	gain	BASIS
PD22355	DNMT3A	cna	gain	BASIS
PD23562	DNMT3A	cna	gain	BASIS
PD23578	DNMT3A	cna	gain	BASIS
PD24186	DNMT3A	cna	gain	BASIS
PD3890	DNMT3A	cna	hetloss	BASIS
PD3905	DNMT3A	cna	gain	BASIS
PD4006	DNMT3A	cna	gain	BASIS
PD4826	DNMT3A	cna	hetloss	BASIS
PD5930	DNMT3A	cna	gain	BASIS
PD5935	DNMT3A	cna	gain	BASIS
PD5945	DNMT3A	cna	gain	BASIS
PD5948	DNMT3A	cna	gain	BASIS
PD6406	DNMT3A	cna	hetloss	BASIS
PD7067	DNMT3A	cna	gain	BASIS
PD9004	DNMT3A	cna	gain	BASIS
PD9585	DNMT3A	cna	hetloss	BASIS

PD9702	DNMT3A	cna	gain	BASIS
TCGA-AN-A0XU	EPHA4	cna	hetloss	TCGA
TCGA-AO-A0JL	EPHA4	cna	gain	TCGA
TCGA-BH-A0AW	EPHA4	cna	hetloss	TCGA
TCGA-C8-A12L	EPHA4	cna	hetloss	TCGA
TCGA-D8-A27M	EPHA4	cna	gain	TCGA
TCGA-E2-A1L7	EPHA4	cna	hetloss	TCGA
TCGA-E9-A1NC	EPHA4	cna	hetloss	TCGA
MB-2827	EPHA4	cna	hetloss	METABRIC
MB-6060	EPHA4	cna	hetloss	METABRIC
MB-6098	EPHA4	cna	hetloss	METABRIC
MB-6271	EPHA4	cna	hetloss	METABRIC
MB-7038	EPHA4	cna	gain	METABRIC
PD10014	EPHA4	cna	gain	BASIS
PD11327	EPHA4	cna	gain	BASIS
PD13296	EPHA4	cna	hetloss	BASIS
PD13297	EPHA4	cna	hetloss	BASIS
PD23578	EPHA4	cna	hetloss	BASIS
PD24186	EPHA4	cna	gain	BASIS
PD24337	EPHA4	cna	hetloss	BASIS
PD3890	EPHA4	cna	hetloss	BASIS
PD3905	EPHA4	cna	gain	BASIS
PD4107	EPHA4	cna	gain	BASIS
PD4967	EPHA4	cna	hetloss	BASIS
PD5935	EPHA4	cna	gain	BASIS
PD5945	EPHA4	cna	gain	BASIS
PD5948	EPHA4	cna	gain	BASIS
PD6413	EPHA4	cna	hetloss	BASIS
PD6731	EPHA4	cna	hetloss	BASIS
PD7067	EPHA4	cna	gain	BASIS
PD7215	EPHA4	cna	gain	BASIS
PD8980	EPHA4	cna	hetloss	BASIS
PD9585	EPHA4	cna	hetloss	BASIS
TCGA-AO-A0JL	EPHA6	cna	gain	TCGA
TCGA-BH-A0C0	EPHA6	cna	hetloss	TCGA
TCGA-BH-A18R	EPHA6	cna	gain	TCGA
TCGA-BH-A1FU	EPHA6	cna	hetloss	TCGA
TCGA-C8-A12L	EPHA6	cna	gain	TCGA
TCGA-E2-A1L7	EPHA6	cna	hetloss	TCGA
TCGA-LL-A5YP	EPHA6	cna	gain	TCGA
MB-0346	EPHA6	cna	hetloss	METABRIC
MTS-T0064	EPHA6	cna	gain	METABRIC
PD11327	EPHA6	cna	gain	BASIS
PD13296	EPHA6	cna	hetloss	BASIS
PD13299	EPHA6	cna	gain	BASIS
PD23561	EPHA6	cna	hetloss	BASIS
PD23562	EPHA6	cna	gain	BASIS

PD23574	EPHA6	cna	gain	BASIS
PD24186	EPHA6	cna	gain	BASIS
PD24206	EPHA6	cna	gain	BASIS
PD3905	EPHA6	cna	gain	BASIS
PD4005	EPHA6	cna	hetloss	BASIS
PD4006	EPHA6	cna	hetloss	BASIS
PD4107	EPHA6	cna	gain	BASIS
PD4826	EPHA6	cna	gain	BASIS
PD5930	EPHA6	cna	hetloss	BASIS
PD5935	EPHA6	cna	gain	BASIS
PD5945	EPHA6	cna	amp	BASIS
PD5948	EPHA6	cna	gain	BASIS
PD7067	EPHA6	cna	gain	BASIS
PD7215	EPHA6	cna	gain	BASIS
PD8621	EPHA6	cna	gain	BASIS
PD8980	EPHA6	cna	gain	BASIS
PD9004	EPHA6	cna	gain	BASIS
PD9702	EPHA6	cna	gain	BASIS
TCGA-AO-A0JL	ERBB4	cna	gain	TCGA
TCGA-BH-A0AW	ERBB4	cna	hetloss	TCGA
TCGA-C8-A12L	ERBB4	cna	gain	TCGA
TCGA-D8-A27M	ERBB4	cna	amp	TCGA
TCGA-E2-A1L7	ERBB4	cna	hetloss	TCGA
TCGA-E9-A1NC	ERBB4	cna	hetloss	TCGA
MB-2827	ERBB4	cna	hetloss	METABRIC
MB-5465	ERBB4	cna	gain	METABRIC
MB-6098	ERBB4	cna	hetloss	METABRIC
MB-6271	ERBB4	cna	hetloss	METABRIC
MB-7032	ERBB4	cna	gain	METABRIC
PD10014	ERBB4	cna	gain	BASIS
PD11327	ERBB4	cna	gain	BASIS
PD13296	ERBB4	cna	hetloss	BASIS
PD13297	ERBB4	cna	hetloss	BASIS
PD23578	ERBB4	cna	hetloss	BASIS
PD24186	ERBB4	cna	gain	BASIS
PD24206	ERBB4	cna	gain	BASIS
PD3890	ERBB4	cna	homdel	BASIS
PD3905	ERBB4	cna	gain	BASIS
PD4006	ERBB4	cna	hetloss	BASIS
PD4107	ERBB4	cna	gain	BASIS
PD4967	ERBB4	cna	hetloss	BASIS
PD5935	ERBB4	cna	gain	BASIS
PD5945	ERBB4	cna	gain	BASIS
PD5948	ERBB4	cna	gain	BASIS
PD6413	ERBB4	cna	hetloss	BASIS
PD6731	ERBB4	cna	hetloss	BASIS
PD7067	ERBB4	cna	gain	BASIS

PD7215	ERBB4	cna	gain	BASIS
PD9585	ERBB4	cna	hetloss	BASIS
PD9702	ERBB4	cna	gain	BASIS
TCGA-AN-A0XU	EXT2	cna	hetloss	TCGA
TCGA-AO-A0JL	EXT2	cna	hetloss	TCGA
TCGA-BH-A0C0	EXT2	cna	hetloss	TCGA
TCGA-C8-A12L	EXT2	cna	hetloss	TCGA
TCGA-LL-A5YP	EXT2	cna	gain	TCGA
MB-0346	EXT2	cna	hetloss	METABRIC
MB-6060	EXT2	cna	gain	METABRIC
MB-6098	EXT2	cna	hetloss	METABRIC
MB-6271	EXT2	cna	gain	METABRIC
MB-7048	EXT2	cna	hetloss	METABRIC
PD10014	EXT2	cna	hetloss	BASIS
PD13296	EXT2	cna	gain	BASIS
PD23574	EXT2	cna	gain	BASIS
PD24186	EXT2	cna	gain	BASIS
PD24202	EXT2	cna	amp	BASIS
PD24206	EXT2	cna	gain	BASIS
PD3905	EXT2	cna	gain	BASIS
PD4005	EXT2	cna	gain	BASIS
PD4006	EXT2	cna	gain	BASIS
PD4107	EXT2	cna	gain	BASIS
PD4826	EXT2	cna	gain	BASIS
PD5930	EXT2	cna	gain	BASIS
PD5935	EXT2	cna	gain	BASIS
PD5945	EXT2	cna	gain	BASIS
PD5948	EXT2	cna	gain	BASIS
PD6413	EXT2	cna	hetloss	BASIS
PD6731	EXT2	cna	hetloss	BASIS
PD7215	EXT2	cna	amp	BASIS
PD8621	EXT2	cna	gain	BASIS
PD8980	EXT2	cna	hetloss	BASIS
PD9585	EXT2	cna	hetloss	BASIS
PD9702	EXT2	cna	gain	BASIS
TCGA-A2-A25B	FANCG	cna	hetloss	TCGA
TCGA-AN-A0XU	FANCG	cna	hetloss	TCGA
TCGA-AO-A0JL	FANCG	cna	gain	TCGA
TCGA-BH-A0AW	FANCG	cna	hetloss	TCGA
TCGA-BH-A1FU	FANCG	cna	hetloss	TCGA
TCGA-C8-A12L	FANCG	cna	hetloss	TCGA
TCGA-LL-A5YP	FANCG	cna	hetloss	TCGA
MB-0346	FANCG	cna	hetloss	METABRIC
MB-0420	FANCG	cna	hetloss	METABRIC
PD11327	FANCG	cna	hetloss	BASIS
PD13297	FANCG	cna	hetloss	BASIS
PD13299	FANCG	cna	amp	BASIS

PD22355	FANCG	cna	gain	BASIS
PD23562	FANCG	cna	gain	BASIS
PD23574	FANCG	cna	amp	BASIS
PD24202	FANCG	cna	hetloss	BASIS
PD24337	FANCG	cna	hetloss	BASIS
PD3905	FANCG	cna	gain	BASIS
PD4006	FANCG	cna	gain	BASIS
PD4107	FANCG	cna	gain	BASIS
PD4826	FANCG	cna	gain	BASIS
PD4967	FANCG	cna	hetloss	BASIS
PD5930	FANCG	cna	gain	BASIS
PD5935	FANCG	cna	gain	BASIS
PD5945	FANCG	cna	amp	BASIS
PD7067	FANCG	cna	gain	BASIS
PD7215	FANCG	cna	hetloss	BASIS
PD8621	FANCG	cna	gain	BASIS
PD8980	FANCG	cna	hetloss	BASIS
PD9004	FANCG	cna	gain	BASIS
PD9585	FANCG	cna	hetloss	BASIS
PD9702	FANCG	cna	gain	BASIS
TCGA-A2-A25B	FGFR4	cna	hetloss	TCGA
TCGA-AN-A0XU	FGFR4	cna	hetloss	TCGA
TCGA-AO-A0JL	FGFR4	cna	hetloss	TCGA
TCGA-BH-A0C0	FGFR4	cna	hetloss	TCGA
TCGA-BH-A1FU	FGFR4	cna	hetloss	TCGA
TCGA-C8-A12L	FGFR4	cna	hetloss	TCGA
TCGA-D8-A27M	FGFR4	cna	hetloss	TCGA
TCGA-E2-A1L7	FGFR4	cna	hetloss	TCGA
MB-5070	FGFR4	cna	hetloss	METABRIC
MB-6098	FGFR4	cna	hetloss	METABRIC
MB-7038	FGFR4	cna	gain	METABRIC
PD10014	FGFR4	cna	hetloss	BASIS
PD13296	FGFR4	cna	hetloss	BASIS
PD13297	FGFR4	cna	hetloss	BASIS
PD13771	FGFR4	cna	gain	BASIS
PD22355	FGFR4	cna	hetloss	BASIS
PD23578	FGFR4	cna	hetloss	BASIS
PD24186	FGFR4	cna	gain	BASIS
PD24206	FGFR4	cna	gain	BASIS
PD3905	FGFR4	cna	gain	BASIS
PD4005	FGFR4	cna	hetloss	BASIS
PD4006	FGFR4	cna	gain	BASIS
PD4107	FGFR4	cna	hetloss	BASIS
PD4826	FGFR4	cna	gain	BASIS
PD5945	FGFR4	cna	gain	BASIS
PD5948	FGFR4	cna	hetloss	BASIS
PD6406	FGFR4	cna	hetloss	BASIS

PD6413	FGFR4	cna	hetloss	BASIS
PD7067	FGFR4	cna	gain	BASIS
PD7215	FGFR4	cna	amp	BASIS
PD8980	FGFR4	cna	gain	BASIS
PD9702	FGFR4	cna	gain	BASIS
TCGA-BH-A0C0	GSK3B	cna	hetloss	TCGA
TCGA-BH-A18R	GSK3B	cna	gain	TCGA
TCGA-BH-A1FU	GSK3B	cna	hetloss	TCGA
TCGA-C8-A12L	GSK3B	cna	hetloss	TCGA
TCGA-E2-A1L7	GSK3B	cna	hetloss	TCGA
TCGA-LL-A5YP	GSK3B	cna	gain	TCGA
MB-0346	GSK3B	cna	hetloss	METABRIC
MB-5070	GSK3B	cna	hetloss	METABRIC
MB-5107	GSK3B	cna	gain	METABRIC
MB-5465	GSK3B	cna	gain	METABRIC
MB-6098	GSK3B	cna	hetloss	METABRIC
MTS-T0064	GSK3B	cna	hetloss	METABRIC
PD13296	GSK3B	cna	hetloss	BASIS
PD13299	GSK3B	cna	gain	BASIS
PD23561	GSK3B	cna	hetloss	BASIS
PD23574	GSK3B	cna	gain	BASIS
PD24186	GSK3B	cna	gain	BASIS
PD24206	GSK3B	cna	gain	BASIS
PD3905	GSK3B	cna	gain	BASIS
PD4005	GSK3B	cna	hetloss	BASIS
PD4107	GSK3B	cna	gain	BASIS
PD4826	GSK3B	cna	gain	BASIS
PD5935	GSK3B	cna	gain	BASIS
PD5945	GSK3B	cna	gain	BASIS
PD5948	GSK3B	cna	gain	BASIS
PD6731	GSK3B	cna	gain	BASIS
PD7067	GSK3B	cna	gain	BASIS
PD7215	GSK3B	cna	gain	BASIS
PD8621	GSK3B	cna	gain	BASIS
PD8980	GSK3B	cna	gain	BASIS
PD9004	GSK3B	cna	gain	BASIS
PD9702	GSK3B	cna	gain	BASIS
TCGA-A2-A25B	HMGA2	cna	gain	TCGA
TCGA-AO-A0JL	HMGA2	cna	hetloss	TCGA
TCGA-BH-A18R	HMGA2	cna	gain	TCGA
TCGA-BH-A1FU	HMGA2	cna	hetloss	TCGA
TCGA-C8-A12L	HMGA2	cna	gain	TCGA
TCGA-E2-A1L7	HMGA2	cna	hetloss	TCGA
TCGA-LL-A5YP	HMGA2	cna	hetloss	TCGA
MB-0346	HMGA2	cna	gain	METABRIC
MB-2827	HMGA2	cna	hetloss	METABRIC
MB-5107	HMGA2	cna	gain	METABRIC

MB-5465	HMGA2	cna	gain	METABRIC
MB-6098	HMGA2	cna	gain	METABRIC
MB-0420	HMGA2	cna	hetloss	METABRIC
MTS-T0064	HMGA2	cna	gain	METABRIC
PD10014	HMGA2	cna	gain	BASIS
PD22355	HMGA2	cna	hetloss	BASIS
PD23578	HMGA2	cna	gain	BASIS
PD24186	HMGA2	cna	gain	BASIS
PD24202	HMGA2	cna	hetloss	BASIS
PD24206	HMGA2	cna	gain	BASIS
PD3890	HMGA2	cna	hetloss	BASIS
PD3905	HMGA2	cna	gain	BASIS
PD4107	HMGA2	cna	gain	BASIS
PD4826	HMGA2	cna	gain	BASIS
PD5945	HMGA2	cna	gain	BASIS
PD6406	HMGA2	cna	hetloss	BASIS
PD6413	HMGA2	cna	hetloss	BASIS
PD6731	HMGA2	cna	hetloss	BASIS
PD7067	HMGA2	cna	gain	BASIS
PD8621	HMGA2	cna	hetloss	BASIS
PD9585	HMGA2	cna	hetloss	BASIS
PD9702	HMGA2	cna	gain	BASIS
TCGA-A2-A25B	HNRNPA2B1	cna	gain	TCGA
TCGA-BH-A0AW	HNRNPA2B1	cna	gain	TCGA
TCGA-BH-A0C0	HNRNPA2B1	cna	hetloss	TCGA
TCGA-C8-A12L	HNRNPA2B1	cna	gain	TCGA
TCGA-D8-A27M	HNRNPA2B1	cna	hetloss	TCGA
TCGA-LL-A5YP	HNRNPA2B1	cna	hetloss	TCGA
MB-0346	HNRNPA2B1	cna	gain	METABRIC
MB-6098	HNRNPA2B1	cna	hetloss	METABRIC
MB-0420	HNRNPA2B1	cna	hetloss	METABRIC
PD10014	HNRNPA2B1	cna	hetloss	BASIS
PD11327	HNRNPA2B1	cna	gain	BASIS
PD13296	HNRNPA2B1	cna	gain	BASIS
PD13297	HNRNPA2B1	cna	hetloss	BASIS
PD22355	HNRNPA2B1	cna	hetloss	BASIS
PD23574	HNRNPA2B1	cna	gain	BASIS
PD23578	HNRNPA2B1	cna	hetloss	BASIS
PD24186	HNRNPA2B1	cna	gain	BASIS
PD24206	HNRNPA2B1	cna	gain	BASIS
PD24337	HNRNPA2B1	cna	hetloss	BASIS
PD3905	HNRNPA2B1	cna	gain	BASIS
PD4005	HNRNPA2B1	cna	hetloss	BASIS
PD4006	HNRNPA2B1	cna	gain	BASIS
PD4107	HNRNPA2B1	cna	amp	BASIS
PD4826	HNRNPA2B1	cna	gain	BASIS
PD5935	HNRNPA2B1	cna	gain	BASIS

PD5945	HNRNPA2B1	cna	amp	BASIS
PD6406	HNRNPA2B1	cna	hetloss	BASIS
PD6413	HNRNPA2B1	cna	gain	BASIS
PD7215	HNRNPA2B1	cna	gain	BASIS
PD8621	HNRNPA2B1	cna	gain	BASIS
PD9585	HNRNPA2B1	cna	gain	BASIS
PD9702	HNRNPA2B1	cna	gain	BASIS
TCGA-A2-A25B	IGFBP7	cna	hetloss	TCGA
TCGA-AO-A0JL	IGFBP7	cna	gain	TCGA
TCGA-BH-A0C0	IGFBP7	cna	gain	TCGA
TCGA-BH-A1FU	IGFBP7	cna	gain	TCGA
TCGA-C8-A12L	IGFBP7	cna	amp	TCGA
TCGA-D8-A27M	IGFBP7	cna	gain	TCGA
TCGA-E9-A1NC	IGFBP7	cna	hetloss	TCGA
TCGA-LL-A5YP	IGFBP7	cna	gain	TCGA
MB-5465	IGFBP7	cna	gain	METABRIC
MB-6060	IGFBP7	cna	gain	METABRIC
MB-6098	IGFBP7	cna	amp	METABRIC
MB-7038	IGFBP7	cna	gain	METABRIC
MB-0420	IGFBP7	cna	hetloss	METABRIC
PD11742	IGFBP7	cna	hetloss	BASIS
PD13296	IGFBP7	cna	hetloss	BASIS
PD13297	IGFBP7	cna	hetloss	BASIS
PD22355	IGFBP7	cna	hetloss	BASIS
PD23562	IGFBP7	cna	gain	BASIS
PD23574	IGFBP7	cna	gain	BASIS
PD24202	IGFBP7	cna	hetloss	BASIS
PD24206	IGFBP7	cna	gain	BASIS
PD3890	IGFBP7	cna	gain	BASIS
PD3905	IGFBP7	cna	gain	BASIS
PD4006	IGFBP7	cna	hetloss	BASIS
PD4107	IGFBP7	cna	gain	BASIS
PD4826	IGFBP7	cna	gain	BASIS
PD5945	IGFBP7	cna	gain	BASIS
PD5948	IGFBP7	cna	gain	BASIS
PD6413	IGFBP7	cna	gain	BASIS
PD7215	IGFBP7	cna	amp	BASIS
PD8980	IGFBP7	cna	hetloss	BASIS
PD9702	IGFBP7	cna	gain	BASIS
TCGA-AO-A0JL	INSR	cna	gain	TCGA
TCGA-BH-A0C0	INSR	cna	gain	TCGA
TCGA-BH-A1FU	INSR	cna	hetloss	TCGA
TCGA-C8-A12L	INSR	cna	hetloss	TCGA
TCGA-D8-A27M	INSR	cna	hetloss	TCGA
TCGA-EW-A1OX	INSR	cna	hetloss	TCGA
TCGA-LL-A5YP	INSR	cna	hetloss	TCGA
MB-0346	INSR	cna	gain	METABRIC

MB-2827	INSR	cna	hetloss	METABRIC
MB-5465	INSR	cna	gain	METABRIC
MB-6098	INSR	cna	hetloss	METABRIC
MB-7038	INSR	cna	hetloss	METABRIC
MB-0420	INSR	cna	hetloss	METABRIC
PD10014	INSR	cna	hetloss	BASIS
PD11327	INSR	cna	homdel	BASIS
PD14442	INSR	cna	hetloss	BASIS
PD23574	INSR	cna	gain	BASIS
PD24202	INSR	cna	gain	BASIS
PD24206	INSR	cna	homdel	BASIS
PD3905	INSR	cna	gain	BASIS
PD4006	INSR	cna	gain	BASIS
PD4107	INSR	cna	gain	BASIS
PD4826	INSR	cna	gain	BASIS
PD4967	INSR	cna	homdel	BASIS
PD5930	INSR	cna	gain	BASIS
PD5945	INSR	cna	amp	BASIS
PD5948	INSR	cna	gain	BASIS
PD7067	INSR	cna	gain	BASIS
PD7215	INSR	cna	amp	BASIS
PD8621	INSR	cna	gain	BASIS
PD8980	INSR	cna	gain	BASIS
PD9702	INSR	cna	gain	BASIS
TCGA-A2-A25B	KDR	cna	hetloss	TCGA
TCGA-AO-A0JL	KDR	cna	gain	TCGA
TCGA-BH-A0C0	KDR	cna	gain	TCGA
TCGA-BH-A1FU	KDR	cna	gain	TCGA
TCGA-C8-A12L	KDR	cna	gain	TCGA
TCGA-D8-A27M	KDR	cna	gain	TCGA
TCGA-E9-A1NC	KDR	cna	hetloss	TCGA
TCGA-LL-A5YP	KDR	cna	gain	TCGA
MB-5070	KDR	cna	hetloss	METABRIC
MB-5465	KDR	cna	gain	METABRIC
MB-6060	KDR	cna	gain	METABRIC
MB-6098	KDR	cna	amp	METABRIC
MB-7038	KDR	cna	gain	METABRIC
MB-0420	KDR	cna	hetloss	METABRIC
PD11327	KDR	cna	amp	BASIS
PD11742	KDR	cna	hetloss	BASIS
PD13296	KDR	cna	hetloss	BASIS
PD13297	KDR	cna	hetloss	BASIS
PD22355	KDR	cna	hetloss	BASIS
PD23574	KDR	cna	gain	BASIS
PD24206	KDR	cna	gain	BASIS
PD3890	KDR	cna	gain	BASIS
PD3905	KDR	cna	gain	BASIS

PD4006	KDR	cna	gain	BASIS
PD4107	KDR	cna	gain	BASIS
PD4826	KDR	cna	gain	BASIS
PD5935	KDR	cna	gain	BASIS
PD5945	KDR	cna	gain	BASIS
PD5948	KDR	cna	gain	BASIS
PD7215	KDR	cna	amp	BASIS
PD8980	KDR	cna	hetloss	BASIS
PD9702	KDR	cna	gain	BASIS
TCGA-A2-A25B	LRIG3	cna	gain	TCGA
TCGA-AO-A0JL	LRIG3	cna	hetloss	TCGA
TCGA-BH-A18R	LRIG3	cna	gain	TCGA
TCGA-BH-A1FU	LRIG3	cna	hetloss	TCGA
TCGA-C8-A12L	LRIG3	cna	gain	TCGA
TCGA-E2-A1L7	LRIG3	cna	hetloss	TCGA
TCGA-LL-A5YP	LRIG3	cna	hetloss	TCGA
MB-0346	LRIG3	cna	gain	METABRIC
MB-2827	LRIG3	cna	hetloss	METABRIC
MB-5107	LRIG3	cna	gain	METABRIC
MB-6060	LRIG3	cna	gain	METABRIC
MB-6098	LRIG3	cna	gain	METABRIC
MB-0420	LRIG3	cna	hetloss	METABRIC
PD13296	LRIG3	cna	hetloss	BASIS
PD13297	LRIG3	cna	hetloss	BASIS
PD22355	LRIG3	cna	hetloss	BASIS
PD23578	LRIG3	cna	gain	BASIS
PD24186	LRIG3	cna	gain	BASIS
PD24202	LRIG3	cna	hetloss	BASIS
PD24206	LRIG3	cna	amp	BASIS
PD3890	LRIG3	cna	hetloss	BASIS
PD3905	LRIG3	cna	gain	BASIS
PD4006	LRIG3	cna	hetloss	BASIS
PD4107	LRIG3	cna	hetloss	BASIS
PD4826	LRIG3	cna	gain	BASIS
PD5945	LRIG3	cna	gain	BASIS
PD6413	LRIG3	cna	hetloss	BASIS
PD6731	LRIG3	cna	hetloss	BASIS
PD7067	LRIG3	cna	gain	BASIS
PD8621	LRIG3	cna	hetloss	BASIS
PD9585	LRIG3	cna	hetloss	BASIS
PD9702	LRIG3	cna	gain	BASIS
TCGA-A2-A25B	MAP3K7	cna	hetloss	TCGA
TCGA-AN-A0XU	MAP3K7	cna	amp	TCGA
TCGA-AO-A0JL	MAP3K7	cna	hetloss	TCGA
TCGA-C8-A12L	MAP3K7	cna	gain	TCGA
MB-2827	MAP3K7	cna	amp	METABRIC
MB-5107	MAP3K7	cna	hetloss	METABRIC

MB-6060	MAP3K7	cna	hetloss	METABRIC
MB-6098	MAP3K7	cna	hetloss	METABRIC
MB-7032	MAP3K7	cna	hetloss	METABRIC
MB-7038	MAP3K7	cna	gain	METABRIC
MB-7048	MAP3K7	cna	hetloss	METABRIC
PD11327	MAP3K7	cna	gain	BASIS
PD13299	MAP3K7	cna	gain	BASIS
PD13771	MAP3K7	cna	hetloss	BASIS
PD23562	MAP3K7	cna	gain	BASIS
PD23574	MAP3K7	cna	gain	BASIS
PD24186	MAP3K7	cna	gain	BASIS
PD24206	MAP3K7	cna	gain	BASIS
PD24337	MAP3K7	cna	hetloss	BASIS
PD3905	MAP3K7	cna	gain	BASIS
PD4006	MAP3K7	cna	gain	BASIS
PD4107	MAP3K7	cna	hetloss	BASIS
PD4826	MAP3K7	cna	gain	BASIS
PD5935	MAP3K7	cna	gain	BASIS
PD5945	MAP3K7	cna	gain	BASIS
PD6731	MAP3K7	cna	hetloss	BASIS
PD7215	MAP3K7	cna	gain	BASIS
PD8621	MAP3K7	cna	gain	BASIS
PD8980	MAP3K7	cna	gain	BASIS
PD9004	MAP3K7	cna	gain	BASIS
PD9585	MAP3K7	cna	hetloss	BASIS
PD9702	MAP3K7	cna	gain	BASIS
TCGA-AN-A0XU	MYD88	cna	hetloss	TCGA
TCGA-BH-A0C0	MYD88	cna	hetloss	TCGA
TCGA-BH-A1FU	MYD88	cna	hetloss	TCGA
TCGA-C8-A12L	MYD88	cna	hetloss	TCGA
TCGA-D8-A27M	MYD88	cna	hetloss	TCGA
TCGA-E9-A1NC	MYD88	cna	hetloss	TCGA
TCGA-LL-A5YP	MYD88	cna	amp	TCGA
MB-2827	MYD88	cna	hetloss	METABRIC
MB-7038	MYD88	cna	hetloss	METABRIC
MB-7048	MYD88	cna	hetloss	METABRIC
MB-0420	MYD88	cna	hetloss	METABRIC
P-0009557-T01-IM5	MYD88	cna	homdel	MSK-IMPACT
PD10014	MYD88	cna	hetloss	BASIS
PD11742	MYD88	cna	gain	BASIS
PD13296	MYD88	cna	gain	BASIS
PD13299	MYD88	cna	gain	BASIS
PD13771	MYD88	cna	gain	BASIS
PD22355	MYD88	cna	hetloss	BASIS
PD23561	MYD88	cna	hetloss	BASIS
PD23574	MYD88	cna	gain	BASIS
PD23578	MYD88	cna	hetloss	BASIS

PD24202	MYD88	cna	hetloss	BASIS
PD24206	MYD88	cna	gain	BASIS
PD3890	MYD88	cna	hetloss	BASIS
PD3905	MYD88	cna	gain	BASIS
PD4826	MYD88	cna	gain	BASIS
PD5945	MYD88	cna	gain	BASIS
PD6406	MYD88	cna	hetloss	BASIS
PD6731	MYD88	cna	hetloss	BASIS
PD7067	MYD88	cna	gain	BASIS
PD8980	MYD88	cna	gain	BASIS
PD9585	MYD88	cna	hetloss	BASIS
TCGA-A2-A25B	NCOA1	cna	hetloss	TCGA
TCGA-AN-A0XU	NCOA1	cna	gain	TCGA
TCGA-BH-A0AW	NCOA1	cna	gain	TCGA
TCGA-BH-A0C0	NCOA1	cna	gain	TCGA
TCGA-C8-A12L	NCOA1	cna	gain	TCGA
TCGA-D8-A27M	NCOA1	cna	hetloss	TCGA
TCGA-LL-A5YP	NCOA1	cna	gain	TCGA
MB-0346	NCOA1	cna	gain	METABRIC
MB-2827	NCOA1	cna	hetloss	METABRIC
MB-5070	NCOA1	cna	gain	METABRIC
MB-5465	NCOA1	cna	amp	METABRIC
MB-6271	NCOA1	cna	hetloss	METABRIC
MB-7038	NCOA1	cna	gain	METABRIC
PD10014	NCOA1	cna	gain	BASIS
PD11327	NCOA1	cna	gain	BASIS
PD13299	NCOA1	cna	gain	BASIS
PD22355	NCOA1	cna	gain	BASIS
PD23578	NCOA1	cna	gain	BASIS
PD24186	NCOA1	cna	gain	BASIS
PD3890	NCOA1	cna	hetloss	BASIS
PD3905	NCOA1	cna	gain	BASIS
PD4006	NCOA1	cna	gain	BASIS
PD4826	NCOA1	cna	hetloss	BASIS
PD5930	NCOA1	cna	gain	BASIS
PD5935	NCOA1	cna	gain	BASIS
PD5945	NCOA1	cna	gain	BASIS
PD5948	NCOA1	cna	amp	BASIS
PD6406	NCOA1	cna	hetloss	BASIS
PD7067	NCOA1	cna	gain	BASIS
PD9004	NCOA1	cna	gain	BASIS
PD9585	NCOA1	cna	hetloss	BASIS
PD9702	NCOA1	cna	gain	BASIS
TCGA-AO-A0JL	NFE2L2	cna	gain	TCGA
TCGA-BH-A0AW	NFE2L2	cna	gain	TCGA
TCGA-D8-A27M	NFE2L2	cna	gain	TCGA
TCGA-LL-A5YP	NFE2L2	cna	gain	TCGA

MB-2827	NFE2L2	cna	hetloss	METABRIC
MB-5070	NFE2L2	cna	hetloss	METABRIC
MB-5107	NFE2L2	cna	hetloss	METABRIC
MB-6098	NFE2L2	cna	hetloss	METABRIC
MB-6271	NFE2L2	cna	hetloss	METABRIC
MB-7032	NFE2L2	cna	gain	METABRIC
MB-0420	NFE2L2	cna	gain	METABRIC
PD10014	NFE2L2	cna	gain	BASIS
PD11327	NFE2L2	cna	gain	BASIS
PD13299	NFE2L2	cna	gain	BASIS
PD22355	NFE2L2	cna	hetloss	BASIS
PD24186	NFE2L2	cna	gain	BASIS
PD24202	NFE2L2	cna	gain	BASIS
PD24206	NFE2L2	cna	gain	BASIS
PD3890	NFE2L2	cna	hetloss	BASIS
PD3905	NFE2L2	cna	gain	BASIS
PD4006	NFE2L2	cna	gain	BASIS
PD4107	NFE2L2	cna	gain	BASIS
PD4967	NFE2L2	cna	hetloss	BASIS
PD5935	NFE2L2	cna	gain	BASIS
PD5945	NFE2L2	cna	amp	BASIS
PD5948	NFE2L2	cna	gain	BASIS
PD6413	NFE2L2	cna	hetloss	BASIS
PD7067	NFE2L2	cna	gain	BASIS
PD7215	NFE2L2	cna	gain	BASIS
PD9004	NFE2L2	cna	gain	BASIS
PD9585	NFE2L2	cna	hetloss	BASIS
PD9702	NFE2L2	cna	gain	BASIS
TCGA-AN-A0XU	NOTCH2	cna	gain	TCGA
TCGA-AO-A0JL	NOTCH2	cna	gain	TCGA
TCGA-BH-A0AW	NOTCH2	cna	gain	TCGA
TCGA-BH-A0C0	NOTCH2	cna	hetloss	TCGA
TCGA-BH-A18R	NOTCH2	cna	gain	TCGA
TCGA-BH-A1FU	NOTCH2	cna	gain	TCGA
TCGA-C8-A12L	NOTCH2	cna	amp	TCGA
TCGA-E2-A1L7	NOTCH2	cna	gain	TCGA
TCGA-EW-A1OX	NOTCH2	cna	gain	TCGA
TCGA-LL-A5YP	NOTCH2	cna	amp	TCGA
MB-0346	NOTCH2	cna	hetloss	METABRIC
MB-2827	NOTCH2	cna	hetloss	METABRIC
MB-7032	NOTCH2	cna	gain	METABRIC
MB-7038	NOTCH2	cna	gain	METABRIC
PD11327	NOTCH2	cna	gain	BASIS
PD13296	NOTCH2	cna	gain	BASIS
PD13299	NOTCH2	cna	gain	BASIS
PD22355	NOTCH2	cna	hetloss	BASIS
PD23574	NOTCH2	cna	gain	BASIS

PD24186	NOTCH2	cna	gain	BASIS
PD24206	NOTCH2	cna	gain	BASIS
PD3905	NOTCH2	cna	gain	BASIS
PD4006	NOTCH2	cna	gain	BASIS
PD4107	NOTCH2	cna	gain	BASIS
PD5930	NOTCH2	cna	gain	BASIS
PD5945	NOTCH2	cna	amp	BASIS
PD5948	NOTCH2	cna	gain	BASIS
PD6406	NOTCH2	cna	hetloss	BASIS
PD7067	NOTCH2	cna	amp	BASIS
PD7215	NOTCH2	cna	gain	BASIS
PD8621	NOTCH2	cna	gain	BASIS
PD9702	NOTCH2	cna	gain	BASIS
TCGA-AN-A0XU	NOTCH4	cna	gain	TCGA
TCGA-AO-A0JL	NOTCH4	cna	gain	TCGA
TCGA-BH-A0AW	NOTCH4	cna	gain	TCGA
TCGA-BH-A0C0	NOTCH4	cna	hetloss	TCGA
TCGA-BH-A18R	NOTCH4	cna	gain	TCGA
TCGA-C8-A12L	NOTCH4	cna	hetloss	TCGA
TCGA-E2-A1L7	NOTCH4	cna	hetloss	TCGA
TCGA-E9-A1NC	NOTCH4	cna	amp	TCGA
MB-6098	NOTCH4	cna	hetloss	METABRIC
MB-7032	NOTCH4	cna	gain	METABRIC
PD10014	NOTCH4	cna	hetloss	BASIS
PD11327	NOTCH4	cna	hetloss	BASIS
PD13296	NOTCH4	cna	gain	BASIS
PD13299	NOTCH4	cna	gain	BASIS
PD23562	NOTCH4	cna	gain	BASIS
PD23578	NOTCH4	cna	gain	BASIS
PD24186	NOTCH4	cna	gain	BASIS
PD24206	NOTCH4	cna	gain	BASIS
PD3905	NOTCH4	cna	gain	BASIS
PD4006	NOTCH4	cna	gain	BASIS
PD4107	NOTCH4	cna	gain	BASIS
PD4826	NOTCH4	cna	gain	BASIS
PD5935	NOTCH4	cna	gain	BASIS
PD5945	NOTCH4	cna	amp	BASIS
PD5948	NOTCH4	cna	gain	BASIS
PD6731	NOTCH4	cna	hetloss	BASIS
PD7067	NOTCH4	cna	amp	BASIS
PD7215	NOTCH4	cna	gain	BASIS
PD8621	NOTCH4	cna	amp	BASIS
PD9004	NOTCH4	cna	gain	BASIS
PD9585	NOTCH4	cna	gain	BASIS
PD9702	NOTCH4	cna	amp	BASIS
TCGA-AN-A0XU	NR4A2	cna	hetloss	TCGA
TCGA-AO-A0JL	NR4A2	cna	gain	TCGA

TCGA-LL-A5YP	NR4A2	cna	gain	TCGA
MB-2827	NR4A2	cna	hetloss	METABRIC
MB-5107	NR4A2	cna	hetloss	METABRIC
MB-6098	NR4A2	cna	hetloss	METABRIC
MB-6271	NR4A2	cna	hetloss	METABRIC
MB-7032	NR4A2	cna	gain	METABRIC
PD11327	NR4A2	cna	gain	BASIS
PD13297	NR4A2	cna	gain	BASIS
PD13299	NR4A2	cna	gain	BASIS
PD22355	NR4A2	cna	hetloss	BASIS
PD23574	NR4A2	cna	gain	BASIS
PD23578	NR4A2	cna	hetloss	BASIS
PD24186	NR4A2	cna	gain	BASIS
PD24202	NR4A2	cna	hetloss	BASIS
PD24206	NR4A2	cna	gain	BASIS
PD3890	NR4A2	cna	hetloss	BASIS
PD3905	NR4A2	cna	gain	BASIS
PD4005	NR4A2	cna	hetloss	BASIS
PD4006	NR4A2	cna	hetloss	BASIS
PD4107	NR4A2	cna	gain	BASIS
PD4967	NR4A2	cna	hetloss	BASIS
PD5930	NR4A2	cna	hetloss	BASIS
PD5935	NR4A2	cna	gain	BASIS
PD5948	NR4A2	cna	gain	BASIS
PD6406	NR4A2	cna	hetloss	BASIS
PD6413	NR4A2	cna	hetloss	BASIS
PD7067	NR4A2	cna	gain	BASIS
PD7215	NR4A2	cna	gain	BASIS
PD8621	NR4A2	cna	hetloss	BASIS
PD9702	NR4A2	cna	gain	BASIS
TCGA-A2-A25B	NSD1	cna	hetloss	TCGA
TCGA-AN-A0XU	NSD1	cna	hetloss	TCGA
TCGA-AO-A0JL	NSD1	cna	hetloss	TCGA
TCGA-BH-A0C0	NSD1	cna	hetloss	TCGA
TCGA-BH-A1FU	NSD1	cna	hetloss	TCGA
TCGA-C8-A12L	NSD1	cna	hetloss	TCGA
TCGA-D8-A27M	NSD1	cna	hetloss	TCGA
TCGA-E2-A1L7	NSD1	cna	hetloss	TCGA
MB-5070	NSD1	cna	hetloss	METABRIC
MB-6098	NSD1	cna	hetloss	METABRIC
MB-7038	NSD1	cna	gain	METABRIC
PD10014	NSD1	cna	hetloss	BASIS
PD13296	NSD1	cna	hetloss	BASIS
PD13297	NSD1	cna	hetloss	BASIS
PD13771	NSD1	cna	gain	BASIS
PD22355	NSD1	cna	hetloss	BASIS
PD23578	NSD1	cna	hetloss	BASIS

PD24186	NSD1	cna	gain	BASIS
PD24206	NSD1	cna	gain	BASIS
PD3905	NSD1	cna	gain	BASIS
PD4005	NSD1	cna	hetloss	BASIS
PD4006	NSD1	cna	gain	BASIS
PD4107	NSD1	cna	hetloss	BASIS
PD4826	NSD1	cna	gain	BASIS
PD5945	NSD1	cna	gain	BASIS
PD5948	NSD1	cna	hetloss	BASIS
PD6406	NSD1	cna	hetloss	BASIS
PD6413	NSD1	cna	hetloss	BASIS
PD7067	NSD1	cna	gain	BASIS
PD7215	NSD1	cna	amp	BASIS
PD8980	NSD1	cna	gain	BASIS
PD9702	NSD1	cna	gain	BASIS
TCGA-A2-A25B	PATZ1	cna	hetloss	TCGA
TCGA-BH-A0C0	PATZ1	cna	gain	TCGA
TCGA-BH-A1FU	PATZ1	cna	gain	TCGA
TCGA-C8-A12L	PATZ1	cna	gain	TCGA
TCGA-D8-A27M	PATZ1	cna	gain	TCGA
TCGA-E9-A1NC	PATZ1	cna	gain	TCGA
TCGA-LL-A5YP	PATZ1	cna	gain	TCGA
MB-0346	PATZ1	cna	gain	METABRIC
MB-6098	PATZ1	cna	gain	METABRIC
PD11327	PATZ1	cna	gain	BASIS
PD13296	PATZ1	cna	gain	BASIS
PD13297	PATZ1	cna	gain	BASIS
PD13299	PATZ1	cna	gain	BASIS
PD14442	PATZ1	cna	hetloss	BASIS
PD23562	PATZ1	cna	gain	BASIS
PD23574	PATZ1	cna	gain	BASIS
PD24206	PATZ1	cna	homdel	BASIS
PD3905	PATZ1	cna	gain	BASIS
PD4006	PATZ1	cna	gain	BASIS
PD4107	PATZ1	cna	gain	BASIS
PD4826	PATZ1	cna	gain	BASIS
PD4967	PATZ1	cna	hetloss	BASIS
PD5930	PATZ1	cna	gain	BASIS
PD5935	PATZ1	cna	gain	BASIS
PD5945	PATZ1	cna	amp	BASIS
PD5948	PATZ1	cna	gain	BASIS
PD6731	PATZ1	cna	hetloss	BASIS
PD7067	PATZ1	cna	gain	BASIS
PD7215	PATZ1	cna	gain	BASIS
PD8621	PATZ1	cna	gain	BASIS
PD9004	PATZ1	cna	gain	BASIS
PD9702	PATZ1	cna	gain	BASIS

TCGA-A2-A25B	PAX5	cna	hetloss	TCGA
TCGA-AN-A0XU	PAX5	cna	hetloss	TCGA
TCGA-AO-A0JL	PAX5	cna	hetloss	TCGA
TCGA-BH-A0AW	PAX5	cna	hetloss	TCGA
TCGA-BH-A1FU	PAX5	cna	hetloss	TCGA
TCGA-C8-A12L	PAX5	cna	hetloss	TCGA
TCGA-E9-A1NC	PAX5	cna	gain	TCGA
TCGA-LL-A5YP	PAX5	cna	hetloss	TCGA
MB-0346	PAX5	cna	hetloss	METABRIC
MB-6098	PAX5	cna	hetloss	METABRIC
MB-0420	PAX5	cna	hetloss	METABRIC
MTS-T0064	PAX5	cna	amp	METABRIC
PD11327	PAX5	cna	hetloss	BASIS
PD13297	PAX5	cna	hetloss	BASIS
PD13299	PAX5	cna	gain	BASIS
PD23562	PAX5	cna	gain	BASIS
PD23574	PAX5	cna	amp	BASIS
PD3890	PAX5	cna	hetloss	BASIS
PD3905	PAX5	cna	gain	BASIS
PD4006	PAX5	cna	gain	BASIS
PD4107	PAX5	cna	gain	BASIS
PD4826	PAX5	cna	gain	BASIS
PD4967	PAX5	cna	hetloss	BASIS
PD5930	PAX5	cna	gain	BASIS
PD5945	PAX5	cna	amp	BASIS
PD7067	PAX5	cna	gain	BASIS
PD7215	PAX5	cna	hetloss	BASIS
PD8621	PAX5	cna	gain	BASIS
PD8980	PAX5	cna	hetloss	BASIS
PD9004	PAX5	cna	gain	BASIS
PD9585	PAX5	cna	hetloss	BASIS
PD9702	PAX5	cna	gain	BASIS
TCGA-A2-A25B	PHLDA1	cna	gain	TCGA
TCGA-AN-A0XU	PHLDA1	cna	hetloss	TCGA
TCGA-AO-A0JL	PHLDA1	cna	hetloss	TCGA
TCGA-BH-A18R	PHLDA1	cna	gain	TCGA
TCGA-BH-A1FU	PHLDA1	cna	hetloss	TCGA
TCGA-C8-A12L	PHLDA1	cna	gain	TCGA
TCGA-E2-A1L7	PHLDA1	cna	hetloss	TCGA
MB-0346	PHLDA1	cna	gain	METABRIC
MB-2827	PHLDA1	cna	hetloss	METABRIC
MB-5070	PHLDA1	cna	hetloss	METABRIC
MB-5107	PHLDA1	cna	gain	METABRIC
MB-5465	PHLDA1	cna	gain	METABRIC
MB-6098	PHLDA1	cna	hetloss	METABRIC
MTS-T0064	PHLDA1	cna	gain	METABRIC
PD11327	PHLDA1	cna	homdel	BASIS

PD13296	PHLDA1	cna	hetloss	BASIS
PD22355	PHLDA1	cna	hetloss	BASIS
PD23578	PHLDA1	cna	gain	BASIS
PD24202	PHLDA1	cna	hetloss	BASIS
PD24206	PHLDA1	cna	gain	BASIS
PD24337	PHLDA1	cna	gain	BASIS
PD3890	PHLDA1	cna	hetloss	BASIS
PD3905	PHLDA1	cna	gain	BASIS
PD4826	PHLDA1	cna	gain	BASIS
PD5945	PHLDA1	cna	gain	BASIS
PD6406	PHLDA1	cna	hetloss	BASIS
PD6413	PHLDA1	cna	hetloss	BASIS
PD6731	PHLDA1	cna	hetloss	BASIS
PD7067	PHLDA1	cna	gain	BASIS
PD8621	PHLDA1	cna	hetloss	BASIS
PD8980	PHLDA1	cna	hetloss	BASIS
PD9585	PHLDA1	cna	hetloss	BASIS
TCGA-A2-A25B	PMS2	cna	gain	TCGA
TCGA-BH-A0AW	PMS2	cna	gain	TCGA
TCGA-BH-A0C0	PMS2	cna	hetloss	TCGA
TCGA-C8-A12L	PMS2	cna	gain	TCGA
TCGA-E2-A1L7	PMS2	cna	hetloss	TCGA
TCGA-LL-A5YP	PMS2	cna	homdel	TCGA
MB-5465	PMS2	cna	gain	METABRIC
MB-6098	PMS2	cna	hetloss	METABRIC
MB-7038	PMS2	cna	hetloss	METABRIC
MB-7048	PMS2	cna	hetloss	METABRIC
MB-0420	PMS2	cna	hetloss	METABRIC
PD11327	PMS2	cna	homdel	BASIS
PD13771	PMS2	cna	gain	BASIS
PD22355	PMS2	cna	hetloss	BASIS
PD23574	PMS2	cna	gain	BASIS
PD23578	PMS2	cna	hetloss	BASIS
PD24202	PMS2	cna	gain	BASIS
PD24206	PMS2	cna	gain	BASIS
PD24337	PMS2	cna	hetloss	BASIS
PD3905	PMS2	cna	gain	BASIS
PD4006	PMS2	cna	gain	BASIS
PD4107	PMS2	cna	gain	BASIS
PD4826	PMS2	cna	gain	BASIS
PD5935	PMS2	cna	gain	BASIS
PD5945	PMS2	cna	gain	BASIS
PD6406	PMS2	cna	hetloss	BASIS
PD7067	PMS2	cna	gain	BASIS
PD7215	PMS2	cna	gain	BASIS
PD8621	PMS2	cna	gain	BASIS
PD8980	PMS2	cna	hetloss	BASIS

PD9004	PMS2	cna	gain	BASIS
PD9702	PMS2	cna	gain	BASIS
TCGA-AN-A0XU	RBM15	cna	hetloss	TCGA
TCGA-BH-A0AW	RBM15	cna	gain	TCGA
TCGA-BH-A0C0	RBM15	cna	hetloss	TCGA
TCGA-BH-A1FU	RBM15	cna	gain	TCGA
TCGA-E2-A1L7	RBM15	cna	gain	TCGA
TCGA-LL-A5YP	RBM15	cna	hetloss	TCGA
MB-0346	RBM15	cna	hetloss	METABRIC
MB-2827	RBM15	cna	hetloss	METABRIC
MB-7032	RBM15	cna	gain	METABRIC
MB-7038	RBM15	cna	gain	METABRIC
PD11327	RBM15	cna	gain	BASIS
PD11742	RBM15	cna	hetloss	BASIS
PD13296	RBM15	cna	hetloss	BASIS
PD13299	RBM15	cna	gain	BASIS
PD22355	RBM15	cna	hetloss	BASIS
PD23574	RBM15	cna	gain	BASIS
PD24186	RBM15	cna	gain	BASIS
PD24202	RBM15	cna	gain	BASIS
PD24206	RBM15	cna	gain	BASIS
PD3890	RBM15	cna	hetloss	BASIS
PD3905	RBM15	cna	gain	BASIS
PD4006	RBM15	cna	gain	BASIS
PD4107	RBM15	cna	gain	BASIS
PD5930	RBM15	cna	gain	BASIS
PD5945	RBM15	cna	gain	BASIS
PD5948	RBM15	cna	gain	BASIS
PD6413	RBM15	cna	hetloss	BASIS
PD7067	RBM15	cna	gain	BASIS
PD7215	RBM15	cna	gain	BASIS
PD8621	RBM15	cna	gain	BASIS
PD9585	RBM15	cna	hetloss	BASIS
PD9702	RBM15	cna	gain	BASIS
TCGA-A2-A25B	RMRP	cna	hetloss	TCGA
TCGA-AN-A0XU	RMRP	cna	hetloss	TCGA
TCGA-AO-A0JL	RMRP	cna	gain	TCGA
TCGA-BH-A0AW	RMRP	cna	hetloss	TCGA
TCGA-BH-A1FU	RMRP	cna	hetloss	TCGA
TCGA-C8-A12L	RMRP	cna	hetloss	TCGA
TCGA-E9-A1NC	RMRP	cna	gain	TCGA
TCGA-LL-A5YP	RMRP	cna	hetloss	TCGA
MB-0346	RMRP	cna	hetloss	METABRIC
MB-0420	RMRP	cna	hetloss	METABRIC
PD11327	RMRP	cna	hetloss	BASIS
PD13297	RMRP	cna	hetloss	BASIS
PD13299	RMRP	cna	amp	BASIS

PD23562	RMRP	cna	gain	BASIS
PD23574	RMRP	cna	amp	BASIS
PD24202	RMRP	cna	hetloss	BASIS
PD24337	RMRP	cna	gain	BASIS
PD3905	RMRP	cna	gain	BASIS
PD4006	RMRP	cna	gain	BASIS
PD4107	RMRP	cna	gain	BASIS
PD4826	RMRP	cna	gain	BASIS
PD4967	RMRP	cna	hetloss	BASIS
PD5930	RMRP	cna	gain	BASIS
PD5935	RMRP	cna	gain	BASIS
PD5945	RMRP	cna	amp	BASIS
PD7067	RMRP	cna	gain	BASIS
PD7215	RMRP	cna	hetloss	BASIS
PD8621	RMRP	cna	gain	BASIS
PD8980	RMRP	cna	hetloss	BASIS
PD9004	RMRP	cna	gain	BASIS
PD9585	RMRP	cna	hetloss	BASIS
PD9702	RMRP	cna	gain	BASIS
TCGA-A2-A25B	RUNX1	cna	hetloss	TCGA
TCGA-BH-A0C0	RUNX1	cna	hetloss	TCGA
TCGA-BH-A1FU	RUNX1	cna	gain	TCGA
TCGA-C8-A12L	RUNX1	cna	homdel	TCGA
TCGA-D8-A27M	RUNX1	cna	hetloss	TCGA
TCGA-LL-A5YP	RUNX1	cna	gain	TCGA
MB-0346	RUNX1	cna	gain	METABRIC
MB-2827	RUNX1	cna	amp	METABRIC
MB-5070	RUNX1	cna	gain	METABRIC
MB-6098	RUNX1	cna	gain	METABRIC
MB-7048	RUNX1	cna	hetloss	METABRIC
PD13296	RUNX1	cna	gain	BASIS
PD13297	RUNX1	cna	hetloss	BASIS
PD13771	RUNX1	cna	gain	BASIS
PD23562	RUNX1	cna	gain	BASIS
PD23578	RUNX1	cna	gain	BASIS
PD24186	RUNX1	cna	gain	BASIS
PD24202	RUNX1	cna	gain	BASIS
PD24206	RUNX1	cna	gain	BASIS
PD3905	RUNX1	cna	gain	BASIS
PD4107	RUNX1	cna	gain	BASIS
PD4826	RUNX1	cna	gain	BASIS
PD5930	RUNX1	cna	gain	BASIS
PD5945	RUNX1	cna	gain	BASIS
PD5948	RUNX1	cna	gain	BASIS
PD6731	RUNX1	cna	gain	BASIS
PD7067	RUNX1	cna	gain	BASIS
PD7215	RUNX1	cna	gain	BASIS

PD8621	RUNX1	cna	gain	BASIS
PD8980	RUNX1	cna	homdel	BASIS
PD9004	RUNX1	cna	amp	BASIS
PD9702	RUNX1	cna	amp	BASIS
TCGA-AN-A0XU	RYBP	cna	hetloss	TCGA
TCGA-BH-A0C0	RYBP	cna	hetloss	TCGA
TCGA-BH-A18R	RYBP	cna	homdel	TCGA
TCGA-BH-A1FU	RYBP	cna	hetloss	TCGA
TCGA-C8-A12L	RYBP	cna	hetloss	TCGA
TCGA-D8-A27M	RYBP	cna	hetloss	TCGA
TCGA-E2-A1L7	RYBP	cna	homdel	TCGA
TCGA-E9-A1NC	RYBP	cna	hetloss	TCGA
TCGA-LL-A5YP	RYBP	cna	gain	TCGA
MB-0346	RYBP	cna	hetloss	METABRIC
MB-2827	RYBP	cna	hetloss	METABRIC
MB-5465	RYBP	cna	hetloss	METABRIC
MB-6098	RYBP	cna	hetloss	METABRIC
MB-7048	RYBP	cna	hetloss	METABRIC
MB-0420	RYBP	cna	hetloss	METABRIC
MTS-T0064	RYBP	cna	gain	METABRIC
PD10014	RYBP	cna	hetloss	BASIS
PD11742	RYBP	cna	hetloss	BASIS
PD13296	RYBP	cna	hetloss	BASIS
PD13299	RYBP	cna	gain	BASIS
PD13771	RYBP	cna	hetloss	BASIS
PD23561	RYBP	cna	hetloss	BASIS
PD23574	RYBP	cna	gain	BASIS
PD23578	RYBP	cna	hetloss	BASIS
PD24202	RYBP	cna	hetloss	BASIS
PD24337	RYBP	cna	hetloss	BASIS
PD4107	RYBP	cna	gain	BASIS
PD5945	RYBP	cna	gain	BASIS
PD6406	RYBP	cna	hetloss	BASIS
PD6731	RYBP	cna	hetloss	BASIS
PD7067	RYBP	cna	gain	BASIS
PD9585	RYBP	cna	hetloss	BASIS
TCGA-AN-A0XU	SF3B1	cna	gain	TCGA
TCGA-AO-A0JL	SF3B1	cna	gain	TCGA
TCGA-BH-A0AW	SF3B1	cna	gain	TCGA
TCGA-C8-A12L	SF3B1	cna	gain	TCGA
TCGA-D8-A27M	SF3B1	cna	gain	TCGA
MB-2827	SF3B1	cna	hetloss	METABRIC
MB-6098	SF3B1	cna	hetloss	METABRIC
MB-6271	SF3B1	cna	hetloss	METABRIC
MB-7032	SF3B1	cna	gain	METABRIC
MB-0420	SF3B1	cna	amp	METABRIC
PD10014	SF3B1	cna	gain	BASIS

PD11327	SF3B1	cna	gain	BASIS
PD13296	SF3B1	cna	hetloss	BASIS
PD13297	SF3B1	cna	hetloss	BASIS
PD13299	SF3B1	cna	gain	BASIS
PD24186	SF3B1	cna	gain	BASIS
PD24206	SF3B1	cna	gain	BASIS
PD3890	SF3B1	cna	hetloss	BASIS
PD3905	SF3B1	cna	gain	BASIS
PD4006	SF3B1	cna	gain	BASIS
PD4107	SF3B1	cna	gain	BASIS
PD4967	SF3B1	cna	hetloss	BASIS
PD5935	SF3B1	cna	gain	BASIS
PD5945	SF3B1	cna	gain	BASIS
PD5948	SF3B1	cna	gain	BASIS
PD6413	SF3B1	cna	hetloss	BASIS
PD7067	SF3B1	cna	gain	BASIS
PD7215	SF3B1	cna	amp	BASIS
PD8621	SF3B1	cna	gain	BASIS
PD9004	SF3B1	cna	gain	BASIS
PD9585	SF3B1	cna	hetloss	BASIS
PD9702	SF3B1	cna	gain	BASIS
TCGA-AN-A0XU	SMAD3	cna	hetloss	TCGA
TCGA-AO-A0JL	SMAD3	cna	hetloss	TCGA
TCGA-BH-A0AW	SMAD3	cna	hetloss	TCGA
TCGA-BH-A0C0	SMAD3	cna	hetloss	TCGA
TCGA-D8-A27M	SMAD3	cna	hetloss	TCGA
TCGA-E2-A1L7	SMAD3	cna	gain	TCGA
TCGA-LL-A5YP	SMAD3	cna	gain	TCGA
MB-0346	SMAD3	cna	hetloss	METABRIC
MB-6060	SMAD3	cna	gain	METABRIC
MB-7038	SMAD3	cna	gain	METABRIC
MB-7048	SMAD3	cna	hetloss	METABRIC
PD11742	SMAD3	cna	hetloss	BASIS
PD13296	SMAD3	cna	hetloss	BASIS
PD13297	SMAD3	cna	hetloss	BASIS
PD22355	SMAD3	cna	hetloss	BASIS
PD23562	SMAD3	cna	gain	BASIS
PD23574	SMAD3	cna	gain	BASIS
PD23578	SMAD3	cna	gain	BASIS
PD24202	SMAD3	cna	hetloss	BASIS
PD24206	SMAD3	cna	gain	BASIS
PD3890	SMAD3	cna	hetloss	BASIS
PD3905	SMAD3	cna	gain	BASIS
PD4006	SMAD3	cna	gain	BASIS
PD5945	SMAD3	cna	gain	BASIS
PD5948	SMAD3	cna	gain	BASIS
PD6406	SMAD3	cna	hetloss	BASIS

PD6413	SMAD3	cna	gain	BASIS
PD7067	SMAD3	cna	gain	BASIS
PD7215	SMAD3	cna	gain	BASIS
PD8980	SMAD3	cna	gain	BASIS
PD9585	SMAD3	cna	hetloss	BASIS
PD9702	SMAD3	cna	gain	BASIS
TCGA-A2-A25B	SRC	cna	gain	TCGA
TCGA-AO-A0JL	SRC	cna	gain	TCGA
TCGA-BH-A0AW	SRC	cna	gain	TCGA
TCGA-BH-A0C0	SRC	cna	gain	TCGA
TCGA-C8-A12L	SRC	cna	gain	TCGA
TCGA-D8-A27M	SRC	cna	gain	TCGA
TCGA-E2-A1L7	SRC	cna	gain	TCGA
TCGA-E9-A1NC	SRC	cna	hetloss	TCGA
TCGA-LL-A5YP	SRC	cna	hetloss	TCGA
MB-0346	SRC	cna	gain	METABRIC
MB-5107	SRC	cna	gain	METABRIC
MB-6098	SRC	cna	hetloss	METABRIC
PD13296	SRC	cna	hetloss	BASIS
PD13297	SRC	cna	hetloss	BASIS
PD13299	SRC	cna	gain	BASIS
PD13771	SRC	cna	gain	BASIS
PD23578	SRC	cna	hetloss	BASIS
PD24186	SRC	cna	gain	BASIS
PD24202	SRC	cna	hetloss	BASIS
PD3905	SRC	cna	gain	BASIS
PD4005	SRC	cna	hetloss	BASIS
PD4107	SRC	cna	gain	BASIS
PD4826	SRC	cna	gain	BASIS
PD5935	SRC	cna	gain	BASIS
PD5945	SRC	cna	amp	BASIS
PD5948	SRC	cna	amp	BASIS
PD6731	SRC	cna	hetloss	BASIS
PD7067	SRC	cna	gain	BASIS
PD7215	SRC	cna	gain	BASIS
PD8980	SRC	cna	gain	BASIS
PD9004	SRC	cna	gain	BASIS
PD9585	SRC	cna	hetloss	BASIS
TCGA-A2-A25B	TAF1L	cna	hetloss	TCGA
TCGA-AN-A0XU	TAF1L	cna	hetloss	TCGA
TCGA-AO-A0JL	TAF1L	cna	gain	TCGA
TCGA-BH-A0AW	TAF1L	cna	hetloss	TCGA
TCGA-BH-A1FU	TAF1L	cna	hetloss	TCGA
TCGA-C8-A12L	TAF1L	cna	hetloss	TCGA
TCGA-EW-A1OX	TAF1L	cna	gain	TCGA
MB-0346	TAF1L	cna	hetloss	METABRIC
MB-5070	TAF1L	cna	hetloss	METABRIC

MB-0420	TAF1L	cna	hetloss	METABRIC
PD13297	TAF1L	cna	hetloss	BASIS
PD13299	TAF1L	cna	gain	BASIS
PD22355	TAF1L	cna	gain	BASIS
PD23574	TAF1L	cna	amp	BASIS
PD24202	TAF1L	cna	hetloss	BASIS
PD24337	TAF1L	cna	hetloss	BASIS
PD3905	TAF1L	cna	gain	BASIS
PD4005	TAF1L	cna	hetloss	BASIS
PD4006	TAF1L	cna	gain	BASIS
PD4826	TAF1L	cna	gain	BASIS
PD4967	TAF1L	cna	hetloss	BASIS
PD5930	TAF1L	cna	hetloss	BASIS
PD5935	TAF1L	cna	gain	BASIS
PD5945	TAF1L	cna	amp	BASIS
PD6406	TAF1L	cna	hetloss	BASIS
PD7067	TAF1L	cna	gain	BASIS
PD7215	TAF1L	cna	hetloss	BASIS
PD8621	TAF1L	cna	gain	BASIS
PD8980	TAF1L	cna	hetloss	BASIS
PD9004	TAF1L	cna	gain	BASIS
PD9585	TAF1L	cna	hetloss	BASIS
PD9702	TAF1L	cna	gain	BASIS
TCGA-A2-A25B	TAL1	cna	gain	TCGA
TCGA-AN-A0XU	TAL1	cna	gain	TCGA
TCGA-AO-A0JL	TAL1	cna	hetloss	TCGA
TCGA-BH-A0AW	TAL1	cna	gain	TCGA
TCGA-BH-A0C0	TAL1	cna	hetloss	TCGA
TCGA-BH-A1FU	TAL1	cna	gain	TCGA
TCGA-D8-A27M	TAL1	cna	gain	TCGA
TCGA-E2-A1L7	TAL1	cna	gain	TCGA
TCGA-LL-A5YP	TAL1	cna	hetloss	TCGA
MB-0346	TAL1	cna	hetloss	METABRIC
MB-2827	TAL1	cna	hetloss	METABRIC
MB-6060	TAL1	cna	hetloss	METABRIC
MB-6098	TAL1	cna	gain	METABRIC
MB-7038	TAL1	cna	gain	METABRIC
MB-0420	TAL1	cna	hetloss	METABRIC
PD11742	TAL1	cna	hetloss	BASIS
PD13296	TAL1	cna	gain	BASIS
PD13299	TAL1	cna	gain	BASIS
PD24186	TAL1	cna	amp	BASIS
PD24206	TAL1	cna	gain	BASIS
PD3905	TAL1	cna	gain	BASIS
PD4006	TAL1	cna	gain	BASIS
PD4107	TAL1	cna	gain	BASIS
PD4826	TAL1	cna	gain	BASIS

PD5935	TAL1	cna	gain	BASIS
PD5945	TAL1	cna	amp	BASIS
PD5948	TAL1	cna	gain	BASIS
PD7067	TAL1	cna	gain	BASIS
PD7215	TAL1	cna	gain	BASIS
PD8621	TAL1	cna	gain	BASIS
PD9004	TAL1	cna	gain	BASIS
PD9702	TAL1	cna	gain	BASIS
TCGA-AO-A0JL	TPM4	cna	gain	TCGA
TCGA-BH-A0C0	TPM4	cna	gain	TCGA
TCGA-BH-A1FU	TPM4	cna	hetloss	TCGA
TCGA-C8-A12L	TPM4	cna	gain	TCGA
TCGA-D8-A27M	TPM4	cna	hetloss	TCGA
TCGA-EW-A1OX	TPM4	cna	hetloss	TCGA
TCGA-LL-A5YP	TPM4	cna	hetloss	TCGA
MB-0346	TPM4	cna	hetloss	METABRIC
MB-2827	TPM4	cna	hetloss	METABRIC
MB-6098	TPM4	cna	hetloss	METABRIC
MB-0420	TPM4	cna	hetloss	METABRIC
MTS-T0064	TPM4	cna	amp	METABRIC
PD13299	TPM4	cna	gain	BASIS
PD14442	TPM4	cna	hetloss	BASIS
PD22355	TPM4	cna	gain	BASIS
PD23562	TPM4	cna	gain	BASIS
PD23574	TPM4	cna	gain	BASIS
PD24186	TPM4	cna	gain	BASIS
PD24206	TPM4	cna	hetloss	BASIS
PD3905	TPM4	cna	gain	BASIS
PD4006	TPM4	cna	amp	BASIS
PD4107	TPM4	cna	gain	BASIS
PD4826	TPM4	cna	gain	BASIS
PD4967	TPM4	cna	hetloss	BASIS
PD5930	TPM4	cna	gain	BASIS
PD5935	TPM4	cna	gain	BASIS
PD5945	TPM4	cna	amp	BASIS
PD5948	TPM4	cna	gain	BASIS
PD6413	TPM4	cna	gain	BASIS
PD7067	TPM4	cna	gain	BASIS
PD7215	TPM4	cna	amp	BASIS
PD9702	TPM4	cna	gain	BASIS
TCGA-AN-A0XU	WT1	cna	gain	TCGA
TCGA-AO-A0JL	WT1	cna	hetloss	TCGA
TCGA-BH-A0C0	WT1	cna	gain	TCGA
TCGA-C8-A12L	WT1	cna	hetloss	TCGA
TCGA-E2-A1L7	WT1	cna	gain	TCGA
TCGA-LL-A5YP	WT1	cna	gain	TCGA
MB-0346	WT1	cna	hetloss	METABRIC

MB-6060	WT1	cna	amp	METABRIC
MB-6098	WT1	cna	hetloss	METABRIC
MB-6271	WT1	cna	gain	METABRIC
MB-7048	WT1	cna	hetloss	METABRIC
PD10014	WT1	cna	hetloss	BASIS
PD11327	WT1	cna	homdel	BASIS
PD11742	WT1	cna	gain	BASIS
PD13296	WT1	cna	gain	BASIS
PD23578	WT1	cna	hetloss	BASIS
PD24186	WT1	cna	gain	BASIS
PD3905	WT1	cna	gain	BASIS
PD4006	WT1	cna	gain	BASIS
PD4107	WT1	cna	gain	BASIS
PD4826	WT1	cna	gain	BASIS
PD5930	WT1	cna	gain	BASIS
PD5935	WT1	cna	gain	BASIS
PD5945	WT1	cna	gain	BASIS
PD5948	WT1	cna	gain	BASIS
PD6413	WT1	cna	hetloss	BASIS
PD6731	WT1	cna	hetloss	BASIS
PD7215	WT1	cna	gain	BASIS
PD8621	WT1	cna	gain	BASIS
PD8980	WT1	cna	hetloss	BASIS
PD9585	WT1	cna	hetloss	BASIS
PD9702	WT1	cna	gain	BASIS
TCGA-A2-A25B	XBP1	cna	hetloss	TCGA
TCGA-BH-A0C0	XBP1	cna	gain	TCGA
TCGA-BH-A1FU	XBP1	cna	gain	TCGA
TCGA-C8-A12L	XBP1	cna	gain	TCGA
TCGA-D8-A27M	XBP1	cna	gain	TCGA
TCGA-E9-A1NC	XBP1	cna	gain	TCGA
TCGA-LL-A5YP	XBP1	cna	gain	TCGA
MB-0346	XBP1	cna	gain	METABRIC
MB-6060	XBP1	cna	gain	METABRIC
PD11327	XBP1	cna	gain	BASIS
PD13296	XBP1	cna	gain	BASIS
PD13297	XBP1	cna	gain	BASIS
PD13771	XBP1	cna	gain	BASIS
PD14442	XBP1	cna	hetloss	BASIS
PD23562	XBP1	cna	gain	BASIS
PD23574	XBP1	cna	gain	BASIS
PD24206	XBP1	cna	hetloss	BASIS
PD3905	XBP1	cna	gain	BASIS
PD4006	XBP1	cna	gain	BASIS
PD4107	XBP1	cna	gain	BASIS
PD4826	XBP1	cna	gain	BASIS
PD4967	XBP1	cna	hetloss	BASIS

PD5930	XBP1	cna	gain	BASIS
PD5935	XBP1	cna	gain	BASIS
PD5945	XBP1	cna	amp	BASIS
PD5948	XBP1	cna	gain	BASIS
PD6731	XBP1	cna	hetloss	BASIS
PD7067	XBP1	cna	gain	BASIS
PD7215	XBP1	cna	gain	BASIS
PD8621	XBP1	cna	gain	BASIS
PD9004	XBP1	cna	gain	BASIS
PD9702	XBP1	cna	gain	BASIS
TCGA-AN-A0XU	ZNF91	cna	hetloss	TCGA
TCGA-AO-A0JL	ZNF91	cna	gain	TCGA
TCGA-BH-A0AW	ZNF91	cna	gain	TCGA
TCGA-BH-A0C0	ZNF91	cna	gain	TCGA
TCGA-BH-A1FU	ZNF91	cna	hetloss	TCGA
TCGA-C8-A12L	ZNF91	cna	gain	TCGA
TCGA-D8-A27M	ZNF91	cna	hetloss	TCGA
TCGA-E9-A1NC	ZNF91	cna	hetloss	TCGA
TCGA-EW-A1OX	ZNF91	cna	hetloss	TCGA
TCGA-LL-A5YP	ZNF91	cna	amp	TCGA
MB-0346	ZNF91	cna	hetloss	METABRIC
MB-2827	ZNF91	cna	hetloss	METABRIC
MB-6060	ZNF91	cna	amp	METABRIC
MB-6098	ZNF91	cna	gain	METABRIC
MB-0420	ZNF91	cna	hetloss	METABRIC
MTS-T0064	ZNF91	cna	gain	METABRIC
PD13299	ZNF91	cna	gain	BASIS
PD13771	ZNF91	cna	gain	BASIS
PD23562	ZNF91	cna	gain	BASIS
PD23574	ZNF91	cna	gain	BASIS
PD24186	ZNF91	cna	amp	BASIS
PD24206	ZNF91	cna	hetloss	BASIS
PD3905	ZNF91	cna	gain	BASIS
PD4006	ZNF91	cna	gain	BASIS
PD4826	ZNF91	cna	gain	BASIS
PD5930	ZNF91	cna	gain	BASIS
PD5935	ZNF91	cna	gain	BASIS
PD5945	ZNF91	cna	amp	BASIS
PD5948	ZNF91	cna	gain	BASIS
PD7067	ZNF91	cna	gain	BASIS
PD7215	ZNF91	cna	amp	BASIS
PD9702	ZNF91	cna	gain	BASIS
TCGA-A2-A25B	AARS2	cna	hetloss	TCGA
TCGA-AO-A0JL	AARS2	cna	hetloss	TCGA
TCGA-BH-A0AW	AARS2	cna	gain	TCGA
TCGA-BH-A0C0	AARS2	cna	hetloss	TCGA
TCGA-C8-A12L	AARS2	cna	gain	TCGA

TCGA-D8-A27M	AARS2	cna	gain	TCGA
MB-5070	AARS2	cna	gain	METABRIC
MB-6060	AARS2	cna	hetloss	METABRIC
MB-7032	AARS2	cna	gain	METABRIC
MB-7038	AARS2	cna	hetloss	METABRIC
PD11327	AARS2	cna	amp	BASIS
PD13296	AARS2	cna	gain	BASIS
PD13299	AARS2	cna	gain	BASIS
PD22355	AARS2	cna	gain	BASIS
PD23562	AARS2	cna	gain	BASIS
PD24186	AARS2	cna	gain	BASIS
PD24206	AARS2	cna	gain	BASIS
PD3905	AARS2	cna	amp	BASIS
PD4006	AARS2	cna	gain	BASIS
PD4107	AARS2	cna	gain	BASIS
PD4826	AARS2	cna	gain	BASIS
PD5935	AARS2	cna	gain	BASIS
PD5945	AARS2	cna	amp	BASIS
PD5948	AARS2	cna	gain	BASIS
PD7067	AARS2	cna	amp	BASIS
PD7215	AARS2	cna	gain	BASIS
PD8621	AARS2	cna	gain	BASIS
PD8980	AARS2	cna	hetloss	BASIS
PD9004	AARS2	cna	gain	BASIS
PD9585	AARS2	cna	gain	BASIS
PD9702	AARS2	cna	gain	BASIS
TCGA-A2-A25B	ASXL1	cna	gain	TCGA
TCGA-AO-A0JL	ASXL1	cna	gain	TCGA
TCGA-BH-A0AW	ASXL1	cna	gain	TCGA
TCGA-BH-A0C0	ASXL1	cna	gain	TCGA
TCGA-C8-A12L	ASXL1	cna	gain	TCGA
TCGA-D8-A27M	ASXL1	cna	gain	TCGA
TCGA-E2-A1L7	ASXL1	cna	gain	TCGA
TCGA-E9-A1NC	ASXL1	cna	hetloss	TCGA
TCGA-LL-A5YP	ASXL1	cna	gain	TCGA
MB-0346	ASXL1	cna	gain	METABRIC
MB-5070	ASXL1	cna	amp	METABRIC
MB-6060	ASXL1	cna	gain	METABRIC
MB-7038	ASXL1	cna	hetloss	METABRIC
P-0010758-T01-IM5	ASXL1	cna	amp	MSK-IMPACT
PD11327	ASXL1	cna	homdel	BASIS
PD13296	ASXL1	cna	hetloss	BASIS
PD13299	ASXL1	cna	gain	BASIS
PD23574	ASXL1	cna	gain	BASIS
PD24186	ASXL1	cna	gain	BASIS
PD24206	ASXL1	cna	gain	BASIS
PD3905	ASXL1	cna	gain	BASIS

PD4107	ASXL1	cna	amp	BASIS
PD4826	ASXL1	cna	gain	BASIS
PD5930	ASXL1	cna	gain	BASIS
PD5935	ASXL1	cna	gain	BASIS
PD5945	ASXL1	cna	amp	BASIS
PD5948	ASXL1	cna	gain	BASIS
PD7067	ASXL1	cna	gain	BASIS
PD7215	ASXL1	cna	gain	BASIS
PD9004	ASXL1	cna	gain	BASIS
PD9702	ASXL1	cna	gain	BASIS
TCGA-AN-A0XU	ASXL3	cna	hetloss	TCGA
TCGA-AO-A0JL	ASXL3	cna	gain	TCGA
TCGA-BH-A0AW	ASXL3	cna	hetloss	TCGA
TCGA-BH-A0C0	ASXL3	cna	gain	TCGA
TCGA-C8-A12L	ASXL3	cna	gain	TCGA
TCGA-D8-A27M	ASXL3	cna	hetloss	TCGA
TCGA-E2-A1L7	ASXL3	cna	gain	TCGA
TCGA-LL-A5YP	ASXL3	cna	gain	TCGA
MB-0346	ASXL3	cna	hetloss	METABRIC
PD10014	ASXL3	cna	hetloss	BASIS
PD11327	ASXL3	cna	gain	BASIS
PD13297	ASXL3	cna	gain	BASIS
PD13299	ASXL3	cna	gain	BASIS
PD23578	ASXL3	cna	gain	BASIS
PD24186	ASXL3	cna	gain	BASIS
PD24202	ASXL3	cna	hetloss	BASIS
PD24206	ASXL3	cna	gain	BASIS
PD24337	ASXL3	cna	gain	BASIS
PD3905	ASXL3	cna	gain	BASIS
PD4006	ASXL3	cna	gain	BASIS
PD4107	ASXL3	cna	gain	BASIS
PD4967	ASXL3	cna	hetloss	BASIS
PD5930	ASXL3	cna	gain	BASIS
PD5945	ASXL3	cna	gain	BASIS
PD5948	ASXL3	cna	gain	BASIS
PD6406	ASXL3	cna	gain	BASIS
PD7067	ASXL3	cna	amp	BASIS
PD7215	ASXL3	cna	gain	BASIS
PD8980	ASXL3	cna	hetloss	BASIS
PD9004	ASXL3	cna	hetloss	BASIS
PD9702	ASXL3	cna	gain	BASIS
TCGA-AN-A0XU	ATIC	cna	gain	TCGA
TCGA-AO-A0JL	ATIC	cna	gain	TCGA
TCGA-BH-A0AW	ATIC	cna	hetloss	TCGA
TCGA-C8-A12L	ATIC	cna	gain	TCGA
TCGA-D8-A27M	ATIC	cna	gain	TCGA
TCGA-E2-A1L7	ATIC	cna	hetloss	TCGA

TCGA-E9-A1NC	ATIC	cna	hetloss	TCGA
MB-2827	ATIC	cna	hetloss	METABRIC
MB-6098	ATIC	cna	hetloss	METABRIC
MB-6271	ATIC	cna	hetloss	METABRIC
PD10014	ATIC	cna	gain	BASIS
PD11327	ATIC	cna	gain	BASIS
PD13296	ATIC	cna	hetloss	BASIS
PD13297	ATIC	cna	hetloss	BASIS
PD23578	ATIC	cna	hetloss	BASIS
PD24186	ATIC	cna	gain	BASIS
PD24206	ATIC	cna	gain	BASIS
PD24337	ATIC	cna	hetloss	BASIS
PD3890	ATIC	cna	hetloss	BASIS
PD3905	ATIC	cna	gain	BASIS
PD4006	ATIC	cna	gain	BASIS
PD4107	ATIC	cna	gain	BASIS
PD4967	ATIC	cna	hetloss	BASIS
PD5935	ATIC	cna	gain	BASIS
PD5945	ATIC	cna	gain	BASIS
PD5948	ATIC	cna	gain	BASIS
PD6413	ATIC	cna	hetloss	BASIS
PD6731	ATIC	cna	hetloss	BASIS
PD7067	ATIC	cna	gain	BASIS
PD7215	ATIC	cna	gain	BASIS
PD9585	ATIC	cna	hetloss	BASIS
TCGA-AN-A0XU	AXL	cna	hetloss	TCGA
TCGA-AO-A0JL	AXL	cna	hetloss	TCGA
TCGA-BH-A0AW	AXL	cna	gain	TCGA
TCGA-BH-A0C0	AXL	cna	hetloss	TCGA
TCGA-BH-A1FU	AXL	cna	hetloss	TCGA
TCGA-D8-A27M	AXL	cna	gain	TCGA
TCGA-EW-A1OX	AXL	cna	hetloss	TCGA
TCGA-LL-A5YP	AXL	cna	gain	TCGA
MB-0346	AXL	cna	hetloss	METABRIC
MB-6098	AXL	cna	hetloss	METABRIC
PD13296	AXL	cna	hetloss	BASIS
PD13299	AXL	cna	gain	BASIS
PD13771	AXL	cna	hetloss	BASIS
PD23562	AXL	cna	gain	BASIS
PD23574	AXL	cna	gain	BASIS
PD23578	AXL	cna	gain	BASIS
PD24186	AXL	cna	gain	BASIS
PD3905	AXL	cna	gain	BASIS
PD4005	AXL	cna	hetloss	BASIS
PD4006	AXL	cna	gain	BASIS
PD4826	AXL	cna	gain	BASIS
PD5935	AXL	cna	gain	BASIS

PD5945	AXL	cna	gain	BASIS
PD5948	AXL	cna	gain	BASIS
PD6406	AXL	cna	gain	BASIS
PD7067	AXL	cna	gain	BASIS
PD7215	AXL	cna	gain	BASIS
PD8621	AXL	cna	gain	BASIS
PD8980	AXL	cna	hetloss	BASIS
PD9585	AXL	cna	gain	BASIS
PD9702	AXL	cna	gain	BASIS
TCGA-AO-A0JL	BCL3	cna	hetloss	TCGA
TCGA-BH-A0AW	BCL3	cna	gain	TCGA
TCGA-BH-A0C0	BCL3	cna	hetloss	TCGA
TCGA-BH-A1FU	BCL3	cna	hetloss	TCGA
TCGA-D8-A27M	BCL3	cna	gain	TCGA
TCGA-EW-A1OX	BCL3	cna	hetloss	TCGA
TCGA-LL-A5YP	BCL3	cna	gain	TCGA
MB-0346	BCL3	cna	hetloss	METABRIC
MB-6098	BCL3	cna	hetloss	METABRIC
PD11327	BCL3	cna	gain	BASIS
PD13296	BCL3	cna	hetloss	BASIS
PD13299	BCL3	cna	gain	BASIS
PD13771	BCL3	cna	hetloss	BASIS
PD23562	BCL3	cna	gain	BASIS
PD23574	BCL3	cna	gain	BASIS
PD24186	BCL3	cna	gain	BASIS
PD24206	BCL3	cna	hetloss	BASIS
PD3905	BCL3	cna	gain	BASIS
PD4005	BCL3	cna	hetloss	BASIS
PD4006	BCL3	cna	gain	BASIS
PD4826	BCL3	cna	gain	BASIS
PD5935	BCL3	cna	gain	BASIS
PD5945	BCL3	cna	amp	BASIS
PD5948	BCL3	cna	gain	BASIS
PD6406	BCL3	cna	gain	BASIS
PD7067	BCL3	cna	gain	BASIS
PD7215	BCL3	cna	gain	BASIS
PD8621	BCL3	cna	gain	BASIS
PD8980	BCL3	cna	hetloss	BASIS
PD9585	BCL3	cna	gain	BASIS
PD9702	BCL3	cna	gain	BASIS
TCGA-A2-A25B	CCND3	cna	hetloss	TCGA
TCGA-AO-A0JL	CCND3	cna	gain	TCGA
TCGA-BH-A0AW	CCND3	cna	gain	TCGA
TCGA-BH-A0C0	CCND3	cna	hetloss	TCGA
TCGA-C8-A12L	CCND3	cna	hetloss	TCGA
TCGA-E2-A1L7	CCND3	cna	hetloss	TCGA
TCGA-E9-A1NC	CCND3	cna	amp	TCGA

MB-5070	CCND3	cna	gain	METABRIC
MB-6060	CCND3	cna	hetloss	METABRIC
MB-7032	CCND3	cna	gain	METABRIC
PD11327	CCND3	cna	amp	BASIS
PD13299	CCND3	cna	gain	BASIS
PD22355	CCND3	cna	gain	BASIS
PD23562	CCND3	cna	gain	BASIS
PD24186	CCND3	cna	gain	BASIS
PD24206	CCND3	cna	gain	BASIS
PD3905	CCND3	cna	amp	BASIS
PD4006	CCND3	cna	gain	BASIS
PD4107	CCND3	cna	gain	BASIS
PD4826	CCND3	cna	gain	BASIS
PD4967	CCND3	cna	hetloss	BASIS
PD5935	CCND3	cna	gain	BASIS
PD5945	CCND3	cna	amp	BASIS
PD5948	CCND3	cna	gain	BASIS
PD7067	CCND3	cna	amp	BASIS
PD7215	CCND3	cna	gain	BASIS
PD8621	CCND3	cna	gain	BASIS
PD8980	CCND3	cna	hetloss	BASIS
PD9004	CCND3	cna	gain	BASIS
PD9585	CCND3	cna	gain	BASIS
PD9702	CCND3	cna	amp	BASIS
TCGA-AO-A0JL	CD79A	cna	hetloss	TCGA
TCGA-BH-A0AW	CD79A	cna	gain	TCGA
TCGA-BH-A0C0	CD79A	cna	hetloss	TCGA
TCGA-BH-A1FU	CD79A	cna	hetloss	TCGA
TCGA-D8-A27M	CD79A	cna	gain	TCGA
TCGA-E9-A1NC	CD79A	cna	gain	TCGA
TCGA-EW-A1OX	CD79A	cna	hetloss	TCGA
TCGA-LL-A5YP	CD79A	cna	gain	TCGA
MB-0346	CD79A	cna	hetloss	METABRIC
MB-6098	CD79A	cna	hetloss	METABRIC
PD11327	CD79A	cna	gain	BASIS
PD13296	CD79A	cna	hetloss	BASIS
PD13299	CD79A	cna	gain	BASIS
PD13771	CD79A	cna	hetloss	BASIS
PD23562	CD79A	cna	gain	BASIS
PD23574	CD79A	cna	gain	BASIS
PD24186	CD79A	cna	gain	BASIS
PD3905	CD79A	cna	gain	BASIS
PD4005	CD79A	cna	hetloss	BASIS
PD4006	CD79A	cna	gain	BASIS
PD4826	CD79A	cna	gain	BASIS
PD5935	CD79A	cna	gain	BASIS
PD5945	CD79A	cna	gain	BASIS

PD5948	CD79A	cna	gain	BASIS
PD6406	CD79A	cna	gain	BASIS
PD7067	CD79A	cna	gain	BASIS
PD7215	CD79A	cna	gain	BASIS
PD8621	CD79A	cna	gain	BASIS
PD8980	CD79A	cna	hetloss	BASIS
PD9585	CD79A	cna	gain	BASIS
PD9702	CD79A	cna	gain	BASIS
TCGA-A2-A25B	CHEK2	cna	hetloss	TCGA
TCGA-BH-A0C0	CHEK2	cna	gain	TCGA
TCGA-BH-A1FU	CHEK2	cna	gain	TCGA
TCGA-C8-A12L	CHEK2	cna	gain	TCGA
TCGA-D8-A27M	CHEK2	cna	gain	TCGA
TCGA-E9-A1NC	CHEK2	cna	gain	TCGA
TCGA-LL-A5YP	CHEK2	cna	gain	TCGA
MB-0346	CHEK2	cna	gain	METABRIC
MB-6060	CHEK2	cna	gain	METABRIC
PD11327	CHEK2	cna	gain	BASIS
PD13296	CHEK2	cna	gain	BASIS
PD13297	CHEK2	cna	gain	BASIS
PD14442	CHEK2	cna	hetloss	BASIS
PD23562	CHEK2	cna	gain	BASIS
PD23574	CHEK2	cna	gain	BASIS
PD24206	CHEK2	cna	hetloss	BASIS
PD3905	CHEK2	cna	gain	BASIS
PD4006	CHEK2	cna	gain	BASIS
PD4107	CHEK2	cna	gain	BASIS
PD4826	CHEK2	cna	gain	BASIS
PD4967	CHEK2	cna	hetloss	BASIS
PD5930	CHEK2	cna	gain	BASIS
PD5935	CHEK2	cna	gain	BASIS
PD5945	CHEK2	cna	amp	BASIS
PD5948	CHEK2	cna	gain	BASIS
PD6731	CHEK2	cna	hetloss	BASIS
PD7067	CHEK2	cna	gain	BASIS
PD7215	CHEK2	cna	gain	BASIS
PD8621	CHEK2	cna	gain	BASIS
PD9004	CHEK2	cna	gain	BASIS
PD9702	CHEK2	cna	gain	BASIS
TCGA-AN-A0XU	CHIC2	cna	gain	TCGA
TCGA-AO-A0JL	CHIC2	cna	gain	TCGA
TCGA-BH-A0C0	CHIC2	cna	gain	TCGA
TCGA-BH-A1FU	CHIC2	cna	gain	TCGA
TCGA-C8-A12L	CHIC2	cna	gain	TCGA
TCGA-D8-A27M	CHIC2	cna	gain	TCGA
TCGA-E9-A1NC	CHIC2	cna	hetloss	TCGA
TCGA-LL-A5YP	CHIC2	cna	amp	TCGA

MB-5465	CHIC2	cna	hetloss	METABRIC
MB-6060	CHIC2	cna	gain	METABRIC
MB-6098	CHIC2	cna	amp	METABRIC
MB-7038	CHIC2	cna	gain	METABRIC
MB-0420	CHIC2	cna	hetloss	METABRIC
PD11327	CHIC2	cna	amp	BASIS
PD13296	CHIC2	cna	hetloss	BASIS
PD13297	CHIC2	cna	hetloss	BASIS
PD22355	CHIC2	cna	hetloss	BASIS
PD23574	CHIC2	cna	gain	BASIS
PD24206	CHIC2	cna	gain	BASIS
PD3890	CHIC2	cna	gain	BASIS
PD3905	CHIC2	cna	gain	BASIS
PD4006	CHIC2	cna	gain	BASIS
PD4107	CHIC2	cna	gain	BASIS
PD4826	CHIC2	cna	gain	BASIS
PD5935	CHIC2	cna	gain	BASIS
PD5945	CHIC2	cna	gain	BASIS
PD5948	CHIC2	cna	gain	BASIS
PD7215	CHIC2	cna	amp	BASIS
PD8980	CHIC2	cna	hetloss	BASIS
PD9004	CHIC2	cna	gain	BASIS
PD9702	CHIC2	cna	gain	BASIS
TCGA-AO-A0JL	CIC	cna	hetloss	TCGA
TCGA-BH-A0AW	CIC	cna	gain	TCGA
TCGA-BH-A0C0	CIC	cna	hetloss	TCGA
TCGA-BH-A1FU	CIC	cna	hetloss	TCGA
TCGA-D8-A27M	CIC	cna	gain	TCGA
TCGA-E9-A1NC	CIC	cna	gain	TCGA
TCGA-EW-A1OX	CIC	cna	hetloss	TCGA
TCGA-LL-A5YP	CIC	cna	gain	TCGA
MB-0346	CIC	cna	hetloss	METABRIC
MB-6098	CIC	cna	hetloss	METABRIC
PD11327	CIC	cna	gain	BASIS
PD13296	CIC	cna	hetloss	BASIS
PD13299	CIC	cna	gain	BASIS
PD13771	CIC	cna	hetloss	BASIS
PD23562	CIC	cna	gain	BASIS
PD23574	CIC	cna	gain	BASIS
PD24186	CIC	cna	gain	BASIS
PD3905	CIC	cna	gain	BASIS
PD4005	CIC	cna	hetloss	BASIS
PD4006	CIC	cna	gain	BASIS
PD4826	CIC	cna	gain	BASIS
PD5935	CIC	cna	gain	BASIS
PD5945	CIC	cna	gain	BASIS
PD5948	CIC	cna	gain	BASIS

PD6406	CIC	cna	gain	BASIS
PD7067	CIC	cna	gain	BASIS
PD7215	CIC	cna	gain	BASIS
PD8621	CIC	cna	gain	BASIS
PD8980	CIC	cna	hetloss	BASIS
PD9585	CIC	cna	gain	BASIS
PD9702	CIC	cna	gain	BASIS
TCGA-AN-A0XU	DAXX	cna	gain	TCGA
TCGA-AO-A0JL	DAXX	cna	gain	TCGA
TCGA-BH-A0AW	DAXX	cna	gain	TCGA
TCGA-BH-A0C0	DAXX	cna	hetloss	TCGA
TCGA-BH-A18R	DAXX	cna	gain	TCGA
TCGA-C8-A12L	DAXX	cna	hetloss	TCGA
TCGA-E2-A1L7	DAXX	cna	hetloss	TCGA
TCGA-E9-A1NC	DAXX	cna	amp	TCGA
MB-7032	DAXX	cna	gain	METABRIC
PD10014	DAXX	cna	hetloss	BASIS
PD11327	DAXX	cna	hetloss	BASIS
PD13296	DAXX	cna	gain	BASIS
PD13299	DAXX	cna	gain	BASIS
PD23562	DAXX	cna	gain	BASIS
PD23578	DAXX	cna	gain	BASIS
PD24186	DAXX	cna	gain	BASIS
PD24206	DAXX	cna	gain	BASIS
PD3905	DAXX	cna	gain	BASIS
PD4006	DAXX	cna	gain	BASIS
PD4107	DAXX	cna	gain	BASIS
PD4826	DAXX	cna	gain	BASIS
PD5935	DAXX	cna	gain	BASIS
PD5945	DAXX	cna	amp	BASIS
PD5948	DAXX	cna	gain	BASIS
PD6731	DAXX	cna	hetloss	BASIS
PD7067	DAXX	cna	amp	BASIS
PD7215	DAXX	cna	gain	BASIS
PD8621	DAXX	cna	amp	BASIS
PD9004	DAXX	cna	gain	BASIS
PD9585	DAXX	cna	gain	BASIS
PD9702	DAXX	cna	amp	BASIS
TCGA-AN-A0XU	DDB2	cna	hetloss	TCGA
TCGA-AO-A0JL	DDB2	cna	hetloss	TCGA
TCGA-BH-A0C0	DDB2	cna	hetloss	TCGA
TCGA-C8-A12L	DDB2	cna	hetloss	TCGA
TCGA-LL-A5YP	DDB2	cna	gain	TCGA
MB-0346	DDB2	cna	hetloss	METABRIC
MB-6060	DDB2	cna	gain	METABRIC
MB-6098	DDB2	cna	hetloss	METABRIC
MB-6271	DDB2	cna	gain	METABRIC

MB-7038	DDB2	cna	gain	METABRIC
PD10014	DDB2	cna	hetloss	BASIS
PD11327	DDB2	cna	homdel	BASIS
PD13296	DDB2	cna	hetloss	BASIS
PD13771	DDB2	cna	hetloss	BASIS
PD23574	DDB2	cna	gain	BASIS
PD24186	DDB2	cna	gain	BASIS
PD24206	DDB2	cna	gain	BASIS
PD3905	DDB2	cna	gain	BASIS
PD4006	DDB2	cna	gain	BASIS
PD4107	DDB2	cna	gain	BASIS
PD4826	DDB2	cna	gain	BASIS
PD5935	DDB2	cna	gain	BASIS
PD5945	DDB2	cna	gain	BASIS
PD5948	DDB2	cna	gain	BASIS
PD6406	DDB2	cna	hetloss	BASIS
PD6413	DDB2	cna	hetloss	BASIS
PD7215	DDB2	cna	gain	BASIS
PD8621	DDB2	cna	gain	BASIS
PD8980	DDB2	cna	hetloss	BASIS
PD9585	DDB2	cna	hetloss	BASIS
PD9702	DDB2	cna	gain	BASIS
TCGA-A2-A25B	EML4	cna	hetloss	TCGA
TCGA-AN-A0XU	EML4	cna	gain	TCGA
TCGA-AO-A0JL	EML4	cna	gain	TCGA
TCGA-BH-A0AW	EML4	cna	gain	TCGA
TCGA-BH-A0C0	EML4	cna	gain	TCGA
TCGA-C8-A12L	EML4	cna	gain	TCGA
TCGA-D8-A27M	EML4	cna	gain	TCGA
TCGA-LL-A5YP	EML4	cna	gain	TCGA
MB-5465	EML4	cna	gain	METABRIC
MB-6271	EML4	cna	hetloss	METABRIC
PD10014	EML4	cna	gain	BASIS
PD13299	EML4	cna	gain	BASIS
PD23562	EML4	cna	gain	BASIS
PD23578	EML4	cna	gain	BASIS
PD24186	EML4	cna	amp	BASIS
PD24206	EML4	cna	gain	BASIS
PD3890	EML4	cna	hetloss	BASIS
PD3905	EML4	cna	gain	BASIS
PD4006	EML4	cna	amp	BASIS
PD4107	EML4	cna	gain	BASIS
PD5930	EML4	cna	gain	BASIS
PD5935	EML4	cna	gain	BASIS
PD5945	EML4	cna	gain	BASIS
PD5948	EML4	cna	gain	BASIS
PD6406	EML4	cna	hetloss	BASIS

PD7067	EML4	cna	gain	BASIS
PD8621	EML4	cna	gain	BASIS
PD8980	EML4	cna	gain	BASIS
PD9004	EML4	cna	gain	BASIS
PD9585	EML4	cna	hetloss	BASIS
PD9702	EML4	cna	gain	BASIS
TCGA-AO-A0JL	ERCC2	cna	hetloss	TCGA
TCGA-BH-A0AW	ERCC2	cna	gain	TCGA
TCGA-BH-A0C0	ERCC2	cna	hetloss	TCGA
TCGA-BH-A1FU	ERCC2	cna	hetloss	TCGA
TCGA-D8-A27M	ERCC2	cna	gain	TCGA
TCGA-E2-A1L7	ERCC2	cna	hetloss	TCGA
TCGA-EW-A1OX	ERCC2	cna	hetloss	TCGA
TCGA-LL-A5YP	ERCC2	cna	gain	TCGA
MB-0346	ERCC2	cna	hetloss	METABRIC
MB-6098	ERCC2	cna	hetloss	METABRIC
PD11327	ERCC2	cna	gain	BASIS
PD13296	ERCC2	cna	hetloss	BASIS
PD13299	ERCC2	cna	gain	BASIS
PD13771	ERCC2	cna	hetloss	BASIS
PD23562	ERCC2	cna	gain	BASIS
PD23574	ERCC2	cna	gain	BASIS
PD24186	ERCC2	cna	gain	BASIS
PD24206	ERCC2	cna	hetloss	BASIS
PD3905	ERCC2	cna	gain	BASIS
PD4005	ERCC2	cna	hetloss	BASIS
PD4006	ERCC2	cna	gain	BASIS
PD4826	ERCC2	cna	gain	BASIS
PD5935	ERCC2	cna	gain	BASIS
PD5945	ERCC2	cna	amp	BASIS
PD5948	ERCC2	cna	gain	BASIS
PD6406	ERCC2	cna	gain	BASIS
PD7067	ERCC2	cna	gain	BASIS
PD7215	ERCC2	cna	gain	BASIS
PD8621	ERCC2	cna	gain	BASIS
PD8980	ERCC2	cna	hetloss	BASIS
PD9585	ERCC2	cna	gain	BASIS
TCGA-AN-A0XU	FIP1L1	cna	gain	TCGA
TCGA-AO-A0JL	FIP1L1	cna	gain	TCGA
TCGA-BH-A0C0	FIP1L1	cna	gain	TCGA
TCGA-BH-A1FU	FIP1L1	cna	gain	TCGA
TCGA-C8-A12L	FIP1L1	cna	gain	TCGA
TCGA-D8-A27M	FIP1L1	cna	gain	TCGA
TCGA-E9-A1NC	FIP1L1	cna	hetloss	TCGA
TCGA-LL-A5YP	FIP1L1	cna	amp	TCGA
MB-0346	FIP1L1	cna	gain	METABRIC
MB-5465	FIP1L1	cna	hetloss	METABRIC

MB-6060	FIP1L1	cna	gain	METABRIC
MB-6098	FIP1L1	cna	amp	METABRIC
MB-7038	FIP1L1	cna	gain	METABRIC
MB-0420	FIP1L1	cna	hetloss	METABRIC
PD11327	FIP1L1	cna	gain	BASIS
PD13296	FIP1L1	cna	hetloss	BASIS
PD13297	FIP1L1	cna	hetloss	BASIS
PD22355	FIP1L1	cna	hetloss	BASIS
PD23574	FIP1L1	cna	gain	BASIS
PD24206	FIP1L1	cna	gain	BASIS
PD3905	FIP1L1	cna	gain	BASIS
PD4006	FIP1L1	cna	gain	BASIS
PD4107	FIP1L1	cna	gain	BASIS
PD4826	FIP1L1	cna	gain	BASIS
PD5935	FIP1L1	cna	gain	BASIS
PD5945	FIP1L1	cna	gain	BASIS
PD5948	FIP1L1	cna	gain	BASIS
PD7215	FIP1L1	cna	amp	BASIS
PD8980	FIP1L1	cna	hetloss	BASIS
PD9004	FIP1L1	cna	gain	BASIS
PD9702	FIP1L1	cna	gain	BASIS
TCGA-A2-A25B	GLI3	cna	gain	TCGA
TCGA-AO-A0JL	GLI3	cna	gain	TCGA
TCGA-BH-A0AW	GLI3	cna	gain	TCGA
TCGA-BH-A0C0	GLI3	cna	hetloss	TCGA
TCGA-C8-A12L	GLI3	cna	gain	TCGA
TCGA-D8-A27M	GLI3	cna	hetloss	TCGA
TCGA-LL-A5YP	GLI3	cna	gain	TCGA
MB-0346	GLI3	cna	hetloss	METABRIC
MB-5070	GLI3	cna	hetloss	METABRIC
MB-6098	GLI3	cna	hetloss	METABRIC
MB-0420	GLI3	cna	hetloss	METABRIC
PD11327	GLI3	cna	gain	BASIS
PD13296	GLI3	cna	gain	BASIS
PD23578	GLI3	cna	hetloss	BASIS
PD24186	GLI3	cna	gain	BASIS
PD24202	GLI3	cna	hetloss	BASIS
PD24206	GLI3	cna	gain	BASIS
PD24337	GLI3	cna	hetloss	BASIS
PD3905	GLI3	cna	gain	BASIS
PD4006	GLI3	cna	gain	BASIS
PD4107	GLI3	cna	amp	BASIS
PD4826	GLI3	cna	gain	BASIS
PD5930	GLI3	cna	gain	BASIS
PD5945	GLI3	cna	amp	BASIS
PD6406	GLI3	cna	hetloss	BASIS
PD6413	GLI3	cna	gain	BASIS

PD7067	GLI3	cna	gain	BASIS
PD7215	GLI3	cna	gain	BASIS
PD8621	GLI3	cna	gain	BASIS
PD9004	GLI3	cna	hetloss	BASIS
PD9702	GLI3	cna	gain	BASIS
TCGA-AO-A0JL	HOXD11	cna	gain	TCGA
TCGA-D8-A27M	HOXD11	cna	gain	TCGA
TCGA-LL-A5YP	HOXD11	cna	gain	TCGA
MB-2827	HOXD11	cna	hetloss	METABRIC
MB-5070	HOXD11	cna	hetloss	METABRIC
MB-5107	HOXD11	cna	hetloss	METABRIC
MB-6098	HOXD11	cna	hetloss	METABRIC
MB-6271	HOXD11	cna	hetloss	METABRIC
MB-7032	HOXD11	cna	gain	METABRIC
MB-0420	HOXD11	cna	gain	METABRIC
PD10014	HOXD11	cna	gain	BASIS
PD11327	HOXD11	cna	gain	BASIS
PD13299	HOXD11	cna	gain	BASIS
PD22355	HOXD11	cna	hetloss	BASIS
PD24186	HOXD11	cna	gain	BASIS
PD24202	HOXD11	cna	gain	BASIS
PD24206	HOXD11	cna	gain	BASIS
PD3890	HOXD11	cna	hetloss	BASIS
PD3905	HOXD11	cna	gain	BASIS
PD4006	HOXD11	cna	gain	BASIS
PD4107	HOXD11	cna	gain	BASIS
PD4967	HOXD11	cna	hetloss	BASIS
PD5935	HOXD11	cna	gain	BASIS
PD5945	HOXD11	cna	amp	BASIS
PD5948	HOXD11	cna	gain	BASIS
PD6413	HOXD11	cna	hetloss	BASIS
PD7067	HOXD11	cna	gain	BASIS
PD7215	HOXD11	cna	gain	BASIS
PD9004	HOXD11	cna	gain	BASIS
PD9585	HOXD11	cna	hetloss	BASIS
PD9702	HOXD11	cna	gain	BASIS
TCGA-AO-A0JL	HOXD13	cna	gain	TCGA
TCGA-D8-A27M	HOXD13	cna	gain	TCGA
TCGA-LL-A5YP	HOXD13	cna	gain	TCGA
MB-2827	HOXD13	cna	hetloss	METABRIC
MB-5070	HOXD13	cna	hetloss	METABRIC
MB-5107	HOXD13	cna	hetloss	METABRIC
MB-6098	HOXD13	cna	hetloss	METABRIC
MB-6271	HOXD13	cna	hetloss	METABRIC
MB-7032	HOXD13	cna	gain	METABRIC
MB-0420	HOXD13	cna	gain	METABRIC
PD10014	HOXD13	cna	gain	BASIS

PD11327	HOXD13	cna	gain	BASIS
PD13299	HOXD13	cna	gain	BASIS
PD22355	HOXD13	cna	hetloss	BASIS
PD24186	HOXD13	cna	gain	BASIS
PD24202	HOXD13	cna	gain	BASIS
PD24206	HOXD13	cna	gain	BASIS
PD3890	HOXD13	cna	hetloss	BASIS
PD3905	HOXD13	cna	gain	BASIS
PD4006	HOXD13	cna	gain	BASIS
PD4107	HOXD13	cna	gain	BASIS
PD4967	HOXD13	cna	hetloss	BASIS
PD5935	HOXD13	cna	gain	BASIS
PD5945	HOXD13	cna	amp	BASIS
PD5948	HOXD13	cna	gain	BASIS
PD6413	HOXD13	cna	hetloss	BASIS
PD7067	HOXD13	cna	gain	BASIS
PD7215	HOXD13	cna	gain	BASIS
PD9004	HOXD13	cna	gain	BASIS
PD9585	HOXD13	cna	hetloss	BASIS
PD9702	HOXD13	cna	gain	BASIS
TCGA-A2-A25B	HSP90AB1	cna	hetloss	TCGA
TCGA-AO-A0JL	HSP90AB1	cna	hetloss	TCGA
TCGA-BH-A0AW	HSP90AB1	cna	gain	TCGA
TCGA-BH-A0C0	HSP90AB1	cna	hetloss	TCGA
TCGA-C8-A12L	HSP90AB1	cna	gain	TCGA
TCGA-D8-A27M	HSP90AB1	cna	gain	TCGA
MB-5070	HSP90AB1	cna	gain	METABRIC
MB-6060	HSP90AB1	cna	hetloss	METABRIC
MB-7032	HSP90AB1	cna	gain	METABRIC
MB-7038	HSP90AB1	cna	hetloss	METABRIC
PD11327	HSP90AB1	cna	amp	BASIS
PD13296	HSP90AB1	cna	gain	BASIS
PD13299	HSP90AB1	cna	gain	BASIS
PD22355	HSP90AB1	cna	gain	BASIS
PD23562	HSP90AB1	cna	gain	BASIS
PD24186	HSP90AB1	cna	gain	BASIS
PD24206	HSP90AB1	cna	gain	BASIS
PD3905	HSP90AB1	cna	amp	BASIS
PD4006	HSP90AB1	cna	gain	BASIS
PD4107	HSP90AB1	cna	gain	BASIS
PD4826	HSP90AB1	cna	gain	BASIS
PD5935	HSP90AB1	cna	gain	BASIS
PD5945	HSP90AB1	cna	amp	BASIS
PD5948	HSP90AB1	cna	gain	BASIS
PD7067	HSP90AB1	cna	amp	BASIS
PD7215	HSP90AB1	cna	gain	BASIS
PD8621	HSP90AB1	cna	gain	BASIS

PD8980	HSP90AB1	cna	hetloss	BASIS
PD9004	HSP90AB1	cna	gain	BASIS
PD9585	HSP90AB1	cna	gain	BASIS
PD9702	HSP90AB1	cna	gain	BASIS
TCGA-AN-A0XU	IDH1	cna	gain	TCGA
TCGA-AO-A0JL	IDH1	cna	gain	TCGA
TCGA-BH-A0AW	IDH1	cna	hetloss	TCGA
TCGA-C8-A12L	IDH1	cna	gain	TCGA
TCGA-D8-A27M	IDH1	cna	amp	TCGA
TCGA-E9-A1NC	IDH1	cna	hetloss	TCGA
MB-2827	IDH1	cna	hetloss	METABRIC
MB-6098	IDH1	cna	hetloss	METABRIC
MB-6271	IDH1	cna	hetloss	METABRIC
MB-7032	IDH1	cna	gain	METABRIC
MB-0420	IDH1	cna	gain	METABRIC
PD10014	IDH1	cna	gain	BASIS
PD11327	IDH1	cna	gain	BASIS
PD13297	IDH1	cna	hetloss	BASIS
PD23578	IDH1	cna	hetloss	BASIS
PD24186	IDH1	cna	gain	BASIS
PD24206	IDH1	cna	gain	BASIS
PD3890	IDH1	cna	hetloss	BASIS
PD3905	IDH1	cna	gain	BASIS
PD4006	IDH1	cna	gain	BASIS
PD4107	IDH1	cna	gain	BASIS
PD4967	IDH1	cna	hetloss	BASIS
PD5935	IDH1	cna	gain	BASIS
PD5945	IDH1	cna	gain	BASIS
PD5948	IDH1	cna	gain	BASIS
PD6413	IDH1	cna	hetloss	BASIS
PD6731	IDH1	cna	hetloss	BASIS
PD7067	IDH1	cna	gain	BASIS
PD7215	IDH1	cna	gain	BASIS
PD9004	IDH1	cna	gain	BASIS
PD9585	IDH1	cna	hetloss	BASIS
TCGA-A2-A25B	INHBA	cna	gain	TCGA
TCGA-AO-A0JL	INHBA	cna	gain	TCGA
TCGA-BH-A0AW	INHBA	cna	gain	TCGA
TCGA-BH-A0C0	INHBA	cna	hetloss	TCGA
TCGA-C8-A12L	INHBA	cna	gain	TCGA
TCGA-D8-A27M	INHBA	cna	hetloss	TCGA
TCGA-LL-A5YP	INHBA	cna	hetloss	TCGA
MB-0346	INHBA	cna	hetloss	METABRIC
MB-5070	INHBA	cna	hetloss	METABRIC
MB-6098	INHBA	cna	hetloss	METABRIC
MB-0420	INHBA	cna	hetloss	METABRIC
PD11327	INHBA	cna	gain	BASIS

PD13296	INHBA	cna	gain	BASIS
PD23578	INHBA	cna	hetloss	BASIS
PD24186	INHBA	cna	gain	BASIS
PD24202	INHBA	cna	hetloss	BASIS
PD24206	INHBA	cna	gain	BASIS
PD24337	INHBA	cna	hetloss	BASIS
PD3905	INHBA	cna	gain	BASIS
PD4006	INHBA	cna	gain	BASIS
PD4107	INHBA	cna	amp	BASIS
PD4826	INHBA	cna	gain	BASIS
PD5930	INHBA	cna	gain	BASIS
PD5945	INHBA	cna	amp	BASIS
PD6406	INHBA	cna	hetloss	BASIS
PD6413	INHBA	cna	gain	BASIS
PD7067	INHBA	cna	gain	BASIS
PD7215	INHBA	cna	gain	BASIS
PD8621	INHBA	cna	gain	BASIS
PD9004	INHBA	cna	hetloss	BASIS
PD9702	INHBA	cna	gain	BASIS
TCGA-AN-A0XU	IRS1	cna	hetloss	TCGA
TCGA-AO-A0JL	IRS1	cna	gain	TCGA
TCGA-BH-A0AW	IRS1	cna	hetloss	TCGA
TCGA-C8-A12L	IRS1	cna	hetloss	TCGA
TCGA-D8-A27M	IRS1	cna	gain	TCGA
TCGA-E2-A1L7	IRS1	cna	hetloss	TCGA
TCGA-E9-A1NC	IRS1	cna	hetloss	TCGA
MB-2827	IRS1	cna	hetloss	METABRIC
MB-6060	IRS1	cna	hetloss	METABRIC
MB-6098	IRS1	cna	hetloss	METABRIC
MB-6271	IRS1	cna	hetloss	METABRIC
MB-7038	IRS1	cna	gain	METABRIC
PD10014	IRS1	cna	gain	BASIS
PD11327	IRS1	cna	gain	BASIS
PD13296	IRS1	cna	hetloss	BASIS
PD13297	IRS1	cna	hetloss	BASIS
PD23578	IRS1	cna	hetloss	BASIS
PD24186	IRS1	cna	gain	BASIS
PD3890	IRS1	cna	hetloss	BASIS
PD4005	IRS1	cna	hetloss	BASIS
PD4967	IRS1	cna	hetloss	BASIS
PD5935	IRS1	cna	gain	BASIS
PD5945	IRS1	cna	gain	BASIS
PD5948	IRS1	cna	gain	BASIS
PD6406	IRS1	cna	hetloss	BASIS
PD6413	IRS1	cna	hetloss	BASIS
PD6731	IRS1	cna	hetloss	BASIS
PD7067	IRS1	cna	gain	BASIS

PD7215	IRS1	cna	gain	BASIS
PD8980	IRS1	cna	hetloss	BASIS
PD9585	IRS1	cna	hetloss	BASIS
TCGA-AO-A0JL	KIT	cna	gain	TCGA
TCGA-BH-A0C0	KIT	cna	gain	TCGA
TCGA-BH-A1FU	KIT	cna	gain	TCGA
TCGA-C8-A12L	KIT	cna	gain	TCGA
TCGA-D8-A27M	KIT	cna	gain	TCGA
TCGA-E9-A1NC	KIT	cna	hetloss	TCGA
TCGA-LL-A5YP	KIT	cna	gain	TCGA
MB-5465	KIT	cna	gain	METABRIC
MB-6060	KIT	cna	gain	METABRIC
MB-6098	KIT	cna	amp	METABRIC
MB-7038	KIT	cna	gain	METABRIC
MB-0420	KIT	cna	hetloss	METABRIC
PD11327	KIT	cna	amp	BASIS
PD11742	KIT	cna	hetloss	BASIS
PD13296	KIT	cna	hetloss	BASIS
PD13297	KIT	cna	hetloss	BASIS
PD22355	KIT	cna	hetloss	BASIS
PD23574	KIT	cna	gain	BASIS
PD24206	KIT	cna	gain	BASIS
PD3890	KIT	cna	gain	BASIS
PD3905	KIT	cna	gain	BASIS
PD4006	KIT	cna	gain	BASIS
PD4107	KIT	cna	gain	BASIS
PD4826	KIT	cna	gain	BASIS
PD5935	KIT	cna	gain	BASIS
PD5945	KIT	cna	gain	BASIS
PD5948	KIT	cna	gain	BASIS
PD7215	KIT	cna	amp	BASIS
PD8980	KIT	cna	hetloss	BASIS
PD9004	KIT	cna	gain	BASIS
PD9702	KIT	cna	gain	BASIS
TCGA-A2-A25B	KLF4	cna	gain	TCGA
TCGA-AN-A0XU	KLF4	cna	hetloss	TCGA
TCGA-AO-A0JL	KLF4	cna	hetloss	TCGA
TCGA-BH-A1FU	KLF4	cna	hetloss	TCGA
TCGA-C8-A12L	KLF4	cna	hetloss	TCGA
TCGA-E2-A1L7	KLF4	cna	hetloss	TCGA
TCGA-E9-A1NC	KLF4	cna	hetloss	TCGA
TCGA-LL-A5YP	KLF4	cna	hetloss	TCGA
MB-0346	KLF4	cna	hetloss	METABRIC
MB-5070	KLF4	cna	hetloss	METABRIC
MB-5107	KLF4	cna	hetloss	METABRIC
MB-6098	KLF4	cna	hetloss	METABRIC
MB-7038	KLF4	cna	hetloss	METABRIC

MB-0420	KLF4	cna	hetloss	METABRIC
PD11327	KLF4	cna	hetloss	BASIS
PD13296	KLF4	cna	hetloss	BASIS
PD23562	KLF4	cna	gain	BASIS
PD23578	KLF4	cna	hetloss	BASIS
PD24186	KLF4	cna	amp	BASIS
PD24202	KLF4	cna	hetloss	BASIS
PD24206	KLF4	cna	gain	BASIS
PD3905	KLF4	cna	gain	BASIS
PD4005	KLF4	cna	hetloss	BASIS
PD4006	KLF4	cna	gain	BASIS
PD4107	KLF4	cna	hetloss	BASIS
PD5945	KLF4	cna	amp	BASIS
PD6406	KLF4	cna	hetloss	BASIS
PD6731	KLF4	cna	hetloss	BASIS
PD7215	KLF4	cna	gain	BASIS
PD8621	KLF4	cna	gain	BASIS
PD9585	KLF4	cna	hetloss	BASIS
TCGA-AN-A0XU	MAPK8IP1	cna	hetloss	TCGA
TCGA-AO-A0JL	MAPK8IP1	cna	hetloss	TCGA
TCGA-BH-A0C0	MAPK8IP1	cna	gain	TCGA
TCGA-C8-A12L	MAPK8IP1	cna	hetloss	TCGA
TCGA-LL-A5YP	MAPK8IP1	cna	gain	TCGA
MB-0346	MAPK8IP1	cna	hetloss	METABRIC
MB-6098	MAPK8IP1	cna	hetloss	METABRIC
MB-6271	MAPK8IP1	cna	gain	METABRIC
MB-7048	MAPK8IP1	cna	hetloss	METABRIC
PD10014	MAPK8IP1	cna	hetloss	BASIS
PD11327	MAPK8IP1	cna	homdel	BASIS
PD13296	MAPK8IP1	cna	hetloss	BASIS
PD13771	MAPK8IP1	cna	hetloss	BASIS
PD23574	MAPK8IP1	cna	gain	BASIS
PD24186	MAPK8IP1	cna	gain	BASIS
PD24206	MAPK8IP1	cna	gain	BASIS
PD3905	MAPK8IP1	cna	gain	BASIS
PD4005	MAPK8IP1	cna	gain	BASIS
PD4006	MAPK8IP1	cna	gain	BASIS
PD4107	MAPK8IP1	cna	gain	BASIS
PD4826	MAPK8IP1	cna	gain	BASIS
PD5935	MAPK8IP1	cna	gain	BASIS
PD5945	MAPK8IP1	cna	gain	BASIS
PD5948	MAPK8IP1	cna	gain	BASIS
PD6406	MAPK8IP1	cna	hetloss	BASIS
PD6413	MAPK8IP1	cna	hetloss	BASIS
PD7215	MAPK8IP1	cna	gain	BASIS
PD8621	MAPK8IP1	cna	gain	BASIS
PD8980	MAPK8IP1	cna	hetloss	BASIS

PD9585	MAPK8IP1	cna	hetloss	BASIS
PD9702	MAPK8IP1	cna	gain	BASIS
TCGA-AN-A0XU	MDC1	cna	gain	TCGA
TCGA-AO-A0JL	MDC1	cna	gain	TCGA
TCGA-BH-A0AW	MDC1	cna	gain	TCGA
TCGA-BH-A0C0	MDC1	cna	hetloss	TCGA
TCGA-BH-A18R	MDC1	cna	gain	TCGA
TCGA-C8-A12L	MDC1	cna	hetloss	TCGA
TCGA-E9-A1NC	MDC1	cna	amp	TCGA
MB-5465	MDC1	cna	gain	METABRIC
MB-6098	MDC1	cna	hetloss	METABRIC
MB-7032	MDC1	cna	gain	METABRIC
PD11327	MDC1	cna	hetloss	BASIS
PD13296	MDC1	cna	gain	BASIS
PD13299	MDC1	cna	gain	BASIS
PD23562	MDC1	cna	gain	BASIS
PD24186	MDC1	cna	gain	BASIS
PD24202	MDC1	cna	gain	BASIS
PD24206	MDC1	cna	gain	BASIS
PD3905	MDC1	cna	gain	BASIS
PD4006	MDC1	cna	gain	BASIS
PD4107	MDC1	cna	gain	BASIS
PD4826	MDC1	cna	gain	BASIS
PD5935	MDC1	cna	gain	BASIS
PD5945	MDC1	cna	amp	BASIS
PD5948	MDC1	cna	gain	BASIS
PD6731	MDC1	cna	hetloss	BASIS
PD7067	MDC1	cna	amp	BASIS
PD7215	MDC1	cna	gain	BASIS
PD8621	MDC1	cna	amp	BASIS
PD9004	MDC1	cna	gain	BASIS
PD9585	MDC1	cna	gain	BASIS
PD9702	MDC1	cna	gain	BASIS
TCGA-A2-A25B	MLLT3	cna	hetloss	TCGA
TCGA-AO-A0JL	MLLT3	cna	gain	TCGA
TCGA-BH-A0AW	MLLT3	cna	hetloss	TCGA
TCGA-BH-A1FU	MLLT3	cna	hetloss	TCGA
TCGA-C8-A12L	MLLT3	cna	hetloss	TCGA
TCGA-E9-A1NC	MLLT3	cna	hetloss	TCGA
MB-0346	MLLT3	cna	hetloss	METABRIC
MB-5070	MLLT3	cna	hetloss	METABRIC
MB-6098	MLLT3	cna	hetloss	METABRIC
MB-0420	MLLT3	cna	hetloss	METABRIC
MTS-T0064	MLLT3	cna	amp	METABRIC
PD13296	MLLT3	cna	hetloss	BASIS
PD13297	MLLT3	cna	hetloss	BASIS
PD13299	MLLT3	cna	amp	BASIS

PD22355	MLLT3	cna	gain	BASIS
PD23574	MLLT3	cna	amp	BASIS
PD24202	MLLT3	cna	hetloss	BASIS
PD24337	MLLT3	cna	hetloss	BASIS
PD3905	MLLT3	cna	gain	BASIS
PD4005	MLLT3	cna	hetloss	BASIS
PD4006	MLLT3	cna	gain	BASIS
PD4826	MLLT3	cna	hetloss	BASIS
PD5945	MLLT3	cna	amp	BASIS
PD6406	MLLT3	cna	homdel	BASIS
PD6413	MLLT3	cna	gain	BASIS
PD7067	MLLT3	cna	homdel	BASIS
PD8621	MLLT3	cna	gain	BASIS
PD8980	MLLT3	cna	homdel	BASIS
PD9004	MLLT3	cna	gain	BASIS
PD9585	MLLT3	cna	hetloss	BASIS
PD9702	MLLT3	cna	gain	BASIS
TCGA-A2-A25B	MN1	cna	hetloss	TCGA
TCGA-BH-A0C0	MN1	cna	gain	TCGA
TCGA-BH-A1FU	MN1	cna	gain	TCGA
TCGA-C8-A12L	MN1	cna	gain	TCGA
TCGA-E9-A1NC	MN1	cna	gain	TCGA
TCGA-LL-A5YP	MN1	cna	gain	TCGA
MB-0346	MN1	cna	gain	METABRIC
MB-6060	MN1	cna	gain	METABRIC
PD11327	MN1	cna	gain	BASIS
PD11742	MN1	cna	hetloss	BASIS
PD13296	MN1	cna	gain	BASIS
PD13297	MN1	cna	gain	BASIS
PD14442	MN1	cna	hetloss	BASIS
PD23562	MN1	cna	gain	BASIS
PD23574	MN1	cna	gain	BASIS
PD24206	MN1	cna	hetloss	BASIS
PD3905	MN1	cna	gain	BASIS
PD4006	MN1	cna	gain	BASIS
PD4107	MN1	cna	gain	BASIS
PD4826	MN1	cna	gain	BASIS
PD4967	MN1	cna	hetloss	BASIS
PD5930	MN1	cna	gain	BASIS
PD5935	MN1	cna	gain	BASIS
PD5945	MN1	cna	amp	BASIS
PD5948	MN1	cna	gain	BASIS
PD6731	MN1	cna	hetloss	BASIS
PD7067	MN1	cna	gain	BASIS
PD7215	MN1	cna	gain	BASIS
PD8621	MN1	cna	gain	BASIS
PD9004	MN1	cna	gain	BASIS

PD9702	MN1	cna	gain	BASIS
TCGA-A2-A25B	MUTYH	cna	gain	TCGA
TCGA-AN-A0XU	MUTYH	cna	gain	TCGA
TCGA-AO-A0JL	MUTYH	cna	hetloss	TCGA
TCGA-BH-A0AW	MUTYH	cna	gain	TCGA
TCGA-BH-A0C0	MUTYH	cna	gain	TCGA
TCGA-BH-A1FU	MUTYH	cna	gain	TCGA
TCGA-D8-A27M	MUTYH	cna	gain	TCGA
TCGA-E2-A1L7	MUTYH	cna	gain	TCGA
TCGA-LL-A5YP	MUTYH	cna	hetloss	TCGA
MB-0346	MUTYH	cna	hetloss	METABRIC
MB-2827	MUTYH	cna	hetloss	METABRIC
MB-6060	MUTYH	cna	hetloss	METABRIC
MB-6098	MUTYH	cna	gain	METABRIC
MB-7038	MUTYH	cna	gain	METABRIC
MB-0420	MUTYH	cna	hetloss	METABRIC
PD13296	MUTYH	cna	gain	BASIS
PD13299	MUTYH	cna	gain	BASIS
PD24186	MUTYH	cna	amp	BASIS
PD3905	MUTYH	cna	gain	BASIS
PD4006	MUTYH	cna	gain	BASIS
PD4107	MUTYH	cna	gain	BASIS
PD4826	MUTYH	cna	gain	BASIS
PD5935	MUTYH	cna	gain	BASIS
PD5945	MUTYH	cna	amp	BASIS
PD5948	MUTYH	cna	gain	BASIS
PD7067	MUTYH	cna	gain	BASIS
PD7215	MUTYH	cna	gain	BASIS
PD8621	MUTYH	cna	gain	BASIS
PD8980	MUTYH	cna	gain	BASIS
PD9004	MUTYH	cna	gain	BASIS
PD9702	MUTYH	cna	gain	BASIS
TCGA-A2-A25B	NACA	cna	gain	TCGA
TCGA-AO-A0JL	NACA	cna	hetloss	TCGA
TCGA-BH-A18R	NACA	cna	gain	TCGA
TCGA-BH-A1FU	NACA	cna	hetloss	TCGA
TCGA-C8-A12L	NACA	cna	gain	TCGA
TCGA-D8-A27M	NACA	cna	hetloss	TCGA
TCGA-E2-A1L7	NACA	cna	hetloss	TCGA
TCGA-LL-A5YP	NACA	cna	hetloss	TCGA
MB-0346	NACA	cna	gain	METABRIC
MB-2827	NACA	cna	hetloss	METABRIC
MB-6098	NACA	cna	hetloss	METABRIC
MB-7038	NACA	cna	hetloss	METABRIC
MB-0420	NACA	cna	hetloss	METABRIC
PD11327	NACA	cna	homdel	BASIS
PD13296	NACA	cna	hetloss	BASIS

PD13297	NACA	cna	hetloss	BASIS
PD13771	NACA	cna	gain	BASIS
PD24186	NACA	cna	gain	BASIS
PD24202	NACA	cna	hetloss	BASIS
PD24206	NACA	cna	gain	BASIS
PD24337	NACA	cna	hetloss	BASIS
PD3890	NACA	cna	hetloss	BASIS
PD3905	NACA	cna	gain	BASIS
PD4826	NACA	cna	gain	BASIS
PD5945	NACA	cna	gain	BASIS
PD6413	NACA	cna	hetloss	BASIS
PD6731	NACA	cna	hetloss	BASIS
PD7067	NACA	cna	gain	BASIS
PD8621	NACA	cna	hetloss	BASIS
PD9585	NACA	cna	hetloss	BASIS
PD9702	NACA	cna	gain	BASIS
TCGA-AN-A0XU	PALB2	cna	gain	TCGA
TCGA-AO-A0JL	PALB2	cna	gain	TCGA
TCGA-BH-A0AW	PALB2	cna	gain	TCGA
TCGA-BH-A0C0	PALB2	cna	gain	TCGA
TCGA-C8-A12L	PALB2	cna	gain	TCGA
TCGA-E2-A1L7	PALB2	cna	hetloss	TCGA
TCGA-LL-A5YP	PALB2	cna	gain	TCGA
MB-0346	PALB2	cna	gain	METABRIC
MB-6271	PALB2	cna	gain	METABRIC
MB-7032	PALB2	cna	gain	METABRIC
MB-7038	PALB2	cna	hetloss	METABRIC
MB-0420	PALB2	cna	hetloss	METABRIC
PD10014	PALB2	cna	hetloss	BASIS
PD11327	PALB2	cna	hetloss	BASIS
PD11742	PALB2	cna	gain	BASIS
PD13299	PALB2	cna	gain	BASIS
PD24186	PALB2	cna	gain	BASIS
PD24202	PALB2	cna	hetloss	BASIS
PD3905	PALB2	cna	gain	BASIS
PD4006	PALB2	cna	gain	BASIS
PD4107	PALB2	cna	gain	BASIS
PD4826	PALB2	cna	gain	BASIS
PD5930	PALB2	cna	gain	BASIS
PD5935	PALB2	cna	gain	BASIS
PD5945	PALB2	cna	amp	BASIS
PD5948	PALB2	cna	gain	BASIS
PD7067	PALB2	cna	gain	BASIS
PD7215	PALB2	cna	amp	BASIS
PD8621	PALB2	cna	gain	BASIS
PD8980	PALB2	cna	gain	BASIS
PD9004	PALB2	cna	gain	BASIS

TCGA-AN-A0XU	PAX3	cna	hetloss	TCGA
TCGA-AO-A0JL	PAX3	cna	gain	TCGA
TCGA-BH-A0AW	PAX3	cna	hetloss	TCGA
TCGA-C8-A12L	PAX3	cna	hetloss	TCGA
TCGA-D8-A27M	PAX3	cna	gain	TCGA
TCGA-E2-A1L7	PAX3	cna	hetloss	TCGA
TCGA-E9-A1NC	PAX3	cna	hetloss	TCGA
MB-2827	PAX3	cna	hetloss	METABRIC
MB-6060	PAX3	cna	hetloss	METABRIC
MB-6098	PAX3	cna	hetloss	METABRIC
MB-6271	PAX3	cna	hetloss	METABRIC
MB-7038	PAX3	cna	gain	METABRIC
PD10014	PAX3	cna	gain	BASIS
PD11327	PAX3	cna	gain	BASIS
PD13296	PAX3	cna	hetloss	BASIS
PD13297	PAX3	cna	hetloss	BASIS
PD23578	PAX3	cna	hetloss	BASIS
PD24186	PAX3	cna	gain	BASIS
PD24337	PAX3	cna	hetloss	BASIS
PD3890	PAX3	cna	hetloss	BASIS
PD3905	PAX3	cna	gain	BASIS
PD4967	PAX3	cna	hetloss	BASIS
PD5935	PAX3	cna	gain	BASIS
PD5945	PAX3	cna	gain	BASIS
PD5948	PAX3	cna	gain	BASIS
PD6413	PAX3	cna	hetloss	BASIS
PD6731	PAX3	cna	hetloss	BASIS
PD7067	PAX3	cna	gain	BASIS
PD7215	PAX3	cna	gain	BASIS
PD8980	PAX3	cna	hetloss	BASIS
PD9585	PAX3	cna	hetloss	BASIS
TCGA-AN-A0XU	PDGFRA	cna	gain	TCGA
TCGA-AO-A0JL	PDGFRA	cna	gain	TCGA
TCGA-BH-A0C0	PDGFRA	cna	gain	TCGA
TCGA-BH-A1FU	PDGFRA	cna	gain	TCGA
TCGA-C8-A12L	PDGFRA	cna	gain	TCGA
TCGA-D8-A27M	PDGFRA	cna	gain	TCGA
TCGA-E9-A1NC	PDGFRA	cna	hetloss	TCGA
TCGA-LL-A5YP	PDGFRA	cna	amp	TCGA
MB-5465	PDGFRA	cna	hetloss	METABRIC
MB-6060	PDGFRA	cna	gain	METABRIC
MB-6098	PDGFRA	cna	amp	METABRIC
MB-7038	PDGFRA	cna	gain	METABRIC
MB-0420	PDGFRA	cna	hetloss	METABRIC
PD11327	PDGFRA	cna	amp	BASIS
PD13296	PDGFRA	cna	hetloss	BASIS
PD13297	PDGFRA	cna	hetloss	BASIS

PD22355	PDGFRA	cna	hetloss	BASIS
PD23574	PDGFRA	cna	gain	BASIS
PD24206	PDGFRA	cna	gain	BASIS
PD3890	PDGFRA	cna	gain	BASIS
PD3905	PDGFRA	cna	gain	BASIS
PD4006	PDGFRA	cna	gain	BASIS
PD4107	PDGFRA	cna	gain	BASIS
PD4826	PDGFRA	cna	gain	BASIS
PD5935	PDGFRA	cna	gain	BASIS
PD5945	PDGFRA	cna	gain	BASIS
PD5948	PDGFRA	cna	gain	BASIS
PD7215	PDGFRA	cna	amp	BASIS
PD8980	PDGFRA	cna	hetloss	BASIS
PD9004	PDGFRA	cna	gain	BASIS
PD9702	PDGFRA	cna	gain	BASIS
TCGA-A2-A25B	PIK3R3	cna	gain	TCGA
TCGA-AN-A0XU	PIK3R3	cna	gain	TCGA
TCGA-AO-A0JL	PIK3R3	cna	hetloss	TCGA
TCGA-BH-A0AW	PIK3R3	cna	gain	TCGA
TCGA-BH-A0C0	PIK3R3	cna	gain	TCGA
TCGA-BH-A1FU	PIK3R3	cna	gain	TCGA
TCGA-D8-A27M	PIK3R3	cna	gain	TCGA
TCGA-E2-A1L7	PIK3R3	cna	gain	TCGA
TCGA-LL-A5YP	PIK3R3	cna	hetloss	TCGA
MB-0346	PIK3R3	cna	hetloss	METABRIC
MB-2827	PIK3R3	cna	hetloss	METABRIC
MB-6060	PIK3R3	cna	hetloss	METABRIC
MB-6098	PIK3R3	cna	gain	METABRIC
MB-7038	PIK3R3	cna	gain	METABRIC
MB-0420	PIK3R3	cna	hetloss	METABRIC
PD13296	PIK3R3	cna	gain	BASIS
PD13299	PIK3R3	cna	gain	BASIS
PD24186	PIK3R3	cna	amp	BASIS
PD24206	PIK3R3	cna	gain	BASIS
PD3905	PIK3R3	cna	gain	BASIS
PD4006	PIK3R3	cna	gain	BASIS
PD4107	PIK3R3	cna	gain	BASIS
PD4826	PIK3R3	cna	gain	BASIS
PD5935	PIK3R3	cna	gain	BASIS
PD5945	PIK3R3	cna	amp	BASIS
PD5948	PIK3R3	cna	gain	BASIS
PD7067	PIK3R3	cna	gain	BASIS
PD7215	PIK3R3	cna	gain	BASIS
PD8621	PIK3R3	cna	gain	BASIS
PD9004	PIK3R3	cna	gain	BASIS
PD9702	PIK3R3	cna	gain	BASIS
TCGA-A2-A25B	PIM1	cna	hetloss	TCGA

TCGA-AN-A0XU	PIM1	cna	hetloss	TCGA
TCGA-AO-A0JL	PIM1	cna	gain	TCGA
TCGA-BH-A0AW	PIM1	cna	gain	TCGA
TCGA-BH-A0C0	PIM1	cna	hetloss	TCGA
TCGA-C8-A12L	PIM1	cna	hetloss	TCGA
TCGA-E2-A1L7	PIM1	cna	hetloss	TCGA
TCGA-E9-A1NC	PIM1	cna	amp	TCGA
MB-6098	PIM1	cna	gain	METABRIC
MB-7032	PIM1	cna	gain	METABRIC
PD11327	PIM1	cna	hetloss	BASIS
PD13299	PIM1	cna	gain	BASIS
PD22355	PIM1	cna	gain	BASIS
PD23562	PIM1	cna	gain	BASIS
PD24186	PIM1	cna	gain	BASIS
PD24206	PIM1	cna	gain	BASIS
PD24337	PIM1	cna	gain	BASIS
PD3905	PIM1	cna	gain	BASIS
PD4006	PIM1	cna	gain	BASIS
PD4107	PIM1	cna	gain	BASIS
PD4826	PIM1	cna	gain	BASIS
PD5935	PIM1	cna	gain	BASIS
PD5945	PIM1	cna	amp	BASIS
PD5948	PIM1	cna	gain	BASIS
PD7067	PIM1	cna	amp	BASIS
PD7215	PIM1	cna	gain	BASIS
PD8621	PIM1	cna	gain	BASIS
PD8980	PIM1	cna	homdel	BASIS
PD9004	PIM1	cna	gain	BASIS
PD9585	PIM1	cna	gain	BASIS
PD9702	PIM1	cna	gain	BASIS
TCGA-A2-A25B	PNRC1	cna	hetloss	TCGA
TCGA-AN-A0XU	PNRC1	cna	amp	TCGA
TCGA-AO-A0JL	PNRC1	cna	hetloss	TCGA
TCGA-C8-A12L	PNRC1	cna	gain	TCGA
MB-2827	PNRC1	cna	amp	METABRIC
MB-5107	PNRC1	cna	hetloss	METABRIC
MB-6098	PNRC1	cna	hetloss	METABRIC
MB-7032	PNRC1	cna	hetloss	METABRIC
MB-7038	PNRC1	cna	gain	METABRIC
MB-7048	PNRC1	cna	hetloss	METABRIC
PD11327	PNRC1	cna	hetloss	BASIS
PD13299	PNRC1	cna	gain	BASIS
PD13771	PNRC1	cna	hetloss	BASIS
PD23562	PNRC1	cna	gain	BASIS
PD23574	PNRC1	cna	gain	BASIS
PD23578	PNRC1	cna	gain	BASIS
PD24186	PNRC1	cna	gain	BASIS

PD24206	PNRC1	cna	gain	BASIS
PD24337	PNRC1	cna	hetloss	BASIS
PD3905	PNRC1	cna	gain	BASIS
PD4006	PNRC1	cna	gain	BASIS
PD4826	PNRC1	cna	gain	BASIS
PD5935	PNRC1	cna	gain	BASIS
PD5945	PNRC1	cna	gain	BASIS
PD6731	PNRC1	cna	hetloss	BASIS
PD7215	PNRC1	cna	gain	BASIS
PD8621	PNRC1	cna	gain	BASIS
PD8980	PNRC1	cna	gain	BASIS
PD9004	PNRC1	cna	gain	BASIS
PD9585	PNRC1	cna	hetloss	BASIS
PD9702	PNRC1	cna	gain	BASIS
TCGA-AN-A0XU	POU5F1	cna	gain	TCGA
TCGA-AO-A0JL	POU5F1	cna	gain	TCGA
TCGA-BH-A0AW	POU5F1	cna	gain	TCGA
TCGA-BH-A0C0	POU5F1	cna	hetloss	TCGA
TCGA-BH-A18R	POU5F1	cna	gain	TCGA
TCGA-C8-A12L	POU5F1	cna	hetloss	TCGA
TCGA-E2-A1L7	POU5F1	cna	hetloss	TCGA
TCGA-E9-A1NC	POU5F1	cna	amp	TCGA
MB-6098	POU5F1	cna	hetloss	METABRIC
MB-7032	POU5F1	cna	gain	METABRIC
PD11327	POU5F1	cna	hetloss	BASIS
PD13296	POU5F1	cna	gain	BASIS
PD13299	POU5F1	cna	gain	BASIS
PD23562	POU5F1	cna	gain	BASIS
PD24186	POU5F1	cna	gain	BASIS
PD24202	POU5F1	cna	gain	BASIS
PD24206	POU5F1	cna	gain	BASIS
PD3905	POU5F1	cna	gain	BASIS
PD4006	POU5F1	cna	gain	BASIS
PD4107	POU5F1	cna	gain	BASIS
PD4826	POU5F1	cna	gain	BASIS
PD5935	POU5F1	cna	gain	BASIS
PD5945	POU5F1	cna	amp	BASIS
PD5948	POU5F1	cna	gain	BASIS
PD6731	POU5F1	cna	hetloss	BASIS
PD7067	POU5F1	cna	amp	BASIS
PD7215	POU5F1	cna	gain	BASIS
PD8621	POU5F1	cna	amp	BASIS
PD9004	POU5F1	cna	gain	BASIS
PD9585	POU5F1	cna	gain	BASIS
PD9702	POU5F1	cna	amp	BASIS
TCGA-A2-A25B	RAD54L	cna	gain	TCGA
TCGA-AN-A0XU	RAD54L	cna	gain	TCGA

TCGA-AO-A0JL	RAD54L	cna	hetloss	TCGA
TCGA-BH-A0AW	RAD54L	cna	gain	TCGA
TCGA-BH-A0C0	RAD54L	cna	gain	TCGA
TCGA-BH-A1FU	RAD54L	cna	gain	TCGA
TCGA-D8-A27M	RAD54L	cna	gain	TCGA
TCGA-E2-A1L7	RAD54L	cna	gain	TCGA
TCGA-LL-A5YP	RAD54L	cna	hetloss	TCGA
MB-0346	RAD54L	cna	hetloss	METABRIC
MB-2827	RAD54L	cna	hetloss	METABRIC
MB-6060	RAD54L	cna	hetloss	METABRIC
MB-6098	RAD54L	cna	gain	METABRIC
MB-7038	RAD54L	cna	gain	METABRIC
MB-0420	RAD54L	cna	hetloss	METABRIC
PD13296	RAD54L	cna	gain	BASIS
PD13299	RAD54L	cna	gain	BASIS
PD24186	RAD54L	cna	amp	BASIS
PD24206	RAD54L	cna	gain	BASIS
PD3905	RAD54L	cna	gain	BASIS
PD4006	RAD54L	cna	gain	BASIS
PD4107	RAD54L	cna	gain	BASIS
PD4826	RAD54L	cna	gain	BASIS
PD5935	RAD54L	cna	gain	BASIS
PD5945	RAD54L	cna	amp	BASIS
PD5948	RAD54L	cna	gain	BASIS
PD7067	RAD54L	cna	gain	BASIS
PD7215	RAD54L	cna	gain	BASIS
PD8621	RAD54L	cna	gain	BASIS
PD9004	RAD54L	cna	gain	BASIS
PD9702	RAD54L	cna	gain	BASIS
TCGA-A2-A25B	RASGEF1A	cna	hetloss	TCGA
TCGA-AN-A0XU	RASGEF1A	cna	hetloss	TCGA
TCGA-AO-A0JL	RASGEF1A	cna	hetloss	TCGA
TCGA-BH-A0C0	RASGEF1A	cna	gain	TCGA
TCGA-C8-A12L	RASGEF1A	cna	gain	TCGA
TCGA-E9-A1NC	RASGEF1A	cna	hetloss	TCGA
TCGA-LL-A5YP	RASGEF1A	cna	gain	TCGA
MB-5070	RASGEF1A	cna	gain	METABRIC
MB-6098	RASGEF1A	cna	hetloss	METABRIC
MB-7038	RASGEF1A	cna	hetloss	METABRIC
PD11327	RASGEF1A	cna	gain	BASIS
PD13296	RASGEF1A	cna	gain	BASIS
PD13299	RASGEF1A	cna	gain	BASIS
PD23562	RASGEF1A	cna	gain	BASIS
PD23574	RASGEF1A	cna	amp	BASIS
PD24186	RASGEF1A	cna	gain	BASIS
PD24206	RASGEF1A	cna	gain	BASIS
PD3905	RASGEF1A	cna	gain	BASIS

PD4006	RASGEF1A	cna	gain	BASIS
PD4107	RASGEF1A	cna	gain	BASIS
PD4826	RASGEF1A	cna	gain	BASIS
PD5930	RASGEF1A	cna	gain	BASIS
PD5945	RASGEF1A	cna	gain	BASIS
PD5948	RASGEF1A	cna	gain	BASIS
PD7067	RASGEF1A	cna	gain	BASIS
PD7215	RASGEF1A	cna	gain	BASIS
PD8621	RASGEF1A	cna	gain	BASIS
PD8980	RASGEF1A	cna	hetloss	BASIS
PD9004	RASGEF1A	cna	gain	BASIS
PD9585	RASGEF1A	cna	hetloss	BASIS
PD9702	RASGEF1A	cna	gain	BASIS
TCGA-A2-A25B	RET	cna	hetloss	TCGA
TCGA-AN-A0XU	RET	cna	hetloss	TCGA
TCGA-AO-A0JL	RET	cna	hetloss	TCGA
TCGA-BH-A0C0	RET	cna	gain	TCGA
TCGA-C8-A12L	RET	cna	gain	TCGA
TCGA-E9-A1NC	RET	cna	gain	TCGA
TCGA-LL-A5YP	RET	cna	gain	TCGA
MB-5070	RET	cna	gain	METABRIC
MB-6098	RET	cna	hetloss	METABRIC
MB-7038	RET	cna	hetloss	METABRIC
PD11327	RET	cna	gain	BASIS
PD13296	RET	cna	gain	BASIS
PD13299	RET	cna	gain	BASIS
PD23562	RET	cna	gain	BASIS
PD23574	RET	cna	amp	BASIS
PD24186	RET	cna	gain	BASIS
PD24206	RET	cna	gain	BASIS
PD3905	RET	cna	gain	BASIS
PD4006	RET	cna	gain	BASIS
PD4107	RET	cna	gain	BASIS
PD4826	RET	cna	gain	BASIS
PD5930	RET	cna	gain	BASIS
PD5945	RET	cna	gain	BASIS
PD5948	RET	cna	gain	BASIS
PD7067	RET	cna	gain	BASIS
PD7215	RET	cna	gain	BASIS
PD8621	RET	cna	gain	BASIS
PD8980	RET	cna	hetloss	BASIS
PD9004	RET	cna	gain	BASIS
PD9585	RET	cna	hetloss	BASIS
PD9702	RET	cna	gain	BASIS
TCGA-AO-A0JL	RPS19	cna	hetloss	TCGA
TCGA-BH-A0AW	RPS19	cna	gain	TCGA
TCGA-BH-A0C0	RPS19	cna	hetloss	TCGA

TCGA-BH-A1FU	RPS19	cna	hetloss	TCGA
TCGA-D8-A27M	RPS19	cna	gain	TCGA
TCGA-E9-A1NC	RPS19	cna	gain	TCGA
TCGA-EW-A1OX	RPS19	cna	hetloss	TCGA
TCGA-LL-A5YP	RPS19	cna	gain	TCGA
MB-0346	RPS19	cna	hetloss	METABRIC
MB-6098	RPS19	cna	hetloss	METABRIC
PD11327	RPS19	cna	gain	BASIS
PD13296	RPS19	cna	hetloss	BASIS
PD13299	RPS19	cna	gain	BASIS
PD13771	RPS19	cna	hetloss	BASIS
PD23562	RPS19	cna	gain	BASIS
PD23574	RPS19	cna	gain	BASIS
PD24186	RPS19	cna	gain	BASIS
PD3905	RPS19	cna	gain	BASIS
PD4005	RPS19	cna	hetloss	BASIS
PD4006	RPS19	cna	gain	BASIS
PD4826	RPS19	cna	gain	BASIS
PD5935	RPS19	cna	gain	BASIS
PD5945	RPS19	cna	gain	BASIS
PD5948	RPS19	cna	gain	BASIS
PD6406	RPS19	cna	gain	BASIS
PD7067	RPS19	cna	gain	BASIS
PD7215	RPS19	cna	gain	BASIS
PD8621	RPS19	cna	gain	BASIS
PD8980	RPS19	cna	hetloss	BASIS
PD9585	RPS19	cna	gain	BASIS
PD9702	RPS19	cna	gain	BASIS
TCGA-AO-A0JL	TFG	cna	gain	TCGA
TCGA-BH-A0C0	TFG	cna	hetloss	TCGA
TCGA-BH-A18R	TFG	cna	gain	TCGA
TCGA-BH-A1FU	TFG	cna	hetloss	TCGA
TCGA-C8-A12L	TFG	cna	gain	TCGA
TCGA-E2-A1L7	TFG	cna	hetloss	TCGA
TCGA-LL-A5YP	TFG	cna	gain	TCGA
MB-0346	TFG	cna	hetloss	METABRIC
MTS-T0064	TFG	cna	gain	METABRIC
PD11327	TFG	cna	gain	BASIS
PD13296	TFG	cna	gain	BASIS
PD13299	TFG	cna	gain	BASIS
PD23561	TFG	cna	hetloss	BASIS
PD23574	TFG	cna	gain	BASIS
PD24186	TFG	cna	gain	BASIS
PD24206	TFG	cna	gain	BASIS
PD24337	TFG	cna	gain	BASIS
PD3905	TFG	cna	amp	BASIS
PD4005	TFG	cna	hetloss	BASIS

PD4107	TFG	cna	gain	BASIS
PD4826	TFG	cna	gain	BASIS
PD5930	TFG	cna	hetloss	BASIS
PD5935	TFG	cna	gain	BASIS
PD5945	TFG	cna	amp	BASIS
PD5948	TFG	cna	gain	BASIS
PD7067	TFG	cna	gain	BASIS
PD7215	TFG	cna	gain	BASIS
PD8621	TFG	cna	gain	BASIS
PD8980	TFG	cna	gain	BASIS
PD9004	TFG	cna	gain	BASIS
PD9702	TFG	cna	gain	BASIS
TCGA-AN-A0XU	AAMP	cna	gain	TCGA
TCGA-AO-A0JL	AAMP	cna	gain	TCGA
TCGA-BH-A0AW	AAMP	cna	hetloss	TCGA
TCGA-C8-A12L	AAMP	cna	hetloss	TCGA
TCGA-D8-A27M	AAMP	cna	gain	TCGA
TCGA-E2-A1L7	AAMP	cna	hetloss	TCGA
TCGA-E9-A1NC	AAMP	cna	hetloss	TCGA
MB-2827	AAMP	cna	hetloss	METABRIC
MB-6098	AAMP	cna	hetloss	METABRIC
MB-6271	AAMP	cna	hetloss	METABRIC
PD10014	AAMP	cna	gain	BASIS
PD11327	AAMP	cna	hetloss	BASIS
PD13296	AAMP	cna	hetloss	BASIS
PD13297	AAMP	cna	hetloss	BASIS
PD24186	AAMP	cna	gain	BASIS
PD24337	AAMP	cna	hetloss	BASIS
PD3890	AAMP	cna	hetloss	BASIS
PD3905	AAMP	cna	gain	BASIS
PD4006	AAMP	cna	gain	BASIS
PD4107	AAMP	cna	gain	BASIS
PD4967	AAMP	cna	hetloss	BASIS
PD5935	AAMP	cna	amp	BASIS
PD5945	AAMP	cna	amp	BASIS
PD5948	AAMP	cna	gain	BASIS
PD6413	AAMP	cna	hetloss	BASIS
PD6731	AAMP	cna	hetloss	BASIS
PD7067	AAMP	cna	gain	BASIS
PD7215	AAMP	cna	gain	BASIS
PD8980	AAMP	cna	gain	BASIS
PD9585	AAMP	cna	hetloss	BASIS
TCGA-AN-A0XU	ACSL3	cna	hetloss	TCGA
TCGA-AO-A0JL	ACSL3	cna	gain	TCGA
TCGA-BH-A0AW	ACSL3	cna	hetloss	TCGA
TCGA-C8-A12L	ACSL3	cna	hetloss	TCGA
TCGA-D8-A27M	ACSL3	cna	gain	TCGA

TCGA-E2-A1L7	ACSL3	cna	hetloss	TCGA
TCGA-E9-A1NC	ACSL3	cna	hetloss	TCGA
MB-2827	ACSL3	cna	hetloss	METABRIC
MB-6060	ACSL3	cna	hetloss	METABRIC
MB-6098	ACSL3	cna	hetloss	METABRIC
MB-6271	ACSL3	cna	hetloss	METABRIC
MB-7038	ACSL3	cna	gain	METABRIC
PD10014	ACSL3	cna	gain	BASIS
PD11327	ACSL3	cna	gain	BASIS
PD13296	ACSL3	cna	hetloss	BASIS
PD13297	ACSL3	cna	hetloss	BASIS
PD23578	ACSL3	cna	hetloss	BASIS
PD24186	ACSL3	cna	gain	BASIS
PD24337	ACSL3	cna	hetloss	BASIS
PD3890	ACSL3	cna	hetloss	BASIS
PD4967	ACSL3	cna	hetloss	BASIS
PD5935	ACSL3	cna	gain	BASIS
PD5945	ACSL3	cna	gain	BASIS
PD5948	ACSL3	cna	gain	BASIS
PD6413	ACSL3	cna	hetloss	BASIS
PD6731	ACSL3	cna	hetloss	BASIS
PD7067	ACSL3	cna	gain	BASIS
PD7215	ACSL3	cna	gain	BASIS
PD8980	ACSL3	cna	hetloss	BASIS
PD9585	ACSL3	cna	hetloss	BASIS
TCGA-A2-A25B	AKAP9	cna	hetloss	TCGA
TCGA-AN-A0XU	AKAP9	cna	hetloss	TCGA
TCGA-AO-A0JL	AKAP9	cna	gain	TCGA
TCGA-BH-A0AW	AKAP9	cna	gain	TCGA
TCGA-C8-A12L	AKAP9	cna	hetloss	TCGA
TCGA-D8-A27M	AKAP9	cna	gain	TCGA
TCGA-E2-A1L7	AKAP9	cna	hetloss	TCGA
TCGA-E9-A1NC	AKAP9	cna	gain	TCGA
MB-0346	AKAP9	cna	hetloss	METABRIC
MB-7048	AKAP9	cna	gain	METABRIC
PD10014	AKAP9	cna	gain	BASIS
PD11327	AKAP9	cna	amp	BASIS
PD13296	AKAP9	cna	hetloss	BASIS
PD23574	AKAP9	cna	gain	BASIS
PD24186	AKAP9	cna	amp	BASIS
PD24202	AKAP9	cna	hetloss	BASIS
PD3890	AKAP9	cna	hetloss	BASIS
PD3905	AKAP9	cna	gain	BASIS
PD4006	AKAP9	cna	gain	BASIS
PD4107	AKAP9	cna	gain	BASIS
PD4826	AKAP9	cna	gain	BASIS
PD5930	AKAP9	cna	gain	BASIS

PD5935	AKAP9	cna	gain	BASIS
PD5945	AKAP9	cna	amp	BASIS
PD5948	AKAP9	cna	gain	BASIS
PD7067	AKAP9	cna	amp	BASIS
PD7215	AKAP9	cna	gain	BASIS
PD8621	AKAP9	cna	gain	BASIS
PD9004	AKAP9	cna	gain	BASIS
PD9702	AKAP9	cna	gain	BASIS
TCGA-AN-A0XU	AKT2	cna	gain	TCGA
TCGA-AO-A0JL	AKT2	cna	hetloss	TCGA
TCGA-BH-A0AW	AKT2	cna	gain	TCGA
TCGA-BH-A0C0	AKT2	cna	hetloss	TCGA
TCGA-BH-A1FU	AKT2	cna	hetloss	TCGA
TCGA-D8-A27M	AKT2	cna	gain	TCGA
TCGA-EW-A1OX	AKT2	cna	hetloss	TCGA
TCGA-LL-A5YP	AKT2	cna	gain	TCGA
MB-0346	AKT2	cna	hetloss	METABRIC
MB-6098	AKT2	cna	gain	METABRIC
MB-7038	AKT2	cna	gain	METABRIC
MB-7048	AKT2	cna	hetloss	METABRIC
PD13296	AKT2	cna	hetloss	BASIS
PD13299	AKT2	cna	gain	BASIS
PD23562	AKT2	cna	gain	BASIS
PD23574	AKT2	cna	gain	BASIS
PD23578	AKT2	cna	gain	BASIS
PD24186	AKT2	cna	gain	BASIS
PD3905	AKT2	cna	gain	BASIS
PD4006	AKT2	cna	gain	BASIS
PD4826	AKT2	cna	gain	BASIS
PD5935	AKT2	cna	gain	BASIS
PD5945	AKT2	cna	amp	BASIS
PD5948	AKT2	cna	gain	BASIS
PD6406	AKT2	cna	gain	BASIS
PD7067	AKT2	cna	gain	BASIS
PD7215	AKT2	cna	gain	BASIS
PD8621	AKT2	cna	gain	BASIS
PD9585	AKT2	cna	gain	BASIS
PD9702	AKT2	cna	gain	BASIS
TCGA-A2-A25B	ALK	cna	hetloss	TCGA
TCGA-AN-A0XU	ALK	cna	gain	TCGA
TCGA-BH-A0AW	ALK	cna	gain	TCGA
TCGA-BH-A0C0	ALK	cna	gain	TCGA
TCGA-C8-A12L	ALK	cna	gain	TCGA
TCGA-D8-A27M	ALK	cna	gain	TCGA
TCGA-LL-A5YP	ALK	cna	gain	TCGA
MB-0346	ALK	cna	gain	METABRIC
MB-5465	ALK	cna	amp	METABRIC

MB-6060	ALK	cna	gain	METABRIC
MB-6271	ALK	cna	hetloss	METABRIC
MTS-T0064	ALK	cna	gain	METABRIC
PD10014	ALK	cna	gain	BASIS
PD11327	ALK	cna	gain	BASIS
PD13299	ALK	cna	gain	BASIS
PD23562	ALK	cna	gain	BASIS
PD23574	ALK	cna	gain	BASIS
PD24206	ALK	cna	gain	BASIS
PD3890	ALK	cna	hetloss	BASIS
PD3905	ALK	cna	gain	BASIS
PD4005	ALK	cna	hetloss	BASIS
PD4006	ALK	cna	gain	BASIS
PD5930	ALK	cna	gain	BASIS
PD5935	ALK	cna	gain	BASIS
PD5945	ALK	cna	gain	BASIS
PD5948	ALK	cna	gain	BASIS
PD6406	ALK	cna	hetloss	BASIS
PD7067	ALK	cna	gain	BASIS
PD9004	ALK	cna	gain	BASIS
PD9702	ALK	cna	gain	BASIS
TCGA-A2-A25B	ASXL2	cna	hetloss	TCGA
TCGA-AN-A0XU	ASXL2	cna	gain	TCGA
TCGA-BH-A0AW	ASXL2	cna	gain	TCGA
TCGA-BH-A0C0	ASXL2	cna	gain	TCGA
TCGA-C8-A12L	ASXL2	cna	gain	TCGA
TCGA-D8-A27M	ASXL2	cna	hetloss	TCGA
TCGA-LL-A5YP	ASXL2	cna	gain	TCGA
MB-0346	ASXL2	cna	gain	METABRIC
MB-2827	ASXL2	cna	hetloss	METABRIC
MB-5465	ASXL2	cna	amp	METABRIC
MB-6271	ASXL2	cna	hetloss	METABRIC
MB-7038	ASXL2	cna	gain	METABRIC
PD10014	ASXL2	cna	gain	BASIS
PD11327	ASXL2	cna	gain	BASIS
PD13299	ASXL2	cna	gain	BASIS
PD23562	ASXL2	cna	gain	BASIS
PD23578	ASXL2	cna	gain	BASIS
PD24186	ASXL2	cna	gain	BASIS
PD3890	ASXL2	cna	hetloss	BASIS
PD3905	ASXL2	cna	gain	BASIS
PD4006	ASXL2	cna	gain	BASIS
PD4826	ASXL2	cna	hetloss	BASIS
PD5930	ASXL2	cna	gain	BASIS
PD5935	ASXL2	cna	gain	BASIS
PD5945	ASXL2	cna	gain	BASIS
PD5948	ASXL2	cna	gain	BASIS

PD6406	ASXL2	cna	hetloss	BASIS
PD7067	ASXL2	cna	gain	BASIS
PD9004	ASXL2	cna	gain	BASIS
PD9702	ASXL2	cna	gain	BASIS
TCGA-A2-A25B	BTG1	cna	gain	TCGA
TCGA-AO-A0JL	BTG1	cna	hetloss	TCGA
TCGA-BH-A0C0	BTG1	cna	hetloss	TCGA
TCGA-BH-A18R	BTG1	cna	gain	TCGA
TCGA-BH-A1FU	BTG1	cna	hetloss	TCGA
TCGA-C8-A12L	BTG1	cna	gain	TCGA
TCGA-E2-A1L7	BTG1	cna	hetloss	TCGA
TCGA-LL-A5YP	BTG1	cna	hetloss	TCGA
MB-0346	BTG1	cna	gain	METABRIC
MB-2827	BTG1	cna	hetloss	METABRIC
MB-5070	BTG1	cna	hetloss	METABRIC
MB-5107	BTG1	cna	gain	METABRIC
MB-5465	BTG1	cna	gain	METABRIC
MB-6098	BTG1	cna	hetloss	METABRIC
PD10014	BTG1	cna	gain	BASIS
PD13296	BTG1	cna	hetloss	BASIS
PD22355	BTG1	cna	hetloss	BASIS
PD23578	BTG1	cna	gain	BASIS
PD3890	BTG1	cna	hetloss	BASIS
PD4005	BTG1	cna	hetloss	BASIS
PD4826	BTG1	cna	gain	BASIS
PD5945	BTG1	cna	gain	BASIS
PD6406	BTG1	cna	hetloss	BASIS
PD6413	BTG1	cna	hetloss	BASIS
PD6731	BTG1	cna	hetloss	BASIS
PD7067	BTG1	cna	gain	BASIS
PD7215	BTG1	cna	hetloss	BASIS
PD8621	BTG1	cna	hetloss	BASIS
PD8980	BTG1	cna	hetloss	BASIS
PD9585	BTG1	cna	hetloss	BASIS
TCGA-AN-A0XU	CASP8	cna	gain	TCGA
TCGA-AO-A0JL	CASP8	cna	gain	TCGA
TCGA-BH-A0AW	CASP8	cna	gain	TCGA
TCGA-C8-A12L	CASP8	cna	gain	TCGA
TCGA-D8-A27M	CASP8	cna	gain	TCGA
MB-2827	CASP8	cna	hetloss	METABRIC
MB-6098	CASP8	cna	hetloss	METABRIC
MB-6271	CASP8	cna	hetloss	METABRIC
MB-7032	CASP8	cna	gain	METABRIC
MB-0420	CASP8	cna	gain	METABRIC
PD10014	CASP8	cna	gain	BASIS
PD13296	CASP8	cna	hetloss	BASIS
PD13297	CASP8	cna	hetloss	BASIS

PD13299	CASP8	cna	gain	BASIS
PD24186	CASP8	cna	gain	BASIS
PD24206	CASP8	cna	gain	BASIS
PD3905	CASP8	cna	gain	BASIS
PD4006	CASP8	cna	gain	BASIS
PD4107	CASP8	cna	gain	BASIS
PD4967	CASP8	cna	hetloss	BASIS
PD5935	CASP8	cna	gain	BASIS
PD5945	CASP8	cna	gain	BASIS
PD5948	CASP8	cna	gain	BASIS
PD6413	CASP8	cna	hetloss	BASIS
PD6731	CASP8	cna	hetloss	BASIS
PD7067	CASP8	cna	gain	BASIS
PD7215	CASP8	cna	amp	BASIS
PD9004	CASP8	cna	gain	BASIS
PD9585	CASP8	cna	hetloss	BASIS
PD9702	CASP8	cna	gain	BASIS
TCGA-AO-A0JL	CBLC	cna	hetloss	TCGA
TCGA-BH-A0AW	CBLC	cna	gain	TCGA
TCGA-BH-A0C0	CBLC	cna	hetloss	TCGA
TCGA-BH-A1FU	CBLC	cna	hetloss	TCGA
TCGA-D8-A27M	CBLC	cna	gain	TCGA
TCGA-EW-A1OX	CBLC	cna	hetloss	TCGA
TCGA-LL-A5YP	CBLC	cna	gain	TCGA
MB-0346	CBLC	cna	hetloss	METABRIC
MB-6098	CBLC	cna	hetloss	METABRIC
PD11327	CBLC	cna	gain	BASIS
PD13296	CBLC	cna	hetloss	BASIS
PD13299	CBLC	cna	gain	BASIS
PD13771	CBLC	cna	hetloss	BASIS
PD23562	CBLC	cna	gain	BASIS
PD23574	CBLC	cna	gain	BASIS
PD24186	CBLC	cna	gain	BASIS
PD24206	CBLC	cna	hetloss	BASIS
PD3905	CBLC	cna	gain	BASIS
PD4005	CBLC	cna	hetloss	BASIS
PD4006	CBLC	cna	gain	BASIS
PD4826	CBLC	cna	gain	BASIS
PD5935	CBLC	cna	gain	BASIS
PD5945	CBLC	cna	amp	BASIS
PD5948	CBLC	cna	gain	BASIS
PD6406	CBLC	cna	gain	BASIS
PD7067	CBLC	cna	gain	BASIS
PD7215	CBLC	cna	gain	BASIS
PD8621	CBLC	cna	gain	BASIS
PD8980	CBLC	cna	hetloss	BASIS
PD9585	CBLC	cna	gain	BASIS

TCGA-AN-A0XU	CD22	cna	gain	TCGA
TCGA-AO-A0JL	CD22	cna	hetloss	TCGA
TCGA-BH-A0AW	CD22	cna	gain	TCGA
TCGA-BH-A0C0	CD22	cna	hetloss	TCGA
TCGA-BH-A1FU	CD22	cna	hetloss	TCGA
TCGA-C8-A12L	CD22	cna	gain	TCGA
TCGA-D8-A27M	CD22	cna	amp	TCGA
TCGA-EW-A1OX	CD22	cna	hetloss	TCGA
TCGA-LL-A5YP	CD22	cna	gain	TCGA
MB-0346	CD22	cna	hetloss	METABRIC
MB-6098	CD22	cna	gain	METABRIC
MB-7038	CD22	cna	gain	METABRIC
PD13296	CD22	cna	gain	BASIS
PD13299	CD22	cna	gain	BASIS
PD23562	CD22	cna	gain	BASIS
PD23574	CD22	cna	gain	BASIS
PD24186	CD22	cna	gain	BASIS
PD3905	CD22	cna	gain	BASIS
PD4006	CD22	cna	gain	BASIS
PD4826	CD22	cna	gain	BASIS
PD5935	CD22	cna	gain	BASIS
PD5945	CD22	cna	gain	BASIS
PD5948	CD22	cna	gain	BASIS
PD6406	CD22	cna	gain	BASIS
PD7067	CD22	cna	gain	BASIS
PD7215	CD22	cna	gain	BASIS
PD8621	CD22	cna	gain	BASIS
PD8980	CD22	cna	hetloss	BASIS
PD9585	CD22	cna	gain	BASIS
PD9702	CD22	cna	gain	BASIS
TCGA-AN-A0XU	CD58	cna	hetloss	TCGA
TCGA-BH-A0AW	CD58	cna	gain	TCGA
TCGA-BH-A0C0	CD58	cna	hetloss	TCGA
TCGA-BH-A1FU	CD58	cna	gain	TCGA
TCGA-C8-A12L	CD58	cna	amp	TCGA
TCGA-E2-A1L7	CD58	cna	gain	TCGA
TCGA-LL-A5YP	CD58	cna	gain	TCGA
MB-0346	CD58	cna	hetloss	METABRIC
MB-2827	CD58	cna	hetloss	METABRIC
MB-7032	CD58	cna	gain	METABRIC
MB-7038	CD58	cna	gain	METABRIC
PD11327	CD58	cna	gain	BASIS
PD13296	CD58	cna	hetloss	BASIS
PD13299	CD58	cna	gain	BASIS
PD22355	CD58	cna	hetloss	BASIS
PD23574	CD58	cna	gain	BASIS
PD24186	CD58	cna	gain	BASIS

PD24206	CD58	cna	gain	BASIS
PD3905	CD58	cna	gain	BASIS
PD4006	CD58	cna	gain	BASIS
PD4107	CD58	cna	gain	BASIS
PD5930	CD58	cna	gain	BASIS
PD5945	CD58	cna	gain	BASIS
PD5948	CD58	cna	gain	BASIS
PD7067	CD58	cna	amp	BASIS
PD7215	CD58	cna	gain	BASIS
PD8621	CD58	cna	gain	BASIS
PD9004	CD58	cna	hetloss	BASIS
PD9585	CD58	cna	hetloss	BASIS
PD9702	CD58	cna	gain	BASIS
TCGA-A2-A25B	CDK6	cna	hetloss	TCGA
TCGA-AN-A0XU	CDK6	cna	hetloss	TCGA
TCGA-AO-A0JL	CDK6	cna	gain	TCGA
TCGA-BH-A0AW	CDK6	cna	gain	TCGA
TCGA-C8-A12L	CDK6	cna	hetloss	TCGA
TCGA-D8-A27M	CDK6	cna	gain	TCGA
TCGA-E2-A1L7	CDK6	cna	hetloss	TCGA
TCGA-E9-A1NC	CDK6	cna	gain	TCGA
MB-0346	CDK6	cna	hetloss	METABRIC
MB-7048	CDK6	cna	gain	METABRIC
PD10014	CDK6	cna	gain	BASIS
PD11327	CDK6	cna	amp	BASIS
PD13296	CDK6	cna	hetloss	BASIS
PD23574	CDK6	cna	gain	BASIS
PD24186	CDK6	cna	amp	BASIS
PD24202	CDK6	cna	hetloss	BASIS
PD3890	CDK6	cna	hetloss	BASIS
PD3905	CDK6	cna	gain	BASIS
PD4006	CDK6	cna	gain	BASIS
PD4107	CDK6	cna	gain	BASIS
PD4826	CDK6	cna	gain	BASIS
PD5930	CDK6	cna	gain	BASIS
PD5935	CDK6	cna	gain	BASIS
PD5945	CDK6	cna	amp	BASIS
PD5948	CDK6	cna	gain	BASIS
PD7067	CDK6	cna	amp	BASIS
PD7215	CDK6	cna	gain	BASIS
PD8621	CDK6	cna	gain	BASIS
PD9004	CDK6	cna	gain	BASIS
PD9702	CDK6	cna	gain	BASIS
TCGA-A2-A25B	CDKN2A	cna	hetloss	TCGA
TCGA-AO-A0JL	CDKN2A	cna	gain	TCGA
TCGA-BH-A0AW	CDKN2A	cna	hetloss	TCGA
TCGA-BH-A1FU	CDKN2A	cna	hetloss	TCGA

TCGA-C8-A12L	CDKN2A	cna	hetloss	TCGA
TCGA-E9-A1NC	CDKN2A	cna	homdel	TCGA
MB-0346	CDKN2A	cna	hetloss	METABRIC
MB-5070	CDKN2A	cna	hetloss	METABRIC
MB-6098	CDKN2A	cna	hetloss	METABRIC
MB-0420	CDKN2A	cna	homdel	METABRIC
MTS-T0064	CDKN2A	cna	homdel	METABRIC
PD13297	CDKN2A	cna	hetloss	BASIS
PD13299	CDKN2A	cna	amp	BASIS
PD22355	CDKN2A	cna	gain	BASIS
PD23574	CDKN2A	cna	gain	BASIS
PD24202	CDKN2A	cna	hetloss	BASIS
PD24337	CDKN2A	cna	hetloss	BASIS
PD3905	CDKN2A	cna	gain	BASIS
PD4005	CDKN2A	cna	hetloss	BASIS
PD4006	CDKN2A	cna	gain	BASIS
PD4826	CDKN2A	cna	hetloss	BASIS
PD5945	CDKN2A	cna	amp	BASIS
PD5948	CDKN2A	cna	hetloss	BASIS
PD6406	CDKN2A	cna	homdel	BASIS
PD7067	CDKN2A	cna	homdel	BASIS
PD8621	CDKN2A	cna	gain	BASIS
PD8980	CDKN2A	cna	homdel	BASIS
PD9004	CDKN2A	cna	gain	BASIS
PD9585	CDKN2A	cna	hetloss	BASIS
PD9702	CDKN2A	cna	gain	BASIS
TCGA-A2-A25B	CDKN2B	cna	hetloss	TCGA
TCGA-AO-A0JL	CDKN2B	cna	gain	TCGA
TCGA-BH-A0AW	CDKN2B	cna	hetloss	TCGA
TCGA-BH-A1FU	CDKN2B	cna	hetloss	TCGA
TCGA-C8-A12L	CDKN2B	cna	hetloss	TCGA
TCGA-E9-A1NC	CDKN2B	cna	hetloss	TCGA
MB-0346	CDKN2B	cna	hetloss	METABRIC
MB-5070	CDKN2B	cna	hetloss	METABRIC
MB-6098	CDKN2B	cna	hetloss	METABRIC
MB-0420	CDKN2B	cna	homdel	METABRIC
MTS-T0064	CDKN2B	cna	homdel	METABRIC
PD13297	CDKN2B	cna	hetloss	BASIS
PD13299	CDKN2B	cna	amp	BASIS
PD22355	CDKN2B	cna	gain	BASIS
PD23574	CDKN2B	cna	gain	BASIS
PD24202	CDKN2B	cna	hetloss	BASIS
PD24337	CDKN2B	cna	hetloss	BASIS
PD3905	CDKN2B	cna	gain	BASIS
PD4005	CDKN2B	cna	hetloss	BASIS
PD4006	CDKN2B	cna	gain	BASIS
PD4826	CDKN2B	cna	hetloss	BASIS

PD5945	CDKN2B	cna	amp	BASIS
PD5948	CDKN2B	cna	hetloss	BASIS
PD6406	CDKN2B	cna	homdel	BASIS
PD7067	CDKN2B	cna	homdel	BASIS
PD8621	CDKN2B	cna	gain	BASIS
PD8980	CDKN2B	cna	homdel	BASIS
PD9004	CDKN2B	cna	gain	BASIS
PD9585	CDKN2B	cna	hetloss	BASIS
PD9702	CDKN2B	cna	gain	BASIS
TCGA-AO-A0JL	CPS1	cna	gain	TCGA
TCGA-BH-A0AW	CPS1	cna	hetloss	TCGA
TCGA-D8-A27M	CPS1	cna	amp	TCGA
TCGA-E2-A1L7	CPS1	cna	hetloss	TCGA
TCGA-E9-A1NC	CPS1	cna	hetloss	TCGA
MB-2827	CPS1	cna	hetloss	METABRIC
MB-6098	CPS1	cna	hetloss	METABRIC
MB-6271	CPS1	cna	hetloss	METABRIC
MB-7032	CPS1	cna	gain	METABRIC
PD10014	CPS1	cna	gain	BASIS
PD11327	CPS1	cna	gain	BASIS
PD13296	CPS1	cna	hetloss	BASIS
PD13297	CPS1	cna	hetloss	BASIS
PD23578	CPS1	cna	hetloss	BASIS
PD24186	CPS1	cna	gain	BASIS
PD24206	CPS1	cna	gain	BASIS
PD3890	CPS1	cna	hetloss	BASIS
PD3905	CPS1	cna	gain	BASIS
PD4006	CPS1	cna	gain	BASIS
PD4107	CPS1	cna	gain	BASIS
PD4967	CPS1	cna	hetloss	BASIS
PD5935	CPS1	cna	gain	BASIS
PD5945	CPS1	cna	gain	BASIS
PD5948	CPS1	cna	gain	BASIS
PD6413	CPS1	cna	hetloss	BASIS
PD6731	CPS1	cna	hetloss	BASIS
PD7067	CPS1	cna	gain	BASIS
PD7215	CPS1	cna	gain	BASIS
PD9004	CPS1	cna	gain	BASIS
PD9585	CPS1	cna	hetloss	BASIS
TCGA-AN-A0XU	CREB3L1	cna	hetloss	TCGA
TCGA-AO-A0JL	CREB3L1	cna	hetloss	TCGA
TCGA-BH-A0C0	CREB3L1	cna	gain	TCGA
TCGA-C8-A12L	CREB3L1	cna	hetloss	TCGA
TCGA-LL-A5YP	CREB3L1	cna	gain	TCGA
MB-0346	CREB3L1	cna	hetloss	METABRIC
MB-6098	CREB3L1	cna	hetloss	METABRIC
MB-6271	CREB3L1	cna	gain	METABRIC

MB-7048	CREB3L1	cna	hetloss	METABRIC
PD10014	CREB3L1	cna	hetloss	BASIS
PD11327	CREB3L1	cna	homdel	BASIS
PD13296	CREB3L1	cna	hetloss	BASIS
PD13771	CREB3L1	cna	hetloss	BASIS
PD23574	CREB3L1	cna	gain	BASIS
PD24186	CREB3L1	cna	gain	BASIS
PD24206	CREB3L1	cna	gain	BASIS
PD3905	CREB3L1	cna	gain	BASIS
PD4006	CREB3L1	cna	gain	BASIS
PD4107	CREB3L1	cna	gain	BASIS
PD4826	CREB3L1	cna	gain	BASIS
PD5935	CREB3L1	cna	gain	BASIS
PD5945	CREB3L1	cna	gain	BASIS
PD5948	CREB3L1	cna	gain	BASIS
PD6406	CREB3L1	cna	hetloss	BASIS
PD6413	CREB3L1	cna	hetloss	BASIS
PD7215	CREB3L1	cna	gain	BASIS
PD8621	CREB3L1	cna	gain	BASIS
PD8980	CREB3L1	cna	hetloss	BASIS
PD9585	CREB3L1	cna	hetloss	BASIS
PD9702	CREB3L1	cna	gain	BASIS
TCGA-A2-A25B	CRKL	cna	hetloss	TCGA
TCGA-BH-A0C0	CRKL	cna	gain	TCGA
TCGA-BH-A1FU	CRKL	cna	gain	TCGA
TCGA-C8-A12L	CRKL	cna	hetloss	TCGA
TCGA-E9-A1NC	CRKL	cna	gain	TCGA
TCGA-LL-A5YP	CRKL	cna	gain	TCGA
MB-0346	CRKL	cna	gain	METABRIC
MB-6098	CRKL	cna	hetloss	METABRIC
PD11327	CRKL	cna	gain	BASIS
PD11742	CRKL	cna	hetloss	BASIS
PD13296	CRKL	cna	gain	BASIS
PD14442	CRKL	cna	hetloss	BASIS
PD23574	CRKL	cna	hetloss	BASIS
PD23578	CRKL	cna	hetloss	BASIS
PD3890	CRKL	cna	hetloss	BASIS
PD3905	CRKL	cna	gain	BASIS
PD4005	CRKL	cna	gain	BASIS
PD4006	CRKL	cna	gain	BASIS
PD4107	CRKL	cna	gain	BASIS
PD4826	CRKL	cna	gain	BASIS
PD4967	CRKL	cna	hetloss	BASIS
PD5930	CRKL	cna	gain	BASIS
PD5935	CRKL	cna	gain	BASIS
PD5945	CRKL	cna	amp	BASIS
PD5948	CRKL	cna	gain	BASIS

PD6406	CRKL	cna	hetloss	BASIS
PD7067	CRKL	cna	gain	BASIS
PD8621	CRKL	cna	gain	BASIS
PD9004	CRKL	cna	gain	BASIS
PD9702	CRKL	cna	gain	BASIS
TCGA-AN-A0XU	CTLA4	cna	gain	TCGA
TCGA-AO-A0JL	CTLA4	cna	gain	TCGA
TCGA-BH-A0AW	CTLA4	cna	gain	TCGA
TCGA-C8-A12L	CTLA4	cna	gain	TCGA
TCGA-D8-A27M	CTLA4	cna	gain	TCGA
MB-2827	CTLA4	cna	hetloss	METABRIC
MB-6098	CTLA4	cna	hetloss	METABRIC
MB-6271	CTLA4	cna	hetloss	METABRIC
MB-7032	CTLA4	cna	gain	METABRIC
MB-0420	CTLA4	cna	gain	METABRIC
PD10014	CTLA4	cna	gain	BASIS
PD11327	CTLA4	cna	gain	BASIS
PD13297	CTLA4	cna	hetloss	BASIS
PD13299	CTLA4	cna	hetloss	BASIS
PD24186	CTLA4	cna	gain	BASIS
PD24206	CTLA4	cna	gain	BASIS
PD3905	CTLA4	cna	gain	BASIS
PD4006	CTLA4	cna	gain	BASIS
PD4107	CTLA4	cna	gain	BASIS
PD4967	CTLA4	cna	hetloss	BASIS
PD5935	CTLA4	cna	gain	BASIS
PD5945	CTLA4	cna	gain	BASIS
PD5948	CTLA4	cna	gain	BASIS
PD6413	CTLA4	cna	hetloss	BASIS
PD6731	CTLA4	cna	hetloss	BASIS
PD7067	CTLA4	cna	gain	BASIS
PD7215	CTLA4	cna	gain	BASIS
PD9004	CTLA4	cna	gain	BASIS
PD9585	CTLA4	cna	hetloss	BASIS
PD9702	CTLA4	cna	gain	BASIS
TCGA-A2-A25B	DDIT3	cna	gain	TCGA
TCGA-AO-A0JL	DDIT3	cna	hetloss	TCGA
TCGA-BH-A18R	DDIT3	cna	gain	TCGA
TCGA-BH-A1FU	DDIT3	cna	hetloss	TCGA
TCGA-C8-A12L	DDIT3	cna	gain	TCGA
TCGA-E2-A1L7	DDIT3	cna	hetloss	TCGA
TCGA-LL-A5YP	DDIT3	cna	hetloss	TCGA
MB-0346	DDIT3	cna	gain	METABRIC
MB-2827	DDIT3	cna	hetloss	METABRIC
MB-6060	DDIT3	cna	gain	METABRIC
MB-6098	DDIT3	cna	hetloss	METABRIC
MB-7038	DDIT3	cna	hetloss	METABRIC

MB-0420	DDIT3	cna	hetloss	METABRIC
PD11327	DDIT3	cna	homdel	BASIS
PD13296	DDIT3	cna	hetloss	BASIS
PD13297	DDIT3	cna	hetloss	BASIS
PD24186	DDIT3	cna	gain	BASIS
PD24202	DDIT3	cna	hetloss	BASIS
PD24206	DDIT3	cna	gain	BASIS
PD24337	DDIT3	cna	hetloss	BASIS
PD3890	DDIT3	cna	hetloss	BASIS
PD3905	DDIT3	cna	gain	BASIS
PD4826	DDIT3	cna	gain	BASIS
PD5945	DDIT3	cna	gain	BASIS
PD6413	DDIT3	cna	hetloss	BASIS
PD6731	DDIT3	cna	hetloss	BASIS
PD7067	DDIT3	cna	gain	BASIS
PD8621	DDIT3	cna	hetloss	BASIS
PD9585	DDIT3	cna	hetloss	BASIS
PD9702	DDIT3	cna	gain	BASIS
TCGA-A2-A25B	DKK1	cna	hetloss	TCGA
TCGA-AN-A0XU	DKK1	cna	hetloss	TCGA
TCGA-AO-A0JL	DKK1	cna	hetloss	TCGA
TCGA-BH-A0C0	DKK1	cna	gain	TCGA
TCGA-C8-A12L	DKK1	cna	gain	TCGA
TCGA-LL-A5YP	DKK1	cna	gain	TCGA
MB-0346	DKK1	cna	amp	METABRIC
MB-5070	DKK1	cna	gain	METABRIC
MB-5107	DKK1	cna	gain	METABRIC
MB-6098	DKK1	cna	hetloss	METABRIC
MB-7038	DKK1	cna	hetloss	METABRIC
PD11327	DKK1	cna	amp	BASIS
PD11742	DKK1	cna	hetloss	BASIS
PD13296	DKK1	cna	gain	BASIS
PD13299	DKK1	cna	gain	BASIS
PD24186	DKK1	cna	gain	BASIS
PD24206	DKK1	cna	gain	BASIS
PD3905	DKK1	cna	gain	BASIS
PD4006	DKK1	cna	gain	BASIS
PD4107	DKK1	cna	gain	BASIS
PD4826	DKK1	cna	gain	BASIS
PD5930	DKK1	cna	gain	BASIS
PD5945	DKK1	cna	gain	BASIS
PD5948	DKK1	cna	gain	BASIS
PD7067	DKK1	cna	gain	BASIS
PD7215	DKK1	cna	gain	BASIS
PD8621	DKK1	cna	gain	BASIS
PD8980	DKK1	cna	hetloss	BASIS
PD9585	DKK1	cna	hetloss	BASIS

PD9702	DKK1	cna	gain	BASIS
TCGA-A2-A25B	DNMT3B	cna	gain	TCGA
TCGA-AO-A0JL	DNMT3B	cna	gain	TCGA
TCGA-BH-A0AW	DNMT3B	cna	gain	TCGA
TCGA-BH-A0C0	DNMT3B	cna	gain	TCGA
TCGA-C8-A12L	DNMT3B	cna	gain	TCGA
TCGA-D8-A27M	DNMT3B	cna	gain	TCGA
TCGA-E2-A1L7	DNMT3B	cna	gain	TCGA
TCGA-E9-A1NC	DNMT3B	cna	hetloss	TCGA
TCGA-LL-A5YP	DNMT3B	cna	gain	TCGA
MB-0346	DNMT3B	cna	gain	METABRIC
MB-6060	DNMT3B	cna	gain	METABRIC
MB-6098	DNMT3B	cna	gain	METABRIC
MB-7038	DNMT3B	cna	hetloss	METABRIC
P-0010758-T01-IM5	DNMT3B	cna	amp	MSK-IMPACT
PD11327	DNMT3B	cna	homdel	BASIS
PD13296	DNMT3B	cna	hetloss	BASIS
PD13299	DNMT3B	cna	gain	BASIS
PD23574	DNMT3B	cna	gain	BASIS
PD24186	DNMT3B	cna	gain	BASIS
PD24206	DNMT3B	cna	gain	BASIS
PD3905	DNMT3B	cna	gain	BASIS
PD4107	DNMT3B	cna	amp	BASIS
PD4826	DNMT3B	cna	gain	BASIS
PD5935	DNMT3B	cna	gain	BASIS
PD5945	DNMT3B	cna	amp	BASIS
PD5948	DNMT3B	cna	gain	BASIS
PD7067	DNMT3B	cna	gain	BASIS
PD7215	DNMT3B	cna	gain	BASIS
PD9004	DNMT3B	cna	gain	BASIS
PD9702	DNMT3B	cna	gain	BASIS
TCGA-AN-A0XU	FEV	cna	gain	TCGA
TCGA-AO-A0JL	FEV	cna	gain	TCGA
TCGA-BH-A0AW	FEV	cna	hetloss	TCGA
TCGA-C8-A12L	FEV	cna	hetloss	TCGA
TCGA-D8-A27M	FEV	cna	gain	TCGA
TCGA-E2-A1L7	FEV	cna	hetloss	TCGA
TCGA-E9-A1NC	FEV	cna	hetloss	TCGA
MB-2827	FEV	cna	hetloss	METABRIC
MB-6098	FEV	cna	hetloss	METABRIC
MB-6271	FEV	cna	hetloss	METABRIC
PD10014	FEV	cna	gain	BASIS
PD11327	FEV	cna	gain	BASIS
PD13296	FEV	cna	hetloss	BASIS
PD13297	FEV	cna	hetloss	BASIS
PD24186	FEV	cna	gain	BASIS
PD24337	FEV	cna	hetloss	BASIS

PD3890	FEV	cna	hetloss	BASIS
PD3905	FEV	cna	gain	BASIS
PD4006	FEV	cna	gain	BASIS
PD4107	FEV	cna	gain	BASIS
PD4967	FEV	cna	hetloss	BASIS
PD5935	FEV	cna	gain	BASIS
PD5945	FEV	cna	amp	BASIS
PD5948	FEV	cna	gain	BASIS
PD6413	FEV	cna	hetloss	BASIS
PD6731	FEV	cna	hetloss	BASIS
PD7067	FEV	cna	gain	BASIS
PD7215	FEV	cna	gain	BASIS
PD8980	FEV	cna	hetloss	BASIS
PD9585	FEV	cna	hetloss	BASIS
TCGA-A2-A25B	GLI1	cna	gain	TCGA
TCGA-AO-A0JL	GLI1	cna	hetloss	TCGA
TCGA-BH-A18R	GLI1	cna	gain	TCGA
TCGA-BH-A1FU	GLI1	cna	hetloss	TCGA
TCGA-C8-A12L	GLI1	cna	gain	TCGA
TCGA-E2-A1L7	GLI1	cna	hetloss	TCGA
TCGA-LL-A5YP	GLI1	cna	hetloss	TCGA
MB-0346	GLI1	cna	gain	METABRIC
MB-2827	GLI1	cna	hetloss	METABRIC
MB-6060	GLI1	cna	gain	METABRIC
MB-6098	GLI1	cna	hetloss	METABRIC
MB-7038	GLI1	cna	hetloss	METABRIC
MB-0420	GLI1	cna	hetloss	METABRIC
PD11327	GLI1	cna	homdel	BASIS
PD13296	GLI1	cna	hetloss	BASIS
PD13297	GLI1	cna	hetloss	BASIS
PD24186	GLI1	cna	gain	BASIS
PD24202	GLI1	cna	hetloss	BASIS
PD24206	GLI1	cna	gain	BASIS
PD24337	GLI1	cna	hetloss	BASIS
PD3890	GLI1	cna	hetloss	BASIS
PD3905	GLI1	cna	gain	BASIS
PD4826	GLI1	cna	gain	BASIS
PD5945	GLI1	cna	gain	BASIS
PD6413	GLI1	cna	hetloss	BASIS
PD6731	GLI1	cna	hetloss	BASIS
PD7067	GLI1	cna	gain	BASIS
PD8621	GLI1	cna	hetloss	BASIS
PD9585	GLI1	cna	hetloss	BASIS
PD9702	GLI1	cna	gain	BASIS
TCGA-AN-A0XU	HIST1H1C	cna	gain	TCGA
TCGA-AO-A0JL	HIST1H1C	cna	gain	TCGA
TCGA-BH-A0AW	HIST1H1C	cna	gain	TCGA

TCGA-BH-A0C0	HIST1H1C	cna	hetloss	TCGA
TCGA-BH-A18R	HIST1H1C	cna	gain	TCGA
TCGA-C8-A12L	HIST1H1C	cna	hetloss	TCGA
MB-5465	HIST1H1C	cna	gain	METABRIC
MB-6098	HIST1H1C	cna	hetloss	METABRIC
MB-7032	HIST1H1C	cna	gain	METABRIC
PD11327	HIST1H1C	cna	hetloss	BASIS
PD13296	HIST1H1C	cna	gain	BASIS
PD13299	HIST1H1C	cna	gain	BASIS
PD23562	HIST1H1C	cna	gain	BASIS
PD23574	HIST1H1C	cna	gain	BASIS
PD24186	HIST1H1C	cna	amp	BASIS
PD24206	HIST1H1C	cna	gain	BASIS
PD3905	HIST1H1C	cna	gain	BASIS
PD4006	HIST1H1C	cna	gain	BASIS
PD4107	HIST1H1C	cna	gain	BASIS
PD4826	HIST1H1C	cna	gain	BASIS
PD5935	HIST1H1C	cna	gain	BASIS
PD5945	HIST1H1C	cna	amp	BASIS
PD5948	HIST1H1C	cna	gain	BASIS
PD6731	HIST1H1C	cna	hetloss	BASIS
PD7067	HIST1H1C	cna	amp	BASIS
PD7215	HIST1H1C	cna	gain	BASIS
PD8621	HIST1H1C	cna	amp	BASIS
PD9004	HIST1H1C	cna	gain	BASIS
PD9585	HIST1H1C	cna	gain	BASIS
PD9702	HIST1H1C	cna	gain	BASIS
TCGA-AN-A0XU	HIST1H2AL	cna	gain	TCGA
TCGA-AO-A0JL	HIST1H2AL	cna	gain	TCGA
TCGA-BH-A0AW	HIST1H2AL	cna	gain	TCGA
TCGA-BH-A0C0	HIST1H2AL	cna	hetloss	TCGA
TCGA-BH-A18R	HIST1H2AL	cna	gain	TCGA
TCGA-C8-A12L	HIST1H2AL	cna	hetloss	TCGA
MB-5465	HIST1H2AL	cna	gain	METABRIC
MB-6098	HIST1H2AL	cna	hetloss	METABRIC
MB-7032	HIST1H2AL	cna	gain	METABRIC
PD11327	HIST1H2AL	cna	hetloss	BASIS
PD13296	HIST1H2AL	cna	gain	BASIS
PD13297	HIST1H2AL	cna	hetloss	BASIS
PD13299	HIST1H2AL	cna	gain	BASIS
PD23562	HIST1H2AL	cna	gain	BASIS
PD24186	HIST1H2AL	cna	gain	BASIS
PD24206	HIST1H2AL	cna	gain	BASIS
PD3905	HIST1H2AL	cna	gain	BASIS
PD4006	HIST1H2AL	cna	gain	BASIS
PD4107	HIST1H2AL	cna	gain	BASIS
PD4826	HIST1H2AL	cna	gain	BASIS

PD5935	HIST1H2AL	cna	gain	BASIS
PD5945	HIST1H2AL	cna	amp	BASIS
PD5948	HIST1H2AL	cna	gain	BASIS
PD6731	HIST1H2AL	cna	hetloss	BASIS
PD7067	HIST1H2AL	cna	amp	BASIS
PD7215	HIST1H2AL	cna	gain	BASIS
PD8621	HIST1H2AL	cna	amp	BASIS
PD9004	HIST1H2AL	cna	gain	BASIS
PD9585	HIST1H2AL	cna	gain	BASIS
PD9702	HIST1H2AL	cna	gain	BASIS
TCGA-AN-A0XU	HIST1H2AM	cna	gain	TCGA
TCGA-AO-A0JL	HIST1H2AM	cna	gain	TCGA
TCGA-BH-A0AW	HIST1H2AM	cna	gain	TCGA
TCGA-BH-A0C0	HIST1H2AM	cna	hetloss	TCGA
TCGA-BH-A18R	HIST1H2AM	cna	gain	TCGA
TCGA-C8-A12L	HIST1H2AM	cna	hetloss	TCGA
MB-5465	HIST1H2AM	cna	gain	METABRIC
MB-6098	HIST1H2AM	cna	hetloss	METABRIC
MB-7032	HIST1H2AM	cna	gain	METABRIC
PD11327	HIST1H2AM	cna	hetloss	BASIS
PD13296	HIST1H2AM	cna	gain	BASIS
PD13297	HIST1H2AM	cna	hetloss	BASIS
PD13299	HIST1H2AM	cna	gain	BASIS
PD23562	HIST1H2AM	cna	gain	BASIS
PD24186	HIST1H2AM	cna	gain	BASIS
PD24206	HIST1H2AM	cna	gain	BASIS
PD3905	HIST1H2AM	cna	gain	BASIS
PD4006	HIST1H2AM	cna	gain	BASIS
PD4107	HIST1H2AM	cna	gain	BASIS
PD4826	HIST1H2AM	cna	gain	BASIS
PD5935	HIST1H2AM	cna	gain	BASIS
PD5945	HIST1H2AM	cna	amp	BASIS
PD5948	HIST1H2AM	cna	gain	BASIS
PD6731	HIST1H2AM	cna	hetloss	BASIS
PD7067	HIST1H2AM	cna	amp	BASIS
PD7215	HIST1H2AM	cna	gain	BASIS
PD8621	HIST1H2AM	cna	amp	BASIS
PD9004	HIST1H2AM	cna	gain	BASIS
PD9585	HIST1H2AM	cna	gain	BASIS
PD9702	HIST1H2AM	cna	gain	BASIS
TCGA-AN-A0XU	HIST1H2BO	cna	gain	TCGA
TCGA-AO-A0JL	HIST1H2BO	cna	gain	TCGA
TCGA-BH-A0AW	HIST1H2BO	cna	gain	TCGA
TCGA-BH-A0C0	HIST1H2BO	cna	hetloss	TCGA
TCGA-BH-A18R	HIST1H2BO	cna	gain	TCGA
TCGA-C8-A12L	HIST1H2BO	cna	hetloss	TCGA
MB-5465	HIST1H2BO	cna	gain	METABRIC

MB-6098	HIST1H2BO	cna	hetloss	METABRIC
MB-7032	HIST1H2BO	cna	gain	METABRIC
PD11327	HIST1H2BO	cna	hetloss	BASIS
PD13296	HIST1H2BO	cna	gain	BASIS
PD13297	HIST1H2BO	cna	hetloss	BASIS
PD13299	HIST1H2BO	cna	gain	BASIS
PD23562	HIST1H2BO	cna	gain	BASIS
PD24186	HIST1H2BO	cna	gain	BASIS
PD24206	HIST1H2BO	cna	gain	BASIS
PD3905	HIST1H2BO	cna	gain	BASIS
PD4006	HIST1H2BO	cna	gain	BASIS
PD4107	HIST1H2BO	cna	gain	BASIS
PD4826	HIST1H2BO	cna	gain	BASIS
PD5935	HIST1H2BO	cna	gain	BASIS
PD5945	HIST1H2BO	cna	amp	BASIS
PD5948	HIST1H2BO	cna	gain	BASIS
PD6731	HIST1H2BO	cna	hetloss	BASIS
PD7067	HIST1H2BO	cna	amp	BASIS
PD7215	HIST1H2BO	cna	gain	BASIS
PD8621	HIST1H2BO	cna	amp	BASIS
PD9004	HIST1H2BO	cna	gain	BASIS
PD9585	HIST1H2BO	cna	gain	BASIS
PD9702	HIST1H2BO	cna	gain	BASIS
TCGA-AN-A0XU	HIST1H3A	cna	gain	TCGA
TCGA-AO-A0JL	HIST1H3A	cna	gain	TCGA
TCGA-BH-A0AW	HIST1H3A	cna	gain	TCGA
TCGA-BH-A0C0	HIST1H3A	cna	hetloss	TCGA
TCGA-BH-A18R	HIST1H3A	cna	gain	TCGA
TCGA-C8-A12L	HIST1H3A	cna	hetloss	TCGA
MB-5465	HIST1H3A	cna	gain	METABRIC
MB-6098	HIST1H3A	cna	hetloss	METABRIC
MB-7032	HIST1H3A	cna	gain	METABRIC
PD11327	HIST1H3A	cna	hetloss	BASIS
PD13296	HIST1H3A	cna	gain	BASIS
PD13299	HIST1H3A	cna	gain	BASIS
PD23562	HIST1H3A	cna	gain	BASIS
PD23574	HIST1H3A	cna	gain	BASIS
PD24186	HIST1H3A	cna	amp	BASIS
PD24206	HIST1H3A	cna	gain	BASIS
PD3905	HIST1H3A	cna	gain	BASIS
PD4006	HIST1H3A	cna	gain	BASIS
PD4107	HIST1H3A	cna	gain	BASIS
PD4826	HIST1H3A	cna	gain	BASIS
PD5935	HIST1H3A	cna	gain	BASIS
PD5945	HIST1H3A	cna	amp	BASIS
PD5948	HIST1H3A	cna	gain	BASIS
PD6731	HIST1H3A	cna	hetloss	BASIS

PD7067	HIST1H3A	cna	amp	BASIS
PD7215	HIST1H3A	cna	gain	BASIS
PD8621	HIST1H3A	cna	amp	BASIS
PD9004	HIST1H3A	cna	gain	BASIS
PD9585	HIST1H3A	cna	gain	BASIS
PD9702	HIST1H3A	cna	gain	BASIS
TCGA-AN-A0XU	HIST1H3B	cna	gain	TCGA
TCGA-AO-A0JL	HIST1H3B	cna	gain	TCGA
TCGA-BH-A0AW	HIST1H3B	cna	gain	TCGA
TCGA-BH-A0C0	HIST1H3B	cna	hetloss	TCGA
TCGA-BH-A18R	HIST1H3B	cna	gain	TCGA
TCGA-C8-A12L	HIST1H3B	cna	hetloss	TCGA
MB-5465	HIST1H3B	cna	gain	METABRIC
MB-6098	HIST1H3B	cna	hetloss	METABRIC
MB-7032	HIST1H3B	cna	gain	METABRIC
PD11327	HIST1H3B	cna	hetloss	BASIS
PD13296	HIST1H3B	cna	gain	BASIS
PD13299	HIST1H3B	cna	gain	BASIS
PD23562	HIST1H3B	cna	gain	BASIS
PD23574	HIST1H3B	cna	gain	BASIS
PD24186	HIST1H3B	cna	amp	BASIS
PD24206	HIST1H3B	cna	gain	BASIS
PD3905	HIST1H3B	cna	gain	BASIS
PD4006	HIST1H3B	cna	gain	BASIS
PD4107	HIST1H3B	cna	gain	BASIS
PD4826	HIST1H3B	cna	gain	BASIS
PD5935	HIST1H3B	cna	gain	BASIS
PD5945	HIST1H3B	cna	amp	BASIS
PD5948	HIST1H3B	cna	gain	BASIS
PD6731	HIST1H3B	cna	hetloss	BASIS
PD7067	HIST1H3B	cna	amp	BASIS
PD7215	HIST1H3B	cna	gain	BASIS
PD8621	HIST1H3B	cna	amp	BASIS
PD9004	HIST1H3B	cna	gain	BASIS
PD9585	HIST1H3B	cna	gain	BASIS
PD9702	HIST1H3B	cna	gain	BASIS
TCGA-AO-A0JL	IKZF2	cna	gain	TCGA
TCGA-BH-A0AW	IKZF2	cna	hetloss	TCGA
TCGA-C8-A12L	IKZF2	cna	gain	TCGA
TCGA-D8-A27M	IKZF2	cna	gain	TCGA
TCGA-E2-A1L7	IKZF2	cna	hetloss	TCGA
TCGA-E9-A1NC	IKZF2	cna	hetloss	TCGA
MB-2827	IKZF2	cna	hetloss	METABRIC
MB-6098	IKZF2	cna	hetloss	METABRIC
MB-6271	IKZF2	cna	hetloss	METABRIC
PD10014	IKZF2	cna	gain	BASIS
PD11327	IKZF2	cna	gain	BASIS

PD13296	IKZF2	cna	hetloss	BASIS
PD13297	IKZF2	cna	hetloss	BASIS
PD23578	IKZF2	cna	hetloss	BASIS
PD24186	IKZF2	cna	gain	BASIS
PD24206	IKZF2	cna	gain	BASIS
PD24337	IKZF2	cna	hetloss	BASIS
PD3890	IKZF2	cna	homdel	BASIS
PD3905	IKZF2	cna	gain	BASIS
PD4107	IKZF2	cna	gain	BASIS
PD4967	IKZF2	cna	hetloss	BASIS
PD5935	IKZF2	cna	gain	BASIS
PD5945	IKZF2	cna	gain	BASIS
PD5948	IKZF2	cna	gain	BASIS
PD6413	IKZF2	cna	hetloss	BASIS
PD6731	IKZF2	cna	hetloss	BASIS
PD7067	IKZF2	cna	gain	BASIS
PD7215	IKZF2	cna	gain	BASIS
PD9585	IKZF2	cna	hetloss	BASIS
PD9702	IKZF2	cna	gain	BASIS
TCGA-AN-A0XU	IL21R	cna	gain	TCGA
TCGA-AO-A0JL	IL21R	cna	gain	TCGA
TCGA-BH-A0AW	IL21R	cna	gain	TCGA
TCGA-BH-A0C0	IL21R	cna	gain	TCGA
TCGA-C8-A12L	IL21R	cna	gain	TCGA
TCGA-E2-A1L7	IL21R	cna	hetloss	TCGA
TCGA-LL-A5YP	IL21R	cna	gain	TCGA
MB-0346	IL21R	cna	gain	METABRIC
MB-6271	IL21R	cna	amp	METABRIC
MB-7032	IL21R	cna	gain	METABRIC
MB-7038	IL21R	cna	hetloss	METABRIC
MB-0420	IL21R	cna	hetloss	METABRIC
PD10014	IL21R	cna	hetloss	BASIS
PD11327	IL21R	cna	hetloss	BASIS
PD11742	IL21R	cna	gain	BASIS
PD13299	IL21R	cna	gain	BASIS
PD24186	IL21R	cna	gain	BASIS
PD24202	IL21R	cna	hetloss	BASIS
PD3905	IL21R	cna	gain	BASIS
PD4006	IL21R	cna	gain	BASIS
PD4107	IL21R	cna	gain	BASIS
PD4826	IL21R	cna	gain	BASIS
PD5930	IL21R	cna	gain	BASIS
PD5935	IL21R	cna	gain	BASIS
PD5945	IL21R	cna	amp	BASIS
PD5948	IL21R	cna	gain	BASIS
PD7067	IL21R	cna	gain	BASIS
PD7215	IL21R	cna	amp	BASIS

PD8980	IL21R	cna	gain	BASIS
PD9004	IL21R	cna	gain	BASIS
TCGA-AN-A0XU	ITGAL	cna	gain	TCGA
TCGA-AO-A0JL	ITGAL	cna	gain	TCGA
TCGA-BH-A0AW	ITGAL	cna	gain	TCGA
TCGA-BH-A0C0	ITGAL	cna	gain	TCGA
TCGA-BH-A18R	ITGAL	cna	gain	TCGA
TCGA-C8-A12L	ITGAL	cna	gain	TCGA
TCGA-E2-A1L7	ITGAL	cna	hetloss	TCGA
TCGA-LL-A5YP	ITGAL	cna	gain	TCGA
MB-0346	ITGAL	cna	gain	METABRIC
MB-6271	ITGAL	cna	amp	METABRIC
MB-7032	ITGAL	cna	gain	METABRIC
MB-7038	ITGAL	cna	hetloss	METABRIC
MB-7048	ITGAL	cna	hetloss	METABRIC
MB-0420	ITGAL	cna	hetloss	METABRIC
PD11327	ITGAL	cna	hetloss	BASIS
PD13299	ITGAL	cna	gain	BASIS
PD24186	ITGAL	cna	gain	BASIS
PD24202	ITGAL	cna	hetloss	BASIS
PD3905	ITGAL	cna	gain	BASIS
PD4006	ITGAL	cna	gain	BASIS
PD4107	ITGAL	cna	gain	BASIS
PD4826	ITGAL	cna	gain	BASIS
PD5930	ITGAL	cna	gain	BASIS
PD5935	ITGAL	cna	gain	BASIS
PD5945	ITGAL	cna	amp	BASIS
PD5948	ITGAL	cna	gain	BASIS
PD7067	ITGAL	cna	gain	BASIS
PD7215	ITGAL	cna	amp	BASIS
PD8980	ITGAL	cna	gain	BASIS
PD9004	ITGAL	cna	gain	BASIS
TCGA-AN-A0XU	LDHA	cna	hetloss	TCGA
TCGA-AO-A0JL	LDHA	cna	hetloss	TCGA
TCGA-BH-A0C0	LDHA	cna	hetloss	TCGA
TCGA-C8-A12L	LDHA	cna	hetloss	TCGA
TCGA-E2-A1L7	LDHA	cna	gain	TCGA
TCGA-E9-A1NC	LDHA	cna	hetloss	TCGA
TCGA-LL-A5YP	LDHA	cna	gain	TCGA
MB-0346	LDHA	cna	hetloss	METABRIC
MB-6271	LDHA	cna	gain	METABRIC
MB-7048	LDHA	cna	homdel	METABRIC
MB-0420	LDHA	cna	gain	METABRIC
PD10014	LDHA	cna	hetloss	BASIS
PD11327	LDHA	cna	hetloss	BASIS
PD13296	LDHA	cna	hetloss	BASIS
PD23574	LDHA	cna	gain	BASIS

PD24186	LDHA	cna	gain	BASIS
PD24206	LDHA	cna	gain	BASIS
PD3905	LDHA	cna	gain	BASIS
PD4107	LDHA	cna	gain	BASIS
PD4826	LDHA	cna	gain	BASIS
PD5930	LDHA	cna	gain	BASIS
PD5935	LDHA	cna	gain	BASIS
PD5945	LDHA	cna	gain	BASIS
PD5948	LDHA	cna	gain	BASIS
PD6413	LDHA	cna	hetloss	BASIS
PD6731	LDHA	cna	hetloss	BASIS
PD7215	LDHA	cna	gain	BASIS
PD8621	LDHA	cna	gain	BASIS
PD8980	LDHA	cna	homdel	BASIS
PD9585	LDHA	cna	hetloss	BASIS
TCGA-A2-A25B	MEN1	cna	hetloss	TCGA
TCGA-AN-A0XU	MEN1	cna	hetloss	TCGA
TCGA-AO-A0JL	MEN1	cna	hetloss	TCGA
TCGA-BH-A18R	MEN1	cna	homdel	TCGA
TCGA-LL-A5YP	MEN1	cna	hetloss	TCGA
MB-0346	MEN1	cna	gain	METABRIC
MB-6060	MEN1	cna	gain	METABRIC
MB-6098	MEN1	cna	hetloss	METABRIC
MB-6271	MEN1	cna	hetloss	METABRIC
PD11327	MEN1	cna	hetloss	BASIS
PD13296	MEN1	cna	hetloss	BASIS
PD13771	MEN1	cna	hetloss	BASIS
PD22355	MEN1	cna	gain	BASIS
PD23574	MEN1	cna	gain	BASIS
PD24186	MEN1	cna	gain	BASIS
PD24206	MEN1	cna	gain	BASIS
PD3905	MEN1	cna	gain	BASIS
PD4006	MEN1	cna	gain	BASIS
PD4107	MEN1	cna	gain	BASIS
PD4826	MEN1	cna	gain	BASIS
PD5930	MEN1	cna	gain	BASIS
PD5935	MEN1	cna	gain	BASIS
PD5945	MEN1	cna	amp	BASIS
PD5948	MEN1	cna	gain	BASIS
PD6413	MEN1	cna	gain	BASIS
PD7067	MEN1	cna	gain	BASIS
PD7215	MEN1	cna	gain	BASIS
PD8621	MEN1	cna	gain	BASIS
PD8980	MEN1	cna	hetloss	BASIS
PD9702	MEN1	cna	gain	BASIS
TCGA-A2-A25B	MTAP	cna	hetloss	TCGA
TCGA-AO-A0JL	MTAP	cna	gain	TCGA

TCGA-BH-A0AW	MTAP	cna	hetloss	TCGA
TCGA-BH-A1FU	MTAP	cna	hetloss	TCGA
TCGA-C8-A12L	MTAP	cna	hetloss	TCGA
TCGA-E9-A1NC	MTAP	cna	homdel	TCGA
MB-0346	MTAP	cna	hetloss	METABRIC
MB-5070	MTAP	cna	hetloss	METABRIC
MB-6098	MTAP	cna	hetloss	METABRIC
MB-0420	MTAP	cna	homdel	METABRIC
MTS-T0064	MTAP	cna	homdel	METABRIC
PD13297	MTAP	cna	hetloss	BASIS
PD13299	MTAP	cna	amp	BASIS
PD22355	MTAP	cna	gain	BASIS
PD23574	MTAP	cna	gain	BASIS
PD24202	MTAP	cna	hetloss	BASIS
PD24337	MTAP	cna	hetloss	BASIS
PD3905	MTAP	cna	gain	BASIS
PD4005	MTAP	cna	hetloss	BASIS
PD4006	MTAP	cna	gain	BASIS
PD4826	MTAP	cna	hetloss	BASIS
PD5945	MTAP	cna	amp	BASIS
PD5948	MTAP	cna	hetloss	BASIS
PD6406	MTAP	cna	homdel	BASIS
PD7067	MTAP	cna	homdel	BASIS
PD8621	MTAP	cna	gain	BASIS
PD8980	MTAP	cna	homdel	BASIS
PD9004	MTAP	cna	gain	BASIS
PD9585	MTAP	cna	hetloss	BASIS
PD9702	MTAP	cna	gain	BASIS
TCGA-A2-A25B	NAV3	cna	gain	TCGA
TCGA-AN-A0XU	NAV3	cna	hetloss	TCGA
TCGA-AO-A0JL	NAV3	cna	hetloss	TCGA
TCGA-BH-A18R	NAV3	cna	gain	TCGA
TCGA-BH-A1FU	NAV3	cna	hetloss	TCGA
TCGA-C8-A12L	NAV3	cna	gain	TCGA
TCGA-E2-A1L7	NAV3	cna	hetloss	TCGA
MB-0346	NAV3	cna	gain	METABRIC
MB-2827	NAV3	cna	hetloss	METABRIC
MB-5070	NAV3	cna	hetloss	METABRIC
MB-5107	NAV3	cna	gain	METABRIC
MB-5465	NAV3	cna	gain	METABRIC
MB-6098	NAV3	cna	hetloss	METABRIC
PD10014	NAV3	cna	gain	BASIS
PD13296	NAV3	cna	hetloss	BASIS
PD22355	NAV3	cna	hetloss	BASIS
PD23578	NAV3	cna	gain	BASIS
PD24202	NAV3	cna	hetloss	BASIS
PD24206	NAV3	cna	gain	BASIS

PD3890	NAV3	cna	hetloss	BASIS
PD3905	NAV3	cna	gain	BASIS
PD4826	NAV3	cna	gain	BASIS
PD5945	NAV3	cna	gain	BASIS
PD6406	NAV3	cna	hetloss	BASIS
PD6413	NAV3	cna	hetloss	BASIS
PD6731	NAV3	cna	hetloss	BASIS
PD7067	NAV3	cna	gain	BASIS
PD8621	NAV3	cna	hetloss	BASIS
PD8980	NAV3	cna	hetloss	BASIS
PD9585	NAV3	cna	hetloss	BASIS
TCGA-A2-A25B	NCOA4	cna	hetloss	TCGA
TCGA-AN-A0XU	NCOA4	cna	hetloss	TCGA
TCGA-AO-A0JL	NCOA4	cna	hetloss	TCGA
TCGA-BH-A0C0	NCOA4	cna	gain	TCGA
TCGA-C8-A12L	NCOA4	cna	gain	TCGA
TCGA-LL-A5YP	NCOA4	cna	gain	TCGA
MB-5070	NCOA4	cna	gain	METABRIC
MB-6098	NCOA4	cna	hetloss	METABRIC
MB-7038	NCOA4	cna	hetloss	METABRIC
PD11327	NCOA4	cna	amp	BASIS
PD11742	NCOA4	cna	hetloss	BASIS
PD13296	NCOA4	cna	gain	BASIS
PD13299	NCOA4	cna	gain	BASIS
PD23562	NCOA4	cna	gain	BASIS
PD23574	NCOA4	cna	amp	BASIS
PD24186	NCOA4	cna	gain	BASIS
PD24206	NCOA4	cna	gain	BASIS
PD3905	NCOA4	cna	gain	BASIS
PD4006	NCOA4	cna	gain	BASIS
PD4107	NCOA4	cna	gain	BASIS
PD4826	NCOA4	cna	gain	BASIS
PD5930	NCOA4	cna	gain	BASIS
PD5935	NCOA4	cna	hetloss	BASIS
PD5945	NCOA4	cna	gain	BASIS
PD7067	NCOA4	cna	gain	BASIS
PD7215	NCOA4	cna	gain	BASIS
PD8621	NCOA4	cna	gain	BASIS
PD8980	NCOA4	cna	hetloss	BASIS
PD9585	NCOA4	cna	hetloss	BASIS
PD9702	NCOA4	cna	gain	BASIS
TCGA-A2-A25B	PC	cna	hetloss	TCGA
TCGA-AN-A0XU	PC	cna	hetloss	TCGA
TCGA-AO-A0JL	PC	cna	hetloss	TCGA
TCGA-BH-A18R	PC	cna	gain	TCGA
TCGA-EW-A1OX	PC	cna	gain	TCGA
TCGA-LL-A5YP	PC	cna	hetloss	TCGA

MB-0346	PC	cna	gain	METABRIC
MB-5107	PC	cna	gain	METABRIC
MB-6060	PC	cna	gain	METABRIC
MB-6271	PC	cna	gain	METABRIC
PD11327	PC	cna	hetloss	BASIS
PD11742	PC	cna	gain	BASIS
PD22355	PC	cna	gain	BASIS
PD23574	PC	cna	gain	BASIS
PD24186	PC	cna	gain	BASIS
PD3905	PC	cna	gain	BASIS
PD4006	PC	cna	amp	BASIS
PD4107	PC	cna	gain	BASIS
PD4826	PC	cna	gain	BASIS
PD5930	PC	cna	gain	BASIS
PD5935	PC	cna	gain	BASIS
PD5945	PC	cna	amp	BASIS
PD5948	PC	cna	amp	BASIS
PD6413	PC	cna	gain	BASIS
PD7067	PC	cna	gain	BASIS
PD7215	PC	cna	gain	BASIS
PD8621	PC	cna	gain	BASIS
PD8980	PC	cna	gain	BASIS
PD9004	PC	cna	gain	BASIS
PD9702	PC	cna	gain	BASIS
TCGA-A2-A25B	POFUT1	cna	gain	TCGA
TCGA-AO-A0JL	POFUT1	cna	gain	TCGA
TCGA-BH-A0AW	POFUT1	cna	gain	TCGA
TCGA-BH-A0C0	POFUT1	cna	gain	TCGA
TCGA-C8-A12L	POFUT1	cna	gain	TCGA
TCGA-D8-A27M	POFUT1	cna	gain	TCGA
TCGA-E2-A1L7	POFUT1	cna	gain	TCGA
TCGA-E9-A1NC	POFUT1	cna	hetloss	TCGA
TCGA-LL-A5YP	POFUT1	cna	gain	TCGA
MB-0346	POFUT1	cna	gain	METABRIC
MB-5070	POFUT1	cna	amp	METABRIC
MB-6060	POFUT1	cna	gain	METABRIC
MB-7038	POFUT1	cna	hetloss	METABRIC
PD11327	POFUT1	cna	homdel	BASIS
PD13296	POFUT1	cna	gain	BASIS
PD13299	POFUT1	cna	gain	BASIS
PD23574	POFUT1	cna	gain	BASIS
PD24186	POFUT1	cna	gain	BASIS
PD24206	POFUT1	cna	gain	BASIS
PD3905	POFUT1	cna	gain	BASIS
PD4107	POFUT1	cna	amp	BASIS
PD4826	POFUT1	cna	gain	BASIS
PD5930	POFUT1	cna	gain	BASIS

PD5935	POFUT1	cna	gain	BASIS
PD5945	POFUT1	cna	amp	BASIS
PD5948	POFUT1	cna	gain	BASIS
PD7067	POFUT1	cna	gain	BASIS
PD7215	POFUT1	cna	gain	BASIS
PD9004	POFUT1	cna	gain	BASIS
PD9702	POFUT1	cna	gain	BASIS
TCGA-AN-A0XU	PTPN22	cna	hetloss	TCGA
TCGA-BH-A0AW	PTPN22	cna	gain	TCGA
TCGA-BH-A0C0	PTPN22	cna	hetloss	TCGA
TCGA-BH-A1FU	PTPN22	cna	gain	TCGA
TCGA-E2-A1L7	PTPN22	cna	gain	TCGA
TCGA-LL-A5YP	PTPN22	cna	gain	TCGA
MB-0346	PTPN22	cna	hetloss	METABRIC
MB-2827	PTPN22	cna	hetloss	METABRIC
MB-7032	PTPN22	cna	gain	METABRIC
MB-7038	PTPN22	cna	gain	METABRIC
PD11327	PTPN22	cna	gain	BASIS
PD11742	PTPN22	cna	hetloss	BASIS
PD13296	PTPN22	cna	hetloss	BASIS
PD13299	PTPN22	cna	gain	BASIS
PD22355	PTPN22	cna	hetloss	BASIS
PD23574	PTPN22	cna	gain	BASIS
PD24186	PTPN22	cna	gain	BASIS
PD24206	PTPN22	cna	gain	BASIS
PD3890	PTPN22	cna	hetloss	BASIS
PD3905	PTPN22	cna	gain	BASIS
PD4006	PTPN22	cna	gain	BASIS
PD4107	PTPN22	cna	gain	BASIS
PD5930	PTPN22	cna	gain	BASIS
PD5945	PTPN22	cna	gain	BASIS
PD5948	PTPN22	cna	gain	BASIS
PD7067	PTPN22	cna	amp	BASIS
PD7215	PTPN22	cna	gain	BASIS
PD8621	PTPN22	cna	gain	BASIS
PD9585	PTPN22	cna	hetloss	BASIS
PD9702	PTPN22	cna	gain	BASIS
TCGA-A2-A25B	TEK	cna	hetloss	TCGA
TCGA-AN-A0XU	TEK	cna	hetloss	TCGA
TCGA-AO-A0JL	TEK	cna	gain	TCGA
TCGA-BH-A0AW	TEK	cna	hetloss	TCGA
TCGA-BH-A1FU	TEK	cna	hetloss	TCGA
TCGA-C8-A12L	TEK	cna	hetloss	TCGA
MB-0346	TEK	cna	hetloss	METABRIC
MB-5070	TEK	cna	hetloss	METABRIC
MB-6098	TEK	cna	gain	METABRIC
MB-0420	TEK	cna	hetloss	METABRIC

MTS-T0064	TEK	cna	homdel	METABRIC
PD13297	TEK	cna	hetloss	BASIS
PD13299	TEK	cna	gain	BASIS
PD22355	TEK	cna	gain	BASIS
PD23574	TEK	cna	gain	BASIS
PD24202	TEK	cna	hetloss	BASIS
PD24337	TEK	cna	hetloss	BASIS
PD3905	TEK	cna	gain	BASIS
PD4005	TEK	cna	hetloss	BASIS
PD4006	TEK	cna	gain	BASIS
PD4826	TEK	cna	gain	BASIS
PD5935	TEK	cna	gain	BASIS
PD5945	TEK	cna	amp	BASIS
PD6406	TEK	cna	homdel	BASIS
PD7067	TEK	cna	gain	BASIS
PD8621	TEK	cna	gain	BASIS
PD8980	TEK	cna	hetloss	BASIS
PD9004	TEK	cna	gain	BASIS
PD9585	TEK	cna	hetloss	BASIS
PD9702	TEK	cna	gain	BASIS
TCGA-A2-A25B	TET3	cna	hetloss	TCGA
TCGA-AN-A0XU	TET3	cna	gain	TCGA
TCGA-AO-A0JL	TET3	cna	gain	TCGA
TCGA-BH-A0AW	TET3	cna	gain	TCGA
TCGA-BH-A0C0	TET3	cna	gain	TCGA
TCGA-D8-A27M	TET3	cna	gain	TCGA
TCGA-LL-A5YP	TET3	cna	gain	TCGA
MB-6098	TET3	cna	gain	METABRIC
MB-6271	TET3	cna	hetloss	METABRIC
PD11327	TET3	cna	gain	BASIS
PD13296	TET3	cna	hetloss	BASIS
PD13299	TET3	cna	gain	BASIS
PD22355	TET3	cna	hetloss	BASIS
PD23562	TET3	cna	amp	BASIS
PD23574	TET3	cna	gain	BASIS
PD23578	TET3	cna	gain	BASIS
PD24186	TET3	cna	gain	BASIS
PD24206	TET3	cna	gain	BASIS
PD3890	TET3	cna	hetloss	BASIS
PD3905	TET3	cna	gain	BASIS
PD4006	TET3	cna	gain	BASIS
PD4967	TET3	cna	hetloss	BASIS
PD5930	TET3	cna	gain	BASIS
PD5935	TET3	cna	gain	BASIS
PD5945	TET3	cna	gain	BASIS
PD7067	TET3	cna	gain	BASIS
PD7215	TET3	cna	gain	BASIS

PD8621	TET3	cna	gain	BASIS
PD9004	TET3	cna	gain	BASIS
PD9702	TET3	cna	gain	BASIS
TCGA-A2-A25B	TFEB	cna	hetloss	TCGA
TCGA-AO-A0JL	TFEB	cna	gain	TCGA
TCGA-BH-A0AW	TFEB	cna	gain	TCGA
TCGA-BH-A0C0	TFEB	cna	hetloss	TCGA
TCGA-C8-A12L	TFEB	cna	hetloss	TCGA
TCGA-E2-A1L7	TFEB	cna	hetloss	TCGA
TCGA-E9-A1NC	TFEB	cna	amp	TCGA
MB-5070	TFEB	cna	gain	METABRIC
MB-6060	TFEB	cna	hetloss	METABRIC
MB-7032	TFEB	cna	gain	METABRIC
PD11327	TFEB	cna	amp	BASIS
PD13299	TFEB	cna	gain	BASIS
PD22355	TFEB	cna	gain	BASIS
PD23562	TFEB	cna	gain	BASIS
PD24186	TFEB	cna	gain	BASIS
PD24206	TFEB	cna	gain	BASIS
PD3905	TFEB	cna	amp	BASIS
PD4006	TFEB	cna	gain	BASIS
PD4107	TFEB	cna	gain	BASIS
PD4826	TFEB	cna	gain	BASIS
PD5935	TFEB	cna	gain	BASIS
PD5945	TFEB	cna	amp	BASIS
PD5948	TFEB	cna	gain	BASIS
PD7067	TFEB	cna	amp	BASIS
PD7215	TFEB	cna	gain	BASIS
PD8621	TFEB	cna	gain	BASIS
PD8980	TFEB	cna	hetloss	BASIS
PD9004	TFEB	cna	gain	BASIS
PD9585	TFEB	cna	gain	BASIS
PD9702	TFEB	cna	gain	BASIS
TCGA-A2-A25B	BACH1	cna	hetloss	TCGA
TCGA-BH-A1FU	BACH1	cna	gain	TCGA
TCGA-D8-A27M	BACH1	cna	amp	TCGA
TCGA-LL-A5YP	BACH1	cna	gain	TCGA
MB-0346	BACH1	cna	gain	METABRIC
MB-2827	BACH1	cna	hetloss	METABRIC
MB-7048	BACH1	cna	hetloss	METABRIC
PD11742	BACH1	cna	hetloss	BASIS
PD13296	BACH1	cna	gain	BASIS
PD13297	BACH1	cna	hetloss	BASIS
PD13771	BACH1	cna	gain	BASIS
PD22355	BACH1	cna	hetloss	BASIS
PD23562	BACH1	cna	gain	BASIS
PD23578	BACH1	cna	gain	BASIS

PD24186	BACH1	cna	gain	BASIS
PD24202	BACH1	cna	gain	BASIS
PD24206	BACH1	cna	hetloss	BASIS
PD3905	BACH1	cna	gain	BASIS
PD4107	BACH1	cna	gain	BASIS
PD4826	BACH1	cna	gain	BASIS
PD5930	BACH1	cna	gain	BASIS
PD5945	BACH1	cna	gain	BASIS
PD6731	BACH1	cna	gain	BASIS
PD7067	BACH1	cna	gain	BASIS
PD7215	BACH1	cna	gain	BASIS
PD8621	BACH1	cna	gain	BASIS
PD8980	BACH1	cna	hetloss	BASIS
PD9004	BACH1	cna	amp	BASIS
PD9702	BACH1	cna	gain	BASIS
TCGA-AO-A0JL	BARD1	cna	gain	TCGA
TCGA-BH-A0AW	BARD1	cna	hetloss	TCGA
TCGA-C8-A12L	BARD1	cna	gain	TCGA
TCGA-D8-A27M	BARD1	cna	gain	TCGA
TCGA-E2-A1L7	BARD1	cna	hetloss	TCGA
TCGA-E9-A1NC	BARD1	cna	hetloss	TCGA
MB-2827	BARD1	cna	hetloss	METABRIC
MB-6098	BARD1	cna	hetloss	METABRIC
MB-6271	BARD1	cna	hetloss	METABRIC
PD10014	BARD1	cna	gain	BASIS
PD11327	BARD1	cna	gain	BASIS
PD13296	BARD1	cna	hetloss	BASIS
PD13297	BARD1	cna	hetloss	BASIS
PD23578	BARD1	cna	hetloss	BASIS
PD24186	BARD1	cna	gain	BASIS
PD24206	BARD1	cna	gain	BASIS
PD24337	BARD1	cna	hetloss	BASIS
PD3890	BARD1	cna	hetloss	BASIS
PD3905	BARD1	cna	gain	BASIS
PD4107	BARD1	cna	gain	BASIS
PD4967	BARD1	cna	hetloss	BASIS
PD5935	BARD1	cna	gain	BASIS
PD5945	BARD1	cna	gain	BASIS
PD5948	BARD1	cna	gain	BASIS
PD6413	BARD1	cna	hetloss	BASIS
PD6731	BARD1	cna	hetloss	BASIS
PD7067	BARD1	cna	gain	BASIS
PD7215	BARD1	cna	gain	BASIS
PD9585	BARD1	cna	hetloss	BASIS
TCGA-A2-A25B	CDC42EP2	cna	hetloss	TCGA
TCGA-AN-A0XU	CDC42EP2	cna	hetloss	TCGA
TCGA-AO-A0JL	CDC42EP2	cna	hetloss	TCGA

TCGA-BH-A18R	CDC42EP2	cna	homdel	TCGA
TCGA-LL-A5YP	CDC42EP2	cna	hetloss	TCGA
MB-0346	CDC42EP2	cna	gain	METABRIC
MB-6060	CDC42EP2	cna	gain	METABRIC
MB-6098	CDC42EP2	cna	hetloss	METABRIC
MB-6271	CDC42EP2	cna	hetloss	METABRIC
PD11327	CDC42EP2	cna	hetloss	BASIS
PD13296	CDC42EP2	cna	hetloss	BASIS
PD13771	CDC42EP2	cna	hetloss	BASIS
PD22355	CDC42EP2	cna	gain	BASIS
PD23574	CDC42EP2	cna	gain	BASIS
PD24186	CDC42EP2	cna	gain	BASIS
PD3905	CDC42EP2	cna	gain	BASIS
PD4006	CDC42EP2	cna	amp	BASIS
PD4107	CDC42EP2	cna	gain	BASIS
PD4826	CDC42EP2	cna	gain	BASIS
PD5930	CDC42EP2	cna	gain	BASIS
PD5935	CDC42EP2	cna	gain	BASIS
PD5945	CDC42EP2	cna	amp	BASIS
PD5948	CDC42EP2	cna	gain	BASIS
PD6413	CDC42EP2	cna	gain	BASIS
PD7067	CDC42EP2	cna	gain	BASIS
PD7215	CDC42EP2	cna	gain	BASIS
PD8621	CDC42EP2	cna	gain	BASIS
PD8980	CDC42EP2	cna	hetloss	BASIS
PD9702	CDC42EP2	cna	gain	BASIS
TCGA-A2-A25B	CDK4	cna	gain	TCGA
TCGA-AO-A0JL	CDK4	cna	hetloss	TCGA
TCGA-BH-A18R	CDK4	cna	gain	TCGA
TCGA-BH-A1FU	CDK4	cna	hetloss	TCGA
TCGA-C8-A12L	CDK4	cna	gain	TCGA
TCGA-E2-A1L7	CDK4	cna	hetloss	TCGA
TCGA-LL-A5YP	CDK4	cna	hetloss	TCGA
MB-0346	CDK4	cna	gain	METABRIC
MB-2827	CDK4	cna	hetloss	METABRIC
MB-6060	CDK4	cna	gain	METABRIC
MB-6098	CDK4	cna	hetloss	METABRIC
MB-0420	CDK4	cna	hetloss	METABRIC
PD11327	CDK4	cna	homdel	BASIS
PD13296	CDK4	cna	hetloss	BASIS
PD13297	CDK4	cna	hetloss	BASIS
PD24186	CDK4	cna	gain	BASIS
PD24202	CDK4	cna	hetloss	BASIS
PD24206	CDK4	cna	gain	BASIS
PD24337	CDK4	cna	hetloss	BASIS
PD3890	CDK4	cna	hetloss	BASIS
PD3905	CDK4	cna	gain	BASIS

PD4826	CDK4	cna	gain	BASIS
PD5945	CDK4	cna	gain	BASIS
PD6413	CDK4	cna	hetloss	BASIS
PD6731	CDK4	cna	hetloss	BASIS
PD7067	CDK4	cna	gain	BASIS
PD8621	CDK4	cna	hetloss	BASIS
PD9585	CDK4	cna	hetloss	BASIS
PD9702	CDK4	cna	gain	BASIS
TCGA-AN-A0XU	CDKN1A	cna	hetloss	TCGA
TCGA-AO-A0JL	CDKN1A	cna	gain	TCGA
TCGA-BH-A0AW	CDKN1A	cna	gain	TCGA
TCGA-BH-A0C0	CDKN1A	cna	hetloss	TCGA
TCGA-C8-A12L	CDKN1A	cna	hetloss	TCGA
TCGA-E2-A1L7	CDKN1A	cna	hetloss	TCGA
TCGA-E9-A1NC	CDKN1A	cna	amp	TCGA
MB-6098	CDKN1A	cna	gain	METABRIC
MB-7032	CDKN1A	cna	gain	METABRIC
PD11327	CDKN1A	cna	hetloss	BASIS
PD13296	CDKN1A	cna	hetloss	BASIS
PD13299	CDKN1A	cna	gain	BASIS
PD23562	CDKN1A	cna	gain	BASIS
PD24186	CDKN1A	cna	gain	BASIS
PD24206	CDKN1A	cna	gain	BASIS
PD3905	CDKN1A	cna	gain	BASIS
PD4006	CDKN1A	cna	gain	BASIS
PD4107	CDKN1A	cna	gain	BASIS
PD4826	CDKN1A	cna	gain	BASIS
PD5935	CDKN1A	cna	gain	BASIS
PD5945	CDKN1A	cna	amp	BASIS
PD5948	CDKN1A	cna	gain	BASIS
PD6731	CDKN1A	cna	hetloss	BASIS
PD7067	CDKN1A	cna	amp	BASIS
PD7215	CDKN1A	cna	gain	BASIS
PD8621	CDKN1A	cna	amp	BASIS
PD9004	CDKN1A	cna	gain	BASIS
PD9585	CDKN1A	cna	gain	BASIS
PD9702	CDKN1A	cna	gain	BASIS
TCGA-AO-A0JL	CEBPA	cna	hetloss	TCGA
TCGA-BH-A0AW	CEBPA	cna	gain	TCGA
TCGA-BH-A0C0	CEBPA	cna	hetloss	TCGA
TCGA-BH-A1FU	CEBPA	cna	hetloss	TCGA
TCGA-C8-A12L	CEBPA	cna	gain	TCGA
TCGA-D8-A27M	CEBPA	cna	amp	TCGA
TCGA-EW-A1OX	CEBPA	cna	hetloss	TCGA
TCGA-LL-A5YP	CEBPA	cna	gain	TCGA
MB-0346	CEBPA	cna	hetloss	METABRIC
MB-6098	CEBPA	cna	gain	METABRIC

MB-7038	CEBPA	cna	gain	METABRIC
PD13296	CEBPA	cna	gain	BASIS
PD13299	CEBPA	cna	gain	BASIS
PD23562	CEBPA	cna	gain	BASIS
PD23574	CEBPA	cna	gain	BASIS
PD24186	CEBPA	cna	gain	BASIS
PD3905	CEBPA	cna	gain	BASIS
PD4006	CEBPA	cna	gain	BASIS
PD4826	CEBPA	cna	gain	BASIS
PD4967	CEBPA	cna	gain	BASIS
PD5935	CEBPA	cna	gain	BASIS
PD5945	CEBPA	cna	gain	BASIS
PD5948	CEBPA	cna	gain	BASIS
PD7067	CEBPA	cna	gain	BASIS
PD7215	CEBPA	cna	gain	BASIS
PD8621	CEBPA	cna	gain	BASIS
PD8980	CEBPA	cna	hetloss	BASIS
PD9585	CEBPA	cna	gain	BASIS
PD9702	CEBPA	cna	gain	BASIS
TCGA-AO-A0JL	ERCC3	cna	gain	TCGA
TCGA-D8-A27M	ERCC3	cna	gain	TCGA
TCGA-E9-A1NC	ERCC3	cna	gain	TCGA
TCGA-LL-A5YP	ERCC3	cna	hetloss	TCGA
MB-2827	ERCC3	cna	hetloss	METABRIC
MB-5107	ERCC3	cna	hetloss	METABRIC
MB-6098	ERCC3	cna	hetloss	METABRIC
MB-6271	ERCC3	cna	hetloss	METABRIC
PD11327	ERCC3	cna	gain	BASIS
PD11742	ERCC3	cna	hetloss	BASIS
PD13297	ERCC3	cna	hetloss	BASIS
PD13299	ERCC3	cna	gain	BASIS
PD23574	ERCC3	cna	gain	BASIS
PD23578	ERCC3	cna	hetloss	BASIS
PD24186	ERCC3	cna	gain	BASIS
PD24202	ERCC3	cna	hetloss	BASIS
PD24206	ERCC3	cna	gain	BASIS
PD3905	ERCC3	cna	gain	BASIS
PD4107	ERCC3	cna	gain	BASIS
PD4967	ERCC3	cna	hetloss	BASIS
PD5935	ERCC3	cna	hetloss	BASIS
PD5945	ERCC3	cna	gain	BASIS
PD5948	ERCC3	cna	gain	BASIS
PD6406	ERCC3	cna	hetloss	BASIS
PD6413	ERCC3	cna	hetloss	BASIS
PD7067	ERCC3	cna	gain	BASIS
PD7215	ERCC3	cna	gain	BASIS
PD9004	ERCC3	cna	gain	BASIS

PD9702	ERCC3	cna	gain	BASIS
TCGA-AN-A0XU	FAM46C	cna	hetloss	TCGA
TCGA-BH-A0AW	FAM46C	cna	gain	TCGA
TCGA-BH-A0C0	FAM46C	cna	hetloss	TCGA
TCGA-BH-A1FU	FAM46C	cna	gain	TCGA
TCGA-C8-A12L	FAM46C	cna	amp	TCGA
TCGA-E2-A1L7	FAM46C	cna	gain	TCGA
TCGA-LL-A5YP	FAM46C	cna	gain	TCGA
MB-0346	FAM46C	cna	hetloss	METABRIC
MB-2827	FAM46C	cna	hetloss	METABRIC
MB-7032	FAM46C	cna	gain	METABRIC
MB-7038	FAM46C	cna	gain	METABRIC
PD11327	FAM46C	cna	gain	BASIS
PD13296	FAM46C	cna	hetloss	BASIS
PD13299	FAM46C	cna	gain	BASIS
PD22355	FAM46C	cna	hetloss	BASIS
PD23574	FAM46C	cna	gain	BASIS
PD24186	FAM46C	cna	gain	BASIS
PD24206	FAM46C	cna	gain	BASIS
PD3905	FAM46C	cna	gain	BASIS
PD4006	FAM46C	cna	gain	BASIS
PD4107	FAM46C	cna	gain	BASIS
PD5930	FAM46C	cna	gain	BASIS
PD5945	FAM46C	cna	gain	BASIS
PD5948	FAM46C	cna	gain	BASIS
PD7067	FAM46C	cna	amp	BASIS
PD7215	FAM46C	cna	gain	BASIS
PD8621	FAM46C	cna	gain	BASIS
PD9585	FAM46C	cna	hetloss	BASIS
PD9702	FAM46C	cna	gain	BASIS
TCGA-AN-A0XU	FANCF	cna	hetloss	TCGA
TCGA-AO-A0JL	FANCF	cna	hetloss	TCGA
TCGA-BH-A0C0	FANCF	cna	hetloss	TCGA
TCGA-C8-A12L	FANCF	cna	hetloss	TCGA
TCGA-LL-A5YP	FANCF	cna	gain	TCGA
MB-0346	FANCF	cna	hetloss	METABRIC
MB-6098	FANCF	cna	hetloss	METABRIC
MB-6271	FANCF	cna	gain	METABRIC
MB-7048	FANCF	cna	hetloss	METABRIC
MB-0420	FANCF	cna	amp	METABRIC
PD10014	FANCF	cna	hetloss	BASIS
PD13296	FANCF	cna	gain	BASIS
PD23574	FANCF	cna	gain	BASIS
PD24186	FANCF	cna	gain	BASIS
PD24206	FANCF	cna	gain	BASIS
PD3905	FANCF	cna	gain	BASIS
PD4006	FANCF	cna	hetloss	BASIS

PD4107	FANCF	cna	gain	BASIS
PD4826	FANCF	cna	gain	BASIS
PD5930	FANCF	cna	gain	BASIS
PD5935	FANCF	cna	gain	BASIS
PD5945	FANCF	cna	gain	BASIS
PD5948	FANCF	cna	gain	BASIS
PD6413	FANCF	cna	hetloss	BASIS
PD6731	FANCF	cna	hetloss	BASIS
PD7215	FANCF	cna	gain	BASIS
PD8621	FANCF	cna	gain	BASIS
PD8980	FANCF	cna	hetloss	BASIS
PD9585	FANCF	cna	hetloss	BASIS
TCGA-AN-A0XU	HIST1H1D	cna	gain	TCGA
TCGA-AO-A0JL	HIST1H1D	cna	gain	TCGA
TCGA-BH-A0AW	HIST1H1D	cna	gain	TCGA
TCGA-BH-A0C0	HIST1H1D	cna	hetloss	TCGA
TCGA-BH-A18R	HIST1H1D	cna	gain	TCGA
TCGA-C8-A12L	HIST1H1D	cna	hetloss	TCGA
MB-5465	HIST1H1D	cna	gain	METABRIC
MB-6098	HIST1H1D	cna	hetloss	METABRIC
MB-7032	HIST1H1D	cna	gain	METABRIC
PD11327	HIST1H1D	cna	hetloss	BASIS
PD13296	HIST1H1D	cna	gain	BASIS
PD13299	HIST1H1D	cna	gain	BASIS
PD23562	HIST1H1D	cna	gain	BASIS
PD24186	HIST1H1D	cna	amp	BASIS
PD24206	HIST1H1D	cna	gain	BASIS
PD3905	HIST1H1D	cna	gain	BASIS
PD4006	HIST1H1D	cna	gain	BASIS
PD4107	HIST1H1D	cna	gain	BASIS
PD4826	HIST1H1D	cna	gain	BASIS
PD5935	HIST1H1D	cna	gain	BASIS
PD5945	HIST1H1D	cna	amp	BASIS
PD5948	HIST1H1D	cna	gain	BASIS
PD6731	HIST1H1D	cna	hetloss	BASIS
PD7067	HIST1H1D	cna	amp	BASIS
PD7215	HIST1H1D	cna	gain	BASIS
PD8621	HIST1H1D	cna	amp	BASIS
PD9004	HIST1H1D	cna	gain	BASIS
PD9585	HIST1H1D	cna	gain	BASIS
PD9702	HIST1H1D	cna	gain	BASIS
TCGA-AN-A0XU	HIST1H1E	cna	gain	TCGA
TCGA-AO-A0JL	HIST1H1E	cna	gain	TCGA
TCGA-BH-A0AW	HIST1H1E	cna	gain	TCGA
TCGA-BH-A0C0	HIST1H1E	cna	hetloss	TCGA
TCGA-BH-A18R	HIST1H1E	cna	gain	TCGA
TCGA-C8-A12L	HIST1H1E	cna	hetloss	TCGA

MB-5465	HIST1H1E	cna	gain	METABRIC
MB-6098	HIST1H1E	cna	hetloss	METABRIC
MB-7032	HIST1H1E	cna	gain	METABRIC
PD11327	HIST1H1E	cna	hetloss	BASIS
PD13296	HIST1H1E	cna	gain	BASIS
PD13299	HIST1H1E	cna	gain	BASIS
PD23562	HIST1H1E	cna	gain	BASIS
PD24186	HIST1H1E	cna	amp	BASIS
PD24206	HIST1H1E	cna	gain	BASIS
PD3905	HIST1H1E	cna	gain	BASIS
PD4006	HIST1H1E	cna	gain	BASIS
PD4107	HIST1H1E	cna	gain	BASIS
PD4826	HIST1H1E	cna	gain	BASIS
PD5935	HIST1H1E	cna	gain	BASIS
PD5945	HIST1H1E	cna	amp	BASIS
PD5948	HIST1H1E	cna	gain	BASIS
PD6731	HIST1H1E	cna	hetloss	BASIS
PD7067	HIST1H1E	cna	amp	BASIS
PD7215	HIST1H1E	cna	gain	BASIS
PD8621	HIST1H1E	cna	amp	BASIS
PD9004	HIST1H1E	cna	gain	BASIS
PD9585	HIST1H1E	cna	gain	BASIS
PD9702	HIST1H1E	cna	gain	BASIS
TCGA-AN-A0XU	HIST1H2AC	cna	gain	TCGA
TCGA-AO-A0JL	HIST1H2AC	cna	gain	TCGA
TCGA-BH-A0AW	HIST1H2AC	cna	gain	TCGA
TCGA-BH-A0C0	HIST1H2AC	cna	hetloss	TCGA
TCGA-BH-A18R	HIST1H2AC	cna	gain	TCGA
TCGA-C8-A12L	HIST1H2AC	cna	hetloss	TCGA
MB-5465	HIST1H2AC	cna	gain	METABRIC
MB-6098	HIST1H2AC	cna	hetloss	METABRIC
MB-7032	HIST1H2AC	cna	gain	METABRIC
PD11327	HIST1H2AC	cna	hetloss	BASIS
PD13296	HIST1H2AC	cna	gain	BASIS
PD13299	HIST1H2AC	cna	gain	BASIS
PD23562	HIST1H2AC	cna	gain	BASIS
PD24186	HIST1H2AC	cna	amp	BASIS
PD24206	HIST1H2AC	cna	gain	BASIS
PD3905	HIST1H2AC	cna	gain	BASIS
PD4006	HIST1H2AC	cna	gain	BASIS
PD4107	HIST1H2AC	cna	gain	BASIS
PD4826	HIST1H2AC	cna	gain	BASIS
PD5935	HIST1H2AC	cna	gain	BASIS
PD5945	HIST1H2AC	cna	amp	BASIS
PD5948	HIST1H2AC	cna	gain	BASIS
PD6731	HIST1H2AC	cna	hetloss	BASIS
PD7067	HIST1H2AC	cna	amp	BASIS

PD7215	HIST1H2AC	cna	gain	BASIS
PD8621	HIST1H2AC	cna	amp	BASIS
PD9004	HIST1H2AC	cna	gain	BASIS
PD9585	HIST1H2AC	cna	gain	BASIS
PD9702	HIST1H2AC	cna	gain	BASIS
TCGA-AN-A0XU	HIST1H2AG	cna	gain	TCGA
TCGA-AO-A0JL	HIST1H2AG	cna	gain	TCGA
TCGA-BH-A0AW	HIST1H2AG	cna	gain	TCGA
TCGA-BH-A0C0	HIST1H2AG	cna	hetloss	TCGA
TCGA-BH-A18R	HIST1H2AG	cna	gain	TCGA
TCGA-C8-A12L	HIST1H2AG	cna	hetloss	TCGA
MB-5465	HIST1H2AG	cna	gain	METABRIC
MB-6098	HIST1H2AG	cna	hetloss	METABRIC
MB-7032	HIST1H2AG	cna	gain	METABRIC
PD11327	HIST1H2AG	cna	hetloss	BASIS
PD13296	HIST1H2AG	cna	gain	BASIS
PD13299	HIST1H2AG	cna	gain	BASIS
PD23562	HIST1H2AG	cna	gain	BASIS
PD24186	HIST1H2AG	cna	gain	BASIS
PD24206	HIST1H2AG	cna	gain	BASIS
PD3905	HIST1H2AG	cna	gain	BASIS
PD4006	HIST1H2AG	cna	gain	BASIS
PD4107	HIST1H2AG	cna	gain	BASIS
PD4826	HIST1H2AG	cna	gain	BASIS
PD5935	HIST1H2AG	cna	gain	BASIS
PD5945	HIST1H2AG	cna	amp	BASIS
PD5948	HIST1H2AG	cna	gain	BASIS
PD6731	HIST1H2AG	cna	hetloss	BASIS
PD7067	HIST1H2AG	cna	amp	BASIS
PD7215	HIST1H2AG	cna	gain	BASIS
PD8621	HIST1H2AG	cna	amp	BASIS
PD9004	HIST1H2AG	cna	gain	BASIS
PD9585	HIST1H2AG	cna	gain	BASIS
PD9702	HIST1H2AG	cna	gain	BASIS
TCGA-AN-A0XU	HIST1H2BC	cna	gain	TCGA
TCGA-AO-A0JL	HIST1H2BC	cna	gain	TCGA
TCGA-BH-A0AW	HIST1H2BC	cna	gain	TCGA
TCGA-BH-A0C0	HIST1H2BC	cna	hetloss	TCGA
TCGA-BH-A18R	HIST1H2BC	cna	gain	TCGA
TCGA-C8-A12L	HIST1H2BC	cna	hetloss	TCGA
MB-5465	HIST1H2BC	cna	gain	METABRIC
MB-6098	HIST1H2BC	cna	hetloss	METABRIC
MB-7032	HIST1H2BC	cna	gain	METABRIC
PD11327	HIST1H2BC	cna	hetloss	BASIS
PD13296	HIST1H2BC	cna	gain	BASIS
PD13299	HIST1H2BC	cna	gain	BASIS
PD23562	HIST1H2BC	cna	gain	BASIS

PD24186	HIST1H2BC	cna	amp	BASIS
PD24206	HIST1H2BC	cna	gain	BASIS
PD3905	HIST1H2BC	cna	gain	BASIS
PD4006	HIST1H2BC	cna	gain	BASIS
PD4107	HIST1H2BC	cna	gain	BASIS
PD4826	HIST1H2BC	cna	gain	BASIS
PD5935	HIST1H2BC	cna	gain	BASIS
PD5945	HIST1H2BC	cna	amp	BASIS
PD5948	HIST1H2BC	cna	gain	BASIS
PD6731	HIST1H2BC	cna	hetloss	BASIS
PD7067	HIST1H2BC	cna	amp	BASIS
PD7215	HIST1H2BC	cna	gain	BASIS
PD8621	HIST1H2BC	cna	amp	BASIS
PD9004	HIST1H2BC	cna	gain	BASIS
PD9585	HIST1H2BC	cna	gain	BASIS
PD9702	HIST1H2BC	cna	gain	BASIS
TCGA-AN-A0XU	HIST1H2BD	cna	gain	TCGA
TCGA-AO-A0JL	HIST1H2BD	cna	gain	TCGA
TCGA-BH-A0AW	HIST1H2BD	cna	gain	TCGA
TCGA-BH-A0C0	HIST1H2BD	cna	hetloss	TCGA
TCGA-BH-A18R	HIST1H2BD	cna	gain	TCGA
TCGA-C8-A12L	HIST1H2BD	cna	hetloss	TCGA
MB-5465	HIST1H2BD	cna	gain	METABRIC
MB-6098	HIST1H2BD	cna	hetloss	METABRIC
MB-7032	HIST1H2BD	cna	gain	METABRIC
PD11327	HIST1H2BD	cna	hetloss	BASIS
PD13296	HIST1H2BD	cna	gain	BASIS
PD13299	HIST1H2BD	cna	gain	BASIS
PD23562	HIST1H2BD	cna	gain	BASIS
PD24186	HIST1H2BD	cna	amp	BASIS
PD24206	HIST1H2BD	cna	gain	BASIS
PD3905	HIST1H2BD	cna	gain	BASIS
PD4006	HIST1H2BD	cna	gain	BASIS
PD4107	HIST1H2BD	cna	gain	BASIS
PD4826	HIST1H2BD	cna	gain	BASIS
PD5935	HIST1H2BD	cna	gain	BASIS
PD5945	HIST1H2BD	cna	amp	BASIS
PD5948	HIST1H2BD	cna	gain	BASIS
PD6731	HIST1H2BD	cna	hetloss	BASIS
PD7067	HIST1H2BD	cna	amp	BASIS
PD7215	HIST1H2BD	cna	gain	BASIS
PD8621	HIST1H2BD	cna	amp	BASIS
PD9004	HIST1H2BD	cna	gain	BASIS
PD9585	HIST1H2BD	cna	gain	BASIS
PD9702	HIST1H2BD	cna	gain	BASIS
TCGA-AN-A0XU	HIST1H2BJ	cna	gain	TCGA
TCGA-AO-A0JL	HIST1H2BJ	cna	gain	TCGA

TCGA-BH-A0AW	HIST1H2BJ	cna	gain	TCGA
TCGA-BH-A0C0	HIST1H2BJ	cna	hetloss	TCGA
TCGA-BH-A18R	HIST1H2BJ	cna	gain	TCGA
TCGA-C8-A12L	HIST1H2BJ	cna	hetloss	TCGA
MB-5465	HIST1H2BJ	cna	gain	METABRIC
MB-6098	HIST1H2BJ	cna	hetloss	METABRIC
MB-7032	HIST1H2BJ	cna	gain	METABRIC
PD11327	HIST1H2BJ	cna	hetloss	BASIS
PD13296	HIST1H2BJ	cna	gain	BASIS
PD13299	HIST1H2BJ	cna	gain	BASIS
PD23562	HIST1H2BJ	cna	gain	BASIS
PD24186	HIST1H2BJ	cna	gain	BASIS
PD24206	HIST1H2BJ	cna	gain	BASIS
PD3905	HIST1H2BJ	cna	gain	BASIS
PD4006	HIST1H2BJ	cna	gain	BASIS
PD4107	HIST1H2BJ	cna	gain	BASIS
PD4826	HIST1H2BJ	cna	gain	BASIS
PD5935	HIST1H2BJ	cna	gain	BASIS
PD5945	HIST1H2BJ	cna	amp	BASIS
PD5948	HIST1H2BJ	cna	gain	BASIS
PD6731	HIST1H2BJ	cna	hetloss	BASIS
PD7067	HIST1H2BJ	cna	amp	BASIS
PD7215	HIST1H2BJ	cna	gain	BASIS
PD8621	HIST1H2BJ	cna	amp	BASIS
PD9004	HIST1H2BJ	cna	gain	BASIS
PD9585	HIST1H2BJ	cna	gain	BASIS
PD9702	HIST1H2BJ	cna	gain	BASIS
TCGA-AN-A0XU	HIST1H2BK	cna	gain	TCGA
TCGA-AO-A0JL	HIST1H2BK	cna	gain	TCGA
TCGA-BH-A0AW	HIST1H2BK	cna	gain	TCGA
TCGA-BH-A0C0	HIST1H2BK	cna	hetloss	TCGA
TCGA-BH-A18R	HIST1H2BK	cna	gain	TCGA
TCGA-C8-A12L	HIST1H2BK	cna	hetloss	TCGA
MB-5465	HIST1H2BK	cna	gain	METABRIC
MB-6098	HIST1H2BK	cna	hetloss	METABRIC
MB-7032	HIST1H2BK	cna	gain	METABRIC
PD11327	HIST1H2BK	cna	hetloss	BASIS
PD13296	HIST1H2BK	cna	gain	BASIS
PD13299	HIST1H2BK	cna	gain	BASIS
PD23562	HIST1H2BK	cna	gain	BASIS
PD24186	HIST1H2BK	cna	gain	BASIS
PD24206	HIST1H2BK	cna	gain	BASIS
PD3905	HIST1H2BK	cna	gain	BASIS
PD4006	HIST1H2BK	cna	gain	BASIS
PD4107	HIST1H2BK	cna	gain	BASIS
PD4826	HIST1H2BK	cna	gain	BASIS
PD5935	HIST1H2BK	cna	gain	BASIS

PD5945	HIST1H2BK	cna	amp	BASIS
PD5948	HIST1H2BK	cna	gain	BASIS
PD6731	HIST1H2BK	cna	hetloss	BASIS
PD7067	HIST1H2BK	cna	amp	BASIS
PD7215	HIST1H2BK	cna	gain	BASIS
PD8621	HIST1H2BK	cna	amp	BASIS
PD9004	HIST1H2BK	cna	gain	BASIS
PD9585	HIST1H2BK	cna	gain	BASIS
PD9702	HIST1H2BK	cna	gain	BASIS
TCGA-AN-A0XU	HIST1H4I	cna	gain	TCGA
TCGA-AO-A0JL	HIST1H4I	cna	gain	TCGA
TCGA-BH-A0AW	HIST1H4I	cna	gain	TCGA
TCGA-BH-A0C0	HIST1H4I	cna	hetloss	TCGA
TCGA-BH-A18R	HIST1H4I	cna	gain	TCGA
TCGA-C8-A12L	HIST1H4I	cna	hetloss	TCGA
MB-5465	HIST1H4I	cna	gain	METABRIC
MB-6098	HIST1H4I	cna	hetloss	METABRIC
MB-7032	HIST1H4I	cna	gain	METABRIC
PD11327	HIST1H4I	cna	hetloss	BASIS
PD13296	HIST1H4I	cna	gain	BASIS
PD13299	HIST1H4I	cna	gain	BASIS
PD23562	HIST1H4I	cna	gain	BASIS
PD24186	HIST1H4I	cna	gain	BASIS
PD24206	HIST1H4I	cna	gain	BASIS
PD3905	HIST1H4I	cna	gain	BASIS
PD4006	HIST1H4I	cna	gain	BASIS
PD4107	HIST1H4I	cna	gain	BASIS
PD4826	HIST1H4I	cna	gain	BASIS
PD5935	HIST1H4I	cna	gain	BASIS
PD5945	HIST1H4I	cna	amp	BASIS
PD5948	HIST1H4I	cna	gain	BASIS
PD6731	HIST1H4I	cna	hetloss	BASIS
PD7067	HIST1H4I	cna	amp	BASIS
PD7215	HIST1H4I	cna	gain	BASIS
PD8621	HIST1H4I	cna	amp	BASIS
PD9004	HIST1H4I	cna	gain	BASIS
PD9585	HIST1H4I	cna	gain	BASIS
PD9702	HIST1H4I	cna	gain	BASIS
TCGA-A2-A25B	JAZF1	cna	gain	TCGA
TCGA-BH-A0AW	JAZF1	cna	gain	TCGA
TCGA-BH-A0C0	JAZF1	cna	hetloss	TCGA
TCGA-C8-A12L	JAZF1	cna	gain	TCGA
TCGA-D8-A27M	JAZF1	cna	hetloss	TCGA
TCGA-LL-A5YP	JAZF1	cna	hetloss	TCGA
MB-0346	JAZF1	cna	gain	METABRIC
MB-5465	JAZF1	cna	gain	METABRIC
MB-6098	JAZF1	cna	hetloss	METABRIC

MB-0420	JAZF1	cna	hetloss	METABRIC
PD10014	JAZF1	cna	hetloss	BASIS
PD11327	JAZF1	cna	gain	BASIS
PD13296	JAZF1	cna	gain	BASIS
PD13297	JAZF1	cna	hetloss	BASIS
PD23578	JAZF1	cna	hetloss	BASIS
PD24186	JAZF1	cna	gain	BASIS
PD24206	JAZF1	cna	gain	BASIS
PD24337	JAZF1	cna	hetloss	BASIS
PD3905	JAZF1	cna	gain	BASIS
PD4006	JAZF1	cna	gain	BASIS
PD4107	JAZF1	cna	amp	BASIS
PD4826	JAZF1	cna	gain	BASIS
PD5945	JAZF1	cna	amp	BASIS
PD6406	JAZF1	cna	hetloss	BASIS
PD6413	JAZF1	cna	gain	BASIS
PD7215	JAZF1	cna	gain	BASIS
PD8621	JAZF1	cna	gain	BASIS
PD9585	JAZF1	cna	gain	BASIS
PD9702	JAZF1	cna	gain	BASIS
TCGA-A2-A25B	MALAT1	cna	hetloss	TCGA
TCGA-AN-A0XU	MALAT1	cna	hetloss	TCGA
TCGA-AO-A0JL	MALAT1	cna	hetloss	TCGA
TCGA-BH-A18R	MALAT1	cna	homdel	TCGA
TCGA-LL-A5YP	MALAT1	cna	hetloss	TCGA
MB-0346	MALAT1	cna	gain	METABRIC
MB-6060	MALAT1	cna	gain	METABRIC
MB-6271	MALAT1	cna	hetloss	METABRIC
PD11327	MALAT1	cna	hetloss	BASIS
PD13296	MALAT1	cna	hetloss	BASIS
PD13771	MALAT1	cna	hetloss	BASIS
PD22355	MALAT1	cna	gain	BASIS
PD23574	MALAT1	cna	gain	BASIS
PD24186	MALAT1	cna	gain	BASIS
PD3905	MALAT1	cna	gain	BASIS
PD4006	MALAT1	cna	amp	BASIS
PD4107	MALAT1	cna	gain	BASIS
PD4826	MALAT1	cna	gain	BASIS
PD5930	MALAT1	cna	gain	BASIS
PD5935	MALAT1	cna	gain	BASIS
PD5948	MALAT1	cna	gain	BASIS
PD6406	MALAT1	cna	amp	BASIS
PD6413	MALAT1	cna	gain	BASIS
PD7067	MALAT1	cna	gain	BASIS
PD7215	MALAT1	cna	gain	BASIS
PD8621	MALAT1	cna	gain	BASIS
PD8980	MALAT1	cna	gain	BASIS

PD9004	MALAT1	cna	gain	BASIS
PD9702	MALAT1	cna	gain	BASIS
TCGA-A2-A25B	MAPK1	cna	hetloss	TCGA
TCGA-BH-A0C0	MAPK1	cna	gain	TCGA
TCGA-BH-A1FU	MAPK1	cna	gain	TCGA
TCGA-C8-A12L	MAPK1	cna	hetloss	TCGA
TCGA-E9-A1NC	MAPK1	cna	gain	TCGA
TCGA-LL-A5YP	MAPK1	cna	gain	TCGA
MB-0346	MAPK1	cna	gain	METABRIC
MB-6098	MAPK1	cna	hetloss	METABRIC
PD11327	MAPK1	cna	gain	BASIS
PD11742	MAPK1	cna	hetloss	BASIS
PD13296	MAPK1	cna	gain	BASIS
PD14442	MAPK1	cna	hetloss	BASIS
PD22355	MAPK1	cna	hetloss	BASIS
PD23562	MAPK1	cna	gain	BASIS
PD23574	MAPK1	cna	hetloss	BASIS
PD23578	MAPK1	cna	hetloss	BASIS
PD3905	MAPK1	cna	gain	BASIS
PD4006	MAPK1	cna	gain	BASIS
PD4107	MAPK1	cna	gain	BASIS
PD4826	MAPK1	cna	gain	BASIS
PD4967	MAPK1	cna	hetloss	BASIS
PD5930	MAPK1	cna	gain	BASIS
PD5935	MAPK1	cna	gain	BASIS
PD5945	MAPK1	cna	amp	BASIS
PD5948	MAPK1	cna	gain	BASIS
PD7067	MAPK1	cna	gain	BASIS
PD8621	MAPK1	cna	gain	BASIS
PD9004	MAPK1	cna	gain	BASIS
PD9702	MAPK1	cna	gain	BASIS
TCGA-AN-A0XU	MYOD1	cna	hetloss	TCGA
TCGA-AO-A0JL	MYOD1	cna	hetloss	TCGA
TCGA-BH-A0C0	MYOD1	cna	hetloss	TCGA
TCGA-C8-A12L	MYOD1	cna	hetloss	TCGA
TCGA-E2-A1L7	MYOD1	cna	gain	TCGA
TCGA-E9-A1NC	MYOD1	cna	hetloss	TCGA
TCGA-LL-A5YP	MYOD1	cna	gain	TCGA
MB-0346	MYOD1	cna	hetloss	METABRIC
MB-6271	MYOD1	cna	gain	METABRIC
MB-7048	MYOD1	cna	hetloss	METABRIC
MB-0420	MYOD1	cna	gain	METABRIC
PD10014	MYOD1	cna	hetloss	BASIS
PD11327	MYOD1	cna	hetloss	BASIS
PD13296	MYOD1	cna	hetloss	BASIS
PD23574	MYOD1	cna	gain	BASIS
PD24186	MYOD1	cna	gain	BASIS

PD24206	MYOD1	cna	amp	BASIS
PD3905	MYOD1	cna	gain	BASIS
PD4107	MYOD1	cna	gain	BASIS
PD4826	MYOD1	cna	gain	BASIS
PD5930	MYOD1	cna	gain	BASIS
PD5935	MYOD1	cna	gain	BASIS
PD5945	MYOD1	cna	gain	BASIS
PD5948	MYOD1	cna	gain	BASIS
PD6413	MYOD1	cna	hetloss	BASIS
PD6731	MYOD1	cna	hetloss	BASIS
PD7215	MYOD1	cna	gain	BASIS
PD8980	MYOD1	cna	hetloss	BASIS
PD9585	MYOD1	cna	hetloss	BASIS
MB-2827	NKX2-1	cna	hetloss	METABRIC
MB-5070	NKX2-1	cna	hetloss	METABRIC
MB-5465	NKX2-1	cna	hetloss	METABRIC
MB-6060	NKX2-1	cna	gain	METABRIC
PD10014	NKX2-1	cna	hetloss	BASIS
PD13297	NKX2-1	cna	hetloss	BASIS
PD22355	NKX2-1	cna	hetloss	BASIS
PD23578	NKX2-1	cna	hetloss	BASIS
PD24186	NKX2-1	cna	gain	BASIS
PD24202	NKX2-1	cna	hetloss	BASIS
PD24206	NKX2-1	cna	gain	BASIS
PD24337	NKX2-1	cna	hetloss	BASIS
PD3890	NKX2-1	cna	hetloss	BASIS
PD3905	NKX2-1	cna	gain	BASIS
PD4005	NKX2-1	cna	hetloss	BASIS
PD4006	NKX2-1	cna	hetloss	BASIS
PD4107	NKX2-1	cna	hetloss	BASIS
PD4826	NKX2-1	cna	gain	BASIS
PD5930	NKX2-1	cna	hetloss	BASIS
PD5945	NKX2-1	cna	gain	BASIS
PD5948	NKX2-1	cna	gain	BASIS
PD6406	NKX2-1	cna	hetloss	BASIS
PD7067	NKX2-1	cna	gain	BASIS
PD7215	NKX2-1	cna	gain	BASIS
PD8621	NKX2-1	cna	gain	BASIS
PD8980	NKX2-1	cna	gain	BASIS
PD9004	NKX2-1	cna	gain	BASIS
PD9585	NKX2-1	cna	gain	BASIS
PD9702	NKX2-1	cna	gain	BASIS
TCGA-AN-A0XU	NRAS	cna	hetloss	TCGA
TCGA-BH-A0AW	NRAS	cna	gain	TCGA
TCGA-BH-A0C0	NRAS	cna	hetloss	TCGA
TCGA-BH-A1FU	NRAS	cna	gain	TCGA
TCGA-E2-A1L7	NRAS	cna	gain	TCGA

TCGA-LL-A5YP	NRAS	cna	gain	TCGA
MB-0346	NRAS	cna	hetloss	METABRIC
MB-2827	NRAS	cna	hetloss	METABRIC
MB-7032	NRAS	cna	gain	METABRIC
MB-7038	NRAS	cna	gain	METABRIC
PD11327	NRAS	cna	gain	BASIS
PD11742	NRAS	cna	hetloss	BASIS
PD13296	NRAS	cna	hetloss	BASIS
PD13299	NRAS	cna	gain	BASIS
PD22355	NRAS	cna	hetloss	BASIS
PD23574	NRAS	cna	gain	BASIS
PD24186	NRAS	cna	gain	BASIS
PD24206	NRAS	cna	gain	BASIS
PD3905	NRAS	cna	gain	BASIS
PD4006	NRAS	cna	gain	BASIS
PD4107	NRAS	cna	gain	BASIS
PD5930	NRAS	cna	gain	BASIS
PD5945	NRAS	cna	gain	BASIS
PD5948	NRAS	cna	gain	BASIS
PD7067	NRAS	cna	amp	BASIS
PD7215	NRAS	cna	gain	BASIS
PD8621	NRAS	cna	gain	BASIS
PD9585	NRAS	cna	hetloss	BASIS
PD9702	NRAS	cna	gain	BASIS
TCGA-A2-A25B	STAT6	cna	gain	TCGA
TCGA-AO-A0JL	STAT6	cna	hetloss	TCGA
TCGA-BH-A18R	STAT6	cna	gain	TCGA
TCGA-BH-A1FU	STAT6	cna	hetloss	TCGA
TCGA-C8-A12L	STAT6	cna	gain	TCGA
TCGA-E2-A1L7	STAT6	cna	hetloss	TCGA
TCGA-LL-A5YP	STAT6	cna	hetloss	TCGA
MB-0346	STAT6	cna	gain	METABRIC
MB-2827	STAT6	cna	hetloss	METABRIC
MB-6098	STAT6	cna	hetloss	METABRIC
MB-7038	STAT6	cna	hetloss	METABRIC
MB-0420	STAT6	cna	hetloss	METABRIC
PD11327	STAT6	cna	homdel	BASIS
PD13296	STAT6	cna	hetloss	BASIS
PD13297	STAT6	cna	hetloss	BASIS
PD24186	STAT6	cna	gain	BASIS
PD24202	STAT6	cna	hetloss	BASIS
PD24206	STAT6	cna	gain	BASIS
PD24337	STAT6	cna	hetloss	BASIS
PD3890	STAT6	cna	hetloss	BASIS
PD3905	STAT6	cna	gain	BASIS
PD4826	STAT6	cna	gain	BASIS
PD5945	STAT6	cna	gain	BASIS

PD6413	STAT6	cna	hetloss	BASIS
PD6731	STAT6	cna	hetloss	BASIS
PD7067	STAT6	cna	gain	BASIS
PD8621	STAT6	cna	hetloss	BASIS
PD9585	STAT6	cna	hetloss	BASIS
PD9702	STAT6	cna	gain	BASIS
TCGA-AN-A0XU	TRIM27	cna	gain	TCGA
TCGA-AO-A0JL	TRIM27	cna	gain	TCGA
TCGA-BH-A0AW	TRIM27	cna	gain	TCGA
TCGA-BH-A0C0	TRIM27	cna	hetloss	TCGA
TCGA-BH-A18R	TRIM27	cna	gain	TCGA
TCGA-C8-A12L	TRIM27	cna	hetloss	TCGA
MB-5465	TRIM27	cna	gain	METABRIC
MB-6098	TRIM27	cna	hetloss	METABRIC
MB-7032	TRIM27	cna	gain	METABRIC
PD11327	TRIM27	cna	hetloss	BASIS
PD13296	TRIM27	cna	gain	BASIS
PD13299	TRIM27	cna	gain	BASIS
PD23562	TRIM27	cna	gain	BASIS
PD24186	TRIM27	cna	gain	BASIS
PD24206	TRIM27	cna	gain	BASIS
PD3905	TRIM27	cna	gain	BASIS
PD4006	TRIM27	cna	gain	BASIS
PD4107	TRIM27	cna	gain	BASIS
PD4826	TRIM27	cna	gain	BASIS
PD5935	TRIM27	cna	gain	BASIS
PD5945	TRIM27	cna	amp	BASIS
PD5948	TRIM27	cna	gain	BASIS
PD6731	TRIM27	cna	hetloss	BASIS
PD7067	TRIM27	cna	amp	BASIS
PD7215	TRIM27	cna	gain	BASIS
PD8621	TRIM27	cna	amp	BASIS
PD9004	TRIM27	cna	gain	BASIS
PD9585	TRIM27	cna	gain	BASIS
PD9702	TRIM27	cna	gain	BASIS
TCGA-AN-A0XU	TRIM33	cna	hetloss	TCGA
TCGA-BH-A0AW	TRIM33	cna	gain	TCGA
TCGA-BH-A0C0	TRIM33	cna	hetloss	TCGA
TCGA-BH-A1FU	TRIM33	cna	gain	TCGA
TCGA-E2-A1L7	TRIM33	cna	gain	TCGA
TCGA-LL-A5YP	TRIM33	cna	gain	TCGA
MB-0346	TRIM33	cna	hetloss	METABRIC
MB-2827	TRIM33	cna	hetloss	METABRIC
MB-7032	TRIM33	cna	gain	METABRIC
MB-7038	TRIM33	cna	gain	METABRIC
PD11327	TRIM33	cna	gain	BASIS
PD11742	TRIM33	cna	hetloss	BASIS

PD13296	TRIM33	cna	hetloss	BASIS
PD13299	TRIM33	cna	gain	BASIS
PD22355	TRIM33	cna	hetloss	BASIS
PD23574	TRIM33	cna	gain	BASIS
PD24186	TRIM33	cna	gain	BASIS
PD24206	TRIM33	cna	gain	BASIS
PD3905	TRIM33	cna	gain	BASIS
PD4006	TRIM33	cna	gain	BASIS
PD4107	TRIM33	cna	gain	BASIS
PD5930	TRIM33	cna	gain	BASIS
PD5945	TRIM33	cna	gain	BASIS
PD5948	TRIM33	cna	gain	BASIS
PD7067	TRIM33	cna	amp	BASIS
PD7215	TRIM33	cna	gain	BASIS
PD8621	TRIM33	cna	gain	BASIS
PD9585	TRIM33	cna	hetloss	BASIS
PD9702	TRIM33	cna	gain	BASIS
TCGA-AN-A0XU	VTCN1	cna	hetloss	TCGA
TCGA-BH-A0AW	VTCN1	cna	gain	TCGA
TCGA-BH-A0C0	VTCN1	cna	hetloss	TCGA
TCGA-BH-A1FU	VTCN1	cna	gain	TCGA
TCGA-C8-A12L	VTCN1	cna	amp	TCGA
TCGA-E2-A1L7	VTCN1	cna	gain	TCGA
TCGA-LL-A5YP	VTCN1	cna	gain	TCGA
MB-0346	VTCN1	cna	hetloss	METABRIC
MB-2827	VTCN1	cna	hetloss	METABRIC
MB-7032	VTCN1	cna	gain	METABRIC
MB-7038	VTCN1	cna	gain	METABRIC
PD11327	VTCN1	cna	gain	BASIS
PD13296	VTCN1	cna	hetloss	BASIS
PD13299	VTCN1	cna	gain	BASIS
PD22355	VTCN1	cna	hetloss	BASIS
PD23574	VTCN1	cna	gain	BASIS
PD24186	VTCN1	cna	gain	BASIS
PD24206	VTCN1	cna	gain	BASIS
PD3905	VTCN1	cna	gain	BASIS
PD4006	VTCN1	cna	gain	BASIS
PD4107	VTCN1	cna	gain	BASIS
PD5930	VTCN1	cna	gain	BASIS
PD5945	VTCN1	cna	gain	BASIS
PD5948	VTCN1	cna	gain	BASIS
PD7067	VTCN1	cna	amp	BASIS
PD7215	VTCN1	cna	gain	BASIS
PD8621	VTCN1	cna	gain	BASIS
PD9585	VTCN1	cna	hetloss	BASIS
PD9702	VTCN1	cna	gain	BASIS
TCGA-A2-A25B	ARID5B	cna	hetloss	TCGA

TCGA-AN-A0XU	ARID5B	cna	hetloss	TCGA
TCGA-AO-A0JL	ARID5B	cna	gain	TCGA
TCGA-BH-A0C0	ARID5B	cna	gain	TCGA
TCGA-C8-A12L	ARID5B	cna	gain	TCGA
TCGA-LL-A5YP	ARID5B	cna	hetloss	TCGA
MB-0346	ARID5B	cna	gain	METABRIC
MB-5070	ARID5B	cna	gain	METABRIC
MB-6098	ARID5B	cna	hetloss	METABRIC
MB-7038	ARID5B	cna	hetloss	METABRIC
PD11327	ARID5B	cna	amp	BASIS
PD11742	ARID5B	cna	hetloss	BASIS
PD13299	ARID5B	cna	gain	BASIS
PD23574	ARID5B	cna	gain	BASIS
PD24186	ARID5B	cna	gain	BASIS
PD24206	ARID5B	cna	gain	BASIS
PD4005	ARID5B	cna	gain	BASIS
PD4006	ARID5B	cna	gain	BASIS
PD4107	ARID5B	cna	gain	BASIS
PD4826	ARID5B	cna	gain	BASIS
PD5930	ARID5B	cna	gain	BASIS
PD5945	ARID5B	cna	amp	BASIS
PD7067	ARID5B	cna	amp	BASIS
PD7215	ARID5B	cna	gain	BASIS
PD8621	ARID5B	cna	gain	BASIS
PD8980	ARID5B	cna	hetloss	BASIS
PD9585	ARID5B	cna	hetloss	BASIS
PD9702	ARID5B	cna	gain	BASIS
TCGA-AN-A0XU	BCL2L11	cna	hetloss	TCGA
TCGA-AO-A0JL	BCL2L11	cna	gain	TCGA
TCGA-D8-A27M	BCL2L11	cna	gain	TCGA
TCGA-LL-A5YP	BCL2L11	cna	gain	TCGA
MB-5465	BCL2L11	cna	hetloss	METABRIC
MB-6060	BCL2L11	cna	gain	METABRIC
MB-6271	BCL2L11	cna	hetloss	METABRIC
PD11327	BCL2L11	cna	gain	BASIS
PD13297	BCL2L11	cna	homdel	BASIS
PD13299	BCL2L11	cna	gain	BASIS
PD23578	BCL2L11	cna	hetloss	BASIS
PD24186	BCL2L11	cna	gain	BASIS
PD24202	BCL2L11	cna	hetloss	BASIS
PD24206	BCL2L11	cna	gain	BASIS
PD3905	BCL2L11	cna	gain	BASIS
PD4005	BCL2L11	cna	hetloss	BASIS
PD4107	BCL2L11	cna	gain	BASIS
PD4967	BCL2L11	cna	hetloss	BASIS
PD5945	BCL2L11	cna	gain	BASIS
PD5948	BCL2L11	cna	gain	BASIS

PD6413	BCL2L11	cna	hetloss	BASIS
PD7067	BCL2L11	cna	gain	BASIS
PD7215	BCL2L11	cna	gain	BASIS
PD8621	BCL2L11	cna	gain	BASIS
PD8980	BCL2L11	cna	hetloss	BASIS
PD9004	BCL2L11	cna	gain	BASIS
PD9585	BCL2L11	cna	hetloss	BASIS
PD9702	BCL2L11	cna	gain	BASIS
TCGA-A2-A25B	CAD	cna	hetloss	TCGA
TCGA-AN-A0XU	CAD	cna	gain	TCGA
TCGA-BH-A0AW	CAD	cna	gain	TCGA
TCGA-BH-A0C0	CAD	cna	gain	TCGA
TCGA-C8-A12L	CAD	cna	gain	TCGA
TCGA-D8-A27M	CAD	cna	gain	TCGA
TCGA-LL-A5YP	CAD	cna	gain	TCGA
MB-0346	CAD	cna	gain	METABRIC
MB-5465	CAD	cna	amp	METABRIC
MB-6271	CAD	cna	hetloss	METABRIC
MB-7038	CAD	cna	gain	METABRIC
PD10014	CAD	cna	gain	BASIS
PD11327	CAD	cna	gain	BASIS
PD13299	CAD	cna	gain	BASIS
PD23562	CAD	cna	gain	BASIS
PD24186	CAD	cna	gain	BASIS
PD3890	CAD	cna	hetloss	BASIS
PD3905	CAD	cna	gain	BASIS
PD4006	CAD	cna	gain	BASIS
PD4826	CAD	cna	hetloss	BASIS
PD5930	CAD	cna	gain	BASIS
PD5935	CAD	cna	gain	BASIS
PD5945	CAD	cna	gain	BASIS
PD5948	CAD	cna	gain	BASIS
PD6406	CAD	cna	hetloss	BASIS
PD7067	CAD	cna	gain	BASIS
PD9004	CAD	cna	gain	BASIS
PD9702	CAD	cna	gain	BASIS
TCGA-A2-A25B	CCDC6	cna	hetloss	TCGA
TCGA-AN-A0XU	CCDC6	cna	hetloss	TCGA
TCGA-AO-A0JL	CCDC6	cna	hetloss	TCGA
TCGA-BH-A0C0	CCDC6	cna	gain	TCGA
TCGA-C8-A12L	CCDC6	cna	gain	TCGA
TCGA-LL-A5YP	CCDC6	cna	hetloss	TCGA
MB-0346	CCDC6	cna	gain	METABRIC
MB-5070	CCDC6	cna	gain	METABRIC
MB-6098	CCDC6	cna	hetloss	METABRIC
MB-7038	CCDC6	cna	hetloss	METABRIC
PD11327	CCDC6	cna	amp	BASIS

PD11742	CCDC6	cna	hetloss	BASIS
PD13296	CCDC6	cna	gain	BASIS
PD13299	CCDC6	cna	gain	BASIS
PD24186	CCDC6	cna	gain	BASIS
PD24206	CCDC6	cna	gain	BASIS
PD4005	CCDC6	cna	gain	BASIS
PD4006	CCDC6	cna	gain	BASIS
PD4107	CCDC6	cna	gain	BASIS
PD4826	CCDC6	cna	gain	BASIS
PD5930	CCDC6	cna	gain	BASIS
PD5945	CCDC6	cna	gain	BASIS
PD7067	CCDC6	cna	amp	BASIS
PD7215	CCDC6	cna	gain	BASIS
PD8621	CCDC6	cna	gain	BASIS
PD8980	CCDC6	cna	hetloss	BASIS
PD9585	CCDC6	cna	hetloss	BASIS
PD9702	CCDC6	cna	gain	BASIS
TCGA-A2-A25B	CLTCL1	cna	hetloss	TCGA
TCGA-BH-A0C0	CLTCL1	cna	gain	TCGA
TCGA-BH-A1FU	CLTCL1	cna	gain	TCGA
TCGA-C8-A12L	CLTCL1	cna	hetloss	TCGA
TCGA-E9-A1NC	CLTCL1	cna	gain	TCGA
TCGA-LL-A5YP	CLTCL1	cna	gain	TCGA
MB-0346	CLTCL1	cna	gain	METABRIC
MB-6098	CLTCL1	cna	hetloss	METABRIC
PD11327	CLTCL1	cna	gain	BASIS
PD11742	CLTCL1	cna	hetloss	BASIS
PD13296	CLTCL1	cna	gain	BASIS
PD14442	CLTCL1	cna	hetloss	BASIS
PD23578	CLTCL1	cna	hetloss	BASIS
PD3890	CLTCL1	cna	hetloss	BASIS
PD3905	CLTCL1	cna	gain	BASIS
PD4005	CLTCL1	cna	gain	BASIS
PD4006	CLTCL1	cna	gain	BASIS
PD4826	CLTCL1	cna	gain	BASIS
PD4967	CLTCL1	cna	hetloss	BASIS
PD5930	CLTCL1	cna	gain	BASIS
PD5935	CLTCL1	cna	gain	BASIS
PD5945	CLTCL1	cna	amp	BASIS
PD5948	CLTCL1	cna	gain	BASIS
PD6406	CLTCL1	cna	hetloss	BASIS
PD7067	CLTCL1	cna	gain	BASIS
PD8621	CLTCL1	cna	gain	BASIS
PD9004	CLTCL1	cna	gain	BASIS
PD9702	CLTCL1	cna	gain	BASIS
TCGA-AN-A0XU	DUSP2	cna	gain	TCGA
TCGA-AO-A0JL	DUSP2	cna	gain	TCGA

TCGA-BH-A0AW	DUSP2	cna	amp	TCGA
TCGA-BH-A0C0	DUSP2	cna	gain	TCGA
TCGA-D8-A27M	DUSP2	cna	gain	TCGA
TCGA-LL-A5YP	DUSP2	cna	gain	TCGA
MB-6060	DUSP2	cna	gain	METABRIC
MB-6098	DUSP2	cna	gain	METABRIC
MB-6271	DUSP2	cna	hetloss	METABRIC
PD13297	DUSP2	cna	gain	BASIS
PD13299	DUSP2	cna	gain	BASIS
PD23562	DUSP2	cna	amp	BASIS
PD23578	DUSP2	cna	gain	BASIS
PD24186	DUSP2	cna	gain	BASIS
PD24206	DUSP2	cna	gain	BASIS
PD3905	DUSP2	cna	gain	BASIS
PD4005	DUSP2	cna	hetloss	BASIS
PD4006	DUSP2	cna	gain	BASIS
PD4107	DUSP2	cna	gain	BASIS
PD4967	DUSP2	cna	hetloss	BASIS
PD5930	DUSP2	cna	gain	BASIS
PD5945	DUSP2	cna	amp	BASIS
PD5948	DUSP2	cna	gain	BASIS
PD7067	DUSP2	cna	amp	BASIS
PD7215	DUSP2	cna	gain	BASIS
PD8621	DUSP2	cna	gain	BASIS
PD9004	DUSP2	cna	gain	BASIS
PD9702	DUSP2	cna	gain	BASIS
TCGA-A2-A25B	ELN	cna	hetloss	TCGA
TCGA-AN-A0XU	ELN	cna	hetloss	TCGA
TCGA-AO-A0JL	ELN	cna	gain	TCGA
TCGA-BH-A0AW	ELN	cna	gain	TCGA
TCGA-C8-A12L	ELN	cna	hetloss	TCGA
TCGA-D8-A27M	ELN	cna	gain	TCGA
MB-0346	ELN	cna	gain	METABRIC
MB-2827	ELN	cna	hetloss	METABRIC
MB-7048	ELN	cna	hetloss	METABRIC
PD11327	ELN	cna	hetloss	BASIS
PD13296	ELN	cna	hetloss	BASIS
PD23562	ELN	cna	gain	BASIS
PD23574	ELN	cna	gain	BASIS
PD24186	ELN	cna	gain	BASIS
PD24206	ELN	cna	homdel	BASIS
PD3890	ELN	cna	hetloss	BASIS
PD3905	ELN	cna	gain	BASIS
PD4006	ELN	cna	gain	BASIS
PD4107	ELN	cna	gain	BASIS
PD4826	ELN	cna	gain	BASIS
PD4967	ELN	cna	hetloss	BASIS

PD5930	ELN	cna	gain	BASIS
PD5935	ELN	cna	gain	BASIS
PD5945	ELN	cna	amp	BASIS
PD5948	ELN	cna	gain	BASIS
PD7215	ELN	cna	gain	BASIS
PD9004	ELN	cna	gain	BASIS
PD9702	ELN	cna	gain	BASIS
TCGA-AN-A0XU	FUS	cna	gain	TCGA
TCGA-AO-A0JL	FUS	cna	gain	TCGA
TCGA-BH-A0AW	FUS	cna	gain	TCGA
TCGA-BH-A0C0	FUS	cna	gain	TCGA
TCGA-C8-A12L	FUS	cna	gain	TCGA
TCGA-E2-A1L7	FUS	cna	hetloss	TCGA
TCGA-LL-A5YP	FUS	cna	gain	TCGA
MB-0346	FUS	cna	gain	METABRIC
MB-6271	FUS	cna	gain	METABRIC
MB-7032	FUS	cna	gain	METABRIC
MB-7048	FUS	cna	hetloss	METABRIC
MB-0420	FUS	cna	hetloss	METABRIC
PD11327	FUS	cna	hetloss	BASIS
PD13299	FUS	cna	gain	BASIS
PD24186	FUS	cna	gain	BASIS
PD24202	FUS	cna	hetloss	BASIS
PD3905	FUS	cna	gain	BASIS
PD4006	FUS	cna	gain	BASIS
PD4107	FUS	cna	gain	BASIS
PD4826	FUS	cna	gain	BASIS
PD5930	FUS	cna	gain	BASIS
PD5935	FUS	cna	gain	BASIS
PD5945	FUS	cna	amp	BASIS
PD5948	FUS	cna	gain	BASIS
PD7067	FUS	cna	gain	BASIS
PD7215	FUS	cna	amp	BASIS
PD8980	FUS	cna	gain	BASIS
PD9004	FUS	cna	gain	BASIS
TCGA-A2-A25B	HIP1	cna	hetloss	TCGA
TCGA-AN-A0XU	HIP1	cna	hetloss	TCGA
TCGA-AO-A0JL	HIP1	cna	gain	TCGA
TCGA-BH-A0AW	HIP1	cna	gain	TCGA
TCGA-C8-A12L	HIP1	cna	hetloss	TCGA
TCGA-D8-A27M	HIP1	cna	gain	TCGA
MB-0346	HIP1	cna	hetloss	METABRIC
MB-2827	HIP1	cna	hetloss	METABRIC
MB-7048	HIP1	cna	hetloss	METABRIC
PD11327	HIP1	cna	hetloss	BASIS
PD13296	HIP1	cna	hetloss	BASIS
PD23562	HIP1	cna	gain	BASIS

PD23574	HIP1	cna	gain	BASIS
PD24186	HIP1	cna	amp	BASIS
PD24206	HIP1	cna	hetloss	BASIS
PD3890	HIP1	cna	hetloss	BASIS
PD3905	HIP1	cna	gain	BASIS
PD4006	HIP1	cna	gain	BASIS
PD4107	HIP1	cna	gain	BASIS
PD4826	HIP1	cna	gain	BASIS
PD4967	HIP1	cna	hetloss	BASIS
PD5930	HIP1	cna	gain	BASIS
PD5935	HIP1	cna	gain	BASIS
PD5945	HIP1	cna	amp	BASIS
PD5948	HIP1	cna	gain	BASIS
PD7215	HIP1	cna	gain	BASIS
PD9004	HIP1	cna	gain	BASIS
PD9702	HIP1	cna	gain	BASIS
TCGA-A2-A25B	HOXA11	cna	gain	TCGA
TCGA-BH-A0AW	HOXA11	cna	gain	TCGA
TCGA-BH-A0C0	HOXA11	cna	hetloss	TCGA
TCGA-C8-A12L	HOXA11	cna	gain	TCGA
TCGA-D8-A27M	HOXA11	cna	hetloss	TCGA
TCGA-LL-A5YP	HOXA11	cna	hetloss	TCGA
MB-0346	HOXA11	cna	gain	METABRIC
MB-6098	HOXA11	cna	hetloss	METABRIC
MB-0420	HOXA11	cna	hetloss	METABRIC
PD10014	HOXA11	cna	hetloss	BASIS
PD11327	HOXA11	cna	gain	BASIS
PD13296	HOXA11	cna	gain	BASIS
PD13297	HOXA11	cna	hetloss	BASIS
PD23578	HOXA11	cna	hetloss	BASIS
PD24186	HOXA11	cna	gain	BASIS
PD24206	HOXA11	cna	gain	BASIS
PD24337	HOXA11	cna	hetloss	BASIS
PD3905	HOXA11	cna	gain	BASIS
PD4006	HOXA11	cna	gain	BASIS
PD4107	HOXA11	cna	amp	BASIS
PD4826	HOXA11	cna	gain	BASIS
PD5945	HOXA11	cna	amp	BASIS
PD6406	HOXA11	cna	hetloss	BASIS
PD6413	HOXA11	cna	gain	BASIS
PD7215	HOXA11	cna	gain	BASIS
PD8621	HOXA11	cna	gain	BASIS
PD9585	HOXA11	cna	gain	BASIS
PD9702	HOXA11	cna	gain	BASIS
TCGA-A2-A25B	HOXA13	cna	gain	TCGA
TCGA-BH-A0AW	HOXA13	cna	gain	TCGA
TCGA-BH-A0C0	HOXA13	cna	hetloss	TCGA

TCGA-C8-A12L	HOXA13	cna	gain	TCGA
TCGA-D8-A27M	HOXA13	cna	hetloss	TCGA
TCGA-LL-A5YP	HOXA13	cna	hetloss	TCGA
MB-0346	HOXA13	cna	gain	METABRIC
MB-6098	HOXA13	cna	hetloss	METABRIC
MB-0420	HOXA13	cna	hetloss	METABRIC
PD10014	HOXA13	cna	hetloss	BASIS
PD11327	HOXA13	cna	gain	BASIS
PD13296	HOXA13	cna	gain	BASIS
PD13297	HOXA13	cna	hetloss	BASIS
PD23578	HOXA13	cna	hetloss	BASIS
PD24186	HOXA13	cna	gain	BASIS
PD24206	HOXA13	cna	gain	BASIS
PD24337	HOXA13	cna	hetloss	BASIS
PD3905	HOXA13	cna	gain	BASIS
PD4006	HOXA13	cna	gain	BASIS
PD4107	HOXA13	cna	amp	BASIS
PD4826	HOXA13	cna	gain	BASIS
PD5945	HOXA13	cna	amp	BASIS
PD6406	HOXA13	cna	hetloss	BASIS
PD6413	HOXA13	cna	gain	BASIS
PD7215	HOXA13	cna	gain	BASIS
PD8621	HOXA13	cna	gain	BASIS
PD9585	HOXA13	cna	gain	BASIS
PD9702	HOXA13	cna	gain	BASIS
TCGA-A2-A25B	HOXA9	cna	gain	TCGA
TCGA-BH-A0AW	HOXA9	cna	gain	TCGA
TCGA-BH-A0C0	HOXA9	cna	hetloss	TCGA
TCGA-C8-A12L	HOXA9	cna	gain	TCGA
TCGA-D8-A27M	HOXA9	cna	hetloss	TCGA
TCGA-LL-A5YP	HOXA9	cna	hetloss	TCGA
MB-0346	HOXA9	cna	gain	METABRIC
MB-6098	HOXA9	cna	hetloss	METABRIC
MB-0420	HOXA9	cna	hetloss	METABRIC
PD10014	HOXA9	cna	hetloss	BASIS
PD11327	HOXA9	cna	gain	BASIS
PD13296	HOXA9	cna	gain	BASIS
PD13297	HOXA9	cna	hetloss	BASIS
PD23578	HOXA9	cna	hetloss	BASIS
PD24186	HOXA9	cna	gain	BASIS
PD24206	HOXA9	cna	gain	BASIS
PD24337	HOXA9	cna	hetloss	BASIS
PD3905	HOXA9	cna	gain	BASIS
PD4006	HOXA9	cna	gain	BASIS
PD4107	HOXA9	cna	amp	BASIS
PD4826	HOXA9	cna	gain	BASIS
PD5945	HOXA9	cna	amp	BASIS

PD6406	HOXA9	cna	hetloss	BASIS
PD6413	HOXA9	cna	gain	BASIS
PD7215	HOXA9	cna	gain	BASIS
PD8621	HOXA9	cna	gain	BASIS
PD9585	HOXA9	cna	gain	BASIS
PD9702	HOXA9	cna	gain	BASIS
TCGA-A2-A25B	MAGI2	cna	hetloss	TCGA
TCGA-AN-A0XU	MAGI2	cna	hetloss	TCGA
TCGA-AO-A0JL	MAGI2	cna	gain	TCGA
TCGA-BH-A0AW	MAGI2	cna	gain	TCGA
TCGA-C8-A12L	MAGI2	cna	hetloss	TCGA
TCGA-D8-A27M	MAGI2	cna	gain	TCGA
TCGA-E9-A1NC	MAGI2	cna	amp	TCGA
MB-0346	MAGI2	cna	hetloss	METABRIC
MB-2827	MAGI2	cna	hetloss	METABRIC
MB-7048	MAGI2	cna	hetloss	METABRIC
PD11327	MAGI2	cna	amp	BASIS
PD13296	MAGI2	cna	hetloss	BASIS
PD23562	MAGI2	cna	gain	BASIS
PD23574	MAGI2	cna	gain	BASIS
PD24186	MAGI2	cna	amp	BASIS
PD3890	MAGI2	cna	hetloss	BASIS
PD3905	MAGI2	cna	gain	BASIS
PD4006	MAGI2	cna	gain	BASIS
PD4107	MAGI2	cna	gain	BASIS
PD4826	MAGI2	cna	gain	BASIS
PD5930	MAGI2	cna	gain	BASIS
PD5935	MAGI2	cna	gain	BASIS
PD5945	MAGI2	cna	amp	BASIS
PD5948	MAGI2	cna	gain	BASIS
PD6406	MAGI2	cna	hetloss	BASIS
PD7215	MAGI2	cna	gain	BASIS
PD9004	MAGI2	cna	gain	BASIS
PD9702	MAGI2	cna	gain	BASIS
TCGA-A2-A25B	NOD1	cna	gain	TCGA
TCGA-AO-A0JL	NOD1	cna	gain	TCGA
TCGA-BH-A0AW	NOD1	cna	gain	TCGA
TCGA-BH-A0C0	NOD1	cna	hetloss	TCGA
TCGA-C8-A12L	NOD1	cna	gain	TCGA
TCGA-D8-A27M	NOD1	cna	hetloss	TCGA
TCGA-LL-A5YP	NOD1	cna	hetloss	TCGA
MB-0346	NOD1	cna	gain	METABRIC
MB-6098	NOD1	cna	hetloss	METABRIC
MB-0420	NOD1	cna	hetloss	METABRIC
PD10014	NOD1	cna	hetloss	BASIS
PD11327	NOD1	cna	gain	BASIS
PD13297	NOD1	cna	hetloss	BASIS

PD23578	NOD1	cna	hetloss	BASIS
PD24186	NOD1	cna	gain	BASIS
PD24206	NOD1	cna	gain	BASIS
PD24337	NOD1	cna	hetloss	BASIS
PD3905	NOD1	cna	gain	BASIS
PD4006	NOD1	cna	gain	BASIS
PD4107	NOD1	cna	amp	BASIS
PD4826	NOD1	cna	gain	BASIS
PD5945	NOD1	cna	amp	BASIS
PD6406	NOD1	cna	hetloss	BASIS
PD6413	NOD1	cna	gain	BASIS
PD7215	NOD1	cna	gain	BASIS
PD8621	NOD1	cna	gain	BASIS
PD9585	NOD1	cna	gain	BASIS
PD9702	NOD1	cna	gain	BASIS
TCGA-A2-A25B	OLIG2	cna	hetloss	TCGA
TCGA-BH-A0C0	OLIG2	cna	hetloss	TCGA
TCGA-BH-A1FU	OLIG2	cna	gain	TCGA
TCGA-D8-A27M	OLIG2	cna	hetloss	TCGA
TCGA-LL-A5YP	OLIG2	cna	gain	TCGA
MB-0346	OLIG2	cna	gain	METABRIC
MB-7048	OLIG2	cna	hetloss	METABRIC
PD11742	OLIG2	cna	hetloss	BASIS
PD13296	OLIG2	cna	gain	BASIS
PD13297	OLIG2	cna	hetloss	BASIS
PD23562	OLIG2	cna	gain	BASIS
PD23578	OLIG2	cna	gain	BASIS
PD24186	OLIG2	cna	gain	BASIS
PD24202	OLIG2	cna	gain	BASIS
PD24206	OLIG2	cna	hetloss	BASIS
PD3905	OLIG2	cna	gain	BASIS
PD4107	OLIG2	cna	gain	BASIS
PD4826	OLIG2	cna	gain	BASIS
PD5930	OLIG2	cna	gain	BASIS
PD5945	OLIG2	cna	gain	BASIS
PD5948	OLIG2	cna	gain	BASIS
PD6731	OLIG2	cna	gain	BASIS
PD7067	OLIG2	cna	gain	BASIS
PD7215	OLIG2	cna	gain	BASIS
PD8621	OLIG2	cna	gain	BASIS
PD8980	OLIG2	cna	hetloss	BASIS
PD9004	OLIG2	cna	amp	BASIS
PD9702	OLIG2	cna	gain	BASIS
TCGA-AN-A0XU	PRSS8	cna	gain	TCGA
TCGA-AO-A0JL	PRSS8	cna	gain	TCGA
TCGA-BH-A0AW	PRSS8	cna	gain	TCGA
TCGA-BH-A0C0	PRSS8	cna	gain	TCGA

TCGA-C8-A12L	PRSS8	cna	gain	TCGA
TCGA-E2-A1L7	PRSS8	cna	hetloss	TCGA
TCGA-LL-A5YP	PRSS8	cna	gain	TCGA
MB-0346	PRSS8	cna	gain	METABRIC
MB-6271	PRSS8	cna	gain	METABRIC
MB-7032	PRSS8	cna	gain	METABRIC
MB-7048	PRSS8	cna	hetloss	METABRIC
MB-0420	PRSS8	cna	hetloss	METABRIC
PD11327	PRSS8	cna	hetloss	BASIS
PD13299	PRSS8	cna	gain	BASIS
PD24186	PRSS8	cna	gain	BASIS
PD24202	PRSS8	cna	hetloss	BASIS
PD3905	PRSS8	cna	gain	BASIS
PD4006	PRSS8	cna	gain	BASIS
PD4107	PRSS8	cna	gain	BASIS
PD4826	PRSS8	cna	gain	BASIS
PD5930	PRSS8	cna	gain	BASIS
PD5935	PRSS8	cna	gain	BASIS
PD5945	PRSS8	cna	amp	BASIS
PD5948	PRSS8	cna	gain	BASIS
PD7067	PRSS8	cna	gain	BASIS
PD7215	PRSS8	cna	amp	BASIS
PD8980	PRSS8	cna	gain	BASIS
PD9004	PRSS8	cna	gain	BASIS
TCGA-A2-A25B	RPS6KA4	cna	hetloss	TCGA
TCGA-AN-A0XU	RPS6KA4	cna	hetloss	TCGA
TCGA-AO-A0JL	RPS6KA4	cna	hetloss	TCGA
TCGA-BH-A18R	RPS6KA4	cna	homdel	TCGA
TCGA-LL-A5YP	RPS6KA4	cna	hetloss	TCGA
MB-0346	RPS6KA4	cna	gain	METABRIC
MB-6060	RPS6KA4	cna	gain	METABRIC
MB-6098	RPS6KA4	cna	hetloss	METABRIC
MB-6271	RPS6KA4	cna	hetloss	METABRIC
PD11327	RPS6KA4	cna	hetloss	BASIS
PD13296	RPS6KA4	cna	hetloss	BASIS
PD13771	RPS6KA4	cna	hetloss	BASIS
PD24186	RPS6KA4	cna	gain	BASIS
PD24206	RPS6KA4	cna	gain	BASIS
PD3905	RPS6KA4	cna	gain	BASIS
PD4006	RPS6KA4	cna	gain	BASIS
PD4107	RPS6KA4	cna	gain	BASIS
PD4826	RPS6KA4	cna	gain	BASIS
PD5930	RPS6KA4	cna	gain	BASIS
PD5935	RPS6KA4	cna	gain	BASIS
PD5945	RPS6KA4	cna	amp	BASIS
PD5948	RPS6KA4	cna	gain	BASIS
PD6413	RPS6KA4	cna	gain	BASIS

PD7067	RPS6KA4	cna	gain	BASIS
PD7215	RPS6KA4	cna	gain	BASIS
PD8621	RPS6KA4	cna	gain	BASIS
PD8980	RPS6KA4	cna	hetloss	BASIS
PD9702	RPS6KA4	cna	gain	BASIS
TCGA-AN-A0XU	SRSF3	cna	hetloss	TCGA
TCGA-AO-A0JL	SRSF3	cna	gain	TCGA
TCGA-BH-A0AW	SRSF3	cna	gain	TCGA
TCGA-BH-A0C0	SRSF3	cna	hetloss	TCGA
TCGA-C8-A12L	SRSF3	cna	hetloss	TCGA
TCGA-E2-A1L7	SRSF3	cna	hetloss	TCGA
TCGA-E9-A1NC	SRSF3	cna	amp	TCGA
MB-7032	SRSF3	cna	gain	METABRIC
PD11327	SRSF3	cna	hetloss	BASIS
PD13296	SRSF3	cna	hetloss	BASIS
PD13299	SRSF3	cna	gain	BASIS
PD23562	SRSF3	cna	gain	BASIS
PD24186	SRSF3	cna	gain	BASIS
PD24206	SRSF3	cna	gain	BASIS
PD3905	SRSF3	cna	gain	BASIS
PD4006	SRSF3	cna	gain	BASIS
PD4107	SRSF3	cna	gain	BASIS
PD4826	SRSF3	cna	gain	BASIS
PD5935	SRSF3	cna	gain	BASIS
PD5945	SRSF3	cna	amp	BASIS
PD5948	SRSF3	cna	gain	BASIS
PD6731	SRSF3	cna	hetloss	BASIS
PD7067	SRSF3	cna	amp	BASIS
PD7215	SRSF3	cna	gain	BASIS
PD8621	SRSF3	cna	amp	BASIS
PD9004	SRSF3	cna	gain	BASIS
PD9585	SRSF3	cna	gain	BASIS
PD9702	SRSF3	cna	gain	BASIS
TCGA-AN-A0XU	TMEM127	cna	gain	TCGA
TCGA-AO-A0JL	TMEM127	cna	gain	TCGA
TCGA-BH-A0AW	TMEM127	cna	amp	TCGA
TCGA-BH-A0C0	TMEM127	cna	gain	TCGA
TCGA-D8-A27M	TMEM127	cna	gain	TCGA
TCGA-LL-A5YP	TMEM127	cna	gain	TCGA
MB-6060	TMEM127	cna	gain	METABRIC
MB-6098	TMEM127	cna	gain	METABRIC
MB-6271	TMEM127	cna	hetloss	METABRIC
PD13297	TMEM127	cna	gain	BASIS
PD13299	TMEM127	cna	gain	BASIS
PD23562	TMEM127	cna	amp	BASIS
PD23578	TMEM127	cna	gain	BASIS
PD24186	TMEM127	cna	gain	BASIS

PD24206	TMEM127	cna	gain	BASIS
PD3905	TMEM127	cna	gain	BASIS
PD4005	TMEM127	cna	hetloss	BASIS
PD4006	TMEM127	cna	gain	BASIS
PD4107	TMEM127	cna	gain	BASIS
PD4967	TMEM127	cna	hetloss	BASIS
PD5930	TMEM127	cna	gain	BASIS
PD5945	TMEM127	cna	amp	BASIS
PD5948	TMEM127	cna	gain	BASIS
PD7067	TMEM127	cna	amp	BASIS
PD7215	TMEM127	cna	gain	BASIS
PD8621	TMEM127	cna	gain	BASIS
PD9004	TMEM127	cna	gain	BASIS
PD9702	TMEM127	cna	gain	BASIS
TCGA-A2-A25B	SEPT5	cna	hetloss	TCGA
TCGA-BH-A0C0	SEPT5	cna	gain	TCGA
TCGA-BH-A1FU	SEPT5	cna	gain	TCGA
TCGA-C8-A12L	SEPT5	cna	hetloss	TCGA
TCGA-E9-A1NC	SEPT5	cna	gain	TCGA
TCGA-LL-A5YP	SEPT5	cna	gain	TCGA
PD11327	SEPT5	cna	gain	BASIS
PD11742	SEPT5	cna	hetloss	BASIS
PD13296	SEPT5	cna	gain	BASIS
PD14442	SEPT5	cna	hetloss	BASIS
PD23578	SEPT5	cna	hetloss	BASIS
PD3890	SEPT5	cna	hetloss	BASIS
PD3905	SEPT5	cna	gain	BASIS
PD4005	SEPT5	cna	gain	BASIS
PD4006	SEPT5	cna	gain	BASIS
PD4107	SEPT5	cna	gain	BASIS
PD4826	SEPT5	cna	gain	BASIS
PD4967	SEPT5	cna	hetloss	BASIS
PD5930	SEPT5	cna	gain	BASIS
PD5935	SEPT5	cna	gain	BASIS
PD5945	SEPT5	cna	amp	BASIS
PD5948	SEPT5	cna	gain	BASIS
PD6406	SEPT5	cna	hetloss	BASIS
PD7067	SEPT5	cna	gain	BASIS
PD8621	SEPT5	cna	gain	BASIS
PD9004	SEPT5	cna	gain	BASIS
PD9702	SEPT5	cna	gain	BASIS
TCGA-A2-A25B	ABCB1	cna	hetloss	TCGA
TCGA-AN-A0XU	ABCB1	cna	hetloss	TCGA
TCGA-AO-A0JL	ABCB1	cna	gain	TCGA
TCGA-BH-A0AW	ABCB1	cna	gain	TCGA
TCGA-C8-A12L	ABCB1	cna	hetloss	TCGA
TCGA-D8-A27M	ABCB1	cna	gain	TCGA

TCGA-E2-A1L7	ABCB1	cna	hetloss	TCGA
TCGA-E9-A1NC	ABCB1	cna	gain	TCGA
MB-0346	ABCB1	cna	hetloss	METABRIC
PD11327	ABCB1	cna	gain	BASIS
PD13296	ABCB1	cna	hetloss	BASIS
PD23574	ABCB1	cna	gain	BASIS
PD24186	ABCB1	cna	amp	BASIS
PD3890	ABCB1	cna	hetloss	BASIS
PD3905	ABCB1	cna	gain	BASIS
PD4006	ABCB1	cna	gain	BASIS
PD4107	ABCB1	cna	gain	BASIS
PD4826	ABCB1	cna	gain	BASIS
PD5930	ABCB1	cna	gain	BASIS
PD5935	ABCB1	cna	gain	BASIS
PD5945	ABCB1	cna	amp	BASIS
PD5948	ABCB1	cna	gain	BASIS
PD7067	ABCB1	cna	gain	BASIS
PD7215	ABCB1	cna	gain	BASIS
PD8621	ABCB1	cna	gain	BASIS
PD9004	ABCB1	cna	gain	BASIS
PD9702	ABCB1	cna	gain	BASIS
TCGA-A2-A25B	CD36	cna	hetloss	TCGA
TCGA-AN-A0XU	CD36	cna	hetloss	TCGA
TCGA-AO-A0JL	CD36	cna	gain	TCGA
TCGA-BH-A0AW	CD36	cna	gain	TCGA
TCGA-C8-A12L	CD36	cna	hetloss	TCGA
TCGA-D8-A27M	CD36	cna	gain	TCGA
TCGA-E9-A1NC	CD36	cna	gain	TCGA
MB-0346	CD36	cna	hetloss	METABRIC
MB-5465	CD36	cna	gain	METABRIC
PD11327	CD36	cna	amp	BASIS
PD13296	CD36	cna	hetloss	BASIS
PD23562	CD36	cna	gain	BASIS
PD23574	CD36	cna	gain	BASIS
PD24186	CD36	cna	amp	BASIS
PD3890	CD36	cna	hetloss	BASIS
PD3905	CD36	cna	gain	BASIS
PD4006	CD36	cna	gain	BASIS
PD4107	CD36	cna	gain	BASIS
PD4826	CD36	cna	gain	BASIS
PD5930	CD36	cna	gain	BASIS
PD5935	CD36	cna	gain	BASIS
PD5945	CD36	cna	amp	BASIS
PD5948	CD36	cna	gain	BASIS
PD6406	CD36	cna	hetloss	BASIS
PD7215	CD36	cna	gain	BASIS
PD9004	CD36	cna	gain	BASIS

PD9702	CD36	cna	gain	BASIS
TCGA-A2-A25B	CLP1	cna	gain	TCGA
TCGA-AN-A0XU	CLP1	cna	hetloss	TCGA
TCGA-AO-A0JL	CLP1	cna	hetloss	TCGA
TCGA-LL-A5YP	CLP1	cna	gain	TCGA
MB-0346	CLP1	cna	gain	METABRIC
MB-6060	CLP1	cna	gain	METABRIC
MB-6098	CLP1	cna	hetloss	METABRIC
MB-6271	CLP1	cna	gain	METABRIC
MB-0420	CLP1	cna	gain	METABRIC
PD11327	CLP1	cna	homdel	BASIS
PD13296	CLP1	cna	gain	BASIS
PD13299	CLP1	cna	gain	BASIS
PD13771	CLP1	cna	hetloss	BASIS
PD24186	CLP1	cna	gain	BASIS
PD24206	CLP1	cna	gain	BASIS
PD3905	CLP1	cna	gain	BASIS
PD4006	CLP1	cna	gain	BASIS
PD4107	CLP1	cna	amp	BASIS
PD4826	CLP1	cna	gain	BASIS
PD5935	CLP1	cna	gain	BASIS
PD5945	CLP1	cna	gain	BASIS
PD5948	CLP1	cna	gain	BASIS
PD6413	CLP1	cna	hetloss	BASIS
PD7067	CLP1	cna	gain	BASIS
PD7215	CLP1	cna	gain	BASIS
PD8980	CLP1	cna	hetloss	BASIS
PD9702	CLP1	cna	gain	BASIS
TCGA-A2-A25B	EGFR	cna	hetloss	TCGA
TCGA-AN-A0XU	EGFR	cna	hetloss	TCGA
TCGA-AO-A0JL	EGFR	cna	gain	TCGA
TCGA-BH-A0AW	EGFR	cna	gain	TCGA
TCGA-C8-A12L	EGFR	cna	gain	TCGA
TCGA-D8-A27M	EGFR	cna	gain	TCGA
TCGA-LL-A5YP	EGFR	cna	hetloss	TCGA
MB-0346	EGFR	cna	hetloss	METABRIC
MB-2827	EGFR	cna	amp	METABRIC
MB-6098	EGFR	cna	hetloss	METABRIC
MB-0420	EGFR	cna	hetloss	METABRIC
PD11327	EGFR	cna	gain	BASIS
PD13299	EGFR	cna	hetloss	BASIS
PD23562	EGFR	cna	gain	BASIS
PD24186	EGFR	cna	gain	BASIS
PD24206	EGFR	cna	gain	BASIS
PD3905	EGFR	cna	gain	BASIS
PD4006	EGFR	cna	gain	BASIS
PD4107	EGFR	cna	gain	BASIS

PD4826	EGFR	cna	gain	BASIS
PD5930	EGFR	cna	gain	BASIS
PD5945	EGFR	cna	amp	BASIS
PD6406	EGFR	cna	hetloss	BASIS
PD6413	EGFR	cna	gain	BASIS
PD7215	EGFR	cna	gain	BASIS
PD8621	EGFR	cna	gain	BASIS
PD9004	EGFR	cna	gain	BASIS
TCGA-AN-A0XU	FANCE	cna	hetloss	TCGA
TCGA-AO-A0JL	FANCE	cna	gain	TCGA
TCGA-BH-A0AW	FANCE	cna	gain	TCGA
TCGA-BH-A0C0	FANCE	cna	hetloss	TCGA
TCGA-C8-A12L	FANCE	cna	hetloss	TCGA
TCGA-E2-A1L7	FANCE	cna	hetloss	TCGA
TCGA-E9-A1NC	FANCE	cna	amp	TCGA
MB-7032	FANCE	cna	gain	METABRIC
PD11327	FANCE	cna	hetloss	BASIS
PD13296	FANCE	cna	gain	BASIS
PD13299	FANCE	cna	gain	BASIS
PD23562	FANCE	cna	gain	BASIS
PD24186	FANCE	cna	gain	BASIS
PD24206	FANCE	cna	gain	BASIS
PD3905	FANCE	cna	gain	BASIS
PD4006	FANCE	cna	gain	BASIS
PD4107	FANCE	cna	gain	BASIS
PD4826	FANCE	cna	gain	BASIS
PD5935	FANCE	cna	gain	BASIS
PD5945	FANCE	cna	amp	BASIS
PD5948	FANCE	cna	gain	BASIS
PD6731	FANCE	cna	hetloss	BASIS
PD7067	FANCE	cna	amp	BASIS
PD7215	FANCE	cna	gain	BASIS
PD8621	FANCE	cna	amp	BASIS
PD9585	FANCE	cna	gain	BASIS
PD9702	FANCE	cna	gain	BASIS
TCGA-A2-A25B	HGF	cna	hetloss	TCGA
TCGA-AN-A0XU	HGF	cna	hetloss	TCGA
TCGA-AO-A0JL	HGF	cna	gain	TCGA
TCGA-BH-A0AW	HGF	cna	gain	TCGA
TCGA-C8-A12L	HGF	cna	hetloss	TCGA
TCGA-D8-A27M	HGF	cna	gain	TCGA
TCGA-E9-A1NC	HGF	cna	gain	TCGA
MB-0346	HGF	cna	hetloss	METABRIC
MB-5465	HGF	cna	gain	METABRIC
PD11327	HGF	cna	amp	BASIS
PD13296	HGF	cna	hetloss	BASIS
PD23562	HGF	cna	gain	BASIS

PD23574	HGF	cna	gain	BASIS
PD24186	HGF	cna	amp	BASIS
PD3890	HGF	cna	hetloss	BASIS
PD3905	HGF	cna	gain	BASIS
PD4006	HGF	cna	gain	BASIS
PD4107	HGF	cna	gain	BASIS
PD4826	HGF	cna	gain	BASIS
PD5930	HGF	cna	gain	BASIS
PD5935	HGF	cna	gain	BASIS
PD5945	HGF	cna	amp	BASIS
PD5948	HGF	cna	gain	BASIS
PD6406	HGF	cna	hetloss	BASIS
PD7215	HGF	cna	gain	BASIS
PD9004	HGF	cna	gain	BASIS
PD9702	HGF	cna	gain	BASIS
TCGA-AN-A0XU	HMGA1	cna	gain	TCGA
TCGA-AO-A0JL	HMGA1	cna	gain	TCGA
TCGA-BH-A0AW	HMGA1	cna	gain	TCGA
TCGA-BH-A0C0	HMGA1	cna	hetloss	TCGA
TCGA-C8-A12L	HMGA1	cna	hetloss	TCGA
TCGA-E2-A1L7	HMGA1	cna	hetloss	TCGA
TCGA-E9-A1NC	HMGA1	cna	amp	TCGA
MB-7032	HMGA1	cna	gain	METABRIC
PD11327	HMGA1	cna	hetloss	BASIS
PD13296	HMGA1	cna	gain	BASIS
PD13299	HMGA1	cna	gain	BASIS
PD23562	HMGA1	cna	gain	BASIS
PD24186	HMGA1	cna	gain	BASIS
PD24206	HMGA1	cna	gain	BASIS
PD3905	HMGA1	cna	gain	BASIS
PD4006	HMGA1	cna	gain	BASIS
PD4107	HMGA1	cna	gain	BASIS
PD4826	HMGA1	cna	gain	BASIS
PD5935	HMGA1	cna	gain	BASIS
PD5945	HMGA1	cna	amp	BASIS
PD5948	HMGA1	cna	gain	BASIS
PD6731	HMGA1	cna	hetloss	BASIS
PD7067	HMGA1	cna	amp	BASIS
PD7215	HMGA1	cna	gain	BASIS
PD8621	HMGA1	cna	amp	BASIS
PD9585	HMGA1	cna	gain	BASIS
PD9702	HMGA1	cna	amp	BASIS
TCGA-AN-A0XU	INPP4A	cna	gain	TCGA
TCGA-AO-A0JL	INPP4A	cna	gain	TCGA
TCGA-D8-A27M	INPP4A	cna	gain	TCGA
TCGA-LL-A5YP	INPP4A	cna	amp	TCGA
MB-6060	INPP4A	cna	gain	METABRIC

MB-6098	INPP4A	cna	gain	METABRIC
MB-6271	INPP4A	cna	hetloss	METABRIC
PD11327	INPP4A	cna	gain	BASIS
PD13297	INPP4A	cna	hetloss	BASIS
PD13299	INPP4A	cna	gain	BASIS
PD23562	INPP4A	cna	amp	BASIS
PD23578	INPP4A	cna	gain	BASIS
PD24186	INPP4A	cna	gain	BASIS
PD24206	INPP4A	cna	gain	BASIS
PD24337	INPP4A	cna	gain	BASIS
PD3905	INPP4A	cna	gain	BASIS
PD4005	INPP4A	cna	hetloss	BASIS
PD4107	INPP4A	cna	gain	BASIS
PD4967	INPP4A	cna	hetloss	BASIS
PD5930	INPP4A	cna	hetloss	BASIS
PD5945	INPP4A	cna	amp	BASIS
PD5948	INPP4A	cna	gain	BASIS
PD7067	INPP4A	cna	gain	BASIS
PD7215	INPP4A	cna	gain	BASIS
PD8621	INPP4A	cna	gain	BASIS
PD9004	INPP4A	cna	gain	BASIS
PD9702	INPP4A	cna	gain	BASIS
TCGA-AN-A0XU	MAP4K4	cna	gain	TCGA
TCGA-AO-A0JL	MAP4K4	cna	gain	TCGA
TCGA-D8-A27M	MAP4K4	cna	gain	TCGA
TCGA-LL-A5YP	MAP4K4	cna	gain	TCGA
MB-5465	MAP4K4	cna	hetloss	METABRIC
MB-6060	MAP4K4	cna	amp	METABRIC
MB-6271	MAP4K4	cna	hetloss	METABRIC
PD11327	MAP4K4	cna	gain	BASIS
PD13296	MAP4K4	cna	gain	BASIS
PD13297	MAP4K4	cna	hetloss	BASIS
PD13299	MAP4K4	cna	gain	BASIS
PD23562	MAP4K4	cna	amp	BASIS
PD23578	MAP4K4	cna	gain	BASIS
PD24186	MAP4K4	cna	gain	BASIS
PD24206	MAP4K4	cna	gain	BASIS
PD3905	MAP4K4	cna	gain	BASIS
PD4005	MAP4K4	cna	hetloss	BASIS
PD4107	MAP4K4	cna	gain	BASIS
PD4967	MAP4K4	cna	hetloss	BASIS
PD5945	MAP4K4	cna	amp	BASIS
PD5948	MAP4K4	cna	gain	BASIS
PD7067	MAP4K4	cna	gain	BASIS
PD7215	MAP4K4	cna	gain	BASIS
PD8621	MAP4K4	cna	gain	BASIS
PD8980	MAP4K4	cna	homdel	BASIS

PD9004	MAP4K4	cna	gain	BASIS
PD9702	MAP4K4	cna	gain	BASIS
TCGA-A2-A25B	SDHAF2	cna	gain	TCGA
TCGA-AN-A0XU	SDHAF2	cna	hetloss	TCGA
TCGA-AO-A0JL	SDHAF2	cna	hetloss	TCGA
TCGA-BH-A18R	SDHAF2	cna	homdel	TCGA
TCGA-EW-A1OX	SDHAF2	cna	amp	TCGA
TCGA-LL-A5YP	SDHAF2	cna	hetloss	TCGA
MB-0346	SDHAF2	cna	gain	METABRIC
MB-6060	SDHAF2	cna	gain	METABRIC
MB-6098	SDHAF2	cna	hetloss	METABRIC
MB-6271	SDHAF2	cna	hetloss	METABRIC
PD11327	SDHAF2	cna	hetloss	BASIS
PD13771	SDHAF2	cna	hetloss	BASIS
PD24186	SDHAF2	cna	gain	BASIS
PD24202	SDHAF2	cna	hetloss	BASIS
PD24206	SDHAF2	cna	gain	BASIS
PD3905	SDHAF2	cna	gain	BASIS
PD4006	SDHAF2	cna	gain	BASIS
PD4826	SDHAF2	cna	gain	BASIS
PD5935	SDHAF2	cna	gain	BASIS
PD5945	SDHAF2	cna	amp	BASIS
PD5948	SDHAF2	cna	gain	BASIS
PD6413	SDHAF2	cna	gain	BASIS
PD7067	SDHAF2	cna	gain	BASIS
PD7215	SDHAF2	cna	gain	BASIS
PD8621	SDHAF2	cna	gain	BASIS
PD8980	SDHAF2	cna	hetloss	BASIS
PD9702	SDHAF2	cna	gain	BASIS
TCGA-AN-A0XU	AFF3	cna	gain	TCGA
TCGA-AO-A0JL	AFF3	cna	gain	TCGA
TCGA-D8-A27M	AFF3	cna	gain	TCGA
TCGA-LL-A5YP	AFF3	cna	amp	TCGA
MB-5465	AFF3	cna	hetloss	METABRIC
MB-6060	AFF3	cna	amp	METABRIC
MB-6271	AFF3	cna	hetloss	METABRIC
PD11327	AFF3	cna	gain	BASIS
PD13296	AFF3	cna	gain	BASIS
PD13297	AFF3	cna	hetloss	BASIS
PD13299	AFF3	cna	gain	BASIS
PD23562	AFF3	cna	amp	BASIS
PD23578	AFF3	cna	gain	BASIS
PD24186	AFF3	cna	gain	BASIS
PD24206	AFF3	cna	gain	BASIS
PD3905	AFF3	cna	gain	BASIS
PD4005	AFF3	cna	hetloss	BASIS
PD4107	AFF3	cna	gain	BASIS

PD4967	AFF3	cna	hetloss	BASIS
PD5945	AFF3	cna	amp	BASIS
PD5948	AFF3	cna	gain	BASIS
PD7067	AFF3	cna	gain	BASIS
PD7215	AFF3	cna	gain	BASIS
PD8621	AFF3	cna	gain	BASIS
PD9004	AFF3	cna	gain	BASIS
PD9702	AFF3	cna	gain	BASIS
TCGA-A2-A25B	IKZF1	cna	gain	TCGA
TCGA-AN-A0XU	IKZF1	cna	hetloss	TCGA
TCGA-AO-A0JL	IKZF1	cna	gain	TCGA
TCGA-BH-A0AW	IKZF1	cna	gain	TCGA
TCGA-BH-A0C0	IKZF1	cna	hetloss	TCGA
TCGA-C8-A12L	IKZF1	cna	gain	TCGA
TCGA-D8-A27M	IKZF1	cna	gain	TCGA
TCGA-LL-A5YP	IKZF1	cna	hetloss	TCGA
MB-0346	IKZF1	cna	hetloss	METABRIC
MB-2827	IKZF1	cna	hetloss	METABRIC
MB-6098	IKZF1	cna	hetloss	METABRIC
MB-0420	IKZF1	cna	hetloss	METABRIC
PD11327	IKZF1	cna	gain	BASIS
PD24186	IKZF1	cna	gain	BASIS
PD24202	IKZF1	cna	hetloss	BASIS
PD24206	IKZF1	cna	gain	BASIS
PD3905	IKZF1	cna	gain	BASIS
PD4006	IKZF1	cna	gain	BASIS
PD4107	IKZF1	cna	gain	BASIS
PD4826	IKZF1	cna	gain	BASIS
PD5930	IKZF1	cna	gain	BASIS
PD5945	IKZF1	cna	amp	BASIS
PD6406	IKZF1	cna	hetloss	BASIS
PD6413	IKZF1	cna	gain	BASIS
PD7215	IKZF1	cna	gain	BASIS
PD8621	IKZF1	cna	gain	BASIS
TCGA-A2-A25B	PCLO	cna	hetloss	TCGA
TCGA-AN-A0XU	PCLO	cna	hetloss	TCGA
TCGA-AO-A0JL	PCLO	cna	gain	TCGA
TCGA-BH-A0AW	PCLO	cna	gain	TCGA
TCGA-C8-A12L	PCLO	cna	hetloss	TCGA
TCGA-D8-A27M	PCLO	cna	gain	TCGA
TCGA-E9-A1NC	PCLO	cna	gain	TCGA
MB-0346	PCLO	cna	hetloss	METABRIC
MB-5465	PCLO	cna	gain	METABRIC
PD11327	PCLO	cna	gain	BASIS
PD13296	PCLO	cna	hetloss	BASIS
PD23574	PCLO	cna	gain	BASIS
PD24186	PCLO	cna	amp	BASIS

PD3890	PCLO	cna	hetloss	BASIS
PD3905	PCLO	cna	gain	BASIS
PD4006	PCLO	cna	gain	BASIS
PD4107	PCLO	cna	gain	BASIS
PD4826	PCLO	cna	gain	BASIS
PD5930	PCLO	cna	gain	BASIS
PD5935	PCLO	cna	gain	BASIS
PD5945	PCLO	cna	amp	BASIS
PD5948	PCLO	cna	gain	BASIS
PD6406	PCLO	cna	hetloss	BASIS
PD7215	PCLO	cna	gain	BASIS
PD9004	PCLO	cna	gain	BASIS
PD9702	PCLO	cna	gain	BASIS
TCGA-A2-A25B	GRM3	cna	hetloss	TCGA
TCGA-AN-A0XU	GRM3	cna	hetloss	TCGA
TCGA-AO-A0JL	GRM3	cna	gain	TCGA
TCGA-BH-A0AW	GRM3	cna	gain	TCGA
TCGA-C8-A12L	GRM3	cna	hetloss	TCGA
TCGA-D8-A27M	GRM3	cna	gain	TCGA
TCGA-E2-A1L7	GRM3	cna	hetloss	TCGA
TCGA-E9-A1NC	GRM3	cna	gain	TCGA
MB-0346	GRM3	cna	hetloss	METABRIC
PD11327	GRM3	cna	gain	BASIS
PD13296	GRM3	cna	hetloss	BASIS
PD23574	GRM3	cna	gain	BASIS
PD24186	GRM3	cna	amp	BASIS
PD3890	GRM3	cna	hetloss	BASIS
PD3905	GRM3	cna	gain	BASIS
PD4006	GRM3	cna	gain	BASIS
PD4107	GRM3	cna	gain	BASIS
PD4826	GRM3	cna	gain	BASIS
PD5930	GRM3	cna	gain	BASIS
PD5935	GRM3	cna	gain	BASIS
PD5945	GRM3	cna	amp	BASIS
PD5948	GRM3	cna	gain	BASIS
PD7215	GRM3	cna	gain	BASIS
PD9004	GRM3	cna	gain	BASIS
PD9702	GRM3	cna	gain	BASIS
MB-5465	HLA-A	cna	gain	METABRIC
MB-6098	HLA-A	cna	hetloss	METABRIC
MB-7032	HLA-A	cna	gain	METABRIC
MB-7038	HLA-A	cna	gain	METABRIC
PD11327	HLA-A	cna	hetloss	BASIS
PD13296	HLA-A	cna	gain	BASIS
PD13299	HLA-A	cna	gain	BASIS
PD23562	HLA-A	cna	gain	BASIS
PD24186	HLA-A	cna	gain	BASIS

PD24206	HLA-A	cna	gain	BASIS
PD3905	HLA-A	cna	gain	BASIS
PD4005	HLA-A	cna	amp	BASIS
PD4006	HLA-A	cna	gain	BASIS
PD4107	HLA-A	cna	gain	BASIS
PD4826	HLA-A	cna	gain	BASIS
PD5935	HLA-A	cna	gain	BASIS
PD5945	HLA-A	cna	amp	BASIS
PD5948	HLA-A	cna	gain	BASIS
PD6731	HLA-A	cna	hetloss	BASIS
PD7067	HLA-A	cna	amp	BASIS
PD7215	HLA-A	cna	gain	BASIS
PD8621	HLA-A	cna	gain	BASIS
PD9004	HLA-A	cna	gain	BASIS
PD9585	HLA-A	cna	gain	BASIS
PD9702	HLA-A	cna	gain	BASIS
TCGA-AN-A0XU	TTL	cna	hetloss	TCGA
TCGA-AO-A0JL	TTL	cna	gain	TCGA
TCGA-D8-A27M	TTL	cna	gain	TCGA
TCGA-LL-A5YP	TTL	cna	gain	TCGA
MB-5465	TTL	cna	hetloss	METABRIC
MB-6271	TTL	cna	hetloss	METABRIC
PD13297	TTL	cna	hetloss	BASIS
PD13299	TTL	cna	gain	BASIS
PD23578	TTL	cna	hetloss	BASIS
PD24186	TTL	cna	gain	BASIS
PD24202	TTL	cna	hetloss	BASIS
PD24206	TTL	cna	gain	BASIS
PD3905	TTL	cna	gain	BASIS
PD4005	TTL	cna	hetloss	BASIS
PD4107	TTL	cna	gain	BASIS
PD4967	TTL	cna	hetloss	BASIS
PD5945	TTL	cna	gain	BASIS
PD5948	TTL	cna	gain	BASIS
PD6413	TTL	cna	hetloss	BASIS
PD7067	TTL	cna	gain	BASIS
PD7215	TTL	cna	gain	BASIS
PD8621	TTL	cna	gain	BASIS
PD9004	TTL	cna	gain	BASIS
PD9585	TTL	cna	hetloss	BASIS
PD9702	TTL	cna	gain	BASIS
PD10014	TMSB4XP8	cna	gain	BASIS
PD13296	TMSB4XP8	cna	hetloss	BASIS
PD13297	TMSB4XP8	cna	hetloss	BASIS
PD13771	TMSB4XP8	cna	gain	BASIS
PD22355	TMSB4XP8	cna	hetloss	BASIS
PD23562	TMSB4XP8	cna	gain	BASIS

PD23578	TMSB4XP8	cna	hetloss	BASIS
PD24202	TMSB4XP8	cna	hetloss	BASIS
PD24206	TMSB4XP8	cna	gain	BASIS
PD3890	TMSB4XP8	cna	gain	BASIS
PD3905	TMSB4XP8	cna	gain	BASIS
PD4006	TMSB4XP8	cna	hetloss	BASIS
PD4107	TMSB4XP8	cna	gain	BASIS
PD5930	TMSB4XP8	cna	hetloss	BASIS
PD5935	TMSB4XP8	cna	gain	BASIS
PD5945	TMSB4XP8	cna	gain	BASIS
PD5948	TMSB4XP8	cna	gain	BASIS
PD6406	TMSB4XP8	cna	gain	BASIS
PD6731	TMSB4XP8	cna	hetloss	BASIS
PD7067	TMSB4XP8	cna	gain	BASIS
PD7215	TMSB4XP8	cna	gain	BASIS
PD8621	TMSB4XP8	cna	hetloss	BASIS
PD8980	TMSB4XP8	cna	hetloss	BASIS
PD9585	TMSB4XP8	cna	hetloss	BASIS
PD10014	CRLF2	cna	hetloss	BASIS
PD11742	CRLF2	cna	gain	BASIS
PD13771	CRLF2	cna	hetloss	BASIS
PD23562	CRLF2	cna	hetloss	BASIS
PD23574	CRLF2	cna	gain	BASIS
PD24206	CRLF2	cna	gain	BASIS
PD3890	CRLF2	cna	hetloss	BASIS
PD3905	CRLF2	cna	amp	BASIS
PD4005	CRLF2	cna	hetloss	BASIS
PD4006	CRLF2	cna	hetloss	BASIS
PD4107	CRLF2	cna	hetloss	BASIS
PD4826	CRLF2	cna	gain	BASIS
PD4967	CRLF2	cna	hetloss	BASIS
PD5930	CRLF2	cna	hetloss	BASIS
PD5935	CRLF2	cna	gain	BASIS
PD5945	CRLF2	cna	hetloss	BASIS
PD5948	CRLF2	cna	hetloss	BASIS
PD6406	CRLF2	cna	gain	BASIS
PD7067	CRLF2	cna	gain	BASIS
PD7215	CRLF2	cna	gain	BASIS
PD8621	CRLF2	cna	gain	BASIS
PD8980	CRLF2	cna	hetloss	BASIS
PD9004	CRLF2	cna	gain	BASIS
MB-6098	HLA-B	cna	hetloss	METABRIC
MB-7032	HLA-B	cna	gain	METABRIC
PD10014	HLA-B	cna	hetloss	BASIS
PD11327	HLA-B	cna	hetloss	BASIS
PD13296	HLA-B	cna	gain	BASIS
PD13299	HLA-B	cna	gain	BASIS

PD23562	HLA-B	cna	gain	BASIS
PD24186	HLA-B	cna	gain	BASIS
PD24206	HLA-B	cna	gain	BASIS
PD3905	HLA-B	cna	gain	BASIS
PD4006	HLA-B	cna	gain	BASIS
PD4107	HLA-B	cna	gain	BASIS
PD4826	HLA-B	cna	gain	BASIS
PD5935	HLA-B	cna	gain	BASIS
PD5945	HLA-B	cna	amp	BASIS
PD5948	HLA-B	cna	gain	BASIS
PD6731	HLA-B	cna	hetloss	BASIS
PD7067	HLA-B	cna	amp	BASIS
PD7215	HLA-B	cna	gain	BASIS
PD8621	HLA-B	cna	amp	BASIS
PD9004	HLA-B	cna	gain	BASIS
PD9585	HLA-B	cna	gain	BASIS
PD9702	HLA-B	cna	amp	BASIS
TCGA-AO-A0JL	PAX8	cna	gain	TCGA
TCGA-D8-A27M	PAX8	cna	gain	TCGA
TCGA-LL-A5YP	PAX8	cna	gain	TCGA
MB-5465	PAX8	cna	hetloss	METABRIC
MB-6271	PAX8	cna	hetloss	METABRIC
PD11742	PAX8	cna	gain	BASIS
PD13297	PAX8	cna	hetloss	BASIS
PD13299	PAX8	cna	gain	BASIS
PD23578	PAX8	cna	hetloss	BASIS
PD24186	PAX8	cna	gain	BASIS
PD24202	PAX8	cna	hetloss	BASIS
PD24206	PAX8	cna	gain	BASIS
PD3905	PAX8	cna	gain	BASIS
PD4107	PAX8	cna	gain	BASIS
PD4967	PAX8	cna	hetloss	BASIS
PD5945	PAX8	cna	gain	BASIS
PD5948	PAX8	cna	gain	BASIS
PD6413	PAX8	cna	hetloss	BASIS
PD7067	PAX8	cna	gain	BASIS
PD7215	PAX8	cna	gain	BASIS
PD8621	PAX8	cna	gain	BASIS
PD9004	PAX8	cna	gain	BASIS
PD9702	PAX8	cna	gain	BASIS
PD10014	ASMTL	cna	hetloss	BASIS
PD11742	ASMTL	cna	gain	BASIS
PD13771	ASMTL	cna	hetloss	BASIS
PD23562	ASMTL	cna	hetloss	BASIS
PD23574	ASMTL	cna	gain	BASIS
PD24206	ASMTL	cna	gain	BASIS
PD3890	ASMTL	cna	hetloss	BASIS

PD3905	ASMTL	cna	amp	BASIS
PD4005	ASMTL	cna	hetloss	BASIS
PD4006	ASMTL	cna	hetloss	BASIS
PD4107	ASMTL	cna	hetloss	BASIS
PD4826	ASMTL	cna	gain	BASIS
PD4967	ASMTL	cna	hetloss	BASIS
PD5930	ASMTL	cna	hetloss	BASIS
PD5945	ASMTL	cna	hetloss	BASIS
PD5948	ASMTL	cna	hetloss	BASIS
PD6406	ASMTL	cna	gain	BASIS
PD7067	ASMTL	cna	gain	BASIS
PD7215	ASMTL	cna	gain	BASIS
PD8621	ASMTL	cna	gain	BASIS
PD8980	ASMTL	cna	hetloss	BASIS
PD9004	ASMTL	cna	gain	BASIS
PD10014	P2RY8	cna	hetloss	BASIS
PD11742	P2RY8	cna	gain	BASIS
PD13771	P2RY8	cna	hetloss	BASIS
PD23562	P2RY8	cna	hetloss	BASIS
PD23574	P2RY8	cna	gain	BASIS
PD24206	P2RY8	cna	gain	BASIS
PD3890	P2RY8	cna	hetloss	BASIS
PD3905	P2RY8	cna	amp	BASIS
PD4005	P2RY8	cna	hetloss	BASIS
PD4006	P2RY8	cna	hetloss	BASIS
PD4107	P2RY8	cna	hetloss	BASIS
PD4826	P2RY8	cna	gain	BASIS
PD4967	P2RY8	cna	hetloss	BASIS
PD5930	P2RY8	cna	hetloss	BASIS
PD5945	P2RY8	cna	hetloss	BASIS
PD5948	P2RY8	cna	hetloss	BASIS
PD6406	P2RY8	cna	gain	BASIS
PD7067	P2RY8	cna	gain	BASIS
PD7215	P2RY8	cna	gain	BASIS
PD8621	P2RY8	cna	gain	BASIS
PD8980	P2RY8	cna	hetloss	BASIS
PD9004	P2RY8	cna	gain	BASIS
