# Supplementary material for: Comparative oncogenomics identifies combinations of driver genes and drug targets in BRCA1-mutated breast cancer
Source: Nat Commun. 2019 Jan 23;10:397. doi: 10.1038/s41467-019-08301-2 (PMC6344487; doi:10.1038/s41467-019-08301-2)
Supplement: Supplementary file 3 — Reporting Summary [file 41467_2019_8301_MOESM3_ESM.pdf]

nature research

Corresponding author(s): *Double-blind peer review submissions: write DBPR and your manuscript number here instead of author names.*

Reporting Summary

Nature Research wishes to improve the reproducibility of the work that we publish. This form provides structure for consistency and transparency in reporting. For further information on Nature Research policies, see Authors & Referees and the Editorial Policy Checklist.

Statistical parameters

When statistical analyses are reported, confirm that the following items are present in the relevant location (e.g. figure legend, table legend, main text, or Methods section).

n/a	Confirmed	
☐	☒	The exact sample size (n) for each experimental group/condition, given as a discrete number and unit of measurement
☐	☒	An indication of whether measurements were taken from distinct samples or whether the same sample was measured repeatedly
☐	☒	The statistical test(s) used AND whether they are one- or two-sided<br>*Only common tests should be described solely by name; describe more complex techniques in the Methods section.*
☒	☐	A description of all covariates tested
☐	☒	A description of any assumptions or corrections, such as tests of normality and adjustment for multiple comparisons
☐	☒	A full description of the statistics including central tendency (e.g. means) or other basic estimates (e.g. regression coefficient) AND variation (e.g. standard deviation) or associated estimates of uncertainty (e.g. confidence intervals)
☐	☒	For null hypothesis testing, the test statistic (e.g. F, t, r) with confidence intervals, effect sizes, degrees of freedom and P value noted<br>*Give P values as exact values whenever suitable.*
☒	☐	For Bayesian analysis, information on the choice of priors and Markov chain Monte Carlo settings
☒	☐	For hierarchical and complex designs, identification of the appropriate level for tests and full reporting of outcomes
☒	☐	Estimates of effect sizes (e.g. Cohen's d, Pearson's r), indicating how they were calculated
☐	☒	Clearly defined error bars<br>*State explicitly what error bars represent (e.g. SD, SE, CI)*

Our web collection on statistics for biologists may be useful.

Software and code

Policy information about availability of computer code

Data collection	FlowJo software version 7.6.5., TIDE (http://tide.nki.nl), MAGeCK (https://sourceforge.net/p/mageck/wiki/Home), Align-GVGD (http://agvgd.iarc.fr), STAR (version 2.5.3a). QC statistics from Fastqc (version 0.11.5), Multiqc (version 1.1), featureCounts (version 1.5.2), Cutadapt (version 1.15), BWA aln (version 0.7.15), QDNAseq (version 1.14.0), RUBIC (version 1.0.3), Snakemake (version 4.3.1), ImageScope software version 12.0.0 (Aperio)
Data analysis	Graphpad Prism 7.03., Microsoft Excel, Python 3.5

For manuscripts utilizing custom algorithms or software that are central to the research but not yet described in published literature, software must be made available to editors/reviewers upon request. We strongly encourage code deposition in a community repository (e.g. GitHub). See the Nature Research guidelines for submitting code & software for further information.

Data

Policy information about availability of data

All manuscripts must include a data availability statement. This statement should provide the following information, where applicable:

- Accession codes, unique identifiers, or web links for publicly available datasets
- A list of figures that have associated raw data
- A description of any restrictions on data availability

All sequence data that support the findings of this study are available in the European Nucleotide Archive under accession number PRJEB30443. Any additional data are available from the corresponding author upon reasonable request.

Field-specific reporting

Please select the best fit for your research. If you are not sure, read the appropriate sections before making your selection.

☒ Life sciences ☐ Behavioural & social sciences ☐ Ecological, evolutionary & environmental sciences

For a reference copy of the document with all sections, see nature.com/authors/policies/ReportingSummary-flat.pdf

Life sciences study design

All studies must disclose on these points even when the disclosure is negative.

Sample size	No sample size calculation was performed. Sample sizes were chosen based on experience with previous experiments
Data exclusions	No samples were excluded from analysis
Replication	All attempts at replication were successful
Randomization	Cells and mice were randomly allocated to the various conditions.
Blinding	Investigators were blinded to group allocation during data collection

Reporting for specific materials, systems and methods

Materials & experimental systems

n/a	Involved in the study	
☐	☒	Unique biological materials
☐	☒	Antibodies
☐	☒	Eukaryotic cell lines
☒	☐	Palaeontology
☐	☒	Animals and other organisms
☒	☐	Human research participants

Methods

n/a	Involved in the study	
☒	☐	ChIP-seq
☐	☒	Flow cytometry
☒	☐	MRI-based neuroimaging

Unique biological materials

Policy information about availability of materials

Obtaining unique materials	All unique materials are readily available from the authors

Antibodies

Antibodies used	anti-Met (1:1000, Cell Signaling 4560S)<br>anti-phosphoMet (1:1000, Cell Signaling 3077S)<br>anti-Myc (1:1000, Abcam ab32072)<br>anti-Mcl1 (1:1000, Cell Signaling 94296S)

Validation	Commercially available antibodies were validated by the manufacturer and all of them are have been previously used in multiple publications. For further details including lists of publications please refer to the suppliers websites.

Eukaryotic cell lines

Policy information about cell lines

Cell line source(s)	293T: American Tissue Culture Collection (ATCC)
Authentication	None of the cell lines were authenicated
Mycoplasma contamination	All cell lines were tested negative for Mycoplasma contamination
Commonly misidentified lines (See ICLAC register)	No commonly misidentified cell lines were used

Animals and other organisms

Policy information about studies involving animals; ARRIVE guidelines recommended for reporting animal research

Laboratory animals	The investigators used in house genetically modified female FVB mice of various ages as indicated in material and method sections and or figure legends
Wild animals	The study did not involve wild animals
Field-collected samples	The study did not contain samples collect from the field

Flow Cytometry

Plots

Confirm that:

☒ The axis labels state the marker and fluorochrome used (e.g. CD4-FITC).

☒ The axis scales are clearly visible. Include numbers along axes only for bottom left plot of group (a 'group' is an analysis of identical markers).

☒ All plots are contour plots with outliers or pseudocolor plots.

☒ A numerical value for number of cells or percentage (with statistics) is provided.

Methodology

Sample preparation	Cells were collected 5 days after transduction, washed in PBS, fixed in Fixation Buffer (BD Biosciences) and permeabilized with Perm Buffer III (BD Biosciences). They were then stained using the primary rabbit antibody anti-Myc (1:1000, Abcam ab32072) or anti-Mcl1 (1:1000, Cell Signaling 94296S) for 30 minutes at 4 degrees, washed in PBS and incubated for 15 minutes with an AlexaFluor647-conjugated secondary anti-rabbit antibody (1:1000, Thermofisher)
Instrument	BD LSR Fortessa Cell Analyzer
Software	The data was collected using BD FACSDiva Software and analysed using FlowJo (BD Inc, USA)
Cell population abundance	No cell sorting was performed
Gating strategy	Based on FSC-A vs SSC-A, cellular debris were excluded. Next, FSC-A vs FSC-H was used to exclude doublets.

☒ Tick this box to confirm that a figure exemplifying the gating strategy is provided in the Supplementary Information.
